# Nutraceuticals and phytoceuticals for the treatment of major depressive disorder: a systematic review and network meta-analysis

**DOI:** 10.1038/s41380-026-03759-1

**Published:** 2026-09-01

**Authors:** Michele Fornaro, Chiara Di Lorenzo, Nicolas Nunez, Alessandro Miola, Raffaella Calati, André Ferrer Carvalho, Luca Steardo, Brendon Stubbs, David Mischoulon, Christoph U. Correll, Marco Solmi

**Affiliations:** 1 Section of Psychiatry, Federico II University of Naples, Naples, Italy; 2 Federico II University of Naples, Naples, Italy; 3 Department of Psychiatry & Psychology, Mayo Clinic, Rochester, MN USA; 4 Department of Psychiatry, University of Utah, Salt Lake City, UT USA; 5 Department of Neuroscience (DNS), Padua Neuroscience Center, University of Padova, Padua, Italy; 6 University of Milano-Bicocca, Milan, Italy; 7 Innovation in Mental and Physical Health and Clinical Treatment (IMPACT) Strategic Research Centre, School of Medicine, Barwon Health, Deakin University, Geelong, VIC Australia; 8 Psychiatric Unit, Department of Health Sciences, University Magna Graecia of Catanzaro, Catanzaro, Italy; 9 Department of Psychological Medicine, Institute of Psychiatry, Psychology and Neuroscience, King’s College London, London, WC2R 2LS UK; 10 Comprehensive Center for Clinical Neurosciences and Mental Health, Medical University of Vienna, 1090 Vienna, Austria; 11 Division of Social Psychiatry, Department of Psychiatry and Psychotherapy, Medical University of Vienna, 1090 Vienna, Austria; 12 Division of Psychology and Mental Health, University of Manchester, Manchester Academic Health Science Centre, Manchester, UK; 13 Depression Clinical and Research Program, Massachusetts General Hospital, Harvard Medical School, Boston, 02114 USA; 14 Department of Child and Adolescent Psychiatry, Charité Universitätsmedizin, Berlin, Germany; 15 The Zucker Hillside Hospital, Department of Psychiatry, Northwell Health, Glen Oaks, NY USA; 16 Donald and Barbara Zucker School of Medicine at Hofstra/Northwell, Department of Psychiatry and Molecular Medicine, Hempstead, NY USA; 17 Partner Site Berlin-Potsdam, German Center for Mental Health (DZPG), Berlin, Germany; 18 Department of Psychiatry, Faculty of Medicine, University of Ottawa, Ottawa, ON Canada; 19 Department of Mental Health, The Ottawa Hospital, Ottawa, ON Canada; 20 Ottawa Hospital Research Institute, OHRI, Ottawa, ON Canada

**Keywords:** Depression, Neuroscience

## Abstract

**Background:**

“Nutraceuticals” and “phytoceuticals” may have antidepressant activity, prompting an in-depth network meta-analysis of randomized clinical trials (RCTs).

**Methods:**

Systematic review and network meta-analysis (PubMed/MEDLINE/EMBASE/Scopus/PsycINFO/CENTRAL/ClinicalTrials.gov (until 01/07/2025) of RCTs vs. placebo or treatment as usual (TAU = antidepressants) testing nutraceuticals/phytoceuticals in major depressive disorder (MDD). Change in depressive symptomatology (standardized-mean-difference = SMD), treatment response, and all-cause discontinuation (acceptability) (risk ratio = RR) were co-primary outcomes. Secondary outcomes were anxiety symptom changes, adverse event-related discontinuation (“tolerability”), and symptom remission. We conducted subgroup, transitivity, and sensitivity analyses to explore/reduce heterogeneity, and assessed global/local inconsistency, publication bias, risk of bias (RoB), and confidence in the evidence (CINeMA/AMSTAR-Plus).

**Results:**

Across 163 studies (n = 15,757, distinct compounds’ combinations n = 137), several molecules/classes outperformed placebo with very large effect sizes. Restricting analyses to compounds with ≥2RCTs/non-TAU antidepressants/low RoB/non-sponsored trials/non-outliers, higher-than-expected/unrealistic effect sizes emerged vs. placebo for eicosapentaenoic acid (EPA) (k = 20, n = 8483; SMD = −1.67;95%C.I. = −2.38;−0.97), vitamin D3 (k = 3, n = 344; SMD = −1.59;95%C.I. = −2.71;−0.46), and St. John’s-wort-extract-ZE117 (k = 3,n = 207;SMD = −1.02;95%C.I = −1.90;−0.15) regarding depressive symptomatology. Substantial heterogeneity (I^2^ = 85.3%;95%C.I. = 81.9%;88.0%) and global inconsistency (Q-between-designs = 186.91, p < 0.0001) emerged, explained by subgroup and sensitivity analyses, without publication bias. Additionally, a medium effect size emerged vs. placebo for curcumin (k = 2,n = 89;SMD = −0.58;95%C.I. = −0.99;−0.18) regarding anxiety reduction in low RoB analyses. Shugan granules, EPA, EPA+Docosahexaenoic Acid, ZE117, and fluoxetine+EPA response and St. John’s wort extract WS5570/ZE117 remission significantly outperformed placebo. Citicoline was associated with significantly more dropouts than placebo. A meta-regression of efficacy effect sizes against adapted AMSTAR-Plus scores showed that larger SMDs were associated with lower AMSTAR scores, indicating lower study quality. Confidence in the evidence was low to very low.

**Conclusions:**

Upon filtering low-quality evidence, only a few nutraceutical/phytoceuticals showed potential for depressive symptoms, warranting more rigorous trials.

## Introduction

Integrative and complementary medicine, including nutrient-based “nutraceuticals” or plant-based “phytoceuticals,” has gained attention in recent years as a potential monotherapy or adjunctive therapy to standard antidepressants [1, 2], being systematically appraised by international treatment guidelines informed by meta-analytic evidence for major psychiatric disorders, including major depressive disorder (MDD) [1, 3].

A recent network meta-analysis (NMA) of nutraceuticals for depression aimed to expand prior knowledge but failed to account for substantial heterogeneity across most outcomes [4].

Since MDD affects millions of people worldwide, posing a significant burden to the suffering ones and their caregivers [5], and nutraceuticals and phytoceuticals are broadly used, especially in specific geographical regions and cultures relying on secular lore [6] further appraisal of such compounds is needed, especially considering that they are usually perceived as “safe” or “natural” and “cost-effective” interventions to relieve depression, even in the absence of mandatory regulatory approvals by most national agencies [7].

Well-conducted NMAs enable simultaneous comparison of multiple interventions, providing exploratory estimates of comparisons between interventions that have not been directly compared in single randomized controlled trials (RCTs) [8].

The present NMA aims to inform clinical practice by investigating the efficacy, discontinuation due to adverse events (hereinafter “tolerability”), and discontinuation due to any cause (hereinafter “acceptability”) of a broad range of nutraceuticals and phytoceuticals in the treatment of MDD, with the aim to fill the gap of previous evidence about unexplained heterogeneity, and inconsistency mining the overall credibility of the findings.

## Methods

The present systematic review (SR) and NMA adhered to the Preferred Reporting Items for Systematic Reviews and Meta-analyses (PRISMA) [9], following an a priori-designed protocol registered with PROSPERO (CRD420251085073), and replicated the methods previously applied elsewhere [10]. Protocol amendments appear in the supplementary material (S1a).

### Literature search

We systematically searched PubMed/MEDLINE/EMBASE/Scopus/PsycINFO/CENTRAL/ClinicalTrials.gov databases from their inception until July 1, 2025. A manual search was conducted, augmenting this search strategy (detailed in Supplementary material-S1b). Two authors (MF; CDL) independently screened identified records at the title/abstract and full-text levels, and a third author (NN) resolved any discrepancies.

### Inclusion and exclusion criteria

Inclusion criteria were: (i) pharmacological RCTs involving either monotherapy or combination and augmentation therapies of nutraceuticals (considered as any pharmaceutical-grade nutrient) and phytoceuticals (any plant-derived molecule that is active on a biological system); (ii) adult participants with a primary diagnosis of MDD (“unipolar depression”) according to the Diagnostic and Statistical Manual for Mental Disorders (DSM) or the International Classification of Diseases (ICD) criteria, any edition.

Comparators/controls were either a placebo or “treatment as usual” (TAU) antidepressants, i.e., “stable antidepressant regimen that was continued unchanged”.

Exclusion criteria were: (i) lack of current diagnosis of MDD; (ii) non-randomized trials/observational studies; (iii) trials involving pediatric samples only.

We considered cross-over trials only when pre-cross endpoint effects were available.

### Data extraction

Two independent authors (CDL, MF) identified and extracted data from eligible trials. Inconsistencies were resolved by consulting a third author (NN). Extracted information was age, sex, diagnostic criteria, (semi-)structured diagnostic interview, comorbidities, trial duration, blinding status, intervention(s) with dosage and administration schedule, control condition, concurrent psychiatric treatments and their mean dose, sponsorship, response/remission operational definition, composition of the herbal compound (molecule and class, regarded as node-1 and node-2, respectively – see supplementary material S4), country, publication year, data-analysis (intention-to-treat [ITT] vs. per-protocol), outcome assessment (main rating scales); severity of depression and anxiety at baseline and endpoint/change from baseline.

Relevant information was extracted from the study baseline and endpoint records. WebPlotDigitizer [11] was used to extract data from figures when relevant information was not otherwise disclosed. In addition, we planned up to two attempts to contact the author regarding potentially missing data.

### Outcomes

Co-primary efficacy outcomes were: (i) mean change from baseline as standardized mean difference (SMD) of depressive symptoms according to validated rating scales such as the Hamilton Depression Rating Scale (HAM-D) [12] and the Montgomery-Äsberg Depression Rating Scale (MADRS) [13], and the Beck Depression Inventory (BDI) version I [14] or II [15] (an improvement in depressive symptoms is defined by a negative value of SMD); (ii) response as per author definition – Risk Ratio (RR), and (iii) acceptability effects.

Mean change from baseline (as SMD) of anxious symptomatology associated with MDD according to the Hamilton Anxiety Rating Scale (HAM-A) [16], the Beck Anxiety Inventory (BAI) [17], and the State-Trait Anxiety Inventory (STAI) [18] (an improvement in depressive symptoms is defined by a negative value of SMD), tolerability (discontinuation due to adverse effects) and remission (as per author definition) – RRs – were the co-secondary outcomes.

### Risk-of-bias evaluation

Four authors (CDL, MF, NN, AM) assessed the risk of bias at the study level using version 2 of the Cochrane risk-of-bias tool for randomized trials and randomized crossover trials (RoB2 [19], based solely on pre-cross-over data.

### Data analysis

We performed a random-effects NMA for each outcome within the frequentist framework. Specifically, the adopted random-effects model assumes that observed treatment effect estimates vary due to study heterogeneity arising from sampling variability (e.g., age, intervention doses, study methodology and quality, follow-up length, and other known and unknown variables), as well as differences in treatment effects [20]. Before conducting the NMA, we inspected the connection’s structure to determine whether multiple independent subnetworks were present. We analyzed only networks that included at least 10 studies.

Node-1 (molecule) and node-2 (nutraceutical/phytoceuticals compound/antidepressant class) (Table 1 and S4) were appraised for estimates of the variability proportion and the true variance’s absolute value (heterogeneity) measured using the I² statistical test and a τ² statistic, respectively. The proportion of total variability attributable to heterogeneity (I^2^) was considered high if ≥50% [21]. We also assessed the statistical difference between direct and indirect effect sizes by evaluating global inconsistency using a Q-statistic test: a statistically significant Q-statistic (p-value < 0.05) indicates inconsistency [22]. Local inconsistency was measured using a loop-specific approach to assess agreement between direct and indirect estimates [23]. A hierarchy of the evaluated drugs was calculated for each outcome using the cumulative ranking curve (P-score) in a frequentist NMA [24].

**Table 1:** Individual compounds and corresponding classes of molecules.

Molecule	Class (either pharmacological or nutraceutical/phytoceutical)
Aphrodite	Herbal compound
Ayahuasca	Herbal compound
Bifidobacterium longum	Probiotic
Biokult	Probiotic
Sodium Benzoate	Coenzyme
L-Carnitine	Amino acids
Chromium picolinate	Minerals
Cinnamomum tamala	Lauraceae
Citicoline	Nucleotide
Clomipramine	TCA
Coenzyme Q10	Coenzyme
Creatine	Amino acids
Citalopram	SSRI
Curcumin	Zingiberaceae
Cuscuta planiflora	Convolvulaceae
Chlorella Vulgaris extract	Microalgae
Docosahexaenoic acid (DHA)	LCPUFA
Dracocephalum ruyschiana	Lamiaceae
Ellagic acid	Polyphenols
Echium amoenum compound syrup	Herbal compound
Essential oil of Perilla frutescens	LCPUFA
Eicosapentanoic acid	LCPUFA
Escitalopram	SSRI
Free amino acids	Amino acids
Fluoxetine	SSRI
Fish oil	LCPUFA
Folic Acid	Vitamins
Ginkgo biloba dropping pill	Ginkgoaceae
Gami Guibitang (Korean traditional medicinal herb)	Herbal compound
Galactooligosaccharide	Polysaccharide
Lactobacillus Plantarum HEAL9	Probiotic
Hypericum perforatum	Hypericaceae
Imipramine	TCA
Inositol	Vitamins
Inulin	Polysaccharide
Jiaotai pill	Herbal compound
Krill oil	LCPUFA
Lavandula angustifolia	Lamiaceae
Lactobacillus acidophilus	Probiotic
Lactobacillus helveticus	Probiotic
LoHyp57 (hypericum extract)	Hypericaceae
LI-160 St John’s wort extract	Hypericaceae
L-methyl-folate	Vitamins
Lactobacillus Plantarum299v	Probiotic
Lactosucrose	Polysaccharide
Melissa Officinalis	Lamiaceae
Magnesium	Minerals
Mirtazapine	NaSSA
N-acetylcysteine	Amino acids
Nepeta menthoides	Lamiaceae
Nutraceutical combination (SAMe+folinic acid+B12+omega3 + 5-HTP+zinc+B6+vitC+magnesium)	Coenzyme+Vitamins+LCPUFA+Minerals+Amino acids
OmniBiotic drink	Probiotic
Paroxetine	SSRI
Propolis	Flavonoid+Polyphenols
Lactobacillus Plantarum PS128	Probiotic
Rhodiola Rosea	Crassulaceae
Saffron (Crocus sativus)	Iridaceae
S-adenosylmethionine	Coenzyme
Sarcosine (N-methyl-glycine)	Amino acids
Scutellaria	Herbal compound
Sertraline	SSRI
Shugan granule	Herbal compound
Silexan (active substance from Lav.angustifolia)	Lamiaceae
St John’s wort	Hypericaceae
D-Serine	Amino acids
Sulbutiamine	Vitamins
Synbiotic	Probiotic
Shen Zi Ling	Herbal compound
Tensilen	Coenzyme
L-theanine	Amino acids
Thiamine (Vitamin B1)	Vitamins
Üstikuddus sherbiti	Herbal compound
Venlafaxine	SNRI
Vitamin D	Vitamins
Hypericum perforatum extract WS5570	Hypericaceae
St John’s wort extract WS 5572	Hypericaceae
St John’s wort extract WS 5573	Hypericaceae
Yueju	Herbal compound
Hypericum extract ZE	Hypericaceae
St John’s wort extract ZE 117	Hypericaceae
Zinc	Minerals
Zishen pingchan granules	Herbal compound

Planned sensitivity analyses for co-primary and co-secondary outcomes were: (i) inclusion of low-risk of bias studies only; (ii) exclusion of sponsored trials/undisclosed financial support; (iii) exclusion of the pre-DSM-IV [25] studies and outlier RCTs identified by transitivity analyses. Specifically, transitivity analyses accounted for the following potential effect modifiers: (i) proportion of female participants; (ii) mean age; and (iii) baseline severity of depression measured by the HAM-D or equivalent rating scale. Planned sub-group analyses vs. placebo considered: (i) clinical (semi-)structured interview vs. no-interview; (ii) sponsorship (both industry and academic) vs. no-sponsorship; (iii) setting of the study (outpatients vs. inpatients); (iv) nutraceutical vs. phytoceuticals compounds; (v) geographical region (continent); (vi) single- vs. multicenter trial; (vii) classes of molecules; and (viii) treatment-resistant depression (TRD) population vs. non-TRD population. Planned sub-group analyses involving. TAU antidepressants(i.e., background antidepressant treatment) considered: TAU antidepressants-augmentation with the nutraceuticals or phytoceuticals (e.g., TAU antidepressants +curcumin vs. TAU antidepressants +placebo) vs. no-augmentation of the nutraceutical or phytoceuticals (e.g., curcumin vs. TAU antidepressants only). We assessed the normality of the distributions of baseline depression severity, trial length (in weeks), and mean age using the Shapiro-Wilk test. We applied the Wilcoxon test to compare variables with non-normal distributions between subgroups. We used the SMD to measure change in depression severity (lower SMD indicated better performance in reducing the total depression score at the study endpoint compared with baseline). We used RR to assess response, remission, acceptability, and tolerability. All outcomes were evaluated with the corresponding 95% confidence intervals (CIs), with statistical significance set at p = .05. Following the Cochrane Handbook, second edition, missing standard deviations (SDs) were imputed by computing the weighted mean of the available SDs [26].

We assessed publication bias and the small-study effect using funnel-plot inspection and Egger’s test [27] for all analyses involving more than 10 studies. All analyses were conducted using R version 4.3.2 [28] and netmeta v2.8-2 [29].

### Confidence in evidence

Confidence in the evidence for co-primary outcomes (node-1) was assessed using the Confidence in Network Meta-Analysis (CINeMA) framework [30].

### Interpretation of network meta-analysis findings in the context of real trial data

Findings from NMA can be confounded by suboptimal network connectivity, indirect comparisons, small sample sizes, trial quality, and other clinical and methodological factors. It is essential that NMA results are critically interpreted and that they also account for the real data, i.e., individual RCTs. Hence, in addition to conducting sensitivity analyses, we critically compared NMA findings with those from individual RCTs and qualitatively assessed the overall body of evidence in our discussion of the results. Moreover, the interpretation of results was also informed by the consistency of efficacy findings across outcomes and by clinical credibility. Finally, an adapted version of the AMSTAR plus content total score [31] was employed to rate the quality of the included RCTs, weighted for the number of comparisons (score range = 0–7, with a higher score indicating higher quality, using the following adapted items: (1) Blinding in studies yes/no (score range 0–1); (2) sample size (n < 20 score = 0, n = 20–100 score 1, n > 100 score = 2); (3) findings replicated in ≥1 comparison (1 point), (4) n > 20 in ≥1study arm (1 point); 5) ITT (no = 0, yes = 1); (6) High (score = 0) or low risk (score = 1) of bias at the Rob2; the sum score for each RCT per compound with ≥2 studies was then regressed against the primary outcomes effect size of each compound-based RCT.

## Results

### Study characteristics

We identified 25,559 records across various sources and, after removing duplicates, screened 24,254 records at the title and abstract level. We retrieved 613 full texts.

**Fig. 1:**
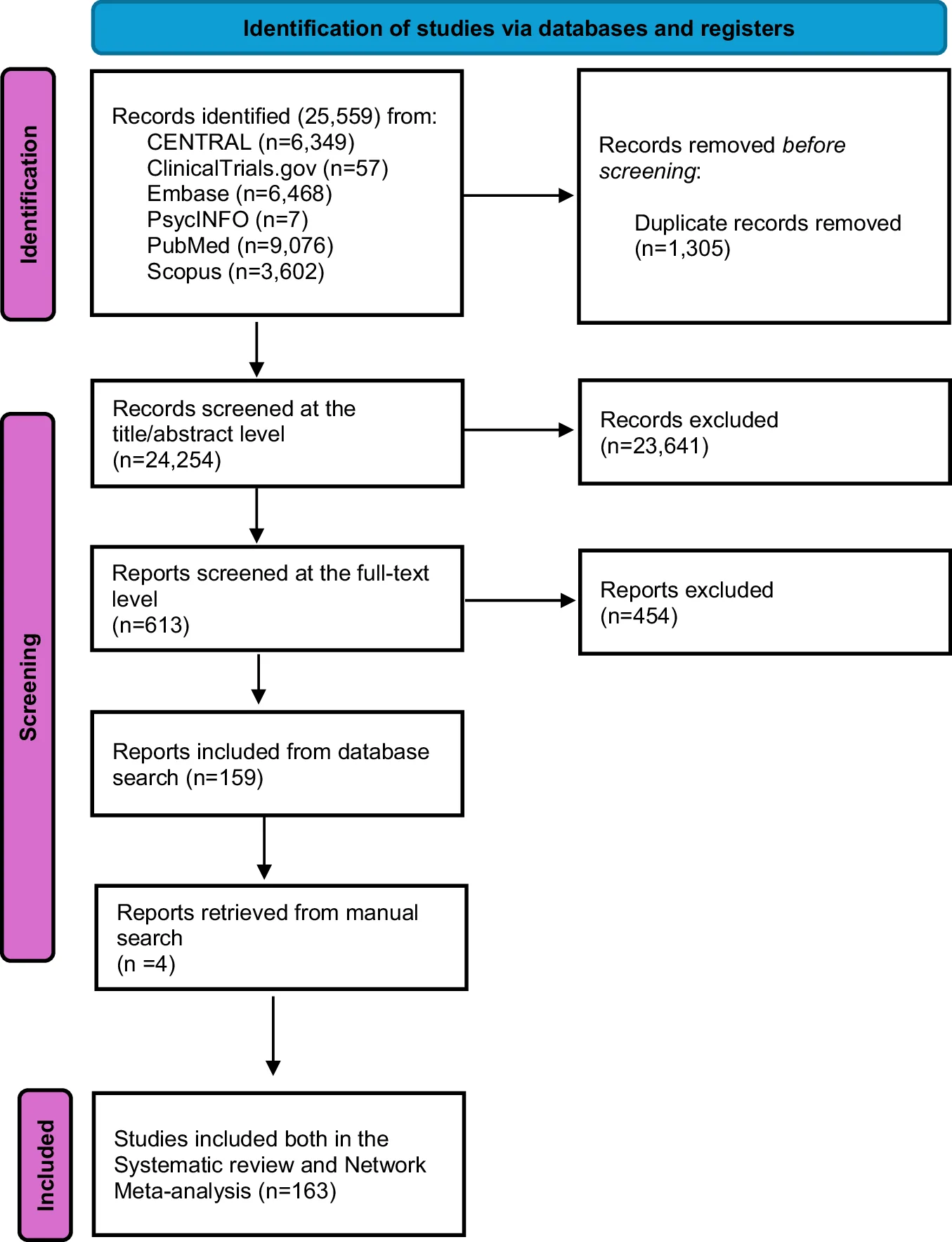
The PRISMA 2020 flow diagram for new systematic reviews includes database, register, and other source searches (adapted). **Note for** Fig. 1: BMJ 2021; 372 https://doi.org/10.1136/bmj.n71 (Published 29 March 2021). This work is licensed under CC BY 4.0. To view a copy of this license, visit https://creativecommons.org/licenses/by/4.0/.

The review included 163 studies. Details on the study flow and the adopted search strategy are provided in the PRISMA diagram (Fig. 1) and in S1. The characteristics of the included studies are detailed in Table 2. Excluded studies, with reason(s), are detailed in S2. The legend of the appraised compounds appears in S4.

**Table 2:** Characteristics of the included studies.

Author(s), year	Treatment	Mean dose	Country	ITT or PP	Sample size per arm	n of female per arm (%)	Mean age (SD)	Diagnosis	Multiple/single site	Treatment resistant depression	Diagnostic criteria	(semi)structured interview	Comorbidities	Concurrent psychiatric treatment (mean dose)	Setting	Length of the trial (weeks)	Sponsor	Outcome(s) assessment	Response definition	Remission definition	RoB2
(Açik, Çakiroğlu et al. 2025) [106]	FOIL	2 × 1000 mg/day	Turkey	PP	19 randomized, 16 analyzed	10 (58.8)	33.3 (12.9)	MDD	single center	no	DSM-5		no comorbidities	no	outpatients	8	no sponsor	HAM-D, DASS-21	no response outcome	no remission outcome	Some concerns
(Açik, Çakiroğlu et al. 2025) [106]	KOIL	2 × 1000 mg/day	Turkey	PP	19 randomized, 17 analyzed	13 (76.5)	39.7 (11.6)	MDD	single center	no	DSM-5		no comorbidities	no	outpatients	8	no sponsor	HAM-D, DASS-21	no response outcome	no remission outcome	Some concerns
(Açik, Çakiroğlu et al. 2025) [106]	PLB		Turkey	PP	19 randomized, 17 analyzed	13 (81.2)	35.1 (13.3)	MDD	single center	no	DSM-5		no comorbidities	no	outpatients	8	no sponsor	HAM-D, DASS-21	no response outcome	no remission outcome	Some concerns
(Ahmadpanah, Ramezanshams et al. 2019) [54]	SAFF	60 mg/day	Iran	ITT	25	6 (24)	64.48 (2.63)	MDD	single center	no	DSM-5	NA	no comorbidities	no	outpatients	6	no sponsor	HAM-D-21 item, response rate, remission rate	improvement of 50% or higher of HDRS scores	HDRS scores of 8 points or lower	Low
(Ahmadpanah, Ramezanshams et al. 2019) [54]	SER	100 mg/day	Iran	ITT	25	9 (36)	66.72 (5.34)	MDD	single center	no	DSM-5	NA	no comorbidities	no	outpatients	6	no sponsor	HAM-D-21 item, response rate, remission rate	improvement of 50% or higher of HDRS scores	HDRS scores of 8 points or lower	Low
(Akhondzadeh Basti, Moshiri et al. 2007) [107]	SAFF	30 mg/day	Iran	ITT	20	10 (50)	35.55 (8.05)	MDD	single center	no	DSM-IV	SCID for DSM-IV	no comorbidities	no	outpatients	8	Tehran University of Medical Sciences	HAM-D-17 item, response rate, remission rate	improvement of 50% or higher of HDRS scores	HAM-D total score of ≤7	Some concerns
(Akhondzadeh Basti, Moshiri et al. 2007) [107]	FLUO	20 mg/day	Iran	ITT	20	11 (55)	34.09 (5.5)	MDD	single center	no	DSM-IV	SCID for DSM-IV	no comorbidities	no	outpatients	8	Tehran University of Medical Sciences	HAM-D-17 item, response rate, remission rate	improvement of 50% or higher of HDRS scores	HAM-D total score of ≤7	Some concerns
(Akhondzadeh, Fallah-Pour et al. 2004) [55]	IMI	100 mg/day	Iran	ITT	15	8 (53)	32.53 (8.10)	MDD	single center	no	DSM-IV	SCID-IV	no comorbidities	no	outpatients	6	Iranian National Research Center of Medical Sciences and Red Crescent		no response outcome	no remission outcome	High
(Akhondzadeh, Fallah-Pour et al. 2004) [55]	SAFF	30 mg/day	Iran	ITT	15	9 (60)	35.3 (10.28)	MDD	single center	no	DSM-IV	SCID-IV	no comorbidities	no	outpatients	6	Iranian National Research Center of Medical Sciences and Red Crescent		no response outcome	no remission outcome	High
(Akhondzadeh, Mostafavi et al. 2020) [56]	SAF	15 mg 2/day	Iran	ITT	36	36 (100)	37 (10.3)	MDD	single center	no	DSM-5	NA	overweight (BMI > 25)	no	outpatients	12	NA	BDI-I	no response outcome	no remission outcome	Some concerns
(Akhondzadeh, Mostafavi et al. 2020) [56]	PLB	2/day	Iran	ITT	37	37 (100)	39.8 (9.2)	MDD	single center	no	DSM-5	NA	overweight (BMI > 25)	no	outpatients	12	NA	BDI-I	no response outcome	no remission outcome	Some concerns
(Akhondzadeh, Mostafavi et al. 2022) [108]	MEL	3 mg/day	Iran	PP	26 randomized, 21 analyzed	NA	35.3 (10.6)	MDD (mild to moderate)	single center	no	DSM-5	SCID	overweight (BMI > 25), sleep-wake disorders	no	outpatients	12	Tehran University of Medical Sciences	BDI-II	no response outcome	no remission outcome	Some concerns
(Akhondzadeh, Mostafavi et al. 2022) [108]	PLB		Iran	PP	26 randomized, 22 analyzed	NA	38.5 (8.7)	MDD (mild to moderate)	single center	no	DSM-5	SCID	overweight (BMI > 25), sleep-wake disorders	no	outpatients	12	Tehran University of Medical Sciences	BDI-II	no response outcome	no remission outcome	Some concerns
(Akhondzadeh, Kashani et al. 2003) [109]	LAV	60 drops/day	Iran	PP	16 randomized, 15 analyzed	7 (46.7)	33.53 (8.81)	MDD (mild to moderate)	single center	no	DSM-IV	SCID for DSM-IV	no comorbidities	no	outpatients	4	Ministry of Health and Medical Education of Iran	HAM-D-17 item	no response outcome	no remission outcome	Some concerns
(Akhondzadeh, Kashani et al. 2003) [109]	IMI	100 mg/day	Iran	PP	16 randomized, 15 analyzed	8 (53.3)	33.93 (11.38)	MDD (mild to moderate)	single center	no	DSM-IV	SCID for DSM-IV	no comorbidities	no	outpatients	4	Ministry of Health and Medical Education of Iran	HAM-D-17 item	no response outcome	no remission outcome	Some concerns
(Akhondzadeh, Kashani et al. 2003) [109]	LAV+IMI	60 drops/day+100 mg/day	Iran	PP	16 randomized, 15 analyzed	6 (40)	31.53 (10.22)	MDD (mild to moderate)	single center	no	DSM-IV	SCID for DSM-IV	no comorbidities	no	outpatients	4	Ministry of Health and Medical Education of Iran	HAM-D-17 item	no response outcome	no remission outcome	Some concerns
(Akkasheh, Kashani-Poor et al. 2016) [110]	PROB	1 cp/day	Iran	ITT	20	17 (85)	38.3 (12.1)	MDD	single center	no	DSM-IV	NA	no comorbidities	no	outpatients	8	Kashan University of Medical Sciences	BDI	no response outcome	no remission outcome	Low
(Akkasheh, Kashani-Poor et al. 2016) [110]	PLB		Iran	ITT	20	17 (85)	36.2 (8.2)	MDD	single center	no	DSM-IV	NA	no comorbidities	no	outpatients	8	Kashan University of Medical Sciences	BDI	no response outcome	no remission outcome	Low
(Almeida, Ford et al. 2014) [111]	FOL + vitB12 + vitB6 + CTP	0.5 mg+25 mg+2 mg+20–40 mg	New Zealand	ITT	77	38 (49.3)	63.4 (7.4)	MDD	single center	no	DSM-IV-TR	MINI	no comorbidities	no	outpatients	52	National Health and Medical Research Council of Australia	MADRS, response rate, remission rate	≥50% reduction in MADRS score at week 12	remission according to DSM-5 criteria at week 52	Low
(Almeida, Ford et al. 2014) [111]	CTP + PLB	20–40 mg	New Zealand	ITT	76	48 (63.2)	61.7 (8.2)	MDD	single center	no	DSM-IV-TR	MINI	no comorbidities	no	outpatients	52	National Health and Medical Research Council of Australia	MADRS, response rate, remission rate	≥50% reduction in MADRS score at week 12	remission according to DSM-5 criteria at week 52	Low
(Amini, Amani et al. 2022) [112]	vitD3-Ca	50000 IU+500 mg/day	Iran	PP	26	26 (100)	26.88 (0.97)	Post-partum depression (PPD)	single center	no	DSM-IV	NA	no comorbidities	no	outpatients	8	Vice-Chancellor for Research Affairs of Ahvaz Jundishapur University of Medical Sciences	Edinburg Postpartum Depression Scale (EPDS)	no response outcome	no remission outcome	Some concerns
(Amini, Amani et al. 2022) [112]	vitD3	50000 IU/day	Iran	PP	26	26 (100)	29.25 (1.43)	Post-partum depression (PPD)	single center	no	DSM-IV	NA	no comorbidities	no	outpatients	8	Vice-Chancellor for Research Affairs of Ahvaz Jundishapur University of Medical Sciences	Edinburg Postpartum Depression Scale (EPDS)	no response outcome	no remission outcome	Some concerns
(Amini, Amani et al. 2022) [112]	PLB	NA	Iran	PP	24	24 (100)	28.92 (1.62)	Post-partum depression (PPD)	single center	no	DSM-IV	NA	no comorbidities	no	outpatients	8	Vice-Chancellor for Research Affairs of Ahvaz Jundishapur University of Medical Sciences	Edinburg Postpartum Depression Scale (EPDS)	no response outcome	no remission outcome	Some concerns
(Anushiravani, Manteghi et al. 2019) [113]	EACS	30 ml	Iran	PP	25 randomized, 21 analyzed	12 (58)	NA	MDD	single center	no	DSM-5	NA	no comorbidities	no	outpatients	8	NA	HAM-D-17 item	no response outcome	no remission outcome	High
(Anushiravani, Manteghi et al. 2019) [113]	CTP	20 mg/day	Iran	PP	25 randomized, 21 analyzed	9 (42)	NA	MDD	single center	no	DSM-5	NA	no comorbidities	no	outpatients	8	NA	HAM-D-17 item	no response outcome	no remission outcome	High
(Araj-Khodaei, Noorbala et al. 2020) [114]	MEL	2 g 1/day	Iran	PP	16	10 (66.7)	37.4 (3.3)	mild to moderate depression	single center	no	DSM-5	NA	no comorbidities	no	outpatients	8	Deputy of Research of Shahed University, Tehran	HAM-D	no response outcome	no remission outcome	High
(Araj-Khodaei, Noorbala et al. 2020) [114]	LAV	2 g 1/day	Iran	PP	17	11 (73.3)	37.9 (2.4)	mild to moderate depression	single center	no	DSM-5	NA	no comorbidities	no	outpatients	8	Deputy of Research of Shahed University, Tehran	HAM-D	no response outcome	no remission outcome	High
(Araj-Khodaei, Noorbala et al. 2020) [114]	FLUO	20 mg 1/day	Iran	PP	17	11 (73.3)	33.4 (2.7)	mild to moderate depression	single center	no	DSM-5	NA	no comorbidities	no	outpatients	8	Deputy of Research of Shahed University, Tehran	HAM-D	no response outcome	no remission outcome	High
(Baião, Capitão et al. 2023) [115]	BIOKULT	4 cp/day	UK	ITT	35	21 (60)	27.94 (6.99)	MDD	single center	no	DSM-IV	SCID	no comorbidities	no	outpatients	4	ADM Protexin Ltd	PHQ-9	no response outcome	no remission outcome	High
(Baião, Capitão et al. 2023) [115]	PLB		UK	ITT	36	23 (64)	29.64 (10.52)	MDD	single center	no	DSM-IV	SCID	no comorbidities	no	outpatients	4	ADM Protexin Ltd	PHQ-9	no response outcome	no remission outcome	High
(Basoglu, Ates et al. 2009) [116]	ESC + FOL	10 mg+2.5 mg	Turkey	ITT	20	11 (55)	29	MDD	single center	no	DSM IV	SCID for DSM-IV	no comorbidities	no	outpatients	6	NA	MADRS, response rate, CGI-S	at least a 50% reduction in baseline MADRS total score	no remission outcome	Low
(Basoglu, Ates et al. 2009) [116]	ESC	10 mg	Turkey	ITT	15	8 (53.3)	30	MDD	single center	no	DSM IV	SCID for DSM-IV	no comorbidities	no	outpatients	6	NA	MADRS, response rate, CGI-S	at least a 50% reduction in baseline MADRS total score	no remission outcome	Low
(Behnke, Jensen et al. 2002) [117]	SJW	300 mg/day	Denmark	ITT	35	24 (68.57)	51.4 (10.9)	MDD	multi-center	no	ICD-10	NA	no comorbidities	no	outpatients	6	NA	HAM-D-17 item, response rate	≥ 50 % reduction in the HAM-D score	no remission outcome	Low
(Behnke, Jensen et al. 2002) [117]	FLUO	40 mg/day	Denmark	ITT	35	23 (65.71)	48 (12.6)	MDD	multi-center	no	ICD-10	NA	no comorbidities	no	outpatients	6	NA	HAM-D-17 item, response rate	≥ 50 % reduction in the HAM-D score	no remission outcome	Low
(Bell, Plon et al. 1988) [67]	SAMe	200–400 mg/day	USA	iTT	9	NA	NA	MDD	single center	no	DSM-III		no comorbidities	no	outpatients	2	John D. and Catherine T. MacArthur Foundation	HAM-D, response rate	a reduction of more than 50%	no remission outcome	High
(Bell, Plon et al. 1988) [67]	IMI	150 mg/day	USA	ITT	9	NA	NA	MDD	single center	no	DSM-III		no comorbidities	no	outpatients	2	John D. and Catherine T. MacArthur Foundation	HAM-D, response rate	a reduction of more than 50%	no remission outcome	High
(Bergman, Miodownik et al. 2013) [118]	CUR	500 mg/day	Israel	ITT	20	9 (45)	65.8 (10.7)	MDD	single center	no	DSM-IV	NA	no comorbidities	venlafaxine or escitalopram (107.3 mg/day; 11.4 mg/day)	inpatients	5	no sponsor	MADRS, HAM-D-17 item, CGI-S	no response outcome	no remission outcome	Low
(Bergman, Miodownik et al. 2013) [118]	PLB		Israel	ITT	20	14 (70)	61.3 (15.2)	MDD	single center	no	DSM-IV	NA	no comorbidities	venlafaxine or escitalopram (107.3 mg/day; 11.4 mg/day)	inpatients	5	no sponsor	MADRS, HAM-D-17 item, CGI-S	no response outcome	no remission outcome	Low
(Berk, Dean et al. 2014) [119]	NAC + TAU	2000 mg/day	Australia	ITT	127	84 (66.1)	49.9 (13)	MDD	single center	no	DSM-IV-TR	MINI plus	no comorbidities	46 no psychotropic medication; the remaining: antidepressants, BDZ, antipsychotic, mood stabilizers	Inpatients	12	National Health and Medical research Council and Australian Rotary Health, NHMRC Senior Principal Research Fellowship	MADRS, HAM-A, SOFAS, GAF, LIFE-RIFT, SLICE-LIFE, Q-LES-Q, response rate, remission rate	decrease of 50% or more in MADRS at week 16	MADRS score of ≤7	Low
(Berk, Dean et al. 2014) [119]	PLB + TAU	NA	Australia	ITT	125	75 (60)	50.5 (12.5)	MDD	single center	no	DSM-IV-TR	MINI plus	no comorbidities	33 no psychotropic medication; the remaining: antidepressants, BDZ, antipsychotic, mood stabilizers	Inpatients	12	National Health and Medical research Council and Australian Rotary Health, NHMRC Senior Principal Research Fellowship	MADRS, HAM-A, SOFAS, GAF, LIFE-RIFT, SLICE-LIFE, Q-LES-Q, response rate, remission rate	decrease of 50% or more in MADRS at week 16	MADRS score of ≤7	Low
(Bidzan-Wiącek, Tomczyk et al. 2024) [32]	EPA + DHA	4000 mg/day	Poland	ITT	25	NA	36.04 (5.43)	MDD	single center	no	DSM-5		no comorbidities	no	outpatients	12	Narodowe Centrum Nauki	DASS-21	no response outcome	no remission outcome	Low
(Bidzan-Wiącek, Tomczyk et al. 2024) [32]	PLB		Poland	ITT	22	NA	36.79 (7.17)	MDD	single center	no	DSM-5		no comorbidities	no	outpatients	12	Narodowe Centrum Nauki	DASS-21	no response outcome	no remission outcome	Low
(Bjerkenstedt, Edman et al. 2005) [79]	LI-160	900 mg/day	Sweden	ITT	59 randomized, 54 analyzed	43 (79.62)	49.1 (12)	MDD	multi-center	no	DSM-IV	NA	no comorbidities	no	outpatients	4	Lichtwer Pharma GmbH, Berlin, Germany	HAMD-21	HAM-D reduction >50%	HAM-D < 8	High
(Bjerkenstedt, Edman et al. 2005) [79]	FLUO	20 mg/day	Sweden	ITT	57 randomized, 54 analyzed	41 (75.92)	50.4 (11.6)	MDD	multi-center	no	DSM-IV	NA	no comorbidities	no	outpatients	4	Lichtwer Pharma GmbH, Berlin, Germany	HAMD-21	HAM-D reduction >50%	HAM-D < 8	High
(Bjerkenstedt, Edman et al. 2005) [79]	PLB	NA	Sweden	ITT	57 randomized, 55 analyzed	45 (81.8)	51.4 (11.8)	MDD	multi-center	no	DSM-IV	NA	no comorbidities	no	outpatients	4	Lichtwer Pharma GmbH,Berlin, Germany	HAMD-21	HAM-D reduction >50%	HAM-D < 8	High
(Bot, Pouwer et al. 2010) [33]	EPA	1 g/day	Netherlands	ITT	13	8 (62)	53.1 (13.8)	MDD	single center	no	DSM-IV	CIDI	5DM1, 8DM2	SSRIs, TCAs, Noradrenergic and specific serotoninergic antidepressant	outpatients	12	Dutch Diabetes Research Foundation, Minami Nutrition, Belgium	MADRS	no response outcome	no remission outcome	Low
(Bot, Pouwer et al. 2010) [33]	PLB		Netherlands	ITT	12	5 (42)	55 (8.6)	MDD	single center	no	DSM-IV	CIDI	5DM1, 7DM2	SSRIs, TCAs, Noradrenergic and specific serotoninergic antidepressant	outpatients	12	Dutch Diabetes Research Foundation, Minami Nutrition, Belgium	MADRS	no response outcome	no remission outcome	Low
(Brenner, Azbel et al. 2000) [80]	LI-160	900 mg/day	USA	ITT	15	10 (66.7)	44.2 (12.9)	MDD	single center	no	DSM-IV	NA	no comorbidities	no	outpatients	6	Lichtwer Pharma AG, Berlin, Germany	HAM-D-17 item, CGI	≥ 50 % reduction in the HAM-D score	no remission outcome	Low
(Brenner, Azbel et al. 2000) [80]	SER	75 mg/day	USA	ITT	15	9 (60)	46.9 (12.3)	MDD	single center	no	DSM-IV	NA	no comorbidities	no	outpatients	6	Lichtwer Pharma AG, Berlin, Germany	HAM-D-17 item, CGI	≥ 50 % reduction in the HAM-D score	no remission outcome	Low
(Carney, Freedland et al. 2019) [34]	SER + EPA	50 mg/day + 2 g/day	USA	ITT	71	26 (36.6)	58.5 (9.6)	MDD	single center	no	DSM-5	NA	coronary heart disease	no	outpatients	10	NA	BDI-II, HAM-D-17, response rate, BAI scores, remission rate	>50% reduction from baseline BDI-II scores	BDI-II<8	Low
(Carney, Freedland et al. 2019) [34]	SER + PLB	50 mg/day	USA	ITT	73	30 (41.1)	60.5 (9.3)	MDD	single center	no	DSM-5	NA	coronary heart disease	no	outpatients	10	NA	BDI-II, HAM-D-17, response rate, BAI scores, remission rate	>50% reduction from baseline BDI-II scores	BDI-II<8	Low
(Carney, Steinmeyer et al. 2016) [35]	SER + EPA + DHA	50 mg+930 mg+750 mg/day	USA	ITT	62	NA	NA	MDD	single center	no	DSM-IV		CHD defined as ≥50% stenosis in ≥1 major coronary artery, a history of revascularization, or hospitalization for an acute coronary syndrome (ACS)	no	outpatients		National Heart, Lung, and Blood Institute, Glaxo-Smith-Kline, Inc., Pfizer, Inc.		no response outcome	HAM-D-17 <7	Low
(Carney, Steinmeyer et al. 2016) [35]	SER + PLB	50 mg/day	USA	ITT	60	NA	NA	MDD	single center	no	DSM-IV		CHD defined as ≥50% stenosis in ≥1 major coronary artery, a history of revascularization, or hospitalization for an acute coronary syndrome (ACS)	no	outpatients		National Heart, Lung, and Blood Institute, Glaxo-Smith-Kline, Inc., Pfizer, Inc.		no response outcome	HAM-D-17 <7	Low
(Caruso, Fumagalli et al. 1987) [68]	SAMe	200 mg/day	Italy	PP	30	25 (83.3)	50.2	MDD	single center	no	DSM-III		rheumatoid arthritic	no	outpatients	3	NA	HAM-D	no response outcome	no remission outcome	Some concerns
(Caruso, Fumagalli et al. 1987) [68]	PLB		Italy	PP	30 randomized, 29 analyzed	26 (86.7)	55.2	MDD	single center	no	DSM-III		rheumatoid arthritic	no	outpatients	3	NA	HAM-D	no response outcome	no remission outcome	Some concerns
(Chang, Chang et al. 2020) [120]	OMEGA3	2 g EPA+1 g DHA/day	China	ITT	30	12 (40)	61.10 (9.14)	MDD	single center	no	DSM-IV or ICD-10	MINI	cardiovascular diseases	no	outpatients	12	Ministry of Science and Technology, Taiwan; the National Health Research Institutes, Taiwan; and Chinese Medicine Research Center from the China Medical University, Taiwan.	HAMD-21, BDI-II	no response outcome	no remission outcome	Low
(Chang, Chang et al. 2020) [120]	PLB		China	ITT	29	9 (31)	61.93 (8.95)	MDD	single center	no	DSM-IV or ICD-10	MINI	cardiovascular diseases	no	outpatients	12	Ministry of Science and Technology, Taiwan; the National Health Research Institutes, Taiwan; and Chinese Medicine Research Center from the China Medical University, Taiwan.	HAMD-21, BDI-II	no response outcome	no remission outcome	Low
(Cheng, Chen et al. 2025) [36]	EPA + DHA	1.2 g+1 g/day	Taiwan	ITT	19	15 (78.94)	68.36 (6.27)	MDD (late life depression)	single center	no	DSM-5		no comorbidities	no	outpatients	52	Taipei city government	HAM-D, HAM-A	no response outcome	no remission outcome	Low
(Cheng, Chen et al. 2025) [36]	PLB		Taiwan	ITT	20	17 (85)	69.36 (6.27)	MDD (late life depression)	single center	no	DSM-5		no comorbidities	no	outpatients	52	Taipei city government	HAM-D, HAM-A	no response outcome	no remission outcome	Low
(Chopra and Kalyani 2020) [57]	SAFF	2 cp/day	India	ITT	20	8 (20)	41.9	MDD	single center	no	DSM-IV-TR		CV diseases	no	outpatients	6	NA	HAM-D	no response outcome	no remission outcome	High
(Chopra and Kalyani 2020) [57]	FLUO		India	ITT	20	6 (20)	44.7	MDD	single center	no	DSM-IV-TR		CV diseases	no	outpatients	6	NA	HAM-D	no response outcome	no remission outcome	High
(Coppen and Bailey 2000) [121]	FOL + FLUO	500 mcg/day+20 mg/day	UK	ITT	62	33 (68)	41.9 (12)	MDD	multi-center	no	DSM-III-R	NA	no comorbidities	no	outpatients	10	Scotia Pharmaceuticals	HAM-D-17-item	improvement > 50% of HDRS scores	recovered patients were defined as those who scored ≤9 on the HDRS at completition	Low
(Coppen and Bailey 2000) [121]	PLB + FLUO	20 mg/day	UK	ITT	65	36 (62)	44.3 (14.6)	MDD	multi-center	no	DSM-III-R	NA	no comorbidities	no	outpatients	10	Scotia Pharmaceuticals	HAM-D-17-item	improvement > 50% of HDRS scores	recovered patients were defined as those who scored ≤9 on the HDRS at completition	Low
(Darbinyan, Aslanyan et al. 2007) [122]	RHO	340 or 680 mg/day	Armenia	ITT	60	38 (63.3)	44.75 (18.25)	MDD	single center	no	DSM-IV	NA	no comorbidities	no	inpatients and outpatients	6	Swedish Herbal Institute	HAM-D-21 item, BDI-13 item	no response outcome	no remission outcome	Some concerns
(Darbinyan, Aslanyan et al. 2007) [122]	PLB	NA	Armenia	ITT	29	15 (51.7)	42.8 (12.87)	MDD	single center	no	DSM-IV	NA	no comorbidities	no	inpatients and outpatients	6	Swedish Herbal Institute	HAM-D-21 item, BDI-13 item	no response outcome	no remission outcome	Some concerns
(Davidson, Abraham et al. 2003) [123]	CHRO	600 mcg	USA	ITT	10	7 (70)	46.1 (10.5)	MDD, atypical features	single center	no	DSM-IV	MINI	no comorbidities	no	outpatients	8	Nutrition 21		a drop of at least 66% on the HAM-D from baseline long with very much improve- men at the CGI-I	remission outcome HAM-D of 8	Low
(Davidson, Abraham et al. 2003) [123]	PLB		USA	ITT	5	5 (100)	47.0 (12.8)	MDD, atypical features	single center	no	DSM-IV	MINI	no comorbidities	no	outpatients	8	Nutrition 22		a drop of at least 66% on the HAM-D from baseline long with very much improvement on the CGI-I	remission outcome HAM-D of 8	Low
(Davidson, Gadde et al. 2002) [81]	LI-160	900–1500 mg/day	USA	ITT	113	73 (64.5)	43.1 (13.5)	MDD	single center	no	DSM IV	SCID	no comorbidities	zolpidem (5–10 mg 2/week)	outpatients	8	National Center for Complementary and Alternative Medicine and the National Institute of Mental Health to Duke University Medical Center, Lichtwer Pharma and Pfizer.	HAM-D, BDI, CGI-S	no response outcome	no remission outcome	Low
(Davidson, Gadde et al. 2002) [81]	SER	50–100 mg/day	USA	ITT	111	74 (66.7)	43.9 (13.9)	MDD	single center	no	DSM IV	SCID	no comorbidities	zolpidem (5–10 mg 2/week)	outpatients	8	National Center for Complementary and Alternative Medicine and the National Institute of Mental Health to Duke University Medical Center, Lichtwer Pharma and Pfizer.	HAM-D, BDI, CGI-S	no response outcome	no remission outcome	Low
(Davidson, Gadde et al. 2002) [81]	PLB		USA	ITT	116	77 (66.4)	40.1 (12.2)	MDD	single center	no	DSM IV	SCID	no comorbidities	Zolpidem (5–10 mg 2/week)	outpatients	8	National Center for Complementary and Alternative Medicine and the National Institute of Mental Health to Duke University Medical Center, Lichtwer Pharma and Pfizer.	HAM-D, BDI, CGI-S	no response outcome	no remission outcome	Low
(De Leo 1987) [69]	SAMe	200 mg/day	Italy	PP	20	9 (45)	44.55 (13.16)	MDD	single center	no	DSM-III		no comorbidities	no	outpatients	4	NA	CGI-S	no response outcome	no remission outcome	Some concerns
(De Leo 1987) [69]	PLB		Italy	PP	20	13 (65)	47.50 (13.70)	MDD	single center	no	DSM-III		no comorbidities	no	outpatients	4	NA	CGI-S	no response outcome	no remission outcome	Some concerns
(Djokic, Korcok et al. 2017) [124]	TEN	1 cp/day	Serbia	ITT	30	19 (63.3)	49.63 (15.57)	MDD	single center	no	ICD-10	MINI	no comorbidities	no	outpatients	12	no sponsor	HAM-D, CGI_S	no response outcome	no remission outcome	Low
(Djokic, Korcok et al. 2017) [124]	PLB		Serbia	ITT	30	15 (50)	51.1 (14.0)	MDD	single center	no	ICD-10	MINI	no comorbidities	no	outpatients	12	no sponsor	HAM-D, CGI_S	no response outcome	no remission outcome	Low
(Docherty, Sack et al. 2005) [125]	CHRO	600 mcg/day	USA	ITT	70	54 (77)	46.4 (11.7)	MDD, atypical type	multi-center	no	DSM-IV	MINI	no comorbidities	no	outpatients	8	Nutrition 21, Inc., Purchase, NY	HAM-D-29 item, response rate, remission rate	reduction of at least 50% in HAM-D-29 scores and a CGI-I scores of 1 or 2	no remission outcome	Low
(Docherty, Sack et al. 2005) [125]	PLB	NA	USA	ITT	40	24 (60)	45.5 (10.2)	MDD, atypical type	multi-center	no	DSM-IV	MINI	no comorbidities	no	outpatients	8	Nutrition 21, Inc., Purchase, NY	HAM-D-29 item, response rate, remission rate	reduction of at least 50% in HAM-D-29 scores and a CGI-I scores of 1 or 2	no remission outcome	Low
(Dormal, Suchareau et al. 2025) [126]	SCU	20 mg 1/day	Belgium	ITT	42	31 (73.8)	38.3 (14.2)	MDD	single center	no	DSM-5		no comorbidities	no	outpatients	6	Comercial Quimica Massó	HAM-D, BDI	no response outcome	no remission outcome	Low
(Dormal, Suchareau et al. 2025) [126]	SAFF + SCU	20+30 mg/day	Belgium	ITT	48	35 (72.9)	33.3 (14.7)	MDD	single center	no	DSM-5		no comorbidities	no	outpatients	6	Comercial Quimica Massó	HAM-D, BDI	no response outcome	no remission outcome	Low
(Dormal, Suchareau et al. 2025) [126]	SAFF	30 mg/day	Belgium	ITT	43	30 (69.8)	34.0 (15.2)	MDD	single center	no	DSM-5		no comorbidities	no	outpatients	6	Comercial Quimica Massó	HAM-D, BDI	no response outcome	no remission outcome	Low
(Dormal, Suchareau et al. 2025) [126]	PLB		Belgium	ITT	47	33 (70.2)	35.6 (15.9)	MDD	single center	no	DSM-5		no comorbidities	no	outpatients	6	Comercial Quimica Massó	HAM-D, BDI	no response outcome	no remission outcome	Low
(Fava, Alpert et al. 2005) [82]	LI-160	900 mg/day	USA	ITT	45	24 (53)	37.4 (11.7)	MDD	single center	no	DSM-IV	SCID for DSM-IV	no comorbidities	no	outpatients	12	Lichtwer Pharma AG (Berlin, Germany)	HAM-D-17 item, BDI, CGI-S, remission rate	no response outcome	HAM-D score < 8	Low
(Fava, Alpert et al. 2005) [82]	FLUO	20 mg/day	USA	ITT	47	25 (53)	36.7 (9.6)	MDD	single center	no	DSM-IV	SCID for DSM-IV	no comorbidities	no	outpatients	12	Lichtwer Pharma AG (Berlin, Germany)	HAM-D-17 item, BDI, CGI-S, remission rate	no response outcome	HAM-D score < 8	Low
(Fava, Alpert et al. 2005) [82]	PLB	NA	USA	ITT	43	28 (65)	37.8 (12)	MDD	single center	no	DSM-IV	SCID for DSM-IV	no comorbidities	no	outpatients	12	Lichtwer Pharma AG (Berlin, Germany)	HAM-D-17 item, BDI, CGI-S, remission rate	no response outcome	HAM-D score < 8	Low
(Firoozabadi, Zarshenas et al. 2015) [127]	NEP + TAU	400 mg/day	Iran	ITT	15	NA	NA	MDD	single center	no	DSM-IV	NA	no comorbidities	combination of TCA and SSRI	outpatients	8	no sponsor	HAM-D, BDI	no response outcome	no remission outcome	Low
(Firoozabadi, Zarshenas et al. 2015) [127]	CUS + TAU	500 mg/day	Iran	ITT	13	NA	NA	MDD	single center	no	DSM-IV	NA	no comorbidities	combination of TCA and SSRI	outpatients	8	no sponsor	HAM-D, BDI	no response outcome	no remission outcome	Low
(Firoozabadi, Zarshenas et al. 2015) [127]	TAU	NA	Iran	ITT	15	NA	NA	MDD	single center	no	DSM-IV	NA	no comorbidities	combination of TCA and SSRI	outpatients	8	no sponsor	HAM-D, BDI	no response outcome	no remission outcome	Low
(Firoozeei, Barekatain et al. 2020) [128]	LAV + PLB	5 ml/day	Iran	PP	28 randomized, 25 analyzed	20 (80)	36.1 (9.2)	MDD with anxious distress	single center	no	DSM-5	SCID	no comorbidities	no	outpatients	6	Tehran University of Medical Sciences	HAM-D-17 item, HAM-A-14 item, response rate, remission rate	HAM-D reduction of 50% or more from baseline	HAM-D total score of ≤7	Some concerns
(Firoozeei, Barekatain et al. 2020) [128]	CTP + PLB	20 mg/day	Iran	PP	28 randomized, 25 analyzed	20 (80)	38.6 (6.6)	MDD with anxious distress	single center	no	DSM-5	SCID	no comorbidities	no	outpatients	6	Tehran University of Medical Sciences	HAM-D-17 item, HAM-A-14 item, response rate, remission rate	HAM-D reduction of 50% or more from baseline	HAM-D total score of ≤7	Some concerns
(Freeman, Davis et al. 2008) [37]	EPA + DHA	1.9 g/day	USA	PP	31 randomized, 28 analyzed	31 (100)	31.0 (5.8)	MDD	single center	no	DSM IV	SCID	pregnant or within 6 months post-partum	no	outpatients	8	NA	HAM-D, EPDS	no response outcome	no remission outcome	Some concerns
(Freeman, Davis et al. 2008) [37]	PLB		USA	PP	28 randomized, 23 analyzed	28 (100)	29.7 (6.2)	MDD	single center	no	DSM IV	SCID	pregnant or within 6 months post-partum	no	outpatients	8	NA	HAM-D, EPDS	no response outcome	no remission outcome	Some concerns
(Friede, Henneicke von Zepelin et al. 2001) [129]	ZE117	500 mg/day	Germany	ITT	126	90 (71)	46 (19)	MDD (mild to moderate)	single center	no	ICD-10		no comorbidities	no	inpatients	6	NA	HAM-D, response rate	no response outcome	no remission outcome	High
(Friede, Henneicke von Zepelin et al. 2001) [129]	FLUO	20 mg/day	Germany	ITT	114	67 (59)	47 (17)	MDD (mild to moderate)	single center	no	ICD-10		no comorbidities	no	inpatients	6	NA	HAM-D, response rate	no response outcome	no remission outcome	High
(Gao, Wu et al. 2020) [130]	SER + PLB	NA	China	ITT	32	17 (53.1)	35 (13.2)	MDD	single center	no	DSM-IV	SCID for DSM-IV	no comorbidities	no	outpatients	12	Fujian Provincial Health Commission, National Natural Science Foundation of China and the Natural Science Foundation of the Fujian Province	HAM-D-17 item, BDI	no response outcome	no remission outcome	Low
(Gao, Wu et al. 2020) [130]	RHO + SER	0.3–0.6 g/day	China	ITT	66	36 (54.5)	38.5 (11.75)	MDD	single center	no	DSM-IV	SCID for DSM-IV	no comorbidities	no	outpatients	12	Fujian Provincial Health Commission, National Natural Science Foundation of China and the Natural Science Foundation of the Fujian Province	HAM-D-17 item, BDI	no response outcome	no remission outcome	Low
(Gelenberg, Shelton et al. 2004) [83]	LI-160	900–1200 mg/day	USA	ITT	43	26 (60.5)	40.8 (12.4)	MDD	multi-center	no	DSM-IV		no comorbidities	11 citalopram, 1 lorazepam,1 fluvoxamine, 1 nortriptyline, 11 paroxetine, 19 fluoxetine, 1 mirtazapine, 2 nefazodone, 2 trazodone, 4 venlafaxine, 7 bupropion, 1 zolpidem, 28 sertraline (28.2 mg, 1 mg, 12 mg, 75 mg, 20.9 mg, 25 mg, 15 mg, 200 mg, 25 mg, 49.7 mg, 150 mg, 5 mg, 92.9 mg)	outpatients	23	Pfizer Inc, New York	HAM-D, response rate	HAM-D <= 12 and CGI-I <= 2	no remission outcome	Low
(Gelenberg, Shelton et al. 2004) [83]	PLB		USA	ITT	55	40 (72.7)	42.3 (14.3)	MDD	multi-center	no	DSM-IV		no comorbidities	11 citalopram, 1 lorazepam,1 fluvoxamine, 1 nortriptyline, 11 paroxetine, 19 fluoxetine, 1 mirtazapine, 2 nefazodone, 2 trazodone, 4 venlafaxine, 7 bupropion, 1 zolpidem, 28 sertraline (28.2 mg, 1 mg, 12 mg, 75 mg, 20.9 mg, 25 mg, 15 mg, 200 mg, 25 mg, 49.7 mg, 150 mg, 5 mg, 92.9 mg)	outpatients	23	Pfizer Inc, New York	HAM-D, response rate	HAM-D <= 12 and CGI-I <= 2	no remission outcome	Low
(Ghaffari, Ghobadi et al. 2020) [131]	SER + PLB	100 mg2/day	Iran	PP	25 randomized, 19 analyzed	17 (89.5)	32.10 (10.98)	MDD	single center	no	DSM-5	NA	no comorbidities	no	outpatients	6	NA	HAM-D, remission rate, response rate	50%reductionintheHAM-Dscore	HAM-D score <= 7	Some concerns
(Ghaffari, Ghobadi et al. 2020) [131]	SER + CIN	100 mg2/day+500 mg/day	Iran	PP	25 randomized, 23 analyzed	18 (78.3)	37 (10.04)	MDD	single center	no	DSM-5	NA	no comorbidities	no	outpatients	6	NA	HAM-D, remission rate, response rate	50%reductionintheHAM-Dscore	HAM-D score <= 7	Some concerns
(Ghajar, Neishabouri et al. 2017) [58]	SAFF	30 mg/day	Iran	ITT	30	19 (63.3)	37.9 (11.56)	MDD with anxious distress	multi-center	no	DSM-5	NA	no comorbidities	no	outpatients	6	Tehran University of Medical Sciences	HAM-D-17 item; HAM-A-14 item	≥ 50 % reduction in the HAM-D score	HAM-D score ≤ 7	Low
(Ghajar, Neishabouri et al. 2017) [58]	CTP	40 mg/day	Iran	ITT	30	15 (50)	34.17 (10.41)	MDD with anxious distress	multi-center	no	DSM-5	NA	no comorbidities	no	outpatients	6	Tehran University of Medical Sciences	HAM-D-17 item; HAM-A-14 item	≥ 50 % reduction in the HAM-D score	HAM-D score ≤ 7	Low
(Ghaleiha, Davari et al. 2016) [132]	THI + FLUO	300 mg/day+20 mg/day	Iran	ITT	25	13 (52)	35.28 (9.9)	MDD	single center	no	DSM-5	NA	no comorbidities	no	Inpatients	12	no sponsor	HAM-D-21 item, response rate, remission rate	decrease of 50% or more in HAM-D score from baseline	HAM-D total score of ≤8	Low
(Ghaleiha, Davari et al. 2016) [132]	PLB + FLUO	20 mg/day	Iran	ITT	26	14 (53.8)	35.08 (8.35)	MDD	single center	no	DSM-5	NA	no comorbidities	no	inpatients	12	no sponsor	HAM-D-21 item, response rate, remission rate	decrease of 50% or more in HAM-D score from baseline	HAM-D total score of ≤8	Low
(Ghorbani, Nazari et al. 2018) [133]	SYN	1 cp/day	Iran	ITT	20	14 (70)	34.45 (3.95)	MDD	single center	no	DSM-5	SCID	no comorbidities	fluoxetine	outpatients	10	National nutrition and food technology research institute, Shahid Beheshti University of Medical Sciences	HAM-D	no response outcome	no remission outcome	Low
(Ghorbani, Nazari et al. 2018) [133]	PLB		Iran	ITT	20	14 (70)	35.50 (5.27)	MDD	single center	no	DSM-5	SCID	no comorbidities	fluoxetine	outpatients	10	National nutrition and food technology research institute, Shahid Beheshti University of Medical Sciences	HAM-D	no response outcome	no remission outcome	Low
(Grenyer, Crowe et al. 2007) [134]	OMEGA3 + TAU	3 g/day	Australia	ITT	40	NA	NA	MDD	single center	no	DSM-IV	SCID for DSM-IV	no comorbidities	33 out of 83 SSRIs, 4 out of 83 TCAs	outpatients	16	Clover Corporation in collaboration with the University of Wollongong, and Australian Research Council	BDI, HAM-D, GAF	no response outcome	no remission outcome	Some concerns
(Grenyer, Crowe et al. 2007) [134]	PLB + TAU	NA	Australia	ITT	43	NA	NA	MDD	single center	no	DSM-IV	SCID for DSM-IV	no comorbidities	33 out of 83 SSRIs, 4 out of 83 TCAs	outpatients	16	Clover Corporation in collaboration with the University of Wollongong, and by the Australian Research Council	BDI, HAM-D, GAF	no response outcome	no remission outcome	Some concerns
(Gul, Memtily et al. 2019) [135]	US	50 ml	China	PP	54 randomized, 50 analyzed	40 (80)	48.80 (13.66)	MDD + mixed anxiety	single center	no	ICD-10		no comorbidities	no	outpatients	6	Xinjiang Local Production New Drug Research & Development Project for Traditional Chinese Medicine and Ethnic Medicine		no response outcome	no remission outcome	High
(Gul, Memtily et al. 2019) [135]	FLUO	20 mg 1/day	China	PP	53 randomized, 50 analyzed	31 (62)	47.7 (13.43)	MDD + mixed anxiety	single center	no	ICD-10		no comorbidities	no	outpatients	6	Xinjiang Local Production New Drug Research & Development Project for Traditional Chinese Medicine and Ethnic Medicine		no response outcome	no remission outcome	High
(Gul, Memtily et al. 2019) [135]	FLUO + US	20 mg+50 ml/day	China	PP	55 randomized, 50 analyzed	32 (64)	45.66 (12.30)	MDD + mixed anxiety	single center	no	ICD-10		no comorbidities	no	outpatients	6	Xinjiang Local Production New Drug Research & Development Project for Traditional Chinese Medicine and Ethnic Medicine		no response outcome	no remission outcome	High
(Han, Chung et al. 2012) [136]	GGBT	4000 mg/day	Republic of Korea	ITT	82	71 (86.59)	47.85 (1.46)	MDD	single center	no	DSM IV		no comorbidities	no	outpatients	8	Oriental Medicine R&D Project, Ministry for Health & Welfare & Family Affairs, Republic of Korea	HAM-D	no response outcome	no remission outcome	Low
(Han, Chung et al. 2012) [136]	PLB		Republic of Korea	ITT	82	64 (78.05)	48.05 (1.37)	MDD	single center	no	DSM IV		no comorbidities	no	outpatients	8	Oriental Medicine R&D Project, Ministry for Health & Welfare & Family Affairs, Republic of Korea	HAM-D	no response outcome	no remission outcome	Low
(Harrer, Schmidt et al. 1999) [137]	LH57	800 mg/day	Germany	ITT	70	60 (85.71)	68.40 (0.6)	MDD	single center	no	ICD-10	NA	no comorbidities	no	outpatients	6	NA	HAM-D, response rate	HAMD score ≤ 10 points or score reduction of at least 50%	no remission outcome	Low
(Harrer, Schmidt et al. 1999) [137]	FLUO	20 mg/day	Germany	ITT	79	69 (87.34)	69.09 (9.9)	MDD	single center	no	ICD-10	NA	no comorbidities	no	outpatients	6	NA	HAM-D, response rate	HAMD score ≤ 10 points or score reduction of at least 50%	no remission outcome	Low
(He, Liu et al. 2024) [138]	GBDP	5 cp/3 day	China	ITT	34	14 (41.2)	59.79 (8.93)	MDD	multi-center	no	ICD-10		Coronary Heart Disease, Stable Angina Pectoris	no	outpatients	12	National Key Research and Development Plan, the National Natural Science Foundation of China, the National Clinical Research Base of Traditional Chinese Medicine, the second batch of the national Traditional Chinese Medicine (TCM) clinical research base project, the Research Project of Guangdong Administration of Traditional Chinese Medicine, the Natural Science Foundation of Guangdong Province, the Guangzhou Science and Technology Program Key Projects, and the Excellent Doctoral Dissertation Cultivation Project of the First Clinical School of Guangzhou University of Chinese Medicine.	HAM-D, response rate	reduction rate of the HAMD total score (a reduction rate of ≥50% was considered effective	no remission outcome	Low
(He, Liu et al. 2024) [138]	PLB		China	ITT	34	8 (23.5)	59.38 (10.27)	MDD	multi-center	no	ICD-10		Coronary Heart Disease, Stable Angina Pectoris	no	outpatients	12	National Key Research and Development Plan, the National Natural Science Foundation of China, the National Clinical Research Base of Traditional Chinese Medicine, the second batch of the national Traditional Chinese Medicine (TCM) clinical research base project, the Research Project of Guangdong Administration of Traditional Chinese Medicine, the Natural Science Foundation of Guangdong Province, the Guangzhou Science and Technology Program Key Projects, and the Excellent Doctoral Dissertation Cultivation Project of the First Clinical School of Guangzhou University of Chinese Medicine.	HAM-D, response rate	reduction rate of the HAMD total score (a reduction rate of ≥50% was considered effective	no remission outcome	Low
(Hosseini, Bagherniya et al. 2024) [139]	EA	200 mg	Iran	ITT	20	NA	54.18 (5.85)	MDD	single center	no	DSM-5	NA	no comorbidities	Fluoxetine (20–60 mg/day)	outpatients	8	no sponsor	HAM-D, BDI	no response outcome	no remission outcome	High
(Hosseini, Bagherniya et al. 2024) [139]	PLB		Iran	ITT	20	NA	53.29 (6.19)	MDD	single center	no	DSM-5	NA	no comorbidities	Fluoxetine (20–60 mg/day)	outpatients	8	no sponsor	HAM-D, BDI	no response outcome	no remission outcome	High
(Hu, Chen et al. 2021) [140]	FLUO + PLB	20 mg	China	ITT	66	44 (66.67)	46.03 (11.92)	MDD	multi-center	no	DSM-IV	NA	no comorbidities	no	outpatients	8	National Natural Science Foundation of China and the Project of the Ministry of Science and Technology of the People’s Republic of China	HAM-D-17 item, SDS, N-back evaluation	no response outcome	no remission outcome	Some concerns
(Hu, Chen et al. 2021) [140]	SZL + PLB	3.2 g/day	China	ITT	63	39 (61.90)	47.14 (14.29)	MDD	multi-center	no	DSM-IV	NA	no comorbidities	no	outpatients	8	National Natural Science Foundation of China and the Project of the Ministry of Science and Technology of the People’s Republic of China	HAM-D-17 item, SDS, N-back evaluation	no response outcome	no remission outcome	Some concerns
(Hvas, Juul et al. 2004) [141]	vitB12	1 mg	Denmark	ITT	70	50 (71)	75	MDD	single center	no	DSM-IV or ICD-10		no comorbidities	no	outpatients	4	Danish Medical Research Council, The Health Found of ‘danmark’s’ Sygeforsiking, EU Biomed (BMH4-98-3549), The Institute of Experimental Clinical Research, Aarhus University, E. Danielsen and Wife’s Foundation, The Novo Nordisk Foundation, Hans and Nora Buchard Foundation, Mogens Svarre Mogensen Foundation, Velux Foundation, and The Family Hede Nielsen Foundation.	MDI	no response outcome	no remission outcome	Low
(Hvas, Juul et al. 2004) [141]	PLB		Denmark	ITT	70	48 (69)	74	MDD	single center	no	DSM-IV or ICD-10		no comorbidities	no	outpatients	4	Danish Medical Research Council, The Health Found of ‘danmark’s’ Sygeforsiking, EU Biomed (BMH4-98-3549), The Institute of Experimental Clinical Research, Aarhus University, E. Danielsen and Wife’s Foundation, The Novo Nordisk Foundation, Hans and Nora Buchard Foundation, Mogens Svarre Mogensen Foundation, Velux Foundation, and The Family Hede Nielsen Foundation.	MDI	no response outcome	no remission outcome	Low
(Ille, Spona et al. 2007) [142]	fAA + MIR	NA	Austria	ITT	20	17 (85)	48.9 (12)	MDD	single center	no	DSM-IV	NA	no comorbidities	no	Inpatients	4	NA	BDI, HAM-D, CGI	no response outcome	no remission outcome	High
(Ille, Spona et al. 2007) [142]	PLB + MIR	NA	Austria	ITT	20	15 (75)	43.8 (12)	MDD	single center	no	DSM-IV	NA	no comorbidities	no	Inpatients	4	NA	BDI, HAM-D, CGI	no response outcome	no remission outcome	High
(Jahangard, Sadeghi et al. 2018) [143]	OMEGA3 + SER	1000 mg/day+50–200 mg/day	Iran	ITT	25	8 (32)	41.28 (11.56)	MDD	single center	no	DSM-5	clinical interview	no comorbidities	no	outpatients	12	no sponsor	MADRS, BDI, anxiety sensitivity index, insomnia severity index	no response outcome	no remission outcome	Low
(Jahangard, Sadeghi et al. 2018) [143]	PLB + SER	50–200 mg/day	Iran	ITT	25	8 (32)	43.64 (11.29)	MDD	single center	no	DSM-5	clinical interview	no comorbidities	no	outpatients	12	no sponsor	MADRS, BDI, anxiety sensitivity index, insomnia severity index	no response outcome	no remission outcome	Low
(Jazayeri, Tehrani-Doost et al. 2008) [38]	EPA	1 g/day	Iran	ITT	16	12 (75)	34.9 (8.7)	MDD	single center	no	DSM-IV	SCID	no comorbidities	no	outpatients	8	Vice-Chancellor for Research, Tehran University of Medical Sciences, Tehran, Iran.	HAM-D-17 item, response rate	≥50% reduction in score on HAM-D	no remission outcome	High
(Jazayeri, Tehrani-Doost et al. 2008) [38]	FLUO	20 mg 7 day	Iran	ITT	16	12 (75)	35.1 (9.4)	MDD	single center	no	DSM-IV	SCID	no comorbidities	no	outpatients	8	Vice-Chancellor for Research, Tehran University of Medical Sciences, Tehran, Iran.	HAM-D-17 item, response rate	≥50% reduction in score on HAM-D	no remission outcome	High
(Jazayeri, Tehrani-Doost et al. 2008) [38]	EPA + FLUO	1 g/day+20 mg/day	Iran	ITT	16	9 (56)	34.5 (11.3)	MDD	single center	no	DSM-IV	SCID	no comorbidities	no	outpatients	8	Vice-Chancellor for Research, Tehran University of Medical Sciences, Tehran, Iran.	HAM-D-17 item, response rate	≥50% reduction in score on HAM-D	no remission outcome	High
(Jelodar, Javid et al. 2018) [59]	FLUO + PLB	20 mg/day	Iran	ITT	20	NA	NA	MDD	single center	no	DSM-IV	SCID for DSM-IV	no comorbidities	no	outpatients	4	personally funded by the authors	BDI	no response outcome	no remission outcome	Low
(Jelodar, Javid et al. 2018) [59]	FLUO + SAFF	20 mg+30 mg/day	Iran	ITT	20	NA	NA	MDD	single center	no	DSM-IV	SCID for DSM-IV	no comorbidities	no	outpatients	4	personally funded by the authors	BDI	no response outcome	no remission outcome	Low
(Kagan, Sultzer et al. 1990) [70]	SAMe	200 mg/day	USA	ITT	9	0 (0)	NA	MDD	single center	no	DSM-III		no comorbidities	no	inpatients	3	NA	HAM-D, response rate	reductions of more than 50%.	no remission outcome	High
(Kagan, Sultzer et al. 1990) [70]	PLB		USA	ITT	6	0 (0)	NA	MDD	single center	no	DSM-III		no comorbidities	no	inpatients	3	NA	HAM-D, response rate	reductions of more than 50%.	no remission outcome	High
(Kalb, Trautmann-Sponsel et al. 2001) [144]	WS5572	900 mg/day	Germany	ITT	37	26 (70.3)	48 (11)	MDD	multi-center	no	DSM-IV	NA	no comorbidities	no	outpatients	6	Dr. Willmar Schwabe Pharmaceutical, Karlsruhe, Germany	HAMD-17 items, response rate	decrease ≥ 50% in total HAM-D score	no remission outcome	Low
(Kalb, Trautmann-Sponsel et al. 2001) [144]	PROB	NA	Germany	ITT	35	22 (62.9)	49 (10)	MDD	multi-center	no	DSM-IV	NA	no comorbidities	no	outpatients	6	Dr. Willmar Schwabe Pharmaceutical, Karlsruhe, Germany	HAMD-17 items, response rate	decrease ≥ 50% in total HAM-D score	no remission outcome	Low
(Kanchanatawan, Tangwongchai et al. 2018) [145]	CUR	1500 mg/day	Thailand	ITT	33	27 (81.8)	42.6 (13.6)	MDD	single center	no	DSM-IV-TR	MINI 6.0	no comorbidities	12 fluoxetine, 13 SSRIs, 5 sodium valproate, 1 lamotrigine, 16 clonazepam (10–50 mg/day, 40–125 mg/day, 200–1000 mg/day, 200 mg/day)	outpatients	12	Agricultural Research Developmental Agency (ARDA), Bangkok, Thailand	MADRS, HAM-A	no response outcome	no remission outcome	Low
(Kanchanatawan, Tangwongchai et al. 2018) [145]	PLB	NA	Thailand	ITT	32	22 (68.75)	46.2 (13.4)	MDD	single center	no	DSM-IV-TR	MINI 6.0	no comorbidities	12 fluoxetine, 13 SSRIs, 5 sodium valproate, 1 lamotrigine, 16 clonazepam (10–50 mg/day, 40–125 mg/day, 200–1000 mg/day, 200 mg/day)	outpatients	12	Agricultural Research Developmental Agency (ARDA), Bangkok, Thailand	MADRS, HAM-A	no response outcome	no remission outcome	Low
(Kashani, Esalatmanesh et al. 2018) [146]	SAFF	30 mg/day	Iran	ITT	28	28 (100)	55.71 (6.57)	MDD (mild to moderate)	multi-center	no	DSM-IV-TR	SCID for DSM-IV	post-menopausal hot flashes	no	outpatients	6	Tehran University of Medical Sciences	HAM-D-17 item, response rate, remission rate	improvement of 50% or higher of HDRS scores	HAM-D total score of ≤7	Low
(Kashani, Esalatmanesh et al. 2018) [146]	PLB	NA	Iran	ITT	28	28 (100)	55.43 (5.46)	MDD (mild to moderate)	multi-center	no	DSM-IV-TR	SCID for DSM-IV	post-menopausal hot flashes	no	outpatients	6	Tehran University of Medical Sciences	HAM-D-17 item, response rate, remission rate	improvement of 50% or higher of HDRS scores	HAM-D total score of ≤7	Low
(Kashani, Eslatmanesh et al. 2017) [60]	SAFF	30 mg/day	Iran	PP	34 randomized,32 analyzed	32 (100)	29.21 (7.69)	MDD post-partum	multi-center	no	DSM-IV-TR	SCID for DSM-IV	no comorbidities	no	outpatients	6	Tehran University of Medical Sciences	HAM-D-17 item, response rate, remission rate	improvement of 50% or higher of HDRS scores	HAM-D total score of ≤7	Some concerns
(Kashani, Eslatmanesh et al. 2017) [60]	FLUO	40 mg/day	Iran	PP	34 randomized,32 analyzed	32 (100)	32.09 (4.99)	MDD post-partum	multi-center	no	DSM-IV-TR	SCID for DSM-IV	no comorbidities	no	outpatients	6	Tehran University of Medical Sciences	HAM-D-17 item, response rate, remission rate	improvement of 50% or higher of HDRS scores	HAM-D total score of ≤7	Some concerns
(Kasper, Anghelescu et al. 2006) [147]	WS5570	600 or 1200 mg/day	Germany	ITT	243	149 (61)	46.05 (11.09)	MDD	multi-center	no	DSM-IV	NA	no comorbidities	no	outpatients	6	NA	HAMD-17, MADRS	decrease ≥ 50% in total HAM-D score	≤ 7 points in total HAM-D score	Low
(Kasper, Anghelescu et al. 2006) [147]	PLB	NA	Germany	ITT	81	56 (69.1)	46.9 (11.8)	MDD	multi-center	no	DSM-IV	NA	no comorbidities	no	outpatients	6	NA	HAMD-17, MADRS	decrease ≥ 50% in total HAM-D score	≤ 7 points in total HAM-D score	Low
(Kasper, Anghelescu et al. 2007) [148]	WS5570	1200 mg/day	Austria	ITT	68	48 (70.6)	46.6 (11.3)	MDD	multi-center	no	DSM-IV		no comorbidities	no	outpatients	16	NA	HAM-D, MADRS, BDI, response rate	HAMD reduction ≥ 50%	no remission outcome	Low
(Kasper, Anghelescu et al. 2007) [148]	PLB		Austria	ITT	24	18 (75)	47.0 (12.2)	MDD	multi-center	no	DSM-IV		no comorbidities	no	outpatients	16	NA	HAM-D, MADRS, BDI, response rate	HAMD reduction ≥ 50%	no remission outcome	Low
(Kasper, Anghelescu et al. 2015) [149]	SIL	1 cp/day	Germany	PP	159	105 (66)	47.7 (12.6)	MADD	multi-center	no	ICD-10	clinical interview	no comorbidities	no	outpatients	10	Dr. Willmar Schwabe GmbH & Co	HAMA, MADRS	at least 50% of the baseline value HAMA and MADRS	a total score o10 points (HAMA) and r10 points (MADRS) at treatment end	Low
(Kasper, Anghelescu et al. 2015) [149]	PLB		Germany	PP	156	113 (72.4)	47.9 (12.6)	MADD	multi-center	no	ICD-10	clinical interview	no comorbidities	no	outpatients	10	Dr. Willmar Schwabe GmbH & Co	HAMA, MADRS	“at least 50% of the baseline value HAMA and MADRS”	a total score o10 points (HAMA) and r10 points (MADRS) at treatment end	Low
(Kasper, Volz et al. 2024) [149]	SIL	80 mg/day	Austria	PP	170 randomized, 149 analyzed	118 (69.4)	45.8 (14.5)	MDD	single center	no	ICD-10	MINI	no comorbidities	no	outpatients	8	Medical University of Vienna	response rate, remission rate	a total MADRS score decrease by at least 50% of the baseline value	a total MADRS score of < 10 points	High
(Kasper, Volz et al. 2024) [149]	SER	50 mg/day	Austria	PP	171 randomized, 148 analyzed	110 (64.3)	44.7 (14.4)	MDD	single center	no	ICD-10	MINI	no comorbidities	no	outpatients	8	Medical University of Vienna	response rate, remission rate	a total MADRS score decrease by at least 50% of the baseline value	a total MADRS score of < 10 points	High
(Kasper, Volz et al. 2024) [150]	PLB		Austria	PP	157 randomized, 138 analyzed	99 (63.1)	46.6 (15.7)	MDD	single center	no	ICD-10	MINI	no comorbidities	no	outpatients	8	Medical University of Vienna	response rate, remission rate	a total MADRS score decrease by at least 50% of the baseline value	a total MADRS score of < 10 points	High
(Kaviani, Nikooyeh et al. 2022) [150]	vitD	50,000 IU/2wks	Iran	ITT	28	27 (96.4)	43.14 (9.25)	MDD (mild to moderate)	single center	no	DSM IV		no comorbidities	no	outpatients	8	National Nutrition and Food Technology Research Institute (NNFTRI)	BDI-II	no response outcome	no remission outcome	Low
(Kaviani, Nikooyeh et al. 2022) [151]	PLB		Iran	ITT	28	23 (82.1)	42.86 (8.01)	MDD (mild to moderate)	single center	no	DSM IV		no comorbidities	no	outpatients	8	National Nutrition and Food Technology Research Institute (NNFTRI)	BDI-II	no response outcome	no remission outcome	Low
(Khoraminya, Tehrani-Doost et al. 2013) [152]	vitD3 + FLUO	1500IU/day+20 mg/day	Iran	PP	20	17 (85)	38.1 (10.07)	MDD	single center	no	DSM-IV	NA	vitamin D-deficient	no	outpatients	8	no sponsor	HAM-D-17 item, BDI	no response outcome	no remission outcome	Some concerns
(Khoraminya, Tehrani-Doost et al. 2013) [152]	FLUO	20 mg 7 day	Iran	PP	20	17 (85)	39.65 (8.27)	MDD	single center	no	DSM-IV	NA	vitamin D-deficient	no	outpatients	8	no sponsor	HAM-D-17 item, BDI	no response outcome	no remission outcome	Some concerns
(Kolouri, Firoozabadi et al. 2016) [153]	NEP	400 mg/day	Iran	ITT	33	25 (75.8)	35.42 (9.4)	MDD	single center	no	DSM-5	SCID	no comorbidities	no	outpatients	4	Shi-raz University of Medical Sciences	BDI-II	no response outcome	no remission outcome	High
(Kolouri, Firoozabadi et al. 2016) [153]	SER	50 mg/day	Iran	ITT	33	25 (75.8)	35.12 (10.06)	MDD	single center	no	DSM-5	SCID	no comorbidities	no	outpatients	4	Shi-raz University of Medical Sciences	BDI-II	no response outcome	no remission outcome	High
(Kreuzer, Reiter et al. 2022) [154]	OMB	3g	Austria	ITT	28	21 (75)	44.63 (15.12)	MDD	single center	no	ICD-10	MINI	no comorbidities	no	inpatients	4	Swiss National Science Foundation, Austrian Science Fund, Austrian Research Promotion Agency, the Integrative Metabolism Research Center Graz, Austrian Infrastructure Program 2016/2017, Styrian Government (Zukunftsfonds, doc.funds program), The City of Graz and BIOTECHMED-GRAZ (Flagship project DYNIMO).	HAM-D, BDI	no response outcome	no remission outcome	Low
(Kreuzer, Reiter et al. 2022) [154]	PLB		Austria	ITT	29	24 (82.8)	40.38 (11.30)	MDD	single center	no	ICD-10	MINI	no comorbidities	no	inpatients	4	Swiss National Science Foundation, Austrian Science Fund, Austrian Research Promotion Agency, the Integrative Metabolism Research Center Graz, Austrian Infrastructure Program 2016/2017, Styrian Government (Zukunftsfonds, doc.funds program), The City of Graz and BIOTECHMED-GRAZ (Flagship project DYNIMO).	HAM-D, BDI	no response outcome	no remission outcome	Low
(Kumar, Menon et al. 2022) [155]	ESC + vit.D	10–20 mg/day + 60000IU every 5 days	India	ITT	31	NA	34.9 (10.5)	MDD	single center	no	DSM-5	NA	Vit.D deficiency (<20 ng/ml)	clonazepam (2 mg/day)	outpatients	12	no sponsor	MADRS, HAM-D-17 item, CGI-S, BDI, CGI-I	no response outcome	no remission outcome	Low
(Kumar, Menon et al. 2022) [155]	ESC + PLB	10–20 mg/day	India	ITT	28	NA	39.3 (11.8)	MDD	single center	no	DSM-5	NA	Vit.D deficiency (<20 ng/ml)	clonazepam (2 mg/day)	outpatients	12	no sponsor	MADRS, HAM-D-17 item, CGI-S, BDI, CGI-I	no response outcome	no remission outcome	Low
(Laakmann 1998) [156]	WS5573	3 x 300 mg/day	Germany	ITT	49	42 (85.7)	48.7 (1 1.8)	MDD (mild to moderate)	multi-center	no	DSM-IV		no comorbidities	no	outpatients	6	NA	HAM-D, response rate	HAMD total score reduction >=50%	no remission outcome	Low
(Laakmann 1998) [156]	WS5572	3 x 300 mg/day	Germany	ITT	49	40 (81.6)	47.3 (11.8)	MDD (mild to moderate)	multi-center	no	DSM-IV		no comorbidities	no	outpatients	6	NA	HAM-D, response rate	HAMD total score reduction >=50%	no remission outcome	Low
(Laakmann 1998) [156]	PLB		Germany	ITT	49	35 (71.4)	51.0 (12.7)	MDD (mild to moderate)	multi-center	no	DSM-IV		no comorbidities	no	outpatients	6	NA	HAM-D, response rate	HAMD total score reduction >=50%	no remission outcome	Low
(Lecrubier, Clerc et al. 2002) [157]	WS5570	900 mg/day	France	ITT	186	142 (76.3)	40.2 (11.7)	MDD	single center	no	DSM-IV	MINI	no comorbidities	no	outpatients	6	Dr. Willmar Schwabe Pharmaceuticals, Karlsruhe, Germany	HAM-D-17 item	at least 50% decreases in Hamilton score between baseline and treatment end	score of 6 or less on Hamilton scale at treatment end	Low
(Lecrubier, Clerc et al. 2002) [157]	PLB	NA	France	ITT	189	145 (76.7)	41.2 (11.4)	MDD	single center	no	DSM-IV	MINI	no comorbidities	no	outpatients	6	Dr. Willmar Schwabe Pharmaceuticals, Karlsruhe, Germany	HAM-D-17 item	at least 50% decreases in Hamilton score between baseline and treatment end	score of 6 or less on Hamilton scale at treatment end	Low
(Levine, Barak et al. 1995) [158]	PLB		Israel	PP	15	6 (40)	50.5	13 MDD, 2 bipolar depression	single center	no	DSM-III_R		no comorbidities	oxazepam (up to 15 mg/day)	outpatients	4	NA	HAM-D	a decrease of 15 or more points on the Hamilton depression scale	no remission outcome	Some concerns
(Levine, Barak et al. 1995) [158]	INO	12 g/day	Israel	PP	13	10 (76.9)	63.7	9 MDD, 4 bipolar depression	single center	no	DSM-III_R		no comorbidities	oxazepam (up to 15 mg/day)	outpatients	4	NA	HAM-D	a decrease of 15 or more points on the Hamilton depression scale	no remission outcome	Some concerns
(Levine, Barak et al. 1995) [159]	INO	12 g/day	Israel	PP	13	10 (76.9)	63.7	9 MDD, 4 bipolar depression	single center	no	DSM-III_R		no comorbidities	oxazepam (up to 15 mg/day)	outpatients	8	NA	HAM-D	a decrease of 15 or more points on the Hamilton depression scale	no remission outcome	Some concerns
(Levine, Barak et al. 1995) [159]	PLB		Israel	PP	14	6 (40)	50.5	13 MDD, 2 bipolar depression	single center	no	DSM-III_R		no comorbidities	oxazepam (up to 15 mg/day)	outpatients	8	NA	HAM-D	a decrease of 15 or more points on the Hamilton depression scale	no remission outcome	Some concerns
(Li, Li et al. 2022) [94]	SHU	6 g/day	China	ITT	194	165 (85)	43,68 (11.07)	MADD	multi-center	no	DSM IV		no comorbidities	no	outpatients	8	Kunming Traditional Chinese Medicine Factory Co., Ltd.	HAM-D, HAMA, response rate, remission rate, CGI-S	HAMA/HAMD-17 score reduction rate ≥50%	HAMA score <7 and HAMD-17 score ≤7	Low
(Li, Li et al. 2022) [94]	PLB		China	ITT	191	158 (83)	45,20 (11.77)	MADD	multi-center	no	DSM IV		no comorbidities	no	outpatients	8	Kunming Traditional Chinese Medicine Factory Co., Ltd.	HAM-D, HAMA, response rate, remission rate, CGI-S	HAMA/HAMD-17 score reduction rate ≥50%	HAMA score <7 and HAMD-17 score ≤7	Low
(Lin, Kuo et al. 2024) [160]	PS128 + TAU	300 mg 2/day	Taiwan	PP	17 randomized, 16 analyzed	12 (75)	39,31 (13.96)	MDD	single center	no	DSM-5	SCID-IV	no comorbidities	10 out of 16 SSRIs or SNRIs	outpatients	8	National Science and Technology Council	HAM-D	no response outcome	no remission outcome	Some concerns
(Lin, Kuo et al. 2024) [160]	PLB + TAU		Taiwan	PP	17 randomized, 16 analyzed	12 (75)	36,81 (13.17)	MDD	single center	no	DSM-5	SCID-IV	no comorbidities	6 out of 16 SSRIs or SNRIs	outpatients	8	National Science and Technology Council	HAM-D	no response outcome	no remission outcome	Some concerns
(Lin, Wang et al. 2022) [161]	BZT	250–1500 mg/day	Taiwan	PP	39	27 (69.2)	66.4 (8.1)	MDD	multi-center	no	DSM IV		no comorbidities	lorazepam (up to 4 mg/d)	outpatients	8	no sponsor	HAM-D, CGI_S, GDS	no response outcome	no remission outcome	Some concerns
(Lin, Wang et al. 2022) [161]	SER	25–150 mg/day	Taiwan	PP	39	34 (67.2)	66,9 (7.2)	MDD	multi-center	no	DSM IV		no comorbidities	lorazepam (up to 4 mg/d)	outpatients	8	no sponsor	HAM-D, CGI_S, GDS	no response outcome	no remission outcome	Some concerns
(Lin, Wang et al. 2022) [161]	PLB		Taiwan	PP	39	29 (74.4)	70.5 (7.3)	MDD	multi-center	no	DSM IV		no comorbidities	lorazepam (up to 4 mg/d)	outpatients	8	no sponsor	HAM-D, CGI_S, GDS	no response outcome	no remission outcome	Some concerns
(Lôo, Poirier et al. 2000) [162]	SUL + CLO	600 mg/day+75–100 mg/day	France	PP	43	30 (70)	40.9 (9.7)	MDD	multi-center	no	DSM-III_R		no comorbidities	hypnotics and anxiolytics without potential activity on mood	outpatients		NA	HAM-A, MADRS, CGI	decrease of 50% or more in MADRS	no remission outcome	Some concerns
(Lôo, Poirier et al. 2000) [162]	PLB + CLO	75–100 mg/day	France	PP	47	32 (68)	46.3 (10.1)	MDD	multi-center	no	DSM-III_R		no comorbidities	hypnotics and anxiolytics without potential activity on mood	outpatients		NA	HAM-A, MADRS, CGI	decrease of 50% or more in MADRS	no remission outcome	Some concerns
(Lopresti and Drummond 2017) [61]	CUR	500 or 1000 mg/day	Australia	ITT	61	55 (90.16)	43.69	MDD	single center	no	DSM-IV	MINI 6.0	no comorbidities	34 out of 61 antidepressants	outpatients	12	NA	Inventory of Depressive Symptomatology self-rated version (IDS-SR30), STAI	> 50% reduction in IDS score	no remission outcome	Low
(Lopresti and Drummond 2017) [61]	CUR + SAFF	500 mg/day+30 mg/day	Australia	ITT	26	22 (85)	41.12	MDD	single center	no	DSM-IV	MINI 6.0	no comorbidities	13 out of 26 antidepressants	outpatients	12	NA	Inventory of Depressive Symptomatology self-rated version (IDS-SR30), STAI	> 50% reduction in IDS score	no remission outcome	Some concerns
(Lopresti and Drummond 2017) [61]	PLB	NA	Australia	ITT	36	32 (90)	42.11	MDD	single center	no	DSM-IV	MINI 6.0	no comorbidities	15 out of 32 antidepressants	outpatients	12	NA	Inventory of Depressive Symptomatology self-rated version (IDS-SR30), STAI	> 50% reduction in IDS score	no remission outcome	Some concerns
(Lopresti, Maes et al. 2014) [163]	CUR	1000 mg/day	Australia	ITT	28	18 (72)	44.04 (11.94)	MDD	single center	no	DSM-IV	MINI 6.0	no comorbidities	antidepressant 10/28; no antidepressant 18/28	outpatients	8	Arjuna Natural Extracts Limited	Inventory of Depressive Symptomatology self-rated version (IDS-SR30), STAI	no response outcome	no remission outcome	Low
(Lopresti, Maes et al. 2014) [163]	PLB	NA	Australia	ITT	28	17 (60.7)	48.54 (11.73)	MDD	single center	no	DSM-IV	MINI 6.0	no comorbidities	antidepressant 9/28; no antidepressant 19/28	outpatients	8	Arjuna Natural Extracts Limited	Inventory of Depressive Symptomatology self-rated version (IDS-SR30), STAI	no response outcome	no remission outcome	Low
(Lyoo, Yoon et al. 2012) [164]	ESC + CREA	20 mg/day+5g/day	South Corea	ITT	25	25 (100)	45.7 (12.7)	MDD	single center	no	DSM-IV	SCID-IV	no comorbidities	10 out of 25 lorazepam, 6 out of 25 zolpidem (1,5 mg)	outpatients	8	Brain Research Center of the 21st Century Frontier Research Program (Dr. Lyoo), Global Research Network Program, the Korea Research Foundation (Dr. Lyoo), the Mental Illness Research, Education and Clinical Center of Veterans Integrated Service Network 19 (Dr. Renshaw), National Institute on Drug Abuse (Dr. Renshaw and Drs. Renshaw and Lyoo), NIMH (Dr. Renshaw).	HAM-D, MADRS, CGI-S, response rate, remission rate	decrease of 50% or more in HAM-D score from baseline	HAM-D score of 7 or less	Low
(Lyoo, Yoon et al. 2012) [164]	ESC + PLB	20 mg/day	South Corea	ITT	27	27 (100)	47.5 (9.5)	MDD	single center	no	DSM-IV	SCID-IV	no comorbidities	12 out of 27 lorazepam, 9 out of 27 zolpidem (10 mg)	outpatients	8	Brain Research Center of the 21st Century Frontier Research Program (Dr. Lyoo), Global Research Network Program, the Korea Research Foundation (Dr. Lyoo), the Mental Illness Research, Education and Clinical Center of Veterans Integrated Service Network 19 (Dr. Renshaw), National Institute on Drug Abuse (Dr. Renshaw and Drs. Renshaw and Lyoo), NIMH (Dr. Renshaw).	HAM-D, MADRS, CGI-S, response rate, remission rate	decrease of 50% or more in HAM-D score from baseline	HAM-D score of 7 or less	Low
(Majmasanaye, Mehrpooya et al. 2024) [165]	CQ10 + TAU	200 mg	Iran	PP	46 randomized, 36 analyzed	24 (66.6)	45.03 (6.71)	MDD	single center	no	DSM-5	SCID	no comorbidities	SSRIs (adequate dosages that was defined as ≥50 mg/d of sertraline)	outpatients	8	Iran University of Medical Sciences, Tehran, Iran.	MADRS	no response outcome	no remission outcome	Some concerns
(Majmasanaye, Mehrpooya et al. 2024) [165]	PLB + TAU		Iran	PP	46 randomized, 33 analyzed	26 (78.7)	47.7 (7.44)	MDD	single center	no	DSM-5	SCID	no comorbidities	SSRIs (adequate dosages that was defined as ≥50 mg/d of sertraline)	outpatients	8	Iran University of Medical Sciences, Tehran, Iran.	MADRS	no response outcome	no remission outcome	Some concerns
(Mao, Xie et al. 2015) [166]	RHO	340 mg/day	USA	ITT	20	8 (40)	46.9 (16.9)	MDD	single center	no	DSM-IV	SCID for DSM-IV	no comorbidities	no	outpatients	12	National Institutes of Health Center for Complementary and Alternative Medicine (NCCAM), the Jack Warsaw Fund for Research in Biological Psychiatry, University of Pennsylvania	HAM-D-17 item, BDI	no response outcome	no remission outcome	High
(Mao, Xie et al. 2015) [166]	SER	50 mg/day	USA	ITT	19	10 (53)	41.4 (14.6)	MDD	single center	no	DSM-IV	SCID for DSM-IV	no comorbidities	no	outpatients	12	National Institutes of Health Center for Complementary and Alternative Medicine (NCCAM), the Jack Warsaw Fund for Research in Biological Psychiatry, University of Pennsylvania	HAM-D-17 item, BDI	no response outcome	no remission outcome	High
(Mao, Xie et al. 2015) [166]	PLB		USA	ITT	18	8 (44)	46.7 (15.2)	MDD	single center	no	DSM-IV	SCID for DSM-IV	no comorbidities	no	outpatients	12	National Institutes of Health Center for Complementary and Alternative Medicine (NCCAM), the Jack Warsaw Fund for Research in Biological Psychiatry, University of Pennsylvania	HAM-D-17 item, BDI	no response outcome	no remission outcome	High
(Marangell, Martinez et al. 2003) [53]	PLB	NA	USA	ITT	17	14 (82.4)	47.9 (11.2)	MDD	single center	no	DSM-IV	SCID	no comorbidities	no	outpatients	6	Martek Biosciences Corporation	HDRS-28 item, MADRS, GAF	≥50% reduction in score on MADRS	no remission outcome	Low
(Marangell, Martinez et al. 2003) [53]	DHA	2 g/day	USA	ITT	18	14 (77.8)	46.8 (11.6)	MDD	single center	no	DSM-IV	SCID	no comorbidities	no	outpatients	6	Martek Biosciences Corporation	HDRS-28 item, MADRS, GAF	≥50% reduction in score on MADRS	no remission outcome	Low
(Masoumi, Kazemi et al. 2016) [167]	CTP + PLB	20 mg 1/day	Iran	ITT	30	30 (100)	55.67 (6.06)	MDD	single center	no	DSM IV		post-menopausal	no	outpatients	4	Hamadan University of Medical Sciences	BDI	no response outcome	no remission outcome	High
(Masoumi, Kazemi et al. 2016) [167]	CTP + OMEGA3	20 mg+1g	Iran	ITT	30	30 (100)	55.17 (7.33)	MDD	single center	no	DSM IV		post-menopausal	no	outpatients	4	Hamadan University of Medical Sciences	BDI	no response outcome	no remission outcome	High
(Mazidi, Shemshian et al. 2016) [62]	SAF	50 mg 2/day	Iran	ITT	30	22 (73.3)	42.8 (10.65)	MDD + mixed anxiety	single center	no	DSM IV	NA	no comorbidities	no	outpatients	12	National Science Foundation, Birjand University of Medical Sciences and Mashhad University of Medical Sciences	BDI, BAI	no response outcome	no remission outcome	Low
(Mazidi, Shemshian et al. 2016) [62]	PLB		Iran	ITT	30	20 (66.7)	43.6 (8.83)	MDD + mixed anxiety	single center	no	DSM IV	NA	no comorbidities	no	outpatients	12	National Science Foundation, Birjand University of Medical Sciences and Mashhad University of Medical Sciences	BDI, BAI	no response outcome	no remission outcome	Low
(Mech and Farah 2016) [168]	vitB	1 cp/day	USA	PP	170	NA	NA	MDD	single center	no	DSM-5	NA	MTHFR C677T or A1298C polymorphism	no	outpatients	8	Jaymac Pharmaceuticals of Sunset, Louisiana	MADRS	no response outcome	MADRS<7	Some concerns
(Mech and Farah 2016) [168]	PLB	NA	USA	PP	160	NA	NA	MDD	single center	no	DSM-5	NA	MTHFR C677T or A1298C polymorphism	no	outpatients	8	Jaymac Pharmaceuticals of Sunset, Louisiana	MADRS	no response outcome	MADRS<7	Some concerns
(Mischoulon, Papakostas et al. 2009) [40]	EPA	1 g/day	USA	ITT	16	NA	NA	MDD	single center	no	DSM-IV	SCID for DSM-IV	no comorbidities	no	outpatients	8	National Center for Complementary and Alternative Medicine (NCCAM).	HAM-D, response rate, remission rate	≥50% reduction in score on HAM-D	HAM-D score of 7 or less	Low
(Mischoulon, Papakostas et al. 2009) [40]	PLB	NA	USA	ITT	19	NA	NA	MDD	single center	no	DSM-IV	SCID for DSM-IV	no comorbidities	no	outpatients	8	National Center for Complementary and Alternative Medicine (NCCAM).	HAM-D, response rate, remission rate	≥50% reduction in score on HAM-D	HAM-D score of 7 or less	Low
(Mischoulon, Dunlop et al. 2022) [39]	EPA	4 g/day	USA	PP	16	11 (68.8)	45.8 (13.0)	MDD	single center	no	DSM-5	MINI 7.0	no comorbidities	no	outpatients	12	National Center for Complementary and Integrative Health (NCCIH)	response rate IDS-C30	a ≥ 50% decrease of Inventory of Depressive Symptomatology, Clinician-Rated version (IDS-C30) scores.	no remission outcome	Some concerns
(Mischoulon, Dunlop et al. 2022) [39]	PLB		USA	PP	15	12 (80)	50.3 (12.0)	MDD	single center	no	DSM-5	MINI 7.0	no comorbidities	no	outpatients	12	National Center for Complementary and Integrative Health (NCCIH)	response rate IDS-C30	a ≥ 50% decrease of Inventory of Depressive Symptomatology, Clinician-Rated version (IDS-C30) scores.	no remission outcome	Some concerns
(Mischoulon, Price et al. 2014) [71]	SAMe	1600–3200 mg/day	USA	ITT	64	NA	NA	MDD	single center	no	DSM-IV	SCID-I/P	no comorbidities	no	outpatients	12	National Institutes of Health (NIH) and the National Center for Complementary and Alternative Medicine (NCCAM)	HAM-D-17, CGI-S, CGI-I, IDS-C, IDS-SR, response rate, remission rate	decrease of 50% or more in HAM-D score from baseline	HAM-D score of 7 or less	Low
(Mischoulon, Price et al. 2014) [71]	ESC	10–20 mg/day	USA	ITT	65	NA	NA	MDD	single center	no	DSM-IV	SCID-I/P	no comorbidities	no	outpatients	12	National Institutes of Health (NIH) and the National Center for Complementary and Alternative Medicine (NCCAM)	HAM-D-17, CGI-S, CGI-I, IDS-C, IDS-SR, response rate, remission rate	decrease of 50% or more in HAM-D score from baseline	HAM-D score of 7 or less	Low
(Mischoulon, Price et al. 2014) [71]	PLB		USA	ITT	60	NA	NA	MDD	single center	no	DSM-IV	SCID-I/P	no comorbidities	no	outpatients	12	National Institutes of Health (NIH) and the National Center for Complementary and Alternative Medicine (NCCAM)	HAM-D-17, CGI-S, CGI-I, IDS-C, IDS-SR, response rate, remission rate	decrease of 50% or more in HAM-D score from baseline	HAM-D score of 7 or less	Low
(Moshiri, Basti et al. 2006) [63]	SAFF	30 mg/day	Iran	ITT	20	9 (45)	35.45 (8.19)	MDD	single center	no	DSM-IV	SCID for DSM-IV	no comorbidities	no	outpatients	6	Tehran University of Medical Sciences	HAM-D-17 item	no response outcome	no remission outcome	Low
(Moshiri, Basti et al. 2006) [63]	PROB	NA	Iran	ITT	20	8 (40)	35.85 (5.63)	MDD	single center	no	DSM-IV	SCID for DSM-IV	no comorbidities	no	outpatients	6	Tehran University of Medical Sciences	HAM-D-17 item	no response outcome	no remission outcome	Low
(Mozaffari-Khosravi, Nabizade et al. 2013) [52]	EPA	1 g/day	Iran	ITT	21	13 (61.9)	37.5 (12.4)	MDD	single center	no	DSM-IV	NA	no comorbidities	antidepressant as usual	outpatients	12	Shahid Sadoughi University of Medical Sciences,Yazd,Iran	HAM-D-17 item, response rate, remission rate	≥50% reduction in score on HAM-D	HAM-D score of 7 or less	Low
(Mozaffari-Khosravi, Nabizade et al. 2013) [52]	DHA	1 g/day	Iran	ITT	20	12 (60)	34 (13.1)	MDD	single center	no	DSM-IV	NA	no comorbidities	antidepressant as usual	outpatients	12	Shahid Sadoughi University of Medical Sciences,Yazd,Iran	HAM-D-17 item, response rate, remission rate	≥50% reduction in score on HAM-D	HAM-D score of 7 or less	Low
(Mozaffari-Khosravi, Nabizade et al. 2013) [52]	PLB	NA	Iran	ITT	21	13 (61.9)	33.8 (9.9)	MDD	single center	no	DSM-IV	NA	no comorbidities	antidepressant as usual	outpatients	12	Shahid Sadoughi University of Medical Sciences,Yazd,Iran	HAM-D-17 item, response rate, remission rate	≥50% reduction in score on HAM-D	HAM-D score of 7 or less	Low
(Nemets and Levine 2013) [169]	TAU + CREA	5g	Israel	ITT	5	5 (100)	60.8 (9.8)	MDD	single center	yes	DSM-IV-TR	NA	no comorbidities	1 out of 5 citalopram, 3 out of 5 fluoxetine, 1 out of 5 paroxetine (citalopram 40 mg, fluoxetine 20 mg, fluoxetine 40 mg, paroxetine 40 mg)	outpatients	4	NARSAD Independent Investigator Award	HAM-D, response rate	a sustained decrease of at least 50% in the HDRS total score at 4 weeks of treatment versus baseline	no remission outcome	Low
(Nemets and Levine 2013) [169]	TAU + PLB		Israel	ITT	5	3 (60)	50.0 (15.1)	MDD	single center	yes	DSM-IV-TR	NA	no comorbidities	2 out of 5 paroxetine, 2 out of 5 citalopram, 1 out of 5 mirtazapine (paroxetine 60 mg, citalopram 20 mg, citalopram 40 mg, mirtazapine 30 mg)	outpatients	4	NARSAD Independent Investigator Award	HAM-D, response rate	a sustained decrease of at least 50% in the HDRS total score at 4 weeks of treatment versus baseline	no remission outcome	Low
(Nemets and Levine 2013) [169]	TAU + CREA	10g	Israel	ITT	4	4 (100)	60.8 (9.8)	MDD	single center	yes	DSM-IV-TR	NA	no comorbidities	2 out of 4 fluvoxamine, 1 out of 4 fluoxetine, 1 out of 4 escitalopram (fluvoxamine 200 mg, fluvoxamine 250 mg, fluoxetine 20 mg, escitalopram 10 mg)	outpatients	4	NARSAD Independent Investigator Award	HAM-D, response rate	a sustained decrease of at least 50% in the HDRS total score at 4 weeks of treatment versus baseline	no remission outcome	Low
(Nemets and Levine 2013) [169]	TAU + PLB		Israel	ITT	4	2 (50)	50.0 (15.1)	MDD	single center	yes	DSM-IV-TR	NA	no comorbidities	1 out of 4 mirtazapine, 2 out of 4 paroxetine, 1 out of 4 venlafaxine (mirtazapine 30 mg, paroxetine 20 mg, venlafaxine 300 mg)	outpatients	4	NARSAD Independent Investigator Award	HAM-D, response rate	a sustained decrease of at least 50% in the HDRS total score at 4 weeks of treatment versus baseline	no remission outcome	Low
(Nemets, Stahl et al. 2002) [41]	EPA + TAU	2 g/day	Israel	ITT	10	9 (90)	54.2 (13.9)	MDD	single center	no	DSM-IV	NA	no comorbidities	paroxetine, fluoxetine, mirtazapine, moclobemide, citalopram	outpatients	4	NA	HAM-D-24 item	no response outcome	no remission outcome	Low
(Nemets, Stahl et al. 2002) [41]	PLB + TAU	NA	Israel	ITT	10	8 (80)	52.1 (10.2)	MDD	single center	no	DSM-IV	NA	no comorbidities	paroxetine, fluoxetine, mirtazapine, moclobemide, citalopram	outpatients	4	NA	HAM-D-24 item	no response outcome	no remission outcome	Low
(Ning, Zhou et al. 2022) [170]	ZPG	6.75 g x 2/day	China	ITT	89	47 (53)	68.1 (8.0)	MDD (mild to moderate)	multi-center	no	DSM-5		Parkinson's disease	pramipexole (≥ 0.75 mg/day)	outpatients	12	National Key Research and Development Program of China, General Program of National Natural Science Foundation of China, Science and Technology Program of Jiangsu Province, General Program of Jiangsu Provincial Natural Science Foundation of China, National Natural Science, Foundation of China Youth Science Fund Project, Special Project for the Development of traditional Chinese Medicine Science and Technology in Jiangsu Province	HAM-D-17 item, response rate, remission rate	a ≥ 50% reduction in HAM-D score	HAM-D score < 8	Low
(Ning, Zhou et al. 2022) [170]	PLB		China	ITT	87	42 (60)	66.4 (9.8)	MDD (mild to moderate)	multi-center	no	DSM-5		Parkinson's disease	pramipexole (≥ 0.75 mg/day)	outpatients	12	National Key Research and Development Program of China, General Program of National Natural Science Foundation of China, Science and Technology Program of Jiangsu Province, General Program of Jiangsu Provincial Natural Science Foundation of China, National Natural Science, Foundation of China Youth Science Fund Project, Special Project for the Development of traditional Chinese Medicine Science and Technology in Jiangsu Province	HAM-D-17 item, response rate, remission rate	a ≥ 50% reduction in HAM-D score	HAM-D score < 8	Low
(Noorbala, Akhondzadeh et al. 2005) [64]	SAFF	30 mg/day	Iran	ITT	20	9 (45)	37.3 (8.56)	MDD	single center	no	DSM-IV	SCID for DSM-IV	no comorbidities	no	outpatients	6	Tehran University of Medical Sciences and Iranian Red Crescent	HAM-D-17 item	no response outcome	no remission outcome	Low
(Noorbala, Akhondzadeh et al. 2005) [64]	FLUO	20 mg/day	Iran	ITT	20	11 (55)	36.5 (7.27)	MDD	single center	no	DSM-IV	SCID for DSM-IV	no comorbidities	no	outpatients	6	Tehran University of Medical Sciences and Iranian Red Crescent	HAM-D-17 item	no response outcome	no remission outcome	Low
(Nowak, Siwek et al. 2003) [171]	ZNC + TAU	25 mg/day	Poland	PP	10	2 (20)	42.2 (13.7)	MDD	single center	no	DSM IV		no comorbidities	clomipramine, amitriptyline, citalopram, fluoxetine (125–150 mg, 125–150 mg, 20 mg, 20–40 mg)	outpatients	12	NA	HAM-D, BDI	no response outcome	no remission outcome	Some concerns
(Nowak, Siwek et al. 2003) [171]	PLB + TAU		Poland	PP	10	6 (60)	43.4 (8.5)	MDD	single center	no	DSM IV		no comorbidities	clomipramine, amitriptyline, citalopram, fluoxetine (125–150 mg, 125–150 mg, 20 mg, 20–40 mg)	outpatients	12	NA	HAM-D, BDI	no response outcome	no remission outcome	Some concerns
(Padhan, Mohapatra et al. 2024) [172]	TAU + SAR	500 mg/day	India	ITT	30	15 (50)	44 (12.7)	MDD	single center	no	DSM-5		no comorbidities	24 out of 30 sertraline, 5 out of 30 escitalopram, 1 out of 30 paroxetine (50 mg/day)	outpatients	8	Indian Council of Medical Research (ICMR)	MADRS, response rate, remission rate, CGI-S	proportion of patients showing a ≥50% decrease in MADRS scores from baseline to 8 weeks	percentage of patients achieving MADRS scores <7 at 8 weeks	Low
(Padhan, Mohapatra et al. 2024) [172]	TAU + PLB		India	ITT	30	14 (46.7)	39.5 (12.8)	MDD	single center	no	DSM-5		no comorbidities	25 out of 30 sertraline, 4 out of 30 escitalopram, 1 out of 30 paroxetine (50 mg/day)	outpatients	8	Indian Council of Medical Research (ICMR)	MADRS, response rate, remission rate, CGI-S	proportion of patients showing a ≥50% decrease in MADRS scores from baseline to 8 weeks	percentage of patients achieving MADRS scores <7 at 8 weeks	Low
Palhano-Fontes F. et al., (2016) [173]	AYA	1 ml/kg/day	Brazil	PP	17 randomized, 14 analyzed	11 (78.6)	39.71 (11.26)	MDD	single center	yes	DSM IV		no comorbidities	no	outpatients	1	Brazilian National Council for Scientific and Technological Development, CAPES Foundation within the Ministry of Education	response rate, remission rate	a reduction of 50% or more in baseline HAMD scores	HAM-D≤7 or MADRS≤10	Some concerns
Palhano-Fontes F. et al., (2016) [173]	PLB		Brazil	PP	18 randomized, 15 analyzed	10 (66.7)	44.2 (11.98)	MDD	single center	yes	DSM IV		no comorbidities	no	outpatients	1	Brazilian National Council for Scientific and Technological Development, CAPES Foundation within the Ministry of Education	response rate, remission rate	a reduction of 50% or more in baseline HAMD scores	HAM-D≤7 or MADRS≤10	Some concerns
(Panahi, Badeli et al. 2015) [174]	CVE + TAU	1800 mg/day	Iran	PP	42	24 (57.1)	40.79 (10.87)	MDD	single center	no	DSM-IV	NA	no comorbidities	TCA, BDZ, SSRI, SNRI, beta-blockers	outpatients	6	NA	BDI-II	no response outcome	no remission outcome	High
(Panahi, Badeli et al. 2015) [174]	TAU	NA	Iran	PP	50	29 (58)	40.40 (9.56)	MDD	single center	no	DSM-IV	NA	no comorbidities	TCA, BDZ, SSRI, SNRI, beta-blockers	outpatients	6	NA	BDI-II	no response outcome	no remission outcome	High
(Pancheri, Scapicchio et al. 2002) [72]	SAMe	400 mg/die (i.m.)	Italy	ITT	146	102 (70)	48.2 (12.2)	MDE	multi-center	no	DSM-IV	NA	no comorbidities	no	outpatients	4	Knoll Farmaceutici Spa, Liscate, Milan (Italy)	HAM-D-21 item, CGI, MADRS, response rate	decrease of 50% or more in HAM-D score from baseline	no remission outcome	High
(Pancheri, Scapicchio et al. 2002) [72]	IMI	75 mg/day	Italy	ITT	147	83 (56.4)	48.8 (14)	MDE	multi-center	no	DSM-IV	NA	no comorbidities	no	outpatients	4	Knoll Farmaceutici Spa, Liscate, Milan (Italy)	HAM-D-21 item, CGI, MADRS, response rate	decrease of 50% or more in HAM-D score from baseline	no remission outcome	High
(Papakostas, Mischoulon et al. 2010) [73]	SAMe + TAU	800 mg/day	USA	ITT	39	21 (53.8)	NA	MDD	single center	no	DSM-IV	SCID-I/P	no comorbidities	fluoxetine, sertraline, paroxetine, citalopram, escitalopram, venlafaxine, duloxetine (minimally adequate dose was defined as 20 mg/day or more of fluoxetine, citalopram, or paroxetine; 10 mg/day or more of escitalopram; 50 mg/day or more of sertraline; 60 mg/day or more of duloxetine; and 150 mg/day or more of venlafaxine)	outpatients	6	National Institute of Mental Health	response rate, remission rate according to HDRS	decrease of 50% or more in HAM-D score from baseline	HAM-D score of 7 or less	Low
(Papakostas, Mischoulon et al. 2010) [73]	PLB + TAU		USA	ITT	34	23 (67.6)	NA	MDD	single center	no	DSM-IV	SCID-I/P	no comorbidities	fluoxetine, sertraline, paroxetine, citalopram, escitalopram, venlafaxine, duloxetine (minimally adequate dose was defined as 20 mg/day or more of fluoxetine, citalopram, or paroxetine; 10 mg/day or more of escitalopram; 50 mg/day or more of sertraline; 60 mg/day or more of duloxetine; and 150 mg/day or more of venlafaxine)	outpatients	6	National Institute of Mental Health	response rate, remission rate according to HDRS	decrease of 50% or more in HAM-D score from baseline	HAM-D score of 7 or less	Low
(Papakostas, Shelton et al. 2012) [175]	LMF	7.5 mg/day	USA	ITT	36	NA	NA	MDD	multi-center	yes	DSM-IV	SCID for DSM-IV	no comorbidities	sertraline, escitalopram, fluoxetine, citalopram, paroxetine	outpatients	4	Pamlab	HAM-D, CGI-S, response rate, remission rate	reduction of ≥50% in HAM-D	HAM-D score≤ 7	Low
(Papakostas, Shelton et al. 2012) [175]	PLB	NA	USA	ITT	112	NA	NA	MDD	multi-center	yes	DSM-IV	SCID for DSM-IV	no comorbidities	sertraline, escitalopram, fluoxetine, citalopram, paroxetine	outpatients	4	Pamlab	HAM-D, CGI-S, response rate, remission rate	reduction of ≥50% in HAM-D	HAM-D score≤ 7	Low
(Papakostas, Shelton et al. 2012) [175]	LMF	15 mg/day	USA	ITT	19	NA	NA	MDD	multi-center	yes	DSM-IV	SCID for DSM-IV	no comorbidities	sertraline, escitalopram, fluoxetine, citalopram, paroxetine	outpatients	4	Pamlab	HAM-D, CGI-S, response rate, remission rate	reduction of ≥50% in HAM-D	HAM-D score≤ 7	Low
(Papakostas, Shelton et al. 2012) [175]	PLB	NA	USA	ITT	56	NA	NA	MDD	multi-center	yes	DSM-IV	SCID for DSM-IV	no comorbidities	sertraline, escitalopram, fluoxetine, citalopram, paroxetine	outpatients	4	Pamlab	HAM-D, CGI-S, response rate, remission rate	reduction of ≥50% in HAM-D	HAM-D score≤ 7	Low
(Park, Park et al. 2015) [176]	OMEGA3 + TAU	1.14 mgEPA+600 mgDHA/day	Korea	ITT	18	14 (77.8)	43.5 (3.72)	MDD	single center	no	DSM-IV	NA	no comorbidities	antidepressants	outpatients	12	Korea Research Foundation, Korean Government	HAM-D-17 item, CGI-S	no response outcome	no remission outcome	Low
(Park, Park et al. 2015) [176]	PLB + TAU	NA	Korea	ITT	17	13 (76.5)	39.41 (3.58)	MDD	single center	no	DSM-IV	NA	no comorbidities	antidepressants	outpatients	12	Korea Research Foundation, Korean Government	HAM-D-17 item, CGI-S	no response outcome	no remission outcome	Low
(Piperoglou, Hopwood et al. 2023) [42]	DHA + EPA	130 mg+35 mg	Australia	ITT	13	4 (29.2)	46.7 (9.3)	MDD	single center	no	DSM-5		no comorbidities	no	outpatients	8	Bionutrients, Pty, Ltd	HAM-D, HAM-A	no response outcome	no remission outcome	Low
(Piperoglou, Hopwood et al. 2023) [42]	PLB		Australia	ITT	8	5 (55)	45.5 (13.1)	MDD	single center	no	DSM-5		no comorbidities	no	outpatients	8	Bionutrients, Pty, Ltd	HAM-D, HAM-A	no response outcome	no remission outcome	Low
(Ramezani, Kheirkhah et al. 2022) [177]	CAR + SER	1000 mg + 100 mg	Iran	ITT	30	16 (53.3)	38,13 (11.18)	MDD	single center	no	DSM-5	clinical interview	no comorbidities	no	outpatients	6	Babol University of Medical Sciences	BDI and response definition	50% reduction of the baseline BDI score	no remission outcome	Low
(Ramezani, Kheirkhah et al. 2022) [177]	PLB + SER	100 mg	Iran	ITT	30	19 (63.3)	35.87 (8. 0)	MDD	single center	no	DSM-5	clinical interview	no comorbidities	no	outpatients	6	Babol University of Medical Sciences	BDI and response definition	50% reduction of the baseline BDI score	no remission outcome	Low
(Ranjbar, Shams et al. 2014) [178]	ZNC + TAU	25 mg/day	Iran	PP	22 randomized, 21 analyzed	20 (95)	37 (9)	MDD	single center	no	DSM-IV	NA	no comorbidities	citalopram or fluoxetine (20–60 mg/day)	outpatients	12	Center for Neuroscience Research, Shahid Beheshti University of Medical Sciences.	HAM-D-24 item	no response outcome	no remission outcome	Some concerns
(Ranjbar, Shams et al. 2014) [178]	TAU	NA	Iran	PP	22 randomized, 17 analyzed	14 (82)	37.5 (8)	MDD	single center	no	DSM-IV	NA	no comorbidities	citalopram or fluoxetine (20–60 mg/day)	outpatients	12	Center for Neuroscience Research, Shahid Beheshti University of Medical Sciences.	HAM-D-24 item	no response outcome	no remission outcome	Some concerns
(Rees, Austin et al. 2008) [43]	EPA + DHA	6g/day	Australia	ITT	13	13 (100)	31.2 (4.4)	MDD	single center	no	DSM IV	CIDI	from the third trimester of pregnancy to 6 months postnatal.	no	outpatients	6	NSW Institute of Psychiatry, Pfizer Australia, NHMRC, and NSW Department of Health.	HAM-D, MADRS, EPDS	no response outcome	no remission outcome	Low
(Rees, Austin et al. 2008) [43]	PLB		Australia	ITT	13	13 (100)	34.5 (3.8)	MDD	single center	no	DSM IV	CIDI	from the third trimester of pregnancy to 6 months postnatal.	no	outpatients	6	NSW Institute of Psychiatry, Pfizer Australia, NHMRC, and NSW Department of Health.	HAM-D, MADRS, EPDS	no response outcome	no remission outcome	Low
(Rondanelli, Giacosa et al. 2010) [44]	EPA + DHA	1,67g/day+0,83g/day	Italy	ITT	22	22 (100)	84.9 (6.9)	MDD, dysthymia	single center	no	DSM-IV	NA	no comorbidities	no	Inpatients	8	Regione Lombardia	GDS, SF-36	no response outcome	no remission outcome	Low
(Rondanelli, Giacosa et al. 2010) [44]	PLB		Italy	ITT	24	24 (100)	83.0 (7.3)	MDD, dysthymia	single center	no	DSM-IV	NA	no comorbidities	no	Inpatients	8	Regione Lombardia	GDS, SF-36	no response outcome	no remission outcome	Low
(Roohi-Azizi, Arabzadeh et al. 2017) [97]	CIT + CTP	200 mg/day+20–40 mg/day	Iran	PP	25	16 (64)	36 (8.11)	MDD	single center	no	DSM-IV-TR	NA	no comorbidities	no	outpatients	6	NA	HAM-D 17 item, response rate, remission rate	reduction of ≥50% in HAM-D	HAM-D score≤ 7	Some concerns
(Roohi-Azizi, Arabzadeh et al. 2017) [97]	PLB + CTP	20–40 mg/day	Iran	PP	25	19 (76)	35.8 (11.1)	MDD	single center	no	DSM-IV-TR	NA	no comorbidities	no	outpatients	6	NA	HAM-D 17 item, response rate, remission rate	reduction of ≥50% in HAM-D	HAM-D score≤ 7	Some concerns
(Rudzki, Ostrowska et al. 2019) [179]	LP299v + TAU	2 capsules/day; each capsule contains 10×109CFU of Lactobacillus Plantarum 299v	Poland	PP	30	23 (76.7)	39.13 (9.96)	MDD	single center	no	DSM-IV-TR	NA	no comorbidities	25 out of 30 escitalopram, 4 out of 30 sertraline, 1 out of 30 paroxetine	outpatients	8	Medical University of Bialystok, Poland.	HAM-D 17 item	no response outcome	no remission outcome	Some concerns
(Rudzki, Ostrowska et al. 2019) [179]	PLB + TAU	NA	Poland	PP	30	20 (66.7)	38.9 (12)	MDD	single center	no	DSM-IV-TR	NA	no comorbidities	23 out of 30 escitalopram, 4 out of 30 sertraline, 1 out of 30 paroxetine, 2 out of 30 fluoxetine	outpatients	8	Medical University of Bialystok, Poland.	HAM-D 17 item	no response outcome	no remission outcome	Some concerns
(Ryszewska-Pokraśniewicz, Mach et al. 2018) [180]	mg+FLUO	120 mg/day+20–40 mg/day	Poland	PP	17	11 (65)	48.1 (15.5)	MDD	single center	no	ICD-10	NA	no comorbidities	no	outpatients	8	National Science Center [Narodowe Centrum Nauki], Medical University ofWarsaw and Institute of Pharmacology PAS, Kraków	HAM-D-21 item, HAM-A, CGI-S, response rate, remission rate	reduction of 50% or more in the HAM-D	HAM-D score of 6 or less	Some concerns
(Ryszewska-Pokraśniewicz, Mach et al. 2018) [180]	PLB + FLUO	20–40 mg/day	Poland	PP	20	10 (50)	49.7 (12.3)	MDD	single center	no	ICD-10	NA	no comorbidities	no	outpatients	8	National Science Center [Narodowe Centrum Nauki], Medical University ofWarsaw and Institute of Pharmacology PAS, Kraków	HAM-D-21 item, HAM-A, CGI-S, response rate, remission rate	reduction of 50% or more in the HAM-D	HAM-D score of 6 or less	Some concerns
(Saccarello, Montarsolo et al. 2020) [74]	SAMe + HEAL9	200 mg + 1 cp/day	Italy	ITT	46	38 (84.4)	48.6 (10.67)	MDD (mild to moderate)	single center	no	ICD-10		no comorbidities	no	outpatients	6	NA	ZSDS	no response outcome	no remission outcome	Low
(Saccarello, Montarsolo et al. 2020) [74]	PLB		Italy	ITT	44	35 (79.5)	47.5 (11.90)	MDD (mild to moderate)	single center	no	ICD-10		no comorbidities	no	outpatients	6	NA	ZSDS	no response outcome	no remission outcome	Low
(Sadeghi, Ghorayshi et al. 2023) [181]	EACS + SJW	100 mg+250 mg/2a day	Iran	PP	37 randomized, 23 analyzed	12 (52.2)	34.7 (8.65)	MDD	single center	no	DSM-IV-TR	SCID	no comorbidities	no	outpatients	8	Barij Essence Research Center	HAM-D	no response outcome	no remission outcome	High
(Sadeghi, Ghorayshi et al. 2023) [181]	FLUO	20 mg/2a day	Iran	PP	37 randomized, 28 analyzed	16 (57.1)	36.8 (11.15)	MDD	single center	no	DSM-IV-TR	SCID	no comorbidities	no	outpatients	8	Barij Essence Research Center	HAM-D	no response outcome	no remission outcome	High
(Sahoo, Jarnail et al. 2015) [45]	SER + EPA + DHA	50 mg+1 mg/day	India	PP	33 randomized, 30 analyzed	17 (56.7)	32.87 (10.22)	MDD	single center	no	ICD-10		3 out of 30 diabetes, 2 out of 30 hypertension	no	outpatients	8	NA	BSI	no response outcome	no remission outcome	High
(Sahoo, Jarnail et al. 2015) [45]	SER + 5HTP	50 mg+100 mg/day	India	PP	37 randomized, 30 analyzed	15 (50)	31.50 (10.59)	MDD	single center	no	ICD-10		4 out of 30 diabetes, 3 out of 30 hypertension	no	outpatients	8	NA	BSI	no response outcome	no remission outcome	High
(Sahoo, Jarnail et al. 2015) [45]	SER	50 mg/day	India	PP	33 randomized, 30 analyzed	16 (53.3)	31.23 (9.10)	MDD	single center	no	ICD-10		1 out of 30 diabetes, 1 out of 30 hypertension	no	outpatients	8	NA	BSI	no response outcome	no remission outcome	High
(Sahraian, Ghanizadeh et al. 2015) [182]	vitC + CTP	1g/day+up to 60 mg/day	Iran	ITT	21	15 (71.4)	32.7 (8.4)	MDD	single center	no	DSM-IV	NA	no comorbidities	no	outpatients	8	Shiraz University of Medical Sciences	HDRS-21 item, BDI, response rate, remission rate	improvement of 50% or higher of HDRS scores	HAM-D total score of ≤7	Low
(Sahraian, Ghanizadeh et al. 2015) [182]	PLB + CTP	up to 60 mg/day	Iran	ITT	22	17 (77.2)	34.2 (10.3)	MDD	single center	no	DSM-IV	NA	no comorbidities	no	outpatients	8	Shiraz University of Medical Sciences	HDRS-21 item, BDI, response rate, remission rate	improvement of 50% or higher of HDRS scores	HAM-D total score of ≤7	Low
(Sahraian, Jelodar et al. 2016) [183]	FLUO + SAFF	1 cp + 20 mg	Iran	ITT	20	NA	NA	MDD	single center	no	DSM-IV-TR	SCID	no comorbidities	no	outpatients	4	NA	BDI	no response outcome	no remission outcome	Low
(Sahraian, Jelodar et al. 2016) [183]	FLUO + PLB	20 mg	Iran	ITT	20	NA	NA	MDD	single center	no	DSM-IV-TR	SCID	no comorbidities	no	outpatients	4	NA	BDI	no response outcome	no remission outcome	Low
(Sakurai, L. Carpenter et al. 2020) [75]	SAMe	3,200 mg/day	USA	ITT	16	NA	NA	MDD	single center	no	DSM IV	SCID-IV	no comorbidities	no	outpatients	6	NIH and the National Center for Complementary and Alternative Medicine (NCCAM)	HAM-D, CGI_S, response rate, remission rate	no response outcome	no remission outcome	Low
(Sakurai, L. Carpenter et al. 2020) [75]	ESC	20 mg/day	USA	ITT	23	NA	NA	MDD	single center	no	DSM IV	SCID-IV	no comorbidities	no	outpatients	6	NIH and the National Center for Complementary and Alternative Medicine (NCCAM)	HAM-D, CGI_S, response rate, remission rate	no response outcome	no remission outcome	Low
(Sakurai, L. Carpenter et al. 2020) [75]	PLB		USA	ITT	26	NA	NA	MDD	single center	no	DSM IV	SCID-IV	no comorbidities	no	outpatients	6	NIH and the National Center for Complementary and Alternative Medicine (NCCAM)	HAM-D, CGI_S, response rate, remission rate	no response outcome	no remission outcome	Low
(Sanmukhani, Satodia et al. 2014) [184]	CUR	1000 mg/day	India	ITT	20	15 (75)	37.7 (9.9)	MDD	single center	no	DSM-IV	NA	no comorbidities	diazepam (5 mg/day)	outpatients	6	Ministry of Health and FamilyWelfare, Government of Gujarat, India	HAM-D-17 item, CGI-S, response rate, remission rate	50% or more decrease in HAM-D score	HAM-D score≤7	Low
(Sanmukhani, Satodia et al. 2014) [184]	FLUO	20 mg/day	India	ITT	20	10 (50)	33.6	MDD	single center	no	DSM-IV	NA	no comorbidities	diazepam (5 mg/day)	outpatients	6	Ministry of Health and FamilyWelfare, Government of Gujarat, India	HAM-D-17 item, CGI-S, response rate, remission rate	50% or more decrease in HAM-D score	HAM-D score≤7	Low
(Sanmukhani, Satodia et al. 2014) [184]	CUR + FLUO	1000 mg+20 mg/day	India	ITT	20	14 (70)	40.4	MDD	single center	no	DSM-IV	NA	no comorbidities	diazepam (5 mg/day)	outpatients	6	Ministry of Health and FamilyWelfare, Government of Gujarat, India	HAM-D-17 item, CGI-S, response rate, remission rate	50% or more decrease in HAM-D score	HAM-D score≤7	Low
(Sarris, Byrne et al. 2018) [76]	SAMe + TAU	800 mg/day	Australia	ITT	55	43 (78.2)	43.4 (12.6)	MDD	single center	no	DSM-5	MINI 6.0	no comorbidities	SSRI, SNRI, no RI, mirtazapne, agomelatine	outpatients	8	National Health and Medical Re- search Council (NHMRC), FIT-BioCeuticals, NHMRC Fellowship, NHMRC Senior Principal Research Fellowship	MADRS, BDI-II, response rate, remission rate	MADRS decrease of ≥50%	MADRS score < 10	Low
(Sarris, Byrne et al. 2018) [76]	PLB + TAU	NA	Australia	ITT	52	39 (75)	45.9 (12.7)	MDD	single center	no	DSM-5	MINI 6.0	no comorbidities	SSRI, SNRI, no RI, mirtazapine, agomelatine	outpatients	8	National Health and Medical Re- search Council (NHMRC), FIT-BioCeuticals, NHMRC Fellowship, NHMRC Senior Principal Research Fellowship	MADRS, BDI-II, response rate, remission rate	MADRS decrease of ≥50%	MADRS score < 10	Low
(Sarris, Byrne et al. 2019) [185]	NUT	800 mg+500mcg+200mcg+100 mgEPA+656 mgDHA+200 mg+30 mg+100 mg+60 mg+40 mg/day	Australia	ITT	81	61 (75.3)	40.2 (12.3)	MDD	single center	no	DSM-5	MINI	no comorbidities	antidepressants	outpatients	8	Australian National Health and Medical Research Council project, FIT-BioCeuticals	MADRS, BDI-II, HAM-A, CGI-S	no response outcome	no remission outcome	Low
(Sarris, Byrne et al. 2019) [185]	PLB	NA	Australia	ITT	77	61 (79.2)	44.7 (12.6)	MDD	single center	no	DSM-5	MINI	no comorbidities	antidepressants	outpatients	8	Australian National Health and Medical Research Council project, FIT-BioCeuticals	MADRS, BDI-II, HAM-A, CGI-S	no response outcome	no remission outcome	Low
(Sarris, Fava et al. 2012) [186]	LI-160	900–1500 mg/day	Australia	ITT	35	NA	45 (12.3)	MDD	single center	no	DSM-IV		no comorbidities	no	outpatients	16	the mgH Depression Clinical and Research Program’s clinical practice	remission rate	no response outcome	HAM-D ≤ 7	Low
(Sarris, Fava et al. 2012) [186]	SER	50–100 mg/day	Australia	ITT	49	NA	46 (13.0)	MDD	single center	no	DSM-IV		no comorbidities	no	outpatients	16	the mgH Depression Clinical and Research Program’s clinical practice	remission rate	no response outcome	HAM-D ≤ 7	Low
(Sarris, Fava et al. 2012) [186]	PLB		Australia	ITT	40	NA	42 (13.0)	MDD	single center	no	DSM-IV		no comorbidities	no	outpatients	16	the mgH Depression Clinical and Research Program’s clinical practice	remission rate	no response outcome	HAM-D ≤ 7	Low
(Sarris, Murphy et al. 2020) [77]	SAMe	800 mg/day	Australia	PP	25	18 (72)	45.2 (12.6)	MDD	single center	no	DSM-5	MINI 6.0	no comorbidities	no	outpatients	8	National Health and Medical Research Council (NHMRC), FIT-BioCeuticals	MADRS, BDI-II, HAM-A, response rate, remission rate, BDI-II, HAM-A, SF-12, LSEQ, CGI-I, CGI-S	decrease > 50% in MADRS score from baseline	MADRS score <10	Some concerns
(Sarris, Murphy et al. 2020) [77]	PLB	NA	Australia	PP	24	21 (88)	42 (12.3)	MDD	single center	no	DSM-5	MINI 6.0	no comorbidities	no	outpatients	8	National Health and Medical Research Council (NHMRC), FIT-BioCeuticals	MADRS, BDI-II, HAM-A, response rate, remission rate, BDI-II, HAM-A, SF-12, LSEQ, CGI-I, CGI-S	decrease > 50% in MADRS score from baseline	MADRS score <10	Some concerns
(Schrader 2000) [187]	ZE117	500 mg/day	Germany	ITT	126	90 (71)	46 (19)	MDD	single center	no	ICD-10	NA	no comorbidities	no	outpatients	6	German medical insurance system	HAM-D-21 item	reduction by 50% from baseline or score ≤10	no remission outcome	Low
(Schrader 2000) [187]	FLUO + PLB	20 mg/day	Germany	ITT	114	67 (59)	47 (17)	MDD	single center	no	ICD-10	NA	no comorbidities	no	outpatients	6	German medical insurance system	HAM-D-21 item	reduction by 50% from baseline or score ≤10	no remission outcome	Low
(Schrader, Meier et al. 1998) [188]	ZE117	500 mg/day	Germany	ITT	81	58 (71.6)	NA	MDD	multi-center	no	ICD-10	NA	no comorbidities	no	outpatients	6	NA	HAM-D-21 item, CGI, response rate, remission rate	50% or greater reduction in HAM-D score or HAM-D score of 10 or less	improvement of more than 4 points in the HAMD score	Low
(Schrader, Meier et al. 1998) [188]	PROB	NA	Germany	ITT	81	50 (61.7)	NA	MDD	multi-center	no	ICD-10	NA	no comorbidities	no	outpatients	6	NA	HAM-D-21 item, CGI, response rate, remission rate	50% or greater reduction in HAM-D score or HAM-D score of 10 or less	improvement of more than 4 points in the HAMD score	Low
(Sempach, Schaub et al. 2025) [189]	SRN + TAU	2 g/day	Switzerland	PP	26 randomized, 22 analyzed	10 (45)	44.5 (11.3)	MDD (moderate to severe)	single center	no	ICD-10		no comorbidities	antidepressant as usual	inpatients	6	Gertrud Thalmann Foundation of the University Psychiatric Clinics (UPK) Basel (AS), and the Stiftung zur Forderung der gastroenterologischen und allgemeinen klinischen Forschung sowie der medizinischen Bildauswertung (AS)	HAM-D, STA-I	no response outcome	no remission outcome	Some concerns
(Sempach, Schaub et al. 2025) [189]	PLB + TAU		Switzerland	PP	26 randomized, 22 analyzed	10 (45)	42.5 (11.8)	MDD (moderate to severe)	single center	no	ICD-10		no comorbidities	antidepressant as usual	inpatients	6	Gertrud Thalmann Foundation of the University Psychiatric Clinics (UPK) Basel (AS), and the Stiftung zur Forderung der gastroenterologischen und allgemeinen klinischen Forschung sowie der medizinischen Bildauswertung (AS)	HAM-D, STA-I	no response outcome	no remission outcome	Some concerns
(Sepehrmanesh, Kolahdooz et al. 2016) [190]	vitD	50000 IU/week	Iran	PP	20	17 (85)	36.5 (8.7)	MDD	single center	no	DSM-IV	NA	no comorbidities	no	outpatients	8	NA	BDI	no response outcome	no remission outcome	Some concerns
(Sepehrmanesh, Kolahdooz et al. 2016) [190]	PLB	NA	Iran	PP	20	17 (85)	36.1 (6.9)	MDD	single center	no	DSM-IV	NA	no comorbidities	no	outpatients	8	NA	BDI	no response outcome	no remission outcome	Some concerns
(Sepehrmanesh, Omidi et al. 2016) [191]	CTP + FOL	20 mg+2.5 mg	Iran	ITT	45	26 (57.8)	35.11 (8.62)	MDD	single center	no	DSM-IV-TR		no comorbidities	no	outpatients	8	Kashan University of Medical Sciences	BDI-II, response rate	A decrease more of than 50% in a patient’s primary BDI-II score	no remission outcome	Low
(Sepehrmanesh, Omidi et al. 2016) [191]	CTP + PLB	20 mg	Iran	ITT	45	30 (66.7)	36.35 (10.49)	MDD	single center	no	DSM-IV-TR		no comorbidities	no	outpatients	8	Kashan University of Medical Sciences	BDI-II, response rate	A decrease more of than 50% in a patient’s primary BDI-II score	no remission outcome	Low
(Shafiee-Kandjani, Khalili et al. 2023) [192]	SER + LAV + DRA	100 mg+5ml/day	Iran	ITT	35	10 (29)	44.3 (10.2)	MDD	single center	no	DSM-IV	SCID for DSM-IV	no comorbidities	no	outpatients	6	Research Center of Psychiatry and Behavioral Sciences, Tabriz University of Medical Sciences, Iran		no response outcome	no remission outcome	High
(Shafiee-Kandjani, Khalili et al. 2023) [192]	SER + PLB	1000 mg/day	Iran	ITT	35	11 (31.5)	45.2 (10.8)	MDD	single center	no	DSM-IV	SCID for DSM-IV	no comorbidities	no	outpatients	6	Research Center of Psychiatry and Behavioral Sciences, Tabriz University of Medical Sciences, Iran		no response outcome	no remission outcome	High
(Shahmansouri, Farokhnia et al. 2014) [65]	SAFF	30 mg/day	Iran	PP	22 randomized, 20 analyzed	11 (55)	52.05 (8.92)	MDD	single center	no	DSM-IV-TR	NA	performing percutaneous coronary intervention (PCI).	no	outpatients	6	Tehran University of Medical Sciences	HAM-D, remission rate, response rate	complete and partial response rates (50% and 25% decrease in HDRS score, respectively)	HDRS score <= 7	Some concerns
(Shahmansouri, Farokhnia et al. 2014) [65]	FLUO	40 mg/day	Iran	PP	22 randomized, 20 analyzed	14 (70)	53.10 (8.47)	MDD	single center	no	DSM-IV-TR	NA	performing percutaneous coronary intervention (PCI).	no	outpatients	6	Tehran University of Medical Sciences	HAM-D, remission rate, response rate	complete and partial response rates (50% and 25% decrease in HDRS score, respectively)	HDRS score <= 7	Some concerns
(Shahmoradi, Davarinejad et al. 2023) [193]	APH	2 cp/day	Iran	PP	27 randomized, 21 analyzed	21 (100)	36.64 (8.35)	MDD	single center	no	DSM-5	clinical interview	SSRI-induced sexual dysfunctions	no	outpatients	8	no sponsor		no response outcome	no remission outcome	Some concerns
(Shahmoradi, Davarinejad et al. 2023) [193]	PLB		Iran	PP	27 randomized, 19 analyzed	19 (100)	34.47 (8.79)	MDD	single center	no	DSM-5	clinical interview	SSRI-induced sexual dysfunctions	no	outpatients	8	no sponsor		no response outcome	no remission outcome	Some concerns
(Shamabadi, Fattollahzadeh-Noor et al. 2023) [194]	SER + THE	100 mg/day+200 mg/day	Iran	PP	30 randomized, 25 analyzed	13 (52)	34.44 (5.79)	MDD	single center	no	DSM-5	NA	no comorbidities	no	outpatients	6	Tehran University of Medical Sciences (TUMS)	HAM-D, remission rate, response rate	≥50 % reduction in HDRS score	HDRS score ≤7	Some concerns
(Shamabadi, Fattollahzadeh-Noor et al. 2023) [194]	SER + PLB	100 mg/day	Iran	PP	30 randomized, 25 analyzed	9 (36)	32.52 (6.47)	MDD	single center	no	DSM-5	NA	no comorbidities	no	outpatients	6	Tehran University of Medical Sciences (TUMS)	HAM-D, remission rate, response rate	≥50 % reduction in HDRS score	HDRS score ≤7	Some concerns
(Sharifan, Hosseini et al. 2017) [195]	EOPF	434 g/week	Iran	ITT	60	31 (51.7)	67.9 (9.6)	MDD	single center	no	DSM-IV-TR	SCID	no comorbidities	no	outpatients	24	no sponsor	BDI	no response outcome	no remission outcome	Low
(Sharifan, Hosseini et al. 2017) [195]	PLB		Iran	ITT	60	27 (45)	63.9 (7.9)	MDD	single center	no	DSM-IV-TR	SCID	no comorbidities	no	outpatients	24	no sponsor	BDI	no response outcome	no remission outcome	Low
(Shelton, Keller et al. 2001) [196]	SJW	900 mg/day, increased to 1200 mg/day	USA	ITT	98	64 (64.9)	41.4 (12.5)	MDD	multi-center	no	DSM-IV	NA	no comorbidities	zolpidem tartrate, allowed for sleep for the first 3 weeks of the trial) (up to 10 mg/d)	outpatients	8	Pfizer Inc., NIMH	HAM-D-17 item, CGI-S, HAM-A	HAM-D score of 12 or less (representing at least a 50% improvement in HAM-D scores from baseline) and a CGI-I score of 1 or 2.	HAM-D score of 7 or less with a CGI score of 1 or 2	Low
(Shelton, Keller et al. 2001) [196]	PLB	NA	USA	ITT	102	64 (62.8)	43.3 (13.7)	MDD	multi-center	no	DSM-IV	NA	no comorbidities	zolpidem tartrate, allowed for sleep for the first 3 weeks of the trial) (up to 10 mg/d)	outpatients	8	Pfizer Inc., NIMH	HAM-D-17 item, CGI-S, HAM-A	HAM-D score of 12 or less (representing at least a 50% improvement in HAM-D scores from baseline) and a CGI-I score of 1 or 2.	HAM-D score of 7 or less with a CGI score of 1 or 2	Low
(Sherwood Brown and Gabrielson 2012) [98]	CIT	2000 mg/day	USA	ITT	17	15 (53.6)	41.6 (9.9)	MDD, BD1, BD2, BD NOS	single center	no	DSM-IV	SCID-IV	amphetamine dependence	1 out of 28 lithium, 3 out of 28 anticonvulsants, 9 out of 28 antidepressants, 1 out of 28 antipsychotics, 1 out of 28 sedative, hypnotics or anxiolytics	outpatients	12	Stanley Medical Research Institute.	IDS-C scores	no response outcome	no remission outcome	Low
(Sherwood Brown and Gabrielson 2012) [98]	PLB		USA	ITT	14	7 (35.0)	34.0 (7.3)	MDD, BD1, BD2, BD NOS	single center	no	DSM-IV	SCID-IV	amphetamine dependence	1 out of 20 lithium, 1 out of 20 anticonvulsants, 1 out of 20 antipsychotics, 1 out of 28 sedative, hypnotics or anxiolytics	outpatients	12	Stanley Medical Research Institute.	IDS-C scores	no response outcome	no remission outcome	Low
(Shinto, Marracci et al. 2016) [46]	EPA + DHA	1.95g+1.35g/day	USA	ITT	21	19 (90.5)	50.7 (11.6)	MDD	single center	yes	DSM-IV	SCID for DSM-IV	multiple sclerosis (relapsing remitting and progressive types)	antidepressants	outpatients	4	National Institutes of Health/National Center for Complementary and Alternative Medicine; National Institutes of Health/National Center for Advancing Translational Research	MADRS, response rate	50% or greater improvement in MADRS	no remission outcome	Low
(Shinto, Marracci et al. 2016) [46]	PLB	NA	USA	ITT	18	17 (94.4)	51.9 (10)	MDD	single center	yes	DSM-IV	SCID for DSM-IV	multiple sclerosis (relapsing remitting and progressive types)	antidepressants	outpatients	4	National Institutes of Health/National Center for Complementary and Alternative Medicine; National Institutes of Health/National Center for Advancing Translational Research	MADRS, response rate	50% or greater improvement in MADRS	no remission outcome	Low
(Siwek, Dudek et al. 2010) [197]	ZNC + IMI	25 mg/day+140 mg/day	Poland	PP	30	19 (63)	46.2 (5.8)	MDD	single center	yes	DSM-IV	NA	no comorbidities	no	outpatients	12	Funds for Statutory Activity of Collegium Medicum, Jagiellonian University Krakow and the Institute of Pharmacology	HAM-D-17 item, BDI, CGI, MADRS, response rate, remission rate	“much” or “very much improved” in CGI scale plus at least 50% reduction of HADRS scores (HADRS/CGI criteria)	“very much improved” on the CGI scale plus scores on HADRS (HADRS/CGI criteria)≤7	Some concerns
(Siwek, Dudek et al. 2010) [197]	IMI + PLB	140 mg/day	Poland	PP	30	21 (70)	45.7 (6.1)	MDD	single center	yes	DSM-IV	NA	no comorbidities	no	outpatients	12	Funds for Statutory Activity of Collegium Medicum, Jagiellonian University Krakow and the Institute of Pharmacology	HAM-D-17 item, BDI, CGI, MADRS, response rate, remission rate	“much” or “very much improved” in CGI scale plus at least 50% reduction of HADRS scores (HADRS/CGI criteria)	“very much improved” on the CGI scale plus scores on HADRS (HADRS/CGI criteria) ≤7	Some concerns
(Su, Huang et al. 2003) [47]	EPA + DHA	9.6g/day	Taiwan	PP	12	10 (83)	35.2 (11.6)	MDD	single center	no	DSM-IV		NA	1 fluvoxamine, 1 trazodone, 3 moclobemide, 6 fluoxetine (23 mg/day fluoxetine equivalent)	outpatients	8	NSC	HAM-D-21 item	no response outcome	no remission outcome	Some concerns
(Su, Huang et al. 2003) [47]	PLB	NA	Taiwan	PP	10	8 (80)	42.3 (10.7)	MDD	single center	no	DSM-IV		NA	1 fluvoxamine, 1 trazodone, 2 moclobemide, 5 fluoxetine (17.1 mg/day fluoxetine equivalent)	outpatients	8	NSC	HAM-D-21 item	no response outcome	no remission outcome	Some concerns
(Su, Huang et al. 2008) [48]	EPA + DHA	3.4g/day	Taiwan	ITT	18	18 (100)	30.9 (3.9)	MDD with peripartum onset	single center	no	DSM-IV	NA	pregnant women	no	outpatients	8	National Science Council; Department of Health; and China Medical University and Hospital in Taiwan	HAM-D, EPDS, BDI	improvement of at least 50% in HAM-D	HAM-D score of 7 or less	Low
(Su, Huang et al. 2008) [48]	PLB	NA	Taiwan	ITT	18	18 (100)	31.3 (5.7)	MDD with peripartum onset	single center	no	DSM-IV	NA	pregnant women	no	outpatients	8	National Science Council; Department of Health; and China Medical University and Hospital in Taiwan	HAM-D, EPDS, BDI	improvement of at least 50% in HAM-D	HAM-D score of 7 or less	Low
(Sultana and Rahman 2023) [198]	MG	200 mg	Bangladesh	PP	45 randomized, 40 analyzed	31 (68.89)	30.68 (10.22)	MDD	single center	no	DSM-5		no comorbidities	SSRIs	outpatients	8	NA		no response outcome	no remission outcome	Some concerns
(Sultana and Rahman 2023) [198]	PLB		Bangladesh	PP	45 randomized, 38 analyzed	29 (64.44)	32.56 (11.79)	MDD	single center	no	DSM-5		no comorbidities	SSRIs	outpatients	8	NA		no response outcome	no remission outcome	Some concerns
(Szegedi, Kohnen et al. 2005) [199]	WS5570	900 mg/day	Germany	ITT	122	85 (70)	49 (11)	MDD	single center	no	DSM-IV	NA	no comorbidities	no	outpatients	8	Dr Willmar Schwabe Pharmaceuticals, manufacturer of WS 5570	HAMD-17, MADRS, BDI	no response outcome	no remission outcome	Low
(Szegedi, Kohnen et al. 2005) [199]	PAR	20 mg/day	Germany	ITT	122	83 (68)	45.5 (11.5)	MDD	single center	no	DSM-IV	NA	no comorbidities	no	outpatients	8	Dr Willmar Schwabe Pharmaceuticals, manufacturer of WS 5571	HAMD-17, MADRS, BDI	no response outcome	no remission outcome	Low
(Talaei, Hassanpour Moghadam et al. 2015) [66]	SAFF + TAU	30 mg/day	Iran	PP	23 randomized, 20 analyzed	18 (90)	35.9 (7.1)	MDD	single center	no	DSM-IV	SCID for DSM-IV	no comorbidities	fluoxetine or sertraline or citalopram (20 mg/day, 50 mg/day, 20 mg/day)	outpatients	4	Vice Chancellor of Research, Mashhad University of Medical Sciences	BDI, BAI, GHQ	no response outcome	no remission outcome	Some concerns
(Talaei, Hassanpour Moghadam et al. 2015) [66]	PLB + TAU		Iran	PP	23 randomized, 20 analyzed	16 (80)	36.5 (7.67)	MDD	single center	no	DSM-IV	SCID for DSM-IV	no comorbidities	fluoxetine or sertraline or citalopram (20 mg/day, 50 mg/day, 20 mg/day)	outpatients	4	Vice Chancellor of Research, Mashhad University of Medical Sciences	BDI, BAI, GHQ	no response outcome	no remission outcome	Some concerns
(Talaei, Noori et al. 2023) [200]	SER + CUR	40 mg/day	Iran	PP	23 randomized, 20 analyzed	18 (78.2)	36.60 (10.54)	MDD	single center	no	DSM-5		no comorbidities	no	outpatients	8	Mashhad University of Medical Sciences and Ibn-e-Sina hospital	BDI-II, BAI	no response outcome	no remission outcome	Some concerns
(Talaei, Noori et al. 2023) [200]	SER + PLB		Iran	PP	22 randomized, 20 analyzed	12 (52.17)	36.50 (11.53)	MDD	single center	no	DSM-5		no comorbidities	no	outpatients	8	Mashhad University of Medical Sciences and Ibn-e-Sina hospital	BDI-II, BAI	no response outcome	no remission outcome	Some concerns
(Targum, Cameron et al. 2018) [78]	SAMe + TAU	800 mg/day	USA	ITT	118	78 (66.1)	48.3 (10.6)	MDD	multi-center	no	DSM-IV-TR	NA	no comorbidities	25 out of 118 sertraline, 17 out of 118 bupropion, 23 out of 118 citalopram, 14 out of 118 escitalopram, 20 out of 118 fluoxetine, 12 out of 118 venlafaxine, 4 out of 118 paroxetine, 4 out of 118 duloxetine, 3 out of 118 desvenlafaxine, 2 out of 118 mirtazapine	outpatients	6	NA	HAM-D-17 item, MADRS, CGI-S	no response outcome	no remission outcome	Low
(Targum, Cameron et al. 2018) [78]	PLB + TAU	NA	USA	ITT	116	79 (68.1)	46.1 (10.89)	MDD	multi-center	no	DSM-IV-TR	NA	no comorbidities	30 out of 116 sertraline, 21 out of 116 citalopram, 20 out of 116 escitalopram, 12 out of 116 fluoxetine, 9 out of 116 bupropion, 4 out of 116 venlafaxine, 9 out of 116 paroxetine, 3 out of 116 duloxetine, 2 out of 116 desvenlafaxine, 2 out of 116 mirtazapine	outpatients	6	NA	HAM-D-17 item, MADRS, CGI-S	no response outcome	no remission outcome	Low
(Tarutani, Omori et al. 2022) [201]	LS	7 g	Japan	ITT	9	8 (89)	53 (22.95)	MDD	single center	no	ICD-10	NA	no comorbidities	no	outpatients	24	Baika Women’s University, Doshisha Women’s College, and Osaka Institute of Clinical Psychiatry		no response outcome	no remission outcome	Low
(Tarutani, Omori et al. 2022) [201]	PLB		Japan	ITT	11	9 (82)	53 (21.98)	MDD	single center	no	ICD-10	NA	no comorbidities	no	outpatients	24	Baika Women’s University, Doshisha Women’s College, and Osaka Institute of Clinical Psychiatry		no response outcome	no remission outcome	Low
(Vaghef-Mehrabany, Ranjbar et al. 2021) [202]	INU	10 g/day	Iran	ITT	31	31 (100)	37.45 (6.77)	MDD	single center	no	DSM-5		obesity	no	outpatients	8	NA		no response outcome	no remission outcome	Low
(Vaghef-Mehrabany, Ranjbar et al. 2021) [202]	PLB		Iran	ITT	31	31 (100)	40.00 (8.6)	MDD	single center	no	DSM-5		obesity	no	outpatients	8	NA		no response outcome	no remission outcome	Low
(van Gurp, Meterissian et al. 2002) [203]	SJW	900–1800 mg/day	Canada	ITT	44	28 (63.6)	40.9 (11.6)	MDD	single center	no	DSM-IV	NA	no comorbidities	no	outpatients	12	St Mary’s Hospital Centre in Montreal, Que, Pfizer Canada	HAM-D-17 item, BDI	no response outcome	no remission outcome	Some concerns
(van Gurp, Meterissian et al. 2002) [203]	SER	50–100 mg/day	Canada	ITT	43	24 (58.5)	39.1 (10.2)	MDD	single center	no	DSM-IV	NA	no comorbidities	no	outpatients	12	St Mary’s Hospital Centre in Montreal, Que, Pfizer Canada	HAM-D-17 item, BDI	no response outcome	no remission outcome	Some concerns
(Varzaghani, Sharifi et al. 2022) [204]	PRO	1,000 mg/day	Iran	ITT	28	14 (53.8)	38.8 (10.6)	MDD	single center	no	DSM-5	SCID	no comorbidities	10 out of 28 citalopram, 12 out of 28 sertraline, 5 out of 28 fluoxetine, 5 out of 28 fluvoxamine	outpatients	6	The Academic Center for Education, Culture, and Research (ACECR)	HAM-D, response rate	30% reduction in the depression score	no remission outcome	High
(Varzaghani, Sharifi et al. 2022) [204]	PLB		Iran	ITT	26	16 (57.1)	44.9 (11.5)	MDD	single center	no	DSM-5	SCID	no comorbidities	14 out of 26 citalopram, 9 out of 26 sertraline, 5 out of 26 fluoxetine, 3 out of 26 fluvoxamine	outpatients	6	The Academic Center for Education, Culture, and Research (ACECR)	HAM-D, response rate	30% reduction in the depression score	no remission outcome	High
(Vellekkatt, Menon et al. 2020) [87]	vitD3 + TAU	300000 IU	India	ITT	23	17 (73.9)	36.2 (12.3)	MDD	single center	no	DSM-5	MINI 6.0	no comorbidities	fluoxetine (22 patients), amitriptyline (1 patient)	inpatients and outpatients	12	Jawaharlal Institute of Post Graduate Medical Education and Research (JIPMER)	HAM-D-17 item, CGI-S, Quality of Life Enjoyment and Satisfaction Questionnaire-Short Form (QLES)	no response outcome	no remission outcome	Low
(Vellekkatt, Menon et al. 2020) [87]	PROB + TAU		India	ITT	23	14 (60.9)	35.8 (11.2)	MDD	single center	no	DSM-5	MINI 6.0	no comorbidities	fluoxetine (19 patients), escitalopram (3 patients), mirtazapine (1 patient)	inpatients and outpatients	12	Jawaharlal Institute of Post Graduate Medical Education and Research (JIPMER)	HAM-D-17 item, CGI-S, Quality of Life Enjoyment and Satisfaction Questionnaire-Short Form (QLES)	no response outcome	no remission outcome	Low
(Verma and Das 2016) [205]	ZNC + TAU	20 mg/day	India	ITT	30	10 (33.4)	28.83 (6.61)	MDD	single center	no	ICD-10		no comorbidities	no	Inpatients	8	NA	HAM-D, BDI, HAM-A	no response outcome	no remission outcome	Low
(Verma and Das 2016) [205]	TAU		India	ITT	30	10 (33.4)	33.37 (10.69)	MDD	single center	no	ICD-10		no comorbidities	no	Inpatients	8	NA	HAM-D, BDI, HAM-A	no response outcome	no remission outcome	Low
(Vorbach, Arnoldt et al. 1997) [84]	LI-160	1800 mg/day	Germany	ITT	107	78 (73)	48.8 (12)	MDD	multi-center	no	ICD-10	NA	no comorbidities	no	outpatients	6	NA	HAM-D 17	reduction by 50% or more on HAM-D score	no remission outcome	Low
(Vorbach, Arnoldt et al. 1997) [84]	IMI	150 mg/day	Germany	ITT	102	76 (75)	50.1 (11.8)	MDD	multi-center	no	ICD-10	NA	no comorbidities	no	outpatients	6	NA	HAM-D 17	reduction by 50% or more on HAM-D score	no remission outcome	Low
(Wang, Liu et al. 2016) [206]	vitD3	50000 IU/week	China	ITT	362	141 (39)	NA	58.6% MDD, 41.4% VaD	multi-center	no	DSM-IV	NA	vitD deficiency and dialysis	no	inpatients	52	NA	BDI-II	no response outcome	no remission outcome	Low
(Wang, Liu et al. 2016) [206]	PLB	NA	China	ITT	364	210 (57.7)	NA	56% MDD, 44% VaD	multi-center	no	DSM-IV	NA	vitD deficiency and dialysis	no	inpatients	52	NA	BDI-II	no response outcome	no remission outcome	Low
(Wheatley 1997) [85]	LI-160	2400 mg/day	Germany	ITT	83	70 (84.3)	42	MDD	multi-center	no	DSM-IV	NA	no comorbidities	temazepam, zopiclone, zolpidem (10–20 mg/die, 7,5 mg/die, 5–10 mg/die)	outpatients	6	NA	HAM-D-17 item, response rate	HAM-D score of less than 10 or reduction of at least 50% in HAM-D score	no remission outcome	Low
(Wheatley 1997) [85]	AMI	75 mg/day	Germany	ITT	73	56 (76.6)	38	MDD	multi-center	no	DSM-IV	NA	no comorbidities	temazepam, zopiclone, zolpidem (10–20 mg/die, 7,5 mg/die, 5–10 mg/die)	outpatients	6	NA	HAM-D-17 item, response rate	HAM-D score of less than 10 or reduction of at least 50% in HAM-D score	no remission outcome	Low
(Woelk 2000) [207]	ZE	500 mg/day	Germany	ITT	157	112 (71)	46.5 (12.7)	MDD	multi-center	no	ICD-10	NA	no comorbidities	diazepam (up to 10 mg max 3 times in 6 weeks)	outpatients	6	Bayer AG, Beromed, Berlin	HAM-D, response rate	reduction by 50% or more on HAM-D score	no remission outcome	Low
(Woelk 2000) [207]	TAU	150 mg/day	Germany	ITT	167	119 (71)	45.4 (12.8)	MDD	multi-center	no	ICD-10	NA	no comorbidities	diazepam (up to 10 mg max 3 times in 6 weeks)	outpatients	6	Bayer AG, Beromed, Berlin	HAM-D, response rate	reduction by 50% or more on HAM-D score	no remission outcome	Low
(Wu, Yang et al. 2024) [49]	EPA + DHA	3.2 g	Taiwan	ITT	30	25 (83.3)	35	MDD	single center	no	DSM IV		no comorbidities	no	outpatients	12	National Science and Technology Council (NSTC), Taiwan; the China Medical University Hospital, Taichung, Taiwan	HAM-D-17 item, response rate, remission rate	a reduction in HRSD score of at least 50%	an HRSD score of less than 8	Low
(Wu, Yang et al. 2024) [49]	PLB		Taiwan	ITT	30	25 (83.3)	35	MDD	single center	no	DSM IV		no comorbidities	no	outpatients	12	National Science and Technology Council (NSTC), Taiwan; the China Medical University Hospital, Taichung, Taiwan	HAM-D-17 item, response rate, remission rate	a reduction in HRSD score of at least 50%	an HRSD score of less than 8	Low
(Wu, Zhu et al. 2015) [208]	YUE + FLUO	23 g/day+20 mg/day	China	PP	10	NA	45.70 (4.62)	MDD	single center	no	DSM-IV		no comorbidities	no	outpatients	1	Priority Academic Program Development of Jiangsu Higher Education Institutions (PAPD)	response and remission rates	a 30% decrease in HDRS-24 score from the baseline for this 7-day drug administration regimen.	a score of ≤8 on HDRS-24	Some concerns
(Wu, Zhu et al. 2015) [208]	FLUO + PLB	20 mg/day	China	PP	8	NA	47.38 (5.92)	MDD	single center	no	DSM-IV		no comorbidities	no	outpatients	1	Priority Academic Program Development of Jiangsu Higher Education Institutions (PAPD)	response and remission rates	a 30% decrease in HDRS-24 score from the baseline for this 7-day drug administration regimen.	a score of ≤8 on HDRS-24	Some concerns
(Yang, Wang et al. 2022) [50]	EPA + DHA + VEN	1440 mg+960 mg+75–225 mg/day	China	PP	36 randomized, 21 analyzed	21 (58.3)	26.33 (8.07)	MDD	single center	no	DSM-IV	SCID	no comorbidities	no	outpatients	12	The National Natural Science Foundation of China, the Science Foundation of Xiangya Hospital for Young Scholar, the Natural Science Foundation of Hunan Province, Key-Area Research and Development Program of Guangdong Province, and Key Research Project of Ningxia Hui Autonomous Region in 2021 (Major Project)	HAM-D, HAM-A, BDI	a 50% or greater decrease in HAMD or HAMA score	The absolute value of HAMD or HAMA score less than 8.	Some concerns
(Yang, Wang et al. 2022) [50]	PLB + VEN	75–225 mg/day	China	PP	36 randomized, 27 analyzed	25 (69.4)	27.11 (8.14)	MDD	single center	no	DSM-IV	SCID	no comorbidities	no	outpatients	12	The National Natural Science Foundation of China, the Science Foundation of Xiangya Hospital for Young Scholar, the Natural Science Foundation of Hunan Province, Key-Area Research and Development Program of Guangdong Province, and Key Research Project of Ningxia Hui Autonomous Region in 2021 (Major Project)	HAM-D, HAM-A, BDI	a 50% or greater decrease in HAMD or HAMA score	The absolute value of HAMD or HAMA score less than 8.	Some concerns
(Yu, Pei et al. 2015) [209]	CUR + ESC	2g/day+5–15 mg/day	China	PP	50	0	44.14 (8.02)	MDD	single center	no	DSM-IV	SCID	no comorbidities	no	outpatients	6	NA	HDRS-17, MADRS	50% or greater reduction on the HDRS-17 score	score HDRS-17 ≤7	Some concerns
(Yu, Pei et al. 2015) [209]	PLB + ESC	5–15 mg/day	China	PP	50	0	45.22 (7.68)	MDD	single center	no	DSM-IV	SCID	no comorbidities	no	outpatients	6	NA	HDRS-17, MADRS	50% or greater reduction on the HDRS-17 score	score HDRS-17 ≤7	Some concerns
(Zhang, Wang et al. 2018) [210]	vitD3	100,000 IU/week	China	ITT	56	8 (13.8)	38.3 (12.4)	MDD	single center	no	DSM IV		pulmonary tuberculosis	no	outpatients	8	NA	BDI-II, response rate	[BDI baseline – post]/baseline > 50%	no remission outcome	Low
(Zhang, Wang et al. 2018) [210]	PLB		China	ITT	64	12 (18.5)	40.2 (11.9)	MDD	single center	no	DSM IV		pulmonary tuberculosis	no	outpatients	8	NA	BDI-II, response rate	[BDI baseline – post]/baseline > 50%	no remission outcome	Low

### Risk of bias assessment

Ninety-five studies (58.3%) had low risk of bias; 45 (27.6%) had some concerns; and 23 (14.1%) were deemed high-risk (Tables 2 & S3).

### Systematic review: individual trial findings by molecule

The 163 studies included in the NMA reported on 137 individual nutraceutical/phytoceutical treatments (see Table 2 for full details). Studies were published between 1987 and 2025; 113 (68.9%) trials were monotherapy trials, and 50 (31.1%) were augmentation studies of TAU antidepressants. Altogether, 120 (73.6%) trials had placebo as a comparator and were double-blind, 43 (26.4%) trials were double-blind and had TAU antidepressants or another nutraceutical/phytoceutical as a comparator. The median trial duration was 8 weeks, interquartile range [IQR] = 6–10 weeks. Included in the meta-analyzed studies were 15,757 adults (median sample size, n = 33; IQR = 20, 52), median age = 43.4 years (IQR = 36.5, 47.9), and 66.4% were female. Altogether, only 6/163 [3.6%] trials were conducted in patients with treatment-resistant depression (TRD). S12 summarizes dropouts, adverse outcomes, inefficacy, and safety-related outcomes across different treatments/studies.

Twenty studies (n = 8483) assessed eicosapentaenoic acid (EPA) [32–38, 39, 40, 41–50]. Sixteen studies (n = 7117) evaluated docosahexaenoic acid [32, 35–37, 42–53], and thirteen studies (n = 11,968) reported on saffron (Crocus Sativus) [54–57, 58–64, 65, 66]. Twelve studies (n = 14,840) appraised S-adenosylmethionine [67–78] and eight studies (n = 10,166) considered St. John’s wort (Hypericum Perforatum) extract LI-160 [79–85].

### Network meta-analysis: efficacy, depressive, and anxious symptoms change from baseline

#### Node-1, depressive symptoms

**Fig. 2:**
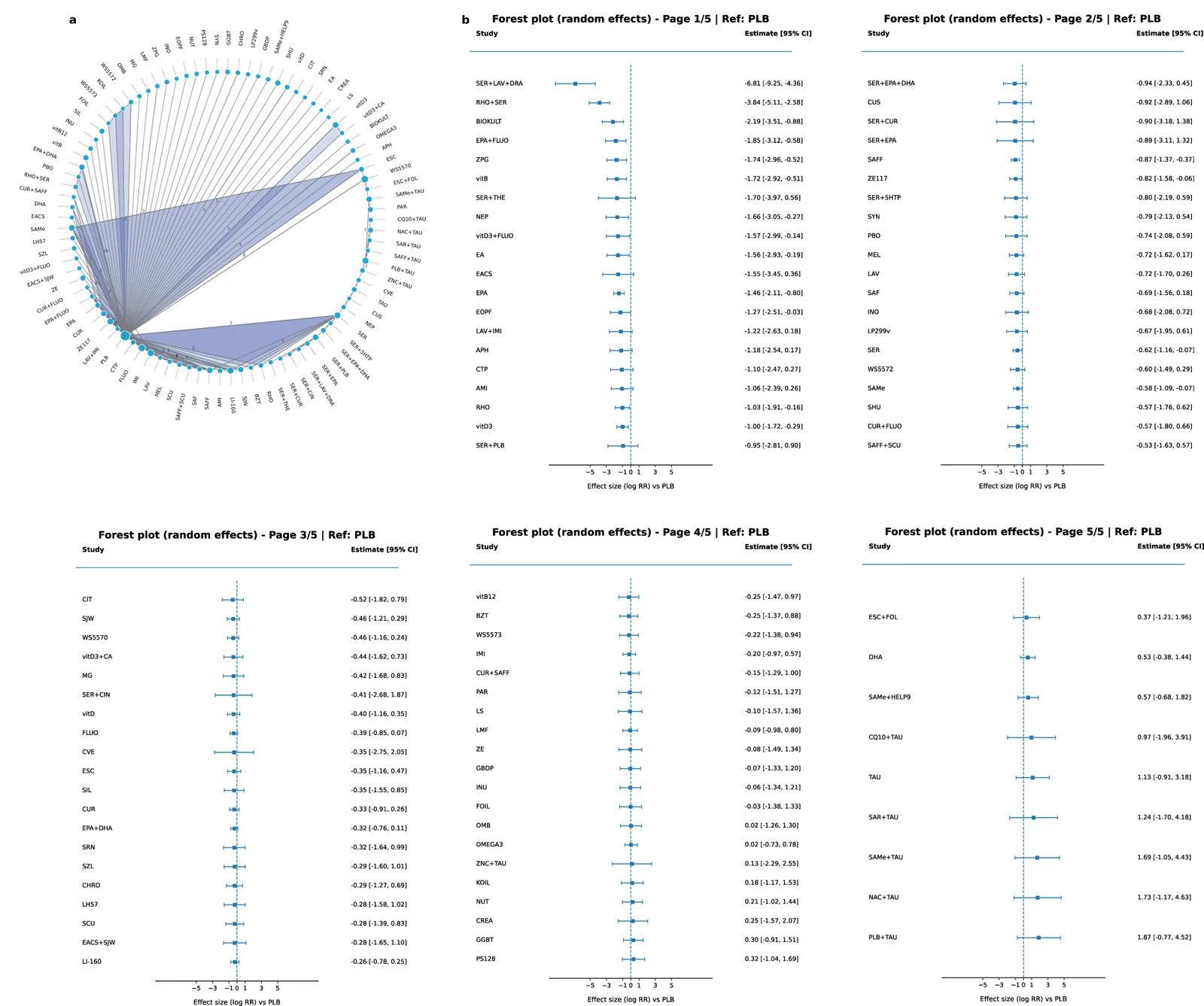
Efficacy node 1 (based on SMD). **a** Netgraph. The line thickness is related to the sample size of the studies in the network. Darker shadows indicate a larger number of comparisons. **b** Forest plot (reference is placebo).

Altogether, 149 studies yielded 197 pairwise comparisons encompassing 13,530 people and informing about 121 individual treatments. The comparisons formed 13 networks (S5). We analyzed the largest subnetwork, which included 131 RCTs informing about 91 treatments and 179 pairwise comparisons. Eighteen treatments’ efficacy was significantly higher than placebo: Sertraline+Lavandula Angustifolia+Dracocephalum ruyschiana (k = 1, n = 35; SMD = −6.81;95%C.I. = −9.25;−4.36); Rhodiola Rosea+Sertraline (k = 1, n = 34; SMD = −3.84;95%C.I. = −5.11;−2.58); Biokult (k = 1, n = 52; SMD = −2.19;95%C.I. = −3.51;−0.88); Tensilen^®^ (k = 1, n = 30; SMD = −2.14;95%C.I. = −3.47;−0.81); EPA+Fluoxetine (k = 1, n = 16; SMD = −1.85;95%C.I. = −3.12;−0.58); Zishen pingchan granules (k = 1, n = 89; SMD = −1.74;95%C.I. = −2.96;−0.51); vitamin B (k = 1, n = 170; SMD = −1.72;95%C.I. = −2.92;−0.51); Sertraline+L-theanine (k = 1, n = 46; SMD = −1.70;95%C.I. = −3.97;−0.56); Nepeta menthoides (k = 1, n = 33; SMD = −1.66;95%C.I. = −3.05;−0.27); vitamin D3+Fluoxetine (k = 1, n = 20; SMD = −1.57;95%C.I. = −2.99;−0.14); Ellagic acid (k = 1, n = 20; SMD = −1.56;95%C.I. = −2.93;−0.19); EPA (k = 20, n = 8483; SMD = −1.46;95%C.I. = −2.11;−0.80); Essential oil of Perilla frutescens (k = 1, n = 60; SMD = −1.27;95%C.I. = −2.51;−0.03); Rhodiola Rosea (k = 2, n = 41; SMD = −1.03;95%C.I. = −1.91;−0.16); vitamin D3 (k = 3, n = 344; SMD = −1.00;95%C.I. = −1.72;−0.29); Saffron (k = 13, n = 11,968; SMD = −0.87;95%C.I. = −1.37;−0.37); St. John’s wort extract ZE117 (k = 3, n = 207; SMD = −0.82;95%C.I. = −1.58;−0.06); S-adenosylmethionine (k = 12, n = 14,840; SMD = −0.58;95%C.I. = −1.09;−0.07) – S6 and Fig. 2a-b). Funnel plot visual inspection and Egger’s test excluded publication bias or small-study effect (S6). Head-to-head comparisons are detailed in the league table in e-material 1 (https://osf.io/cv65g/overview?view_only=a1716f5bf0bf47ce8d777096b44344b3). However, substantial heterogeneity emerged across the studies considered for efficacy measures (I^2^ = 85.3%; 95%C.I. = 81.9%; 88.0%), with global inconsistency (Q between designs = 186.91, p < 0.0001) – S7.

**Fig. 3:**
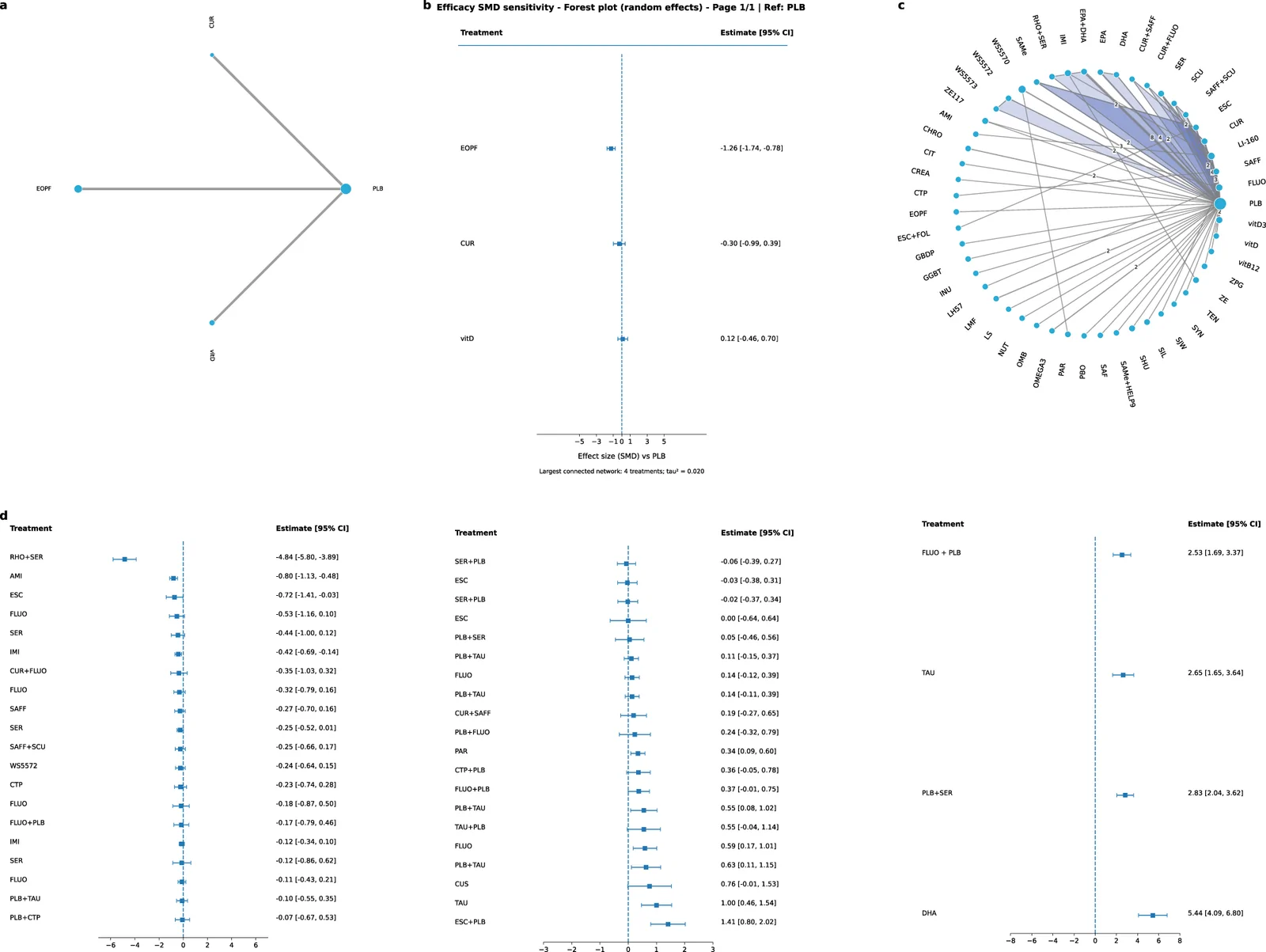
Efficacy node 1 – sensitivity analysis. **a** Netgraph of sensitivity analysis retaining only low risk of bias studies and non-sponsorship studies. The line thickness is related to the sample size of the studies in the network. Darker shadows indicate more comparisons. **b** Forest plots of sensitivity analysis retaining only low risk of bias studies and non-sponsorship studies. **c** Netgraph of sensitivity analysis retaining only low risk of bias studies and no potential effect modifiers outliers. The line thickness is related to the sample size of the studies in the network. Darker shadows indicate a larger number of comparisons. **d** Forest plots of sensitivity analysis retaining only low risk of bias studies and no potential effect modifiers outliers (reference is placebo).

Subgroup (e-material 2 - https://osf.io/cv65g/overview?view_only=a1716f5bf0bf47ce8d777096b44344b3, S10), transitivity (S8), and just a subset of sensitivity analyses (Fig. 3a-b, S9) satisfactorily explained the heterogeneity. Specifically, subgroup analysis assessing the validation of MDD diagnosis using (semi)structured clinical interviews (Q between groups = 1.76; p = 0.0967), geographical region (Q between groups = 12.82; p = 0.0050), single- vs. multicenter trials (Q between groups = 0.58; p = 0.4462), nutraceutical vs. phytoceutical(Q between groups = 1.59; p = 0.2070), inpatient vs outpatient setting (Q between groups = 1.54; p = 0.2152), TAU (antidepressants) augmentation with the nutraceuticals or phytoceuticals (e.g., TAU antidepressants +curcumin vs. TAU antidepressants +placebo) vs. no-augmentation of the nutraceutical or phytoceuticals (e.g., curcumin vs. TAU antidepressants only) (Q between groups = 5.52; p = 0.0188), TRD vs. non-TRD population (Q between groups = 5.39; p = 0.0202), and overt sponsorship (Q between groups = 3.18; p = 0.0747) resulted in I^2^ = 0.9%, yet with variable tau^2^ values (0.23–1.12). The baseline severity of depression, measured by the HAM-D total score or equivalent rating (W = 404.5, p = 0.79), length of trial (W = 362.5; p = 0.33), and mean age (W = 215; p = 0.112) did not differ between studies that outperformed placebo and those that did not.

Sensitivity analyses retained only low-risk-of-bias studies and those trials that clearly disclosed non-sponsorship (Fig. 3a-b, S9). In these analyses, only the superiority of the essential oil of Perilla frutescens (k = 1, n = 60; SMD = −1.27;95%C.I. = −2.51;−0.03) over placebo was confirmed in both sensitivity analyses.

S8 details the transitivity analyses for potential effect modifiers, including mean age, proportion of females, and baseline HAM-D severity (or equivalent ratings), across study arms. Fine-tuned sensitivity analyses of low-risk-of-bias studies, concurrently excluding outlier studies identified by any of the above transitivity analyses alongside with pre-DSM-IV studies (to minimize the risk of diagnostic shift), are presented in Fig. 3c-d and S9, highlighting the superior efficacy of EPA (k = 20, n = 8483; SMD = −1.67;95%C.I. = −2.38;−0.97), vitamin D3 (k = 3, n = 344; SMD = −1.59;95%C.I. = −2.71;−0.46), and St. John’s wort extract ZE117 (k = 3, n = 207; SMD = −1.02;95%C.I = −1.90;−0.15) compared to placebo. Notably, non-sponsored trials could not be included in this refined analysis due to insufficient data.

#### Node-1, anxious symptoms in major depressive disorder

Twenty-six studies yielded 30 pairwise comparisons, encompassing 2575 people and informing about 35 treatments; these comparisons formed 10 networks (S5). We analyzed the largest one. The latter included 12 RCTs informing about 11 treatments and 14 pairwise comparisons. Four treatments’ efficacy were statistically significantly superior to placebo: saffron (k = 1, n = 30; SMD = −1.18;95%C.I. = −1.82;−0.54); curcumin+saffron (k = 1, n = 26; SMD = −0.67;95%C.I. = −1.25;−0.10); Shugan granules (k = 1, n = 194; SMD = −0.59;95%C.I. = −0.98;−0.21); curcumin (k = 2, n = 89; SMD = −0.58;95%C.I. = −0.99;−0.18); the funnel plot ruled out publication bias or small-study effects (S6). Head-to-head comparisons are detailed in the league table in e-material 1. Low heterogeneity emerged across the studies considered for efficacy measures (I^2^ = 24.8%;95%C.I. = 0.0%;92.2%), with global consistency (Q-between designs = 2.57, p = 0.1090) (S7). Sensitivity analyses were conducted, excluding studies with high or medium risk of bias, confirming that saffron, curcumin, and Shugan granules outperformed placebo (S9).

There was insufficient information for any additional sensitivity analysis.

#### Node-2, depressive symptoms

One hundred forty-nine studies yielded 188 pairwise comparisons encompassing 13,530 people and informing about 59 treatment classes. S5 details the pertinent four subnetworks. We analyzed the largest dataset, which included 146 RCTs reporting on 53 classes of treatments (S4) and 185 pairwise comparisons. Fourteen classes of treatments’ efficacy were statistically significantly superior to placebo: selective serotonin reuptake inhibitors (SSRIs)+lamiaceae (k = 1, n = 35; SMD = −5.93;95%C.I. = −7.72;−4.14); crassulaceae+SSRI (k = 1, n = 66; SMD = −3.93;95%C.I. = −5.15;−2.70); long chain polyunsaturate fatty acids (LCPUFA) + SSRI (k = 2, n = 41; SMD = −2.00;95%C.I. = −2.98;−1.02); polyphenols (k = 1, n = 20; SMD = −1.56;95%C.I. = −2.90;−0.22); lamiaceae+tryciclic antidepressants (TCAs) (k = 1, n = 15; SMD = −1.44;95%C.I. = −2.70;−0.19); crassulaceae (k = 2, n = 80; SMD = −0.97;95%C.I. = −1.81;−0.13); lamiaceae(k = 6, n = 12,464;SMD = −0.83;95%C.I. = −1.40;−0.26); iridaceae (k = 13, n = 8787; SMD = −0.77;95%C.I. = −1.15;−0.38); coenzymes (k = 10, n = 9556; SMD = −0.72;95%C.I. = −1.13;−0.31); probiotics (k = 6, n = 8556; SMD = −0.68;95%C.I. = −1.21;−0.16); minerals (k=7, n=5,118; SMD = -0.93; 95%C.I.=-1.34;-0.51); vitamins (k = 11, n = 11,913; SMD = −0.66;95%C.I. = −1.03;−0.28); herbal compounds (k = 7; n = 12,292; SMD = −0.61;95%C.I. = −1.07;−0.15); LCPUFA (k = 21, n = 11,563; SMD = −0.49; 95%C.I = −0.78;-0.20); hypericaceae (k = 21, n = 11,378; SMD = −0.41;95%C.I. = −0.71;−0.12). No publication bias or small-study effect were detected (S6). E-material 1 details the head-to-head comparisons. Substantial between-studies heterogeneity (I^2^ = 86.8%;95%C.I. = 84.7%; 88.6%) and global inconsistency (Q between designs = 173.14, p = <0.0001) existed (S7). Sensitivity analyses were conducted by removing high- and medium-risk studies and overtly sponsored studies (S9), confirming that LCPUFA outperformed placebo.

#### Node-2, anxious symptoms in major depressive disorder

Twenty-six studies yielded 30 pairwise comparisons, encompassing 2575 people, and informed about 28 treatment classes. The comparisons formed 6 networks (S5). We analyzed the largest one, which included 14 RCTs across 12 treatment classes and 18 pairwise comparisons. Five classes of treatments’ efficacy were statistically significantly superior to placebo: SSRI+herbal compounds (k = 1, n = 50; SMD = −1.18;95%C.I. = −1.68;−0.87); iridaceae (k = 2, n = 60; SMD = −0.95;95%C.I. = −1.42;−0.48); zingiberaceae+iridaceae(k = 1; n = 26; SMD = −0.87;95%C.I. = −1.20;−0.14); herbal compounds (k = 3, n = 265; SMD = −0.60;95%C.I. = −0.87;−0.34); zingiberaceae (k = 2, n = 89; SMD = −0.59;95%C.I. = −0.96;−0.22), without publication bias or small-study effect (S6). Head-to-head comparisons are detailed in the league table in e-material 1. Low heterogeneity emerged (I^2^ = 17.1%;95%C.I. = 0.0%; 82.8%), with high global consistency (Q between designs = 4.32, p = 0.1154) (S7). Sensitivity analyses excluding high and medium-risk-of-bias confirmed that iridaceae, zingiberaceae+iridaceae, zingiberaceae, and herbal compounds outperformed placebo (S9).

Insufficient information precluded additional sensitivity analysis, including analyses on overtly sponsored studies.

### Network meta-analysis: treatment response

#### Node-1, treatment response

**Fig. 4:**
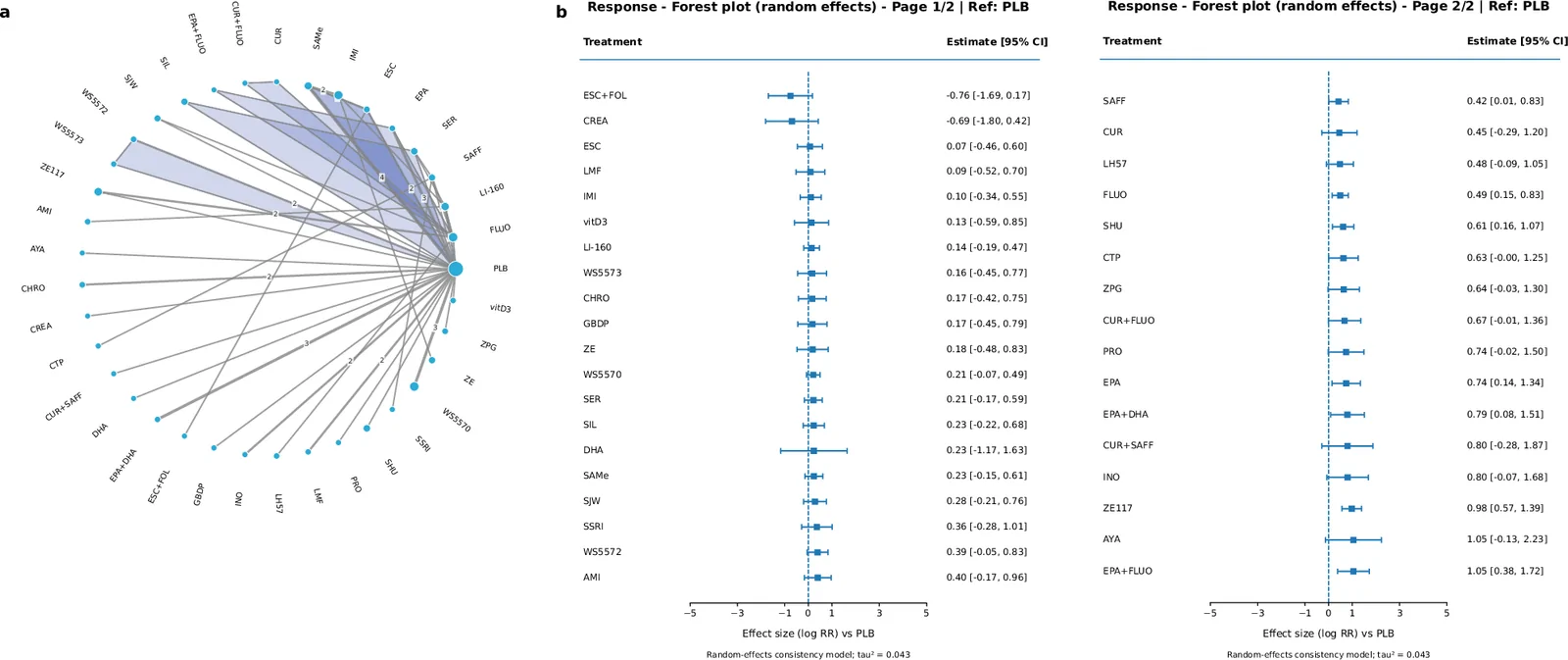
Response node 1 (based on RR). **a** Netgraph. The line thickness is related to the sample size of the studies in the network. Darker shadows indicate a larger number of comparisons. **b** Forest plots (reference is placebo).

**Fig. 5:**
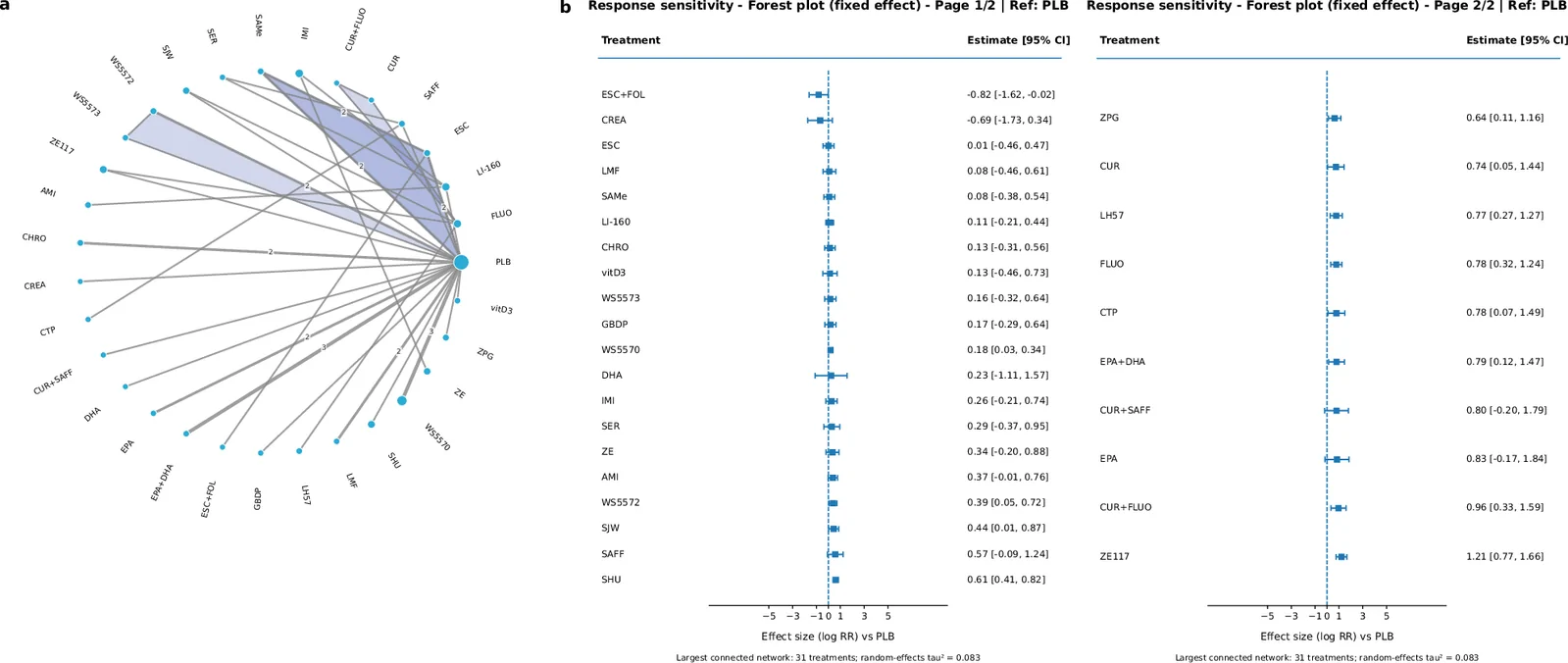
Response node 1 – sensitivity analysis retaining only low risk of bias studies. **a** Netgraph. The line thickness is related to the sample size of the studies in the network. Darker shadows indicate a larger number of comparisons. **b** Forest plots (reference is placebo).

Altogether, 72 studies yielded 86 comparisons, encompassing 7322 individuals and informing about 65 treatments. The comparisons formed 12 networks; we analyzed the largest one (S5), which included 68 RCTs informing about 37 treatments and 68 pairwise comparisons. Shugan granules, EPA, EPA + DHA, ZE117, and fluoxetine+EPA response significantly outperformed placebo (Fig. 4a-b without publication bias or small-study effect (S6). E-material 1 provides the corresponding league tables for head-to-head comparisons. No global heterogeneity (I^2^ = 0%;95%C.I. = 0.0%; 43.2%) or inconsistency (Q between designs = 0.20; p-value = 1) emerged (S7). Sensitivity analyses, which retained only low-risk-of-bias studies, confirmed main analysis results (Fig. 5a-b, S9), although there was insufficient information to concurrently exclude overtly sponsored trials.

#### Node-2, treatment response

Altogether, 72 studies yielded 82 comparisons, encompassing 7322 people and informing about 35 treatment classes. The comparisons formed one single network. No treatment class significantly outperformed placebo (S6), and no publication bias or small-study effects were observed (S6). E-material 1 provides the corresponding league tables for head-to-head comparisons. No overall heterogeneity (I^2^ = 0%;95%C.I. = 0.0%;34.8%) or global inconsistency (Q between designs = 0.59; p-value = 1) emerged (S7). Sensitivity analyses that retained only low-risk-of-bias studies confirmed the main analysis results (S9). Insufficient information precluded additional sensitivity analysis to exclude overtly sponsored studies. Subgroup analysis classes of nutraceutical and phytoceutical compounds (S4) could explain almost all the heterogeneity (I^2^ = 0.9%, tau^2^ = 0.87).

### Network meta-analysis: remission

#### Node-1, remission

Altogether, 48 studies yielded 62 comparisons, involving 4966 people and covering 51 treatments. The comparisons formed 11 networks; we analyzed the largest one (S5), which included 44 RCTs informing about 24 treatments and 44 pairwise comparisons. St. John’s wort extract WS5570, and ZE117 significantly outperformed placebo; however, there was no publication bias or small-study effect (S6). E-material 1 provides the corresponding league tables for head-to-head comparisons. Null heterogeneity (I^2^ = 0%;95%C.I. = 0.0%;53.6%) and negligible global inconsistency (Q between designs = 0.26; p-value = 0.9983) emerged (S7). Sensitivity analyses that retained only low-risk-of-bias studies confirmed the main analysis results, although insufficient information precluded the exclusion of overtly sponsored trials (S9).

#### Node-2, remission

Altogether, 48 studies yielded 62 comparisons, encompassing 4966 people and informing about 30 treatment classes. We analyzed the largest subnetwork (45 RCTs informing 24 treatments and 59 pairwise comparisons) from four subnetworks (S5). Hypericaceae and SSRI+aminoacids significantly outperformed placebo, despite publication bias and a small-study effect (S6). E-material 1 provides the league tables for head-to-head comparisons. No heterogeneity (I^2^ = 0%;95%C.I. = 0.0%;41.3%) or global inconsistency emerged (Q between designs = 0.21; p-value = 0.998) (S7).

Sensitivity analyses, which retained only low-risk-of-bias studies (yet were unable to concurrently exclude overtly sponsored trials due to the paucity of corresponding information), confirmed main analysis results (S9).

### Network meta-analysis: acceptability

#### Node-1, acceptability

**Fig. 6:**
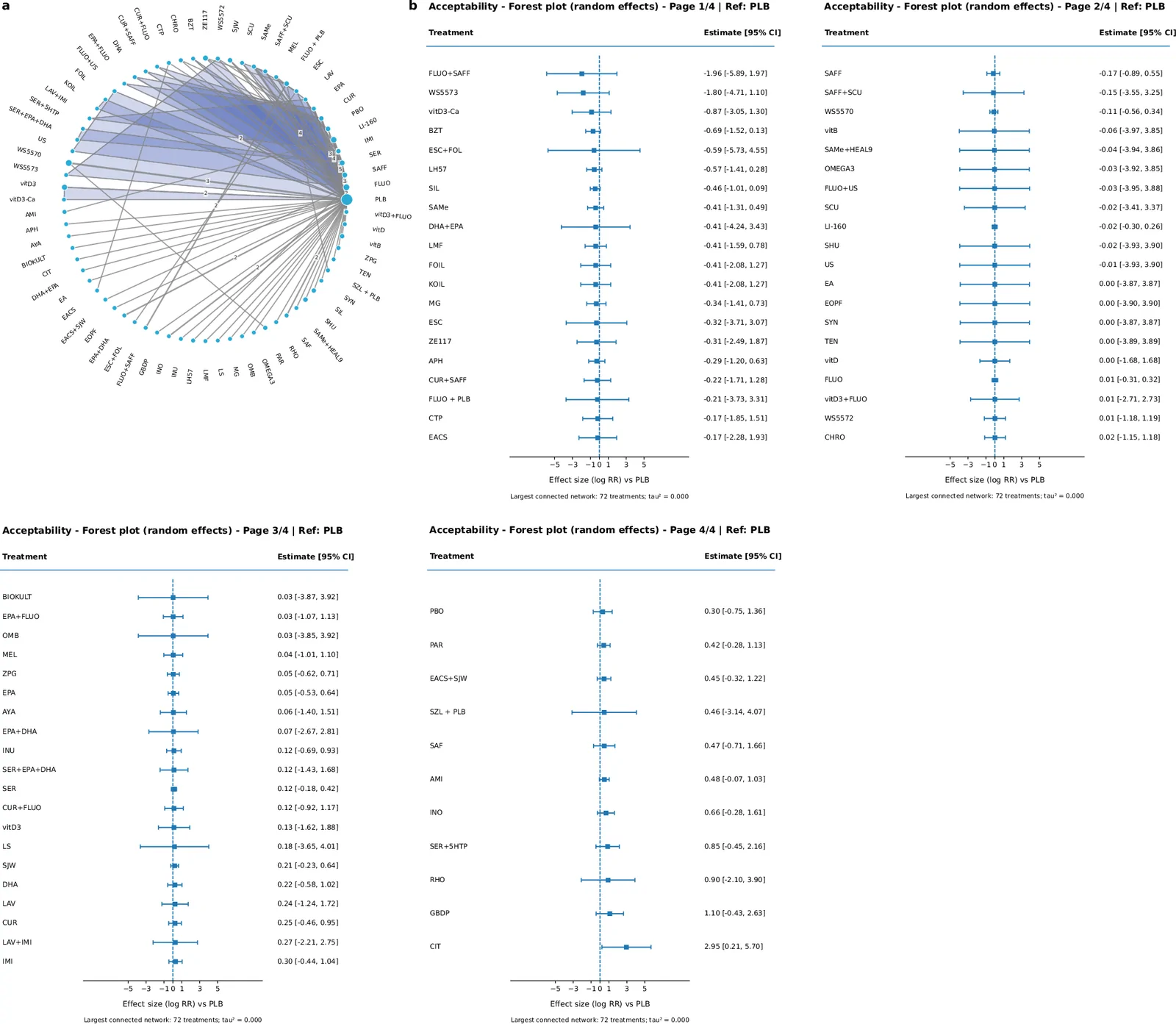
Acceptability node 1 (based on logRR). **a** Netgraph. The line thickness is related to the sample size of the studies in the network. Darker shadows indicate larger number of comparisons. **b** Forest plots (reference is placebo).

**Fig. 7:**
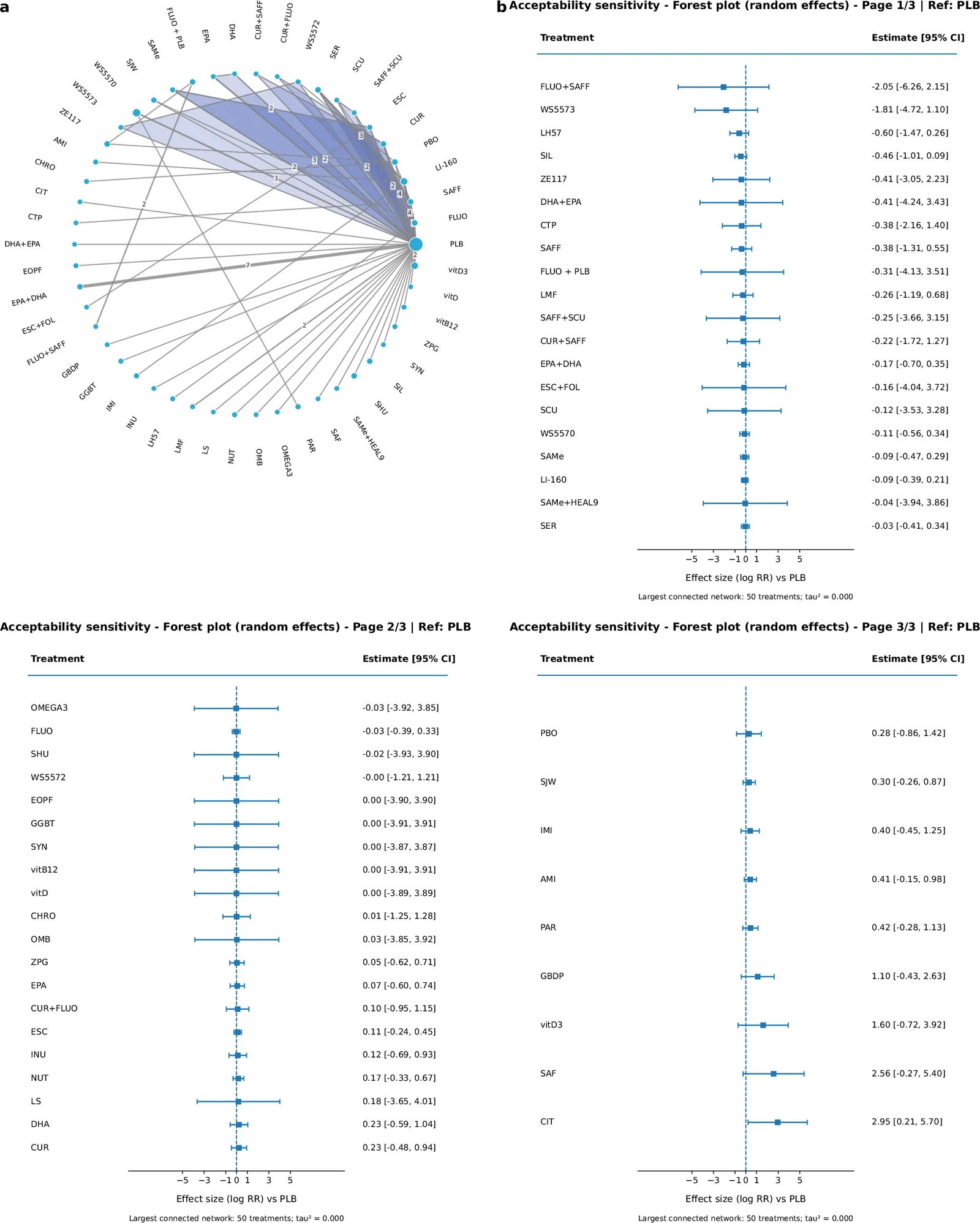
Acceptability node 1 – sensitivity analysis retaining only low risk of bias studies. **a** Netgraph. The line thickness is related to the sample size of the studies in the network. Darker shadows indicate a larger number of comparisons. **b** Forest plots (reference is placebo).

Altogether, 162 studies yielded 209 comparisons, involving 15,452 people and covering 138 treatments. The comparisons yielded 19 networks; we analyzed the largest, which included 116 RCTs encompassing 77 treatments and 161 pairwise comparisons, and the second largest, which included 18 studies, yielding 20 comparisons and informing about 16 treatments (e-Figs. 1–2 for e-table 6 in S5). Citicoline was associated with significantly more dropouts than placebo (Fig. 6a-b), without evidence of publication bias or a small-study effect (S6). E-material 1 provides the corresponding league tables for head-to-head comparisons. No heterogeneity (I^2^ = 0%;95%C.I. = 0.0%;34.8%) emerged, with negligible global inconsistency (Q between designs = 0.35; p = 1.0) (S7). Sensitivity analyses that retained only low-risk-of-bias studies but were unable to control for overtly sponsored trials confirmed main analysis results (Fig. 7a-b, S9).

#### Node-2, acceptability

Altogether, 162 studies yielded 201 comparisons, encompassing 15,452 participants and informing 73 treatment classes. The comparisons formed four networks; we analyzed the largest one, which included 158 RCTs reporting 66 treatments and 197 pairwise comparisons (Fig. 7a-b), without evidence of publication bias or a small-study effect (S6). No nutraceuticals or phytoceutical classes were less acceptable than placebo, while antidepressants (indirect comparisons) were associated with significantly more dropouts than placebo. E-material 1 provides the corresponding league tables for head-to-head comparisons. No heterogeneity (I^2^ = 0%;95%C.I. = 0.0%;28.7%) or global inconsistency (Q between designs = 0.34; p-value = 1) emerged (S7). Sensitivity analyses that retained only low-risk-of-bias studies confirmed the main analysis results, although insufficient information precluded the exclusion of overtly sponsored trials at the same time (S9).

### Network meta-analysis: tolerability

#### Node-1, tolerability

Altogether, 163 studies yielded 206 comparisons, encompassing 15,473 individuals and informing about 137 treatments. We analyzed the largest network of 18, comprising 121 RCTs, 81 treatments, and 162 pairwise comparisons (S5). No nutraceuticals or phytoceuticals’ tolerability differed statistically significantly from placebo; sertraline and imipramine were less tolerable than placebo (indirect comparisons), despite publication bias and a small-study effect (S6). E-material 1 provides the corresponding league tables for head-to-head comparisons. No overall heterogeneity (I^2^ = 0%;95%C.I. = 0.0%;47.0%) or global inconsistency emerged (Q between designs = 0.15; p = 1.0) (S7). Sensitivity analyses, which retained only low-risk-of-bias studies (yet were unable to concurrently exclude overtly sponsored trials due to the paucity of corresponding information), confirmed that no nutraceutical or phytoceutical treatment was worse than placebo (S9).

#### Node-2, tolerability

Altogether, 163 studies yielded 200 comparisons, encompassing 15,473 people and informing about 62 treatment classes. The comparisons formed 4 networks (S5). We analyzed the largest dataset, which included 159 RCTs reporting on 55 treatments and 196 pairwise comparisons. No nutraceutical or phytoceutical class’s tolerability differed statistically significantly from placebo; SSRIs and TCAs were less tolerable than placebo (indirect comparisons), without evidence of publication bias or a small-study effect (S6). E-material 1 provides the corresponding league tables for head-to-head comparisons. No overall heterogeneity (I^2^ = 0%;95%C.I. = 0.0%;36.2%) or global inconsistency (Q between designs = 0.45; p = 1.0) emerged (S7). Sensitivity analyses, which retained only low-risk-of-bias studies (yet were unable to concurrently exclude overtly sponsored trials due to the paucity of corresponding information), confirmed that no nutraceutical or phytoceutical treatment class performed worse than placebo (S9).

### Network meta-analysis: CINeMA confidence in evidence of the primary outcome

S11 and e-material 3 (https://osf.io/cv65g/overview?view_only=a1716f5bf0bf47ce8d777096b44344b3) present CINeMA methods and domain rating reasoning for co-primary outcomes, retaining just the low RoB2 studies included in the largest subnetworks. Regarding changes in depression symptoms, confidence in the evidence was low for 1752 comparisons and very low for the remaining 79. Regarding depression symptom treatment response, 536 low-evidence ratings were identified, and the remaining 26 comparisons were rated very low. Regarding acceptability, evidence was very low in 20 comparisons and low in the remaining 279.

Likewise, meta-regression analysis consistently documented a trend toward more negative SMDs (larger effect sizes) as the adapted AMSTAR-Plus score for low-RoB-RCTs documenting compounds reported by ≥2 primary studies decreased across different outcomes (e.g., total depressive symptoms change=β = 0.42, 95%CI = 0.11–0.72, p = <0.01).

## Discussion

### Comparison with previous evidence, and critical appraisal of the findings

The present NMA concurrently appraised 137 interventions, expanding the range of treatments included in the treatment guidelines [3] beyond probiotics alone [1]. The present NMA also refines the information provided by a previous NMA on the topic [4], which found EPA + DHA, SAMe, curcumin, zinc, tryptophan, and folate in augmentation to antidepressants, and that EPA + DHA, SAMe, curcumin, and saffron monotherapies outperform placebo. Specifically, whilst informative, the latter NMA [4] considered “[…depressive symptoms above the threshold according to validated scales.]” beyond MDD diagnosis according to DSM or ICD criteria. Further, that NMA [4] considered antidepressants “as the same intervention group”, performing sensitivity analysis by excluding high-risk-of-bias studies only, instead of retaining low-risk-of-bias studies only (thus excluding both the high- and medium-/some-concern risk-of-bias studies), which is critical considering that (S3) RoB2 assessment in this NMA resulted in a much higher proportion (94/163 = 57.7%) of low-risk-of-bias studies included in the analysis compared to the previous NMA (144/192 = 75%) [4]. In addition, the previous NMA [4] used odds ratios (ORs) instead of RRs, which may have overstated effect sizes because the outcomes are frequent, thereby reducing clinical interpretability. Additionally, we performed a sensitivity analysis on pre- vs. post-DSM-IV-criteria-complainant studies rather than on “publication year”. Overall, the present NMA investigated sources of heterogeneity through a broad range of subgroup and sensitivity analyses, whereas the previous NMA conducted only limited additional analyses beyond acceptability outcomes [4]. Moreover, after excluding studies with high or uncertain risk of bias, we identified a few compounds and observed inconsistencies in efficacy findings between continuous and binary outcomes, further underscoring the need for caution when interpreting the claimed efficacy of nutraceuticals and phytoceuticals.

Our analyses indicated the efficacy of EPA(SMD = −1.67;95%C.I. = −2.38;−0.97), vitamin D3(SMD = −1.59;−2.71;−0.46), and St. John’s wort extract ZE117 (SMD = −1.02;−1.90;−0.15) vs. placebo as the relatively more reliable findings. EPA and ZE117 also outperformed placebo for response, together with DHA and Shugan granules; ZE117 and St. John’s wort extract WS5570 achieved significantly higher remission rates than placebo. EPA (k = 20) and DHA (k = 16) findings were based on a higher number of trials and thus emerged as established evidence, whereas the other compounds (k < 5) with large or very large effects yielded exploratory, unreplicated findings. Higher fish intake has been associated with lower rates of depression, and LCPUFA, particularly EPA, are proposed as protective factors [40]. The essential oil of Perilla frutescens is a combination of LCPUFAs and anti-inflammatory agents, and is suggested to be a modulator of the neuro-immune network modulator [86]. The anti-inflammatory properties of vitamin D are mediated by its effects on neuronal calcium homeostasis, gene signaling, and protein expression [87]. St. John’s wort is considered effective for mild-to-moderate depression, with a benign profile of adverse drug events, including lack of sedation [82]. Nonetheless, it is remarkable that those compounds had effect sizes >1, which is highly unusual in any high-quality double-blind, placebo-controlled trial for any pharmaceutical compound, including depression [88]. CINeMA analyses also highlighted the very low credibility of the evidence.

Concerning anxious symptoms in MDD, low-risk-of-bias studies supported saffron (SMD = −1.18;95%C.I. = −1.82;−0.54), Shugan granules(SMD = −0.59;95%C.I. = −0.98;−0.21), and curcumin (SMD = −0.58;95%C.I. = −0.99;−0.18) superior efficacy vs. placebo. Although pseudospecificity cannot be ruled out, i.e., that anxiety symptoms are reduced due to an improvement in depressive symptoms, several potential mechanistic explanations can be put forward. For example, saffron’s active constituents, particularly crocin and safranal, inhibit the serotonin transporter, increasing synaptic serotonin levels - a putative mechanism like that exerted by the SSRIs [89]. Additionally, these compounds may modulate the reuptake of dopamine and norepinephrine, contributing to mood enhancement and stabilization, and reduce neuroinflammation and [89] receptor activation, particularly at the GluN2B subunit, which is implicated in glutamate-induced excitotoxicity [89]. By blocking excessive NMDA activation, curcumin may help protect hippocampal neurons from damage [90]. Additionally, saffron may upregulate brain**-**derived neurotrophic factor (BDNF) expression and enhance synaptic plasticity [90], which is often impaired in depression and anxiety. These effects would resemble those seen with rapid-acting antidepressants like ketamine [90]. Shugan granules are a composition of roots and herbal compounds, including licorice and peppermint [91]. Glycyrrhizin, a major active compound in licorice root, influences the expression of certain genes and proteins involved in the stress response, including regulation of the hypothalamic-pituitary-adrenal axis [92]. These findings suggest that glycyrrhizin may modulate neuroendocrine pathways to alleviate anxiety symptoms. The anxiolytic effects of peppermint essential oil are attributed to its chemical composition, which is primarily comprised of menthol, menthone, and linalool. These compounds have been shown to influence neurotransmitter systems involved in mood regulation. For instance, linalool modulates GABAergic activity, a process that plays a crucial role in reducing neuronal excitability and promoting relaxation [92]. Linalool is also the active ingredient of Lavandula angustifolia and is a calcium channel agent [93]. However, it must be noted that the efficacy of Shugan granules [94] and saffron [58] for anxious symptoms of depression relied on single RCTs, which, although rated as low risk of bias according to the Rob2 (S3), originated from Iran that consistently documented much higher-than-average SMDs (with non-significant 95%CIs overall), as detailed in S10 and e-material 2. It cannot be excluded that these results might be due to cultural differences in symptom reporting or specific dietary and environmental factors.

From a class-wide perspective (“node-2”), minerals resulted in the most efficacious class of compound to reduce depression and anxiety (SMD = −0.93; 95%CI = −1.34;−0.51). In particular, magnesium acts as a natural blocker of the NMDA receptor, helping to regulate the hypothalamic-pituitary-adrenal axis, which controls the stress response [95]. Zinc increases BDNF expression, promoting synaptic growth and resilience, and modulates glutamate receptor activity, including NMDA and AMPA receptors [96]. In a subgroup analysis of 78 studies, nutraceuticals (SMD = –0.6326; 95%CI = –0.6190;–0.2297) showed higher efficacy than phytoceuticals (SMD= –0.42; 95%CI = –0.88;–0.39). No matter the class (either nutraceuticals or phytoceuticals), such interventions showed much higher efficacy as TAU antidepressants augmentation (SMD = –0.89; 95%CI = –1.40;–0.38) rather than monotherapy without TAU antidepressants (SMD = –0.11; 95%CI = –0.62;0.41) overall, suggesting TAU antidepressants (usually an established antidepressant drug, namely: fluoxetine, imipramine, citalopram, amitriptyline, sertraline, escitalopram, mirtazapine, and paroxetine) as possible confounders of the antidepressant response.

Citicoline emerged as less acceptable than placebo. Citicoline is a naturally occurring nutrient (a precursor to phosphatidylcholine and acetylcholine) that supports brain cell health and membrane synthesis [97]. Studies have also explored its benefits in depressed patients dealing with substance use disorders (like methamphetamine or cocaine dependence), where it showed promise in stabilizing mood and reducing substance cravings [98]. Citicoline is widely considered safe and well-tolerated in clinical studies. However, it may cause mild side effects in some individuals, including mild gastrointestinal disturbances (stomach pain, constipation), transient headaches, restlessness, or sleep disturbances [97].

### Limitations

The first limitations of the present NMA include the lack of clarity regarding certain design features in many studies (including sponsorship) and implausibly high effect sizes for depression symptom reduction. Second, the CINeMA rated the credibility of the present NMA’s findings as low for most comparisons, underscoring that more high-quality studies are needed and that the results of this NMA should be interpreted with caution, especially considering that the higher  the AMSTAR score, the less negative the value of the SMD, meaning that the effect decreases when study quality increases (since “more negative” SMDs indicate larger differences between post- and pre-treatment). Thus, larger effect sizes should be interpreted with caution, since they rely on lower-quality studies. Third, results were substantially affected by heterogeneity and global inconsistency, and only small/insufficient subgroup analyses could be performed without overt study sponsorship. Fourth, we set an arbitrary threshold, based on the number of included studies, to select the most relevant subnetworks for each outcome. However, in the excluded subnetworks, many molecules relied on only one trial, seriously undermining the credibility of the unreplicated evidence.Fifth few safety outcomes were collected because such data were not available in the original studies. For example, the risk of suicidality and long-term safety outcomes could not be systematically assessed. These shortcomings should prompt future studies of nutraceuticals/phytoceuticals that assess and report in detail safety/tolerability (standardized adverse events) outcomes in addition to efficacy results.

Sixth, although in the meta-analyzed trials, <4% were reported to have had TRD, this disease category was not always assessed/reported, and this number is likely an underestimate. Future trials should formally assess and diagnose TRD and stratify analyses and, ideally, randomization by the presence of TRD.

### Conclusions

The term “nutraceutical” was coined in 1989 by Stephen DeFelice of the Foundation for Innovative Medicine and encompasses dietary supplements, isolated nutrients, and herbal products [99]. However, the link between “ante-litteram” nutraceuticals (and phytoceuticals) and health, including depression, has been noted since antiquity, tracing back to Hippocrates, who nonetheless distinguished between food and medicine [100]. The dichotomy between food- or plant-based treatments and well-established antidepressants has persisted in modern-day medicine, especially considering that nutraceutical and phytoceutical interventions have been questioned in terms of the credibility of their findings, often due to non-rigorous trial design, an uncertain putative mechanism of action, as well as a lack of regulatory agency oversight for quality assurance purposes, and inconsistent nomenclature systems [101, 102]; for example, in Japan such products are called “food for special health use”, and “Natural health products” in Canada, where a government database maintains up-to-date information on product safety and efficacy [99]. Furthermore, marketers of dietary supplements often tout nonspecific/nebulous “antidepressant” benefits such as “relief of nervous tension and or stress”, “improves mental and physical performance”, “enhances overall vitality”, or “body cleaners”, variables that are hard to qualify or quantify and to translate into tangible targets when treating MDD, warranting sensible definition of what “nutraceutical psychiatry” is, indeed [103]. While nutraceutical and phytoceutical compounds often appeal to many patients with depression, and their proponents sometimes conflate “natural” with “safe”, especially considering their traditional utilization reinforced by cultural lore (e.g., “used for centuries” by ancient civilizations), insights about the efficacy and acceptability of nutraceuticals and phytoceuticals represents a priority for the final recipients suffering from burdensome conditions such as MDD, and for regulatory agencies, warranting rigorous trials [104] and greater research investment to strengthen evidence base [105]. From this perspective, we reinforce the relevance of the present network analysis and aim to provide credible evidence on the matter.

## Supplementary information

Supplementary Material
