# Supplementary material for: Nutraceuticals and phytoceuticals for the treatment of major depressive disorder: a systematic review and network meta-analysis
Source: Mol Psychiatry. 2026 Sep 1;31(12):7013–60. doi: 10.1038/s41380-026-03759-1 (PMC13736492; doi:10.1038/s41380-026-03759-1)
Supplement: Supplementary file 1 [file 41380_2026_3759_MOESM1_ESM.docx]

**Nutraceuticals and phytoceuticals for the treatment of major depressive disorder: a systematic review and network meta-analysis**

Table of Contents

[S1a: Protocol amendments.	4](#_Toc231050236)

[S1b: Description of the search strategy	5](#_Toc231050237)

[S1.1: Citations by each source	12](#_Toc231050238)

[S2: Excluded studies, with the reason(s).	13](#_Toc231050239)

[S3a: Risk of bias assessment.	55](#_Toc231050240)

[S3.1: Legend of RoB2.	57](#_Toc231050241)

[S3b: AMSTAR-Plus score applied to low-RoB2-RCTs documenting compounds reported in at least 2 RCTs.	58](#_Toc231050242)

[S4: Legend of molecules and classes.	65](#_Toc231050243)

[S5: Subnetworks for each outcome and specific node analysis.	71](#_Toc231050244)

[S6: Analyses (Network plots, forest plots, funnel plots, and cumulative rankings).	223](#_Toc231050245)

[S7a: Evaluation of heterogeneity and inconsistency – Multiple outcomes and effect sizes.	273](#_Toc231050246)

[S7a.1: Heterogeneity	273](#_Toc231050247)

[S7a.2: Global consistency	274](#_Toc231050248)

[S7a.3: Local consistency – split of direct and indirect evidence.	275](#_Toc231050249)

[S7b: Meta-regression analysis.	285](#_Toc231050250)

[S8: Transitivity analyses.	286](#_Toc231050251)

[S9: Sensitivity analyses	369](#_Toc231050252)

[S10: Subgroup analyses	402](#_Toc231050253)

[S10.1: No (semi)structured clinical interview vs. (semi)structured clinical interview	402](#_Toc231050254)

[S10.2: Geographical region	402](#_Toc231050255)

[S10.3: Single center vs. Multi center	403](#_Toc231050256)

[S10.4: Phytoceutical vs. Nutraceutical	403](#_Toc231050257)

[S10.5: Clinical setting	403](#_Toc231050258)

[S10.6: Sponsorship	404](#_Toc231050259)

[S10.7: TAU augmentation vs no TAU augmentation	404](#_Toc231050260)

[S10.8: Classes of molecules	405](#_Toc231050261)

[S10.9: Treatment-resistant depression	405](#_Toc231050262)

[S11: Evaluating the confidence in NMA	406](#_Toc231050263)

[S11.1: CINeMA methods and reasoning	406](#_Toc231050264)

[S11.2 NMA confidence rating for *efficacy, symptom change from baseline*	407](#_Toc231050265)

[S11.3 NMA confidence rating for *response*.	523](#_Toc231050266)

[S11.4 NMA confidence rating for *acceptability*.	549](#_Toc231050267)

[S12: Dropouts, adverse outcomes, safety related outcomes.	569](#_Toc231050268)

**e-material 1**: League tables of node 1 and node 2 primary and secondary outcomes.

**e-material 2:** Subgroup analyses.

**e-material 3:** CINeMA indirectness and RoB2 charts for primary outcomes.

# **S1a**: Protocol amendments.

• We included nutraceutical and phytoceutical compounds based on the WFSBP/CANMAT guidelines and supplemented with additional compounds retrieved from the included RCTs (see Table 1 for the complete list).

• We assessed treatment response (as defined by study authors) as co-primary outcome and remission (as defined by study authors) as co-secondary outcome.

• We conducted sensitivity analyses excluding studies with high and some concerns risk of bias, as well as declared sponsored trials.

• We explored sources of heterogeneity through transitivity analyses (mean age, number of females per arm, and baseline depression severity) and conducted a sensitivity analysis excluding outliers and studies using pre-DSM-IV diagnostic criteria for MDD.

• We performed subgroup analyses stratifying studies by: sponsorship (sponsored vs. non-sponsored); type of compound (nutraceutical vs. phytoceutical); geographical region; study design (multisite vs. single-site); clinical setting; compound class; treatment-resistant depression population vs. non treatment-resistant depression population; and placebo performance (outperforming vs. non-outperforming placebo), also assessing between-group variance in mean age, trial duration, and baseline depression severity.

# **S1b**: Description of the search strategy

PubMed (up until July 1^st^, 2025)

#1: ("depress*"[Title/Abstract])

638,160 results

#2: (vitamin*[Title/Abstract]) OR (mineral*[Title/Abstract])) OR (nutrient*[Title/Abstract])) OR (food supplement*[Title/Abstract])) OR (meal replacement*[Title/Abstract])) OR (nutritional supplement*[Title/Abstract])) OR (health supplement*[Title/Abstract])) OR (multivitamin*[Title/Abstract])) OR ("omega 3"[Title/Abstract])) OR (fish oil*[Title/Abstract])) OR ("alpha lipoic acid"[Title/Abstract])) OR ("alpha linolenic acid"[Title/Abstract])) OR ("alpha linoleic acid"[Title/Abstract])) OR ("eicosapentaenoic"[Title/Abstract])) OR ("docosahexaenoic"[Title/Abstract])) OR (fatty acid*[Title/Abstract])) OR (amino acid*[Title/Abstract])) OR ("taurine"[Title/Abstract])) OR ("sadenosylmethionine"[Title/Abstract])) OR ("creatine"[Title/Abstract])) OR ("acetylcysteine"[Title/Abstract])) OR ("cysteine"[Title/Abstract])) OR (probiotic*[Title/Abstract])) OR (prebiotic*[Title/abstract])) OR (psychobiotic*[Title/Abstract])) OR (postbiotic*[Title/Abstract])) OR (synbiotic*[Title/abstract])) OR ("tryptophan"[Title/Abstract])) OR ("tocopherol"[Title/Abstract])) OR ("alphatocopherol"[Title/Abstract])) OR ("carotene"[Title/Abstract])) OR ("retinol"[Title/Abstract])) OR ("thiamine"[Title/Abstract])) OR ("riboflavin"[Title/Abstract])) OR ("niacin"[Title/Abstract])) OR ("niacinamide"[Title/Abstract])) OR ("nicotinic acid"[Title/Abstract])) OR ("pantothenic"[Title/Abstract])) OR (pyridox*[Title/Abstract])) OR ("biotin"[Title/Abstract])) OR ("methylfolate"[Title/Abstract])) OR (5-MTH*[Title/Abstract])) OR ("levomefolic acid"[Title/Abstract])) OR ("folate"[Title/Abstract])) OR ("folinic acid"[Title/Abstract])) OR ("folic acid"[Title/Abstract])) OR ("inositol"[Title/Abstract])) OR ("cyanocobalamin"[Title/Abstract])) OR ("methylcobalamin"[Title/Abstract])) OR ("cobalamin"[Title/Abstract])) OR ("ascorbic acid"[Title/Abstract])) OR ("cholecalciferol"[Title/Abstract])) OR ("iron"[Title/Abstract])) OR ("ferrous"[Title/Abstract])) OR ("tocopherols"[Title/Abstract])) OR ("trace element"[Title/Abstract])) OR ("calcium"[Title/Abstract])) OR ("phosphorus"[Title/Abstract])) OR ("magnesium"[Title/Abstract])) OR ("potassium"[Title/Abstract])) OR ("manganese"[Title/Abstract])) OR ("zinc"[Title/Abstract])) OR ("selenium"[Title/Abstract])) OR ("boron"[Title/Abstract])) OR ("chromium"[Title/Abstract])) OR ("lycopene"[Title/Abstract])) OR (isoflav*[Title/Abstract])) OR (flavonoid*[Title/Abstract])) OR (bioflavonoid*[Title/Abstract])) OR ("micronutrient"[Title/Abstract])) OR ("carnitine"[Title/Abstract])) OR ("herbal"[Title/Abstract])) OR ("herbal medicine"[Title/Abstract])) OR ("plant medicine"[Title/Abstract])) OR ("phytomedicine"[Title/Abstract])) OR ("supplement"[Title/Abstract])) OR ("st john s wort"[Title/Abstract])) OR ("kava"[Title/Abstract])) OR ("ginseng"[Title/Abstract])) OR ("saffron"[Title/Abstract])) OR ("curcumin"[Title/Abstract])) OR ("valerian"[Title/Abstract])) OR ("ginkgo"[Title/Abstract])) OR ("rhodiola"[Title/Abstract])) OR ("bacopa monniera"[Title/Abstract])) OR ("centella asiatica"[Title/Abstract])) OR ("crocus sativus"[Title/Abstract])) OR ("curcuma longa"[Title/Abstract])) OR ("hypericum perforatum"[Title/Abstract])) OR ("galphimia"[Title/Abstract])) OR ("ginkgo biloba"[Title/Abstract])) OR ("lavandula"[Title/Abstract])) OR ("matricaria"[Title/Abstract])) OR ("panax ginseng"[Title/Abstract])) OR ("passiflora incarnata"[Title/Abstract])) OR ("piper methysticum"[Title/Abstract])) OR ("rhodiola rosea"[Title/Abstract])) OR ("valeriana"[Title/Abstract])) OR ("withania somnifera"[Title/Abstract])) OR ("melissa officinalis"[Title/Abstract])) OR ("sarsasapogenin"[Title/Abstract])) OR ("mangosteen"[Title/Abstract])) OR ("scutellaria"[Title/Abstract])) OR ("silexan"[Title/Abstract])) OR ("citicoline"[Title/Abstract])) OR ("coenzyme q10"[Title/Abstract])) OR ("theanine"[Title/Abstract])

**3,172,346** results

#3

("depress*"[Title/Abstract]) AND (((vitamin*[Title/Abstract]) OR (mineral*[Title/Abstract])) OR (nutrient*[Title/Abstract])) OR (food supplement*[Title/Abstract])) OR (meal replacement*[Title/Abstract])) OR (nutritional supplement*[Title/Abstract])) OR (health supplement*[Title/Abstract])) OR (multivitamin*[Title/Abstract])) OR ("omega 3"[Title/Abstract])) OR (fish oil*[Title/Abstract])) OR ("alpha lipoic acid"[Title/Abstract])) OR ("alpha linolenic acid"[Title/Abstract])) OR ("alpha linoleic acid"[Title/Abstract])) OR ("eicosapentaenoic"[Title/Abstract])) OR ("docosahexaenoic"[Title/Abstract])) OR (fatty acid*[Title/Abstract])) OR (amino acid*[Title/Abstract])) OR ("taurine"[Title/Abstract])) OR ("sadenosylmethionine"[Title/Abstract])) OR ("creatine"[Title/Abstract])) OR ("acetylcysteine"[Title/Abstract])) OR ("cysteine"[Title/Abstract])) OR (probiotic*[Title/Abstract])) OR (prebiotic*[Title/abstract])) OR (psychobiotic*[Title/Abstract])) OR (postbiotic*[Title/Abstract])) OR (synbiotic*[Title/abstract])) OR ("tryptophan"[Title/Abstract])) OR ("tocopherol"[Title/Abstract])) OR ("alphatocopherol"[Title/Abstract])) OR ("carotene"[Title/Abstract])) OR ("retinol"[Title/Abstract])) OR ("thiamine"[Title/Abstract])) OR ("riboflavin"[Title/Abstract])) OR ("niacin"[Title/Abstract])) OR ("niacinamide"[Title/Abstract])) OR ("nicotinic acid"[Title/Abstract])) OR ("pantothenic"[Title/Abstract])) OR (pyridox*[Title/Abstract])) OR ("biotin"[Title/Abstract])) OR ("methylfolate"[Title/Abstract])) OR (5-MTH*[Title/Abstract])) OR ("levomefolic acid"[Title/Abstract])) OR ("folate"[Title/Abstract])) OR ("folinic acid"[Title/Abstract])) OR ("folic acid"[Title/Abstract])) OR ("inositol"[Title/Abstract])) OR ("cyanocobalamin"[Title/Abstract])) OR ("methylcobalamin"[Title/Abstract])) OR ("cobalamin"[Title/Abstract])) OR ("ascorbic acid"[Title/Abstract])) OR ("cholecalciferol"[Title/Abstract])) OR ("iron"[Title/Abstract])) OR ("ferrous"[Title/Abstract])) OR ("tocopherols"[Title/Abstract])) OR ("trace element"[Title/Abstract])) OR ("calcium"[Title/Abstract])) OR ("phosphorus"[Title/Abstract])) OR ("magnesium"[Title/Abstract])) OR ("potassium"[Title/Abstract])) OR ("manganese"[Title/Abstract])) OR ("zinc"[Title/Abstract])) OR ("selenium"[Title/Abstract])) OR ("boron"[Title/Abstract])) OR ("chromium"[Title/Abstract])) OR ("lycopene"[Title/Abstract])) OR (isoflav*[Title/Abstract])) OR (flavonoid*[Title/Abstract])) OR (bioflavonoid*[Title/Abstract])) OR ("micronutrient"[Title/Abstract])) OR ("carnitine"[Title/Abstract])) OR ("herbal"[Title/Abstract])) OR ("herbal medicine"[Title/Abstract])) OR ("plant medicine"[Title/Abstract])) OR ("phytomedicine"[Title/Abstract])) OR ("supplement"[Title/Abstract])) OR ("st john s wort"[Title/Abstract])) OR ("kava"[Title/Abstract])) OR ("ginseng"[Title/Abstract])) OR ("saffron"[Title/Abstract])) OR ("curcumin"[Title/Abstract])) OR ("valerian"[Title/Abstract])) OR ("ginkgo"[Title/Abstract])) OR ("rhodiola"[Title/Abstract])) OR ("bacopa monniera"[Title/Abstract])) OR ("centella asiatica"[Title/Abstract])) OR ("crocus sativus"[Title/Abstract])) OR ("curcuma longa"[Title/Abstract])) OR ("hypericum perforatum"[Title/Abstract])) OR ("galphimia"[Title/Abstract])) OR ("ginkgo biloba"[Title/Abstract])) OR ("lavandula"[Title/Abstract])) OR ("matricaria"[Title/Abstract])) OR ("panax ginseng"[Title/Abstract])) OR ("passiflora incarnata"[Title/Abstract])) OR ("piper methysticum"[Title/Abstract])) OR ("rhodiola rosea"[Title/Abstract])) OR ("valeriana"[Title/Abstract])) OR ("withania somnifera"[Title/Abstract])) OR ("melissa officinalis"[Title/Abstract])) OR ("sarsasapogenin"[Title/Abstract])) OR ("mangosteen"[Title/Abstract])) OR ("scutellaria"[Title/Abstract])) OR ("silexan"[Title/Abstract])) OR ("citicoline"[Title/Abstract])) OR ("coenzyme q10"[Title/Abstract])) OR ("theanine"[Title/Abstract])) AND (randomized controlled trial[Title/Abstract])

**9,076** results

Cochrane Central (Trials) (up until July 1^st^, 2025)

#1	MeSH descriptor: [Depress*]	142993 results

#2	MeSH descriptor: [Nutrients]	13601 results

#3	MeSH descriptor: [Trace Elements]	1745 results

#4	MeSH descriptor: [Dietary Supplements]	30553 results

#5	MeSH descriptor: [Dietary Fats, Unsaturated]	1337 results

#6	MeSH descriptor: [Fatty Acids, Unsaturated]	1944 results

#7	MeSH descriptor: [Oils]	17880 results

#8	MeSH descriptor: [Thioctic Acid]	482 results

#9	MeSH descriptor: [Inositol]	962 results

#10	MeSH descriptor: [Vitamin B 12] 4852 results

#11	MeSH descriptor: [Ascorbic Acid]	4373 results

#12	MeSH descriptor: [Herbal Medicine]	9226 results

#13	MeSH descriptor: [Plant Preparations]	2490 results

#14	MeSH descriptor: [Amino Acids] 12073 results

#15	MeSH descriptor: [Creatine]	7721 results

#16	MeSH descriptor: [Taurine]	862 results

#17	MeSH descriptor: [Cysteine]	1838 results

#18	MeSH descriptor: [Tryptophan]	2111 results

#19	MeSH descriptor: [S-Adenosylmethionine]	270 results

#20	MeSH descriptor: [Ubiquinone]	800 results

#21	MeSH descriptor: [Melissa]	333 results

#22	MeSH descriptor: [Withania]	288 results

#23	MeSH descriptor: [Pantothenic Acid]	267 results

#24	MeSH descriptor: [Thiamine]	1012 results

#25	MeSH descriptor: [Vitamin A]	35302 results

#26	MeSH descriptor: [Carotenoids]	1806 results

#27	MeSH descriptor: [Riboflavin]	1126 results

#28	MeSH descriptor: [Nicotinic Acids]	1478 results

#29	MeSH descriptor: [Biotin] 400 results

#30	MeSH descriptor: [Folic Acid]	5555 results

#31	MeSH descriptor: [Leucovorin]	4047 results

#32	MeSH descriptor: [Tetrahydrofolates]	227 results

#33	MeSH descriptor: [Tocopherols]	2815 results

#34	MeSH descriptor: [Cholecalciferol]	3856 results

#35	MeSH descriptor: [Vitamin D]	20930 results

#36	MeSH descriptor: [Carnitine]	2142 results

#37	MeSH descriptor: [Flavonoids] 1977 results

#38	MeSH descriptor: [Calcium]	34891 results

#39	MeSH descriptor: [Magnesium]	10281 results

#40	MeSH descriptor: [Phosphorus]	3822 results

#41	MeSH descriptor: [Iron Compounds]	2016 results

#42	MeSH descriptor: [Potassium]	14979 results

#43	MeSH descriptor: [Manganese]	447 results

#44	MeSH descriptor: [Zinc]	7420 results

#45	MeSH descriptor: [Selenium]	2444 results

#46	MeSH descriptor: [Chromium]	1517 results

#47	MeSH descriptor: [Boron] 526 results

#48	MeSH descriptor: [Vitamin B 6] 5192 results

#49	MeSH descriptor: [Hypericum] 481 results

#50	MeSH descriptor: [Kava] 156 results

#51	MeSH descriptor: [Panax] 645 results

#52	MeSH descriptor: [Crocus] 327 results

#53	MeSH descriptor: [Curcumin] 2019 results

#54	MeSH descriptor: [Valerian] 269 results

#55	MeSH descriptor: [Ginkgo biloba] 1086 results

#56	MeSH descriptor: [Curcuma] 647 results

#57	MeSH descriptor: [Lavandula] 384 results

#58	MeSH descriptor: [Passiflora] 107 results

#59	MeSH descriptor: [Rhodiola] 132 results

#60	MeSH descriptor: [Galphimia] 30 results

#61	MeSH descriptor: [Centella] 200 results

#62	MeSH descriptor: [Bacopa] 152 results

#63	MeSH descriptor: [Matricaria] 170 results

#64	MeSH descriptor: [Cytidine Diphosphate Choline] 190 results

#65	MeSH descriptor: [Clinical Trial]	1440590 results

#66	(random*):ti,ab,kw	1489636 results

#67	(control*):ti,ab,kw	2263313 results

#68	(placebo):ti,ab,kw	410439 results

#69	(depress*) AND (nutrients OR trace elements OR dietary supplements OR dietary fats, unsaturated OR Fatty Acids, Unsaturated OR Oils OR Thioctic Acid OR. Inositol OR Vitamin B 12 OR Ascorbic Acid OR Herbal Medicine OR Plant Preparations OR Amino Acids OR Creatine OR Taurine OR Cysteine OR Tryptophan OR S-Adenosylmethionine OR Ubiquinone OR Melissa OR Withania OR Pantothenic Acid OR Thiamine OR Vitamin A OR Carotenoids OR Riboflavin OR Nicotinic Acids OR Biotin OR Folic Acid OR Leucovorin OR Tetrahydrofolates OR Tocopherols OR Cholecalciferol OR Vitamin D OR Carnitine OR Flavonoids OR Calcium OR Magnesium OR phosphorus OR Iron Compounds OR Potassium OR Manganese OR zinc OR Selenium OR Chromium OR Boron OR Vitamin B 6 OR Hypericum OR Kava OR Panax OR Crocus OR curcumin OR Valerian OR Ginkgo blob OR curcuma OR Lavandula OR passiflora OR rhodiola OR galphimia OR centella OR bacopa OR Matricaria OR cytidine diphosphate choline) AND (Clinical Trial OR random OR Control OR placebo)	6,349 results

ClinicalTrial.gov (up until July 1^st^, 2025)

Studies With Results | Interventional Studies | Depression | dietary supplement OR herbal OR phytoceutical OR nutraceutical OR fatty acid OR vitamin OR trace element OR probiotic OR prebiotic

57 **results**

EMBASE (up until July 1^st^, 2025)

#1: (((((((((((((depress*:ti,ab.)))))))))))))

873,901 results

#2: ((((((((((((((((((((((((((((((((((((((((((((((((((((((((((((((((((((((((((((((((((((((((((((((((((((((((((vitamin*:ti,ab) OR (mineral*:ti,ab)) OR (nutrient*:ti,ab)) OR ('food supplement*':ti,ab)) OR ('meal replacement*':ti,ab)) OR ('nutritional supplement*':ti,ab)) OR ('health supplement*':ti,ab)) OR (multivitamin*:ti,ab)) OR ('omega 3':ti,ab)) OR ('fish oil*':ti,ab)) OR ('alpha lipoic acid':ti,ab)) OR ('alpha linolenic acid':ti,ab)) OR ('alpha linoleic acid':ti,ab)) OR (eicosapentaenoic:ti,ab)) OR (docosahexaenoic:ti,ab)) OR ('fatty acid*':ti,ab)) OR ('amino acid*':ti,ab)) OR (taurine:ti,ab)) OR (sadenosylmethionine:ti,ab)) OR (creatine:ti,ab)) OR (acetylcysteine:ti,ab)) OR (cysteine:ti,ab)) OR (probiotic*:ti,ab)) OR (prebiotic*:ti,ab)) OR (psychobiotic*:ti,ab)) OR (postbiotic*:ti,ab)) OR (synbiotic*:ti,ab)) OR (tryptophan:ti,ab)) OR (tocopherol:ti,ab)) OR (alphatocopherol:ti,ab)) OR (carotene:ti,ab)) OR (retinol:ti,ab)) OR (thiamine:ti,ab)) OR (riboflavin:ti,ab)) OR (niacin:ti,ab)) OR (niacinamide:ti,ab)) OR ('nicotinic acid':ti,ab)) OR (pantothenic:ti,ab)) OR (pyridox*:ti,ab)) OR (biotin:ti,ab)) OR (methylfolate:ti,ab)) OR (5-MTH*:ti,ab)) OR ('levomefolic acid':ti,ab)) OR (folate:ti,ab)) OR ('folinic acid':ti,ab)) OR ('folic acid':ti,ab)) OR (inositol:ti,ab)) OR (cyanocobalamin:ti,ab)) OR (methylcobalamin:ti,ab)) OR (cobalamin:ti,ab)) OR ('ascorbic acid':ti,ab)) OR (cholecalciferol:ti,ab)) OR (iron:ti,ab)) OR (ferrous:ti,ab)) OR (tocopherols:ti,ab)) OR ('trace element':ti,ab)) OR (calcium:ti,ab)) OR (phosphorus:ti,ab)) OR (magnesium:ti,ab)) OR (potassium:ti,ab)) OR (manganese:ti,ab)) OR (zinc:ti,ab)) OR (selenium:ti,ab)) OR (boron:ti,ab)) OR (chromium:ti,ab)) OR (lycopene:ti,ab)) OR (isoflav*:ti,ab)) OR (flavonoid*:ti,ab)) OR (bioflavonoid*:ti,ab)) OR (micronutrient:ti,ab)) OR (carnitine:ti,ab)) OR (herbal:ti,ab)) OR ('herbal medicine':ti,ab)) OR ('plant medicine':ti,ab)) OR (phytomedicine:ti,ab)) OR (supplement:ti,ab)) OR ('st john s wort':ti,ab)) OR (kava:ti,ab)) OR (ginseng:ti,ab)) OR (saffron:ti,ab)) OR (curcumin:ti,ab)) OR (valerian:ti,ab)) OR (ginkgo:ti,ab)) OR (rhodiola:ti,ab)) OR ('bacopa monniera':ti,ab)) OR ('centella asiatica':ti,ab)) OR ('crocus sativus':ti,ab)) OR ('curcuma longa':ti,ab)) OR ('hypericum perforatum':ti,ab)) OR (galphimia:ti,ab)) OR ('ginkgo biloba':ti,ab)) OR (lavandula:ti,ab)) OR (matricaria:ti,ab)) OR ('panax ginseng':ti,ab)) OR ('passiflora incarnata':ti,ab)) OR ('piper methysticum':ti,ab)) OR ('rhodiola rosea':ti,ab)) OR (valeriana:ti,ab)) OR ('withania somnifera':ti,ab)) OR ('melissa officinalis':ti,ab)) OR (sarsasapogenin:ti,ab)) OR (mangosteen:ti,ab)) OR (scutellaria:ti,ab)) OR (silexan:ti,ab)) OR (citicoline:ti,ab)) OR ('coenzyme q10':ti,ab)) OR (theanine:ti,ab)

3,772,899 results

#3 depress*:ti,ab AND (vitamin*:ti,ab OR mineral*:ti,ab OR nutrient*:ti,ab OR 'food supplement*':ti,ab OR 'meal replacement*':ti,ab OR 'nutritional supplement*':ti,ab OR 'health supplement*':ti,ab OR multivitamin*:ti,ab OR 'omega 3':ti,ab OR 'fish oil*':ti,ab OR 'alpha lipoic acid':ti,ab OR 'alpha linolenic acid':ti,ab OR 'alpha linoleic acid':ti,ab OR eicosapentaenoic:ti,ab OR docosahexaenoic:ti,ab OR 'fatty acid*':ti,ab OR 'amino acid*':ti,ab OR taurine:ti,ab OR sadenosylmethionine:ti,ab OR creatine:ti,ab OR acetylcysteine:ti,ab OR cysteine:ti,ab OR probiotic*:ti,ab OR prebiotic*:ti,ab OR psychobiotic*:ti,ab OR postbiotic*:ti,ab OR synbiotic*:ti,ab OR tryptophan:ti,ab OR tocopherol:ti,ab OR alphatocopherol:ti,ab OR carotene:ti,ab OR retinol:ti,ab OR thiamine:ti,ab OR riboflavin:ti,ab OR niacin:ti,ab OR niacinamide:ti,ab OR 'nicotinic acid':ti,ab OR pantothenic:ti,ab OR pyridox*:ti,ab OR biotin:ti,ab OR methylfolate:ti,ab OR '5 mth*':ti,ab OR 'levomefolic acid':ti,ab OR folate:ti,ab OR 'folinic acid':ti,ab OR 'folic acid':ti,ab OR inositol:ti,ab OR cyanocobalamin:ti,ab OR methylcobalamin:ti,ab OR cobalamin:ti,ab OR 'ascorbic acid':ti,ab OR cholecalciferol:ti,ab OR iron:ti,ab OR ferrous:ti,ab OR tocopherols:ti,ab OR 'trace element':ti,ab OR calcium:ti,ab OR phosphorus:ti,ab OR magnesium:ti,ab OR potassium:ti,ab OR manganese:ti,ab OR zinc:ti,ab OR selenium:ti,ab OR boron:ti,ab OR chromium:ti,ab OR lycopene:ti,ab OR isoflav*:ti,ab OR flavonoid*:ti,ab OR bioflavonoid*:ti,ab OR micronutrient:ti,ab OR carnitine:ti,ab OR herbal:ti,ab OR 'herbal medicine':ti,ab OR 'plant medicine':ti,ab OR phytomedicine:ti,ab OR supplement:ti,ab OR 'st john s wort':ti,ab OR kava:ti,ab OR ginseng:ti,ab OR saffron:ti,ab OR curcumin:ti,ab OR valerian:ti,ab OR ginkgo:ti,ab OR rhodiola:ti,ab OR 'bacopa monniera':ti,ab OR 'centella asiatica':ti,ab OR 'crocus sativus':ti,ab OR 'curcuma longa':ti,ab OR 'hypericum perforatum':ti,ab OR galphimia:ti,ab OR 'ginkgo biloba':ti,ab OR lavandula:ti,ab OR matricaria:ti,ab OR 'panax ginseng':ti,ab OR 'passiflora incarnata':ti,ab OR 'piper methysticum':ti,ab OR 'rhodiola rosea':ti,ab OR valeriana:ti,ab OR 'withania somnifera':ti,ab OR 'melissa officinalis':ti,ab OR sarsasapogenin:ti,ab OR mangosteen:ti,ab OR scutellaria:ti,ab OR silexan:ti,ab OR citicoline:ti,ab OR 'coenzyme q10':ti,ab OR theanine:ti,ab) AND ('clinical trial'/de OR 'controlled clinical trial'/de OR 'randomized controlled trial'/de)

6,468 results

Scopus (up until July 1^st^, 2025)

#1: (((((((((((((TITLE-ABS(depress*))))))))))))))

910,348 results

#2: TITLE-ABS-KEY ( nutraceutical OR phytoceutical OR nutrient OR vitamin OR mineral OR nutritional ORdietary OR "omega 3 fatty" OR "omega 6 fatty" OR icosapentaenoic OR docosahexaenoic OR fatty ANDacid OR thioctic OR linolenic OR linoleic OR aminoacid OR taurine OR ademetionine OR creatine ORacetylcysteine OR cysteine OR probiotic OR probiotic OR psychobiotic OR synbiotic OR tryptophan ORtocopherol OR retinol OR thiamine OR carotene OR riboflavin OR nicotinic OR biotin OR methylfolateOR folic OR folate OR pantothenic OR pyridoxine OR inositol OR cyanocobalamin OR mecobalamin ORcobalamin OR ascorbic OR cholecalciferol OR iron OR ferrous OR phosphorus OR magnesium ORcalcium OR zinc OR chromium OR potassium OR boron OR lycopene OR selenium OR flavonoid ORbioflavonoid OR carnitine OR herbal OR plant OR saffron OR crocus OR ginseng OR curcumin ORginkgo OR rhodiola OR bacopa OR centella OR curcuma OR hypericum OR passiflora OR matricaria ORlavender OR valeriana OR withania OR scutellaria OR melissa OR citicoline OR theanine OR ubiquinoneOR kava OR mangosteen OR silexan )

1,856,785 results

#3: TITLE-ABS-KEY ( depress* ) AND TITLE-ABS-KEY ( nutraceutical OR phytoceutical OR nutrient ORvitamin OR mineral OR nutritional OR dietary OR "omega 3 fatty" OR "omega 6 fatty" ORicosapentaenoic OR docosahexaenoic OR fatty AND acid OR thioctic OR linolenic OR linoleic ORaminoacid OR taurine OR ademetionine OR creatine OR acetylcysteine OR cysteine OR probiotic ORprobiotic OR psychobiotic OR synbiotic OR tryptophan OR tocopherol OR retinol OR thiamine ORcarotene OR riboflavin OR nicotinic OR biotin OR methylfolate OR folic OR folate OR pantothenic ORpyridoxine OR inositol OR cyanocobalamin OR mecobalamin OR cobalamin OR ascorbic ORcholecalciferol OR iron OR ferrous OR phosphorus OR magnesium OR calcium OR zinc OR chromiumOR potassium OR boron OR lycopene OR selenium OR flavonoid OR bioflavonoid OR carnitine ORherbal OR plant OR saffron OR crocus OR ginseng OR curcumin OR ginkgo OR rhodiola OR bacopa ORcentella OR curcuma OR hypericum OR passiflora OR matricaria OR lavender OR valeriana ORwithania OR scutellaria OR melissa OR citicoline OR theanine OR ubiquinone OR kava OR mangosteenOR silexan ) AND TITLE-ABS-KEY ( randomized OR randomised OR controlled AND trial )

3,602 results

PsycInfo (up until July 1^st^, 2025)

#1: (((((((((((((depress*)))))))))))))

**491,402 results**

#2: (nutraceuticals) OR (phytoceuticals) OR (nutrients) OR (polyunsaturated fatty acids) OR (vitamins) OR (amino acids) OR (minerals) OR (trace element) OR (probiotics) OR (prebiotics) OR (herbal) OR (curcumin) OR (curcuma) OR (mangosteen) OR (food supplement) OR (nutritional supplement) OR (multivitamin) OR (omega 3) OR (alpha lipoic acid) OR (alpha linolenic acid) OR (alpha linoleic acid) OR (eicosapentaenoic) OR (docosahexaenoic) OR (taurine) OR (sadenosylmethionine) OR (creatine) OR (acetylcysteine) OR (cysteine) OR (psychobiotics) OR (postbiotics) OR (synbiotic) OR (tryptophan) OR (tocopherol) OR (retinol) OR (thiamine) OR (riboflavin) OR (niacin) OR (niacinamide) OR (nicotinic acid) OR (pantothenic) OR (pyridoxine) OR (biotin) OR (methylfolate) OR (5-MTH) OR (levomefolic acid) OR (folate) OR (folic acid) OR (inositol) OR (cyanocobalamin) OR (methylcobalamin) OR (cobalamin) OR (ascorbic acid) OR (vitamin D) OR (iron) OR (tocopherols) OR (calcium) OR (phosphorus) OR (magnesium) OR (potassium) OR (manganese) OR (zinc) OR (selenium) OR (boron) OR (chromium) OR (lycopene) OR (flavonoids) OR (micronutrient) OR (carnitine) OR (st john s wort) OR (kava) OR (ginseng) OR (saffron) OR (ginkgo) OR (rhodiola) OR (bacopa monniera) OR (centella asiatica) OR (crocus sativus) OR (curcuma longa) OR (hypericum perforatum) OR (galphimia) OR (ginkgo biloba) OR (lavandula) OR (matricaria) OR (panax ginseng) OR (passiflora incarnata) OR (piper methysticum) OR (rhodiola rosea) OR (valeriana) OR (withania somnifera) OR (melissa officinalis) OR (sarsasapogenin) OR (scutellaria) OR (silexan) OR (citicoline) OR (coenzyme q10) OR (theanine)

**80,378 results**

#3: (depress*) AND ((nutraceuticals) OR (phytoceuticals) OR (nutrients) OR (polyunsaturated fatty acids) OR (vitamins) OR (amino acids) OR (minerals) OR (trace element) OR (probiotics) OR (prebiotics) OR (herbal) OR (curcumin) OR (curcuma) OR (mangosteen) OR (food supplement) OR (nutritional supplement) OR (multivitamin) OR (omega 3) OR (alpha lipoic acid) OR (alpha linolenic acid) OR (alpha linoleic acid) OR (eicosapentaenoic) OR (docosahexaenoic) OR (taurine) OR (sadenosylmethionine) OR (creatine) OR (acetylcysteine) OR (cysteine) OR (psychobiotics) OR (postbiotics) OR (synbiotic) OR (tryptophan) OR (tocopherol) OR (retinol) OR (thiamine) OR (riboflavin) OR (niacin) OR (niacinamide) OR (nicotinic acid) OR (pantothenic) OR (pyridoxine) OR (biotin) OR (methylfolate) OR (5-MTH) OR (levomefolic acid) OR (folate) OR (folic acid) OR (inositol) OR (cyanocobalamin) OR (methylcobalamin) OR (cobalamin) OR (ascorbic acid) OR (vitamin D) OR (iron) OR (tocopherols) OR (calcium) OR (phosphorus) OR (magnesium) OR (potassium) OR (manganese) OR (zinc) OR (selenium) OR (boron) OR (chromium) OR (lycopene) OR (flavonoids) OR (micronutrient) OR (carnitine) OR (st john s wort) OR (kava) OR (ginseng) OR (saffron) OR (ginkgo) OR (rhodiola) OR (bacopa monniera) OR (centella asiatica) OR (crocus sativus) OR (curcuma longa) OR (hypericum perforatum) OR (galphimia) OR (ginkgo biloba) OR (lavandula) OR (matricaria) OR (panax ginseng) OR (passiflora incarnata) OR (piper methysticum) OR (rhodiola rosea) OR (valeriana) OR (withania somnifera) OR (melissa officinalis) OR (sarsasapogenin) OR (scutellaria) OR (silexan) OR (citicoline) OR (coenzyme q10) OR (theanine)) AND ((randomized) OR (randomised) OR (controlled))

**1,578 results identified, 7 retrieved**

| **S1.1: Citations by each source** | |
| --- | --- |
| **Databases, trial registers, and other sources** | **Citations** |
| **Databases:** |  |
| PubMed | 9,076 |
| EMBASE | 6,468 |
| Scopus | 3,602 |
| PsycINFO | 7 |
| CENTRAL | 6,349 |
| **Total (databases)** | **25,502** |
|  |  |
| **Trial registers:** |  |
| ClinicalTrials.gov | 57 |
| **Total (trial registers)** | **57** |
|  |  |
| **Other sources (Cross-references and manual search):** | **4** |
|  |  |
| **Total** | **25,563** |

# **S2:** Excluded studies, with the reason(s).

| **Author, year** | **Reasons** |
| --- | --- |
| ^1^(2000)(2000)NCT00005013 | Trial registry record |
| ^2^[NCT00042380](https://clinicaltrials.gov/study/NCT00042380) | Trial registry record |
| ^3^PMID: 12070867 | Foreign language |
| ^4^PMID: **12085821** | Narrative article |
| ^5^[NCT00066859](https://clinicaltrials.gov/study/NCT00066859) | Trial registry record |
| ^6^[NCT00067301](https://clinicaltrials.gov/study/NCT00067301) | Trial registry record |
| ^7^ISSN 20786190 | Narrative article |
| ^8^[NCT00122954](https://clinicaltrials.gov/study/NCT00122954) | Trial registry record |
| ^9^[NCT00223236](https://clinicaltrials.gov/study/NCT00223236) | Trial registry record |
| ^10^[NCT00226356](https://clinicaltrials.gov/study/NCT00226356) | Trial registry record |
| ^11^[NCT00256412](https://clinicaltrials.gov/study/NCT00256412) | Trial registry record |
| ^12^[NCT00480207](https://clinicaltrials.gov/study/NCT00480207) | Trial registry record |
| ^13^[NCT00539513](https://clinicaltrials.gov/study/NCT00539513) | Trial registry record |
| ^14^[NCT00693680](https://clinicaltrials.gov/study/NCT00693680) | Trial registry record |
| ^15^[NCT00711971](https://clinicaltrials.gov/study/NCT00711971) | Trial registry record |
| ^16^[NCT00729755](https://clinicaltrials.gov/study/NCT00729755) | Trial registry record |
| ^17^[NCT00816322](https://clinicaltrials.gov/study/NCT00816322) | Trial registry record |
| ^18^[NCT00861978](https://clinicaltrials.gov/study/NCT00861978) | Trial registry record |
| ^19^[NCT00955955](https://clinicaltrials.gov/study/NCT00955955) | Trial registry record |
| ^20^[NCT00997490](https://clinicaltrials.gov/study/NCT00997490) | Trial registry record |
| ^21^[NCT01022632](https://clinicaltrials.gov/study/NCT01022632) | Trial registry record |
| ^22^Gilbert, G.E.; Wahlquist A.H. | Poster/Conference abstract |
| ^23^[NCT01175616](https://clinicaltrials.gov/study/NCT01175616) | Withdrawn trial |
| ^24^[NCT01185574](https://clinicaltrials.gov/study/NCT01185574) | Trial registry record |
| ^25^[NCT01223729](https://clinicaltrials.gov/study/NCT01223729) | Wrong population |
| ^26^[NCT01390662](https://clinicaltrials.gov/study/NCT01390662) | Trial registry record |
| ^27^[NCT01416818](https://clinicaltrials.gov/study/NCT01416818) | Trial registry record |
| ^28^[NCT01462058](https://clinicaltrials.gov/study/NCT01462058) | Trial registry record |
| ^29^[NCT01750359](https://clinicaltrials.gov/study/NCT01750359) | Trial registry record |
| ^30^[NCT02189915](https://clinicaltrials.gov/study/NCT02189915) | Trial registry record |
| ^31^[NCT02223299](https://clinicaltrials.gov/study/NCT02223299) | Trial registry record |
| ^32^[NCT02359266](https://clinicaltrials.gov/study/NCT02359266) | Wrong population |
| ^33^[NCT02381600](https://clinicaltrials.gov/study/NCT02381600) | Trial registry record |
| ^34^[NCT02568878](https://clinicaltrials.gov/study/NCT02568878) | Trial registry record |
| ^35^[NCT02793648](https://clinicaltrials.gov/study/NCT02793648) | Trial registry record |
| ^36^[NCT02838043](https://clinicaltrials.gov/study/NCT02838043) | Trial registry record |
| ^37^[NCT02890212](https://clinicaltrials.gov/study/NCT02890212) | Trial registry record |
| ^38^[NCT02922725](https://clinicaltrials.gov/study/NCT02922725) | Trial registry record |
| ^39^[NCT03081065](https://clinicaltrials.gov/study/NCT03081065) | Trial registry record |
| ^40^[NCT03323697](https://clinicaltrials.gov/study/NCT03323697) | Trial registry record |
| ^41^[NCT03380091](https://clinicaltrials.gov/study/NCT03380091) | Trial registry record |
| ^42^[NCT03754712](https://clinicaltrials.gov/study/NCT03754712) | Trial registry record |
| ^43^[NCT03766074](https://clinicaltrials.gov/study/NCT03766074) | Trial registry record |
| ^44^[NCT03932474](https://clinicaltrials.gov/study/NCT03932474) | Trial registry record |
| ^45^[NCT04006977](https://clinicaltrials.gov/study/NCT04006977) | Trial registry record |
| ^46^[NCT04032795](https://clinicaltrials.gov/study/NCT04032795) | Trial registry record |
| ^47^[NCT04124276](https://clinicaltrials.gov/study/NCT04124276) | Trial registry record |
| ^48^[NCT04333277](https://clinicaltrials.gov/study/NCT04333277) | Trial registry record |
| ^49^[NCT04353921](https://clinicaltrials.gov/study/NCT04353921) | No statistical significance and low enrollment |
| ^50^[NCT04482296](https://clinicaltrials.gov/study/NCT04482296) | Trial registry record |
| ^51^[NCT04567147](https://clinicaltrials.gov/study/NCT04567147) | Trial registry record |
| ^52^[NCT04570072](https://clinicaltrials.gov/study/NCT04570072) | Trial registry record |
| ^53^[NCT04803071](https://clinicaltrials.gov/study/NCT04803071) | Trial registry record |
| ^54^[NCT04832178](https://clinicaltrials.gov/study/NCT04832178) | Trial registry record |
| ^55^[NCT04917458](https://clinicaltrials.gov/study/NCT04917458) | Wrong population |
| ^56^[NCT04975100](https://clinicaltrials.gov/study/NCT04975100) | Trial registry record |
| ^57^[NCT05315401](https://clinicaltrials.gov/study/NCT05315401) | Trial registry record |
| ^58^[NCT05564767](https://clinicaltrials.gov/study/NCT05564767) | Trial registry record |
| ^59^[NCT06651424](https://clinicaltrials.gov/study/NCT06651424) | Trial registry record |
| ^60^[NCT05751746](https://clinicaltrials.gov/study/NCT05751746) | Trial registry record |
| ^61^[NCT05774665](https://clinicaltrials.gov/study/NCT05774665) | Trial registry record |
| ^62^[NCT05817383](https://clinicaltrials.gov/study/NCT05817383) | Study pilot |
| ^63^[NCT05931965](https://clinicaltrials.gov/study/NCT05931965) | No randomization |
| ^64^[NCT06580145](https://clinicaltrials.gov/study/NCT06580145) | Trial registry record |
| ^65^[NCT06629441](https://clinicaltrials.gov/study/NCT06629441) | Trial registry record |
| ^66^[NCT06723249](https://clinicaltrials.gov/study/NCT06723249) | Trial registry record |
| ^67^ | Trial registry record |
| ^68^ | Wrong population |
| ^69^ | Wrong population |
| ^70^ | Wrong population |
| ^71^ | Wrong population |
| ^72^ | Wrong or no control group |
| ^73^ | Duplicated |
| ^74^ | Duplicated |
| ^75^ | Wrong population |
| ^76^ | Wrong population |
| ^77^ | Poster/Conference abstract |
| ^78^ | Wrong population |
| ^79^ | Wrong study design |
| ^80^ | Wrong population |
| ^81^ | Wrong population |
| ^82^ | Wrong study design |
| ^83^ | Wrong outcome |
| ^84^ | Poster/Conference abstract |
| ^85^ | Wrong population |
| ^86^ | Wrong population |
| ^87^ | Wrong study design |
| ^88^ | Wrong population |
| ^89^ | Wrong population |
| ^90^ | Wrong population |
| ^91^ | Wrong study design |
| ^92^ | Wrong study design |
| ^93^ | Wrong or no control group |
| ^94^ | Wrong population |
| ^95^ | Wrong population |
| ^96^ | Wrong study design |
| ^97^ | Wrong population |
| ^98^ | Wrong population |
| ^99^ | Trial registry record |
| ^100^ | Wrong study design |
| ^101^ | Wrong study design |
| ^102^ | Wrong population |
| ^103^ | Wrong population |
| ^104^ | Poster/Conference abstract |
| ^105^ | Duplicated |
| ^106^ | Wrong or no control group |
| ^107^ | Wrong study design |
| ^108^ | Wrong outcome |
| ^109^ | Wrong population |
| ^110^ | Wrong population |
| ^111^ | Trial registry record |
| ^112^ | Wrong or no control group |
| ^113^ | Wrong outcome |
| ^114^ | Trial registry record |
| ^115^ | Wrong or no control group |
| ^116^ | Wrong study design |
| ^117^ | Wrong population |
| ^118^ | Wrong population |
| ^119^ | Wrong or no control group |
| ^120^ | Trial registry record |
| ^121^ | Wrong population |
| ^122^ | Wrong population |
| ^123^ | Wrong population |
| ^124^ | Wrong population |
| ^125^ | Wrong population |
| ^126^ | Wrong population |
| ^127^ | Wrong population |
| ^128^ | Wrong population |
| ^129^ | Wrong study design |
| ^130^ | Wrong type of intervention |
| ^131^ | Trial registry record |
| ^132^ | Poster/Conference abstract |
| ^133^ | Poster/Conference abstract |
| ^134^ | Trial registry record |
| ^135^ | Wrong population |
| ^136^ | Wrong or no control group |
| ^137^ | Wrong or no control group |
| ^138^ | Wrong or no control group |
| ^139^ | Wrong or no control group |
| ^140^ | Wrong population |
| ^141^ | Wrong population |
| ^142^ | Wrong or no control group |
| ^143^ | Wrong population |
| ^144^ | Wrong population |
| ^145^ | Wrong population |
| ^146^ | Wrong outcome |
| ^147^ | Study pilot |
| ^147^ | Study pilot |
| ^148^ | Poster/Conference abstract |
| ^149^ | Wrong outcome |
| ^150^ | Wrong population |
| ^151^ | Wrong population |
| ^152^ | Wrong study design |
| ^153^ | Wrong outcome |
| ^154^ | Wrong population |
| ^155^ | Wrong population |
| ^156^ | Wrong study design |
| ^157^ | Wrong population |
| ^158^ | Wrong population |
| ^159^ | Wrong population |
| ^160^ | Wrong outcome |
| ^161^ | Wrong or no control group |
| ^162^ | Wrong or no control group |
| ^163^ | Wrong population |
| ^164^ | Poster/Conference abstract |
| ^165^ | Wrong population |
| ^166^ | Wrong population |
| ^167^ | Trial registry record |
| ^168^ | Trial registry record |
| ^169^ | Trial registry record |
| ^170^ | Trial registry record |
| ^171^ | Trial registry record |
| ^172^ | Trial registry record |
| ^173^ | Trial registry record |
| ^174^ | Trial registry record |
| ^175^ | Wrong population |
| ^176^ | Trial registry record |
| ^177^ | Trial registry record |
| ^178^ | Wrong population |
| ^179^ | Wrong population |
| ^180^ | Wrong or no control group |
| ^181^ | Wrong population |
| ^182^ | Foreign language |
| ^183^ | Wrong population |
| ^184^ | Wrong population |
| ^185^ | Wrong population |
| ^186^ | Study pilot |
| ^186^ | Study pilot |
| ^187^ | Foreign language |
| ^188^ | Wrong study design |
| ^189^ | Foreign language |
| ^190^ | Poster/Conference abstract |
| ^191^ | Wrong study design |
| ^192^ | Wrong population |
| ^193^ | Wrong population |
| ^194^ | Wrong outcome |
| ^195^ | Wrong outcome |
| ^196^ | Poster/Conference abstract |
| ^197^ | Wrong population |
| ^198^ | Wrong population |
| ^199^ | Poster/Conference abstract |
| ^200^ | Wrong population |
| ^201^ | Wrong population |
| ^202^ | Poster/Conference abstract |
| ^203^ | Wrong study design |
| ^204^ | Study pilot |
| ^205^ | Wrong or no control group |
| ^206^ | Wrong population |
| ^207^ | Wrong population |
| ^208^ | Wrong population |
| ^209^ | Wrong outcome |
| ^210^ | Wrong population |
| ^211^ | Duplicated |
| ^212^ | Wrong population |
| ^213^ | Wrong population |
| ^214^ | Book chapter |
| ^215^ | Wrong population |
| ^216^ | Wrong outcome |
| ^217^ | Wrong study design |
| ^218^ | Wrong population |
| ^219^ | Wrong population |
| ^220^ | Trial registry record |
| ^221^ | Duplicated |
| ^222^ | Wrong population |
| ^223^ | Wrong population |
| ^224^ | Wrong population |
| ^225^ | Wrong population |
| ^226^ | Wrong study design |
| ^227^ | Wrong population |
| ^228^ | Wrong study design |
| ^229^ | Poster/Conference abstract |
| ^230^ | Wrong study design |
| ^231^ | Wrong outcome |
| ^232^ | Wrong population |
| ^233^ | Wrong population |
| ^234^ | Wrong population |
| ^235^ | Wrong population |
| ^236^ | Poster/Conference abstract |
| ^237^ | Wrong population |
| ^238^ | Wrong study design |
| ^239^ | Wrong population |
| ^240^ | Wrong population |
| ^241^ | Wrong population |
| ^242^ | Study pilot |
| ^243^ | Poster/Conference abstract |
| ^244^ | Wrong or no control group |
| ^245^ | Duplicated |
| ^246^ | Lack of recruitment |
| ^247^ | Wrong study design |
| ^248^ | Wrong or no control group |
| ^249^ | Wrong population |
| ^250^ | Trial registry record |
| ^251^ | Study pilot |
| ^252^ | Wrong or no control group |
| ^253^ | Wrong study design |
| ^254^ | Wrong or no control group |
| ^255^ | Duplicated |
| ^256^ | Poster/Conference abstract |
| ^257^ | Wrong population |
| ^258^ | Wrong population |
| ^259^ | Wrong outcome |
| ^260^ | Poster/Conference abstract |
| ^261^ | Wrong population |
| ^262^ | Wrong population |
| ^263^ | Wrong population |
| ^264^ | Wrong population |
| ^265^ | Wrong population |
| ^265^ | Wrong population |
| ^266^ | Poster/Conference abstract |
| ^267^ | Wrong study design |
| ^268^ | Foreign language |
| ^269^ | Poster/Conference abstract |
| ^270^ | Wrong population |
| ^271^ | Wrong population |
| ^272^ | Wrong or no control group |
| ^273^ | Wrong population |
| ^274^ | Wrong study design |
| ^275^ | Poster/Conference abstract |
| ^276^ | Wrong population |
| ^277^ | Wrong population |
| ^278^ | Trial registry record |
| ^279^ | Trial registry record |
| ^280^ | Trial registry record |
| ^281^ | Trial registry record |
| ^282^ | Trial registry record |
| ^283^ | Trial registry record |
| ^284^ | Trial registry record |
| ^285^ | Wrong population |
| ^286^ | Trial registry record |
| ^287^ | Trial registry record |
| ^288^ | Trial registry record |
| ^289^ | Trial registry record |
| ^290^ | Trial registry record |
| ^291^ | Ongoing trial |
| ^291^ | Trial registry record |
| ^292^ | Trial registry record |
| ^293^ | Trial registry record |
| ^294^ | Trial registry record |
| ^295^ | Trial registry record |
| ^296^ | Duplicated |
| ^297^ | Foreign language |
| ^298^ | Wrong outcome |
| ^299^ | Poster/Conference abstract |
| ^300^ | Wrong population |
| ^301^ | Wrong outcome |
| ^302^ | Duplicated |
| ^303^ | Poster/Conference abstract |
| ^304^ | Poster/Conference abstract |
| ^305^ | Wrong population |
| ^306^ | Wrong population |
| ^307^ | Foreign language |
| ^308^ | Foreign language |
| ^309^ | Poster/Conference abstract |
| ^310^ | Wrong population |
| ^311^ | Wrong study design |
| ^312^ | Wrong population |
| ^313^ | Wrong population |
| ^314^ | Wrong population |
| ^315^ | Wrong population |
| ^316^ | Wrong or no control group |
| ^316^ | Poster/Conference abstract |
| ^317^ | Poster/Conference abstract |
| ^318^ | Wrong population |
| ^319^ | Wrong or no control group |
| ^320^ | Wrong population |
| ^321^ | Wrong population |
| ^322^ | Wrong study design |
| ^323^ | Wrong population |
| ^324^ | Duplicated |
| ^325^ | Wrong population |
| ^326^ | Wrong population |
| ^327^ | Wrong population |
| ^328^ | Wrong population |
| ^329^ | Foreign language |
| ^330^ | Wrong population |
| ^331^ | Wrong study design |
| ^332^ | Duplicated |
| ^333^ | Poster/Conference abstract |
| ^334^ | Poster/Conference abstract |
| ^335^ | Wrong population |
| ^336^ | Poster/Conference abstract |
| ^337^ | Wrong or no control group |
| ^337^ | Wrong or no control group |
| ^338^ | Wrong population |
| ^339^ | Poster/Conference abstract |
| ^340^ | Wrong population |
| ^341^ | Wrong outcome |
| ^342^ | Wrong or no control group |
| ^343^ | Wrong study design |
| ^344^ | Letter to the editor |
| ^345^ | Wrong outcome |
| ^346^ | Duplicated |
| ^347^ | Wrong population |
| ^348^ | Wrong population |
| ^349^ | Foreign language |
| ^350^ | Wrong study design |
| ^351^ | Wrong or no control group |
| ^352^ | Wrong study design |
| ^353^ | Wrong population |
| ^354^ | Narrative article |
| ^355^ | Short communication |
| ^356^ | Wrong population |
| ^357^ | Narrative article |
| ^358^ | Poster/Conference abstract |
| ^359^ | Foreign language |
| ^360^ | Wrong study design |
| ^361^ | Wrong outcome |
| ^362^ | Wrong study design |
| ^362^ | Wrong study design |
| ^363^ | Wrong study design |
| ^363^ | Narrative article |
| ^363^ | Narrative article |
| ^364^ | Foreign language |
| ^365^ | Foreign language |
| ^366^ | Trial registry record |
| ^367^ | Wrong study design |
| ^368^ | Wrong population |
| ^369^ | Wrong population |
| ^370^ | Poster/Conference abstract |
| ^371^ | Wrong or no control group |
| ^372^ | Wrong or no control group |
| ^373^ | Wrong or no control group |
| ^374^ | Wrong or no control group |
| ^375^ | Wrong population |
| ^376^ | Wrong outcome |
| ^377^ | Wrong population |
| ^378^ | Wrong population |
| ^379^ | Wrong or no control group |
| ^380^ | Wrong population |
| ^381^ | Wrong population |
| ^382^ | Wrong study design |
| ^383^ | Wrong population |
| ^384^ | Wrong study design |
| ^385^ | Wrong population |
| ^386^ | Wrong outcome |
| ^387^ | Duplicated |
| ^388^ | Wrong population |
| ^389^ | Wrong outcome |
| ^390^ | Wrong population |
| ^391^ | Wrong type of intervention |
| ^392^ | Wrong population |
| ^393^ | Foreign language |
| ^394^ | Wrong population |
| ^395^ | Wrong population |
| ^396^ | Wrong population |
| ^396^ | Wrong population |
| ^397^ | Narrative article |
| ^398^ | Wrong population |
| ^399^ | Wrong population |
| ^400^ | Wrong population |
| ^401^ | Duplicated |
| ^402^ | Wrong population |
| ^403^ | Wrong study design |
| ^404^ | Wrong population |
| ^405^ | Wrong study design |
| ^406^ | Wrong population |
| ^407^ | Wrong population |
| ^408^ | Wrong study design |
| ^409^ | Wrong population |
| ^410^ | Wrong population |
| ^411^ | Wrong population |
| ^412^ | Wrong population |
| ^413^ | Wrong population |
| ^414^ | Wrong population |
| ^415^ | Poster/Conference abstract |
| ^416^ | Wrong population |
| ^417^ | Wrong population |
| ^418^ | Wrong or no control group |
| ^419^ | Wrong population |
| ^420^ | Wrong population |
| ^421^ | Wrong population |
| ^422^ | Wrong population |
| ^423^ | Wrong outcome |
| ^424^ | Wrong population |
| ^425^ | Wrong or no control group |
| ^425^ | Wrong or no control group |
| ^426^ | Wrong or no control group |
| ^427^ | Wrong study design |
| ^428^ | Narrative article |
| ^429^ | Duplicated |
| ^430^ | Wrong study design |
| ^431^ | Wrong population |
| ^432^ | Wrong outcome |
| ^433^ | Poster/Conference abstract |
| ^434^ | Poster/Conference abstract |
| ^435^ | Wrong or no control group |
| ^436^ | Wrong or no control group |
| ^437^ | Study pilot |
| ^438^ | Duplicated |
| ^439^ | Wrong or no control group |
| ^440^ | Wrong outcome |
| ^441^ | Wrong population |
| ^442^ | Poster/Conference abstract |
| ^443^ | Wrong population |
| ^444^ | Wrong or no control group |
| ^445^ | Poster/Conference abstract |
| ^446^ | Wrong population |
| ^447^ | Poster/Conference abstract |
| ^448^ | Wrong population |
| ^449^ | Duplicated |
| ^450^ | Poster/Conference abstract |
| ^451^ | Wrong population |
| ^452^ | Poster/Conference abstract |
| ^453^ | Study pilot |
| ^454^ | Wrong population |

**References of the excluded studies** (n=454 total records, 453 distinct records).

(2016). Double Blind, Placebo Controlled Pilot Study to Evaluate the Use of Selenium as an Augmentation Treatment for Sertraline Resistant Major Depression. clinicaltrials.gov.

(2016). The Efficacy, Safety, and Tolerability of Probiotics on the Mood and Cognition of Depressed Patients. clinicaltrials.gov.

(2016). A Randomized, Double-blind, Placebo-controlled Trial of 5-hydroxytryptophan and Creatine for SSRI or SNRI Augmentation in Treatment Resistant Depression Associated With Hypobaric Hypoxia in Females. clinicaltrials.gov.

(2017). The Chinese Herb SZ-05 for the Treatment of Depression and Anxiety Disorders; Efficiency, Safety and Biological Correlates. clinicaltrials.gov.

(2017). Metformin, Vitamin D, and Depression in Polycystic Ovary Syndrome (PCOS) Trial: The MINDD Trial. clinicaltrials.gov.

(2017). Nutritional Intervention With Mediterranean Diet in the Prevention of Recurrence of Depression. clinicaltrials.gov.

(2018). Effect of Vitamin D Administration Along With Selective Serotonin Reuptake Inhibitors (SSRIs) in Patients With Major Depressive Disorder (MDD). clinicaltrials.gov.

(2018). Evaluation of Vitamin D Supplementation Effects on Serum 25(OH)D3, PTH, Pro-inflammatory Biomarkers and Neurotransmitters Involved in Depression, and Depression Status in Depressive Patients. clinicaltrials.gov.

(2019). DOUBLE BLIND, RANDOMIZED, PLACEBO CONTROLLED STUDY TO EVALUATE THE EFFICACY OF SAMEUP IN SUBJECTS CHARACTERIZED BY MILD TO MODERATE DEPRESSION SYMPTOMS. clinicaltrials.gov.

(2019). Evaluation of Efficacy of Lycium Barbarum Polysaccharide in Patients With Major Depressive Disorder: a Randomized,Placebo-controled Trial. clinicaltrials.gov.

(2019). Evaluation of Efficacy of Lycium Barbarum Polysaccharide in People With Subthreshold Depression: a Randomized, Placebo-controlled Trial. clinicaltrials.gov.

(2019). Multistrain Probiotic Product (De Simone Formulation) Reduces Depression and Anxiety Scores: a Randomized Pilot Study in Patients With Ulcerative Colitis. clinicaltrials.gov.

(2020). Double-blind Placebo-controlled Clinical Trial of Lactobacillus Helveticus as an add-on Strategy for the Treatment of Major Depression. clinicaltrials.gov.

(2020). Effect of Probiotic on Chinese Patients With Depression and Constipated Comorbidity. clinicaltrials.gov.

(2020). Effect of Probiotic on Depressive Symptom in Chinese Patients. clinicaltrials.gov.

(2020). Effect of Zinc Supplementation on Depression in Selective Serotonin Reuptake Inhibitors-treated Major Depressive Disorder Patients. clinicaltrials.gov.

(2020). A Two-Year Observational Follow-up Study of Subjects With Major Depressive Disorder Following a Randomized, Double-Blind Single-Dose of Psilocybin or Niacin-Control. clinicaltrials.gov.

(2021). Effects of Cholecalciferol on Depressive Symptoms in Type 2 Diabetes Mellitus Patients: Study of Neurotrophin-3, Serotonin, and C-Peptide. clinicaltrials.gov.

(2021). Evaluation of Efficacy and Safety of add-on Sarcosine in Patients With Major Depressive Disorder: A Randomized Controlled Trial. clinicaltrials.gov.

(2021). Omega-3 Unsaturated Fatty Acid Improves Cognitive Function in Patients With Depression: a 12 Week Randomized, Double-blind, Placebo-controlled Study. clinicaltrials.gov.

(2021). Use of Sumor in the Antidepressant Therapy of Subjects With Major Depression: a Randomized Placebo-controlled Clinical Trial. clinicaltrials.gov.

(2022). Effect of Two Probiotic Formulations on Mental Health and Mood Biomarkers in Adults With Depressive Symptoms: A Pilot Randomized Placebo-controlled Trial. clinicaltrials.gov.

(2022). The Efficacy of Probiotic Compared With Placebo and Acceptance and Commitment Therapy for the Treatment of Major Depressive Disorder Among Adult Females. clinicaltrials.gov.

(2022). Multicenter, Randomized, Double-blind, Parallel-group Controlled Nutritional Intervention Study to Evaluate the Effect of a Combination of Probiotic Strain on Postpartum Depression. clinicaltrials.gov.

(2023). Blueberries, Gut Microbiota, and Metabolites in Depressed Older Adults - A Pilot Study. clinicaltrials.gov.

(2023). Comparative Efficacy of Antidepressants With L-methylfolate, B12, and Magnesium in Depressive Disorders. clinicaltrials.gov.

(2023). The Effect of Peripartum Magnesium Sulfate Use on the Occurrence of Postpartum Depression. clinicaltrials.gov.

(2023). Specialized Pro-resolving Lipid Mediators and Treatment Resistant Depression. clinicaltrials.gov.

(2024). The Effect of Stingless Bee Honey on BDNF, Oxidative Stress, Depressive Symptoms and Verbal Learning and Memory in Major Depressive Disorders: A Randomized Clinical Trial. clinicaltrials.gov.

(2024). Effectiveness and Safety of Probiotics, BC99 in Relieving Anxiety and Depression in Adults. clinicaltrials.gov.

(2024). Leucine as a Probe of Kynurenine-Induced Glutamate and Neural Circuit Dysfunction in Midlife Depression. clinicaltrials.gov.

12070867, P. (2002). "St. John's Wort for depression. 900 mg per day should an effective dose." MMW Fortschritte der Medizin **Suppl 2**: 87.

12085821, P. (2002). "Study shows St. John's wort ineffective for major depression." FDA consumer **36**(3): 8.

20786190, I. (2005). "St John's wort = SSRI for moderate-severe depression." South African Family Practice **47**(4): 18.

Actrn (2017). "A micronutrient intervention for adults experiencing symptoms of anxiety and depression."

Adams, P. W., D. P. Rose, J. Folkard, V. Wynn, M. Seed and R. Strong (1973). "Effect of pyridoxine hydrochloride (vitamin B 6 ) upon depression associated with oral contraception." Lancet **1**(7809): 899-904.

Afsharfar, M., M. Shahraki, M. Shakiba, O. Asbaghi and A. Dashipour (2021). "The effects of magnesium supplementation on serum level of brain derived neurotrophic factor (BDNF) and depression status in patients with depression." Clinical Nutrition ESPEN **42**: 381-386.

Afzali, A., Z. Vakili, S. Goli, H. Bagheri, S. Mirhosseini and H. Ebrahimi (2021). "A Randomized Clinical Trial of the Effect of Zinc Supplement on Depression and Anxiety in the Elderly." Open Public Health Journal **14**(1): 537-544.

Ahmad, S., A. Khan, H. Rafiq, P. Tikmani, H. Mesiya and F. Amin (2023). "Co-administration of saffron and chamomile: to determine the efficacy as an adjuvant therapy for mild to moderate depression in human subjects. A pilot randomized clinical trial." Journal of the Pakistan Medical Association **73**(6): 1245-1250.

Akhondzadeh Basti, A., S. A. Choreishi, A. A. Noorbala, S. H. Akhondzadeh and S. Rezazadeh (2008). "Petal and stigma of Crocus sativus L. in the treatment of depression: A pilot double - blind randomized trial." Journal of Medicinal Plants **7**(SUPPL. 4): 29-36.

Akhondzadeh, S., H. Fallah-Pour, K. Afkham, A. H. Jamshidi and F. Khalighi-Cigaroudi (2004). "Comparison of Crocus sativus L. and imipramine in the treatment of mild to moderate depression: a pilot double-blind randomized trial." **4**.

Akhondzadeh, S., H. Fallah-Pour, K. Afkham, A. H. Jamshidi, F. Khalighi-Cigaroudi and L. G. Miller (2005). "A comparative trial of Crocus sativus L. (saffron) and imipramine in mild to moderate depression." Focus on Alternative and Complementary Therapies **10**(1): 22-23.

Alavi, N. M., S. Khademalhoseini, Z. Vakili and F. Assarian (2019). "Effect of vitamin D supplementation on depression in elderly patients: A randomized clinical trial." Clinical Nutrition **38**(5): 2065-2070.

Alcocer-Gómez, E., J. A. Sánchez-Alcázar and M. D. Cordero (2014). "Coenzyme Q10 regulates serotonin levels and depressive symptoms in fibromyalgia patients: Results of a small clinical trial." Journal of Clinical Psychopharmacology **34**(2): 277-278.

Amminger, G. P., B. Nelson, H. P. Yuen, C. Markulev, M. R. Schafer, M. Schlogelhofer, N. Mossaheb, S. Smesny, I. Hickie, G. Berger and et al. (2018). "Neurapro revisited: increases in long-chain omega-3 fatty acids improve functional and symptomatic outcomes in ultrahigh risk patients." **44**: S228

C223 - EMBASE 621901637.

Amsterdam, J. D., Q. S. Li, S. X. Xie and J. J. Mao (2020). "Putative Antidepressant Effect of Chamomile (Matricaria chamomilla L.) Oral Extract in Subjects with Comorbid Generalized Anxiety Disorder and Depression." Journal of Alternative and Complementary Medicine **26**(9): 813-819.

Andrade, C. (2021). "N-Acetylcysteine Augmentation for Patients With Major Depressive Disorder and Bipolar Depression." Journal of Clinical Psychiatry **82**(1 C7 - 21f13891).

Andreeva, V. A., P. Galan, M. Torrès, C. Julia, S. Hercberg and E. Kesse-Guyot (2012). "Supplementation with B vitamins or N-3 fatty acids and depressive symptoms in cardiovascular disease survivors: Ancillary findings from the SUpplementation with FOLate, vitamins B-6 and B-12 and/or OMega-3 fatty acids (SU.FOL.OM3) randomized trial." American Journal of Clinical Nutrition **96**(1): 208-214.

Antypa, N., A. H. M. Smelt, A. Strengholt and A. J. W. Van Der Does (2012). "Effects of omega-3 fatty acid supplementation on mood and emotional information processing in recovered depressed individuals." Journal of Psychopharmacology **26**(5): 738-743.

Appleton, K. M., P. D. Voyias, H. M. Sallis, S. Dawson, A. R. Ness, R. Churchill and R. Perry (2021). "Omega-3 fatty acids for depression in adults." Cochrane Database of Systematic Reviews **2021**(11 C7 - CD004692).

Arnold, L. E., A. S. Young, M. A. Belury, R. M. Cole, B. Gracious, A. M. Seidenfeld, H. Wolfson and M. A. Fristad (2017). "Omega-3 fatty acid plasma levels before and after supplementation: Correlations with mood and clinical outcomes in the omega-3 and therapy studies." Journal of Child and Adolescent Psychopharmacology **27**(3): 223-233.

Arnold, R., J. Murphy-Smith, C. H. Ng, D. Mischoulon, G. J. Byrne, C. A. Bousman, C. Stough, M. Berk and J. Sarris (2024). "Predictors of the placebo response in a nutraceutical randomized controlled trial for depression." Journal of Integrative Medicine **22**(1): 46-53.

Asadi, S., M. S. Gholami, F. Siassi, M. Qorbani and G. Sotoudeh (2019). "Beneficial effects of nano-curcumin supplement on depression and anxiety in diabetic patients with peripheral neuropathy: A randomized, double-blind, placebo-controlled clinical trial." Phytotherapy research : PTR.

Astorg, P., A. Couthouis, G. P. De Courcy, S. Bertrais, N. Arnault, P. Meneton, P. Galan and S. Hercberg (2008). "Association of folate intake with the occurrence of depressive episodes in middle-aged French men and women." British Journal of Nutrition **100**(1): 183-187.

Augusto da Silva Meleiro, A. M. (2000). "Treatment of mild and moderate depression: Evaluation of the effectiveness and tolerability of LI 160 S Hypericum perforatum extract." Revista Brasileira de Medicina **57**(11): 1313-1323.

Badrasawi, M. M., S. Shahar, Z. Abd Manaf and H. Haron (2013). "Effect of Talbinah food consumption on depressive symptoms among elderly individuals in long term care facilities, randomized clinical trial." **8**: 279‐285

C273 - PUBMED 23493965.

Bahrami, A., F. Nikoomanesh, H. Dehghani, H. Aramjoo and G. A. Ferns (2024). "Curcumin Ameliorates Depression, and Anxiety in Patients with Premenstrual Syndrome and Dysmenorrhea: A Triple-Blinded Placebo-Controlled Trial." Journal of Advances in Medical and Biomedical Research **32**(152): 174-184.

Barragán-Rodríguez, L., M. Rodríguez-Morán and F. Guerrero-Romero (2008). "Efficacy and safety of oral magnesium supplementation in the treatment of depression in the elderly with type 2 diabetes: A randomized, equivalent trial." Magnesium Research **21**(4): 218-223.

Bartova, L., M. Dold, H. P. Volz, E. Seifritz, H. J. Möller and S. Kasper (2023). "Beneficial effects of Silexan on co-occurring depressive symptoms in patients with subthreshold anxiety and anxiety disorders: randomized, placebo-controlled trials revisited." European Archives of Psychiatry and Clinical Neuroscience **273**(1): 51-63.

Bazinet, R. P., A. H. Metherel, C. T. Chen, S. R. Shaikh, A. Nadjar, C. Joffre and S. Layé (2020). "Brain eicosapentaenoic acid metabolism as a lead for novel therapeutics in major depression." Brain, Behavior, and Immunity **85**: 21-28.

Bazrafshan, M. R., M. Jokar, N. Shokrpour and H. Delam (2020). "The effect of lavender herbal tea on the anxiety and depression of the elderly: A randomized clinical trial." Complementary Therapies in Medicine **50**.

Bitarafan, S., A. Saboor-Yaraghi, M. A. Sahraian, D. Soltani, S. Nafissi, M. Togha, N. Beladi Moghadam, T. Roostaei, N. Mohammadzadeh Honarvar and M. H. Harirchian (2016). "Effect of Vitamin A Supplementation on fatigue and depression in Multiple Sclerosis patients: a Double-Blind Placebo-Controlled Clinical Trial." **15**(1): 13‐19

C13 - PUBMED 26996107,EMBASE 608134953.

Blampied, M., J. M. Tylianakis, C. Bell, C. Gilbert and J. J. Rucklidge (2023). "Efficacy and safety of a vitamin-mineral intervention for symptoms of anxiety and depression in adults: A randomised placebo-controlled trial “NoMAD”." Journal of Affective Disorders **339**: 954-964.

Borja-Hart, N. L. and J. Marino (2010). "Role of omega-3 fatty acids for prevention or treatment of perinatal depression." Pharmacotherapy **30**(2): 210-216.

Bot, M., I. A. Brouwer, M. Roca, E. Kohls, B. W. J. H. Penninx, E. Watkins, G. Van Grootheest, M. Cabout, U. Hegerl, M. Gili, M. Owens and M. Visser (2019). "Effect of Multinutrient Supplementation and Food-Related Behavioral Activation Therapy on Prevention of Major Depressive Disorder among Overweight or Obese Adults with Subsyndromal Depressive Symptoms: The MooDFOOD Randomized Clinical Trial." JAMA - Journal of the American Medical Association **321**(9): 858-868.

Bradley, H. A., E. Moltchanova, R. T. Mulder, L. Dixon, J. Henderson and J. J. Rucklidge (2024). "Efficacy and safety of a mineral and vitamin treatment on symptoms of antenatal depression: 12-week fully blinded randomised placebo-controlled trial (NUTRIMUM)." BJPsych Open **10**(4 C7 - e119).

Bradley, H. A., R. T. Mulder, J. M. T. Henderson, L. Dixon, E. Moltchanova and J. J. Rucklidge (2022). "A NUTRITIONAL APPROACH TO TREATING SYMPTOMS OF ANTENATAL DEPRESSION AND ANXIETY. THE NUTRIMUM TRIAL: A DOUBLE-BLIND, RANDOMISED CONTROLLED TRIAL." Journal of Paediatrics and Child Health **58**(SUPPL 2): 23.

Brattström, A. (2009). "Long-term effects of St. John's wort (Hypericum perforatum) treatment: A 1-year safety study in mild to moderate depression." Phytomedicine **16**(4): 277-283.

Brendler, T., J. A. Brinckmann, U. Feiter, N. Gericke, L. Lang, O. N. Pozharitskaya, A. N. Shikov, M. Smith and B. E. van Wyk (2021). "Sceletium for managing anxiety, depression and cognitive impairment: A traditional herbal medicine in modern-day regulatory systems." Current Neuropharmacology **19**(9): 1384-1400.

Brennan, B. P., J. E. Jensen, J. I. Hudson, C. E. Coit, A. Beaulieu, H. G. Pope, P. F. Renshaw and B. M. Cohen (2013). "A placebo-controlled trial of acetyl-l-carnitine and α-lipoic acid in the treatment of bipolar depression." Journal of Clinical Psychopharmacology **33**(5): 627-635.

Browne, P. D., A. C. Bolte, I. Besseling-van der Vaart, E. Claassen and C. de Weerth (2021). "Probiotics as a treatment for prenatal maternal anxiety and depression: a double-blind randomized pilot trial." Sci Rep **11**(1): 3051.

C. Curtin, A. and C. S. Johnston (2023). "Vitamin B6 Supplementation Reduces Symptoms of Depression in College Women Taking Oral Contraceptives: A Randomized, Double-Blind Crossover Trial." Journal of Dietary Supplements **20**(4): 550-562.

Cao, X. J., X. C. Huang and X. Wang (2019). "Effectiveness of Chinese herbal medicine granules and traditional Chinese medicine-based psychotherapy for perimenopausal depression in Chinese women: a randomized controlled trial." Menopause **26**(10): 1193-1203.

Cao, X. W., D. Guo, J. W. Liu, W. Niu, J. Liu, J. K. Pan, H. Xie, W. W. Ouyang and D. K. Lin (2016). "The efficacy and safety of the Shaoyao Shujin tablet for knee osteoarthritis: study protocol for a randomized controlled trial." Trials **17**: 3.

Carnegie, R., M. C. Borges, H. J. Jones, J. Zheng, P. Haycock, J. Evans and R. M. Martin (2024). "Omega-3 fatty acids and major depression: a Mendelian randomization study." Translational Psychiatry **14**(1 C7 - 222).

Carnegie, R. E., J. Zheng, M. C. Borges, H. J. Jones, K. H. Wade, H. M. Sallis, S. J. Lewis, D. M. Evans, J. A. Revez, J. Evans and R. M. Martin (2024). "Micronutrients and Major Depression: A Mendelian Randomisation Study." Nutrients **16**(21 C7 - 3690).

Chaban, О., O. O. Khaustova, D. O. Assonov and L. V. Sak (2023). "SAFETY AND EFFICACY OF THE COMPLEX DEPRILIUM® IN REDUCING SUBCLINICAL SYMPTOMS OF DEPRESSION IN PATIENTS WITH CHRONIC NON-COMMUNICABLE DISEASES: DOUBLE-BLIND RANDOMIZED CONTROLLED STUDY." Wiadomosci lekarskie (Warsaw, Poland : 1960) **76**(1): 136-144.

Chahwan, B., S. Kwan, A. Isik, S. van Hemert, C. Burke and L. Roberts (2019). "Gut feelings: A randomised, triple-blind, placebo-controlled trial of probiotics for depressive symptoms." Journal of Affective Disorders **253**: 317-326.

Chang, J. P. C., S. S. Chang, H. T. Chen, K. P. Su and C. M. Pariante (2018). "A double blind-placebo controlled trial of omega-3 polyunsaturated fatty acids (n-3 PUFAs) in patients with cardiovascular diseases (CVDs) and comorbid major depressive disorder (MDD)." Nutritional Neuroscience **21**: S11.

Chen, H. M., P. H. Kuo, C. Y. Hsu, Y. H. Chiu, Y. W. Liu, M. L. Lu and C. H. Chen (2021). "Psychophysiological effects of lactobacillus plantarum ps128 in patients with major depressive disorder: A preliminary 8-week open trial." Nutrients **13**(11 C7 - 3731).

Chhetry, B. T., A. Hezghia, J. M. Miller, S. Lee, H. Rubin-Falcone, T. B. Cooper, M. A. Oquendo, J. J. Mann and M. E. Sublette (2016). "Omega-3 polyunsaturated fatty acid supplementation and white matter changes in major depression." Journal of Psychiatric Research **75**: 65-74.

Chi, C. I. (2017). "Safety, effectiveness, and mechanism of fish oil as adjunct treatment for Major Depressive Disorder - a 12-month randomized, placebo controlled clinical trial."

Chinna Meyyappan, A., E. Forth and R. Milev (2022). "Microbial Ecosystem Therapeutic-2 Intervention in People With Major Depressive Disorder and Generalized Anxiety Disorder: Phase 1, Open-Label Study." Interact J Med Res **11**(1): e32234.

Chong, M. F. F., J. X. Y. Wong, M. Colega, L. W. Chen, R. M. van Dam, C. S. Tan, A. L. Lim, S. Cai, B. F. P. Broekman, Y. S. Lee, S. M. Saw, K. Kwek, K. M. Godfrey, Y. S. Chong, P. Gluckman, M. J. Meaney, H. Chen, P. Agarwal, A. Biswas, C. L. Bong, J. K. Yen Chan, Y. H. Chan, C. Y. I. Chee, Y. B. Cheung, A. Chia, A. Chinnadurai, C. K. Chng, S. C. Chong, M. C. Chua, C. M. Ding, E. A. Finkelstein, D. Fok, M. Fortier, A. E. N. Goh, Y. T. D. Goh, J. J. Gooley, W. M. Han, M. Hanson, C. J. Henry, J. D. Holbrook, C. Y. Hsu, H. Inskip, J. Kapur, I. Yee-Man Lau, B. W. Lee, N. Lek, S. B. Lim, Y. L. Low, I. Magiati, L. M. Daniel, C. Ngo, K. Naiduvaje, W. W. Pang, A. Qiu, B. L. Quah, V. S. Rajadurai, M. Rauff, S. A. Rebello, J. L. Richmond, A. Rifkin-Graboi, L. Pei-Chi Shek, A. Sheppard, B. Shuter, L. Singh, S. E. Soh, W. Stunkel, L. L. Su, K. H. Tan, O. H. Teoh, M. T. Tint, H. P. S. van Bever, I. B. Yun Wong, P. C. Wong, F. Yap and G. S. Heong Yeo (2014). "Relationships of maternal folate and vitamin B12 status during pregnancy with perinatal depression: The GUSTO study." Journal of Psychiatric Research **55**(1): 110-116.

Choukri, M. A., T. S. Conner, J. J. Haszard, M. J. Harper and L. A. Houghton (2018). "Effect of vitamin D supplementation on depressive symptoms and psychological wellbeing in healthy adult women: A double-blind randomised controlled clinical trial." Journal of Nutritional Science **7**.

Coët, A., F. M. Blampied and J. J. Rucklidge (2025). "1-year naturalistic follow-up of a Randomised Double-Blind, Placebo-Controlled Trial (“NoMAD”) Exploring the Effectiveness of Micronutrients in Improving Symptoms of Anxiety and Depression." Journal of Affective Disorders Reports **20**.

Cropley, M., A. P. Banks and J. Boyle (2015). "The Effects of Rhodiola rosea L. Extract on Anxiety, Stress, Cognition and Other Mood Symptoms." Phytotherapy Research **29**(12): 1934-1939.

Ctis (2025). "EFFECTIVENESS OF SUPPLEMENTATION WITH VITAMIN D IN PATIENTS WITH DEPRESSIVE DISORDER OR BIPOLAR DISORDER: a RANDOMIZED CONTROLLED CLINICAL TRIAL."

Dabbaghmanesh, M. H., F. Vaziri, F. Najib, S. Nasiri and S. Pourahmad (2019). "The effect of vitamin D consumption during pregnancy on maternal thyroid function and depression: A randomized, placebo-controlled, clinical trial." Jundishapur Journal of Natural Pharmaceutical Products **14**(2 C7 - e65328).

de Koning, E. J., P. Lips, B. Penninx, P. J. M. Elders, A. C. Heijboer, M. den Heijer, P. M. Bet, H. W. J. van Marwijk and N. M. van Schoor (2019). "Vitamin D supplementation for the prevention of depression and poor physical function in older persons: the D-Vitaal study, a randomized clinical trial." **110**(5): 1119‐1130

C1113 - PUBMED 31340012,EMBASE 628896621.

de Koning, E. J., N. L. van der Zwaluw, J. P. van Wijngaarden, E. Sohl, E. M. Brouwer-Brolsma, H. W. van Marwijk, A. W. Enneman, K. M. Swart, S. C. van Dijk, A. C. Ham, N. van der Velde, A. G. Uitterlinden, B. W. Penninx, P. J. Elders, P. Lips, R. A. Dhonukshe-Rutten, N. M. van Schoor and L. C. de Groot (2016). "Effects of Two-Year Vitamin B(12) and Folic Acid Supplementation on Depressive Symptoms and Quality of Life in Older Adults with Elevated Homocysteine Concentrations: Additional Results from the B-PROOF Study, an RCT." Nutrients **8**(11).

de Sousa-Muñoz, R. L. and R. G. Filizola (2009). "Efficacy of soy isoflavones for depressive symptoms of the climacteric syndrome." Maturitas **63**(1): 89-93.

Di Pierro, F., R. Orsi and R. Settembre (2015). "Role of betaine in improving the antidepressant effect of S-adenosyl-methionine in patients with mild-to-moderate depression." Journal of Multidisciplinary Healthcare **8**: 39-45.

Effati Daryani, F., S. M. A. Charandabi, S. Zarei, A. Mohammadi and M. Mirghafourvand (2017). "Effect of lavender cream with or without footbath on anxiety, stress and depression of women in postpartum: A clinical randomized controlled trial." Iranian Journal of Obstetrics, Gynecology and Infertility **20**(10): 52-61.

Esmaily, H., A. Sahebkar, M. Iranshahi, S. Ganjali, A. Mohammadi, G. Ferns and M. Ghayour-Mobarhan (2015). "An investigation of the effects of curcumin on anxiety and depression in obese individuals: A randomized controlled trial." Chin J Integr Med **21**(5): 332-338.

Fard, F. E., M. Mirghafourvand, S. Mohammad-Alizadeh Charandabi, A. Farshbaf-Khalili, Y. Javadzadeh and H. Asgharian (2017). "Effects of zinc and magnesium supplements on postpartum depression and anxiety: A randomized controlled clinical trial." Women and Health **57**(9): 1115-1128.

Farioli-Vecchioli, S., S. Sacchetti, N. V. di Robilant and D. Cutuli (2018). "The role of physical exercise and omega-3 fatty acids in depressive illness in the elderly." Current Neuropharmacology **16**(3): 308-326.

Fava, M., J. S. Borus, J. E. Alpert, A. A. Nierenberg, J. F. Rosenbaum and T. Bottiglieri (1997). "Folate, vitamin B12, and homocysteine in major depressive disorder." American Journal of Psychiatry **154**(3): 426-428.

Fava, M., R. Gomeni, J. Komorowski, J. Docherty, C. Cusin, D. Soskin and D. Mischoulon (2012). "A re-analysis using a population-enrichment strategy of a double-blind, placebo-controlled study of chromium picolinate in atypical depression." Neuropsychopharmacology **38**: S341-S342.

Fava, M., S. Lamon-Fava, J. So, D. Mischoulon, A. Nierenberg, B. Dunlop, P. Schettler, B. Kinkead, T. Ziegler and M. Rapaport (2019). "Eicosapentaenoic acid (EPA) supplementation in major depressive disorder patients with chronic inflammation: Dose-dependent increases in EPA metabolites and specialized pro-resolving lipid mediators." Neuropsychopharmacology **44**: 451-452.

Fedotova, J., Z. Bereza and T. Dudnichenko (2020). "Efficacy of escitalopram in combination with vitamin D3 supplementation in the depressed perimenopausal women." European Psychiatry **63**: S109-S110.

Firoozabadi, A., S. Kolouri, M. M. Zarshenas, A. Salehi, S. H. Mosavat and S. A. Dastgheib (2016). "Efficacy of Nepeta Menthoides Boiss and Buhse Freeze-Dried Aqueous Extract on Anxiety of Patients with Depression: A Double-Blind Randomized Controlled Clinical Trial." **41**(3 Suppl): S4

C3 - PUBMED 27840470.

Firoozabadi, A., S. Kolouri, M. M. Zarshenas, A. Salehi, S. H. Mosavat and S. A. Dastgheib (2017). "Efficacy of a freeze-dried aqueous extract of Nepeta menthoides Boiss. & Buhse in the treatment of anxiety in patients with depression: A double-blind, randomized, controlled trial." Journal of Herbal Medicine **10**: 17-23.

Foshati, S., A. Ghanizadeh and M. Akhlaghi (2022). "Extra-Virgin Olive Oil Improves Depression Symptoms Without Affecting Salivary Cortisol and Brain-Derived Neurotrophic Factor in Patients With Major Depression: a Double-Blind Randomized Controlled Trial." **122**(2): 284‐297.e281

C283 - PUBMED 34358723,EMBASE 635814426.

Freeman, M. P., J. R. Hibbeln, M. Silver, A. M. Hirschberg, B. Wang, A. M. Yule, L. F. Petrillo, E. Pascuillo, N. I. Economou, H. Joffe and L. S. Cohen (2011). "Omega-3 fatty acids for major depressive disorder associated with the menopausal transition: A preliminary open trial." Menopause **18**(3): 279-284.

Freeman, M. P., J. R. Hibbeln, K. L. Wisner, B. H. Brumbach, M. Watchman and A. J. Gelenberg (2006). "Randomized dose-ranging pilot trial of omega-3 fatty acids for postpartum depression." Acta Psychiatrica Scandinavica **113**(1): 31-35.

Freeman, M. P., J. R. Hibbeln, K. L. Wisner, M. Watchman and A. J. Gelenberg (2006). An open trial of Omega-3 fatty acids for depression in pregnancy. Acta Neuropsychiatrica.

Freeman, M. P., G. M. Savella, T. R. Church, L. Goéz-Mogollón, A. Z. Sosinsky, O. B. Noe, A. Kaimal and L. S. Cohen (2019). "A prenatal supplement with methylfolate for the treatment and prevention of depression in women trying to conceive and during pregnancy." Annals of Clinical Psychiatry **31**(1): 4-16.

Fries, L. R., M. Boehme, L. Lavalle, O. Sakwinska, F. Chughlay, S. Keddani, N. Porta, M. Vicario, G. Bergonzelli, I. Silva Zolezzi and S. Y. Chan (2025). "The impact of ingestion of Bifidobacterium longum NCC3001 on perinatal anxiety and depressive symptoms: a randomized controlled trial." Sci Rep **15**(1): 11250.

Fulzele, A. V., N. Ingle, M. N. Huda and D. S. Mishra (2014). "Comparative study to evaluate the effect of a herbal preparation & shirodhara in the management of major depressive disorder." International Journal of PharmTech Research **6**(2): 506-511.

Gabbay, V., R. D. Freed, C. M. Alonso, S. Senger, J. Stadterman, B. A. Davison and R. G. Klein (2018). "A double-blind placebo-controlled trial of omega-3 fatty acids as a monotherapy for adolescent depression." Journal of Clinical Psychiatry **79**(4 C7 - 17m11596).

Gariballa, S. and S. Forster (2007). "Effects of dietary supplements on depressive symptoms in older patients: A randomised double-blind placebo-controlled trial." Clinical Nutrition **26**(5): 545-551.

Gawlik-Kotelnicka, O., A. Margulska, A. Skowrońska and D. Strzelecki (2023). "PRO-DEMET Randomized Controlled Trial on Probiotics in Depression-Pilot Study Results." Nutrients **15**(6).

Gawlik-Kotelnicka, O., J. Rogalski, K. H. Czarnecka-Chrebelska, J. Burzyński, P. Jakubowska, A. Skowrońska and D. Strzelecki (2024). "The Interplay Between Depression, Probiotics, Diet, Immunometabolic Health, the Gut, and the Liver—A Secondary Analysis of the Pro-Demet Randomized Clinical Trial." Nutrients **16**(23 C7 - 4024).

Gawlik-Kotelnicka, O., A. Skowrońska, A. Margulska, K. H. Czarnecka-Chrebelska, I. Łoniewski, K. Skonieczna-żydecka and D. Strzelecki (2021). "The influence of probiotic supplementation on depressive symptoms, inflammation, and oxidative stress parameters and fecal microbiota in patients with depression depending on metabolic syndrome comorbidity—pro-demet randomized study protocol." Journal of Clinical Medicine **10**(7 C7 - 1342).

Gawlik-Kotelnicka, O., A. Skowrońska, A. Margulska and D. Strzelecki (2023). "The Influence of Probiotic Supplementation on Depression, Anxiety, and Stress Level, as well as Inflammation, Anthropometric and Metabolic Parameters in Patients with Depressive Disorders-preliminary results of an RCT." European Psychiatry **66**: S841-S842.

Gecele, M., G. Francesetti and A. Meluzzi (1991). "Acetyl-L-carnitine in aged subjects with major depression: Clinical efficacy and effects on the circadian rhythm of cortisol." Dementia **2**(6): 333-337.

Ghamchini, V. M., M. Salami, G. R. Mohammadi, Z. Moradi, A. Kavosi, A. Movahedi, M. Bidkhori and M. R. Aryaeefar (2019). "The effect of chamomile tea on anxiety and depression in cancer patients treated with chemotherapy." Journal of Young Pharmacists **11**(3): 309-312.

Gharekhani, A., M. R. Khatami, S. Dashti-Khavidaki, E. Razeghi, A. A. Noorbala, S. S. Hashemi-Nazari and M. A. Mansournia (2014). "The effect of omega-3 fatty acids on depressive symptoms and inflammatory markers in maintenance hemodialysis patients: A randomized, placebo-controlled clinical trial." European Journal of Clinical Pharmacology **70**(6): 655-665.

Gilbert, G. E. W. A. H. (2010). "Omega-3 fatty acids not effective for depression in patients with coronary heart disease." Journal of the National Medical Association **102**(2): 151.

Ginsberg, D. L. (2003). "Chromium Picolinate Treatment of Atypical Depression." Primary Psychiatry **10**(4): 23-24.

Grobler, A. C., G. Matthews and G. Molenberghs (2014). "The impact of missing data on clinical trials: A re-analysis of a placebo controlled trial of Hypericum perforatum (St Johns wort) and sertraline in major depressive disorder." Psychopharmacology **231**(9): 1987-1999.

Haberka, M., K. Mizia-Stec, M. Mizia, K. Gieszczyk, A. Chmiel, K. Sitnik-Warchulska and Z. Gasior (2012). "Effects of n-3 polyunsaturated fatty acids on depressive symptoms, anxiety and emotional state in patients with acute myocardial infarction." **19**(1): S42

C43 - EMBASE 71706389.

Haghighat, N., S. Rajabi and M. Mohammadshahi (2021). "Effect of synbiotic and probiotic supplementation on serum brain-derived neurotrophic factor level, depression and anxiety symptoms in hemodialysis patients: a randomized, double-blinded, clinical trial." Nutritional Neuroscience **24**(6): 490-499.

Hallahan, B., T. Ryan, J. R. Hibbeln, I. T. Murray, S. Glynn, C. E. Ramsden, J. P. SanGiovanni and J. M. Davis (2016). "Efficacy of omega-3 highly unsaturated fatty acids in the treatment of depression." British Journal of Psychiatry **209**(3): 192-201.

Hamlbar, E. P., M. Mirghafourvand, E. Shaseb and M. Kamalifard (2025). "The effect of curcumin on postpartum depression and anxiety in primiparous women: a double-blind randomized placebo-controlled clinical trial." BMC Complementary Medicine and Therapies **25**(1).

Hatton, D. C., J. Harrison-Hohner, J. Matarazzo, P. Edwards, A. Lewy and L. Davis (2007). "Missed antenatal depression among high risk women: A secondary analysis." Archives of Women's Mental Health **10**(3): 121-123.

Haybar, H., A. Z. Javid, M. H. Haghighizadeh, E. Valizadeh, S. M. Mohaghegh and A. Mohammadzadeh (2018). "The effects of Melissa officinalis supplementation on depression, anxiety, stress, and sleep disorder in patients with chronic stable angina." Clinical Nutrition ESPEN **26**: 47-52.

Heaton, J. L., S. A. Campbell, H. A. Bradley, R. T. Mulder, L. Dixon, J. Henderson and J. J. Rucklidge (2025). "Broad-Spectrum Micronutrients or Antidepressants for Antenatal Depression: Effect on Maternal and Infant Birth Outcomes in an Observational Secondary Analysis of NUTRIMUM." Journal of Clinical Psychopharmacology **45**(1): 4-15.

Hellem, T. L., Y.-H. Sung, X.-F. Shi, M. A. Pett, G. Latendresse, J. Morgan, R. S. Huber, D. Kuykendall, K. J. Lundberg and P. F. Renshaw (2015). "Creatine as a novel treatment for depression in females using methamphetamine: A pilot study." Journal of Dual Diagnosis **11**(3-4): 189-202.

Hidese, S., M. Ota, C. Wakabayashi, T. Noda, H. Ozawa, T. Okubo and H. Kunugi (2017). "Effects of chronic l-theanine administration in patients with major depressive disorder: An open-label study." Acta Neuropsychiatrica **29**(2): 72-79.

Ho, Y. T., Y. C. Tsai, T. B. J. Kuo and C. C. H. Yang (2021). "Effects of lactobacillus plantarum ps128 on depressive symptoms and sleep quality in self-reported insomniacs: A randomized, double-blind, placebo-controlled pilot trial." Nutrients **13**(8 C7 - 2820).

Holsboen, F., O. Benkert, L. Meier and A. Kreuz-Kersting (1985). "Combined estradiol and vitamin B6 treatment in women with major depression." The American journal of psychiatry **142**(5): 658.

Hulkkonen, P., E. L. Kataja, T. Vahlberg, E. Koivuniemi, N. Houttu, O. Pellonperä, K. Mokkala, H. Karlsson and K. Laitinen (2021). "The efficacy of probiotics and/or n-3 long-chain polyunsaturated fatty acids intervention on maternal prenatal and postnatal depressive and anxiety symptoms among overweight and obese women." Journal of Affective Disorders **289**: 21-30.

Ibero-Baraibar, I., A. Perez-Cornago, M. J. Ramirez, J. A. Martinez and M. A. Zulet (2015). "An increase in plasma homovanillic acid with cocoa extract consumption is associated with the alleviation of depressive symptoms in overweight or obese adults on an energy restricted diet in a randomized controlled trial." **146**(4): 897S‐904S

C893 - PUBMED 26962189,EMBASE 610003794.

Irct138901233695N (2010). "Effect of vitamins in treatment of depression."

Irct201109297669N (2011). "The impact of Omga-3 fatty acids on depression."

Irct2014041916465N (2014). "Evaluating the effects of Crocus sativus L. (Saffron) on weight and depression."

Irct2014082518922N (2014). "Effect of Folic acid supplementation on the therapeutic regimen for major depressive disorder."

Irct2017082612438N (2017). "The effect of barij deprex capsule on Depressive symptoms."

Irct20170311033004N (2022). "Effects of alkaloids of harmala seeds on depression."

Irct20240107060642N (2024). "Investigating the effect of folic acid supplementation on postpartum depression in women."

Irct20240619062173N (2024). "Effect of Cuscuta epithymum on depression."

Islambulchilar, M., M. Morovati, Y. Morsali, S. Rezaee and A. Esmaeilzadeh (2023). "Could one-milligram daily folic acid supplementation change the intensity of the disorder in outpatients with depression who use citalopram?" Koomesh **25**(5).

Isrctn (2024). "Probiotic supplementation for maternal depression."

j57vj, R. B. R. (2024). "Effect of Magnesium Chloride supplementation on Depression."

Jam, I. N., A. H. Sahebkar, S. Eslami, N. Mokhber, M. Nosrati, M. Khademi, M. Foroutan-Tanha, M. Ghayour-Mobarhan, F. Hadizadeh, G. Ferns and M. Abbasi (2017). "The effects of crocin on the symptoms of depression in subjects with metabolic syndrome." Advances in Clinical and Experimental Medicine **26**(6): 925-930.

Javadinia, S. A., R. Salek, M. Dehghani, S. A. Mohajeri, A. Talaei and A. Fanipakdel (2021). "Amelioration of anxiety, depression, and chemotherapy related toxicity after crocin administration during chemotherapy of breast cancer: A double blind, randomized clinical trial." Annals of Oncology **32**: S439-S440.

Jeong, H. G., Y. H. Ko, S. Y. Oh, C. Han, T. Kim and S. H. Joe (2015). "Effect of Korean Red Ginseng as an adjuvant treatment for women with residual symptoms of major depression." Asia-Pacific Psychiatry **7**(3): 330-336.

Jiang, W., D. J. Whellan, K. F. Adams, M. A. Babyak, S. H. Boyle, J. L. Wilson, C. B. Patel, J. G. Rogers, W. S. Harris and C. M. O'Connor (2018). "Long-Chain Omega-3 Fatty Acid Supplements in Depressed Heart Failure Patients: Results of the OCEAN Trial." JACC: Heart Failure **6**(10): 833-843.

Jokar, A. and F. Farahi (2014). "Effect of vitamin C on depression in menopausal women with balanced diet: A randomized clinical trial." Iranian Journal of Obstetrics, Gynecology and Infertility **17**(121): 18-23.

Jorde, R. and J. Kubiak (2018). "No improvement in depressive symptoms by vitamin D supplementation: Results from a randomised controlled trial." Journal of Nutritional Science **7**.

Jorde, R., M. Sneve, Y. Figenschau, J. Svartberg and K. Waterloo (2008). "Effects of vitamin D supplementation on symptoms of depression in overweight and obese subjects: Randomized double blind trial." Journal of Internal Medicine **264**(6): 599-609.

Jurcic, J., J. A. Pereira and D. Kavanaugh (2007). "St John's wort versus paroxetine for depression." Canadian Family Physician **53**(9): 1511-1513.

Karakula-Juchnowicz, H., J. Rog, D. Juchnowicz, I. Łoniewski, K. Skonieczna-Ydecka, P. Krukow, M. Futyma-Jedrzejewska and M. Kaczmarczyk (2019). "The study evaluating the effect of probiotic supplementation on the mental status, inflammation, and intestinal barrier in major depressive disorder patients using gluten-free or gluten-containing diet (SANGUT study): A 12-week, randomized, double-blind, and placebo-controlled clinical study protocol." Nutrition Journal **18**(1).

Karimi, F. Z., H. Hosseini, S. R. Mazloom and H. Rakhshandeh (2021). "Effect of oral capsule of Ocimum basilicum leaf extract on depression in menopausal women: A randomized clinical trial." Iranian Journal of Obstetrics, Gynecology and Infertility **24**(2): 50-58.

Kasper, D. C., J. A. Widness, N. Haiden, A. Berger, M. Hayde, A. Pollak and K. R. Herkner (2009). "Characterization and differentiation of iron status in anemic very low birth weight infants using a diagnostic nomogram." Neonatology **95**(2): 164-171.

Kasper, S., F. Caraci, B. Forti, F. Drago and E. Aguglia (2010). "Efficacy and tolerability of Hypericum extract for the treatment of mild to moderate depression." European Neuropsychopharmacology **20**(11): 747-765.

Kasper, S., M. Gastpar, W. E. Müller, H. P. Volz, A. Dienel and H. J. Möller (2008). "Treatment of depression with St. John's wort extract WS® 5570. No influence on patients' weight." Psychopharmakotherapie **15**(1): 6-10.

Kasper, S., E. Seifritz and H. P. Volz (2024). "Efficacy of Silexan in Patients with a Major Depressive Episode - First Results from a Multi-centre, Doubleblind, Randomised, Placebo- and Reference-controlled Phase III Trial." European Psychiatry **67**: S224-S225.

Katrenčíková, B., M. Vaváková, Z. Paduchová, Z. Nagyová, I. Garaiova, J. Muchová, Z. Ďuračková and J. Trebatická (2021). "Oxidative stress markers and antioxidant enzymes in children and adolescents with depressive disorder and impact of omega-3 fatty acids in randomised clinical trial." Antioxidants **10**(8 C7 - 1256).

Kazemi, A., A. A. Noorbala, K. Azam, M. H. Eskandari and K. Djafarian (2019). "Effect of probiotic and prebiotic vs placebo on psychological outcomes in patients with major depressive disorder: A randomized clinical trial." Clinical Nutrition **38**(2): 522-528.

Kazemi, A., A. A. Noorbala and K. Djafarian (2020). "Effect of probiotic and prebiotic versus placebo on appetite in patients with major depressive disorder: post hoc analysis of a randomised clinical trial." Journal of Human Nutrition and Dietetics **33**(1): 56-65.

Keshavarz, S. A., S. A. Mostafavi, S. Akhondzadeh, M. R. Mohammadi, S. Hosseini, M. R. Eshraghian and M. Chamari (2018). "Omega-3 supplementation effects on body weight and depression among dieter women with co-morbidity of depression and obesity compared with the placebo: A randomized clinical trial." Clinical Nutrition ESPEN **25**: 37-43.

Khaddage, R., M. Bassil and B. Rahi (2023). "Vitamin D Supplementation Improves Depressive Symptomatology Among Vitamin D Deficient Saudi Arabian Adults - A Randomized Clinical Trial." Current Developments in Nutrition **7**.

Khajehnasiri, F., S. Akhondzadeh, S. B. Mortazavi, A. Allameh, G. Sotoudeh, A. Khavanin and Z. Zamanian (2015). "Are supplementation of omega-3 and ascorbic acid effective in reducing oxidative stress and depression among depressed shift workers?" International Journal for Vitamin and Nutrition Research **85**(5-6): 299-310.

Khalatbari Mohseni, G., S. A. Hosseini, N. Majdinasab and B. Cheraghian (2023). "Effects of N-acetylcysteine on oxidative stress biomarkers, depression, and anxiety symptoms in patients with multiple sclerosis." Neuropsychopharmacology Reports **43**(3): 382-390.

Khalili, M., H. Karimi and S. Sadri (2011). "Effect of fish oil supplement and weight loss diet on depression in obese women." Obesity Reviews **12**: 70.

Khanna, A., F. John, S. Das, J. Thomas, J. Rao, B. Maliakel and K. Im (2020). "Efficacy of a novel extract of fenugreek seeds in alleviating vasomotor symptoms and depression in perimenopausal women: A randomized, double-blinded, placebo-controlled study." Journal of food biochemistry **44**(12): e13507.

Khodadad, M., P. Bahadoran, G. Kheirabadi and A. Sabzghabaee (2021). "Can Vitamin B6 help to prevent postpartum depression? A randomized controlled trial." International Journal of Preventive Medicine **12**(1 C7 - 12).

Khorvash, F., P. Hajihashemi, G. Askari, M. Noorian and M. R. Maracy (2019). "The effects of combined supplementation of coenzyme Q10 with L-carnitine on migraine-related disability and depression among patient with migraine: A randomized, placebo-controlled, double-blind trial." Journal of the Neurological Sciences **405**: 106.

Kieser, M. and A. Szegedi (2005). "Predicting stable treatment response in patients with major depression treated with hypericum extract WS® 5570/5572." Pharmacopsychiatry **38**(5): 194-200.

Kim, Y., Y. Choi, M. Y. Lee, S. H. Cho, I. C. Jung, D. H. Kang and C. Yang (2023). "Bangpungtongsung-san for patients with major depressive disorder: study protocol for a randomized controlled phase II clinical trial." BMC Complement Med Ther **23**(1): 114.

Kious, B. M., H. Sabic, Y.-H. Sung, D. G. Kondo and P. Renshaw (2017). "An open-label pilot study of combined augmentation with creatine monohydrate and 5-hydroxytryptophan for selective serotonin reuptake inhibitor–or serotonin-norepinephrine reuptake inhibitor–resistant depression in adult women." Journal of clinical psychopharmacology **37**(5): 578-583.

Komorniak, N., M. Kaczmarczyk, I. Łoniewski, A. Martynova-Van Kley, A. Nalian, M. Wroński, K. Kaseja, B. Kowalewski, M. Folwarski and E. Stachowska (2023). "Analysis of the Efficacy of Diet and Short-Term Probiotic Intervention on Depressive Symptoms in Patients after Bariatric Surgery: A Randomized Double-Blind Placebo Controlled Pilot Study." Nutrients **15**(23 C7 - 4905).

Kotlega, D., A. Zembron-Lacny, M. Golab-Janowska, P. Nowacki and M. Szczuko (2020). "The association of free fatty acids and eicosanoids with the severity of depressive symptoms in stroke patients." International Journal of Molecular Sciences **21**(15 C7 - 5220): 1-18.

Lagzi, N., A. Bateni, R. Goli and N. Talebiazar (2023). "The effect of multivitamins on anxiety and depression in patients undergoing methadone maintenance treatment: A double-blind randomized controlled trial." International Journal of Psychiatry in Medicine **58**(6): 576-590.

Lamon-Fava, S., M. Liu, B. W. Dunlop, B. Kinkead, P. J. Schettler, J. C. Felger, T. R. Ziegler, M. Fava, D. Mischoulon and M. H. Rapaport (2023). "Clinical response to EPA supplementation in patients with major depressive disorder is associated with higher plasma concentrations of pro-resolving lipid mediators." Neuropsychopharmacology **48**(6): 929-935.

Lc, R., B. Em, H. Db, S. H and P. Ar (2021). "A pilot randomized controlled trial testing supplements of omega-3 fatty acids, probiotics, combination or placebo on symptoms of depression, anxiety and stress." Journal of Affective Disorders Reports **5 C7 - 100141**.

Lecrubier, Y., G. Clerc, R. Didi and M. Kieser (2002). "Efficacy of St. John's wort extract WS 5570 in major depression: A double-blind, placebo-controlled trial." American Journal of Psychiatry **159**(8): 1361-1366.

Lee, Y. J. and K. Y. Sohng (2019). "Effects of Vitamin D on depression, cognitive function, and physical function in elderly individuals living alone." International Journal of Gerontology **13**(3): 196-201.

Lespérance, F., N. Frasure-Smith, E. St-André, G. Turecki, P. Lespérance and S. R. Wisniewski (2011). "The efficacy of omega-3 supplementation for major depression: A randomized controlled trial." Journal of Clinical Psychiatry **72**(8): 1054-1062.

Levant, B. (2011). Brain N-3 (Omega-3) fatty acids in neurochemistry, behavior and mood: Implications for the treatment and prevention of non-puerperal and postpartum depression. Lipids: Categories, Biological Functions and Metabolism, Nutrition and Health**:** 127-152.

Liang, Z. H., Y. B. Jia, M. L. Wang, Z. R. Li, M. Li, Y. L. Yun and R. X. Zhu (2019). "Efficacy of ginkgo biloba extract as augmentation of venlafaxine in treating post-stroke depression." Neuropsychiatric Disease and Treatment **15**: 2551-2557.

Lin, C., S. H. Lee, C. M. Huang, Y. W. Wu, Y. X. Chang, H. L. Liu, S. H. Ng, Y. C. Cheng, C. C. Chiu and S. C. Wu (2024). "Cognitive protection and brain entropy changes from omega-3 polyunsaturated fatty acids supplement in late-life depression: A 52-week randomized controlled trial." Journal of Affective Disorders **351**: 15-23.

Lin, K., Y. Li, E. D. Toit, L. Wendt and J. Sun (2021). "Effects of Polyphenol Supplementations on Improving Depression, Anxiety, and Quality of Life in Patients With Depression." Frontiers in Psychiatry **12 C7 - 765485**.

Lingærde, O., A. R. Føreland and A. Magnusson (1999). "Can winter depression be prevented by Ginkgo biloba extract? A placebo- controlled trial." Acta Psychiatrica Scandinavica **100**(1): 62-66.

Lipovac, M., P. Chedraui, C. Gruenhut, A. Gocan, M. Stammler and M. Imhof (2010). "Improvement of postmenopausal depressive and anxiety symptoms after treatment with isoflavones derived from red clover extracts." Maturitas **65**(3): 258-261.

Loas, G., D. Rose, P. Nowaczkowski, P. Lernout and B. Duron (1996). "Proportion of non-responsive patient to amitriptyline: Exploratory pilot study of the French center. A report from the WHO project on the combined utilization of an antidepressant (amitriptyline) and an antioxidant (ginkgo biloba) in the treatment of therapy-resistant depression." Annales Medico-Psychologiques **154**(3): 202-203.

Lopresti, A. L., M. Maes, G. L. Maker, S. D. Hood and P. D. Drummond (2014). "Curcumin for the treatment of major depression: A randomised, double-blind, placebo controlled study." Journal of Affective Disorders **167**: 368-375.

Lopresti, A. L., S. J. Smith, S. D. Hood and P. D. Drummond (2019). "Efficacy of a standardised saffron extract (affron®) as an add-on to antidepressant medication for the treatment of persistent depressive symptoms in adults: A randomised, double-blind, placebo-controlled study." Journal of Psychopharmacology **33**(11): 1415-1427.

Lopresti, A. L., S. J. Smith, H. Malvi and R. Kodgule (2019). "An investigation into the stress-relieving and pharmacological actions of an ashwagandha (Withania somnifera) extract: a randomized, double-blind, placebo-controlled study." **98**(37): e17186

C17183 - PUBMED 31517876.

Lopresti, A. L., S. J. Smith, W. Marx, M. Díez-Municio and M. I. Morán-Valero (2025). "An Examination into the Effects of a Saffron Extract (Affron) on Mood and General Wellbeing in Adults Experiencing Low Mood: A Randomized, Double-Blind, Placebo-Controlled Trial." Journal of Nutrition.

Loria-Kohen, V., C. Gómez-Candela, S. Palma-Milla, B. Amador-Sastre, A. Hernanz and L. M. Bermejo (2013). "A pilot study of folic acid supplementation for improving homocysteine levels, cognitive and depressive status in eating disorders." Nutricion Hospitalaria **28**(3): 807-815.

Lu, J., H. Mao, Y. Tan and G. Luo (2024). "Associations of Dietary Intake of Vitamin B6 and Plasma Pyridoxal 5′-Phosphate Level With Depression in US Adults: Findings From NHANES 2005–2010." Brain and Behavior **14**(11 C7 - e70128).

Lucas, M., G. Asselin, C. Mérette, M. J. Poulin and S. Dodin (2009). "Ethyl-eicosapentaenoic acid for the treatment of psychological distress and depressive symptoms in middle-aged women: A double-blind, placebo-controlled, randomized clinical trial." American Journal of Clinical Nutrition **89**(2): 641-651.

Lucas, M., F. Mirzaei, E. J. O'Reilly, A. Pan, W. C. Willett, I. Kawachi, K. Koenen and A. Ascherio (2011). "Dietary intake of n-3 and n-6 fatty acids and the risk of clinical depression in women: A 10-y prospective follow-up study." American Journal of Clinical Nutrition **93**(6): 1337-1343.

Luger, M., R. Kruschitz, R. Marculescu, C. Klammer, M. Adelfang, M. Kral, F. Hoppichler, F. Langer, G. Prager, K. Schindler and et al. (2015). "Depressive symptoms and associations with low vitamin d levels and adherence to mediterranean diet in morbidly obese patients." **8**: 188

C183 - EMBASE 71907835.

Luke, A. K., E. W. Evans, D. S. Bond and J. G. Thomas (2016). "Associations between omega fatty acid consumption and depressive symptoms among individuals seeking behavioural weight loss treatment." Obesity Science and Practice **2**(1): 75-82.

Mac Giollabhui, N., D. Mischoulon, B. W. Dunlop, B. Kinkead, P. J. Schettler, R. T. Liu, O. I. Okereke, S. Lamon-Fava, M. Fava and M. H. Rapaport (2023). "Individuals with depression exhibiting a pro-inflammatory phenotype receiving omega-3 polyunsaturated fatty acids experience improved motivation-related cognitive function: Preliminary results from a randomized controlled trial." Brain, Behavior, and Immunity - Health **32 C7 - 100666**.

Mahboobi, S., M. Ghasvarian, H. Ghaem, H. Alipour, S. Alipour and M. H. Eftekhari (2022). "Effects of probiotic and magnesium co-supplementation on mood, cognition, intestinal barrier function and inflammation in individuals with obesity and depressed mood: A randomized, double-blind placebo-controlled clinical trial." Frontiers in Nutrition **9 C7 - 1018357**.

Majeed, M., K. Nagabhushanam, A. Murali, D. T. Vishwanathan, R. V. Mamidala and L. Mundkur (2024). "A Standardized Withania somnifera (Linn.) Root Extract with Piperine Alleviates the Symptoms of Anxiety and Depression by Increasing Serotonin Levels: A Double-Blind, Randomized, Placebo-Controlled Study." Journal of Integrative and Complementary Medicine **30**(5): 459-468.

Malaguarnera, G., G. Bertino, G. Chisari, M. Motta, M. Vecchio, M. Vacante, F. Caraci, C. Greco, F. Drago, G. Nunnari and et al. (2016). "Silybin supplementation during HCV therapy with pegylated interferon-a plus ribavirin reduces depression and anxiety and increases work ability." **16**(1).

Mannel, M., U. Kuhn, U. Schmidt, M. Ploch and H. Murck (2010). "St. John's wort extract LI160 for the treatment of depression with atypical features - A double-blind, randomized, and placebo-controlled trial." Journal of Psychiatric Research **44**(12): 760-767.

Maroufi, M., F. Kianvash and M. R. Alavi (2010). "Comparison of Citrus aurantium and imipramine for treatment of major depressive disorder." European Neuropsychopharmacology **20**: S369.

Matthews, J., S. J. Torres, C. M. Milte, I. Hopkins, S. Kukuljan, C. A. Nowson and R. M. Daly (2020). "Effects of a multicomponent exercise program combined with calcium–vitamin D3-enriched milk on health-related quality of life and depressive symptoms in older men: secondary analysis of a randomized controlled trial." European Journal of Nutrition **59**(3): 1081-1091.

Mayers, A. G., D. S. Baldwin, R. Dyson, R. W. Middleton and A. Mustapha (2003). "Use of St. John's wort (Hypericum perforatum L) in members of a depression self-help organisation: A 12-week open prospective pilot study using the HADS scale." Primary Care Psychiatry **9**(1): 15-20.

Mazaherioun, M., A. Saedisomeolia, M. H. Javanbakht, F. Koohdani, M. Zarei, S. Ansari, F. Khoshkhoo Bazargani and M. Djalali (2018). "Long chain n-3 fatty acids improve depression syndrome in type 2 diabetes mellitus." Iranian Journal of Public Health **47**(4): 575-583.

Mazereeuw, G., N. Herrmann, P. I. Oh, D. W. L. Ma, C. T. Wang, A. Kiss and K. L. Lanctôt (2016). "Omega-3 fatty acids, depressive symptoms, and cognitive performance in patients with coronary artery disease." Journal of Clinical Psychopharmacology **36**(5): 436-444.

Mazloom, Z., M. Ekramzadeh and N. Hejazi (2013). "Efficacy of supplementary vitamins C and E on anxiety, depression and stress in type 2 diabetic patients: A randomized, single-blind, placebo-controlled trial." Pakistan Journal of Biological Sciences **16**(22): 1597-1600.

McKetin, R., O. M. Dean, A. Turner, P. J. Kelly, B. Quinn, D. I. Lubman, P. Dietze, G. Carter, P. Higgs, A. L. Baker and et al. (2019). "A study protocol for the N-ICE trial: a randomised double-blind placebo-controlled study of the safety and efficacy of N-acetyl-cysteine (NAC) as a pharmacotherapy for methamphetamine ("ice") dependence." **20**(1): 325

C323 - PUBMED 31164169.

Mech, A. W. and B. A. Farah (2016). "Reduced B vitamin therapy for MDD in MTHFR C677t/A1298c patients-clinical response correlates with homocysteine reduction: A double-blind, placebo-controlled study." CNS Spectrums **21**(1): 116-117.

Mehdi, S., K. Manohar, A. Shariff, N. Kinattingal, S. U. D. Wani, S. Alshehri, M. T. Imam, F. Shakeel and K. L. Krishna (2023). "Omega-3 Fatty Acids Supplementation in the Treatment of Depression: An Observational Study." Journal of Personalized Medicine **13**(2 C7 - 224).

Mehdi, S. M., S. E. Atlas, S. Qadir, D. Musselman, S. Goldberg, J. M. Woolger, R. Corredor, M. H. Abbas, L. Arosemena, S. Caccamo and et al. (2017). "Double-blind, randomized crossover study of intravenous infusion of magnesium sulfate versus 5% dextrose on depressive symptoms in adults with treatment-resistant depression." **71**(3): 204‐211

C203 - PUBMED 27862658,EMBASE 613942099.

Mehdi, S. M. A., S. E. Atlas, S. Qadir, D. Musselman, S. Goldberg, J. M. Woolger, R. Corredor, M. H. Abbas, L. Arosemena, S. Caccamo, C. S. G. Campbell, A. Farooqi, J. Gao, J. Konefal, L. C. Lages, L. Lantigua, J. Lopez, V. Padilla, A. Rasul, A. M. Ray, H. G. Simões, E. Tiozzo and J. E. Lewis (2017). "Double-blind, randomized crossover study of intravenous infusion of magnesium sulfate versus 5% dextrose on depressive symptoms in adults with treatment-resistant depression." Psychiatry and Clinical Neurosciences **71**(3): 204-211.

Melzer, J., R. Brignoli, M. E. Keck and R. Saller (2010). "A hypericum extract in the treatment of depressive symptoms in outpatients: an open study." Forschende Komplementärmedizin (2006) **17**(1): 7-14.

Merens, W., L. Booij, R. Markus, F. G. Zitman, W. Onkenhout and A. J. Van der Does (2005). "The effects of a diet enriched with alpha-lactalbumin on mood and cortisol response in unmedicated recovered depressed subjects and controls." **94**(3): 415‐422

C413 - PUBMED 16176613.

Mesbahi, S. F., A. M. Sadegh, E. M. Asl, H. Boostani and N. S. Irani (2025). "Effectiveness of Ginseng on the Symptoms of Depression and Anxiety in Patients Referred to Addiction Treatment Clinics in Ahvaz: A Randomized Clinical Trial." Jundishapur Journal of Natural Pharmaceutical Products **20**(1).

Mikola, T., S. M. Lehto, K. Honkalampi, M. Valkonen-Korhonen, H. Koivumaa-Honkanen, T. Tolmunen, V. Laukkanen, M. Pakarinen and A. Ruusunen (2024). "A follow-up study with a double-blinded, randomized controlled vitamin D supplementation trial in patients with major depressive episode (DepFuD): a study protocol and baseline characteristics." **12**(10): 8454‐8469

C8453 - PUBMED 39479665.

Millar, C. L., A. Wolfe, K. Baldyga, A. B. Dufour and L. A. Lipsitz (2025). "Berries and Steps: a protocol of a randomized, placebo-controlled pilot study testing freeze-dried blueberry powder in sedentary older adults with mild depressive symptoms." Nutrition Journal **24**(1 C7 - 87).

Mirzavandi, F., S. Babaie, S. Rahimpour, E. Razmpoosh, N. Talenezhad, S. M. Aghaei Zarch and H. Mozaffari-Khosravi (2020). "The effect of high dose of intramuscular vitamin D supplement injections on depression in patients with type 2 diabetes and vitamin D deficiency: A randomized controlled clinical trial." Obesity Medicine **17**.

Mischoulon, D., C. Best-Popescu, M. Laposata, W. Merens, J. L. Murakami, S. L. Wu, G. I. Papakostas, C. M. Dording, S. B. Sonawalla, A. A. Nierenberg, J. E. Alpert and M. Fava (2008). "A double-blind dose-finding pilot study of docosahexaenoic acid (DHA) for major depressive disorder." European Neuropsychopharmacology **18**(9): 639-645.

Mischoulon, D. and M. Fava (2000). "Docosahexanoic acid and ω-3 fatty acids in depression." Psychiatric Clinics of North America **23**(4): 785-794.

Mischoulon, D., G. I. Papakostas, C. M. Dording, A. H. Farabaugh, S. B. Sonawalla, A. M. Agoston, J. Smith, E. C. Beaumont, L. E. Dahan, J. E. Alpert, A. A. Nierenberg and M. Fava (2009). "A double-blind, randomized controlled trial of ethyl-eicosapentaenoate for major depressive disorder." Journal of Clinical Psychiatry **70**(12): 1636-1644.

Missaoui, M., A. Hassine, S. Naija, J. Emna and S. Ben Amor (2021). "Effect of vitamin D supplementation on anxiety-depressive disorder at six months of stroke: A randomized clinical trial." European Journal of Neurology **28**(SUPPL 1): 864.

Mitchell, M., H. Bradley, N. M. Blampied, R. T. Mulder and J. J. Rucklidge (2025). "Protective effect of micronutrients used to treat antenatal depression on rates of postnatal depression at six months: A secondary analysis of NUTRIMUM." Journal of Affective Disorders **388 C7 - 119560**.

Moazen-Zadeh, E., S. H. Abbasi, H. Safi-Aghdam, N. Shahmansouri, A. Arjmandi-Beglar, A. Hajhosseinn Talasaz, A. Salehiomran, S. Forghani and S. Akhondzadeh (2018). "Effects of Saffron on Cognition, Anxiety, and Depression in Patients Undergoing Coronary Artery Bypass Grafting: A Randomized Double-Blind Placebo-Controlled Trial." Journal of Alternative and Complementary Medicine **24**(4): 361-368.

Mocking, R. J. T., J. Assies, M. Bot, E. H. J. M. Jansen, A. H. Schene and F. Pouwer (2012). "Biological Effects of Add-On Eicosapentaenoic Acid Supplementation in Diabetes Mellitus and Co-Morbid Depression: A Randomized Controlled Trial." PLoS ONE **7**(11 C7 - e49431).

Mohajeri, S., M. Hassanpour Moghadam, A. Talaei and S. Sajadi Tabassi (2014). "Effect of crocin on major depressive disorder: a double blind clinical trial." **115**: 187

C183 - EMBASE 71549613.

Mohamed, S.-A., G. M. Khafagy, I. T. A. Alsaid and H. A. Hussein (2024). "The gut brain axis, effect of dietary changes and probiotics supplement on depression symptoms." **30**.

Mohsenpour, M. A., F. Mohammadi, Z. Sohrabi, M. Niakousari, M. Jeddi and M. H. Eftekhari (2025). "Mental Health and Health-related Quality of Life Response to Curcumin-Probiotic Drink Powder Co-supplementation in Adults with Overweight/Obesity and Metabolic Syndrome: a Four-arm Randomized, Double-blind, Placebo-controlled, Clinical Trial." British Journal of Nutrition.

Mokhber, N., M. Namjoo, F. Tara, H. Boskabadi, M. P. Rayman, M. Ghayour-Mobarhan, A. Sahebkar, M. R. Majdi, S. Tavallaie, M. Azimi-Nezhad and et al. (2011). "Effect of supplementation with selenium on postpartum depression: a randomized double-blind placebo-controlled trial." **24**(1): 104‐108

C103 - PUBMED 20528216.

Moludi, J., M. Alizadeh, M. H. S. Mohammadzad and M. Davari (2019). "The Effect of Probiotic Supplementation on Depressive Symptoms and Quality of Life in Patients after Myocardial Infarction: Results of a Preliminary Double-Blind Clinical Trial." Psychosomatic Medicine **81**(9): 770-777.

Moludi, J., H. Khedmatgozar, S. M. Nachvak, H. Abdollahzad, M. Moradinazar and A. Sadeghpour tabaei (2022). "The effects of co-administration of probiotics and prebiotics on chronic inflammation, and depression symptoms in patients with coronary artery diseases: a randomized clinical trial." Nutritional Neuroscience **25**(8): 1659-1668.

Moniello, G., I. Bonfitto, F. Simonetti, S. Dimalta, M. R. Rizzo and A. Bellomo (2024). "Role of L-Arginine supplementation in Long Covidrelated Fatigue and Depression in Elderly Outpatients." European Psychiatry **67**: S106.

Moore, K., C. F. Hughes, L. Hoey, M. Ward, C. Cunningham, A. M. Molloy, J. J. Strain, K. McCarroll, M. C. Casey, F. Tracey, E. Laird, M. O'Kane and H. McNulty (2019). "B-vitamins in Relation to Depression in Older Adults Over 60 Years of Age: The Trinity Ulster Department of Agriculture (TUDA) Cohort Study." Journal of the American Medical Directors Association **20**(5): 551-557.e551.

Moosavi, S. M., M. Ahmadi, M. Amini and B. Vazirzadeh (2014). "The effects of 40 and 80 mg hydro- alcoholic extract of crocus sativus in the treatment of mild to moderate depression." Journal of Mazandaran University of Medical Sciences **24**(113): 47-53.

Moschonis, G., K. Sarapis, S. Resciniti, R. Hall, K. Yim, M. Tonkovic, C. Fitzgerald, F. Anixiadis, Q. Nhu Dinh, M. Hale, B. Wright, M. Pane, C. J. Tuck and J. R. Biesiekierski (2024). "The effectiveness of a Lactobacillus probiotic on measures of psychosocial health in adults diagnosed with subthreshold depression: a double-blind, randomised, placebo-controlled trial." Proceedings of the Nutrition Society **83**(OCE1): E58.

Moschonis, G., K. Sarapis, S. Resciniti, R. Hall, K. Yim, M. Tonkovic, C. Fitzgerald, F. Anixiadis, A. Vinh, Q. N. Dinh, R. A. Cronin, M. W. Hale, B. J. Wright, M. Pane, C. J. Tuck and J. R. Biesiekierski (2024). "Evaluation of a probiotic blend on psychosocial health and biomarkers of inflammatory, immune and stress response in adults with subthreshold depression: A double-blind, randomised, placebo-controlled trial." British Journal of Nutrition.

Mousa, A., N. Naderpoor, M. P. J. de Courten and B. de Courten (2018). "Vitamin D and symptoms of depression in overweight or obese adults: A cross-sectional study and randomized placebo-controlled trial." Journal of Steroid Biochemistry and Molecular Biology **177**: 200-208.

Mozaffari-Khosravi, H., L. Nabizade, S. M. Yassini-Ardakani, H. Hadinedoushan and K. Barzegar (2013). "The effect of 2 different single injections of high dose of vitamin D on improving the depression in depressed patients with vitamin D deficiency: A randomized clinical trial." Journal of Clinical Psychopharmacology **33**(3): 378-385.

Mueller, B. M. (1998). "St. John's wort for depressive disorders: Results of an outpatient study with the hypericum preparation HYP 811." Advances in Therapy **15**(2): 109-116.

Mueller, M., R. Ganesh and S. Bonnes (2020). "Gut Health = Mental Health? The Impact of Diet and Dietary Supplements on Mood Disorders." Current Nutrition Reports **9**(4): 361-368.

Nabimeybodi, R., A. M. Ranjbar, A. Javaheri, B. Heydari, F. Hosseini, S. Kardan and V. Ramezani (2024). "Assessment of the Effect of Grape Molasses on Preventing Postpartum Depression in Women Referred to Shahid Sadoughi Hospital in Yazd." Traditional and Integrative Medicine **9**: 4.

Nagayasu, Y., D. Fujita, A. Daimon, M. Nunode, M. Sawada, T. Sano and M. Ohmichi (2021). "Possible prevention of post-partum depression by intake of omega-3 polyunsaturated fatty acids and its relationship with interleukin 6." Journal of Obstetrics and Gynaecology Research **47**(4): 1371-1379.

Najafabady, M. T., P. Y. Baghbadoranee, E. Moghimipour, M. H. Haghighizadeh and H. Boostani (2019). "Comparison the effect of Echium amoenum extract with fluoxetine on depression in menopausal women. A double-blind randomized controlled trial." Asian Journal of Pharmaceutics **13**(3): 271-275.

NCT00005013 (2000). A Placebo Controlled Clinical Trial of a Standardized Extract of Hypericum Perforatum in Major Depressive Disorder. clinicaltrials.gov.

NCT00042380 (2002). A Phase 2A Study of Soy Isoflavones in the Treatment of Major Depression. clinicaltrials.gov.

NCT00066859 (2003). A Phase III Double-Blind Randomized Trial Comparing Sertraline (Zoloft) And Hypericum Perforatum (St. John's Wort) In Cancer Patients With Mild To Moderate Depression. clinicaltrials.gov.

NCT00067301 (2003). PUFA Augmentation in Treatment of Major Depression. clinicaltrials.gov.

NCT00122954 (2005). Fish Oil as an Adjunct Therapy for Depression in Multiple Sclerosis. clinicaltrials.gov.

NCT00223236 (2005). A Double-blind, Placebo-controlled Trial of Citicoline add-on Therapy in Patients With a History of Mania or Hypomania and Cocaine Abuse/Dependence. clinicaltrials.gov.

NCT00226356 (2005). Phase 4 Study: An Open Study of the Efficacy and Tolerability in Unipolar Depression of Augmentation of the One-carbon Cycle With L-methionine, Betaine and Folate. clinicaltrials.gov.

NCT00256412 (2005). Essential Fatty Acids in Management of MDD - A Pilot Study. clinicaltrials.gov.

NCT00480207 (2007). Randomized Double-blind Controlled Clinical Trial to Assess the Impact of Folic Acid and Omega-3 Fatty Acids Supplementation on the Severity of Depressive Symptoms in Older Adults With Identified Depression. clinicaltrials.gov.

NCT00539513 (2007). A Double-Blind Study of N-Acetylcysteine Augmentation in Serotonin Reuptake Inhibitor-Refractory Obsessive-Compulsive Disorder and Depression. clinicaltrials.gov.

NCT00693680 (2008). The Role of Zinc in Therapy of Unipolar Depression. clinicaltrials.gov.

NCT00711971 (2008). Does EPA or DHA Prevent Depressive Symptoms in Pregnancy and Postpartum? clinicaltrials.gov.

NCT00729755 (2008). Efficacy and Safety of Augmentation of Creatine for the Patients With Major Depressive Disorder. clinicaltrials.gov.

NCT00816322 (2008). The Effect of Fish Oil in Major Depressive Disorder: a Double-blind Placebo-controlled Monotherapy Trail and a RCT for Recurrence Prevention. clinicaltrials.gov.

NCT00861978 (2009). St. John's Wort Extract LI 160 for the Treatment of Atypical Depression. clinicaltrials.gov.

NCT00955955 (2009). TRD - 2: A Double-Blind, Placebo Controlled Study of 6(S)-5-MTHF Among SSRI-Resistant Outpatients With Major Depressive Disorder (MDD). clinicaltrials.gov.

NCT00997490 (2009). Phase III Placebo-controlled, Randomised, Single-centre, Double-blind Study of the Antidepressant Efficacy of a Combination Herbal Drug Preparation. clinicaltrials.gov.

NCT01022632 (2009). A Randomized, Active Controlled, Open Label, Parallel Group Study to Compare the Efficacy of Extract of Curcuma Longa (Turmeric) With Fluoxetine and to Study Its Effect as an Add on Therapy to Fluoxetine in Patients of Depression. clinicaltrials.gov.

NCT01175616 (2010). Creatine Augmentation in Female & Male Veterans With Selective Serotonin Reuptake Inhibitor-Resistant Major Depressive Disorder. clinicaltrials.gov.

NCT01185574 (2010). Vitamin D for Improving Metabolic Control and Depressive Symptoms in Women With Diabetes; The Sunshine Study. clinicaltrials.gov.

NCT01223729 (2010). Acetyl-L-Carnitine Reduces Depression and Improves Quality of Life in Patients With Minimal Hepatic Encephalopathy. clinicaltrials.gov.

NCT01390662 (2011). Phase 4 Study of Vitamin D3 Supplementation for Outcomes in Patients With Unipolar Depression. clinicaltrials.gov.

NCT01416818 (2011). A Randomized, Double-Blind, Placebo-Controlled Study of "Xiaoyao Pill" Treatment of Depression in Patients With Parkinson's Disease. clinicaltrials.gov.

NCT01462058 (2011). The Role of Vitamin D Supplementation on Well Being and Symptoms of Depression During the Winter Season in Health Service Staff. clinicaltrials.gov.

NCT01750359 (2012). The Efficacy and Safety of Adjunctive Curcumin for Treatment of Depression: A Randomized, Double-blind, Placebo-controlled Study. clinicaltrials.gov.

NCT02189915 (2014). Open-Label Creatine Monohydrate Supplementation in Depressed Female Methamphetamine Users. clinicaltrials.gov.

NCT02223299 (2014). A Randomized Double-Blind Placebo-Controlled Pilot Study of L-tyrosine and L-tryptophan in Depressed Patients Receiving L-methylfolate and and SSRI. clinicaltrials.gov.

NCT02359266 (2015). Clinical Trial Investigating the Role of Vitamin D in the Treatment of Depression in Patients With Chronic Liver Disease. clinicaltrials.gov.

NCT02381600 (2015). An Evaluation of the Effects of Vitamin D Supplementation on Specific Cognitive Functions and Depressive Symptoms of Older Subjects With Low Levels of This Vitamin. clinicaltrials.gov.

NCT02568878 (2015). An Open-Label Pilot Study of Creatine for Depressed Male and Female Methamphetamine Users. clinicaltrials.gov.

NCT02793648 (2015). Phase II, Double-blind, Randomized, 1-way Cross-over, to Investigate the Effectiveness of the Combination of Ascorbic Acid (Vitamin C) and Tocopherol (Vitamin E) Versus Placebo for the Treatment of Depressive Disorders in Elderly. clinicaltrials.gov.

Nct (2002). "Examination of a Soy-Based Supplement for Major Depression."

Nct (2007). "Folate Augmentation of Treatment - Evaluation for Depression: a Randomised Controlled Trial."

Nct (2007). "Omega-3 Fatty Acids as Adjunct Treatment for Major Depressive Disorder."

Nct (2007). "Omega-3 Fatty Acids for Treating Adults With Major Depression."

Nct (2007). "St. John's Wort And Kava In The Treatment Of Major Depressive Disorder With Comorbid Anxiety."

Nct (2009). "Comparison of Vitamin B12 Supplementation to Selective Serotonin Reuptake Inhibitor (SSRI) Versus SSRI Antidepressant Treatment Alone."

Nct (2010). "Rhodiola Rosea Therapy of Major Depressive Disorder."

Nct (2010). "Vitamin B6, B12, Folic Acid and Exercise in Parkinson's Disease."

Nct (2012). "N-3 Polyunsaturated Fatty Acids in the Prevention and Treatment for IFN-induced Depression."

Nct (2015). "Effect of Lactobacillus Plantarum 299v Supplementation on Major Depression Treatment."

Nct (2016). "Selenium as Augmentation Treatment for Sertraline Resistant Major Depression."

Nct (2018). "Role of Omega-3 Polyunsaturated Fatty Acid in the Management of Major Depressive Disorder."

Nct (2019). "Effects of Nutrients Supplementation in Antidepressant Treated Depressive Disorder Patients."

Nct (2021). "Cholecalciferol on Depressive Symptoms in Type 2 Diabetes Mellitus Patients."

Nct (2021). "RCT of Adjunctive Curcumin and the Meru Health Program."

Nct (2023). "Supplementation and Depressive Symptomatology Incidence Among Saudi Arabian Adults."

Nct (2024). "Comparison of Krill and Fish Oil on Clinical and Biochemical Outcomes in Depression."

Nemets, B. (2006). "A Double-Blind, Parallel, Randomized, Add-on Clinical Trial of Creatine Versus Placebo Added to Antidepressant Treatment of Patients With Major Depressive Episode."

Nemets, B., Z. Stahl and R. H. Belmaker (2002). "Addition of omega-3 fatty acid to maintenance medication treatment for recurrent unipolar depressive disorder." American Journal of Psychiatry **159**(3): 477-479.

Nikfarjam, M., I. Goudarzi, S. Heidari, L. R. Vardanjani and N. Parvin (2012). "Effect of Ginkgo biloba pill on patients with major depression treated with electroconvulsive therapy." Journal of Mazandaran University of Medical Sciences **22**(88): 64-72.

Nikfarjam, M., R. Rakhshan and H. Ghaderi (2017). "Comparison of effect of Lavandula officinalis and venlafaxine in treating depression: A double blind clinical trial." Journal of Clinical and Diagnostic Research **11**(7): KC01-KC04.

Nikolova, V., A. Cleare, A. Young and J. Stone (2023). "Probiotics as Adjunctive Treatment in Major Depressive Disorder: Estimates of Treatment Effect and Underlying Mechanisms From a Double-Blind Placebo-Controlled Randomised Pilot Trial." BJPsych Open **9**: S64-S65.

Nikolova, V. L., A. J. Cleare, A. H. Young and J. M. Stone (2023). "Acceptability, Tolerability, and Estimates of Putative Treatment Effects of Probiotics as Adjunctive Treatment in Patients with Depression: A Randomized Clinical Trial." JAMA Psychiatry **80**(8): 842-847.

Nikolova, V. L., A. J. Cleare, A. H. Young and J. M. Stone (2025). "Exploring the mechanisms of action of probiotics in depression: Results from a randomized controlled pilot trial." Journal of Affective Disorders **376**: 241-250.

Ning, H., H. Zhou, N. Yang, J. Ren, H. Wang, W. Liu and Y. Zhao (2023). "Effect of Zishen pingchan granules combined with pramipexole on serum BDNF, IL-1β, IL-6, CRP, TNF-α levels in depressed patients with Parkinson's disease: Results of a randomized, double-blind, controlled study." Experimental Gerontology **182**.

Nishi, D., Y. Matsuoka and K. Su (2015). Are omega-3 PUFAs effective for depression during pregnancy in countries where fish consumptions are high? PSYCHOTHERAPY AND PSYCHOSOMATICS, KARGER ALLSCHWILERSTRASSE 10, CH-4009 BASEL, SWITZERLAND.

Nishi, D., K. P. Su, K. Usuda, J. P. C. Chang, K. Hamazaki, T. Ishima, K. Hashimoto and Y. J. Matsuoka (2019). "The effect of omega-3 fatty acids for depressive symptoms among pregnant women in Japan and Taiwan: A randomized, double-blind, placebo-controlled trial and biomarker study." Psychotherapy and Psychosomatics **88**: 96-97.

Nishi, D., K. P. Su, K. Usuda, J. P. C. Chang, K. Hamazaki, T. Ishima, Y. Sano, H. Ito, K. Isaka, Y. Tachibana, S. Tanigaki, T. Suzuki, K. Hashimoto and Y. J. Matsuoka (2020). "Plasma estradiol levels and antidepressant effects of omega-3 fatty acids in pregnant women." Brain, Behavior, and Immunity **85**: 29-34.

Noori, M., Z. Shateri, S. Babajafari, M. H. Eskandari, K. Parastouei, M. Ghasemi, H. Afshari and M. Samadi (2025). "The effect of probiotic-fortified kefir on depression, appetite, oxidative stress, and inflammatory parameters in Iranian overweight and obese elderly: a randomized, double-blind, placebo-controlled clinical trial." Journal of health, population, and nutrition **44**(1): 30.

Novak, F., M. Vecka, E. Meisnerova, S. Sevela, L. Vavrova, J. Rychlikova, L. Dolezalova, D. Myslivcova, A. Zak, L. Vitek and et al. (2019). "Fish oil supplementation with various lipid emulsions suppresses in vitro cytokine release in home parenteral nutrition patients: a crossover study." **72**: 70‐79

C73 - PUBMED 31759770,EMBASE 2003873276.

Novak, F., M. Vecka, E. Meisnerova, S. Sevela, L. Vavrova, J. Rychlikova, L. Dolezalova, D. Myslivcova, A. Zak, L. Vitek and O. Novakova (2019). "Fish oil supplementation with various lipid emulsions suppresses in vitro cytokine release in home parenteral nutrition patients: a crossover study." Nutrition Research **72**: 70-79.

Odebrecht Vargas Nunes, S., M. Porcu, H. Odebrecht Vargas and M. Urbano (2017). "N-acetylcysteine as an adjunctive treatment to reduce depressive symptom severity when C-reactive protein levels are increased." European Neuropsychopharmacology **27**: S788.

Okronipa, H., S. Adu-Afarwuah, A. Lartey, P. Ashorn, S. A. Vosti, R. R. Young and K. G. Dewey (2018). "Maternal supplementation with small-quantity lipid-based nutrient supplements during pregnancy and lactation does not reduce depressive symptoms at 6 months postpartum in Ghanaian women: a randomized controlled trial." Archives of Women's Mental Health **21**(1): 55-63.

Olagunju, A. E. and H. Gaddey (2020). "Leveraging CAM to treat depression and anxiety." Journal of Family Practice **69**(5): 221-227.

Omidian, M., M. Mahmoudi, M. Abshirini, M. R. Eshraghian, M. H. Javanbakht, M. Zarei, H. Hasani and M. Djalali (2019). "Effects of vitamin D supplementation on depressive symptoms in type 2 diabetes mellitus patients: Randomized placebo-controlled double-blind clinical trial." Diabetes Metab Syndr **13**(4): 2375-2380.

Opiyo, R. O., P. S. Nyasulu, R. K. Koigi, A. Obondo, D. Ogoyi and W. Kogi-Makau (2018). "Effect of fish oil omega-3 fatty acids on reduction of depressive symptoms among HIV-seropositive pregnant women: a randomized, double-blind controlled trial." Annals of General Psychiatry **17**(1 C7 - 49).

Osali, A. and A. Rostami (2023). "Effect of 6 weeks of aerobic training with nanocurcumin consumption on IL1β, nitric oxide, and depression in women with metabolic syndrome." International Journal of Diabetes in Developing Countries **43**(6): 1007-1014.

Pakseresht, S., H. Boustani, M. E. Azemi, J. Nilsaz, R. Babapour and M. R. Haghdust (2012). "Evaluation of pharmaceutical products of st. john's wort efficacy added on tricyclic antidepressants in treating major depressive disorder: A double blind randomized control trial." Jundishapur Journal of Natural Pharmaceutical Products **7**(3): 106-110.

Parletta, N., D. Zarnowiecki, J. Cho, A. Wilson, S. Bogomolova, A. Villani, C. Itsiopoulos, B. Meyer, L. Segal and K. O'Dea (2018). "Effects of a Mediterranean-style diet supplemented with fish oil on mental health and quality of life in people with depression." Nutritional Neuroscience **21**: S41.

Parvin, N., S. Farzaneh, M. Nikfarjam, N. Shahinfard and N. Asarzadegan (2011). "The effect of lavandula angustifolia in the treatment of depression." European Psychiatry **26**.

Peet, M. and D. F. Horrobin (2002). "A dose-ranging study of the effects of ethyl-eicosapentaenoate in patients with ongoing depression despite apparently adequate treatment with standard drugs." Archives of General Psychiatry **59**(10): 913-919.

Penckofer, S., M. Ridosh, W. Adams, M. Grzesiak, J. Woo, M. Byrn, J. Kouba, P. Sheean, C. Kordish, R. Durazo-Arvizu and et al. (2022). "Vitamin D Supplementation for the Treatment of Depressive Symptoms in Women with Type 2 Diabetes: a Randomized Clinical Trial." **2022**: 4090807

C4090803 - PUBMED 35280228,EMBASE 2017280621.

Pinto-Sanchez, M. I., G. B. Hall, K. Ghajar, A. Nardelli, C. Bolino, J. T. Lau, F. P. Martin, O. Cominetti, C. Welsh, A. Rieder and et al. (2017). "Probiotic Bifidobacterium longum NCC3001 Reduces Depression Scores and Alters Brain Activity: a Pilot Study in Patients With Irritable Bowel Syndrome." **153**(2): 448‐459.e448

C443 - PUBMED 28483500,EMBASE 617425533.

Putranto, R., S. Setiati, M. W. Nasrun, F. Witjaksono, S. Immanuel, I. Subekti, K. Harimurti, A. Siswanto, H. Shatri, S. Suwarto and et al. (2024). "Effects of cholecalciferol supplementation on depressive symptoms, C-peptide, serotonin, and neurotrophin-3 in type 2 diabetes mellitus: a double-blind, randomized, placebo-controlled trial." **4**(3): e1342

C1343 - PUBMED 39816051.

Raeder, M. B., V. M. Steen, S. E. Vollset and I. Bjelland (2007). "Associations between cod liver oil use and symptoms of depression: The Hordaland Health Study." Journal of Affective Disorders **101**(1-3): 245-249.

Rahman, S. T., M. Waterhouse, B. D. Romero, C. Baxter, D. R. English, O. P. Almeida, M. Berk, P. R. Ebeling, B. K. Armstrong, D. S. A. McLeod, G. Hartel, R. L. O'Connell, H. Pham, J. G. Scott, J. C. van der Pols, A. J. Venn, P. M. Webb, D. C. Whiteman and R. E. Neale (2023). "Effect of vitamin D supplementation on depression in older Australian adults." Int J Geriatr Psychiatry **38**(1): e5847.

Rahman, S. T., M. Waterhouse, B. D. Romero, C. Baxter, D. R. English, O. P. Almeida, M. Berk, P. R. Ebeling, B. K. Armstrong, D. S. A. McLeod, G. Hartel, R. L. O’Connell, H. Pham, J. G. Scott, J. C. van der Pols, A. J. Venn, P. M. Webb, D. C. Whiteman and R. E. Neale (2023). "Effect of vitamin D supplementation on depression in older Australian adults." International Journal of Geriatric Psychiatry **38**(1 C7 - e5847).

Rajizadeh, A., H. Mozaffari-Khosravi, M. Yassini-Ardakani and A. Dehghani (2017). "Effect of magnesium supplementation on depression status in depressed patients with magnesium deficiency: A randomized, double-blind, placebo-controlled trial." Nutrition **35**: 56-60.

Ranjbar, M., A. Firoozabadi, A. Salehi, Z. Ghorbanifar, M. M. Zarshenas, K. Sadeghniiat-Haghighi and H. Rezaeizadeh (2018). "Effects of Herbal combination (Melissa officinalis L. and Nepeta menthoides Boiss. & Buhse) on insomnia severity, anxiety and depression in insomniacs: Randomized placebo controlled trial." Integrative Medicine Research **7**(4): 328-332.

Rao, G. P., S. Kariya, P. Chaudhari and M. Bhondave (2024). "A STUDY TO ASSESS EFFICACY AND SAFETY OF ADD-ON ACETYL-L-CARNITINE AND AGMATINE (REJIYANA®) WITH STANDARD OF CARE (SOC) TO VALIDATE VISIBLE CHANGES IN MAJOR DEPRESSIVE DISORDER A RANDOMIZED CONTROLLED TRIAL: REVIVE TRIAL." International Journal of Medicine and Public Health **14**(2): 597-604.

Rashmi, K. and P. Madhuri (2017). "Evaluation of therapeutic effect of Vachadi ghrita in patients of mild to moderate depression." International Journal of Research in Ayurveda and Pharmacy **8**: 137-140.

Rastegar, M., N. J. Shoorab, R. Salari and A. Saki (2024). "The Effect of Oral Probiotic Capsules on Postpartum Depression in Primiparous Women: A Triple-blind Randomized Control trial." Iranian Journal of Obstetrics, Gynecology and Infertility **27**(3): 64-75.

Ravi, S., H. Khalili, L. Abbasian, M. Arbabi and P. Ghaeli (2016). "Effect of Omega-3 Fatty Acids on Depressive Symptoms in HIV-Positive Individuals: A Randomized, Placebo-Controlled Clinical Trial." Annals of Pharmacotherapy **50**(10): 797-807.

Raza, M. L., S. T. Hassan, S. Jamil, W. Fatima and M. Fatima (2025). "Nutritional interventions in depression: The role of vitamin D and omega-3 fatty acids in neuropsychiatric health." Clinical Nutrition **45**: 270-280.

Rees, A. M., M. P. Austin and G. B. Parker (2008). "Omega-3 fatty acids as a treatment for perinatal depression: Randomized double-blind placebo-controlled trial." Australian and New Zealand Journal of Psychiatry **42**(3): 199-205.

Rees, A. M., G. Parker and M. P. Austin (2006). "A randomized controlled trial to assess the efficacy of omega-3 fatty acids as a treatment for depression during the perinatal period." Acta Neuropsychiatr **18**(6): 267-268.

Regueme, S. C., R. Ostan, A. Pinto, L. Valentini, J. Durrieu, C. Blanc-Bisson, M. L. Donini, H. Lochs, C. Franceschi and I. Bourdel-Marchasson (2016). "Effects of a 2-month dietand nutraceutical intervention on quality of life and depression with respect to inflammaging: Ristomed, an open label intervention trial." Clinical Nutrition **35**: S84.

Reininghaus, E., K. Riedrich, S. Bengesser, S. Mörkl, C. Hamm, R. Queissner, D. Otti, H. Schöggl, T. Lahousen, W. Wurm, N. Dalkner, F. Fellendorf, M. Platzer, T. Bastiaanssen and B. Reininghaus (2020). "Intake of probiotic supplements for the treatment of depression." Bipolar Disorders **22**: 101.

Reininghaus, E. Z., M. Platzer, A. Kohlhammer-Dohr, C. Hamm, S. Mörkl, S. A. Bengesser, F. T. Fellendorf, T. Lahousen-Luxenberger, B. Leitner-Afschar, H. Schöggl, D. Amberger-Otti, W. Wurm, R. Queissner, A. Birner, V. S. Falzberger, A. Painold, W. Fitz, J. Wagner-Skacel, M. Brunnmayr, A. Rieger, A. Maget, R. Unterweger, K. Schwalsberger, B. Reininghaus, M. Lenger, T. F. S. Bastiaanssen and N. Dalkner (2020). "PROVIT: Supplementary Probiotic Treatment and Vitamin B7 in Depression-A Randomized Controlled Trial." Nutrients **12**(11).

Rocco, A. D., J. D. Rogers, R. Brown, P. Werner and T. Bottiglieri (2000). "S-adenosyl-methionine improves depression in patients with Parkinson's disease in an open-label clinical trial." Movement Disorders **15**(6): 1225-1229.

Rodríguez-Morán, M., F. Guerrero-Romero, J. Barragán-Zuñiga, C. I. Gamboa-Gómez, Y. Weyman-Vela, M. Arce-Quiñones, L. E. Simental-Mendía and G. Martínez-Aguilar (2024). "Combined oral supplementation with magnesium plus vitamin D alleviates mild to moderate depressive symptoms related to long-COVID: an open-label randomized, controlled clinical trial." Magnesium Research **37**(3): 49-57.

Rolf, L., A. H. Muris, Y. Bol, J. Damoiseaux, J. Smolders and R. Hupperts (2016). "High dose vitamin D3 supplementation does not ameliorate depressive symptoms in relapsing remitting multiple sclerosis: Results of a randomized placebo-controlled trial." Multiple Sclerosis **22**: 379.

Romijn, A. R., J. J. Rucklidge, R. G. Kuijer and C. Frampton (2017). "A double-blind, randomized, placebo-controlled trial of Lactobacillus helveticus and Bifidobacterium longum for the symptoms of depression." Australian and New Zealand Journal of Psychiatry **51**(8): 810-821.

Rondanelli, M., A. Giacosa, A. Opizzi, C. Pelucchi, C. La Vecchia, G. Montorfano, M. Negroni, B. Berra, P. Politi and A. M. Rizzo (2011). "Long chain omega 3 polyunsaturated fatty acids supplementation in the treatment of elderly depression: Effects on depressive symptoms, on phospholipids fatty acids profile and on health-related quality of life." Journal of Nutrition, Health and Aging **15**(1): 37-44.

Rosenbaum, J. F., M. Fava, W. E. Falk, M. H. Pollack, L. S. Cohen, B. M. Cohen and G. S. Zubenko (1988). "An open-label pilot study of oral S-adenosylmethionine in major depression. An interim report." The Alabama journal of medical sciences **25**(3): 301-306.

Ross, S. M. (2014). "Rhodiola rosea (SHR-5), Part 2: A standardized extract of Rhodiola rosea is shown to be effective in the treatment of mild to moderate depression." Holistic nursing practice **28**(3): 217-221.

Rucklidge, J. J. and A. J. Richardson (2019). "Multinutrient Supplementation for Prevention of Major Depressive Disorder in Overweight Adults." JAMA - Journal of the American Medical Association **322**(4): 366.

Russell, S. E., D. R. Skvarc, M. Mohebbi, D. Camfield, L. K. Byrne, A. Turner, M. M. Ashton, M. Berk, S. Dodd, G. S. Malhi, S. M. Cotton, A. I. Bush and O. M. Dean (2023). "The Impact of N-acetylcysteine on Major Depression: Qualitative Observation and Mixed Methods Analysis of Participant Change during a 12-week Randomised Controlled Trial." Clinical Psychopharmacology and Neuroscience **21**(2): 320-331.

Rychlik, R., H. Siedentop, V. von den Driesch and S. Kasper (2001). "St. John's wort extract WS 5572 in minor to moderately severe depression. Effectiveness and tolerance of 600 and 1200 mg active ingredient daily." Fortschritte der Medizin. Originalien **119**(3-4): 119-128.

Safa, M., S. Fallah Tafti, F. Ghassem Boroujerdi and F. Talischi (2013). "Clinical trial in the treatment of 80 Iranian patients with major depression disorder by the combination of omega 3 fatty acid and a selective serotonin reuptake inhibitor." Therapeutic Advances in Psychopharmacology **3**(4): 186-190.

Safari, M., A. Asadi, N. Aryaeian, H. F. Huseini, F. shidfar, S. Jazayeri, M. Malek, A. F. Hosseini and Z. hamidi (2023). "The effects of melissa officinalis on depression and anxiety in type 2 diabetes patients with depression: a randomized double-blinded placebo-controlled clinical trial." BMC Complementary Medicine and Therapies **23**(1 C7 - 140).

Salehi, L., M. Farajnejad and S. Esmailzadeh Saieh (2024). "The effect of aromatherapy with Polianthes’s tuberosa on postpartum depression in high-risk pregnancies: A double blind randomized placebo-controlled clinical trial." The Iranian Journal of Obstetrics, Gynecology and Infertility **27**(4): 18-27.

Sánchez-Villegas, A., J. Álvarez-Pérez, E. Toledo, J. Salas-Salvadó, C. Ortega-Azorín, M. D. Zomeño, J. Vioque, J. A. Martínez, D. Romaguera, J. Pérez-López, J. López-Miranda, R. Estruch, A. Bueno-Cavanillas, F. Arós, J. A. Tur, F. J. Tinahones, O. Lecea, V. Martín, M. Ortega-Calvo, C. Vázquez, X. Pintó, J. Vidal, L. Daimiel, M. Delgado-Rodríguez, P. Matía, D. Corella, A. Díaz-López, N. Babio, M. Á. Muñoz, M. Fitó, M. G. de la Hera, I. Abete, A. García-Rios, E. Ros, M. Ruíz-Canela, M. Á. Martínez-González, M. Izquierdo and L. Serra-Majem (2018). "Seafood consumption, omega-3 fatty acids intake, and life-time prevalence of depression in the PREDIMED-plus trial." Nutrients **10**(12 C7 - 2000).

Sánchez-Villegas, A., M. A. Martínez-González, R. Estruch, J. Salas-Salvadó, D. Corella, M. I. Covas, F. Arós, D. Romaguera, E. Gómez-Gracia, J. Lapetra and et al. (2013). "Mediterranean dietary pattern and depression: the PREDIMED randomized trial." **11**: 208

C203 - PUBMED 24229349,EMBASE 52789647.

Sanders, A. E., E. D. Weatherspoon, B. M. Ehrmann, P. S. Soma, S. R. Shaikh, J. S. Preisser, R. Ohrbach, R. B. Fillingim and G. D. Slade (2022). "Ratio of Omega-6/Omega-3 Polyunsaturated Fatty Acids Associated With Somatic and Depressive Symptoms in People With Painful Temporomandibular Disorder and Irritable Bowel Syndrome." Journal of Pain **23**(10): 1737-1748.

Sarkawi, M., R. A. Raja Ali, N. Abdul Wahab, N. D. Abdul Rathi and N. M. Mokhtar (2024). "A randomized, double-blinded, placebo-controlled clinical trial on Lactobacillus-containing cultured milk drink as adjuvant therapy for depression in irritable bowel syndrome." Scientific reports **14**(1): 9478.

Sarris, J. (2010). "Positive effects of Piper methysticum (kava) in patients with anxiety and depression." Focus on Alternative and Complementary Therapies **15**(1): 27-28.

Sarris, J., D. J. Kavanagh, J. Adams, K. Bone and G. Byrne (2009). "Kava Anxiety Depression Spectrum Study (KADSS): A mixed methods RCT using an aqueous extract of Piper methysticum." Complementary Therapies in Medicine **17**(3): 176-178.

Sarris, J., D. J. Kavanagh, G. Byrne, K. M. Bone, J. Adams and G. Deed (2009). "The Kava Anxiety Depression Spectrum Study (KADSS): A randomized, placebo-controlled crossover trial using an aqueous extract of Piper methysticum." Psychopharmacology **205**(3): 399-407.

Savović, J. (2005). "Neither Hypericum perforatum nor fluoxetine are more effective than placebo in short-term treatment of mild to moderate depression." Focus on Alternative and Complementary Therapies **10**(4): 286-287.

Schifano, F. and F. Garofoli (1993). "Dothiepin vs. S-adenosylmethionine (SAMe) for the treatment of major depression in weaned alcoholics: A pilot study." European Neuropsychopharmacology **3**(3): 330.

Schmidt, U., J. Maisenbacher, G. Harrer and U. Kuhn (1995). "Psychomotoric performance improvement: Antidepressant therapy with St John's wort." Therapiewoche **45**(2): 106+108+110+112.

Schmidt, U. and H. Sommer (1993). "St. John's wort extract in the ambulatory therapy of depression. Attention and reaction ability are preserved." Fortschritte der Medizin **111**(19): 339-342.

Schneider, E., J. P. K. Doll, N. Schweinfurth, C. Kettelhack, A. C. Schaub, G. Yamanbaeva, N. Varghese, L. Mählmann, S. Brand, A. Eckert and et al. (2023). "Effect of short-term, high-dose probiotic supplementation on cognition, related brain functions and BDNF in patients with depression: a secondary analysis of a randomized controlled trial." **48**(1): E23‐E33

C23 - PUBMED 36653035.

Schulz, V. (2002). "Clinical study with hypericum extracts in depressive patients. Results, comparison, conclusions, for anti-depressive pharmacotherapy." Zeitschrift fur Phytotherapie **23**(1): 11-15.

Schulz, V. (2005). "St John's wort in the treatment of moderate to severe depression." Zeitschrift fur Phytotherapie **26**(3): 127-128.

Schulz, V. (2008). "Single dose of St John's wort versus citalopram in patients with moderate depression." Zeitschrift fur Phytotherapie **29**(3): 131-132.

Schulzz, V. (2009). "Rhodiola rosea radix extract for the treatment of mild to moderate depression? Results of a placebo-controlled clinical study." KIM - Komplementare und Integrative Medizin, Artztezeitschrift fur Naturheilverfahren **50**(3): 53-54.

Sempels, C. and P. Sienaert (2007). "The role of omega-3 fatty acids in the treatment of bipolar disorders: The current situation." Tijdschrift voor Psychiatrie **49**(9): 639-647.

Severus, W. E. (2006). "Effects of omega-3 polyunsaturated fatty acids on depression." Herz **31 Suppl 3**: 69-74.

Sharifi, A., H. Vahedi, S. Nedjat, A. Mohamadkhani and M. J. Hosseinzadeh Attar (2019). "Vitamin D Decreases Beck Depression Inventory Score in Patients with Mild to Moderate Ulcerative Colitis: A Double-Blind Randomized Placebo-Controlled Trial." Journal of Dietary Supplements **16**(5): 541-549.

Sheikh, M., S. Hantoushzadeh, M. Shariat, Z. Farahani and O. Ebrahiminasab (2017). "The efficacy of early iron supplementation on postpartum depression, a randomized double-blind placebo-controlled trial." European Journal of Nutrition **56**(2): 901-908.

Shemshian, M., A. Sadeghi, T. Kermani, H. M. Seyed, S. Tavalaie and A. Norouzi (2011). "Saffron (crocus sativus) in the treatment of anxiety and depression: a double-blind, randomized and placebo-controlled trial."

Sherpa, N. N., R. De Giorgi, E. G. Ostinelli, A. Choudhury, T. Dolma and S. Dorjee (2025). "Efficacy and safety profile of oral creatine monohydrate in add-on to cognitive-behavioural therapy in depression: An 8-week pilot, double-blind, randomised, placebo-controlled feasibility and exploratory trial in an under-resourced area." European Neuropsychopharmacology **90**: 28-35.

Shiloh, R., A. Weizman, N. Weizer, P. Dorfman-Etrog and H. Munitz (2001). "Antidepressive effect of pyridoxine (vitamin B6) in neuroleptic-treated schizophrenic patients with co-morbid minor depression - Preliminary open-label trial." Harefuah **140**(5): 369-373+456.

Shipowick, C. D., C. B. Moore, C. Corbett and R. Bindler (2009). "Vitamin D and depressive symptoms in women during the winter: A pilot study." Applied Nursing Research **22**(3): 221-225.

Shippy, R. A., D. Mendez, K. Jones, I. Cergnul and S. E. Karpiak (2004). "S-adenosylmethionine (SAM-e) for the treatment of depression in people living with HIV/AIDS." BMC psychiatry **4**.

Silvers, K. M., C. C. Woolley, F. C. Hamilton, P. M. Watts and R. A. Watson (2005). "Randomised double-blind placebo-controlled trial of fish oil in the treatment of depression." Prostaglandins Leukotrienes and Essential Fatty Acids **72**(3): 211-218.

Singer, A., M. Schmidt, W. Hauke and K. Stade (2011). "Duration of response after treatment of mild to moderate depression with Hypericum extract STW 3-VI, citalopram and placebo: A reanalysis of data from a controlled clinical trial." Phytomedicine.

Sinn, N., C. M. Milte, S. J. Street, J. D. Buckley, A. M. Coates, J. Petkov and P. R. Howe (2012). "Effects of n-3 fatty acids, EPA v. DHA, on depressive symptoms, quality of life, memory and executive function in older adults with mild cognitive impairment: a 6-month randomised controlled trial." **107**(11): 1682‐1693

C1683 - PUBMED 21929835.

Slykerman, R. F., F. Hood, K. Wickens, J. M. D. Thompson, C. Barthow, R. Murphy, J. Kang, J. Rowden, P. Stone, J. Crane, T. Stanley, P. Abels, G. Purdie, R. Maude and E. A. Mitchell (2017). "Effect of Lactobacillus rhamnosus HN001 in Pregnancy on Postpartum Symptoms of Depression and Anxiety: A Randomised Double-blind Placebo-controlled Trial." EBioMedicine **24**: 159-165.

Smith, D. J., J. Sarris, N. Dowling, M. O'Connor and C. H. Ng (2018). "Adjunctive low-dose docosahexaenoic acid (DHA) for major depression: An open-label pilot trial." Nutritional Neuroscience **21**(3): 224-228.

Solati, Z., S. Jazayeri, M. Tehrani-Doost, S. Mahmoodianfard and M. R. Gohari (2015). "Zinc monotherapy increases serum brain-derived neurotrophic factor (BDNF) levels and decreases depressive symptoms in overweight or obese subjects: a double-blind, randomized, placebo-controlled trial." **18**(4): 162‐168

C163 - PUBMED 24621065,EMBASE 603838110.

Soltani, M., M. J. Hosseinzadeh-Attar, M. Rezaei, E. Alipoor, A. Vasheghani-Farahani, M. Yaseri and S. M. Rezayat (2024). "Effect of nano-curcumin supplementation on cardiometabolic risk factors, physical and psychological quality of life, and depression in patients with coronary slow flow phenomenon: a randomized double-blind clinical trial." Trials **25**(1 C7 - 515).

Song, Y. K., J. Y. Lee and W. Park (2016). "Modulatory Effect of Eui-E-In-Tang on Serum Leptin Concentration in Obese Korean Female Adults: A Randomized Controlled Trial." Evid Based Complement Alternat Med **2016**: 1894837.

Sousa, T. M. D. and L. C. D. Santos (2020). "Dietary fatty acids, omega-6/omega-3 ratio and cholesterol intake associated with depressive symptoms in low-risk pregnancy." Nutritional Neuroscience: 1-6.

Stevinson, C. and E. Ernst (1999). "Hypericum for depression: An update of the clinical evidence." European Neuropsychopharmacology **9**(6): 501-505.

Stewart, R. C., P. Ashorn, E. Umar, K. G. Dewey, U. Ashorn, F. Creed, A. Rahman, B. Tomenson, E. L. Prado and K. Maleta (2017). "The impact of maternal diet fortification with lipid-based nutrient supplements on postpartum depression in rural Malawi: a randomised-controlled trial." Maternal and Child Nutrition **13**(2 C7 - e12299).

Strodl, E., M. Bambling, S. Parnam, G. Ritchie, S. Cramb and L. Vitetta (2024). "Probiotics and magnesium orotate for the treatment of major depressive disorder: a randomised double blind controlled trial." Scientific reports **14**(1): 20841.

Su, K. P., S. Y. Huang, T. H. Chiu, K. C. Huang, C. L. Huang, H. C. Chang and C. M. Pariante (2008). "Omega-3 fatty acids for major depressive disorder during pregnancy: Results from a randomized, double-blind, placebo-controlled trial." Journal of Clinical Psychiatry **69**(4): 644-651.

Su, K. P., H. C. Lai, H. T. Yang, W. P. Su, C. Y. Peng, J. P. Chang, H. C. Chang and C. M. Pariante (2014). "Omega-3 fatty acids in the prevention of interferon-alpha-induced depression: results from a randomized, controlled trial." Biol Psychiatry **76**(7): 559-566.

Suzuki, H., S. Masuki, A. Morikawa, Y. Ogawa, Y. I. Kamijo, K. Takahashi, M. Nakajima and H. Nose (2018). "Effects of 5-aminolevulinic acid supplementation on home-based walking training achievement in middle-aged depressive women: Randomized, double-blind, crossover pilot study." Scientific Reports **8**(1 C7 - 7151).

Syed, E. U., M. Wasay and S. Awan (2013). "Vitamin B12 supplementation in treating major depressive disorder: A randomized controlled trial." Open Neurology Journal **7**(1): 44-48.

Tabasi, M., S. A. Mostafavi, H. Oreyzi, M. R. Mohammadi and A. Khaleghi (2024). "The Effectiveness of Transcranial Direct Current Stimulation (tDCS) and Omega-3 on Food Craving, Executive Functions, Weight, and Depressive Symptoms in Patients with Depression and Overweight: A Randomized Controlled Trial." Iranian Journal of Psychiatry **19**(2): 158-170.

Tabeshpour, J., F. Sobhani, S. A. Sadjadi, H. Hosseinzadeh, S. A. Mohajeri, O. Rajabi, Z. Taherzadeh and S. Eslami (2017). "A double-blind, randomized, placebo-controlled trial of saffron stigma (Crocus sativus L.) in mothers suffering from mild-to-moderate postpartum depression." Phytomedicine **36**: 145-152.

Taheri, M., K. Irandoust, A. Sadeghi and S. Yari (2018). "The effect of omega-3 fatty acid supplement and aerobic exercise on lipid profile and depression in obese women." Acta Medica Mediterranea **34**(3): 865-870.

Tajalizadekhoob, Y., F. Sharifi, H. Fakhrzadeh, M. Mirarefin, M. Ghaderpanahi, Z. Badamchizade and S. Azimipour (2011). "The effect of low-dose omega 3 fatty acids on the treatment of mild to moderate depression in the elderly: A double-blind, randomized, placebo-controlled study." European Archives of Psychiatry and Clinical Neuroscience **261**(8): 539-549.

Tarkesh, F., B. Namavar Jahromi, N. Hejazi and G. Hoseini (2022). "Effect of vitamin K2 administration on depression status in patients with polycystic ovary syndrome: a randomized clinical trial." BMC Women's Health **22**(1).

Tarleton, E. K., B. Littenberg, C. D. MacLean, A. G. Kennedy and C. Daley (2017). "Role of magnesium supplementation in the treatment of depression: A randomized clinical trial." PLoS ONE **12**(6 C7 - e0180067).

Tatum, P. and E. J. Lindbloom (2001). "Is St. John's wort an effective treatment for major depression?" Journal of Family Practice **50**(7): 624.

Tayama, J., S. Ogawa, N. Nakaya, T. Sone, T. Hamaguchi, A. Takeoka, K. Hamazaki, H. Okamura, J. Yajima, M. Kobayashi and et al. (2019). "Omega-3 polyunsaturated fatty acids and psychological intervention for workers with mild to moderate depression: a double-blind randomized controlled trial." **245**: 364‐370

C363 - PUBMED 30423463,EMBASE 2001261185.

Tayebi, N., M. Emamghoreishi and M. Akbarzadeh (2021). "Effect of vitex agnus-castus on depression of postmenopausal women: A randomized clinical trial." Shiraz E Medical Journal **22**(6).

Tayebi, N., M. Emamghoreishi and M. Akbarzadeh (2024). "The Impact of Salvia officinalis Extract on Depression in Postmenopausal Women: A Randomized Clinical Trial." International Journal of Women's Health and Reproduction Sciences **12**(2).

Taylor, C. L., P. R. Thomas, J. F. Aloia, P. S. Millard and C. J. Rosen (2015). "Questions about Vitamin D for Primary Care Practice: Input from an NIH Conference." American Journal of Medicine **128**(11): 1167-1170.

Tessou, K. D., H. Lemus, F. C. Hsu, J. Pierce, S. Hong, L. Brown and T. Wu (2021). "Independent and joint impacts of acid-producing diets and depression on physical health among breast cancer survivors." Nutrients **13**(7 C7 - 2422).

Thesing, C. S., Y. Milaneschi, M. Bot, I. A. Brouwer, M. Owens, U. Hegerl, M. Gili, M. Roca, E. Kohls, E. Watkins and et al. (2020). "Supplementation-induced increase in circulating omega-3 serum levels is not associated with a reduction in depressive symptoms: results from the MooDFOOD depression prevention trial." **37**(11): 1079‐1088

C1073 - PUBMED 32845021,EMBASE 2005970673.

Thesing, C. S., Y. Milaneschi, M. Bot, I. A. Brouwer, M. Owens, U. Hegerl, M. Gili, M. Roca, E. Kohls, E. Watkins, M. Visser and B. Penninx (2020). "Supplementation-induced increase in circulating omega-3 serum levels is not associated with a reduction in depressive symptoms: Results from the MooDFOOD depression prevention trial." Depress Anxiety **37**(11): 1079-1088.

Thomson, M. and V. Sharma (2023). "Alternative Treatments for Postpartum Depression." Current Treatment Options in Psychiatry **10**(3): 288-312.

Tian, P., Y. Chen, H. Zhu, L. Wang, X. Qian, R. Zou, J. Zhao, H. Zhang, L. Qian, Q. Wang and et al. (2022). "Bifidobacterium breve CCFM1025 attenuates major depression disorder via regulating gut microbiome and tryptophan metabolism: a randomized clinical trial." **100**: 233‐241

C233 - PUBMED 34875345.

Tian, P., H. Yang, F. Hang, G. Wang, X. Mao, X. Jin and J. Zhao (2024). "Evaluation of the clinical efficacy of Pediococcus acidilactici CCFM6432 in alleviating depression." Microbiome Research Reports **3**(4 C7 - 49).

Tiemeier, H., H. Ruud van Tuijl, A. Hofman, J. Meijer, A. J. Kiliaan and M. M. B. Breteler (2002). "Vitamin B12, folate, and homocysteine in depression: The Rotterdam study." American Journal of Psychiatry **159**(12): 2099-2101.

Tomko, R. L., N. L. Baker, C. O. Hood, A. K. Gilmore, E. A. McClure, L. M. Squeglia, A. L. McRae-Clark, S. C. Sonne and K. M. Gray (2020). "Depressive symptoms and cannabis use in a placebo-controlled trial of N-Acetylcysteine for adult cannabis use disorder." Psychopharmacology **237**(2): 479-490.

Tomko, R. L., A. K. Gilmore and K. M. Gray (2018). "The role of depressive symptoms in treatment of adolescent cannabis use disorder with N-Acetylcysteine." Addictive Behaviors **85**: 26-30.

Tzikos, G., E. Chamalidou, D. Christopoulou, A. Apostolopoulou, S. Gkarmiri, M. Pertsikapa, A. E. Menni, I. M. Theodorou, G. Stavrou, N. D. Doutsini, A. D. Shrewsbury, T. Papavramidis, J. K. Tsetis, H. Theodorou, A. Konsta and K. Kotzampassi (2025). "Psychobiotics Ameliorate Depression and Anxiety Status in Surgical Oncology Patients: Results from the ProDeCa Study." Nutrients **17**(5).

Ullah, H., A. Di Minno, C. Esposito, H. R. El-Seedi, S. A. M. Khalifa, A. Baldi, A. Greco, S. Santonastaso, V. Cioffi, R. Sperandeo and et al. (2022). "Efficacy of a food supplement based on S-adenosyl methionine and probiotic strains in subjects with subthreshold depression and mild-to-moderate depression: a monocentric, randomized, cross-over, double-blind, placebo-controlled clinical trial." **156**: 113930

C113933 - PUBMED 36411659.

Van De Rest, O., J. De Goede, F. Sytsma, L. M. Oude Griep, J. M. Geleijnse, D. Kromhout and E. J. Giltay (2010). "Association of n-3 long-chain PUFA and fish intake with depressive symptoms and low dispositional optimism in older subjects with a history of myocardial infarction." British Journal of Nutrition **103**(9): 1381-1387.

Vaz, J., D. Farias, A. Adegboye, A. Nardi and G. Kac (2018). "N-3 polyunsaturated fatty acids (PUFAs) supplementation from pregnancy to postpartum to prevent depressive symptoms: A randomized placebo-controlled trial." Nutritional Neuroscience **21**: S1.

Vaz, J. D. S., D. R. Farias, A. R. A. Adegboye, A. E. Nardi and G. Kac (2017). "Omega-3 supplementation from pregnancy to postpartum to prevent depressive symptoms: a randomized placebo-controlled trial." BMC Pregnancy Childbirth **17**(1): 180.

Vaziri, F., S. Nasiri, Z. Tavana, M. H. Dabbaghmanesh, F. Sharif and P. Jafari (2016). "A randomized controlled trial of vitamin D supplementation on perinatal depression: In Iranian pregnant mothers." BMC Pregnancy and Childbirth **16**(1 C7 - 239).

Velichkov, M., Z. Bezur, C. M. van Reekum and C. M. Williams (2024). "A biphasic response to blueberry supplementation on depressive symptoms in emerging adults: a double-blind randomized controlled trial." European Journal of Nutrition **63**(4): 1071-1088.

Venkatasubramanian, R., C. N. Kumar and R. S. Pandey (2013). "A randomized double-blind comparison of fluoxetine augmentation by high and low dosage folic acid in patients with depressive episodes." Journal of Affective Disorders **150**(2): 644-648.

Vreijling, S. R., B. Penninx, M. Bot, E. Watkins, M. Owens, E. Kohls, U. Hegerl, M. Roca, M. Gili, I. A. Brouwer and et al. (2021). "Effects of dietary interventions on depressive symptom profiles: results from the MooDFOOD depression prevention study." **52**(15): 1‐10

C13 - PUBMED 33823960,EMBASE 634757999.

Vyas, C. M., D. Mischoulon, G. Chang, N. R. Cook, A. Weinberg, T. Copeland, J. H. Kang, V. Bubes, G. Friedenberg, M. S. LeBoff and et al. (2023). "Effects of Vitamin D3 and Marine Omega-3 Fatty Acids Supplementation on Indicated and Selective Prevention of Depression in Older Adults: results From the Clinical Center Sub-Cohort of the VITamin D and OmegA-3 TriaL (VITAL)." **84**(4).

Wadud, S., S. Fatima and M. Irfan (2024). "ROLE OF ASCORBIC ACID IN IMPROVING MOOD IN PATIENTS WITH DEPRESSION: A RANDOMIZED CONTROLLED TRIAL." Journal of Postgraduate Medical Institute **38**(2): 97-101.

Walker, J. G., P. J. Batterham, A. J. Mackinnon, A. F. Jorm, I. Hickie, M. Fenech, M. Kljakovic, D. Crisp and H. Christensen (2012). "Oral folic acid and vitamin B-12 supplementation to prevent cognitive decline in community-dwelling older adults with depressive symptoms - The Beyond Ageing Project: A randomized controlled trial (American Journal of Clinical Nutrition (2012) 95, (194-203))." American Journal of Clinical Nutrition **96**(2): 448.

Walker, J. G., A. J. Mackinnon, P. Batterham, A. F. Jorm, I. Hickie, A. McCarthy, M. Fenech and H. Christensen (2010). "Mental health literacy, folic acid and vitamin B12, and physical activity for the prevention of depression in older adults: Randomised controlled trial." British Journal of Psychiatry **197**(1): 45-54.

Walker, S. P., S. M. Chang, C. A. Powell, E. Simonoff and S. M. Grantham-McGregor (2006). "Effects of psychosocial stimulation and dietary supplementation in early childhood on psychosocial functioning in late adolescence: follow-up of randomised controlled trial." **333**(7566): 472

C473 - PUBMED 16877454,EMBASE 44343443.

Wallace, C. J. K. and R. V. Milev (2021). "The Efficacy, Safety, and Tolerability of Probiotics on Depression: Clinical Results From an Open-Label Pilot Study." Frontiers in Psychiatry **12 C7 - 618279**.

Wang, L., Y. Zhang, L. Chen, D. Yuan, X. Feng, J. Zheng, X. Li and C. Yuan (2024). "A two-center randomized controlled trial of a repairing mask as an adjunctive treatment for mild to moderate rosacea." J Cosmet Dermatol **23**(10): 3281-3286.

Wang, X. J., J. Li, Q. D. Zou and L. Jin (2009). "Wuling Capsule for climacteric patients with depression and anxiety state: A randomized, positive parallel controlled trial." Journal of Chinese Integrative Medicine **7**(11): 1042-1046.

Wasielewski, S. (2003). "Herbal psychotropic drugs for the treatment of mild depression?" Deutsche Apotheker Zeitung **143**(39): 38-39.

Wheatley, D. (1997). "LI 160, an extract of St. John's wort, versus amitriptyline in mildly to moderately depressed outpatients--a controlled 6-week clinical trial." **30 Suppl 2**: 77‐80

C73 - PUBMED 9342764.

Wu, S. K., K. J. Yang, W. C. Liu, I. A. Malau, H. Zailani, C. H. Chang, S. Y. Huang, J. P. C. Chang, W. C. Chiu and K. P. Su (2024). "The Efficacy of Omega-3 Fatty Acids as the Monotherapy for Depression: A Randomized, Double-Blind, Placebo-Controlled Pilot Study." Nutrients **16**(21 C7 - 3688).

Xiao, F., L. Liu, W. P. Zhang, X. Y. Chang and Y. F. Zhang (2016). "Effect of adding magnesium sulfate to intrathecal low-dose of bupivacaine for patients with severe pre-eclampsia undergoing cesarean delivery." **9**(10): 19749‐19756

C19743 - EMBASE 612964624.

Xue, Y., L. Wang, T. Liu, T. Zhao, K. Xie, J. Guo, J. Chen, H. Tang and M. Tang (2024). "Omega-3 polyunsaturated fatty acids supplementation improves memory in first-diagnosed, drug-naïve patients with depression: Secondary analysis of data from a randomized controlled trial." Journal of Affective Disorders **350**: 403-410.

Yalamanchili, V. and J. C. Gallagher (2016). "Comparison of the effect of vitamin D and 1,25 dihydroxyvitamin D treatment on depression scores in older women." Endocrine Reviews **37**(2).

Yalamanchili, V. and J. C. Gallagher (2017). "Effect of vitamin D supplementation on depression scores in younger caucasian and Africa-American women." Endocrine Reviews **38**(3).

Yalamanchili, V. and J. C. Gallagher (2018). "Dose ranging effects of vitamin D3 on the geriatric depression score: A clinical trial." Journal of Steroid Biochemistry and Molecular Biology **178**: 60-64.

Yamada, K., G. Yagi and S. Kanba (2005). "Effectiveness of herbal medicine (Rokumigan and Hachimijiogan) for fatigue or loss of energy in patients with partial remitted major depressive disorder." Psychiatry and Clinical Neurosciences **59**(5): 610-612.

Yang, B., L. Lin, R. P. Bazinet, Y. C. Chien, J. P. C. Chang, S. K. Satyanarayanan, H. Su and K. P. Su (2019). "Clinical Efficacy and Biological Regulations of ω-3 PUFA-Derived Endocannabinoids in Major Depressive Disorder." Psychotherapy and Psychosomatics **88**(4): 215-224.

Yang, C., F. J. Bosker, J. Li and R. A. Schoevers (2018). "N-acetylcysteine as add-on to antidepressant medication in therapy refractory major depressive disorder patients with increased inflammatory activity: Study protocol of a double-blind randomized placebo-controlled trial." BMC Psychiatry **18**(1).

Yang, T. J., C. H. Chiu, C. L. Wu, Y. S. Liao and C. K. Chang (2021). "Thirty minutes of moderate-intensity downhill or level running has no effect on postprandial lipemia: A randomized controlled trial." Chin J Physiol **64**(5): 244-250.

Yoon, S., J. E. Kim, J. Hwang, T. S. Kim, H. J. Kang, E. Namgung, S. Ban, S. Oh, J. Yang, P. F. Renshaw and I. K. Lyoo (2016). "Effects of Creatine Monohydrate Augmentation on Brain Metabolic and Network Outcome Measures in Women With Major Depressive Disorder." Biological Psychiatry **80**(6): 439-447.

Yosaee, S., S. Soltani, A. Esteghamati, S. A. Motevalian, M. Tehrani-Doost, C. C. T. Clark and S. Jazayeri (2020). "Effects of zinc, vitamin D, and their co-supplementation on mood, serum cortisol, and brain-derived neurotrophic factor in patients with obesity and mild to moderate depressive symptoms: A phase II, 12-wk, 2 × 2 factorial design, double-blind, randomized, placebo-controlled trial." Nutrition **71 C7 - 110601**.

Yu, J., P. Chukaew and M. Rasenick (2020). "A Molecular Rationale for n-3 PUFA Augmentation of Antidepressant Action." Biological Psychiatry **87**(9): S40.

Zailani, H., S. K. Wu, K. J. Yang, I. A. Malau, H. F. Liao, Y. L. Chung, J. P. Chang, W. C. Chiu and K. P. Su (2024). "Omega-3 polyunsaturated fatty acids in the prevention of relapse in patients with stable bipolar disorder: A 6-month pilot randomized controlled trial." Psychiatry Res **331**: 115633.

Zajecka, J. M., M. Fava, R. C. Shelton, L. W. Barrentine, P. Young and G. I. Papakostas (2016). "Long-Term Efficacy, Safety, and Tolerability of l-Methylfolate Calcium 15 mg as Adjunctive Therapy With Selective Serotonin Reuptake Inhibitors: A 12-Month, Open-Label Study Following a Placebo-Controlled Acute Study." Journal of Clinical Psychiatry **77**(5): 654-660.

Zaromytidou, E., T. Koufakis, G. Dimakopoulos, D. Drivakou, S. Konstantinidou, P. Rakitzi, M. Grammatiki, E. Manthou, A. Notopoulos, I. Iakovou and et al. (2022). "Vitamin D Alleviates Anxiety and Depression in Elderly People with Prediabetes: a Randomized Controlled Study." **12**(10 C3 - PUBMED 36295786,EMBASE 2019781038).

Zaromytidou, E., T. Koufakis, G. Dimakopoulos, D. Drivakou, G. Meristoudis, S. Konstantinidou, G. Karaliolios, E. Melidou, P. Rakitzi, M. Grammatiki and et al. (2022). "Vitamin D Supplementation Improves Anxiety and Depression Status in Elderly People with Prediabetes: an Open-Label 12-Month Randomized Controlled Study." **71 C3 - EMBASE 641933907**.

Zhang, Y., Y. Cao and L. Wang (2020). "The effects of a new, improved Chinese medicine, Gengnianchun formula granules, on hot flushes, depression, anxiety, and sleep in Chinese peri-and postmenopausal women: A randomized placebo-controlled trial." Menopause **27**(8): 899-905.

Zhang, Z. J. (2012). "Herbal medicine and acupuncture for unipolar and bipolar depression: Randomized controlled trials." Bipolar Disorders **14**: 137.

Zhao, A. M., W. R. Qiu, L. J. Mao, J. G. Ren, L. Xu, M. J. Yao, K. Bilinksi, D. Chang and J. X. Liu (2018). "The efficacy and safety of Jiedu Tongluo granules for treating post-stroke depression with qi deficiency and blood stasis syndrome: study protocol for a randomized controlled trial." Trials **19**(1): 275.

Zheng, S., L. Tu, F. Cicuttini, W. Han, Z. Zhu, B. Antony, A. Wluka, T. Winzenberg, T. Meng, D. Aitken, L. Blizzard, G. Jones and C. Ding (2019). "Effect of Vitamin D Supplementation on Depressive Symptoms in Patients With Knee Osteoarthritis." Journal of the American Medical Directors Association **20**(12): 1634-1640.e1631.

Zheng, S., L. Tu, F. Cicuttini, W. Han, Z. Zhu, B. Antony, A. Wluka, T. Winzenberg, T. Meng, L. Blizzard, G. Jones and C. Ding (2019). "Vitamin D supplementation improves depressive symptoms in knee osteoarthritis patients over 24 months." European Journal of Immunology **49**: 1421.

Zheng, Y., L. J. Xu, L. Shi, B. Pang and F. Q. Xu (2019). "Modified erxian decoction for peri-menopausal depression: A randomized controlled clinical trial." Psychotherapy and Psychosomatics **88**: 142.

Zhou, Z., S. Fu, Y. Liu, Y. Wang, H. Bu, Y. Mei, Y. Tong and C. Yu (2020). "Study of efficacy and safety of Jiaotai pill in the treatment of depression." Medicine (United States) **99**(18): E19999.

Zimmer, R., T. Riemer, B. Rauch, S. Schneider, R. Schiele, H. Gohlke, F. Diller, G. Steinbeck, H. Katus and J. Senges (2013). "Effects of 1-year treatment with highly purified omega-3 fatty acids on depression after myocardial infarction: Results from the OMEGA trial." Journal of Clinical Psychiatry **74**(11): e1037-e1045.

# **S3a**: Risk of bias assessment.

**e-table 1.** Risk of bias summary of randomized Intention-to-treat studies (Cochrane risk-of-bias tool for randomized trials (RoB 2)).

**e-table 2.** Risk of bias summary of randomized Per-Protocol studies (Cochrane risk-of-bias tool for randomized cross-over trials (RoB 2)).

## **S3.1:** Legend of RoB2.

# **S3b**: AMSTAR-Plus score applied to low-RoB2-RCTs documenting compounds reported in at least 2 RCTs.

| **Author and year of the MA** | **1** | **2** | **3** | **4** | **5** | **6** | **7** | **8** | **9** | **10** | **11** | **Methodological quality** | **1+** | **2+** | **3+** | **4+** | **5+** | **6+** | **Content quality** | **Quality (AMSTAR-PLUS) of the MA** |
| --- | --- | --- | --- | --- | --- | --- | --- | --- | --- | --- | --- | --- | --- | --- | --- | --- | --- | --- | --- | --- |
| Ahmadpanah M et al., 2019 | 1 | 0 | 1 | 0 | 1 | 1 | 0 | 1 | 1 | 0 | 1 | 7 | 1 | 0 | 1 | 0 | 1 | 0 | 3 | low |
| Akkasheh G et al., 2015 | 1 | 0 | 1 | 0 | 1 | 1 | 0 | 1 | 1 | 1 | 1 | 8 | 1 | 0 | 0 | 0 | 1 | 1 | 3 | low |
| Basoglu C. et al., 2009 | 1 | 1 | 0 | 1 | 1 | 1 | 1 | 1 | 1 | 1 | 1 | 10 | 1 | 0 | 0 | 0 | 1 | 1 | 3 | low |
| Behnke K. et al., 2002 | 1 | 0 | 1 | 0 | 1 | 1 | 0 | 1 | 1 | 1 | 1 | 8 | 1 | 0 | 1 | 0 | 1 | 0 | 3 | low |
| Bergman J et al., 2013 | 1 | 0 | 1 | 0 | 1 | 1 | 0 | 1 | 1 | 1 | 1 | 8 | 1 | 0 | 0 | 0 | 1 | 1 | 3 | low |
| Bidzan-Wiacek M. et al., 2024 | 1 | 1 | 1 | 1 | 1 | 1 | 1 | 1 | 1 | 1 | 1 | 11 | 1 | 0 | 0 | 1 | 1 | 1 | 4 | low |
| Bot M et al., 2010 | 1 | 0 | 1 | 0 | 1 | 1 | 0 | 1 | 1 | 1 | 1 | 8 | 1 | 0 | 1 | 0 | 1 | 0 | 4 | low |
| Brenner R et al., 2000 | 1 | 1 | 1 | 1 | 1 | 1 | 1 | 1 | 1 | 1 | 1 | 11 | 0 | 1 | 0 | 1 | 1 | 1 | 4 | low |
| Carney R.M. et al., 2016 | 1 | 1 | 1 | 1 | 1 | 1 | 1 | 1 | 1 | 1 | 1 | 11 | 1 | 0 | 1 | 0 | 1 | 0 | 4 | low |
| Cheng Y. et al., 2025 | 1 | 1 | 1 | 1 | 1 | 1 | 1 | 1 | 1 | 1 | 1 | 11 | 1 | 0 | 1 | 0 | 1 | 0 | 4 | low |
| Davidson J.R.T. et al., 2002 | 1 | 1 | 1 | 1 | 1 | 1 | 1 | 1 | 1 | 1 | 1 | 11 | 1 | 0 | 1 | 0 | 1 | 0 | 3 | low |
| Docherty JP et al., 2005 | 1 | 1 | 1 | 1 | 1 | 1 | 1 | 1 | 1 | 1 | 1 | 11 | 1 | 0 | 1 | 0 | 1 | 0 | 3 | low |
| Dormal V. et al., 2025 | 1 | 1 | 1 | 1 | 1 | 1 | 1 | 1 | 1 | 1 | 1 | 11 | 1 | 0 | 1 | 0 | 1 | 1 | 4 | low |
| Fava M et al., 2005 | 1 | 0 | 1 | 0 | 1 | 1 | 0 | 1 | 1 | 1 | 1 | 8 | 1 | 0 | 1 | 0 | 1 | 1 | 4 | low |
| Gao L et al., 2020 | 1 | 0 | 1 | 0 | 1 | 1 | 0 | 1 | 1 | 1 | 1 | 8 | 0 | 1 | 1 | 0 | 1 | 1 | 4 | low |
| Gelenberg A. et al., 2004 | 1 | 1 | 1 | 1 | 1 | 1 | 1 | 1 | 1 | 1 | 1 | 11 | 0 | 1 | 0 | 0 | 1 | 1 | 3 | low |
| Ghajar A et al., 2016 | 1 | 1 | 1 | 1 | 1 | 1 | 1 | 1 | 1 | 1 | 1 | 11 | 0 | 1 | 0 | 0 | 1 | 1 | 3 | low |
| Harrer G et al., 1999 | 1 | 0 | 1 | 0 | 1 | 1 | 0 | 1 | 1 | 1 | 1 | 8 | 1 | 0 | 1 | 0 | 1 | 0 | 3 | low |
| Jelodar G. et al., 2017 | 1 | 1 | 0 | 1 | 1 | 1 | 1 | 1 | 1 | 1 | 1 | 10 | 0 | 1 | 0 | 1 | 1 | 1 | 4 | low |
| Kalb R. et al., 2001 | 1 | 1 | 1 | 1 | 1 | 1 | 1 | 1 | 1 | 1 | 1 | 11 | 0 | 1 | 0 | 0 | 1 | 1 | 3 | low |
| Kanchanatawan B et al., 2018 | 1 | 0 | 1 | 0 | 1 | 1 | 0 | 1 | 1 | 0 | 1 | 7 | 0 | 0 | 1 | 1 | 1 | 1 | 4 | low |
| Kashani L et al., 2018 | 1 | 0 | 1 | 0 | 1 | 1 | 0 | 1 | 1 | 1 | 1 | 8 | 1 | 0 | 1 | 0 | 1 | 0 | 3 | low |
| Kasper S. et al., 2006 | 1 | 1 | 1 | 1 | 1 | 1 | 1 | 1 | 1 | 1 | 1 | 11 | 0 | 1 | 0 | 0 | 1 | 1 | 3 | low |
| Kasper S. et al., 2007 | 1 | 0 | 1 | 0 | 1 | 1 | 0 | 1 | 1 | 1 | 1 | 8 | 0 | 1 | 0 | 0 | 1 | 1 | 3 | low |
| Laakmann G et al., 1998 | 1 | 1 | 1 | 1 | 1 | 1 | 1 | 1 | 1 | 1 | 1 | 11 | 1 | 1 | 0 | 0 | 1 | 1 | 4 | low |
| Lecrubier Y. et al., 2002 | 1 | 1 | 1 | 1 | 1 | 1 | 1 | 1 | 1 | 1 | 1 | 11 | 1 | 0 | 1 | 0 | 1 | 0 | 3 | low |
| Lopresti AL et al., 2014 | 1 | 0 | 1 | 0 | 1 | 1 | 0 | 1 | 1 | 1 | 1 | 8 | 0 | 0 | 0 | 1 | 1 | 1 | 3 | low |
| Lopresti AL et al., 2017 | 1 | 1 | 1 | 1 | 1 | 1 | 1 | 1 | 1 | 1 | 1 | 11 | 0 | 1 | 0 | 1 | 1 | 1 | 4 | low |
| Marangell LB et al., 2003 | 1 | 0 | 1 | 0 | 1 | 1 | 0 | 1 | 1 | 0 | 1 | 7 | 0 | 0 | 0 | 1 | 1 | 1 | 3 | low |
| Mazidi, 2016 | 1 | 1 | 0 | 1 | 1 | 1 | 1 | 1 | 1 | 1 | 1 | 10 | 0 | 0 | 0 | 1 | 1 | 1 | 3 | low |
| Mischoulon D et al., 2009 | 1 | 1 | 1 | 1 | 1 | 1 | 1 | 1 | 1 | 1 | 1 | 11 | 0 | 0 | 0 | 1 | 1 | 1 | 3 | low |
| Mischoulon D et al., 2014 | 1 | 0 | 1 | 0 | 1 | 1 | 0 | 1 | 1 | 0 | 1 | 7 | 1 | 0 | 1 | 0 | 1 | 0 | 3 | low |
| Moshiri E et al., 2006 | 1 | 1 | 1 | 1 | 1 | 1 | 1 | 1 | 1 | 1 | 1 | 11 | 0 | 0 | 0 | 1 | 1 | 1 | 3 | low |
| Mozaffari-Khosravi H et al., 2012 | 1 | 1 | 1 | 1 | 1 | 1 | 1 | 1 | 1 | 1 | 1 | 11 | 0 | 0 | 0 | 0 | 1 | 1 | 2 | low |
| Noorbala AA et al., 2005 | 1 | 0 | 1 | 0 | 1 | 1 | 0 | 1 | 1 | 1 | 1 | 8 | 1 | 0 | 0 | 1 | 1 | 1 | 4 | low |
| Papakostas GI et al., 2010 | 1 | 1 | 0 | 1 | 1 | 1 | 1 | 1 | 1 | 1 | 1 | 10 | 0 | 0 | 0 | 0 | 1 | 1 | 2 | low |
| Papakostas GI et al., 2012 (a) | 1 | 0 | 1 | 0 | 1 | 1 | 0 | 1 | 1 | 0 | 1 | 7 | 0 | 0 | 0 | 0 | 1 | 1 | 2 | low |
| Papakostas GI et al., 2012 (b) | 1 | 1 | 1 | 1 | 1 | 1 | 1 | 1 | 1 | 1 | 1 | 11 | 1 | 0 | 0 | 1 | 1 | 1 | 4 | low |
| Rees AM et al., 2008 | 1 | 1 | 1 | 1 | 1 | 1 | 1 | 1 | 1 | 1 | 1 | 11 | 0 | 0 | 0 | 0 | 1 | 1 | 2 | low |
| Rondanelli M. et al., 2010 | 1 | 0 | 1 | 0 | 1 | 1 | 0 | 1 | 1 | 1 | 1 | 8 | 0 | 0 | 1 | 1 | 1 | 1 | 4 | low |
| Sahraian A. et al., 2015 | 1 | 1 | 1 | 1 | 1 | 1 | 1 | 1 | 1 | 1 | 1 | 11 | 1 | 0 | 0 | 1 | 1 | 1 | 4 | low |
| Sakurai H. et al., 2021 | 1 | 1 | 1 | 1 | 1 | 1 | 1 | 1 | 1 | 1 | 1 | 11 | 1 | 0 | 1 | 0 | 1 | 1 | 4 | low |
| Sanmukhani J et al., 2013 | 1 | 0 | 1 | 0 | 1 | 1 | 0 | 1 | 1 | 0 | 1 | 7 | 0 | 1 | 0 | 1 | 1 | 1 | 4 | low |
| Sarris J et al., 2018 | 1 | 1 | 1 | 1 | 1 | 1 | 1 | 1 | 1 | 1 | 1 | 11 | 0 | 0 | 1 | 1 | 1 | 1 | 4 | low |
| Sarris J. et al., 2012 | 1 | 1 | 1 | 1 | 1 | 1 | 1 | 1 | 1 | 1 | 1 | 11 | 0 | 0 | 1 | 1 | 1 | 1 | 4 | low |
| Schrader e et al., 1999 | 1 | 0 | 1 | 0 | 1 | 1 | 0 | 1 | 1 | 1 | 1 | 8 | 1 | 0 | 1 | 1 | 0 | 1 | 4 | low |
| Schrader E et al., 2000 | 1 | 1 | 1 | 1 | 1 | 1 | 1 | 1 | 1 | 1 | 1 | 11 | 1 | 0 | 1 | 0 | 1 | 1 | 4 | low |
| Shelton RC et al., 2001 | 1 | 1 | 0 | 1 | 1 | 1 | 1 | 1 | 1 | 1 | 1 | 10 | 1 | 1 | 1 | 0 | 1 | 0 | 4 | low |
| Shinto L et al., 2016 | 1 | 1 | 1 | 1 | 1 | 1 | 1 | 1 | 1 | 1 | 1 | 11 | 1 | 0 | 1 | 0 | 0 | 1 | 3 | low |
| Su K-P et al., 2008 | 1 | 0 | 1 | 0 | 1 | 1 | 0 | 1 | 1 | 0 | 1 | 7 | 0 | 0 | 1 | 1 | 1 | 1 | 4 | low |
| Szegedi A et al. 2005 | 1 | 1 | 1 | 1 | 1 | 1 | 1 | 1 | 1 | 1 | 1 | 11 | 1 | 0 | 1 | 0 | 0 | 1 | 3 | low |
| Targum SD et al., 2018 | 1 | 0 | 1 | 0 | 1 | 1 | 0 | 1 | 1 | 1 | 1 | 8 | 1 | 0 | 1 | 0 | 0 | 1 | 3 | low |
| Vorbach EU et al., 1997 | 1 | 1 | 0 | 1 | 1 | 1 | 1 | 1 | 1 | 1 | 1 | 10 | 1 | 0 | 0 | 1 | 0 | 1 | 3 | low |
| Wang Y et al., 2016 | 1 | 1 | 1 | 1 | 1 | 1 | 1 | 1 | 1 | 1 | 1 | 11 | 0 | 0 | 0 | 1 | 0 | 1 | 2 | low |
| Wheatley D et al., 1997 | 1 | 1 | 1 | 1 | 1 | 1 | 1 | 1 | 1 | 1 | 1 | 11 | 1 | 0 | 0 | 1 | 0 | 1 | 3 | low |
| Wu SK et al., 2024 | 1 | 0 | 1 | 0 | 1 | 1 | 0 | 1 | 1 | 1 | 1 | 8 | 0 | 0 | 0 | 1 | 0 | 1 | 2 | low |
| Zhang L. et al., 2018 | 1 | 0 | 1 | 0 | 1 | 1 | 0 | 1 | 1 | 0 | 1 | 7 | 0 | 0 | 0 | 1 | 0 | 1 | 2 | low |

**Legend for table:** + = AMSTAR-Plus Content score items

AMSTAR-Plus quantifies both the methodological quality of MAs and NMAs with the first 11 items (AMSTAR) and of included RCTs with six additional items (AMSTAR-Content). Methodological quality was categorized into low (<4), medium (4-7), and high (>7). Content quality was categorized into low (<4), medium (4-6), and high (>6).

Note: **The lower of the two scores between methodological and content quality determined the overall MA or NMA quality.**

# **S4:** Legend of molecules and classes.

| **LABEL** | **Molecule** | **Class (either pharmacological or nutraceutical/phytoceutical)** |
| --- | --- | --- |
| APH | Aphrodite | Herbal compound |
| AYA | Ayahuasca | Herbal compound |
| BBL | Bifidobacterium longum | Probiotic |
| BZT | Sodium Benzoate | Coenzyme |
| CAR | L-Carnitine | Amino acids |
| CHRO | Chromium picolinate | Minerals |
| CIN | Cinnamomum Tamala | Lauraceae |
| CIT | Citicoline | Nucleotide |
| CLO | Clomipramine | TCA |
| CQ10 | Coenzyme Q10 | Coenzyme |
| CREA | Creatine | Amino acids |
| CTP | Citalopram | SSRI |
| CUR | Curcumin | Zingiberaceae |
| CUS | Cuscuta planiflora | Convolvulaceae |
| CVE | Chlorella Vulgaris extract | Microalgae |
| DHA | Docosahesanoic acid | LCPUFA |
| DRA | Dracocephalum ruyschiana | Lamiaceae |
| EA | Ellagic acid | Polyphenols |
| EACS | Echium amoenum compound syrup | Herbal compound |
| EOPF | essential oil of Perilla frutescens | LCPUFA |
| EPA | Eicosapentanoic acid | LCPUFA |
| ESC | Escitalopram | SSRI |
| fAA | Free amino acids | Amino acids |
| FLUO | Fluoxetine | SSRI |
| FOIL | Fish oil | LCPUFA |
| FOL | Acid Folic | Vitamins |
| GBDP | Ginkgo biloba dropping pill | Ginkgoaceae |
| GGBT | Gami Guibitang (Korean traditional medicinal herb) | Herbal compound |
| GOS | Galactooligosaccharide | Polysaccarid |
| HEAL9 | Lactobacillus Plantarum HEAL9 | Probiotic |
| HYP | Hypericum perforatum | Hypericum |
| IMI | Imipramine | TCA |
| INO | Inositol | Vitamins |
| INU | Inulin | Polysaccarid |
| JTP | Jiaotai pill | Herbal compound |
| KOIL | Krill oil | LCPUFA |
| LAV | Lavandula angostifulia | Lamiaceae |
| LBA | Lactobacillus acidophilus | Probiotic |
| LBH | Lactobacillus helveticus | Probiotic |
| LCPUFA |  | Long chain polyunsaturate fatty acids |
| LH57 | LoHyp57 (hypericum extract) | Hypericum |
| LI-160 | LI-160 St John’s wort extract | Hypericum |
| LMF | l-methyl-folate | vitamins |
| LP299v | Lactobacillus Plantarum299v | PROB |
| LS | Lactosucrose | Polysaccharide |
| MEL | Melissa Officinalis | Lamiaceae |
| MG | Magnesium | Minerals |
| MIR | Mirtazapine | NASSA |
| NAC | N-acetylcysteine | Amino acids |
| NaSSA |  | Noradrenergic and specific serotonergic antidepressant |
| NEP | Nepeta menthoides | Lamiaceae |
| NUT | Nutraceutical combination (SAMe+folinic acid+B12+omega3+5-HTP+zinc+B6+vitC+magnesium) | Coenzyme+vitamins+LCPUFA+minerals+amino acids |
| OMB | OmniBiotic drink | Probiotic |
| PAR | Paroxetine | SSRI |
| PROB |  | Probiotic |
| PLB |  | Placebo |
| PRO | Propolis | Flavonoid+Polyphenols |
| PS128 | Lactobacillus Plantarum PS128 | PROB |
| RHO | Rhodiola Rosea | Crassulaceae |
| SAF | Saffron (=Crocus sativus) | Iridaceae |
| SAMe | S-adenosylmethionine | Coenzyme |
| SAR | Sarcosine (N-methyl-glycine) | Amino acids |
| SCU | Scutellaria | Herbal compound |
| SER | Sertraline | SSRI |
| SHU | Shugan granule | Herbal compound |
| SIL | Silexan (active substance from Lav.angustifolia) | Lamiaceae |
| SJW | St John's wort | Hypericum |
| SNRI |  | Serotonin-Norepinephrine Reuptake Inhibitor |
| SRN | D-Serine | Amino acids |
| SSRI |  | Selective Serotonin Reuptake Inhibitor |
| SUL | Sulbutiamine | vitamins |
| SYN | Synbiotic | Probiotic |
| SZL | Shen Zi Ling | Herbal compound |
| TEN | Tensilen | Coenzyme |
| THE | L-theanine | Amino acids |
| THI | Thiamine (Vitamin B1) | vitamins |
| US | Üstikuddus sherbiti | Herbal compound |
| VEN | Venlafaxine | SNRI |
| WS5570 | Hypericum perforatum extract WS5570 | Hypericum |
| WS5572 | St John's wort extract WS 5572 | Hypericum |
| WS5573 | St John's wort extract WS 5573 | Hypericum |
| YUE | Yueju | Herbal compound |
| ZE | Hypericum extract ZE | Hypericum |
| ZE117 | St John's wort extract ZE 117 | Hypericum |
| ZNC | Zinc | Minerals |
| ZPG | Zishen pingchan granules | Herbal compound |

# **S5**: Subnetworks for each outcome and specific node analysis.

**e-table 3**: Efficacy node 1 subnetworks.

| **Author(s), year** | **Treatment** | **n** | **Subnetwork** |
| --- | --- | --- | --- |
| ^455^ | FOIL | 16 | 1 |
| ^455^ | KOIL | 17 | 1 |
| ^455^ | PLB | 17 | 1 |
| ^456^ | SAFF | 25 | 1 |
| ^456^ | SER | 25 | 1 |
| ^457^ | SAFF | 20 | 1 |
| ^457^ | FLUO | 20 | 1 |
| ^458^ | SAFF | 15 | 1 |
| ^458^ | IMI | 15 | 1 |
| ^459^ | SAF | 27 | 1 |
| ^459^ | PLB | 25 | 1 |
| ^460^ | MEL | 21 | 1 |
| ^460^ | PLB | 22 | 1 |
| ^460^ | LAV | 15 | 1 |
| ^460^ | IMI | 15 | 1 |
| ^460^ | LAV+IMI | 15 | 1 |
| ^461^ | PROB | 20 | 1 |
| ^461^ | PLB | 20 | 1 |
| ^462^ | vitD3+CA | 26 | 1 |
| ^462^ | vitD3 | 26 | 1 |
| ^462^ | PLB | 24 | 1 |
| ^463^ | EACS | 21 | 1 |
| ^463^ | CTP | 21 | 1 |
| ^464^ | MEL | 15 | 1 |
| ^464^ | LAV | 15 | 1 |
| ^464^ | FLUO | 15 | 1 |
| ^465^ | BIOKULT | 35 | 1 |
| ^465^ | PLB | 36 | 1 |
| ^466^ | ESC+FOL | 20 | 1 |
| ^466^ | ESC | 15 | 1 |
| ^467^ | SJW | 35 | 1 |
| ^467^ | FLUO | 35 | 1 |
| ^468^ | SAMe | 9 | 1 |
| ^468^ | IMI | 9 | 1 |
| ^469^ | CUR | 19 | 1 |
| ^469^ | PLB | 20 | 1 |
| ^470^ | NAC+TAU | 125 | 1 |
| ^470^ | PLB+TAU | 127 | 1 |
| ^471^ | EPA+DHA | 25 | 1 |
| ^471^ | PLB | 22 | 1 |
| ^472^ | LI-160 | 54 | 1 |
| ^472^ | FLUO | 54 | 1 |
| ^472^ | PLB | 55 | 1 |
| ^473^ | EPA | 12 | 1 |
| ^473^ | PLB | 12 | 1 |
| ^474^ | LI-160 | 13 | 1 |
| ^474^ | SER | 15 | 1 |
| ^475^ | SER+EPA | 71 | 1 |
| ^475^ | SER+PLB | 73 | 1 |
| ^476^ | SER+EPA+DHA | 62 | 1 |
| ^476^ | SER+PLB | 60 | 1 |
| ^477^ | SAMe | 30 | 1 |
| ^477^ | PLB | 29 | 1 |
| ^478^ | OMEGA3 | 30 | 1 |
| ^478^ | PLB | 29 | 1 |
| ^479^ | EPA+DHA | 19 | 1 |
| ^479^ | PLB | 20 | 1 |
| ^480^ | SAFF | 20 | 1 |
| ^480^ | FLUO | 20 | 1 |
| ^481^ | RHO | 60 | 1 |
| ^481^ | PLB | 29 | 1 |
| ^482^ | CHRO | 10 | 1 |
| ^482^ | PLB | 5 | 1 |
| ^483^ | LI-160 | 113 | 1 |
| ^483^ | SER | 109 | 1 |
| ^483^ | PLB | 116 | 1 |
| ^484^ | SAMe | 19 | 1 |
| ^484^ | PLB | 17 | 1 |
| ^485^ | TEN | 30 | 1 |
| ^485^ | PLB | 30 | 1 |
| ^486^ | CHRO | 70 | 1 |
| ^486^ | PLB | 40 | 1 |
| ^487^ | SCU | 42 | 1 |
| ^487^ | SAFF+SCU | 48 | 1 |
| ^487^ | SAFF | 41 | 1 |
| ^487^ | PLB | 46 | 1 |
| ^488^ | LI-160 | 45 | 1 |
| ^488^ | FLUO | 47 | 1 |
| ^488^ | PLB | 43 | 1 |
| ^489^ | NEP | 15 | 1 |
| ^489^ | CUS | 13 | 1 |
| ^489^ | TAU | 15 | 1 |
| ^490^ | EPA+DHA | 28 | 1 |
| ^490^ | PLB | 23 | 1 |
| ^491^ | ZE117 | 126 | 1 |
| ^491^ | FLUO | 114 | 1 |
| ^492^ | RHO+SER | 66 | 1 |
| ^492^ | SER | 32 | 1 |
| ^493^ | LI-160 | 43 | 1 |
| ^493^ | PLB | 55 | 1 |
| ^494^ | SER+PLB | 23 | 1 |
| ^494^ | SER+CIN | 19 | 1 |
| ^495^ | SAFF | 30 | 1 |
| ^495^ | CTP | 30 | 1 |
| ^496^ | SYN | 20 | 1 |
| ^496^ | PLB | 20 | 1 |
| ^497^ | OMEGA3 | 40 | 1 |
| ^497^ | PLB | 43 | 1 |
| ^498^ | GGBT | 82 | 1 |
| ^498^ | PLB | 82 | 1 |
| ^499^ | LH57 | 70 | 1 |
| ^499^ | FLUO | 79 | 1 |
| ^500^ | GBDP | 34 | 1 |
| ^500^ | PLB | 34 | 1 |
| ^501^ | EA | 20 | 1 |
| ^501^ | PLB | 20 | 1 |
| ^502^ | SZL | 63 | 1 |
| ^502^ | FLUO | 66 | 1 |
| ^503^ | vitB12 | 70 | 1 |
| ^503^ | PLB | 70 | 1 |
| ^504^ | EPA | 16 | 1 |
| ^504^ | FLUO | 16 | 1 |
| ^504^ | EPA+FLUO | 16 | 1 |
| ^505^ | SAMe | 9 | 1 |
| ^505^ | PLB | 6 | 1 |
| ^506^ | WS5572 | 37 | 1 |
| ^506^ | PLB | 35 | 1 |
| ^507^ | CUR | 33 | 1 |
| ^507^ | PLB | 32 | 1 |
| ^508^ | SAFF | 28 | 1 |
| ^508^ | PLB | 28 | 1 |
| ^509^ | SAFF | 32 | 1 |
| ^509^ | FLUO | 32 | 1 |
| ^510^ | WS5570 | 243 | 1 |
| ^510^ | PLB | 81 | 1 |
| ^511^ | WS5570 | 68 | 1 |
| ^511^ | PLB | 24 | 1 |
| ^512^ | SIL | 141 | 1 |
| ^512^ | PLB | 128 | 1 |
| ^513^ | vitD | 28 | 1 |
| ^513^ | PLB | 28 | 1 |
| ^514^ | vitD3+FLUO | 20 | 1 |
| ^514^ | FLUO | 20 | 1 |
| ^515^ | NEP | 33 | 1 |
| ^515^ | SER | 33 | 1 |
| ^516^ | OMB | 28 | 1 |
| ^516^ | PLB | 29 | 1 |
| ^517^ | vitD | 31 | 1 |
| ^517^ | PLB | 28 | 1 |
| ^518^ | WS5573 | 49 | 1 |
| ^518^ | WS5572 | 49 | 1 |
| ^518^ | PLB | 49 | 1 |
| ^211^ | WS5570 | 186 | 1 |
| ^211^ | PLB | 189 | 1 |
| ^519^ | INO | 13 | 1 |
| ^519^ | PLB | 15 | 1 |
| ^520^ | SHU | 194 | 1 |
| ^520^ | PLB | 191 | 1 |
| ^521^ | PS128 | 16 | 1 |
| ^521^ | PLB | 16 | 1 |
| ^522^ | BZT | 37 | 1 |
| ^522^ | SER | 34 | 1 |
| ^522^ | PLB | 38 | 1 |
| ^523^ | CUR | 61 | 1 |
| ^523^ | CUR+SAFF | 26 | 1 |
| ^523^ | PLB | 36 | 1 |
| ^221^ | CUR | 28 | 1 |
| ^221^ | PLB | 28 | 1 |
| ^524^ | CQ10+TAU | 36 | 1 |
| ^524^ | PLB+TAU | 33 | 1 |
| ^525^ | RHO | 20 | 1 |
| ^525^ | SER | 19 | 1 |
| ^525^ | PLB | 18 | 1 |
| ^526^ | DHA | 18 | 1 |
| ^526^ | PLB | 17 | 1 |
| ^527^ | SAF | 30 | 1 |
| ^527^ | PLB | 30 | 1 |
| ^528^ | vitB | 159 | 1 |
| ^528^ | PLB | 123 | 1 |
| ^254^ | EPA | 16 | 1 |
| ^254^ | PLB | 19 | 1 |
| ^529^ | SAMe | 64 | 1 |
| ^529^ | ESC | 65 | 1 |
| ^529^ | PLB | 60 | 1 |
| ^530^ | SAFF | 20 | 1 |
| ^530^ | PLB | 20 | 1 |
| ^272^ | EPA | 21 | 1 |
| ^272^ | DHA | 20 | 1 |
| ^272^ | PLB | 21 | 1 |
| ^531^ | CREA | 4 | 1 |
| ^531^ | PLB | 4 | 1 |
| ^296^ | EPA | 10 | 1 |
| ^296^ | PLB | 10 | 1 |
| ^532^ | ZPG | 89 | 1 |
| ^532^ | PLB | 87 | 1 |
| ^533^ | SAFF | 20 | 1 |
| ^533^ | FLUO | 20 | 1 |
| ^534^ | ZNC+TAU | 6 | 1 |
| ^534^ | PLB+TAU | 8 | 1 |
| ^535^ | SAR+TAU | 30 | 1 |
| ^535^ | PLB+TAU | 30 | 1 |
| ^536^ | CVE | 42 | 1 |
| ^536^ | TAU | 50 | 1 |
| ^537^ | SAMe | 146 | 1 |
| ^537^ | IMI | 147 | 1 |
| ^538^ | SAMe+TAU | 39 | 1 |
| ^538^ | PLB+TAU | 34 | 1 |
| ^539^ | LMF | 36 | 1 |
| ^539^ | PLB | 112 | 1 |
| ^539^ | LMF | 19 | 1 |
| ^539^ | PLB | 56 | 1 |
| ^540^ | OMEGA3 | 12 | 1 |
| ^540^ | PLB | 13 | 1 |
| ^541^ | EPA+DHA | 14 | 1 |
| ^541^ | PLB | 9 | 1 |
| ^542^ | ZNC+TAU | 20 | 1 |
| ^542^ | PLB+TAU | 17 | 1 |
| ^332^ | EPA+DHA | 13 | 1 |
| ^332^ | PLB | 13 | 1 |
| ^543^ | EPA+DHA | 22 | 1 |
| ^543^ | PLB | 24 | 1 |
| ^544^ | LP299v | 30 | 1 |
| ^544^ | PLB | 30 | 1 |
| ^545^ | SAMe+HELP9 | 46 | 1 |
| ^545^ | PLB | 44 | 1 |
| ^546^ | EACS+SJW | 23 | 1 |
| ^546^ | FLUO | 28 | 1 |
| ^547^ | SER+EPA+DHA | 30 | 1 |
| ^547^ | SER+5HTP | 30 | 1 |
| ^547^ | SER | 30 | 1 |
| ^548^ | SAMe | 16 | 1 |
| ^548^ | ESC | 23 | 1 |
| ^548^ | PLB | 26 | 1 |
| ^549^ | CUR | 16 | 1 |
| ^549^ | FLUO | 17 | 1 |
| ^549^ | CUR+FLUO | 18 | 1 |
| ^550^ | SAMe+TAU | 37 | 1 |
| ^550^ | PLB+TAU | 39 | 1 |
| ^551^ | NUT | 56 | 1 |
| ^551^ | PLB | 57 | 1 |
| ^552^ | SAMe | 22 | 1 |
| ^552^ | PLB | 19 | 1 |
| ^553^ | ZE117 | 125 | 1 |
| ^553^ | FLUO | 113 | 1 |
| ^554^ | ZE117 | 80 | 1 |
| ^554^ | PLB | 79 | 1 |
| ^555^ | SRN | 22 | 1 |
| ^555^ | PLB | 22 | 1 |
| ^556^ | vitD | 18 | 1 |
| ^556^ | PLB | 18 | 1 |
| ^557^ | SER+LAV+DRA | 35 | 1 |
| ^557^ | SER+PLB | 35 | 1 |
| ^558^ | SAFF | 20 | 1 |
| ^558^ | FLUO | 20 | 1 |
| ^559^ | APH | 21 | 1 |
| ^559^ | PLB | 19 | 1 |
| ^560^ | SER+THE | 25 | 1 |
| ^560^ | SER+PLB | 25 | 1 |
| ^561^ | EOPF | 60 | 1 |
| ^561^ | PLB | 60 | 1 |
| ^562^ | SJW | 98 | 1 |
| ^562^ | PLB | 102 | 1 |
| ^563^ | CIT | 28 | 1 |
| ^563^ | PLB | 20 | 1 |
| ^564^ | EPA+DHA | 15 | 1 |
| ^564^ | PLB | 16 | 1 |
| ^565^ | EPA+DHA | 12 | 1 |
| ^565^ | PLB | 10 | 1 |
| ^387^ | EPA+DHA | 17 | 1 |
| ^387^ | PLB | 16 | 1 |
| ^566^ | MG | 40 | 1 |
| ^566^ | PLB | 38 | 1 |
| ^567^ | WS5570 | 122 | 1 |
| ^567^ | PAR | 122 | 1 |
| ^568^ | SER+CUR | 20 | 1 |
| ^568^ | SER+PLB | 20 | 1 |
| ^569^ | SAMe+TAU | 118 | 1 |
| ^569^ | PLB+TAU | 116 | 1 |
| ^570^ | LS | 9 | 1 |
| ^570^ | PLB | 11 | 1 |
| ^571^ | INU | 31 | 1 |
| ^571^ | PLB | 31 | 1 |
| ^572^ | SJW | 44 | 1 |
| ^572^ | SER | 43 | 1 |
| ^573^ | ZNC+TAU | 30 | 1 |
| ^573^ | TAU | 30 | 1 |
| ^574^ | LI-160 | 107 | 1 |
| ^574^ | IMI | 102 | 1 |
| ^575^ | vitD3 | 212 | 1 |
| ^575^ | PLB | 204 | 1 |
| ^429^ | LI-160 | 83 | 1 |
| ^429^ | AMI | 73 | 1 |
| ^576^ | ZE | 157 | 1 |
| ^576^ | IMI | 167 | 1 |
| ^577^ | EPA+DHA | 30 | 1 |
| ^577^ | PLB | 30 | 1 |
| ^578^ | vitD3 | 56 | 1 |
| ^578^ | PLB | 64 | 1 |
| ^579^ | LAV+PLB | 25 | 2 |
| ^579^ | CTP+PLB | 25 | 2 |
| ^580^ | CTP+PLB | 30 | 2 |
| ^580^ | CTP+OMEGA3 | 30 | 2 |
| ^581^ | FOL+CTP | 45 | 2 |
| ^581^ | CTP+PLB | 45 | 2 |
| ^582^ | THI+FLUO | 25 | 3 |
| ^582^ | PLB+FLUO | 26 | 3 |
| ^583^ | MG+FLUO | 17 | 3 |
| ^583^ | PLB+FLUO | 15 | 3 |
| ^584^ | FOL+FLUO | 51 | 4 |
| ^584^ | FLUO+PLB | 58 | 4 |
| ^585^ | SAFF+FLUO | 20 | 4 |
| ^585^ | FLUO+PLB | 20 | 4 |
| ^586^ | CIT+CTP | 25 | 5 |
| ^586^ | PLB+CTP | 25 | 5 |
| ^585^ | vitC+CTP | 21 | 5 |
| ^585^ | PLB+CTP | 22 | 5 |
| ^587^ | OMEGA3+SER | 25 | 6 |
| ^587^ | PLB+SER | 25 | 6 |
| ^588^ | CAR+SER | 30 | 6 |
| ^588^ | PLB+SER | 30 | 6 |
| ^589^ | vitD3+TAU | 23 | 7 |
| ^589^ | TAU+PLB | 23 | 7 |
| ^590^ | SAFF+TAU | 20 | 8 |
| ^590^ | PLB*TAU | 20 | 8 |
| ^591^ | FLUO+SAFF | 20 | 9 |
| ^591^ | FLUO + PLB | 20 | 9 |
| ^592^ | fAA+MIR | 20 | 10 |
| ^592^ | PLB+MIR | 20 | 10 |
| ^593^ | ESC+CREA | 25 | 11 |
| ^593^ | ESC+PLB | 27 | 11 |
| ^594^ | CUR+ESC | 50 | 12 |
| ^594^ | PLB+ESC | 50 | 12 |
| ^595^ | EPA+DHA+VEN | 21 | 13 |
| ^595^ | PLB+VEN | 27 | 13 |

**Reference for e-table 3**

1.	Açik M, Çakiroğlu FP, Tekin A, Egeli A. The effect of fish oil versus krill oil intervention on clinical symptoms and cardiometabolic risk factors in patients with major depressive disorder: A randomized placebo-controlled double-blind trial. Journal of Affective Disorders. 2025;380:171-80.

2.	Ahmadpanah M, Ramezanshams F, Ghaleiha A, Akhondzadeh S, Sadeghi Bahmani D, Brand S. Crocus Sativus L. (saffron) versus sertraline on symptoms of depression among older people with major depressive disorders–a double-blind, randomized intervention study. Psychiatry Research. 2019;282.

3.	Akhondzadeh Basti A, Moshiri E, Noorbala AA, Jamshidi AH, Abbasi SH, Akhondzadeh S. Comparison of petal of Crocus sativus L. and fluoxetine in the treatment of depressed outpatients: A pilot double-blind randomized trial. Progress in Neuro-Psychopharmacology and Biological Psychiatry. 2007;31(2):439-42.

4.	Akhondzadeh S, Fallah-Pour H, Afkham K, Jamshidi AH, Khalighi-Cigaroudi F. Comparison of Crocus sativus L. and imipramine in the treatment of mild to moderate depression: A pilot double-blind randomized trial [ISRCTN45683816]. BMC Complementary and Alternative Medicine. 2004;4.

5.	Akhondzadeh S, Mostafavi SA, Keshavarz SA, Mohammadi MR, Hosseini S, Eshraghian MR. A placebo controlled randomized clinical trial of Crocus sativus L. (saffron) on depression and food craving among overweight women with mild to moderate depression. Journal of Clinical Pharmacy and Therapeutics. 2020;45(1):134-43.

6.	Akhondzadeh S, Mostafavi SA, Keshavarz SA, Mohammadi MR, Chamari M. Melatonin Effects in Women With Comorbidities of Overweight, Depression, and Sleep Disturbance: A Randomized Placebo Controlled Clinical Trial. Sleep Med Res. 2022;13(1):22-30.

7.	Akkasheh G, Kashani-Poor Z, Tajabadi-Ebrahimi M, Jafari P, Akbari H, Taghizadeh M, et al. Clinical and metabolic response to probiotic administration in patients with major depressive disorder: A randomized, double-blind, placebo-controlled trial. Nutrition. 2016;32(3):315-20.

8.	Amini L, Yaghini O, Ghazavi M, Aslani N. L-carnitine versus Propranolol for pediatric migraine prophylaxis. Iran J Child Neurol. 2021;15(2):77-86.

9.	Anushiravani M, Manteghi AA, Taghipur A, Eslami M. Comparing Effectiveness of a Combined Herbal Drug Based on Echium Amoenum with Citalopram in the Treatment of Major Depressive Disorder. Curr Drug Discov Technol. 2019;16(2):232-8.

10.	Araj-Khodaei M, Noorbala AA, Yarani R, Emadi F, Emaratkar E, Faghihzadeh S, et al. A double-blind, randomized pilot study for comparison of Melissa officinalis L. And Lavandula angustifolia Mill. And Fluoxetine for the treatment of depression. BMC Complementary Medicine and Therapies. 2020;20(1).

11.	Baião R, Capitão LP, Higgins C, Browning M, Harmer CJ, Burnet PWJ. Multispecies probiotic administration reduces emotional salience and improves mood in subjects with moderate depression: a randomised, double-blind, placebo-controlled study. Psychological medicine. 2023;53(8):3437-47.

12.	Basoglu C, Ates MA, Algul A, Ipcioglu OM, Gecici O, Yilmaz O, et al. Adjuvant folate with escitalopram treatment and homocystein, folate, vitamin B-12 levels in patients with major depressive disorder. 2009;19(2):135‐42

C3 - EMBASE 354832073.

13.	Behnke K, Jensen GS, Graubaum HJ, Gruenwald J. Hypericum perforatum versus fluoxetine in the treatment of mild to moderate depression. Advances in Therapy. 2002;19(1):43-52.

14.	Bell KM, Plon L, Bunney Jr WE, Potkin SG. S-adenosylmethionine treatment of depression: A controlled clinical trial. American Journal of Psychiatry. 1988;145(9):1110-4.

15.	Bergman J, Miodownik C, Bersudsky Y, Sokolik S, Lerner PP, Kreinin A, et al. Curcumin as an add-on to antidepressive treatment: A randomized, double-blind, placebo-controlled, pilot clinical study. Clinical Neuropharmacology. 2013;36(3):73-7.

16.	Berk M, Dean OM, Cotton SM, Jeavons S, Tanious M, Kohlmann K, et al. The efficacy of adjunctive N-acetylcysteine in major depressive disorder: A double-blind, randomized, placebo-controlled trial. Journal of Clinical Psychiatry. 2014;75(6):628-36.

17.	Bidzan-Wiącek M, Tomczyk M, Błażek M, Mika A, Antosiewicz J. No Effects of Omega-3 Supplementation on Kynurenine Pathway, Inflammation, Depressive Symptoms, and Stress Response in Males: A Placebo-Controlled Trial. Nutrients. 2024;16(21 C7 - 3744).

18.	Bjerkenstedt L, Edman GV, Alken RG, Mannel M. Hypericum extract LI 160 and fluoxetine in mild to moderate depression: A randomized, placebo-controlled multi-center study in outpatients. European Archives of Psychiatry and Clinical Neuroscience. 2005;255(1):40-7.

19.	Bot M, Pouwer F, Assies J, Jansen EH, Diamant M, Snoek FJ, et al. Eicosapentaenoic acid as an add-on to antidepressant medication for co-morbid major depression in patients with diabetes mellitus: a randomized, double-blind placebo-controlled study. J Affect Disord. 2010;126(1-2):282-6.

20.	Brenner R, Azbel V, Madhusoodanan S, Pawlowska M. Comparison of an extract of hypericum (LI 160) and sertraline in the treatment of depression: A double-blind, randomized pilot study. Clinical Therapeutics. 2000;22(4):411-9.

21.	Carney RM, Freedland KE, Rubin EH, Rich MW, Steinmeyer BC, Harris WS. A randomized placebo-controlled trial of omega-3 and sertraline in depressed patients with or at risk for coronary heart disease. Journal of Clinical Psychiatry. 2019;80(4 C7 - 19m12742).

22.	Carney RM, Steinmeyer BC, Freedland KE, Rubin EH, Rich MW, Harris WS. Baseline blood levels of omega-3 and depression remission: A secondary analysis of data from a placebo-controlled trial of omega-3 supplements. Journal of Clinical Psychiatry. 2016;77(2):e138-e43.

23.	Caruso I, Fumagalli M, Boccassini L, Puttini SP, Santandrea S, Giniselli G, et al. Treatment of depression in rheumatoid arthritic patients. A comparison of S-adenosylmethionine (Samyr) and placebo in a double-blind study. CLIN TRIALS J. 1987;24(4):305-10.

24.	Chang JP, Chang SS, Yang HT, Chen HT, Chien YC, Yang B, et al. Omega-3 polyunsaturated fatty acids in cardiovascular diseases comorbid major depressive disorder - Results from a randomized controlled trial. Brain Behav Immun. 2020;85:14-20.

25.	Cheng YC, Chen WY, Lin C, Lee SH, Chiu CC, Kuo PH. The N-3 polyunsaturated fatty acids supplementation to prevent depression recurrence in patients with late-life depression: A 52-week double-blind, placebo-controlled trial. Journal of Affective Disorders. 2025;369:8-15.

26.	Chopra A, Kalyani AK. Comparison of efficacy of Crocus sativus L. with fluoxetine for improving depression among patients with cardiovascular diseases. Int J Life Sci Biotechnol Pharma Res. 2020;9(2):151-4.

27.	Darbinyan V, Aslanyan G, Amroyan E, Gabrielyan E, Malmström C, Panossian A. Clinical trial of Rhodiola rosea L. extract SHR-5 in the treatment of mild to moderate depression. Nordic Journal of Psychiatry. 2007;61(5):343-8.

28.	Davidson JR, Gadde KM, Fairbank JA, Krishnan KRR, Califf RM, Binanay C, et al. Effect of Hypericum perforatum (St John's wort) in major depressive disorder: a randomized controlled trial. 2002;287(14):1807‐14

C3 - PUBMED 11939866,EMBASE 34303625.

29.	Davidson JRT, Gadde KM, Fairbank JA, Ranga Rama Krishnan K, Califf RM, Binanay C, et al. Effect of Hypericum perforatum (St John's wort) in major depressive disorder: A randomized controlled trial. JAMA. 2002;287(14):1807-14.

30.	De Leo D. S-Adenosylmethionine as an antidepressant. A double-blind trial versus placebo. CURR THER RES CLIN EXP. 1987;41(6):865-70.

31.	Djokic G, Korcok D, Djordjevic V, Agic A, Rankovic A, Djukic Dejanovic S. The effects of s-adenosyl-L-methionine-vitamin b complex on mild and moderate depressive symptoms. Hippokratia. 2017;21(3):140-3.

32.	Docherty JP, Sack DA, Roffman M, Finch M, Komorowski JR. A double-blind, placebo-controlled, exploratory trial of chromium picolinate in atypical depression: Effect on carbohydrate craving. Journal of Psychiatric Practice. 2005;11(5):302-14.

33.	Dormal V, Jonniaux L, Buchet M, Simar L, Copine S, Deldicque L. Effect of Hydroponically Grown Red Panax Ginseng on Perceived Stress Level, Emotional Processing, and Cognitive Functions in Moderately Stressed Adults: A Randomized, Double-Blind, Placebo-Controlled Study. Nutrients. 2025;17(6 C7 - 955).

34.	Fava M, Alpert J, Nierenberg AA, Mischoulon D, Otto MW, Zajecka J, et al. A double-blind, randomized trial of St John's wort, fluoxetine, and placebo in major depressive disorder. Journal of Clinical Psychopharmacology. 2005;25(5):441-7.

35.	Firoozabadi A, Zarshenas MM, Salehi A, Jahanbin S, Mohagheghzadeh A. Effectiveness of Cuscuta planiflora Ten. and Nepeta menthoides Boiss. & Buhse in major depression: a triple-blind randomized controlled trial study. J Evid Based Complementary Altern Med. 2015;20(2):94-7.

36.	Freeman MP, Davis M, Sinha P, Wisner KL, Hibbeln JR, Gelenberg AJ. Omega-3 fatty acids and supportive psychotherapy for perinatal depression: A randomized placebo-controlled study. Journal of Affective Disorders. 2008;110(1-2):142-8.

37.	Friede M, Henneicke von Zepelin HH, Freudenstein J. Differential therapy of mild to moderate depressive episodes (ICD-10 F 32.0; F 32.1) with St. John's wort. Pharmacopsychiatry. 2001;34(SUPPL. 1):S38-S41.

38.	Gao L, Wu C, Liao Y, Wang J. Antidepressants effects of Rhodiola capsule combined with sertraline for major depressive disorder: A randomized double-blind placebo-controlled clinical trial. Journal of Affective Disorders. 2020;265:99-103.

39.	Gelenberg AJ, Shelton RC, Crits-Christoph P, Keller MB, Dunner DL, Hirschfeld RM, et al. The effectiveness of St. John's Wort in major depressive disorder: a naturalistic phase 2 follow-up in which nonresponders were provided alternate medication. 2004;65(8):1114‐9

C3 - PUBMED 15323598.

40.	Ghaffari S, Ghobadi A, Jamshidi AH, Mortazavi SH, Naderi S, Aqamolaei A, et al. Cinnamomum tamala as an adjuvant therapy in the treatment of major depressive disorder: A double-blind, randomized, placebo-controlled clinical trial with placebo control. Advances in Integrative Medicine. 2020;7(3):141-7.

41.	Ghajar A, Neishabouri SM, Velayati N, Jahangard L, Matinnia N, Haghighi M, et al. Crocus sativus L versus Citalopram in the Treatment of Major Depressive Disorder with Anxious Distress: A Double-Blind, Controlled Clinical Trial. Pharmacopsychiatry. 2017;50(4):152-60.

42.	Ghorbani Z, Nazari S, Etesam F, Nourimajd S, Ahmadpanah M, Jahromi SR. The effect of synbiotic as an adjuvant therapy to fluoxetine in moderate depression: A randomized multicenter trial. Arch Neurosci. 2018;5(2).

43.	Grenyer BF, Crowe T, Meyer B, Owen AJ, Grigonis-Deane EM, Caputi P, et al. Fish oil supplementation in the treatment of major depression: a randomised double-blind placebo-controlled trial. 2007;31(7):1393‐6

C3 - PUBMED 17659823.

44.	Han CH, Chung MI, Bose S, Lim C, Kim H, Koo BS, et al. The effect of Gami Guibitang (GGBT), a medicinal herbal formulation, on moderate depression. European Journal of Integrative Medicine. 2012;4(4):e400-e7.

45.	Harrer G, Schmidt U, Kuhn U, Biller A. Comparison of equivalence between the St. John's wort extract LoHyp-57 and fluoxetine. Arzneimittel-Forschung/Drug Research. 1999;49(4):289-96.

46.	He X, Liu D, Ni S, Li Z, Li S, Wu T, et al. Efficacy and safety evaluation of Ginkgo biloba dropping pill (GBDP) on stable angina pectoris complicated with depression: A placebo-controlled, randomized, double-blind, multicenter study. Phytomedicine. 2024;126.

47.	Hosseini H, Bagherniya M, Sahebkar A, Iraj B, Majeed M, Askari G. The effect of curcumin-piperine supplementation on lipid profile, glycemic index, inflammation, and blood pressure in patients with type 2 diabetes mellitus and hypertriglyceridemia. Phytotherapy Research. 2024;38(11):5150-61.

48.	Hu Y, Chen C, Wang Y, Yang W, Wang Y, Zhu W, et al. The effects of KaiXinSan on depression and its association with lipid profiles: a randomized, double-blinded, placebo-controlled trial. 2021;83:153467

C3 - PUBMED 33516143,EMBASE 2010802711.

49.	Hvas AM, Juul S, Lauritzen L, Nexø E, Ellegaard J. No effect of vitamin B-12 treatment on cognitive function and depression: A randomized placebo controlled study. Journal of Affective Disorders. 2004;81(3):269-73.

50.	Jazayeri S, Tehrani-Doost M, Keshavarz SA, Hosseini M, Djazayery A, Amini H, et al. Comparison of therapeutic effects of omega-3 fatty acid eicosapentaenoic acid and fluoxetine, separately and in combination, in major depressive disorder. Australian and New Zealand Journal of Psychiatry. 2008;42(3):192-8.

51.	Kagan BL, Sultzer DL, Rosenlicht N, Gerner RH. Oral S-adenosylmethionine in depression: a randomized, double-blind, placebo-controlled trial. 1990;147(5):591‐5

C3 - PUBMED 2183633.

52.	Kalb R, Trautmann-Sponsel RD, Kieser M. Efficacy and tolerability of Hypericum extract WS 5572 versus placebo in mildly to moderately depressed patients: A randomized double-blind multicenter clinical trial. Pharmacopsychiatry. 2001;34(3):96-103.

53.	Kanchanatawan B, Tangwongchai S, Sughondhabhirom A, Suppapitiporn S, Hemrunrojn S, Carvalho AF, et al. Add-on Treatment with Curcumin Has Antidepressive Effects in Thai Patients with Major Depression: Results of a Randomized Double-Blind Placebo-Controlled Study. Neurotoxic Res. 2018;33(3):621-33.

54.	Kashani L, Esalatmanesh S, Eftekhari F, Salimi S, Foroughifar T, Etesam F, et al. Efficacy of Crocus sativus (saffron) in treatment of major depressive disorder associated with post-menopausal hot flashes: a double-blind, randomized, placebo-controlled trial. Arch Gynecol Obstet. 2018;297(3):717-24.

55.	Kashani L, Eslatmanesh S, Saedi N, Niroomand N, Ebrahimi M, Hosseinian M, et al. Comparison of Saffron versus Fluoxetine in Treatment of Mild to Moderate Postpartum Depression: A Double-Blind, Randomized Clinical Trial. Pharmacopsychiatry. 2017;50(2):64-8.

56.	Kasper S, Anghelescu IG, Szegedi A, Dienel A, Kieser M. Superior efficacy of St John's wort extract WS 5570 compared to placebo in patients with major depression: a randomized, double-blind, placebo-controlled, multi-center trial. 2006;4:14

C3 - PUBMED 16796730,EMBASE 44227549.

57.	Kasper S, Anghelescu IG, Szegedi A, Dienel A, Kieser M. Placebo controlled continuation treatment with Hypericum extract WS® 5570 after recovery from a mild or moderate depressive episode. Wiener Medizinische Wochenschrift. 2007;157(13-14):362-6.

58.	Kasper S, Anghelescu I, Dienel A. Efficacy of orally administered Silexan in patients with anxiety-related restlessness and disturbed sleep--A randomized, placebo-controlled trial. 2015;25(11):1960‐7

C3 - PUBMED 26293583,EMBASE 605643089.

59.	Kaviani M, Nikooyeh B, Etesam F, Behnagh SJ, Kangarani HM, Arefi M, et al. Effects of vitamin D supplementation on depression and some selected pro-inflammatory biomarkers: a double-blind randomized clinical trial. BMC Psychiatry. 2022;22(1 C7 - 694).

60.	Khoraminya N, Tehrani-Doost M, Jazayeri S, Hosseini A, Djazayery A. Therapeutic effects of vitamin D as adjunctive therapy to fluoxetine in patients with major depressive disorder. Australian and New Zealand Journal of Psychiatry. 2013;47(3):271-5.

61.	Kolouri S, Firoozabadi A, Salehi A, Zarshenas MM, Dastgheib SA, Heydari M, et al. Nepeta menthoides Boiss. & Buhse freeze-dried aqueous extract versus sertraline in the treatment of major depression: A double blind randomized controlled trial. Complement Ther Med. 2016;26:164-70.

62.	Kreuzer K, Reiter A, Birkl-Töglhofer AM, Dalkner N, Mörkl S, Mairinger M, et al. The PROVIT Study—Effects of Multispecies Probiotic Add-on Treatment on Metabolomics in Major Depressive Disorder—A Randomized, Placebo-Controlled Trial. Metabolites. 2022;12(8).

63.	Kumar PNS, Menon V, Andrade C. A randomized, double-blind, placebo-controlled, 12-week trial of vitamin D augmentation in major depressive disorder associated with vitamin D deficiency. Journal of Affective Disorders. 2022;314:143-9.

64.	Laakmann G. St. John's Wort in mild to moderate depression: The relevance of hyperforin for the clinical efficacy. Pharmacopsychiatry. 1998;31(SUPPL. 1):54-9.

65.	Lecrubier Y, Clerc G, Didi R, Kieser M. Efficacy of St. John's wort extract WS 5570 in major depression: A double-blind, placebo-controlled trial. American Journal of Psychiatry. 2002;159(8):1361-6.

66.	Levine J, Barak Y, Gonzalves M, Szor H, Elizur A, Kofman O, et al. Double-blind, controlled trial of inositol treatment of depression. American Journal of Psychiatry. 1995;152(5):792-4.

67.	Li Y, Li L, Guo R, Yu H, Wang X, Wang B, et al. Clinical efficacy of Shugan granule in the treatment of mixed anxiety-depressive disorder: a multicenter, randomized, double-blind, placebo-controlled trial. 2022;290:115032

C3 - PUBMED 35085742,EMBASE 637131287.

68.	Lin SKK, Kuo PH, Hsu CY, Chiu YH, Chen CH. The effects of Lactobacillus plantarum PS128 in patients with major depressive disorder: an eight-week double-blind, placebo-controlled study. Asian Journal of Psychiatry. 2024;101 C7 - 104210.

69.	Lin CH, Wang SH, Lane HY. Effects of Sodium Benzoate, a D-Amino Acid Oxidase Inhibitor, on Perceived Stress and Cognitive Function Among Patients With Late-Life Depression: A Randomized, Double-Blind, Sertraline- and Placebo-Controlled Trial. International Journal of Neuropsychopharmacology. 2022;25(7):545-55.

70.	Lopresti AL, Drummond PD. Efficacy of curcumin, and a saffron/curcumin combination for the treatment of major depression: A randomised, double-blind, placebo-controlled study. Journal of Affective Disorders. 2017;207:188-96.

71.	Lopresti AL, Maes M, Maker GL, Hood SD, Drummond PD. Curcumin for the treatment of major depression: A randomised, double-blind, placebo controlled study. Journal of Affective Disorders. 2014;167:368-75.

72.	Majmasanaye M, Mehrpooya M, Amiri H, Eshraghi A. Discovering the Potential Value of Coenzyme Q10 as an Adjuvant Treatment in Patients with Depression. Journal of Clinical Psychopharmacology. 2024;44(3):232-9.

73.	Mao JJ, Xie SX, Zee J, Soeller I, Li QS, Rockwell K, et al. Rhodiola rosea versus sertraline for major depressive disorder: A randomized placebo-controlled trial. Phytomedicine. 2015;22(3):394-9.

74.	Marangell LB, Martinez JM, Zboyan HA, Kertz B, Kim HFS, Puryear LJ. A double-blind, placebo-controlled study of the omega-3 fatty acid docosahexaenoic acid in the treatment of major depression. American Journal of Psychiatry. 2003;160(5):996-8.

75.	Mazidi M, Shemshian M, Mousavi SH, Norouzy A, Kermani T, Moghiman T, et al. A double-blind, randomized and placebo-controlled trial of Saffron (Crocus sativus L.) in the treatment of anxiety and depression. 2016;13(2):195‐9

C3 - PUBMED 27101556,EMBASE 610483123.

76.	Mech AW, Farah A. Correlation of clinical response with homocysteine reduction during therapy with reduced b vitamins in patients with mdd who are positive for MTHFR C677T or A1298C polymorphism: A Randomized, double-blind, placebo-controlled study. Journal of Clinical Psychiatry. 2016;77(5):668-71.

77.	Mischoulon D, Best-Popescu C, Laposata M, Merens W, Murakami JL, Wu SL, et al. A double-blind dose-finding pilot study of docosahexaenoic acid (DHA) for major depressive disorder. European Neuropsychopharmacology. 2008;18(9):639-45.

78.	Mischoulon D, Price LH, Carpenter LL, Tyrka AR, Papakostas GI, Baer L, et al. A double-blind, randomized, placebo-controlled clinical trial of S-adenosyl-L-methionine (SAMe) versus escitalopram in major depressive disorder. J Clin Psychiatry. 2014;75(4):370-6.

79.	Moshiri E, Basti AA, Noorbala AA, Jamshidi AH, Hesameddin Abbasi S, Akhondzadeh S. Crocus sativus L. (petal) in the treatment of mild-to-moderate depression: A double-blind, randomized and placebo-controlled trial. Phytomedicine. 2006;13(9-10):607-11.

80.	Mozaffari-Khosravi H, Nabizade L, Yassini-Ardakani SM, Hadinedoushan H, Barzegar K. The effect of 2 different single injections of high dose of vitamin D on improving the depression in depressed patients with vitamin D deficiency: A randomized clinical trial. Journal of Clinical Psychopharmacology. 2013;33(3):378-85.

81.	Nemets B, Levine J. A pilot dose-finding clinical trial of creatine monohydrate augmentation to SSRIs/SNRIs/NASA antidepressant treatment in major depression. International Clinical Psychopharmacology. 2013;28(3):127-33.

82.	Nemets B, Stahl Z, Belmaker RH. Addition of omega-3 fatty acid to maintenance medication treatment for recurrent unipolar depressive disorder. American Journal of Psychiatry. 2002;159(3):477-9.

83.	Ning H, Zhou H, Ren J, Zhou G, Yang N, Wang Z, et al. Zishen pingchan granules combined with pramipexole in the improvement of depressive symptoms in Parkinson's disease: a prospective, multicenter, randomized, double-blind, controlled clinical study. J Transl Med. 2022;20(1):357.

84.	Noorbala AA, Akhondzadeh S, Tahmacebi-Pour N, Jamshidi AH. Hydro-alcoholic extract of Crocus sativus L. versus fluoxetine in the treatment of mild to moderate depression: A double-blind, randomized pilot trial. Journal of Ethnopharmacology. 2005;97(2):281-4.

85.	Nowak G, Siwek M, Dudek D, Zieba A, Pilc A. Effect of zinc supplementation on antidepressant therapy in unipolar depression: A preliminary placebo-controlled study. Polish Journal of Pharmacology. 2003;55(6):1143-7.

86.	Padhan M, Mohapatra D, Mishra BR, Maiti R, Jena M. Efficacy and safety of add-on sarcosine in patients with major depressive disorder: A randomized controlled trial. Journal of Psychiatric Research. 2024;178:298-304.

87.	Panahi Y, Badeli R, Karami GR, Badeli Z, Sahebkar A. A randomized controlled trial of 6-week Chlorella vulgaris supplementation in patients with major depressive disorder. Complement Ther Med. 2015;23(4):598-602.

88.	Pancheri P, Scapicchio P, Delle Chiaie R. A double-blind, randomized parallel-group, efficacy and safety study of intramuscular S-adenosyl-L-methionine 1, 4-butanedisulphonate (SAMe) versus imipramine in patients with major depressive disorder. International Journal of Neuropsychopharmacology. 2002;5(4):287-94.

89.	Papakostas GI, Mischoulon D, Shyu I, Alpert JE, Fava M. S-adenosyl methionine (SAMe) augmentation of serotonin reuptake inhibitors for antidepressant nonresponders with major depressive disorder: a double-blind, randomized clinical trial. 2010;167(8):942‐8

C3 - PUBMED 20595412.

90.	Papakostas GI, Shelton RC, Zajecka JM, Etemad B, Rickels K, Clain A, et al. L-methylfolate as adjunctive therapy for SSRI-resistant major depression: results of two randomized, double-blind, parallel-sequential trials. 2012;169(12):1267‐74

C3 - PUBMED 23212058.

91.	Park Y, Park YS, Kim SH, Oh DH, Park YC. Supplementation of n-3 polyunsaturated fatty acids for major depressive disorder: A randomized, double-blind, 12-week, placebo-controlled trial in Korea. Annals of Nutrition and Metabolism. 2015;66(2-3):141-8.

92.	Piperoglou M, Hopwood M, Norman TR. Adjunctive Docosahexaenoic Acid in Residual Symptoms of Depression and Anxiety. Journal of Clinical Psychopharmacology. 2023;43(6):493-7.

93.	Ranjbar E, Shams J, Sabetkasaei M, M-Shirazi M, Rashidkhani B, Mostafavi A, et al. Effects of zinc supplementation on efficacy of antidepressant therapy, inflammatory cytokines, and brain-derived neurotrophic factor in patients with major depression. Nutritional Neuroscience. 2014;17(2):65-71.

94.	Rees AM, Austin MP, Parker GB. Omega-3 fatty acids as a treatment for perinatal depression: Randomized double-blind placebo-controlled trial. Australian and New Zealand Journal of Psychiatry. 2008;42(3):199-205.

95.	Rondanelli M, Giacosa A, Opizzi A, Pelucchi C, Vecchia CL, Montorfano G, et al. Effect of omega-3 fatty acids supplementation on depressive symptoms and on health-related quality of life in the treatment of elderly women with depression: A double-blind, placebo-controlled, randomized clinical trial. Journal of the American College of Nutrition. 2010;29(1):55-64.

96.	Rudzki L, Ostrowska L, Pawlak D, Małus A, Pawlak K, Waszkiewicz N, et al. Probiotic Lactobacillus Plantarum 299v decreases kynurenine concentration and improves cognitive functions in patients with major depression: A double-blind, randomized, placebo controlled study. Psychoneuroendocrinology. 2019;100:213-22.

97.	Saccarello A, Montarsolo P, Massardo I, Picciotto R, Pedemonte A, Castagnaro R, et al. Oral Administration of S-Adenosylmethionine (SAMe) and Lactobacillus Plantarum HEAL9 Improves the Mild-To-Moderate Symptoms of Depression: a Randomized, Double-Blind, Placebo-Controlled Study. 2020;22(4 C3 - PUBMED 32589828,EMBASE 632201612).

98.	Sadeghi A, Ghorayshi F, Baghshahi H, Akbari H, Memarzadeh MR, Taghizadeh M, et al. The antidepressant effect of combined extracts of Hypericum perforatum and Echium amoenum supplementation in patients with depression symptoms: A randomized clinical trial. Avicenna J Phytomedicine. 2023;13(4):328-37.

99.	Sahoo JP, Jarnail S, Khurana H, Singh P. Omega-3 Fatty Acids Versus 5-hydroxytryptophan as Add on Therapy in Patients with Depression: A Comparative Study. Indian Journal of Pharmacology. 2015;47(7):S23-S4.

100.	Sakurai H, L. Carpenter L, R. Tyrka A, Price LH, I. Papakostas G, Dording CM, et al. Dose increase of S-Adenosyl-Methionine and escitalopram in a randomized clinical trial for major depressive disorder. Journal of Affective Disorders. 2020;262:118-25.

101.	Sanmukhani J, Satodia V, Trivedi J, Patel T, Tiwari D, Panchal B, et al. Efficacy and safety of curcumin in major depressive disorder: a randomized controlled trial. Phytother Res. 2014;28(4):579-85.

102.	Sarris J, Byrne GJ, Bousman C, Stough C, Murphy J, MacDonald P, et al. Adjunctive S-adenosylmethionine (SAMe) in treating non-remittent major depressive disorder: An 8-week double-blind, randomized, controlled trial. European Neuropsychopharmacology. 2018;28(10):1126-36.

103.	Sarris J, Byrne GJ, Stough C, Bousman C, Mischoulon D, Murphy J, et al. Nutraceuticals for major depressive disorder- more is not merrier: An 8-week double-blind, randomised, controlled trial. Journal of Affective Disorders. 2019;245:1007-15.

104.	Sarris J, Murphy J, Stough C, Mischoulon D, Bousman C, MacDonald P, et al. S-Adenosylmethionine (SAMe) monotherapy for depression: an 8-week double-blind, randomised, controlled trial. 2020;237(1):209‐18

C3 - PUBMED 31712971,EMBASE 2003633556.

105.	Schrader E. Equivalence of St John's wort extract (Ze 117) and fluoxetine: A randomized, controlled study in mild-moderate depression. International Clinical Psychopharmacology. 2000;15(2):61-8.

106.	Schrader E, Meier B, Brattström A. Hypericum treatment of mild-moderate depression in a placebo-controlled study. A prospective, double-blind, randomized, placebo-controlled, multicentre study. Human Psychopharmacology. 1998;13(3):163-9.

107.	Sempach L, Schaub A-C, Brühl AB, Sterzer P, Lang UE, Schmidt A. Adjunctive d-serine treatment for major depressive disorder: A randomized clinical trial. Journal of Affective Disorders. 2025:119504.

108.	Sepehrmanesh Z, Kolahdooz F, Abedi F, Mazroii N, Assarian A, Asemi Z, et al. Vitamin D supplementation affects the beck depression inventory, insulin resistance, and biomarkers of oxidative stress in patients with major depressive disorder: A randomized, controlled clinical trial. Journal of Nutrition. 2016;146(2):243-8.

109.	Shafiee-Kandjani A, Khalili M, Malek A, Farhang S, Ranjbari Y, Khalili Y. The therapeutic effect of the extracts of Lavandula angustifolia and Dracocephalum ruyschiana besides sertraline on patients with mild to moderate depression: A double-blind controlled trial. Phytomedicine Plus. 2023;3(2).

110.	Shahmansouri N, Farokhnia M, Abbasi SH, Kassaian SE, Noorbala Tafti AA, Gougol A, et al. A randomized, double-blind, clinical trial comparing the efficacy and safety of Crocus sativus L. with fluoxetine for improving mild to moderate depression in post percutaneous coronary intervention patients. 2014;155:216‐22

C3 - PUBMED 24289892,EMBASE 52891114.

111.	Shahmoradi N, Davarinejad O, Brühl AB, Brand S. Effects of Aphrodite (an Herbal Compound) on SSRI-Induced Sexual Dysfunctions and Depression in Females with Major Depressive Disorder: Findings from a Randomized Clinical Trial. Medicina (Kaunas, Lithuania). 2023;59(9).

112.	Shamabadi A, Fattollahzadeh-Noor S, Fallahpour B, A. Basti F, Khodaei Ardakani MR, Akhondzadeh S. L-Theanine adjunct to risperidone in the treatment of chronic schizophrenia inpatients: a randomized, double-blind, placebo-controlled clinical trial. Psychopharmacology. 2023;240(12):2631-40.

113.	Sharifan P, Hosseini MS, Sharifan A. The interventional relationship between frequent fish consumption and depression symptoms in aging adults: a randomized controlled trial. International Journal of Geriatric Psychiatry. 2017;32(12):e116-e22.

114.	Shelton RC, Keller MB, Gelenberg A, Dunner DL, Hirschfeld R, Thase ME, et al. Effectiveness of St John's wort in major depression: A randomized controlled trial. JAMA. 2001;285(15):1978-86.

115.	Sherwood Brown E, Gabrielson B. A randomized, double-blind, placebo-controlled trial of citicoline for bipolar and unipolar depression and methamphetamine dependence. Journal of Affective Disorders. 2012;143(1-3):257-60.

116.	Shinto L, Marracci G, Mohr DC, Bumgarner L, Murchison C, Senders A, et al. Omega-3 fatty acids for depression in multiple sclerosis: A randomized pilot study. PLoS ONE. 2016;11(1 C7 - e0147195).

117.	Su KP, Huang SY, Chiu CC, Shen WW. Omega-3 fatty acids in major depressive disorder: A preliminary double-blind, placebo-controlled trial. European Neuropsychopharmacology. 2003;13(4):267-71.

118.	Su KP, Huang SY, Chiu TH, Huang KC, Huang CL, Chang HC, et al. Omega-3 fatty acids for major depressive disorder during pregnancy: Results from a randomized, double-blind, placebo-controlled trial. Journal of Clinical Psychiatry. 2008;69(4):644-51.

119.	Sultana S, Rahman MS. Effect of magnesium supplementation in selective serotonin reuptake inhibitors treated major depressive disorder patients: A randomized, double-blind, placebo-controlled trial. EC Nutr. 2023;18:25-34.

120.	Szegedi A, Kohnen R, Dienel A, Kieser M. Acute treatment of moderate to severe depression with hypericum extract WS 5570 (St John's wort): Randomised controlled double blind non-inferiority trial versus paroxetine. British Medical Journal. 2005;330(7490):503-6.

121.	Talaei A, Noori R, Rezaei Ardani A, Mohammadpour AH, Azimipoor Z, Afzaljavan F. The Effect of Adding Curcumin to Sertraline in the Treatment of Severe Major Depressive Disorder: A Randomized, Double-Blind Clinical Trial. Clinical Neuropharmacology. 2023;46(4):135-9.

122.	Targum SD, Cameron BR, Ferreira L, MacDonald ID. An augmentation study of MSI-195 (S-adenosylmethionine) in Major Depressive Disorder. Journal of Psychiatric Research. 2018;107:86-96.

123.	Tarutani S, Omori M, Ido Y, Yano M, Komatsu T, Okamura T. Effects of 4G-beta-D-Galactosylsucrose in patients with depression: A randomized, double-blinded, placebo-controlled, parallel-group comparative study. J Psychiatr Res. 2022;148:110-20.

124.	Vaghef-Mehrabany E, Ranjbar F, Asghari-Jafarabadi M, Hosseinpour-Arjmand S, Ebrahimi-Mameghani M. Calorie restriction in combination with prebiotic supplementation in obese women with depression: effects on metabolic and clinical response. Nutritional Neuroscience. 2021;24(5):339-53.

125.	van Gurp G, Meterissian GB, Haiek LN, McCusker J, Bellavance F. St John's wort or sertraline? Randomized controlled trial in primary care. Can Fam Physician. 2002;48:905-12.

126.	Verma DK, Das B. Effect of zinc supplementation on antidepressant therapy and serum zinc level in unipolar depression: A randomized double blind controlled study. Indian Journal of Psychiatry. 2016;58(5):S47.

127.	Vorbach EU, Arnoldt KH, Hubner WD. Efficacy and tolerability of St. John's wort extract LI 160 versus imipramine in patients with severe depressive episodes according to ICD-10. Pharmacopsychiatry. 1997;30(SUPPL.):81-5.

128.	Wang L, Bordi PL, Fleming JA, Hill AM, Kris-Etherton PM. Effect of a moderate fat diet with and without avocados on lipoprotein particle number, size and subclasses in overweight and obese adults: a randomized, controlled trial. J Am Heart Assoc. 2015;4(1):e001355.

129.	Wheatley D. LI 160, an extract of St. John's wort, versus amitriptyline in mildly to moderately depressed outpatients--a controlled 6-week clinical trial. 1997;30 Suppl 2:77‐80

C3 - PUBMED 9342764.

130.	Woelk H. Comparison of St John's wort and imipramine for treating depression: Randomised controlled trial. British Medical Journal. 2000;321(7260):536-9.

131.	Wu SK, Yang KJ, Liu WC, Malau IA, Zailani H, Chang CH, et al. The Efficacy of Omega-3 Fatty Acids as the Monotherapy for Depression: A Randomized, Double-Blind, Placebo-Controlled Pilot Study. Nutrients. 2024;16(21).

132.	Zhang L, Wang S, Zhu Y, Yang T. Vitamin D3 as adjunctive therapy in the treatment of depression in tuberculosis patients: A short-term pilot randomized double-blind controlled study. Neuropsychiatric Disease and Treatment. 2018;14:3103-9.

133.	Firoozeei TS, Barekatain M, Karimi M, Zargaran A, Akhondzadeh S, Rezaeizadeh H. Lavender and dodder combined herbal syrup versus citalopram in major depressive disorder with anxious distress: A double-blind randomized trial. Journal of Integrative Medicine. 2020;18(5):409-15.

134.	Masoumi SZ, Kazemi F, Tavakolian S, Rahimi A, Oshvandi K, Soltanian A, et al. Effect of citalopram in combination with omega-3 on depression in post-menopausal women: A triple blind randomized controlled trial. Journal of Clinical and Diagnostic Research. 2016;10(10):QC01-QC5.

135.	Sepehrmanesh Z, Kolahdooz F, Abedi F, Mazroii N, Assarian A, Asemi Z, et al. Vitamin D Supplementation Affects the Beck Depression Inventory, Insulin Resistance, and Biomarkers of Oxidative Stress in Patients with Major Depressive Disorder: A Randomized, Controlled Clinical Trial [Retraction notice in CN-02298463]. 2016;146(2):243‐8

C3 - PUBMED 26609167,EMBASE 608794678.

136.	Ghaleiha A, Davari H, Jahangard L, Haghighi M, Ahmadpanah M, Seifrabie MA, et al. Adjuvant thiamine improved standard treatment in patients with major depressive disorder: results from a randomized, double-blind, and placebo-controlled clinical trial. European Archives of Psychiatry and Clinical Neuroscience. 2016;266(8):695-702.

137.	Ryszewska-Pokraśniewicz B, Mach A, Skalski M, Januszko P, Wawrzyniak ZM, Poleszak E, et al. Effects of magnesium supplementation on unipolar depression: A placebo-controlled study and review of the importance of dosing and magnesium status in the therapeutic response. Nutrients. 2018;10(8 C7 - 1014).

138.	Coppen A, Bailey J. Enhancement of the antidepressant action of fluoxetine by folic acid: A randomised, placebo controlled trial. Journal of Affective Disorders. 2000;60(2):121-30.

139.	Sahraian A, Ghanizadeh A, Kazemeini F. Vitamin C as an adjuvant for treating major depressive disorder and suicidal behavior, a randomized placebo-controlled clinical trial. Trials. 2015;16(1 C7 - 94).

140.	Roohi-Azizi M, Arabzadeh S, Amidfar M, Salimi S, Zarindast MR, Talaei A, et al. Citicoline combination therapy for major depressive disorder: A randomized, double-blind, placebo-controlled trial. Clinical Neuropharmacology. 2017;40(1):1-5.

141.	Jahangard L, Sadeghi A, Ahmadpanah M, Holsboer-Trachsler E, Sadeghi Bahmani D, Haghighi M, et al. Influence of adjuvant omega-3-polyunsaturated fatty acids on depression, sleep, and emotion regulation among outpatients with major depressive disorders - Results from a double-blind, randomized and placebo-controlled clinical trial. Journal of Psychiatric Research. 2018;107:48-56.

142.	Ramezani A, Kheirkhah F, Zabihi E, Shirafkan H, Moudi S. Effectiveness of L-carnitine Supplementation to Sertraline for Treatment of Major Depressive Disorder: A Double-blind Randomized Placebo-controlled Trial. Glob J Med Pharm Biomed Update. 2022;17.

143.	Vellekkatt F, Menon V, Rajappa M, Sahoo J. Effect of adjunctive single dose parenteral Vitamin D supplementation in major depressive disorder with concurrent vitamin D deficiency: A double-blind randomized placebo-controlled trial. J Psychiatr Res. 2020;129:250-6.

144.	Talaei A, Hassanpour Moghadam M, Sajadi Tabassi SA, Mohajeri SA. Crocin, the main active saffron constituent, as an adjunctive treatment in major depressive disorder: A randomized, double-blind, placebo-controlled, pilot clinical trial. Journal of Affective Disorders. 2015;174:51-6.

145.	Jelodar G, Javid Z, Sahraian A, Jelodar S. Saffron improved depression and reduced homocysteine level in patients with major depression: A Randomized, double-blind study. Avicenna J Phytomedicine. 2018;8(1):43-50.

146.	Ille R, Spona J, Zickl M, Hofmann P, Lahousen T, Dittrich N, et al. "Add-On"-therapy with an individualized preparation consisting of free amino acids for patients with a major depression. European Archives of Psychiatry and Clinical Neuroscience. 2007;257(4):222-9.

147.	Lyoo IK, Yoon S, Kim TS, Hwang J, Kim JE, Won W, et al. A randomized, double-blind placebo-controlled trial of oral creatine monohydrate augmentation for enhanced response to a selective serotonin reuptake inhibitor in women with major depressive disorder. American Journal of Psychiatry. 2012;169(9):937-45.

148.	Yu JJ, Pei LB, Zhang Y, Wen ZY, Yang JL. Chronic Supplementation of Curcumin Enhances the Efficacy of Antidepressants in Major Depressive Disorder: A Randomized, Double-Blind, Placebo-Controlled Pilot Study. Journal of Clinical Psychopharmacology. 2015;35(4):406-10.

149.	Yang R, Wang L, Jin K, Cao S, Wu C, Guo J, et al. Omega-3 Polyunsaturated Fatty Acids Supplementation Alleviate Anxiety Rather Than Depressive Symptoms Among First-Diagnosed, Drug-Naïve Major Depressive Disorder Patients: A Randomized Clinical Trial. Frontiers in Nutrition. 2022;9 C7 - 876152.

**e-table 4:** Efficacy node 2 subnetworks.

| **Author(s), year** | **Treatment** | **N** | **Subnetwork** |
| --- | --- | --- | --- |
| ^555^ | Aminoacids+Antidepressants | 22 | 1 |
| ^555^ | Antidepressants+PLB | 22 | 1 |
| ^525^ | Crassulaceae | 20 | 1 |
| ^525^ | SSRI | 19 | 1 |
| ^525^ | PLB | 18 | 1 |
| ^529^ | Coenzyme | 64 | 1 |
| ^529^ | SSRI | 65 | 1 |
| ^529^ | PLB | 60 | 1 |
| ^537^ | Coenzyme | 146 | 1 |
| ^537^ | TCA | 147 | 1 |
| ^498^ | Herbal compound | 82 | 1 |
| ^498^ | PLB | 82 | 1 |
| ^505^ | Coenzyme | 9 | 1 |
| ^505^ | PLB | 6 | 1 |
| ^387^ | LCPUFA | 12 | 1 |
| ^387^ | PLB | 10 | 1 |
| ^596^ | LCPUFA | 17 | 1 |
| ^596^ | PLB | 16 | 1 |
| ^490^ | LCPUFA | 15 | 1 |
| ^490^ | PLB | 16 | 1 |
| ^515^ | LCPUFA | 28 | 1 |
| ^515^ | PLB | 23 | 1 |
| ^597^ | LCPUFA | 13 | 1 |
| ^597^ | PLB | 13 | 1 |
| ^578^ | Lamiaceae | 33 | 1 |
| ^578^ | SSRI | 33 | 1 |
| ^577^ | Vitamins | 56 | 1 |
| ^577^ | PLB | 64 | 1 |
| ^551^ | LCPUFA | 30 | 1 |
| ^551^ | PLB | 30 | 1 |
| ^471^ | Coenzyme+Vitamins+LCPUFA+Minerals+Aminoacids | 56 | 1 |
| ^471^ | PLB | 57 | 1 |
| ^503^ | LCPUFA | 25 | 1 |
| ^503^ | PLB | 22 | 1 |
| ^524^ | Vitamins | 70 | 1 |
| ^524^ | PLB | 70 | 1 |
| ^539^ | Vitamins | 19 | 1 |
| ^539^ | PLB | 56 | 1 |
| ^539^ | Coenzyme+Antidepressants | 36 | 1 |
| ^539^ | Antidepressants+PLB | 33 | 1 |
| ^598^ | Vitamins | 36 | 1 |
| ^598^ | PLB | 112 | 1 |
| ^468^ | Coenzyme | 9 | 1 |
| ^468^ | TCA | 9 | 1 |
| ^552^ | Coenzyme | 22 | 1 |
| ^552^ | PLB | 19 | 1 |
| ^272^ | LCPUFA | 41 | 1 |
| ^272^ | PLB | 21 | 1 |
| ^473^ | LCPUFA | 12 | 1 |
| ^473^ | PLB | 12 | 1 |
| ^511^ | HYP | 68 | 1 |
| ^511^ | PLB | 24 | 1 |
| ^469^ | Zingiberaceae | 19 | 1 |
| ^469^ | PLB | 20 | 1 |
| ^532^ | Herbal compound | 89 | 1 |
| ^532^ | PLB | 87 | 1 |
| ^491^ | HYP | 126 | 1 |
| ^491^ | SSRI | 114 | 1 |
| ^545^ | Coenzyme+PROB | 46 | 1 |
| ^545^ | PLB | 44 | 1 |
| ^478^ | LCPUFA | 30 | 1 |
| ^478^ | PLB | 29 | 1 |
| ^518^ | HYP | 98 | 1 |
| ^518^ | PLB | 49 | 1 |
| ^547^ | SSRI+LCPUFA | 30 | 1 |
| ^547^ | SSRI+Aminoacids | 30 | 1 |
| ^547^ | SSRI | 30 | 1 |
| ^556^ | Vitamins | 18 | 1 |
| ^556^ | PLB | 18 | 1 |
| ^514^ | Vitamins+SSRI | 20 | 1 |
| ^514^ | SSRI | 20 | 1 |
| ^516^ | PROB | 28 | 1 |
| ^516^ | PLB | 29 | 1 |
| ^479^ | LCPUFA | 19 | 1 |
| ^479^ | PLB | 20 | 1 |
| ^484^ | Coenzyme | 19 | 1 |
| ^484^ | PLB | 17 | 1 |
| ^540^ | LCPUFA | 12 | 1 |
| ^540^ | PLB | 13 | 1 |
| ^546^ | Herbal compound+HYP | 23 | 1 |
| ^546^ | SSRI | 28 | 1 |
| ^510^ | HYP | 243 | 1 |
| ^510^ | PLB | 81 | 1 |
| ^568^ | SSRI+Zingiberaceae | 20 | 1 |
| ^568^ | SSRI+PLB | 20 | 1 |
| ^557^ | SSRI+Lamiaceae | 35 | 1 |
| ^557^ | SSRI+PLB | 35 | 1 |
| ^493^ | HYP | 43 | 1 |
| ^493^ | PLB | 55 | 1 |
| ^538^ | Coenzyme+Antidepressants | 39 | 1 |
| ^538^ | PLB+Antidepressants | 34 | 1 |
| ^455^ | LCPUFA | 33 | 1 |
| ^455^ | PLB | 17 | 1 |
| ^496^ | PROB | 20 | 1 |
| ^496^ | PLB | 20 | 1 |
| ^462^ | Vitamins+CA | 26 | 1 |
| ^462^ | Vitamins | 26 | 1 |
| ^462^ | PLB | 24 | 1 |
| ^584^ | Vitamins+SSRI | 51 | 1 |
| ^584^ | SSRI+PLB | 58 | 1 |
| ^553^ | HYP | 125 | 1 |
| ^553^ | SSRI | 113 | 1 |
| ^554^ | HYP | 80 | 1 |
| ^554^ | PLB | 79 | 1 |
| ^467^ | HYP | 35 | 1 |
| ^467^ | SSRI | 35 | 1 |
| ^472^ | HYP | 54 | 1 |
| ^472^ | SSRI | 54 | 1 |
| ^472^ | PLB | 55 | 1 |
| ^533^ | Iridaceae | 20 | 1 |
| ^533^ | SSRI | 20 | 1 |
| ^221^ | Zingiberaceae | 28 | 1 |
| ^221^ | PLB | 28 | 1 |
| ^470^ | Aminoacids+Antidepressants | 125 | 1 |
| ^470^ | Antidepressants+PLB | 127 | 1 |
| ^509^ | Iridaceae | 32 | 1 |
| ^509^ | SSRI | 32 | 1 |
| ^499^ | HYP | 70 | 1 |
| ^499^ | SSRI | 79 | 1 |
| ^530^ | Iridaceae | 20 | 1 |
| ^530^ | PLB | 20 | 1 |
| ^576^ | HYP | 157 | 1 |
| ^576^ | TCA | 167 | 1 |
| ^456^ | Iridaceae | 25 | 1 |
| ^456^ | SSRI | 25 | 1 |
| ^504^ | LCPUFA | 16 | 1 |
| ^504^ | SSRI | 16 | 1 |
| ^504^ | LCPUFA+SSRI | 16 | 1 |
| ^495^ | Iridaceae | 30 | 1 |
| ^495^ | SSRI | 30 | 1 |
| ^528^ | Vitamins | 159 | 1 |
| ^528^ | PLB | 123 | 1 |
| ^534^ | Minerals+Antidepressants | 6 | 1 |
| ^534^ | Antidepressants+PLB | 8 | 1 |
| ^580^ | SSRI+PLB | 30 | 1 |
| ^580^ | SSRI+LCPUFA | 30 | 1 |
| ^589^ | Vitamins+Antidepressants | 23 | 1 |
| ^589^ | Antidepressants+PLB | 23 | 1 |
| ^566^ | Minerals | 40 | 1 |
| ^566^ | PLB | 38 | 1 |
| ^483^ | HYP | 113 | 1 |
| ^483^ | SSRI | 109 | 1 |
| ^483^ | PLB | 116 | 1 |
| ^520^ | Minerals+Antidepressants | 30 | 1 |
| ^520^ | Antidepressants | 30 | 1 |
| ^571^ | Herbal compound | 194 | 1 |
| ^571^ | PLB | 191 | 1 |
| ^429^ | Polysaccarid | 31 | 1 |
| ^429^ | PLB | 31 | 1 |
| ^480^ | HYP | 83 | 1 |
| ^480^ | TCA | 73 | 1 |
| ^561^ | Iridaceae | 20 | 1 |
| ^561^ | SSRI | 20 | 1 |
| ^599^ | LCPUFA | 60 | 1 |
| ^599^ | PLB | 60 | 1 |
| ^476^ | SSRI+LCPUFA | 62 | 1 |
| ^476^ | SSRI+PLB | 60 | 1 |
| ^474^ | HYP | 13 | 1 |
| ^474^ | SSRI | 15 | 1 |
| ^461^ | PROB | 20 | 1 |
| ^461^ | PLB | 20 | 1 |
| ^463^ | Herbal compound | 21 | 1 |
| ^463^ | SSRI | 21 | 1 |
| ^586^ | Nucleotide+SSRI | 25 | 1 |
| ^586^ | PLB+SSRI | 25 | 1 |
| ^594^ | Zingiberaceae+SSRI | 50 | 1 |
| ^594^ | PLB+SSRI | 50 | 1 |
| ^477^ | Coenzyme | 30 | 1 |
| ^477^ | PLB | 29 | 1 |
| ^296^ | LCPUFA | 10 | 1 |
| ^296^ | PLB | 10 | 1 |
| ^487^ | Herbal compound | 42 | 1 |
| ^487^ | Iridaceae+Herbal compound | 48 | 1 |
| ^487^ | Iridaceae | 41 | 1 |
| ^487^ | PLB | 46 | 1 |
| ^492^ | LCPUFA | 14 | 1 |
| ^492^ | PLB | 9 | 1 |
| Palhano-Fontes F. et al., ^600^ | SSRI+Vitamins | 20 | 1 |
| Palhano-Fontes F. et al., ^600^ | SSRI | 15 | 1 |
| ^466^ | Vitamins+SSRI | 25 | 1 |
| ^466^ | SSRI+PLB | 26 | 1 |
| ^582^ | Coenzyme+Antidepressants | 37 | 1 |
| ^582^ | PLB+Antidepressants | 39 | 1 |
| ^550^ | Zingiberaceae | 33 | 1 |
| ^550^ | PLB | 32 | 1 |
| ^507^ | HYP | 122 | 1 |
| ^507^ | SSRI | 122 | 1 |
| ^567^ | Vitamins+SSRI | 45 | 1 |
| ^567^ | SSRI+PLB | 45 | 1 |
| ^601^ | HYP | 37 | 1 |
| ^601^ | PLB | 35 | 1 |
| ^602^ | Zingiberaceae | 16 | 1 |
| ^602^ | SSRI | 17 | 1 |
| ^602^ | Zingiberaceae+SSRI | 18 | 1 |
| ^549^ | Vitamins | 13 | 1 |
| ^549^ | PLB | 15 | 1 |
| ^519^ | Zingiberaceae+Iridaceae | 26 | 1 |
| ^519^ | PLB | 36 | 1 |
| ^523^ | HYP | 107 | 1 |
| ^523^ | TCA | 102 | 1 |
| ^574^ | SSRI | 23 | 1 |
| ^574^ | PLB | 26 | 1 |
| ^548^ | Aminoacids+Antidepressants | 30 | 1 |
| ^548^ | PLB+Antidepressants | 30 | 1 |
| ^535^ | Ginkgoaceae | 34 | 1 |
| ^535^ | PLB | 34 | 1 |
| ^500^ | Minerals+Antidepressants | 20 | 1 |
| ^500^ | PLB+Antidepressants | 17 | 1 |
| ^542^ | Vitamins | 28 | 1 |
| ^542^ | PLB | 28 | 1 |
| ^513^ | SSRI | 34 | 1 |
| ^513^ | PLB | 38 | 1 |
| ^522^ | Minerals+SSRI | 17 | 1 |
| ^522^ | PLB+SSRI | 15 | 1 |
| ^583^ | Coenzyme | 30 | 1 |
| ^583^ | PLB | 30 | 1 |
| ^485^ | Iridaceae+SSRI | 20 | 1 |
| ^485^ | SSRI+PLB | 20 | 1 |
| ^512^ | Vitamins | 212 | 1 |
| ^512^ | PLB | 204 | 1 |
| ^575^ | Minerals | 10 | 1 |
| ^575^ | PLB | 5 | 1 |
| ^482^ | HYP | 44 | 1 |
| ^482^ | SSRI | 43 | 1 |
| ^572^ | Herbal compound | 21 | 1 |
| ^572^ | PLB | 19 | 1 |
| ^559^ | Polysaccarid | 9 | 1 |
| ^559^ | PLB | 11 | 1 |
| ^570^ | PROB | 98 | 1 |
| ^570^ | PLB | 102 | 1 |
| ^588^ | Aminoacids+SSRI | 30 | 1 |
| ^588^ | SSRI+PLB | 30 | 1 |
| ^489^ | Lamiaceae | 15 | 1 |
| ^489^ | Convolvulaceae | 13 | 1 |
| ^489^ | Antidepressants | 15 | 1 |
| ^536^ | Microalgae | 42 | 1 |
| ^536^ | Antidepressants | 50 | 1 |
| ^558^ | Iridaceae | 20 | 1 |
| ^558^ | SSRI | 20 | 1 |
| ^531^ | Aminoacids | 4 | 1 |
| ^531^ | PLB | 4 | 1 |
| ^488^ | HYP | 45 | 1 |
| ^488^ | SSRI | 47 | 1 |
| ^488^ | PLB | 43 | 1 |
| ^486^ | Minerals | 70 | 1 |
| ^486^ | PLB | 40 | 1 |
| ^526^ | LCPUFA | 18 | 1 |
| ^526^ | PLB | 17 | 1 |
| ^254^ | LCPUFA | 16 | 1 |
| ^254^ | PLB | 19 | 1 |
| ^458^ | TCA | 15 | 1 |
| ^458^ | Iridaceae | 15 | 1 |
| ^521^ | PROB | 16 | 1 |
| ^521^ | PLB | 16 | 1 |
| ^585^ | Vitamins+SSRI | 21 | 1 |
| ^585^ | SSRI+PLB | 22 | 1 |
| ^585^ | Lamiaceae | 141 | 1 |
| ^585^ | PLB | 128 | 1 |
| ^544^ | PROB | 30 | 1 |
| ^544^ | PLB | 30 | 1 |
| ^465^ | PROB | 35 | 1 |
| ^465^ | PLB | 36 | 1 |
| ^460^ | Lamiaceae | 21 | 1 |
| ^460^ | PLB | 22 | 1 |
| ^460^ | Lamiaceae | 15 | 1 |
| ^460^ | TCA | 15 | 1 |
| ^460^ | Lamiaceae+TCA | 15 | 1 |
| ^579^ | Lamiaceae+PLB | 25 | 1 |
| ^579^ | SSRI+PLB | 25 | 1 |
| ^560^ | SSRI+Aminoacids | 25 | 1 |
| ^560^ | SSRI+PLB | 25 | 1 |
| ^587^ | LCPUFA+SSRI | 25 | 1 |
| ^587^ | SSRI+PLB | 25 | 1 |
| ^497^ | LCPUFA | 40 | 1 |
| ^497^ | PLB | 43 | 1 |
| ^508^ | Iridaceae | 28 | 1 |
| ^508^ | PLB | 28 | 1 |
| ^211^ | HYP | 186 | 1 |
| ^211^ | PLB | 189 | 1 |
| ^501^ | Polyphenols | 20 | 1 |
| ^501^ | PLB | 20 | 1 |
| ^543^ | LCPUFA | 22 | 1 |
| ^543^ | PLB | 24 | 1 |
| ^457^ | Iridaceae | 20 | 1 |
| ^457^ | SSRI | 20 | 1 |
| ^590^ | Iridaceae+Antidepressants | 20 | 1 |
| ^590^ | Antidepressants+PLB | 20 | 1 |
| ^481^ | Crassulaceae | 60 | 1 |
| ^481^ | PLB | 29 | 1 |
| ^494^ | SSRI+PLB | 23 | 1 |
| ^494^ | SSRI+Lauraceae | 19 | 1 |
| ^569^ | Coenzyme+Antidepressants | 118 | 1 |
| ^569^ | PLB+Antidepressants | 116 | 1 |
| ^502^ | Herbal compound | 63 | 1 |
| ^502^ | SSRI | 66 | 1 |
| ^517^ | Vitamins | 31 | 1 |
| ^517^ | PLB | 28 | 1 |
| ^563^ | Nucleotide | 28 | 1 |
| ^563^ | PLB | 20 | 1 |
| ^593^ | SSRI+Aminoacids | 25 | 1 |
| ^593^ | SSRI+PLB | 27 | 1 |
| ^475^ | SSRI+LCPUFA | 71 | 1 |
| ^475^ | SSRI+PLB | 73 | 1 |
| ^459^ | Iridaceae | 27 | 1 |
| ^459^ | PLB | 25 | 1 |
| ^464^ | Lamiaceae | 30 | 1 |
| ^464^ | SSRI | 15 | 1 |
| ^464^ | Iridaceae | 15 | 1 |
| ^527^ | Iridaceae | 30 | 1 |
| ^527^ | PLB | 30 | 1 |
| ^591^ | SSRI+Iridaceae | 20 | 2 |
| ^591^ | SSRI + PLB | 20 | 2 |
| ^592^ | Aminoacids+NASSA | 20 | 3 |
| ^592^ | MIR+PLB | 20 | 3 |
| ^603^ | LCPUFA+SNRI | 21 | 4 |
| ^603^ | PLB+SNRI | 27 | 4 |

**Reference for e-table 4**

1.	Mazidi M, Shemshian M, Mousavi SH, Norouzy A, Kermani T, Moghiman T, et al. A double-blind, randomized and placebo-controlled trial of Saffron (Crocus sativus L.) in the treatment of anxiety and depression. 2016;13(2):195‐9

C3 - PUBMED 27101556,EMBASE 610483123.

2.	Araj-Khodaei M, Noorbala AA, Yarani R, Emadi F, Emaratkar E, Faghihzadeh S, et al. A double-blind, randomized pilot study for comparison of Melissa officinalis L. And Lavandula angustifolia Mill. And Fluoxetine for the treatment of depression. BMC Complementary Medicine and Therapies. 2020;20(1).

3.	Akhondzadeh S, Mostafavi SA, Keshavarz SA, Mohammadi MR, Hosseini S, Eshraghian MR. A placebo controlled randomized clinical trial of Crocus sativus L. (saffron) on depression and food craving among overweight women with mild to moderate depression. Journal of Clinical Pharmacy and Therapeutics. 2020;45(1):134-43.

4.	Carney RM, Freedland KE, Rubin EH, Rich MW, Steinmeyer BC, Harris WS. A randomized placebo-controlled trial of omega-3 and sertraline in depressed patients with or at risk for coronary heart disease. Journal of Clinical Psychiatry. 2019;80(4 C7 - 19m12742).

5.	Lyoo IK, Yoon S, Kim TS, Hwang J, Kim JE, Won W, et al. A randomized, double-blind placebo-controlled trial of oral creatine monohydrate augmentation for enhanced response to a selective serotonin reuptake inhibitor in women with major depressive disorder. American Journal of Psychiatry. 2012;169(9):937-45.

6.	Sherwood Brown E, Gabrielson B. A randomized, double-blind, placebo-controlled trial of citicoline for bipolar and unipolar depression and methamphetamine dependence. Journal of Affective Disorders. 2012;143(1-3):257-60.

7.	Kumar PNS, Menon V, Andrade C. A randomized, double-blind, placebo-controlled, 12-week trial of vitamin D augmentation in major depressive disorder associated with vitamin D deficiency. Journal of Affective Disorders. 2022;314:143-9.

8.	Hu Y, Chen C, Wang Y, Yang W, Wang Y, Zhu W, et al. The effects of KaiXinSan on depression and its association with lipid profiles: a randomized, double-blinded, placebo-controlled trial. 2021;83:153467

C3 - PUBMED 33516143,EMBASE 2010802711.

9.	Targum SD, Cameron BR, Ferreira L, MacDonald ID. An augmentation study of MSI-195 (S-adenosylmethionine) in Major Depressive Disorder. Journal of Psychiatric Research. 2018;107:86-96.

10.	Ghaffari S, Ghobadi A, Jamshidi AH, Mortazavi SH, Naderi S, Aqamolaei A, et al. Cinnamomum tamala as an adjuvant therapy in the treatment of major depressive disorder: A double-blind, randomized, placebo-controlled clinical trial with placebo control. Advances in Integrative Medicine. 2020;7(3):141-7.

11.	Darbinyan V, Aslanyan G, Amroyan E, Gabrielyan E, Malmström C, Panossian A. Clinical trial of Rhodiola rosea L. extract SHR-5 in the treatment of mild to moderate depression. Nordic Journal of Psychiatry. 2007;61(5):343-8.

12.	Talaei A, Hassanpour Moghadam M, Sajadi Tabassi SA, Mohajeri SA. Crocin, the main active saffron constituent, as an adjunctive treatment in major depressive disorder: A randomized, double-blind, placebo-controlled, pilot clinical trial. Journal of Affective Disorders. 2015;174:51-6.

13.	Akhondzadeh Basti A, Moshiri E, Noorbala AA, Jamshidi AH, Abbasi SH, Akhondzadeh S. Comparison of petal of Crocus sativus L. and fluoxetine in the treatment of depressed outpatients: A pilot double-blind randomized trial. Progress in Neuro-Psychopharmacology and Biological Psychiatry. 2007;31(2):439-42.

14.	Rondanelli M, Giacosa A, Opizzi A, Pelucchi C, Vecchia CL, Montorfano G, et al. Effect of omega-3 fatty acids supplementation on depressive symptoms and on health-related quality of life in the treatment of elderly women with depression: A double-blind, placebo-controlled, randomized clinical trial. Journal of the American College of Nutrition. 2010;29(1):55-64.

15.	Hosseini H, Bagherniya M, Sahebkar A, Iraj B, Majeed M, Askari G. The effect of curcumin-piperine supplementation on lipid profile, glycemic index, inflammation, and blood pressure in patients with type 2 diabetes mellitus and hypertriglyceridemia. Phytotherapy Research. 2024;38(11):5150-61.

16.	Lecrubier Y, Clerc G, Didi R, Kieser M. Efficacy of St. John's wort extract WS 5570 in major depression: A double-blind, placebo-controlled trial. American Journal of Psychiatry. 2002;159(8):1361-6.

17.	Kashani L, Esalatmanesh S, Eftekhari F, Salimi S, Foroughifar T, Etesam F, et al. Efficacy of Crocus sativus (saffron) in treatment of major depressive disorder associated with post-menopausal hot flashes: a double-blind, randomized, placebo-controlled trial. Arch Gynecol Obstet. 2018;297(3):717-24.

18.	Grenyer BF, Crowe T, Meyer B, Owen AJ, Grigonis-Deane EM, Caputi P, et al. Fish oil supplementation in the treatment of major depression: a randomised double-blind placebo-controlled trial. 2007;31(7):1393‐6

C3 - PUBMED 17659823.

19.	Jahangard L, Sadeghi A, Ahmadpanah M, Holsboer-Trachsler E, Sadeghi Bahmani D, Haghighi M, et al. Influence of adjuvant omega-3-polyunsaturated fatty acids on depression, sleep, and emotion regulation among outpatients with major depressive disorders - Results from a double-blind, randomized and placebo-controlled clinical trial. Journal of Psychiatric Research. 2018;107:48-56.

20.	Shamabadi A, Fattollahzadeh-Noor S, Fallahpour B, A. Basti F, Khodaei Ardakani MR, Akhondzadeh S. L-Theanine adjunct to risperidone in the treatment of chronic schizophrenia inpatients: a randomized, double-blind, placebo-controlled clinical trial. Psychopharmacology. 2023;240(12):2631-40.

21.	Firoozeei TS, Barekatain M, Karimi M, Zargaran A, Akhondzadeh S, Rezaeizadeh H. Lavender and dodder combined herbal syrup versus citalopram in major depressive disorder with anxious distress: A double-blind randomized trial. Journal of Integrative Medicine. 2020;18(5):409-15.

22.	Akhondzadeh S, Mostafavi SA, Keshavarz SA, Mohammadi MR, Chamari M. Melatonin Effects in Women With Comorbidities of Overweight, Depression, and Sleep Disturbance: A Randomized Placebo Controlled Clinical Trial. Sleep Med Res. 2022;13(1):22-30.

23.	Baião R, Capitão LP, Higgins C, Browning M, Harmer CJ, Burnet PWJ. Multispecies probiotic administration reduces emotional salience and improves mood in subjects with moderate depression: a randomised, double-blind, placebo-controlled study. Psychological medicine. 2023;53(8):3437-47.

24.	Rudzki L, Ostrowska L, Pawlak D, Małus A, Pawlak K, Waszkiewicz N, et al. Probiotic Lactobacillus Plantarum 299v decreases kynurenine concentration and improves cognitive functions in patients with major depression: A double-blind, randomized, placebo controlled study. Psychoneuroendocrinology. 2019;100:213-22.

25.	Sahraian A, Ghanizadeh A, Kazemeini F. Vitamin C as an adjuvant for treating major depressive disorder and suicidal behavior, a randomized placebo-controlled clinical trial. Trials. 2015;16(1 C7 - 94).

26.	Ille R, Spona J, Zickl M, Hofmann P, Lahousen T, Dittrich N, et al. "Add-On"-therapy with an individualized preparation consisting of free amino acids for patients with a major depression. European Archives of Psychiatry and Clinical Neuroscience. 2007;257(4):222-9.

27.	Lin SKK, Kuo PH, Hsu CY, Chiu YH, Chen CH. The effects of Lactobacillus plantarum PS128 in patients with major depressive disorder: an eight-week double-blind, placebo-controlled study. Asian Journal of Psychiatry. 2024;101 C7 - 104210.

28.	Akhondzadeh S, Fallah-Pour H, Afkham K, Jamshidi AH, Khalighi-Cigaroudi F. Comparison of Crocus sativus L. and imipramine in the treatment of mild to moderate depression: A pilot double-blind randomized trial [ISRCTN45683816]. BMC Complementary and Alternative Medicine. 2004;4.

29.	Mischoulon D, Best-Popescu C, Laposata M, Merens W, Murakami JL, Wu SL, et al. A double-blind dose-finding pilot study of docosahexaenoic acid (DHA) for major depressive disorder. European Neuropsychopharmacology. 2008;18(9):639-45.

30.	Marangell LB, Martinez JM, Zboyan HA, Kertz B, Kim HFS, Puryear LJ. A double-blind, placebo-controlled study of the omega-3 fatty acid docosahexaenoic acid in the treatment of major depression. American Journal of Psychiatry. 2003;160(5):996-8.

31.	Docherty JP, Sack DA, Roffman M, Finch M, Komorowski JR. A double-blind, placebo-controlled, exploratory trial of chromium picolinate in atypical depression: Effect on carbohydrate craving. Journal of Psychiatric Practice. 2005;11(5):302-14.

32.	Fava M, Alpert J, Nierenberg AA, Mischoulon D, Otto MW, Zajecka J, et al. A double-blind, randomized trial of St John's wort, fluoxetine, and placebo in major depressive disorder. Journal of Clinical Psychopharmacology. 2005;25(5):441-7.

33.	Nemets B, Levine J. A pilot dose-finding clinical trial of creatine monohydrate augmentation to SSRIs/SNRIs/NASA antidepressant treatment in major depression. International Clinical Psychopharmacology. 2013;28(3):127-33.

34.	Shahmansouri N, Farokhnia M, Abbasi SH, Kassaian SE, Noorbala Tafti AA, Gougol A, et al. A randomized, double-blind, clinical trial comparing the efficacy and safety of Crocus sativus L. with fluoxetine for improving mild to moderate depression in post percutaneous coronary intervention patients. 2014;155:216‐22

C3 - PUBMED 24289892,EMBASE 52891114.

35.	Panahi Y, Badeli R, Karami GR, Badeli Z, Sahebkar A. A randomized controlled trial of 6-week Chlorella vulgaris supplementation in patients with major depressive disorder. Complement Ther Med. 2015;23(4):598-602.

36.	Firoozabadi A, Zarshenas MM, Salehi A, Jahanbin S, Mohagheghzadeh A. Effectiveness of Cuscuta planiflora Ten. and Nepeta menthoides Boiss. & Buhse in major depression: a triple-blind randomized controlled trial study. J Evid Based Complementary Altern Med. 2015;20(2):94-7.

37.	Ramezani A, Kheirkhah F, Zabihi E, Shirafkan H, Moudi S. Effectiveness of L-carnitine Supplementation to Sertraline for Treatment of Major Depressive Disorder: A Double-blind Randomized Placebo-controlled Trial. Glob J Med Pharm Biomed Update. 2022;17.

38.	Tarutani S, Omori M, Ido Y, Yano M, Komatsu T, Okamura T. Effects of 4G-beta-D-Galactosylsucrose in patients with depression: A randomized, double-blinded, placebo-controlled, parallel-group comparative study. J Psychiatr Res. 2022;148:110-20.

39.	Shahmoradi N, Davarinejad O, Brühl AB, Brand S. Effects of Aphrodite (an Herbal Compound) on SSRI-Induced Sexual Dysfunctions and Depression in Females with Major Depressive Disorder: Findings from a Randomized Clinical Trial. Medicina (Kaunas, Lithuania). 2023;59(9).

40.	van Gurp G, Meterissian GB, Haiek LN, McCusker J, Bellavance F. St John's wort or sertraline? Randomized controlled trial in primary care. Can Fam Physician. 2002;48:905-12.

41.	Davidson JR, Gadde KM, Fairbank JA, Krishnan KRR, Califf RM, Binanay C, et al. Effect of Hypericum perforatum (St John's wort) in major depressive disorder: a randomized controlled trial. 2002;287(14):1807‐14

C3 - PUBMED 11939866,EMBASE 34303625.

42.	Wang L, Bordi PL, Fleming JA, Hill AM, Kris-Etherton PM. Effect of a moderate fat diet with and without avocados on lipoprotein particle number, size and subclasses in overweight and obese adults: a randomized, controlled trial. J Am Heart Assoc. 2015;4(1):e001355.

43.	Kasper S, Anghelescu I, Dienel A. Efficacy of orally administered Silexan in patients with anxiety-related restlessness and disturbed sleep--A randomized, placebo-controlled trial. 2015;25(11):1960‐7

C3 - PUBMED 26293583,EMBASE 605643089.

44.	Djokic G, Korcok D, Djordjevic V, Agic A, Rankovic A, Djukic Dejanovic S. The effects of s-adenosyl-L-methionine-vitamin b complex on mild and moderate depressive symptoms. Hippokratia. 2017;21(3):140-3.

45.	Ryszewska-Pokraśniewicz B, Mach A, Skalski M, Januszko P, Wawrzyniak ZM, Poleszak E, et al. Effects of magnesium supplementation on unipolar depression: A placebo-controlled study and review of the importance of dosing and magnesium status in the therapeutic response. Nutrients. 2018;10(8 C7 - 1014).

46.	Lin CH, Wang SH, Lane HY. Effects of Sodium Benzoate, a D-Amino Acid Oxidase Inhibitor, on Perceived Stress and Cognitive Function Among Patients With Late-Life Depression: A Randomized, Double-Blind, Sertraline- and Placebo-Controlled Trial. International Journal of Neuropsychopharmacology. 2022;25(7):545-55.

47.	Kaviani M, Nikooyeh B, Etesam F, Behnagh SJ, Kangarani HM, Arefi M, et al. Effects of vitamin D supplementation on depression and some selected pro-inflammatory biomarkers: a double-blind randomized clinical trial. BMC Psychiatry. 2022;22(1 C7 - 694).

48.	Ranjbar E, Shams J, Sabetkasaei M, M-Shirazi M, Rashidkhani B, Mostafavi A, et al. Effects of zinc supplementation on efficacy of antidepressant therapy, inflammatory cytokines, and brain-derived neurotrophic factor in patients with major depression. Nutritional Neuroscience. 2014;17(2):65-71.

49.	He X, Liu D, Ni S, Li Z, Li S, Wu T, et al. Efficacy and safety evaluation of Ginkgo biloba dropping pill (GBDP) on stable angina pectoris complicated with depression: A placebo-controlled, randomized, double-blind, multicenter study. Phytomedicine. 2024;126.

50.	Padhan M, Mohapatra D, Mishra BR, Maiti R, Jena M. Efficacy and safety of add-on sarcosine in patients with major depressive disorder: A randomized controlled trial. Journal of Psychiatric Research. 2024;178:298-304.

51.	Sakurai H, L. Carpenter L, R. Tyrka A, Price LH, I. Papakostas G, Dording CM, et al. Dose increase of S-Adenosyl-Methionine and escitalopram in a randomized clinical trial for major depressive disorder. Journal of Affective Disorders. 2020;262:118-25.

52.	Vorbach EU, Arnoldt KH, Hubner WD. Efficacy and tolerability of St. John's wort extract LI 160 versus imipramine in patients with severe depressive episodes according to ICD-10. Pharmacopsychiatry. 1997;30(SUPPL.):81-5.

53.	Lopresti AL, Drummond PD. Efficacy of curcumin, and a saffron/curcumin combination for the treatment of major depression: A randomised, double-blind, placebo-controlled study. Journal of Affective Disorders. 2017;207:188-96.

54.	Levine J, Barak Y, Gonzalves M, Szor H, Elizur A, Kofman O, et al. Double-blind, controlled trial of inositol treatment of depression. American Journal of Psychiatry. 1995;152(5):792-4.

55.	Sanmukhani J, Satodia V, Trivedi J, Patel T, Tiwari D, Panchal B, et al. Efficacy and safety of curcumin in major depressive disorder: a randomized controlled trial. Phytother Res. 2014;28(4):579-85.

56.	Gul A, Memtily N, Mijit P, Imin M, Rui H, Wushuer P, et al. The Üstikuddus sherbiti in the treatment of abnormal cold and dry-type depression patients with comorbid anxiety: A randomized and controlled clinical trial. Trad Med Mod Med. 2019;2(3):127-33.

57.	Wu R, Zhu D, Xia Y, Wang H, Tao W, Xue W, et al. A role of Yueju in fast-onset antidepressant action on major depressive disorder and serum BDNF expression: A randomly double-blind, fluoxetine-adjunct, placebo-controlled, pilot clinical study. Neuropsychiatric Disease and Treatment. 2015;11:2013-21.

58.	Szegedi A, Kohnen R, Dienel A, Kieser M. Acute treatment of moderate to severe depression with hypericum extract WS 5570 (St John's wort): Randomised controlled double blind non-inferiority trial versus paroxetine. British Medical Journal. 2005;330(7490):503-6.

59.	Kanchanatawan B, Tangwongchai S, Sughondhabhirom A, Suppapitiporn S, Hemrunrojn S, Carvalho AF, et al. Add-on Treatment with Curcumin Has Antidepressive Effects in Thai Patients with Major Depression: Results of a Randomized Double-Blind Placebo-Controlled Study. Neurotoxic Res. 2018;33(3):621-33.

60.	Sarris J, Byrne GJ, Bousman C, Stough C, Murphy J, MacDonald P, et al. Adjunctive S-adenosylmethionine (SAMe) in treating non-remittent major depressive disorder: An 8-week double-blind, randomized, controlled trial. European Neuropsychopharmacology. 2018;28(10):1126-36.

61.	Ghaleiha A, Davari H, Jahangard L, Haghighi M, Ahmadpanah M, Seifrabie MA, et al. Adjuvant thiamine improved standard treatment in patients with major depressive disorder: results from a randomized, double-blind, and placebo-controlled clinical trial. European Archives of Psychiatry and Clinical Neuroscience. 2016;266(8):695-702.

62.	Basoglu C, Ates MA, Algul A, Ipcioglu OM, Gecici O, Yilmaz O, et al. Adjuvant folate with escitalopram treatment and homocystein, folate, vitamin B-12 levels in patients with major depressive disorder. 2009;19(2):135‐42

C3 - EMBASE 354832073.

63.	Antidepressant Effects of Ayahuasca: a Randomized Placebo Controlled Trial in Treatment Resistant Depression. clinicaltrialsgov2016.

64.	Gao L, Wu C, Liao Y, Wang J. Antidepressants effects of Rhodiola capsule combined with sertraline for major depressive disorder: A randomized double-blind placebo-controlled clinical trial. Journal of Affective Disorders. 2020;265:99-103.

65.	Dormal V, Jonniaux L, Buchet M, Simar L, Copine S, Deldicque L. Effect of Hydroponically Grown Red Panax Ginseng on Perceived Stress Level, Emotional Processing, and Cognitive Functions in Moderately Stressed Adults: A Randomized, Double-Blind, Placebo-Controlled Study. Nutrients. 2025;17(6 C7 - 955).

66.	Nemets B, Stahl Z, Belmaker RH. Addition of omega-3 fatty acid to maintenance medication treatment for recurrent unipolar depressive disorder. American Journal of Psychiatry. 2002;159(3):477-9.

67.	Caruso I, Fumagalli M, Boccassini L, Puttini SP, Santandrea S, Giniselli G, et al. Treatment of depression in rheumatoid arthritic patients. A comparison of S-adenosylmethionine (Samyr) and placebo in a double-blind study. CLIN TRIALS J. 1987;24(4):305-10.

68.	Yu JJ, Pei LB, Zhang Y, Wen ZY, Yang JL. Chronic Supplementation of Curcumin Enhances the Efficacy of Antidepressants in Major Depressive Disorder: A Randomized, Double-Blind, Placebo-Controlled Pilot Study. Journal of Clinical Psychopharmacology. 2015;35(4):406-10.

69.	Roohi-Azizi M, Arabzadeh S, Amidfar M, Salimi S, Zarindast MR, Talaei A, et al. Citicoline combination therapy for major depressive disorder: A randomized, double-blind, placebo-controlled trial. Clinical Neuropharmacology. 2017;40(1):1-5.

70.	Anushiravani M, Manteghi AA, Taghipur A, Eslami M. Comparing Effectiveness of a Combined Herbal Drug Based on Echium Amoenum with Citalopram in the Treatment of Major Depressive Disorder. Curr Drug Discov Technol. 2019;16(2):232-8.

71.	Akkasheh G, Kashani-Poor Z, Tajabadi-Ebrahimi M, Jafari P, Akbari H, Taghizadeh M, et al. Clinical and metabolic response to probiotic administration in patients with major depressive disorder: A randomized, double-blind, placebo-controlled trial. Nutrition. 2016;32(3):315-20.

72.	Brenner R, Azbel V, Madhusoodanan S, Pawlowska M. Comparison of an extract of hypericum (LI 160) and sertraline in the treatment of depression: A double-blind, randomized pilot study. Clinical Therapeutics. 2000;22(4):411-9.

73.	Carney RM, Steinmeyer BC, Freedland KE, Rubin EH, Rich MW, Harris WS. Baseline blood levels of omega-3 and depression remission: A secondary analysis of data from a placebo-controlled trial of omega-3 supplements. Journal of Clinical Psychiatry. 2016;77(2):e138-e43.

74.	Almeida OP, Ford AH, Hirani V, Singh V, VanBockxmeer FM, McCaul K, et al. B vitamins to enhance treatment response to antidepressants in middle-aged and older adults: Results from the B-VITAGE randomised, double-blind, placebo-controlled trial. British Journal of Psychiatry. 2014;205(6):450-7.

75.	Sharifan P, Hosseini MS, Sharifan A. The interventional relationship between frequent fish consumption and depression symptoms in aging adults: a randomized controlled trial. International Journal of Geriatric Psychiatry. 2017;32(12):e116-e22.

76.	Chopra A, Kalyani AK. Comparison of efficacy of Crocus sativus L. with fluoxetine for improving depression among patients with cardiovascular diseases. Int J Life Sci Biotechnol Pharma Res. 2020;9(2):151-4.

77.	Wheatley D. LI 160, an extract of St. John's wort, versus amitriptyline in mildly to moderately depressed outpatients--a controlled 6-week clinical trial. 1997;30 Suppl 2:77‐80

C3 - PUBMED 9342764.

78.	Vaghef-Mehrabany E, Ranjbar F, Asghari-Jafarabadi M, Hosseinpour-Arjmand S, Ebrahimi-Mameghani M. Calorie restriction in combination with prebiotic supplementation in obese women with depression: effects on metabolic and clinical response. Nutritional Neuroscience. 2021;24(5):339-53.

79.	Li Y, Li L, Guo R, Yu H, Wang X, Wang B, et al. Clinical efficacy of Shugan granule in the treatment of mixed anxiety-depressive disorder: a multicenter, randomized, double-blind, placebo-controlled trial. 2022;290:115032

C3 - PUBMED 35085742,EMBASE 637131287.

80.	Davidson JRT, Gadde KM, Fairbank JA, Ranga Rama Krishnan K, Califf RM, Binanay C, et al. Effect of Hypericum perforatum (St John's wort) in major depressive disorder: A randomized controlled trial. JAMA. 2002;287(14):1807-14.

81.	Sultana S, Rahman MS. Effect of magnesium supplementation in selective serotonin reuptake inhibitors treated major depressive disorder patients: A randomized, double-blind, placebo-controlled trial. EC Nutr. 2023;18:25-34.

82.	Vellekkatt F, Menon V, Rajappa M, Sahoo J. Effect of adjunctive single dose parenteral Vitamin D supplementation in major depressive disorder with concurrent vitamin D deficiency: A double-blind randomized placebo-controlled trial. J Psychiatr Res. 2020;129:250-6.

83.	Masoumi SZ, Kazemi F, Tavakolian S, Rahimi A, Oshvandi K, Soltanian A, et al. Effect of citalopram in combination with omega-3 on depression in post-menopausal women: A triple blind randomized controlled trial. Journal of Clinical and Diagnostic Research. 2016;10(10):QC01-QC5.

84.	Nowak G, Siwek M, Dudek D, Zieba A, Pilc A. Effect of zinc supplementation on antidepressant therapy in unipolar depression: A preliminary placebo-controlled study. Polish Journal of Pharmacology. 2003;55(6):1143-7.

85.	Mech AW, Farah A. Correlation of clinical response with homocysteine reduction during therapy with reduced b vitamins in patients with mdd who are positive for MTHFR C677T or A1298C polymorphism: A Randomized, double-blind, placebo-controlled study. Journal of Clinical Psychiatry. 2016;77(5):668-71.

86.	Ghajar A, Neishabouri SM, Velayati N, Jahangard L, Matinnia N, Haghighi M, et al. Crocus sativus L versus Citalopram in the Treatment of Major Depressive Disorder with Anxious Distress: A Double-Blind, Controlled Clinical Trial. Pharmacopsychiatry. 2017;50(4):152-60.

87.	Jazayeri S, Tehrani-Doost M, Keshavarz SA, Hosseini M, Djazayery A, Amini H, et al. Comparison of therapeutic effects of omega-3 fatty acid eicosapentaenoic acid and fluoxetine, separately and in combination, in major depressive disorder. Australian and New Zealand Journal of Psychiatry. 2008;42(3):192-8.

88.	Ahmadpanah M, Ramezanshams F, Ghaleiha A, Akhondzadeh S, Sadeghi Bahmani D, Brand S. Crocus Sativus L. (saffron) versus sertraline on symptoms of depression among older people with major depressive disorders–a double-blind, randomized intervention study. Psychiatry Research. 2019;282.

89.	Woelk H. Comparison of St John's wort and imipramine for treating depression: Randomised controlled trial. British Medical Journal. 2000;321(7260):536-9.

90.	Moshiri E, Basti AA, Noorbala AA, Jamshidi AH, Hesameddin Abbasi S, Akhondzadeh S. Crocus sativus L. (petal) in the treatment of mild-to-moderate depression: A double-blind, randomized and placebo-controlled trial. Phytomedicine. 2006;13(9-10):607-11.

91.	Harrer G, Schmidt U, Kuhn U, Biller A. Comparison of equivalence between the St. John's wort extract LoHyp-57 and fluoxetine. Arzneimittel-Forschung/Drug Research. 1999;49(4):289-96.

92.	Kashani L, Eslatmanesh S, Saedi N, Niroomand N, Ebrahimi M, Hosseinian M, et al. Comparison of Saffron versus Fluoxetine in Treatment of Mild to Moderate Postpartum Depression: A Double-Blind, Randomized Clinical Trial. Pharmacopsychiatry. 2017;50(2):64-8.

93.	Berk M, Dean OM, Cotton SM, Jeavons S, Tanious M, Kohlmann K, et al. The efficacy of adjunctive N-acetylcysteine in major depressive disorder: A double-blind, randomized, placebo-controlled trial. Journal of Clinical Psychiatry. 2014;75(6):628-36.

94.	Lopresti AL, Maes M, Maker GL, Hood SD, Drummond PD. Curcumin for the treatment of major depression: A randomised, double-blind, placebo controlled study. Journal of Affective Disorders. 2014;167:368-75.

95.	Noorbala AA, Akhondzadeh S, Tahmacebi-Pour N, Jamshidi AH. Hydro-alcoholic extract of Crocus sativus L. versus fluoxetine in the treatment of mild to moderate depression: A double-blind, randomized pilot trial. Journal of Ethnopharmacology. 2005;97(2):281-4.

96.	Bjerkenstedt L, Edman GV, Alken RG, Mannel M. Hypericum extract LI 160 and fluoxetine in mild to moderate depression: A randomized, placebo-controlled multi-center study in outpatients. European Archives of Psychiatry and Clinical Neuroscience. 2005;255(1):40-7.

97.	Behnke K, Jensen GS, Graubaum HJ, Gruenwald J. Hypericum perforatum versus fluoxetine in the treatment of mild to moderate depression. Advances in Therapy. 2002;19(1):43-52.

98.	Schrader E, Meier B, Brattström A. Hypericum treatment of mild-moderate depression in a placebo-controlled study. A prospective, double-blind, randomized, placebo-controlled, multicentre study. Human Psychopharmacology. 1998;13(3):163-9.

99.	Schrader E. Equivalence of St John's wort extract (Ze 117) and fluoxetine: A randomized, controlled study in mild-moderate depression. International Clinical Psychopharmacology. 2000;15(2):61-8.

100.	Coppen A, Bailey J. Enhancement of the antidepressant action of fluoxetine by folic acid: A randomised, placebo controlled trial. Journal of Affective Disorders. 2000;60(2):121-30.

101.	Amini L, Yaghini O, Ghazavi M, Aslani N. L-carnitine versus Propranolol for pediatric migraine prophylaxis. Iran J Child Neurol. 2021;15(2):77-86.

102.	Ghorbani Z, Nazari S, Etesam F, Nourimajd S, Ahmadpanah M, Jahromi SR. The effect of synbiotic as an adjuvant therapy to fluoxetine in moderate depression: A randomized multicenter trial. Arch Neurosci. 2018;5(2).

103.	Açik M, Çakiroğlu FP, Tekin A, Egeli A. The effect of fish oil versus krill oil intervention on clinical symptoms and cardiometabolic risk factors in patients with major depressive disorder: A randomized placebo-controlled double-blind trial. Journal of Affective Disorders. 2025;380:171-80.

104.	Papakostas GI, Mischoulon D, Shyu I, Alpert JE, Fava M. S-adenosyl methionine (SAMe) augmentation of serotonin reuptake inhibitors for antidepressant nonresponders with major depressive disorder: a double-blind, randomized clinical trial. 2010;167(8):942‐8

C3 - PUBMED 20595412.

105.	Gelenberg AJ, Shelton RC, Crits-Christoph P, Keller MB, Dunner DL, Hirschfeld RM, et al. The effectiveness of St. John's Wort in major depressive disorder: a naturalistic phase 2 follow-up in which nonresponders were provided alternate medication. 2004;65(8):1114‐9

C3 - PUBMED 15323598.

106.	Shafiee-Kandjani A, Khalili M, Malek A, Farhang S, Ranjbari Y, Khalili Y. The therapeutic effect of the extracts of Lavandula angustifolia and Dracocephalum ruyschiana besides sertraline on patients with mild to moderate depression: A double-blind controlled trial. Phytomedicine Plus. 2023;3(2).

107.	Talaei A, Noori R, Rezaei Ardani A, Mohammadpour AH, Azimipoor Z, Afzaljavan F. The Effect of Adding Curcumin to Sertraline in the Treatment of Severe Major Depressive Disorder: A Randomized, Double-Blind Clinical Trial. Clinical Neuropharmacology. 2023;46(4):135-9.

108.	Kasper S, Anghelescu IG, Szegedi A, Dienel A, Kieser M. Superior efficacy of St John's wort extract WS 5570 compared to placebo in patients with major depression: a randomized, double-blind, placebo-controlled, multi-center trial. 2006;4:14

C3 - PUBMED 16796730,EMBASE 44227549.

109.	Sadeghi A, Ghorayshi F, Baghshahi H, Akbari H, Memarzadeh MR, Taghizadeh M, et al. The antidepressant effect of combined extracts of Hypericum perforatum and Echium amoenum supplementation in patients with depression symptoms: A randomized clinical trial. Avicenna J Phytomedicine. 2023;13(4):328-37.

110.	Park Y, Park YS, Kim SH, Oh DH, Park YC. Supplementation of n-3 polyunsaturated fatty acids for major depressive disorder: A randomized, double-blind, 12-week, placebo-controlled trial in Korea. Annals of Nutrition and Metabolism. 2015;66(2-3):141-8.

111.	De Leo D. S-Adenosylmethionine as an antidepressant. A double-blind trial versus placebo. CURR THER RES CLIN EXP. 1987;41(6):865-70.

112.	Cheng YC, Chen WY, Lin C, Lee SH, Chiu CC, Kuo PH. The N-3 polyunsaturated fatty acids supplementation to prevent depression recurrence in patients with late-life depression: A 52-week double-blind, placebo-controlled trial. Journal of Affective Disorders. 2025;369:8-15.

113.	Kreuzer K, Reiter A, Birkl-Töglhofer AM, Dalkner N, Mörkl S, Mairinger M, et al. The PROVIT Study—Effects of Multispecies Probiotic Add-on Treatment on Metabolomics in Major Depressive Disorder—A Randomized, Placebo-Controlled Trial. Metabolites. 2022;12(8).

114.	Jelodar G, Javid Z, Sahraian A, Jelodar S. Saffron improved depression and reduced homocysteine level in patients with major depression: A Randomized, double-blind study. Avicenna J Phytomedicine. 2018;8(1):43-50.

115.	Khoraminya N, Tehrani-Doost M, Jazayeri S, Hosseini A, Djazayery A. Therapeutic effects of vitamin D as adjunctive therapy to fluoxetine in patients with major depressive disorder. Australian and New Zealand Journal of Psychiatry. 2013;47(3):271-5.

116.	Sepehrmanesh Z, Kolahdooz F, Abedi F, Mazroii N, Assarian A, Asemi Z, et al. Vitamin D supplementation affects the beck depression inventory, insulin resistance, and biomarkers of oxidative stress in patients with major depressive disorder: A randomized, controlled clinical trial. Journal of Nutrition. 2016;146(2):243-8.

117.	Sahoo JP, Jarnail S, Khurana H, Singh P. Omega-3 Fatty Acids Versus 5-hydroxytryptophan as Add on Therapy in Patients with Depression: A Comparative Study. Indian Journal of Pharmacology. 2015;47(7):S23-S4.

118.	Laakmann G. St. John's Wort in mild to moderate depression: The relevance of hyperforin for the clinical efficacy. Pharmacopsychiatry. 1998;31(SUPPL. 1):54-9.

119.	Chang JP, Chang SS, Yang HT, Chen HT, Chien YC, Yang B, et al. Omega-3 polyunsaturated fatty acids in cardiovascular diseases comorbid major depressive disorder - Results from a randomized controlled trial. Brain Behav Immun. 2020;85:14-20.

120.	Saccarello A, Montarsolo P, Massardo I, Picciotto R, Pedemonte A, Castagnaro R, et al. Oral Administration of S-Adenosylmethionine (SAMe) and Lactobacillus Plantarum HEAL9 Improves the Mild-To-Moderate Symptoms of Depression: a Randomized, Double-Blind, Placebo-Controlled Study. 2020;22(4 C3 - PUBMED 32589828,EMBASE 632201612).

121.	Friede M, Henneicke von Zepelin HH, Freudenstein J. Differential therapy of mild to moderate depressive episodes (ICD-10 F 32.0; F 32.1) with St. John's wort. Pharmacopsychiatry. 2001;34(SUPPL. 1):S38-S41.

122.	Ning H, Zhou H, Ren J, Zhou G, Yang N, Wang Z, et al. Zishen pingchan granules combined with pramipexole in the improvement of depressive symptoms in Parkinson's disease: a prospective, multicenter, randomized, double-blind, controlled clinical study. J Transl Med. 2022;20(1):357.

123.	Bergman J, Miodownik C, Bersudsky Y, Sokolik S, Lerner PP, Kreinin A, et al. Curcumin as an add-on to antidepressive treatment: A randomized, double-blind, placebo-controlled, pilot clinical study. Clinical Neuropharmacology. 2013;36(3):73-7.

124.	Kasper S, Anghelescu IG, Szegedi A, Dienel A, Kieser M. Placebo controlled continuation treatment with Hypericum extract WS® 5570 after recovery from a mild or moderate depressive episode. Wiener Medizinische Wochenschrift. 2007;157(13-14):362-6.

125.	Bot M, Pouwer F, Assies J, Jansen EH, Diamant M, Snoek FJ, et al. Eicosapentaenoic acid as an add-on to antidepressant medication for co-morbid major depression in patients with diabetes mellitus: a randomized, double-blind placebo-controlled study. J Affect Disord. 2010;126(1-2):282-6.

126.	Mozaffari-Khosravi H, Nabizade L, Yassini-Ardakani SM, Hadinedoushan H, Barzegar K. The effect of 2 different single injections of high dose of vitamin D on improving the depression in depressed patients with vitamin D deficiency: A randomized clinical trial. Journal of Clinical Psychopharmacology. 2013;33(3):378-85.

127.	Sarris J, Murphy J, Stough C, Mischoulon D, Bousman C, MacDonald P, et al. S-Adenosylmethionine (SAMe) monotherapy for depression: an 8-week double-blind, randomised, controlled trial. 2020;237(1):209‐18

C3 - PUBMED 31712971,EMBASE 2003633556.

128.	Bell KM, Plon L, Bunney Jr WE, Potkin SG. S-adenosylmethionine treatment of depression: A controlled clinical trial. American Journal of Psychiatry. 1988;145(9):1110-4.

129.	Siwek M, Dudek D, Schlegel-Zawadzka M, Morawska A, Piekoszewski W, Opoka W, et al. Serum zinc level in depressed patients during zinc supplementation of imipramine treatment. Journal of Affective Disorders. 2010;126(3):447-52.

130.	Papakostas GI, Shelton RC, Zajecka JM, Etemad B, Rickels K, Clain A, et al. L-methylfolate as adjunctive therapy for SSRI-resistant major depression: results of two randomized, double-blind, parallel-sequential trials. 2012;169(12):1267‐74

C3 - PUBMED 23212058.

131.	Majmasanaye M, Mehrpooya M, Amiri H, Eshraghi A. Discovering the Potential Value of Coenzyme Q10 as an Adjuvant Treatment in Patients with Depression. Journal of Clinical Psychopharmacology. 2024;44(3):232-9.

132.	Hvas AM, Juul S, Lauritzen L, Nexø E, Ellegaard J. No effect of vitamin B-12 treatment on cognitive function and depression: A randomized placebo controlled study. Journal of Affective Disorders. 2004;81(3):269-73.

133.	Bidzan-Wiącek M, Tomczyk M, Błażek M, Mika A, Antosiewicz J. No Effects of Omega-3 Supplementation on Kynurenine Pathway, Inflammation, Depressive Symptoms, and Stress Response in Males: A Placebo-Controlled Trial. Nutrients. 2024;16(21 C7 - 3744).

134.	Sarris J, Byrne GJ, Stough C, Bousman C, Mischoulon D, Murphy J, et al. Nutraceuticals for major depressive disorder- more is not merrier: An 8-week double-blind, randomised, controlled trial. Journal of Affective Disorders. 2019;245:1007-15.

135.	Wu SK, Yang KJ, Liu WC, Malau IA, Zailani H, Chang CH, et al. The Efficacy of Omega-3 Fatty Acids as the Monotherapy for Depression: A Randomized, Double-Blind, Placebo-Controlled Pilot Study. Nutrients. 2024;16(21).

136.	Zhang L, Wang S, Zhu Y, Yang T. Vitamin D3 as adjunctive therapy in the treatment of depression in tuberculosis patients: A short-term pilot randomized double-blind controlled study. Neuropsychiatric Disease and Treatment. 2018;14:3103-9.

137.	Kasper S, Volz HP, Möller HJ, Schläfke S, Klement S, Anghelescu IG, et al. Lavender oil preparation Silexan is effective in mild-to-moderate major depression: a randomized, placebo- and reference-controlled trial. European Archives of Psychiatry and Clinical Neuroscience. 2024.

138.	Kolouri S, Firoozabadi A, Salehi A, Zarshenas MM, Dastgheib SA, Heydari M, et al. Nepeta menthoides Boiss. & Buhse freeze-dried aqueous extract versus sertraline in the treatment of major depression: A double blind randomized controlled trial. Complement Ther Med. 2016;26:164-70.

139.	Freeman MP, Davis M, Sinha P, Wisner KL, Hibbeln JR, Gelenberg AJ. Omega-3 fatty acids and supportive psychotherapy for perinatal depression: A randomized placebo-controlled study. Journal of Affective Disorders. 2008;110(1-2):142-8.

140.	Varzaghani V, Sharifi M, Hajiaghaee R, Bagheri S, Momtaz S, Tarassoli Z, et al. Propolis add-on therapy alleviates depressive symptoms; A randomized placebo-controlled clinical trial. Phytotherapy Research. 2022;36(3):1258-67.

141.	Su KP, Huang SY, Chiu TH, Huang KC, Huang CL, Chang HC, et al. Omega-3 fatty acids for major depressive disorder during pregnancy: Results from a randomized, double-blind, placebo-controlled trial. Journal of Clinical Psychiatry. 2008;69(4):644-51.

142.	Mischoulon D, Dunlop BW, Kinkead B, Schettler PJ, Lamon-Fava S, Rakofsky JJ, et al. Omega-3 Fatty Acids for Major Depressive Disorder With High Inflammation: A Randomized Dose-Finding Clinical Trial. Journal of Clinical Psychiatry. 2022;83(5 C7 - 21m14074).

143.	Kagan BL, Sultzer DL, Rosenlicht N, Gerner RH. Oral S-adenosylmethionine in depression: a randomized, double-blind, placebo-controlled trial. 1990;147(5):591‐5

C3 - PUBMED 2183633.

144.	Han CH, Chung MI, Bose S, Lim C, Kim H, Koo BS, et al. The effect of Gami Guibitang (GGBT), a medicinal herbal formulation, on moderate depression. European Journal of Integrative Medicine. 2012;4(4):e400-e7.

145.	Pancheri P, Scapicchio P, Delle Chiaie R. A double-blind, randomized parallel-group, efficacy and safety study of intramuscular S-adenosyl-L-methionine 1, 4-butanedisulphonate (SAMe) versus imipramine in patients with major depressive disorder. International Journal of Neuropsychopharmacology. 2002;5(4):287-94.

146.	Mischoulon D, Price LH, Carpenter LL, Tyrka AR, Papakostas GI, Baer L, et al. A double-blind, randomized, placebo-controlled clinical trial of S-adenosyl-L-methionine (SAMe) versus escitalopram in major depressive disorder. J Clin Psychiatry. 2014;75(4):370-6.

147.	Mao JJ, Xie SX, Zee J, Soeller I, Li QS, Rockwell K, et al. Rhodiola rosea versus sertraline for major depressive disorder: A randomized placebo-controlled trial. Phytomedicine. 2015;22(3):394-9.

148.	Sempach L, Schaub A-C, Brühl AB, Sterzer P, Lang UE, Schmidt A. Adjunctive d-serine treatment for major depressive disorder: A randomized clinical trial. Journal of Affective Disorders. 2025:119504.

**e-table 5:** Response node 1 subnetworks.

| **Author(s), year** | **Treatment** | **N** | **Subnetwork** |
| --- | --- | --- | --- |
| ^600^ | AYA | 14 | 1 |
| ^600^ | PLB | 15 | 1 |
| ^456^ | SAFF | 25 | 1 |
| ^456^ | SER | 25 | 1 |
| ^457^ | SAFF | 20 | 1 |
| ^457^ | FLUO | 20 | 1 |
| ^466^ | ESC+FOL | 20 | 1 |
| ^466^ | ESC | 15 | 1 |
| ^467^ | SJW | 35 | 1 |
| ^467^ | FLUO | 35 | 1 |
| ^468^ | SAMe | 9 | 1 |
| ^468^ | IMI | 9 | 1 |
| ^472^ | LI-160 | 54 | 1 |
| ^472^ | FLUO | 54 | 1 |
| ^472^ | PLB | 55 | 1 |
| ^474^ | LI-160 | 15 | 1 |
| ^474^ | SER | 15 | 1 |
| ^482^ | CHRO | 10 | 1 |
| ^482^ | PLB | 5 | 1 |
| ^486^ | CHRO | 70 | 1 |
| ^486^ | PLB | 40 | 1 |
| ^491^ | ZE117 | 126 | 1 |
| ^491^ | FLUO | 114 | 1 |
| ^493^ | LI-160 | 41 | 1 |
| ^493^ | PLB | 54 | 1 |
| ^495^ | SAFF | 30 | 1 |
| ^495^ | CTP | 30 | 1 |
| ^499^ | LH57 | 70 | 1 |
| ^499^ | FLUO | 79 | 1 |
| ^500^ | GBDP | 34 | 1 |
| ^500^ | PLB | 34 | 1 |
| ^504^ | EPA | 16 | 1 |
| ^504^ | FLUO | 16 | 1 |
| ^504^ | EPA+FLUO | 16 | 1 |
| ^505^ | SAMe | 9 | 1 |
| ^505^ | PLB | 6 | 1 |
| ^506^ | WS5572 | 37 | 1 |
| ^506^ | PLB | 35 | 1 |
| ^508^ | SAFF | 28 | 1 |
| ^508^ | PLB | 28 | 1 |
| ^509^ | SAFF | 32 | 1 |
| ^509^ | FLUO | 32 | 1 |
| ^510^ | WS5570 | 243 | 1 |
| ^510^ | PLB | 81 | 1 |
| ^511^ | WS5570 | 68 | 1 |
| ^511^ | PLB | 24 | 1 |
| ^597^ | SIL | 154 | 1 |
| ^597^ | SER | 151 | 1 |
| ^597^ | PLB | 141 | 1 |
| ^518^ | WS5573 | 49 | 1 |
| ^518^ | WS5572 | 49 | 1 |
| ^518^ | PLB | 49 | 1 |
| ^211^ | WS5570 | 186 | 1 |
| ^211^ | PLB | 189 | 1 |
| ^519^ | INO | 13 | 1 |
| ^519^ | PLB | 15 | 1 |
| ^604^ | INO | 13 | 1 |
| ^604^ | PLB | 14 | 1 |
| ^520^ | SHU | 194 | 1 |
| ^520^ | PLB | 191 | 1 |
| ^523^ | CUR+SAFF | 26 | 1 |
| ^523^ | PLB | 36 | 1 |
| ^526^ | DHA | 18 | 1 |
| ^526^ | PLB | 17 | 1 |
| ^254^ | EPA | 16 | 1 |
| ^254^ | PLB | 19 | 1 |
| ^603^ | EPA | 11 | 1 |
| ^603^ | PLB | 10 | 1 |
| ^529^ | SAMe | 64 | 1 |
| ^529^ | ESC | 65 | 1 |
| ^529^ | PLB | 60 | 1 |
| ^272^ | EPA | 21 | 1 |
| ^272^ | PLB | 21 | 1 |
| ^531^ | CREA | 9 | 1 |
| ^531^ | PLB | 9 | 1 |
| ^532^ | ZPG | 89 | 1 |
| ^532^ | PLB | 87 | 1 |
| ^537^ | SAMe | 146 | 1 |
| ^537^ | IMI | 147 | 1 |
| ^539^ | LMF | 36 | 1 |
| ^539^ | PLB | 112 | 1 |
| ^539^ | LMF | 19 | 1 |
| ^539^ | PLB | 56 | 1 |
| ^548^ | SAMe | 16 | 1 |
| ^548^ | ESC | 23 | 1 |
| ^548^ | PLB | 26 | 1 |
| ^549^ | CUR | 16 | 1 |
| ^549^ | FLUO | 17 | 1 |
| ^549^ | CUR+FLUO | 18 | 1 |
| ^552^ | SAMe | 22 | 1 |
| ^552^ | PLB | 19 | 1 |
| ^553^ | ZE117 | 125 | 1 |
| ^553^ | FLUO | 113 | 1 |
| ^554^ | ZE117 | 80 | 1 |
| ^554^ | PLB | 79 | 1 |
| ^558^ | SAFF | 20 | 1 |
| ^558^ | SSRI | 20 | 1 |
| ^562^ | SJW | 98 | 1 |
| ^562^ | PLB | 102 | 1 |
| ^564^ | EPA+DHA | 15 | 1 |
| ^564^ | PLB | 16 | 1 |
| ^387^ | EPA+DHA | 13 | 1 |
| ^387^ | PLB | 11 | 1 |
| ^596^ | PRO | 28 | 1 |
| ^596^ | PLB | 26 | 1 |
| ^574^ | LI-160 | 107 | 1 |
| ^574^ | IMI | 102 | 1 |
| ^429^ | LI-160 | 83 | 1 |
| ^429^ | AMI | 73 | 1 |
| ^576^ | ZE | 157 | 1 |
| ^576^ | IMI | 167 | 1 |
| ^577^ | EPA+DHA | 30 | 1 |
| ^577^ | PLB | 30 | 1 |
| ^578^ | vitD3 | 56 | 1 |
| ^578^ | PLB | 64 | 1 |
| ^475^ | SER+EPA | 71 | 2 |
| ^475^ | SER+PLB | 73 | 2 |
| ^494^ | SER+PLB | 19 | 2 |
| ^494^ | SER+CIN | 23 | 2 |
| ^560^ | SER+THE | 25 | 2 |
| ^560^ | SER+PLB | 25 | 2 |
| ^535^ | SAR+TAU | 30 | 3 |
| ^535^ | PLB+TAU | 30 | 3 |
| ^538^ | SAMe+TAU | 39 | 3 |
| ^538^ | PLB+TAU | 34 | 3 |
| ^550^ | SAMe+TAU | 37 | 3 |
| ^550^ | PLB+TAU | 39 | 3 |
| ^582^ | THI+FLUO | 25 | 4 |
| ^582^ | PLB+FLUO | 26 | 4 |
| ^583^ | MG+FLUO | 17 | 4 |
| ^583^ | PLB+FLUO | 15 | 4 |
| ^579^ | CTP+PLB | 25 | 5 |
| ^579, 585^ | LAV+PLB | 25 | 5 |
| ^581^ | FOL+CTP | 45 | 5 |
| ^581^ | CTP+PLB | 45 | 5 |
| ^586^ | CIT+CTP | 25 | 6 |
| ^586^ | PLB+CTP | 25 | 6 |
| ^585^ | vitC+CTP | 21 | 6 |
| ^585^ | PLB+CTP | 22 | 6 |
| ^605^ | SUL+CLO | 36 | 7 |
| ^605^ | PLB+CLO | 39 | 7 |
| ^598^ | ZNC+IMI | 27 | 8 |
| ^598^ | IMI+PLB | 25 | 8 |
| ^584^ | FOL+FLUO | 51 | 9 |
| ^584^ | FLUO+PLB | 58 | 9 |
| ^593^ | ESC+CREA | 25 | 10 |
| ^593^ | ESC+PLB | 27 | 10 |
| ^594^ | CUR+ESC | 50 | 11 |
| ^594^ | PLB+ESC | 50 | 11 |
| ^595^ | EPA+DHA+VEN | 21 | 12 |
| ^595^ | PLB+VEN | 27 | 12 |

**Reference for e-table 5**

1.	Antidepressant Effects of Ayahuasca: a Randomized Placebo Controlled Trial in Treatment Resistant Depression. clinicaltrialsgov2016.

2.	Ahmadpanah M, Ramezanshams F, Ghaleiha A, Akhondzadeh S, Sadeghi Bahmani D, Brand S. Crocus Sativus L. (saffron) versus sertraline on symptoms of depression among older people with major depressive disorders–a double-blind, randomized intervention study. Psychiatry Research. 2019;282.

3.	Akhondzadeh Basti A, Moshiri E, Noorbala AA, Jamshidi AH, Abbasi SH, Akhondzadeh S. Comparison of petal of Crocus sativus L. and fluoxetine in the treatment of depressed outpatients: A pilot double-blind randomized trial. Progress in Neuro-Psychopharmacology and Biological Psychiatry. 2007;31(2):439-42.

4.	Basoglu C, Ates MA, Algul A, Ipcioglu OM, Gecici O, Yilmaz O, et al. Adjuvant folate with escitalopram treatment and homocystein, folate, vitamin B-12 levels in patients with major depressive disorder. 2009;19(2):135‐42

C3 - EMBASE 354832073.

5.	Behnke K, Jensen GS, Graubaum HJ, Gruenwald J. Hypericum perforatum versus fluoxetine in the treatment of mild to moderate depression. Advances in Therapy. 2002;19(1):43-52.

6.	Bell KM, Plon L, Bunney Jr WE, Potkin SG. S-adenosylmethionine treatment of depression: A controlled clinical trial. American Journal of Psychiatry. 1988;145(9):1110-4.

7.	Bjerkenstedt L, Edman GV, Alken RG, Mannel M. Hypericum extract LI 160 and fluoxetine in mild to moderate depression: A randomized, placebo-controlled multi-center study in outpatients. European Archives of Psychiatry and Clinical Neuroscience. 2005;255(1):40-7.

8.	Brenner R, Azbel V, Madhusoodanan S, Pawlowska M. Comparison of an extract of hypericum (LI 160) and sertraline in the treatment of depression: A double-blind, randomized pilot study. Clinical Therapeutics. 2000;22(4):411-9.

9.	Davidson JR, Gadde KM, Fairbank JA, Krishnan KRR, Califf RM, Binanay C, et al. Effect of Hypericum perforatum (St John's wort) in major depressive disorder: a randomized controlled trial. 2002;287(14):1807‐14

C3 - PUBMED 11939866,EMBASE 34303625.

10.	Docherty JP, Sack DA, Roffman M, Finch M, Komorowski JR. A double-blind, placebo-controlled, exploratory trial of chromium picolinate in atypical depression: Effect on carbohydrate craving. Journal of Psychiatric Practice. 2005;11(5):302-14.

11.	Friede M, Henneicke von Zepelin HH, Freudenstein J. Differential therapy of mild to moderate depressive episodes (ICD-10 F 32.0; F 32.1) with St. John's wort. Pharmacopsychiatry. 2001;34(SUPPL. 1):S38-S41.

12.	Gelenberg AJ, Shelton RC, Crits-Christoph P, Keller MB, Dunner DL, Hirschfeld RM, et al. The effectiveness of St. John's Wort in major depressive disorder: a naturalistic phase 2 follow-up in which nonresponders were provided alternate medication. 2004;65(8):1114‐9

C3 - PUBMED 15323598.

13.	Ghajar A, Neishabouri SM, Velayati N, Jahangard L, Matinnia N, Haghighi M, et al. Crocus sativus L versus Citalopram in the Treatment of Major Depressive Disorder with Anxious Distress: A Double-Blind, Controlled Clinical Trial. Pharmacopsychiatry. 2017;50(4):152-60.

14.	Harrer G, Schmidt U, Kuhn U, Biller A. Comparison of equivalence between the St. John's wort extract LoHyp-57 and fluoxetine. Arzneimittel-Forschung/Drug Research. 1999;49(4):289-96.

15.	He X, Liu D, Ni S, Li Z, Li S, Wu T, et al. Efficacy and safety evaluation of Ginkgo biloba dropping pill (GBDP) on stable angina pectoris complicated with depression: A placebo-controlled, randomized, double-blind, multicenter study. Phytomedicine. 2024;126.

16.	Jazayeri S, Tehrani-Doost M, Keshavarz SA, Hosseini M, Djazayery A, Amini H, et al. Comparison of therapeutic effects of omega-3 fatty acid eicosapentaenoic acid and fluoxetine, separately and in combination, in major depressive disorder. Australian and New Zealand Journal of Psychiatry. 2008;42(3):192-8.

17.	Kagan BL, Sultzer DL, Rosenlicht N, Gerner RH. Oral S-adenosylmethionine in depression: a randomized, double-blind, placebo-controlled trial. 1990;147(5):591‐5

C3 - PUBMED 2183633.

18.	Kalb R, Trautmann-Sponsel RD, Kieser M. Efficacy and tolerability of Hypericum extract WS 5572 versus placebo in mildly to moderately depressed patients: A randomized double-blind multicenter clinical trial. Pharmacopsychiatry. 2001;34(3):96-103.

19.	Kashani L, Esalatmanesh S, Eftekhari F, Salimi S, Foroughifar T, Etesam F, et al. Efficacy of Crocus sativus (saffron) in treatment of major depressive disorder associated with post-menopausal hot flashes: a double-blind, randomized, placebo-controlled trial. Arch Gynecol Obstet. 2018;297(3):717-24.

20.	Kashani L, Eslatmanesh S, Saedi N, Niroomand N, Ebrahimi M, Hosseinian M, et al. Comparison of Saffron versus Fluoxetine in Treatment of Mild to Moderate Postpartum Depression: A Double-Blind, Randomized Clinical Trial. Pharmacopsychiatry. 2017;50(2):64-8.

21.	Kasper S, Anghelescu IG, Szegedi A, Dienel A, Kieser M. Superior efficacy of St John's wort extract WS 5570 compared to placebo in patients with major depression: a randomized, double-blind, placebo-controlled, multi-center trial. 2006;4:14

C3 - PUBMED 16796730,EMBASE 44227549.

22.	Kasper S, Anghelescu IG, Szegedi A, Dienel A, Kieser M. Placebo controlled continuation treatment with Hypericum extract WS® 5570 after recovery from a mild or moderate depressive episode. Wiener Medizinische Wochenschrift. 2007;157(13-14):362-6.

23.	Kasper S, Volz HP, Möller HJ, Schläfke S, Klement S, Anghelescu IG, et al. Lavender oil preparation Silexan is effective in mild-to-moderate major depression: a randomized, placebo- and reference-controlled trial. European Archives of Psychiatry and Clinical Neuroscience. 2024.

24.	Laakmann G. St. John's Wort in mild to moderate depression: The relevance of hyperforin for the clinical efficacy. Pharmacopsychiatry. 1998;31(SUPPL. 1):54-9.

25.	Lecrubier Y, Clerc G, Didi R, Kieser M. Efficacy of St. John's wort extract WS 5570 in major depression: A double-blind, placebo-controlled trial. American Journal of Psychiatry. 2002;159(8):1361-6.

26.	Levine J, Barak Y, Gonzalves M, Szor H, Elizur A, Kofman O, et al. Double-blind, controlled trial of inositol treatment of depression. American Journal of Psychiatry. 1995;152(5):792-4.

27.	Levine J, Barak Y, Kofman O, Belmaker RH. Follow-up and relapse analysis of an inositol study of depression. Isr J Psychiatry Relat Sci. 1995;32(1):14-21.

28.	Li Y, Li L, Guo R, Yu H, Wang X, Wang B, et al. Clinical efficacy of Shugan granule in the treatment of mixed anxiety-depressive disorder: a multicenter, randomized, double-blind, placebo-controlled trial. 2022;290:115032

C3 - PUBMED 35085742,EMBASE 637131287.

29.	Lopresti AL, Drummond PD. Efficacy of curcumin, and a saffron/curcumin combination for the treatment of major depression: A randomised, double-blind, placebo-controlled study. Journal of Affective Disorders. 2017;207:188-96.

30.	Marangell LB, Martinez JM, Zboyan HA, Kertz B, Kim HFS, Puryear LJ. A double-blind, placebo-controlled study of the omega-3 fatty acid docosahexaenoic acid in the treatment of major depression. American Journal of Psychiatry. 2003;160(5):996-8.

31.	Mischoulon D, Best-Popescu C, Laposata M, Merens W, Murakami JL, Wu SL, et al. A double-blind dose-finding pilot study of docosahexaenoic acid (DHA) for major depressive disorder. European Neuropsychopharmacology. 2008;18(9):639-45.

32.	Mischoulon D, Dunlop BW, Kinkead B, Schettler PJ, Lamon-Fava S, Rakofsky JJ, et al. Omega-3 Fatty Acids for Major Depressive Disorder With High Inflammation: A Randomized Dose-Finding Clinical Trial. Journal of Clinical Psychiatry. 2022;83(5 C7 - 21m14074).

33.	Mischoulon D, Price LH, Carpenter LL, Tyrka AR, Papakostas GI, Baer L, et al. A double-blind, randomized, placebo-controlled clinical trial of S-adenosyl-L-methionine (SAMe) versus escitalopram in major depressive disorder. J Clin Psychiatry. 2014;75(4):370-6.

34.	Mozaffari-Khosravi H, Nabizade L, Yassini-Ardakani SM, Hadinedoushan H, Barzegar K. The effect of 2 different single injections of high dose of vitamin D on improving the depression in depressed patients with vitamin D deficiency: A randomized clinical trial. Journal of Clinical Psychopharmacology. 2013;33(3):378-85.

35.	Nemets B, Levine J. A pilot dose-finding clinical trial of creatine monohydrate augmentation to SSRIs/SNRIs/NASA antidepressant treatment in major depression. International Clinical Psychopharmacology. 2013;28(3):127-33.

36.	Ning H, Zhou H, Ren J, Zhou G, Yang N, Wang Z, et al. Zishen pingchan granules combined with pramipexole in the improvement of depressive symptoms in Parkinson's disease: a prospective, multicenter, randomized, double-blind, controlled clinical study. J Transl Med. 2022;20(1):357.

37.	Pancheri P, Scapicchio P, Delle Chiaie R. A double-blind, randomized parallel-group, efficacy and safety study of intramuscular S-adenosyl-L-methionine 1, 4-butanedisulphonate (SAMe) versus imipramine in patients with major depressive disorder. International Journal of Neuropsychopharmacology. 2002;5(4):287-94.

38.	Papakostas GI, Shelton RC, Zajecka JM, Etemad B, Rickels K, Clain A, et al. L-methylfolate as adjunctive therapy for SSRI-resistant major depression: results of two randomized, double-blind, parallel-sequential trials. 2012;169(12):1267‐74

C3 - PUBMED 23212058.

39.	Sakurai H, L. Carpenter L, R. Tyrka A, Price LH, I. Papakostas G, Dording CM, et al. Dose increase of S-Adenosyl-Methionine and escitalopram in a randomized clinical trial for major depressive disorder. Journal of Affective Disorders. 2020;262:118-25.

40.	Sanmukhani J, Satodia V, Trivedi J, Patel T, Tiwari D, Panchal B, et al. Efficacy and safety of curcumin in major depressive disorder: a randomized controlled trial. Phytother Res. 2014;28(4):579-85.

41.	Sarris J, Murphy J, Stough C, Mischoulon D, Bousman C, MacDonald P, et al. S-Adenosylmethionine (SAMe) monotherapy for depression: an 8-week double-blind, randomised, controlled trial. 2020;237(1):209‐18

C3 - PUBMED 31712971,EMBASE 2003633556.

42.	Schrader E. Equivalence of St John's wort extract (Ze 117) and fluoxetine: A randomized, controlled study in mild-moderate depression. International Clinical Psychopharmacology. 2000;15(2):61-8.

43.	Schrader E, Meier B, Brattström A. Hypericum treatment of mild-moderate depression in a placebo-controlled study. A prospective, double-blind, randomized, placebo-controlled, multicentre study. Human Psychopharmacology. 1998;13(3):163-9.

44.	Shahmansouri N, Farokhnia M, Abbasi SH, Kassaian SE, Noorbala Tafti AA, Gougol A, et al. A randomized, double-blind, clinical trial comparing the efficacy and safety of Crocus sativus L. with fluoxetine for improving mild to moderate depression in post percutaneous coronary intervention patients. 2014;155:216‐22

C3 - PUBMED 24289892,EMBASE 52891114.

45.	Shelton RC, Keller MB, Gelenberg A, Dunner DL, Hirschfeld R, Thase ME, et al. Effectiveness of St John's wort in major depression: A randomized controlled trial. JAMA. 2001;285(15):1978-86.

46.	Shinto L, Marracci G, Mohr DC, Bumgarner L, Murchison C, Senders A, et al. Omega-3 fatty acids for depression in multiple sclerosis: A randomized pilot study. PLoS ONE. 2016;11(1 C7 - e0147195).

47.	Su KP, Huang SY, Chiu TH, Huang KC, Huang CL, Chang HC, et al. Omega-3 fatty acids for major depressive disorder during pregnancy: Results from a randomized, double-blind, placebo-controlled trial. Journal of Clinical Psychiatry. 2008;69(4):644-51.

48.	Varzaghani V, Sharifi M, Hajiaghaee R, Bagheri S, Momtaz S, Tarassoli Z, et al. Propolis add-on therapy alleviates depressive symptoms; A randomized placebo-controlled clinical trial. Phytotherapy Research. 2022;36(3):1258-67.

49.	Vorbach EU, Arnoldt KH, Hubner WD. Efficacy and tolerability of St. John's wort extract LI 160 versus imipramine in patients with severe depressive episodes according to ICD-10. Pharmacopsychiatry. 1997;30(SUPPL.):81-5.

50.	Wheatley D. LI 160, an extract of St. John's wort, versus amitriptyline in mildly to moderately depressed outpatients--a controlled 6-week clinical trial. 1997;30 Suppl 2:77‐80

C3 - PUBMED 9342764.

51.	Woelk H. Comparison of St John's wort and imipramine for treating depression: Randomised controlled trial. British Medical Journal. 2000;321(7260):536-9.

52.	Wu SK, Yang KJ, Liu WC, Malau IA, Zailani H, Chang CH, et al. The Efficacy of Omega-3 Fatty Acids as the Monotherapy for Depression: A Randomized, Double-Blind, Placebo-Controlled Pilot Study. Nutrients. 2024;16(21).

53.	Zhang L, Wang S, Zhu Y, Yang T. Vitamin D3 as adjunctive therapy in the treatment of depression in tuberculosis patients: A short-term pilot randomized double-blind controlled study. Neuropsychiatric Disease and Treatment. 2018;14:3103-9.

54.	Carney RM, Freedland KE, Rubin EH, Rich MW, Steinmeyer BC, Harris WS. A randomized placebo-controlled trial of omega-3 and sertraline in depressed patients with or at risk for coronary heart disease. Journal of Clinical Psychiatry. 2019;80(4 C7 - 19m12742).

55.	Ghaffari S, Ghobadi A, Jamshidi AH, Mortazavi SH, Naderi S, Aqamolaei A, et al. Cinnamomum tamala as an adjuvant therapy in the treatment of major depressive disorder: A double-blind, randomized, placebo-controlled clinical trial with placebo control. Advances in Integrative Medicine. 2020;7(3):141-7.

56.	Shamabadi A, Fattollahzadeh-Noor S, Fallahpour B, A. Basti F, Khodaei Ardakani MR, Akhondzadeh S. L-Theanine adjunct to risperidone in the treatment of chronic schizophrenia inpatients: a randomized, double-blind, placebo-controlled clinical trial. Psychopharmacology. 2023;240(12):2631-40.

57.	Padhan M, Mohapatra D, Mishra BR, Maiti R, Jena M. Efficacy and safety of add-on sarcosine in patients with major depressive disorder: A randomized controlled trial. Journal of Psychiatric Research. 2024;178:298-304.

58.	Papakostas GI, Mischoulon D, Shyu I, Alpert JE, Fava M. S-adenosyl methionine (SAMe) augmentation of serotonin reuptake inhibitors for antidepressant nonresponders with major depressive disorder: a double-blind, randomized clinical trial. 2010;167(8):942‐8

C3 - PUBMED 20595412.

59.	Sarris J, Byrne GJ, Bousman C, Stough C, Murphy J, MacDonald P, et al. Adjunctive S-adenosylmethionine (SAMe) in treating non-remittent major depressive disorder: An 8-week double-blind, randomized, controlled trial. European Neuropsychopharmacology. 2018;28(10):1126-36.

60.	Ghaleiha A, Davari H, Jahangard L, Haghighi M, Ahmadpanah M, Seifrabie MA, et al. Adjuvant thiamine improved standard treatment in patients with major depressive disorder: results from a randomized, double-blind, and placebo-controlled clinical trial. European Archives of Psychiatry and Clinical Neuroscience. 2016;266(8):695-702.

61.	Ryszewska-Pokraśniewicz B, Mach A, Skalski M, Januszko P, Wawrzyniak ZM, Poleszak E, et al. Effects of magnesium supplementation on unipolar depression: A placebo-controlled study and review of the importance of dosing and magnesium status in the therapeutic response. Nutrients. 2018;10(8 C7 - 1014).

62.	Firoozeei TS, Barekatain M, Karimi M, Zargaran A, Akhondzadeh S, Rezaeizadeh H. Lavender and dodder combined herbal syrup versus citalopram in major depressive disorder with anxious distress: A double-blind randomized trial. Journal of Integrative Medicine. 2020;18(5):409-15.

63.	Sahraian A, Ghanizadeh A, Kazemeini F. Vitamin C as an adjuvant for treating major depressive disorder and suicidal behavior, a randomized placebo-controlled clinical trial. Trials. 2015;16(1 C7 - 94).

64.	Sepehrmanesh Z, Kolahdooz F, Abedi F, Mazroii N, Assarian A, Asemi Z, et al. Vitamin D Supplementation Affects the Beck Depression Inventory, Insulin Resistance, and Biomarkers of Oxidative Stress in Patients with Major Depressive Disorder: A Randomized, Controlled Clinical Trial [Retraction notice in CN-02298463]. 2016;146(2):243‐8

C3 - PUBMED 26609167,EMBASE 608794678.

65.	Roohi-Azizi M, Arabzadeh S, Amidfar M, Salimi S, Zarindast MR, Talaei A, et al. Citicoline combination therapy for major depressive disorder: A randomized, double-blind, placebo-controlled trial. Clinical Neuropharmacology. 2017;40(1):1-5.

66.	Lôo H, Poirier MF, Ollat H, Elatki S. Effects of sulbutiamine (Arcalion 200) on psycho-behavioral inhibition in major depressive episodes. 2000;26(2):70‐5

C3 - PUBMED 10858919,EMBASE 30337124.

67.	Siwek M, Dudek D, Schlegel-Zawadzka M, Morawska A, Piekoszewski W, Opoka W, et al. Serum zinc level in depressed patients during zinc supplementation of imipramine treatment. Journal of Affective Disorders. 2010;126(3):447-52.

68.	Coppen A, Bailey J. Enhancement of the antidepressant action of fluoxetine by folic acid: A randomised, placebo controlled trial. Journal of Affective Disorders. 2000;60(2):121-30.

69.	Lyoo IK, Yoon S, Kim TS, Hwang J, Kim JE, Won W, et al. A randomized, double-blind placebo-controlled trial of oral creatine monohydrate augmentation for enhanced response to a selective serotonin reuptake inhibitor in women with major depressive disorder. American Journal of Psychiatry. 2012;169(9):937-45.

70.	Yu JJ, Pei LB, Zhang Y, Wen ZY, Yang JL. Chronic Supplementation of Curcumin Enhances the Efficacy of Antidepressants in Major Depressive Disorder: A Randomized, Double-Blind, Placebo-Controlled Pilot Study. Journal of Clinical Psychopharmacology. 2015;35(4):406-10.

71.	Yang R, Wang L, Jin K, Cao S, Wu C, Guo J, et al. Omega-3 Polyunsaturated Fatty Acids Supplementation Alleviate Anxiety Rather Than Depressive Symptoms Among First-Diagnosed, Drug-Naïve Major Depressive Disorder Patients: A Randomized Clinical Trial. Frontiers in Nutrition. 2022;9 C7 - 876152.

**e-table 6:** Acceptability node 1 subnetworks.

| **Author(s), year** | **Treatment** | **N** | **Subnetwork** |
| --- | --- | --- | --- |
| ^455^ | FOIL | 19 | 1 |
| ^455^ | KOIL | 19 | 1 |
| ^455^ | PLB | 19 | 1 |
| ^457^ | SAFF | 20 | 1 |
| ^457^ | FLUO | 20 | 1 |
| ^458^ | SAFF | 15 | 1 |
| ^458^ | IMI | 15 | 1 |
| ^459^ | SAF | 36 | 1 |
| ^459^ | PLB | 37 | 1 |
| ^460^ | MEL | 26 | 1 |
| ^460^ | PLB | 26 | 1 |
| ^460^ | LAV+IMI | 16 | 1 |
| ^460^ | LH57 | 70 | 1 |
| ^460^ | FLUO | 79 | 1 |
| ^461^ | PROB | 20 | 1 |
| ^461^ | PLB | 20 | 1 |
| ^599^ | EOPF | 60 | 1 |
| ^599^ | PLB | 60 | 1 |
| ^462^ | vitD3-Ca | 26 | 1 |
| ^462^ | vitD3 | 26 | 1 |
| ^462^ | PLB | 24 | 1 |
| ^463^ | EACS | 25 | 1 |
| ^463^ | CTP | 25 | 1 |
| ^464^ | MEL | 16 | 1 |
| ^464^ | LAV | 17 | 1 |
| ^464^ | FLUO | 17 | 1 |
| ^465^ | BIOKULT | 35 | 1 |
| ^465^ | PLB | 36 | 1 |
| ^466^ | ESC+FOL | 20 | 1 |
| ^466^ | ESC | 15 | 1 |
| ^467^ | SJW | 35 | 1 |
| ^467^ | FLUO | 35 | 1 |
| ^468^ | SAMe | 9 | 1 |
| ^468^ | IMI | 9 | 1 |
| ^469^ | CUR | 20 | 1 |
| ^469^ | PLB | 20 | 1 |
| ^470^ | CUR | 28 | 1 |
| ^470^ | PLB | 28 | 1 |
| ^471^ | EPA+DHA | 25 | 1 |
| ^471^ | PLB | 22 | 1 |
| ^473^ | EPA | 13 | 1 |
| ^473^ | PLB | 12 | 1 |
| ^474^ | LI-160 | 15 | 1 |
| ^474^ | SER | 15 | 1 |
| ^477^ | SAMe | 30 | 1 |
| ^477^ | PLB | 30 | 1 |
| ^478^ | OMEGA3 | 30 | 1 |
| ^478^ | PLB | 29 | 1 |
| ^479^ | EPA+DHA | 19 | 1 |
| ^479^ | PLB | 20 | 1 |
| ^480^ | LI-160 | 83 | 1 |
| ^480^ | AMI | 73 | 1 |
| ^481^ | RHO | 60 | 1 |
| ^481^ | PLB | 29 | 1 |
| ^482^ | CHRO | 10 | 1 |
| ^482^ | PLB | 5 | 1 |
| ^483^ | PLB | 116 | 1 |
| ^483^ | MG | 45 | 1 |
| ^483^ | PLB | 45 | 1 |
| ^484^ | SAMe | 20 | 1 |
| ^484^ | PLB | 20 | 1 |
| ^485^ | TEN | 30 | 1 |
| ^485^ | PLB | 30 | 1 |
| ^486^ | CHRO | 70 | 1 |
| ^486^ | PROB | 40 | 1 |
| ^487^ | SCU | 42 | 1 |
| ^487^ | SAFF+SCU | 48 | 1 |
| ^487^ | SAFF | 43 | 1 |
| ^487^ | PLB | 47 | 1 |
| ^488^ | LI-160 | 45 | 1 |
| ^488^ | FLUO | 47 | 1 |
| ^488^ | PLB | 43 | 1 |
| ^490^ | EPA+DHA | 31 | 1 |
| ^490^ | PLB | 28 | 1 |
| ^491^ | ZE117 | 126 | 1 |
| ^491^ | FLUO | 114 | 1 |
| ^493^ | LI-160 | 43 | 1 |
| ^493^ | PLB | 55 | 1 |
| ^495^ | EPA | 16 | 1 |
| ^495^ | FLUO | 16 | 1 |
| ^496^ | SYN | 20 | 1 |
| ^496^ | PLB | 20 | 1 |
| ^602^ | US | 54 | 1 |
| ^602^ | FLUO | 53 | 1 |
| ^602^ | FLUO+US | 55 | 1 |
| ^498^ | GGBT | 82 | 1 |
| ^498^ | PLB | 82 | 1 |
| ^499^ | SAFF | 34 | 1 |
| ^499^ | FLUO | 34 | 1 |
| ^500^ | GBDP | 34 | 1 |
| ^500^ | PLB | 34 | 1 |
| ^501^ | EA | 20 | 1 |
| ^501^ | PLB | 20 | 1 |
| ^502^ | SZL + PLB | 63 | 1 |
| ^502^ | FLUO + PLB | 66 | 1 |
| ^503^ | vitB12 | 70 | 1 |
| ^503^ | PLB | 70 | 1 |
| ^504^ | EPA+FLUO | 16 | 1 |
| ^504^ | SAFF | 25 | 1 |
| ^504^ | SER | 25 | 1 |
| ^591^ | FLUO+SAFF | 20 | 1 |
| ^591^ | FLUO + PLB | 20 | 1 |
| ^505^ | SAMe | 9 | 1 |
| ^505^ | PLB | 6 | 1 |
| ^506^ | WS5572 | 37 | 1 |
| ^506^ | PROB | 35 | 1 |
| ^507^ | CUR | 33 | 1 |
| ^507^ | PLB | 32 | 1 |
| ^508^ | SAFF | 28 | 1 |
| ^508^ | PLB | 28 | 1 |
| ^510^ | WS5570 | 243 | 1 |
| ^510^ | PLB | 81 | 1 |
| ^511^ | WS5570 | 68 | 1 |
| ^511^ | PLB | 24 | 1 |
| ^512^ | SIL | 159 | 1 |
| ^512^ | PLB | 156 | 1 |
| ^597^ | SIL | 170 | 1 |
| ^597^ | SER | 171 | 1 |
| ^597^ | PLB | 157 | 1 |
| ^513^ | vitD | 28 | 1 |
| ^513^ | PLB | 28 | 1 |
| ^514^ | vitD3+FLUO | 20 | 1 |
| ^514^ | FLUO | 20 | 1 |
| ^515^ | NEP | 33 | 1 |
| ^515^ | SER | 33 | 1 |
| ^516^ | OMB | 28 | 1 |
| ^516^ | PLB | 29 | 1 |
| ^518^ | WS5573 | 49 | 1 |
| ^518^ | WS5572 | 49 | 1 |
| ^518^ | PLB | 49 | 1 |
| ^211^ | WS5570 | 186 | 1 |
| ^211^ | PLB | 189 | 1 |
| ^519^ | INO | 13 | 1 |
| ^519^ | PLB | 15 | 1 |
| ^604^ | LI-160 | 113 | 1 |
| ^604^ | SER | 111 | 1 |
| ^522^ | BZT | 39 | 1 |
| ^522^ | SER | 39 | 1 |
| ^522^ | PLB | 39 | 1 |
| ^605^ | SAFF | 20 | 1 |
| ^605^ | FLUO | 20 | 1 |
| ^523^ | CUR | 61 | 1 |
| ^523^ | CUR+SAFF | 26 | 1 |
| ^523^ | PLB | 36 | 1 |
| ^525^ | RHO | 20 | 1 |
| ^525^ | SER | 19 | 1 |
| ^525^ | PLB | 18 | 1 |
| ^526^ | DHA | 18 | 1 |
| ^526^ | PLB | 17 | 1 |
| ^527^ | SAF | 30 | 1 |
| ^527^ | PLB | 30 | 1 |
| ^528^ | SAFF | 30 | 1 |
| ^528^ | CTP | 30 | 1 |
| ^254^ | EPA | 16 | 1 |
| ^254^ | PLB | 19 | 1 |
| ^603^ | EPA | 16 | 1 |
| ^603^ | PLB | 15 | 1 |
| ^529^ | SAMe | 64 | 1 |
| ^529^ | ESC | 65 | 1 |
| ^529^ | PLB | 60 | 1 |
| ^530^ | LAV | 16 | 1 |
| ^530^ | IMI | 16 | 1 |
| ^272^ | EPA | 21 | 1 |
| ^272^ | DHA | 20 | 1 |
| ^272^ | PLB | 21 | 1 |
| ^532^ | ZPG | 89 | 1 |
| ^532^ | PLB | 87 | 1 |
| ^533^ | LI-160 | 59 | 1 |
| ^533^ | FLUO | 57 | 1 |
| ^534^ | vitB | 170 | 1 |
| ^534^ | PLB | 160 | 1 |
| ^537^ | SAMe | 146 | 1 |
| ^537^ | IMI | 147 | 1 |
| ^539^ | LMF | 36 | 1 |
| ^539^ | PLB | 112 | 1 |
| ^539^ | LMF | 19 | 1 |
| ^539^ | PLB | 56 | 1 |
| ^541^ | DHA+EPA | 14 | 1 |
| ^541^ | PLB | 9 | 1 |
| ^332^ | EPA+DHA | 13 | 1 |
| ^332^ | PLB | 13 | 1 |
| ^543^ | EPA+DHA | 22 | 1 |
| ^543^ | PLB | 24 | 1 |
| ^545^ | SAMe+HEAL9 | 46 | 1 |
| ^545^ | PLB | 44 | 1 |
| ^546^ | EACS+SJW | 37 | 1 |
| ^546^ | FLUO | 37 | 1 |
| ^547^ | SER+EPA+DHA | 33 | 1 |
| ^547^ | SER+5HTP | 37 | 1 |
| ^547^ | SER | 33 | 1 |
| ^585^ | FLUO+SAFF | 20 | 1 |
| ^585^ | FLUO + PLB | 20 | 1 |
| ^548^ | SAMe | 16 | 1 |
| ^548^ | ESC | 23 | 1 |
| ^548^ | PLB | 26 | 1 |
| ^549^ | CUR | 20 | 1 |
| ^549^ | FLUO | 20 | 1 |
| ^549^ | CUR+FLUO | 20 | 1 |
| ^551^ | NUT | 81 | 1 |
| ^551^ | PLB | 77 | 1 |
| ^606^ | LI-160 | 35 | 1 |
| ^606^ | SER | 49 | 1 |
| ^606^ | PLB | 40 | 1 |
| ^552^ | SAMe | 25 | 1 |
| ^552^ | PLB | 24 | 1 |
| ^553^ | ZE117 | 126 | 1 |
| ^553^ | FLUO + PLB | 114 | 1 |
| ^554^ | ZE117 | 81 | 1 |
| ^554^ | PROB | 81 | 1 |
| ^556^ | vitD | 20 | 1 |
| ^556^ | PLB | 20 | 1 |
| ^558^ | SAFF | 22 | 1 |
| ^558^ | FLUO | 22 | 1 |
| ^559^ | APH | 27 | 1 |
| ^559^ | PLB | 27 | 1 |
| ^561^ | SAFF | 20 | 1 |
| ^561^ | FLUO | 20 | 1 |
| ^562^ | SJW | 98 | 1 |
| ^562^ | PLB | 102 | 1 |
| ^563^ | CIT | 17 | 1 |
| ^563^ | PLB | 14 | 1 |
| ^564^ | EPA+DHA | 21 | 1 |
| ^564^ | PLB | 18 | 1 |
| ^565^ | EPA+DHA | 12 | 1 |
| ^565^ | PLB | 10 | 1 |
| ^387^ | EPA+DHA | 18 | 1 |
| ^387^ | PLB | 18 | 1 |
| ^567^ | WS5570 | 122 | 1 |
| ^567^ | PAR | 122 | 1 |
| ^570^ | LS | 9 | 1 |
| ^570^ | PLB | 11 | 1 |
| ^571^ | SHU | 194 | 1 |
| ^571^ | PLB | 191 | 1 |
| ^572^ | SJW | 44 | 1 |
| ^572^ | SER | 43 | 1 |
| ^596^ | PRO | 28 | 1 |
| ^596^ | PLB | 26 | 1 |
| ^573^ | INO | 13 | 1 |
| ^573^ | PLB | 14 | 1 |
| ^574^ | LI-160 | 107 | 1 |
| ^574^ | IMI | 102 | 1 |
| ^575^ | vitD3 | 362 | 1 |
| ^575^ | PLB | 364 | 1 |
| ^429^ | INU | 31 | 1 |
| ^429^ | PLB | 31 | 1 |
| ^576^ | SAFF | 20 | 1 |
| ^576^ | PROB | 20 | 1 |
| ^577^ | EPA+DHA | 30 | 1 |
| ^577^ | PLB | 30 | 1 |
| ^578^ | vitD3 | 56 | 1 |
| ^578^ | PLB | 64 | 1 |
| Palhano-Fontes F. et al., ^600^ | AYA | 17 | 1 |
| Palhano-Fontes F. et al., ^600^ | PLB | 18 | 1 |
| ^456^ | ZE | 157 | 2 |
| ^456^ | TAU | 167 | 2 |
| ^489^ | NEP+TAU | 15 | 2 |
| ^489^ | CUS+TAU | 13 | 2 |
| ^489^ | TAU | 15 | 2 |
| ^497^ | OMEGA3+TAU | 40 | 2 |
| ^497^ | PLB+TAU | 43 | 2 |
| ^509^ | NAC+TAU | 127 | 2 |
| ^509^ | PLB+TAU | 125 | 2 |
| ^520^ | ZNC+TAU | 30 | 2 |
| ^520^ | TAU | 30 | 2 |
| ^521^ | PS128+TAU | 16 | 2 |
| ^521^ | PLB+TAU | 16 | 2 |
| ^524^ | CQ10+TAU | 46 | 2 |
| ^524^ | PLB+TAU | 46 | 2 |
| ^580^ | ZNC+TAU | 10 | 2 |
| ^580^ | PLB+TAU | 10 | 2 |
| ^296^ | EPA+TAU | 10 | 2 |
| ^296^ | PLB+TAU | 10 | 2 |
| ^536^ | CVE+TAU | 42 | 2 |
| ^536^ | TAU | 50 | 2 |
| ^538^ | SAMe+TAU | 39 | 2 |
| ^538^ | PLB+TAU | 34 | 2 |
| ^540^ | OMEGA3+TAU | 18 | 2 |
| ^540^ | PLB+TAU | 17 | 2 |
| ^542^ | ZNC+TAU | 21 | 2 |
| ^542^ | TAU | 22 | 2 |
| ^544^ | LP299v+TAU | 30 | 2 |
| ^544^ | PLB+TAU | 30 | 2 |
| ^550^ | SAMe+TAU | 55 | 2 |
| ^550^ | PLB+TAU | 52 | 2 |
| ^555^ | SRN+TAU | 26 | 2 |
| ^555^ | PLB+TAU | 26 | 2 |
| ^590^ | SAFF+TAU | 23 | 2 |
| ^590^ | PLB+TAU | 23 | 2 |
| ^569^ | SAMe+TAU | 118 | 2 |
| ^569^ | PLB+TAU | 116 | 2 |
| ^492^ | SER+PLB | 32 | 3 |
| ^492^ | RHO+SER | 66 | 3 |
| ^557^ | SER+LAV+DRA | 35 | 3 |
| ^557^ | SER+PLB | 35 | 3 |
| ^560^ | SER+THE | 30 | 3 |
| ^560^ | SER+PLB | 30 | 3 |
| ^568^ | SER+CUR | 23 | 3 |
| ^568^ | SER+PLB | 22 | 3 |
| ^584^ | FOL+FLUO | 62 | 4 |
| ^584^ | PLB+FLUO | 65 | 4 |
| ^582^ | THI+FLUO | 25 | 4 |
| ^582^ | PLB+FLUO | 26 | 4 |
| ^583^ | MG+FLUO | 17 | 4 |
| ^583^ | PLB+FLUO | 20 | 4 |
| ^579^ | LAV +PLB | 28 | 5 |
| ^579^ | CTP + PLB | 28 | 5 |
| ^581^ | CTP+FOL | 45 | 5 |
| ^581^ | CTP + PLB | 45 | 5 |
| ^589^ | CTP + PLB | 30 | 5 |
| ^589^ | CTP+OMEGA3 | 30 | 5 |
| ^531^ | TAU+CREA | 4 | 6 |
| ^531^ | TAU+PLB | 4 | 6 |
| ^535^ | TAU+SAR | 30 | 6 |
| ^535^ | TAU+PLB | 30 | 6 |
| ^475^ | SER + EPA | 71 | 7 |
| ^475^ | SER + PLB | 73 | 7 |
| ^494^ | SER + PLB | 19 | 7 |
| ^494^ | SER+CIN | 25 | 7 |
| ^586^ | CIT+CTP | 25 | 8 |
| ^586^ | PLB+CTP | 25 | 8 |
| ^585^ | vitC+CTP | 21 | 8 |
| ^585^ | PLB+CTP | 22 | 8 |
| ^587^ | OMEGA3+SER | 25 | 9 |
| ^587^ | PLB+SER | 25 | 9 |
| ^588^ | CAR+SER | 30 | 9 |
| ^588^ | PLB+SER | 30 | 9 |
| ^221^ | SUL+CLO | 43 | 10 |
| ^221^ | PLB+CLO | 47 | 10 |
| ^566^ | vitD3+TAU | 23 | 11 |
| ^566^ | PROB+TAU | 23 | 11 |
| ^598^ | ZNC+IMI | 30 | 12 |
| ^598^ | IMI+PLB | 30 | 12 |
| ^601^ | YUE+FLUO | 10 | 13 |
| ^601^ | FLUO+PLB | 8 | 13 |
| ^592^ | fAA+MIR | 20 | 14 |
| ^592^ | PLB+MIR | 20 | 14 |
| ^593^ | ESC+CREA | 25 | 15 |
| ^593^ | ESC+PLB | 27 | 15 |
| ^517^ | ESC + vitD | 31 | 16 |
| ^517^ | ESC + PLB | 28 | 16 |
| ^595^ | EPA+DHA+VEN | 36 | 17 |
| ^595^ | PLB+VEN | 36 | 17 |
| ^476^ | FOL+vitB12+vitB6+CTP | 77 | 18 |
| ^476^ | CTP+PLB | 76 | 18 |
| ^594^ | CUR+ESC | 50 | 19 |
| ^594^ | PLB+ESC | 50 | 19 |

**Reference for e-table 6**

1.	Açik M, Çakiroğlu FP, Tekin A, Egeli A. The effect of fish oil versus krill oil intervention on clinical symptoms and cardiometabolic risk factors in patients with major depressive disorder: A randomized placebo-controlled double-blind trial. Journal of Affective Disorders. 2025;380:171-80.

2.	Akhondzadeh Basti A, Moshiri E, Noorbala AA, Jamshidi AH, Abbasi SH, Akhondzadeh S. Comparison of petal of Crocus sativus L. and fluoxetine in the treatment of depressed outpatients: A pilot double-blind randomized trial. Progress in Neuro-Psychopharmacology and Biological Psychiatry. 2007;31(2):439-42.

3.	Akhondzadeh S, Fallah-Pour H, Afkham K, Jamshidi AH, Khalighi-Cigaroudi F. Comparison of Crocus sativus L. and imipramine in the treatment of mild to moderate depression: A pilot double-blind randomized trial [ISRCTN45683816]. BMC Complementary and Alternative Medicine. 2004;4.

4.	Akhondzadeh S, Mostafavi SA, Keshavarz SA, Mohammadi MR, Hosseini S, Eshraghian MR. A placebo controlled randomized clinical trial of Crocus sativus L. (saffron) on depression and food craving among overweight women with mild to moderate depression. Journal of Clinical Pharmacy and Therapeutics. 2020;45(1):134-43.

5.	Akhondzadeh S, Mostafavi SA, Keshavarz SA, Mohammadi MR, Chamari M. Melatonin Effects in Women With Comorbidities of Overweight, Depression, and Sleep Disturbance: A Randomized Placebo Controlled Clinical Trial. Sleep Med Res. 2022;13(1):22-30.

6.	Akkasheh G, Kashani-Poor Z, Tajabadi-Ebrahimi M, Jafari P, Akbari H, Taghizadeh M, et al. Clinical and metabolic response to probiotic administration in patients with major depressive disorder: A randomized, double-blind, placebo-controlled trial. Nutrition. 2016;32(3):315-20.

7.	Almeida OP, Ford AH, Hirani V, Singh V, VanBockxmeer FM, McCaul K, et al. B vitamins to enhance treatment response to antidepressants in middle-aged and older adults: Results from the B-VITAGE randomised, double-blind, placebo-controlled trial. British Journal of Psychiatry. 2014;205(6):450-7.

8.	Amini L, Yaghini O, Ghazavi M, Aslani N. L-carnitine versus Propranolol for pediatric migraine prophylaxis. Iran J Child Neurol. 2021;15(2):77-86.

9.	Anushiravani M, Manteghi AA, Taghipur A, Eslami M. Comparing Effectiveness of a Combined Herbal Drug Based on Echium Amoenum with Citalopram in the Treatment of Major Depressive Disorder. Curr Drug Discov Technol. 2019;16(2):232-8.

10.	Araj-Khodaei M, Noorbala AA, Yarani R, Emadi F, Emaratkar E, Faghihzadeh S, et al. A double-blind, randomized pilot study for comparison of Melissa officinalis L. And Lavandula angustifolia Mill. And Fluoxetine for the treatment of depression. BMC Complementary Medicine and Therapies. 2020;20(1).

11.	Baião R, Capitão LP, Higgins C, Browning M, Harmer CJ, Burnet PWJ. Multispecies probiotic administration reduces emotional salience and improves mood in subjects with moderate depression: a randomised, double-blind, placebo-controlled study. Psychological medicine. 2023;53(8):3437-47.

12.	Basoglu C, Ates MA, Algul A, Ipcioglu OM, Gecici O, Yilmaz O, et al. Adjuvant folate with escitalopram treatment and homocystein, folate, vitamin B-12 levels in patients with major depressive disorder. 2009;19(2):135‐42

C3 - EMBASE 354832073.

13.	Behnke K, Jensen GS, Graubaum HJ, Gruenwald J. Hypericum perforatum versus fluoxetine in the treatment of mild to moderate depression. Advances in Therapy. 2002;19(1):43-52.

14.	Bell KM, Plon L, Bunney Jr WE, Potkin SG. S-adenosylmethionine treatment of depression: A controlled clinical trial. American Journal of Psychiatry. 1988;145(9):1110-4.

15.	Bergman J, Miodownik C, Bersudsky Y, Sokolik S, Lerner PP, Kreinin A, et al. Curcumin as an add-on to antidepressive treatment: A randomized, double-blind, placebo-controlled, pilot clinical study. Clinical Neuropharmacology. 2013;36(3):73-7.

16.	Berk M, Dean OM, Cotton SM, Jeavons S, Tanious M, Kohlmann K, et al. The efficacy of adjunctive N-acetylcysteine in major depressive disorder: A double-blind, randomized, placebo-controlled trial. Journal of Clinical Psychiatry. 2014;75(6):628-36.

17.	Bidzan-Wiącek M, Tomczyk M, Błażek M, Mika A, Antosiewicz J. No Effects of Omega-3 Supplementation on Kynurenine Pathway, Inflammation, Depressive Symptoms, and Stress Response in Males: A Placebo-Controlled Trial. Nutrients. 2024;16(21 C7 - 3744).

18.	Bot M, Pouwer F, Assies J, Jansen EH, Diamant M, Snoek FJ, et al. Eicosapentaenoic acid as an add-on to antidepressant medication for co-morbid major depression in patients with diabetes mellitus: a randomized, double-blind placebo-controlled study. J Affect Disord. 2010;126(1-2):282-6.

19.	Brenner R, Azbel V, Madhusoodanan S, Pawlowska M. Comparison of an extract of hypericum (LI 160) and sertraline in the treatment of depression: A double-blind, randomized pilot study. Clinical Therapeutics. 2000;22(4):411-9.

20.	Caruso I, Fumagalli M, Boccassini L, Puttini SP, Santandrea S, Giniselli G, et al. Treatment of depression in rheumatoid arthritic patients. A comparison of S-adenosylmethionine (Samyr) and placebo in a double-blind study. CLIN TRIALS J. 1987;24(4):305-10.

21.	Chang JP, Chang SS, Yang HT, Chen HT, Chien YC, Yang B, et al. Omega-3 polyunsaturated fatty acids in cardiovascular diseases comorbid major depressive disorder - Results from a randomized controlled trial. Brain Behav Immun. 2020;85:14-20.

22.	Cheng YC, Chen WY, Lin C, Lee SH, Chiu CC, Kuo PH. The N-3 polyunsaturated fatty acids supplementation to prevent depression recurrence in patients with late-life depression: A 52-week double-blind, placebo-controlled trial. Journal of Affective Disorders. 2025;369:8-15.

23.	Chopra A, Kalyani AK. Comparison of efficacy of Crocus sativus L. with fluoxetine for improving depression among patients with cardiovascular diseases. Int J Life Sci Biotechnol Pharma Res. 2020;9(2):151-4.

24.	Darbinyan V, Aslanyan G, Amroyan E, Gabrielyan E, Malmström C, Panossian A. Clinical trial of Rhodiola rosea L. extract SHR-5 in the treatment of mild to moderate depression. Nordic Journal of Psychiatry. 2007;61(5):343-8.

25.	Davidson JR, Gadde KM, Fairbank JA, Krishnan KRR, Califf RM, Binanay C, et al. Effect of Hypericum perforatum (St John's wort) in major depressive disorder: a randomized controlled trial. 2002;287(14):1807‐14

C3 - PUBMED 11939866,EMBASE 34303625.

26.	Davidson JRT, Gadde KM, Fairbank JA, Ranga Rama Krishnan K, Califf RM, Binanay C, et al. Effect of Hypericum perforatum (St John's wort) in major depressive disorder: A randomized controlled trial. JAMA. 2002;287(14):1807-14.

27.	De Leo D. S-Adenosylmethionine as an antidepressant. A double-blind trial versus placebo. CURR THER RES CLIN EXP. 1987;41(6):865-70.

28.	Djokic G, Korcok D, Djordjevic V, Agic A, Rankovic A, Djukic Dejanovic S. The effects of s-adenosyl-L-methionine-vitamin b complex on mild and moderate depressive symptoms. Hippokratia. 2017;21(3):140-3.

29.	Docherty JP, Sack DA, Roffman M, Finch M, Komorowski JR. A double-blind, placebo-controlled, exploratory trial of chromium picolinate in atypical depression: Effect on carbohydrate craving. Journal of Psychiatric Practice. 2005;11(5):302-14.

30.	Dormal V, Jonniaux L, Buchet M, Simar L, Copine S, Deldicque L. Effect of Hydroponically Grown Red Panax Ginseng on Perceived Stress Level, Emotional Processing, and Cognitive Functions in Moderately Stressed Adults: A Randomized, Double-Blind, Placebo-Controlled Study. Nutrients. 2025;17(6 C7 - 955).

31.	Fava M, Alpert J, Nierenberg AA, Mischoulon D, Otto MW, Zajecka J, et al. A double-blind, randomized trial of St John's wort, fluoxetine, and placebo in major depressive disorder. Journal of Clinical Psychopharmacology. 2005;25(5):441-7.

32.	Freeman MP, Davis M, Sinha P, Wisner KL, Hibbeln JR, Gelenberg AJ. Omega-3 fatty acids and supportive psychotherapy for perinatal depression: A randomized placebo-controlled study. Journal of Affective Disorders. 2008;110(1-2):142-8.

33.	Friede M, Henneicke von Zepelin HH, Freudenstein J. Differential therapy of mild to moderate depressive episodes (ICD-10 F 32.0; F 32.1) with St. John's wort. Pharmacopsychiatry. 2001;34(SUPPL. 1):S38-S41.

34.	Gelenberg AJ, Shelton RC, Crits-Christoph P, Keller MB, Dunner DL, Hirschfeld RM, et al. The effectiveness of St. John's Wort in major depressive disorder: a naturalistic phase 2 follow-up in which nonresponders were provided alternate medication. 2004;65(8):1114‐9

C3 - PUBMED 15323598.

35.	Ghajar A, Neishabouri SM, Velayati N, Jahangard L, Matinnia N, Haghighi M, et al. Crocus sativus L versus Citalopram in the Treatment of Major Depressive Disorder with Anxious Distress: A Double-Blind, Controlled Clinical Trial. Pharmacopsychiatry. 2017;50(4):152-60.

36.	Ghorbani Z, Nazari S, Etesam F, Nourimajd S, Ahmadpanah M, Jahromi SR. The effect of synbiotic as an adjuvant therapy to fluoxetine in moderate depression: A randomized multicenter trial. Arch Neurosci. 2018;5(2).

37.	Gul A, Memtily N, Mijit P, Imin M, Rui H, Wushuer P, et al. The Üstikuddus sherbiti in the treatment of abnormal cold and dry-type depression patients with comorbid anxiety: A randomized and controlled clinical trial. Trad Med Mod Med. 2019;2(3):127-33.

38.	Han CH, Chung MI, Bose S, Lim C, Kim H, Koo BS, et al. The effect of Gami Guibitang (GGBT), a medicinal herbal formulation, on moderate depression. European Journal of Integrative Medicine. 2012;4(4):e400-e7.

39.	Harrer G, Schmidt U, Kuhn U, Biller A. Comparison of equivalence between the St. John's wort extract LoHyp-57 and fluoxetine. Arzneimittel-Forschung/Drug Research. 1999;49(4):289-96.

40.	He X, Liu D, Ni S, Li Z, Li S, Wu T, et al. Efficacy and safety evaluation of Ginkgo biloba dropping pill (GBDP) on stable angina pectoris complicated with depression: A placebo-controlled, randomized, double-blind, multicenter study. Phytomedicine. 2024;126.

41.	Hosseini H, Bagherniya M, Sahebkar A, Iraj B, Majeed M, Askari G. The effect of curcumin-piperine supplementation on lipid profile, glycemic index, inflammation, and blood pressure in patients with type 2 diabetes mellitus and hypertriglyceridemia. Phytotherapy Research. 2024;38(11):5150-61.

42.	Hu Y, Chen C, Wang Y, Yang W, Wang Y, Zhu W, et al. The effects of KaiXinSan on depression and its association with lipid profiles: a randomized, double-blinded, placebo-controlled trial. 2021;83:153467

C3 - PUBMED 33516143,EMBASE 2010802711.

43.	Hvas AM, Juul S, Lauritzen L, Nexø E, Ellegaard J. No effect of vitamin B-12 treatment on cognitive function and depression: A randomized placebo controlled study. Journal of Affective Disorders. 2004;81(3):269-73.

44.	Jazayeri S, Tehrani-Doost M, Keshavarz SA, Hosseini M, Djazayery A, Amini H, et al. Comparison of therapeutic effects of omega-3 fatty acid eicosapentaenoic acid and fluoxetine, separately and in combination, in major depressive disorder. Australian and New Zealand Journal of Psychiatry. 2008;42(3):192-8.

45.	Jelodar G, Javid Z, Sahraian A, Jelodar S. Saffron improved depression and reduced homocysteine level in patients with major depression: A Randomized, double-blind study. Avicenna J Phytomedicine. 2018;8(1):43-50.

46.	Kagan BL, Sultzer DL, Rosenlicht N, Gerner RH. Oral S-adenosylmethionine in depression: a randomized, double-blind, placebo-controlled trial. 1990;147(5):591‐5

C3 - PUBMED 2183633.

47.	Kalb R, Trautmann-Sponsel RD, Kieser M. Efficacy and tolerability of Hypericum extract WS 5572 versus placebo in mildly to moderately depressed patients: A randomized double-blind multicenter clinical trial. Pharmacopsychiatry. 2001;34(3):96-103.

48.	Kanchanatawan B, Tangwongchai S, Sughondhabhirom A, Suppapitiporn S, Hemrunrojn S, Carvalho AF, et al. Add-on Treatment with Curcumin Has Antidepressive Effects in Thai Patients with Major Depression: Results of a Randomized Double-Blind Placebo-Controlled Study. Neurotoxic Res. 2018;33(3):621-33.

49.	Kashani L, Esalatmanesh S, Eftekhari F, Salimi S, Foroughifar T, Etesam F, et al. Efficacy of Crocus sativus (saffron) in treatment of major depressive disorder associated with post-menopausal hot flashes: a double-blind, randomized, placebo-controlled trial. Arch Gynecol Obstet. 2018;297(3):717-24.

50.	Kasper S, Anghelescu IG, Szegedi A, Dienel A, Kieser M. Superior efficacy of St John's wort extract WS 5570 compared to placebo in patients with major depression: a randomized, double-blind, placebo-controlled, multi-center trial. 2006;4:14

C3 - PUBMED 16796730,EMBASE 44227549.

51.	Kasper S, Anghelescu IG, Szegedi A, Dienel A, Kieser M. Placebo controlled continuation treatment with Hypericum extract WS® 5570 after recovery from a mild or moderate depressive episode. Wiener Medizinische Wochenschrift. 2007;157(13-14):362-6.

52.	Kasper S, Anghelescu I, Dienel A. Efficacy of orally administered Silexan in patients with anxiety-related restlessness and disturbed sleep--A randomized, placebo-controlled trial. 2015;25(11):1960‐7

C3 - PUBMED 26293583,EMBASE 605643089.

53.	Kasper S, Volz HP, Möller HJ, Schläfke S, Klement S, Anghelescu IG, et al. Lavender oil preparation Silexan is effective in mild-to-moderate major depression: a randomized, placebo- and reference-controlled trial. European Archives of Psychiatry and Clinical Neuroscience. 2024.

54.	Kaviani M, Nikooyeh B, Etesam F, Behnagh SJ, Kangarani HM, Arefi M, et al. Effects of vitamin D supplementation on depression and some selected pro-inflammatory biomarkers: a double-blind randomized clinical trial. BMC Psychiatry. 2022;22(1 C7 - 694).

55.	Khoraminya N, Tehrani-Doost M, Jazayeri S, Hosseini A, Djazayery A. Therapeutic effects of vitamin D as adjunctive therapy to fluoxetine in patients with major depressive disorder. Australian and New Zealand Journal of Psychiatry. 2013;47(3):271-5.

56.	Kolouri S, Firoozabadi A, Salehi A, Zarshenas MM, Dastgheib SA, Heydari M, et al. Nepeta menthoides Boiss. & Buhse freeze-dried aqueous extract versus sertraline in the treatment of major depression: A double blind randomized controlled trial. Complement Ther Med. 2016;26:164-70.

57.	Kreuzer K, Reiter A, Birkl-Töglhofer AM, Dalkner N, Mörkl S, Mairinger M, et al. The PROVIT Study—Effects of Multispecies Probiotic Add-on Treatment on Metabolomics in Major Depressive Disorder—A Randomized, Placebo-Controlled Trial. Metabolites. 2022;12(8).

58.	Laakmann G. St. John's Wort in mild to moderate depression: The relevance of hyperforin for the clinical efficacy. Pharmacopsychiatry. 1998;31(SUPPL. 1):54-9.

59.	Lecrubier Y, Clerc G, Didi R, Kieser M. Efficacy of St. John's wort extract WS 5570 in major depression: A double-blind, placebo-controlled trial. American Journal of Psychiatry. 2002;159(8):1361-6.

60.	Levine J, Barak Y, Gonzalves M, Szor H, Elizur A, Kofman O, et al. Double-blind, controlled trial of inositol treatment of depression. American Journal of Psychiatry. 1995;152(5):792-4.

61.	Levine J, Barak Y, Kofman O, Belmaker RH. Follow-up and relapse analysis of an inositol study of depression. Isr J Psychiatry Relat Sci. 1995;32(1):14-21.

62.	Lin CH, Wang SH, Lane HY. Effects of Sodium Benzoate, a D-Amino Acid Oxidase Inhibitor, on Perceived Stress and Cognitive Function Among Patients With Late-Life Depression: A Randomized, Double-Blind, Sertraline- and Placebo-Controlled Trial. International Journal of Neuropsychopharmacology. 2022;25(7):545-55.

63.	Lôo H, Poirier MF, Ollat H, Elatki S. Effects of sulbutiamine (Arcalion 200) on psycho-behavioral inhibition in major depressive episodes. 2000;26(2):70‐5

C3 - PUBMED 10858919,EMBASE 30337124.

64.	Lopresti AL, Drummond PD. Efficacy of curcumin, and a saffron/curcumin combination for the treatment of major depression: A randomised, double-blind, placebo-controlled study. Journal of Affective Disorders. 2017;207:188-96.

65.	Mao JJ, Xie SX, Zee J, Soeller I, Li QS, Rockwell K, et al. Rhodiola rosea versus sertraline for major depressive disorder: A randomized placebo-controlled trial. Phytomedicine. 2015;22(3):394-9.

66.	Marangell LB, Martinez JM, Zboyan HA, Kertz B, Kim HFS, Puryear LJ. A double-blind, placebo-controlled study of the omega-3 fatty acid docosahexaenoic acid in the treatment of major depression. American Journal of Psychiatry. 2003;160(5):996-8.

67.	Mazidi M, Shemshian M, Mousavi SH, Norouzy A, Kermani T, Moghiman T, et al. A double-blind, randomized and placebo-controlled trial of Saffron (Crocus sativus L.) in the treatment of anxiety and depression. 2016;13(2):195‐9

C3 - PUBMED 27101556,EMBASE 610483123.

68.	Mech AW, Farah A. Correlation of clinical response with homocysteine reduction during therapy with reduced b vitamins in patients with mdd who are positive for MTHFR C677T or A1298C polymorphism: A Randomized, double-blind, placebo-controlled study. Journal of Clinical Psychiatry. 2016;77(5):668-71.

69.	Mischoulon D, Best-Popescu C, Laposata M, Merens W, Murakami JL, Wu SL, et al. A double-blind dose-finding pilot study of docosahexaenoic acid (DHA) for major depressive disorder. European Neuropsychopharmacology. 2008;18(9):639-45.

70.	Mischoulon D, Dunlop BW, Kinkead B, Schettler PJ, Lamon-Fava S, Rakofsky JJ, et al. Omega-3 Fatty Acids for Major Depressive Disorder With High Inflammation: A Randomized Dose-Finding Clinical Trial. Journal of Clinical Psychiatry. 2022;83(5 C7 - 21m14074).

71.	Mischoulon D, Price LH, Carpenter LL, Tyrka AR, Papakostas GI, Baer L, et al. A double-blind, randomized, placebo-controlled clinical trial of S-adenosyl-L-methionine (SAMe) versus escitalopram in major depressive disorder. J Clin Psychiatry. 2014;75(4):370-6.

72.	Moshiri E, Basti AA, Noorbala AA, Jamshidi AH, Hesameddin Abbasi S, Akhondzadeh S. Crocus sativus L. (petal) in the treatment of mild-to-moderate depression: A double-blind, randomized and placebo-controlled trial. Phytomedicine. 2006;13(9-10):607-11.

73.	Mozaffari-Khosravi H, Nabizade L, Yassini-Ardakani SM, Hadinedoushan H, Barzegar K. The effect of 2 different single injections of high dose of vitamin D on improving the depression in depressed patients with vitamin D deficiency: A randomized clinical trial. Journal of Clinical Psychopharmacology. 2013;33(3):378-85.

74.	Ning H, Zhou H, Ren J, Zhou G, Yang N, Wang Z, et al. Zishen pingchan granules combined with pramipexole in the improvement of depressive symptoms in Parkinson's disease: a prospective, multicenter, randomized, double-blind, controlled clinical study. J Transl Med. 2022;20(1):357.

75.	Noorbala AA, Akhondzadeh S, Tahmacebi-Pour N, Jamshidi AH. Hydro-alcoholic extract of Crocus sativus L. versus fluoxetine in the treatment of mild to moderate depression: A double-blind, randomized pilot trial. Journal of Ethnopharmacology. 2005;97(2):281-4.

76.	Nowak G, Siwek M, Dudek D, Zieba A, Pilc A. Effect of zinc supplementation on antidepressant therapy in unipolar depression: A preliminary placebo-controlled study. Polish Journal of Pharmacology. 2003;55(6):1143-7.

77.	Pancheri P, Scapicchio P, Delle Chiaie R. A double-blind, randomized parallel-group, efficacy and safety study of intramuscular S-adenosyl-L-methionine 1, 4-butanedisulphonate (SAMe) versus imipramine in patients with major depressive disorder. International Journal of Neuropsychopharmacology. 2002;5(4):287-94.

78.	Papakostas GI, Shelton RC, Zajecka JM, Etemad B, Rickels K, Clain A, et al. L-methylfolate as adjunctive therapy for SSRI-resistant major depression: results of two randomized, double-blind, parallel-sequential trials. 2012;169(12):1267‐74

C3 - PUBMED 23212058.

79.	Piperoglou M, Hopwood M, Norman TR. Adjunctive Docosahexaenoic Acid in Residual Symptoms of Depression and Anxiety. Journal of Clinical Psychopharmacology. 2023;43(6):493-7.

80.	Rees AM, Austin MP, Parker GB. Omega-3 fatty acids as a treatment for perinatal depression: Randomized double-blind placebo-controlled trial. Australian and New Zealand Journal of Psychiatry. 2008;42(3):199-205.

81.	Rondanelli M, Giacosa A, Opizzi A, Pelucchi C, Vecchia CL, Montorfano G, et al. Effect of omega-3 fatty acids supplementation on depressive symptoms and on health-related quality of life in the treatment of elderly women with depression: A double-blind, placebo-controlled, randomized clinical trial. Journal of the American College of Nutrition. 2010;29(1):55-64.

82.	Saccarello A, Montarsolo P, Massardo I, Picciotto R, Pedemonte A, Castagnaro R, et al. Oral Administration of S-Adenosylmethionine (SAMe) and Lactobacillus Plantarum HEAL9 Improves the Mild-To-Moderate Symptoms of Depression: a Randomized, Double-Blind, Placebo-Controlled Study. 2020;22(4 C3 - PUBMED 32589828,EMBASE 632201612).

83.	Sadeghi A, Ghorayshi F, Baghshahi H, Akbari H, Memarzadeh MR, Taghizadeh M, et al. The antidepressant effect of combined extracts of Hypericum perforatum and Echium amoenum supplementation in patients with depression symptoms: A randomized clinical trial. Avicenna J Phytomedicine. 2023;13(4):328-37.

84.	Sahoo JP, Jarnail S, Khurana H, Singh P. Omega-3 Fatty Acids Versus 5-hydroxytryptophan as Add on Therapy in Patients with Depression: A Comparative Study. Indian Journal of Pharmacology. 2015;47(7):S23-S4.

85.	Sahraian A, Ghanizadeh A, Kazemeini F. Vitamin C as an adjuvant for treating major depressive disorder and suicidal behavior, a randomized placebo-controlled clinical trial. Trials. 2015;16(1 C7 - 94).

86.	Sakurai H, L. Carpenter L, R. Tyrka A, Price LH, I. Papakostas G, Dording CM, et al. Dose increase of S-Adenosyl-Methionine and escitalopram in a randomized clinical trial for major depressive disorder. Journal of Affective Disorders. 2020;262:118-25.

87.	Sanmukhani J, Satodia V, Trivedi J, Patel T, Tiwari D, Panchal B, et al. Efficacy and safety of curcumin in major depressive disorder: a randomized controlled trial. Phytother Res. 2014;28(4):579-85.

88.	Sarris J, Byrne GJ, Stough C, Bousman C, Mischoulon D, Murphy J, et al. Nutraceuticals for major depressive disorder- more is not merrier: An 8-week double-blind, randomised, controlled trial. Journal of Affective Disorders. 2019;245:1007-15.

89.	Sarris J, Fava M, Schweitzer I, Mischoulon D. St Johns Wort (Hypericum perforatum) versus sertraline and placebo in major depressive disorder: Continuation data from a 26-week RCT. Pharmacopsychiatry. 2012;45(7):275-8.

90.	Sarris J, Murphy J, Stough C, Mischoulon D, Bousman C, MacDonald P, et al. S-Adenosylmethionine (SAMe) monotherapy for depression: an 8-week double-blind, randomised, controlled trial. 2020;237(1):209‐18

C3 - PUBMED 31712971,EMBASE 2003633556.

91.	Schrader E. Equivalence of St John's wort extract (Ze 117) and fluoxetine: A randomized, controlled study in mild-moderate depression. International Clinical Psychopharmacology. 2000;15(2):61-8.

92.	Schrader E, Meier B, Brattström A. Hypericum treatment of mild-moderate depression in a placebo-controlled study. A prospective, double-blind, randomized, placebo-controlled, multicentre study. Human Psychopharmacology. 1998;13(3):163-9.

93.	Sepehrmanesh Z, Kolahdooz F, Abedi F, Mazroii N, Assarian A, Asemi Z, et al. Vitamin D supplementation affects the beck depression inventory, insulin resistance, and biomarkers of oxidative stress in patients with major depressive disorder: A randomized, controlled clinical trial. Journal of Nutrition. 2016;146(2):243-8.

94.	Shahmansouri N, Farokhnia M, Abbasi SH, Kassaian SE, Noorbala Tafti AA, Gougol A, et al. A randomized, double-blind, clinical trial comparing the efficacy and safety of Crocus sativus L. with fluoxetine for improving mild to moderate depression in post percutaneous coronary intervention patients. 2014;155:216‐22

C3 - PUBMED 24289892,EMBASE 52891114.

95.	Shahmoradi N, Davarinejad O, Brühl AB, Brand S. Effects of Aphrodite (an Herbal Compound) on SSRI-Induced Sexual Dysfunctions and Depression in Females with Major Depressive Disorder: Findings from a Randomized Clinical Trial. Medicina (Kaunas, Lithuania). 2023;59(9).

96.	Sharifan P, Hosseini MS, Sharifan A. The interventional relationship between frequent fish consumption and depression symptoms in aging adults: a randomized controlled trial. International Journal of Geriatric Psychiatry. 2017;32(12):e116-e22.

97.	Shelton RC, Keller MB, Gelenberg A, Dunner DL, Hirschfeld R, Thase ME, et al. Effectiveness of St John's wort in major depression: A randomized controlled trial. JAMA. 2001;285(15):1978-86.

98.	Sherwood Brown E, Gabrielson B. A randomized, double-blind, placebo-controlled trial of citicoline for bipolar and unipolar depression and methamphetamine dependence. Journal of Affective Disorders. 2012;143(1-3):257-60.

99.	Shinto L, Marracci G, Mohr DC, Bumgarner L, Murchison C, Senders A, et al. Omega-3 fatty acids for depression in multiple sclerosis: A randomized pilot study. PLoS ONE. 2016;11(1 C7 - e0147195).

100.	Su KP, Huang SY, Chiu CC, Shen WW. Omega-3 fatty acids in major depressive disorder: A preliminary double-blind, placebo-controlled trial. European Neuropsychopharmacology. 2003;13(4):267-71.

101.	Su KP, Huang SY, Chiu TH, Huang KC, Huang CL, Chang HC, et al. Omega-3 fatty acids for major depressive disorder during pregnancy: Results from a randomized, double-blind, placebo-controlled trial. Journal of Clinical Psychiatry. 2008;69(4):644-51.

102.	Szegedi A, Kohnen R, Dienel A, Kieser M. Acute treatment of moderate to severe depression with hypericum extract WS 5570 (St John's wort): Randomised controlled double blind non-inferiority trial versus paroxetine. British Medical Journal. 2005;330(7490):503-6.

103.	Tarutani S, Omori M, Ido Y, Yano M, Komatsu T, Okamura T. Effects of 4G-beta-D-Galactosylsucrose in patients with depression: A randomized, double-blinded, placebo-controlled, parallel-group comparative study. J Psychiatr Res. 2022;148:110-20.

104.	Vaghef-Mehrabany E, Ranjbar F, Asghari-Jafarabadi M, Hosseinpour-Arjmand S, Ebrahimi-Mameghani M. Calorie restriction in combination with prebiotic supplementation in obese women with depression: effects on metabolic and clinical response. Nutritional Neuroscience. 2021;24(5):339-53.

105.	van Gurp G, Meterissian GB, Haiek LN, McCusker J, Bellavance F. St John's wort or sertraline? Randomized controlled trial in primary care. Can Fam Physician. 2002;48:905-12.

106.	Varzaghani V, Sharifi M, Hajiaghaee R, Bagheri S, Momtaz S, Tarassoli Z, et al. Propolis add-on therapy alleviates depressive symptoms; A randomized placebo-controlled clinical trial. Phytotherapy Research. 2022;36(3):1258-67.

107.	Verma DK, Das B. Effect of zinc supplementation on antidepressant therapy and serum zinc level in unipolar depression: A randomized double blind controlled study. Indian Journal of Psychiatry. 2016;58(5):S47.

108.	Vorbach EU, Arnoldt KH, Hubner WD. Efficacy and tolerability of St. John's wort extract LI 160 versus imipramine in patients with severe depressive episodes according to ICD-10. Pharmacopsychiatry. 1997;30(SUPPL.):81-5.

109.	Wang L, Bordi PL, Fleming JA, Hill AM, Kris-Etherton PM. Effect of a moderate fat diet with and without avocados on lipoprotein particle number, size and subclasses in overweight and obese adults: a randomized, controlled trial. J Am Heart Assoc. 2015;4(1):e001355.

110.	Wheatley D. LI 160, an extract of St. John's wort, versus amitriptyline in mildly to moderately depressed outpatients--a controlled 6-week clinical trial. 1997;30 Suppl 2:77‐80

C3 - PUBMED 9342764.

111.	Woelk H. Comparison of St John's wort and imipramine for treating depression: Randomised controlled trial. British Medical Journal. 2000;321(7260):536-9.

112.	Wu SK, Yang KJ, Liu WC, Malau IA, Zailani H, Chang CH, et al. The Efficacy of Omega-3 Fatty Acids as the Monotherapy for Depression: A Randomized, Double-Blind, Placebo-Controlled Pilot Study. Nutrients. 2024;16(21).

113.	Zhang L, Wang S, Zhu Y, Yang T. Vitamin D3 as adjunctive therapy in the treatment of depression in tuberculosis patients: A short-term pilot randomized double-blind controlled study. Neuropsychiatric Disease and Treatment. 2018;14:3103-9.

114.	Antidepressant Effects of Ayahuasca: a Randomized Placebo Controlled Trial in Treatment Resistant Depression. clinicaltrialsgov2016.

115.	Ahmadpanah M, Ramezanshams F, Ghaleiha A, Akhondzadeh S, Sadeghi Bahmani D, Brand S. Crocus Sativus L. (saffron) versus sertraline on symptoms of depression among older people with major depressive disorders–a double-blind, randomized intervention study. Psychiatry Research. 2019;282.

116.	Firoozabadi A, Zarshenas MM, Salehi A, Jahanbin S, Mohagheghzadeh A. Effectiveness of Cuscuta planiflora Ten. and Nepeta menthoides Boiss. & Buhse in major depression: a triple-blind randomized controlled trial study. J Evid Based Complementary Altern Med. 2015;20(2):94-7.

117.	Grenyer BF, Crowe T, Meyer B, Owen AJ, Grigonis-Deane EM, Caputi P, et al. Fish oil supplementation in the treatment of major depression: a randomised double-blind placebo-controlled trial. 2007;31(7):1393‐6

C3 - PUBMED 17659823.

118.	Kashani L, Eslatmanesh S, Saedi N, Niroomand N, Ebrahimi M, Hosseinian M, et al. Comparison of Saffron versus Fluoxetine in Treatment of Mild to Moderate Postpartum Depression: A Double-Blind, Randomized Clinical Trial. Pharmacopsychiatry. 2017;50(2):64-8.

119.	Li Y, Li L, Guo R, Yu H, Wang X, Wang B, et al. Clinical efficacy of Shugan granule in the treatment of mixed anxiety-depressive disorder: a multicenter, randomized, double-blind, placebo-controlled trial. 2022;290:115032

C3 - PUBMED 35085742,EMBASE 637131287.

120.	Lin SKK, Kuo PH, Hsu CY, Chiu YH, Chen CH. The effects of Lactobacillus plantarum PS128 in patients with major depressive disorder: an eight-week double-blind, placebo-controlled study. Asian Journal of Psychiatry. 2024;101 C7 - 104210.

121.	Majmasanaye M, Mehrpooya M, Amiri H, Eshraghi A. Discovering the Potential Value of Coenzyme Q10 as an Adjuvant Treatment in Patients with Depression. Journal of Clinical Psychopharmacology. 2024;44(3):232-9.

122.	Masoumi SZ, Kazemi F, Tavakolian S, Rahimi A, Oshvandi K, Soltanian A, et al. Effect of citalopram in combination with omega-3 on depression in post-menopausal women: A triple blind randomized controlled trial. Journal of Clinical and Diagnostic Research. 2016;10(10):QC01-QC5.

123.	Nemets B, Stahl Z, Belmaker RH. Addition of omega-3 fatty acid to maintenance medication treatment for recurrent unipolar depressive disorder. American Journal of Psychiatry. 2002;159(3):477-9.

124.	Panahi Y, Badeli R, Karami GR, Badeli Z, Sahebkar A. A randomized controlled trial of 6-week Chlorella vulgaris supplementation in patients with major depressive disorder. Complement Ther Med. 2015;23(4):598-602.

125.	Papakostas GI, Mischoulon D, Shyu I, Alpert JE, Fava M. S-adenosyl methionine (SAMe) augmentation of serotonin reuptake inhibitors for antidepressant nonresponders with major depressive disorder: a double-blind, randomized clinical trial. 2010;167(8):942‐8

C3 - PUBMED 20595412.

126.	Park Y, Park YS, Kim SH, Oh DH, Park YC. Supplementation of n-3 polyunsaturated fatty acids for major depressive disorder: A randomized, double-blind, 12-week, placebo-controlled trial in Korea. Annals of Nutrition and Metabolism. 2015;66(2-3):141-8.

127.	Ranjbar E, Shams J, Sabetkasaei M, M-Shirazi M, Rashidkhani B, Mostafavi A, et al. Effects of zinc supplementation on efficacy of antidepressant therapy, inflammatory cytokines, and brain-derived neurotrophic factor in patients with major depression. Nutritional Neuroscience. 2014;17(2):65-71.

128.	Rudzki L, Ostrowska L, Pawlak D, Małus A, Pawlak K, Waszkiewicz N, et al. Probiotic Lactobacillus Plantarum 299v decreases kynurenine concentration and improves cognitive functions in patients with major depression: A double-blind, randomized, placebo controlled study. Psychoneuroendocrinology. 2019;100:213-22.

129.	Sarris J, Byrne GJ, Bousman C, Stough C, Murphy J, MacDonald P, et al. Adjunctive S-adenosylmethionine (SAMe) in treating non-remittent major depressive disorder: An 8-week double-blind, randomized, controlled trial. European Neuropsychopharmacology. 2018;28(10):1126-36.

130.	Sempach L, Schaub A-C, Brühl AB, Sterzer P, Lang UE, Schmidt A. Adjunctive d-serine treatment for major depressive disorder: A randomized clinical trial. Journal of Affective Disorders. 2025:119504.

131.	Talaei A, Hassanpour Moghadam M, Sajadi Tabassi SA, Mohajeri SA. Crocin, the main active saffron constituent, as an adjunctive treatment in major depressive disorder: A randomized, double-blind, placebo-controlled, pilot clinical trial. Journal of Affective Disorders. 2015;174:51-6.

132.	Targum SD, Cameron BR, Ferreira L, MacDonald ID. An augmentation study of MSI-195 (S-adenosylmethionine) in Major Depressive Disorder. Journal of Psychiatric Research. 2018;107:86-96.

133.	Gao L, Wu C, Liao Y, Wang J. Antidepressants effects of Rhodiola capsule combined with sertraline for major depressive disorder: A randomized double-blind placebo-controlled clinical trial. Journal of Affective Disorders. 2020;265:99-103.

134.	Shafiee-Kandjani A, Khalili M, Malek A, Farhang S, Ranjbari Y, Khalili Y. The therapeutic effect of the extracts of Lavandula angustifolia and Dracocephalum ruyschiana besides sertraline on patients with mild to moderate depression: A double-blind controlled trial. Phytomedicine Plus. 2023;3(2).

135.	Shamabadi A, Fattollahzadeh-Noor S, Fallahpour B, A. Basti F, Khodaei Ardakani MR, Akhondzadeh S. L-Theanine adjunct to risperidone in the treatment of chronic schizophrenia inpatients: a randomized, double-blind, placebo-controlled clinical trial. Psychopharmacology. 2023;240(12):2631-40.

136.	Talaei A, Noori R, Rezaei Ardani A, Mohammadpour AH, Azimipoor Z, Afzaljavan F. The Effect of Adding Curcumin to Sertraline in the Treatment of Severe Major Depressive Disorder: A Randomized, Double-Blind Clinical Trial. Clinical Neuropharmacology. 2023;46(4):135-9.

137.	Coppen A, Bailey J. Enhancement of the antidepressant action of fluoxetine by folic acid: A randomised, placebo controlled trial. Journal of Affective Disorders. 2000;60(2):121-30.

138.	Ghaleiha A, Davari H, Jahangard L, Haghighi M, Ahmadpanah M, Seifrabie MA, et al. Adjuvant thiamine improved standard treatment in patients with major depressive disorder: results from a randomized, double-blind, and placebo-controlled clinical trial. European Archives of Psychiatry and Clinical Neuroscience. 2016;266(8):695-702.

139.	Ryszewska-Pokraśniewicz B, Mach A, Skalski M, Januszko P, Wawrzyniak ZM, Poleszak E, et al. Effects of magnesium supplementation on unipolar depression: A placebo-controlled study and review of the importance of dosing and magnesium status in the therapeutic response. Nutrients. 2018;10(8 C7 - 1014).

140.	Firoozeei TS, Barekatain M, Karimi M, Zargaran A, Akhondzadeh S, Rezaeizadeh H. Lavender and dodder combined herbal syrup versus citalopram in major depressive disorder with anxious distress: A double-blind randomized trial. Journal of Integrative Medicine. 2020;18(5):409-15.

141.	Sepehrmanesh Z, Kolahdooz F, Abedi F, Mazroii N, Assarian A, Asemi Z, et al. Vitamin D Supplementation Affects the Beck Depression Inventory, Insulin Resistance, and Biomarkers of Oxidative Stress in Patients with Major Depressive Disorder: A Randomized, Controlled Clinical Trial [Retraction notice in CN-02298463]. 2016;146(2):243‐8

C3 - PUBMED 26609167,EMBASE 608794678.

142.	Vellekkatt F, Menon V, Rajappa M, Sahoo J. Effect of adjunctive single dose parenteral Vitamin D supplementation in major depressive disorder with concurrent vitamin D deficiency: A double-blind randomized placebo-controlled trial. J Psychiatr Res. 2020;129:250-6.

143.	Nemets B, Levine J. A pilot dose-finding clinical trial of creatine monohydrate augmentation to SSRIs/SNRIs/NASA antidepressant treatment in major depression. International Clinical Psychopharmacology. 2013;28(3):127-33.

144.	Padhan M, Mohapatra D, Mishra BR, Maiti R, Jena M. Efficacy and safety of add-on sarcosine in patients with major depressive disorder: A randomized controlled trial. Journal of Psychiatric Research. 2024;178:298-304.

145.	Carney RM, Freedland KE, Rubin EH, Rich MW, Steinmeyer BC, Harris WS. A randomized placebo-controlled trial of omega-3 and sertraline in depressed patients with or at risk for coronary heart disease. Journal of Clinical Psychiatry. 2019;80(4 C7 - 19m12742).

146.	Ghaffari S, Ghobadi A, Jamshidi AH, Mortazavi SH, Naderi S, Aqamolaei A, et al. Cinnamomum tamala as an adjuvant therapy in the treatment of major depressive disorder: A double-blind, randomized, placebo-controlled clinical trial with placebo control. Advances in Integrative Medicine. 2020;7(3):141-7.

147.	Roohi-Azizi M, Arabzadeh S, Amidfar M, Salimi S, Zarindast MR, Talaei A, et al. Citicoline combination therapy for major depressive disorder: A randomized, double-blind, placebo-controlled trial. Clinical Neuropharmacology. 2017;40(1):1-5.

148.	Jahangard L, Sadeghi A, Ahmadpanah M, Holsboer-Trachsler E, Sadeghi Bahmani D, Haghighi M, et al. Influence of adjuvant omega-3-polyunsaturated fatty acids on depression, sleep, and emotion regulation among outpatients with major depressive disorders - Results from a double-blind, randomized and placebo-controlled clinical trial. Journal of Psychiatric Research. 2018;107:48-56.

149.	Ramezani A, Kheirkhah F, Zabihi E, Shirafkan H, Moudi S. Effectiveness of L-carnitine Supplementation to Sertraline for Treatment of Major Depressive Disorder: A Double-blind Randomized Placebo-controlled Trial. Glob J Med Pharm Biomed Update. 2022;17.

150.	Lopresti AL, Maes M, Maker GL, Hood SD, Drummond PD. Curcumin for the treatment of major depression: A randomised, double-blind, placebo controlled study. Journal of Affective Disorders. 2014;167:368-75.

151.	Sultana S, Rahman MS. Effect of magnesium supplementation in selective serotonin reuptake inhibitors treated major depressive disorder patients: A randomized, double-blind, placebo-controlled trial. EC Nutr. 2023;18:25-34.

152.	Siwek M, Dudek D, Schlegel-Zawadzka M, Morawska A, Piekoszewski W, Opoka W, et al. Serum zinc level in depressed patients during zinc supplementation of imipramine treatment. Journal of Affective Disorders. 2010;126(3):447-52.

153.	Wu R, Zhu D, Xia Y, Wang H, Tao W, Xue W, et al. A role of Yueju in fast-onset antidepressant action on major depressive disorder and serum BDNF expression: A randomly double-blind, fluoxetine-adjunct, placebo-controlled, pilot clinical study. Neuropsychiatric Disease and Treatment. 2015;11:2013-21.

154.	Ille R, Spona J, Zickl M, Hofmann P, Lahousen T, Dittrich N, et al. "Add-On"-therapy with an individualized preparation consisting of free amino acids for patients with a major depression. European Archives of Psychiatry and Clinical Neuroscience. 2007;257(4):222-9.

155.	Lyoo IK, Yoon S, Kim TS, Hwang J, Kim JE, Won W, et al. A randomized, double-blind placebo-controlled trial of oral creatine monohydrate augmentation for enhanced response to a selective serotonin reuptake inhibitor in women with major depressive disorder. American Journal of Psychiatry. 2012;169(9):937-45.

156.	Kumar PNS, Menon V, Andrade C. A randomized, double-blind, placebo-controlled, 12-week trial of vitamin D augmentation in major depressive disorder associated with vitamin D deficiency. Journal of Affective Disorders. 2022;314:143-9.

157.	Yang R, Wang L, Jin K, Cao S, Wu C, Guo J, et al. Omega-3 Polyunsaturated Fatty Acids Supplementation Alleviate Anxiety Rather Than Depressive Symptoms Among First-Diagnosed, Drug-Naïve Major Depressive Disorder Patients: A Randomized Clinical Trial. Frontiers in Nutrition. 2022;9 C7 - 876152.

158.	Carney RM, Steinmeyer BC, Freedland KE, Rubin EH, Rich MW, Harris WS. Baseline blood levels of omega-3 and depression remission: A secondary analysis of data from a placebo-controlled trial of omega-3 supplements. Journal of Clinical Psychiatry. 2016;77(2):e138-e43.

159.	Yu JJ, Pei LB, Zhang Y, Wen ZY, Yang JL. Chronic Supplementation of Curcumin Enhances the Efficacy of Antidepressants in Major Depressive Disorder: A Randomized, Double-Blind, Placebo-Controlled Pilot Study. Journal of Clinical Psychopharmacology. 2015;35(4):406-10.

**e-figure 1 for e-table 6:** Network plot of **acceptability node 1 - second largest subnetwork** (based on RR). Nodes are proportional to sample size; edges’ width is proportional to the number of comparisons.

**e-figure 2 for e-table 6:** Forest plot of **acceptability node 1 - second largest subnetwork** (based on RR). Reference is placebo + TAU.

**e-table7:** Acceptability node 2: subnetworks.

| **Author(s), year** | **Treatment** | **N** | **Subnetwork** |
| --- | --- | --- | --- |
| (Mazidi, Shemshian et al. 2016)^527^ | Iridaceae | 30 | 1 |
| (Mazidi, Shemshian et al. 2016)^527^ | PLB | 30 | 1 |
| (Araj-Khodaei, Noorbala et al. 2020)^464^ | Lamiaceae | 33 | 1 |
| (Araj-Khodaei, Noorbala et al. 2020)^464^ | SSRI | 17 | 1 |
| (Akhondzadeh, Mostafavi et al. 2020)^459^ | Iridaceae | 36 | 1 |
| (Akhondzadeh, Mostafavi et al. 2020)^459^ | PLB | 37 | 1 |
| (Carney, Freedland et al. 2019)^475^ | SSRI+LCPUFA | 71 | 1 |
| (Carney, Freedland et al. 2019)^475^ | SSRI+PLB | 73 | 1 |
| (Lyoo, Yoon et al. 2012)^593^ | SSRI+Aminoacids | 25 | 1 |
| (Lyoo, Yoon et al. 2012)^593^ | SSRI+PLB | 27 | 1 |
| (Sherwood Brown and Gabrielson 2012)^563^ | Nucleotide | 17 | 1 |
| (Sherwood Brown and Gabrielson 2012)^563^ | PLB | 14 | 1 |
| (Kumar, Menon et al. 2022)^517^ | SSRI+Vitamins | 31 | 1 |
| (Kumar, Menon et al. 2022)^517^ | SSRI+PLB | 28 | 1 |
| (Hu, Chen et al. 2021)^502^ | Herbal compound + PLB | 63 | 1 |
| (Hu, Chen et al. 2021)^502^ | SSRI+PLB | 66 | 1 |
| (Targum, Cameron et al. 2018)^569^ | Coenzyme+Antidepressants | 118 | 1 |
| (Targum, Cameron et al. 2018)^569^ | Antidepressants+PLB | 116 | 1 |
| (Ghaffari, Ghobadi et al. 2020)^494^ | SSRI+PLB | 19 | 1 |
| (Ghaffari, Ghobadi et al. 2020)^494^ | SSRI+Lauraceae | 25 | 1 |
| (Darbinyan, Aslanyan et al. 2007)^481^ | Crassulaceae | 60 | 1 |
| (Darbinyan, Aslanyan et al. 2007)^481^ | PLB | 29 | 1 |
| (Talaei, Hassanpour Moghadam et al. 2015)^590^ | Iridaceae+Antidepressants | 23 | 1 |
| (Talaei, Hassanpour Moghadam et al. 2015)^590^ | Antidepressants+PLB | 23 | 1 |
| (Akhondzadeh Basti, Moshiri et al. 2007)^457^ | Iridaceae | 20 | 1 |
| (Akhondzadeh Basti, Moshiri et al. 2007)^457^ | SSRI | 20 | 1 |
| (Rondanelli, Giacosa et al. 2010)^543^ | LCPUFA | 22 | 1 |
| (Rondanelli, Giacosa et al. 2010)^543^ | PLB | 24 | 1 |
| (Hosseini, Bagherniya et al. 2024)^501^ | Polyphenols | 20 | 1 |
| (Hosseini, Bagherniya et al. 2024)^501^ | PLB | 20 | 1 |
| (Lecrubier, Clerc et al. 2002)^211^ | HYP | 186 | 1 |
| (Lecrubier, Clerc et al. 2002)^211^ | PLB | 189 | 1 |
| (Kashani, Esalatmanesh et al. 2018)^508^ | Iridaceae | 28 | 1 |
| (Kashani, Esalatmanesh et al. 2018)^508^ | PLB | 28 | 1 |
| (Grenyer, Crowe et al. 2007)^497^ | LCPUFA+Antidepressants | 40 | 1 |
| (Grenyer, Crowe et al. 2007)^497^ | Antidepressants+PLB | 43 | 1 |
| (Jahangard, Sadeghi et al. 2018)^587^ | LCPUFA+SSRI | 25 | 1 |
| (Jahangard, Sadeghi et al. 2018)^587^ | PLB+SSRI | 25 | 1 |
| (Shamabadi, Fattollahzadeh-Noor et al. 2023)^560^ | SSRI+Aminoacids | 30 | 1 |
| (Shamabadi, Fattollahzadeh-Noor et al. 2023)^560^ | SSRI+PLB | 30 | 1 |
| (Firoozeei, Barekatain et al. 2020)^579^ | Lamiaceae +PLB | 28 | 1 |
| (Firoozeei, Barekatain et al. 2020)^579^ | SSRI+PLB | 28 | 1 |
| (Akhondzadeh, Mostafavi et al. 2022)^460^ | Lamiaceae | 26 | 1 |
| (Akhondzadeh, Mostafavi et al. 2022)^460^ | PLB | 26 | 1 |
| (Baião, Capitão et al. 2023)^465^ | PROB | 35 | 1 |
| (Baião, Capitão et al. 2023)^465^ | PLB | 36 | 1 |
| (Rudzki, Ostrowska et al. 2019)^544^ | PROB+Antidepressants | 30 | 1 |
| (Rudzki, Ostrowska et al. 2019)^544^ | Antidepressants+PLB | 30 | 1 |
| (Sahraian, Ghanizadeh et al. 2015)^585^ | Vitamins+SSRI | 21 | 1 |
| (Sahraian, Ghanizadeh et al. 2015)^585^ | SSRI+PLB | 22 | 1 |
| (Ille, Spona et al. 2007)^592^ | Aminoacids+NASSA | 20 | 3 |
| (Ille, Spona et al. 2007)^592^ | NASSA+PLB | 20 | 3 |
| (Lin, Kuo et al. 2024)^521^ | PROB+Antidepressants | 16 | 1 |
| (Lin, Kuo et al. 2024)^521^ | Antidepressants+PLB | 16 | 1 |
| (Akhondzadeh, Fallah-Pour et al. 2004)^458^ | Iridaceae | 15 | 1 |
| (Akhondzadeh, Fallah-Pour et al. 2004)^458^ | TCA | 15 | 1 |
| (Mischoulon, Best-Popescu et al. 2008)^254^ | LCPUFA | 16 | 1 |
| (Mischoulon, Best-Popescu et al. 2008)^254^ | PLB | 19 | 1 |
| (Marangell, Martinez et al. 2003)^526^ | LCPUFA | 18 | 1 |
| (Marangell, Martinez et al. 2003)^526^ | PLB | 17 | 1 |
| (Docherty, Sack et al. 2005)^486^ | Minerals | 70 | 1 |
| (Docherty, Sack et al. 2005)^486^ | PROB | 40 | 1 |
| (Fava, Alpert et al. 2005)^488^ | HYP | 45 | 1 |
| (Fava, Alpert et al. 2005)^488^ | SSRI | 47 | 1 |
| (Fava, Alpert et al. 2005)^488^ | PLB | 43 | 1 |
| (Nemets and Levine 2013)^531^ | Antidepressants+Aminoacids | 4 | 1 |
| (Nemets and Levine 2013)^531^ | Antidepressants+PLB | 4 | 1 |
| (Shahmansouri, Farokhnia et al. 2014)^558^ | Iridaceae | 22 | 1 |
| (Shahmansouri, Farokhnia et al. 2014)^558^ | SSRI | 22 | 1 |
| (Panahi, Badeli et al. 2015)^536^ | Microalgae+Antidepressants | 42 | 1 |
| (Panahi, Badeli et al. 2015)^536^ | Antidepressants | 50 | 1 |
| (Firoozabadi, Zarshenas et al. 2015)^489^ | Lamiaceae+Antidepressants | 15 | 1 |
| (Firoozabadi, Zarshenas et al. 2015)^489^ | Convolvulaceae+Antidepressants | 13 | 1 |
| (Firoozabadi, Zarshenas et al. 2015)^489^ | Antidepressants | 15 | 1 |
| (Ramezani, Kheirkhah et al. 2022)^588^ | Aminoacids+SSRI | 30 | 1 |
| (Ramezani, Kheirkhah et al. 2022)^588^ | SSRI + PLB | 30 | 1 |
| (Shelton, Keller et al. 2001)^562^ | HYP | 98 | 1 |
| (Shelton, Keller et al. 2001)^562^ | PLB | 102 | 1 |
| (Tarutani, Omori et al. 2022)^570^ | Polysaccarids | 9 | 1 |
| (Tarutani, Omori et al. 2022)^570^ | PLB | 11 | 1 |
| (Shahmoradi, Davarinejad et al. 2023)^559^ | Herbal compound | 27 | 1 |
| (Shahmoradi, Davarinejad et al. 2023)^559^ | PLB | 27 | 1 |
| (van Gurp, Meterissian et al. 2002)^572^ | HYP | 44 | 1 |
| (van Gurp, Meterissian et al. 2002)^572^ | SSRI | 43 | 1 |
| (Davidson, Gadde et al. 2002)^482^ | Minerals | 10 | 1 |
| (Davidson, Gadde et al. 2002)^482^ | PLB | 5 | 1 |
| (Wang, Bordi et al. 2015)^575^ | Vitamins | 362 | 1 |
| (Wang, Bordi et al. 2015)^575^ | PLB | 364 | 1 |
| (Kasper, Anghelescu et al. 2015)^512^ | Lamiaceae | 159 | 1 |
| (Kasper, Anghelescu et al. 2015)^512^ | PLB | 156 | 1 |
| (Sahraian, Ghanizadeh et al. 2015)^585^ | SSRI+IridaceaeF | 20 | 1 |
| (Sahraian, Ghanizadeh et al. 2015)^585^ | SSRI + PLB | 20 | 1 |
| (Djokic, Korcok et al. 2017)^485^ | Aminoacids | 30 | 1 |
| (Djokic, Korcok et al. 2017)^485^ | PLB | 30 | 1 |
| (Ryszewska-Pokraśniewicz, Mach et al. 2018)^583^ | Minerals+SSRI | 17 | 1 |
| (Ryszewska-Pokraśniewicz, Mach et al. 2018)^583^ | PLB+SSRI | 20 | 1 |
| (Lin, Wang et al. 2022)^522^ | Coenzyme | 39 | 1 |
| (Lin, Wang et al. 2022)^522^ | SSRI | 39 | 1 |
| (Lin, Wang et al. 2022)^522^ | PLB | 39 | 1 |
| (Kaviani, Nikooyeh et al. 2022)^513^ | Vitamins | 28 | 1 |
| (Kaviani, Nikooyeh et al. 2022)^513^ | PLB | 28 | 1 |
| (Ranjbar, Shams et al. 2014)^542^ | Minerals+Antidepressants | 21 | 1 |
| (Ranjbar, Shams et al. 2014)^542^ | Antidepressants | 22 | 1 |
| (He, Liu et al. 2024)^500^ | Ginkgoaceae | 34 | 1 |
| (He, Liu et al. 2024)^500^ | PLB | 34 | 1 |
| (Padhan, Mohapatra et al. 2024)^535^ | Antidepressants+Aminoacids | 30 | 1 |
| (Padhan, Mohapatra et al. 2024)^535^ | Antidepressants+PLB | 30 | 1 |
| (Sakurai, L. Carpenter et al. 2020)^548^ | Coenzyme | 16 | 1 |
| (Sakurai, L. Carpenter et al. 2020)^548^ | SSRI | 23 | 1 |
| (Sakurai, L. Carpenter et al. 2020)^548^ | PLB | 26 | 1 |
| (Vorbach, Arnoldt et al. 1997)^574^ | HYP | 107 | 1 |
| (Vorbach, Arnoldt et al. 1997)^574^ | TCA | 102 | 1 |
| (Lopresti and Drummond 2017)^523^ | Zingiberaceae | 61 | 1 |
| (Lopresti and Drummond 2017)^523^ | Zingiberaceae+Iridaceae | 26 | 1 |
| (Lopresti and Drummond 2017)^523^ | PLB | 36 | 1 |
| (Levine, Barak et al. 1995)^519^ | Vitamins | 13 | 1 |
| (Levine, Barak et al. 1995)^519^ | PLB | 15 | 1 |
| (Sanmukhani, Satodia et al. 2014)^549^ | Zingiberaceae | 20 | 1 |
| (Sanmukhani, Satodia et al. 2014)^549^ | SSRI | 20 | 1 |
| (Sanmukhani, Satodia et al. 2014)^549^ | Zingiberaceae+SSRI | 20 | 1 |
| (Gul, Memtily et al. 2019)^602^ | Herbal compound | 54 | 1 |
| (Gul, Memtily et al. 2019)^602^ | SSRI | 53 | 1 |
| (Gul, Memtily et al. 2019)^602^ | SSRI+Herbal compound | 55 | 1 |
| (Kalb, Trautmann-Sponsel et al. 2001)^506^ | HYP | 37 | 1 |
| (Kalb, Trautmann-Sponsel et al. 2001)^506^ | PROB | 35 | 1 |
| (Wu, Zhu et al. 2015)^601^ | Herbal compound+SSRI | 10 | 1 |
| (Wu, Zhu et al. 2015)^601^ | SSRI+PLB | 8 | 1 |
| (Sepehrmanesh, Kolahdooz et al. 2016)^581^ | SSRI+Vitamins | 45 | 1 |
| (Sepehrmanesh, Kolahdooz et al. 2016)^581^ | SSRI + PLB | 45 | 1 |
| (Szegedi, Kohnen et al. 2005)^567^ | HYP | 122 | 1 |
| (Szegedi, Kohnen et al. 2005)^567^ | SSRI | 122 | 1 |
| (Kanchanatawan, Tangwongchai et al. 2018)^507^ | Zingiberaceae | 33 | 1 |
| (Kanchanatawan, Tangwongchai et al. 2018)^507^ | PLB | 32 | 1 |
| (Sarris, Byrne et al. 2018)^550^ | Coenzyme+Antidepressants | 55 | 1 |
| (Sarris, Byrne et al. 2018)^550^ | PLB+Antidepressants | 52 | 1 |
| (Ghaleiha, Davari et al. 2016)^582^ | Vitamins+SSRI | 25 | 1 |
| (Ghaleiha, Davari et al. 2016)^582^ | PLB+SSRI | 26 | 1 |
| (Basoglu, Ates et al. 2009)^466^ | SSRI+Vitamins | 20 | 1 |
| (Basoglu, Ates et al. 2009)^466^ | SSRI | 15 | 1 |
| Palhano-Fontes F. et al., (2016)^600^ | Herbal compound | 17 | 1 |
| Palhano-Fontes F. et al., (2016)^600^ | PLB | 18 | 1 |
| (Piperoglou, Hopwood et al. 2023)^541^ | LCPUFA | 14 | 1 |
| (Piperoglou, Hopwood et al. 2023)^541^ | PLB | 9 | 1 |
| (Gao, Wu et al. 2020)^492^ | SSRI+PLB | 32 | 1 |
| (Gao, Wu et al. 2020)^492^ | Crassulaceae+SSRI | 66 | 1 |
| (Dormal, Jonniaux et al. 2025)^487^ | Herbal compound | 42 | 1 |
| (Dormal, Jonniaux et al. 2025)^487^ | Iridaceae+Herbal compound | 48 | 1 |
| (Dormal, Jonniaux et al. 2025)^487^ | Iridaceae | 43 | 1 |
| (Dormal, Jonniaux et al. 2025)^487^ | PLB | 47 | 1 |
| (Nemets, Stahl et al. 2002)^296^ | LCPUFA+Antidepressants | 10 | 1 |
| (Nemets, Stahl et al. 2002)^296^ | PLB+Antidepressants | 10 | 1 |
| (Caruso, Fumagalli et al. 1987)^477^ | Coenzyme | 30 | 1 |
| (Caruso, Fumagalli et al. 1987)^477^ | PLB | 30 | 1 |
| (Yu, Pei et al. 2015)^594^ | Zingiberaceae+SSRI | 50 | 1 |
| (Yu, Pei et al. 2015)^594^ | PLB+SSRI | 50 | 1 |
| (Roohi-Azizi, Arabzadeh et al. 2017)^586^ | Nucleotide+SSRI | 25 | 1 |
| (Roohi-Azizi, Arabzadeh et al. 2017)^586^ | PLB+SSRI | 25 | 1 |
| (Anushiravani, Manteghi et al. 2019)^463^ | Herbal compound | 25 | 1 |
| (Anushiravani, Manteghi et al. 2019)^463^ | SSRI | 25 | 1 |
| (Akkasheh, Kashani-Poor et al. 2016)^461^ | PROB | 20 | 1 |
| (Akkasheh, Kashani-Poor et al. 2016)^461^ | PLB | 20 | 1 |
| (Brenner, Azbel et al. 2000)^474^ | HYP | 15 | 1 |
| (Brenner, Azbel et al. 2000)^474^ | SSRI | 15 | 1 |
| (Carney, Steinmeyer et al. 2016)^476^ | Vitamins+Vitamins12+Vitamins6+SSRI | 77 | 1 |
| (Carney, Steinmeyer et al. 2016)^476^ | SSRI+PLB | 76 | 1 |
| (Almeida, Ford et al. 2014)^599^ | LCPUFA | 60 | 1 |
| (Almeida, Ford et al. 2014)^599^ | PLB | 60 | 1 |
| (Sharifan, Hosseini et al. 2017)^561^ | Iridaceae | 20 | 1 |
| (Sharifan, Hosseini et al. 2017)^561^ | SSRI | 20 | 1 |
| (Chopra and Kalyani 2020)^480^ | HYP | 83 | 1 |
| (Chopra and Kalyani 2020)^480^ | TCA | 73 | 1 |
| (Wheatley 1997)^429^ | Polysaccarids | 31 | 1 |
| (Wheatley 1997)^429^ | PLB | 31 | 1 |
| (Vaghef-Mehrabany, Ranjbar et al. 2021)^571^ | Herbal compound | 194 | 1 |
| (Vaghef-Mehrabany, Ranjbar et al. 2021)^571^ | PLB | 191 | 1 |
| (Li, Li et al. 2022)^520^ | Minerals+Antidepressants | 30 | 1 |
| (Li, Li et al. 2022)^520^ | Antidepressants | 30 | 1 |
| (Verma and Das 2016)^573^ | Vitamins | 13 | 1 |
| (Verma and Das 2016)^573^ | PLB | 14 | 1 |
| (Levine, Barak et al. 1995)^604^ | HYP | 113 | 1 |
| (Levine, Barak et al. 1995)^604^ | SSRI | 111 | 1 |
| (Davidson, Gadde et al. 2002)^483, 607^ | PLB | 116 | 1 |
| (Davidson, Gadde et al. 2002)^483, 607^ | Minerals | 45 | 1 |
| (Davidson, Gadde et al. 2002)^483, 607^ | PLB | 45 | 1 |
| (Sultana and Rahman 2023)^566^ | Vitamins+Antidepressants | 23 | 1 |
| (Sultana and Rahman 2023)^566^ | PROB+Antidepressants | 23 | 1 |
| (Vellekkatt, Menon et al. 2020)^589^ | SSRI + PLB | 30 | 1 |
| (Vellekkatt, Menon et al. 2020)^589^ | SSRI+LCPUFA | 30 | 1 |
| (Masoumi, Kazemi et al. 2016)^580^ | Minerals+Antidepressants | 10 | 1 |
| (Masoumi, Kazemi et al. 2016)^580^ | PLB+Antidepressants | 10 | 1 |
| (Nowak, Siwek et al. 2003)^534^ | Vitamins | 170 | 1 |
| (Nowak, Siwek et al. 2003)^534^ | PLB | 160 | 1 |
| (Mech and Farah 2016)^528^ | Iridaceae | 30 | 1 |
| (Mech and Farah 2016)^528^ | SSRI | 30 | 1 |
| (Ghajar, Neishabouri et al. 2017)^495^ | LCPUFA | 16 | 1 |
| (Ghajar, Neishabouri et al. 2017)^495^ | SSRI | 16 | 1 |
| (Jazayeri, Tehrani-Doost et al. 2008)^504^ | LCPUFA+SSRI | 16 | 1 |
| (Jazayeri, Tehrani-Doost et al. 2008)^504^ | Iridaceae | 25 | 1 |
| (Jazayeri, Tehrani-Doost et al. 2008)^504^ | SSRI | 25 | 1 |
| (Ahmadpanah, Ramezanshams et al. 2019)^456^ | HYP | 157 | 1 |
| (Ahmadpanah, Ramezanshams et al. 2019)^456^ | Antidepressants | 167 | 1 |
| (Woelk 2000)^576^ | Iridaceae | 20 | 1 |
| (Woelk 2000)^576^ | PROB | 20 | 1 |
| (Moshiri, Basti et al. 2006)^530^ | Lamiaceae | 16 | 1 |
| (Moshiri, Basti et al. 2006)^530^ | TCA | 16 | 1 |
| (Akhondzadeh, Mostafavi et al. 2022)^460^ | Lamiaceae+TCA | 16 | 1 |
| (Akhondzadeh, Mostafavi et al. 2022)^460^ | HYP | 70 | 1 |
| (Akhondzadeh, Mostafavi et al. 2022)^460^ | SSRI | 79 | 1 |
| (Harrer, Schmidt et al. 1999)^499^ | Iridaceae | 34 | 1 |
| (Harrer, Schmidt et al. 1999)^499^ | SSRI | 34 | 1 |
| (Kashani, Eslatmanesh et al. 2017)^509^ | Aminoacids+Antidepressants | 127 | 1 |
| (Kashani, Eslatmanesh et al. 2017)^509^ | PLB+Antidepressants | 125 | 1 |
| (Berk, Dean et al. 2014)^470^ | Zingiberaceae | 28 | 1 |
| (Berk, Dean et al. 2014)^470^ | PLB | 28 | 1 |
| (Lopresti, Maes et al. 2014)^221^ | Vitamins+TCA | 43 | 2 |
| (Lopresti, Maes et al. 2014)^221^ | TCA+PLB | 47 | 2 |
| (Lôo, Poirier et al. 2000)^605^ | Iridaceae | 20 | 1 |
| (Lôo, Poirier et al. 2000)^605^ | SSRI | 20 | 1 |
| (Noorbala, Akhondzadeh et al. 2005)^533^ | HYP | 59 | 1 |
| (Noorbala, Akhondzadeh et al. 2005)^533^ | SSRI | 57 | 1 |
| (Behnke, Jensen et al. 2002)^467^ | HYP | 35 | 1 |
| (Behnke, Jensen et al. 2002)^467^ | SSRI | 35 | 1 |
| (Schrader, Meier et al. 1998)^554^ | HYP | 81 | 1 |
| (Schrader, Meier et al. 1998)^554^ | PROB | 81 | 1 |
| (Schrader 2000)^553^ | HYP | 126 | 1 |
| (Schrader 2000)^553^ | SSRI + PLB | 114 | 1 |
| (Coppen and Bailey 2000)^584^ | Vitamins+SSRI | 62 | 1 |
| (Coppen and Bailey 2000)^584^ | PLB+SSRI | 65 | 1 |
| (Amini, Yaghini et al. 2021)^462^ | Vitamins+Minerals | 26 | 1 |
| (Amini, Yaghini et al. 2021)^462^ | Vitamins | 26 | 1 |
| (Amini, Yaghini et al. 2021)^462^ | PLB | 24 | 1 |
| (Ghorbani, Nazari et al. 2018)^496^ | PROB | 20 | 1 |
| (Ghorbani, Nazari et al. 2018)^496^ | PLB | 20 | 1 |
| (Açik, Çakiroğlu et al. 2025)^455^ | LCPUFA | 38 | 1 |
| (Açik, Çakiroğlu et al. 2025)^455^ | PLB | 19 | 1 |
| (Papakostas, Mischoulon et al. 2010)^538^ | Coenzyme+Antidepressants | 39 | 1 |
| (Papakostas, Mischoulon et al. 2010)^538^ | PLB+Antidepressants | 34 | 1 |
| (Gelenberg, Shelton et al. 2004)^493^ | HYP | 43 | 1 |
| (Gelenberg, Shelton et al. 2004)^493^ | PLB | 55 | 1 |
| (Shafiee-Kandjani, Khalili et al. 2023)^557^ | SSRI+Lamiaceae+DRA | 35 | 1 |
| (Shafiee-Kandjani, Khalili et al. 2023)^557^ | SSRI+PLB | 35 | 1 |
| (Talaei, Noori et al. 2023)^568^ | SSRI+Zingiberaceae | 23 | 1 |
| (Talaei, Noori et al. 2023)^568^ | SSRI+PLB | 22 | 1 |
| (Kasper, Anghelescu et al. 2006)^510^ | HYP | 243 | 1 |
| (Kasper, Anghelescu et al. 2006)^510^ | PLB | 81 | 1 |
| (Sadeghi, Ghorayshi et al. 2023)^546^ | Herbal compound+HYP | 37 | 1 |
| (Sadeghi, Ghorayshi et al. 2023)^546^ | SSRI | 37 | 1 |
| (Park, Park et al. 2015)^540^ | LCPUFA+Antidepressants | 18 | 1 |
| (Park, Park et al. 2015)^540^ | Antidepressants+PLB | 17 | 1 |
| (De Leo 1987)^484^ | Coenzyme | 20 | 1 |
| (De Leo 1987)^484^ | PLB | 20 | 1 |
| (Cheng, Chen et al. 2025)^479^ | LCPUFA | 19 | 1 |
| (Cheng, Chen et al. 2025)^479^ | PLB | 20 | 1 |
| (Kreuzer, Reiter et al. 2022)^516, 608^ | PROB | 28 | 1 |
| (Kreuzer, Reiter et al. 2022)^516, 608^ | PLB | 29 | 1 |
| (Sarris, Fava et al. 2012)^606^ | HYP | 35 | 1 |
| (Sarris, Fava et al. 2012)^606^ | SSRI | 49 | 1 |
| (Sarris, Fava et al. 2012)^606^ | PLB | 40 | 1 |
| (Jelodar, Javid et al. 2018)^591^ | SSRI+IridaceaeF | 20 | 1 |
| (Jelodar, Javid et al. 2018)^591^ | SSRI + PLB | 20 | 1 |
| (Khoraminya, Tehrani-Doost et al. 2013)^514^ | Vitamins+SSRI | 20 | 1 |
| (Khoraminya, Tehrani-Doost et al. 2013)^514^ | SSRI | 20 | 1 |
| (Sepehrmanesh, Kolahdooz et al. 2016)^556^ | Vitamins | 20 | 1 |
| (Sepehrmanesh, Kolahdooz et al. 2016)^556^ | PLB | 20 | 1 |
| (Sahoo, Jarnail et al. 2015)^547^ | SSRI+LCPUFA | 33 | 1 |
| (Sahoo, Jarnail et al. 2015)^547^ | SSRI+Aminoacids | 37 | 1 |
| (Sahoo, Jarnail et al. 2015)^547^ | SSRI | 33 | 1 |
| (Laakmann 1998)^518^ | HYP | 98 | 1 |
| (Laakmann 1998)^518^ | PLB | 49 | 1 |
| (Chang, Chang et al. 2020)^478^ | LCPUFA | 30 | 1 |
| (Chang, Chang et al. 2020)^478^ | PLB | 29 | 1 |
| (Saccarello, Montarsolo et al. 2020)^545^ | Coenzyme+PROB | 46 | 1 |
| (Saccarello, Montarsolo et al. 2020)^545^ | PLB | 44 | 1 |
| (Friede, Henneicke von Zepelin et al. 2001)^491^ | HYP | 126 | 1 |
| (Friede, Henneicke von Zepelin et al. 2001)^491^ | SSRI | 114 | 1 |
| (Ning, Zhou et al. 2022)^532^ | HYP | 89 | 1 |
| (Ning, Zhou et al. 2022)^532^ | PLB | 87 | 1 |
| (Bergman, Miodownik et al. 2013)^469^ | Zingiberaceae | 20 | 1 |
| (Bergman, Miodownik et al. 2013)^469^ | PLB | 20 | 1 |
| (Kasper, Anghelescu et al. 2007)^511^ | HYP | 68 | 1 |
| (Kasper, Anghelescu et al. 2007)^511^ | PLB | 24 | 1 |
| (Bot, Pouwer et al. 2010)^473^ | LCPUFA | 13 | 1 |
| (Bot, Pouwer et al. 2010)^473^ | PLB | 12 | 1 |
| (Mozaffari-Khosravi, Nabizade et al. 2013)^272^ | LCPUFA | 41 | 1 |
| (Mozaffari-Khosravi, Nabizade et al. 2013)^272^ | PLB | 21 | 1 |
| (Sarris, Murphy et al. 2020)^552^ | Coenzyme | 25 | 1 |
| (Sarris, Murphy et al. 2020)^552^ | PLB | 24 | 1 |
| (Bell, Plon et al. 1988)^468^ | Coenzyme | 9 | 1 |
| (Bell, Plon et al. 1988)^468^ | IMI | 9 | 1 |
| (Siwek, Dudek et al. 2010)^598^ | Minerals+TCA | 30 | 2 |
| (Siwek, Dudek et al. 2010)^598^ | TCA+PLB | 30 | 2 |
| (Papakostas, Shelton et al. 2012)^539^ | Vitamins | 36 | 1 |
| (Papakostas, Shelton et al. 2012)^539^ | PLB | 112 | 1 |
| (Papakostas, Shelton et al. 2012)^539^ | Vitamins | 19 | 1 |
| (Papakostas, Shelton et al. 2012)^539^ | PLB | 56 | 1 |
| (Majmasanaye, Mehrpooya et al. 2024)^524^ | Coenzyme+Antidepressants | 46 | 1 |
| (Majmasanaye, Mehrpooya et al. 2024)^524^ | PLB+Antidepressants | 46 | 1 |
| (Hvas, Juul et al. 2004)^503^ | Vitamins | 70 | 1 |
| (Hvas, Juul et al. 2004)^503^ | PLB | 70 | 1 |
| (Bidzan-Wiącek, Tomczyk et al. 2024)^471^ | LCPUFA | 25 | 1 |
| (Bidzan-Wiącek, Tomczyk et al. 2024)^471^ | PLB | 22 | 1 |
| (Sarris, Byrne et al. 2019)^551^ | Coenzyme+Vitamins+LCPUFA+Minerals+Aminoacids | 81 | 1 |
| (Sarris, Byrne et al. 2019)^551^ | PLB | 77 | 1 |
| (Wu, Yang et al. 2024)^577^ | LCPUFA | 30 | 1 |
| (Wu, Yang et al. 2024)^577^ | PLB | 30 | 1 |
| (Zhang, Wang et al. 2018)^578^ | Vitamins | 56 | 1 |
| (Zhang, Wang et al. 2018)^578^ | PLB | 64 | 1 |
| (Kasper, Volz et al. 2024)^597^ | Lamiaceae | 170 | 1 |
| (Kasper, Volz et al. 2024)^597^ | SSRI | 171 | 1 |
| (Kasper, Volz et al. 2024)^597^ | PLB | 157 | 1 |
| (Kolouri, Firoozabadi et al. 2016)^515^ | Lamiaceae | 33 | 1 |
| (Kolouri, Firoozabadi et al. 2016)^515^ | SSRI | 33 | 1 |
| (Rees, Austin et al. 2008)^332^ | LCPUFA | 13 | 1 |
| (Rees, Austin et al. 2008)^332^ | PLB | 13 | 1 |
| (Freeman, Davis et al. 2008)^490^ | LCPUFA | 31 | 1 |
| (Freeman, Davis et al. 2008)^490^ | PLB | 28 | 1 |
| (Varzaghani, Sharifi et al. 2022)^596^ | PRO | 28 | 1 |
| (Varzaghani, Sharifi et al. 2022)^596^ | PLB | 26 | 1 |
| (Shinto, Marracci et al. 2016)^564^ | LCPUFA | 21 | 1 |
| (Shinto, Marracci et al. 2016)^564^ | PLB | 18 | 1 |
| (Su, Huang et al. 2008)^387^ | LCPUFA | 18 | 1 |
| (Su, Huang et al. 2008)^387^ | PLB | 18 | 1 |
| (Mischoulon, Dunlop et al. 2022)^603^ | LCPUFA | 16 | 1 |
| (Mischoulon, Dunlop et al. 2022)^603^ | PLB | 15 | 1 |
| (Su, Huang et al. 2003)^565^ | LCPUFA | 12 | 1 |
| (Su, Huang et al. 2003)^565^ | PLB | 10 | 1 |
| (Yang, Wang et al. 2022)^595^ | LCPUFA+SNRI | 36 | 4 |
| (Yang, Wang et al. 2022)^595^ | SNRI+PLB | 36 | 4 |
| (Kagan, Sultzer et al. 1990)^505^ | Coenzyme | 9 | 1 |
| (Kagan, Sultzer et al. 1990)^505^ | PLB | 6 | 1 |
| (Han, Chung et al. 2012)^498^ | Herbal compound | 82 | 1 |
| (Han, Chung et al. 2012)^498^ | PLB | 82 | 1 |
| (Pancheri, Scapicchio et al. 2002)^537^ | Coenzyme | 146 | 1 |
| (Pancheri, Scapicchio et al. 2002)^537^ | TCA | 147 | 1 |
| (Mischoulon, Price et al. 2014)^529^ | Coenzyme | 64 | 1 |
| (Mischoulon, Price et al. 2014)^529^ | SSRI | 65 | 1 |
| (Mischoulon, Price et al. 2014)^529^ | PLB | 60 | 1 |
| (Mao, Xie et al. 2015)^525^ | Crassulaceae | 20 | 1 |
| (Mao, Xie et al. 2015)^525^ | SSRI | 19 | 1 |
| (Mao, Xie et al. 2015)^525^ | PLB | 18 | 1 |
| (Sempach, Schaub et al. 2025)^555^ | Aminoacids+Antidepressants | 26 | 1 |
| (Sempach, Schaub et al. 2025)^555^ | Antidepressants+PLB | 26 | 1 |

**Reference for e-table 7**

1.	Mazidi M, Shemshian M, Mousavi SH, Norouzy A, Kermani T, Moghiman T, et al. A double-blind, randomized and placebo-controlled trial of Saffron (Crocus sativus L.) in the treatment of anxiety and depression. 2016;13(2):195‐9

C3 - PUBMED 27101556,EMBASE 610483123.

2.	Araj-Khodaei M, Noorbala AA, Yarani R, Emadi F, Emaratkar E, Faghihzadeh S, et al. A double-blind, randomized pilot study for comparison of Melissa officinalis L. And Lavandula angustifolia Mill. And Fluoxetine for the treatment of depression. BMC Complementary Medicine and Therapies. 2020;20(1).

3.	Akhondzadeh S, Mostafavi SA, Keshavarz SA, Mohammadi MR, Hosseini S, Eshraghian MR. A placebo controlled randomized clinical trial of Crocus sativus L. (saffron) on depression and food craving among overweight women with mild to moderate depression. Journal of Clinical Pharmacy and Therapeutics. 2020;45(1):134-43.

4.	Carney RM, Freedland KE, Rubin EH, Rich MW, Steinmeyer BC, Harris WS. A randomized placebo-controlled trial of omega-3 and sertraline in depressed patients with or at risk for coronary heart disease. Journal of Clinical Psychiatry. 2019;80(4 C7 - 19m12742).

5.	Lyoo IK, Yoon S, Kim TS, Hwang J, Kim JE, Won W, et al. A randomized, double-blind placebo-controlled trial of oral creatine monohydrate augmentation for enhanced response to a selective serotonin reuptake inhibitor in women with major depressive disorder. American Journal of Psychiatry. 2012;169(9):937-45.

6.	Sherwood Brown E, Gabrielson B. A randomized, double-blind, placebo-controlled trial of citicoline for bipolar and unipolar depression and methamphetamine dependence. Journal of Affective Disorders. 2012;143(1-3):257-60.

7.	Kumar PNS, Menon V, Andrade C. A randomized, double-blind, placebo-controlled, 12-week trial of vitamin D augmentation in major depressive disorder associated with vitamin D deficiency. Journal of Affective Disorders. 2022;314:143-9.

8.	Hu Y, Chen C, Wang Y, Yang W, Wang Y, Zhu W, et al. The effects of KaiXinSan on depression and its association with lipid profiles: a randomized, double-blinded, placebo-controlled trial. 2021;83:153467

C3 - PUBMED 33516143,EMBASE 2010802711.

9.	Targum SD, Cameron BR, Ferreira L, MacDonald ID. An augmentation study of MSI-195 (S-adenosylmethionine) in Major Depressive Disorder. Journal of Psychiatric Research. 2018;107:86-96.

10.	Ghaffari S, Ghobadi A, Jamshidi AH, Mortazavi SH, Naderi S, Aqamolaei A, et al. Cinnamomum tamala as an adjuvant therapy in the treatment of major depressive disorder: A double-blind, randomized, placebo-controlled clinical trial with placebo control. Advances in Integrative Medicine. 2020;7(3):141-7.

11.	Darbinyan V, Aslanyan G, Amroyan E, Gabrielyan E, Malmström C, Panossian A. Clinical trial of Rhodiola rosea L. extract SHR-5 in the treatment of mild to moderate depression. Nordic Journal of Psychiatry. 2007;61(5):343-8.

12.	Talaei A, Hassanpour Moghadam M, Sajadi Tabassi SA, Mohajeri SA. Crocin, the main active saffron constituent, as an adjunctive treatment in major depressive disorder: A randomized, double-blind, placebo-controlled, pilot clinical trial. Journal of Affective Disorders. 2015;174:51-6.

13.	Akhondzadeh Basti A, Moshiri E, Noorbala AA, Jamshidi AH, Abbasi SH, Akhondzadeh S. Comparison of petal of Crocus sativus L. and fluoxetine in the treatment of depressed outpatients: A pilot double-blind randomized trial. Progress in Neuro-Psychopharmacology and Biological Psychiatry. 2007;31(2):439-42.

14.	Rondanelli M, Giacosa A, Opizzi A, Pelucchi C, Vecchia CL, Montorfano G, et al. Effect of omega-3 fatty acids supplementation on depressive symptoms and on health-related quality of life in the treatment of elderly women with depression: A double-blind, placebo-controlled, randomized clinical trial. Journal of the American College of Nutrition. 2010;29(1):55-64.

15.	Hosseini H, Bagherniya M, Sahebkar A, Iraj B, Majeed M, Askari G. The effect of curcumin-piperine supplementation on lipid profile, glycemic index, inflammation, and blood pressure in patients with type 2 diabetes mellitus and hypertriglyceridemia. Phytotherapy Research. 2024;38(11):5150-61.

16.	Lecrubier Y, Clerc G, Didi R, Kieser M. Efficacy of St. John's wort extract WS 5570 in major depression: A double-blind, placebo-controlled trial. American Journal of Psychiatry. 2002;159(8):1361-6.

17.	Kashani L, Esalatmanesh S, Eftekhari F, Salimi S, Foroughifar T, Etesam F, et al. Efficacy of Crocus sativus (saffron) in treatment of major depressive disorder associated with post-menopausal hot flashes: a double-blind, randomized, placebo-controlled trial. Arch Gynecol Obstet. 2018;297(3):717-24.

18.	Grenyer BF, Crowe T, Meyer B, Owen AJ, Grigonis-Deane EM, Caputi P, et al. Fish oil supplementation in the treatment of major depression: a randomised double-blind placebo-controlled trial. 2007;31(7):1393‐6

C3 - PUBMED 17659823.

19.	Jahangard L, Sadeghi A, Ahmadpanah M, Holsboer-Trachsler E, Sadeghi Bahmani D, Haghighi M, et al. Influence of adjuvant omega-3-polyunsaturated fatty acids on depression, sleep, and emotion regulation among outpatients with major depressive disorders - Results from a double-blind, randomized and placebo-controlled clinical trial. Journal of Psychiatric Research. 2018;107:48-56.

20.	Shamabadi A, Fattollahzadeh-Noor S, Fallahpour B, A. Basti F, Khodaei Ardakani MR, Akhondzadeh S. L-Theanine adjunct to risperidone in the treatment of chronic schizophrenia inpatients: a randomized, double-blind, placebo-controlled clinical trial. Psychopharmacology. 2023;240(12):2631-40.

21.	Firoozeei TS, Barekatain M, Karimi M, Zargaran A, Akhondzadeh S, Rezaeizadeh H. Lavender and dodder combined herbal syrup versus citalopram in major depressive disorder with anxious distress: A double-blind randomized trial. Journal of Integrative Medicine. 2020;18(5):409-15.

22.	Akhondzadeh S, Mostafavi SA, Keshavarz SA, Mohammadi MR, Chamari M. Melatonin Effects in Women With Comorbidities of Overweight, Depression, and Sleep Disturbance: A Randomized Placebo Controlled Clinical Trial. Sleep Med Res. 2022;13(1):22-30.

23.	Baião R, Capitão LP, Higgins C, Browning M, Harmer CJ, Burnet PWJ. Multispecies probiotic administration reduces emotional salience and improves mood in subjects with moderate depression: a randomised, double-blind, placebo-controlled study. Psychological medicine. 2023;53(8):3437-47.

24.	Rudzki L, Ostrowska L, Pawlak D, Małus A, Pawlak K, Waszkiewicz N, et al. Probiotic Lactobacillus Plantarum 299v decreases kynurenine concentration and improves cognitive functions in patients with major depression: A double-blind, randomized, placebo controlled study. Psychoneuroendocrinology. 2019;100:213-22.

25.	Sahraian A, Ghanizadeh A, Kazemeini F. Vitamin C as an adjuvant for treating major depressive disorder and suicidal behavior, a randomized placebo-controlled clinical trial. Trials. 2015;16(1 C7 - 94).

26.	Ille R, Spona J, Zickl M, Hofmann P, Lahousen T, Dittrich N, et al. "Add-On"-therapy with an individualized preparation consisting of free amino acids for patients with a major depression. European Archives of Psychiatry and Clinical Neuroscience. 2007;257(4):222-9.

27.	Lin SKK, Kuo PH, Hsu CY, Chiu YH, Chen CH. The effects of Lactobacillus plantarum PS128 in patients with major depressive disorder: an eight-week double-blind, placebo-controlled study. Asian Journal of Psychiatry. 2024;101 C7 - 104210.

28.	Akhondzadeh S, Fallah-Pour H, Afkham K, Jamshidi AH, Khalighi-Cigaroudi F. Comparison of Crocus sativus L. and imipramine in the treatment of mild to moderate depression: A pilot double-blind randomized trial [ISRCTN45683816]. BMC Complementary and Alternative Medicine. 2004;4.

29.	Mischoulon D, Best-Popescu C, Laposata M, Merens W, Murakami JL, Wu SL, et al. A double-blind dose-finding pilot study of docosahexaenoic acid (DHA) for major depressive disorder. European Neuropsychopharmacology. 2008;18(9):639-45.

30.	Marangell LB, Martinez JM, Zboyan HA, Kertz B, Kim HFS, Puryear LJ. A double-blind, placebo-controlled study of the omega-3 fatty acid docosahexaenoic acid in the treatment of major depression. American Journal of Psychiatry. 2003;160(5):996-8.

31.	Docherty JP, Sack DA, Roffman M, Finch M, Komorowski JR. A double-blind, placebo-controlled, exploratory trial of chromium picolinate in atypical depression: Effect on carbohydrate craving. Journal of Psychiatric Practice. 2005;11(5):302-14.

32.	Fava M, Alpert J, Nierenberg AA, Mischoulon D, Otto MW, Zajecka J, et al. A double-blind, randomized trial of St John's wort, fluoxetine, and placebo in major depressive disorder. Journal of Clinical Psychopharmacology. 2005;25(5):441-7.

33.	Nemets B, Levine J. A pilot dose-finding clinical trial of creatine monohydrate augmentation to SSRIs/SNRIs/NASA antidepressant treatment in major depression. International Clinical Psychopharmacology. 2013;28(3):127-33.

34.	Shahmansouri N, Farokhnia M, Abbasi SH, Kassaian SE, Noorbala Tafti AA, Gougol A, et al. A randomized, double-blind, clinical trial comparing the efficacy and safety of Crocus sativus L. with fluoxetine for improving mild to moderate depression in post percutaneous coronary intervention patients. 2014;155:216‐22

C3 - PUBMED 24289892,EMBASE 52891114.

35.	Panahi Y, Badeli R, Karami GR, Badeli Z, Sahebkar A. A randomized controlled trial of 6-week Chlorella vulgaris supplementation in patients with major depressive disorder. Complement Ther Med. 2015;23(4):598-602.

36.	Firoozabadi A, Zarshenas MM, Salehi A, Jahanbin S, Mohagheghzadeh A. Effectiveness of Cuscuta planiflora Ten. and Nepeta menthoides Boiss. & Buhse in major depression: a triple-blind randomized controlled trial study. J Evid Based Complementary Altern Med. 2015;20(2):94-7.

37.	Ramezani A, Kheirkhah F, Zabihi E, Shirafkan H, Moudi S. Effectiveness of L-carnitine Supplementation to Sertraline for Treatment of Major Depressive Disorder: A Double-blind Randomized Placebo-controlled Trial. Glob J Med Pharm Biomed Update. 2022;17.

38.	Shelton RC, Keller MB, Gelenberg A, Dunner DL, Hirschfeld R, Thase ME, et al. Effectiveness of St John's wort in major depression: A randomized controlled trial. JAMA. 2001;285(15):1978-86.

39.	Tarutani S, Omori M, Ido Y, Yano M, Komatsu T, Okamura T. Effects of 4G-beta-D-Galactosylsucrose in patients with depression: A randomized, double-blinded, placebo-controlled, parallel-group comparative study. J Psychiatr Res. 2022;148:110-20.

40.	Shahmoradi N, Davarinejad O, Brühl AB, Brand S. Effects of Aphrodite (an Herbal Compound) on SSRI-Induced Sexual Dysfunctions and Depression in Females with Major Depressive Disorder: Findings from a Randomized Clinical Trial. Medicina (Kaunas, Lithuania). 2023;59(9).

41.	van Gurp G, Meterissian GB, Haiek LN, McCusker J, Bellavance F. St John's wort or sertraline? Randomized controlled trial in primary care. Can Fam Physician. 2002;48:905-12.

42.	Davidson JR, Gadde KM, Fairbank JA, Krishnan KRR, Califf RM, Binanay C, et al. Effect of Hypericum perforatum (St John's wort) in major depressive disorder: a randomized controlled trial. 2002;287(14):1807‐14

C3 - PUBMED 11939866,EMBASE 34303625.

43.	Wang L, Bordi PL, Fleming JA, Hill AM, Kris-Etherton PM. Effect of a moderate fat diet with and without avocados on lipoprotein particle number, size and subclasses in overweight and obese adults: a randomized, controlled trial. J Am Heart Assoc. 2015;4(1):e001355.

44.	Kasper S, Anghelescu I, Dienel A. Efficacy of orally administered Silexan in patients with anxiety-related restlessness and disturbed sleep--A randomized, placebo-controlled trial. 2015;25(11):1960‐7

C3 - PUBMED 26293583,EMBASE 605643089.

45.	Djokic G, Korcok D, Djordjevic V, Agic A, Rankovic A, Djukic Dejanovic S. The effects of s-adenosyl-L-methionine-vitamin b complex on mild and moderate depressive symptoms. Hippokratia. 2017;21(3):140-3.

46.	Ryszewska-Pokraśniewicz B, Mach A, Skalski M, Januszko P, Wawrzyniak ZM, Poleszak E, et al. Effects of magnesium supplementation on unipolar depression: A placebo-controlled study and review of the importance of dosing and magnesium status in the therapeutic response. Nutrients. 2018;10(8 C7 - 1014).

47.	Lin CH, Wang SH, Lane HY. Effects of Sodium Benzoate, a D-Amino Acid Oxidase Inhibitor, on Perceived Stress and Cognitive Function Among Patients With Late-Life Depression: A Randomized, Double-Blind, Sertraline- and Placebo-Controlled Trial. International Journal of Neuropsychopharmacology. 2022;25(7):545-55.

48.	Kaviani M, Nikooyeh B, Etesam F, Behnagh SJ, Kangarani HM, Arefi M, et al. Effects of vitamin D supplementation on depression and some selected pro-inflammatory biomarkers: a double-blind randomized clinical trial. BMC Psychiatry. 2022;22(1 C7 - 694).

49.	Ranjbar E, Shams J, Sabetkasaei M, M-Shirazi M, Rashidkhani B, Mostafavi A, et al. Effects of zinc supplementation on efficacy of antidepressant therapy, inflammatory cytokines, and brain-derived neurotrophic factor in patients with major depression. Nutritional Neuroscience. 2014;17(2):65-71.

50.	He X, Liu D, Ni S, Li Z, Li S, Wu T, et al. Efficacy and safety evaluation of Ginkgo biloba dropping pill (GBDP) on stable angina pectoris complicated with depression: A placebo-controlled, randomized, double-blind, multicenter study. Phytomedicine. 2024;126.

51.	Padhan M, Mohapatra D, Mishra BR, Maiti R, Jena M. Efficacy and safety of add-on sarcosine in patients with major depressive disorder: A randomized controlled trial. Journal of Psychiatric Research. 2024;178:298-304.

52.	Sakurai H, L. Carpenter L, R. Tyrka A, Price LH, I. Papakostas G, Dording CM, et al. Dose increase of S-Adenosyl-Methionine and escitalopram in a randomized clinical trial for major depressive disorder. Journal of Affective Disorders. 2020;262:118-25.

53.	Vorbach EU, Arnoldt KH, Hubner WD. Efficacy and tolerability of St. John's wort extract LI 160 versus imipramine in patients with severe depressive episodes according to ICD-10. Pharmacopsychiatry. 1997;30(SUPPL.):81-5.

54.	Lopresti AL, Drummond PD. Efficacy of curcumin, and a saffron/curcumin combination for the treatment of major depression: A randomised, double-blind, placebo-controlled study. Journal of Affective Disorders. 2017;207:188-96.

55.	Levine J, Barak Y, Gonzalves M, Szor H, Elizur A, Kofman O, et al. Double-blind, controlled trial of inositol treatment of depression. American Journal of Psychiatry. 1995;152(5):792-4.

56.	Sanmukhani J, Satodia V, Trivedi J, Patel T, Tiwari D, Panchal B, et al. Efficacy and safety of curcumin in major depressive disorder: a randomized controlled trial. Phytother Res. 2014;28(4):579-85.

57.	Gul A, Memtily N, Mijit P, Imin M, Rui H, Wushuer P, et al. The Üstikuddus sherbiti in the treatment of abnormal cold and dry-type depression patients with comorbid anxiety: A randomized and controlled clinical trial. Trad Med Mod Med. 2019;2(3):127-33.

58.	Kalb R, Trautmann-Sponsel RD, Kieser M. Efficacy and tolerability of Hypericum extract WS 5572 versus placebo in mildly to moderately depressed patients: A randomized double-blind multicenter clinical trial. Pharmacopsychiatry. 2001;34(3):96-103.

59.	Wu R, Zhu D, Xia Y, Wang H, Tao W, Xue W, et al. A role of Yueju in fast-onset antidepressant action on major depressive disorder and serum BDNF expression: A randomly double-blind, fluoxetine-adjunct, placebo-controlled, pilot clinical study. Neuropsychiatric Disease and Treatment. 2015;11:2013-21.

60.	Sepehrmanesh Z, Kolahdooz F, Abedi F, Mazroii N, Assarian A, Asemi Z, et al. Vitamin D Supplementation Affects the Beck Depression Inventory, Insulin Resistance, and Biomarkers of Oxidative Stress in Patients with Major Depressive Disorder: A Randomized, Controlled Clinical Trial [Retraction notice in CN-02298463]. 2016;146(2):243‐8

C3 - PUBMED 26609167,EMBASE 608794678.

61.	Szegedi A, Kohnen R, Dienel A, Kieser M. Acute treatment of moderate to severe depression with hypericum extract WS 5570 (St John's wort): Randomised controlled double blind non-inferiority trial versus paroxetine. British Medical Journal. 2005;330(7490):503-6.

62.	Kanchanatawan B, Tangwongchai S, Sughondhabhirom A, Suppapitiporn S, Hemrunrojn S, Carvalho AF, et al. Add-on Treatment with Curcumin Has Antidepressive Effects in Thai Patients with Major Depression: Results of a Randomized Double-Blind Placebo-Controlled Study. Neurotoxic Res. 2018;33(3):621-33.

63.	Sarris J, Byrne GJ, Bousman C, Stough C, Murphy J, MacDonald P, et al. Adjunctive S-adenosylmethionine (SAMe) in treating non-remittent major depressive disorder: An 8-week double-blind, randomized, controlled trial. European Neuropsychopharmacology. 2018;28(10):1126-36.

64.	Ghaleiha A, Davari H, Jahangard L, Haghighi M, Ahmadpanah M, Seifrabie MA, et al. Adjuvant thiamine improved standard treatment in patients with major depressive disorder: results from a randomized, double-blind, and placebo-controlled clinical trial. European Archives of Psychiatry and Clinical Neuroscience. 2016;266(8):695-702.

65.	Basoglu C, Ates MA, Algul A, Ipcioglu OM, Gecici O, Yilmaz O, et al. Adjuvant folate with escitalopram treatment and homocystein, folate, vitamin B-12 levels in patients with major depressive disorder. 2009;19(2):135‐42

C3 - EMBASE 354832073.

66.	Antidepressant Effects of Ayahuasca: a Randomized Placebo Controlled Trial in Treatment Resistant Depression. clinicaltrialsgov2016.

67.	Piperoglou M, Hopwood M, Norman TR. Adjunctive Docosahexaenoic Acid in Residual Symptoms of Depression and Anxiety. Journal of Clinical Psychopharmacology. 2023;43(6):493-7.

68.	Gao L, Wu C, Liao Y, Wang J. Antidepressants effects of Rhodiola capsule combined with sertraline for major depressive disorder: A randomized double-blind placebo-controlled clinical trial. Journal of Affective Disorders. 2020;265:99-103.

69.	Dormal V, Jonniaux L, Buchet M, Simar L, Copine S, Deldicque L. Effect of Hydroponically Grown Red Panax Ginseng on Perceived Stress Level, Emotional Processing, and Cognitive Functions in Moderately Stressed Adults: A Randomized, Double-Blind, Placebo-Controlled Study. Nutrients. 2025;17(6 C7 - 955).

70.	Nemets B, Stahl Z, Belmaker RH. Addition of omega-3 fatty acid to maintenance medication treatment for recurrent unipolar depressive disorder. American Journal of Psychiatry. 2002;159(3):477-9.

71.	Caruso I, Fumagalli M, Boccassini L, Puttini SP, Santandrea S, Giniselli G, et al. Treatment of depression in rheumatoid arthritic patients. A comparison of S-adenosylmethionine (Samyr) and placebo in a double-blind study. CLIN TRIALS J. 1987;24(4):305-10.

72.	Yu JJ, Pei LB, Zhang Y, Wen ZY, Yang JL. Chronic Supplementation of Curcumin Enhances the Efficacy of Antidepressants in Major Depressive Disorder: A Randomized, Double-Blind, Placebo-Controlled Pilot Study. Journal of Clinical Psychopharmacology. 2015;35(4):406-10.

73.	Roohi-Azizi M, Arabzadeh S, Amidfar M, Salimi S, Zarindast MR, Talaei A, et al. Citicoline combination therapy for major depressive disorder: A randomized, double-blind, placebo-controlled trial. Clinical Neuropharmacology. 2017;40(1):1-5.

74.	Anushiravani M, Manteghi AA, Taghipur A, Eslami M. Comparing Effectiveness of a Combined Herbal Drug Based on Echium Amoenum with Citalopram in the Treatment of Major Depressive Disorder. Curr Drug Discov Technol. 2019;16(2):232-8.

75.	Akkasheh G, Kashani-Poor Z, Tajabadi-Ebrahimi M, Jafari P, Akbari H, Taghizadeh M, et al. Clinical and metabolic response to probiotic administration in patients with major depressive disorder: A randomized, double-blind, placebo-controlled trial. Nutrition. 2016;32(3):315-20.

76.	Brenner R, Azbel V, Madhusoodanan S, Pawlowska M. Comparison of an extract of hypericum (LI 160) and sertraline in the treatment of depression: A double-blind, randomized pilot study. Clinical Therapeutics. 2000;22(4):411-9.

77.	Carney RM, Steinmeyer BC, Freedland KE, Rubin EH, Rich MW, Harris WS. Baseline blood levels of omega-3 and depression remission: A secondary analysis of data from a placebo-controlled trial of omega-3 supplements. Journal of Clinical Psychiatry. 2016;77(2):e138-e43.

78.	Almeida OP, Ford AH, Hirani V, Singh V, VanBockxmeer FM, McCaul K, et al. B vitamins to enhance treatment response to antidepressants in middle-aged and older adults: Results from the B-VITAGE randomised, double-blind, placebo-controlled trial. British Journal of Psychiatry. 2014;205(6):450-7.

79.	Sharifan P, Hosseini MS, Sharifan A. The interventional relationship between frequent fish consumption and depression symptoms in aging adults: a randomized controlled trial. International Journal of Geriatric Psychiatry. 2017;32(12):e116-e22.

80.	Chopra A, Kalyani AK. Comparison of efficacy of Crocus sativus L. with fluoxetine for improving depression among patients with cardiovascular diseases. Int J Life Sci Biotechnol Pharma Res. 2020;9(2):151-4.

81.	Wheatley D. LI 160, an extract of St. John's wort, versus amitriptyline in mildly to moderately depressed outpatients--a controlled 6-week clinical trial. 1997;30 Suppl 2:77‐80

C3 - PUBMED 9342764.

82.	Vaghef-Mehrabany E, Ranjbar F, Asghari-Jafarabadi M, Hosseinpour-Arjmand S, Ebrahimi-Mameghani M. Calorie restriction in combination with prebiotic supplementation in obese women with depression: effects on metabolic and clinical response. Nutritional Neuroscience. 2021;24(5):339-53.

83.	Li Y, Li L, Guo R, Yu H, Wang X, Wang B, et al. Clinical efficacy of Shugan granule in the treatment of mixed anxiety-depressive disorder: a multicenter, randomized, double-blind, placebo-controlled trial. 2022;290:115032

C3 - PUBMED 35085742,EMBASE 637131287.

84.	Verma DK, Das B. Effect of zinc supplementation on antidepressant therapy and serum zinc level in unipolar depression: A randomized double blind controlled study. Indian Journal of Psychiatry. 2016;58(5):S47.

85.	Levine J, Barak Y, Kofman O, Belmaker RH. Follow-up and relapse analysis of an inositol study of depression. Isr J Psychiatry Relat Sci. 1995;32(1):14-21.

86.	Davidson JRT, Gadde KM, Fairbank JA, Ranga Rama Krishnan K, Califf RM, Binanay C, et al. Effect of Hypericum perforatum (St John's wort) in major depressive disorder: A randomized controlled trial. JAMA. 2002;287(14):1807-14.

87.	Sultana S, Rahman MS. Effect of magnesium supplementation in selective serotonin reuptake inhibitors treated major depressive disorder patients: A randomized, double-blind, placebo-controlled trial. EC Nutr. 2023;18:25-34.

88.	Vellekkatt F, Menon V, Rajappa M, Sahoo J. Effect of adjunctive single dose parenteral Vitamin D supplementation in major depressive disorder with concurrent vitamin D deficiency: A double-blind randomized placebo-controlled trial. J Psychiatr Res. 2020;129:250-6.

89.	Masoumi SZ, Kazemi F, Tavakolian S, Rahimi A, Oshvandi K, Soltanian A, et al. Effect of citalopram in combination with omega-3 on depression in post-menopausal women: A triple blind randomized controlled trial. Journal of Clinical and Diagnostic Research. 2016;10(10):QC01-QC5.

90.	Nowak G, Siwek M, Dudek D, Zieba A, Pilc A. Effect of zinc supplementation on antidepressant therapy in unipolar depression: A preliminary placebo-controlled study. Polish Journal of Pharmacology. 2003;55(6):1143-7.

91.	Mech AW, Farah A. Correlation of clinical response with homocysteine reduction during therapy with reduced b vitamins in patients with mdd who are positive for MTHFR C677T or A1298C polymorphism: A Randomized, double-blind, placebo-controlled study. Journal of Clinical Psychiatry. 2016;77(5):668-71.

92.	Ghajar A, Neishabouri SM, Velayati N, Jahangard L, Matinnia N, Haghighi M, et al. Crocus sativus L versus Citalopram in the Treatment of Major Depressive Disorder with Anxious Distress: A Double-Blind, Controlled Clinical Trial. Pharmacopsychiatry. 2017;50(4):152-60.

93.	Jazayeri S, Tehrani-Doost M, Keshavarz SA, Hosseini M, Djazayery A, Amini H, et al. Comparison of therapeutic effects of omega-3 fatty acid eicosapentaenoic acid and fluoxetine, separately and in combination, in major depressive disorder. Australian and New Zealand Journal of Psychiatry. 2008;42(3):192-8.

94.	Ahmadpanah M, Ramezanshams F, Ghaleiha A, Akhondzadeh S, Sadeghi Bahmani D, Brand S. Crocus Sativus L. (saffron) versus sertraline on symptoms of depression among older people with major depressive disorders–a double-blind, randomized intervention study. Psychiatry Research. 2019;282.

95.	Woelk H. Comparison of St John's wort and imipramine for treating depression: Randomised controlled trial. British Medical Journal. 2000;321(7260):536-9.

96.	Moshiri E, Basti AA, Noorbala AA, Jamshidi AH, Hesameddin Abbasi S, Akhondzadeh S. Crocus sativus L. (petal) in the treatment of mild-to-moderate depression: A double-blind, randomized and placebo-controlled trial. Phytomedicine. 2006;13(9-10):607-11.

97.	Harrer G, Schmidt U, Kuhn U, Biller A. Comparison of equivalence between the St. John's wort extract LoHyp-57 and fluoxetine. Arzneimittel-Forschung/Drug Research. 1999;49(4):289-96.

98.	Kashani L, Eslatmanesh S, Saedi N, Niroomand N, Ebrahimi M, Hosseinian M, et al. Comparison of Saffron versus Fluoxetine in Treatment of Mild to Moderate Postpartum Depression: A Double-Blind, Randomized Clinical Trial. Pharmacopsychiatry. 2017;50(2):64-8.

99.	Berk M, Dean OM, Cotton SM, Jeavons S, Tanious M, Kohlmann K, et al. The efficacy of adjunctive N-acetylcysteine in major depressive disorder: A double-blind, randomized, placebo-controlled trial. Journal of Clinical Psychiatry. 2014;75(6):628-36.

100.	Lopresti AL, Maes M, Maker GL, Hood SD, Drummond PD. Curcumin for the treatment of major depression: A randomised, double-blind, placebo controlled study. Journal of Affective Disorders. 2014;167:368-75.

101.	Lôo H, Poirier MF, Ollat H, Elatki S. Effects of sulbutiamine (Arcalion 200) on psycho-behavioral inhibition in major depressive episodes. 2000;26(2):70‐5

C3 - PUBMED 10858919,EMBASE 30337124.

102.	Noorbala AA, Akhondzadeh S, Tahmacebi-Pour N, Jamshidi AH. Hydro-alcoholic extract of Crocus sativus L. versus fluoxetine in the treatment of mild to moderate depression: A double-blind, randomized pilot trial. Journal of Ethnopharmacology. 2005;97(2):281-4.

103.	Behnke K, Jensen GS, Graubaum HJ, Gruenwald J. Hypericum perforatum versus fluoxetine in the treatment of mild to moderate depression. Advances in Therapy. 2002;19(1):43-52.

104.	Schrader E, Meier B, Brattström A. Hypericum treatment of mild-moderate depression in a placebo-controlled study. A prospective, double-blind, randomized, placebo-controlled, multicentre study. Human Psychopharmacology. 1998;13(3):163-9.

105.	Schrader E. Equivalence of St John's wort extract (Ze 117) and fluoxetine: A randomized, controlled study in mild-moderate depression. International Clinical Psychopharmacology. 2000;15(2):61-8.

106.	Coppen A, Bailey J. Enhancement of the antidepressant action of fluoxetine by folic acid: A randomised, placebo controlled trial. Journal of Affective Disorders. 2000;60(2):121-30.

107.	Amini L, Yaghini O, Ghazavi M, Aslani N. L-carnitine versus Propranolol for pediatric migraine prophylaxis. Iran J Child Neurol. 2021;15(2):77-86.

108.	Ghorbani Z, Nazari S, Etesam F, Nourimajd S, Ahmadpanah M, Jahromi SR. The effect of synbiotic as an adjuvant therapy to fluoxetine in moderate depression: A randomized multicenter trial. Arch Neurosci. 2018;5(2).

109.	Açik M, Çakiroğlu FP, Tekin A, Egeli A. The effect of fish oil versus krill oil intervention on clinical symptoms and cardiometabolic risk factors in patients with major depressive disorder: A randomized placebo-controlled double-blind trial. Journal of Affective Disorders. 2025;380:171-80.

110.	Papakostas GI, Mischoulon D, Shyu I, Alpert JE, Fava M. S-adenosyl methionine (SAMe) augmentation of serotonin reuptake inhibitors for antidepressant nonresponders with major depressive disorder: a double-blind, randomized clinical trial. 2010;167(8):942‐8

C3 - PUBMED 20595412.

111.	Gelenberg AJ, Shelton RC, Crits-Christoph P, Keller MB, Dunner DL, Hirschfeld RM, et al. The effectiveness of St. John's Wort in major depressive disorder: a naturalistic phase 2 follow-up in which nonresponders were provided alternate medication. 2004;65(8):1114‐9

C3 - PUBMED 15323598.

112.	Shafiee-Kandjani A, Khalili M, Malek A, Farhang S, Ranjbari Y, Khalili Y. The therapeutic effect of the extracts of Lavandula angustifolia and Dracocephalum ruyschiana besides sertraline on patients with mild to moderate depression: A double-blind controlled trial. Phytomedicine Plus. 2023;3(2).

113.	Talaei A, Noori R, Rezaei Ardani A, Mohammadpour AH, Azimipoor Z, Afzaljavan F. The Effect of Adding Curcumin to Sertraline in the Treatment of Severe Major Depressive Disorder: A Randomized, Double-Blind Clinical Trial. Clinical Neuropharmacology. 2023;46(4):135-9.

114.	Kasper S, Anghelescu IG, Szegedi A, Dienel A, Kieser M. Superior efficacy of St John's wort extract WS 5570 compared to placebo in patients with major depression: a randomized, double-blind, placebo-controlled, multi-center trial. 2006;4:14

C3 - PUBMED 16796730,EMBASE 44227549.

115.	Sadeghi A, Ghorayshi F, Baghshahi H, Akbari H, Memarzadeh MR, Taghizadeh M, et al. The antidepressant effect of combined extracts of Hypericum perforatum and Echium amoenum supplementation in patients with depression symptoms: A randomized clinical trial. Avicenna J Phytomedicine. 2023;13(4):328-37.

116.	Park Y, Park YS, Kim SH, Oh DH, Park YC. Supplementation of n-3 polyunsaturated fatty acids for major depressive disorder: A randomized, double-blind, 12-week, placebo-controlled trial in Korea. Annals of Nutrition and Metabolism. 2015;66(2-3):141-8.

117.	De Leo D. S-Adenosylmethionine as an antidepressant. A double-blind trial versus placebo. CURR THER RES CLIN EXP. 1987;41(6):865-70.

118.	Cheng YC, Chen WY, Lin C, Lee SH, Chiu CC, Kuo PH. The N-3 polyunsaturated fatty acids supplementation to prevent depression recurrence in patients with late-life depression: A 52-week double-blind, placebo-controlled trial. Journal of Affective Disorders. 2025;369:8-15.

119.	Kreuzer K, Reiter A, Birkl-Töglhofer AM, Dalkner N, Mörkl S, Mairinger M, et al. The PROVIT Study—Effects of Multispecies Probiotic Add-on Treatment on Metabolomics in Major Depressive Disorder—A Randomized, Placebo-Controlled Trial. Metabolites. 2022;12(8).

120.	Sarris J, Fava M, Schweitzer I, Mischoulon D. St Johns Wort (Hypericum perforatum) versus sertraline and placebo in major depressive disorder: Continuation data from a 26-week RCT. Pharmacopsychiatry. 2012;45(7):275-8.

121.	Jelodar G, Javid Z, Sahraian A, Jelodar S. Saffron improved depression and reduced homocysteine level in patients with major depression: A Randomized, double-blind study. Avicenna J Phytomedicine. 2018;8(1):43-50.

122.	Khoraminya N, Tehrani-Doost M, Jazayeri S, Hosseini A, Djazayery A. Therapeutic effects of vitamin D as adjunctive therapy to fluoxetine in patients with major depressive disorder. Australian and New Zealand Journal of Psychiatry. 2013;47(3):271-5.

123.	Sepehrmanesh Z, Kolahdooz F, Abedi F, Mazroii N, Assarian A, Asemi Z, et al. Vitamin D supplementation affects the beck depression inventory, insulin resistance, and biomarkers of oxidative stress in patients with major depressive disorder: A randomized, controlled clinical trial. Journal of Nutrition. 2016;146(2):243-8.

124.	Sahoo JP, Jarnail S, Khurana H, Singh P. Omega-3 Fatty Acids Versus 5-hydroxytryptophan as Add on Therapy in Patients with Depression: A Comparative Study. Indian Journal of Pharmacology. 2015;47(7):S23-S4.

125.	Laakmann G. St. John's Wort in mild to moderate depression: The relevance of hyperforin for the clinical efficacy. Pharmacopsychiatry. 1998;31(SUPPL. 1):54-9.

126.	Chang JP, Chang SS, Yang HT, Chen HT, Chien YC, Yang B, et al. Omega-3 polyunsaturated fatty acids in cardiovascular diseases comorbid major depressive disorder - Results from a randomized controlled trial. Brain Behav Immun. 2020;85:14-20.

127.	Saccarello A, Montarsolo P, Massardo I, Picciotto R, Pedemonte A, Castagnaro R, et al. Oral Administration of S-Adenosylmethionine (SAMe) and Lactobacillus Plantarum HEAL9 Improves the Mild-To-Moderate Symptoms of Depression: a Randomized, Double-Blind, Placebo-Controlled Study. 2020;22(4 C3 - PUBMED 32589828,EMBASE 632201612).

128.	Friede M, Henneicke von Zepelin HH, Freudenstein J. Differential therapy of mild to moderate depressive episodes (ICD-10 F 32.0; F 32.1) with St. John's wort. Pharmacopsychiatry. 2001;34(SUPPL. 1):S38-S41.

129.	Ning H, Zhou H, Ren J, Zhou G, Yang N, Wang Z, et al. Zishen pingchan granules combined with pramipexole in the improvement of depressive symptoms in Parkinson's disease: a prospective, multicenter, randomized, double-blind, controlled clinical study. J Transl Med. 2022;20(1):357.

130.	Bergman J, Miodownik C, Bersudsky Y, Sokolik S, Lerner PP, Kreinin A, et al. Curcumin as an add-on to antidepressive treatment: A randomized, double-blind, placebo-controlled, pilot clinical study. Clinical Neuropharmacology. 2013;36(3):73-7.

131.	Kasper S, Anghelescu IG, Szegedi A, Dienel A, Kieser M. Placebo controlled continuation treatment with Hypericum extract WS® 5570 after recovery from a mild or moderate depressive episode. Wiener Medizinische Wochenschrift. 2007;157(13-14):362-6.

132.	Bot M, Pouwer F, Assies J, Jansen EH, Diamant M, Snoek FJ, et al. Eicosapentaenoic acid as an add-on to antidepressant medication for co-morbid major depression in patients with diabetes mellitus: a randomized, double-blind placebo-controlled study. J Affect Disord. 2010;126(1-2):282-6.

133.	Mozaffari-Khosravi H, Nabizade L, Yassini-Ardakani SM, Hadinedoushan H, Barzegar K. The effect of 2 different single injections of high dose of vitamin D on improving the depression in depressed patients with vitamin D deficiency: A randomized clinical trial. Journal of Clinical Psychopharmacology. 2013;33(3):378-85.

134.	Sarris J, Murphy J, Stough C, Mischoulon D, Bousman C, MacDonald P, et al. S-Adenosylmethionine (SAMe) monotherapy for depression: an 8-week double-blind, randomised, controlled trial. 2020;237(1):209‐18

C3 - PUBMED 31712971,EMBASE 2003633556.

135.	Bell KM, Plon L, Bunney Jr WE, Potkin SG. S-adenosylmethionine treatment of depression: A controlled clinical trial. American Journal of Psychiatry. 1988;145(9):1110-4.

136.	Siwek M, Dudek D, Schlegel-Zawadzka M, Morawska A, Piekoszewski W, Opoka W, et al. Serum zinc level in depressed patients during zinc supplementation of imipramine treatment. Journal of Affective Disorders. 2010;126(3):447-52.

137.	Papakostas GI, Shelton RC, Zajecka JM, Etemad B, Rickels K, Clain A, et al. L-methylfolate as adjunctive therapy for SSRI-resistant major depression: results of two randomized, double-blind, parallel-sequential trials. 2012;169(12):1267‐74

C3 - PUBMED 23212058.

138.	Majmasanaye M, Mehrpooya M, Amiri H, Eshraghi A. Discovering the Potential Value of Coenzyme Q10 as an Adjuvant Treatment in Patients with Depression. Journal of Clinical Psychopharmacology. 2024;44(3):232-9.

139.	Hvas AM, Juul S, Lauritzen L, Nexø E, Ellegaard J. No effect of vitamin B-12 treatment on cognitive function and depression: A randomized placebo controlled study. Journal of Affective Disorders. 2004;81(3):269-73.

140.	Bidzan-Wiącek M, Tomczyk M, Błażek M, Mika A, Antosiewicz J. No Effects of Omega-3 Supplementation on Kynurenine Pathway, Inflammation, Depressive Symptoms, and Stress Response in Males: A Placebo-Controlled Trial. Nutrients. 2024;16(21 C7 - 3744).

141.	Sarris J, Byrne GJ, Stough C, Bousman C, Mischoulon D, Murphy J, et al. Nutraceuticals for major depressive disorder- more is not merrier: An 8-week double-blind, randomised, controlled trial. Journal of Affective Disorders. 2019;245:1007-15.

142.	Wu SK, Yang KJ, Liu WC, Malau IA, Zailani H, Chang CH, et al. The Efficacy of Omega-3 Fatty Acids as the Monotherapy for Depression: A Randomized, Double-Blind, Placebo-Controlled Pilot Study. Nutrients. 2024;16(21).

143.	Zhang L, Wang S, Zhu Y, Yang T. Vitamin D3 as adjunctive therapy in the treatment of depression in tuberculosis patients: A short-term pilot randomized double-blind controlled study. Neuropsychiatric Disease and Treatment. 2018;14:3103-9.

144.	Kasper S, Volz HP, Möller HJ, Schläfke S, Klement S, Anghelescu IG, et al. Lavender oil preparation Silexan is effective in mild-to-moderate major depression: a randomized, placebo- and reference-controlled trial. European Archives of Psychiatry and Clinical Neuroscience. 2024.

145.	Kolouri S, Firoozabadi A, Salehi A, Zarshenas MM, Dastgheib SA, Heydari M, et al. Nepeta menthoides Boiss. & Buhse freeze-dried aqueous extract versus sertraline in the treatment of major depression: A double blind randomized controlled trial. Complement Ther Med. 2016;26:164-70.

146.	Rees AM, Austin MP, Parker GB. Omega-3 fatty acids as a treatment for perinatal depression: Randomized double-blind placebo-controlled trial. Australian and New Zealand Journal of Psychiatry. 2008;42(3):199-205.

147.	Freeman MP, Davis M, Sinha P, Wisner KL, Hibbeln JR, Gelenberg AJ. Omega-3 fatty acids and supportive psychotherapy for perinatal depression: A randomized placebo-controlled study. Journal of Affective Disorders. 2008;110(1-2):142-8.

148.	Varzaghani V, Sharifi M, Hajiaghaee R, Bagheri S, Momtaz S, Tarassoli Z, et al. Propolis add-on therapy alleviates depressive symptoms; A randomized placebo-controlled clinical trial. Phytotherapy Research. 2022;36(3):1258-67.

149.	Shinto L, Marracci G, Mohr DC, Bumgarner L, Murchison C, Senders A, et al. Omega-3 fatty acids for depression in multiple sclerosis: A randomized pilot study. PLoS ONE. 2016;11(1 C7 - e0147195).

150.	Su KP, Huang SY, Chiu TH, Huang KC, Huang CL, Chang HC, et al. Omega-3 fatty acids for major depressive disorder during pregnancy: Results from a randomized, double-blind, placebo-controlled trial. Journal of Clinical Psychiatry. 2008;69(4):644-51.

151.	Mischoulon D, Dunlop BW, Kinkead B, Schettler PJ, Lamon-Fava S, Rakofsky JJ, et al. Omega-3 Fatty Acids for Major Depressive Disorder With High Inflammation: A Randomized Dose-Finding Clinical Trial. Journal of Clinical Psychiatry. 2022;83(5 C7 - 21m14074).

152.	Su KP, Huang SY, Chiu CC, Shen WW. Omega-3 fatty acids in major depressive disorder: A preliminary double-blind, placebo-controlled trial. European Neuropsychopharmacology. 2003;13(4):267-71.

153.	Yang R, Wang L, Jin K, Cao S, Wu C, Guo J, et al. Omega-3 Polyunsaturated Fatty Acids Supplementation Alleviate Anxiety Rather Than Depressive Symptoms Among First-Diagnosed, Drug-Naïve Major Depressive Disorder Patients: A Randomized Clinical Trial. Frontiers in Nutrition. 2022;9 C7 - 876152.

154.	Kagan BL, Sultzer DL, Rosenlicht N, Gerner RH. Oral S-adenosylmethionine in depression: a randomized, double-blind, placebo-controlled trial. 1990;147(5):591‐5

C3 - PUBMED 2183633.

155.	Han CH, Chung MI, Bose S, Lim C, Kim H, Koo BS, et al. The effect of Gami Guibitang (GGBT), a medicinal herbal formulation, on moderate depression. European Journal of Integrative Medicine. 2012;4(4):e400-e7.

156.	Pancheri P, Scapicchio P, Delle Chiaie R. A double-blind, randomized parallel-group, efficacy and safety study of intramuscular S-adenosyl-L-methionine 1, 4-butanedisulphonate (SAMe) versus imipramine in patients with major depressive disorder. International Journal of Neuropsychopharmacology. 2002;5(4):287-94.

157.	Mischoulon D, Price LH, Carpenter LL, Tyrka AR, Papakostas GI, Baer L, et al. A double-blind, randomized, placebo-controlled clinical trial of S-adenosyl-L-methionine (SAMe) versus escitalopram in major depressive disorder. J Clin Psychiatry. 2014;75(4):370-6.

158.	Mao JJ, Xie SX, Zee J, Soeller I, Li QS, Rockwell K, et al. Rhodiola rosea versus sertraline for major depressive disorder: A randomized placebo-controlled trial. Phytomedicine. 2015;22(3):394-9.

159.	Sempach L, Schaub A-C, Brühl AB, Sterzer P, Lang UE, Schmidt A. Adjunctive d-serine treatment for major depressive disorder: A randomized clinical trial. Journal of Affective Disorders. 2025:119504.

**e-table 8:** Anxious symptoms node 1: subnetworks.

| **Author(s), year** | **Treatment** | **N** | **Subnetwork** |
| --- | --- | --- | --- |
| (Sarris, Byrne et al. 2019)^551^ | NUT | 56 | 1 |
| (Sarris, Byrne et al. 2019)^551^ | PLB | 57 | 1 |
| (Bidzan-Wiącek, Tomczyk et al. 2024)^471^ | EPA+DHA | 25 | 1 |
| (Bidzan-Wiącek, Tomczyk et al. 2024)^471^ | PLB | 22 | 1 |
| (Sarris, Murphy et al. 2020)^552^ | SAMe | 22 | 1 |
| (Sarris, Murphy et al. 2020)^552^ | PLB | 19 | 1 |
| (Cheng, Chen et al. 2025)^479^ | EPA+DHA | 19 | 1 |
| (Cheng, Chen et al. 2025)^479^ | PLB | 20 | 1 |
| (Lopresti, Maes et al. 2014)^221^ | CUR | 28 | 1 |
| (Lopresti, Maes et al. 2014)^221^ | PLB | 28 | 1 |
| (Li, Li et al. 2022)^520^ | SHU | 194 | 1 |
| (Li, Li et al. 2022)^520^ | PLB | 191 | 1 |
| (Piperoglou, Hopwood et al. 2023)^541^ | EPA+DHA | 14 | 1 |
| (Piperoglou, Hopwood et al. 2023)^541^ | PLB | 9 | 1 |
| (Lopresti and Drummond 2017)^523^ | CUR | 61 | 1 |
| (Lopresti and Drummond 2017)^523^ | CUR+SAFF | 26 | 1 |
| (Lopresti and Drummond 2017)^523^ | PLB | 36 | 1 |
| (Kasper, Anghelescu et al. 2015)^512^ | SIL | 141 | 1 |
| (Kasper, Anghelescu et al. 2015)^512^ | PLB | 128 | 1 |
| (Shahmoradi, Davarinejad et al. 2023)^559^ | APH | 21 | 1 |
| (Shahmoradi, Davarinejad et al. 2023)^559^ | PLB | 19 | 1 |
| (Shelton, Keller et al. 2001)^562^ | SJW | 98 | 1 |
| (Shelton, Keller et al. 2001)^562^ | PLB | 102 | 1 |
| (Mazidi, Shemshian et al. 2016)^527^ | SAF | 30 | 1 |
| (Mazidi, Shemshian et al. 2016)^527^ | PLB | 30 | 1 |
| (Sempach, Schaub et al. 2025)^555^ | SRN+TAU | 22 | 2 |
| (Sempach, Schaub et al. 2025)^555^ | PLB+TAU | 22 | 2 |
| (Berk, Dean et al. 2014)^470^ | NAC+TAU | 125 | 2 |
| (Berk, Dean et al. 2014)^470^ | PLB+TAU | 127 | 2 |
| (Sarris, Byrne et al. 2018)^550^ | SAMe+TAU | 37 | 2 |
| (Sarris, Byrne et al. 2018)^550^ | PLB+TAU | 39 | 2 |
| (Talaei, Hassanpour Moghadam et al. 2015)^590^ | SAFF+TAU | 20 | 2 |
| (Talaei, Hassanpour Moghadam et al. 2015)^590^ | PLB+TAU | 20 | 2 |
| (Talaei, Noori et al. 2023)^568^ | SER+CUR | 20 | 3 |
| (Talaei, Noori et al. 2023)^568^ | SER+PLB | 20 | 3 |
| (Carney, Freedland et al. 2019)^475^ | SER+EPA | 71 | 3 |
| (Carney, Freedland et al. 2019)^475^ | SER+PLB | 73 | 3 |
| (Gul, Memtily et al. 2019)^602^ | US | 50 | 4 |
| (Gul, Memtily et al. 2019)^602^ | FLUO | 50 | 4 |
| (Gul, Memtily et al. 2019)^602^ | US+FLUO | 50 | 4 |
| (Sepehrmanesh, Kolahdooz et al. 2016)^581^ | FOL+CTP | 45 | 5 |
| (Sepehrmanesh, Kolahdooz et al. 2016)^581^ | CTP+PLB | 45 | 5 |
| (Firoozeei, Barekatain et al. 2020)^579^ | LAV+PLB | 25 | 5 |
| (Firoozeei, Barekatain et al. 2020)^579^ | CTP+PLB | 25 | 5 |
| (Verma and Das 2016)^573^ | ZNC+TAU | 30 | 6 |
| (Verma and Das 2016)^573^ | TAU | 30 | 6 |
| (Lôo, Poirier et al. 2000)^605^ | SUL+CLO | 36 | 7 |
| (Lôo, Poirier et al. 2000)^605^ | PLB+CLO | 39 | 7 |
| (Jahangard, Sadeghi et al. 2018)^587^ | OMEGA3+SER | 25 | 8 |
| (Jahangard, Sadeghi et al. 2018)^587^ | PLB+SER | 25 | 8 |
| (Ghajar, Neishabouri et al. 2017)^495^ | SAFF | 30 | 9 |
| (Ghajar, Neishabouri et al. 2017)^495^ | CTP | 30 | 9 |
| (Yang, Wang et al. 2022)^595^ | EPA+DHA+VEN | 21 | 10 |
| (Yang, Wang et al. 2022)^595^ | PLB+VEN | 27 | 10 |

**Reference for e-table 8**

1.	Mazidi M, Shemshian M, Mousavi SH, Norouzy A, Kermani T, Moghiman T, et al. A double-blind, randomized and placebo-controlled trial of Saffron (Crocus sativus L.) in the treatment of anxiety and depression. 2016;13(2):195‐9

C3 - PUBMED 27101556,EMBASE 610483123.

2.	Carney RM, Freedland KE, Rubin EH, Rich MW, Steinmeyer BC, Harris WS. A randomized placebo-controlled trial of omega-3 and sertraline in depressed patients with or at risk for coronary heart disease. Journal of Clinical Psychiatry. 2019;80(4 C7 - 19m12742).

3.	Talaei A, Hassanpour Moghadam M, Sajadi Tabassi SA, Mohajeri SA. Crocin, the main active saffron constituent, as an adjunctive treatment in major depressive disorder: A randomized, double-blind, placebo-controlled, pilot clinical trial. Journal of Affective Disorders. 2015;174:51-6.

4.	Jahangard L, Sadeghi A, Ahmadpanah M, Holsboer-Trachsler E, Sadeghi Bahmani D, Haghighi M, et al. Influence of adjuvant omega-3-polyunsaturated fatty acids on depression, sleep, and emotion regulation among outpatients with major depressive disorders - Results from a double-blind, randomized and placebo-controlled clinical trial. Journal of Psychiatric Research. 2018;107:48-56.

5.	Firoozeei TS, Barekatain M, Karimi M, Zargaran A, Akhondzadeh S, Rezaeizadeh H. Lavender and dodder combined herbal syrup versus citalopram in major depressive disorder with anxious distress: A double-blind randomized trial. Journal of Integrative Medicine. 2020;18(5):409-15.

6.	Shelton RC, Keller MB, Gelenberg A, Dunner DL, Hirschfeld R, Thase ME, et al. Effectiveness of St John's wort in major depression: A randomized controlled trial. JAMA. 2001;285(15):1978-86.

7.	Shahmoradi N, Davarinejad O, Brühl AB, Brand S. Effects of Aphrodite (an Herbal Compound) on SSRI-Induced Sexual Dysfunctions and Depression in Females with Major Depressive Disorder: Findings from a Randomized Clinical Trial. Medicina (Kaunas, Lithuania). 2023;59(9).

8.	Kasper S, Anghelescu I, Dienel A. Efficacy of orally administered Silexan in patients with anxiety-related restlessness and disturbed sleep--A randomized, placebo-controlled trial. 2015;25(11):1960‐7

C3 - PUBMED 26293583,EMBASE 605643089.

9.	Lopresti AL, Drummond PD. Efficacy of curcumin, and a saffron/curcumin combination for the treatment of major depression: A randomised, double-blind, placebo-controlled study. Journal of Affective Disorders. 2017;207:188-96.

10.	Gul A, Memtily N, Mijit P, Imin M, Rui H, Wushuer P, et al. The Üstikuddus sherbiti in the treatment of abnormal cold and dry-type depression patients with comorbid anxiety: A randomized and controlled clinical trial. Trad Med Mod Med. 2019;2(3):127-33.

11.	Sepehrmanesh Z, Kolahdooz F, Abedi F, Mazroii N, Assarian A, Asemi Z, et al. Vitamin D Supplementation Affects the Beck Depression Inventory, Insulin Resistance, and Biomarkers of Oxidative Stress in Patients with Major Depressive Disorder: A Randomized, Controlled Clinical Trial [Retraction notice in CN-02298463]. 2016;146(2):243‐8

C3 - PUBMED 26609167,EMBASE 608794678.

12.	Sarris J, Byrne GJ, Bousman C, Stough C, Murphy J, MacDonald P, et al. Adjunctive S-adenosylmethionine (SAMe) in treating non-remittent major depressive disorder: An 8-week double-blind, randomized, controlled trial. European Neuropsychopharmacology. 2018;28(10):1126-36.

13.	Piperoglou M, Hopwood M, Norman TR. Adjunctive Docosahexaenoic Acid in Residual Symptoms of Depression and Anxiety. Journal of Clinical Psychopharmacology. 2023;43(6):493-7.

14.	Li Y, Li L, Guo R, Yu H, Wang X, Wang B, et al. Clinical efficacy of Shugan granule in the treatment of mixed anxiety-depressive disorder: a multicenter, randomized, double-blind, placebo-controlled trial. 2022;290:115032

C3 - PUBMED 35085742,EMBASE 637131287.

15.	Verma DK, Das B. Effect of zinc supplementation on antidepressant therapy and serum zinc level in unipolar depression: A randomized double blind controlled study. Indian Journal of Psychiatry. 2016;58(5):S47.

16.	Ghajar A, Neishabouri SM, Velayati N, Jahangard L, Matinnia N, Haghighi M, et al. Crocus sativus L versus Citalopram in the Treatment of Major Depressive Disorder with Anxious Distress: A Double-Blind, Controlled Clinical Trial. Pharmacopsychiatry. 2017;50(4):152-60.

17.	Berk M, Dean OM, Cotton SM, Jeavons S, Tanious M, Kohlmann K, et al. The efficacy of adjunctive N-acetylcysteine in major depressive disorder: A double-blind, randomized, placebo-controlled trial. Journal of Clinical Psychiatry. 2014;75(6):628-36.

18.	Lopresti AL, Maes M, Maker GL, Hood SD, Drummond PD. Curcumin for the treatment of major depression: A randomised, double-blind, placebo controlled study. Journal of Affective Disorders. 2014;167:368-75.

19.	Lôo H, Poirier MF, Ollat H, Elatki S. Effects of sulbutiamine (Arcalion 200) on psycho-behavioral inhibition in major depressive episodes. 2000;26(2):70‐5

C3 - PUBMED 10858919,EMBASE 30337124.

20.	Talaei A, Noori R, Rezaei Ardani A, Mohammadpour AH, Azimipoor Z, Afzaljavan F. The Effect of Adding Curcumin to Sertraline in the Treatment of Severe Major Depressive Disorder: A Randomized, Double-Blind Clinical Trial. Clinical Neuropharmacology. 2023;46(4):135-9.

21.	Cheng YC, Chen WY, Lin C, Lee SH, Chiu CC, Kuo PH. The N-3 polyunsaturated fatty acids supplementation to prevent depression recurrence in patients with late-life depression: A 52-week double-blind, placebo-controlled trial. Journal of Affective Disorders. 2025;369:8-15.

22.	Sarris J, Murphy J, Stough C, Mischoulon D, Bousman C, MacDonald P, et al. S-Adenosylmethionine (SAMe) monotherapy for depression: an 8-week double-blind, randomised, controlled trial. 2020;237(1):209‐18

C3 - PUBMED 31712971,EMBASE 2003633556.

23.	Bidzan-Wiącek M, Tomczyk M, Błażek M, Mika A, Antosiewicz J. No Effects of Omega-3 Supplementation on Kynurenine Pathway, Inflammation, Depressive Symptoms, and Stress Response in Males: A Placebo-Controlled Trial. Nutrients. 2024;16(21 C7 - 3744).

24.	Sarris J, Byrne GJ, Stough C, Bousman C, Mischoulon D, Murphy J, et al. Nutraceuticals for major depressive disorder- more is not merrier: An 8-week double-blind, randomised, controlled trial. Journal of Affective Disorders. 2019;245:1007-15.

25.	Yang R, Wang L, Jin K, Cao S, Wu C, Guo J, et al. Omega-3 Polyunsaturated Fatty Acids Supplementation Alleviate Anxiety Rather Than Depressive Symptoms Among First-Diagnosed, Drug-Naïve Major Depressive Disorder Patients: A Randomized Clinical Trial. Frontiers in Nutrition. 2022;9 C7 - 876152.

26.	Sempach L, Schaub A-C, Brühl AB, Sterzer P, Lang UE, Schmidt A. Adjunctive d-serine treatment for major depressive disorder: A randomized clinical trial. Journal of Affective Disorders. 2025:119504.

**e-table 9:** Anxious symptoms node 2.

| **Author(s), year** | **Treatment** | **N** | **Subnetwork** |
| --- | --- | --- | --- |
| (Sarris, Byrne et al. 2019)^551^ | Coenzyme+Vitamins+LCPUFA+Minerals+Aminoacids | 56 | 1 |
| (Sarris, Byrne et al. 2019)^551^ | PLB | 57 | 1 |
| (Bidzan-Wiącek, Tomczyk et al. 2024)^471^ | LCPUFA | 25 | 1 |
| (Bidzan-Wiącek, Tomczyk et al. 2024)^471^ | PLB | 22 | 1 |
| (Sarris, Murphy et al. 2020)^552^ | Coenzyme | 22 | 1 |
| (Sarris, Murphy et al. 2020)^552^ | PLB | 19 | 1 |
| (Cheng, Chen et al. 2025)^479^ | LCPUFA | 19 | 1 |
| (Cheng, Chen et al. 2025)^479^ | PLB | 20 | 1 |
| (Lopresti, Maes et al. 2014)^221^ | Zingiberaceae | 28 | 1 |
| (Lopresti, Maes et al. 2014)^221^ | PLB | 28 | 1 |
| (Ghajar, Neishabouri et al. 2017)^495^ | Iridaceae | 30 | 1 |
| (Ghajar, Neishabouri et al. 2017)^495^ | SSRI | 30 | 1 |
| (Li, Li et al. 2022)^520^ | Herbal compound | 194 | 1 |
| (Li, Li et al. 2022)^520^ | PLB | 191 | 1 |
| (Piperoglou, Hopwood et al. 2023)^541^ | LCPUFA | 14 | 1 |
| (Piperoglou, Hopwood et al. 2023)^541^ | PLB | 9 | 1 |
| (Gul, Memtily et al. 2019)^602^ | Herbal compound | 50 | 1 |
| (Gul, Memtily et al. 2019)^602^ | SSRI | 50 | 1 |
| (Gul, Memtily et al. 2019)^602^ | Herbal compound+SSRI | 50 | 1 |
| (Lopresti and Drummond 2017)^523^ | Zingiberaceae | 61 | 1 |
| (Lopresti and Drummond 2017)^523^ | Zingiberaceae+Iridaceae | 26 | 1 |
| (Lopresti and Drummond 2017)^523^ | PLB | 36 | 1 |
| (Kasper, Anghelescu et al. 2015)^512^ | Lamiaceae | 141 | 1 |
| (Kasper, Anghelescu et al. 2015)^512^ | PLB | 128 | 1 |
| (Shahmoradi, Davarinejad et al. 2023)^559^ | Herbal compound | 21 | 1 |
| (Shahmoradi, Davarinejad et al. 2023)^559^ | PLB | 19 | 1 |
| (Shelton, Keller et al. 2001)^562^ | HYP | 98 | 1 |
| (Shelton, Keller et al. 2001)^562^ | PLB | 102 | 1 |
| (Mazidi, Shemshian et al. 2016)^527^ | Iridaceae | 30 | 1 |
| (Mazidi, Shemshian et al. 2016)^527^ | PLB | 30 | 1 |
| (Talaei, Noori et al. 2023)^568^ | SSRI+Zingiberaceae | 20 | 2 |
| (Talaei, Noori et al. 2023)^568^ | SSRI+PLB | 20 | 2 |
| (Sepehrmanesh, Kolahdooz et al. 2016)^581^ | Vitamins+SSRI | 45 | 2 |
| (Sepehrmanesh, Kolahdooz et al. 2016)^581^ | SSRI+PLB | 45 | 2 |
| (Firoozeei, Barekatain et al. 2020)^579^ | Lamiaceae+PLB | 25 | 2 |
| (Firoozeei, Barekatain et al. 2020)^579^ | SSRI+PLB | 25 | 2 |
| (Jahangard, Sadeghi et al. 2018)^587^ | LCPUFA+SSRI | 25 | 2 |
| (Jahangard, Sadeghi et al. 2018)^587^ | SSRI+PLB | 25 | 2 |
| (Carney, Freedland et al. 2019)^475^ | SSRI+LCPUFA | 71 | 2 |
| (Carney, Freedland et al. 2019)^475^ | SSRI+PLB | 73 | 2 |
| (Sempach, Schaub et al. 2025)^555^ | Aminoacids+Antidepressants | 22 | 3 |
| (Sempach, Schaub et al. 2025)^555^ | Antidepressant+PLB | 22 | 3 |
| (Berk, Dean et al. 2014)^470^ | Aminoacids+Antidepressants | 125 | 3 |
| (Berk, Dean et al. 2014)^470^ | Antidepressant+PLB | 127 | 3 |
| (Sarris, Byrne et al. 2018)^550^ | Coenzyme+Antidepressants | 37 | 3 |
| (Sarris, Byrne et al. 2018)^550^ | Antidepressant+PLB | 39 | 3 |
| (Talaei, Hassanpour Moghadam et al. 2015)^590^ | Iridaceae+Antidepressant | 20 | 3 |
| (Talaei, Hassanpour Moghadam et al. 2015)^590^ | Antidepressant+PLB | 20 | 3 |
| (Lôo, Poirier et al. 2000)^605^ | Vitamins+TCA | 36 | 4 |
| (Lôo, Poirier et al. 2000)^605^ | TCA+PLB | 39 | 4 |
| (Verma and Das 2016)^573^ | Minerals+Antidepressants | 30 | 5 |
| (Verma and Das 2016)^573^ | Antidepressant | 30 | 5 |
| (Yang, Wang et al. 2022)^595^ | LCPUFA+SNRI | 21 | 6 |
| (Yang, Wang et al. 2022)^595^ | SNRI+PLB | 27 | 6 |

**Reference for e-table 9**

1.	Mazidi M, Shemshian M, Mousavi SH, Norouzy A, Kermani T, Moghiman T, et al. A double-blind, randomized and placebo-controlled trial of Saffron (Crocus sativus L.) in the treatment of anxiety and depression. 2016;13(2):195‐9

C3 - PUBMED 27101556,EMBASE 610483123.

2.	Carney RM, Freedland KE, Rubin EH, Rich MW, Steinmeyer BC, Harris WS. A randomized placebo-controlled trial of omega-3 and sertraline in depressed patients with or at risk for coronary heart disease. Journal of Clinical Psychiatry. 2019;80(4 C7 - 19m12742).

3.	Talaei A, Hassanpour Moghadam M, Sajadi Tabassi SA, Mohajeri SA. Crocin, the main active saffron constituent, as an adjunctive treatment in major depressive disorder: A randomized, double-blind, placebo-controlled, pilot clinical trial. Journal of Affective Disorders. 2015;174:51-6.

4.	Jahangard L, Sadeghi A, Ahmadpanah M, Holsboer-Trachsler E, Sadeghi Bahmani D, Haghighi M, et al. Influence of adjuvant omega-3-polyunsaturated fatty acids on depression, sleep, and emotion regulation among outpatients with major depressive disorders - Results from a double-blind, randomized and placebo-controlled clinical trial. Journal of Psychiatric Research. 2018;107:48-56.

5.	Firoozeei TS, Barekatain M, Karimi M, Zargaran A, Akhondzadeh S, Rezaeizadeh H. Lavender and dodder combined herbal syrup versus citalopram in major depressive disorder with anxious distress: A double-blind randomized trial. Journal of Integrative Medicine. 2020;18(5):409-15.

6.	Shelton RC, Keller MB, Gelenberg A, Dunner DL, Hirschfeld R, Thase ME, et al. Effectiveness of St John's wort in major depression: A randomized controlled trial. JAMA. 2001;285(15):1978-86.

7.	Shahmoradi N, Davarinejad O, Brühl AB, Brand S. Effects of Aphrodite (an Herbal Compound) on SSRI-Induced Sexual Dysfunctions and Depression in Females with Major Depressive Disorder: Findings from a Randomized Clinical Trial. Medicina (Kaunas, Lithuania). 2023;59(9).

8.	Kasper S, Anghelescu I, Dienel A. Efficacy of orally administered Silexan in patients with anxiety-related restlessness and disturbed sleep--A randomized, placebo-controlled trial. 2015;25(11):1960‐7

C3 - PUBMED 26293583,EMBASE 605643089.

9.	Lopresti AL, Drummond PD. Efficacy of curcumin, and a saffron/curcumin combination for the treatment of major depression: A randomised, double-blind, placebo-controlled study. Journal of Affective Disorders. 2017;207:188-96.

10.	Gul A, Memtily N, Mijit P, Imin M, Rui H, Wushuer P, et al. The Üstikuddus sherbiti in the treatment of abnormal cold and dry-type depression patients with comorbid anxiety: A randomized and controlled clinical trial. Trad Med Mod Med. 2019;2(3):127-33.

11.	Sepehrmanesh Z, Kolahdooz F, Abedi F, Mazroii N, Assarian A, Asemi Z, et al. Vitamin D Supplementation Affects the Beck Depression Inventory, Insulin Resistance, and Biomarkers of Oxidative Stress in Patients with Major Depressive Disorder: A Randomized, Controlled Clinical Trial [Retraction notice in CN-02298463]. 2016;146(2):243‐8

C3 - PUBMED 26609167,EMBASE 608794678.

12.	Sarris J, Byrne GJ, Bousman C, Stough C, Murphy J, MacDonald P, et al. Adjunctive S-adenosylmethionine (SAMe) in treating non-remittent major depressive disorder: An 8-week double-blind, randomized, controlled trial. European Neuropsychopharmacology. 2018;28(10):1126-36.

13.	Piperoglou M, Hopwood M, Norman TR. Adjunctive Docosahexaenoic Acid in Residual Symptoms of Depression and Anxiety. Journal of Clinical Psychopharmacology. 2023;43(6):493-7.

14.	Li Y, Li L, Guo R, Yu H, Wang X, Wang B, et al. Clinical efficacy of Shugan granule in the treatment of mixed anxiety-depressive disorder: a multicenter, randomized, double-blind, placebo-controlled trial. 2022;290:115032

C3 - PUBMED 35085742,EMBASE 637131287.

15.	Verma DK, Das B. Effect of zinc supplementation on antidepressant therapy and serum zinc level in unipolar depression: A randomized double blind controlled study. Indian Journal of Psychiatry. 2016;58(5):S47.

16.	Ghajar A, Neishabouri SM, Velayati N, Jahangard L, Matinnia N, Haghighi M, et al. Crocus sativus L versus Citalopram in the Treatment of Major Depressive Disorder with Anxious Distress: A Double-Blind, Controlled Clinical Trial. Pharmacopsychiatry. 2017;50(4):152-60.

17.	Berk M, Dean OM, Cotton SM, Jeavons S, Tanious M, Kohlmann K, et al. The efficacy of adjunctive N-acetylcysteine in major depressive disorder: A double-blind, randomized, placebo-controlled trial. Journal of Clinical Psychiatry. 2014;75(6):628-36.

18.	Lopresti AL, Maes M, Maker GL, Hood SD, Drummond PD. Curcumin for the treatment of major depression: A randomised, double-blind, placebo controlled study. Journal of Affective Disorders. 2014;167:368-75.

19.	Lôo H, Poirier MF, Ollat H, Elatki S. Effects of sulbutiamine (Arcalion 200) on psycho-behavioral inhibition in major depressive episodes. 2000;26(2):70‐5

C3 - PUBMED 10858919,EMBASE 30337124.

20.	Talaei A, Noori R, Rezaei Ardani A, Mohammadpour AH, Azimipoor Z, Afzaljavan F. The Effect of Adding Curcumin to Sertraline in the Treatment of Severe Major Depressive Disorder: A Randomized, Double-Blind Clinical Trial. Clinical Neuropharmacology. 2023;46(4):135-9.

21.	Cheng YC, Chen WY, Lin C, Lee SH, Chiu CC, Kuo PH. The N-3 polyunsaturated fatty acids supplementation to prevent depression recurrence in patients with late-life depression: A 52-week double-blind, placebo-controlled trial. Journal of Affective Disorders. 2025;369:8-15.

22.	Sarris J, Murphy J, Stough C, Mischoulon D, Bousman C, MacDonald P, et al. S-Adenosylmethionine (SAMe) monotherapy for depression: an 8-week double-blind, randomised, controlled trial. 2020;237(1):209‐18

C3 - PUBMED 31712971,EMBASE 2003633556.

23.	Bidzan-Wiącek M, Tomczyk M, Błażek M, Mika A, Antosiewicz J. No Effects of Omega-3 Supplementation on Kynurenine Pathway, Inflammation, Depressive Symptoms, and Stress Response in Males: A Placebo-Controlled Trial. Nutrients. 2024;16(21 C7 - 3744).

24.	Sarris J, Byrne GJ, Stough C, Bousman C, Mischoulon D, Murphy J, et al. Nutraceuticals for major depressive disorder- more is not merrier: An 8-week double-blind, randomised, controlled trial. Journal of Affective Disorders. 2019;245:1007-15.

25.	Yang R, Wang L, Jin K, Cao S, Wu C, Guo J, et al. Omega-3 Polyunsaturated Fatty Acids Supplementation Alleviate Anxiety Rather Than Depressive Symptoms Among First-Diagnosed, Drug-Naïve Major Depressive Disorder Patients: A Randomized Clinical Trial. Frontiers in Nutrition. 2022;9 C7 - 876152.

26.	Sempach L, Schaub A-C, Brühl AB, Sterzer P, Lang UE, Schmidt A. Adjunctive d-serine treatment for major depressive disorder: A randomized clinical trial. Journal of Affective Disorders. 2025:119504.

**e-table 10:** Remission node 1 subnetworks.

| **Author(s), year** | **Treatment** | **N** | **Subnetwork** |
| --- | --- | --- | --- |
| (Mischoulon, Price et al. 2014)^529^ | SAMe | 64 | 1 |
| (Mischoulon, Price et al. 2014)^529^ | ESC | 65 | 1 |
| (Mischoulon, Price et al. 2014)^529^ | PLB | 60 | 1 |
| (Su, Huang et al. 2008)^387^ | EPA+DHA | 13 | 1 |
| (Su, Huang et al. 2008)^387^ | PLB | 11 | 1 |
| (Kasper, Volz et al. 2024)^597^ | SIL | 154 | 1 |
| (Kasper, Volz et al. 2024)^597^ | SER | 151 | 1 |
| (Kasper, Volz et al. 2024)^597^ | PLB | 141 | 1 |
| (Wu, Yang et al. 2024)^577^ | EPA+DHA | 30 | 1 |
| (Wu, Yang et al. 2024)^577^ | PLB | 30 | 1 |
| (Papakostas, Shelton et al. 2012)^539^ | LMF | 36 | 1 |
| (Papakostas, Shelton et al. 2012)^539^ | PLB | 112 | 1 |
| (Papakostas, Shelton et al. 2012)^539^ | LMF | 19 | 1 |
| (Papakostas, Shelton et al. 2012)^539^ | PLB | 56 | 1 |
| (Sarris, Murphy et al. 2020)^552^ | SAMe | 22 | 1 |
| (Sarris, Murphy et al. 2020)^552^ | PLB | 19 | 1 |
| (Mozaffari-Khosravi, Nabizade et al. 2013)^272^ | EPA | 21 | 1 |
| (Mozaffari-Khosravi, Nabizade et al. 2013)^272^ | PLB | 21 | 1 |
| (Ning, Zhou et al. 2022)^532^ | ZPG | 89 | 1 |
| (Ning, Zhou et al. 2022)^532^ | PLB | 87 | 1 |
| (Sarris, Fava et al. 2012)^606^ | Li-160 | 35 | 1 |
| (Sarris, Fava et al. 2012)^606^ | SER | 49 | 1 |
| (Sarris, Fava et al. 2012)^606^ | PLB | 40 | 1 |
| (Kasper, Anghelescu et al. 2006)^510^ | WS5570 | 243 | 1 |
| (Kasper, Anghelescu et al. 2006)^510^ | PLB | 81 | 1 |
| (Gelenberg, Shelton et al. 2004)^493^ | LI-160 | 41 | 1 |
| (Gelenberg, Shelton et al. 2004)^493^ | PLB | 54 | 1 |
| (Schrader, Meier et al. 1998)^554^ | ZE117 | 80 | 1 |
| (Schrader, Meier et al. 1998)^554^ | PLB | 79 | 1 |
| (Bjerkenstedt, Edman et al. 2005)^472^ | LI-160 | 54 | 1 |
| (Bjerkenstedt, Edman et al. 2005)^472^ | FLUO | 54 | 1 |
| (Bjerkenstedt, Edman et al. 2005)^472^ | PLB | 55 | 1 |
| (Kashani, Eslatmanesh et al. 2017)^509^ | SAFF | 32 | 1 |
| (Kashani, Eslatmanesh et al. 2017)^509^ | FLUO | 32 | 1 |
| (Ahmadpanah, Ramezanshams et al. 2019)^456^ | SAFF | 25 | 1 |
| (Ahmadpanah, Ramezanshams et al. 2019)^456^ | SER | 25 | 1 |
| (Ghajar, Neishabouri et al. 2017)^495^ | SAFF | 30 | 1 |
| (Ghajar, Neishabouri et al. 2017)^495^ | CTP | 30 | 1 |
| (Li, Li et al. 2022)^520^ | SHU | 194 | 1 |
| (Li, Li et al. 2022)^520^ | PLB | 191 | 1 |
| (2016)^600^ | AYA | 14 | 1 |
| (2016)^600^ | PLB | 15 | 1 |
| (Wu, Zhu et al. 2015)^601^ | YEU | 10 | 1 |
| (Wu, Zhu et al. 2015)^601^ | FLUO | 8 | 1 |
| (Sanmukhani, Satodia et al. 2014)^549^ | CUR | 16 | 1 |
| (Sanmukhani, Satodia et al. 2014)^549^ | FLUO | 17 | 1 |
| (Sanmukhani, Satodia et al. 2014)^549^ | CUR+FLUO | 18 | 1 |
| (Sakurai, L. Carpenter et al. 2020)^548^ | SAMe | 16 | 1 |
| (Sakurai, L. Carpenter et al. 2020)^548^ | ESC | 23 | 1 |
| (Sakurai, L. Carpenter et al. 2020)^548^ | PLB | 26 | 1 |
| (Davidson, Gadde et al. 2002)^482^ | CHRO | 10 | 1 |
| (Davidson, Gadde et al. 2002)^482^ | PLB | 5 | 1 |
| (Shelton, Keller et al. 2001)^562^ | SJW | 98 | 1 |
| (Shelton, Keller et al. 2001)^562^ | PLB | 102 | 1 |
| (Shahmansouri, Farokhnia et al. 2014)^558^ | SAFF | 20 | 1 |
| (Shahmansouri, Farokhnia et al. 2014)^558^ | FLUO | 20 | 1 |
| (Fava, Alpert et al. 2005)^488^ | LI-160 | 45 | 1 |
| (Fava, Alpert et al. 2005)^488^ | FLUO | 47 | 1 |
| (Fava, Alpert et al. 2005)^488^ | PLB | 43 | 1 |
| (Mischoulon, Best-Popescu et al. 2008)^254^ | EPA | 16 | 1 |
| (Mischoulon, Best-Popescu et al. 2008)^254^ | PLB | 19 | 1 |
| (Kashani, Esalatmanesh et al. 2018)^508^ | SAFF | 28 | 1 |
| (Kashani, Esalatmanesh et al. 2018)^508^ | PLB | 28 | 1 |
| (Lecrubier, Clerc et al. 2002)^211^ | WS5570 | 186 | 1 |
| (Lecrubier, Clerc et al. 2002)^211^ | PLB | 189 | 1 |
| (Akhondzadeh Basti, Moshiri et al. 2007)^457^ | SAFF | 20 | 1 |
| (Akhondzadeh Basti, Moshiri et al. 2007)^457^ | FLUO | 20 | 1 |
| (Carney, Steinmeyer et al. 2016)^476^ | SER+EPA+DHA | 62 | 2 |
| (Carney, Steinmeyer et al. 2016)^476^ | SER+PLB | 60 | 2 |
| (Shamabadi, Fattollahzadeh-Noor et al. 2023)^560^ | SER+THE | 25 | 2 |
| (Shamabadi, Fattollahzadeh-Noor et al. 2023)^560^ | SER+PLB | 25 | 2 |
| (Ghaffari, Ghobadi et al. 2020)^494^ | SER+PLB | 19 | 2 |
| (Ghaffari, Ghobadi et al. 2020)^494^ | SER+CIN | 23 | 2 |
| (Carney, Freedland et al. 2019)^475^ | SER+EPA | 71 | 2 |
| (Carney, Freedland et al. 2019)^475^ | SER+PLB | 73 | 2 |
| (Papakostas, Mischoulon et al. 2010)^538^ | SAMe+TAU | 39 | 3 |
| (Papakostas, Mischoulon et al. 2010)^538^ | PLB+TAU | 34 | 3 |
| (Sarris, Byrne et al. 2018)^550^ | SAMe+TAU | 37 | 3 |
| (Sarris, Byrne et al. 2018)^550^ | PLB+TAU | 39 | 3 |
| (Padhan, Mohapatra et al. 2024)^535^ | SAR+TAU | 30 | 3 |
| (Padhan, Mohapatra et al. 2024)^535^ | PLB+TAU | 30 | 3 |
| (Ghaleiha, Davari et al. 2016)^582^ | THI+FLUO | 25 | 4 |
| (Ghaleiha, Davari et al. 2016)^582^ | PLB+FLUO | 26 | 4 |
| (Ryszewska-Pokraśniewicz, Mach et al. 2018)^583^ | MG+FLUO | 17 | 4 |
| (Ryszewska-Pokraśniewicz, Mach et al. 2018)^583^ | PLB+FLUO | 15 | 4 |
| (Almeida, Ford et al. 2014)^599^ | FOL+vitB12+vitB6+CTP | 62 | 5 |
| (Almeida, Ford et al. 2014)^599^ | CTP+PLB | 66 | 5 |
| (Firoozeei, Barekatain et al. 2020)^579^ | LAV+PLB | 25 | 5 |
| (Firoozeei, Barekatain et al. 2020)^579^ | CTP+PLB | 25 | 5 |
| (Roohi-Azizi, Arabzadeh et al. 2017)^586^ | CIT+CTP | 25 | 6 |
| (Roohi-Azizi, Arabzadeh et al. 2017)^586^ | PLB+CTP | 25 | 6 |
| (Sahraian, Ghanizadeh et al. 2015)^585^ | vitC+CTP | 21 | 6 |
| (Sahraian, Ghanizadeh et al. 2015)^585^ | PLB+CTP | 22 | 6 |
| (Siwek, Dudek et al. 2010)^598^ | ZNC+IMI | 27 | 7 |
| (Siwek, Dudek et al. 2010)^598^ | IMI+PLB | 25 | 7 |
| (Coppen and Bailey 2000)^584^ | FOL+FLUO | 51 | 8 |
| (Coppen and Bailey 2000)^584^ | FLUO+PLB | 58 | 8 |
| (Lyoo, Yoon et al. 2012)^593^ | ESC+CREA | 25 | 9 |
| (Lyoo, Yoon et al. 2012)^593^ | ESC+PLB | 27 | 9 |
| (Yu, Pei et al. 2015)^594^ | CUR+ESC | 50 | 10 |
| (Yu, Pei et al. 2015)^594^ | PLB+ESC | 50 | 10 |
| (Yang, Wang et al. 2022)^595^ | EPA+DHA+VEN | 21 | 11 |
| (Yang, Wang et al. 2022)^595^ | PLB+VEN | 27 | 11 |

**Reference for e-table 10**

1.	Carney RM, Freedland KE, Rubin EH, Rich MW, Steinmeyer BC, Harris WS. A randomized placebo-controlled trial of omega-3 and sertraline in depressed patients with or at risk for coronary heart disease. Journal of Clinical Psychiatry. 2019;80(4 C7 - 19m12742).

2.	Lyoo IK, Yoon S, Kim TS, Hwang J, Kim JE, Won W, et al. A randomized, double-blind placebo-controlled trial of oral creatine monohydrate augmentation for enhanced response to a selective serotonin reuptake inhibitor in women with major depressive disorder. American Journal of Psychiatry. 2012;169(9):937-45.

3.	Ghaffari S, Ghobadi A, Jamshidi AH, Mortazavi SH, Naderi S, Aqamolaei A, et al. Cinnamomum tamala as an adjuvant therapy in the treatment of major depressive disorder: A double-blind, randomized, placebo-controlled clinical trial with placebo control. Advances in Integrative Medicine. 2020;7(3):141-7.

4.	Akhondzadeh Basti A, Moshiri E, Noorbala AA, Jamshidi AH, Abbasi SH, Akhondzadeh S. Comparison of petal of Crocus sativus L. and fluoxetine in the treatment of depressed outpatients: A pilot double-blind randomized trial. Progress in Neuro-Psychopharmacology and Biological Psychiatry. 2007;31(2):439-42.

5.	Lecrubier Y, Clerc G, Didi R, Kieser M. Efficacy of St. John's wort extract WS 5570 in major depression: A double-blind, placebo-controlled trial. American Journal of Psychiatry. 2002;159(8):1361-6.

6.	Kashani L, Esalatmanesh S, Eftekhari F, Salimi S, Foroughifar T, Etesam F, et al. Efficacy of Crocus sativus (saffron) in treatment of major depressive disorder associated with post-menopausal hot flashes: a double-blind, randomized, placebo-controlled trial. Arch Gynecol Obstet. 2018;297(3):717-24.

7.	Shamabadi A, Fattollahzadeh-Noor S, Fallahpour B, A. Basti F, Khodaei Ardakani MR, Akhondzadeh S. L-Theanine adjunct to risperidone in the treatment of chronic schizophrenia inpatients: a randomized, double-blind, placebo-controlled clinical trial. Psychopharmacology. 2023;240(12):2631-40.

8.	Firoozeei TS, Barekatain M, Karimi M, Zargaran A, Akhondzadeh S, Rezaeizadeh H. Lavender and dodder combined herbal syrup versus citalopram in major depressive disorder with anxious distress: A double-blind randomized trial. Journal of Integrative Medicine. 2020;18(5):409-15.

9.	Sahraian A, Ghanizadeh A, Kazemeini F. Vitamin C as an adjuvant for treating major depressive disorder and suicidal behavior, a randomized placebo-controlled clinical trial. Trials. 2015;16(1 C7 - 94).

10.	Mischoulon D, Best-Popescu C, Laposata M, Merens W, Murakami JL, Wu SL, et al. A double-blind dose-finding pilot study of docosahexaenoic acid (DHA) for major depressive disorder. European Neuropsychopharmacology. 2008;18(9):639-45.

11.	Fava M, Alpert J, Nierenberg AA, Mischoulon D, Otto MW, Zajecka J, et al. A double-blind, randomized trial of St John's wort, fluoxetine, and placebo in major depressive disorder. Journal of Clinical Psychopharmacology. 2005;25(5):441-7.

12.	Shahmansouri N, Farokhnia M, Abbasi SH, Kassaian SE, Noorbala Tafti AA, Gougol A, et al. A randomized, double-blind, clinical trial comparing the efficacy and safety of Crocus sativus L. with fluoxetine for improving mild to moderate depression in post percutaneous coronary intervention patients. 2014;155:216‐22

C3 - PUBMED 24289892,EMBASE 52891114.

13.	Shelton RC, Keller MB, Gelenberg A, Dunner DL, Hirschfeld R, Thase ME, et al. Effectiveness of St John's wort in major depression: A randomized controlled trial. JAMA. 2001;285(15):1978-86.

14.	Davidson JR, Gadde KM, Fairbank JA, Krishnan KRR, Califf RM, Binanay C, et al. Effect of Hypericum perforatum (St John's wort) in major depressive disorder: a randomized controlled trial. 2002;287(14):1807‐14

C3 - PUBMED 11939866,EMBASE 34303625.

15.	Ryszewska-Pokraśniewicz B, Mach A, Skalski M, Januszko P, Wawrzyniak ZM, Poleszak E, et al. Effects of magnesium supplementation on unipolar depression: A placebo-controlled study and review of the importance of dosing and magnesium status in the therapeutic response. Nutrients. 2018;10(8 C7 - 1014).

16.	Padhan M, Mohapatra D, Mishra BR, Maiti R, Jena M. Efficacy and safety of add-on sarcosine in patients with major depressive disorder: A randomized controlled trial. Journal of Psychiatric Research. 2024;178:298-304.

17.	Sakurai H, L. Carpenter L, R. Tyrka A, Price LH, I. Papakostas G, Dording CM, et al. Dose increase of S-Adenosyl-Methionine and escitalopram in a randomized clinical trial for major depressive disorder. Journal of Affective Disorders. 2020;262:118-25.

18.	Sanmukhani J, Satodia V, Trivedi J, Patel T, Tiwari D, Panchal B, et al. Efficacy and safety of curcumin in major depressive disorder: a randomized controlled trial. Phytother Res. 2014;28(4):579-85.

19.	Wu R, Zhu D, Xia Y, Wang H, Tao W, Xue W, et al. A role of Yueju in fast-onset antidepressant action on major depressive disorder and serum BDNF expression: A randomly double-blind, fluoxetine-adjunct, placebo-controlled, pilot clinical study. Neuropsychiatric Disease and Treatment. 2015;11:2013-21.

20.	Sarris J, Byrne GJ, Bousman C, Stough C, Murphy J, MacDonald P, et al. Adjunctive S-adenosylmethionine (SAMe) in treating non-remittent major depressive disorder: An 8-week double-blind, randomized, controlled trial. European Neuropsychopharmacology. 2018;28(10):1126-36.

21.	Ghaleiha A, Davari H, Jahangard L, Haghighi M, Ahmadpanah M, Seifrabie MA, et al. Adjuvant thiamine improved standard treatment in patients with major depressive disorder: results from a randomized, double-blind, and placebo-controlled clinical trial. European Archives of Psychiatry and Clinical Neuroscience. 2016;266(8):695-702.

22.	Antidepressant Effects of Ayahuasca: a Randomized Placebo Controlled Trial in Treatment Resistant Depression. clinicaltrialsgov2016.

23.	Yu JJ, Pei LB, Zhang Y, Wen ZY, Yang JL. Chronic Supplementation of Curcumin Enhances the Efficacy of Antidepressants in Major Depressive Disorder: A Randomized, Double-Blind, Placebo-Controlled Pilot Study. Journal of Clinical Psychopharmacology. 2015;35(4):406-10.

24.	Roohi-Azizi M, Arabzadeh S, Amidfar M, Salimi S, Zarindast MR, Talaei A, et al. Citicoline combination therapy for major depressive disorder: A randomized, double-blind, placebo-controlled trial. Clinical Neuropharmacology. 2017;40(1):1-5.

25.	Carney RM, Steinmeyer BC, Freedland KE, Rubin EH, Rich MW, Harris WS. Baseline blood levels of omega-3 and depression remission: A secondary analysis of data from a placebo-controlled trial of omega-3 supplements. Journal of Clinical Psychiatry. 2016;77(2):e138-e43.

26.	Almeida OP, Ford AH, Hirani V, Singh V, VanBockxmeer FM, McCaul K, et al. B vitamins to enhance treatment response to antidepressants in middle-aged and older adults: Results from the B-VITAGE randomised, double-blind, placebo-controlled trial. British Journal of Psychiatry. 2014;205(6):450-7.

27.	Li Y, Li L, Guo R, Yu H, Wang X, Wang B, et al. Clinical efficacy of Shugan granule in the treatment of mixed anxiety-depressive disorder: a multicenter, randomized, double-blind, placebo-controlled trial. 2022;290:115032

C3 - PUBMED 35085742,EMBASE 637131287.

28.	Ghajar A, Neishabouri SM, Velayati N, Jahangard L, Matinnia N, Haghighi M, et al. Crocus sativus L versus Citalopram in the Treatment of Major Depressive Disorder with Anxious Distress: A Double-Blind, Controlled Clinical Trial. Pharmacopsychiatry. 2017;50(4):152-60.

29.	Ahmadpanah M, Ramezanshams F, Ghaleiha A, Akhondzadeh S, Sadeghi Bahmani D, Brand S. Crocus Sativus L. (saffron) versus sertraline on symptoms of depression among older people with major depressive disorders–a double-blind, randomized intervention study. Psychiatry Research. 2019;282.

30.	Kashani L, Eslatmanesh S, Saedi N, Niroomand N, Ebrahimi M, Hosseinian M, et al. Comparison of Saffron versus Fluoxetine in Treatment of Mild to Moderate Postpartum Depression: A Double-Blind, Randomized Clinical Trial. Pharmacopsychiatry. 2017;50(2):64-8.

31.	Bjerkenstedt L, Edman GV, Alken RG, Mannel M. Hypericum extract LI 160 and fluoxetine in mild to moderate depression: A randomized, placebo-controlled multi-center study in outpatients. European Archives of Psychiatry and Clinical Neuroscience. 2005;255(1):40-7.

32.	Schrader E, Meier B, Brattström A. Hypericum treatment of mild-moderate depression in a placebo-controlled study. A prospective, double-blind, randomized, placebo-controlled, multicentre study. Human Psychopharmacology. 1998;13(3):163-9.

33.	Coppen A, Bailey J. Enhancement of the antidepressant action of fluoxetine by folic acid: A randomised, placebo controlled trial. Journal of Affective Disorders. 2000;60(2):121-30.

34.	Papakostas GI, Mischoulon D, Shyu I, Alpert JE, Fava M. S-adenosyl methionine (SAMe) augmentation of serotonin reuptake inhibitors for antidepressant nonresponders with major depressive disorder: a double-blind, randomized clinical trial. 2010;167(8):942‐8

C3 - PUBMED 20595412.

35.	Gelenberg AJ, Shelton RC, Crits-Christoph P, Keller MB, Dunner DL, Hirschfeld RM, et al. The effectiveness of St. John's Wort in major depressive disorder: a naturalistic phase 2 follow-up in which nonresponders were provided alternate medication. 2004;65(8):1114‐9

C3 - PUBMED 15323598.

36.	Kasper S, Anghelescu IG, Szegedi A, Dienel A, Kieser M. Superior efficacy of St John's wort extract WS 5570 compared to placebo in patients with major depression: a randomized, double-blind, placebo-controlled, multi-center trial. 2006;4:14

C3 - PUBMED 16796730,EMBASE 44227549.

37.	Sarris J, Fava M, Schweitzer I, Mischoulon D. St Johns Wort (Hypericum perforatum) versus sertraline and placebo in major depressive disorder: Continuation data from a 26-week RCT. Pharmacopsychiatry. 2012;45(7):275-8.

38.	Ning H, Zhou H, Ren J, Zhou G, Yang N, Wang Z, et al. Zishen pingchan granules combined with pramipexole in the improvement of depressive symptoms in Parkinson's disease: a prospective, multicenter, randomized, double-blind, controlled clinical study. J Transl Med. 2022;20(1):357.

39.	Mozaffari-Khosravi H, Nabizade L, Yassini-Ardakani SM, Hadinedoushan H, Barzegar K. The effect of 2 different single injections of high dose of vitamin D on improving the depression in depressed patients with vitamin D deficiency: A randomized clinical trial. Journal of Clinical Psychopharmacology. 2013;33(3):378-85.

40.	Sarris J, Murphy J, Stough C, Mischoulon D, Bousman C, MacDonald P, et al. S-Adenosylmethionine (SAMe) monotherapy for depression: an 8-week double-blind, randomised, controlled trial. 2020;237(1):209‐18

C3 - PUBMED 31712971,EMBASE 2003633556.

41.	Siwek M, Dudek D, Schlegel-Zawadzka M, Morawska A, Piekoszewski W, Opoka W, et al. Serum zinc level in depressed patients during zinc supplementation of imipramine treatment. Journal of Affective Disorders. 2010;126(3):447-52.

42.	Papakostas GI, Shelton RC, Zajecka JM, Etemad B, Rickels K, Clain A, et al. L-methylfolate as adjunctive therapy for SSRI-resistant major depression: results of two randomized, double-blind, parallel-sequential trials. 2012;169(12):1267‐74

C3 - PUBMED 23212058.

43.	Wu SK, Yang KJ, Liu WC, Malau IA, Zailani H, Chang CH, et al. The Efficacy of Omega-3 Fatty Acids as the Monotherapy for Depression: A Randomized, Double-Blind, Placebo-Controlled Pilot Study. Nutrients. 2024;16(21).

44.	Kasper S, Volz HP, Möller HJ, Schläfke S, Klement S, Anghelescu IG, et al. Lavender oil preparation Silexan is effective in mild-to-moderate major depression: a randomized, placebo- and reference-controlled trial. European Archives of Psychiatry and Clinical Neuroscience. 2024.

45.	Su KP, Huang SY, Chiu TH, Huang KC, Huang CL, Chang HC, et al. Omega-3 fatty acids for major depressive disorder during pregnancy: Results from a randomized, double-blind, placebo-controlled trial. Journal of Clinical Psychiatry. 2008;69(4):644-51.

46.	Yang R, Wang L, Jin K, Cao S, Wu C, Guo J, et al. Omega-3 Polyunsaturated Fatty Acids Supplementation Alleviate Anxiety Rather Than Depressive Symptoms Among First-Diagnosed, Drug-Naïve Major Depressive Disorder Patients: A Randomized Clinical Trial. Frontiers in Nutrition. 2022;9 C7 - 876152.

47.	Mischoulon D, Price LH, Carpenter LL, Tyrka AR, Papakostas GI, Baer L, et al. A double-blind, randomized, placebo-controlled clinical trial of S-adenosyl-L-methionine (SAMe) versus escitalopram in major depressive disorder. J Clin Psychiatry. 2014;75(4):370-6.

**e-table 11**: Remission node 2 subnetworks.

| **Author(s), year** | **Treatment** | **N** | **Subnetwork** |
| --- | --- | --- | --- |
| ^475^ | SSRI+LCPUFA | 71 | 1 |
| ^475^ | SSRI | 73 | 1 |
| ^593^ | SSRI+aminoacids | 25 | 1 |
| ^593^ | SSRI | 27 | 1 |
| ^494^ | SSRI | 19 | 1 |
| ^494^ | SSRI+Lauraceae | 23 | 1 |
| ^457^ | Iridaceae | 20 | 1 |
| ^457^ | SSRI | 20 | 1 |
| ^211^ | HYP | 186 | 1 |
| ^211^ | PLB | 189 | 1 |
| ^508^ | Iridaceae | 28 | 1 |
| ^508^ | PLB | 28 | 1 |
| ^560^ | SSRI+Aminoacids | 25 | 1 |
| ^560^ | SSRI+PLB | 25 | 1 |
| ^585^ | vitamin +SSRI | 21 | 1 |
| ^585^ | PLB+SSRI | 22 | 1 |
| ^254^ | LCPUFA | 16 | 1 |
| ^254^ | PLB | 19 | 1 |
| ^488^ | PLB | 43 | 1 |
| ^488^ | HYP | 45 | 1 |
| ^488^ | SSRI | 47 | 1 |
| ^558^ | Iridaceae | 20 | 1 |
| ^558^ | SSRI | 20 | 1 |
| ^562^ | HYP | 98 | 1 |
| ^562^ | PLB | 102 | 1 |
| ^482^ | PLB | 5 | 1 |
| ^482^ | Minerals | 10 | 1 |
| ^583^ | PLB+SSRI | 15 | 1 |
| ^583^ | Minerals+SSRI | 17 | 1 |
| ^535^ | Amminoacids+SSRI | 30 | 1 |
| ^535^ | SSRI+PLB | 30 | 1 |
| ^548^ | Coenzyme | 16 | 1 |
| ^548^ | SSRI | 23 | 1 |
| ^548^ | PLB | 26 | 1 |
| ^549^ | Zingiberaceae | 16 | 1 |
| ^549^ | SSRI | 17 | 1 |
| ^549^ | Zingiberaceae+SSRI | 18 | 1 |
| ^601^ | SSRI | 8 | 1 |
| ^601^ | Herbal compound | 10 | 1 |
| ^582^ | PLB+SSRI | 26 | 1 |
| ^582^ | vitamin+SSRI | 25 | 1 |
| ^600^ | Herbal compound | 14 | 1 |
| ^600^ | PLB | 15 | 1 |
| ^594^ | Zingiberaceae+SSRI | 50 | 1 |
| ^594^ | PLB+SSRI | 50 | 1 |
| ^579^ | Nucleotide+SSRI | 25 | 1 |
| ^579^ | PLB+SSRI | 25 | 1 |
| ^476^ | SSRI+PLB | 60 | 1 |
| ^476^ | SSRI+LCPUFA | 62 | 1 |
| ^599^ | vitamin+SSRI | 62 | 1 |
| ^599^ | SSRI+PLB | 66 | 1 |
| ^520^ | PLB | 191 | 1 |
| ^520^ | Herbal compound | 194 | 1 |
| ^495^ | Iridaceae | 30 | 1 |
| ^495^ | SSRI | 30 | 1 |
| ^586^ | Iridaceae | 25 | 1 |
| ^586^ | SSRI | 25 | 1 |
| ^509^ | Iridaceae | 32 | 1 |
| ^509^ | SSRI | 32 | 1 |
| ^472^ | HYP | 54 | 1 |
| ^472^ | SSRI | 54 | 1 |
| ^472^ | PLB | 55 | 1 |
| ^554^ | PLB | 79 | 1 |
| ^554^ | HYP | 80 | 1 |
| ^584^ | vitamin+SSRI | 51 | 1 |
| ^584^ | SSRI+PLB | 58 | 1 |
| ^538^ | PLB+SSRI | 34 | 1 |
| ^550^ | Coenzyme+SSRI | 39 | 1 |
| ^493^ | HYP | 41 | 1 |
| ^493^ | PLB | 54 | 1 |
| ^510^ | PLB | 81 | 1 |
| ^510^ | HYP | 243 | 1 |
| ^606^ | HYP | 35 | 1 |
| ^606^ | PLB | 40 | 1 |
| ^606^ | SSRI | 49 | 1 |
| ^532^ | PLB | 87 | 1 |
| ^532^ | Herbal compound | 89 | 1 |
| ^272^ | LCPUFA | 21 | 1 |
| ^272^ | PLB | 21 | 1 |
| ^552^ | PLB | 19 | 1 |
| ^552^ | Coenzyme | 22 | 1 |
| ^539^ | vitamins | 36 | 1 |
| ^539^ | PLB | 112 | 1 |
| ^539^ | vitamins | 19 | 1 |
| ^539^ | PLB | 56 | 1 |
| ^577^ | LCPUFA | 30 | 1 |
| ^577^ | PLB | 30 | 1 |
| ^597^ | PLB | 141 | 1 |
| ^597^ | SSRI | 151 | 1 |
| ^597^ | Lamiaceae | 154 | 1 |
| ^387^ | PLB | 11 | 1 |
| ^387^ | LCPUFA | 13 | 1 |
| ^595^ | LCPUFA | 21 | 1 |
| ^595^ | PLB | 27 | 1 |
| ^529^ | PLB | 60 | 1 |
| ^529^ | Coenzyme | 64 | 1 |
| ^529^ | SSRI | 65 | 1 |
| ^538^ | Mineral+TCA | 27 | 2 |
| ^538^ | TCA+PLB | 25 | 2 |
| ^456^ | PLB+Antidepressant | 39 | 3 |
| ^456^ | Coenzyme+Antidepressant | 37 | 3 |
| ^598^ | Lamiaceae + PLB | 25 | 4 |
| ^598^ | SSRI + PLB | 25 | 4 |

**Reference for e-table 11**

1.	Carney RM, Freedland KE, Rubin EH, Rich MW, Steinmeyer BC, Harris WS. A randomized placebo-controlled trial of omega-3 and sertraline in depressed patients with or at risk for coronary heart disease. Journal of Clinical Psychiatry. 2019;80(4 C7 - 19m12742).

2.	Lyoo IK, Yoon S, Kim TS, Hwang J, Kim JE, Won W, et al. A randomized, double-blind placebo-controlled trial of oral creatine monohydrate augmentation for enhanced response to a selective serotonin reuptake inhibitor in women with major depressive disorder. American Journal of Psychiatry. 2012;169(9):937-45.

3.	Ghaffari S, Ghobadi A, Jamshidi AH, Mortazavi SH, Naderi S, Aqamolaei A, et al. Cinnamomum tamala as an adjuvant therapy in the treatment of major depressive disorder: A double-blind, randomized, placebo-controlled clinical trial with placebo control. Advances in Integrative Medicine. 2020;7(3):141-7.

4.	Akhondzadeh Basti A, Moshiri E, Noorbala AA, Jamshidi AH, Abbasi SH, Akhondzadeh S. Comparison of petal of Crocus sativus L. and fluoxetine in the treatment of depressed outpatients: A pilot double-blind randomized trial. Progress in Neuro-Psychopharmacology and Biological Psychiatry. 2007;31(2):439-42.

5.	Lecrubier Y, Clerc G, Didi R, Kieser M. Efficacy of St. John's wort extract WS 5570 in major depression: A double-blind, placebo-controlled trial. American Journal of Psychiatry. 2002;159(8):1361-6.

6.	Kashani L, Esalatmanesh S, Eftekhari F, Salimi S, Foroughifar T, Etesam F, et al. Efficacy of Crocus sativus (saffron) in treatment of major depressive disorder associated with post-menopausal hot flashes: a double-blind, randomized, placebo-controlled trial. Arch Gynecol Obstet. 2018;297(3):717-24.

7.	Shamabadi A, Fattollahzadeh-Noor S, Fallahpour B, A. Basti F, Khodaei Ardakani MR, Akhondzadeh S. L-Theanine adjunct to risperidone in the treatment of chronic schizophrenia inpatients: a randomized, double-blind, placebo-controlled clinical trial. Psychopharmacology. 2023;240(12):2631-40.

8.	Sahraian A, Ghanizadeh A, Kazemeini F. Vitamin C as an adjuvant for treating major depressive disorder and suicidal behavior, a randomized placebo-controlled clinical trial. Trials. 2015;16(1 C7 - 94).

9.	Mischoulon D, Best-Popescu C, Laposata M, Merens W, Murakami JL, Wu SL, et al. A double-blind dose-finding pilot study of docosahexaenoic acid (DHA) for major depressive disorder. European Neuropsychopharmacology. 2008;18(9):639-45.

10.	Fava M, Alpert J, Nierenberg AA, Mischoulon D, Otto MW, Zajecka J, et al. A double-blind, randomized trial of St John's wort, fluoxetine, and placebo in major depressive disorder. Journal of Clinical Psychopharmacology. 2005;25(5):441-7.

11.	Shahmansouri N, Farokhnia M, Abbasi SH, Kassaian SE, Noorbala Tafti AA, Gougol A, et al. A randomized, double-blind, clinical trial comparing the efficacy and safety of Crocus sativus L. with fluoxetine for improving mild to moderate depression in post percutaneous coronary intervention patients. 2014;155:216‐22

C3 - PUBMED 24289892,EMBASE 52891114.

12.	Shelton RC, Keller MB, Gelenberg A, Dunner DL, Hirschfeld R, Thase ME, et al. Effectiveness of St John's wort in major depression: A randomized controlled trial. JAMA. 2001;285(15):1978-86.

13.	Davidson JR, Gadde KM, Fairbank JA, Krishnan KRR, Califf RM, Binanay C, et al. Effect of Hypericum perforatum (St John's wort) in major depressive disorder: a randomized controlled trial. 2002;287(14):1807‐14

C3 - PUBMED 11939866,EMBASE 34303625.

14.	Ryszewska-Pokraśniewicz B, Mach A, Skalski M, Januszko P, Wawrzyniak ZM, Poleszak E, et al. Effects of magnesium supplementation on unipolar depression: A placebo-controlled study and review of the importance of dosing and magnesium status in the therapeutic response. Nutrients. 2018;10(8 C7 - 1014).

15.	Padhan M, Mohapatra D, Mishra BR, Maiti R, Jena M. Efficacy and safety of add-on sarcosine in patients with major depressive disorder: A randomized controlled trial. Journal of Psychiatric Research. 2024;178:298-304.

16.	Sakurai H, L. Carpenter L, R. Tyrka A, Price LH, I. Papakostas G, Dording CM, et al. Dose increase of S-Adenosyl-Methionine and escitalopram in a randomized clinical trial for major depressive disorder. Journal of Affective Disorders. 2020;262:118-25.

17.	Sanmukhani J, Satodia V, Trivedi J, Patel T, Tiwari D, Panchal B, et al. Efficacy and safety of curcumin in major depressive disorder: a randomized controlled trial. Phytother Res. 2014;28(4):579-85.

18.	Wu R, Zhu D, Xia Y, Wang H, Tao W, Xue W, et al. A role of Yueju in fast-onset antidepressant action on major depressive disorder and serum BDNF expression: A randomly double-blind, fluoxetine-adjunct, placebo-controlled, pilot clinical study. Neuropsychiatric Disease and Treatment. 2015;11:2013-21.

19.	Ghaleiha A, Davari H, Jahangard L, Haghighi M, Ahmadpanah M, Seifrabie MA, et al. Adjuvant thiamine improved standard treatment in patients with major depressive disorder: results from a randomized, double-blind, and placebo-controlled clinical trial. European Archives of Psychiatry and Clinical Neuroscience. 2016;266(8):695-702.

20.	Antidepressant Effects of Ayahuasca: a Randomized Placebo Controlled Trial in Treatment Resistant Depression. clinicaltrialsgov2016.

21.	Yu JJ, Pei LB, Zhang Y, Wen ZY, Yang JL. Chronic Supplementation of Curcumin Enhances the Efficacy of Antidepressants in Major Depressive Disorder: A Randomized, Double-Blind, Placebo-Controlled Pilot Study. Journal of Clinical Psychopharmacology. 2015;35(4):406-10.

22.	Firoozeei TS, Barekatain M, Karimi M, Zargaran A, Akhondzadeh S, Rezaeizadeh H. Lavender and dodder combined herbal syrup versus citalopram in major depressive disorder with anxious distress: A double-blind randomized trial. Journal of Integrative Medicine. 2020;18(5):409-15.

23.	Carney RM, Steinmeyer BC, Freedland KE, Rubin EH, Rich MW, Harris WS. Baseline blood levels of omega-3 and depression remission: A secondary analysis of data from a placebo-controlled trial of omega-3 supplements. Journal of Clinical Psychiatry. 2016;77(2):e138-e43.

24.	Almeida OP, Ford AH, Hirani V, Singh V, VanBockxmeer FM, McCaul K, et al. B vitamins to enhance treatment response to antidepressants in middle-aged and older adults: Results from the B-VITAGE randomised, double-blind, placebo-controlled trial. British Journal of Psychiatry. 2014;205(6):450-7.

25.	Li Y, Li L, Guo R, Yu H, Wang X, Wang B, et al. Clinical efficacy of Shugan granule in the treatment of mixed anxiety-depressive disorder: a multicenter, randomized, double-blind, placebo-controlled trial. 2022;290:115032

C3 - PUBMED 35085742,EMBASE 637131287.

26.	Ghajar A, Neishabouri SM, Velayati N, Jahangard L, Matinnia N, Haghighi M, et al. Crocus sativus L versus Citalopram in the Treatment of Major Depressive Disorder with Anxious Distress: A Double-Blind, Controlled Clinical Trial. Pharmacopsychiatry. 2017;50(4):152-60.

27.	Roohi-Azizi M, Arabzadeh S, Amidfar M, Salimi S, Zarindast MR, Talaei A, et al. Citicoline combination therapy for major depressive disorder: A randomized, double-blind, placebo-controlled trial. Clinical Neuropharmacology. 2017;40(1):1-5.

28.	Kashani L, Eslatmanesh S, Saedi N, Niroomand N, Ebrahimi M, Hosseinian M, et al. Comparison of Saffron versus Fluoxetine in Treatment of Mild to Moderate Postpartum Depression: A Double-Blind, Randomized Clinical Trial. Pharmacopsychiatry. 2017;50(2):64-8.

29.	Bjerkenstedt L, Edman GV, Alken RG, Mannel M. Hypericum extract LI 160 and fluoxetine in mild to moderate depression: A randomized, placebo-controlled multi-center study in outpatients. European Archives of Psychiatry and Clinical Neuroscience. 2005;255(1):40-7.

30.	Schrader E, Meier B, Brattström A. Hypericum treatment of mild-moderate depression in a placebo-controlled study. A prospective, double-blind, randomized, placebo-controlled, multicentre study. Human Psychopharmacology. 1998;13(3):163-9.

31.	Coppen A, Bailey J. Enhancement of the antidepressant action of fluoxetine by folic acid: A randomised, placebo controlled trial. Journal of Affective Disorders. 2000;60(2):121-30.

32.	Papakostas GI, Mischoulon D, Shyu I, Alpert JE, Fava M. S-adenosyl methionine (SAMe) augmentation of serotonin reuptake inhibitors for antidepressant nonresponders with major depressive disorder: a double-blind, randomized clinical trial. 2010;167(8):942‐8

C3 - PUBMED 20595412.

33.	Sarris J, Byrne GJ, Bousman C, Stough C, Murphy J, MacDonald P, et al. Adjunctive S-adenosylmethionine (SAMe) in treating non-remittent major depressive disorder: An 8-week double-blind, randomized, controlled trial. European Neuropsychopharmacology. 2018;28(10):1126-36.

34.	Gelenberg AJ, Shelton RC, Crits-Christoph P, Keller MB, Dunner DL, Hirschfeld RM, et al. The effectiveness of St. John's Wort in major depressive disorder: a naturalistic phase 2 follow-up in which nonresponders were provided alternate medication. 2004;65(8):1114‐9

C3 - PUBMED 15323598.

35.	Kasper S, Anghelescu IG, Szegedi A, Dienel A, Kieser M. Superior efficacy of St John's wort extract WS 5570 compared to placebo in patients with major depression: a randomized, double-blind, placebo-controlled, multi-center trial. 2006;4:14

C3 - PUBMED 16796730,EMBASE 44227549.

36.	Sarris J, Fava M, Schweitzer I, Mischoulon D. St Johns Wort (Hypericum perforatum) versus sertraline and placebo in major depressive disorder: Continuation data from a 26-week RCT. Pharmacopsychiatry. 2012;45(7):275-8.

37.	Ning H, Zhou H, Ren J, Zhou G, Yang N, Wang Z, et al. Zishen pingchan granules combined with pramipexole in the improvement of depressive symptoms in Parkinson's disease: a prospective, multicenter, randomized, double-blind, controlled clinical study. J Transl Med. 2022;20(1):357.

38.	Mozaffari-Khosravi H, Nabizade L, Yassini-Ardakani SM, Hadinedoushan H, Barzegar K. The effect of 2 different single injections of high dose of vitamin D on improving the depression in depressed patients with vitamin D deficiency: A randomized clinical trial. Journal of Clinical Psychopharmacology. 2013;33(3):378-85.

39.	Sarris J, Murphy J, Stough C, Mischoulon D, Bousman C, MacDonald P, et al. S-Adenosylmethionine (SAMe) monotherapy for depression: an 8-week double-blind, randomised, controlled trial. 2020;237(1):209‐18

C3 - PUBMED 31712971,EMBASE 2003633556.

40.	Papakostas GI, Shelton RC, Zajecka JM, Etemad B, Rickels K, Clain A, et al. L-methylfolate as adjunctive therapy for SSRI-resistant major depression: results of two randomized, double-blind, parallel-sequential trials. 2012;169(12):1267‐74

C3 - PUBMED 23212058.

41.	Wu SK, Yang KJ, Liu WC, Malau IA, Zailani H, Chang CH, et al. The Efficacy of Omega-3 Fatty Acids as the Monotherapy for Depression: A Randomized, Double-Blind, Placebo-Controlled Pilot Study. Nutrients. 2024;16(21).

42.	Kasper S, Volz HP, Möller HJ, Schläfke S, Klement S, Anghelescu IG, et al. Lavender oil preparation Silexan is effective in mild-to-moderate major depression: a randomized, placebo- and reference-controlled trial. European Archives of Psychiatry and Clinical Neuroscience. 2024.

43.	Su KP, Huang SY, Chiu TH, Huang KC, Huang CL, Chang HC, et al. Omega-3 fatty acids for major depressive disorder during pregnancy: Results from a randomized, double-blind, placebo-controlled trial. Journal of Clinical Psychiatry. 2008;69(4):644-51.

44.	Yang R, Wang L, Jin K, Cao S, Wu C, Guo J, et al. Omega-3 Polyunsaturated Fatty Acids Supplementation Alleviate Anxiety Rather Than Depressive Symptoms Among First-Diagnosed, Drug-Naïve Major Depressive Disorder Patients: A Randomized Clinical Trial. Frontiers in Nutrition. 2022;9 C7 - 876152.

45.	Mischoulon D, Price LH, Carpenter LL, Tyrka AR, Papakostas GI, Baer L, et al. A double-blind, randomized, placebo-controlled clinical trial of S-adenosyl-L-methionine (SAMe) versus escitalopram in major depressive disorder. J Clin Psychiatry. 2014;75(4):370-6.

46.	Ahmadpanah M, Ramezanshams F, Ghaleiha A, Akhondzadeh S, Sadeghi Bahmani D, Brand S. Crocus Sativus L. (saffron) versus sertraline on symptoms of depression among older people with major depressive disorders–a double-blind, randomized intervention study. Psychiatry Research. 2019;282.

47.	Siwek M, Dudek D, Schlegel-Zawadzka M, Morawska A, Piekoszewski W, Opoka W, et al. Serum zinc level in depressed patients during zinc supplementation of imipramine treatment. Journal of Affective Disorders. 2010;126(3):447-52.

**e-table 12**: Tolerability node 2 subnetworks.

| **Author(s), year** | **Treatment** | **n** | **Subnetwork** |
| --- | --- | --- | --- |
| (Mazidi, Shemshian et al. 2016)^527^ | Iridaceae | 30 | 1 |
| (Mazidi, Shemshian et al. 2016)^527^ | PLB | 30 | 1 |
| (Araj-Khodaei, Noorbala et al. 2020)^464^ | Lamiaceae | 33 | 1 |
| (Araj-Khodaei, Noorbala et al. 2020)^464^ | SSRI | 17 | 1 |
| (Akhondzadeh, Mostafavi et al. 2020)^459^ | Iridaceae | 36 | 1 |
| (Akhondzadeh, Mostafavi et al. 2020)^459^ | PLB | 37 | 1 |
| (Carney, Freedland et al. 2019)^475^ | SSRI+LCPUFA | 71 | 1 |
| (Carney, Freedland et al. 2019)^475^ | SSRI+PLB | 73 | 1 |
| (Lyoo, Yoon et al. 2012)^593^ | SSRI+Aminoacids | 25 | 1 |
| (Lyoo, Yoon et al. 2012)^593^ | SSRI+PLB | 27 | 1 |
| (Sherwood Brown and Gabrielson 2012)^563^ | Nucleotide | 17 | 1 |
| (Sherwood Brown and Gabrielson 2012)^563^ | PLB | 14 | 1 |
| (Kumar, Menon et al. 2022)^517^ | SSRI+Vitamins | 31 | 1 |
| (Kumar, Menon et al. 2022)^517^ | SSRI+PLB | 28 | 1 |
| (Hu, Chen et al. 2021)^502^ | Herbal compound + PLB | 63 | 1 |
| (Hu, Chen et al. 2021)^502^ | SSRI+PLB | 66 | 1 |
| (Targum, Cameron et al. 2018)^569^ | Coenzyme+Antidepressants | 118 | 1 |
| (Targum, Cameron et al. 2018)^569^ | Antidepressants+PLB | 116 | 1 |
| (Ghaffari, Ghobadi et al. 2020)^494^ | SSRI+PLB | 19 | 1 |
| (Ghaffari, Ghobadi et al. 2020)^494^ | SSRI+Lauraceae | 23 | 1 |
| (Darbinyan, Aslanyan et al. 2007)^481^ | Crassulaceae | 60 | 1 |
| (Darbinyan, Aslanyan et al. 2007)^481^ | PLB | 29 | 1 |
| (Talaei, Hassanpour Moghadam et al. 2015)^590^ | Iridaceae+Antidepressants | 20 | 1 |
| (Talaei, Hassanpour Moghadam et al. 2015)^590^ | Antidepressants+PLB | 20 | 1 |
| (Akhondzadeh Basti, Moshiri et al. 2007)^457^ | Iridaceae | 20 | 1 |
| (Akhondzadeh Basti, Moshiri et al. 2007)^457^ | SSRI | 20 | 1 |
| (Rondanelli, Giacosa et al. 2010)^543^ | LCPUFA | 22 | 1 |
| (Rondanelli, Giacosa et al. 2010)^543^ | PLB | 24 | 1 |
| (Hosseini, Bagherniya et al. 2024)^501^ | Polyphenols | 20 | 1 |
| (Hosseini, Bagherniya et al. 2024)^501^ | PLB | 20 | 1 |
| (Lecrubier, Clerc et al. 2002)^211^ | HYP | 186 | 1 |
| (Lecrubier, Clerc et al. 2002)^211^ | PLB | 189 | 1 |
| (Kashani, Esalatmanesh et al. 2018)^508^ | Iridaceae | 28 | 1 |
| (Kashani, Esalatmanesh et al. 2018)^508^ | PLB | 28 | 1 |
| (Grenyer, Crowe et al. 2007)^497^ | LCPUFA+Antidepressants | 40 | 1 |
| (Grenyer, Crowe et al. 2007)^497^ | Antidepressants+PLB | 43 | 1 |
| (Jahangard, Sadeghi et al. 2018)^587^ | SSRI+LCPUFA | 25 | 1 |
| (Jahangard, Sadeghi et al. 2018)^587^ | SSRI+PLB | 25 | 1 |
| (Shamabadi, Fattollahzadeh-Noor et al. 2023)^560^ | SSRI+Aminoacids | 25 | 1 |
| (Shamabadi, Fattollahzadeh-Noor et al. 2023)^560^ | SSRI+PLB | 25 | 1 |
| (Firoozeei, Barekatain et al. 2020)^579^ | Lamiaceae+PLB | 25 | 1 |
| (Firoozeei, Barekatain et al. 2020)^579^ | SSRI+PLB | 25 | 1 |
| (Akhondzadeh, Mostafavi et al. 2022)^460^ | Lamiaceae | 21 | 1 |
| (Akhondzadeh, Mostafavi et al. 2022)^460^ | PLB | 22 | 1 |
| (Baião, Capitão et al. 2023)^465^ | PROB | 35 | 1 |
| (Baião, Capitão et al. 2023)^465^ | PLB | 36 | 1 |
| (Rudzki, Ostrowska et al. 2019)^544^ | Antidepressants+PROB | 30 | 1 |
| (Rudzki, Ostrowska et al. 2019)^544^ | Antidepressants+PLB | 30 | 1 |
| (Sahraian, Ghanizadeh et al. 2015)^585^ | SSRI+Vitamins | 21 | 1 |
| (Sahraian, Ghanizadeh et al. 2015)^585^ | SSRI+PLB | 22 | 1 |
| (Ille, Spona et al. 2007)^592^ | Aminoacids+NASSA | 20 | 3 |
| (Ille, Spona et al. 2007)^592^ | NASSA+PLB | 20 | 3 |
| (Lin, Kuo et al. 2024)^521^ | Antidepressants+PROB | 17 | 1 |
| (Lin, Kuo et al. 2024)^521^ | Antidepressants+PLB | 17 | 1 |
| (Akhondzadeh, Fallah-Pour et al. 2004)^458^ | Iridaceae | 15 | 1 |
| (Akhondzadeh, Fallah-Pour et al. 2004)^458^ | TCA | 15 | 1 |
| (Mischoulon, Best-Popescu et al. 2008)^254^ | LCPUFA | 16 | 1 |
| (Mischoulon, Best-Popescu et al. 2008)^254^ | PLB | 19 | 1 |
| (Marangell, Martinez et al. 2003)^526^ | LCPUFA | 18 | 1 |
| (Marangell, Martinez et al. 2003)^526^ | PLB | 17 | 1 |
| (Docherty, Sack et al. 2005)^486^ | Minerals | 70 | 1 |
| (Docherty, Sack et al. 2005)^486^ | PROB | 40 | 1 |
| (Fava, Alpert et al. 2005)^488^ | HYP | 45 | 1 |
| (Fava, Alpert et al. 2005)^488^ | SSRI | 47 | 1 |
| (Fava, Alpert et al. 2005)^488^ | PLB | 43 | 1 |
| (Nemets and Levine 2013)^531^ | Antidepressants+Aminoacids | 4 | 1 |
| (Nemets and Levine 2013)^531^ | Antidepressants+PLB | 4 | 1 |
| (Shahmansouri, Farokhnia et al. 2014)^558^ | Iridaceae | 22 | 1 |
| (Shahmansouri, Farokhnia et al. 2014)^558^ | SSRI | 22 | 1 |
| (Panahi, Badeli et al. 2015)^536^ | Microalgae+Antidepressants | 42 | 1 |
| (Panahi, Badeli et al. 2015)^536^ | Antidepressants | 50 | 1 |
| (Firoozabadi, Zarshenas et al. 2015)^489^ | Antidepressants+Lamiaceae | 15 | 1 |
| (Firoozabadi, Zarshenas et al. 2015)^489^ | Antidepressants+Convolvulaceae | 13 | 1 |
| (Firoozabadi, Zarshenas et al. 2015)^489^ | Antidepressants | 15 | 1 |
| (Ramezani, Kheirkhah et al. 2022)^588^ | SSRI+Aminoacids | 30 | 1 |
| (Ramezani, Kheirkhah et al. 2022)^588^ | SSRI+PLB | 30 | 1 |
| (Shelton, Keller et al. 2001)^562^ | HYP | 98 | 1 |
| (Shelton, Keller et al. 2001)^562^ | PLB | 102 | 1 |
| (Tarutani, Omori et al. 2022)^570^ | Polysaccarids | 9 | 1 |
| (Tarutani, Omori et al. 2022)^570^ | PLB | 11 | 1 |
| (Shahmoradi, Davarinejad et al. 2023)^559^ | Herbal compound | 27 | 1 |
| (Shahmoradi, Davarinejad et al. 2023)^559^ | PLB | 27 | 1 |
| (van Gurp, Meterissian et al. 2002)^572^ | HYP | 44 | 1 |
| (van Gurp, Meterissian et al. 2002)^572^ | SSRI | 43 | 1 |
| (Davidson, Gadde et al. 2002)^482^ | Minerals | 10 | 1 |
| (Davidson, Gadde et al. 2002)^482^ | PLB | 5 | 1 |
| (Wang, Bordi et al. 2015)^575^ | Vitamins | 362 | 1 |
| (Wang, Bordi et al. 2015)^575^ | PLB | 364 | 1 |
| (Kasper, Anghelescu et al. 2015)^512^ | Lamiaceae | 159 | 1 |
| (Kasper, Anghelescu et al. 2015)^512^ | PLB | 156 | 1 |
| (Sahraian, Ghanizadeh et al. 2015)^585^ | SSRI+Iridaceae | 20 | 1 |
| (Sahraian, Ghanizadeh et al. 2015)^585^ | SSRI+PLB | 20 | 1 |
| (Djokic, Korcok et al. 2017)^485^ | Coenzyme | 30 | 1 |
| (Djokic, Korcok et al. 2017)^485^ | PLB | 30 | 1 |
| (Ryszewska-Pokraśniewicz, Mach et al. 2018)^583^ | Minerals+SSRI | 17 | 1 |
| (Ryszewska-Pokraśniewicz, Mach et al. 2018)^583^ | SSRI+PLB | 20 | 1 |
| (Lin, Wang et al. 2022)^522^ | Coenzyme | 39 | 1 |
| (Lin, Wang et al. 2022)^522^ | SSRI | 39 | 1 |
| (Lin, Wang et al. 2022)^522^ | PLB | 39 | 1 |
| (Kaviani, Nikooyeh et al. 2022)^513^ | Vitamins | 28 | 1 |
| (Kaviani, Nikooyeh et al. 2022)^513^ | PLB | 28 | 1 |
| (Ranjbar, Shams et al. 2014)^542^ | Antidepressants+Minerals | 22 | 1 |
| (Ranjbar, Shams et al. 2014)^542^ | Antidepressants | 22 | 1 |
| (He, Liu et al. 2024)^500^ | Ginkgoaceae | 34 | 1 |
| (He, Liu et al. 2024)^500^ | PLB | 34 | 1 |
| (Padhan, Mohapatra et al. 2024)^535^ | Antidepressants+Aminoacids | 30 | 1 |
| (Padhan, Mohapatra et al. 2024)^535^ | Antidepressants+PLB | 30 | 1 |
| (Sakurai, L. Carpenter et al. 2020)^548^ | Coenzyme | 16 | 1 |
| (Sakurai, L. Carpenter et al. 2020)^548^ | SSRI | 23 | 1 |
| (Sakurai, L. Carpenter et al. 2020)^548^ | PLB | 26 | 1 |
| (Vorbach, Arnoldt et al. 1997)^574^ | HYP | 107 | 1 |
| (Vorbach, Arnoldt et al. 1997)^574^ | TCA | 102 | 1 |
| (Lopresti and Drummond 2017)^523^ | Zingiberaceae | 61 | 1 |
| (Lopresti and Drummond 2017)^523^ | Zingiberaceae+Iridaceae | 26 | 1 |
| (Lopresti and Drummond 2017)^523^ | PLB | 36 | 1 |
| (Levine, Barak et al. 1995)^519^ | Vitamins | 13 | 1 |
| (Levine, Barak et al. 1995)^519^ | PLB | 15 | 1 |
| (Sanmukhani, Satodia et al. 2014)^549^ | Zingiberaceae | 20 | 1 |
| (Sanmukhani, Satodia et al. 2014)^549^ | SSRI | 20 | 1 |
| (Sanmukhani, Satodia et al. 2014)^549^ | SSRI+Zingiberaceae | 20 | 1 |
| (Gul, Memtily et al. 2019)^602^ | Herbal compound | 54 | 1 |
| (Gul, Memtily et al. 2019)^602^ | SSRI | 53 | 1 |
| (Gul, Memtily et al. 2019)^602^ | SSRI+Herbal compound | 53 | 1 |
| (Kalb, Trautmann-Sponsel et al. 2001)^506^ | HYP | 37 | 1 |
| (Kalb, Trautmann-Sponsel et al. 2001)^506^ | PROB | 35 | 1 |
| (Wu, Zhu et al. 2015)^601^ | SSRI+Herbal compound | 10 | 1 |
| (Wu, Zhu et al. 2015)^601^ | SSRI+PLB | 8 | 1 |
| (Sepehrmanesh, Kolahdooz et al. 2016)^581^ | SSRI+Vitamins | 45 | 1 |
| (Sepehrmanesh, Kolahdooz et al. 2016)^581^ | SSRI+PLB | 45 | 1 |
| (Szegedi, Kohnen et al. 2005)^567^ | HYP | 122 | 1 |
| (Szegedi, Kohnen et al. 2005)^567^ | SSRI | 122 | 1 |
| (Kanchanatawan, Tangwongchai et al. 2018)^507^ | Zingiberaceae | 33 | 1 |
| (Kanchanatawan, Tangwongchai et al. 2018)^507^ | PLB | 32 | 1 |
| (Sarris, Byrne et al. 2018)^550^ | Antidepressants+Coenzyme | 55 | 1 |
| (Sarris, Byrne et al. 2018)^550^ | Antidepressants+PLB | 52 | 1 |
| (Ghaleiha, Davari et al. 2016)^582^ | SSRI+Vitamins | 25 | 1 |
| (Ghaleiha, Davari et al. 2016)^582^ | SSRI+PLB | 26 | 1 |
| (Basoglu, Ates et al. 2009)^466^ | SSRI+Vitamins | 20 | 1 |
| (Basoglu, Ates et al. 2009)^466^ | SSRI | 15 | 1 |
| Palhano-Fontes F. et al., (2016)^600^ | Herbal compound | 17 | 1 |
| Palhano-Fontes F. et al., (2016)^600^ | PLB | 18 | 1 |
| (Piperoglou, Hopwood et al. 2023)^541^ | LCPUFA | 14 | 1 |
| (Piperoglou, Hopwood et al. 2023)^541^ | PLB | 9 | 1 |
| (Gao, Wu et al. 2020)^492^ | SSRI+PLB | 32 | 1 |
| (Gao, Wu et al. 2020)^492^ | SSRI+Crassulaceae | 66 | 1 |
| (Dormal, Jonniaux et al. 2025)^487^ | Herbal compound | 42 | 1 |
| (Dormal, Jonniaux et al. 2025)^487^ | Iridaceae+Herbal compound | 48 | 1 |
| (Dormal, Jonniaux et al. 2025)^487^ | Iridaceae | 43 | 1 |
| (Dormal, Jonniaux et al. 2025)^487^ | PLB | 47 | 1 |
| (Nemets, Stahl et al. 2002)^296^ | Antidepressants+LCPUFA | 10 | 1 |
| (Nemets, Stahl et al. 2002)^296^ | Antidepressants+PLB | 10 | 1 |
| (Caruso, Fumagalli et al. 1987)^477^ | Coenzyme | 30 | 1 |
| (Caruso, Fumagalli et al. 1987)^477^ | PLB | 30 | 1 |
| (Yu, Pei et al. 2015)^594^ | SSRI+Zingiberaceae | 50 | 1 |
| (Yu, Pei et al. 2015)^594^ | SSRI+PLB | 50 | 1 |
| (Roohi-Azizi, Arabzadeh et al. 2017)^586^ | SSRI+Nucleotide | 25 | 1 |
| (Roohi-Azizi, Arabzadeh et al. 2017)^586^ | SSRI+PLB | 25 | 1 |
| (Anushiravani, Manteghi et al. 2019)^463^ | Herbal compound | 25 | 1 |
| (Anushiravani, Manteghi et al. 2019)^463^ | SSRI | 25 | 1 |
| (Akkasheh, Kashani-Poor et al. 2016)^461^ | PROB | 20 | 1 |
| (Akkasheh, Kashani-Poor et al. 2016)^461^ | PLB | 20 | 1 |
| (Brenner, Azbel et al. 2000)^474^ | HYP | 15 | 1 |
| (Brenner, Azbel et al. 2000)^474^ | SSRI | 15 | 1 |
| (Carney, Steinmeyer et al. 2016)^476^ | SSRI+LCPUFA | 62 | 1 |
| (Carney, Steinmeyer et al. 2016)^476^ | SSRI+PLB | 60 | 1 |
| (Almeida, Ford et al. 2014)^599^ | SSRI+Vitamins | 77 | 1 |
| (Almeida, Ford et al. 2014)^599^ | SSRI+PLB | 76 | 1 |
| (Sharifan, Hosseini et al. 2017)^561^ | LCPUFA | 60 | 1 |
| (Sharifan, Hosseini et al. 2017)^561^ | PLB | 60 | 1 |
| (Chopra and Kalyani 2020)^480^ | Iridaceae | 20 | 1 |
| (Chopra and Kalyani 2020)^480^ | SSRI | 20 | 1 |
| (Wheatley 1997)^429^ | HYP | 83 | 1 |
| (Wheatley 1997)^429^ | TCA | 73 | 1 |
| (Vaghef-Mehrabany, Ranjbar et al. 2021)^571^ | Polysaccarids | 31 | 1 |
| (Vaghef-Mehrabany, Ranjbar et al. 2021)^571^ | PLB | 31 | 1 |
| (Li, Li et al. 2022)^520^ | Herbal compound | 194 | 1 |
| (Li, Li et al. 2022)^520^ | PLB | 191 | 1 |
| (Verma and Das 2016)^573^ | Antidepressants+Minerals | 30 | 1 |
| (Verma and Das 2016)^573^ | Antidepressants | 30 | 1 |
| (Levine, Barak et al. 1995)^604^ | Vitamins | 13 | 1 |
| (Levine, Barak et al. 1995)^604^ | PLB | 14 | 1 |
| (Davidson, Gadde et al. 2002)^483, 607^ | HYP | 113 | 1 |
| (Davidson, Gadde et al. 2002)^483, 607^ | SSRI | 111 | 1 |
| (Davidson, Gadde et al. 2002)^483, 607^ | PLB | 116 | 1 |
| (Sultana and Rahman 2023)^566^ | Minerals | 45 | 1 |
| (Sultana and Rahman 2023)^566^ | PLB | 45 | 1 |
| (Vellekkatt, Menon et al. 2020)^589^ | Antidepressants+Vitamins | 23 | 1 |
| (Vellekkatt, Menon et al. 2020)^589^ | Antidepressants+PROB | 23 | 1 |
| (Masoumi, Kazemi et al. 2016)^580^ | SSRI+PLB | 30 | 1 |
| (Masoumi, Kazemi et al. 2016)^580^ | SSRI+LCPUFA | 30 | 1 |
| (Nowak, Siwek et al. 2003)^534^ | Antidepressants+Minerals | 10 | 1 |
| (Nowak, Siwek et al. 2003)^534^ | Antidepressants+PLB | 10 | 1 |
| (Mech and Farah 2016)^528^ | Vitamins | 170 | 1 |
| (Mech and Farah 2016)^528^ | PLB | 160 | 1 |
| (Ghajar, Neishabouri et al. 2017)^495^ | Iridaceae | 30 | 1 |
| (Ghajar, Neishabouri et al. 2017)^495^ | SSRI | 30 | 1 |
| (Jazayeri, Tehrani-Doost et al. 2008)^504^ | LCPUFA | 16 | 1 |
| (Jazayeri, Tehrani-Doost et al. 2008)^504^ | SSRI | 16 | 1 |
| (Jazayeri, Tehrani-Doost et al. 2008)^504^ | SSRI+LCPUFA | 16 | 1 |
| (Ahmadpanah, Ramezanshams et al. 2019)^456^ | Iridaceae | 25 | 1 |
| (Ahmadpanah, Ramezanshams et al. 2019)^456^ | SSRI | 25 | 1 |
| (Woelk 2000)^576^ | HYP | 157 | 1 |
| (Woelk 2000)^576^ | Antidepressants | 167 | 1 |
| (Moshiri, Basti et al. 2006)^530^ | Iridaceae | 20 | 1 |
| (Moshiri, Basti et al. 2006)^530^ | PROB | 20 | 1 |
| (Akhondzadeh, Mostafavi et al. 2022)^460^ | Lamiaceae | 16 | 1 |
| (Akhondzadeh, Mostafavi et al. 2022)^460^ | TCA | 16 | 1 |
| (Akhondzadeh, Mostafavi et al. 2022)^460^ | TCA+Lamiaceae | 16 | 1 |
| (Harrer, Schmidt et al. 1999)^499^ | HYP | 70 | 1 |
| (Harrer, Schmidt et al. 1999)^499^ | SSRI | 79 | 1 |
| (Kashani, Eslatmanesh et al. 2017)^509^ | Iridaceae | 34 | 1 |
| (Kashani, Eslatmanesh et al. 2017)^509^ | SSRI | 34 | 1 |
| (Berk, Dean et al. 2014)^470^ | Antidepressants+Aminoacids | 127 | 1 |
| (Berk, Dean et al. 2014)^470^ | Antidepressants+PLB | 125 | 1 |
| (Lopresti, Maes et al. 2014)^221^ | Zingiberaceae | 28 | 1 |
| (Lopresti, Maes et al. 2014)^221^ | PLB | 28 | 1 |
| (Lôo, Poirier et al. 2000)^605^ | TCA+Vitamins | 43 | 2 |
| (Lôo, Poirier et al. 2000)^605^ | TCA+PLB | 47 | 2 |
| (Noorbala, Akhondzadeh et al. 2005)^533^ | Iridaceae | 20 | 1 |
| (Noorbala, Akhondzadeh et al. 2005)^533^ | SSRI | 20 | 1 |
| (Bjerkenstedt, Edman et al. 2005)^472^ | HYP | 59 | 1 |
| (Bjerkenstedt, Edman et al. 2005)^472^ | SSRI | 57 | 1 |
| (Bjerkenstedt, Edman et al. 2005)^472^ | PLB | 57 | 1 |
| (Behnke, Jensen et al. 2002)^467^ | HYP | 35 | 1 |
| (Behnke, Jensen et al. 2002)^467^ | SSRI | 35 | 1 |
| (Schrader, Meier et al. 1998)^554^ | HYP | 81 | 1 |
| (Schrader, Meier et al. 1998)^554^ | PROB | 81 | 1 |
| (Schrader 2000)^553^ | HYP | 126 | 1 |
| (Schrader 2000)^553^ | SSRI+PLB | 114 | 1 |
| (Coppen and Bailey 2000)^584^ | SSRI+Vitamins | 62 | 1 |
| (Coppen and Bailey 2000)^584^ | SSRI+PLB | 65 | 1 |
| (Amini, Yaghini et al. 2021)^462^ | Vitamins+Minerals | 26 | 1 |
| (Amini, Yaghini et al. 2021)^462^ | Vitamins | 26 | 1 |
| (Amini, Yaghini et al. 2021)^462^ | PLB | 24 | 1 |
| (Ghorbani, Nazari et al. 2018)^496^ | PROB | 20 | 1 |
| (Ghorbani, Nazari et al. 2018)^496^ | PLB | 20 | 1 |
| (Açik, Çakiroğlu et al. 2025)^455^ | LCPUFA | 38 | 1 |
| (Açik, Çakiroğlu et al. 2025)^455^ | PLB | 19 | 1 |
| (Papakostas, Mischoulon et al. 2010)^538^ | Antidepressants+Coenzyme | 39 | 1 |
| (Papakostas, Mischoulon et al. 2010)^538^ | Antidepressants+PLB | 34 | 1 |
| (Gelenberg, Shelton et al. 2004)^493^ | HYP | 43 | 1 |
| (Gelenberg, Shelton et al. 2004)^493^ | PLB | 55 | 1 |
| (Shafiee-Kandjani, Khalili et al. 2023)^557^ | SSRI+Lamiaceae | 35 | 1 |
| (Shafiee-Kandjani, Khalili et al. 2023)^557^ | SSRI+PLB | 35 | 1 |
| (Talaei, Noori et al. 2023)^568^ | SSRI+Zingiberaceae | 23 | 1 |
| (Talaei, Noori et al. 2023)^568^ | SSRI+PLB | 22 | 1 |
| (Kasper, Anghelescu et al. 2006)^510^ | HYP | 243 | 1 |
| (Kasper, Anghelescu et al. 2006)^510^ | PLB | 81 | 1 |
| (Sadeghi, Ghorayshi et al. 2023)^546^ | LCPUFA+HYP | 37 | 1 |
| (Sadeghi, Ghorayshi et al. 2023)^546^ | SSRI | 37 | 1 |
| (Park, Park et al. 2015)^540^ | Antidepressants+LCPUFA | 18 | 1 |
| (Park, Park et al. 2015)^540^ | Antidepressants+PLB | 17 | 1 |
| (De Leo 1987)^484^ | Coenzyme | 20 | 1 |
| (De Leo 1987)^484^ | PLB | 20 | 1 |
| (Cheng, Chen et al. 2025)^479^ | LCPUFA | 19 | 1 |
| (Cheng, Chen et al. 2025)^479^ | PLB | 20 | 1 |
| (Kreuzer, Reiter et al. 2022)^516, 608^ | PROB | 28 | 1 |
| (Kreuzer, Reiter et al. 2022)^516, 608^ | PLB | 29 | 1 |
| (Sarris, Fava et al. 2012)^606^ | HYP | 35 | 1 |
| (Sarris, Fava et al. 2012)^606^ | SSRI | 49 | 1 |
| (Sarris, Fava et al. 2012)^606^ | PLB | 40 | 1 |
| (Jelodar, Javid et al. 2018)^591^ | SSRI+Iridaceae | 20 | 1 |
| (Jelodar, Javid et al. 2018)^591^ | SSRI+PLB | 20 | 1 |
| (Khoraminya, Tehrani-Doost et al. 2013)^514^ | SSRI+Vitamins | 20 | 1 |
| (Khoraminya, Tehrani-Doost et al. 2013)^514^ | SSRI | 20 | 1 |
| (Sepehrmanesh, Kolahdooz et al. 2016)^556^ | Vitamins | 20 | 1 |
| (Sepehrmanesh, Kolahdooz et al. 2016)^556^ | PLB | 20 | 1 |
| (Sahoo, Jarnail et al. 2015)^547^ | SSRI+LCPUFA | 33 | 1 |
| (Sahoo, Jarnail et al. 2015)^547^ | SSRI+Aminoacids | 37 | 1 |
| (Sahoo, Jarnail et al. 2015)^547^ | SSRI | 33 | 1 |
| (Laakmann 1998)^518^ | HYP | 98 | 1 |
| (Laakmann 1998)^518^ | PLB | 49 | 1 |
| (Chang, Chang et al. 2020)^478^ | LCPUFA | 30 | 1 |
| (Chang, Chang et al. 2020)^478^ | PLB | 29 | 1 |
| (Saccarello, Montarsolo et al. 2020)^545^ | Coenzyme+PROB | 46 | 1 |
| (Saccarello, Montarsolo et al. 2020)^545^ | PLB | 44 | 1 |
| (Friede, Henneicke von Zepelin et al. 2001)^491^ | HYP | 126 | 1 |
| (Friede, Henneicke von Zepelin et al. 2001)^491^ | SSRI | 114 | 1 |
| (Ning, Zhou et al. 2022)^532^ | Herbal compound | 89 | 1 |
| (Ning, Zhou et al. 2022)^532^ | PLB | 87 | 1 |
| (Bergman, Miodownik et al. 2013)^469^ | Zingiberaceae | 20 | 1 |
| (Bergman, Miodownik et al. 2013)^469^ | PLB | 20 | 1 |
| (Kasper, Anghelescu et al. 2007)^511^ | HYP | 68 | 1 |
| (Kasper, Anghelescu et al. 2007)^511^ | PLB | 24 | 1 |
| (Bot, Pouwer et al. 2010)^473^ | LCPUFA | 13 | 1 |
| (Bot, Pouwer et al. 2010)^473^ | PLB | 12 | 1 |
| (Mozaffari-Khosravi, Nabizade et al. 2013)^272^ | LCPUFA | 41 | 1 |
| (Mozaffari-Khosravi, Nabizade et al. 2013)^272^ | PLB | 21 | 1 |
| (Sarris, Murphy et al. 2020)^552^ | Coenzyme | 25 | 1 |
| (Sarris, Murphy et al. 2020)^552^ | PLB | 24 | 1 |
| (Bell, Plon et al. 1988)^468^ | Coenzyme | 9 | 1 |
| (Bell, Plon et al. 1988)^468^ | TCA | 9 | 1 |
| (Siwek, Dudek et al. 2010)^598^ | TCA+Minerals | 30 | 2 |
| (Siwek, Dudek et al. 2010)^598^ | TCA+PLB | 30 | 2 |
| (Papakostas, Shelton et al. 2012)^539^ | Vitamins | 36 | 1 |
| (Papakostas, Shelton et al. 2012)^539^ | PLB | 112 | 1 |
| (Papakostas, Shelton et al. 2012)^539^ | Vitamins | 19 | 1 |
| (Papakostas, Shelton et al. 2012)^539^ | PLB | 56 | 1 |
| (Majmasanaye, Mehrpooya et al. 2024)^524^ | Antidepressants+Coenzyme | 46 | 1 |
| (Majmasanaye, Mehrpooya et al. 2024)^524^ | Antidepressants+PLB | 46 | 1 |
| (Hvas, Juul et al. 2004)^503^ | Vitamins | 70 | 1 |
| (Hvas, Juul et al. 2004)^503^ | PLB | 70 | 1 |
| (Bidzan-Wiącek, Tomczyk et al. 2024)^471^ | LCPUFA | 25 | 1 |
| (Bidzan-Wiącek, Tomczyk et al. 2024)^471^ | PLB | 22 | 1 |
| (Sarris, Byrne et al. 2019)^551^ | Coenzyme+Vitamins+LCPUFA+Minerals+Aminoacids | 81 | 1 |
| (Sarris, Byrne et al. 2019)^551^ | PLB | 77 | 1 |
| (Wu, Yang et al. 2024)^577^ | LCPUFA | 30 | 1 |
| (Wu, Yang et al. 2024)^577^ | PLB | 30 | 1 |
| (Zhang, Wang et al. 2018)^578^ | Vitamins | 56 | 1 |
| (Zhang, Wang et al. 2018)^578^ | PLB | 64 | 1 |
| (Kasper, Volz et al. 2024)^597^ | Lamiaceae | 170 | 1 |
| (Kasper, Volz et al. 2024)^597^ | SSRI | 171 | 1 |
| (Kasper, Volz et al. 2024)^597^ | PLB | 157 | 1 |
| (Kolouri, Firoozabadi et al. 2016)^515^ | Lamiaceae | 33 | 1 |
| (Kolouri, Firoozabadi et al. 2016)^515^ | SSRI | 33 | 1 |
| (Rees, Austin et al. 2008)^332^ | LCPUFA | 13 | 1 |
| (Rees, Austin et al. 2008)^332^ | PLB | 13 | 1 |
| (Freeman, Davis et al. 2008)^490^ | LCPUFA | 31 | 1 |
| (Freeman, Davis et al. 2008)^490^ | PLB | 28 | 1 |
| (Varzaghani, Sharifi et al. 2022)^596^ | Flavonoid+Polyphenols | 28 | 1 |
| (Varzaghani, Sharifi et al. 2022)^596^ | PLB | 26 | 1 |
| (Shinto, Marracci et al. 2016)^564^ | LCPUFA | 21 | 1 |
| (Shinto, Marracci et al. 2016)^564^ | PLB | 18 | 1 |
| (Su, Huang et al. 2008)^387^ | LCPUFA | 18 | 1 |
| (Su, Huang et al. 2008)^387^ | PLB | 18 | 1 |
| (Mischoulon, Dunlop et al. 2022)^603^ | LCPUFA | 16 | 1 |
| (Mischoulon, Dunlop et al. 2022)^603^ | PLB | 15 | 1 |
| (Su, Huang et al. 2003)^565^ | LCPUFA | 12 | 1 |
| (Su, Huang et al. 2003)^565^ | PLB | 10 | 1 |
| (Yang, Wang et al. 2022)^595^ | SNRI+LCPUFA | 36 | 4 |
| (Yang, Wang et al. 2022)^595^ | SNRI+PLB | 36 | 4 |
| (Kagan, Sultzer et al. 1990)^505^ | Coenzyme | 9 | 1 |
| (Kagan, Sultzer et al. 1990)^505^ | PLB | 6 | 1 |
| (Han, Chung et al. 2012)^498^ | Herbal compound | 82 | 1 |
| (Han, Chung et al. 2012)^498^ | PLB | 82 | 1 |
| (Pancheri, Scapicchio et al. 2002)^537^ | Coenzyme | 146 | 1 |
| (Pancheri, Scapicchio et al. 2002)^537^ | TCA | 147 | 1 |
| (Mischoulon, Price et al. 2014)^529^ | Coenzyme | 64 | 1 |
| (Mischoulon, Price et al. 2014)^529^ | SSRI | 65 | 1 |
| (Mischoulon, Price et al. 2014)^529^ | PLB | 60 | 1 |
| (Mao, Xie et al. 2015)^525^ | SSRI | 19 | 1 |
| (Mao, Xie et al. 2015)^525^ | PLB | 18 | 1 |
| (Sempach, Schaub et al. 2025)^555^ | Aminoacids+Antidepressants | 26 | 1 |
| (Sempach, Schaub et al. 2025)^555^ | Antidepressants+PLB | 26 | 1 |

**Reference for e-table 12**

1.	Mazidi M, Shemshian M, Mousavi SH, Norouzy A, Kermani T, Moghiman T, et al. A double-blind, randomized and placebo-controlled trial of Saffron (Crocus sativus L.) in the treatment of anxiety and depression. 2016;13(2):195‐9

C3 - PUBMED 27101556,EMBASE 610483123.

2.	Araj-Khodaei M, Noorbala AA, Yarani R, Emadi F, Emaratkar E, Faghihzadeh S, et al. A double-blind, randomized pilot study for comparison of Melissa officinalis L. And Lavandula angustifolia Mill. And Fluoxetine for the treatment of depression. BMC Complementary Medicine and Therapies. 2020;20(1).

3.	Akhondzadeh S, Mostafavi SA, Keshavarz SA, Mohammadi MR, Hosseini S, Eshraghian MR. A placebo controlled randomized clinical trial of Crocus sativus L. (saffron) on depression and food craving among overweight women with mild to moderate depression. Journal of Clinical Pharmacy and Therapeutics. 2020;45(1):134-43.

4.	Carney RM, Freedland KE, Rubin EH, Rich MW, Steinmeyer BC, Harris WS. A randomized placebo-controlled trial of omega-3 and sertraline in depressed patients with or at risk for coronary heart disease. Journal of Clinical Psychiatry. 2019;80(4 C7 - 19m12742).

5.	Lyoo IK, Yoon S, Kim TS, Hwang J, Kim JE, Won W, et al. A randomized, double-blind placebo-controlled trial of oral creatine monohydrate augmentation for enhanced response to a selective serotonin reuptake inhibitor in women with major depressive disorder. American Journal of Psychiatry. 2012;169(9):937-45.

6.	Sherwood Brown E, Gabrielson B. A randomized, double-blind, placebo-controlled trial of citicoline for bipolar and unipolar depression and methamphetamine dependence. Journal of Affective Disorders. 2012;143(1-3):257-60.

7.	Kumar PNS, Menon V, Andrade C. A randomized, double-blind, placebo-controlled, 12-week trial of vitamin D augmentation in major depressive disorder associated with vitamin D deficiency. Journal of Affective Disorders. 2022;314:143-9.

8.	Hu Y, Chen C, Wang Y, Yang W, Wang Y, Zhu W, et al. The effects of KaiXinSan on depression and its association with lipid profiles: a randomized, double-blinded, placebo-controlled trial. 2021;83:153467

C3 - PUBMED 33516143,EMBASE 2010802711.

9.	Targum SD, Cameron BR, Ferreira L, MacDonald ID. An augmentation study of MSI-195 (S-adenosylmethionine) in Major Depressive Disorder. Journal of Psychiatric Research. 2018;107:86-96.

10.	Ghaffari S, Ghobadi A, Jamshidi AH, Mortazavi SH, Naderi S, Aqamolaei A, et al. Cinnamomum tamala as an adjuvant therapy in the treatment of major depressive disorder: A double-blind, randomized, placebo-controlled clinical trial with placebo control. Advances in Integrative Medicine. 2020;7(3):141-7.

11.	Darbinyan V, Aslanyan G, Amroyan E, Gabrielyan E, Malmström C, Panossian A. Clinical trial of Rhodiola rosea L. extract SHR-5 in the treatment of mild to moderate depression. Nordic Journal of Psychiatry. 2007;61(5):343-8.

12.	Talaei A, Hassanpour Moghadam M, Sajadi Tabassi SA, Mohajeri SA. Crocin, the main active saffron constituent, as an adjunctive treatment in major depressive disorder: A randomized, double-blind, placebo-controlled, pilot clinical trial. Journal of Affective Disorders. 2015;174:51-6.

13.	Akhondzadeh Basti A, Moshiri E, Noorbala AA, Jamshidi AH, Abbasi SH, Akhondzadeh S. Comparison of petal of Crocus sativus L. and fluoxetine in the treatment of depressed outpatients: A pilot double-blind randomized trial. Progress in Neuro-Psychopharmacology and Biological Psychiatry. 2007;31(2):439-42.

14.	Rondanelli M, Giacosa A, Opizzi A, Pelucchi C, Vecchia CL, Montorfano G, et al. Effect of omega-3 fatty acids supplementation on depressive symptoms and on health-related quality of life in the treatment of elderly women with depression: A double-blind, placebo-controlled, randomized clinical trial. Journal of the American College of Nutrition. 2010;29(1):55-64.

15.	Hosseini H, Bagherniya M, Sahebkar A, Iraj B, Majeed M, Askari G. The effect of curcumin-piperine supplementation on lipid profile, glycemic index, inflammation, and blood pressure in patients with type 2 diabetes mellitus and hypertriglyceridemia. Phytotherapy Research. 2024;38(11):5150-61.

16.	Lecrubier Y, Clerc G, Didi R, Kieser M. Efficacy of St. John's wort extract WS 5570 in major depression: A double-blind, placebo-controlled trial. American Journal of Psychiatry. 2002;159(8):1361-6.

17.	Kashani L, Esalatmanesh S, Eftekhari F, Salimi S, Foroughifar T, Etesam F, et al. Efficacy of Crocus sativus (saffron) in treatment of major depressive disorder associated with post-menopausal hot flashes: a double-blind, randomized, placebo-controlled trial. Arch Gynecol Obstet. 2018;297(3):717-24.

18.	Grenyer BF, Crowe T, Meyer B, Owen AJ, Grigonis-Deane EM, Caputi P, et al. Fish oil supplementation in the treatment of major depression: a randomised double-blind placebo-controlled trial. 2007;31(7):1393‐6

C3 - PUBMED 17659823.

19.	Jahangard L, Sadeghi A, Ahmadpanah M, Holsboer-Trachsler E, Sadeghi Bahmani D, Haghighi M, et al. Influence of adjuvant omega-3-polyunsaturated fatty acids on depression, sleep, and emotion regulation among outpatients with major depressive disorders - Results from a double-blind, randomized and placebo-controlled clinical trial. Journal of Psychiatric Research. 2018;107:48-56.

20.	Shamabadi A, Fattollahzadeh-Noor S, Fallahpour B, A. Basti F, Khodaei Ardakani MR, Akhondzadeh S. L-Theanine adjunct to risperidone in the treatment of chronic schizophrenia inpatients: a randomized, double-blind, placebo-controlled clinical trial. Psychopharmacology. 2023;240(12):2631-40.

21.	Firoozeei TS, Barekatain M, Karimi M, Zargaran A, Akhondzadeh S, Rezaeizadeh H. Lavender and dodder combined herbal syrup versus citalopram in major depressive disorder with anxious distress: A double-blind randomized trial. Journal of Integrative Medicine. 2020;18(5):409-15.

22.	Akhondzadeh S, Mostafavi SA, Keshavarz SA, Mohammadi MR, Chamari M. Melatonin Effects in Women With Comorbidities of Overweight, Depression, and Sleep Disturbance: A Randomized Placebo Controlled Clinical Trial. Sleep Med Res. 2022;13(1):22-30.

23.	Baião R, Capitão LP, Higgins C, Browning M, Harmer CJ, Burnet PWJ. Multispecies probiotic administration reduces emotional salience and improves mood in subjects with moderate depression: a randomised, double-blind, placebo-controlled study. Psychological medicine. 2023;53(8):3437-47.

24.	Rudzki L, Ostrowska L, Pawlak D, Małus A, Pawlak K, Waszkiewicz N, et al. Probiotic Lactobacillus Plantarum 299v decreases kynurenine concentration and improves cognitive functions in patients with major depression: A double-blind, randomized, placebo controlled study. Psychoneuroendocrinology. 2019;100:213-22.

25.	Sahraian A, Ghanizadeh A, Kazemeini F. Vitamin C as an adjuvant for treating major depressive disorder and suicidal behavior, a randomized placebo-controlled clinical trial. Trials. 2015;16(1 C7 - 94).

26.	Ille R, Spona J, Zickl M, Hofmann P, Lahousen T, Dittrich N, et al. "Add-On"-therapy with an individualized preparation consisting of free amino acids for patients with a major depression. European Archives of Psychiatry and Clinical Neuroscience. 2007;257(4):222-9.

27.	Lin SKK, Kuo PH, Hsu CY, Chiu YH, Chen CH. The effects of Lactobacillus plantarum PS128 in patients with major depressive disorder: an eight-week double-blind, placebo-controlled study. Asian Journal of Psychiatry. 2024;101 C7 - 104210.

28.	Akhondzadeh S, Fallah-Pour H, Afkham K, Jamshidi AH, Khalighi-Cigaroudi F. Comparison of Crocus sativus L. and imipramine in the treatment of mild to moderate depression: A pilot double-blind randomized trial [ISRCTN45683816]. BMC Complementary and Alternative Medicine. 2004;4.

29.	Mischoulon D, Best-Popescu C, Laposata M, Merens W, Murakami JL, Wu SL, et al. A double-blind dose-finding pilot study of docosahexaenoic acid (DHA) for major depressive disorder. European Neuropsychopharmacology. 2008;18(9):639-45.

30.	Marangell LB, Martinez JM, Zboyan HA, Kertz B, Kim HFS, Puryear LJ. A double-blind, placebo-controlled study of the omega-3 fatty acid docosahexaenoic acid in the treatment of major depression. American Journal of Psychiatry. 2003;160(5):996-8.

31.	Docherty JP, Sack DA, Roffman M, Finch M, Komorowski JR. A double-blind, placebo-controlled, exploratory trial of chromium picolinate in atypical depression: Effect on carbohydrate craving. Journal of Psychiatric Practice. 2005;11(5):302-14.

32.	Fava M, Alpert J, Nierenberg AA, Mischoulon D, Otto MW, Zajecka J, et al. A double-blind, randomized trial of St John's wort, fluoxetine, and placebo in major depressive disorder. Journal of Clinical Psychopharmacology. 2005;25(5):441-7.

33.	Nemets B, Levine J. A pilot dose-finding clinical trial of creatine monohydrate augmentation to SSRIs/SNRIs/NASA antidepressant treatment in major depression. International Clinical Psychopharmacology. 2013;28(3):127-33.

34.	Shahmansouri N, Farokhnia M, Abbasi SH, Kassaian SE, Noorbala Tafti AA, Gougol A, et al. A randomized, double-blind, clinical trial comparing the efficacy and safety of Crocus sativus L. with fluoxetine for improving mild to moderate depression in post percutaneous coronary intervention patients. 2014;155:216‐22

C3 - PUBMED 24289892,EMBASE 52891114.

35.	Panahi Y, Badeli R, Karami GR, Badeli Z, Sahebkar A. A randomized controlled trial of 6-week Chlorella vulgaris supplementation in patients with major depressive disorder. Complement Ther Med. 2015;23(4):598-602.

36.	Firoozabadi A, Zarshenas MM, Salehi A, Jahanbin S, Mohagheghzadeh A. Effectiveness of Cuscuta planiflora Ten. and Nepeta menthoides Boiss. & Buhse in major depression: a triple-blind randomized controlled trial study. J Evid Based Complementary Altern Med. 2015;20(2):94-7.

37.	Ramezani A, Kheirkhah F, Zabihi E, Shirafkan H, Moudi S. Effectiveness of L-carnitine Supplementation to Sertraline for Treatment of Major Depressive Disorder: A Double-blind Randomized Placebo-controlled Trial. Glob J Med Pharm Biomed Update. 2022;17.

38.	Shelton RC, Keller MB, Gelenberg A, Dunner DL, Hirschfeld R, Thase ME, et al. Effectiveness of St John's wort in major depression: A randomized controlled trial. JAMA. 2001;285(15):1978-86.

39.	Tarutani S, Omori M, Ido Y, Yano M, Komatsu T, Okamura T. Effects of 4G-beta-D-Galactosylsucrose in patients with depression: A randomized, double-blinded, placebo-controlled, parallel-group comparative study. J Psychiatr Res. 2022;148:110-20.

40.	Shahmoradi N, Davarinejad O, Brühl AB, Brand S. Effects of Aphrodite (an Herbal Compound) on SSRI-Induced Sexual Dysfunctions and Depression in Females with Major Depressive Disorder: Findings from a Randomized Clinical Trial. Medicina (Kaunas, Lithuania). 2023;59(9).

41.	van Gurp G, Meterissian GB, Haiek LN, McCusker J, Bellavance F. St John's wort or sertraline? Randomized controlled trial in primary care. Can Fam Physician. 2002;48:905-12.

42.	Davidson JR, Gadde KM, Fairbank JA, Krishnan KRR, Califf RM, Binanay C, et al. Effect of Hypericum perforatum (St John's wort) in major depressive disorder: a randomized controlled trial. 2002;287(14):1807‐14

C3 - PUBMED 11939866,EMBASE 34303625.

43.	Wang L, Bordi PL, Fleming JA, Hill AM, Kris-Etherton PM. Effect of a moderate fat diet with and without avocados on lipoprotein particle number, size and subclasses in overweight and obese adults: a randomized, controlled trial. J Am Heart Assoc. 2015;4(1):e001355.

44.	Kasper S, Anghelescu I, Dienel A. Efficacy of orally administered Silexan in patients with anxiety-related restlessness and disturbed sleep--A randomized, placebo-controlled trial. 2015;25(11):1960‐7

C3 - PUBMED 26293583,EMBASE 605643089.

45.	Djokic G, Korcok D, Djordjevic V, Agic A, Rankovic A, Djukic Dejanovic S. The effects of s-adenosyl-L-methionine-vitamin b complex on mild and moderate depressive symptoms. Hippokratia. 2017;21(3):140-3.

46.	Ryszewska-Pokraśniewicz B, Mach A, Skalski M, Januszko P, Wawrzyniak ZM, Poleszak E, et al. Effects of magnesium supplementation on unipolar depression: A placebo-controlled study and review of the importance of dosing and magnesium status in the therapeutic response. Nutrients. 2018;10(8 C7 - 1014).

47.	Lin CH, Wang SH, Lane HY. Effects of Sodium Benzoate, a D-Amino Acid Oxidase Inhibitor, on Perceived Stress and Cognitive Function Among Patients With Late-Life Depression: A Randomized, Double-Blind, Sertraline- and Placebo-Controlled Trial. International Journal of Neuropsychopharmacology. 2022;25(7):545-55.

48.	Kaviani M, Nikooyeh B, Etesam F, Behnagh SJ, Kangarani HM, Arefi M, et al. Effects of vitamin D supplementation on depression and some selected pro-inflammatory biomarkers: a double-blind randomized clinical trial. BMC Psychiatry. 2022;22(1 C7 - 694).

49.	Ranjbar E, Shams J, Sabetkasaei M, M-Shirazi M, Rashidkhani B, Mostafavi A, et al. Effects of zinc supplementation on efficacy of antidepressant therapy, inflammatory cytokines, and brain-derived neurotrophic factor in patients with major depression. Nutritional Neuroscience. 2014;17(2):65-71.

50.	He X, Liu D, Ni S, Li Z, Li S, Wu T, et al. Efficacy and safety evaluation of Ginkgo biloba dropping pill (GBDP) on stable angina pectoris complicated with depression: A placebo-controlled, randomized, double-blind, multicenter study. Phytomedicine. 2024;126.

51.	Padhan M, Mohapatra D, Mishra BR, Maiti R, Jena M. Efficacy and safety of add-on sarcosine in patients with major depressive disorder: A randomized controlled trial. Journal of Psychiatric Research. 2024;178:298-304.

52.	Sakurai H, L. Carpenter L, R. Tyrka A, Price LH, I. Papakostas G, Dording CM, et al. Dose increase of S-Adenosyl-Methionine and escitalopram in a randomized clinical trial for major depressive disorder. Journal of Affective Disorders. 2020;262:118-25.

53.	Vorbach EU, Arnoldt KH, Hubner WD. Efficacy and tolerability of St. John's wort extract LI 160 versus imipramine in patients with severe depressive episodes according to ICD-10. Pharmacopsychiatry. 1997;30(SUPPL.):81-5.

54.	Lopresti AL, Drummond PD. Efficacy of curcumin, and a saffron/curcumin combination for the treatment of major depression: A randomised, double-blind, placebo-controlled study. Journal of Affective Disorders. 2017;207:188-96.

55.	Levine J, Barak Y, Gonzalves M, Szor H, Elizur A, Kofman O, et al. Double-blind, controlled trial of inositol treatment of depression. American Journal of Psychiatry. 1995;152(5):792-4.

56.	Sanmukhani J, Satodia V, Trivedi J, Patel T, Tiwari D, Panchal B, et al. Efficacy and safety of curcumin in major depressive disorder: a randomized controlled trial. Phytother Res. 2014;28(4):579-85.

57.	Gul A, Memtily N, Mijit P, Imin M, Rui H, Wushuer P, et al. The Üstikuddus sherbiti in the treatment of abnormal cold and dry-type depression patients with comorbid anxiety: A randomized and controlled clinical trial. Trad Med Mod Med. 2019;2(3):127-33.

58.	Kalb R, Trautmann-Sponsel RD, Kieser M. Efficacy and tolerability of Hypericum extract WS 5572 versus placebo in mildly to moderately depressed patients: A randomized double-blind multicenter clinical trial. Pharmacopsychiatry. 2001;34(3):96-103.

59.	Wu R, Zhu D, Xia Y, Wang H, Tao W, Xue W, et al. A role of Yueju in fast-onset antidepressant action on major depressive disorder and serum BDNF expression: A randomly double-blind, fluoxetine-adjunct, placebo-controlled, pilot clinical study. Neuropsychiatric Disease and Treatment. 2015;11:2013-21.

60.	Sepehrmanesh Z, Kolahdooz F, Abedi F, Mazroii N, Assarian A, Asemi Z, et al. Vitamin D Supplementation Affects the Beck Depression Inventory, Insulin Resistance, and Biomarkers of Oxidative Stress in Patients with Major Depressive Disorder: A Randomized, Controlled Clinical Trial [Retraction notice in CN-02298463]. 2016;146(2):243‐8

C3 - PUBMED 26609167,EMBASE 608794678.

61.	Szegedi A, Kohnen R, Dienel A, Kieser M. Acute treatment of moderate to severe depression with hypericum extract WS 5570 (St John's wort): Randomised controlled double blind non-inferiority trial versus paroxetine. British Medical Journal. 2005;330(7490):503-6.

62.	Kanchanatawan B, Tangwongchai S, Sughondhabhirom A, Suppapitiporn S, Hemrunrojn S, Carvalho AF, et al. Add-on Treatment with Curcumin Has Antidepressive Effects in Thai Patients with Major Depression: Results of a Randomized Double-Blind Placebo-Controlled Study. Neurotoxic Res. 2018;33(3):621-33.

63.	Sarris J, Byrne GJ, Bousman C, Stough C, Murphy J, MacDonald P, et al. Adjunctive S-adenosylmethionine (SAMe) in treating non-remittent major depressive disorder: An 8-week double-blind, randomized, controlled trial. European Neuropsychopharmacology. 2018;28(10):1126-36.

64.	Ghaleiha A, Davari H, Jahangard L, Haghighi M, Ahmadpanah M, Seifrabie MA, et al. Adjuvant thiamine improved standard treatment in patients with major depressive disorder: results from a randomized, double-blind, and placebo-controlled clinical trial. European Archives of Psychiatry and Clinical Neuroscience. 2016;266(8):695-702.

65.	Basoglu C, Ates MA, Algul A, Ipcioglu OM, Gecici O, Yilmaz O, et al. Adjuvant folate with escitalopram treatment and homocystein, folate, vitamin B-12 levels in patients with major depressive disorder. 2009;19(2):135‐42

C3 - EMBASE 354832073.

66.	Antidepressant Effects of Ayahuasca: a Randomized Placebo Controlled Trial in Treatment Resistant Depression. clinicaltrialsgov2016.

67.	Piperoglou M, Hopwood M, Norman TR. Adjunctive Docosahexaenoic Acid in Residual Symptoms of Depression and Anxiety. Journal of Clinical Psychopharmacology. 2023;43(6):493-7.

68.	Gao L, Wu C, Liao Y, Wang J. Antidepressants effects of Rhodiola capsule combined with sertraline for major depressive disorder: A randomized double-blind placebo-controlled clinical trial. Journal of Affective Disorders. 2020;265:99-103.

69.	Dormal V, Jonniaux L, Buchet M, Simar L, Copine S, Deldicque L. Effect of Hydroponically Grown Red Panax Ginseng on Perceived Stress Level, Emotional Processing, and Cognitive Functions in Moderately Stressed Adults: A Randomized, Double-Blind, Placebo-Controlled Study. Nutrients. 2025;17(6 C7 - 955).

70.	Nemets B, Stahl Z, Belmaker RH. Addition of omega-3 fatty acid to maintenance medication treatment for recurrent unipolar depressive disorder. American Journal of Psychiatry. 2002;159(3):477-9.

71.	Caruso I, Fumagalli M, Boccassini L, Puttini SP, Santandrea S, Giniselli G, et al. Treatment of depression in rheumatoid arthritic patients. A comparison of S-adenosylmethionine (Samyr) and placebo in a double-blind study. CLIN TRIALS J. 1987;24(4):305-10.

72.	Yu JJ, Pei LB, Zhang Y, Wen ZY, Yang JL. Chronic Supplementation of Curcumin Enhances the Efficacy of Antidepressants in Major Depressive Disorder: A Randomized, Double-Blind, Placebo-Controlled Pilot Study. Journal of Clinical Psychopharmacology. 2015;35(4):406-10.

73.	Roohi-Azizi M, Arabzadeh S, Amidfar M, Salimi S, Zarindast MR, Talaei A, et al. Citicoline combination therapy for major depressive disorder: A randomized, double-blind, placebo-controlled trial. Clinical Neuropharmacology. 2017;40(1):1-5.

74.	Anushiravani M, Manteghi AA, Taghipur A, Eslami M. Comparing Effectiveness of a Combined Herbal Drug Based on Echium Amoenum with Citalopram in the Treatment of Major Depressive Disorder. Curr Drug Discov Technol. 2019;16(2):232-8.

75.	Akkasheh G, Kashani-Poor Z, Tajabadi-Ebrahimi M, Jafari P, Akbari H, Taghizadeh M, et al. Clinical and metabolic response to probiotic administration in patients with major depressive disorder: A randomized, double-blind, placebo-controlled trial. Nutrition. 2016;32(3):315-20.

76.	Brenner R, Azbel V, Madhusoodanan S, Pawlowska M. Comparison of an extract of hypericum (LI 160) and sertraline in the treatment of depression: A double-blind, randomized pilot study. Clinical Therapeutics. 2000;22(4):411-9.

77.	Carney RM, Steinmeyer BC, Freedland KE, Rubin EH, Rich MW, Harris WS. Baseline blood levels of omega-3 and depression remission: A secondary analysis of data from a placebo-controlled trial of omega-3 supplements. Journal of Clinical Psychiatry. 2016;77(2):e138-e43.

78.	Almeida OP, Ford AH, Hirani V, Singh V, VanBockxmeer FM, McCaul K, et al. B vitamins to enhance treatment response to antidepressants in middle-aged and older adults: Results from the B-VITAGE randomised, double-blind, placebo-controlled trial. British Journal of Psychiatry. 2014;205(6):450-7.

79.	Sharifan P, Hosseini MS, Sharifan A. The interventional relationship between frequent fish consumption and depression symptoms in aging adults: a randomized controlled trial. International Journal of Geriatric Psychiatry. 2017;32(12):e116-e22.

80.	Chopra A, Kalyani AK. Comparison of efficacy of Crocus sativus L. with fluoxetine for improving depression among patients with cardiovascular diseases. Int J Life Sci Biotechnol Pharma Res. 2020;9(2):151-4.

81.	Wheatley D. LI 160, an extract of St. John's wort, versus amitriptyline in mildly to moderately depressed outpatients--a controlled 6-week clinical trial. 1997;30 Suppl 2:77‐80

C3 - PUBMED 9342764.

82.	Vaghef-Mehrabany E, Ranjbar F, Asghari-Jafarabadi M, Hosseinpour-Arjmand S, Ebrahimi-Mameghani M. Calorie restriction in combination with prebiotic supplementation in obese women with depression: effects on metabolic and clinical response. Nutritional Neuroscience. 2021;24(5):339-53.

83.	Li Y, Li L, Guo R, Yu H, Wang X, Wang B, et al. Clinical efficacy of Shugan granule in the treatment of mixed anxiety-depressive disorder: a multicenter, randomized, double-blind, placebo-controlled trial. 2022;290:115032

C3 - PUBMED 35085742,EMBASE 637131287.

84.	Verma DK, Das B. Effect of zinc supplementation on antidepressant therapy and serum zinc level in unipolar depression: A randomized double blind controlled study. Indian Journal of Psychiatry. 2016;58(5):S47.

85.	Levine J, Barak Y, Kofman O, Belmaker RH. Follow-up and relapse analysis of an inositol study of depression. Isr J Psychiatry Relat Sci. 1995;32(1):14-21.

86.	Davidson JRT, Gadde KM, Fairbank JA, Ranga Rama Krishnan K, Califf RM, Binanay C, et al. Effect of Hypericum perforatum (St John's wort) in major depressive disorder: A randomized controlled trial. JAMA. 2002;287(14):1807-14.

87.	Sultana S, Rahman MS. Effect of magnesium supplementation in selective serotonin reuptake inhibitors treated major depressive disorder patients: A randomized, double-blind, placebo-controlled trial. EC Nutr. 2023;18:25-34.

88.	Vellekkatt F, Menon V, Rajappa M, Sahoo J. Effect of adjunctive single dose parenteral Vitamin D supplementation in major depressive disorder with concurrent vitamin D deficiency: A double-blind randomized placebo-controlled trial. J Psychiatr Res. 2020;129:250-6.

89.	Masoumi SZ, Kazemi F, Tavakolian S, Rahimi A, Oshvandi K, Soltanian A, et al. Effect of citalopram in combination with omega-3 on depression in post-menopausal women: A triple blind randomized controlled trial. Journal of Clinical and Diagnostic Research. 2016;10(10):QC01-QC5.

90.	Nowak G, Siwek M, Dudek D, Zieba A, Pilc A. Effect of zinc supplementation on antidepressant therapy in unipolar depression: A preliminary placebo-controlled study. Polish Journal of Pharmacology. 2003;55(6):1143-7.

91.	Mech AW, Farah A. Correlation of clinical response with homocysteine reduction during therapy with reduced b vitamins in patients with mdd who are positive for MTHFR C677T or A1298C polymorphism: A Randomized, double-blind, placebo-controlled study. Journal of Clinical Psychiatry. 2016;77(5):668-71.

92.	Ghajar A, Neishabouri SM, Velayati N, Jahangard L, Matinnia N, Haghighi M, et al. Crocus sativus L versus Citalopram in the Treatment of Major Depressive Disorder with Anxious Distress: A Double-Blind, Controlled Clinical Trial. Pharmacopsychiatry. 2017;50(4):152-60.

93.	Jazayeri S, Tehrani-Doost M, Keshavarz SA, Hosseini M, Djazayery A, Amini H, et al. Comparison of therapeutic effects of omega-3 fatty acid eicosapentaenoic acid and fluoxetine, separately and in combination, in major depressive disorder. Australian and New Zealand Journal of Psychiatry. 2008;42(3):192-8.

94.	Ahmadpanah M, Ramezanshams F, Ghaleiha A, Akhondzadeh S, Sadeghi Bahmani D, Brand S. Crocus Sativus L. (saffron) versus sertraline on symptoms of depression among older people with major depressive disorders–a double-blind, randomized intervention study. Psychiatry Research. 2019;282.

95.	Woelk H. Comparison of St John's wort and imipramine for treating depression: Randomised controlled trial. British Medical Journal. 2000;321(7260):536-9.

96.	Moshiri E, Basti AA, Noorbala AA, Jamshidi AH, Hesameddin Abbasi S, Akhondzadeh S. Crocus sativus L. (petal) in the treatment of mild-to-moderate depression: A double-blind, randomized and placebo-controlled trial. Phytomedicine. 2006;13(9-10):607-11.

97.	Harrer G, Schmidt U, Kuhn U, Biller A. Comparison of equivalence between the St. John's wort extract LoHyp-57 and fluoxetine. Arzneimittel-Forschung/Drug Research. 1999;49(4):289-96.

98.	Kashani L, Eslatmanesh S, Saedi N, Niroomand N, Ebrahimi M, Hosseinian M, et al. Comparison of Saffron versus Fluoxetine in Treatment of Mild to Moderate Postpartum Depression: A Double-Blind, Randomized Clinical Trial. Pharmacopsychiatry. 2017;50(2):64-8.

99.	Berk M, Dean OM, Cotton SM, Jeavons S, Tanious M, Kohlmann K, et al. The efficacy of adjunctive N-acetylcysteine in major depressive disorder: A double-blind, randomized, placebo-controlled trial. Journal of Clinical Psychiatry. 2014;75(6):628-36.

100.	Lopresti AL, Maes M, Maker GL, Hood SD, Drummond PD. Curcumin for the treatment of major depression: A randomised, double-blind, placebo controlled study. Journal of Affective Disorders. 2014;167:368-75.

101.	Lôo H, Poirier MF, Ollat H, Elatki S. Effects of sulbutiamine (Arcalion 200) on psycho-behavioral inhibition in major depressive episodes. 2000;26(2):70‐5

C3 - PUBMED 10858919,EMBASE 30337124.

102.	Noorbala AA, Akhondzadeh S, Tahmacebi-Pour N, Jamshidi AH. Hydro-alcoholic extract of Crocus sativus L. versus fluoxetine in the treatment of mild to moderate depression: A double-blind, randomized pilot trial. Journal of Ethnopharmacology. 2005;97(2):281-4.

103.	Bjerkenstedt L, Edman GV, Alken RG, Mannel M. Hypericum extract LI 160 and fluoxetine in mild to moderate depression: A randomized, placebo-controlled multi-center study in outpatients. European Archives of Psychiatry and Clinical Neuroscience. 2005;255(1):40-7.

104.	Behnke K, Jensen GS, Graubaum HJ, Gruenwald J. Hypericum perforatum versus fluoxetine in the treatment of mild to moderate depression. Advances in Therapy. 2002;19(1):43-52.

105.	Schrader E, Meier B, Brattström A. Hypericum treatment of mild-moderate depression in a placebo-controlled study. A prospective, double-blind, randomized, placebo-controlled, multicentre study. Human Psychopharmacology. 1998;13(3):163-9.

106.	Schrader E. Equivalence of St John's wort extract (Ze 117) and fluoxetine: A randomized, controlled study in mild-moderate depression. International Clinical Psychopharmacology. 2000;15(2):61-8.

107.	Coppen A, Bailey J. Enhancement of the antidepressant action of fluoxetine by folic acid: A randomised, placebo controlled trial. Journal of Affective Disorders. 2000;60(2):121-30.

108.	Amini L, Yaghini O, Ghazavi M, Aslani N. L-carnitine versus Propranolol for pediatric migraine prophylaxis. Iran J Child Neurol. 2021;15(2):77-86.

109.	Ghorbani Z, Nazari S, Etesam F, Nourimajd S, Ahmadpanah M, Jahromi SR. The effect of synbiotic as an adjuvant therapy to fluoxetine in moderate depression: A randomized multicenter trial. Arch Neurosci. 2018;5(2).

110.	Açik M, Çakiroğlu FP, Tekin A, Egeli A. The effect of fish oil versus krill oil intervention on clinical symptoms and cardiometabolic risk factors in patients with major depressive disorder: A randomized placebo-controlled double-blind trial. Journal of Affective Disorders. 2025;380:171-80.

111.	Papakostas GI, Mischoulon D, Shyu I, Alpert JE, Fava M. S-adenosyl methionine (SAMe) augmentation of serotonin reuptake inhibitors for antidepressant nonresponders with major depressive disorder: a double-blind, randomized clinical trial. 2010;167(8):942‐8

C3 - PUBMED 20595412.

112.	Gelenberg AJ, Shelton RC, Crits-Christoph P, Keller MB, Dunner DL, Hirschfeld RM, et al. The effectiveness of St. John's Wort in major depressive disorder: a naturalistic phase 2 follow-up in which nonresponders were provided alternate medication. 2004;65(8):1114‐9

C3 - PUBMED 15323598.

113.	Shafiee-Kandjani A, Khalili M, Malek A, Farhang S, Ranjbari Y, Khalili Y. The therapeutic effect of the extracts of Lavandula angustifolia and Dracocephalum ruyschiana besides sertraline on patients with mild to moderate depression: A double-blind controlled trial. Phytomedicine Plus. 2023;3(2).

114.	Talaei A, Noori R, Rezaei Ardani A, Mohammadpour AH, Azimipoor Z, Afzaljavan F. The Effect of Adding Curcumin to Sertraline in the Treatment of Severe Major Depressive Disorder: A Randomized, Double-Blind Clinical Trial. Clinical Neuropharmacology. 2023;46(4):135-9.

115.	Kasper S, Anghelescu IG, Szegedi A, Dienel A, Kieser M. Superior efficacy of St John's wort extract WS 5570 compared to placebo in patients with major depression: a randomized, double-blind, placebo-controlled, multi-center trial. 2006;4:14

C3 - PUBMED 16796730,EMBASE 44227549.

116.	Sadeghi A, Ghorayshi F, Baghshahi H, Akbari H, Memarzadeh MR, Taghizadeh M, et al. The antidepressant effect of combined extracts of Hypericum perforatum and Echium amoenum supplementation in patients with depression symptoms: A randomized clinical trial. Avicenna J Phytomedicine. 2023;13(4):328-37.

117.	Park Y, Park YS, Kim SH, Oh DH, Park YC. Supplementation of n-3 polyunsaturated fatty acids for major depressive disorder: A randomized, double-blind, 12-week, placebo-controlled trial in Korea. Annals of Nutrition and Metabolism. 2015;66(2-3):141-8.

118.	De Leo D. S-Adenosylmethionine as an antidepressant. A double-blind trial versus placebo. CURR THER RES CLIN EXP. 1987;41(6):865-70.

119.	Cheng YC, Chen WY, Lin C, Lee SH, Chiu CC, Kuo PH. The N-3 polyunsaturated fatty acids supplementation to prevent depression recurrence in patients with late-life depression: A 52-week double-blind, placebo-controlled trial. Journal of Affective Disorders. 2025;369:8-15.

120.	Kreuzer K, Reiter A, Birkl-Töglhofer AM, Dalkner N, Mörkl S, Mairinger M, et al. The PROVIT Study—Effects of Multispecies Probiotic Add-on Treatment on Metabolomics in Major Depressive Disorder—A Randomized, Placebo-Controlled Trial. Metabolites. 2022;12(8).

121.	Sarris J, Fava M, Schweitzer I, Mischoulon D. St Johns Wort (Hypericum perforatum) versus sertraline and placebo in major depressive disorder: Continuation data from a 26-week RCT. Pharmacopsychiatry. 2012;45(7):275-8.

122.	Jelodar G, Javid Z, Sahraian A, Jelodar S. Saffron improved depression and reduced homocysteine level in patients with major depression: A Randomized, double-blind study. Avicenna J Phytomedicine. 2018;8(1):43-50.

123.	Khoraminya N, Tehrani-Doost M, Jazayeri S, Hosseini A, Djazayery A. Therapeutic effects of vitamin D as adjunctive therapy to fluoxetine in patients with major depressive disorder. Australian and New Zealand Journal of Psychiatry. 2013;47(3):271-5.

124.	Sepehrmanesh Z, Kolahdooz F, Abedi F, Mazroii N, Assarian A, Asemi Z, et al. Vitamin D supplementation affects the beck depression inventory, insulin resistance, and biomarkers of oxidative stress in patients with major depressive disorder: A randomized, controlled clinical trial. Journal of Nutrition. 2016;146(2):243-8.

125.	Sahoo JP, Jarnail S, Khurana H, Singh P. Omega-3 Fatty Acids Versus 5-hydroxytryptophan as Add on Therapy in Patients with Depression: A Comparative Study. Indian Journal of Pharmacology. 2015;47(7):S23-S4.

126.	Laakmann G. St. John's Wort in mild to moderate depression: The relevance of hyperforin for the clinical efficacy. Pharmacopsychiatry. 1998;31(SUPPL. 1):54-9.

127.	Chang JP, Chang SS, Yang HT, Chen HT, Chien YC, Yang B, et al. Omega-3 polyunsaturated fatty acids in cardiovascular diseases comorbid major depressive disorder - Results from a randomized controlled trial. Brain Behav Immun. 2020;85:14-20.

128.	Saccarello A, Montarsolo P, Massardo I, Picciotto R, Pedemonte A, Castagnaro R, et al. Oral Administration of S-Adenosylmethionine (SAMe) and Lactobacillus Plantarum HEAL9 Improves the Mild-To-Moderate Symptoms of Depression: a Randomized, Double-Blind, Placebo-Controlled Study. 2020;22(4 C3 - PUBMED 32589828,EMBASE 632201612).

129.	Friede M, Henneicke von Zepelin HH, Freudenstein J. Differential therapy of mild to moderate depressive episodes (ICD-10 F 32.0; F 32.1) with St. John's wort. Pharmacopsychiatry. 2001;34(SUPPL. 1):S38-S41.

130.	Ning H, Zhou H, Ren J, Zhou G, Yang N, Wang Z, et al. Zishen pingchan granules combined with pramipexole in the improvement of depressive symptoms in Parkinson's disease: a prospective, multicenter, randomized, double-blind, controlled clinical study. J Transl Med. 2022;20(1):357.

131.	Bergman J, Miodownik C, Bersudsky Y, Sokolik S, Lerner PP, Kreinin A, et al. Curcumin as an add-on to antidepressive treatment: A randomized, double-blind, placebo-controlled, pilot clinical study. Clinical Neuropharmacology. 2013;36(3):73-7.

132.	Kasper S, Anghelescu IG, Szegedi A, Dienel A, Kieser M. Placebo controlled continuation treatment with Hypericum extract WS® 5570 after recovery from a mild or moderate depressive episode. Wiener Medizinische Wochenschrift. 2007;157(13-14):362-6.

133.	Bot M, Pouwer F, Assies J, Jansen EH, Diamant M, Snoek FJ, et al. Eicosapentaenoic acid as an add-on to antidepressant medication for co-morbid major depression in patients with diabetes mellitus: a randomized, double-blind placebo-controlled study. J Affect Disord. 2010;126(1-2):282-6.

134.	Mozaffari-Khosravi H, Nabizade L, Yassini-Ardakani SM, Hadinedoushan H, Barzegar K. The effect of 2 different single injections of high dose of vitamin D on improving the depression in depressed patients with vitamin D deficiency: A randomized clinical trial. Journal of Clinical Psychopharmacology. 2013;33(3):378-85.

135.	Sarris J, Murphy J, Stough C, Mischoulon D, Bousman C, MacDonald P, et al. S-Adenosylmethionine (SAMe) monotherapy for depression: an 8-week double-blind, randomised, controlled trial. 2020;237(1):209‐18

C3 - PUBMED 31712971,EMBASE 2003633556.

136.	Bell KM, Plon L, Bunney Jr WE, Potkin SG. S-adenosylmethionine treatment of depression: A controlled clinical trial. American Journal of Psychiatry. 1988;145(9):1110-4.

137.	Siwek M, Dudek D, Schlegel-Zawadzka M, Morawska A, Piekoszewski W, Opoka W, et al. Serum zinc level in depressed patients during zinc supplementation of imipramine treatment. Journal of Affective Disorders. 2010;126(3):447-52.

138.	Papakostas GI, Shelton RC, Zajecka JM, Etemad B, Rickels K, Clain A, et al. L-methylfolate as adjunctive therapy for SSRI-resistant major depression: results of two randomized, double-blind, parallel-sequential trials. 2012;169(12):1267‐74

C3 - PUBMED 23212058.

139.	Majmasanaye M, Mehrpooya M, Amiri H, Eshraghi A. Discovering the Potential Value of Coenzyme Q10 as an Adjuvant Treatment in Patients with Depression. Journal of Clinical Psychopharmacology. 2024;44(3):232-9.

140.	Hvas AM, Juul S, Lauritzen L, Nexø E, Ellegaard J. No effect of vitamin B-12 treatment on cognitive function and depression: A randomized placebo controlled study. Journal of Affective Disorders. 2004;81(3):269-73.

141.	Bidzan-Wiącek M, Tomczyk M, Błażek M, Mika A, Antosiewicz J. No Effects of Omega-3 Supplementation on Kynurenine Pathway, Inflammation, Depressive Symptoms, and Stress Response in Males: A Placebo-Controlled Trial. Nutrients. 2024;16(21 C7 - 3744).

142.	Sarris J, Byrne GJ, Stough C, Bousman C, Mischoulon D, Murphy J, et al. Nutraceuticals for major depressive disorder- more is not merrier: An 8-week double-blind, randomised, controlled trial. Journal of Affective Disorders. 2019;245:1007-15.

143.	Wu SK, Yang KJ, Liu WC, Malau IA, Zailani H, Chang CH, et al. The Efficacy of Omega-3 Fatty Acids as the Monotherapy for Depression: A Randomized, Double-Blind, Placebo-Controlled Pilot Study. Nutrients. 2024;16(21).

144.	Zhang L, Wang S, Zhu Y, Yang T. Vitamin D3 as adjunctive therapy in the treatment of depression in tuberculosis patients: A short-term pilot randomized double-blind controlled study. Neuropsychiatric Disease and Treatment. 2018;14:3103-9.

145.	Kasper S, Volz HP, Möller HJ, Schläfke S, Klement S, Anghelescu IG, et al. Lavender oil preparation Silexan is effective in mild-to-moderate major depression: a randomized, placebo- and reference-controlled trial. European Archives of Psychiatry and Clinical Neuroscience. 2024.

146.	Kolouri S, Firoozabadi A, Salehi A, Zarshenas MM, Dastgheib SA, Heydari M, et al. Nepeta menthoides Boiss. & Buhse freeze-dried aqueous extract versus sertraline in the treatment of major depression: A double blind randomized controlled trial. Complement Ther Med. 2016;26:164-70.

147.	Rees AM, Austin MP, Parker GB. Omega-3 fatty acids as a treatment for perinatal depression: Randomized double-blind placebo-controlled trial. Australian and New Zealand Journal of Psychiatry. 2008;42(3):199-205.

148.	Freeman MP, Davis M, Sinha P, Wisner KL, Hibbeln JR, Gelenberg AJ. Omega-3 fatty acids and supportive psychotherapy for perinatal depression: A randomized placebo-controlled study. Journal of Affective Disorders. 2008;110(1-2):142-8.

149.	Varzaghani V, Sharifi M, Hajiaghaee R, Bagheri S, Momtaz S, Tarassoli Z, et al. Propolis add-on therapy alleviates depressive symptoms; A randomized placebo-controlled clinical trial. Phytotherapy Research. 2022;36(3):1258-67.

150.	Shinto L, Marracci G, Mohr DC, Bumgarner L, Murchison C, Senders A, et al. Omega-3 fatty acids for depression in multiple sclerosis: A randomized pilot study. PLoS ONE. 2016;11(1 C7 - e0147195).

151.	Su KP, Huang SY, Chiu TH, Huang KC, Huang CL, Chang HC, et al. Omega-3 fatty acids for major depressive disorder during pregnancy: Results from a randomized, double-blind, placebo-controlled trial. Journal of Clinical Psychiatry. 2008;69(4):644-51.

152.	Mischoulon D, Dunlop BW, Kinkead B, Schettler PJ, Lamon-Fava S, Rakofsky JJ, et al. Omega-3 Fatty Acids for Major Depressive Disorder With High Inflammation: A Randomized Dose-Finding Clinical Trial. Journal of Clinical Psychiatry. 2022;83(5 C7 - 21m14074).

153.	Su KP, Huang SY, Chiu CC, Shen WW. Omega-3 fatty acids in major depressive disorder: A preliminary double-blind, placebo-controlled trial. European Neuropsychopharmacology. 2003;13(4):267-71.

154.	Yang R, Wang L, Jin K, Cao S, Wu C, Guo J, et al. Omega-3 Polyunsaturated Fatty Acids Supplementation Alleviate Anxiety Rather Than Depressive Symptoms Among First-Diagnosed, Drug-Naïve Major Depressive Disorder Patients: A Randomized Clinical Trial. Frontiers in Nutrition. 2022;9 C7 - 876152.

155.	Kagan BL, Sultzer DL, Rosenlicht N, Gerner RH. Oral S-adenosylmethionine in depression: a randomized, double-blind, placebo-controlled trial. 1990;147(5):591‐5

C3 - PUBMED 2183633.

156.	Han CH, Chung MI, Bose S, Lim C, Kim H, Koo BS, et al. The effect of Gami Guibitang (GGBT), a medicinal herbal formulation, on moderate depression. European Journal of Integrative Medicine. 2012;4(4):e400-e7.

157.	Pancheri P, Scapicchio P, Delle Chiaie R. A double-blind, randomized parallel-group, efficacy and safety study of intramuscular S-adenosyl-L-methionine 1, 4-butanedisulphonate (SAMe) versus imipramine in patients with major depressive disorder. International Journal of Neuropsychopharmacology. 2002;5(4):287-94.

158.	Mischoulon D, Price LH, Carpenter LL, Tyrka AR, Papakostas GI, Baer L, et al. A double-blind, randomized, placebo-controlled clinical trial of S-adenosyl-L-methionine (SAMe) versus escitalopram in major depressive disorder. J Clin Psychiatry. 2014;75(4):370-6.

159.	Mao JJ, Xie SX, Zee J, Soeller I, Li QS, Rockwell K, et al. Rhodiola rosea versus sertraline for major depressive disorder: A randomized placebo-controlled trial. Phytomedicine. 2015;22(3):394-9.

160.	Sempach L, Schaub A-C, Brühl AB, Sterzer P, Lang UE, Schmidt A. Adjunctive d-serine treatment for major depressive disorder: A randomized clinical trial. Journal of Affective Disorders. 2025:119504.

# **S6:** Analyses (Network plots, forest plots, funnel plots, and cumulative rankings).

***In this section, PBO refers to Probiotics, not placebo (PLB)!***

**e-figure 1:** Cumulative **efficacy** ranking **node 1** based on SMD (decrease in depressive symptoms): P-SCORE, which is the rank of treatment within the range of treatments, measured on a scale from 0 (worst) to 1 (best).

**e-figure 2**: Visual inspection of the funnel plot and Egger’s test for **efficacy node 1**.

**e-figure 3a:** Network plot of **efficacy node 2** (based on SMD). Nodes are proportional to sample size; edges’ width is proportional to the number of comparisons.

**e-figure 3b:** Forest plot for **efficacy node 2** (reference is placebo).

**e-figure 4a:** Cumulative **efficacy** ranking **node 2** based on SMD (decrease in depressive symptoms): P-SCORE, which is the rank of treatment within the range of treatments, measured on a scale from 0 (worst) to 1 (best): how each intervention is likely to perform better than every alternative one.

**e-figure 4b:** Visual inspection of the funnel plot and Egger’s test for **efficacy node 2**

**e-figure 5:** Cumulative **response** ranking **node 1** based on SMD (decrease in depressive symptoms): P-Score, which is the rank of treatment within the range of treatments, measured on a scale from 0 (worst) to 1 (best): how each intervention is likely to perform better than every alternative one.

**e-figure 6:** Visual inspection of the funnel plot and Egger’s test for **response node 1**.

**e-figure 7a:** Network plot of **response node 2** (based on RR). Nodes are proportional to sample size; edges’ width is proportional to the number of comparisons.

**e-figure 7b:** Forest plot for **response node 2** (reference is placebo),

**e-figure 8a:** Cumulative **response** ranking **node 2** based on SMD (decrease in depressive symptoms): P-SCORE, which is the rank of treatment within the range of treatments, measured on a scale from 0 (worst) to 1 (best).

**e-figure 8b:** Visual inspection of the funnel plot and Egger’s test for **response node 2**

**e-figure 9:** Cumulative **acceptability** ranking **node 1** based on SMD (decrease in depressive symptoms): P-SCORE, which is the rank of treatment within the range of treatments, measured on a scale from 0 (worst) to 1 (best): how each intervention is likely to perform better than every alternative one.

**e-figure 10:** Visual inspection of the funnel plot and Egger’s test for **acceptability node 1**

**e-figure 11a:** Network plot of **acceptability node 2** (based on RR). Nodes are proportional to sample size; edges’ width is proportional to the number of comparisons.

**e-figure 11b:** Forest plot for **acceptability node 2** (reference is placebo).

**e-figure 12a:** Cumulative **acceptability** ranking **node 2** based on SMD (decrease in depressive symptoms): P-score, which is the rank of treatment within the range of treatments, measured on a scale from 0 (worst) to 1 (best): how each intervention is likely to perform better than every alternative one.

**e-figure 12b:** Visual inspection of the funnel plot and Egger’s test for **acceptability node 2.**

**e-figure 13:** Network plot of **anxious symptoms node 1** (based on SMD). Nodes are proportional to sample size; edges’ width is proportional to the number of comparisons.

**e-figure 14:** Forest plot of **anxious symptoms node 1** (reference is placebo)

**e-figure 15:** Cumulative **anxious symptoms** ranking **node 1** based on SMD (decrease in depressive symptoms): P-score, which is the rank of treatment within the range of treatments, measured on a scale from 0 (worst) to 1 (best): how each intervention is likely to perform better than every alternative one.

**e-figure 16:** Visual inspection of the funnel plot and Egger’s test for **anxious symptoms node 1**.

**e-figure 17:** Network plot of **anxious symptoms node 2** (based on SMD). Nodes are proportional to sample size; edges’ width is proportional to the number of comparisons.

**e-figure 18:** Forest plot of **anxious symptoms node 2** (reference is placebo)

**e-figure 19:** Cumulative **anxious symptoms** ranking **node 2** based on SMD (decrease in depressive symptoms):P-score, which is the rank of treatment within the range of treatments, measured on a scale from 0 (worst) to 1 (best): how each intervention is likely to perform better than every alternative one.

**e-figure 20:** Visual inspection of the funnel plot and Egger’s test for **anxious symptoms node 2**

**e-figure 21:** Network plot of **remission node 1**. Nodes are proportional to sample size; edges’ width is proportional to the number of comparisons.

**e-figure 22:** Forest plot of **remission node 1** (reference is placebo)

**e-figure 23:** Cumulative **remission node 1** ranking: P-SCORE, which is the rank of treatment within the range of treatments, measured on a scale from 0 (worst) to 1 (best): how each intervention is likely to perform better than every alternative one.

**e-figure 24:** Visual inspection of the funnel plot and Egger’s test for **remission node 1**

**e-figure 25:** Network plot of **remission node 2**. Nodes are proportional to sample size; edges’ width is proportional to the number of comparisons.

**e-figure 26:** Forest plot of **remission node 2** (reference is placebo).

**e-figure 27:** Cumulative **remission node 2** ranking: P-SCORE, which is the rank of treatment within the range of treatments, measured on a scale from 0 (worst) to 1 (best): how each intervention is likely to perform better than every alternative one.

**e-figure 28:** Visual inspection of the funnel plot and Egger’s test for **remission node 2**.

**e-figure 29:** Network plot of **tolerability node 1.** Nodes are proportional to sample size; edges’ width is proportional to the number of comparisons.

**e-figure 30:** Forest plot of **tolerability node 1** (reference is placebo)

**e-figure 31:** Cumulative **tolerability node 1** ranking: P-score, which is the rank of treatment within the range of treatments, measured on a scale from 0 (worst) to 1 (best): how each intervention is likely to perform better than every alternative one.

**e-figure 32:** Visual inspection of the funnel plot and Egger’s test for **tolerability node 1**

**e-figure 33:** Network plot of **tolerability node 2.** Nodes are proportional to sample size; edges’ width is proportional to the number of comparisons.

**e-figure 34:** Forest plot of **tolerability node 2** (reference is placebo).

**e-figure 35:** Cumulative **tolerability node 2** ranking: P-score, which is the rank of treatment within the range of treatments, measured on a scale from 0 (worst) to 1 (best).

**e-figure 36:** Visual inspection of the funnel plot and Egger’s test for **tolerability node 2**

# **S7a:** Evaluation of heterogeneity and inconsistency – Multiple outcomes and effect sizes.

| **Measure** | **What it measures** | **Interpretation** |
| --- | --- | --- |
| **Q total** | The **overall variability** in the network (heterogeneity + inconsistency) | It is the sum of **Q within** and **Q between**. High value = possible inconsistency or heterogeneity. |
| **Q within** | **Within-comparison heterogeneity** (variation among studies within the same contrast) | High value = studies comparing the same treatments show different results. |
| **Q between** | **Inconsistency** between direct and indirect evidence | High value = disagreement between direct and indirect estimates in the network. |
| **τ² (tau-squared)** | The **variance** of true effect sizes across studies (random-effects model) | The higher it is, the more the treatment effects vary across studies. |
| **I²** | The **percentage of variability** not due to chance | Values >50% often indicate substantial heterogeneity. |

## **S7a.1:** Heterogeneity

**e-table 13.** Between-study variance and heterogeneity – all studies node 1

.

**e-table 14.** Between-study variance and heterogeneity – all studies node 2

## **S7a.2:** Global consistency

**e-table 15** Global inconsistency under the assumption of a full-design-by-treatment interaction model – all studies node 1

**e-table 16** Global inconsistency under the assumption of a full-design-by-treatment interaction model – all studies node 2

## **S7a.3:** Local consistency – split of direct and indirect evidence.

**e-table 17.** Local inconsistency is measured as a ratio of ratios or difference between direct and indirect estimates for **efficacy node 1**. Only the relevant comparisons for the network estimate are shown herein.

**e-table 18.** Local inconsistency is measured as a ratio of ratios or difference between direct and indirect estimates for **efficacy node 2**. Only the relevant comparisons for the network estimate are shown herein.

**e-table 19.** Local inconsistency is measured as a ratio of ratios or difference between direct and indirect estimates for **response node 1**. Only the relevant comparisons for the network estimate are shown herein.

**e-table 20.** Local inconsistency is measured as a ratio of ratios or difference between direct and indirect estimates for **response node 2.** Only the relevant comparisons for the network estimate are shown herein.

**e-table 21.** Local inconsistency is measured as a ratio of ratios or difference between direct and indirect estimates for **acceptability node 1**. Only the relevant comparisons for the network estimate are shown herein.

**e-table 22.** Local inconsistency is measured as a ratio of ratios or difference between direct and indirect estimates for **acceptability node 2**. Only the relevant comparisons for the network estimate are shown herein.

**e-table 23.** Local inconsistency is measured as a ratio of ratios or difference between direct and indirect estimates for **anxious symptoms node 1**. Only the relevant comparisons for the network estimate are shown herein.

**e-table 24.** Local inconsistency is measured as a ratio of ratios or difference between direct and indirect estimates for **anxious symptoms node 2.** Only the relevant comparisons for the network estimate are shown herein.

**e-table 25.** Local inconsistency is measured as a ratio of ratios or difference between direct and indirect estimates for **remission node 1**. Only the relevant comparisons for the network estimate are shown herein.

**e-table 26.** Local inconsistency is measured as a ratio of ratios or difference between direct and indirect estimates for **remission node 2**. Only the relevant comparisons for the network estimate are shown herein.

**e-table 27.** Local inconsistency is measured as a ratio of ratios or difference between direct and indirect estimates for **tolerability node 1**. Only the relevant comparisons for the network estimate are shown herein.

**e-table 28.** Local inconsistency is measured as a ratio of ratios or difference between direct and indirect estimates for **tolerability node 2**. Only the relevant comparisons for the network estimate are shown herein.

# **S7b**: Meta-regression analysis.

**Linear regression: SMD ~ AMSTAR plus**

Total depressive symptoms change β=0.42, 95%CI=0.11-0.72, p=<0.01

**Interpretation:** The higher the AMSTAR score, the higher the SMD, meaning that the effect decreases when the quality of the study increases (more negative SMDs indicate higher differences between post- and pre-treatment). Thus, larger effect sizes should be interpreted with caution since they rely on studies rated lower quality.

# **S8:** Transitivity analyses.

**e-figure 37:** Age distribution across different study arms.

**e-table 29**: Outliers for mean age

| **age** | **author_year** |
| --- | --- |
| **84.9** | Rondanelli M. et al., 2010 |
| **83** | Rondanelli M. et al., 2010 |
| **66.4** | Lin C. et al., 2022 |
| **66.9** | Lin C. et al., 2022 |
| **70.5** | Lin C. et al., 2022 |
| **67.9** | Sharifan P. et al., 2017 |
| **66.72** | Ahmadpanah M et al., 2019 |
| **68.4** | Harrer G et al., 1999 |
| **69.1** | Harrer G et al., 1999 |
| **68.36** | Cheng Y. et al., 2025 |
| **69.36** | Cheng Y. et al., 2025 |
| **68.1** | Ning H. et al., 2022 |
| **66.4** | Ning H. et al., 2022 |
| **65.8** | Bergman J et al., 2013 |
| **75** | Hvas AM et al., 2004 |
| **74** | Hvas AM et al., 2004 |

**e-figure 38:** Proportion of females across different study arms.

**e-table 30**: Outliers for proportion of females

| **prop** | **author_year** |
| --- | --- |
| **0** | Kumar PNS et al., 2022 |
| **0** | Kumar PNS et al., 2022 |
| **0** | Hosseini S.H. et al., 2024 |
| **0** | Hosseini S.H. et al., 2024 |
| **0** | Shahmansouri N. et al., 2013 |
| **138** | Zhang L. et al., 2018 |
| **0** | Kagan BL et al., 1990 |
| **0** | Kagan BL et al., 1990 |

**e-table 31**: HAM-D mean and SD at baseline.

| **Author, year** | **Treatment** | **n** | **HAM-D mean at baseline** | **HAM-D SD at baseline** | **Efficacy outcome assessment** |
| --- | --- | --- | --- | --- | --- |
| ^464^ | MEL | 16 | 17.8 | 3.04 | HAM-D |
| ^464^ | LAV | 17 | 17.2 | 3.61 | HAM-D |
| ^464^ | FLUO | 17 | 18.4 | 3.06 | HAM-D |
| eh, Mostafavi et al. 2020) | SAF | 36 | 21.82 | 7.77 | BDI-II |
| eh, Mostafavi et al. 2020) | PLB | 37 | 21.4 | 6.8 | BDI-II |
| ^475^ | SER + EPA | 71 | 17.4 | 5.4 | BDI-II |
| ^475^ | SER + PLB | 73 | 17.0 | 5.0 | BDI-II |
| ^593^ | ESC+CREA | 25 | 27.0 | 3.9 | HAM-D |
| ^593^ | ESC+PLB | 27 | 26.7 | 4.3 | HAM-D |
| ^517^ | ESC + vitD | 31 | 25.7 | 8.0 | HAM-D |
| ^517^ | ESC + PLB | 28 | 25.8 | 10.3 | HAM-D |
| ^502^ | SZL + PLB | 63 | 18.79 | 2.09 | HAM-D |
| ^502^ | FLUO + PLB | 66 | 18.53 | 1.95 | HAM-D |
| ^569^ | SAMe+TAU | 118 | 21.2 | 3.8 | HAM-D |
| ^569^ | PLB+TAU | 116 | 21.4 | 3.5 | HAM-D |
| ^494^ | SER + PLB | 25 | 24.6 | 4.79 | HAM-D |
| ^494^ | SER+CIN | 25 | 25.4 | 3.73 | HAM-D |
| ^481^ | RHO | 60 | 23.79 | 1.7 | HAM-D |
| ^481^ | PLB | 29 | 24.17 | 1.7 | HAM-D |
| ^501^ | EA | 20 | 177565 | 4.4 | HAM-D |
| ^501^ | PLB | 20 | 17368 | 4.42 | HAM-D |
| ^211^ | WS5570 | 186 | 21.9 | 1.7 | HAM-D |
| ^211^ | PLB | 189 | 21.9 | 1.7 | HAM-D |
| (Kashani, Esalatmanesh et al. 2018 | SAFF | 28 | 15.29 | 6.05 | HAM-D |
| (Kashani, Esalatmanesh et al. 2018 | PLB | 28 | 15.96 | 4.26 | HAM-D |
| ^587^ | OMEGA3+SER | 25 | 29312 | 6.5 | MADRS |
| ^587^ | PLB+SER | 25 | 25356 | 6.4 | MADRS |
| ^560^ | SER+THE | 30 | 26.88 | 3.03 | HAM-D |
| ^560^ | SER+PLB | 30 | 26.52 | 3.77 | HAM-D |
| ^579^ | LAV +PLB | 28 | 20.12 | 1.94 | HAM-D |
| ^579^ | CTP + PLB | 25 | 19.72 | 1.69 | HAM-D |
| ^460^ | MEL | 21 | 21085 | 12285 | BDI |
| ^460^ | PLB | 22 | 21.82 | 9.45 | BDI |
| ^544^ | LP299v+TAU | 30 | 21.53 | 6.03 | HAM-D |
| ^544^ | PLB+TAU | 30 | 22.00 | 7.92 | HAM-D |
| ^585^ | vitC+CTP | 21 | 21.4 | 4.1 | HAM-D |
| ^585^ | PLB+CTP | 22 | 22.4 | 3 | HAM-D |
| ^592^ | fAA+MIR | 20 | 26.7 | 5.9 | HAM-D |
| ^592^ | PLB+MIR | 20 | 20.7 | 3.6 | HAM-D |
| ^521^ | PS128+TAU | 17 | 20.38 | 5.63 | HAM-D |
| ^521^ | PLB+TAU | 17 | 19.88 | 4.33 | HAM-D |
| ^458^ | SAFF | 15 | 19.20 | 0.42 | HAM-D |
| ^458^ | IMI | 15 | 19.00 | 0.444 | HAM-D |
| ^254^ | EPA | 16 | 21.3 | 3 | HAM-D |
| ^254^ | PLB | 19 | 20.5 | 3.8 | HAM-D |
| ^526^ | DHA | 18 | 23.5 | 3.1 | HAM-D |
| ^526^ | PLB | 17 | 28.5 | 4.5 | HAM-D |
| ^486^ | CHRO | 70 | 35.3 | 1.2 | HAM-D |
| ^486^ | PROB | 40 | 33.6 | 1.2 | HAM-D |
| ^488^ | LI-160 | 45 | 19.6 | 3.5 | HAM-D |
| ^488^ | FLUO | 47 | 19.6 | 3.1 | HAM-D |
| ^488^ | PLB | 43 | 19.9 | 2.9 | HAM-D |
| ^531^ | TAU+CREA | 4 | 22.3 | 5.3 | HAM-D |
| ^531^ | TAU+PLB | 4 | 27.8 | 5.7 | HAM-D |
| ^558^ | SAFF | 22 | 17.00 | 1.77 | HAM-D |
| ^558^ | FLUO | 22 | 16.80 | 1.79 | HAM-D |
| ^536^ | CVE+TAU | 42 | 17.52 | 3.06 | BDI |
| ^536^ | TAU | 50 | 20.26 | 3.64 | BDI |
| ^489^ | NEP+TAU | 15 | 29.6 | 5.5 | HAM-D |
| ^489^ | CUS+TAU | 13 | 27.2 | 5.2 | HAM-D |
| ^489^ | TAU | 15 | 24.7 | 5.7 | HAM-D |
| ^588^ | CAR+SER | 30 | 29.8 | 9681 | BDI |
| ^588^ | PLB+SER | 30 | 306715 | 10.5 | BDI |
| ^562^ | SJW | 98 | 17.4 | 5.1 | HAM-D |
| ^562^ | PLB | 102 | 19.7 | 6.8 | HAM-D |
| ^570^ | LS | 9 | 11.94 | 6.23 | MADRS |
| ^570^ | PLB | 11 | 14.57 | 6.23 | MADRS |
| ^559^ | APH | 27 | 18.13 | 6.85 | BDI |
| ^559^ | PLB | 27 | 16.57 | 7.48 | BDI |
| ^572^ | SJW | 44 | 18.9 | 3.6 | HAM-D |
| ^572^ | SER | 43 | 19.7 | 3.5 | HAM-D |
| ^482^ | CHRO | 10 | 30.9 | 6 | HAM-D |
| ^482^ | PLB | 5 | 29.8 | 5.2 | HAM-D |
| ^575^ | vitD3 | 362 | 22.7 | 4.3 | BDI |
| ^575^ | PLB | 364 | 21.9 | 5.4 | BDI |
| ^512^ | SIL | 159 | 16.27 | 5504 | HAM-D |
| ^512^ | PLB | 156 | 16356 | 5246 | HAM-D |
| ^585^ | FLUO+SAFF | 20 | 21736 | 6.23 | BDI |
| ^585^ | FLUO + PLB | 20 | 22177 | 6.23 | BDI |
| ^485^ | TEN | 30 | 20.17 | 3.89 | HAM-D |
| ^485^ | PLB | 30 | 19.4 | 3.6 | HAM-D |
| ^583^ | MG+FLUO | 17 | 30.5 | 6 | HAM-D |
| ^583^ | PLB+FLUO | 20 | 27.5 | 5.5 | HAM-D |
| ^522^ | BZT | 39 | 26.1 | 5.1 | HAM-D |
| ^522^ | SER | 39 | 27.0 | 5.2 | HAM-D |
| ^522^ | PLB | 39 | 25.4 | 6 | HAM-D |
| ^513^ | vitD | 28 | 23563 | 5.76 | BDI-II |
| ^513^ | PLB | 28 | 112255 | 6426 | BDI-II |
| ^500^ | GBDP | 34 | 13.68 | 4.63 | HAM-D |
| ^500^ | PLB | 34 | 12.88 | 3.82 | HAM-D |
| ^535^ | TAU+SAR | 30 | 13518 | 4558 | MADRS |
| ^535^ | TAU+PLB | 30 | 14808 | 3784 | MADRS |
| ^548^ | SAMe | 16 | 18.6 | 4.2 | HAM-D |
| ^548^ | ESC | 23 | 20.1 | 3.6 | HAM-D |
| ^548^ | PLB | 26 | 19.8 | 3.8 | HAM-D |
| ^574^ | LI-160 | 107 | 25.3 | 4.7 | HAM-D |
| ^574^ | IMI | 102 | 26.1 | 4.8 | HAM-D |
| ^519^ | INO | 13 | 29.2 | 7 | HAM-D |
| ^519^ | PLB | 15 | 32.9 | 5 | HAM-D |
| ^549^ | CUR | 20 | 18.3 | 6.3 | HAM-D |
| ^549^ | FLUO | 20 | 21 | 6.3 | HAM-D |
| ^549^ | CUR+FLUO | 20 | 21.9 | 6.3 | HAM-D |
| ^506^ | WS5572 | 37 | 19.7 | 3.4 | HAM-D |
| ^506^ | PLB | 35 | 20.12 | 2.6 | HAM-D |
| ^601^ | YUE+FLUO | 10 | 23.80 | 1.51 | HAM-D |
| ^601^ | FLUO+PLB | 8 | 25.38 | 1.45 | HAM-D |
| ^581^ | CTP+FOL | 45 | 34.24 | 9.01 | BDI-II |
| ^581^ | CTP + PLB | 45 | 35.82 | 9.57 | BDI-II |
| ^567^ | WS5570 | 122 | 25.5 | 2.7 | HAM-D |
| ^567^ | PAR | 122 | 25.5 | 2.9 | HAM-D |
| ^507^ | CUR | 33 | 21086 | 4902 | MADRS |
| ^507^ | PLB | 32 | 21258 | 3784 | MADRS |
| ^550^ | SAMe+TAU | 55 | 19452 | 4.53 | MADRS |
| ^550^ | PLB+TAU | 52 | 19366 | 4.76 | MADRS |
| ^582^ | THI+FLUO | 25 | 31.40 | 4.1 | HAM-D |
| ^582^ | PLB+FLUO | 26 | 32.77 | 4.22 | HAM-D |
| ^466^ | ESC+FOL | 20 | 24.44 | 6.3 | MADRS |
| ^466^ | ESC | 15 | 24.87 | 6.3 | MADRS |
| Palhano-Fontes F. et al., ^600^ | AYA | 17 | 24.07 | 5.34 | HAM-D |
| Palhano-Fontes F. et al., ^600^ | PLB | 18 | 19.73 | 4.59 | HAM-D |
| ^541^ | DHA+EPA | 14 | 8.0 | 5.6 | HAM-D |
| ^541^ | PLB | 9 | 8.4 | 6.4 | HAM-D |
| ^492^ | SER+PLB | 32 | 23.2 | 2.6 | HAM-D |
| ^492^ | RHO+SER | 66 | 23.3 | 1.8 | HAM-D |
| ^487^ | SCU | 42 | 17.3 | 3 | HAM-D |
| ^487^ | SAFF+SCU | 48 | 16.8 | 3.1 | HAM-D |
| ^487^ | SAFF | 43 | 16.5 | 3.3 | HAM-D |
| ^487^ | PLB | 47 | 16.2 | 3.3 | HAM-D |
| ^296^ | EPA+TAU | 10 | 24.0 | 2.9 | HAM-D |
| ^296^ | PLB+TAU | 10 | 22.3 | 2.8 | HAM-D |
| ^477^ | SAMe | 30 | 45.1 | 6.7 | HAM-D |
| ^477^ | PLB | 30 | 42.4 | 5.1 | HAM-D |
| ^594^ | CUR+ESC | 50 | 14.06 | 3.01 | HAM-D |
| ^594^ | PLB+ESC | 50 | 14.28 | 3.02 | HAM-D |
| ^586^ | CIT+CTP | 25 | 24.76 | 4.4 | HAM-D |
| ^586^ | PLB+CTP | 25 | 24.40 | 4.24 | HAM-D |
| ^463^ | EACS | 25 | 17.33 | 5.51 | HAM-D |
| ^463^ | CTP | 25 | 18.86 | 4.54 | HAM-D |
| ^474^ | LI-160 | 15 | 21.3 | 3.2 | HAM-D |
| ^474^ | SER | 15 | 21.7 | 2.7 | HAM-D |
| ^599^ | FOL+vitB12+vitB6+CTP | 77 | 19.71 | 6.3 | MADRS |
| ^599^ | CTP+PLB | 76 | 20.57 | 6.3 | MADRS |
| ^561^ | EOPF | 60 | 26.9 | 4.8 | BDI |
| ^561^ | PLB | 60 | 19.7 | 5.7 | BDI |
| ^480^ | SAFF | 20 | 17.3 | 6.3 | HAM-D |
| ^480^ | FLUO | 20 | 16.7 | 6.3 | HAM-D |
| ^429^ | LI-160 | 83 | 20.6 | 2.1 | HAM-D |
| ^429^ | AMI | 73 | 20.8 | 2.3 | HAM-D |
| ^571^ | INU | 31 | 13.06 | 4.82 | HAM-D |
| ^571^ | PLB | 31 | 12.87 | 4.81 | HAM-D |
| ^520^ | SHU | 194 | 11.36 | 3.2 | HAM-D |
| ^520^ | PLB | 191 | 11.36 | 3.07 | HAM-D |
| ^573^ | ZNC+TAU | 30 | 29.77 | 4.28 | HAM-D |
| ^573^ | TAU | 30 | 29.40 | 4.56 | HAM-D |
| ^604^ | INO | 13 | 2.77 | 0.6 | HAM-D |
| ^604^ | PLB | 14 | 2.67 | 0.6 | HAM-D |
| ^483^ | LI-160 | 113 | 23.1 | 2.7 | HAM-D |
| ^483^ | SER | 111 | 22.5 | 2.5 | HAM-D |
| ^483^ | PLB | 116 | 22.7 | 2.7 | HAM-D |
| ^566^ | MG | 45 | 21.10 | 3.04 | HAM-D |
| ^566^ | PLB | 45 | 21.10 | 3.83 | HAM-D |
| ^589^ | vitD3+TAU | 23 | 19.4 | 4 | HAM-D |
| ^589^ | PROB+TAU | 23 | 17.44 | 3.1 | HAM-D |
| ^580^ | CTP + PLB | 30 | 26.35 | 9.89 | BDI |
| ^580^ | CTP+OMEGA3 | 30 | 25.89 | 10.56 | BDI |
| ^534^ | ZNC+TAU | 10 | 32.11 | 5.67 | HAM-D |
| ^534^ | PLB+TAU | 10 | 33475 | 5985 | HAM-D |
| ^495^ | SAFF | 30 | 17.20 | 1.4 | HAM-D |
| ^495^ | CTP | 30 | 17.50 | 0.63 | HAM-D |
| ^504^ | EPA | 16 | 29.94 | 4.9 | HAM-D |
| ^504^ | FLUO | 16 | 29.31 | 6.5 | HAM-D |
| ^504^ | EPA+FLUO | 16 | 31.63 | 5.8 | HAM-D |
| ^456^ | SAFF | 25 | 21.12 | 2.35 | HAM-D |
| ^456^ | SER | 25 | 21.40 | 2.86 | HAM-D |
| ^576^ | ZE | 157 | 22.4 | 3.4 | HAM-D |
| ^576^ | TAU | 167 | 22.1 | 2.9 | HAM-D |
| ^509^ | SAFF | 34 | 16.53 | 1.48 | HAM-D |
| ^509^ | FLUO | 34 | 16.65 | 1.12 | HAM-D |
| ^470^ | NAC+TAU | 127 | 19.4 | 3.6 | MADRS |
| ^470^ | PLB+TAU | 125 | 19.4 | 3.6 | MADRS |
| ^605^ | SUL+CLO | 43 | 24956 | 5246 | HAM-D |
| ^605^ | PLB+CLO | 47 | 25386 | 5.59 | HAM-D |
| ^472^ | LI-160 | 54 | 24.9 | 4.8 | HAM-D |
| ^472^ | FLUO | 54 | 23.8 | 3.6 | HAM-D |
| ^472^ | PLB | 55 | 25.2 | 4.4 | HAM-D |
| ^467^ | SJW | 35 | 20.0 | 3.2 | HAM-D |
| ^467^ | FLUO | 35 | 20.7 | 2.9 | HAM-D |
| ^554^ | ZE117 | 126 | 19.5 | 3.1 | HAM-D |
| ^554^ | FLUO + PLB | 114 | 19.5 | 2.4 | HAM-D |
| ^584^ | FOL+FLUO | 62 | 26.8 | 5.0 | HAM-D |
| ^584^ | PLB+FLUO | 65 | 26.6 | 4.0 | HAM-D |
| ^496^ | SYN | 20 | 22.90 | 6.3 | HAM-D |
| ^496^ | PLB | 20 | 22.55 | 6.3 | HAM-D |
| ^538^ | SAMe+TAU | 39 | 19.1 | 3.1 | HAM-D |
| ^538^ | PLB+TAU | 34 | 19.9 | 3.0 | HAM-D |
| ^493^ | LI-160 | 43 | 18.3 | 5.3 | HAM-D |
| ^493^ | PLB | 55 | 18.1 | 4.5 | HAM-D |
| ^557^ | SER+LAV+DRA | 35 | 23.4 | 10584 | BDI |
| ^557^ | SER+PLB | 35 | 21.7 | 1.72 | BDI |
| ^568^ | SER+CUR | 20 | 39.20 | 6.49 | BDI-II |
| ^568^ | SER+PLB | 20 | 39.75 | 6.32 | BDI-II |
| ^510^ | WS5570 | 243 | 22.9 | 3.1 | HAM-D |
| ^510^ | PLB | 81 | 22.5 | 3.4 | HAM-D |
| ^546^ | EACS+SJW | 23 | 27.22 | 2.94 | HAM-D |
| ^546^ | FLUO | 28 | 27.64 | 2.57 | HAM-D |
| ^540^ | OMEGA3+TAU | 18 | 21.50 | 1.63 | HAM-D |
| ^540^ | PLB+TAU | 17 | 20.31 | 1.24 | HAM-D |
| ^479^ | EPA+DHA | 19 | 6.16 | 3.91 | HAM-D |
| ^479^ | PLB | 20 | 7.8 | 6.28 | HAM-D |
| ^516^ | OMB | 28 | 15.14 | 6.25 | HAM-D |
| ^516^ | PLB | 29 | 14.45 | 4.44 | HAM-D |
| ^591^ | FLUO+SAFF | 20 | 21.61 | 1029 | BDI |
| ^591^ | FLUO + PLB | 20 | 171265 | 1.14 | BDI |
| ^514^ | vitD3+FLUO | 20 | 29.4 | 5.23 | HAM-D |
| ^514^ | FLUO | 20 | 30.2 | 5.83 | HAM-D |
| ^556^ | vitD | 20 | 24.97 | 9.66 | BDI |
| ^556^ | PLB | 20 | 28.72 | 11.34 | BDI |
| ^518^ | WS5573 | 49 | 20.3 | 2.7 | HAM-D |
| ^518^ | WS5572 | 49 | 20.9 | 3.1 | HAM-D |
| ^518^ | PLB | 49 | 21.2 | 3.3 | HAM-D |
| ^478^ | OMEGA3 | 30 | 19 | 4.21 | HAM-D |
| ^478^ | PLB | 29 | 19.17 | 3.58 | HAM-D |
| ^532^ | ZPG | 89 | 13.00 | 4.24 | HAM-D |
| ^532^ | PLB | 87 | 13.48 | 3.97 | HAM-D |
| ^469^ | CUR | 20 | 32.7 | 4.9 | HAM-D |
| ^469^ | PLB | 20 | 32.7 | 5.9 | HAM-D |
| ^511^ | WS5570 | 68 | 22.8 | 3.3 | HAM-D |
| ^511^ | PLB | 24 | 23.6 | 4.2 | HAM-D |
| ^473^ | EPA | 13 | 19968 | 7052 | MADRS |
| ^473^ | PLB | 12 | 20054 | 7482 | MADRS |
| ^272^ | EPA | 21 | 15.9 | 2 | HAM-D |
| ^272^ | DHA | 20 | 15.7 | 2.4 | HAM-D |
| ^272^ | PLB | 21 | 15.5 | 2.3 | HAM-D |
| ^552^ | SAMe | 25 | 16614 | 1.7 | MADRS |
| ^552^ | PLB | 24 | 16442 | 2.6 | MADRS |
| ^468^ | SAMe | 9 | 40.0 | 7.3 | HAM-D |
| ^468^ | IMI | 9 | 39.0 | 7.5 | HAM-D |
| ^539^ | LMF | 36 | 18.8 | 4.2 | HAM-D |
| ^539^ | PLB | 112 | 19.9 | 4.8 | HAM-D |
| ^539^ | LMF | 19 | 16.2 | 3.4 | HAM-D |
| ^539^ | PLB | 56 | 16.8 | 4.7 | HAM-D |
| ^524^ | CQ10+TAU | 36 | 25.62 | 4.85 | MADRS |
| ^524^ | PLB+TAU | 33 | 23.43 | 4.84 | MADRS |
| ^551^ | NUT | 81 | 17.99 | 4.03 | MADRS |
| ^551^ | PLB | 77 | 18506 | 4.49 | MADRS |
| ^577^ | EPA+DHA | 30 | 19.17 | 5.14 | HAM-D |
| ^577^ | PLB | 30 | 20.67 | 6.43 | HAM-D |
| ^578^ | vitD3 | 56 | 24.34 | 1.37 | BDI-II |
| ^578^ | PLB | 64 | 22975 | 1.11 | BDI-II |
| ^597^ | SIL | 149 | 17388 | 3.01 | MADRS |
| ^597^ | SER | 148 | 17646 | 2924 | MADRS |
| ^597^ | PLB | 138 | 17474 | 2924 | HAM-D |
| ^515^ | NEP | 33 | 27.42 | 8.77 | BDI-II |
| ^515^ | SER | 33 | 24.51 | 8.55 | BDI-II |
| ^332^ | EPA+DHA | 13 | 19.7 | 4.8 | HAM-D |
| ^332^ | PLB | 13 | 9.0 | 3.5 | HAM-D |
| ^490^ | EPA+DHA | 28 | 18.7 | 3.3 | HAM-D |
| ^490^ | PLB | 23 | 18.5 | 3.7 | HAM-D |
| ^564^ | EPA+DHA | 21 | 13174 | 4558 | MADRS |
| ^564^ | PLB | 18 | 13776 | 3.44 | MADRS |
| ^387^ | EPA+DHA | 18 | 22.5 | 3.9 | HAM-D |
| ^387^ | PLB | 18 | 22.1 | 3.9 | HAM-D |
| ^565^ | EPA+DHA | 12 | 22.1 | 5 | HAM-D |
| ^565^ | PLB | 10 | 21.8 | 3.9 | HAM-D |
| ^595^ | EPA+DHA+VEN | 21 | 29.50 | 6.3 | HAM-D |
| ^595^ | PLB+VEN | 27 | 29.97 | 6.3 | HAM-D |
| ^505^ | SAMe | 9 | 26.6 | 5.5 | HAM-D |
| ^505^ | PLB | 6 | 31.0 | 8.5 | HAM-D |
| ^498^ | GGBT | 82 | 14.96 | 1.84 | HAM-D |
| ^498^ | PLB | 82 | 15.18 | 1.98 | HAM-D |
| ^537^ | SAMe | 146 | 24.3 | 4.0 | HAM-D |
| ^537^ | IMI | 147 | 26.0 | 4.5 | HAM-D |
| ^529^ | SAMe | 64 | 18.98 | 5.09 | HAM-D |
| ^529^ | ESC | 65 | 19.25 | 4.88 | HAM-D |
| ^529^ | PLB | 60 | 19.43 | 4.07 | HAM-D |
| ^525^ | RHO | 20 | 19.0 | 4.2 | HAM-D |
| ^525^ | SER | 19 | 20.8 | 4.1 | HAM-D |
| ^525^ | PLB | 18 | 19.4 | 2.6 | HAM-D |
| ^555^ | SRN+TAU | 22 | 21.2 | 3.8 | HAM-D |
| ^555^ | PLB+TAU | 22 | 21.7 | 3.5 | HAM-D |

Most of the included studies assessed the mean change in Depression symptoms using the Hamilton Depression Rating Scale (HAM-D). Some of the latter studies employed the Montgomery-Åsberg Depression Rating Scale (MADRS) or the Beck Depression Inventory (BDI), either the Type I (BDI-I) or the Type II (BDI-II) version. To analyze our primary outcome (efficacy), we computed mean changes as SMDs, pooling values coming from different scales. In transitivity analyses, we needed to harmonize the baseline scores to pool them together and verify whether the baseline depression score could serve as an effect modifier. Note: For transitivity analyses, we could not pool SMDs from different yet equivalent rating scales, as the primary meta-analytic synthesis requires raw scores to be plotted against computed effect sizes (in this instance, the SMD of efficacy). Thus, we converted MADRS baseline ^609^ and BDI-I and BDI-II scores ^610^ to their HAM-D approximate equivalents, using the conversion tables provided by the respective sources.

**References for e-table 31**

(2016). Antidepressant Effects of Ayahuasca: a Randomized Placebo Controlled Trial in Treatment Resistant Depression. clinicaltrials.gov.

(2016). Double Blind, Placebo Controlled Pilot Study to Evaluate the Use of Selenium as an Augmentation Treatment for Sertraline Resistant Major Depression. clinicaltrials.gov.

(2016). The Efficacy, Safety, and Tolerability of Probiotics on the Mood and Cognition of Depressed Patients. clinicaltrials.gov.

(2016). A Randomized, Double-blind, Placebo-controlled Trial of 5-hydroxytryptophan and Creatine for SSRI or SNRI Augmentation in Treatment Resistant Depression Associated With Hypobaric Hypoxia in Females. clinicaltrials.gov.

(2017). The Chinese Herb SZ-05 for the Treatment of Depression and Anxiety Disorders; Efficiency, Safety and Biological Correlates. clinicaltrials.gov.

(2017). Metformin, Vitamin D, and Depression in Polycystic Ovary Syndrome (PCOS) Trial: The MINDD Trial. clinicaltrials.gov.

(2017). Nutritional Intervention With Mediterranean Diet in the Prevention of Recurrence of Depression. clinicaltrials.gov.

(2018). Effect of Vitamin D Administration Along With Selective Serotonin Reuptake Inhibitors (SSRIs) in Patients With Major Depressive Disorder (MDD). clinicaltrials.gov.

(2018). Evaluation of Vitamin D Supplementation Effects on Serum 25(OH)D3, PTH, Pro-inflammatory Biomarkers and Neurotransmitters Involved in Depression, and Depression Status in Depressive Patients. clinicaltrials.gov.

(2019). DOUBLE BLIND, RANDOMIZED, PLACEBO CONTROLLED STUDY TO EVALUATE THE EFFICACY OF SAMEUP IN SUBJECTS CHARACTERIZED BY MILD TO MODERATE DEPRESSION SYMPTOMS. clinicaltrials.gov.

(2019). Evaluation of Efficacy of Lycium Barbarum Polysaccharide in Patients With Major Depressive Disorder: a Randomized,Placebo-controled Trial. clinicaltrials.gov.

(2019). Evaluation of Efficacy of Lycium Barbarum Polysaccharide in People With Subthreshold Depression: a Randomized, Placebo-controlled Trial. clinicaltrials.gov.

(2019). Multistrain Probiotic Product (De Simone Formulation) Reduces Depression and Anxiety Scores: a Randomized Pilot Study in Patients With Ulcerative Colitis. clinicaltrials.gov.

(2020). Double-blind Placebo-controlled Clinical Trial of Lactobacillus Helveticus as an add-on Strategy for the Treatment of Major Depression. clinicaltrials.gov.

(2020). Effect of Probiotic on Chinese Patients With Depression and Constipated Comorbidity. clinicaltrials.gov.

(2020). Effect of Probiotic on Depressive Symptom in Chinese Patients. clinicaltrials.gov.

(2020). Effect of Zinc Supplementation on Depression in Selective Serotonin Reuptake Inhibitors-treated Major Depressive Disorder Patients. clinicaltrials.gov.

(2020). A Two-Year Observational Follow-up Study of Subjects With Major Depressive Disorder Following a Randomized, Double-Blind Single-Dose of Psilocybin or Niacin-Control. clinicaltrials.gov.

(2021). Effects of Cholecalciferol on Depressive Symptoms in Type 2 Diabetes Mellitus Patients: Study of Neurotrophin-3, Serotonin, and C-Peptide. clinicaltrials.gov.

(2021). Evaluation of Efficacy and Safety of add-on Sarcosine in Patients With Major Depressive Disorder: A Randomized Controlled Trial. clinicaltrials.gov.

(2021). Omega-3 Unsaturated Fatty Acid Improves Cognitive Function in Patients With Depression: a 12 Week Randomized, Double-blind, Placebo-controlled Study. clinicaltrials.gov.

(2021). Use of Sumor in the Antidepressant Therapy of Subjects With Major Depression: a Randomized Placebo-controlled Clinical Trial. clinicaltrials.gov.

(2022). Effect of Two Probiotic Formulations on Mental Health and Mood Biomarkers in Adults With Depressive Symptoms: A Pilot Randomized Placebo-controlled Trial. clinicaltrials.gov.

(2022). The Efficacy of Probiotic Compared With Placebo and Acceptance and Commitment Therapy for the Treatment of Major Depressive Disorder Among Adult Females. clinicaltrials.gov.

(2022). Multicenter, Randomized, Double-blind, Parallel-group Controlled Nutritional Intervention Study to Evaluate the Effect of a Combination of Probiotic Strain on Postpartum Depression. clinicaltrials.gov.

(2023). Blueberries, Gut Microbiota, and Metabolites in Depressed Older Adults - A Pilot Study. clinicaltrials.gov.

(2023). Comparative Efficacy of Antidepressants With L-methylfolate, B12, and Magnesium in Depressive Disorders. clinicaltrials.gov.

(2023). The Effect of Peripartum Magnesium Sulfate Use on the Occurrence of Postpartum Depression. clinicaltrials.gov.

(2023). Specialized Pro-resolving Lipid Mediators and Treatment Resistant Depression. clinicaltrials.gov.

(2024). The Effect of Stingless Bee Honey on BDNF, Oxidative Stress, Depressive Symptoms and Verbal Learning and Memory in Major Depressive Disorders: A Randomized Clinical Trial. clinicaltrials.gov.

(2024). Effectiveness and Safety of Probiotics, BC99 in Relieving Anxiety and Depression in Adults. clinicaltrials.gov.

(2024). Leucine as a Probe of Kynurenine-Induced Glutamate and Neural Circuit Dysfunction in Midlife Depression. clinicaltrials.gov.

12070867, P. (2002). "St. John's Wort for depression. 900 mg per day should an effective dose." MMW Fortschritte der Medizin **Suppl 2**: 87.

12085821, P. (2002). "Study shows St. John's wort ineffective for major depression." FDA consumer **36**(3): 8.

20786190, I. (2005). "St John's wort = SSRI for moderate-severe depression." South African Family Practice **47**(4): 18.

Açik, M., F. P. Çakiroğlu, A. Tekin and A. Egeli (2025). "The effect of fish oil versus krill oil intervention on clinical symptoms and cardiometabolic risk factors in patients with major depressive disorder: A randomized placebo-controlled double-blind trial." Journal of Affective Disorders **380**: 171-180.

Actrn (2017). "A micronutrient intervention for adults experiencing symptoms of anxiety and depression."

Adams, P. W., D. P. Rose, J. Folkard, V. Wynn, M. Seed and R. Strong (1973). "Effect of pyridoxine hydrochloride (vitamin B 6 ) upon depression associated with oral contraception." Lancet **1**(7809): 899-904.

Afsharfar, M., M. Shahraki, M. Shakiba, O. Asbaghi and A. Dashipour (2021). "The effects of magnesium supplementation on serum level of brain derived neurotrophic factor (BDNF) and depression status in patients with depression." Clinical Nutrition ESPEN **42**: 381-386.

Afzali, A., Z. Vakili, S. Goli, H. Bagheri, S. Mirhosseini and H. Ebrahimi (2021). "A Randomized Clinical Trial of the Effect of Zinc Supplement on Depression and Anxiety in the Elderly." Open Public Health Journal **14**(1): 537-544.

Ahmad, S., A. Khan, H. Rafiq, P. Tikmani, H. Mesiya and F. Amin (2023). "Co-administration of saffron and chamomile: to determine the efficacy as an adjuvant therapy for mild to moderate depression in human subjects. A pilot randomized clinical trial." Journal of the Pakistan Medical Association **73**(6): 1245-1250.

Ahmadpanah, M., F. Ramezanshams, A. Ghaleiha, S. Akhondzadeh, D. Sadeghi Bahmani and S. Brand (2019). "Crocus Sativus L. (saffron) versus sertraline on symptoms of depression among older people with major depressive disorders–a double-blind, randomized intervention study." Psychiatry Research **282**.

Akhondzadeh Basti, A., S. A. Choreishi, A. A. Noorbala, S. H. Akhondzadeh and S. Rezazadeh (2008). "Petal and stigma of Crocus sativus L. in the treatment of depression: A pilot double - blind randomized trial." Journal of Medicinal Plants **7**(SUPPL. 4): 29-36.

Akhondzadeh Basti, A., E. Moshiri, A. A. Noorbala, A. H. Jamshidi, S. H. Abbasi and S. Akhondzadeh (2007). "Comparison of petal of Crocus sativus L. and fluoxetine in the treatment of depressed outpatients: A pilot double-blind randomized trial." Progress in Neuro-Psychopharmacology and Biological Psychiatry **31**(2): 439-442.

Akhondzadeh, S., H. Fallah-Pour, K. Afkham, A. H. Jamshidi and F. Khalighi-Cigaroudi (2004). "Comparison of Crocus sativus L. and imipramine in the treatment of mild to moderate depression: a pilot double-blind randomized trial." **4**.

Akhondzadeh, S., H. Fallah-Pour, K. Afkham, A. H. Jamshidi and F. Khalighi-Cigaroudi (2004). "Comparison of Crocus sativus L. and imipramine in the treatment of mild to moderate depression: A pilot double-blind randomized trial [ISRCTN45683816]." BMC Complementary and Alternative Medicine **4**.

Akhondzadeh, S., H. Fallah-Pour, K. Afkham, A. H. Jamshidi, F. Khalighi-Cigaroudi and L. G. Miller (2005). "A comparative trial of Crocus sativus L. (saffron) and imipramine in mild to moderate depression." Focus on Alternative and Complementary Therapies **10**(1): 22-23.

Akhondzadeh, S., S. A. Mostafavi, S. A. Keshavarz, M. R. Mohammadi and M. Chamari (2022). "Melatonin Effects in Women With Comorbidities of Overweight, Depression, and Sleep Disturbance: A Randomized Placebo Controlled Clinical Trial." Sleep Medicine Research **13**(1): 22-30.

Akhondzadeh, S., S. A. Mostafavi, S. A. Keshavarz, M. R. Mohammadi, S. Hosseini and M. R. Eshraghian (2020). "A placebo controlled randomized clinical trial of Crocus sativus L. (saffron) on depression and food craving among overweight women with mild to moderate depression." Journal of Clinical Pharmacy and Therapeutics **45**(1): 134-143.

Akkasheh, G., Z. Kashani-Poor, M. Tajabadi-Ebrahimi, P. Jafari, H. Akbari, M. Taghizadeh, M. R. Memarzadeh, Z. Asemi and A. Esmaillzadeh (2016). "Clinical and metabolic response to probiotic administration in patients with major depressive disorder: A randomized, double-blind, placebo-controlled trial." Nutrition **32**(3): 315-320.

Alavi, N. M., S. Khademalhoseini, Z. Vakili and F. Assarian (2019). "Effect of vitamin D supplementation on depression in elderly patients: A randomized clinical trial." Clinical Nutrition **38**(5): 2065-2070.

Alcocer-Gómez, E., J. A. Sánchez-Alcázar and M. D. Cordero (2014). "Coenzyme Q10 regulates serotonin levels and depressive symptoms in fibromyalgia patients: Results of a small clinical trial." Journal of Clinical Psychopharmacology **34**(2): 277-278.

Almeida, O. P., A. H. Ford, V. Hirani, V. Singh, F. M. VanBockxmeer, K. McCaul and L. Flicker (2014). "B vitamins to enhance treatment response to antidepressants in middle-aged and older adults: Results from the B-VITAGE randomised, double-blind, placebo-controlled trial." British Journal of Psychiatry **205**(6): 450-457.

Amini, L., O. Yaghini, M. Ghazavi and N. Aslani (2021). "L-carnitine versus Propranolol for pediatric migraine prophylaxis." Iran J Child Neurol **15**(2): 77-86.

Amminger, G. P., B. Nelson, H. P. Yuen, C. Markulev, M. R. Schafer, M. Schlogelhofer, N. Mossaheb, S. Smesny, I. Hickie, G. Berger and et al. (2018). "Neurapro revisited: increases in long-chain omega-3 fatty acids improve functional and symptomatic outcomes in ultrahigh risk patients." **44**: S228

C223 - EMBASE 621901637.

Amsterdam, J. D., Q. S. Li, S. X. Xie and J. J. Mao (2020). "Putative Antidepressant Effect of Chamomile (Matricaria chamomilla L.) Oral Extract in Subjects with Comorbid Generalized Anxiety Disorder and Depression." Journal of Alternative and Complementary Medicine **26**(9): 813-819.

Andrade, C. (2021). "N-Acetylcysteine Augmentation for Patients With Major Depressive Disorder and Bipolar Depression." Journal of Clinical Psychiatry **82**(1 C7 - 21f13891).

Andreeva, V. A., P. Galan, M. Torrès, C. Julia, S. Hercberg and E. Kesse-Guyot (2012). "Supplementation with B vitamins or N-3 fatty acids and depressive symptoms in cardiovascular disease survivors: Ancillary findings from the SUpplementation with FOLate, vitamins B-6 and B-12 and/or OMega-3 fatty acids (SU.FOL.OM3) randomized trial." American Journal of Clinical Nutrition **96**(1): 208-214.

Antypa, N., A. H. M. Smelt, A. Strengholt and A. J. W. Van Der Does (2012). "Effects of omega-3 fatty acid supplementation on mood and emotional information processing in recovered depressed individuals." Journal of Psychopharmacology **26**(5): 738-743.

Anushiravani, M., A. A. Manteghi, A. Taghipur and M. Eslami (2019). "Comparing Effectiveness of a Combined Herbal Drug Based on Echium Amoenum with Citalopram in the Treatment of Major Depressive Disorder." Curr Drug Discov Technol **16**(2): 232-238.

Appleton, K. M., P. D. Voyias, H. M. Sallis, S. Dawson, A. R. Ness, R. Churchill and R. Perry (2021). "Omega-3 fatty acids for depression in adults." Cochrane Database of Systematic Reviews **2021**(11 C7 - CD004692).

Araj-Khodaei, M., A. A. Noorbala, R. Yarani, F. Emadi, E. Emaratkar, S. Faghihzadeh, Z. Parsian, F. Alijaniha, M. Kamalinejad and M. Naseri (2020). "A double-blind, randomized pilot study for comparison of Melissa officinalis L. And Lavandula angustifolia Mill. And Fluoxetine for the treatment of depression." BMC Complementary Medicine and Therapies **20**(1).

Arnold, L. E., A. S. Young, M. A. Belury, R. M. Cole, B. Gracious, A. M. Seidenfeld, H. Wolfson and M. A. Fristad (2017). "Omega-3 fatty acid plasma levels before and after supplementation: Correlations with mood and clinical outcomes in the omega-3 and therapy studies." Journal of Child and Adolescent Psychopharmacology **27**(3): 223-233.

Arnold, R., J. Murphy-Smith, C. H. Ng, D. Mischoulon, G. J. Byrne, C. A. Bousman, C. Stough, M. Berk and J. Sarris (2024). "Predictors of the placebo response in a nutraceutical randomized controlled trial for depression." Journal of Integrative Medicine **22**(1): 46-53.

Asadi, S., M. S. Gholami, F. Siassi, M. Qorbani and G. Sotoudeh (2019). "Beneficial effects of nano-curcumin supplement on depression and anxiety in diabetic patients with peripheral neuropathy: A randomized, double-blind, placebo-controlled clinical trial." Phytotherapy research : PTR.

Astorg, P., A. Couthouis, G. P. De Courcy, S. Bertrais, N. Arnault, P. Meneton, P. Galan and S. Hercberg (2008). "Association of folate intake with the occurrence of depressive episodes in middle-aged French men and women." British Journal of Nutrition **100**(1): 183-187.

Augusto da Silva Meleiro, A. M. (2000). "Treatment of mild and moderate depression: Evaluation of the effectiveness and tolerability of LI 160 S Hypericum perforatum extract." Revista Brasileira de Medicina **57**(11): 1313-1323.

Badrasawi, M. M., S. Shahar, Z. Abd Manaf and H. Haron (2013). "Effect of Talbinah food consumption on depressive symptoms among elderly individuals in long term care facilities, randomized clinical trial." **8**: 279‐285

C273 - PUBMED 23493965.

Bahrami, A., F. Nikoomanesh, H. Dehghani, H. Aramjoo and G. A. Ferns (2024). "Curcumin Ameliorates Depression, and Anxiety in Patients with Premenstrual Syndrome and Dysmenorrhea: A Triple-Blinded Placebo-Controlled Trial." Journal of Advances in Medical and Biomedical Research **32**(152): 174-184.

Baião, R., L. P. Capitão, C. Higgins, M. Browning, C. J. Harmer and P. W. J. Burnet (2023). "Multispecies probiotic administration reduces emotional salience and improves mood in subjects with moderate depression: a randomised, double-blind, placebo-controlled study." Psychological medicine **53**(8): 3437-3447.

Barragán-Rodríguez, L., M. Rodríguez-Morán and F. Guerrero-Romero (2008). "Efficacy and safety of oral magnesium supplementation in the treatment of depression in the elderly with type 2 diabetes: A randomized, equivalent trial." Magnesium Research **21**(4): 218-223.

Bartova, L., M. Dold, H. P. Volz, E. Seifritz, H. J. Möller and S. Kasper (2023). "Beneficial effects of Silexan on co-occurring depressive symptoms in patients with subthreshold anxiety and anxiety disorders: randomized, placebo-controlled trials revisited." European Archives of Psychiatry and Clinical Neuroscience **273**(1): 51-63.

Basoglu, C., M. A. Ates, A. Algul, O. M. Ipcioglu, O. Gecici, O. Yilmaz, U. B. Semiz, S. Ebrinc, M. Gulsun, O. Ozcan and et al. (2009). "Adjuvant folate with escitalopram treatment and homocystein, folate, vitamin B-12 levels in patients with major depressive disorder." **19**(2): 135‐142

C133 - EMBASE 354832073.

Bazinet, R. P., A. H. Metherel, C. T. Chen, S. R. Shaikh, A. Nadjar, C. Joffre and S. Layé (2020). "Brain eicosapentaenoic acid metabolism as a lead for novel therapeutics in major depression." Brain, Behavior, and Immunity **85**: 21-28.

Bazrafshan, M. R., M. Jokar, N. Shokrpour and H. Delam (2020). "The effect of lavender herbal tea on the anxiety and depression of the elderly: A randomized clinical trial." Complementary Therapies in Medicine **50**.

Behnke, K., G. S. Jensen, H. J. Graubaum and J. Gruenwald (2002). "Hypericum perforatum versus fluoxetine in the treatment of mild to moderate depression." Advances in Therapy **19**(1): 43-52.

Bell, K. M., L. Plon, W. E. Bunney Jr and S. G. Potkin (1988). "S-adenosylmethionine treatment of depression: A controlled clinical trial." American Journal of Psychiatry **145**(9): 1110-1114.

Bergman, J., C. Miodownik, Y. Bersudsky, S. Sokolik, P. P. Lerner, A. Kreinin, J. Polakiewicz and V. Lerner (2013). "Curcumin as an add-on to antidepressive treatment: A randomized, double-blind, placebo-controlled, pilot clinical study." Clinical Neuropharmacology **36**(3): 73-77.

Berk, M., O. M. Dean, S. M. Cotton, S. Jeavons, M. Tanious, K. Kohlmann, K. Hewitt, K. Moss, C. Allwang, I. Schapkaitz, J. Robbins, H. Cobb, F. Ng, S. Dodd, A. I. Bush and G. S. Malhi (2014). "The efficacy of adjunctive N-acetylcysteine in major depressive disorder: A double-blind, randomized, placebo-controlled trial." Journal of Clinical Psychiatry **75**(6): 628-636.

Bidzan-Wiącek, M., M. Tomczyk, M. Błażek, A. Mika and J. Antosiewicz (2024). "No Effects of Omega-3 Supplementation on Kynurenine Pathway, Inflammation, Depressive Symptoms, and Stress Response in Males: A Placebo-Controlled Trial." Nutrients **16**(21 C7 - 3744).

Bitarafan, S., A. Saboor-Yaraghi, M. A. Sahraian, D. Soltani, S. Nafissi, M. Togha, N. Beladi Moghadam, T. Roostaei, N. Mohammadzadeh Honarvar and M. H. Harirchian (2016). "Effect of Vitamin A Supplementation on fatigue and depression in Multiple Sclerosis patients: a Double-Blind Placebo-Controlled Clinical Trial." **15**(1): 13‐19

C13 - PUBMED 26996107,EMBASE 608134953.

Bjerkenstedt, L., G. V. Edman, R. G. Alken and M. Mannel (2005). "Hypericum extract LI 160 and fluoxetine in mild to moderate depression: A randomized, placebo-controlled multi-center study in outpatients." European Archives of Psychiatry and Clinical Neuroscience **255**(1): 40-47.

Blampied, M., J. M. Tylianakis, C. Bell, C. Gilbert and J. J. Rucklidge (2023). "Efficacy and safety of a vitamin-mineral intervention for symptoms of anxiety and depression in adults: A randomised placebo-controlled trial “NoMAD”." Journal of Affective Disorders **339**: 954-964.

Borja-Hart, N. L. and J. Marino (2010). "Role of omega-3 fatty acids for prevention or treatment of perinatal depression." Pharmacotherapy **30**(2): 210-216.

Bot, M., I. A. Brouwer, M. Roca, E. Kohls, B. W. J. H. Penninx, E. Watkins, G. Van Grootheest, M. Cabout, U. Hegerl, M. Gili, M. Owens and M. Visser (2019). "Effect of Multinutrient Supplementation and Food-Related Behavioral Activation Therapy on Prevention of Major Depressive Disorder among Overweight or Obese Adults with Subsyndromal Depressive Symptoms: The MooDFOOD Randomized Clinical Trial." JAMA - Journal of the American Medical Association **321**(9): 858-868.

Bot, M., F. Pouwer, J. Assies, E. H. Jansen, M. Diamant, F. J. Snoek, A. T. Beekman and P. de Jonge (2010). "Eicosapentaenoic acid as an add-on to antidepressant medication for co-morbid major depression in patients with diabetes mellitus: a randomized, double-blind placebo-controlled study." J Affect Disord **126**(1-2): 282-286.

Bradley, H. A., E. Moltchanova, R. T. Mulder, L. Dixon, J. Henderson and J. J. Rucklidge (2024). "Efficacy and safety of a mineral and vitamin treatment on symptoms of antenatal depression: 12-week fully blinded randomised placebo-controlled trial (NUTRIMUM)." BJPsych Open **10**(4 C7 - e119).

Bradley, H. A., R. T. Mulder, J. M. T. Henderson, L. Dixon, E. Moltchanova and J. J. Rucklidge (2022). "A NUTRITIONAL APPROACH TO TREATING SYMPTOMS OF ANTENATAL DEPRESSION AND ANXIETY. THE NUTRIMUM TRIAL: A DOUBLE-BLIND, RANDOMISED CONTROLLED TRIAL." Journal of Paediatrics and Child Health **58**(SUPPL 2): 23.

Brattström, A. (2009). "Long-term effects of St. John's wort (Hypericum perforatum) treatment: A 1-year safety study in mild to moderate depression." Phytomedicine **16**(4): 277-283.

Brendler, T., J. A. Brinckmann, U. Feiter, N. Gericke, L. Lang, O. N. Pozharitskaya, A. N. Shikov, M. Smith and B. E. van Wyk (2021). "Sceletium for managing anxiety, depression and cognitive impairment: A traditional herbal medicine in modern-day regulatory systems." Current Neuropharmacology **19**(9): 1384-1400.

Brennan, B. P., J. E. Jensen, J. I. Hudson, C. E. Coit, A. Beaulieu, H. G. Pope, P. F. Renshaw and B. M. Cohen (2013). "A placebo-controlled trial of acetyl-l-carnitine and α-lipoic acid in the treatment of bipolar depression." Journal of Clinical Psychopharmacology **33**(5): 627-635.

Brenner, R., V. Azbel, S. Madhusoodanan and M. Pawlowska (2000). "Comparison of an extract of hypericum (LI 160) and sertraline in the treatment of depression: A double-blind, randomized pilot study." Clinical Therapeutics **22**(4): 411-419.

Browne, P. D., A. C. Bolte, I. Besseling-van der Vaart, E. Claassen and C. de Weerth (2021). "Probiotics as a treatment for prenatal maternal anxiety and depression: a double-blind randomized pilot trial." Sci Rep **11**(1): 3051.

C. Curtin, A. and C. S. Johnston (2023). "Vitamin B6 Supplementation Reduces Symptoms of Depression in College Women Taking Oral Contraceptives: A Randomized, Double-Blind Crossover Trial." Journal of Dietary Supplements **20**(4): 550-562.

Cao, X. J., X. C. Huang and X. Wang (2019). "Effectiveness of Chinese herbal medicine granules and traditional Chinese medicine-based psychotherapy for perimenopausal depression in Chinese women: a randomized controlled trial." Menopause **26**(10): 1193-1203.

Cao, X. W., D. Guo, J. W. Liu, W. Niu, J. Liu, J. K. Pan, H. Xie, W. W. Ouyang and D. K. Lin (2016). "The efficacy and safety of the Shaoyao Shujin tablet for knee osteoarthritis: study protocol for a randomized controlled trial." Trials **17**: 3.

Carmody, T. J., A. J. Rush, I. Bernstein, D. Warden, S. Brannan, D. Burnham, A. Woo and M. H. Trivedi (2006). "The Montgomery Äsberg and the Hamilton ratings of depression: a comparison of measures." European Neuropsychopharmacology **16**(8): 601-611.

Carnegie, R., M. C. Borges, H. J. Jones, J. Zheng, P. Haycock, J. Evans and R. M. Martin (2024). "Omega-3 fatty acids and major depression: a Mendelian randomization study." Translational Psychiatry **14**(1 C7 - 222).

Carnegie, R. E., J. Zheng, M. C. Borges, H. J. Jones, K. H. Wade, H. M. Sallis, S. J. Lewis, D. M. Evans, J. A. Revez, J. Evans and R. M. Martin (2024). "Micronutrients and Major Depression: A Mendelian Randomisation Study." Nutrients **16**(21 C7 - 3690).

Carney, R. M., K. E. Freedland, E. H. Rubin, M. W. Rich, B. C. Steinmeyer and W. S. Harris (2019). "A randomized placebo-controlled trial of omega-3 and sertraline in depressed patients with or at risk for coronary heart disease." Journal of Clinical Psychiatry **80**(4 C7 - 19m12742).

Carney, R. M., B. C. Steinmeyer, K. E. Freedland, E. H. Rubin, M. W. Rich and W. S. Harris (2016). "Baseline blood levels of omega-3 and depression remission: A secondary analysis of data from a placebo-controlled trial of omega-3 supplements." Journal of Clinical Psychiatry **77**(2): e138-e143.

Caruso, I., M. Fumagalli, L. Boccassini, S. P. Puttini, S. Santandrea, G. Giniselli and E. Parma (1987). "Treatment of depression in rheumatoid arthritic patients. A comparison of S-adenosylmethionine (Samyr) and placebo in a double-blind study." Clinical Trials Journal **24**(4): 305-310.

Chaban, О., O. O. Khaustova, D. O. Assonov and L. V. Sak (2023). "SAFETY AND EFFICACY OF THE COMPLEX DEPRILIUM® IN REDUCING SUBCLINICAL SYMPTOMS OF DEPRESSION IN PATIENTS WITH CHRONIC NON-COMMUNICABLE DISEASES: DOUBLE-BLIND RANDOMIZED CONTROLLED STUDY." Wiadomosci lekarskie (Warsaw, Poland : 1960) **76**(1): 136-144.

Chahwan, B., S. Kwan, A. Isik, S. van Hemert, C. Burke and L. Roberts (2019). "Gut feelings: A randomised, triple-blind, placebo-controlled trial of probiotics for depressive symptoms." Journal of Affective Disorders **253**: 317-326.

Chang, J. P., S. S. Chang, H. T. Yang, H. T. Chen, Y. C. Chien, B. Yang, H. Su and K. P. Su (2020). "Omega-3 polyunsaturated fatty acids in cardiovascular diseases comorbid major depressive disorder - Results from a randomized controlled trial." Brain Behav Immun **85**: 14-20.

Chang, J. P. C., S. S. Chang, H. T. Chen, K. P. Su and C. M. Pariante (2018). "A double blind-placebo controlled trial of omega-3 polyunsaturated fatty acids (n-3 PUFAs) in patients with cardiovascular diseases (CVDs) and comorbid major depressive disorder (MDD)." Nutritional Neuroscience **21**: S11.

Chen, H. M., P. H. Kuo, C. Y. Hsu, Y. H. Chiu, Y. W. Liu, M. L. Lu and C. H. Chen (2021). "Psychophysiological effects of lactobacillus plantarum ps128 in patients with major depressive disorder: A preliminary 8-week open trial." Nutrients **13**(11 C7 - 3731).

Cheng, Y. C., W. Y. Chen, C. Lin, S. H. Lee, C. C. Chiu and P. H. Kuo (2025). "The N-3 polyunsaturated fatty acids supplementation to prevent depression recurrence in patients with late-life depression: A 52-week double-blind, placebo-controlled trial." Journal of Affective Disorders **369**: 8-15.

Chhetry, B. T., A. Hezghia, J. M. Miller, S. Lee, H. Rubin-Falcone, T. B. Cooper, M. A. Oquendo, J. J. Mann and M. E. Sublette (2016). "Omega-3 polyunsaturated fatty acid supplementation and white matter changes in major depression." Journal of Psychiatric Research **75**: 65-74.

Chi, C. I. (2017). "Safety, effectiveness, and mechanism of fish oil as adjunct treatment for Major Depressive Disorder - a 12-month randomized, placebo controlled clinical trial."

Chinna Meyyappan, A., E. Forth and R. Milev (2022). "Microbial Ecosystem Therapeutic-2 Intervention in People With Major Depressive Disorder and Generalized Anxiety Disorder: Phase 1, Open-Label Study." Interact J Med Res **11**(1): e32234.

Chong, M. F. F., J. X. Y. Wong, M. Colega, L. W. Chen, R. M. van Dam, C. S. Tan, A. L. Lim, S. Cai, B. F. P. Broekman, Y. S. Lee, S. M. Saw, K. Kwek, K. M. Godfrey, Y. S. Chong, P. Gluckman, M. J. Meaney, H. Chen, P. Agarwal, A. Biswas, C. L. Bong, J. K. Yen Chan, Y. H. Chan, C. Y. I. Chee, Y. B. Cheung, A. Chia, A. Chinnadurai, C. K. Chng, S. C. Chong, M. C. Chua, C. M. Ding, E. A. Finkelstein, D. Fok, M. Fortier, A. E. N. Goh, Y. T. D. Goh, J. J. Gooley, W. M. Han, M. Hanson, C. J. Henry, J. D. Holbrook, C. Y. Hsu, H. Inskip, J. Kapur, I. Yee-Man Lau, B. W. Lee, N. Lek, S. B. Lim, Y. L. Low, I. Magiati, L. M. Daniel, C. Ngo, K. Naiduvaje, W. W. Pang, A. Qiu, B. L. Quah, V. S. Rajadurai, M. Rauff, S. A. Rebello, J. L. Richmond, A. Rifkin-Graboi, L. Pei-Chi Shek, A. Sheppard, B. Shuter, L. Singh, S. E. Soh, W. Stunkel, L. L. Su, K. H. Tan, O. H. Teoh, M. T. Tint, H. P. S. van Bever, I. B. Yun Wong, P. C. Wong, F. Yap and G. S. Heong Yeo (2014). "Relationships of maternal folate and vitamin B12 status during pregnancy with perinatal depression: The GUSTO study." Journal of Psychiatric Research **55**(1): 110-116.

Chopra, A. and A. K. Kalyani (2020). "Comparison of efficacy of Crocus sativus L. with fluoxetine for improving depression among patients with cardiovascular diseases." International Journal of Life Sciences Biotechnology and Pharma Research **9**(2): 151-154.

Choukri, M. A., T. S. Conner, J. J. Haszard, M. J. Harper and L. A. Houghton (2018). "Effect of vitamin D supplementation on depressive symptoms and psychological wellbeing in healthy adult women: A double-blind randomised controlled clinical trial." Journal of Nutritional Science **7**.

Coët, A., F. M. Blampied and J. J. Rucklidge (2025). "1-year naturalistic follow-up of a Randomised Double-Blind, Placebo-Controlled Trial (“NoMAD”) Exploring the Effectiveness of Micronutrients in Improving Symptoms of Anxiety and Depression." Journal of Affective Disorders Reports **20**.

Coppen, A. and J. Bailey (2000). "Enhancement of the antidepressant action of fluoxetine by folic acid: A randomised, placebo controlled trial." Journal of Affective Disorders **60**(2): 121-130.

Cropley, M., A. P. Banks and J. Boyle (2015). "The Effects of Rhodiola rosea L. Extract on Anxiety, Stress, Cognition and Other Mood Symptoms." Phytotherapy Research **29**(12): 1934-1939.

Ctis (2025). "EFFECTIVENESS OF SUPPLEMENTATION WITH VITAMIN D IN PATIENTS WITH DEPRESSIVE DISORDER OR BIPOLAR DISORDER: a RANDOMIZED CONTROLLED CLINICAL TRIAL."

Dabbaghmanesh, M. H., F. Vaziri, F. Najib, S. Nasiri and S. Pourahmad (2019). "The effect of vitamin D consumption during pregnancy on maternal thyroid function and depression: A randomized, placebo-controlled, clinical trial." Jundishapur Journal of Natural Pharmaceutical Products **14**(2 C7 - e65328).

Darbinyan, V., G. Aslanyan, E. Amroyan, E. Gabrielyan, C. Malmström and A. Panossian (2007). "Clinical trial of Rhodiola rosea L. extract SHR-5 in the treatment of mild to moderate depression." Nordic Journal of Psychiatry **61**(5): 343-348.

Davidson, J. R., K. M. Gadde, J. A. Fairbank, K. R. R. Krishnan, R. M. Califf, C. Binanay, C. B. Parker, N. Pugh, T. D. Hartwell, B. Vitiello and et al. (2002). "Effect of Hypericum perforatum (St John's wort) in major depressive disorder: a randomized controlled trial." **287**(14): 1807‐1814

C1803 - PUBMED 11939866,EMBASE 34303625.

Davidson, J. R. T., K. M. Gadde, J. A. Fairbank, K. Ranga Rama Krishnan, R. M. Califf, C. Binanay, C. B. Parker, N. Pugh, T. D. Hartwell, B. Vitiello, L. Ritz, J. Severe, J. O. Cole, C. De Battista, P. Murali Doraiswamy, J. P. Feighner, P. Keck, J. Kelsey, K. M. Lin, P. D. Londborg, C. B. Nemeroff, A. F. Schatzberg, D. V. Sheehan, R. K. Srivastava, L. Taylor, M. H. Trivedi and R. H. Weisler (2002). "Effect of Hypericum perforatum (St John's wort) in major depressive disorder: A randomized controlled trial." JAMA **287**(14): 1807-1814.

de Koning, E. J., P. Lips, B. Penninx, P. J. M. Elders, A. C. Heijboer, M. den Heijer, P. M. Bet, H. W. J. van Marwijk and N. M. van Schoor (2019). "Vitamin D supplementation for the prevention of depression and poor physical function in older persons: the D-Vitaal study, a randomized clinical trial." **110**(5): 1119‐1130

C1113 - PUBMED 31340012,EMBASE 628896621.

de Koning, E. J., N. L. van der Zwaluw, J. P. van Wijngaarden, E. Sohl, E. M. Brouwer-Brolsma, H. W. van Marwijk, A. W. Enneman, K. M. Swart, S. C. van Dijk, A. C. Ham, N. van der Velde, A. G. Uitterlinden, B. W. Penninx, P. J. Elders, P. Lips, R. A. Dhonukshe-Rutten, N. M. van Schoor and L. C. de Groot (2016). "Effects of Two-Year Vitamin B(12) and Folic Acid Supplementation on Depressive Symptoms and Quality of Life in Older Adults with Elevated Homocysteine Concentrations: Additional Results from the B-PROOF Study, an RCT." Nutrients **8**(11).

De Leo, D. (1987). "S-Adenosylmethionine as an antidepressant. A double-blind trial versus placebo." Current Therapeutic Research - Clinical and Experimental **41**(6): 865-870.

de Sousa-Muñoz, R. L. and R. G. Filizola (2009). "Efficacy of soy isoflavones for depressive symptoms of the climacteric syndrome." Maturitas **63**(1): 89-93.

Di Pierro, F., R. Orsi and R. Settembre (2015). "Role of betaine in improving the antidepressant effect of S-adenosyl-methionine in patients with mild-to-moderate depression." Journal of Multidisciplinary Healthcare **8**: 39-45.

Djokic, G., D. Korcok, V. Djordjevic, A. Agic, A. Rankovic and S. Djukic Dejanovic (2017). "The effects of s-adenosyl-L-methionine-vitamin b complex on mild and moderate depressive symptoms." Hippokratia **21**(3): 140-143.

Docherty, J. P., D. A. Sack, M. Roffman, M. Finch and J. R. Komorowski (2005). "A double-blind, placebo-controlled, exploratory trial of chromium picolinate in atypical depression: Effect on carbohydrate craving." Journal of Psychiatric Practice **11**(5): 302-314.

Dormal, V., L. Jonniaux, M. Buchet, L. Simar, S. Copine and L. Deldicque (2025). "Effect of Hydroponically Grown Red Panax Ginseng on Perceived Stress Level, Emotional Processing, and Cognitive Functions in Moderately Stressed Adults: A Randomized, Double-Blind, Placebo-Controlled Study." Nutrients **17**(6 C7 - 955).

Effati Daryani, F., S. M. A. Charandabi, S. Zarei, A. Mohammadi and M. Mirghafourvand (2017). "Effect of lavender cream with or without footbath on anxiety, stress and depression of women in postpartum: A clinical randomized controlled trial." Iranian Journal of Obstetrics, Gynecology and Infertility **20**(10): 52-61.

Esmaily, H., A. Sahebkar, M. Iranshahi, S. Ganjali, A. Mohammadi, G. Ferns and M. Ghayour-Mobarhan (2015). "An investigation of the effects of curcumin on anxiety and depression in obese individuals: A randomized controlled trial." Chin J Integr Med **21**(5): 332-338.

Fard, F. E., M. Mirghafourvand, S. Mohammad-Alizadeh Charandabi, A. Farshbaf-Khalili, Y. Javadzadeh and H. Asgharian (2017). "Effects of zinc and magnesium supplements on postpartum depression and anxiety: A randomized controlled clinical trial." Women and Health **57**(9): 1115-1128.

Farioli-Vecchioli, S., S. Sacchetti, N. V. di Robilant and D. Cutuli (2018). "The role of physical exercise and omega-3 fatty acids in depressive illness in the elderly." Current Neuropharmacology **16**(3): 308-326.

Fava, M., J. Alpert, A. A. Nierenberg, D. Mischoulon, M. W. Otto, J. Zajecka, H. Murck and J. F. Rosenbaum (2005). "A double-blind, randomized trial of St John's wort, fluoxetine, and placebo in major depressive disorder." Journal of Clinical Psychopharmacology **25**(5): 441-447.

Fava, M., J. S. Borus, J. E. Alpert, A. A. Nierenberg, J. F. Rosenbaum and T. Bottiglieri (1997). "Folate, vitamin B12, and homocysteine in major depressive disorder." American Journal of Psychiatry **154**(3): 426-428.

Fava, M., R. Gomeni, J. Komorowski, J. Docherty, C. Cusin, D. Soskin and D. Mischoulon (2012). "A re-analysis using a population-enrichment strategy of a double-blind, placebo-controlled study of chromium picolinate in atypical depression." Neuropsychopharmacology **38**: S341-S342.

Fava, M., S. Lamon-Fava, J. So, D. Mischoulon, A. Nierenberg, B. Dunlop, P. Schettler, B. Kinkead, T. Ziegler and M. Rapaport (2019). "Eicosapentaenoic acid (EPA) supplementation in major depressive disorder patients with chronic inflammation: Dose-dependent increases in EPA metabolites and specialized pro-resolving lipid mediators." Neuropsychopharmacology **44**: 451-452.

Fedotova, J., Z. Bereza and T. Dudnichenko (2020). "Efficacy of escitalopram in combination with vitamin D3 supplementation in the depressed perimenopausal women." European Psychiatry **63**: S109-S110.

Firoozabadi, A., S. Kolouri, M. M. Zarshenas, A. Salehi, S. H. Mosavat and S. A. Dastgheib (2016). "Efficacy of Nepeta Menthoides Boiss and Buhse Freeze-Dried Aqueous Extract on Anxiety of Patients with Depression: A Double-Blind Randomized Controlled Clinical Trial." **41**(3 Suppl): S4

C3 - PUBMED 27840470.

Firoozabadi, A., S. Kolouri, M. M. Zarshenas, A. Salehi, S. H. Mosavat and S. A. Dastgheib (2017). "Efficacy of a freeze-dried aqueous extract of Nepeta menthoides Boiss. & Buhse in the treatment of anxiety in patients with depression: A double-blind, randomized, controlled trial." Journal of Herbal Medicine **10**: 17-23.

Firoozabadi, A., M. M. Zarshenas, A. Salehi, S. Jahanbin and A. Mohagheghzadeh (2015). "Effectiveness of Cuscuta planiflora Ten. and Nepeta menthoides Boiss. & Buhse in major depression: a triple-blind randomized controlled trial study." J Evid Based Complementary Altern Med **20**(2): 94-97.

Firoozeei, T. S., M. Barekatain, M. Karimi, A. Zargaran, S. Akhondzadeh and H. Rezaeizadeh (2020). "Lavender and dodder combined herbal syrup versus citalopram in major depressive disorder with anxious distress: A double-blind randomized trial." Journal of Integrative Medicine **18**(5): 409-415.

Foshati, S., A. Ghanizadeh and M. Akhlaghi (2022). "Extra-Virgin Olive Oil Improves Depression Symptoms Without Affecting Salivary Cortisol and Brain-Derived Neurotrophic Factor in Patients With Major Depression: a Double-Blind Randomized Controlled Trial." **122**(2): 284‐297.e281

C283 - PUBMED 34358723,EMBASE 635814426.

Freeman, M. P., M. Davis, P. Sinha, K. L. Wisner, J. R. Hibbeln and A. J. Gelenberg (2008). "Omega-3 fatty acids and supportive psychotherapy for perinatal depression: A randomized placebo-controlled study." Journal of Affective Disorders **110**(1-2): 142-148.

Freeman, M. P., J. R. Hibbeln, M. Silver, A. M. Hirschberg, B. Wang, A. M. Yule, L. F. Petrillo, E. Pascuillo, N. I. Economou, H. Joffe and L. S. Cohen (2011). "Omega-3 fatty acids for major depressive disorder associated with the menopausal transition: A preliminary open trial." Menopause **18**(3): 279-284.

Freeman, M. P., J. R. Hibbeln, K. L. Wisner, B. H. Brumbach, M. Watchman and A. J. Gelenberg (2006). "Randomized dose-ranging pilot trial of omega-3 fatty acids for postpartum depression." Acta Psychiatrica Scandinavica **113**(1): 31-35.

Freeman, M. P., J. R. Hibbeln, K. L. Wisner, M. Watchman and A. J. Gelenberg (2006). An open trial of Omega-3 fatty acids for depression in pregnancy. Acta Neuropsychiatrica.

Freeman, M. P., G. M. Savella, T. R. Church, L. Goéz-Mogollón, A. Z. Sosinsky, O. B. Noe, A. Kaimal and L. S. Cohen (2019). "A prenatal supplement with methylfolate for the treatment and prevention of depression in women trying to conceive and during pregnancy." Annals of Clinical Psychiatry **31**(1): 4-16.

Friede, M., H. H. Henneicke von Zepelin and J. Freudenstein (2001). "Differential therapy of mild to moderate depressive episodes (ICD-10 F 32.0; F 32.1) with St. John's wort." Pharmacopsychiatry **34**(SUPPL. 1): S38-S41.

Fries, L. R., M. Boehme, L. Lavalle, O. Sakwinska, F. Chughlay, S. Keddani, N. Porta, M. Vicario, G. Bergonzelli, I. Silva Zolezzi and S. Y. Chan (2025). "The impact of ingestion of Bifidobacterium longum NCC3001 on perinatal anxiety and depressive symptoms: a randomized controlled trial." Sci Rep **15**(1): 11250.

Fulzele, A. V., N. Ingle, M. N. Huda and D. S. Mishra (2014). "Comparative study to evaluate the effect of a herbal preparation & shirodhara in the management of major depressive disorder." International Journal of PharmTech Research **6**(2): 506-511.

Furukawa, T. A., M. Reijnders, S. Kishimoto, M. Sakata, R. J. DeRubeis, S. Dimidjian, D. J. Dozois, U. Hegerl, S. D. Hollon and R. B. Jarrett (2019). "Translating the BDI and BDI-II into the HAMD and vice versa with equipercentile linking."

Gabbay, V., R. D. Freed, C. M. Alonso, S. Senger, J. Stadterman, B. A. Davison and R. G. Klein (2018). "A double-blind placebo-controlled trial of omega-3 fatty acids as a monotherapy for adolescent depression." Journal of Clinical Psychiatry **79**(4 C7 - 17m11596).

Gao, L., C. Wu, Y. Liao and J. Wang (2020). "Antidepressants effects of Rhodiola capsule combined with sertraline for major depressive disorder: A randomized double-blind placebo-controlled clinical trial." Journal of Affective Disorders **265**: 99-103.

Gariballa, S. and S. Forster (2007). "Effects of dietary supplements on depressive symptoms in older patients: A randomised double-blind placebo-controlled trial." Clinical Nutrition **26**(5): 545-551.

Gawlik-Kotelnicka, O., A. Margulska, A. Skowrońska and D. Strzelecki (2023). "PRO-DEMET Randomized Controlled Trial on Probiotics in Depression-Pilot Study Results." Nutrients **15**(6).

Gawlik-Kotelnicka, O., J. Rogalski, K. H. Czarnecka-Chrebelska, J. Burzyński, P. Jakubowska, A. Skowrońska and D. Strzelecki (2024). "The Interplay Between Depression, Probiotics, Diet, Immunometabolic Health, the Gut, and the Liver—A Secondary Analysis of the Pro-Demet Randomized Clinical Trial." Nutrients **16**(23 C7 - 4024).

Gawlik-Kotelnicka, O., A. Skowrońska, A. Margulska, K. H. Czarnecka-Chrebelska, I. Łoniewski, K. Skonieczna-żydecka and D. Strzelecki (2021). "The influence of probiotic supplementation on depressive symptoms, inflammation, and oxidative stress parameters and fecal microbiota in patients with depression depending on metabolic syndrome comorbidity—pro-demet randomized study protocol." Journal of Clinical Medicine **10**(7 C7 - 1342).

Gawlik-Kotelnicka, O., A. Skowrońska, A. Margulska and D. Strzelecki (2023). "The Influence of Probiotic Supplementation on Depression, Anxiety, and Stress Level, as well as Inflammation, Anthropometric and Metabolic Parameters in Patients with Depressive Disorders-preliminary results of an RCT." European Psychiatry **66**: S841-S842.

Gecele, M., G. Francesetti and A. Meluzzi (1991). "Acetyl-L-carnitine in aged subjects with major depression: Clinical efficacy and effects on the circadian rhythm of cortisol." Dementia **2**(6): 333-337.

Gelenberg, A. J., R. C. Shelton, P. Crits-Christoph, M. B. Keller, D. L. Dunner, R. M. Hirschfeld, M. E. Thase, J. M. Russell, R. B. Lydiard, R. J. Gallop and et al. (2004). "The effectiveness of St. John's Wort in major depressive disorder: a naturalistic phase 2 follow-up in which nonresponders were provided alternate medication." **65**(8): 1114‐1119

C1113 - PUBMED 15323598.

Ghaffari, S., A. Ghobadi, A. H. Jamshidi, S. H. Mortazavi, S. Naderi, A. Aqamolaei, A. Mortezaei, E. Sahebolzamani, A. Shamabadi, S. Jalilvand, B. Daraei, M. R. Shalbafan and S. Akhondzadeh (2020). "Cinnamomum tamala as an adjuvant therapy in the treatment of major depressive disorder: A double-blind, randomized, placebo-controlled clinical trial with placebo control." Advances in Integrative Medicine **7**(3): 141-147.

Ghajar, A., S. M. Neishabouri, N. Velayati, L. Jahangard, N. Matinnia, M. Haghighi, A. Ghaleiha, M. Afarideh, S. Salimi, A. Meysamie and S. Akhondzadeh (2017). "Crocus sativus L versus Citalopram in the Treatment of Major Depressive Disorder with Anxious Distress: A Double-Blind, Controlled Clinical Trial." Pharmacopsychiatry **50**(4): 152-160.

Ghaleiha, A., H. Davari, L. Jahangard, M. Haghighi, M. Ahmadpanah, M. A. Seifrabie, H. Bajoghli, E. Holsboer-Trachsler and S. Brand (2016). "Adjuvant thiamine improved standard treatment in patients with major depressive disorder: results from a randomized, double-blind, and placebo-controlled clinical trial." European Archives of Psychiatry and Clinical Neuroscience **266**(8): 695-702.

Ghamchini, V. M., M. Salami, G. R. Mohammadi, Z. Moradi, A. Kavosi, A. Movahedi, M. Bidkhori and M. R. Aryaeefar (2019). "The effect of chamomile tea on anxiety and depression in cancer patients treated with chemotherapy." Journal of Young Pharmacists **11**(3): 309-312.

Gharekhani, A., M. R. Khatami, S. Dashti-Khavidaki, E. Razeghi, A. A. Noorbala, S. S. Hashemi-Nazari and M. A. Mansournia (2014). "The effect of omega-3 fatty acids on depressive symptoms and inflammatory markers in maintenance hemodialysis patients: A randomized, placebo-controlled clinical trial." European Journal of Clinical Pharmacology **70**(6): 655-665.

Ghorbani, Z., S. Nazari, F. Etesam, S. Nourimajd, M. Ahmadpanah and S. R. Jahromi (2018). "The effect of synbiotic as an adjuvant therapy to fluoxetine in moderate depression: A randomized multicenter trial." Archives of Neuroscience **5**(2).

Gilbert, G. E. W. A. H. (2010). "Omega-3 fatty acids not effective for depression in patients with coronary heart disease." Journal of the National Medical Association **102**(2): 151.

Ginsberg, D. L. (2003). "Chromium Picolinate Treatment of Atypical Depression." Primary Psychiatry **10**(4): 23-24.

Grenyer, B. F., T. Crowe, B. Meyer, A. J. Owen, E. M. Grigonis-Deane, P. Caputi and P. R. Howe (2007). "Fish oil supplementation in the treatment of major depression: a randomised double-blind placebo-controlled trial." **31**(7): 1393‐1396

C1393 - PUBMED 17659823.

Grobler, A. C., G. Matthews and G. Molenberghs (2014). "The impact of missing data on clinical trials: A re-analysis of a placebo controlled trial of Hypericum perforatum (St Johns wort) and sertraline in major depressive disorder." Psychopharmacology **231**(9): 1987-1999.

Gul, A., N. Memtily, P. Mijit, M. Imin, H. Rui, P. Wushuer and A. Talifu (2019). "The Üstikuddus sherbiti in the treatment of abnormal cold and dry-type depression patients with comorbid anxiety: A randomized and controlled clinical trial." Traditional Medicine and Modern Medicine **2**(3): 127-133.

Haberka, M., K. Mizia-Stec, M. Mizia, K. Gieszczyk, A. Chmiel, K. Sitnik-Warchulska and Z. Gasior (2012). "Effects of n-3 polyunsaturated fatty acids on depressive symptoms, anxiety and emotional state in patients with acute myocardial infarction." **19**(1): S42

C43 - EMBASE 71706389.

Haghighat, N., S. Rajabi and M. Mohammadshahi (2021). "Effect of synbiotic and probiotic supplementation on serum brain-derived neurotrophic factor level, depression and anxiety symptoms in hemodialysis patients: a randomized, double-blinded, clinical trial." Nutritional Neuroscience **24**(6): 490-499.

Hallahan, B., T. Ryan, J. R. Hibbeln, I. T. Murray, S. Glynn, C. E. Ramsden, J. P. SanGiovanni and J. M. Davis (2016). "Efficacy of omega-3 highly unsaturated fatty acids in the treatment of depression." British Journal of Psychiatry **209**(3): 192-201.

Hamlbar, E. P., M. Mirghafourvand, E. Shaseb and M. Kamalifard (2025). "The effect of curcumin on postpartum depression and anxiety in primiparous women: a double-blind randomized placebo-controlled clinical trial." BMC Complementary Medicine and Therapies **25**(1).

Han, C. H., M. I. Chung, S. Bose, C. Lim, H. Kim, B. S. Koo and S. C. Pak (2012). "The effect of Gami Guibitang (GGBT), a medicinal herbal formulation, on moderate depression." European Journal of Integrative Medicine **4**(4): e400-e407.

Harrer, G., U. Schmidt, U. Kuhn and A. Biller (1999). "Comparison of equivalence between the St. John's wort extract LoHyp-57 and fluoxetine." Arzneimittel-Forschung/Drug Research **49**(4): 289-296.

Hatton, D. C., J. Harrison-Hohner, J. Matarazzo, P. Edwards, A. Lewy and L. Davis (2007). "Missed antenatal depression among high risk women: A secondary analysis." Archives of Women's Mental Health **10**(3): 121-123.

Hawley, C. J., T. M. Gale, P. S. J. Smith, S. Jain, A. Farag, R. Kondan, C. Avent and J. Graham (2013). "Equations for converting scores between depression scales (MÅDRS, SRS, PHQ‐9 and BDI‐II): Good statistical, but weak idiographic, validity." Human Psychopharmacology: Clinical and Experimental **28**(6): 544-551.

Haybar, H., A. Z. Javid, M. H. Haghighizadeh, E. Valizadeh, S. M. Mohaghegh and A. Mohammadzadeh (2018). "The effects of Melissa officinalis supplementation on depression, anxiety, stress, and sleep disorder in patients with chronic stable angina." Clinical Nutrition ESPEN **26**: 47-52.

He, X., D. Liu, S. Ni, Z. Li, S. Li, T. Wu, X. Dong, X. Zhang, Y. Tang, Y. Ling, H. Liao, J. Kang, Y. Li, H. Wu, J. Luo, X. Wan, D. Zhang, L. Lu, W. Long and Z. Yang (2024). "Efficacy and safety evaluation of Ginkgo biloba dropping pill (GBDP) on stable angina pectoris complicated with depression: A placebo-controlled, randomized, double-blind, multicenter study." Phytomedicine **126**.

Heaton, J. L., S. A. Campbell, H. A. Bradley, R. T. Mulder, L. Dixon, J. Henderson and J. J. Rucklidge (2025). "Broad-Spectrum Micronutrients or Antidepressants for Antenatal Depression: Effect on Maternal and Infant Birth Outcomes in an Observational Secondary Analysis of NUTRIMUM." Journal of Clinical Psychopharmacology **45**(1): 4-15.

Hellem, T. L., Y.-H. Sung, X.-F. Shi, M. A. Pett, G. Latendresse, J. Morgan, R. S. Huber, D. Kuykendall, K. J. Lundberg and P. F. Renshaw (2015). "Creatine as a novel treatment for depression in females using methamphetamine: A pilot study." Journal of Dual Diagnosis **11**(3-4): 189-202.

Hidese, S., M. Ota, C. Wakabayashi, T. Noda, H. Ozawa, T. Okubo and H. Kunugi (2017). "Effects of chronic l-theanine administration in patients with major depressive disorder: An open-label study." Acta Neuropsychiatrica **29**(2): 72-79.

Ho, Y. T., Y. C. Tsai, T. B. J. Kuo and C. C. H. Yang (2021). "Effects of lactobacillus plantarum ps128 on depressive symptoms and sleep quality in self-reported insomniacs: A randomized, double-blind, placebo-controlled pilot trial." Nutrients **13**(8 C7 - 2820).

Holsboen, F., O. Benkert, L. Meier and A. Kreuz-Kersting (1985). "Combined estradiol and vitamin B6 treatment in women with major depression." The American journal of psychiatry **142**(5): 658.

Hosseini, H., M. Bagherniya, A. Sahebkar, B. Iraj, M. Majeed and G. Askari (2024). "The effect of curcumin-piperine supplementation on lipid profile, glycemic index, inflammation, and blood pressure in patients with type 2 diabetes mellitus and hypertriglyceridemia." Phytotherapy Research **38**(11): 5150-5161.

Hu, Y., C. Chen, Y. Wang, W. Yang, Y. Wang, W. Zhu, C. Yan and P. Liu (2021). "The effects of KaiXinSan on depression and its association with lipid profiles: a randomized, double-blinded, placebo-controlled trial." **83**: 153467

C153463 - PUBMED 33516143,EMBASE 2010802711.

Hulkkonen, P., E. L. Kataja, T. Vahlberg, E. Koivuniemi, N. Houttu, O. Pellonperä, K. Mokkala, H. Karlsson and K. Laitinen (2021). "The efficacy of probiotics and/or n-3 long-chain polyunsaturated fatty acids intervention on maternal prenatal and postnatal depressive and anxiety symptoms among overweight and obese women." Journal of Affective Disorders **289**: 21-30.

Hvas, A. M., S. Juul, L. Lauritzen, E. Nexø and J. Ellegaard (2004). "No effect of vitamin B-12 treatment on cognitive function and depression: A randomized placebo controlled study." Journal of Affective Disorders **81**(3): 269-273.

Ibero-Baraibar, I., A. Perez-Cornago, M. J. Ramirez, J. A. Martinez and M. A. Zulet (2015). "An increase in plasma homovanillic acid with cocoa extract consumption is associated with the alleviation of depressive symptoms in overweight or obese adults on an energy restricted diet in a randomized controlled trial." **146**(4): 897S‐904S

C893 - PUBMED 26962189,EMBASE 610003794.

Ille, R., J. Spona, M. Zickl, P. Hofmann, T. Lahousen, N. Dittrich, G. Bertha, K. Hasiba, F. A. Mahnert and H. P. Kapfhammer (2007). ""Add-On"-therapy with an individualized preparation consisting of free amino acids for patients with a major depression." European Archives of Psychiatry and Clinical Neuroscience **257**(4): 222-229.

Irct138901233695N (2010). "Effect of vitamins in treatment of depression."

Irct201109297669N (2011). "The impact of Omga-3 fatty acids on depression."

Irct2014041916465N (2014). "Evaluating the effects of Crocus sativus L. (Saffron) on weight and depression."

Irct2014082518922N (2014). "Effect of Folic acid supplementation on the therapeutic regimen for major depressive disorder."

Irct2017082612438N (2017). "The effect of barij deprex capsule on Depressive symptoms."

Irct20170311033004N (2022). "Effects of alkaloids of harmala seeds on depression."

Irct20240107060642N (2024). "Investigating the effect of folic acid supplementation on postpartum depression in women."

Irct20240619062173N (2024). "Effect of Cuscuta epithymum on depression."

Islambulchilar, M., M. Morovati, Y. Morsali, S. Rezaee and A. Esmaeilzadeh (2023). "Could one-milligram daily folic acid supplementation change the intensity of the disorder in outpatients with depression who use citalopram?" Koomesh **25**(5).

Isrctn (2024). "Probiotic supplementation for maternal depression."

j57vj, R. B. R. (2024). "Effect of Magnesium Chloride supplementation on Depression."

Jahangard, L., A. Sadeghi, M. Ahmadpanah, E. Holsboer-Trachsler, D. Sadeghi Bahmani, M. Haghighi and S. Brand (2018). "Influence of adjuvant omega-3-polyunsaturated fatty acids on depression, sleep, and emotion regulation among outpatients with major depressive disorders - Results from a double-blind, randomized and placebo-controlled clinical trial." Journal of Psychiatric Research **107**: 48-56.

Jam, I. N., A. H. Sahebkar, S. Eslami, N. Mokhber, M. Nosrati, M. Khademi, M. Foroutan-Tanha, M. Ghayour-Mobarhan, F. Hadizadeh, G. Ferns and M. Abbasi (2017). "The effects of crocin on the symptoms of depression in subjects with metabolic syndrome." Advances in Clinical and Experimental Medicine **26**(6): 925-930.

Javadinia, S. A., R. Salek, M. Dehghani, S. A. Mohajeri, A. Talaei and A. Fanipakdel (2021). "Amelioration of anxiety, depression, and chemotherapy related toxicity after crocin administration during chemotherapy of breast cancer: A double blind, randomized clinical trial." Annals of Oncology **32**: S439-S440.

Jazayeri, S., M. Tehrani-Doost, S. A. Keshavarz, M. Hosseini, A. Djazayery, H. Amini, M. Jalali and M. Peet (2008). "Comparison of therapeutic effects of omega-3 fatty acid eicosapentaenoic acid and fluoxetine, separately and in combination, in major depressive disorder." Australian and New Zealand Journal of Psychiatry **42**(3): 192-198.

Jelodar, G., Z. Javid, A. Sahraian and S. Jelodar (2018). "Saffron improved depression and reduced homocysteine level in patients with major depression: A Randomized, double-blind study." Avicenna Journal of Phytomedicine **8**(1): 43-50.

Jeong, H. G., Y. H. Ko, S. Y. Oh, C. Han, T. Kim and S. H. Joe (2015). "Effect of Korean Red Ginseng as an adjuvant treatment for women with residual symptoms of major depression." Asia-Pacific Psychiatry **7**(3): 330-336.

Jiang, W., D. J. Whellan, K. F. Adams, M. A. Babyak, S. H. Boyle, J. L. Wilson, C. B. Patel, J. G. Rogers, W. S. Harris and C. M. O'Connor (2018). "Long-Chain Omega-3 Fatty Acid Supplements in Depressed Heart Failure Patients: Results of the OCEAN Trial." JACC: Heart Failure **6**(10): 833-843.

Jokar, A. and F. Farahi (2014). "Effect of vitamin C on depression in menopausal women with balanced diet: A randomized clinical trial." Iranian Journal of Obstetrics, Gynecology and Infertility **17**(121): 18-23.

Jorde, R. and J. Kubiak (2018). "No improvement in depressive symptoms by vitamin D supplementation: Results from a randomised controlled trial." Journal of Nutritional Science **7**.

Jorde, R., M. Sneve, Y. Figenschau, J. Svartberg and K. Waterloo (2008). "Effects of vitamin D supplementation on symptoms of depression in overweight and obese subjects: Randomized double blind trial." Journal of Internal Medicine **264**(6): 599-609.

Jurcic, J., J. A. Pereira and D. Kavanaugh (2007). "St John's wort versus paroxetine for depression." Canadian Family Physician **53**(9): 1511-1513.

Kagan, B. L., D. L. Sultzer, N. Rosenlicht and R. H. Gerner (1990). "Oral S-adenosylmethionine in depression: a randomized, double-blind, placebo-controlled trial." **147**(5): 591‐595

C593 - PUBMED 2183633.

Kalb, R., R. D. Trautmann-Sponsel and M. Kieser (2001). "Efficacy and tolerability of Hypericum extract WS 5572 versus placebo in mildly to moderately depressed patients: A randomized double-blind multicenter clinical trial." Pharmacopsychiatry **34**(3): 96-103.

Kanchanatawan, B., S. Tangwongchai, A. Sughondhabhirom, S. Suppapitiporn, S. Hemrunrojn, A. F. Carvalho and M. Maes (2018). "Add-on Treatment with Curcumin Has Antidepressive Effects in Thai Patients with Major Depression: Results of a Randomized Double-Blind Placebo-Controlled Study." Neurotoxicity Research **33**(3): 621-633.

Karakula-Juchnowicz, H., J. Rog, D. Juchnowicz, I. Łoniewski, K. Skonieczna-Ydecka, P. Krukow, M. Futyma-Jedrzejewska and M. Kaczmarczyk (2019). "The study evaluating the effect of probiotic supplementation on the mental status, inflammation, and intestinal barrier in major depressive disorder patients using gluten-free or gluten-containing diet (SANGUT study): A 12-week, randomized, double-blind, and placebo-controlled clinical study protocol." Nutrition Journal **18**(1).

Karimi, F. Z., H. Hosseini, S. R. Mazloom and H. Rakhshandeh (2021). "Effect of oral capsule of Ocimum basilicum leaf extract on depression in menopausal women: A randomized clinical trial." Iranian Journal of Obstetrics, Gynecology and Infertility **24**(2): 50-58.

Kashani, L., S. Esalatmanesh, F. Eftekhari, S. Salimi, T. Foroughifar, F. Etesam, H. Safiaghdam, E. Moazen-Zadeh and S. Akhondzadeh (2018). "Efficacy of Crocus sativus (saffron) in treatment of major depressive disorder associated with post-menopausal hot flashes: a double-blind, randomized, placebo-controlled trial." Archives of Gynecology and Obstetrics **297**(3): 717-724.

Kashani, L., S. Eslatmanesh, N. Saedi, N. Niroomand, M. Ebrahimi, M. Hosseinian, T. Foroughifar, S. Salimi and S. Akhondzadeh (2017). "Comparison of Saffron versus Fluoxetine in Treatment of Mild to Moderate Postpartum Depression: A Double-Blind, Randomized Clinical Trial." Pharmacopsychiatry **50**(2): 64-68.

Kasper, D. C., J. A. Widness, N. Haiden, A. Berger, M. Hayde, A. Pollak and K. R. Herkner (2009). "Characterization and differentiation of iron status in anemic very low birth weight infants using a diagnostic nomogram." Neonatology **95**(2): 164-171.

Kasper, S., I. Anghelescu and A. Dienel (2015). "Efficacy of orally administered Silexan in patients with anxiety-related restlessness and disturbed sleep--A randomized, placebo-controlled trial." **25**(11): 1960‐1967

C1963 - PUBMED 26293583,EMBASE 605643089.

Kasper, S., I. G. Anghelescu, A. Szegedi, A. Dienel and M. Kieser (2006). "Superior efficacy of St John's wort extract WS 5570 compared to placebo in patients with major depression: a randomized, double-blind, placebo-controlled, multi-center trial." **4**: 14

C13 - PUBMED 16796730,EMBASE 44227549.

Kasper, S., I. G. Anghelescu, A. Szegedi, A. Dienel and M. Kieser (2007). "Placebo controlled continuation treatment with Hypericum extract WS® 5570 after recovery from a mild or moderate depressive episode." Wiener Medizinische Wochenschrift **157**(13-14): 362-366.

Kasper, S., F. Caraci, B. Forti, F. Drago and E. Aguglia (2010). "Efficacy and tolerability of Hypericum extract for the treatment of mild to moderate depression." European Neuropsychopharmacology **20**(11): 747-765.

Kasper, S., M. Gastpar, W. E. Müller, H. P. Volz, A. Dienel and H. J. Möller (2008). "Treatment of depression with St. John's wort extract WS® 5570. No influence on patients' weight." Psychopharmakotherapie **15**(1): 6-10.

Kasper, S., E. Seifritz and H. P. Volz (2024). "Efficacy of Silexan in Patients with a Major Depressive Episode - First Results from a Multi-centre, Doubleblind, Randomised, Placebo- and Reference-controlled Phase III Trial." European Psychiatry **67**: S224-S225.

Kasper, S., H. P. Volz, H. J. Möller, S. Schläfke, S. Klement, I. G. Anghelescu and E. Seifritz (2024). "Lavender oil preparation Silexan is effective in mild-to-moderate major depression: a randomized, placebo- and reference-controlled trial." European Archives of Psychiatry and Clinical Neuroscience.

Katrenčíková, B., M. Vaváková, Z. Paduchová, Z. Nagyová, I. Garaiova, J. Muchová, Z. Ďuračková and J. Trebatická (2021). "Oxidative stress markers and antioxidant enzymes in children and adolescents with depressive disorder and impact of omega-3 fatty acids in randomised clinical trial." Antioxidants **10**(8 C7 - 1256).

Kaviani, M., B. Nikooyeh, F. Etesam, S. J. Behnagh, H. M. Kangarani, M. Arefi, P. Yaghmaei and T. R. Neyestani (2022). "Effects of vitamin D supplementation on depression and some selected pro-inflammatory biomarkers: a double-blind randomized clinical trial." BMC Psychiatry **22**(1 C7 - 694).

Kazemi, A., A. A. Noorbala, K. Azam, M. H. Eskandari and K. Djafarian (2019). "Effect of probiotic and prebiotic vs placebo on psychological outcomes in patients with major depressive disorder: A randomized clinical trial." Clinical Nutrition **38**(2): 522-528.

Kazemi, A., A. A. Noorbala and K. Djafarian (2020). "Effect of probiotic and prebiotic versus placebo on appetite in patients with major depressive disorder: post hoc analysis of a randomised clinical trial." Journal of Human Nutrition and Dietetics **33**(1): 56-65.

Keshavarz, S. A., S. A. Mostafavi, S. Akhondzadeh, M. R. Mohammadi, S. Hosseini, M. R. Eshraghian and M. Chamari (2018). "Omega-3 supplementation effects on body weight and depression among dieter women with co-morbidity of depression and obesity compared with the placebo: A randomized clinical trial." Clinical Nutrition ESPEN **25**: 37-43.

Khaddage, R., M. Bassil and B. Rahi (2023). "Vitamin D Supplementation Improves Depressive Symptomatology Among Vitamin D Deficient Saudi Arabian Adults - A Randomized Clinical Trial." Current Developments in Nutrition **7**.

Khajehnasiri, F., S. Akhondzadeh, S. B. Mortazavi, A. Allameh, G. Sotoudeh, A. Khavanin and Z. Zamanian (2015). "Are supplementation of omega-3 and ascorbic acid effective in reducing oxidative stress and depression among depressed shift workers?" International Journal for Vitamin and Nutrition Research **85**(5-6): 299-310.

Khalatbari Mohseni, G., S. A. Hosseini, N. Majdinasab and B. Cheraghian (2023). "Effects of N-acetylcysteine on oxidative stress biomarkers, depression, and anxiety symptoms in patients with multiple sclerosis." Neuropsychopharmacology Reports **43**(3): 382-390.

Khalili, M., H. Karimi and S. Sadri (2011). "Effect of fish oil supplement and weight loss diet on depression in obese women." Obesity Reviews **12**: 70.

Khanna, A., F. John, S. Das, J. Thomas, J. Rao, B. Maliakel and K. Im (2020). "Efficacy of a novel extract of fenugreek seeds in alleviating vasomotor symptoms and depression in perimenopausal women: A randomized, double-blinded, placebo-controlled study." Journal of food biochemistry **44**(12): e13507.

Khodadad, M., P. Bahadoran, G. Kheirabadi and A. Sabzghabaee (2021). "Can Vitamin B6 help to prevent postpartum depression? A randomized controlled trial." International Journal of Preventive Medicine **12**(1 C7 - 12).

Khoraminya, N., M. Tehrani-Doost, S. Jazayeri, A. Hosseini and A. Djazayery (2013). "Therapeutic effects of vitamin D as adjunctive therapy to fluoxetine in patients with major depressive disorder." Australian and New Zealand Journal of Psychiatry **47**(3): 271-275.

Khorvash, F., P. Hajihashemi, G. Askari, M. Noorian and M. R. Maracy (2019). "The effects of combined supplementation of coenzyme Q10 with L-carnitine on migraine-related disability and depression among patient with migraine: A randomized, placebo-controlled, double-blind trial." Journal of the Neurological Sciences **405**: 106.

Kieser, M. and A. Szegedi (2005). "Predicting stable treatment response in patients with major depression treated with hypericum extract WS® 5570/5572." Pharmacopsychiatry **38**(5): 194-200.

Kim, Y., Y. Choi, M. Y. Lee, S. H. Cho, I. C. Jung, D. H. Kang and C. Yang (2023). "Bangpungtongsung-san for patients with major depressive disorder: study protocol for a randomized controlled phase II clinical trial." BMC Complement Med Ther **23**(1): 114.

Kious, B. M., H. Sabic, Y.-H. Sung, D. G. Kondo and P. Renshaw (2017). "An open-label pilot study of combined augmentation with creatine monohydrate and 5-hydroxytryptophan for selective serotonin reuptake inhibitor–or serotonin-norepinephrine reuptake inhibitor–resistant depression in adult women." Journal of clinical psychopharmacology **37**(5): 578-583.

Kolouri, S., A. Firoozabadi, A. Salehi, M. M. Zarshenas, S. A. Dastgheib, M. Heydari and H. Rezaeizadeh (2016). "Nepeta menthoides Boiss. & Buhse freeze-dried aqueous extract versus sertraline in the treatment of major depression: A double blind randomized controlled trial." Complement Ther Med **26**: 164-170.

Komorniak, N., M. Kaczmarczyk, I. Łoniewski, A. Martynova-Van Kley, A. Nalian, M. Wroński, K. Kaseja, B. Kowalewski, M. Folwarski and E. Stachowska (2023). "Analysis of the Efficacy of Diet and Short-Term Probiotic Intervention on Depressive Symptoms in Patients after Bariatric Surgery: A Randomized Double-Blind Placebo Controlled Pilot Study." Nutrients **15**(23 C7 - 4905).

Kotlega, D., A. Zembron-Lacny, M. Golab-Janowska, P. Nowacki and M. Szczuko (2020). "The association of free fatty acids and eicosanoids with the severity of depressive symptoms in stroke patients." International Journal of Molecular Sciences **21**(15 C7 - 5220): 1-18.

Kreuzer, K., A. Reiter, A. M. Birkl-Töglhofer, N. Dalkner, S. Mörkl, M. Mairinger, E. Fleischmann, F. Fellendorf, M. Platzer, M. Lenger, T. Färber, M. Seidl, A. Birner, R. Queissner, L. M. S. Mendel, A. Maget, A. Kohlhammer-Dohr, A. Häussl, J. Wagner-Skacel, H. Schöggl, D. Amberger-Otti, A. Painold, T. Lahousen-Luxenberger, B. Leitner-Afschar, J. Haybaeck, H. Habisch, T. Madl, E. Reininghaus and S. Bengesser (2022). "The PROVIT Study—Effects of Multispecies Probiotic Add-on Treatment on Metabolomics in Major Depressive Disorder—A Randomized, Placebo-Controlled Trial." Metabolites **12**(8).

Kumar, P. N. S., V. Menon and C. Andrade (2022). "A randomized, double-blind, placebo-controlled, 12-week trial of vitamin D augmentation in major depressive disorder associated with vitamin D deficiency." Journal of Affective Disorders **314**: 143-149.

Laakmann, G. (1998). "St. John's Wort in mild to moderate depression: The relevance of hyperforin for the clinical efficacy." Pharmacopsychiatry **31**(SUPPL. 1): 54-59.

Lagzi, N., A. Bateni, R. Goli and N. Talebiazar (2023). "The effect of multivitamins on anxiety and depression in patients undergoing methadone maintenance treatment: A double-blind randomized controlled trial." International Journal of Psychiatry in Medicine **58**(6): 576-590.

Lamon-Fava, S., M. Liu, B. W. Dunlop, B. Kinkead, P. J. Schettler, J. C. Felger, T. R. Ziegler, M. Fava, D. Mischoulon and M. H. Rapaport (2023). "Clinical response to EPA supplementation in patients with major depressive disorder is associated with higher plasma concentrations of pro-resolving lipid mediators." Neuropsychopharmacology **48**(6): 929-935.

Lc, R., B. Em, H. Db, S. H and P. Ar (2021). "A pilot randomized controlled trial testing supplements of omega-3 fatty acids, probiotics, combination or placebo on symptoms of depression, anxiety and stress." Journal of Affective Disorders Reports **5 C7 - 100141**.

Lecrubier, Y., G. Clerc, R. Didi and M. Kieser (2002). "Efficacy of St. John's wort extract WS 5570 in major depression: A double-blind, placebo-controlled trial." American Journal of Psychiatry **159**(8): 1361-1366.

Lee, Y. J. and K. Y. Sohng (2019). "Effects of Vitamin D on depression, cognitive function, and physical function in elderly individuals living alone." International Journal of Gerontology **13**(3): 196-201.

Lespérance, F., N. Frasure-Smith, E. St-André, G. Turecki, P. Lespérance and S. R. Wisniewski (2011). "The efficacy of omega-3 supplementation for major depression: A randomized controlled trial." Journal of Clinical Psychiatry **72**(8): 1054-1062.

Levant, B. (2011). Brain N-3 (Omega-3) fatty acids in neurochemistry, behavior and mood: Implications for the treatment and prevention of non-puerperal and postpartum depression. Lipids: Categories, Biological Functions and Metabolism, Nutrition and Health**:** 127-152.

Levine, J., Y. Barak, M. Gonzalves, H. Szor, A. Elizur, O. Kofman and R. H. Belmaker (1995). "Double-blind, controlled trial of inositol treatment of depression." American Journal of Psychiatry **152**(5): 792-794.

Levine, J., Y. Barak, O. Kofman and R. H. Belmaker (1995). "Follow-up and relapse analysis of an inositol study of depression." The Israel journal of psychiatry and related sciences **32**(1): 14-21.

Li, Y., L. Li, R. Guo, H. Yu, X. Wang, B. Wang, Q. Wang, Z. Li, H. Zhao, Q. Temu and et al. (2022). "Clinical efficacy of Shugan granule in the treatment of mixed anxiety-depressive disorder: a multicenter, randomized, double-blind, placebo-controlled trial." **290**: 115032

C115033 - PUBMED 35085742,EMBASE 637131287.

Liang, Z. H., Y. B. Jia, M. L. Wang, Z. R. Li, M. Li, Y. L. Yun and R. X. Zhu (2019). "Efficacy of ginkgo biloba extract as augmentation of venlafaxine in treating post-stroke depression." Neuropsychiatric Disease and Treatment **15**: 2551-2557.

Lin, C., S. H. Lee, C. M. Huang, Y. W. Wu, Y. X. Chang, H. L. Liu, S. H. Ng, Y. C. Cheng, C. C. Chiu and S. C. Wu (2024). "Cognitive protection and brain entropy changes from omega-3 polyunsaturated fatty acids supplement in late-life depression: A 52-week randomized controlled trial." Journal of Affective Disorders **351**: 15-23.

Lin, C. H., S. H. Wang and H. Y. Lane (2022). "Effects of Sodium Benzoate, a D-Amino Acid Oxidase Inhibitor, on Perceived Stress and Cognitive Function Among Patients With Late-Life Depression: A Randomized, Double-Blind, Sertraline- and Placebo-Controlled Trial." International Journal of Neuropsychopharmacology **25**(7): 545-555.

Lin, K., Y. Li, E. D. Toit, L. Wendt and J. Sun (2021). "Effects of Polyphenol Supplementations on Improving Depression, Anxiety, and Quality of Life in Patients With Depression." Frontiers in Psychiatry **12 C7 - 765485**.

Lin, S. K. K., P. H. Kuo, C. Y. Hsu, Y. H. Chiu and C. H. Chen (2024). "The effects of Lactobacillus plantarum PS128 in patients with major depressive disorder: an eight-week double-blind, placebo-controlled study." Asian Journal of Psychiatry **101 C7 - 104210**.

Lingærde, O., A. R. Føreland and A. Magnusson (1999). "Can winter depression be prevented by Ginkgo biloba extract? A placebo- controlled trial." Acta Psychiatrica Scandinavica **100**(1): 62-66.

Lipovac, M., P. Chedraui, C. Gruenhut, A. Gocan, M. Stammler and M. Imhof (2010). "Improvement of postmenopausal depressive and anxiety symptoms after treatment with isoflavones derived from red clover extracts." Maturitas **65**(3): 258-261.

Loas, G., D. Rose, P. Nowaczkowski, P. Lernout and B. Duron (1996). "Proportion of non-responsive patient to amitriptyline: Exploratory pilot study of the French center. A report from the WHO project on the combined utilization of an antidepressant (amitriptyline) and an antioxidant (ginkgo biloba) in the treatment of therapy-resistant depression." Annales Medico-Psychologiques **154**(3): 202-203.

Lôo, H., M. F. Poirier, H. Ollat and S. Elatki (2000). "Effects of sulbutiamine (Arcalion 200) on psycho-behavioral inhibition in major depressive episodes." **26**(2): 70‐75

C73 - PUBMED 10858919,EMBASE 30337124.

Lopresti, A. L. and P. D. Drummond (2017). "Efficacy of curcumin, and a saffron/curcumin combination for the treatment of major depression: A randomised, double-blind, placebo-controlled study." Journal of Affective Disorders **207**: 188-196.

Lopresti, A. L., M. Maes, G. L. Maker, S. D. Hood and P. D. Drummond (2014). "Curcumin for the treatment of major depression: A randomised, double-blind, placebo controlled study." Journal of Affective Disorders **167**: 368-375.

Lopresti, A. L., S. J. Smith, S. D. Hood and P. D. Drummond (2019). "Efficacy of a standardised saffron extract (affron®) as an add-on to antidepressant medication for the treatment of persistent depressive symptoms in adults: A randomised, double-blind, placebo-controlled study." Journal of Psychopharmacology **33**(11): 1415-1427.

Lopresti, A. L., S. J. Smith, H. Malvi and R. Kodgule (2019). "An investigation into the stress-relieving and pharmacological actions of an ashwagandha (Withania somnifera) extract: a randomized, double-blind, placebo-controlled study." **98**(37): e17186

C17183 - PUBMED 31517876.

Lopresti, A. L., S. J. Smith, W. Marx, M. Díez-Municio and M. I. Morán-Valero (2025). "An Examination into the Effects of a Saffron Extract (Affron) on Mood and General Wellbeing in Adults Experiencing Low Mood: A Randomized, Double-Blind, Placebo-Controlled Trial." Journal of Nutrition.

Loria-Kohen, V., C. Gómez-Candela, S. Palma-Milla, B. Amador-Sastre, A. Hernanz and L. M. Bermejo (2013). "A pilot study of folic acid supplementation for improving homocysteine levels, cognitive and depressive status in eating disorders." Nutricion Hospitalaria **28**(3): 807-815.

Lu, J., H. Mao, Y. Tan and G. Luo (2024). "Associations of Dietary Intake of Vitamin B6 and Plasma Pyridoxal 5′-Phosphate Level With Depression in US Adults: Findings From NHANES 2005–2010." Brain and Behavior **14**(11 C7 - e70128).

Lucas, M., G. Asselin, C. Mérette, M. J. Poulin and S. Dodin (2009). "Ethyl-eicosapentaenoic acid for the treatment of psychological distress and depressive symptoms in middle-aged women: A double-blind, placebo-controlled, randomized clinical trial." American Journal of Clinical Nutrition **89**(2): 641-651.

Lucas, M., F. Mirzaei, E. J. O'Reilly, A. Pan, W. C. Willett, I. Kawachi, K. Koenen and A. Ascherio (2011). "Dietary intake of n-3 and n-6 fatty acids and the risk of clinical depression in women: A 10-y prospective follow-up study." American Journal of Clinical Nutrition **93**(6): 1337-1343.

Luger, M., R. Kruschitz, R. Marculescu, C. Klammer, M. Adelfang, M. Kral, F. Hoppichler, F. Langer, G. Prager, K. Schindler and et al. (2015). "Depressive symptoms and associations with low vitamin d levels and adherence to mediterranean diet in morbidly obese patients." **8**: 188

C183 - EMBASE 71907835.

Luke, A. K., E. W. Evans, D. S. Bond and J. G. Thomas (2016). "Associations between omega fatty acid consumption and depressive symptoms among individuals seeking behavioural weight loss treatment." Obesity Science and Practice **2**(1): 75-82.

Lyoo, I. K., S. Yoon, T. S. Kim, J. Hwang, J. E. Kim, W. Won, S. Bae and P. F. Renshaw (2012). "A randomized, double-blind placebo-controlled trial of oral creatine monohydrate augmentation for enhanced response to a selective serotonin reuptake inhibitor in women with major depressive disorder." American Journal of Psychiatry **169**(9): 937-945.

Mac Giollabhui, N., D. Mischoulon, B. W. Dunlop, B. Kinkead, P. J. Schettler, R. T. Liu, O. I. Okereke, S. Lamon-Fava, M. Fava and M. H. Rapaport (2023). "Individuals with depression exhibiting a pro-inflammatory phenotype receiving omega-3 polyunsaturated fatty acids experience improved motivation-related cognitive function: Preliminary results from a randomized controlled trial." Brain, Behavior, and Immunity - Health **32 C7 - 100666**.

Mahboobi, S., M. Ghasvarian, H. Ghaem, H. Alipour, S. Alipour and M. H. Eftekhari (2022). "Effects of probiotic and magnesium co-supplementation on mood, cognition, intestinal barrier function and inflammation in individuals with obesity and depressed mood: A randomized, double-blind placebo-controlled clinical trial." Frontiers in Nutrition **9 C7 - 1018357**.

Majeed, M., K. Nagabhushanam, A. Murali, D. T. Vishwanathan, R. V. Mamidala and L. Mundkur (2024). "A Standardized Withania somnifera (Linn.) Root Extract with Piperine Alleviates the Symptoms of Anxiety and Depression by Increasing Serotonin Levels: A Double-Blind, Randomized, Placebo-Controlled Study." Journal of Integrative and Complementary Medicine **30**(5): 459-468.

Majmasanaye, M., M. Mehrpooya, H. Amiri and A. Eshraghi (2024). "Discovering the Potential Value of Coenzyme Q10 as an Adjuvant Treatment in Patients with Depression." Journal of Clinical Psychopharmacology **44**(3): 232-239.

Malaguarnera, G., G. Bertino, G. Chisari, M. Motta, M. Vecchio, M. Vacante, F. Caraci, C. Greco, F. Drago, G. Nunnari and et al. (2016). "Silybin supplementation during HCV therapy with pegylated interferon-a plus ribavirin reduces depression and anxiety and increases work ability." **16**(1).

Mannel, M., U. Kuhn, U. Schmidt, M. Ploch and H. Murck (2010). "St. John's wort extract LI160 for the treatment of depression with atypical features - A double-blind, randomized, and placebo-controlled trial." Journal of Psychiatric Research **44**(12): 760-767.

Mao, J. J., S. X. Xie, J. Zee, I. Soeller, Q. S. Li, K. Rockwell and J. D. Amsterdam (2015). "Rhodiola rosea versus sertraline for major depressive disorder: A randomized placebo-controlled trial." Phytomedicine **22**(3): 394-399.

Marangell, L. B., J. M. Martinez, H. A. Zboyan, B. Kertz, H. F. S. Kim and L. J. Puryear (2003). "A double-blind, placebo-controlled study of the omega-3 fatty acid docosahexaenoic acid in the treatment of major depression." American Journal of Psychiatry **160**(5): 996-998.

Maroufi, M., F. Kianvash and M. R. Alavi (2010). "Comparison of Citrus aurantium and imipramine for treatment of major depressive disorder." European Neuropsychopharmacology **20**: S369.

Masoumi, S. Z., F. Kazemi, S. Tavakolian, A. Rahimi, K. Oshvandi, A. Soltanian and F. Shobeiri (2016). "Effect of citalopram in combination with omega-3 on depression in post-menopausal women: A triple blind randomized controlled trial." Journal of Clinical and Diagnostic Research **10**(10): QC01-QC05.

Matthews, J., S. J. Torres, C. M. Milte, I. Hopkins, S. Kukuljan, C. A. Nowson and R. M. Daly (2020). "Effects of a multicomponent exercise program combined with calcium–vitamin D3-enriched milk on health-related quality of life and depressive symptoms in older men: secondary analysis of a randomized controlled trial." European Journal of Nutrition **59**(3): 1081-1091.

Mayers, A. G., D. S. Baldwin, R. Dyson, R. W. Middleton and A. Mustapha (2003). "Use of St. John's wort (Hypericum perforatum L) in members of a depression self-help organisation: A 12-week open prospective pilot study using the HADS scale." Primary Care Psychiatry **9**(1): 15-20.

Mazaherioun, M., A. Saedisomeolia, M. H. Javanbakht, F. Koohdani, M. Zarei, S. Ansari, F. Khoshkhoo Bazargani and M. Djalali (2018). "Long chain n-3 fatty acids improve depression syndrome in type 2 diabetes mellitus." Iranian Journal of Public Health **47**(4): 575-583.

Mazereeuw, G., N. Herrmann, P. I. Oh, D. W. L. Ma, C. T. Wang, A. Kiss and K. L. Lanctôt (2016). "Omega-3 fatty acids, depressive symptoms, and cognitive performance in patients with coronary artery disease." Journal of Clinical Psychopharmacology **36**(5): 436-444.

Mazidi, M., M. Shemshian, S. H. Mousavi, A. Norouzy, T. Kermani, T. Moghiman, A. Sadeghi, N. Mokhber, M. Ghayour-Mobarhan and G. A. Ferns (2016). "A double-blind, randomized and placebo-controlled trial of Saffron (Crocus sativus L.) in the treatment of anxiety and depression." **13**(2): 195‐199

C193 - PUBMED 27101556,EMBASE 610483123.

Mazloom, Z., M. Ekramzadeh and N. Hejazi (2013). "Efficacy of supplementary vitamins C and E on anxiety, depression and stress in type 2 diabetic patients: A randomized, single-blind, placebo-controlled trial." Pakistan Journal of Biological Sciences **16**(22): 1597-1600.

McKetin, R., O. M. Dean, A. Turner, P. J. Kelly, B. Quinn, D. I. Lubman, P. Dietze, G. Carter, P. Higgs, A. L. Baker and et al. (2019). "A study protocol for the N-ICE trial: a randomised double-blind placebo-controlled study of the safety and efficacy of N-acetyl-cysteine (NAC) as a pharmacotherapy for methamphetamine ("ice") dependence." **20**(1): 325

C323 - PUBMED 31164169.

Mech, A. W. and A. Farah (2016). "Correlation of clinical response with homocysteine reduction during therapy with reduced b vitamins in patients with mdd who are positive for MTHFR C677T or A1298C polymorphism: A Randomized, double-blind, placebo-controlled study." Journal of Clinical Psychiatry **77**(5): 668-671.

Mech, A. W. and B. A. Farah (2016). "Reduced B vitamin therapy for MDD in MTHFR C677t/A1298c patients-clinical response correlates with homocysteine reduction: A double-blind, placebo-controlled study." CNS Spectrums **21**(1): 116-117.

Mehdi, S., K. Manohar, A. Shariff, N. Kinattingal, S. U. D. Wani, S. Alshehri, M. T. Imam, F. Shakeel and K. L. Krishna (2023). "Omega-3 Fatty Acids Supplementation in the Treatment of Depression: An Observational Study." Journal of Personalized Medicine **13**(2 C7 - 224).

Mehdi, S. M., S. E. Atlas, S. Qadir, D. Musselman, S. Goldberg, J. M. Woolger, R. Corredor, M. H. Abbas, L. Arosemena, S. Caccamo and et al. (2017). "Double-blind, randomized crossover study of intravenous infusion of magnesium sulfate versus 5% dextrose on depressive symptoms in adults with treatment-resistant depression." **71**(3): 204‐211

C203 - PUBMED 27862658,EMBASE 613942099.

Mehdi, S. M. A., S. E. Atlas, S. Qadir, D. Musselman, S. Goldberg, J. M. Woolger, R. Corredor, M. H. Abbas, L. Arosemena, S. Caccamo, C. S. G. Campbell, A. Farooqi, J. Gao, J. Konefal, L. C. Lages, L. Lantigua, J. Lopez, V. Padilla, A. Rasul, A. M. Ray, H. G. Simões, E. Tiozzo and J. E. Lewis (2017). "Double-blind, randomized crossover study of intravenous infusion of magnesium sulfate versus 5% dextrose on depressive symptoms in adults with treatment-resistant depression." Psychiatry and Clinical Neurosciences **71**(3): 204-211.

Melzer, J., R. Brignoli, M. E. Keck and R. Saller (2010). "A hypericum extract in the treatment of depressive symptoms in outpatients: an open study." Forschende Komplementärmedizin (2006) **17**(1): 7-14.

Merens, W., L. Booij, R. Markus, F. G. Zitman, W. Onkenhout and A. J. Van der Does (2005). "The effects of a diet enriched with alpha-lactalbumin on mood and cortisol response in unmedicated recovered depressed subjects and controls." **94**(3): 415‐422

C413 - PUBMED 16176613.

Mesbahi, S. F., A. M. Sadegh, E. M. Asl, H. Boostani and N. S. Irani (2025). "Effectiveness of Ginseng on the Symptoms of Depression and Anxiety in Patients Referred to Addiction Treatment Clinics in Ahvaz: A Randomized Clinical Trial." Jundishapur Journal of Natural Pharmaceutical Products **20**(1).

Mikola, T., S. M. Lehto, K. Honkalampi, M. Valkonen-Korhonen, H. Koivumaa-Honkanen, T. Tolmunen, V. Laukkanen, M. Pakarinen and A. Ruusunen (2024). "A follow-up study with a double-blinded, randomized controlled vitamin D supplementation trial in patients with major depressive episode (DepFuD): a study protocol and baseline characteristics." **12**(10): 8454‐8469

C8453 - PUBMED 39479665.

Millar, C. L., A. Wolfe, K. Baldyga, A. B. Dufour and L. A. Lipsitz (2025). "Berries and Steps: a protocol of a randomized, placebo-controlled pilot study testing freeze-dried blueberry powder in sedentary older adults with mild depressive symptoms." Nutrition Journal **24**(1 C7 - 87).

Mirzavandi, F., S. Babaie, S. Rahimpour, E. Razmpoosh, N. Talenezhad, S. M. Aghaei Zarch and H. Mozaffari-Khosravi (2020). "The effect of high dose of intramuscular vitamin D supplement injections on depression in patients with type 2 diabetes and vitamin D deficiency: A randomized controlled clinical trial." Obesity Medicine **17**.

Mischoulon, D., C. Best-Popescu, M. Laposata, W. Merens, J. L. Murakami, S. L. Wu, G. I. Papakostas, C. M. Dording, S. B. Sonawalla, A. A. Nierenberg, J. E. Alpert and M. Fava (2008). "A double-blind dose-finding pilot study of docosahexaenoic acid (DHA) for major depressive disorder." European Neuropsychopharmacology **18**(9): 639-645.

Mischoulon, D., B. W. Dunlop, B. Kinkead, P. J. Schettler, S. Lamon-Fava, J. J. Rakofsky, A. A. Nierenberg, A. J. Clain, T. M. Crowe, A. Wong, J. C. Felger, L. Sangermano, T. R. Ziegler, C. Cusin, L. B. Fisher, M. Fava and M. H. Rapaport (2022). "Omega-3 Fatty Acids for Major Depressive Disorder With High Inflammation: A Randomized Dose-Finding Clinical Trial." Journal of Clinical Psychiatry **83**(5 C7 - 21m14074).

Mischoulon, D. and M. Fava (2000). "Docosahexanoic acid and ω-3 fatty acids in depression." Psychiatric Clinics of North America **23**(4): 785-794.

Mischoulon, D., G. I. Papakostas, C. M. Dording, A. H. Farabaugh, S. B. Sonawalla, A. M. Agoston, J. Smith, E. C. Beaumont, L. E. Dahan, J. E. Alpert, A. A. Nierenberg and M. Fava (2009). "A double-blind, randomized controlled trial of ethyl-eicosapentaenoate for major depressive disorder." Journal of Clinical Psychiatry **70**(12): 1636-1644.

Mischoulon, D., L. H. Price, L. L. Carpenter, A. R. Tyrka, G. I. Papakostas, L. Baer, C. M. Dording, A. J. Clain, K. Durham and R. Walker (2014). "A double-blind, randomized, placebo-controlled clinical trial of S-adenosyl-L-methionine (SAMe) versus escitalopram in major depressive disorder." J Clin Psychiatry **75**(4): 370-376.

Missaoui, M., A. Hassine, S. Naija, J. Emna and S. Ben Amor (2021). "Effect of vitamin D supplementation on anxiety-depressive disorder at six months of stroke: A randomized clinical trial." European Journal of Neurology **28**(SUPPL 1): 864.

Mitchell, M., H. Bradley, N. M. Blampied, R. T. Mulder and J. J. Rucklidge (2025). "Protective effect of micronutrients used to treat antenatal depression on rates of postnatal depression at six months: A secondary analysis of NUTRIMUM." Journal of Affective Disorders **388 C7 - 119560**.

Moazen-Zadeh, E., S. H. Abbasi, H. Safi-Aghdam, N. Shahmansouri, A. Arjmandi-Beglar, A. Hajhosseinn Talasaz, A. Salehiomran, S. Forghani and S. Akhondzadeh (2018). "Effects of Saffron on Cognition, Anxiety, and Depression in Patients Undergoing Coronary Artery Bypass Grafting: A Randomized Double-Blind Placebo-Controlled Trial." Journal of Alternative and Complementary Medicine **24**(4): 361-368.

Mocking, R. J. T., J. Assies, M. Bot, E. H. J. M. Jansen, A. H. Schene and F. Pouwer (2012). "Biological Effects of Add-On Eicosapentaenoic Acid Supplementation in Diabetes Mellitus and Co-Morbid Depression: A Randomized Controlled Trial." PLoS ONE **7**(11 C7 - e49431).

Mohajeri, S., M. Hassanpour Moghadam, A. Talaei and S. Sajadi Tabassi (2014). "Effect of crocin on major depressive disorder: a double blind clinical trial." **115**: 187

C183 - EMBASE 71549613.

Mohamed, S.-A., G. M. Khafagy, I. T. A. Alsaid and H. A. Hussein (2024). "The gut brain axis, effect of dietary changes and probiotics supplement on depression symptoms." **30**.

Mohsenpour, M. A., F. Mohammadi, Z. Sohrabi, M. Niakousari, M. Jeddi and M. H. Eftekhari (2025). "Mental Health and Health-related Quality of Life Response to Curcumin-Probiotic Drink Powder Co-supplementation in Adults with Overweight/Obesity and Metabolic Syndrome: a Four-arm Randomized, Double-blind, Placebo-controlled, Clinical Trial." British Journal of Nutrition.

Mokhber, N., M. Namjoo, F. Tara, H. Boskabadi, M. P. Rayman, M. Ghayour-Mobarhan, A. Sahebkar, M. R. Majdi, S. Tavallaie, M. Azimi-Nezhad and et al. (2011). "Effect of supplementation with selenium on postpartum depression: a randomized double-blind placebo-controlled trial." **24**(1): 104‐108

C103 - PUBMED 20528216.

Moludi, J., M. Alizadeh, M. H. S. Mohammadzad and M. Davari (2019). "The Effect of Probiotic Supplementation on Depressive Symptoms and Quality of Life in Patients after Myocardial Infarction: Results of a Preliminary Double-Blind Clinical Trial." Psychosomatic Medicine **81**(9): 770-777.

Moludi, J., H. Khedmatgozar, S. M. Nachvak, H. Abdollahzad, M. Moradinazar and A. Sadeghpour tabaei (2022). "The effects of co-administration of probiotics and prebiotics on chronic inflammation, and depression symptoms in patients with coronary artery diseases: a randomized clinical trial." Nutritional Neuroscience **25**(8): 1659-1668.

Moniello, G., I. Bonfitto, F. Simonetti, S. Dimalta, M. R. Rizzo and A. Bellomo (2024). "Role of L-Arginine supplementation in Long Covidrelated Fatigue and Depression in Elderly Outpatients." European Psychiatry **67**: S106.

Moore, K., C. F. Hughes, L. Hoey, M. Ward, C. Cunningham, A. M. Molloy, J. J. Strain, K. McCarroll, M. C. Casey, F. Tracey, E. Laird, M. O'Kane and H. McNulty (2019). "B-vitamins in Relation to Depression in Older Adults Over 60 Years of Age: The Trinity Ulster Department of Agriculture (TUDA) Cohort Study." Journal of the American Medical Directors Association **20**(5): 551-557.e551.

Moosavi, S. M., M. Ahmadi, M. Amini and B. Vazirzadeh (2014). "The effects of 40 and 80 mg hydro- alcoholic extract of crocus sativus in the treatment of mild to moderate depression." Journal of Mazandaran University of Medical Sciences **24**(113): 47-53.

Moschonis, G., K. Sarapis, S. Resciniti, R. Hall, K. Yim, M. Tonkovic, C. Fitzgerald, F. Anixiadis, Q. Nhu Dinh, M. Hale, B. Wright, M. Pane, C. J. Tuck and J. R. Biesiekierski (2024). "The effectiveness of a Lactobacillus probiotic on measures of psychosocial health in adults diagnosed with subthreshold depression: a double-blind, randomised, placebo-controlled trial." Proceedings of the Nutrition Society **83**(OCE1): E58.

Moschonis, G., K. Sarapis, S. Resciniti, R. Hall, K. Yim, M. Tonkovic, C. Fitzgerald, F. Anixiadis, A. Vinh, Q. N. Dinh, R. A. Cronin, M. W. Hale, B. J. Wright, M. Pane, C. J. Tuck and J. R. Biesiekierski (2024). "Evaluation of a probiotic blend on psychosocial health and biomarkers of inflammatory, immune and stress response in adults with subthreshold depression: A double-blind, randomised, placebo-controlled trial." British Journal of Nutrition.

Moshiri, E., A. A. Basti, A. A. Noorbala, A. H. Jamshidi, S. Hesameddin Abbasi and S. Akhondzadeh (2006). "Crocus sativus L. (petal) in the treatment of mild-to-moderate depression: A double-blind, randomized and placebo-controlled trial." Phytomedicine **13**(9-10): 607-611.

Mousa, A., N. Naderpoor, M. P. J. de Courten and B. de Courten (2018). "Vitamin D and symptoms of depression in overweight or obese adults: A cross-sectional study and randomized placebo-controlled trial." Journal of Steroid Biochemistry and Molecular Biology **177**: 200-208.

Mozaffari-Khosravi, H., L. Nabizade, S. M. Yassini-Ardakani, H. Hadinedoushan and K. Barzegar (2013). "The effect of 2 different single injections of high dose of vitamin D on improving the depression in depressed patients with vitamin D deficiency: A randomized clinical trial." Journal of Clinical Psychopharmacology **33**(3): 378-385.

Mueller, B. M. (1998). "St. John's wort for depressive disorders: Results of an outpatient study with the hypericum preparation HYP 811." Advances in Therapy **15**(2): 109-116.

Mueller, M., R. Ganesh and S. Bonnes (2020). "Gut Health = Mental Health? The Impact of Diet and Dietary Supplements on Mood Disorders." Current Nutrition Reports **9**(4): 361-368.

Nabimeybodi, R., A. M. Ranjbar, A. Javaheri, B. Heydari, F. Hosseini, S. Kardan and V. Ramezani (2024). "Assessment of the Effect of Grape Molasses on Preventing Postpartum Depression in Women Referred to Shahid Sadoughi Hospital in Yazd." Traditional and Integrative Medicine **9**: 4.

Nagayasu, Y., D. Fujita, A. Daimon, M. Nunode, M. Sawada, T. Sano and M. Ohmichi (2021). "Possible prevention of post-partum depression by intake of omega-3 polyunsaturated fatty acids and its relationship with interleukin 6." Journal of Obstetrics and Gynaecology Research **47**(4): 1371-1379.

Najafabady, M. T., P. Y. Baghbadoranee, E. Moghimipour, M. H. Haghighizadeh and H. Boostani (2019). "Comparison the effect of Echium amoenum extract with fluoxetine on depression in menopausal women. A double-blind randomized controlled trial." Asian Journal of Pharmaceutics **13**(3): 271-275.

NCT00005013 (2000). A Placebo Controlled Clinical Trial of a Standardized Extract of Hypericum Perforatum in Major Depressive Disorder. clinicaltrials.gov.

NCT00042380 (2002). A Phase 2A Study of Soy Isoflavones in the Treatment of Major Depression. clinicaltrials.gov.

NCT00066859 (2003). A Phase III Double-Blind Randomized Trial Comparing Sertraline (Zoloft) And Hypericum Perforatum (St. John's Wort) In Cancer Patients With Mild To Moderate Depression. clinicaltrials.gov.

NCT00067301 (2003). PUFA Augmentation in Treatment of Major Depression. clinicaltrials.gov.

NCT00122954 (2005). Fish Oil as an Adjunct Therapy for Depression in Multiple Sclerosis. clinicaltrials.gov.

NCT00223236 (2005). A Double-blind, Placebo-controlled Trial of Citicoline add-on Therapy in Patients With a History of Mania or Hypomania and Cocaine Abuse/Dependence. clinicaltrials.gov.

NCT00226356 (2005). Phase 4 Study: An Open Study of the Efficacy and Tolerability in Unipolar Depression of Augmentation of the One-carbon Cycle With L-methionine, Betaine and Folate. clinicaltrials.gov.

NCT00256412 (2005). Essential Fatty Acids in Management of MDD - A Pilot Study. clinicaltrials.gov.

NCT00480207 (2007). Randomized Double-blind Controlled Clinical Trial to Assess the Impact of Folic Acid and Omega-3 Fatty Acids Supplementation on the Severity of Depressive Symptoms in Older Adults With Identified Depression. clinicaltrials.gov.

NCT00539513 (2007). A Double-Blind Study of N-Acetylcysteine Augmentation in Serotonin Reuptake Inhibitor-Refractory Obsessive-Compulsive Disorder and Depression. clinicaltrials.gov.

NCT00693680 (2008). The Role of Zinc in Therapy of Unipolar Depression. clinicaltrials.gov.

NCT00711971 (2008). Does EPA or DHA Prevent Depressive Symptoms in Pregnancy and Postpartum? clinicaltrials.gov.

NCT00729755 (2008). Efficacy and Safety of Augmentation of Creatine for the Patients With Major Depressive Disorder. clinicaltrials.gov.

NCT00816322 (2008). The Effect of Fish Oil in Major Depressive Disorder: a Double-blind Placebo-controlled Monotherapy Trail and a RCT for Recurrence Prevention. clinicaltrials.gov.

NCT00861978 (2009). St. John's Wort Extract LI 160 for the Treatment of Atypical Depression. clinicaltrials.gov.

NCT00955955 (2009). TRD - 2: A Double-Blind, Placebo Controlled Study of 6(S)-5-MTHF Among SSRI-Resistant Outpatients With Major Depressive Disorder (MDD). clinicaltrials.gov.

NCT00997490 (2009). Phase III Placebo-controlled, Randomised, Single-centre, Double-blind Study of the Antidepressant Efficacy of a Combination Herbal Drug Preparation. clinicaltrials.gov.

NCT01022632 (2009). A Randomized, Active Controlled, Open Label, Parallel Group Study to Compare the Efficacy of Extract of Curcuma Longa (Turmeric) With Fluoxetine and to Study Its Effect as an Add on Therapy to Fluoxetine in Patients of Depression. clinicaltrials.gov.

NCT01175616 (2010). Creatine Augmentation in Female & Male Veterans With Selective Serotonin Reuptake Inhibitor-Resistant Major Depressive Disorder. clinicaltrials.gov.

NCT01185574 (2010). Vitamin D for Improving Metabolic Control and Depressive Symptoms in Women With Diabetes; The Sunshine Study. clinicaltrials.gov.

NCT01223729 (2010). Acetyl-L-Carnitine Reduces Depression and Improves Quality of Life in Patients With Minimal Hepatic Encephalopathy. clinicaltrials.gov.

NCT01390662 (2011). Phase 4 Study of Vitamin D3 Supplementation for Outcomes in Patients With Unipolar Depression. clinicaltrials.gov.

NCT01416818 (2011). A Randomized, Double-Blind, Placebo-Controlled Study of "Xiaoyao Pill" Treatment of Depression in Patients With Parkinson's Disease. clinicaltrials.gov.

NCT01462058 (2011). The Role of Vitamin D Supplementation on Well Being and Symptoms of Depression During the Winter Season in Health Service Staff. clinicaltrials.gov.

NCT01750359 (2012). The Efficacy and Safety of Adjunctive Curcumin for Treatment of Depression: A Randomized, Double-blind, Placebo-controlled Study. clinicaltrials.gov.

NCT02189915 (2014). Open-Label Creatine Monohydrate Supplementation in Depressed Female Methamphetamine Users. clinicaltrials.gov.

NCT02223299 (2014). A Randomized Double-Blind Placebo-Controlled Pilot Study of L-tyrosine and L-tryptophan in Depressed Patients Receiving L-methylfolate and and SSRI. clinicaltrials.gov.

NCT02359266 (2015). Clinical Trial Investigating the Role of Vitamin D in the Treatment of Depression in Patients With Chronic Liver Disease. clinicaltrials.gov.

NCT02381600 (2015). An Evaluation of the Effects of Vitamin D Supplementation on Specific Cognitive Functions and Depressive Symptoms of Older Subjects With Low Levels of This Vitamin. clinicaltrials.gov.

NCT02568878 (2015). An Open-Label Pilot Study of Creatine for Depressed Male and Female Methamphetamine Users. clinicaltrials.gov.

NCT02793648 (2015). Phase II, Double-blind, Randomized, 1-way Cross-over, to Investigate the Effectiveness of the Combination of Ascorbic Acid (Vitamin C) and Tocopherol (Vitamin E) Versus Placebo for the Treatment of Depressive Disorders in Elderly. clinicaltrials.gov.

Nct (2002). "Examination of a Soy-Based Supplement for Major Depression."

Nct (2007). "Folate Augmentation of Treatment - Evaluation for Depression: a Randomised Controlled Trial."

Nct (2007). "Omega-3 Fatty Acids as Adjunct Treatment for Major Depressive Disorder."

Nct (2007). "Omega-3 Fatty Acids for Treating Adults With Major Depression."

Nct (2007). "St. John's Wort And Kava In The Treatment Of Major Depressive Disorder With Comorbid Anxiety."

Nct (2009). "Comparison of Vitamin B12 Supplementation to Selective Serotonin Reuptake Inhibitor (SSRI) Versus SSRI Antidepressant Treatment Alone."

Nct (2010). "Rhodiola Rosea Therapy of Major Depressive Disorder."

Nct (2010). "Vitamin B6, B12, Folic Acid and Exercise in Parkinson's Disease."

Nct (2012). "N-3 Polyunsaturated Fatty Acids in the Prevention and Treatment for IFN-induced Depression."

Nct (2015). "Effect of Lactobacillus Plantarum 299v Supplementation on Major Depression Treatment."

Nct (2016). "Selenium as Augmentation Treatment for Sertraline Resistant Major Depression."

Nct (2018). "Role of Omega-3 Polyunsaturated Fatty Acid in the Management of Major Depressive Disorder."

Nct (2019). "Effects of Nutrients Supplementation in Antidepressant Treated Depressive Disorder Patients."

Nct (2021). "Cholecalciferol on Depressive Symptoms in Type 2 Diabetes Mellitus Patients."

Nct (2021). "RCT of Adjunctive Curcumin and the Meru Health Program."

Nct (2023). "Supplementation and Depressive Symptomatology Incidence Among Saudi Arabian Adults."

Nct (2024). "Comparison of Krill and Fish Oil on Clinical and Biochemical Outcomes in Depression."

Nemets, B. (2006). "A Double-Blind, Parallel, Randomized, Add-on Clinical Trial of Creatine Versus Placebo Added to Antidepressant Treatment of Patients With Major Depressive Episode."

Nemets, B. and J. Levine (2013). "A pilot dose-finding clinical trial of creatine monohydrate augmentation to SSRIs/SNRIs/NASA antidepressant treatment in major depression." International Clinical Psychopharmacology **28**(3): 127-133.

Nemets, B., Z. Stahl and R. H. Belmaker (2002). "Addition of omega-3 fatty acid to maintenance medication treatment for recurrent unipolar depressive disorder." American Journal of Psychiatry **159**(3): 477-479.

Nikfarjam, M., I. Goudarzi, S. Heidari, L. R. Vardanjani and N. Parvin (2012). "Effect of Ginkgo biloba pill on patients with major depression treated with electroconvulsive therapy." Journal of Mazandaran University of Medical Sciences **22**(88): 64-72.

Nikfarjam, M., R. Rakhshan and H. Ghaderi (2017). "Comparison of effect of Lavandula officinalis and venlafaxine in treating depression: A double blind clinical trial." Journal of Clinical and Diagnostic Research **11**(7): KC01-KC04.

Nikolova, V., A. Cleare, A. Young and J. Stone (2023). "Probiotics as Adjunctive Treatment in Major Depressive Disorder: Estimates of Treatment Effect and Underlying Mechanisms From a Double-Blind Placebo-Controlled Randomised Pilot Trial." BJPsych Open **9**: S64-S65.

Nikolova, V. L., A. J. Cleare, A. H. Young and J. M. Stone (2023). "Acceptability, Tolerability, and Estimates of Putative Treatment Effects of Probiotics as Adjunctive Treatment in Patients with Depression: A Randomized Clinical Trial." JAMA Psychiatry **80**(8): 842-847.

Nikolova, V. L., A. J. Cleare, A. H. Young and J. M. Stone (2025). "Exploring the mechanisms of action of probiotics in depression: Results from a randomized controlled pilot trial." Journal of Affective Disorders **376**: 241-250.

Ning, H., H. Zhou, J. Ren, G. Zhou, N. Yang, Z. Wang, C. Yuan, Z. Tian, J. Chen, L. Shen, H. Zheng, Y. Zhao, H. Wang, W. Liu and Z. Liu (2022). "Zishen pingchan granules combined with pramipexole in the improvement of depressive symptoms in Parkinson's disease: a prospective, multicenter, randomized, double-blind, controlled clinical study." J Transl Med **20**(1): 357.

Ning, H., H. Zhou, N. Yang, J. Ren, H. Wang, W. Liu and Y. Zhao (2023). "Effect of Zishen pingchan granules combined with pramipexole on serum BDNF, IL-1β, IL-6, CRP, TNF-α levels in depressed patients with Parkinson's disease: Results of a randomized, double-blind, controlled study." Experimental Gerontology **182**.

Nishi, D., Y. Matsuoka and K. Su (2015). Are omega-3 PUFAs effective for depression during pregnancy in countries where fish consumptions are high? PSYCHOTHERAPY AND PSYCHOSOMATICS, KARGER ALLSCHWILERSTRASSE 10, CH-4009 BASEL, SWITZERLAND.

Nishi, D., K. P. Su, K. Usuda, J. P. C. Chang, K. Hamazaki, T. Ishima, K. Hashimoto and Y. J. Matsuoka (2019). "The effect of omega-3 fatty acids for depressive symptoms among pregnant women in Japan and Taiwan: A randomized, double-blind, placebo-controlled trial and biomarker study." Psychotherapy and Psychosomatics **88**: 96-97.

Nishi, D., K. P. Su, K. Usuda, J. P. C. Chang, K. Hamazaki, T. Ishima, Y. Sano, H. Ito, K. Isaka, Y. Tachibana, S. Tanigaki, T. Suzuki, K. Hashimoto and Y. J. Matsuoka (2020). "Plasma estradiol levels and antidepressant effects of omega-3 fatty acids in pregnant women." Brain, Behavior, and Immunity **85**: 29-34.

Noorbala, A. A., S. Akhondzadeh, N. Tahmacebi-Pour and A. H. Jamshidi (2005). "Hydro-alcoholic extract of Crocus sativus L. versus fluoxetine in the treatment of mild to moderate depression: A double-blind, randomized pilot trial." Journal of Ethnopharmacology **97**(2): 281-284.

Noori, M., Z. Shateri, S. Babajafari, M. H. Eskandari, K. Parastouei, M. Ghasemi, H. Afshari and M. Samadi (2025). "The effect of probiotic-fortified kefir on depression, appetite, oxidative stress, and inflammatory parameters in Iranian overweight and obese elderly: a randomized, double-blind, placebo-controlled clinical trial." Journal of health, population, and nutrition **44**(1): 30.

Novak, F., M. Vecka, E. Meisnerova, S. Sevela, L. Vavrova, J. Rychlikova, L. Dolezalova, D. Myslivcova, A. Zak, L. Vitek and et al. (2019). "Fish oil supplementation with various lipid emulsions suppresses in vitro cytokine release in home parenteral nutrition patients: a crossover study." **72**: 70‐79

C73 - PUBMED 31759770,EMBASE 2003873276.

Novak, F., M. Vecka, E. Meisnerova, S. Sevela, L. Vavrova, J. Rychlikova, L. Dolezalova, D. Myslivcova, A. Zak, L. Vitek and O. Novakova (2019). "Fish oil supplementation with various lipid emulsions suppresses in vitro cytokine release in home parenteral nutrition patients: a crossover study." Nutrition Research **72**: 70-79.

Nowak, G., M. Siwek, D. Dudek, A. Zieba and A. Pilc (2003). "Effect of zinc supplementation on antidepressant therapy in unipolar depression: A preliminary placebo-controlled study." Polish Journal of Pharmacology **55**(6): 1143-1147.

Odebrecht Vargas Nunes, S., M. Porcu, H. Odebrecht Vargas and M. Urbano (2017). "N-acetylcysteine as an adjunctive treatment to reduce depressive symptom severity when C-reactive protein levels are increased." European Neuropsychopharmacology **27**: S788.

Okronipa, H., S. Adu-Afarwuah, A. Lartey, P. Ashorn, S. A. Vosti, R. R. Young and K. G. Dewey (2018). "Maternal supplementation with small-quantity lipid-based nutrient supplements during pregnancy and lactation does not reduce depressive symptoms at 6 months postpartum in Ghanaian women: a randomized controlled trial." Archives of Women's Mental Health **21**(1): 55-63.

Olagunju, A. E. and H. Gaddey (2020). "Leveraging CAM to treat depression and anxiety." Journal of Family Practice **69**(5): 221-227.

Omidian, M., M. Mahmoudi, M. Abshirini, M. R. Eshraghian, M. H. Javanbakht, M. Zarei, H. Hasani and M. Djalali (2019). "Effects of vitamin D supplementation on depressive symptoms in type 2 diabetes mellitus patients: Randomized placebo-controlled double-blind clinical trial." Diabetes Metab Syndr **13**(4): 2375-2380.

Opiyo, R. O., P. S. Nyasulu, R. K. Koigi, A. Obondo, D. Ogoyi and W. Kogi-Makau (2018). "Effect of fish oil omega-3 fatty acids on reduction of depressive symptoms among HIV-seropositive pregnant women: a randomized, double-blind controlled trial." Annals of General Psychiatry **17**(1 C7 - 49).

Osali, A. and A. Rostami (2023). "Effect of 6 weeks of aerobic training with nanocurcumin consumption on IL1β, nitric oxide, and depression in women with metabolic syndrome." International Journal of Diabetes in Developing Countries **43**(6): 1007-1014.

Padhan, M., D. Mohapatra, B. R. Mishra, R. Maiti and M. Jena (2024). "Efficacy and safety of add-on sarcosine in patients with major depressive disorder: A randomized controlled trial." Journal of Psychiatric Research **178**: 298-304.

Pakseresht, S., H. Boustani, M. E. Azemi, J. Nilsaz, R. Babapour and M. R. Haghdust (2012). "Evaluation of pharmaceutical products of st. john's wort efficacy added on tricyclic antidepressants in treating major depressive disorder: A double blind randomized control trial." Jundishapur Journal of Natural Pharmaceutical Products **7**(3): 106-110.

Panahi, Y., R. Badeli, G. R. Karami, Z. Badeli and A. Sahebkar (2015). "A randomized controlled trial of 6-week Chlorella vulgaris supplementation in patients with major depressive disorder." Complement Ther Med **23**(4): 598-602.

Pancheri, P., P. Scapicchio and R. Delle Chiaie (2002). "A double-blind, randomized parallel-group, efficacy and safety study of intramuscular S-adenosyl-L-methionine 1, 4-butanedisulphonate (SAMe) versus imipramine in patients with major depressive disorder." International Journal of Neuropsychopharmacology **5**(4): 287-294.

Papakostas, G. I., D. Mischoulon, I. Shyu, J. E. Alpert and M. Fava (2010). "S-adenosyl methionine (SAMe) augmentation of serotonin reuptake inhibitors for antidepressant nonresponders with major depressive disorder: a double-blind, randomized clinical trial." **167**(8): 942‐948

C943 - PUBMED 20595412.

Papakostas, G. I., R. C. Shelton, J. M. Zajecka, B. Etemad, K. Rickels, A. Clain, L. Baer, E. D. Dalton, G. R. Sacco, D. Schoenfeld and et al. (2012). "L-methylfolate as adjunctive therapy for SSRI-resistant major depression: results of two randomized, double-blind, parallel-sequential trials." **169**(12): 1267‐1274

C1263 - PUBMED 23212058.

Park, Y., Y. S. Park, S. H. Kim, D. H. Oh and Y. C. Park (2015). "Supplementation of n-3 polyunsaturated fatty acids for major depressive disorder: A randomized, double-blind, 12-week, placebo-controlled trial in Korea." Annals of Nutrition and Metabolism **66**(2-3): 141-148.

Parletta, N., D. Zarnowiecki, J. Cho, A. Wilson, S. Bogomolova, A. Villani, C. Itsiopoulos, B. Meyer, L. Segal and K. O'Dea (2018). "Effects of a Mediterranean-style diet supplemented with fish oil on mental health and quality of life in people with depression." Nutritional Neuroscience **21**: S41.

Parvin, N., S. Farzaneh, M. Nikfarjam, N. Shahinfard and N. Asarzadegan (2011). "The effect of lavandula angustifolia in the treatment of depression." European Psychiatry **26**.

Peet, M. and D. F. Horrobin (2002). "A dose-ranging study of the effects of ethyl-eicosapentaenoate in patients with ongoing depression despite apparently adequate treatment with standard drugs." Archives of General Psychiatry **59**(10): 913-919.

Penckofer, S., M. Ridosh, W. Adams, M. Grzesiak, J. Woo, M. Byrn, J. Kouba, P. Sheean, C. Kordish, R. Durazo-Arvizu and et al. (2022). "Vitamin D Supplementation for the Treatment of Depressive Symptoms in Women with Type 2 Diabetes: a Randomized Clinical Trial." **2022**: 4090807

C4090803 - PUBMED 35280228,EMBASE 2017280621.

Pinto-Sanchez, M. I., G. B. Hall, K. Ghajar, A. Nardelli, C. Bolino, J. T. Lau, F. P. Martin, O. Cominetti, C. Welsh, A. Rieder and et al. (2017). "Probiotic Bifidobacterium longum NCC3001 Reduces Depression Scores and Alters Brain Activity: a Pilot Study in Patients With Irritable Bowel Syndrome." **153**(2): 448‐459.e448

C443 - PUBMED 28483500,EMBASE 617425533.

Piperoglou, M., M. Hopwood and T. R. Norman (2023). "Adjunctive Docosahexaenoic Acid in Residual Symptoms of Depression and Anxiety." Journal of Clinical Psychopharmacology **43**(6): 493-497.

Putranto, R., S. Setiati, M. W. Nasrun, F. Witjaksono, S. Immanuel, I. Subekti, K. Harimurti, A. Siswanto, H. Shatri, S. Suwarto and et al. (2024). "Effects of cholecalciferol supplementation on depressive symptoms, C-peptide, serotonin, and neurotrophin-3 in type 2 diabetes mellitus: a double-blind, randomized, placebo-controlled trial." **4**(3): e1342

C1343 - PUBMED 39816051.

Raeder, M. B., V. M. Steen, S. E. Vollset and I. Bjelland (2007). "Associations between cod liver oil use and symptoms of depression: The Hordaland Health Study." Journal of Affective Disorders **101**(1-3): 245-249.

Rahman, S. T., M. Waterhouse, B. D. Romero, C. Baxter, D. R. English, O. P. Almeida, M. Berk, P. R. Ebeling, B. K. Armstrong, D. S. A. McLeod, G. Hartel, R. L. O'Connell, H. Pham, J. G. Scott, J. C. van der Pols, A. J. Venn, P. M. Webb, D. C. Whiteman and R. E. Neale (2023). "Effect of vitamin D supplementation on depression in older Australian adults." Int J Geriatr Psychiatry **38**(1): e5847.

Rahman, S. T., M. Waterhouse, B. D. Romero, C. Baxter, D. R. English, O. P. Almeida, M. Berk, P. R. Ebeling, B. K. Armstrong, D. S. A. McLeod, G. Hartel, R. L. O’Connell, H. Pham, J. G. Scott, J. C. van der Pols, A. J. Venn, P. M. Webb, D. C. Whiteman and R. E. Neale (2023). "Effect of vitamin D supplementation on depression in older Australian adults." International Journal of Geriatric Psychiatry **38**(1 C7 - e5847).

Rajizadeh, A., H. Mozaffari-Khosravi, M. Yassini-Ardakani and A. Dehghani (2017). "Effect of magnesium supplementation on depression status in depressed patients with magnesium deficiency: A randomized, double-blind, placebo-controlled trial." Nutrition **35**: 56-60.

Ramezani, A., F. Kheirkhah, E. Zabihi, H. Shirafkan and S. Moudi (2022). "Effectiveness of L-carnitine Supplementation to Sertraline for Treatment of Major Depressive Disorder: A Double-blind Randomized Placebo-controlled Trial." Global Journal of Medical Pharmaceutical and Biomedical Update **17**.

Ranjbar, E., J. Shams, M. Sabetkasaei, M. M-Shirazi, B. Rashidkhani, A. Mostafavi, E. Bornak and J. Nasrollahzadeh (2014). "Effects of zinc supplementation on efficacy of antidepressant therapy, inflammatory cytokines, and brain-derived neurotrophic factor in patients with major depression." Nutritional Neuroscience **17**(2): 65-71.

Ranjbar, M., A. Firoozabadi, A. Salehi, Z. Ghorbanifar, M. M. Zarshenas, K. Sadeghniiat-Haghighi and H. Rezaeizadeh (2018). "Effects of Herbal combination (Melissa officinalis L. and Nepeta menthoides Boiss. & Buhse) on insomnia severity, anxiety and depression in insomniacs: Randomized placebo controlled trial." Integrative Medicine Research **7**(4): 328-332.

Rao, G. P., S. Kariya, P. Chaudhari and M. Bhondave (2024). "A STUDY TO ASSESS EFFICACY AND SAFETY OF ADD-ON ACETYL-L-CARNITINE AND AGMATINE (REJIYANA®) WITH STANDARD OF CARE (SOC) TO VALIDATE VISIBLE CHANGES IN MAJOR DEPRESSIVE DISORDER A RANDOMIZED CONTROLLED TRIAL: REVIVE TRIAL." International Journal of Medicine and Public Health **14**(2): 597-604.

Rashmi, K. and P. Madhuri (2017). "Evaluation of therapeutic effect of Vachadi ghrita in patients of mild to moderate depression." International Journal of Research in Ayurveda and Pharmacy **8**: 137-140.

Rastegar, M., N. J. Shoorab, R. Salari and A. Saki (2024). "The Effect of Oral Probiotic Capsules on Postpartum Depression in Primiparous Women: A Triple-blind Randomized Control trial." Iranian Journal of Obstetrics, Gynecology and Infertility **27**(3): 64-75.

Ravi, S., H. Khalili, L. Abbasian, M. Arbabi and P. Ghaeli (2016). "Effect of Omega-3 Fatty Acids on Depressive Symptoms in HIV-Positive Individuals: A Randomized, Placebo-Controlled Clinical Trial." Annals of Pharmacotherapy **50**(10): 797-807.

Raza, M. L., S. T. Hassan, S. Jamil, W. Fatima and M. Fatima (2025). "Nutritional interventions in depression: The role of vitamin D and omega-3 fatty acids in neuropsychiatric health." Clinical Nutrition **45**: 270-280.

Rees, A. M., M. P. Austin and G. B. Parker (2008). "Omega-3 fatty acids as a treatment for perinatal depression: Randomized double-blind placebo-controlled trial." Australian and New Zealand Journal of Psychiatry **42**(3): 199-205.

Rees, A. M., G. Parker and M. P. Austin (2006). "A randomized controlled trial to assess the efficacy of omega-3 fatty acids as a treatment for depression during the perinatal period." Acta Neuropsychiatr **18**(6): 267-268.

Regueme, S. C., R. Ostan, A. Pinto, L. Valentini, J. Durrieu, C. Blanc-Bisson, M. L. Donini, H. Lochs, C. Franceschi and I. Bourdel-Marchasson (2016). "Effects of a 2-month dietand nutraceutical intervention on quality of life and depression with respect to inflammaging: Ristomed, an open label intervention trial." Clinical Nutrition **35**: S84.

Reininghaus, E., K. Riedrich, S. Bengesser, S. Mörkl, C. Hamm, R. Queissner, D. Otti, H. Schöggl, T. Lahousen, W. Wurm, N. Dalkner, F. Fellendorf, M. Platzer, T. Bastiaanssen and B. Reininghaus (2020). "Intake of probiotic supplements for the treatment of depression." Bipolar Disorders **22**: 101.

Reininghaus, E. Z., M. Platzer, A. Kohlhammer-Dohr, C. Hamm, S. Mörkl, S. A. Bengesser, F. T. Fellendorf, T. Lahousen-Luxenberger, B. Leitner-Afschar, H. Schöggl, D. Amberger-Otti, W. Wurm, R. Queissner, A. Birner, V. S. Falzberger, A. Painold, W. Fitz, J. Wagner-Skacel, M. Brunnmayr, A. Rieger, A. Maget, R. Unterweger, K. Schwalsberger, B. Reininghaus, M. Lenger, T. F. S. Bastiaanssen and N. Dalkner (2020). "PROVIT: Supplementary Probiotic Treatment and Vitamin B7 in Depression-A Randomized Controlled Trial." Nutrients **12**(11).

Rocco, A. D., J. D. Rogers, R. Brown, P. Werner and T. Bottiglieri (2000). "S-adenosyl-methionine improves depression in patients with Parkinson's disease in an open-label clinical trial." Movement Disorders **15**(6): 1225-1229.

Rodríguez-Morán, M., F. Guerrero-Romero, J. Barragán-Zuñiga, C. I. Gamboa-Gómez, Y. Weyman-Vela, M. Arce-Quiñones, L. E. Simental-Mendía and G. Martínez-Aguilar (2024). "Combined oral supplementation with magnesium plus vitamin D alleviates mild to moderate depressive symptoms related to long-COVID: an open-label randomized, controlled clinical trial." Magnesium Research **37**(3): 49-57.

Rolf, L., A. H. Muris, Y. Bol, J. Damoiseaux, J. Smolders and R. Hupperts (2016). "High dose vitamin D3 supplementation does not ameliorate depressive symptoms in relapsing remitting multiple sclerosis: Results of a randomized placebo-controlled trial." Multiple Sclerosis **22**: 379.

Romijn, A. R., J. J. Rucklidge, R. G. Kuijer and C. Frampton (2017). "A double-blind, randomized, placebo-controlled trial of Lactobacillus helveticus and Bifidobacterium longum for the symptoms of depression." Australian and New Zealand Journal of Psychiatry **51**(8): 810-821.

Rondanelli, M., A. Giacosa, A. Opizzi, C. Pelucchi, C. La Vecchia, G. Montorfano, M. Negroni, B. Berra, P. Politi and A. M. Rizzo (2011). "Long chain omega 3 polyunsaturated fatty acids supplementation in the treatment of elderly depression: Effects on depressive symptoms, on phospholipids fatty acids profile and on health-related quality of life." Journal of Nutrition, Health and Aging **15**(1): 37-44.

Rondanelli, M., A. Giacosa, A. Opizzi, C. Pelucchi, C. L. Vecchia, G. Montorfano, M. Negroni, B. Berra, P. Politi and A. M. Rizzo (2010). "Effect of omega-3 fatty acids supplementation on depressive symptoms and on health-related quality of life in the treatment of elderly women with depression: A double-blind, placebo-controlled, randomized clinical trial." Journal of the American College of Nutrition **29**(1): 55-64.

Roohi-Azizi, M., S. Arabzadeh, M. Amidfar, S. Salimi, M. R. Zarindast, A. Talaei and S. Akhondzadeh (2017). "Citicoline combination therapy for major depressive disorder: A randomized, double-blind, placebo-controlled trial." Clinical Neuropharmacology **40**(1): 1-5.

Rosenbaum, J. F., M. Fava, W. E. Falk, M. H. Pollack, L. S. Cohen, B. M. Cohen and G. S. Zubenko (1988). "An open-label pilot study of oral S-adenosylmethionine in major depression. An interim report." The Alabama journal of medical sciences **25**(3): 301-306.

Ross, S. M. (2014). "Rhodiola rosea (SHR-5), Part 2: A standardized extract of Rhodiola rosea is shown to be effective in the treatment of mild to moderate depression." Holistic nursing practice **28**(3): 217-221.

Rucklidge, J. J. and A. J. Richardson (2019). "Multinutrient Supplementation for Prevention of Major Depressive Disorder in Overweight Adults." JAMA - Journal of the American Medical Association **322**(4): 366.

Rudzki, L., L. Ostrowska, D. Pawlak, A. Małus, K. Pawlak, N. Waszkiewicz and A. Szulc (2019). "Probiotic Lactobacillus Plantarum 299v decreases kynurenine concentration and improves cognitive functions in patients with major depression: A double-blind, randomized, placebo controlled study." Psychoneuroendocrinology **100**: 213-222.

Russell, S. E., D. R. Skvarc, M. Mohebbi, D. Camfield, L. K. Byrne, A. Turner, M. M. Ashton, M. Berk, S. Dodd, G. S. Malhi, S. M. Cotton, A. I. Bush and O. M. Dean (2023). "The Impact of N-acetylcysteine on Major Depression: Qualitative Observation and Mixed Methods Analysis of Participant Change during a 12-week Randomised Controlled Trial." Clinical Psychopharmacology and Neuroscience **21**(2): 320-331.

Rychlik, R., H. Siedentop, V. von den Driesch and S. Kasper (2001). "St. John's wort extract WS 5572 in minor to moderately severe depression. Effectiveness and tolerance of 600 and 1200 mg active ingredient daily." Fortschritte der Medizin. Originalien **119**(3-4): 119-128.

Ryszewska-Pokraśniewicz, B., A. Mach, M. Skalski, P. Januszko, Z. M. Wawrzyniak, E. Poleszak, G. Nowak, A. Pilc and M. Radziwoń-Zaleska (2018). "Effects of magnesium supplementation on unipolar depression: A placebo-controlled study and review of the importance of dosing and magnesium status in the therapeutic response." Nutrients **10**(8 C7 - 1014).

Saccarello, A., P. Montarsolo, I. Massardo, R. Picciotto, A. Pedemonte, R. Castagnaro, P. C. Brasesco, V. Guida, P. Picco, P. Fioravanti and et al. (2020). "Oral Administration of S-Adenosylmethionine (SAMe) and Lactobacillus Plantarum HEAL9 Improves the Mild-To-Moderate Symptoms of Depression: a Randomized, Double-Blind, Placebo-Controlled Study." **22**(4 C3 - PUBMED 32589828,EMBASE 632201612).

Sadeghi, A., F. Ghorayshi, H. Baghshahi, H. Akbari, M. R. Memarzadeh, M. Taghizadeh and A. Safaei (2023). "The antidepressant effect of combined extracts of Hypericum perforatum and Echium amoenum supplementation in patients with depression symptoms: A randomized clinical trial." Avicenna Journal of Phytomedicine **13**(4): 328-337.

Safa, M., S. Fallah Tafti, F. Ghassem Boroujerdi and F. Talischi (2013). "Clinical trial in the treatment of 80 Iranian patients with major depression disorder by the combination of omega 3 fatty acid and a selective serotonin reuptake inhibitor." Therapeutic Advances in Psychopharmacology **3**(4): 186-190.

Safari, M., A. Asadi, N. Aryaeian, H. F. Huseini, F. shidfar, S. Jazayeri, M. Malek, A. F. Hosseini and Z. hamidi (2023). "The effects of melissa officinalis on depression and anxiety in type 2 diabetes patients with depression: a randomized double-blinded placebo-controlled clinical trial." BMC Complementary Medicine and Therapies **23**(1 C7 - 140).

Sahoo, J. P., S. Jarnail, H. Khurana and P. Singh (2015). "Omega-3 Fatty Acids Versus 5-hydroxytryptophan as Add on Therapy in Patients with Depression: A Comparative Study." Indian Journal of Pharmacology **47**(7): S23-S24.

Sahraian, A., A. Ghanizadeh and F. Kazemeini (2015). "Vitamin C as an adjuvant for treating major depressive disorder and suicidal behavior, a randomized placebo-controlled clinical trial." Trials **16**(1 C7 - 94).

Sakurai, H., L. L. Carpenter, A. R. Tyrka, L. H. Price, G. I. Papakostas, C. M. Dording, A. S. Yeung, C. Cusin, E. Ludington, R. Bernard-Negron, M. Fava and D. Mischoulon (2020). "Dose increase of S-Adenosyl-Methionine and escitalopram in a randomized clinical trial for major depressive disorder." Journal of Affective Disorders **262**: 118-125.

Salehi, L., M. Farajnejad and S. Esmailzadeh Saieh (2024). "The effect of aromatherapy with Polianthes’s tuberosa on postpartum depression in high-risk pregnancies: A double blind randomized placebo-controlled clinical trial." The Iranian Journal of Obstetrics, Gynecology and Infertility **27**(4): 18-27.

Sánchez-Villegas, A., J. Álvarez-Pérez, E. Toledo, J. Salas-Salvadó, C. Ortega-Azorín, M. D. Zomeño, J. Vioque, J. A. Martínez, D. Romaguera, J. Pérez-López, J. López-Miranda, R. Estruch, A. Bueno-Cavanillas, F. Arós, J. A. Tur, F. J. Tinahones, O. Lecea, V. Martín, M. Ortega-Calvo, C. Vázquez, X. Pintó, J. Vidal, L. Daimiel, M. Delgado-Rodríguez, P. Matía, D. Corella, A. Díaz-López, N. Babio, M. Á. Muñoz, M. Fitó, M. G. de la Hera, I. Abete, A. García-Rios, E. Ros, M. Ruíz-Canela, M. Á. Martínez-González, M. Izquierdo and L. Serra-Majem (2018). "Seafood consumption, omega-3 fatty acids intake, and life-time prevalence of depression in the PREDIMED-plus trial." Nutrients **10**(12 C7 - 2000).

Sánchez-Villegas, A., M. A. Martínez-González, R. Estruch, J. Salas-Salvadó, D. Corella, M. I. Covas, F. Arós, D. Romaguera, E. Gómez-Gracia, J. Lapetra and et al. (2013). "Mediterranean dietary pattern and depression: the PREDIMED randomized trial." **11**: 208

C203 - PUBMED 24229349,EMBASE 52789647.

Sanders, A. E., E. D. Weatherspoon, B. M. Ehrmann, P. S. Soma, S. R. Shaikh, J. S. Preisser, R. Ohrbach, R. B. Fillingim and G. D. Slade (2022). "Ratio of Omega-6/Omega-3 Polyunsaturated Fatty Acids Associated With Somatic and Depressive Symptoms in People With Painful Temporomandibular Disorder and Irritable Bowel Syndrome." Journal of Pain **23**(10): 1737-1748.

Sanmukhani, J., V. Satodia, J. Trivedi, T. Patel, D. Tiwari, B. Panchal, A. Goel and C. B. Tripathi (2014). "Efficacy and safety of curcumin in major depressive disorder: a randomized controlled trial." Phytother Res **28**(4): 579-585.

Sarkawi, M., R. A. Raja Ali, N. Abdul Wahab, N. D. Abdul Rathi and N. M. Mokhtar (2024). "A randomized, double-blinded, placebo-controlled clinical trial on Lactobacillus-containing cultured milk drink as adjuvant therapy for depression in irritable bowel syndrome." Scientific reports **14**(1): 9478.

Sarris, J. (2010). "Positive effects of Piper methysticum (kava) in patients with anxiety and depression." Focus on Alternative and Complementary Therapies **15**(1): 27-28.

Sarris, J., G. J. Byrne, C. Bousman, C. Stough, J. Murphy, P. MacDonald, L. Adams, S. Nazareth, G. Oliver, L. Cribb, K. Savage, R. Menon, S. Chamoli, M. Berk, C. Ng and D. Mischoulon (2018). "Adjunctive S-adenosylmethionine (SAMe) in treating non-remittent major depressive disorder: An 8-week double-blind, randomized, controlled trial." European Neuropsychopharmacology **28**(10): 1126-1136.

Sarris, J., G. J. Byrne, C. Stough, C. Bousman, D. Mischoulon, J. Murphy, P. Macdonald, L. Adams, S. Nazareth, G. Oliver, L. Cribb, K. Savage, R. Menon, S. Chamoli, M. Berk and C. Ng (2019). "Nutraceuticals for major depressive disorder- more is not merrier: An 8-week double-blind, randomised, controlled trial." Journal of Affective Disorders **245**: 1007-1015.

Sarris, J., M. Fava, I. Schweitzer and D. Mischoulon (2012). "St Johns Wort (Hypericum perforatum) versus sertraline and placebo in major depressive disorder: Continuation data from a 26-week RCT." Pharmacopsychiatry **45**(7): 275-278.

Sarris, J., D. J. Kavanagh, J. Adams, K. Bone and G. Byrne (2009). "Kava Anxiety Depression Spectrum Study (KADSS): A mixed methods RCT using an aqueous extract of Piper methysticum." Complementary Therapies in Medicine **17**(3): 176-178.

Sarris, J., D. J. Kavanagh, G. Byrne, K. M. Bone, J. Adams and G. Deed (2009). "The Kava Anxiety Depression Spectrum Study (KADSS): A randomized, placebo-controlled crossover trial using an aqueous extract of Piper methysticum." Psychopharmacology **205**(3): 399-407.

Sarris, J., J. Murphy, C. Stough, D. Mischoulon, C. Bousman, P. MacDonald, L. Adams, S. Nazareth, G. Oliver, L. Cribb and et al. (2020). "S-Adenosylmethionine (SAMe) monotherapy for depression: an 8-week double-blind, randomised, controlled trial." **237**(1): 209‐218

C203 - PUBMED 31712971,EMBASE 2003633556.

Savović, J. (2005). "Neither Hypericum perforatum nor fluoxetine are more effective than placebo in short-term treatment of mild to moderate depression." Focus on Alternative and Complementary Therapies **10**(4): 286-287.

Schifano, F. and F. Garofoli (1993). "Dothiepin vs. S-adenosylmethionine (SAMe) for the treatment of major depression in weaned alcoholics: A pilot study." European Neuropsychopharmacology **3**(3): 330.

Schmidt, U., J. Maisenbacher, G. Harrer and U. Kuhn (1995). "Psychomotoric performance improvement: Antidepressant therapy with St John's wort." Therapiewoche **45**(2): 106+108+110+112.

Schmidt, U. and H. Sommer (1993). "St. John's wort extract in the ambulatory therapy of depression. Attention and reaction ability are preserved." Fortschritte der Medizin **111**(19): 339-342.

Schneider, E., J. P. K. Doll, N. Schweinfurth, C. Kettelhack, A. C. Schaub, G. Yamanbaeva, N. Varghese, L. Mählmann, S. Brand, A. Eckert and et al. (2023). "Effect of short-term, high-dose probiotic supplementation on cognition, related brain functions and BDNF in patients with depression: a secondary analysis of a randomized controlled trial." **48**(1): E23‐E33

C23 - PUBMED 36653035.

Schrader, E. (2000). "Equivalence of St John's wort extract (Ze 117) and fluoxetine: A randomized, controlled study in mild-moderate depression." International Clinical Psychopharmacology **15**(2): 61-68.

Schrader, E., B. Meier and A. Brattström (1998). "Hypericum treatment of mild-moderate depression in a placebo-controlled study. A prospective, double-blind, randomized, placebo-controlled, multicentre study." Human Psychopharmacology **13**(3): 163-169.

Schulz, V. (2002). "Clinical study with hypericum extracts in depressive patients. Results, comparison, conclusions, for anti-depressive pharmacotherapy." Zeitschrift fur Phytotherapie **23**(1): 11-15.

Schulz, V. (2005). "St John's wort in the treatment of moderate to severe depression." Zeitschrift fur Phytotherapie **26**(3): 127-128.

Schulz, V. (2008). "Single dose of St John's wort versus citalopram in patients with moderate depression." Zeitschrift fur Phytotherapie **29**(3): 131-132.

Schulzz, V. (2009). "Rhodiola rosea radix extract for the treatment of mild to moderate depression? Results of a placebo-controlled clinical study." KIM - Komplementare und Integrative Medizin, Artztezeitschrift fur Naturheilverfahren **50**(3): 53-54.

Sempach, L., A.-C. Schaub, A. B. Brühl, P. Sterzer, U. E. Lang and A. Schmidt (2025). "Adjunctive d-serine treatment for major depressive disorder: A randomized clinical trial." Journal of Affective Disorders: 119504.

Sempels, C. and P. Sienaert (2007). "The role of omega-3 fatty acids in the treatment of bipolar disorders: The current situation." Tijdschrift voor Psychiatrie **49**(9): 639-647.

Sepehrmanesh, Z., F. Kolahdooz, F. Abedi, N. Mazroii, A. Assarian, Z. Asemi and A. Esmaillzadeh (2016). "Vitamin D supplementation affects the beck depression inventory, insulin resistance, and biomarkers of oxidative stress in patients with major depressive disorder: A randomized, controlled clinical trial." Journal of Nutrition **146**(2): 243-248.

Sepehrmanesh, Z., F. Kolahdooz, F. Abedi, N. Mazroii, A. Assarian, Z. Asemi and A. Esmaillzadeh (2016). "Vitamin D Supplementation Affects the Beck Depression Inventory, Insulin Resistance, and Biomarkers of Oxidative Stress in Patients with Major Depressive Disorder: A Randomized, Controlled Clinical Trial [Retraction notice in CN-02298463]." **146**(2): 243‐248

C243 - PUBMED 26609167,EMBASE 608794678.

Severus, W. E. (2006). "Effects of omega-3 polyunsaturated fatty acids on depression." Herz **31 Suppl 3**: 69-74.

Shafiee-Kandjani, A., M. Khalili, A. Malek, S. Farhang, Y. Ranjbari and Y. Khalili (2023). "The therapeutic effect of the extracts of Lavandula angustifolia and Dracocephalum ruyschiana besides sertraline on patients with mild to moderate depression: A double-blind controlled trial." Phytomedicine Plus **3**(2).

Shahmansouri, N., M. Farokhnia, S. H. Abbasi, S. E. Kassaian, A. A. Noorbala Tafti, A. Gougol, H. Yekehtaz, S. Forghani, M. Mahmoodian, S. Saroukhani and et al. (2014). "A randomized, double-blind, clinical trial comparing the efficacy and safety of Crocus sativus L. with fluoxetine for improving mild to moderate depression in post percutaneous coronary intervention patients." **155**: 216‐222

C213 - PUBMED 24289892,EMBASE 52891114.

Shahmoradi, N., O. Davarinejad, A. B. Brühl and S. Brand (2023). "Effects of Aphrodite (an Herbal Compound) on SSRI-Induced Sexual Dysfunctions and Depression in Females with Major Depressive Disorder: Findings from a Randomized Clinical Trial." Medicina (Kaunas, Lithuania) **59**(9).

Shamabadi, A., S. Fattollahzadeh-Noor, B. Fallahpour, F. A. Basti, M. R. Khodaei Ardakani and S. Akhondzadeh (2023). "L-Theanine adjunct to risperidone in the treatment of chronic schizophrenia inpatients: a randomized, double-blind, placebo-controlled clinical trial." Psychopharmacology **240**(12): 2631-2640.

Sharifan, P., M. S. Hosseini and A. Sharifan (2017). "The interventional relationship between frequent fish consumption and depression symptoms in aging adults: a randomized controlled trial." International Journal of Geriatric Psychiatry **32**(12): e116-e122.

Sharifi, A., H. Vahedi, S. Nedjat, A. Mohamadkhani and M. J. Hosseinzadeh Attar (2019). "Vitamin D Decreases Beck Depression Inventory Score in Patients with Mild to Moderate Ulcerative Colitis: A Double-Blind Randomized Placebo-Controlled Trial." Journal of Dietary Supplements **16**(5): 541-549.

Sheikh, M., S. Hantoushzadeh, M. Shariat, Z. Farahani and O. Ebrahiminasab (2017). "The efficacy of early iron supplementation on postpartum depression, a randomized double-blind placebo-controlled trial." European Journal of Nutrition **56**(2): 901-908.

Shelton, R. C., M. B. Keller, A. Gelenberg, D. L. Dunner, R. Hirschfeld, M. E. Thase, J. Russell, R. B. Lydiard, P. Crits-Christoph, R. Gallop, L. Todd, D. Hellerstein, P. Goodnick, G. Keitner, S. M. Stahl and U. Halbreich (2001). "Effectiveness of St John's wort in major depression: A randomized controlled trial." JAMA **285**(15): 1978-1986.

Shemshian, M., A. Sadeghi, T. Kermani, H. M. Seyed, S. Tavalaie and A. Norouzi (2011). "Saffron (crocus sativus) in the treatment of anxiety and depression: a double-blind, randomized and placebo-controlled trial."

Sherpa, N. N., R. De Giorgi, E. G. Ostinelli, A. Choudhury, T. Dolma and S. Dorjee (2025). "Efficacy and safety profile of oral creatine monohydrate in add-on to cognitive-behavioural therapy in depression: An 8-week pilot, double-blind, randomised, placebo-controlled feasibility and exploratory trial in an under-resourced area." European Neuropsychopharmacology **90**: 28-35.

Sherwood Brown, E. and B. Gabrielson (2012). "A randomized, double-blind, placebo-controlled trial of citicoline for bipolar and unipolar depression and methamphetamine dependence." Journal of Affective Disorders **143**(1-3): 257-260.

Shiloh, R., A. Weizman, N. Weizer, P. Dorfman-Etrog and H. Munitz (2001). "Antidepressive effect of pyridoxine (vitamin B6) in neuroleptic-treated schizophrenic patients with co-morbid minor depression - Preliminary open-label trial." Harefuah **140**(5): 369-373+456.

Shinto, L., G. Marracci, D. C. Mohr, L. Bumgarner, C. Murchison, A. Senders and D. Bourdette (2016). "Omega-3 fatty acids for depression in multiple sclerosis: A randomized pilot study." PLoS ONE **11**(1 C7 - e0147195).

Shipowick, C. D., C. B. Moore, C. Corbett and R. Bindler (2009). "Vitamin D and depressive symptoms in women during the winter: A pilot study." Applied Nursing Research **22**(3): 221-225.

Shippy, R. A., D. Mendez, K. Jones, I. Cergnul and S. E. Karpiak (2004). "S-adenosylmethionine (SAM-e) for the treatment of depression in people living with HIV/AIDS." BMC psychiatry **4**.

Silvers, K. M., C. C. Woolley, F. C. Hamilton, P. M. Watts and R. A. Watson (2005). "Randomised double-blind placebo-controlled trial of fish oil in the treatment of depression." Prostaglandins Leukotrienes and Essential Fatty Acids **72**(3): 211-218.

Singer, A., M. Schmidt, W. Hauke and K. Stade (2011). "Duration of response after treatment of mild to moderate depression with Hypericum extract STW 3-VI, citalopram and placebo: A reanalysis of data from a controlled clinical trial." Phytomedicine.

Sinn, N., C. M. Milte, S. J. Street, J. D. Buckley, A. M. Coates, J. Petkov and P. R. Howe (2012). "Effects of n-3 fatty acids, EPA v. DHA, on depressive symptoms, quality of life, memory and executive function in older adults with mild cognitive impairment: a 6-month randomised controlled trial." **107**(11): 1682‐1693

C1683 - PUBMED 21929835.

Siwek, M., D. Dudek, M. Schlegel-Zawadzka, A. Morawska, W. Piekoszewski, W. Opoka, A. Ziȩba, A. Pilc, P. Popik and G. Nowak (2010). "Serum zinc level in depressed patients during zinc supplementation of imipramine treatment." Journal of Affective Disorders **126**(3): 447-452.

Slykerman, R. F., F. Hood, K. Wickens, J. M. D. Thompson, C. Barthow, R. Murphy, J. Kang, J. Rowden, P. Stone, J. Crane, T. Stanley, P. Abels, G. Purdie, R. Maude and E. A. Mitchell (2017). "Effect of Lactobacillus rhamnosus HN001 in Pregnancy on Postpartum Symptoms of Depression and Anxiety: A Randomised Double-blind Placebo-controlled Trial." EBioMedicine **24**: 159-165.

Smith, D. J., J. Sarris, N. Dowling, M. O'Connor and C. H. Ng (2018). "Adjunctive low-dose docosahexaenoic acid (DHA) for major depression: An open-label pilot trial." Nutritional Neuroscience **21**(3): 224-228.

Solati, Z., S. Jazayeri, M. Tehrani-Doost, S. Mahmoodianfard and M. R. Gohari (2015). "Zinc monotherapy increases serum brain-derived neurotrophic factor (BDNF) levels and decreases depressive symptoms in overweight or obese subjects: a double-blind, randomized, placebo-controlled trial." **18**(4): 162‐168

C163 - PUBMED 24621065,EMBASE 603838110.

Soltani, M., M. J. Hosseinzadeh-Attar, M. Rezaei, E. Alipoor, A. Vasheghani-Farahani, M. Yaseri and S. M. Rezayat (2024). "Effect of nano-curcumin supplementation on cardiometabolic risk factors, physical and psychological quality of life, and depression in patients with coronary slow flow phenomenon: a randomized double-blind clinical trial." Trials **25**(1 C7 - 515).

Song, Y. K., J. Y. Lee and W. Park (2016). "Modulatory Effect of Eui-E-In-Tang on Serum Leptin Concentration in Obese Korean Female Adults: A Randomized Controlled Trial." Evid Based Complement Alternat Med **2016**: 1894837.

Sousa, T. M. D. and L. C. D. Santos (2020). "Dietary fatty acids, omega-6/omega-3 ratio and cholesterol intake associated with depressive symptoms in low-risk pregnancy." Nutritional Neuroscience: 1-6.

Stevinson, C. and E. Ernst (1999). "Hypericum for depression: An update of the clinical evidence." European Neuropsychopharmacology **9**(6): 501-505.

Stewart, R. C., P. Ashorn, E. Umar, K. G. Dewey, U. Ashorn, F. Creed, A. Rahman, B. Tomenson, E. L. Prado and K. Maleta (2017). "The impact of maternal diet fortification with lipid-based nutrient supplements on postpartum depression in rural Malawi: a randomised-controlled trial." Maternal and Child Nutrition **13**(2 C7 - e12299).

Strodl, E., M. Bambling, S. Parnam, G. Ritchie, S. Cramb and L. Vitetta (2024). "Probiotics and magnesium orotate for the treatment of major depressive disorder: a randomised double blind controlled trial." Scientific reports **14**(1): 20841.

Su, K. P., S. Y. Huang, C. C. Chiu and W. W. Shen (2003). "Omega-3 fatty acids in major depressive disorder: A preliminary double-blind, placebo-controlled trial." European Neuropsychopharmacology **13**(4): 267-271.

Su, K. P., S. Y. Huang, T. H. Chiu, K. C. Huang, C. L. Huang, H. C. Chang and C. M. Pariante (2008). "Omega-3 fatty acids for major depressive disorder during pregnancy: Results from a randomized, double-blind, placebo-controlled trial." Journal of Clinical Psychiatry **69**(4): 644-651.

Su, K. P., H. C. Lai, H. T. Yang, W. P. Su, C. Y. Peng, J. P. Chang, H. C. Chang and C. M. Pariante (2014). "Omega-3 fatty acids in the prevention of interferon-alpha-induced depression: results from a randomized, controlled trial." Biol Psychiatry **76**(7): 559-566.

Sultana, S. and M. S. Rahman (2023). "Effect of magnesium supplementation in selective serotonin reuptake inhibitors treated major depressive disorder patients: A randomized, double-blind, placebo-controlled trial." EC Nutr **18**: 25-34.

Suzuki, H., S. Masuki, A. Morikawa, Y. Ogawa, Y. I. Kamijo, K. Takahashi, M. Nakajima and H. Nose (2018). "Effects of 5-aminolevulinic acid supplementation on home-based walking training achievement in middle-aged depressive women: Randomized, double-blind, crossover pilot study." Scientific Reports **8**(1 C7 - 7151).

Syed, E. U., M. Wasay and S. Awan (2013). "Vitamin B12 supplementation in treating major depressive disorder: A randomized controlled trial." Open Neurology Journal **7**(1): 44-48.

Szegedi, A., R. Kohnen, A. Dienel and M. Kieser (2005). "Acute treatment of moderate to severe depression with hypericum extract WS 5570 (St John's wort): Randomised controlled double blind non-inferiority trial versus paroxetine." British Medical Journal **330**(7490): 503-506.

Tabasi, M., S. A. Mostafavi, H. Oreyzi, M. R. Mohammadi and A. Khaleghi (2024). "The Effectiveness of Transcranial Direct Current Stimulation (tDCS) and Omega-3 on Food Craving, Executive Functions, Weight, and Depressive Symptoms in Patients with Depression and Overweight: A Randomized Controlled Trial." Iranian Journal of Psychiatry **19**(2): 158-170.

Tabeshpour, J., F. Sobhani, S. A. Sadjadi, H. Hosseinzadeh, S. A. Mohajeri, O. Rajabi, Z. Taherzadeh and S. Eslami (2017). "A double-blind, randomized, placebo-controlled trial of saffron stigma (Crocus sativus L.) in mothers suffering from mild-to-moderate postpartum depression." Phytomedicine **36**: 145-152.

Taheri, M., K. Irandoust, A. Sadeghi and S. Yari (2018). "The effect of omega-3 fatty acid supplement and aerobic exercise on lipid profile and depression in obese women." Acta Medica Mediterranea **34**(3): 865-870.

Tajalizadekhoob, Y., F. Sharifi, H. Fakhrzadeh, M. Mirarefin, M. Ghaderpanahi, Z. Badamchizade and S. Azimipour (2011). "The effect of low-dose omega 3 fatty acids on the treatment of mild to moderate depression in the elderly: A double-blind, randomized, placebo-controlled study." European Archives of Psychiatry and Clinical Neuroscience **261**(8): 539-549.

Talaei, A., M. Hassanpour Moghadam, S. A. Sajadi Tabassi and S. A. Mohajeri (2015). "Crocin, the main active saffron constituent, as an adjunctive treatment in major depressive disorder: A randomized, double-blind, placebo-controlled, pilot clinical trial." Journal of Affective Disorders **174**: 51-56.

Talaei, A., R. Noori, A. Rezaei Ardani, A. H. Mohammadpour, Z. Azimipoor and F. Afzaljavan (2023). "The Effect of Adding Curcumin to Sertraline in the Treatment of Severe Major Depressive Disorder: A Randomized, Double-Blind Clinical Trial." Clinical Neuropharmacology **46**(4): 135-139.

Targum, S. D., B. R. Cameron, L. Ferreira and I. D. MacDonald (2018). "An augmentation study of MSI-195 (S-adenosylmethionine) in Major Depressive Disorder." Journal of Psychiatric Research **107**: 86-96.

Tarkesh, F., B. Namavar Jahromi, N. Hejazi and G. Hoseini (2022). "Effect of vitamin K2 administration on depression status in patients with polycystic ovary syndrome: a randomized clinical trial." BMC Women's Health **22**(1).

Tarleton, E. K., B. Littenberg, C. D. MacLean, A. G. Kennedy and C. Daley (2017). "Role of magnesium supplementation in the treatment of depression: A randomized clinical trial." PLoS ONE **12**(6 C7 - e0180067).

Tarutani, S., M. Omori, Y. Ido, M. Yano, T. Komatsu and T. Okamura (2022). "Effects of 4G-beta-D-Galactosylsucrose in patients with depression: A randomized, double-blinded, placebo-controlled, parallel-group comparative study." J Psychiatr Res **148**: 110-120.

Tatum, P. and E. J. Lindbloom (2001). "Is St. John's wort an effective treatment for major depression?" Journal of Family Practice **50**(7): 624.

Tayama, J., S. Ogawa, N. Nakaya, T. Sone, T. Hamaguchi, A. Takeoka, K. Hamazaki, H. Okamura, J. Yajima, M. Kobayashi and et al. (2019). "Omega-3 polyunsaturated fatty acids and psychological intervention for workers with mild to moderate depression: a double-blind randomized controlled trial." **245**: 364‐370

C363 - PUBMED 30423463,EMBASE 2001261185.

Tayebi, N., M. Emamghoreishi and M. Akbarzadeh (2021). "Effect of vitex agnus-castus on depression of postmenopausal women: A randomized clinical trial." Shiraz E Medical Journal **22**(6).

Tayebi, N., M. Emamghoreishi and M. Akbarzadeh (2024). "The Impact of Salvia officinalis Extract on Depression in Postmenopausal Women: A Randomized Clinical Trial." International Journal of Women's Health and Reproduction Sciences **12**(2).

Taylor, C. L., P. R. Thomas, J. F. Aloia, P. S. Millard and C. J. Rosen (2015). "Questions about Vitamin D for Primary Care Practice: Input from an NIH Conference." American Journal of Medicine **128**(11): 1167-1170.

Tessou, K. D., H. Lemus, F. C. Hsu, J. Pierce, S. Hong, L. Brown and T. Wu (2021). "Independent and joint impacts of acid-producing diets and depression on physical health among breast cancer survivors." Nutrients **13**(7 C7 - 2422).

Thesing, C. S., Y. Milaneschi, M. Bot, I. A. Brouwer, M. Owens, U. Hegerl, M. Gili, M. Roca, E. Kohls, E. Watkins and et al. (2020). "Supplementation-induced increase in circulating omega-3 serum levels is not associated with a reduction in depressive symptoms: results from the MooDFOOD depression prevention trial." **37**(11): 1079‐1088

C1073 - PUBMED 32845021,EMBASE 2005970673.

Thesing, C. S., Y. Milaneschi, M. Bot, I. A. Brouwer, M. Owens, U. Hegerl, M. Gili, M. Roca, E. Kohls, E. Watkins, M. Visser and B. Penninx (2020). "Supplementation-induced increase in circulating omega-3 serum levels is not associated with a reduction in depressive symptoms: Results from the MooDFOOD depression prevention trial." Depress Anxiety **37**(11): 1079-1088.

Thomson, M. and V. Sharma (2023). "Alternative Treatments for Postpartum Depression." Current Treatment Options in Psychiatry **10**(3): 288-312.

Tian, P., Y. Chen, H. Zhu, L. Wang, X. Qian, R. Zou, J. Zhao, H. Zhang, L. Qian, Q. Wang and et al. (2022). "Bifidobacterium breve CCFM1025 attenuates major depression disorder via regulating gut microbiome and tryptophan metabolism: a randomized clinical trial." **100**: 233‐241

C233 - PUBMED 34875345.

Tian, P., H. Yang, F. Hang, G. Wang, X. Mao, X. Jin and J. Zhao (2024). "Evaluation of the clinical efficacy of Pediococcus acidilactici CCFM6432 in alleviating depression." Microbiome Research Reports **3**(4 C7 - 49).

Tiemeier, H., H. Ruud van Tuijl, A. Hofman, J. Meijer, A. J. Kiliaan and M. M. B. Breteler (2002). "Vitamin B12, folate, and homocysteine in depression: The Rotterdam study." American Journal of Psychiatry **159**(12): 2099-2101.

Tomko, R. L., N. L. Baker, C. O. Hood, A. K. Gilmore, E. A. McClure, L. M. Squeglia, A. L. McRae-Clark, S. C. Sonne and K. M. Gray (2020). "Depressive symptoms and cannabis use in a placebo-controlled trial of N-Acetylcysteine for adult cannabis use disorder." Psychopharmacology **237**(2): 479-490.

Tomko, R. L., A. K. Gilmore and K. M. Gray (2018). "The role of depressive symptoms in treatment of adolescent cannabis use disorder with N-Acetylcysteine." Addictive Behaviors **85**: 26-30.

Tzikos, G., E. Chamalidou, D. Christopoulou, A. Apostolopoulou, S. Gkarmiri, M. Pertsikapa, A. E. Menni, I. M. Theodorou, G. Stavrou, N. D. Doutsini, A. D. Shrewsbury, T. Papavramidis, J. K. Tsetis, H. Theodorou, A. Konsta and K. Kotzampassi (2025). "Psychobiotics Ameliorate Depression and Anxiety Status in Surgical Oncology Patients: Results from the ProDeCa Study." Nutrients **17**(5).

Ullah, H., A. Di Minno, C. Esposito, H. R. El-Seedi, S. A. M. Khalifa, A. Baldi, A. Greco, S. Santonastaso, V. Cioffi, R. Sperandeo and et al. (2022). "Efficacy of a food supplement based on S-adenosyl methionine and probiotic strains in subjects with subthreshold depression and mild-to-moderate depression: a monocentric, randomized, cross-over, double-blind, placebo-controlled clinical trial." **156**: 113930

C113933 - PUBMED 36411659.

Vaghef-Mehrabany, E., F. Ranjbar, M. Asghari-Jafarabadi, S. Hosseinpour-Arjmand and M. Ebrahimi-Mameghani (2021). "Calorie restriction in combination with prebiotic supplementation in obese women with depression: effects on metabolic and clinical response." Nutritional Neuroscience **24**(5): 339-353.

Van De Rest, O., J. De Goede, F. Sytsma, L. M. Oude Griep, J. M. Geleijnse, D. Kromhout and E. J. Giltay (2010). "Association of n-3 long-chain PUFA and fish intake with depressive symptoms and low dispositional optimism in older subjects with a history of myocardial infarction." British Journal of Nutrition **103**(9): 1381-1387.

van Gurp, G., G. B. Meterissian, L. N. Haiek, J. McCusker and F. Bellavance (2002). "St John's wort or sertraline? Randomized controlled trial in primary care." Can Fam Physician **48**: 905-912.

Varzaghani, V., M. Sharifi, R. Hajiaghaee, S. Bagheri, S. Momtaz, Z. Tarassoli and A. Razmi (2022). "Propolis add-on therapy alleviates depressive symptoms; A randomized placebo-controlled clinical trial." Phytotherapy Research **36**(3): 1258-1267.

Vaz, J., D. Farias, A. Adegboye, A. Nardi and G. Kac (2018). "N-3 polyunsaturated fatty acids (PUFAs) supplementation from pregnancy to postpartum to prevent depressive symptoms: A randomized placebo-controlled trial." Nutritional Neuroscience **21**: S1.

Vaz, J. D. S., D. R. Farias, A. R. A. Adegboye, A. E. Nardi and G. Kac (2017). "Omega-3 supplementation from pregnancy to postpartum to prevent depressive symptoms: a randomized placebo-controlled trial." BMC Pregnancy Childbirth **17**(1): 180.

Vaziri, F., S. Nasiri, Z. Tavana, M. H. Dabbaghmanesh, F. Sharif and P. Jafari (2016). "A randomized controlled trial of vitamin D supplementation on perinatal depression: In Iranian pregnant mothers." BMC Pregnancy and Childbirth **16**(1 C7 - 239).

Velichkov, M., Z. Bezur, C. M. van Reekum and C. M. Williams (2024). "A biphasic response to blueberry supplementation on depressive symptoms in emerging adults: a double-blind randomized controlled trial." European Journal of Nutrition **63**(4): 1071-1088.

Vellekkatt, F., V. Menon, M. Rajappa and J. Sahoo (2020). "Effect of adjunctive single dose parenteral Vitamin D supplementation in major depressive disorder with concurrent vitamin D deficiency: A double-blind randomized placebo-controlled trial." J Psychiatr Res **129**: 250-256.

Venkatasubramanian, R., C. N. Kumar and R. S. Pandey (2013). "A randomized double-blind comparison of fluoxetine augmentation by high and low dosage folic acid in patients with depressive episodes." Journal of Affective Disorders **150**(2): 644-648.

Verma, D. K. and B. Das (2016). "Effect of zinc supplementation on antidepressant therapy and serum zinc level in unipolar depression: A randomized double blind controlled study." Indian Journal of Psychiatry **58**(5): S47.

Vorbach, E. U., K. H. Arnoldt and W. D. Hubner (1997). "Efficacy and tolerability of St. John's wort extract LI 160 versus imipramine in patients with severe depressive episodes according to ICD-10." Pharmacopsychiatry **30**(SUPPL.): 81-85.

Vreijling, S. R., B. Penninx, M. Bot, E. Watkins, M. Owens, E. Kohls, U. Hegerl, M. Roca, M. Gili, I. A. Brouwer and et al. (2021). "Effects of dietary interventions on depressive symptom profiles: results from the MooDFOOD depression prevention study." **52**(15): 1‐10

C13 - PUBMED 33823960,EMBASE 634757999.

Vyas, C. M., D. Mischoulon, G. Chang, N. R. Cook, A. Weinberg, T. Copeland, J. H. Kang, V. Bubes, G. Friedenberg, M. S. LeBoff and et al. (2023). "Effects of Vitamin D3 and Marine Omega-3 Fatty Acids Supplementation on Indicated and Selective Prevention of Depression in Older Adults: results From the Clinical Center Sub-Cohort of the VITamin D and OmegA-3 TriaL (VITAL)." **84**(4).

Wadud, S., S. Fatima and M. Irfan (2024). "ROLE OF ASCORBIC ACID IN IMPROVING MOOD IN PATIENTS WITH DEPRESSION: A RANDOMIZED CONTROLLED TRIAL." Journal of Postgraduate Medical Institute **38**(2): 97-101.

Walker, J. G., P. J. Batterham, A. J. Mackinnon, A. F. Jorm, I. Hickie, M. Fenech, M. Kljakovic, D. Crisp and H. Christensen (2012). "Oral folic acid and vitamin B-12 supplementation to prevent cognitive decline in community-dwelling older adults with depressive symptoms - The Beyond Ageing Project: A randomized controlled trial (American Journal of Clinical Nutrition (2012) 95, (194-203))." American Journal of Clinical Nutrition **96**(2): 448.

Walker, J. G., A. J. Mackinnon, P. Batterham, A. F. Jorm, I. Hickie, A. McCarthy, M. Fenech and H. Christensen (2010). "Mental health literacy, folic acid and vitamin B12, and physical activity for the prevention of depression in older adults: Randomised controlled trial." British Journal of Psychiatry **197**(1): 45-54.

Walker, S. P., S. M. Chang, C. A. Powell, E. Simonoff and S. M. Grantham-McGregor (2006). "Effects of psychosocial stimulation and dietary supplementation in early childhood on psychosocial functioning in late adolescence: follow-up of randomised controlled trial." **333**(7566): 472

C473 - PUBMED 16877454,EMBASE 44343443.

Wallace, C. J. K. and R. V. Milev (2021). "The Efficacy, Safety, and Tolerability of Probiotics on Depression: Clinical Results From an Open-Label Pilot Study." Frontiers in Psychiatry **12 C7 - 618279**.

Wang, L., P. L. Bordi, J. A. Fleming, A. M. Hill and P. M. Kris-Etherton (2015). "Effect of a moderate fat diet with and without avocados on lipoprotein particle number, size and subclasses in overweight and obese adults: a randomized, controlled trial." J Am Heart Assoc **4**(1): e001355.

Wang, L., Y. Zhang, L. Chen, D. Yuan, X. Feng, J. Zheng, X. Li and C. Yuan (2024). "A two-center randomized controlled trial of a repairing mask as an adjunctive treatment for mild to moderate rosacea." J Cosmet Dermatol **23**(10): 3281-3286.

Wang, X. J., J. Li, Q. D. Zou and L. Jin (2009). "Wuling Capsule for climacteric patients with depression and anxiety state: A randomized, positive parallel controlled trial." Journal of Chinese Integrative Medicine **7**(11): 1042-1046.

Wasielewski, S. (2003). "Herbal psychotropic drugs for the treatment of mild depression?" Deutsche Apotheker Zeitung **143**(39): 38-39.

Wheatley, D. (1997). "LI 160, an extract of St. John's wort, versus amitriptyline in mildly to moderately depressed outpatients--a controlled 6-week clinical trial." **30 Suppl 2**: 77‐80

C73 - PUBMED 9342764.

Woelk, H. (2000). "Comparison of St John's wort and imipramine for treating depression: Randomised controlled trial." British Medical Journal **321**(7260): 536-539.

Wu, R., D. Zhu, Y. Xia, H. Wang, W. Tao, W. Xue, B. Xia, L. Ren, X. Zhou, G. Li and G. Chen (2015). "A role of Yueju in fast-onset antidepressant action on major depressive disorder and serum BDNF expression: A randomly double-blind, fluoxetine-adjunct, placebo-controlled, pilot clinical study." Neuropsychiatric Disease and Treatment **11**: 2013-2021.

Wu, S. K., K. J. Yang, W. C. Liu, I. A. Malau, H. Zailani, C. H. Chang, S. Y. Huang, J. P. Chang, W. C. Chiu and K. P. Su (2024). "The Efficacy of Omega-3 Fatty Acids as the Monotherapy for Depression: A Randomized, Double-Blind, Placebo-Controlled Pilot Study." Nutrients **16**(21).

Wu, S. K., K. J. Yang, W. C. Liu, I. A. Malau, H. Zailani, C. H. Chang, S. Y. Huang, J. P. C. Chang, W. C. Chiu and K. P. Su (2024). "The Efficacy of Omega-3 Fatty Acids as the Monotherapy for Depression: A Randomized, Double-Blind, Placebo-Controlled Pilot Study." Nutrients **16**(21 C7 - 3688).

Xiao, F., L. Liu, W. P. Zhang, X. Y. Chang and Y. F. Zhang (2016). "Effect of adding magnesium sulfate to intrathecal low-dose of bupivacaine for patients with severe pre-eclampsia undergoing cesarean delivery." **9**(10): 19749‐19756

C19743 - EMBASE 612964624.

Xue, Y., L. Wang, T. Liu, T. Zhao, K. Xie, J. Guo, J. Chen, H. Tang and M. Tang (2024). "Omega-3 polyunsaturated fatty acids supplementation improves memory in first-diagnosed, drug-naïve patients with depression: Secondary analysis of data from a randomized controlled trial." Journal of Affective Disorders **350**: 403-410.

Yalamanchili, V. and J. C. Gallagher (2016). "Comparison of the effect of vitamin D and 1,25 dihydroxyvitamin D treatment on depression scores in older women." Endocrine Reviews **37**(2).

Yalamanchili, V. and J. C. Gallagher (2017). "Effect of vitamin D supplementation on depression scores in younger caucasian and Africa-American women." Endocrine Reviews **38**(3).

Yalamanchili, V. and J. C. Gallagher (2018). "Dose ranging effects of vitamin D3 on the geriatric depression score: A clinical trial." Journal of Steroid Biochemistry and Molecular Biology **178**: 60-64.

Yamada, K., G. Yagi and S. Kanba (2005). "Effectiveness of herbal medicine (Rokumigan and Hachimijiogan) for fatigue or loss of energy in patients with partial remitted major depressive disorder." Psychiatry and Clinical Neurosciences **59**(5): 610-612.

Yang, B., L. Lin, R. P. Bazinet, Y. C. Chien, J. P. C. Chang, S. K. Satyanarayanan, H. Su and K. P. Su (2019). "Clinical Efficacy and Biological Regulations of ω-3 PUFA-Derived Endocannabinoids in Major Depressive Disorder." Psychotherapy and Psychosomatics **88**(4): 215-224.

Yang, C., F. J. Bosker, J. Li and R. A. Schoevers (2018). "N-acetylcysteine as add-on to antidepressant medication in therapy refractory major depressive disorder patients with increased inflammatory activity: Study protocol of a double-blind randomized placebo-controlled trial." BMC Psychiatry **18**(1).

Yang, R., L. Wang, K. Jin, S. Cao, C. Wu, J. Guo, J. Chen, H. Tang and M. Tang (2022). "Omega-3 Polyunsaturated Fatty Acids Supplementation Alleviate Anxiety Rather Than Depressive Symptoms Among First-Diagnosed, Drug-Naïve Major Depressive Disorder Patients: A Randomized Clinical Trial." Frontiers in Nutrition **9 C7 - 876152**.

Yang, T. J., C. H. Chiu, C. L. Wu, Y. S. Liao and C. K. Chang (2021). "Thirty minutes of moderate-intensity downhill or level running has no effect on postprandial lipemia: A randomized controlled trial." Chin J Physiol **64**(5): 244-250.

Yoon, S., J. E. Kim, J. Hwang, T. S. Kim, H. J. Kang, E. Namgung, S. Ban, S. Oh, J. Yang, P. F. Renshaw and I. K. Lyoo (2016). "Effects of Creatine Monohydrate Augmentation on Brain Metabolic and Network Outcome Measures in Women With Major Depressive Disorder." Biological Psychiatry **80**(6): 439-447.

Yosaee, S., S. Soltani, A. Esteghamati, S. A. Motevalian, M. Tehrani-Doost, C. C. T. Clark and S. Jazayeri (2020). "Effects of zinc, vitamin D, and their co-supplementation on mood, serum cortisol, and brain-derived neurotrophic factor in patients with obesity and mild to moderate depressive symptoms: A phase II, 12-wk, 2 × 2 factorial design, double-blind, randomized, placebo-controlled trial." Nutrition **71 C7 - 110601**.

Yu, J., P. Chukaew and M. Rasenick (2020). "A Molecular Rationale for n-3 PUFA Augmentation of Antidepressant Action." Biological Psychiatry **87**(9): S40.

Yu, J. J., L. B. Pei, Y. Zhang, Z. Y. Wen and J. L. Yang (2015). "Chronic Supplementation of Curcumin Enhances the Efficacy of Antidepressants in Major Depressive Disorder: A Randomized, Double-Blind, Placebo-Controlled Pilot Study." Journal of Clinical Psychopharmacology **35**(4): 406-410.

Zailani, H., S. K. Wu, K. J. Yang, I. A. Malau, H. F. Liao, Y. L. Chung, J. P. Chang, W. C. Chiu and K. P. Su (2024). "Omega-3 polyunsaturated fatty acids in the prevention of relapse in patients with stable bipolar disorder: A 6-month pilot randomized controlled trial." Psychiatry Res **331**: 115633.

Zajecka, J. M., M. Fava, R. C. Shelton, L. W. Barrentine, P. Young and G. I. Papakostas (2016). "Long-Term Efficacy, Safety, and Tolerability of l-Methylfolate Calcium 15 mg as Adjunctive Therapy With Selective Serotonin Reuptake Inhibitors: A 12-Month, Open-Label Study Following a Placebo-Controlled Acute Study." Journal of Clinical Psychiatry **77**(5): 654-660.

Zaromytidou, E., T. Koufakis, G. Dimakopoulos, D. Drivakou, S. Konstantinidou, P. Rakitzi, M. Grammatiki, E. Manthou, A. Notopoulos, I. Iakovou and et al. (2022). "Vitamin D Alleviates Anxiety and Depression in Elderly People with Prediabetes: a Randomized Controlled Study." **12**(10 C3 - PUBMED 36295786,EMBASE 2019781038).

Zaromytidou, E., T. Koufakis, G. Dimakopoulos, D. Drivakou, G. Meristoudis, S. Konstantinidou, G. Karaliolios, E. Melidou, P. Rakitzi, M. Grammatiki and et al. (2022). "Vitamin D Supplementation Improves Anxiety and Depression Status in Elderly People with Prediabetes: an Open-Label 12-Month Randomized Controlled Study." **71 C3 - EMBASE 641933907**.

Zhang, L., S. Wang, Y. Zhu and T. Yang (2018). "Vitamin D3 as adjunctive therapy in the treatment of depression in tuberculosis patients: A short-term pilot randomized double-blind controlled study." Neuropsychiatric Disease and Treatment **14**: 3103-3109.

Zhang, Y., Y. Cao and L. Wang (2020). "The effects of a new, improved Chinese medicine, Gengnianchun formula granules, on hot flushes, depression, anxiety, and sleep in Chinese peri-and postmenopausal women: A randomized placebo-controlled trial." Menopause **27**(8): 899-905.

Zhang, Z. J. (2012). "Herbal medicine and acupuncture for unipolar and bipolar depression: Randomized controlled trials." Bipolar Disorders **14**: 137.

Zhao, A. M., W. R. Qiu, L. J. Mao, J. G. Ren, L. Xu, M. J. Yao, K. Bilinksi, D. Chang and J. X. Liu (2018). "The efficacy and safety of Jiedu Tongluo granules for treating post-stroke depression with qi deficiency and blood stasis syndrome: study protocol for a randomized controlled trial." Trials **19**(1): 275.

Zheng, S., L. Tu, F. Cicuttini, W. Han, Z. Zhu, B. Antony, A. Wluka, T. Winzenberg, T. Meng, D. Aitken, L. Blizzard, G. Jones and C. Ding (2019). "Effect of Vitamin D Supplementation on Depressive Symptoms in Patients With Knee Osteoarthritis." Journal of the American Medical Directors Association **20**(12): 1634-1640.e1631.

Zheng, S., L. Tu, F. Cicuttini, W. Han, Z. Zhu, B. Antony, A. Wluka, T. Winzenberg, T. Meng, L. Blizzard, G. Jones and C. Ding (2019). "Vitamin D supplementation improves depressive symptoms in knee osteoarthritis patients over 24 months." European Journal of Immunology **49**: 1421.

Zheng, Y., L. J. Xu, L. Shi, B. Pang and F. Q. Xu (2019). "Modified erxian decoction for peri-menopausal depression: A randomized controlled clinical trial." Psychotherapy and Psychosomatics **88**: 142.

Zhou, Z., S. Fu, Y. Liu, Y. Wang, H. Bu, Y. Mei, Y. Tong and C. Yu (2020). "Study of efficacy and safety of Jiaotai pill in the treatment of depression." Medicine (United States) **99**(18): E19999.

Zimmer, R., T. Riemer, B. Rauch, S. Schneider, R. Schiele, H. Gohlke, F. Diller, G. Steinbeck, H. Katus and J. Senges (2013). "Effects of 1-year treatment with highly purified omega-3 fatty acids on depression after myocardial infarction: Results from the OMEGA trial." Journal of Clinical Psychiatry **74**(11): e1037-e1045.

**e-figure 39:** Baseline severity of the Hamilton Depression Rating Scale (HAM-D) across different study arms.

**e-table 32:** Outliers for HAM-D at baseline.

| **HAM-D** | **author_year** |
| --- | --- |
| **35.82** | Sepehrmanesh Z. et al., 2016 |
| **8** | Piperoglou M. et al., 2023 |
| **45.1** | Caruso I. et al., 1987 |
| **42.4** | Caruso I. et al., 1987 |
| **2.77** | Levine J. et al., 1995 |
| **2.67** | Levine J. et al., 1995 |
| **39.2** | Talaei A. et al., 2023 |
| **39.75** | Talaei A. et al., 2023 |
| **6.16** | Cheng Y. et al., 2025 |
| **7.8** | Cheng Y. et al., 2025 |
| **40** | Bell K.M. et al., 1988 |
| **39** | Bell K.M. et al., 1988 |

# **S9:** Sensitivity analyses

**e-figure 40:** Network plot of **efficacy node 1** – sensitivity analysis retaining only low risk of bias studies and declared clear-cut no sponsored studies**.** Nodes are proportional to sample size; edges’ width is proportional to the number of comparisons.

**e-figure 41:** Forest plots of **efficacy node 1** – sensitivity analysis retaining only low risk of bias studies and declared clear-cut no sponsored studies

**e-figure 42:** Network plot of **efficacy node 1** – sensitivity analysis retaining only low risk of bias studies and no potential effect modifiers outliers. Nodes are proportional to sample size; edges’ width is proportional to the number of comparisons.

**e-figure 43:** Forest plots of **efficacy node 1** – sensitivity analysis retaining only low risk of bias studies and no potential effect modifiers outliers**.**

**e-figure 44:** Network plot of **efficacy node 2** – sensitivity analysis retaining only low risk of bias studies and declared clear-cut no sponsored studies**.** Nodes are proportional to sample size; edges’ width is proportional to the number of comparisons.

**e-figure 45:** Forest plots of **efficacy node 2** – sensitivity analysis retaining only low risk of bias studies and declared clear-cut no sponsored studies.

**e-figure 46:** Network plot of **response node 1** – sensitivity analysis retaining only low risk of bias studies**.** Nodes are proportional to sample size; edges’ width is proportional to the number of comparisons.

**e-figure 47:** Forest plots of **response node 1** – sensitivity analysis retaining only low risk of bias studies

**e-figure 48:** Network plot of **response node 2** – sensitivity analysis retaining only low risk of bias studies**.** Nodes are proportional to sample size; edges’ width is proportional to the number of comparisons.

**e-figure 49:** Forest plots of **response node 2** – sensitivity analysis retaining only low risk of bias studies

**e-figure 50:** Network plot of **acceptability node 1** – sensitivity analysis retaining only low risk of bias studies**.** Nodes are proportional to sample size; edges’ width is proportional to the number of comparisons.

**e-figure 51:** Forest plots of **acceptability node 1** – sensitivity analysis retaining only low risk of bias studies

**e-figure 52:** Network plot of **acceptability node 2**– sensitivity analysis retaining only low risk of bias studies**.** Nodes are proportional to sample size; edges’ width is proportional to the number of comparisons.

**e-figure 53:** Forest plots of **acceptability node 2** – sensitivity analysis retaining only low risk of bias studies

**e-figure 54:** Network plot of **anxious symptoms node 1**– sensitivity analysis retaining only low risk of bias studies**.** Nodes are proportional to sample size; edges’ width is proportional to the number of comparisons.

**e-figure 53:** Forest plots of **anxious symptoms node 1** – sensitivity analysis retaining only low risk of bias studies

**e-figure 54:** Network plot of **anxious symptoms node 2**– sensitivity analysis retaining only low risk of bias studies**.** Nodes are proportional to sample size; edges’ width is proportional to the number of comparisons.

**e-figure 55:** Forest plots of **anxious symptoms node 2** – sensitivity analysis retaining only low risk of bias studies

**e-figure 56:** Network plot of **remission node 1**– sensitivity analysis retaining only low risk of bias studies**.** Nodes are proportional to sample size; edges’ width is proportional to the number of comparisons.

**e-figure 57:** Forest plots of **remission node 1** – sensitivity analysis retaining only low risk of bias studies

**e-figure 58:** Network plot of **remission node 2**– sensitivity analysis retaining only low risk of bias studies**.** Nodes are proportional to sample size; edges’ width is proportional to the number of comparisons.

**e-figure 59:** Forest plots of **remission node 2** – sensitivity analysis retaining only low risk of bias studies

**e-figure 60:** Network plot of **tolerability node 1**– sensitivity analysis retaining only low risk of bias studies**.** Nodes are proportional to sample size; edges’ width is proportional to the number of comparisons.

**e-figure 61:** Forest plots of **tolerability node 1**– sensitivity analysis retaining only low risk of bias studies

**e-figure 62:** Network plot of **tolerability node 2**– sensitivity analysis retaining only low risk of bias studies**.** Nodes are proportional to sample size; edges’ width is proportional to the number of comparisons.

**e-figure 63:** Forest plots of **tolerability node 2**– sensitivity analysis retaining only low risk of bias studies

# **S10:** Subgroup analyses

## **S10.1:** No (semi)structured clinical interview vs. (semi)structured clinical interview

**e-table 33**: Subgroup analysis results (random effect model)

| **Subgroup** | **k** | **SMD** | **95% CI** | **τ²** | **τ** |
| --- | --- | --- | --- | --- | --- |
| No (semi)structured clinical interview | 48 | -0.6150 | [-0.8416; -0.3885] | 0.3339 | 0.5778 |
| (Semi)structured clinical interview | 37 | -0.3711 | [-0.5622; -0.1799] | 0.3339 | 0.5778 |

**e-table 34**: Test for subgroups differences (random effect model)

| **Source** | **Q Statistic** | **Degrees of Freedom (df)** | **p-value** |
| --- | --- | --- | --- |
| **Between groups** | 2.76 | 1 | 0.0967 |
| **Within groups** | 578.39 | 83 | < 0.0001 |

## **S10.2:** Geographical region

**e-table 35:** Subgroup analysis results (random effect model)

| **Subgroup** | **k** | **SMD** | **95% CI** | **τ²** | **τ** |
| --- | --- | --- | --- | --- | --- |
| Asia | 36 | -0.7759 | [-1.0492; -0.5026] | 0.2991 | 0.5469 |
| North America | 22 | -0.2399 | [-0.4541; -0.0257] | 0.2991 | 0.5469 |
| Europe | 20 | -0.4398 | [-0.7398; -0.1399] | 0.2991 | 0.5469 |
| Oceania | 7 | -0.1841 | [-0.5112; 0.1431] | 0.2991 | 0.5469 |

**e-table 36**: Test for subgroups differences (random effect model)

| **Source** | **Q** | **Degrees of Freedom (df)** | **p-value** |
| --- | --- | --- | --- |
| Between groups | 12.82 | 3 | 0.0050 |
| Within groups | 536.88 | 81 | < 0.0001 |

## **S10.3:** Single center vs. Multi center

**e-table 37:** Subgroup analysis results (random effect model)

| **Subgroup** | **k** | **SMD** | **95% CI** | **τ²** | **τ** |
| --- | --- | --- | --- | --- | --- |
| Single-center | 67 | -0.5374 | [-0.7207; -0.3541] | 0.3473 | 0.5893 |
| Multi-center | 18 | -0.4179 | [-0.6862; -0.1496] | 0.3473 | 0.5893 |

**e-table 38**: Test for subgroups differences (random effect model)

| **Source** | **Q** | **Degrees of Freedom (df)** | **p-value** |
| --- | --- | --- | --- |
| Between groups | 0.58 | 1 | 0.4462 |
| Within groups | 614.38 | 83 | < 0.0001 |

## **S10.4:** Phytoceutical vs. Nutraceutical

**e-table 39:** Subgroup analysis results (random effect model)

| **Subgroup** | **k** | **SMD** | **95% CI** | **τ²** | **τ** |
| --- | --- | --- | --- | --- | --- |
| Phytoceutical | 29 | -0.4305 | [-0.6264; -0.2347] | 0.3655 | 0.6045 |
| Nutraceutical | 49 | -0.6239 | [-0.8645; -0.3832] | 0.3655 | 0.6045 |

**e-table 40**: Test for subgroups differences (random effect model)

| **Source** | **Q** | **Degrees of Freedom (df)** | **p-value** |
| --- | --- | --- | --- |
| Between groups | 1.59 | 1 | 0.2070 |
| Within groups | 554.71 | 76 | < 0.0001 |

## **S10.5:** Clinical setting

**e-table 41:** Subgroup analysis results (random effect model)

| **Subgroup** | **k** | **SMD** | **95% CI** | **τ²** | **τ** |
| --- | --- | --- | --- | --- | --- |
| Outpatients | 79 | -0.4779 | [-0.6318; -0.3240] | 0.3271 | 0.5719 |
| Inpatients | 6 | -0.9243 | [-1.8287; -0.0200] | 0.3271 | 0.5719 |

**e-table 42**: Test for subgroups differences (random effect model)

| **Source** | **Q** | **Degrees of Freedom (df)** | **p-value** |
| --- | --- | --- | --- |
| Between groups | 1.54 | 1 | 0.2152 |
| Within groups | 530.88 | 83 | < 0.0001 |

## **S10.6:** Sponsorship

**e-table 43:** Subgroup analysis results (random effect model)

| **Subgroup** | **k** | **SMD** | **95% CI** | **τ²** | **τ** |
| --- | --- | --- | --- | --- | --- |
| Sponsor | 47 | -0.5136 | [-0.7581; -0.2691] | 0.4507 | 0.6713 |
| No sponsor | 7 | -1.0092 | [-1.6212; -0.3972] | 0.4507 | 0.6713 |

**e-table 44**: Test for subgroups differences (random effect model)

| **Source** | **Q** | **Degrees of Freedom (df)** | **p-value** |
| --- | --- | --- | --- |
| Between groups | 3.18 | 1 | 0.0747 |
| Within groups | 351.84 | 52 | < 0.0001 |

## **S10.7:** TAU augmentation vs no TAU augmentation

**e-table 45:** Subgroup analysis results (random effect model)

| **Subgroup** | **k** | **SMD** | **95% CI** | **τ²** | **τ** |
| --- | --- | --- | --- | --- | --- |
| No TAU augmentation | 31 | -0.0936 | [-0.5943; 0.4071] | 1.1153 | 1.0561 |
| TAU augmentation | 15 | -0.8990 | [-1.4125; -0.3855] | 1.1153 | 1.0561 |

**e-table 46**: Test for subgroups differences (random effect model)

| **Source** | **Q** | **Degrees of Freedom (df)** | **p-value** |
| --- | --- | --- | --- |
| Between groups | 5.52 | 1 | 0.0188 |
| Within groups | 301.86 | 44 | < 0.0001 |

## **S10.8:** Classes of molecules

**e-table 47:** Subgroup analysis results (random effect model)

| **Subgroup** | **k** | **SMD** | **95% CI** | **τ²** | **τ** |
| --- | --- | --- | --- | --- | --- |
| Coenzyme | 8 | -0.3462 | [-1.2546; 0.5622] | 0.8708 | 0.9332 |
| Crassulaceae | 2 | -0.5689 | [-1.1265; -0.0114] | 0.8708 | 0.9332 |
| HYP | 11 | -0.7975 | [-1.3803; -0.2146] | 0.8708 | 0.9332 |
| Iridaceae | 5 | -0.0326 | [-0.4720; 0.4068] | 0.8708 | 0.9332 |
| Lamiaceae | 2 | -0.5153 | [-3.9504; 2.9199] | 0.8708 | 0.9332 |
| LCPUFA | 20 | -0.4148 | [-0.9163; 0.0867] | 0.8708 | 0.9332 |
| Minerals | 3 | -0.8479 | [-1.2595; -0.4364] | 0.8708 | 0.9332 |
| PROB | 6 | -0.7655 | [-1.9241; 0.3931] | 0.8708 | 0.9332 |
| Polysaccarid | 2 | 0.7975 | [-12.9331; 14.5281] | 0.8708 | 0.9332 |
| Vitamins | 11 | -0.1881 | [-0.9764; 0.6002] | 0.8708 | 0.9332 |
| Zingiberaceae | 4 | -1.2787 | [-2.6500; 0.0926] | 0.8708 | 0.9332 |

**e-table 48**: Test for subgroups differences (random effect model)

| **Source** | **Q** | **Degrees of Freedom (df)** | **p-value** |
| --- | --- | --- | --- |
| Between groups | 27.12 | 10 | 0.0025 |
| Within groups | 717.84 | 63 | < 0.0001 |

## **S10.9:** Treatment-resistant depression

**e-table 48:** Subgroup analysis results (random effect model)

| **Subgroup** | **k** | **SMD** | **95% CI** | **τ²** | **τ** |
| --- | --- | --- | --- | --- | --- |
| Treatment-resistant depression | 4 | 0.1262 | [-0.6869; 0.9393] | 0.3314 | 0.5757 |
| Non treatment-resistant depression | 81 | -0.4942 | [-0.6495; -0.3390] | 0.3314 | 0.5757 |

**e-table 49**: Test for subgroups differences (random effect model)

| **Source** | **Q** | **Degrees of Freedom (df)** | **p-value** |
| --- | --- | --- | --- |
| Between groups | 5.39 | 1 | 0.0202 |
| Within groups | 561.18 | 83 | < 0.0001 |

## **S11:** Evaluating the confidence in NMA

## **S11.1**: CINeMA methods and reasoning

The decisions about the domain rating for single studies and whole analysis were undertaken according to the CINeMA methodological guide (10.1371/journal.pmed.1003082).

**Within-study bias. The Cochrane risk-of-bias tool for randomized trials (RoB 2)** (cross-over) assessed studies for randomization, allocation concealment, blinding, missing data, selective reporting, carryover effect, and measure appropriateness. Considering an overall rating, each study was rated as low, unclear, or high risk of bias. All unclearly randomized studies and studies with unclear potential for carryover effect were excluded to avoid hindering the transitivity assumption. The overall *‘within-study bias’* reflects the majority risk of bias for each comparison.

The risk of bias is summarized in net graphs and contribution bar charts, which show study contributions to the risk of bias of a single intervention and each comparison. Low, unclear, and high risk of bias are shown in green, yellow, and red. The colors of nodes reflect the proportion of studies with each risk of bias, and the colors of edges reflect the majority risk of bias within each comparison.

**Reporting bias.** Our decisions about reporting bias reflected: i) small-study effect as per funnel plot visual inspection and Egger’s p-value, ii) search strategy broadness; iii) newly introduced drugs on the market (early evidence may overestimate efficacy and safety); iv) trial funding. Given the high percentage of newly introduced drugs and funded trials in our analyses, notwithstanding the absence of a small-study effect in any evaluated outcomes, we conservatively set the reporting bias to ‘*suspected*’.

**Indirectness.** Each study was assessed according to its relevance to the research question and comparability to other studies. The overall analysis rated all simulated driving studies as having moderate indirectness (or more if other effect modifiers were present). Information for each comparison is provided in a contribution bar chart—green, yellow, and red show low, mild, and high indirectness, respectively. The overall *‘indirectness’* judgment reflects most of the indirectness for each comparison.

**Range of equivalence for imprecision and heterogeneity.** We assumed a clinically meaningful effect as <-0.3 and >0.3 in SMD-based analyses. We assumed a clinically meaningful effect as <0.8 and >1.2 in RR-based analyses. *Small effects were considered important due to the topic's relevance*.

**Overall rating**. According to the CINeMA methodological guide, we downgraded the confidence in evidence by one point for each domain with a *‘some concerns’* rating and two points for each domain with a *‘major concerns’* rating. Considering that *imprecision*, *heterogeneity,* and *incoherence* are interconnected, we downgraded the evidence by one or two points only once whenever multiple domains respectively showed ‘*some* or major concerns.

## **S11.2** NMA confidence rating for *efficacy, symptom change from baseline*

**e-figure 64:** Netgraph. Nodes are represented according to the proportion of studies with each level of risk of bias. Edges are described according to the majority risk of bias within the comparison. Node size is equal due to graphical reasons. Edges’ width is proportional to the sample size. Red stands for high risk of bias, yellow for unclear, and green for low risk of bias

**e-table 50:** CINeMA GRADE evaluation of symptom changes from baseline.

| **Comparison** | **Number of studies** | **Within-study bias** | **Reporting bias** | **Indirectness** | **Imprecision** | **Heterogeneity** | **Incoherence** | **Confidence rating** |
| --- | --- | --- | --- | --- | --- | --- | --- | --- |
| AMI:LI-160 | 1 | No concerns | Low risk | No concerns | Major concerns | No concerns | Major concerns | Low |
| APH:PLB | 1 | No concerns | Low risk | No concerns | Major concerns | No concerns | Major concerns | Low |
| BZT:PLB | 1 | No concerns | Low risk | No concerns | Major concerns | No concerns | No concerns | Low |
| BZT:SER | 1 | No concerns | Low risk | No concerns | Major concerns | No concerns | No concerns | Low |
| CHRO:PLB | 2 | No concerns | Low risk | No concerns | Major concerns | No concerns | Major concerns | Low |
| CIT:PLB | 1 | No concerns | Low risk | No concerns | Major concerns | No concerns | Major concerns | Low |
| CREA:PLB | 1 | No concerns | Low risk | No concerns | Major concerns | No concerns | Major concerns | Low |
| CTP:SAFF | 1 | No concerns | Low risk | No concerns | Major concerns | No concerns | Major concerns | Low |
| CUR:CUR+SAFF | 1 | No concerns | Low risk | No concerns | Major concerns | No concerns | No concerns | Low |
| CUR:PLB | 4 | No concerns | Low risk | No concerns | Major concerns | No concerns | Major concerns | Low |
| CUR+SAFF:PLB | 1 | No concerns | Low risk | No concerns | Major concerns | No concerns | No concerns | Low |
| DHA:EPA | 1 | No concerns | Low risk | No concerns | No concerns | No concerns | Major concerns | Low |
| DHA:PLB | 2 | No concerns | Low risk | No concerns | Major concerns | No concerns | Major concerns | Low |
| EOPF:PLB | 1 | Some concerns | Low risk | No concerns | Some concerns | Some concerns | Major concerns | Very Low |
| EPA:PLB | 4 | No concerns | Low risk | No concerns | No concerns | Some concerns | No concerns | Low |
| EPA+DHA:PLB | 10 | No concerns | Low risk | No concerns | Some concerns | Some concerns | Major concerns | Low |
| EPA+DHA:RHO+SER | 1 | No concerns | Low risk | No concerns | No concerns | No concerns | No concerns | Low |
| ESC:ESC+FOL | 1 | No concerns | Low risk | No concerns | Major concerns | No concerns | Major concerns | Low |
| ESC:PLB | 1 | No concerns | Low risk | No concerns | Major concerns | No concerns | No concerns | Low |
| ESC:SAMe | 1 | No concerns | Low risk | No concerns | Major concerns | No concerns | No concerns | Low |
| FLUO:LH57 | 1 | No concerns | Low risk | Some concerns | Major concerns | No concerns | Major concerns | Very Low |
| FLUO:LI-160 | 1 | No concerns | Low risk | No concerns | Major concerns | No concerns | No concerns | Low |
| FLUO:PLB | 1 | Some concerns | Low risk | No concerns | Major concerns | No concerns | No concerns | Very Low |
| FLUO:SAFF | 3 | No concerns | Low risk | No concerns | No concerns | Major concerns | No concerns | Low |
| FLUO:SJW | 1 | No concerns | Low risk | No concerns | Major concerns | No concerns | No concerns | Low |
| FLUO:SZL | 1 | No concerns | Low risk | No concerns | Major concerns | No concerns | Major concerns | Low |
| FLUO:ZE117 | 1 | No concerns | Low risk | No concerns | Some concerns | Some concerns | No concerns | Low |
| FLUO:vitD3+FLUO | 1 | No concerns | Low risk | No concerns | Major concerns | No concerns | Major concerns | Low |
| FOIL:KOIL | 1 | No concerns | Low risk | No concerns | Major concerns | No concerns | Major concerns | Low |
| FOIL:PLB | 1 | No concerns | Low risk | No concerns | Major concerns | No concerns | Major concerns | Low |
| GBDP:PLB | 1 | No concerns | Low risk | No concerns | Major concerns | No concerns | Major concerns | Low |
| IMI:LAV | 1 | No concerns | Low risk | No concerns | Major concerns | No concerns | Major concerns | Low |
| IMI:LAV+IMI | 1 | No concerns | Low risk | No concerns | Some concerns | Some concerns | Major concerns | Low |
| IMI:LI-160 | 1 | No concerns | Low risk | No concerns | Major concerns | No concerns | Major concerns | Low |
| IMI:ZE | 1 | No concerns | Low risk | No concerns | Major concerns | No concerns | Major concerns | Low |
| INO:PLB | 1 | No concerns | Low risk | No concerns | Major concerns | No concerns | Major concerns | Low |
| INU:PLB | 1 | No concerns | Low risk | No concerns | Major concerns | No concerns | Major concerns | Low |
| KOIL:PLB | 1 | No concerns | Low risk | No concerns | Major concerns | No concerns | Major concerns | Low |
| LAV:LAV+IMI | 1 | No concerns | Low risk | No concerns | Major concerns | No concerns | Major concerns | Low |
| LI-160:PLB | 2 | No concerns | Low risk | No concerns | Major concerns | No concerns | No concerns | Low |
| LI-160:SER | 1 | No concerns | Low risk | No concerns | Major concerns | No concerns | No concerns | Low |
| LMF:PLB | 2 | No concerns | Low risk | No concerns | Major concerns | No concerns | Major concerns | Low |
| LP299v:PLB | 1 | No concerns | Low risk | No concerns | Major concerns | No concerns | Major concerns | Low |
| LS:PLB | 1 | No concerns | Low risk | No concerns | Major concerns | No concerns | Major concerns | Low |
| MEL:PLB | 1 | No concerns | Low risk | No concerns | Major concerns | No concerns | Major concerns | Low |
| MG:PLB | 1 | No concerns | Low risk | No concerns | Major concerns | No concerns | Major concerns | Low |
| NUT:PLB | 1 | No concerns | Low risk | No concerns | Major concerns | No concerns | Major concerns | Low |
| OMB:PLB | 1 | No concerns | Low risk | No concerns | Major concerns | No concerns | Major concerns | Low |
| OMEGA3:PLB | 3 | No concerns | Low risk | No concerns | Major concerns | No concerns | Major concerns | Low |
| PAR:WS5570 | 1 | No concerns | Low risk | No concerns | Major concerns | No concerns | Major concerns | Low |
| PROB:PLB | 1 | No concerns | Low risk | No concerns | Major concerns | No concerns | Major concerns | Low |
| PLB:PS128 | 1 | No concerns | Low risk | No concerns | Major concerns | No concerns | Major concerns | Low |
| PLB:RHO | 1 | No concerns | Low risk | No concerns | No concerns | No concerns | Major concerns | Low |
| PLB:RHO+SER | 1 | No concerns | Low risk | No concerns | No concerns | No concerns | No concerns | Low |
| PLB:SAF | 2 | No concerns | Low risk | Some concerns | Major concerns | No concerns | No concerns | Very Low |
| PLB:SAFF | 3 | No concerns | Low risk | No concerns | No concerns | Major concerns | No concerns | Low |
| PLB:SAFF+SCU | 1 | No concerns | Low risk | No concerns | Major concerns | No concerns | No concerns | Low |
| PLB:SAMe | 4 | No concerns | Low risk | No concerns | Some concerns | Some concerns | Major concerns | Low |
| PLB:SAMe+HELP9 | 1 | No concerns | Low risk | No concerns | Major concerns | No concerns | Major concerns | Low |
| PLB:SCU | 1 | No concerns | Low risk | No concerns | Major concerns | No concerns | No concerns | Low |
| PLB:SER | 1 | Some concerns | Low risk | No concerns | Some concerns | Some concerns | No concerns | Very Low |
| PLB:SJW | 1 | No concerns | Low risk | No concerns | Major concerns | No concerns | No concerns | Low |
| PLB:SRN | 1 | No concerns | Low risk | No concerns | Major concerns | No concerns | Major concerns | Low |
| PLB:TEN | 1 | No concerns | Low risk | No concerns | No concerns | No concerns | Major concerns | Low |
| PLB:WS5570 | 2 | No concerns | Low risk | No concerns | Major concerns | No concerns | Major concerns | Low |
| PLB:WS5572 | 2 | No concerns | Low risk | No concerns | Major concerns | No concerns | Major concerns | Low |
| PLB:WS5573 | 1 | No concerns | Low risk | Some concerns | Major concerns | No concerns | No concerns | Very Low |
| PLB:ZE117 | 1 | No concerns | Low risk | No concerns | Some concerns | Some concerns | No concerns | Low |
| PLB:vitB | 1 | Some concerns | Low risk | No concerns | No concerns | Some concerns | Major concerns | Very Low |
| PLB:vitD | 3 | No concerns | Low risk | No concerns | Major concerns | No concerns | Major concerns | Low |
| PLB:vitD3 | 2 | No concerns | Low risk | No concerns | No concerns | Major concerns | Major concerns | Low |
| PLB:vitD3+CA | 1 | No concerns | Low risk | No concerns | Major concerns | No concerns | No concerns | Low |
| SAF:SJW | 1 | No concerns | Low risk | No concerns | Major concerns | No concerns | No concerns | Low |
| SAFF:SAFF+SCU | 1 | No concerns | Low risk | No concerns | Major concerns | No concerns | No concerns | Low |
| SAFF:SCU | 1 | No concerns | Low risk | No concerns | Major concerns | No concerns | No concerns | Low |
| SAFF:SER | 1 | No concerns | Low risk | No concerns | Major concerns | No concerns | No concerns | Low |
| SAFF+SCU:SCU | 1 | No concerns | Low risk | No concerns | Major concerns | No concerns | Major concerns | Low |
| SER:SJW | 1 | No concerns | Low risk | No concerns | Major concerns | No concerns | No concerns | Low |
| WS5572:WS5573 | 1 | No concerns | Low risk | No concerns | Major concerns | No concerns | No concerns | Low |
| vitD3:vitD3+CA | 1 | Some concerns | Low risk | No concerns | Major concerns | No concerns | No concerns | Very Low |
| AMI:APH | 0 | No concerns | Low risk | No concerns | Major concerns | No concerns | Major concerns | Low |
| AMI:BZT | 0 | No concerns | Low risk | No concerns | Major concerns | No concerns | Major concerns | Low |
| AMI:CHRO | 0 | No concerns | Low risk | No concerns | Major concerns | No concerns | Major concerns | Low |
| AMI:CIT | 0 | No concerns | Low risk | No concerns | Major concerns | No concerns | Major concerns | Low |
| AMI:CREA | 0 | No concerns | Low risk | No concerns | Major concerns | No concerns | Major concerns | Low |
| AMI:CTP | 0 | No concerns | Low risk | No concerns | Major concerns | No concerns | Major concerns | Low |
| AMI:CUR | 0 | No concerns | Low risk | No concerns | Major concerns | No concerns | Major concerns | Low |
| AMI:CUR+SAFF | 0 | No concerns | Low risk | No concerns | Major concerns | No concerns | Major concerns | Low |
| AMI:DHA | 0 | No concerns | Low risk | No concerns | Some concerns | Some concerns | Major concerns | Low |
| AMI:EOPF | 0 | No concerns | Low risk | No concerns | Major concerns | No concerns | Major concerns | Low |
| AMI:EPA | 0 | No concerns | Low risk | No concerns | Major concerns | No concerns | Major concerns | Low |
| AMI:EPA+DHA | 0 | No concerns | Low risk | No concerns | Major concerns | No concerns | Major concerns | Low |
| AMI:ESC | 0 | No concerns | Low risk | No concerns | Major concerns | No concerns | Major concerns | Low |
| AMI:ESC+FOL | 0 | No concerns | Low risk | No concerns | Major concerns | No concerns | Major concerns | Low |
| AMI:FLUO | 0 | No concerns | Low risk | No concerns | Major concerns | No concerns | Major concerns | Low |
| AMI:FOIL | 0 | No concerns | Low risk | No concerns | Major concerns | No concerns | Major concerns | Low |
| AMI:GBDP | 0 | No concerns | Low risk | No concerns | Major concerns | No concerns | Major concerns | Low |
| AMI:IMI | 0 | No concerns | Low risk | No concerns | Major concerns | No concerns | Major concerns | Low |
| AMI:INO | 0 | No concerns | Low risk | No concerns | Major concerns | No concerns | Major concerns | Low |
| AMI:INU | 0 | No concerns | Low risk | No concerns | Major concerns | No concerns | Major concerns | Low |
| AMI:KOIL | 0 | No concerns | Low risk | No concerns | Major concerns | No concerns | Major concerns | Low |
| AMI:LAV | 0 | No concerns | Low risk | No concerns | Major concerns | No concerns | Major concerns | Low |
| AMI:LAV+IMI | 0 | No concerns | Low risk | No concerns | Major concerns | No concerns | Major concerns | Low |
| AMI:LH57 | 0 | No concerns | Low risk | No concerns | Major concerns | No concerns | Major concerns | Low |
| AMI:LMF | 0 | No concerns | Low risk | No concerns | Major concerns | No concerns | Major concerns | Low |
| AMI:LP299v | 0 | Some concerns | Low risk | No concerns | Major concerns | No concerns | Major concerns | Very Low |
| AMI:LS | 0 | No concerns | Low risk | No concerns | Major concerns | No concerns | Major concerns | Low |
| AMI:MEL | 0 | No concerns | Low risk | No concerns | Major concerns | No concerns | Major concerns | Low |
| AMI:MG | 0 | No concerns | Low risk | No concerns | Major concerns | No concerns | Major concerns | Low |
| AMI:NUT | 0 | No concerns | Low risk | No concerns | Major concerns | No concerns | Major concerns | Low |
| AMI:OMB | 0 | No concerns | Low risk | No concerns | Major concerns | No concerns | Major concerns | Low |
| AMI:OMEGA3 | 0 | No concerns | Low risk | No concerns | Major concerns | No concerns | Major concerns | Low |
| AMI:PAR | 0 | No concerns | Low risk | No concerns | Major concerns | No concerns | Major concerns | Low |
| AMI:PROB | 0 | No concerns | Low risk | No concerns | Major concerns | No concerns | Major concerns | Low |
| AMI:PLB | 0 | No concerns | Low risk | No concerns | Major concerns | No concerns | Major concerns | Low |
| AMI:PS128 | 0 | No concerns | Low risk | No concerns | Major concerns | No concerns | Major concerns | Low |
| AMI:RHO | 0 | No concerns | Low risk | No concerns | Major concerns | No concerns | Major concerns | Low |
| AMI:RHO+SER | 0 | No concerns | Low risk | No concerns | No concerns | No concerns | Major concerns | Low |
| AMI:SAF | 0 | No concerns | Low risk | No concerns | Major concerns | No concerns | Major concerns | Low |
| AMI:SAFF | 0 | No concerns | Low risk | No concerns | Major concerns | No concerns | Major concerns | Low |
| AMI:SAFF+SCU | 0 | No concerns | Low risk | No concerns | Major concerns | No concerns | Major concerns | Low |
| AMI:SAMe | 0 | No concerns | Low risk | No concerns | Major concerns | No concerns | Major concerns | Low |
| AMI:SAMe+HELP9 | 0 | No concerns | Low risk | No concerns | Major concerns | No concerns | Major concerns | Low |
| AMI:SCU | 0 | No concerns | Low risk | Some concerns | Major concerns | No concerns | Major concerns | Very Low |
| AMI:SER | 0 | No concerns | Low risk | No concerns | Major concerns | No concerns | Major concerns | Low |
| AMI:SJW | 0 | Some concerns | Low risk | No concerns | Major concerns | No concerns | Major concerns | Very Low |
| AMI:SRN | 0 | No concerns | Low risk | No concerns | Major concerns | No concerns | Major concerns | Low |
| AMI:SZL | 0 | No concerns | Low risk | No concerns | Major concerns | No concerns | Major concerns | Low |
| AMI:TEN | 0 | No concerns | Low risk | No concerns | Major concerns | No concerns | Major concerns | Low |
| AMI:WS5570 | 0 | No concerns | Low risk | No concerns | Major concerns | No concerns | Major concerns | Low |
| AMI:WS5572 | 0 | No concerns | Low risk | No concerns | Major concerns | No concerns | Major concerns | Low |
| AMI:WS5573 | 0 | No concerns | Low risk | No concerns | Major concerns | No concerns | Major concerns | Low |
| AMI:ZE | 0 | No concerns | Low risk | No concerns | Major concerns | No concerns | Major concerns | Low |
| AMI:ZE117 | 0 | No concerns | Low risk | Some concerns | Major concerns | No concerns | Major concerns | Very Low |
| AMI:vitB | 0 | No concerns | Low risk | No concerns | Major concerns | No concerns | Major concerns | Low |
| AMI:vitD | 0 | No concerns | Low risk | No concerns | Major concerns | No concerns | Major concerns | Low |
| AMI:vitD3 | 0 | No concerns | Low risk | No concerns | Major concerns | No concerns | Major concerns | Low |
| AMI:vitD3+CA | 0 | No concerns | Low risk | No concerns | Major concerns | No concerns | Major concerns | Low |
| AMI:vitD3+FLUO | 0 | No concerns | Low risk | No concerns | Major concerns | No concerns | Major concerns | Low |
| APH:BZT | 0 | No concerns | Low risk | No concerns | Major concerns | No concerns | Major concerns | Low |
| APH:CHRO | 0 | No concerns | Low risk | No concerns | Major concerns | No concerns | Major concerns | Low |
| APH:CIT | 0 | No concerns | Low risk | No concerns | Major concerns | No concerns | Major concerns | Low |
| APH:CREA | 0 | No concerns | Low risk | No concerns | Major concerns | No concerns | Major concerns | Low |
| APH:CTP | 0 | No concerns | Low risk | No concerns | Major concerns | No concerns | Major concerns | Low |
| APH:CUR | 0 | No concerns | Low risk | No concerns | Major concerns | No concerns | Major concerns | Low |
| APH:CUR+SAFF | 0 | No concerns | Low risk | No concerns | Major concerns | No concerns | Major concerns | Low |
| APH:DHA | 0 | No concerns | Low risk | No concerns | Some concerns | Some concerns | Major concerns | Low |
| APH:EOPF | 0 | No concerns | Low risk | No concerns | Major concerns | No concerns | Major concerns | Low |
| APH:EPA | 0 | No concerns | Low risk | No concerns | Major concerns | No concerns | Major concerns | Low |
| APH:EPA+DHA | 0 | No concerns | Low risk | No concerns | Major concerns | No concerns | Major concerns | Low |
| APH:ESC | 0 | No concerns | Low risk | No concerns | Major concerns | No concerns | Major concerns | Low |
| APH:ESC+FOL | 0 | No concerns | Low risk | No concerns | Major concerns | No concerns | Major concerns | Low |
| APH:FLUO | 0 | No concerns | Low risk | No concerns | Major concerns | No concerns | Major concerns | Low |
| APH:FOIL | 0 | No concerns | Low risk | No concerns | Major concerns | No concerns | Major concerns | Low |
| APH:GBDP | 0 | No concerns | Low risk | No concerns | Major concerns | No concerns | Major concerns | Low |
| APH:IMI | 0 | No concerns | Low risk | No concerns | Major concerns | No concerns | Major concerns | Low |
| APH:INO | 0 | No concerns | Low risk | No concerns | Major concerns | No concerns | Major concerns | Low |
| APH:INU | 0 | No concerns | Low risk | No concerns | Major concerns | No concerns | Major concerns | Low |
| APH:KOIL | 0 | No concerns | Low risk | No concerns | Major concerns | No concerns | Major concerns | Low |
| APH:LAV | 0 | Some concerns | Low risk | No concerns | Major concerns | No concerns | Major concerns | Very Low |
| APH:LAV+IMI | 0 | No concerns | Low risk | No concerns | Major concerns | No concerns | Major concerns | Low |
| APH:LH57 | 0 | No concerns | Low risk | No concerns | Major concerns | No concerns | Major concerns | Low |
| APH:LI-160 | 0 | No concerns | Low risk | No concerns | Major concerns | No concerns | Major concerns | Low |
| APH:LMF | 0 | No concerns | Low risk | Some concerns | Major concerns | No concerns | Major concerns | Very Low |
| APH:LP299v | 0 | No concerns | Low risk | No concerns | Major concerns | No concerns | Major concerns | Low |
| APH:LS | 0 | No concerns | Low risk | No concerns | Major concerns | No concerns | Major concerns | Low |
| APH:MEL | 0 | No concerns | Low risk | No concerns | Major concerns | No concerns | Major concerns | Low |
| APH:MG | 0 | No concerns | Low risk | No concerns | Major concerns | No concerns | Major concerns | Low |
| APH:NUT | 0 | No concerns | Low risk | No concerns | Major concerns | No concerns | Major concerns | Low |
| APH:OMB | 0 | Some concerns | Low risk | No concerns | Major concerns | No concerns | Major concerns | Very Low |
| APH:OMEGA3 | 0 | No concerns | Low risk | No concerns | Major concerns | No concerns | Major concerns | Low |
| APH:PAR | 0 | No concerns | Low risk | No concerns | Major concerns | No concerns | Major concerns | Low |
| APH:PROB | 0 | No concerns | Low risk | No concerns | Major concerns | No concerns | Major concerns | Low |
| APH:PS128 | 0 | No concerns | Low risk | Some concerns | Major concerns | No concerns | Major concerns | Very Low |
| APH:RHO | 0 | No concerns | Low risk | No concerns | Major concerns | No concerns | Major concerns | Low |
| APH:RHO+SER | 0 | No concerns | Low risk | No concerns | No concerns | No concerns | Major concerns | Low |
| APH:SAF | 0 | No concerns | Low risk | No concerns | Major concerns | No concerns | Major concerns | Low |
| APH:SAFF | 0 | No concerns | Low risk | No concerns | Major concerns | No concerns | Major concerns | Low |
| APH:SAFF+SCU | 0 | No concerns | Low risk | No concerns | Major concerns | No concerns | Major concerns | Low |
| APH:SAMe | 0 | No concerns | Low risk | No concerns | Major concerns | No concerns | Major concerns | Low |
| APH:SAMe+HELP9 | 0 | No concerns | Low risk | No concerns | Major concerns | No concerns | Major concerns | Low |
| APH:SCU | 0 | No concerns | Low risk | No concerns | Major concerns | No concerns | Major concerns | Low |
| APH:SER | 0 | No concerns | Low risk | No concerns | Major concerns | No concerns | Major concerns | Low |
| APH:SJW | 0 | No concerns | Low risk | No concerns | Major concerns | No concerns | Major concerns | Low |
| APH:SRN | 0 | No concerns | Low risk | No concerns | Major concerns | No concerns | Major concerns | Low |
| APH:SZL | 0 | No concerns | Low risk | No concerns | Major concerns | No concerns | Major concerns | Low |
| APH:TEN | 0 | No concerns | Low risk | No concerns | Major concerns | No concerns | Major concerns | Low |
| APH:WS5570 | 0 | No concerns | Low risk | No concerns | Major concerns | No concerns | Major concerns | Low |
| APH:WS5572 | 0 | No concerns | Low risk | No concerns | Major concerns | No concerns | Major concerns | Low |
| APH:WS5573 | 0 | No concerns | Low risk | No concerns | Major concerns | No concerns | Major concerns | Low |
| APH:ZE | 0 | No concerns | Low risk | No concerns | Major concerns | No concerns | Major concerns | Low |
| APH:ZE117 | 0 | No concerns | Low risk | No concerns | Major concerns | No concerns | Major concerns | Low |
| APH:vitB | 0 | No concerns | Low risk | No concerns | Major concerns | No concerns | Major concerns | Low |
| APH:vitD | 0 | No concerns | Low risk | No concerns | Major concerns | No concerns | Major concerns | Low |
| APH:vitD3 | 0 | No concerns | Low risk | No concerns | Major concerns | No concerns | Major concerns | Low |
| APH:vitD3+CA | 0 | No concerns | Low risk | No concerns | Major concerns | No concerns | Major concerns | Low |
| APH:vitD3+FLUO | 0 | No concerns | Low risk | No concerns | Major concerns | No concerns | Major concerns | Low |
| BZT:CHRO | 0 | No concerns | Low risk | No concerns | Major concerns | No concerns | Major concerns | Low |
| BZT:CIT | 0 | Some concerns | Low risk | No concerns | Major concerns | No concerns | Major concerns | Very Low |
| BZT:CREA | 0 | No concerns | Low risk | No concerns | Major concerns | No concerns | Major concerns | Low |
| BZT:CTP | 0 | No concerns | Low risk | No concerns | Major concerns | No concerns | Major concerns | Low |
| BZT:CUR | 0 | No concerns | Low risk | No concerns | Major concerns | No concerns | Major concerns | Low |
| BZT:CUR+SAFF | 0 | No concerns | Low risk | No concerns | Major concerns | No concerns | Major concerns | Low |
| BZT:DHA | 0 | No concerns | Low risk | No concerns | Major concerns | No concerns | Major concerns | Low |
| BZT:EOPF | 0 | No concerns | Low risk | No concerns | Major concerns | No concerns | Major concerns | Low |
| BZT:EPA | 0 | No concerns | Low risk | No concerns | Some concerns | Some concerns | Major concerns | Low |
| BZT:EPA+DHA | 0 | No concerns | Low risk | No concerns | Major concerns | No concerns | Major concerns | Low |
| BZT:ESC | 0 | No concerns | Low risk | No concerns | Major concerns | No concerns | Major concerns | Low |
| BZT:ESC+FOL | 0 | No concerns | Low risk | No concerns | Major concerns | No concerns | Major concerns | Low |
| BZT:FLUO | 0 | No concerns | Low risk | No concerns | Major concerns | No concerns | Major concerns | Low |
| BZT:FOIL | 0 | No concerns | Low risk | No concerns | Major concerns | No concerns | Major concerns | Low |
| BZT:GBDP | 0 | No concerns | Low risk | No concerns | Major concerns | No concerns | Major concerns | Low |
| BZT:IMI | 0 | No concerns | Low risk | No concerns | Major concerns | No concerns | Major concerns | Low |
| BZT:INO | 0 | No concerns | Low risk | No concerns | Major concerns | No concerns | Major concerns | Low |
| BZT:INU | 0 | No concerns | Low risk | No concerns | Major concerns | No concerns | Major concerns | Low |
| BZT:KOIL | 0 | No concerns | Low risk | No concerns | Major concerns | No concerns | Major concerns | Low |
| BZT:LAV | 0 | No concerns | Low risk | No concerns | Major concerns | No concerns | Major concerns | Low |
| BZT:LAV+IMI | 0 | No concerns | Low risk | No concerns | Major concerns | No concerns | Major concerns | Low |
| BZT:LH57 | 0 | No concerns | Low risk | No concerns | Major concerns | No concerns | Major concerns | Low |
| BZT:LI-160 | 0 | No concerns | Low risk | No concerns | Major concerns | No concerns | Major concerns | Low |
| BZT:LMF | 0 | No concerns | Low risk | No concerns | Major concerns | No concerns | Major concerns | Low |
| BZT:LP299v | 0 | No concerns | Low risk | No concerns | Major concerns | No concerns | Major concerns | Low |
| BZT:LS | 0 | No concerns | Low risk | No concerns | Major concerns | No concerns | Major concerns | Low |
| BZT:MEL | 0 | No concerns | Low risk | No concerns | Major concerns | No concerns | Major concerns | Low |
| BZT:MG | 0 | No concerns | Low risk | No concerns | Major concerns | No concerns | Major concerns | Low |
| BZT:NUT | 0 | No concerns | Low risk | No concerns | Major concerns | No concerns | Major concerns | Low |
| BZT:OMB | 0 | No concerns | Low risk | No concerns | Major concerns | No concerns | Major concerns | Low |
| BZT:OMEGA3 | 0 | No concerns | Low risk | No concerns | Major concerns | No concerns | Major concerns | Low |
| BZT:PAR | 0 | No concerns | Low risk | Some concerns | Major concerns | No concerns | Major concerns | Very Low |
| BZT:PROB | 0 | No concerns | Low risk | No concerns | Major concerns | No concerns | Major concerns | Low |
| BZT:PS128 | 0 | No concerns | Low risk | No concerns | Major concerns | No concerns | Major concerns | Low |
| BZT:RHO | 0 | No concerns | Low risk | No concerns | No concerns | Major concerns | Major concerns | Low |
| BZT:RHO+SER | 0 | No concerns | Low risk | No concerns | No concerns | No concerns | Major concerns | Low |
| BZT:SAF | 0 | No concerns | Low risk | No concerns | Major concerns | No concerns | Major concerns | Low |
| BZT:SAFF | 0 | No concerns | Low risk | No concerns | Major concerns | No concerns | Major concerns | Low |
| BZT:SAFF+SCU | 0 | No concerns | Low risk | No concerns | Major concerns | No concerns | Major concerns | Low |
| BZT:SAMe | 0 | No concerns | Low risk | No concerns | Major concerns | No concerns | Major concerns | Low |
| BZT:SAMe+HELP9 | 0 | No concerns | Low risk | No concerns | Major concerns | No concerns | Major concerns | Low |
| BZT:SCU | 0 | No concerns | Low risk | No concerns | Major concerns | No concerns | Major concerns | Low |
| BZT:SJW | 0 | No concerns | Low risk | No concerns | Major concerns | No concerns | Major concerns | Low |
| BZT:SRN | 0 | No concerns | Low risk | No concerns | Major concerns | No concerns | Major concerns | Low |
| BZT:SZL | 0 | No concerns | Low risk | No concerns | Major concerns | No concerns | Major concerns | Low |
| BZT:TEN | 0 | No concerns | Low risk | No concerns | Some concerns | Some concerns | Major concerns | Low |
| BZT:WS5570 | 0 | No concerns | Low risk | No concerns | Major concerns | No concerns | Major concerns | Low |
| BZT:WS5572 | 0 | No concerns | Low risk | No concerns | Major concerns | No concerns | Major concerns | Low |
| BZT:WS5573 | 0 | No concerns | Low risk | No concerns | Major concerns | No concerns | Major concerns | Low |
| BZT:ZE | 0 | No concerns | Low risk | No concerns | Major concerns | No concerns | Major concerns | Low |
| BZT:ZE117 | 0 | No concerns | Low risk | No concerns | Major concerns | No concerns | Major concerns | Low |
| BZT:vitB | 0 | No concerns | Low risk | No concerns | Major concerns | No concerns | Major concerns | Low |
| BZT:vitD | 0 | No concerns | Low risk | No concerns | Major concerns | No concerns | Major concerns | Low |
| BZT:vitD3 | 0 | No concerns | Low risk | No concerns | Major concerns | No concerns | Major concerns | Low |
| BZT:vitD3+CA | 0 | No concerns | Low risk | No concerns | Major concerns | No concerns | Major concerns | Low |
| BZT:vitD3+FLUO | 0 | No concerns | Low risk | No concerns | Major concerns | No concerns | Major concerns | Low |
| CHRO:CIT | 0 | No concerns | Low risk | No concerns | Major concerns | No concerns | Major concerns | Low |
| CHRO:CREA | 0 | No concerns | Low risk | No concerns | Major concerns | No concerns | Major concerns | Low |
| CHRO:CTP | 0 | No concerns | Low risk | No concerns | Major concerns | No concerns | Major concerns | Low |
| CHRO:CUR | 0 | No concerns | Low risk | No concerns | Major concerns | No concerns | Major concerns | Low |
| CHRO:CUR+SAFF | 0 | No concerns | Low risk | No concerns | Major concerns | No concerns | Major concerns | Low |
| CHRO:DHA | 0 | No concerns | Low risk | No concerns | Major concerns | No concerns | Major concerns | Low |
| CHRO:EOPF | 0 | No concerns | Low risk | No concerns | Major concerns | No concerns | Major concerns | Low |
| CHRO:EPA | 0 | No concerns | Low risk | No concerns | Some concerns | Some concerns | Major concerns | Low |
| CHRO:EPA+DHA | 0 | No concerns | Low risk | No concerns | Major concerns | No concerns | Major concerns | Low |
| CHRO:ESC | 0 | No concerns | Low risk | No concerns | Major concerns | No concerns | Major concerns | Low |
| CHRO:ESC+FOL | 0 | No concerns | Low risk | No concerns | Major concerns | No concerns | Major concerns | Low |
| CHRO:FLUO | 0 | No concerns | Low risk | No concerns | Major concerns | No concerns | Major concerns | Low |
| CHRO:FOIL | 0 | No concerns | Low risk | No concerns | Major concerns | No concerns | Major concerns | Low |
| CHRO:GBDP | 0 | No concerns | Low risk | No concerns | Major concerns | No concerns | Major concerns | Low |
| CHRO:IMI | 0 | No concerns | Low risk | No concerns | Major concerns | No concerns | Major concerns | Low |
| CHRO:INO | 0 | No concerns | Low risk | Some concerns | Major concerns | No concerns | Major concerns | Very Low |
| CHRO:INU | 0 | No concerns | Low risk | No concerns | Major concerns | No concerns | Major concerns | Low |
| CHRO:KOIL | 0 | No concerns | Low risk | No concerns | Major concerns | No concerns | Major concerns | Low |
| CHRO:LAV | 0 | No concerns | Low risk | No concerns | Major concerns | No concerns | Major concerns | Low |
| CHRO:LAV+IMI | 0 | No concerns | Low risk | No concerns | Major concerns | No concerns | Major concerns | Low |
| CHRO:LH57 | 0 | No concerns | Low risk | No concerns | Major concerns | No concerns | Major concerns | Low |
| CHRO:LI-160 | 0 | No concerns | Low risk | No concerns | Major concerns | No concerns | Major concerns | Low |
| CHRO:LMF | 0 | No concerns | Low risk | No concerns | Major concerns | No concerns | Major concerns | Low |
| CHRO:LP299v | 0 | No concerns | Low risk | No concerns | Major concerns | No concerns | Major concerns | Low |
| CHRO:LS | 0 | Some concerns | Low risk | No concerns | Major concerns | No concerns | Major concerns | Very Low |
| CHRO:MEL | 0 | No concerns | Low risk | No concerns | Major concerns | No concerns | Major concerns | Low |
| CHRO:MG | 0 | No concerns | Low risk | No concerns | Major concerns | No concerns | Major concerns | Low |
| CHRO:NUT | 0 | No concerns | Low risk | Some concerns | Major concerns | No concerns | Major concerns | Very Low |
| CHRO:OMB | 0 | No concerns | Low risk | No concerns | Major concerns | No concerns | Major concerns | Low |
| CHRO:OMEGA3 | 0 | No concerns | Low risk | No concerns | Major concerns | No concerns | Major concerns | Low |
| CHRO:PAR | 0 | No concerns | Low risk | No concerns | Major concerns | No concerns | Major concerns | Low |
| CHRO:PROB | 0 | No concerns | Low risk | No concerns | Major concerns | No concerns | Major concerns | Low |
| CHRO:PS128 | 0 | No concerns | Low risk | No concerns | Major concerns | No concerns | Major concerns | Low |
| CHRO:RHO | 0 | No concerns | Low risk | No concerns | No concerns | Major concerns | Major concerns | Low |
| CHRO:RHO+SER | 0 | No concerns | Low risk | No concerns | No concerns | No concerns | Major concerns | Low |
| CHRO:SAF | 0 | Some concerns | Low risk | No concerns | Major concerns | No concerns | Major concerns | Very Low |
| CHRO:SAFF | 0 | No concerns | Low risk | No concerns | Major concerns | No concerns | Major concerns | Low |
| CHRO:SAFF+SCU | 0 | No concerns | Low risk | No concerns | Major concerns | No concerns | Major concerns | Low |
| CHRO:SAMe | 0 | No concerns | Low risk | No concerns | Major concerns | No concerns | Major concerns | Low |
| CHRO:SAMe+HELP9 | 0 | No concerns | Low risk | No concerns | Major concerns | No concerns | Major concerns | Low |
| CHRO:SCU | 0 | No concerns | Low risk | No concerns | Major concerns | No concerns | Major concerns | Low |
| CHRO:SER | 0 | No concerns | Low risk | No concerns | Major concerns | No concerns | Major concerns | Low |
| CHRO:SJW | 0 | No concerns | Low risk | No concerns | Major concerns | No concerns | Major concerns | Low |
| CHRO:SRN | 0 | No concerns | Low risk | No concerns | Major concerns | No concerns | Major concerns | Low |
| CHRO:SZL | 0 | No concerns | Low risk | No concerns | Major concerns | No concerns | Major concerns | Low |
| CHRO:TEN | 0 | No concerns | Low risk | No concerns | No concerns | Major concerns | Major concerns | Low |
| CHRO:WS5570 | 0 | No concerns | Low risk | No concerns | Major concerns | No concerns | Major concerns | Low |
| CHRO:WS5572 | 0 | No concerns | Low risk | No concerns | Major concerns | No concerns | Major concerns | Low |
| CHRO:WS5573 | 0 | Some concerns | Low risk | No concerns | Major concerns | No concerns | Major concerns | Very Low |
| CHRO:ZE | 0 | No concerns | Low risk | No concerns | Major concerns | No concerns | Major concerns | Low |
| CHRO:ZE117 | 0 | No concerns | Low risk | No concerns | Major concerns | No concerns | Major concerns | Low |
| CHRO:vitB | 0 | No concerns | Low risk | No concerns | Some concerns | Some concerns | Major concerns | Low |
| CHRO:vitD | 0 | No concerns | Low risk | No concerns | Major concerns | No concerns | Major concerns | Low |
| CHRO:vitD3 | 0 | No concerns | Low risk | No concerns | Major concerns | No concerns | Major concerns | Low |
| CHRO:vitD3+CA | 0 | No concerns | Low risk | No concerns | Major concerns | No concerns | Major concerns | Low |
| CHRO:vitD3+FLUO | 0 | No concerns | Low risk | No concerns | Major concerns | No concerns | Major concerns | Low |
| CIT:CREA | 0 | No concerns | Low risk | No concerns | Major concerns | No concerns | Major concerns | Low |
| CIT:CTP | 0 | No concerns | Low risk | No concerns | Major concerns | No concerns | Major concerns | Low |
| CIT:CUR | 0 | No concerns | Low risk | No concerns | Major concerns | No concerns | Major concerns | Low |
| CIT:CUR+SAFF | 0 | No concerns | Low risk | No concerns | Major concerns | No concerns | Major concerns | Low |
| CIT:DHA | 0 | No concerns | Low risk | No concerns | Major concerns | No concerns | Major concerns | Low |
| CIT:EOPF | 0 | No concerns | Low risk | No concerns | Major concerns | No concerns | Major concerns | Low |
| CIT:EPA | 0 | No concerns | Low risk | No concerns | Major concerns | No concerns | Major concerns | Low |
| CIT:EPA+DHA | 0 | No concerns | Low risk | No concerns | Major concerns | No concerns | Major concerns | Low |
| CIT:ESC | 0 | Some concerns | Low risk | No concerns | Major concerns | No concerns | Major concerns | Very Low |
| CIT:ESC+FOL | 0 | No concerns | Low risk | No concerns | Major concerns | No concerns | Major concerns | Low |
| CIT:FLUO | 0 | No concerns | Low risk | No concerns | Major concerns | No concerns | Major concerns | Low |
| CIT:FOIL | 0 | No concerns | Low risk | No concerns | Major concerns | No concerns | Major concerns | Low |
| CIT:GBDP | 0 | No concerns | Low risk | No concerns | Major concerns | No concerns | Major concerns | Low |
| CIT:IMI | 0 | Some concerns | Low risk | No concerns | Major concerns | No concerns | Major concerns | Very Low |
| CIT:INO | 0 | No concerns | Low risk | No concerns | Major concerns | No concerns | Major concerns | Low |
| CIT:INU | 0 | No concerns | Low risk | No concerns | Major concerns | No concerns | Major concerns | Low |
| CIT:KOIL | 0 | No concerns | Low risk | No concerns | Major concerns | No concerns | Major concerns | Low |
| CIT:LAV | 0 | No concerns | Low risk | No concerns | Major concerns | No concerns | Major concerns | Low |
| CIT:LAV+IMI | 0 | No concerns | Low risk | No concerns | Major concerns | No concerns | Major concerns | Low |
| CIT:LH57 | 0 | No concerns | Low risk | No concerns | Major concerns | No concerns | Major concerns | Low |
| CIT:LI-160 | 0 | No concerns | Low risk | No concerns | Major concerns | No concerns | Major concerns | Low |
| CIT:LMF | 0 | Some concerns | Low risk | No concerns | Major concerns | No concerns | Major concerns | Very Low |
| CIT:LP299v | 0 | No concerns | Low risk | No concerns | Major concerns | No concerns | Major concerns | Low |
| CIT:LS | 0 | No concerns | Low risk | No concerns | Major concerns | No concerns | Major concerns | Low |
| CIT:MEL | 0 | No concerns | Low risk | No concerns | Major concerns | No concerns | Major concerns | Low |
| CIT:MG | 0 | No concerns | Low risk | No concerns | Major concerns | No concerns | Major concerns | Low |
| CIT:NUT | 0 | No concerns | Low risk | No concerns | Major concerns | No concerns | Major concerns | Low |
| CIT:OMB | 0 | No concerns | Low risk | No concerns | Major concerns | No concerns | Major concerns | Low |
| CIT:OMEGA3 | 0 | No concerns | Low risk | No concerns | Major concerns | No concerns | Major concerns | Low |
| CIT:PAR | 0 | No concerns | Low risk | No concerns | Major concerns | No concerns | Major concerns | Low |
| CIT:PROB | 0 | No concerns | Low risk | No concerns | Major concerns | No concerns | Major concerns | Low |
| CIT:PS128 | 0 | No concerns | Low risk | No concerns | Major concerns | No concerns | Major concerns | Low |
| CIT:RHO | 0 | No concerns | Low risk | No concerns | Major concerns | No concerns | Major concerns | Low |
| CIT:RHO+SER | 0 | No concerns | Low risk | No concerns | No concerns | No concerns | Major concerns | Low |
| CIT:SAF | 0 | No concerns | Low risk | No concerns | Major concerns | No concerns | Major concerns | Low |
| CIT:SAFF | 0 | No concerns | Low risk | No concerns | Major concerns | No concerns | Major concerns | Low |
| CIT:SAFF+SCU | 0 | No concerns | Low risk | Some concerns | Major concerns | No concerns | Major concerns | Very Low |
| CIT:SAMe | 0 | No concerns | Low risk | No concerns | Major concerns | No concerns | Major concerns | Low |
| CIT:SAMe+HELP9 | 0 | No concerns | Low risk | No concerns | Major concerns | No concerns | Major concerns | Low |
| CIT:SCU | 0 | Some concerns | Low risk | No concerns | Major concerns | No concerns | Major concerns | Very Low |
| CIT:SER | 0 | No concerns | Low risk | No concerns | Major concerns | No concerns | Major concerns | Low |
| CIT:SJW | 0 | No concerns | Low risk | No concerns | Major concerns | No concerns | Major concerns | Low |
| CIT:SRN | 0 | No concerns | Low risk | No concerns | Major concerns | No concerns | Major concerns | Low |
| CIT:SZL | 0 | No concerns | Low risk | No concerns | Major concerns | No concerns | Major concerns | Low |
| CIT:TEN | 0 | No concerns | Low risk | Some concerns | Major concerns | No concerns | Major concerns | Very Low |
| CIT:WS5570 | 0 | No concerns | Low risk | No concerns | Major concerns | No concerns | Major concerns | Low |
| CIT:WS5572 | 0 | No concerns | Low risk | No concerns | Major concerns | No concerns | Major concerns | Low |
| CIT:WS5573 | 0 | No concerns | Low risk | No concerns | Major concerns | No concerns | Major concerns | Low |
| CIT:ZE | 0 | No concerns | Low risk | No concerns | Major concerns | No concerns | Major concerns | Low |
| CIT:ZE117 | 0 | No concerns | Low risk | No concerns | Major concerns | No concerns | Major concerns | Low |
| CIT:vitB | 0 | No concerns | Low risk | No concerns | Major concerns | No concerns | Major concerns | Low |
| CIT:vitD | 0 | No concerns | Low risk | No concerns | Major concerns | No concerns | Major concerns | Low |
| CIT:vitD3 | 0 | No concerns | Low risk | No concerns | Major concerns | No concerns | Major concerns | Low |
| CIT:vitD3+CA | 0 | No concerns | Low risk | No concerns | Major concerns | No concerns | Major concerns | Low |
| CIT:vitD3+FLUO | 0 | No concerns | Low risk | No concerns | Major concerns | No concerns | Major concerns | Low |
| CREA:CTP | 0 | No concerns | Low risk | No concerns | Major concerns | No concerns | Major concerns | Low |
| CREA:CUR | 0 | Some concerns | Low risk | No concerns | Major concerns | No concerns | Major concerns | Very Low |
| CREA:CUR+SAFF | 0 | No concerns | Low risk | No concerns | Major concerns | No concerns | Major concerns | Low |
| CREA:DHA | 0 | No concerns | Low risk | No concerns | Major concerns | No concerns | Major concerns | Low |
| CREA:EOPF | 0 | No concerns | Low risk | No concerns | Major concerns | No concerns | Major concerns | Low |
| CREA:EPA | 0 | No concerns | Low risk | No concerns | Major concerns | No concerns | Major concerns | Low |
| CREA:EPA+DHA | 0 | No concerns | Low risk | No concerns | Major concerns | No concerns | Major concerns | Low |
| CREA:ESC | 0 | No concerns | Low risk | No concerns | Major concerns | No concerns | Major concerns | Low |
| CREA:ESC+FOL | 0 | No concerns | Low risk | No concerns | Major concerns | No concerns | Major concerns | Low |
| CREA:FLUO | 0 | No concerns | Low risk | No concerns | Major concerns | No concerns | Major concerns | Low |
| CREA:FOIL | 0 | No concerns | Low risk | No concerns | Major concerns | No concerns | Major concerns | Low |
| CREA:GBDP | 0 | No concerns | Low risk | No concerns | Major concerns | No concerns | Major concerns | Low |
| CREA:IMI | 0 | No concerns | Low risk | No concerns | Major concerns | No concerns | Major concerns | Low |
| CREA:INO | 0 | Some concerns | Low risk | No concerns | Major concerns | No concerns | Major concerns | Very Low |
| CREA:INU | 0 | No concerns | Low risk | No concerns | Major concerns | No concerns | Major concerns | Low |
| CREA:KOIL | 0 | No concerns | Low risk | No concerns | Major concerns | No concerns | Major concerns | Low |
| CREA:LAV | 0 | No concerns | Low risk | No concerns | Major concerns | No concerns | Major concerns | Low |
| CREA:LAV+IMI | 0 | No concerns | Low risk | No concerns | Major concerns | No concerns | Major concerns | Low |
| CREA:LH57 | 0 | No concerns | Low risk | No concerns | Major concerns | No concerns | Major concerns | Low |
| CREA:LI-160 | 0 | No concerns | Low risk | No concerns | Major concerns | No concerns | Major concerns | Low |
| CREA:LMF | 0 | No concerns | Low risk | No concerns | Major concerns | No concerns | Major concerns | Low |
| CREA:LP299v | 0 | No concerns | Low risk | No concerns | Major concerns | No concerns | Major concerns | Low |
| CREA:LS | 0 | No concerns | Low risk | No concerns | Major concerns | No concerns | Major concerns | Low |
| CREA:MEL | 0 | No concerns | Low risk | No concerns | Major concerns | No concerns | Major concerns | Low |
| CREA:MG | 0 | No concerns | Low risk | No concerns | Major concerns | No concerns | Major concerns | Low |
| CREA:NUT | 0 | No concerns | Low risk | No concerns | Major concerns | No concerns | Major concerns | Low |
| CREA:OMB | 0 | No concerns | Low risk | No concerns | Major concerns | No concerns | Major concerns | Low |
| CREA:OMEGA3 | 0 | No concerns | Low risk | No concerns | Major concerns | No concerns | Major concerns | Low |
| CREA:PAR | 0 | No concerns | Low risk | No concerns | Major concerns | No concerns | Major concerns | Low |
| CREA:PROB | 0 | No concerns | Low risk | No concerns | Major concerns | No concerns | Major concerns | Low |
| CREA:PS128 | 0 | No concerns | Low risk | No concerns | Major concerns | No concerns | Major concerns | Low |
| CREA:RHO | 0 | No concerns | Low risk | No concerns | No concerns | Major concerns | Major concerns | Low |
| CREA:RHO+SER | 0 | No concerns | Low risk | No concerns | No concerns | No concerns | Major concerns | Low |
| CREA:SAF | 0 | No concerns | Low risk | No concerns | Major concerns | No concerns | Major concerns | Low |
| CREA:SAFF | 0 | Some concerns | Low risk | No concerns | Major concerns | No concerns | Major concerns | Very Low |
| CREA:SAFF+SCU | 0 | No concerns | Low risk | No concerns | Major concerns | No concerns | Major concerns | Low |
| CREA:SAMe | 0 | No concerns | Low risk | No concerns | Major concerns | No concerns | Major concerns | Low |
| CREA:SAMe+HELP9 | 0 | No concerns | Low risk | No concerns | Major concerns | No concerns | Major concerns | Low |
| CREA:SCU | 0 | No concerns | Low risk | No concerns | Major concerns | No concerns | Major concerns | Low |
| CREA:SER | 0 | No concerns | Low risk | No concerns | Major concerns | No concerns | Major concerns | Low |
| CREA:SJW | 0 | No concerns | Low risk | No concerns | Major concerns | No concerns | Major concerns | Low |
| CREA:SRN | 0 | No concerns | Low risk | No concerns | Major concerns | No concerns | Major concerns | Low |
| CREA:SZL | 0 | No concerns | Low risk | No concerns | Major concerns | No concerns | Major concerns | Low |
| CREA:TEN | 0 | No concerns | Low risk | No concerns | Some concerns | Some concerns | Major concerns | Low |
| CREA:WS5570 | 0 | No concerns | Low risk | No concerns | Major concerns | No concerns | Major concerns | Low |
| CREA:WS5572 | 0 | No concerns | Low risk | No concerns | Major concerns | No concerns | Major concerns | Low |
| CREA:WS5573 | 0 | No concerns | Low risk | No concerns | Major concerns | No concerns | Major concerns | Low |
| CREA:ZE | 0 | No concerns | Low risk | No concerns | Major concerns | No concerns | Major concerns | Low |
| CREA:ZE117 | 0 | No concerns | Low risk | No concerns | Major concerns | No concerns | Major concerns | Low |
| CREA:vitB | 0 | No concerns | Low risk | No concerns | Major concerns | No concerns | Major concerns | Low |
| CREA:vitD | 0 | No concerns | Low risk | Some concerns | Major concerns | No concerns | Major concerns | Very Low |
| CREA:vitD3 | 0 | No concerns | Low risk | No concerns | Major concerns | No concerns | Major concerns | Low |
| CREA:vitD3+CA | 0 | No concerns | Low risk | No concerns | Major concerns | No concerns | Major concerns | Low |
| CREA:vitD3+FLUO | 0 | No concerns | Low risk | No concerns | Major concerns | No concerns | Major concerns | Low |
| CTP:CUR | 0 | No concerns | Low risk | No concerns | Major concerns | No concerns | Major concerns | Low |
| CTP:CUR+SAFF | 0 | No concerns | Low risk | No concerns | Major concerns | No concerns | Major concerns | Low |
| CTP:DHA | 0 | No concerns | Low risk | No concerns | Some concerns | Some concerns | Major concerns | Low |
| CTP:EOPF | 0 | No concerns | Low risk | No concerns | Major concerns | No concerns | Major concerns | Low |
| CTP:EPA | 0 | No concerns | Low risk | No concerns | Major concerns | No concerns | Major concerns | Low |
| CTP:EPA+DHA | 0 | No concerns | Low risk | No concerns | Major concerns | No concerns | Major concerns | Low |
| CTP:ESC | 0 | No concerns | Low risk | No concerns | Major concerns | No concerns | Major concerns | Low |
| CTP:ESC+FOL | 0 | No concerns | Low risk | No concerns | Major concerns | No concerns | Major concerns | Low |
| CTP:FLUO | 0 | No concerns | Low risk | No concerns | Major concerns | No concerns | Major concerns | Low |
| CTP:FOIL | 0 | No concerns | Low risk | No concerns | Major concerns | No concerns | Major concerns | Low |
| CTP:GBDP | 0 | No concerns | Low risk | No concerns | Major concerns | No concerns | Major concerns | Low |
| CTP:IMI | 0 | No concerns | Low risk | No concerns | Major concerns | No concerns | Major concerns | Low |
| CTP:INO | 0 | No concerns | Low risk | No concerns | Major concerns | No concerns | Major concerns | Low |
| CTP:INU | 0 | No concerns | Low risk | No concerns | Major concerns | No concerns | Major concerns | Low |
| CTP:KOIL | 0 | Some concerns | Low risk | No concerns | Major concerns | No concerns | Major concerns | Very Low |
| CTP:LAV | 0 | No concerns | Low risk | No concerns | Major concerns | No concerns | Major concerns | Low |
| CTP:LAV+IMI | 0 | No concerns | Low risk | No concerns | Major concerns | No concerns | Major concerns | Low |
| CTP:LH57 | 0 | No concerns | Low risk | No concerns | Major concerns | No concerns | Major concerns | Low |
| CTP:LI-160 | 0 | No concerns | Low risk | No concerns | Major concerns | No concerns | Major concerns | Low |
| CTP:LMF | 0 | No concerns | Low risk | No concerns | Major concerns | No concerns | Major concerns | Low |
| CTP:LP299v | 0 | No concerns | Low risk | No concerns | Major concerns | No concerns | Major concerns | Low |
| CTP:LS | 0 | No concerns | Low risk | No concerns | Major concerns | No concerns | Major concerns | Low |
| CTP:MEL | 0 | Some concerns | Low risk | No concerns | Major concerns | No concerns | Major concerns | Very Low |
| CTP:MG | 0 | No concerns | Low risk | No concerns | Major concerns | No concerns | Major concerns | Low |
| CTP:NUT | 0 | No concerns | Low risk | No concerns | Major concerns | No concerns | Major concerns | Low |
| CTP:OMB | 0 | No concerns | Low risk | No concerns | Major concerns | No concerns | Major concerns | Low |
| CTP:OMEGA3 | 0 | No concerns | Low risk | No concerns | Major concerns | No concerns | Major concerns | Low |
| CTP:PAR | 0 | No concerns | Low risk | No concerns | Major concerns | No concerns | Major concerns | Low |
| CTP:PROB | 0 | Some concerns | Low risk | No concerns | Major concerns | No concerns | Major concerns | Very Low |
| CTP:PLB | 0 | No concerns | Low risk | No concerns | Major concerns | No concerns | Major concerns | Low |
| CTP:PS128 | 0 | No concerns | Low risk | No concerns | Major concerns | No concerns | Major concerns | Low |
| CTP:RHO | 0 | No concerns | Low risk | No concerns | Major concerns | No concerns | Major concerns | Low |
| CTP:RHO+SER | 0 | No concerns | Low risk | No concerns | No concerns | No concerns | Major concerns | Low |
| CTP:SAF | 0 | No concerns | Low risk | No concerns | Major concerns | No concerns | Major concerns | Low |
| CTP:SAFF+SCU | 0 | No concerns | Low risk | No concerns | Major concerns | No concerns | Major concerns | Low |
| CTP:SAMe | 0 | No concerns | Low risk | No concerns | Major concerns | No concerns | Major concerns | Low |
| CTP:SAMe+HELP9 | 0 | No concerns | Low risk | No concerns | Major concerns | No concerns | Major concerns | Low |
| CTP:SCU | 0 | No concerns | Low risk | No concerns | Major concerns | No concerns | Major concerns | Low |
| CTP:SER | 0 | No concerns | Low risk | No concerns | Major concerns | No concerns | Major concerns | Low |
| CTP:SJW | 0 | No concerns | Low risk | No concerns | Major concerns | No concerns | Major concerns | Low |
| CTP:SRN | 0 | No concerns | Low risk | No concerns | Major concerns | No concerns | Major concerns | Low |
| CTP:SZL | 0 | No concerns | Low risk | No concerns | Major concerns | No concerns | Major concerns | Low |
| CTP:TEN | 0 | No concerns | Low risk | No concerns | Major concerns | No concerns | Major concerns | Low |
| CTP:WS5570 | 0 | No concerns | Low risk | No concerns | Major concerns | No concerns | Major concerns | Low |
| CTP:WS5572 | 0 | No concerns | Low risk | No concerns | Major concerns | No concerns | Major concerns | Low |
| CTP:WS5573 | 0 | Some concerns | Low risk | Some concerns | Major concerns | No concerns | Major concerns | Very Low |
| CTP:ZE | 0 | No concerns | Low risk | No concerns | Major concerns | No concerns | Major concerns | Low |
| CTP:ZE117 | 0 | No concerns | Low risk | No concerns | Major concerns | No concerns | Major concerns | Low |
| CTP:vitB | 0 | No concerns | Low risk | No concerns | Major concerns | No concerns | Major concerns | Low |
| CTP:vitD | 0 | No concerns | Low risk | No concerns | Major concerns | No concerns | Major concerns | Low |
| CTP:vitD3 | 0 | No concerns | Low risk | No concerns | Major concerns | No concerns | Major concerns | Low |
| CTP:vitD3+CA | 0 | Some concerns | Low risk | No concerns | Major concerns | No concerns | Major concerns | Very Low |
| CTP:vitD3+FLUO | 0 | No concerns | Low risk | No concerns | Major concerns | No concerns | Major concerns | Low |
| CUR:DHA | 0 | No concerns | Low risk | No concerns | Major concerns | No concerns | Major concerns | Low |
| CUR:EOPF | 0 | No concerns | Low risk | No concerns | Major concerns | No concerns | Major concerns | Low |
| CUR:EPA | 0 | No concerns | Low risk | No concerns | No concerns | Major concerns | Major concerns | Low |
| CUR:EPA+DHA | 0 | No concerns | Low risk | No concerns | Major concerns | No concerns | Major concerns | Low |
| CUR:ESC | 0 | No concerns | Low risk | No concerns | Major concerns | No concerns | Major concerns | Low |
| CUR:ESC+FOL | 0 | No concerns | Low risk | No concerns | Major concerns | No concerns | Major concerns | Low |
| CUR:FLUO | 0 | No concerns | Low risk | No concerns | Major concerns | No concerns | Major concerns | Low |
| CUR:FOIL | 0 | No concerns | Low risk | No concerns | Major concerns | No concerns | Major concerns | Low |
| CUR:GBDP | 0 | No concerns | Low risk | No concerns | Major concerns | No concerns | Major concerns | Low |
| CUR:IMI | 0 | No concerns | Low risk | No concerns | Major concerns | No concerns | Major concerns | Low |
| CUR:INO | 0 | No concerns | Low risk | No concerns | Major concerns | No concerns | Major concerns | Low |
| CUR:INU | 0 | No concerns | Low risk | No concerns | Major concerns | No concerns | Major concerns | Low |
| CUR:KOIL | 0 | No concerns | Low risk | No concerns | Major concerns | No concerns | Major concerns | Low |
| CUR:LAV | 0 | Some concerns | Low risk | No concerns | Major concerns | No concerns | Major concerns | Very Low |
| CUR:LAV+IMI | 0 | No concerns | Low risk | No concerns | Major concerns | No concerns | Major concerns | Low |
| CUR:LH57 | 0 | No concerns | Low risk | No concerns | Major concerns | No concerns | Major concerns | Low |
| CUR:LI-160 | 0 | No concerns | Low risk | No concerns | Major concerns | No concerns | Major concerns | Low |
| CUR:LMF | 0 | Some concerns | Low risk | No concerns | Major concerns | No concerns | Major concerns | Very Low |
| CUR:LP299v | 0 | No concerns | Low risk | No concerns | Major concerns | No concerns | Major concerns | Low |
| CUR:LS | 0 | No concerns | Low risk | No concerns | Major concerns | No concerns | Major concerns | Low |
| CUR:MEL | 0 | No concerns | Low risk | No concerns | Major concerns | No concerns | Major concerns | Low |
| CUR:MG | 0 | No concerns | Low risk | No concerns | Major concerns | No concerns | Major concerns | Low |
| CUR:NUT | 0 | No concerns | Low risk | No concerns | Major concerns | No concerns | Major concerns | Low |
| CUR:OMB | 0 | No concerns | Low risk | No concerns | Major concerns | No concerns | Major concerns | Low |
| CUR:OMEGA3 | 0 | No concerns | Low risk | No concerns | Major concerns | No concerns | Major concerns | Low |
| CUR:PAR | 0 | No concerns | Low risk | No concerns | Major concerns | No concerns | Major concerns | Low |
| CUR:PROB | 0 | No concerns | Low risk | No concerns | Major concerns | No concerns | Major concerns | Low |
| CUR:PS128 | 0 | No concerns | Low risk | No concerns | Major concerns | No concerns | Major concerns | Low |
| CUR:RHO | 0 | No concerns | Low risk | No concerns | No concerns | Some concerns | Major concerns | Low |
| CUR:RHO+SER | 0 | No concerns | Low risk | No concerns | No concerns | No concerns | Major concerns | Low |
| CUR:SAF | 0 | No concerns | Low risk | No concerns | Major concerns | No concerns | Major concerns | Low |
| CUR:SAFF | 0 | No concerns | Low risk | No concerns | Major concerns | No concerns | Major concerns | Low |
| CUR:SAFF+SCU | 0 | No concerns | Low risk | No concerns | Major concerns | No concerns | Major concerns | Low |
| CUR:SAMe | 0 | No concerns | Low risk | No concerns | Major concerns | No concerns | Major concerns | Low |
| CUR:SAMe+HELP9 | 0 | No concerns | Low risk | No concerns | Major concerns | No concerns | Major concerns | Low |
| CUR:SCU | 0 | No concerns | Low risk | No concerns | Major concerns | No concerns | Major concerns | Low |
| CUR:SER | 0 | No concerns | Low risk | No concerns | Major concerns | No concerns | Major concerns | Low |
| CUR:SJW | 0 | No concerns | Low risk | No concerns | Major concerns | No concerns | Major concerns | Low |
| CUR:SRN | 0 | No concerns | Low risk | No concerns | Major concerns | No concerns | Major concerns | Low |
| CUR:SZL | 0 | No concerns | Low risk | No concerns | Major concerns | No concerns | Major concerns | Low |
| CUR:TEN | 0 | No concerns | Low risk | Some concerns | No concerns | Major concerns | Major concerns | Very Low |
| CUR:WS5570 | 0 | No concerns | Low risk | No concerns | Major concerns | No concerns | Major concerns | Low |
| CUR:WS5572 | 0 | No concerns | Low risk | No concerns | Major concerns | No concerns | Major concerns | Low |
| CUR:WS5573 | 0 | No concerns | Low risk | No concerns | Major concerns | No concerns | Major concerns | Low |
| CUR:ZE | 0 | No concerns | Low risk | No concerns | Major concerns | No concerns | Major concerns | Low |
| CUR:ZE117 | 0 | No concerns | Low risk | No concerns | Major concerns | No concerns | Major concerns | Low |
| CUR:vitB | 0 | No concerns | Low risk | No concerns | Some concerns | Some concerns | Major concerns | Low |
| CUR:vitD | 0 | No concerns | Low risk | No concerns | Major concerns | No concerns | Major concerns | Low |
| CUR:vitD3 | 0 | No concerns | Low risk | No concerns | Some concerns | Some concerns | Major concerns | Low |
| CUR:vitD3+CA | 0 | No concerns | Low risk | No concerns | Major concerns | No concerns | Major concerns | Low |
| CUR:vitD3+FLUO | 0 | No concerns | Low risk | No concerns | Major concerns | No concerns | Major concerns | Low |
| CUR+SAFF:DHA | 0 | No concerns | Low risk | No concerns | Major concerns | No concerns | Major concerns | Low |
| CUR+SAFF:EOPF | 0 | No concerns | Low risk | No concerns | Major concerns | No concerns | Major concerns | Low |
| CUR+SAFF:EPA | 0 | No concerns | Low risk | No concerns | Some concerns | Some concerns | Major concerns | Low |
| CUR+SAFF:EPA+DHA | 0 | No concerns | Low risk | No concerns | Major concerns | No concerns | Major concerns | Low |
| CUR+SAFF:ESC | 0 | No concerns | Low risk | No concerns | Major concerns | No concerns | Major concerns | Low |
| CUR+SAFF:ESC+FOL | 0 | No concerns | Low risk | No concerns | Major concerns | No concerns | Major concerns | Low |
| CUR+SAFF:FLUO | 0 | No concerns | Low risk | No concerns | Major concerns | No concerns | Major concerns | Low |
| CUR+SAFF:FOIL | 0 | No concerns | Low risk | No concerns | Major concerns | No concerns | Major concerns | Low |
| CUR+SAFF:GBDP | 0 | Some concerns | Low risk | No concerns | Major concerns | No concerns | Major concerns | Very Low |
| CUR+SAFF:IMI | 0 | No concerns | Low risk | No concerns | Major concerns | No concerns | Major concerns | Low |
| CUR+SAFF:INO | 0 | No concerns | Low risk | No concerns | Major concerns | No concerns | Major concerns | Low |
| CUR+SAFF:INU | 0 | No concerns | Low risk | No concerns | Major concerns | No concerns | Major concerns | Low |
| CUR+SAFF:KOIL | 0 | No concerns | Low risk | No concerns | Major concerns | No concerns | Major concerns | Low |
| CUR+SAFF:LAV | 0 | No concerns | Low risk | No concerns | Major concerns | No concerns | Major concerns | Low |
| CUR+SAFF:LAV+IMI | 0 | No concerns | Low risk | No concerns | Major concerns | No concerns | Major concerns | Low |
| CUR+SAFF:LH57 | 0 | No concerns | Low risk | No concerns | Major concerns | No concerns | Major concerns | Low |
| CUR+SAFF:LI-160 | 0 | No concerns | Low risk | No concerns | Major concerns | No concerns | Major concerns | Low |
| CUR+SAFF:LMF | 0 | No concerns | Low risk | No concerns | Major concerns | No concerns | Major concerns | Low |
| CUR+SAFF:LP299v | 0 | No concerns | Low risk | No concerns | Major concerns | No concerns | Major concerns | Low |
| CUR+SAFF:LS | 0 | No concerns | Low risk | No concerns | Major concerns | No concerns | Major concerns | Low |
| CUR+SAFF:MEL | 0 | No concerns | Low risk | No concerns | Major concerns | No concerns | Major concerns | Low |
| CUR+SAFF:MG | 0 | No concerns | Low risk | No concerns | Major concerns | No concerns | Major concerns | Low |
| CUR+SAFF:NUT | 0 | Some concerns | Low risk | No concerns | Major concerns | No concerns | Major concerns | Very Low |
| CUR+SAFF:OMB | 0 | No concerns | Low risk | No concerns | Major concerns | No concerns | Major concerns | Low |
| CUR+SAFF:OMEGA3 | 0 | No concerns | Low risk | No concerns | Major concerns | No concerns | Major concerns | Low |
| CUR+SAFF:PAR | 0 | No concerns | Low risk | No concerns | Major concerns | No concerns | Major concerns | Low |
| CUR+SAFF:PROB | 0 | No concerns | Low risk | No concerns | Major concerns | No concerns | Major concerns | Low |
| CUR+SAFF:PS128 | 0 | No concerns | Low risk | No concerns | Major concerns | No concerns | Major concerns | Low |
| CUR+SAFF:RHO | 0 | No concerns | Low risk | No concerns | No concerns | Major concerns | Major concerns | Low |
| CUR+SAFF:RHO+SER | 0 | No concerns | Low risk | No concerns | No concerns | No concerns | Major concerns | Low |
| CUR+SAFF:SAF | 0 | Some concerns | Low risk | No concerns | Major concerns | No concerns | Major concerns | Very Low |
| CUR+SAFF:SAFF | 0 | No concerns | Low risk | No concerns | Major concerns | No concerns | Major concerns | Low |
| CUR+SAFF:SAFF+SCU | 0 | No concerns | Low risk | No concerns | Major concerns | No concerns | Major concerns | Low |
| CUR+SAFF:SAMe | 0 | No concerns | Low risk | No concerns | Major concerns | No concerns | Major concerns | Low |
| CUR+SAFF:SAMe+HELP9 | 0 | No concerns | Low risk | No concerns | Major concerns | No concerns | Major concerns | Low |
| CUR+SAFF:SCU | 0 | No concerns | Low risk | No concerns | Major concerns | No concerns | Major concerns | Low |
| CUR+SAFF:SER | 0 | No concerns | Low risk | No concerns | Major concerns | No concerns | Major concerns | Low |
| CUR+SAFF:SJW | 0 | No concerns | Low risk | No concerns | Major concerns | No concerns | Major concerns | Low |
| CUR+SAFF:SRN | 0 | No concerns | Low risk | No concerns | Major concerns | No concerns | Major concerns | Low |
| CUR+SAFF:SZL | 0 | No concerns | Low risk | No concerns | Major concerns | No concerns | Major concerns | Low |
| CUR+SAFF:TEN | 0 | No concerns | Low risk | No concerns | No concerns | Major concerns | Major concerns | Low |
| CUR+SAFF:WS5570 | 0 | No concerns | Low risk | No concerns | Major concerns | No concerns | Major concerns | Low |
| CUR+SAFF:WS5572 | 0 | No concerns | Low risk | No concerns | Major concerns | No concerns | Major concerns | Low |
| CUR+SAFF:WS5573 | 0 | No concerns | Low risk | No concerns | Major concerns | No concerns | Major concerns | Low |
| CUR+SAFF:ZE | 0 | No concerns | Low risk | No concerns | Major concerns | No concerns | Major concerns | Low |
| CUR+SAFF:ZE117 | 0 | No concerns | Low risk | No concerns | Major concerns | No concerns | Major concerns | Low |
| CUR+SAFF:vitB | 0 | No concerns | Low risk | No concerns | Major concerns | No concerns | Major concerns | Low |
| CUR+SAFF:vitD | 0 | No concerns | Low risk | No concerns | Major concerns | No concerns | Major concerns | Low |
| CUR+SAFF:vitD3 | 0 | No concerns | Low risk | No concerns | Major concerns | No concerns | Major concerns | Low |
| CUR+SAFF:vitD3+CA | 0 | No concerns | Low risk | No concerns | Major concerns | No concerns | Major concerns | Low |
| CUR+SAFF:vitD3+FLUO | 0 | No concerns | Low risk | No concerns | Major concerns | No concerns | Major concerns | Low |
| DHA:EOPF | 0 | No concerns | Low risk | No concerns | Some concerns | Some concerns | Major concerns | Low |
| DHA:EPA+DHA | 0 | No concerns | Low risk | No concerns | Major concerns | No concerns | Major concerns | Low |
| DHA:ESC | 0 | Some concerns | Low risk | No concerns | Major concerns | No concerns | Major concerns | Very Low |
| DHA:ESC+FOL | 0 | No concerns | Low risk | No concerns | Major concerns | No concerns | Major concerns | Low |
| DHA:FLUO | 0 | No concerns | Low risk | No concerns | Major concerns | No concerns | Major concerns | Low |
| DHA:FOIL | 0 | No concerns | Low risk | No concerns | Major concerns | No concerns | Major concerns | Low |
| DHA:GBDP | 0 | No concerns | Low risk | No concerns | Major concerns | No concerns | Major concerns | Low |
| DHA:IMI | 0 | No concerns | Low risk | No concerns | Major concerns | No concerns | Major concerns | Low |
| DHA:INO | 0 | No concerns | Low risk | No concerns | Major concerns | No concerns | Major concerns | Low |
| DHA:INU | 0 | No concerns | Low risk | No concerns | Major concerns | No concerns | Major concerns | Low |
| DHA:KOIL | 0 | No concerns | Low risk | No concerns | Major concerns | No concerns | Major concerns | Low |
| DHA:LAV | 0 | No concerns | Low risk | No concerns | Some concerns | Some concerns | Major concerns | Low |
| DHA:LAV+IMI | 0 | No concerns | Low risk | Some concerns | No concerns | Major concerns | Major concerns | Very Low |
| DHA:LH57 | 0 | No concerns | Low risk | No concerns | Major concerns | No concerns | Major concerns | Low |
| DHA:LI-160 | 0 | No concerns | Low risk | No concerns | Major concerns | No concerns | Major concerns | Low |
| DHA:LMF | 0 | No concerns | Low risk | No concerns | Major concerns | No concerns | Major concerns | Low |
| DHA:LP299v | 0 | No concerns | Low risk | No concerns | Major concerns | No concerns | Major concerns | Low |
| DHA:LS | 0 | No concerns | Low risk | No concerns | Major concerns | No concerns | Major concerns | Low |
| DHA:MEL | 0 | No concerns | Low risk | No concerns | Major concerns | No concerns | Major concerns | Low |
| DHA:MG | 0 | Some concerns | Low risk | No concerns | Major concerns | No concerns | Major concerns | Very Low |
| DHA:NUT | 0 | No concerns | Low risk | No concerns | Major concerns | No concerns | Major concerns | Low |
| DHA:OMB | 0 | No concerns | Low risk | No concerns | Major concerns | No concerns | Major concerns | Low |
| DHA:OMEGA3 | 0 | No concerns | Low risk | No concerns | Major concerns | No concerns | Major concerns | Low |
| DHA:PAR | 0 | No concerns | Low risk | No concerns | Major concerns | No concerns | Major concerns | Low |
| DHA:PROB | 0 | No concerns | Low risk | No concerns | Major concerns | No concerns | Major concerns | Low |
| DHA:PS128 | 0 | No concerns | Low risk | No concerns | Major concerns | No concerns | Major concerns | Low |
| DHA:RHO | 0 | No concerns | Low risk | No concerns | No concerns | No concerns | Major concerns | Low |
| DHA:RHO+SER | 0 | No concerns | Low risk | No concerns | No concerns | No concerns | Major concerns | Low |
| DHA:SAF | 0 | No concerns | Low risk | No concerns | Major concerns | No concerns | Major concerns | Low |
| DHA:SAFF | 0 | No concerns | Low risk | No concerns | No concerns | Major concerns | Major concerns | Low |
| DHA:SAFF+SCU | 0 | No concerns | Low risk | No concerns | Major concerns | No concerns | Major concerns | Low |
| DHA:SAMe | 0 | No concerns | Low risk | No concerns | Some concerns | Some concerns | Major concerns | Low |
| DHA:SAMe+HELP9 | 0 | No concerns | Low risk | No concerns | Major concerns | No concerns | Major concerns | Low |
| DHA:SCU | 0 | Some concerns | Low risk | No concerns | Major concerns | No concerns | Major concerns | Very Low |
| DHA:SER | 0 | No concerns | Low risk | No concerns | Some concerns | Some concerns | Major concerns | Low |
| DHA:SJW | 0 | No concerns | Low risk | No concerns | Major concerns | No concerns | Major concerns | Low |
| DHA:SRN | 0 | No concerns | Low risk | No concerns | Major concerns | No concerns | Major concerns | Low |
| DHA:SZL | 0 | No concerns | Low risk | No concerns | Major concerns | No concerns | Major concerns | Low |
| DHA:TEN | 0 | No concerns | Low risk | No concerns | No concerns | No concerns | Major concerns | Low |
| DHA:WS5570 | 0 | No concerns | Low risk | No concerns | Major concerns | No concerns | Major concerns | Low |
| DHA:WS5572 | 0 | No concerns | Low risk | No concerns | Major concerns | No concerns | Major concerns | Low |
| DHA:WS5573 | 0 | No concerns | Low risk | No concerns | Major concerns | No concerns | Major concerns | Low |
| DHA:ZE | 0 | No concerns | Low risk | No concerns | Major concerns | No concerns | Major concerns | Low |
| DHA:ZE117 | 0 | No concerns | Low risk | No concerns | Some concerns | Some concerns | Major concerns | Low |
| DHA:vitB | 0 | No concerns | Low risk | No concerns | No concerns | Some concerns | Major concerns | Low |
| DHA:vitD | 0 | No concerns | Low risk | No concerns | Major concerns | No concerns | Major concerns | Low |
| DHA:vitD3 | 0 | No concerns | Low risk | No concerns | No concerns | Some concerns | Major concerns | Low |
| DHA:vitD3+CA | 0 | No concerns | Low risk | No concerns | Major concerns | No concerns | Major concerns | Low |
| DHA:vitD3+FLUO | 0 | No concerns | Low risk | Some concerns | Major concerns | No concerns | Major concerns | Very Low |
| EOPF:EPA | 0 | No concerns | Low risk | No concerns | Major concerns | No concerns | Major concerns | Low |
| EOPF:EPA+DHA | 0 | No concerns | Low risk | No concerns | Major concerns | No concerns | Major concerns | Low |
| EOPF:ESC | 0 | No concerns | Low risk | No concerns | Major concerns | No concerns | Major concerns | Low |
| EOPF:ESC+FOL | 0 | No concerns | Low risk | No concerns | Major concerns | No concerns | Major concerns | Low |
| EOPF:FLUO | 0 | No concerns | Low risk | No concerns | Major concerns | No concerns | Major concerns | Low |
| EOPF:FOIL | 0 | No concerns | Low risk | No concerns | Major concerns | No concerns | Major concerns | Low |
| EOPF:GBDP | 0 | No concerns | Low risk | No concerns | Major concerns | No concerns | Major concerns | Low |
| EOPF:IMI | 0 | No concerns | Low risk | No concerns | Major concerns | No concerns | Major concerns | Low |
| EOPF:INO | 0 | No concerns | Low risk | No concerns | Major concerns | No concerns | Major concerns | Low |
| EOPF:INU | 0 | No concerns | Low risk | No concerns | Major concerns | No concerns | Major concerns | Low |
| EOPF:KOIL | 0 | No concerns | Low risk | No concerns | Major concerns | No concerns | Major concerns | Low |
| EOPF:LAV | 0 | No concerns | Low risk | No concerns | Major concerns | No concerns | Major concerns | Low |
| EOPF:LAV+IMI | 0 | No concerns | Low risk | No concerns | Major concerns | No concerns | Major concerns | Low |
| EOPF:LH57 | 0 | Some concerns | Low risk | No concerns | Major concerns | No concerns | Major concerns | Very Low |
| EOPF:LI-160 | 0 | No concerns | Low risk | No concerns | Major concerns | No concerns | Major concerns | Low |
| EOPF:LMF | 0 | No concerns | Low risk | No concerns | Major concerns | No concerns | Major concerns | Low |
| EOPF:LP299v | 0 | No concerns | Low risk | No concerns | Major concerns | No concerns | Major concerns | Low |
| EOPF:LS | 0 | No concerns | Low risk | No concerns | Major concerns | No concerns | Major concerns | Low |
| EOPF:MEL | 0 | No concerns | Low risk | No concerns | Major concerns | No concerns | Major concerns | Low |
| EOPF:MG | 0 | No concerns | Low risk | No concerns | Major concerns | No concerns | Major concerns | Low |
| EOPF:NUT | 0 | No concerns | Low risk | No concerns | Major concerns | No concerns | Major concerns | Low |
| EOPF:OMB | 0 | No concerns | Low risk | No concerns | Major concerns | No concerns | Major concerns | Low |
| EOPF:OMEGA3 | 0 | No concerns | Low risk | No concerns | Major concerns | No concerns | Major concerns | Low |
| EOPF:PAR | 0 | No concerns | Low risk | No concerns | Major concerns | No concerns | Major concerns | Low |
| EOPF:PROB | 0 | No concerns | Low risk | No concerns | Major concerns | No concerns | Major concerns | Low |
| EOPF:PS128 | 0 | No concerns | Low risk | No concerns | Major concerns | No concerns | Major concerns | Low |
| EOPF:RHO | 0 | No concerns | Low risk | No concerns | Major concerns | No concerns | Major concerns | Low |
| EOPF:RHO+SER | 0 | No concerns | Low risk | No concerns | No concerns | No concerns | Major concerns | Low |
| EOPF:SAF | 0 | No concerns | Low risk | No concerns | Major concerns | No concerns | Major concerns | Low |
| EOPF:SAFF | 0 | No concerns | Low risk | No concerns | Major concerns | No concerns | Major concerns | Low |
| EOPF:SAFF+SCU | 0 | No concerns | Low risk | No concerns | Major concerns | No concerns | Major concerns | Low |
| EOPF:SAMe | 0 | No concerns | Low risk | No concerns | Major concerns | No concerns | Major concerns | Low |
| EOPF:SAMe+HELP9 | 0 | No concerns | Low risk | No concerns | Some concerns | Some concerns | Major concerns | Low |
| EOPF:SCU | 0 | No concerns | Low risk | No concerns | Major concerns | No concerns | Major concerns | Low |
| EOPF:SER | 0 | No concerns | Low risk | No concerns | Major concerns | No concerns | Major concerns | Low |
| EOPF:SJW | 0 | No concerns | Low risk | No concerns | Major concerns | No concerns | Major concerns | Low |
| EOPF:SRN | 0 | No concerns | Low risk | No concerns | Major concerns | No concerns | Major concerns | Low |
| EOPF:SZL | 0 | No concerns | Low risk | No concerns | Major concerns | No concerns | Major concerns | Low |
| EOPF:TEN | 0 | No concerns | Low risk | No concerns | Major concerns | No concerns | Major concerns | Low |
| EOPF:WS5570 | 0 | No concerns | Low risk | No concerns | Major concerns | No concerns | Major concerns | Low |
| EOPF:WS5572 | 0 | No concerns | Low risk | No concerns | Major concerns | No concerns | Major concerns | Low |
| EOPF:WS5573 | 0 | No concerns | Low risk | No concerns | Major concerns | No concerns | Major concerns | Low |
| EOPF:ZE | 0 | No concerns | Low risk | No concerns | Major concerns | No concerns | Major concerns | Low |
| EOPF:ZE117 | 0 | No concerns | Low risk | No concerns | Major concerns | No concerns | Major concerns | Low |
| EOPF:vitB | 0 | No concerns | Low risk | No concerns | Major concerns | No concerns | Major concerns | Low |
| EOPF:vitD | 0 | No concerns | Low risk | No concerns | Major concerns | No concerns | Major concerns | Low |
| EOPF:vitD3 | 0 | No concerns | Low risk | No concerns | Major concerns | No concerns | Major concerns | Low |
| EOPF:vitD3+CA | 0 | No concerns | Low risk | No concerns | Major concerns | No concerns | Major concerns | Low |
| EOPF:vitD3+FLUO | 0 | No concerns | Low risk | No concerns | Major concerns | No concerns | Major concerns | Low |
| EPA:EPA+DHA | 0 | No concerns | Low risk | No concerns | No concerns | Major concerns | Major concerns | Low |
| EPA:ESC | 0 | No concerns | Low risk | No concerns | Major concerns | No concerns | Major concerns | Low |
| EPA:ESC+FOL | 0 | No concerns | Low risk | No concerns | Major concerns | No concerns | Major concerns | Low |
| EPA:FLUO | 0 | No concerns | Low risk | No concerns | No concerns | Some concerns | Major concerns | Low |
| EPA:FOIL | 0 | No concerns | Low risk | No concerns | Some concerns | Some concerns | Major concerns | Low |
| EPA:GBDP | 0 | No concerns | Low risk | No concerns | Some concerns | Some concerns | Major concerns | Low |
| EPA:IMI | 0 | No concerns | Low risk | No concerns | Major concerns | No concerns | Major concerns | Low |
| EPA:INO | 0 | No concerns | Low risk | No concerns | Major concerns | No concerns | Major concerns | Low |
| EPA:INU | 0 | No concerns | Low risk | No concerns | Some concerns | Some concerns | Major concerns | Low |
| EPA:KOIL | 0 | No concerns | Low risk | No concerns | No concerns | Major concerns | Major concerns | Low |
| EPA:LAV | 0 | No concerns | Low risk | No concerns | Major concerns | No concerns | Major concerns | Low |
| EPA:LAV+IMI | 0 | No concerns | Low risk | No concerns | Major concerns | No concerns | Major concerns | Low |
| EPA:LH57 | 0 | No concerns | Low risk | No concerns | Some concerns | Some concerns | Major concerns | Low |
| EPA:LI-160 | 0 | No concerns | Low risk | No concerns | No concerns | Major concerns | Major concerns | Low |
| EPA:LMF | 0 | No concerns | Low risk | No concerns | No concerns | Major concerns | Major concerns | Low |
| EPA:LP299v | 0 | No concerns | Low risk | No concerns | Major concerns | No concerns | Major concerns | Low |
| EPA:LS | 0 | No concerns | Low risk | No concerns | Major concerns | No concerns | Major concerns | Low |
| EPA:MEL | 0 | No concerns | Low risk | No concerns | Major concerns | No concerns | Major concerns | Low |
| EPA:MG | 0 | No concerns | Low risk | No concerns | Major concerns | No concerns | Major concerns | Low |
| EPA:NUT | 0 | No concerns | Low risk | No concerns | No concerns | Major concerns | Major concerns | Low |
| EPA:OMB | 0 | No concerns | Low risk | No concerns | Some concerns | Some concerns | Major concerns | Low |
| EPA:OMEGA3 | 0 | No concerns | Low risk | No concerns | No concerns | Some concerns | Major concerns | Low |
| EPA:PAR | 0 | No concerns | Low risk | No concerns | Major concerns | No concerns | Major concerns | Low |
| EPA:PROB | 0 | No concerns | Low risk | No concerns | Major concerns | No concerns | Major concerns | Low |
| EPA:PS128 | 0 | No concerns | Low risk | No concerns | No concerns | Major concerns | Major concerns | Low |
| EPA:RHO | 0 | No concerns | Low risk | No concerns | Major concerns | No concerns | Major concerns | Low |
| EPA:RHO+SER | 0 | No concerns | Low risk | No concerns | No concerns | No concerns | Major concerns | Low |
| EPA:SAF | 0 | No concerns | Low risk | No concerns | Major concerns | No concerns | Major concerns | Low |
| EPA:SAFF | 0 | No concerns | Low risk | No concerns | Major concerns | No concerns | Major concerns | Low |
| EPA:SAFF+SCU | 0 | No concerns | Low risk | No concerns | Major concerns | No concerns | Major concerns | Low |
| EPA:SAMe | 0 | No concerns | Low risk | No concerns | Some concerns | Some concerns | Major concerns | Low |
| EPA:SAMe+HELP9 | 0 | No concerns | Low risk | No concerns | No concerns | Some concerns | Major concerns | Low |
| EPA:SCU | 0 | No concerns | Low risk | No concerns | Some concerns | Some concerns | Major concerns | Low |
| EPA:SER | 0 | No concerns | Low risk | No concerns | Some concerns | Some concerns | Major concerns | Low |
| EPA:SJW | 0 | No concerns | Low risk | No concerns | No concerns | Major concerns | Major concerns | Low |
| EPA:SRN | 0 | No concerns | Low risk | No concerns | Major concerns | No concerns | Major concerns | Low |
| EPA:SZL | 0 | No concerns | Low risk | No concerns | Some concerns | Some concerns | Major concerns | Low |
| EPA:TEN | 0 | No concerns | Low risk | Some concerns | Major concerns | No concerns | Major concerns | Very Low |
| EPA:WS5570 | 0 | No concerns | Low risk | No concerns | Some concerns | Some concerns | Major concerns | Low |
| EPA:WS5572 | 0 | No concerns | Low risk | No concerns | Major concerns | No concerns | Major concerns | Low |
| EPA:WS5573 | 0 | No concerns | Low risk | No concerns | Some concerns | Some concerns | Major concerns | Low |
| EPA:ZE | 0 | No concerns | Low risk | No concerns | Major concerns | No concerns | Major concerns | Low |
| EPA:ZE117 | 0 | No concerns | Low risk | No concerns | Major concerns | No concerns | Major concerns | Low |
| EPA:vitB | 0 | No concerns | Low risk | No concerns | Major concerns | No concerns | Major concerns | Low |
| EPA:vitD | 0 | No concerns | Low risk | No concerns | Some concerns | Some concerns | Major concerns | Low |
| EPA:vitD3 | 0 | No concerns | Low risk | No concerns | Major concerns | No concerns | Major concerns | Low |
| EPA:vitD3+CA | 0 | No concerns | Low risk | No concerns | Major concerns | No concerns | Major concerns | Low |
| EPA:vitD3+FLUO | 0 | No concerns | Low risk | No concerns | Major concerns | No concerns | Major concerns | Low |
| EPA+DHA:ESC | 0 | No concerns | Low risk | No concerns | Major concerns | No concerns | Major concerns | Low |
| EPA+DHA:ESC+FOL | 0 | No concerns | Low risk | No concerns | Major concerns | No concerns | Major concerns | Low |
| EPA+DHA:FLUO | 0 | No concerns | Low risk | No concerns | Major concerns | No concerns | Major concerns | Low |
| EPA+DHA:FOIL | 0 | No concerns | Low risk | No concerns | Major concerns | No concerns | Major concerns | Low |
| EPA+DHA:GBDP | 0 | No concerns | Low risk | No concerns | Major concerns | No concerns | Major concerns | Low |
| EPA+DHA:IMI | 0 | No concerns | Low risk | No concerns | Major concerns | No concerns | Major concerns | Low |
| EPA+DHA:INO | 0 | No concerns | Low risk | Some concerns | Major concerns | No concerns | Major concerns | Very Low |
| EPA+DHA:INU | 0 | Some concerns | Low risk | No concerns | Major concerns | No concerns | Major concerns | Very Low |
| EPA+DHA:KOIL | 0 | No concerns | Low risk | No concerns | Major concerns | No concerns | Major concerns | Low |
| EPA+DHA:LAV | 0 | No concerns | Low risk | No concerns | Major concerns | No concerns | Major concerns | Low |
| EPA+DHA:LAV+IMI | 0 | No concerns | Low risk | No concerns | Major concerns | No concerns | Major concerns | Low |
| EPA+DHA:LH57 | 0 | No concerns | Low risk | No concerns | Major concerns | No concerns | Major concerns | Low |
| EPA+DHA:LI-160 | 0 | No concerns | Low risk | No concerns | Major concerns | No concerns | Major concerns | Low |
| EPA+DHA:LMF | 0 | No concerns | Low risk | No concerns | Major concerns | No concerns | Major concerns | Low |
| EPA+DHA:LP299v | 0 | No concerns | Low risk | No concerns | Major concerns | No concerns | Major concerns | Low |
| EPA+DHA:LS | 0 | No concerns | Low risk | No concerns | Major concerns | No concerns | Major concerns | Low |
| EPA+DHA:MEL | 0 | No concerns | Low risk | No concerns | Major concerns | No concerns | Major concerns | Low |
| EPA+DHA:MG | 0 | No concerns | Low risk | No concerns | Major concerns | No concerns | Major concerns | Low |
| EPA+DHA:NUT | 0 | No concerns | Low risk | No concerns | Major concerns | No concerns | Major concerns | Low |
| EPA+DHA:OMB | 0 | No concerns | Low risk | No concerns | Major concerns | No concerns | Major concerns | Low |
| EPA+DHA:OMEGA3 | 0 | No concerns | Low risk | No concerns | Major concerns | No concerns | Major concerns | Low |
| EPA+DHA:PAR | 0 | No concerns | Low risk | No concerns | Major concerns | No concerns | Major concerns | Low |
| EPA+DHA:PROB | 0 | No concerns | Low risk | No concerns | Major concerns | No concerns | Major concerns | Low |
| EPA+DHA:PS128 | 0 | No concerns | Low risk | No concerns | Major concerns | No concerns | Major concerns | Low |
| EPA+DHA:RHO | 0 | No concerns | Low risk | No concerns | No concerns | Some concerns | Major concerns | Low |
| EPA+DHA:SAF | 0 | No concerns | Low risk | No concerns | Major concerns | No concerns | Major concerns | Low |
| EPA+DHA:SAFF | 0 | No concerns | Low risk | No concerns | Some concerns | Some concerns | Major concerns | Low |
| EPA+DHA:SAFF+SCU | 0 | No concerns | Low risk | No concerns | Major concerns | No concerns | Major concerns | Low |
| EPA+DHA:SAMe | 0 | No concerns | Low risk | No concerns | Major concerns | No concerns | Major concerns | Low |
| EPA+DHA:SAMe+HELP9 | 0 | No concerns | Low risk | No concerns | Major concerns | No concerns | Major concerns | Low |
| EPA+DHA:SCU | 0 | No concerns | Low risk | No concerns | Major concerns | No concerns | Major concerns | Low |
| EPA+DHA:SER | 0 | No concerns | Low risk | No concerns | Major concerns | No concerns | Major concerns | Low |
| EPA+DHA:SJW | 0 | No concerns | Low risk | No concerns | Major concerns | No concerns | Major concerns | Low |
| EPA+DHA:SRN | 0 | No concerns | Low risk | No concerns | Major concerns | No concerns | Major concerns | Low |
| EPA+DHA:SZL | 0 | No concerns | Low risk | No concerns | Major concerns | No concerns | Major concerns | Low |
| EPA+DHA:TEN | 0 | No concerns | Low risk | No concerns | No concerns | Major concerns | Major concerns | Low |
| EPA+DHA:WS5570 | 0 | No concerns | Low risk | No concerns | Major concerns | No concerns | Major concerns | Low |
| EPA+DHA:WS5572 | 0 | No concerns | Low risk | No concerns | Major concerns | No concerns | Major concerns | Low |
| EPA+DHA:WS5573 | 0 | No concerns | Low risk | No concerns | Major concerns | No concerns | Major concerns | Low |
| EPA+DHA:ZE | 0 | No concerns | Low risk | Some concerns | Major concerns | No concerns | Major concerns | Very Low |
| EPA+DHA:ZE117 | 0 | No concerns | Low risk | No concerns | Major concerns | No concerns | Major concerns | Low |
| EPA+DHA:vitB | 0 | No concerns | Low risk | No concerns | Some concerns | Some concerns | Major concerns | Low |
| EPA+DHA:vitD | 0 | No concerns | Low risk | No concerns | Major concerns | No concerns | Major concerns | Low |
| EPA+DHA:vitD3 | 0 | No concerns | Low risk | No concerns | Some concerns | Some concerns | Major concerns | Low |
| EPA+DHA:vitD3+CA | 0 | No concerns | Low risk | No concerns | Major concerns | No concerns | Major concerns | Low |
| EPA+DHA:vitD3+FLUO | 0 | No concerns | Low risk | No concerns | Major concerns | No concerns | Major concerns | Low |
| ESC:FLUO | 0 | No concerns | Low risk | No concerns | Major concerns | No concerns | Major concerns | Low |
| ESC:FOIL | 0 | No concerns | Low risk | No concerns | Major concerns | No concerns | Major concerns | Low |
| ESC:GBDP | 0 | No concerns | Low risk | No concerns | Major concerns | No concerns | Major concerns | Low |
| ESC:IMI | 0 | No concerns | Low risk | No concerns | Major concerns | No concerns | Major concerns | Low |
| ESC:INO | 0 | No concerns | Low risk | No concerns | Major concerns | No concerns | Major concerns | Low |
| ESC:INU | 0 | No concerns | Low risk | No concerns | Major concerns | No concerns | Major concerns | Low |
| ESC:KOIL | 0 | No concerns | Low risk | No concerns | Major concerns | No concerns | Major concerns | Low |
| ESC:LAV | 0 | No concerns | Low risk | No concerns | Major concerns | No concerns | Major concerns | Low |
| ESC:LAV+IMI | 0 | No concerns | Low risk | No concerns | Major concerns | No concerns | Major concerns | Low |
| ESC:LH57 | 0 | No concerns | Low risk | No concerns | Major concerns | No concerns | Major concerns | Low |
| ESC:LI-160 | 0 | No concerns | Low risk | No concerns | Major concerns | No concerns | Major concerns | Low |
| ESC:LMF | 0 | No concerns | Low risk | No concerns | Major concerns | No concerns | Major concerns | Low |
| ESC:LP299v | 0 | No concerns | Low risk | No concerns | Major concerns | No concerns | Major concerns | Low |
| ESC:LS | 0 | No concerns | Low risk | Some concerns | Major concerns | No concerns | Major concerns | Very Low |
| ESC:MEL | 0 | No concerns | Low risk | No concerns | Major concerns | No concerns | Major concerns | Low |
| ESC:MG | 0 | No concerns | Low risk | No concerns | Major concerns | No concerns | Major concerns | Low |
| ESC:NUT | 0 | No concerns | Low risk | No concerns | Major concerns | No concerns | Major concerns | Low |
| ESC:OMB | 0 | No concerns | Low risk | No concerns | Major concerns | No concerns | Major concerns | Low |
| ESC:OMEGA3 | 0 | No concerns | Low risk | No concerns | Major concerns | No concerns | Major concerns | Low |
| ESC:PAR | 0 | No concerns | Low risk | No concerns | Major concerns | No concerns | Major concerns | Low |
| ESC:PROB | 0 | No concerns | Low risk | No concerns | Major concerns | No concerns | Major concerns | Low |
| ESC:PS128 | 0 | No concerns | Low risk | No concerns | Major concerns | No concerns | Major concerns | Low |
| ESC:RHO | 0 | No concerns | Low risk | No concerns | Some concerns | Some concerns | Major concerns | Low |
| ESC:RHO+SER | 0 | No concerns | Low risk | No concerns | No concerns | No concerns | Major concerns | Low |
| ESC:SAF | 0 | No concerns | Low risk | No concerns | Major concerns | No concerns | Major concerns | Low |
| ESC:SAFF | 0 | No concerns | Low risk | No concerns | Major concerns | No concerns | Major concerns | Low |
| ESC:SAFF+SCU | 0 | No concerns | Low risk | No concerns | Major concerns | No concerns | Major concerns | Low |
| ESC:SAMe+HELP9 | 0 | No concerns | Low risk | No concerns | Major concerns | No concerns | Major concerns | Low |
| ESC:SCU | 0 | No concerns | Low risk | No concerns | Major concerns | No concerns | Major concerns | Low |
| ESC:SER | 0 | No concerns | Low risk | No concerns | Major concerns | No concerns | Major concerns | Low |
| ESC:SJW | 0 | No concerns | Low risk | No concerns | Major concerns | No concerns | Major concerns | Low |
| ESC:SRN | 0 | No concerns | Low risk | No concerns | Major concerns | No concerns | Major concerns | Low |
| ESC:SZL | 0 | No concerns | Low risk | No concerns | Major concerns | No concerns | Major concerns | Low |
| ESC:TEN | 0 | No concerns | Low risk | No concerns | Some concerns | Some concerns | Major concerns | Low |
| ESC:WS5570 | 0 | No concerns | Low risk | No concerns | Major concerns | No concerns | Major concerns | Low |
| ESC:WS5572 | 0 | No concerns | Low risk | No concerns | Major concerns | No concerns | Major concerns | Low |
| ESC:WS5573 | 0 | No concerns | Low risk | No concerns | Major concerns | No concerns | Major concerns | Low |
| ESC:ZE | 0 | No concerns | Low risk | No concerns | Major concerns | No concerns | Major concerns | Low |
| ESC:ZE117 | 0 | No concerns | Low risk | No concerns | Major concerns | No concerns | Major concerns | Low |
| ESC:vitB | 0 | No concerns | Low risk | No concerns | Major concerns | No concerns | Major concerns | Low |
| ESC:vitD | 0 | No concerns | Low risk | No concerns | Major concerns | No concerns | Major concerns | Low |
| ESC:vitD3 | 0 | No concerns | Low risk | No concerns | Major concerns | No concerns | Major concerns | Low |
| ESC:vitD3+CA | 0 | No concerns | Low risk | No concerns | Major concerns | No concerns | Major concerns | Low |
| ESC:vitD3+FLUO | 0 | No concerns | Low risk | No concerns | Major concerns | No concerns | Major concerns | Low |
| ESC+FOL:FLUO | 0 | No concerns | Low risk | No concerns | Major concerns | No concerns | Major concerns | Low |
| ESC+FOL:FOIL | 0 | No concerns | Low risk | No concerns | Major concerns | No concerns | Major concerns | Low |
| ESC+FOL:GBDP | 0 | No concerns | Low risk | No concerns | Major concerns | No concerns | Major concerns | Low |
| ESC+FOL:IMI | 0 | No concerns | Low risk | No concerns | Major concerns | No concerns | Major concerns | Low |
| ESC+FOL:INO | 0 | No concerns | Low risk | No concerns | Major concerns | No concerns | Major concerns | Low |
| ESC+FOL:INU | 0 | No concerns | Low risk | Some concerns | Major concerns | No concerns | Major concerns | Very Low |
| ESC+FOL:KOIL | 0 | No concerns | Low risk | No concerns | Major concerns | No concerns | Major concerns | Low |
| ESC+FOL:LAV | 0 | No concerns | Low risk | No concerns | Major concerns | No concerns | Major concerns | Low |
| ESC+FOL:LAV+IMI | 0 | No concerns | Low risk | No concerns | Major concerns | No concerns | Major concerns | Low |
| ESC+FOL:LH57 | 0 | No concerns | Low risk | No concerns | Major concerns | No concerns | Major concerns | Low |
| ESC+FOL:LI-160 | 0 | No concerns | Low risk | No concerns | Major concerns | No concerns | Major concerns | Low |
| ESC+FOL:LMF | 0 | No concerns | Low risk | No concerns | Major concerns | No concerns | Major concerns | Low |
| ESC+FOL:LP299v | 0 | No concerns | Low risk | No concerns | Major concerns | No concerns | Major concerns | Low |
| ESC+FOL:LS | 0 | No concerns | Low risk | No concerns | Major concerns | No concerns | Major concerns | Low |
| ESC+FOL:MEL | 0 | No concerns | Low risk | No concerns | Major concerns | No concerns | Major concerns | Low |
| ESC+FOL:MG | 0 | No concerns | Low risk | No concerns | Major concerns | No concerns | Major concerns | Low |
| ESC+FOL:NUT | 0 | No concerns | Low risk | No concerns | Major concerns | No concerns | Major concerns | Low |
| ESC+FOL:OMB | 0 | No concerns | Low risk | Some concerns | Major concerns | No concerns | Major concerns | Very Low |
| ESC+FOL:OMEGA3 | 0 | No concerns | Low risk | No concerns | Major concerns | No concerns | Major concerns | Low |
| ESC+FOL:PAR | 0 | No concerns | Low risk | No concerns | Major concerns | No concerns | Major concerns | Low |
| ESC+FOL:PROB | 0 | No concerns | Low risk | No concerns | Major concerns | No concerns | Major concerns | Low |
| ESC+FOL:PLB | 0 | No concerns | Low risk | No concerns | Major concerns | No concerns | Major concerns | Low |
| ESC+FOL:PS128 | 0 | No concerns | Low risk | No concerns | Major concerns | No concerns | Major concerns | Low |
| ESC+FOL:RHO | 0 | No concerns | Low risk | No concerns | No concerns | Major concerns | Major concerns | Low |
| ESC+FOL:RHO+SER | 0 | No concerns | Low risk | No concerns | No concerns | No concerns | Major concerns | Low |
| ESC+FOL:SAF | 0 | No concerns | Low risk | No concerns | Major concerns | No concerns | Major concerns | Low |
| ESC+FOL:SAFF | 0 | No concerns | Low risk | No concerns | Major concerns | No concerns | Major concerns | Low |
| ESC+FOL:SAFF+SCU | 0 | No concerns | Low risk | No concerns | Major concerns | No concerns | Major concerns | Low |
| ESC+FOL:SAMe | 0 | No concerns | Low risk | No concerns | Major concerns | No concerns | Major concerns | Low |
| ESC+FOL:SAMe+HELP9 | 0 | No concerns | Low risk | No concerns | Major concerns | No concerns | Major concerns | Low |
| ESC+FOL:SCU | 0 | No concerns | Low risk | No concerns | Major concerns | No concerns | Major concerns | Low |
| ESC+FOL:SER | 0 | No concerns | Low risk | No concerns | Major concerns | No concerns | Major concerns | Low |
| ESC+FOL:SJW | 0 | No concerns | Low risk | No concerns | Major concerns | No concerns | Major concerns | Low |
| ESC+FOL:SRN | 0 | No concerns | Low risk | No concerns | Major concerns | No concerns | Major concerns | Low |
| ESC+FOL:SZL | 0 | No concerns | Low risk | No concerns | Major concerns | No concerns | Major concerns | Low |
| ESC+FOL:TEN | 0 | No concerns | Low risk | No concerns | Some concerns | Some concerns | Major concerns | Low |
| ESC+FOL:WS5570 | 0 | No concerns | Low risk | No concerns | Major concerns | No concerns | Major concerns | Low |
| ESC+FOL:WS5572 | 0 | No concerns | Low risk | No concerns | Major concerns | No concerns | Major concerns | Low |
| ESC+FOL:WS5573 | 0 | No concerns | Low risk | No concerns | Major concerns | No concerns | Major concerns | Low |
| ESC+FOL:ZE | 0 | No concerns | Low risk | No concerns | Major concerns | No concerns | Major concerns | Low |
| ESC+FOL:ZE117 | 0 | No concerns | Low risk | No concerns | Major concerns | No concerns | Major concerns | Low |
| ESC+FOL:vitB | 0 | No concerns | Low risk | No concerns | Major concerns | No concerns | Major concerns | Low |
| ESC+FOL:vitD | 0 | No concerns | Low risk | No concerns | Major concerns | No concerns | Major concerns | Low |
| ESC+FOL:vitD3 | 0 | No concerns | Low risk | No concerns | Major concerns | No concerns | Major concerns | Low |
| ESC+FOL:vitD3+CA | 0 | No concerns | Low risk | No concerns | Major concerns | No concerns | Major concerns | Low |
| ESC+FOL:vitD3+FLUO | 0 | No concerns | Low risk | No concerns | Major concerns | No concerns | Major concerns | Low |
| FLUO:FOIL | 0 | No concerns | Low risk | No concerns | Major concerns | No concerns | Major concerns | Low |
| FLUO:GBDP | 0 | No concerns | Low risk | No concerns | Major concerns | No concerns | Major concerns | Low |
| FLUO:IMI | 0 | No concerns | Low risk | No concerns | Major concerns | No concerns | Major concerns | Low |
| FLUO:INO | 0 | No concerns | Low risk | No concerns | Major concerns | No concerns | Major concerns | Low |
| FLUO:INU | 0 | No concerns | Low risk | No concerns | Major concerns | No concerns | Major concerns | Low |
| FLUO:KOIL | 0 | No concerns | Low risk | No concerns | Major concerns | No concerns | Major concerns | Low |
| FLUO:LAV | 0 | No concerns | Low risk | No concerns | Major concerns | No concerns | Major concerns | Low |
| FLUO:LAV+IMI | 0 | No concerns | Low risk | No concerns | Some concerns | Some concerns | Major concerns | Low |
| FLUO:LMF | 0 | No concerns | Low risk | No concerns | Major concerns | No concerns | Major concerns | Low |
| FLUO:LP299v | 0 | No concerns | Low risk | No concerns | Major concerns | No concerns | Major concerns | Low |
| FLUO:LS | 0 | No concerns | Low risk | No concerns | Major concerns | No concerns | Major concerns | Low |
| FLUO:MEL | 0 | No concerns | Low risk | No concerns | Major concerns | No concerns | Major concerns | Low |
| FLUO:MG | 0 | No concerns | Low risk | No concerns | Major concerns | No concerns | Major concerns | Low |
| FLUO:NUT | 0 | No concerns | Low risk | No concerns | Major concerns | No concerns | Major concerns | Low |
| FLUO:OMB | 0 | No concerns | Low risk | No concerns | Major concerns | No concerns | Major concerns | Low |
| FLUO:OMEGA3 | 0 | No concerns | Low risk | No concerns | Major concerns | No concerns | Major concerns | Low |
| FLUO:PAR | 0 | No concerns | Low risk | No concerns | Major concerns | No concerns | Major concerns | Low |
| FLUO:PROB | 0 | No concerns | Low risk | No concerns | Major concerns | No concerns | Major concerns | Low |
| FLUO:PS128 | 0 | No concerns | Low risk | No concerns | Major concerns | No concerns | Major concerns | Low |
| FLUO:RHO | 0 | No concerns | Low risk | No concerns | No concerns | No concerns | Major concerns | Low |
| FLUO:RHO+SER | 0 | No concerns | Low risk | No concerns | No concerns | No concerns | Major concerns | Low |
| FLUO:SAF | 0 | No concerns | Low risk | No concerns | Major concerns | No concerns | Major concerns | Low |
| FLUO:SAFF+SCU | 0 | No concerns | Low risk | No concerns | Major concerns | No concerns | Major concerns | Low |
| FLUO:SAMe | 0 | No concerns | Low risk | No concerns | Major concerns | No concerns | Major concerns | Low |
| FLUO:SAMe+HELP9 | 0 | No concerns | Low risk | No concerns | Major concerns | No concerns | Major concerns | Low |
| FLUO:SCU | 0 | No concerns | Low risk | Some concerns | Major concerns | No concerns | Major concerns | Very Low |
| FLUO:SER | 0 | No concerns | Low risk | No concerns | Major concerns | No concerns | Major concerns | Low |
| FLUO:SRN | 0 | No concerns | Low risk | No concerns | Major concerns | No concerns | Major concerns | Low |
| FLUO:TEN | 0 | No concerns | Low risk | No concerns | No concerns | Some concerns | Major concerns | Low |
| FLUO:WS5570 | 0 | No concerns | Low risk | No concerns | Major concerns | No concerns | Major concerns | Low |
| FLUO:WS5572 | 0 | No concerns | Low risk | No concerns | Major concerns | No concerns | Major concerns | Low |
| FLUO:WS5573 | 0 | No concerns | Low risk | No concerns | Major concerns | No concerns | Major concerns | Low |
| FLUO:ZE | 0 | No concerns | Low risk | No concerns | Major concerns | No concerns | Major concerns | Low |
| FLUO:vitB | 0 | No concerns | Low risk | No concerns | No concerns | Major concerns | Major concerns | Low |
| FLUO:vitD | 0 | No concerns | Low risk | No concerns | Major concerns | No concerns | Major concerns | Low |
| FLUO:vitD3 | 0 | No concerns | Low risk | No concerns | No concerns | Major concerns | Major concerns | Low |
| FLUO:vitD3+CA | 0 | No concerns | Low risk | No concerns | Major concerns | No concerns | Major concerns | Low |
| FOIL:GBDP | 0 | No concerns | Low risk | No concerns | Major concerns | No concerns | Major concerns | Low |
| FOIL:IMI | 0 | No concerns | Low risk | No concerns | Major concerns | No concerns | Major concerns | Low |
| FOIL:INO | 0 | No concerns | Low risk | No concerns | Major concerns | No concerns | Major concerns | Low |
| FOIL:INU | 0 | No concerns | Low risk | No concerns | Major concerns | No concerns | Major concerns | Low |
| FOIL:LAV | 0 | No concerns | Low risk | No concerns | Major concerns | No concerns | Major concerns | Low |
| FOIL:LAV+IMI | 0 | No concerns | Low risk | No concerns | Major concerns | No concerns | Major concerns | Low |
| FOIL:LH57 | 0 | No concerns | Low risk | No concerns | Major concerns | No concerns | Major concerns | Low |
| FOIL:LI-160 | 0 | No concerns | Low risk | No concerns | Major concerns | No concerns | Major concerns | Low |
| FOIL:LMF | 0 | No concerns | Low risk | No concerns | Major concerns | No concerns | Major concerns | Low |
| FOIL:LP299v | 0 | No concerns | Low risk | No concerns | Major concerns | No concerns | Major concerns | Low |
| FOIL:LS | 0 | No concerns | Low risk | No concerns | Major concerns | No concerns | Major concerns | Low |
| FOIL:MEL | 0 | No concerns | Low risk | No concerns | Major concerns | No concerns | Major concerns | Low |
| FOIL:MG | 0 | No concerns | Low risk | No concerns | Major concerns | No concerns | Major concerns | Low |
| FOIL:NUT | 0 | No concerns | Low risk | No concerns | Major concerns | No concerns | Major concerns | Low |
| FOIL:OMB | 0 | No concerns | Low risk | No concerns | Major concerns | No concerns | Major concerns | Low |
| FOIL:OMEGA3 | 0 | No concerns | Low risk | No concerns | Major concerns | No concerns | Major concerns | Low |
| FOIL:PAR | 0 | No concerns | Low risk | No concerns | Major concerns | No concerns | Major concerns | Low |
| FOIL:PROB | 0 | No concerns | Low risk | No concerns | Major concerns | No concerns | Major concerns | Low |
| FOIL:PS128 | 0 | No concerns | Low risk | No concerns | Major concerns | No concerns | Major concerns | Low |
| FOIL:RHO | 0 | No concerns | Low risk | No concerns | No concerns | Major concerns | Major concerns | Low |
| FOIL:RHO+SER | 0 | No concerns | Low risk | No concerns | No concerns | No concerns | Major concerns | Low |
| FOIL:SAF | 0 | No concerns | Low risk | No concerns | Major concerns | No concerns | Major concerns | Low |
| FOIL:SAFF | 0 | No concerns | Low risk | No concerns | Major concerns | No concerns | Major concerns | Low |
| FOIL:SAFF+SCU | 0 | No concerns | Low risk | No concerns | Major concerns | No concerns | Major concerns | Low |
| FOIL:SAMe | 0 | No concerns | Low risk | No concerns | Major concerns | No concerns | Major concerns | Low |
| FOIL:SAMe+HELP9 | 0 | No concerns | Low risk | No concerns | Major concerns | No concerns | Major concerns | Low |
| FOIL:SCU | 0 | No concerns | Low risk | No concerns | Major concerns | No concerns | Major concerns | Low |
| FOIL:SER | 0 | No concerns | Low risk | No concerns | Major concerns | No concerns | Major concerns | Low |
| FOIL:SJW | 0 | No concerns | Low risk | Some concerns | Major concerns | No concerns | Major concerns | Very Low |
| FOIL:SRN | 0 | No concerns | Low risk | No concerns | Major concerns | No concerns | Major concerns | Low |
| FOIL:SZL | 0 | No concerns | Low risk | No concerns | Major concerns | No concerns | Major concerns | Low |
| FOIL:TEN | 0 | No concerns | Low risk | No concerns | Some concerns | Some concerns | Major concerns | Low |
| FOIL:WS5570 | 0 | No concerns | Low risk | No concerns | Major concerns | No concerns | Major concerns | Low |
| FOIL:WS5572 | 0 | No concerns | Low risk | No concerns | Major concerns | No concerns | Major concerns | Low |
| FOIL:WS5573 | 0 | No concerns | Low risk | No concerns | Major concerns | No concerns | Major concerns | Low |
| FOIL:ZE | 0 | No concerns | Low risk | No concerns | Major concerns | No concerns | Major concerns | Low |
| FOIL:ZE117 | 0 | No concerns | Low risk | No concerns | Major concerns | No concerns | Major concerns | Low |
| FOIL:vitB | 0 | No concerns | Low risk | No concerns | Major concerns | No concerns | Major concerns | Low |
| FOIL:vitD | 0 | No concerns | Low risk | No concerns | Major concerns | No concerns | Major concerns | Low |
| FOIL:vitD3 | 0 | No concerns | Low risk | No concerns | Major concerns | No concerns | Major concerns | Low |
| FOIL:vitD3+CA | 0 | No concerns | Low risk | No concerns | Major concerns | No concerns | Major concerns | Low |
| FOIL:vitD3+FLUO | 0 | No concerns | Low risk | No concerns | Major concerns | No concerns | Major concerns | Low |
| GBDP:IMI | 0 | No concerns | Low risk | Some concerns | Major concerns | No concerns | Major concerns | Very Low |
| GBDP:INO | 0 | No concerns | Low risk | No concerns | Major concerns | No concerns | Major concerns | Low |
| GBDP:INU | 0 | No concerns | Low risk | No concerns | Major concerns | No concerns | Major concerns | Low |
| GBDP:KOIL | 0 | No concerns | Low risk | No concerns | Major concerns | No concerns | Major concerns | Low |
| GBDP:LAV | 0 | No concerns | Low risk | No concerns | Major concerns | No concerns | Major concerns | Low |
| GBDP:LAV+IMI | 0 | No concerns | Low risk | No concerns | Major concerns | No concerns | Major concerns | Low |
| GBDP:LH57 | 0 | No concerns | Low risk | No concerns | Major concerns | No concerns | Major concerns | Low |
| GBDP:LI-160 | 0 | No concerns | Low risk | No concerns | Major concerns | No concerns | Major concerns | Low |
| GBDP:LMF | 0 | No concerns | Low risk | No concerns | Major concerns | No concerns | Major concerns | Low |
| GBDP:LP299v | 0 | No concerns | Low risk | No concerns | Major concerns | No concerns | Major concerns | Low |
| GBDP:LS | 0 | No concerns | Low risk | No concerns | Major concerns | No concerns | Major concerns | Low |
| GBDP:MEL | 0 | No concerns | Low risk | No concerns | Major concerns | No concerns | Major concerns | Low |
| GBDP:MG | 0 | No concerns | Low risk | No concerns | Major concerns | No concerns | Major concerns | Low |
| GBDP:NUT | 0 | No concerns | Low risk | No concerns | Major concerns | No concerns | Major concerns | Low |
| GBDP:OMB | 0 | No concerns | Low risk | No concerns | Major concerns | No concerns | Major concerns | Low |
| GBDP:OMEGA3 | 0 | No concerns | Low risk | No concerns | Major concerns | No concerns | Major concerns | Low |
| GBDP:PAR | 0 | No concerns | Low risk | No concerns | Major concerns | No concerns | Major concerns | Low |
| GBDP:PROB | 0 | No concerns | Low risk | No concerns | Major concerns | No concerns | Major concerns | Low |
| GBDP:PS128 | 0 | No concerns | Low risk | No concerns | Major concerns | No concerns | Major concerns | Low |
| GBDP:RHO | 0 | No concerns | Low risk | No concerns | No concerns | Major concerns | Major concerns | Low |
| GBDP:RHO+SER | 0 | No concerns | Low risk | No concerns | No concerns | No concerns | Major concerns | Low |
| GBDP:SAF | 0 | No concerns | Low risk | No concerns | Major concerns | No concerns | Major concerns | Low |
| GBDP:SAFF | 0 | No concerns | Low risk | No concerns | Major concerns | No concerns | Major concerns | Low |
| GBDP:SAFF+SCU | 0 | No concerns | Low risk | No concerns | Major concerns | No concerns | Major concerns | Low |
| GBDP:SAMe | 0 | No concerns | Low risk | No concerns | Major concerns | No concerns | Major concerns | Low |
| GBDP:SAMe+HELP9 | 0 | No concerns | Low risk | No concerns | Major concerns | No concerns | Major concerns | Low |
| GBDP:SCU | 0 | No concerns | Low risk | No concerns | Major concerns | No concerns | Major concerns | Low |
| GBDP:SER | 0 | No concerns | Low risk | No concerns | Major concerns | No concerns | Major concerns | Low |
| GBDP:SJW | 0 | No concerns | Low risk | No concerns | Major concerns | No concerns | Major concerns | Low |
| GBDP:SRN | 0 | No concerns | Low risk | No concerns | Major concerns | No concerns | Major concerns | Low |
| GBDP:SZL | 0 | No concerns | Low risk | No concerns | Major concerns | No concerns | Major concerns | Low |
| GBDP:TEN | 0 | No concerns | Low risk | No concerns | No concerns | Major concerns | Major concerns | Low |
| GBDP:WS5570 | 0 | No concerns | Low risk | No concerns | Major concerns | No concerns | Major concerns | Low |
| GBDP:WS5572 | 0 | No concerns | Low risk | No concerns | Major concerns | No concerns | Major concerns | Low |
| GBDP:WS5573 | 0 | No concerns | Low risk | No concerns | Major concerns | No concerns | Major concerns | Low |
| GBDP:ZE | 0 | No concerns | Low risk | No concerns | Major concerns | No concerns | Major concerns | Low |
| GBDP:ZE117 | 0 | No concerns | Low risk | No concerns | Major concerns | No concerns | Major concerns | Low |
| GBDP:vitB | 0 | No concerns | Low risk | No concerns | Major concerns | No concerns | Major concerns | Low |
| GBDP:vitD | 0 | No concerns | Low risk | No concerns | Major concerns | No concerns | Major concerns | Low |
| GBDP:vitD3 | 0 | No concerns | Low risk | No concerns | Major concerns | No concerns | Major concerns | Low |
| GBDP:vitD3+CA | 0 | No concerns | Low risk | No concerns | Major concerns | No concerns | Major concerns | Low |
| GBDP:vitD3+FLUO | 0 | No concerns | Low risk | No concerns | Major concerns | No concerns | Major concerns | Low |
| IMI:INO | 0 | No concerns | Low risk | No concerns | Major concerns | No concerns | Major concerns | Low |
| IMI:INU | 0 | No concerns | Low risk | No concerns | Major concerns | No concerns | Major concerns | Low |
| IMI:KOIL | 0 | No concerns | Low risk | No concerns | Major concerns | No concerns | Major concerns | Low |
| IMI:LH57 | 0 | No concerns | Low risk | No concerns | Major concerns | No concerns | Major concerns | Low |
| IMI:LMF | 0 | No concerns | Low risk | No concerns | Major concerns | No concerns | Major concerns | Low |
| IMI:LP299v | 0 | No concerns | Low risk | No concerns | Major concerns | No concerns | Major concerns | Low |
| IMI:LS | 0 | No concerns | Low risk | No concerns | Major concerns | No concerns | Major concerns | Low |
| IMI:MEL | 0 | No concerns | Low risk | No concerns | Major concerns | No concerns | Major concerns | Low |
| IMI:MG | 0 | No concerns | Low risk | No concerns | Major concerns | No concerns | Major concerns | Low |
| IMI:NUT | 0 | No concerns | Low risk | No concerns | Major concerns | No concerns | Major concerns | Low |
| IMI:OMB | 0 | No concerns | Low risk | No concerns | Major concerns | No concerns | Major concerns | Low |
| IMI:OMEGA3 | 0 | No concerns | Low risk | No concerns | Major concerns | No concerns | Major concerns | Low |
| IMI:PAR | 0 | No concerns | Low risk | No concerns | Major concerns | No concerns | Major concerns | Low |
| IMI:PROB | 0 | No concerns | Low risk | No concerns | Major concerns | No concerns | Major concerns | Low |
| IMI:PLB | 0 | No concerns | Low risk | No concerns | Major concerns | No concerns | Major concerns | Low |
| IMI:PS128 | 0 | No concerns | Low risk | No concerns | Major concerns | No concerns | Major concerns | Low |
| IMI:RHO | 0 | No concerns | Low risk | No concerns | Major concerns | No concerns | Major concerns | Low |
| IMI:RHO+SER | 0 | No concerns | Low risk | No concerns | No concerns | No concerns | Major concerns | Low |
| IMI:SAF | 0 | No concerns | Low risk | No concerns | Major concerns | No concerns | Major concerns | Low |
| IMI:SAFF | 0 | No concerns | Low risk | No concerns | Major concerns | No concerns | Major concerns | Low |
| IMI:SAFF+SCU | 0 | No concerns | Low risk | No concerns | Major concerns | No concerns | Major concerns | Low |
| IMI:SAMe | 0 | No concerns | Low risk | No concerns | Major concerns | No concerns | Major concerns | Low |
| IMI:SAMe+HELP9 | 0 | No concerns | Low risk | No concerns | Major concerns | No concerns | Major concerns | Low |
| IMI:SCU | 0 | No concerns | Low risk | No concerns | Major concerns | No concerns | Major concerns | Low |
| IMI:SER | 0 | No concerns | Low risk | No concerns | Major concerns | No concerns | Major concerns | Low |
| IMI:SJW | 0 | No concerns | Low risk | No concerns | Major concerns | No concerns | Major concerns | Low |
| IMI:SRN | 0 | No concerns | Low risk | No concerns | Major concerns | No concerns | Major concerns | Low |
| IMI:SZL | 0 | No concerns | Low risk | No concerns | Major concerns | No concerns | Major concerns | Low |
| IMI:TEN | 0 | No concerns | Low risk | No concerns | Major concerns | No concerns | Major concerns | Low |
| IMI:WS5570 | 0 | No concerns | Low risk | No concerns | Major concerns | No concerns | Major concerns | Low |
| IMI:WS5572 | 0 | No concerns | Low risk | Some concerns | Major concerns | No concerns | Major concerns | Very Low |
| IMI:WS5573 | 0 | No concerns | Low risk | No concerns | Major concerns | No concerns | Major concerns | Low |
| IMI:ZE117 | 0 | No concerns | Low risk | No concerns | Major concerns | No concerns | Major concerns | Low |
| IMI:vitB | 0 | No concerns | Low risk | No concerns | Major concerns | No concerns | Major concerns | Low |
| IMI:vitD | 0 | No concerns | Low risk | No concerns | Major concerns | No concerns | Major concerns | Low |
| IMI:vitD3 | 0 | No concerns | Low risk | No concerns | Major concerns | No concerns | Major concerns | Low |
| IMI:vitD3+CA | 0 | No concerns | Low risk | No concerns | Major concerns | No concerns | Major concerns | Low |
| IMI:vitD3+FLUO | 0 | No concerns | Low risk | No concerns | Major concerns | No concerns | Major concerns | Low |
| INO:INU | 0 | No concerns | Low risk | No concerns | Major concerns | No concerns | Major concerns | Low |
| INO:KOIL | 0 | No concerns | Low risk | No concerns | Major concerns | No concerns | Major concerns | Low |
| INO:LAV | 0 | No concerns | Low risk | No concerns | Major concerns | No concerns | Major concerns | Low |
| INO:LAV+IMI | 0 | No concerns | Low risk | No concerns | Major concerns | No concerns | Major concerns | Low |
| INO:LH57 | 0 | No concerns | Low risk | No concerns | Major concerns | No concerns | Major concerns | Low |
| INO:LI-160 | 0 | No concerns | Low risk | No concerns | Major concerns | No concerns | Major concerns | Low |
| INO:LMF | 0 | No concerns | Low risk | No concerns | Major concerns | No concerns | Major concerns | Low |
| INO:LP299v | 0 | No concerns | Low risk | No concerns | Major concerns | No concerns | Major concerns | Low |
| INO:LS | 0 | No concerns | Low risk | No concerns | Major concerns | No concerns | Major concerns | Low |
| INO:MEL | 0 | No concerns | Low risk | No concerns | Major concerns | No concerns | Major concerns | Low |
| INO:MG | 0 | No concerns | Low risk | No concerns | Major concerns | No concerns | Major concerns | Low |
| INO:NUT | 0 | No concerns | Low risk | No concerns | Major concerns | No concerns | Major concerns | Low |
| INO:OMB | 0 | No concerns | Low risk | No concerns | Major concerns | No concerns | Major concerns | Low |
| INO:OMEGA3 | 0 | No concerns | Low risk | No concerns | Major concerns | No concerns | Major concerns | Low |
| INO:PAR | 0 | No concerns | Low risk | No concerns | Major concerns | No concerns | Major concerns | Low |
| INO:PROB | 0 | No concerns | Low risk | No concerns | Major concerns | No concerns | Major concerns | Low |
| INO:PS128 | 0 | No concerns | Low risk | No concerns | Major concerns | No concerns | Major concerns | Low |
| INO:RHO | 0 | No concerns | Low risk | No concerns | Major concerns | No concerns | Major concerns | Low |
| INO:RHO+SER | 0 | No concerns | Low risk | No concerns | No concerns | No concerns | Major concerns | Low |
| INO:SAF | 0 | No concerns | Low risk | No concerns | Major concerns | No concerns | Major concerns | Low |
| INO:SAFF | 0 | No concerns | Low risk | No concerns | Major concerns | No concerns | Major concerns | Low |
| INO:SAFF+SCU | 0 | No concerns | Low risk | No concerns | Major concerns | No concerns | Major concerns | Low |
| INO:SAMe | 0 | No concerns | Low risk | No concerns | Major concerns | No concerns | Major concerns | Low |
| INO:SAMe+HELP9 | 0 | No concerns | Low risk | No concerns | Major concerns | No concerns | Major concerns | Low |
| INO:SCU | 0 | No concerns | Low risk | No concerns | Major concerns | No concerns | Major concerns | Low |
| INO:SER | 0 | No concerns | Low risk | Some concerns | Major concerns | No concerns | Major concerns | Very Low |
| INO:SJW | 0 | No concerns | Low risk | No concerns | Major concerns | No concerns | Major concerns | Low |
| INO:SRN | 0 | No concerns | Low risk | No concerns | Major concerns | No concerns | Major concerns | Low |
| INO:SZL | 0 | No concerns | Low risk | No concerns | Major concerns | No concerns | Major concerns | Low |
| INO:TEN | 0 | No concerns | Low risk | No concerns | Major concerns | No concerns | Major concerns | Low |
| INO:WS5570 | 0 | No concerns | Low risk | No concerns | Major concerns | No concerns | Major concerns | Low |
| INO:WS5572 | 0 | No concerns | Low risk | No concerns | Major concerns | No concerns | Major concerns | Low |
| INO:WS5573 | 0 | No concerns | Low risk | No concerns | Major concerns | No concerns | Major concerns | Low |
| INO:ZE | 0 | No concerns | Low risk | No concerns | Major concerns | No concerns | Major concerns | Low |
| INO:ZE117 | 0 | No concerns | Low risk | No concerns | Major concerns | No concerns | Major concerns | Low |
| INO:vitB | 0 | No concerns | Low risk | No concerns | Major concerns | No concerns | Major concerns | Low |
| INO:vitD | 0 | No concerns | Low risk | No concerns | Major concerns | No concerns | Major concerns | Low |
| INO:vitD3 | 0 | No concerns | Low risk | No concerns | Major concerns | No concerns | Major concerns | Low |
| INO:vitD3+CA | 0 | No concerns | Low risk | No concerns | Major concerns | No concerns | Major concerns | Low |
| INO:vitD3+FLUO | 0 | No concerns | Low risk | No concerns | Major concerns | No concerns | Major concerns | Low |
| INU:KOIL | 0 | No concerns | Low risk | No concerns | Major concerns | No concerns | Major concerns | Low |
| INU:LAV | 0 | No concerns | Low risk | No concerns | Major concerns | No concerns | Major concerns | Low |
| INU:LAV+IMI | 0 | No concerns | Low risk | No concerns | Major concerns | No concerns | Major concerns | Low |
| INU:LH57 | 0 | No concerns | Low risk | No concerns | Major concerns | No concerns | Major concerns | Low |
| INU:LI-160 | 0 | No concerns | Low risk | No concerns | Major concerns | No concerns | Major concerns | Low |
| INU:LMF | 0 | No concerns | Low risk | No concerns | Major concerns | No concerns | Major concerns | Low |
| INU:LP299v | 0 | No concerns | Low risk | No concerns | Major concerns | No concerns | Major concerns | Low |
| INU:LS | 0 | No concerns | Low risk | No concerns | Major concerns | No concerns | Major concerns | Low |
| INU:MEL | 0 | No concerns | Low risk | No concerns | Major concerns | No concerns | Major concerns | Low |
| INU:MG | 0 | No concerns | Low risk | No concerns | Major concerns | No concerns | Major concerns | Low |
| INU:NUT | 0 | No concerns | Low risk | No concerns | Major concerns | No concerns | Major concerns | Low |
| INU:OMB | 0 | No concerns | Low risk | No concerns | Major concerns | No concerns | Major concerns | Low |
| INU:OMEGA3 | 0 | No concerns | Low risk | No concerns | Major concerns | No concerns | Major concerns | Low |
| INU:PAR | 0 | No concerns | Low risk | No concerns | Major concerns | No concerns | Major concerns | Low |
| INU:PROB | 0 | No concerns | Low risk | No concerns | Major concerns | No concerns | Major concerns | Low |
| INU:PS128 | 0 | No concerns | Low risk | No concerns | Major concerns | No concerns | Major concerns | Low |
| INU:RHO | 0 | No concerns | Low risk | No concerns | No concerns | Major concerns | Major concerns | Low |
| INU:RHO+SER | 0 | No concerns | Low risk | No concerns | No concerns | No concerns | Major concerns | Low |
| INU:SAF | 0 | No concerns | Low risk | No concerns | Major concerns | No concerns | Major concerns | Low |
| INU:SAFF | 0 | No concerns | Low risk | No concerns | Major concerns | No concerns | Major concerns | Low |
| INU:SAFF+SCU | 0 | No concerns | Low risk | No concerns | Major concerns | No concerns | Major concerns | Low |
| INU:SAMe | 0 | No concerns | Low risk | No concerns | Major concerns | No concerns | Major concerns | Low |
| INU:SAMe+HELP9 | 0 | No concerns | Low risk | No concerns | Major concerns | No concerns | Major concerns | Low |
| INU:SCU | 0 | No concerns | Low risk | No concerns | Major concerns | No concerns | Major concerns | Low |
| INU:SER | 0 | No concerns | Low risk | No concerns | Major concerns | No concerns | Major concerns | Low |
| INU:SJW | 0 | No concerns | Low risk | No concerns | Major concerns | No concerns | Major concerns | Low |
| INU:SRN | 0 | No concerns | Low risk | No concerns | Major concerns | No concerns | Major concerns | Low |
| INU:SZL | 0 | No concerns | Low risk | No concerns | Major concerns | No concerns | Major concerns | Low |
| INU:TEN | 0 | No concerns | Low risk | No concerns | No concerns | Major concerns | Major concerns | Low |
| INU:WS5570 | 0 | No concerns | Low risk | No concerns | Major concerns | No concerns | Major concerns | Low |
| INU:WS5572 | 0 | No concerns | Low risk | No concerns | Major concerns | No concerns | Major concerns | Low |
| INU:WS5573 | 0 | No concerns | Low risk | No concerns | Major concerns | No concerns | Major concerns | Low |
| INU:ZE | 0 | No concerns | Low risk | No concerns | Major concerns | No concerns | Major concerns | Low |
| INU:ZE117 | 0 | No concerns | Low risk | No concerns | Major concerns | No concerns | Major concerns | Low |
| INU:vitB | 0 | No concerns | Low risk | No concerns | Major concerns | No concerns | Major concerns | Low |
| INU:vitD | 0 | No concerns | Low risk | No concerns | Major concerns | No concerns | Major concerns | Low |
| INU:vitD3 | 0 | No concerns | Low risk | No concerns | Major concerns | No concerns | Major concerns | Low |
| INU:vitD3+CA | 0 | No concerns | Low risk | No concerns | Major concerns | No concerns | Major concerns | Low |
| INU:vitD3+FLUO | 0 | No concerns | Low risk | No concerns | Major concerns | No concerns | Major concerns | Low |
| KOIL:LAV | 0 | No concerns | Low risk | No concerns | Major concerns | No concerns | Major concerns | Low |
| KOIL:LAV+IMI | 0 | No concerns | Low risk | No concerns | Major concerns | No concerns | Major concerns | Low |
| KOIL:LH57 | 0 | No concerns | Low risk | No concerns | Major concerns | No concerns | Major concerns | Low |
| KOIL:LI-160 | 0 | No concerns | Low risk | No concerns | Major concerns | No concerns | Major concerns | Low |
| KOIL:LMF | 0 | No concerns | Low risk | Some concerns | Major concerns | No concerns | Major concerns | Very Low |
| KOIL:LP299v | 0 | No concerns | Low risk | No concerns | Major concerns | No concerns | Major concerns | Low |
| KOIL:LS | 0 | No concerns | Low risk | No concerns | Major concerns | No concerns | Major concerns | Low |
| KOIL:MEL | 0 | No concerns | Low risk | No concerns | Major concerns | No concerns | Major concerns | Low |
| KOIL:MG | 0 | No concerns | Low risk | No concerns | Major concerns | No concerns | Major concerns | Low |
| KOIL:NUT | 0 | No concerns | Low risk | No concerns | Major concerns | No concerns | Major concerns | Low |
| KOIL:OMB | 0 | No concerns | Low risk | No concerns | Major concerns | No concerns | Major concerns | Low |
| KOIL:OMEGA3 | 0 | No concerns | Low risk | No concerns | Major concerns | No concerns | Major concerns | Low |
| KOIL:PAR | 0 | No concerns | Low risk | No concerns | Major concerns | No concerns | Major concerns | Low |
| KOIL:PROB | 0 | No concerns | Low risk | No concerns | Major concerns | No concerns | Major concerns | Low |
| KOIL:PS128 | 0 | No concerns | Low risk | No concerns | Major concerns | No concerns | Major concerns | Low |
| KOIL:RHO | 0 | No concerns | Low risk | No concerns | No concerns | Some concerns | Major concerns | Low |
| KOIL:RHO+SER | 0 | No concerns | Low risk | No concerns | No concerns | No concerns | Major concerns | Low |
| KOIL:SAF | 0 | No concerns | Low risk | No concerns | Major concerns | No concerns | Major concerns | Low |
| KOIL:SAFF | 0 | No concerns | Low risk | No concerns | Major concerns | No concerns | Major concerns | Low |
| KOIL:SAFF+SCU | 0 | No concerns | Low risk | No concerns | Major concerns | No concerns | Major concerns | Low |
| KOIL:SAMe | 0 | No concerns | Low risk | No concerns | Major concerns | No concerns | Major concerns | Low |
| KOIL:SAMe+HELP9 | 0 | No concerns | Low risk | No concerns | Major concerns | No concerns | Major concerns | Low |
| KOIL:SCU | 0 | No concerns | Low risk | No concerns | Major concerns | No concerns | Major concerns | Low |
| KOIL:SER | 0 | No concerns | Low risk | No concerns | Major concerns | No concerns | Major concerns | Low |
| KOIL:SJW | 0 | No concerns | Low risk | No concerns | Major concerns | No concerns | Major concerns | Low |
| KOIL:SRN | 0 | No concerns | Low risk | No concerns | Major concerns | No concerns | Major concerns | Low |
| KOIL:SZL | 0 | No concerns | Low risk | No concerns | Major concerns | No concerns | Major concerns | Low |
| KOIL:TEN | 0 | No concerns | Low risk | No concerns | No concerns | Some concerns | Major concerns | Low |
| KOIL:WS5570 | 0 | No concerns | Low risk | No concerns | Major concerns | No concerns | Major concerns | Low |
| KOIL:WS5572 | 0 | No concerns | Low risk | No concerns | Major concerns | No concerns | Major concerns | Low |
| KOIL:WS5573 | 0 | No concerns | Low risk | No concerns | Major concerns | No concerns | Major concerns | Low |
| KOIL:ZE | 0 | No concerns | Low risk | No concerns | Major concerns | No concerns | Major concerns | Low |
| KOIL:ZE117 | 0 | No concerns | Low risk | No concerns | Major concerns | No concerns | Major concerns | Low |
| KOIL:vitB | 0 | No concerns | Low risk | No concerns | Some concerns | Some concerns | Major concerns | Low |
| KOIL:vitD | 0 | No concerns | Low risk | No concerns | Major concerns | No concerns | Major concerns | Low |
| KOIL:vitD3 | 0 | No concerns | Low risk | No concerns | Some concerns | Some concerns | Major concerns | Low |
| KOIL:vitD3+CA | 0 | No concerns | Low risk | No concerns | Major concerns | No concerns | Major concerns | Low |
| KOIL:vitD3+FLUO | 0 | No concerns | Low risk | No concerns | Major concerns | No concerns | Major concerns | Low |
| LAV:LH57 | 0 | No concerns | Low risk | No concerns | Major concerns | No concerns | Major concerns | Low |
| LAV:LI-160 | 0 | No concerns | Low risk | No concerns | Major concerns | No concerns | Major concerns | Low |
| LAV:LMF | 0 | No concerns | Low risk | No concerns | Major concerns | No concerns | Major concerns | Low |
| LAV:LP299v | 0 | No concerns | Low risk | No concerns | Major concerns | No concerns | Major concerns | Low |
| LAV:LS | 0 | No concerns | Low risk | No concerns | Major concerns | No concerns | Major concerns | Low |
| LAV:MEL | 0 | No concerns | Low risk | No concerns | Major concerns | No concerns | Major concerns | Low |
| LAV:MG | 0 | No concerns | Low risk | No concerns | Major concerns | No concerns | Major concerns | Low |
| LAV:NUT | 0 | No concerns | Low risk | Some concerns | Major concerns | No concerns | Major concerns | Very Low |
| LAV:OMB | 0 | No concerns | Low risk | No concerns | Major concerns | No concerns | Major concerns | Low |
| LAV:OMEGA3 | 0 | No concerns | Low risk | No concerns | Major concerns | No concerns | Major concerns | Low |
| LAV:PAR | 0 | No concerns | Low risk | No concerns | Major concerns | No concerns | Major concerns | Low |
| LAV:PROB | 0 | No concerns | Low risk | No concerns | Major concerns | No concerns | Major concerns | Low |
| LAV:PLB | 0 | No concerns | Low risk | No concerns | Major concerns | No concerns | Major concerns | Low |
| LAV:PS128 | 0 | No concerns | Low risk | No concerns | Major concerns | No concerns | Major concerns | Low |
| LAV:RHO | 0 | No concerns | Low risk | No concerns | Major concerns | No concerns | Major concerns | Low |
| LAV:RHO+SER | 0 | No concerns | Low risk | No concerns | Major concerns | No concerns | Major concerns | Low |
| LAV:SAF | 0 | No concerns | Low risk | No concerns | Major concerns | No concerns | Major concerns | Low |
| LAV:SAFF | 0 | No concerns | Low risk | No concerns | Major concerns | No concerns | Major concerns | Low |
| LAV:SAFF+SCU | 0 | No concerns | Low risk | No concerns | Major concerns | No concerns | Major concerns | Low |
| LAV:SAMe | 0 | No concerns | Low risk | No concerns | Major concerns | No concerns | Major concerns | Low |
| LAV:SAMe+HELP9 | 0 | No concerns | Low risk | No concerns | Some concerns | Some concerns | Major concerns | Low |
| LAV:SCU | 0 | No concerns | Low risk | No concerns | Major concerns | No concerns | Major concerns | Low |
| LAV:SER | 0 | No concerns | Low risk | No concerns | Major concerns | No concerns | Major concerns | Low |
| LAV:SJW | 0 | No concerns | Low risk | No concerns | Major concerns | No concerns | Major concerns | Low |
| LAV:SRN | 0 | No concerns | Low risk | No concerns | Major concerns | No concerns | Major concerns | Low |
| LAV:SZL | 0 | No concerns | Low risk | No concerns | Major concerns | No concerns | Major concerns | Low |
| LAV:TEN | 0 | No concerns | Low risk | No concerns | Major concerns | No concerns | Major concerns | Low |
| LAV:WS5570 | 0 | No concerns | Low risk | Some concerns | Major concerns | No concerns | Major concerns | Very Low |
| LAV:WS5572 | 0 | No concerns | Low risk | No concerns | Major concerns | No concerns | Major concerns | Low |
| LAV:WS5573 | 0 | No concerns | Low risk | No concerns | Major concerns | No concerns | Major concerns | Low |
| LAV:ZE | 0 | No concerns | Low risk | No concerns | Major concerns | No concerns | Major concerns | Low |
| LAV:ZE117 | 0 | No concerns | Low risk | No concerns | Major concerns | No concerns | Major concerns | Low |
| LAV:vitB | 0 | No concerns | Low risk | No concerns | Major concerns | No concerns | Major concerns | Low |
| LAV:vitD | 0 | No concerns | Low risk | No concerns | Major concerns | No concerns | Major concerns | Low |
| LAV:vitD3 | 0 | No concerns | Low risk | No concerns | Major concerns | No concerns | Major concerns | Low |
| LAV:vitD3+CA | 0 | No concerns | Low risk | No concerns | Major concerns | No concerns | Major concerns | Low |
| LAV:vitD3+FLUO | 0 | No concerns | Low risk | No concerns | Major concerns | No concerns | Major concerns | Low |
| LAV+IMI:LH57 | 0 | No concerns | Low risk | No concerns | Major concerns | No concerns | Major concerns | Low |
| LAV+IMI:LI-160 | 0 | No concerns | Low risk | No concerns | Major concerns | No concerns | Major concerns | Low |
| LAV+IMI:LMF | 0 | No concerns | Low risk | No concerns | Major concerns | No concerns | Major concerns | Low |
| LAV+IMI:LP299v | 0 | No concerns | Low risk | No concerns | Major concerns | No concerns | Major concerns | Low |
| LAV+IMI:LS | 0 | No concerns | Low risk | No concerns | Major concerns | No concerns | Major concerns | Low |
| LAV+IMI:MEL | 0 | No concerns | Low risk | No concerns | Major concerns | No concerns | Major concerns | Low |
| LAV+IMI:MG | 0 | No concerns | Low risk | No concerns | Major concerns | No concerns | Major concerns | Low |
| LAV+IMI:NUT | 0 | No concerns | Low risk | No concerns | Major concerns | No concerns | Major concerns | Low |
| LAV+IMI:OMB | 0 | No concerns | Low risk | No concerns | Major concerns | No concerns | Major concerns | Low |
| LAV+IMI:OMEGA3 | 0 | No concerns | Low risk | No concerns | Some concerns | Some concerns | Major concerns | Low |
| LAV+IMI:PAR | 0 | No concerns | Low risk | No concerns | Major concerns | No concerns | Major concerns | Low |
| LAV+IMI:PROB | 0 | No concerns | Low risk | No concerns | Major concerns | No concerns | Major concerns | Low |
| LAV+IMI:PLB | 0 | No concerns | Low risk | No concerns | Some concerns | Some concerns | Major concerns | Low |
| LAV+IMI:PS128 | 0 | No concerns | Low risk | No concerns | Some concerns | Some concerns | Major concerns | Low |
| LAV+IMI:RHO | 0 | No concerns | Low risk | No concerns | Major concerns | No concerns | Major concerns | Low |
| LAV+IMI:RHO+SER | 0 | No concerns | Low risk | No concerns | Major concerns | No concerns | Major concerns | Low |
| LAV+IMI:SAF | 0 | No concerns | Low risk | No concerns | Major concerns | No concerns | Major concerns | Low |
| LAV+IMI:SAFF | 0 | No concerns | Low risk | No concerns | Major concerns | No concerns | Major concerns | Low |
| LAV+IMI:SAFF+SCU | 0 | No concerns | Low risk | No concerns | Major concerns | No concerns | Major concerns | Low |
| LAV+IMI:SAMe | 0 | No concerns | Low risk | No concerns | Major concerns | No concerns | Major concerns | Low |
| LAV+IMI:SAMe+HELP9 | 0 | No concerns | Low risk | No concerns | No concerns | Major concerns | Major concerns | Low |
| LAV+IMI:SCU | 0 | No concerns | Low risk | No concerns | Major concerns | No concerns | Major concerns | Low |
| LAV+IMI:SER | 0 | No concerns | Low risk | No concerns | Major concerns | No concerns | Major concerns | Low |
| LAV+IMI:SJW | 0 | No concerns | Low risk | No concerns | Major concerns | No concerns | Major concerns | Low |
| LAV+IMI:SRN | 0 | No concerns | Low risk | No concerns | Major concerns | No concerns | Major concerns | Low |
| LAV+IMI:SZL | 0 | No concerns | Low risk | No concerns | Major concerns | No concerns | Major concerns | Low |
| LAV+IMI:TEN | 0 | No concerns | Low risk | No concerns | Major concerns | No concerns | Major concerns | Low |
| LAV+IMI:WS5570 | 0 | No concerns | Low risk | No concerns | Major concerns | No concerns | Major concerns | Low |
| LAV+IMI:WS5572 | 0 | No concerns | Low risk | No concerns | Major concerns | No concerns | Major concerns | Low |
| LAV+IMI:WS5573 | 0 | No concerns | Low risk | No concerns | Major concerns | No concerns | Major concerns | Low |
| LAV+IMI:ZE | 0 | No concerns | Low risk | No concerns | Major concerns | No concerns | Major concerns | Low |
| LAV+IMI:ZE117 | 0 | No concerns | Low risk | No concerns | Major concerns | No concerns | Major concerns | Low |
| LAV+IMI:vitB | 0 | No concerns | Low risk | No concerns | Major concerns | No concerns | Major concerns | Low |
| LAV+IMI:vitD | 0 | No concerns | Low risk | No concerns | Major concerns | No concerns | Major concerns | Low |
| LAV+IMI:vitD3 | 0 | No concerns | Low risk | No concerns | Major concerns | No concerns | Major concerns | Low |
| LAV+IMI:vitD3+CA | 0 | No concerns | Low risk | No concerns | Major concerns | No concerns | Major concerns | Low |
| LAV+IMI:vitD3+FLUO | 0 | No concerns | Low risk | No concerns | Major concerns | No concerns | Major concerns | Low |
| LH57:LI-160 | 0 | No concerns | Low risk | No concerns | Major concerns | No concerns | Major concerns | Low |
| LH57:LMF | 0 | No concerns | Low risk | No concerns | Major concerns | No concerns | Major concerns | Low |
| LH57:LP299v | 0 | No concerns | Low risk | No concerns | Major concerns | No concerns | Major concerns | Low |
| LH57:LS | 0 | No concerns | Low risk | No concerns | Major concerns | No concerns | Major concerns | Low |
| LH57:MEL | 0 | No concerns | Low risk | No concerns | Major concerns | No concerns | Major concerns | Low |
| LH57:MG | 0 | No concerns | Low risk | No concerns | Major concerns | No concerns | Major concerns | Low |
| LH57:NUT | 0 | No concerns | Low risk | No concerns | Major concerns | No concerns | Major concerns | Low |
| LH57:OMB | 0 | No concerns | Low risk | No concerns | Major concerns | No concerns | Major concerns | Low |
| LH57:OMEGA3 | 0 | No concerns | Low risk | No concerns | Major concerns | No concerns | Major concerns | Low |
| LH57:PAR | 0 | No concerns | Low risk | No concerns | Major concerns | No concerns | Major concerns | Low |
| LH57:PROB | 0 | No concerns | Low risk | No concerns | Major concerns | No concerns | Major concerns | Low |
| LH57:PLB | 0 | No concerns | Low risk | No concerns | Major concerns | No concerns | Major concerns | Low |
| LH57:PS128 | 0 | No concerns | Low risk | No concerns | Major concerns | No concerns | Major concerns | Low |
| LH57:RHO | 0 | No concerns | Low risk | No concerns | No concerns | Some concerns | Major concerns | Low |
| LH57:RHO+SER | 0 | No concerns | Low risk | No concerns | No concerns | No concerns | Major concerns | Low |
| LH57:SAF | 0 | No concerns | Low risk | No concerns | Major concerns | No concerns | Major concerns | Low |
| LH57:SAFF | 0 | No concerns | Low risk | No concerns | Major concerns | No concerns | Major concerns | Low |
| LH57:SAFF+SCU | 0 | No concerns | Low risk | No concerns | Major concerns | No concerns | Major concerns | Low |
| LH57:SAMe | 0 | No concerns | Low risk | No concerns | Major concerns | No concerns | Major concerns | Low |
| LH57:SAMe+HELP9 | 0 | No concerns | Low risk | No concerns | Major concerns | No concerns | Major concerns | Low |
| LH57:SCU | 0 | No concerns | Low risk | No concerns | Major concerns | No concerns | Major concerns | Low |
| LH57:SER | 0 | No concerns | Low risk | No concerns | Major concerns | No concerns | Major concerns | Low |
| LH57:SJW | 0 | No concerns | Low risk | No concerns | Major concerns | No concerns | Major concerns | Low |
| LH57:SRN | 0 | No concerns | Low risk | No concerns | Major concerns | No concerns | Major concerns | Low |
| LH57:SZL | 0 | No concerns | Low risk | No concerns | Major concerns | No concerns | Major concerns | Low |
| LH57:TEN | 0 | No concerns | Low risk | No concerns | No concerns | Major concerns | Major concerns | Low |
| LH57:WS5570 | 0 | No concerns | Low risk | No concerns | Major concerns | No concerns | Major concerns | Low |
| LH57:WS5572 | 0 | No concerns | Low risk | No concerns | Major concerns | No concerns | Major concerns | Low |
| LH57:WS5573 | 0 | No concerns | Low risk | No concerns | Major concerns | No concerns | Major concerns | Low |
| LH57:ZE | 0 | No concerns | Low risk | No concerns | Major concerns | No concerns | Major concerns | Low |
| LH57:ZE117 | 0 | No concerns | Low risk | No concerns | Major concerns | No concerns | Major concerns | Low |
| LH57:vitB | 0 | No concerns | Low risk | No concerns | Some concerns | Some concerns | Major concerns | Low |
| LH57:vitD | 0 | No concerns | Low risk | No concerns | Major concerns | No concerns | Major concerns | Low |
| LH57:vitD3 | 0 | No concerns | Low risk | No concerns | Major concerns | No concerns | Major concerns | Low |
| LH57:vitD3+CA | 0 | No concerns | Low risk | No concerns | Major concerns | No concerns | Major concerns | Low |
| LH57:vitD3+FLUO | 0 | No concerns | Low risk | No concerns | Major concerns | No concerns | Major concerns | Low |
| LI-160:LMF | 0 | No concerns | Low risk | No concerns | Major concerns | No concerns | Major concerns | Low |
| LI-160:LP299v | 0 | No concerns | Low risk | No concerns | Major concerns | No concerns | Major concerns | Low |
| LI-160:LS | 0 | No concerns | Low risk | No concerns | Major concerns | No concerns | Major concerns | Low |
| LI-160:MEL | 0 | No concerns | Low risk | No concerns | Major concerns | No concerns | Major concerns | Low |
| LI-160:MG | 0 | No concerns | Low risk | No concerns | Major concerns | No concerns | Major concerns | Low |
| LI-160:NUT | 0 | No concerns | Low risk | No concerns | Major concerns | No concerns | Major concerns | Low |
| LI-160:OMB | 0 | No concerns | Low risk | No concerns | Major concerns | No concerns | Major concerns | Low |
| LI-160:OMEGA3 | 0 | No concerns | Low risk | No concerns | Major concerns | No concerns | Major concerns | Low |
| LI-160:PAR | 0 | No concerns | Low risk | No concerns | Major concerns | No concerns | Major concerns | Low |
| LI-160:PROB | 0 | No concerns | Low risk | No concerns | Major concerns | No concerns | Major concerns | Low |
| LI-160:PS128 | 0 | No concerns | Low risk | No concerns | Major concerns | No concerns | Major concerns | Low |
| LI-160:RHO | 0 | No concerns | Low risk | No concerns | No concerns | Major concerns | Major concerns | Low |
| LI-160:RHO+SER | 0 | No concerns | Low risk | No concerns | No concerns | No concerns | Major concerns | Low |
| LI-160:SAF | 0 | No concerns | Low risk | No concerns | Major concerns | No concerns | Major concerns | Low |
| LI-160:SAFF | 0 | No concerns | Low risk | No concerns | Major concerns | No concerns | Major concerns | Low |
| LI-160:SAFF+SCU | 0 | No concerns | Low risk | No concerns | Major concerns | No concerns | Major concerns | Low |
| LI-160:SAMe | 0 | No concerns | Low risk | No concerns | Major concerns | No concerns | Major concerns | Low |
| LI-160:SAMe+HELP9 | 0 | No concerns | Low risk | No concerns | Major concerns | No concerns | Major concerns | Low |
| LI-160:SCU | 0 | No concerns | Low risk | No concerns | Major concerns | No concerns | Major concerns | Low |
| LI-160:SJW | 0 | No concerns | Low risk | No concerns | Major concerns | No concerns | Major concerns | Low |
| LI-160:SRN | 0 | No concerns | Low risk | No concerns | Major concerns | No concerns | Major concerns | Low |
| LI-160:SZL | 0 | No concerns | Low risk | No concerns | Major concerns | No concerns | Major concerns | Low |
| LI-160:TEN | 0 | No concerns | Low risk | No concerns | No concerns | Major concerns | Major concerns | Low |
| LI-160:WS5570 | 0 | No concerns | Low risk | No concerns | Major concerns | No concerns | Major concerns | Low |
| LI-160:WS5572 | 0 | No concerns | Low risk | No concerns | Major concerns | No concerns | Major concerns | Low |
| LI-160:WS5573 | 0 | No concerns | Low risk | No concerns | Major concerns | No concerns | Major concerns | Low |
| LI-160:ZE | 0 | No concerns | Low risk | No concerns | Major concerns | No concerns | Major concerns | Low |
| LI-160:ZE117 | 0 | No concerns | Low risk | No concerns | Major concerns | No concerns | Major concerns | Low |
| LI-160:vitB | 0 | No concerns | Low risk | No concerns | Some concerns | Some concerns | Major concerns | Low |
| LI-160:vitD | 0 | No concerns | Low risk | No concerns | Major concerns | No concerns | Major concerns | Low |
| LI-160:vitD3 | 0 | No concerns | Low risk | No concerns | Major concerns | No concerns | Major concerns | Low |
| LI-160:vitD3+CA | 0 | No concerns | Low risk | Some concerns | Major concerns | No concerns | Major concerns | Very Low |
| LI-160:vitD3+FLUO | 0 | No concerns | Low risk | No concerns | Major concerns | No concerns | Major concerns | Low |
| LMF:LP299v | 0 | No concerns | Low risk | No concerns | Major concerns | No concerns | Major concerns | Low |
| LMF:LS | 0 | No concerns | Low risk | No concerns | Major concerns | No concerns | Major concerns | Low |
| LMF:MEL | 0 | No concerns | Low risk | No concerns | Major concerns | No concerns | Major concerns | Low |
| LMF:MG | 0 | No concerns | Low risk | No concerns | Major concerns | No concerns | Major concerns | Low |
| LMF:NUT | 0 | No concerns | Low risk | No concerns | Major concerns | No concerns | Major concerns | Low |
| LMF:OMB | 0 | No concerns | Low risk | No concerns | Major concerns | No concerns | Major concerns | Low |
| LMF:OMEGA3 | 0 | No concerns | Low risk | No concerns | Major concerns | No concerns | Major concerns | Low |
| LMF:PAR | 0 | No concerns | Low risk | No concerns | Major concerns | No concerns | Major concerns | Low |
| LMF:PROB | 0 | No concerns | Low risk | No concerns | Major concerns | No concerns | Major concerns | Low |
| LMF:PS128 | 0 | No concerns | Low risk | No concerns | Major concerns | No concerns | Major concerns | Low |
| LMF:RHO | 0 | No concerns | Low risk | No concerns | No concerns | Some concerns | Major concerns | Low |
| LMF:RHO+SER | 0 | No concerns | Low risk | No concerns | No concerns | No concerns | Major concerns | Low |
| LMF:SAF | 0 | No concerns | Low risk | No concerns | Major concerns | No concerns | Major concerns | Low |
| LMF:SAFF | 0 | No concerns | Low risk | No concerns | Major concerns | No concerns | Major concerns | Low |
| LMF:SAFF+SCU | 0 | No concerns | Low risk | No concerns | Major concerns | No concerns | Major concerns | Low |
| LMF:SAMe | 0 | No concerns | Low risk | No concerns | Major concerns | No concerns | Major concerns | Low |
| LMF:SAMe+HELP9 | 0 | No concerns | Low risk | No concerns | Major concerns | No concerns | Major concerns | Low |
| LMF:SCU | 0 | No concerns | Low risk | No concerns | Major concerns | No concerns | Major concerns | Low |
| LMF:SER | 0 | No concerns | Low risk | No concerns | Major concerns | No concerns | Major concerns | Low |
| LMF:SJW | 0 | No concerns | Low risk | No concerns | Major concerns | No concerns | Major concerns | Low |
| LMF:SRN | 0 | No concerns | Low risk | No concerns | Major concerns | No concerns | Major concerns | Low |
| LMF:SZL | 0 | No concerns | Low risk | No concerns | Major concerns | No concerns | Major concerns | Low |
| LMF:TEN | 0 | No concerns | Low risk | No concerns | No concerns | Some concerns | Major concerns | Low |
| LMF:WS5570 | 0 | No concerns | Low risk | No concerns | Major concerns | No concerns | Major concerns | Low |
| LMF:WS5572 | 0 | No concerns | Low risk | No concerns | Major concerns | No concerns | Major concerns | Low |
| LMF:WS5573 | 0 | No concerns | Low risk | No concerns | Major concerns | No concerns | Major concerns | Low |
| LMF:ZE | 0 | No concerns | Low risk | No concerns | Major concerns | No concerns | Major concerns | Low |
| LMF:ZE117 | 0 | No concerns | Low risk | No concerns | Major concerns | No concerns | Major concerns | Low |
| LMF:vitB | 0 | No concerns | Low risk | No concerns | Some concerns | Some concerns | Major concerns | Low |
| LMF:vitD | 0 | No concerns | Low risk | No concerns | Major concerns | No concerns | Major concerns | Low |
| LMF:vitD3 | 0 | No concerns | Low risk | No concerns | Some concerns | Some concerns | Major concerns | Low |
| LMF:vitD3+CA | 0 | No concerns | Low risk | No concerns | Major concerns | No concerns | Major concerns | Low |
| LMF:vitD3+FLUO | 0 | No concerns | Low risk | No concerns | Major concerns | No concerns | Major concerns | Low |
| LP299v:LS | 0 | No concerns | Low risk | No concerns | Major concerns | No concerns | Major concerns | Low |
| LP299v:MEL | 0 | No concerns | Low risk | No concerns | Major concerns | No concerns | Major concerns | Low |
| LP299v:MG | 0 | No concerns | Low risk | No concerns | Major concerns | No concerns | Major concerns | Low |
| LP299v:NUT | 0 | No concerns | Low risk | No concerns | Major concerns | No concerns | Major concerns | Low |
| LP299v:OMB | 0 | No concerns | Low risk | No concerns | Major concerns | No concerns | Major concerns | Low |
| LP299v:OMEGA3 | 0 | No concerns | Low risk | No concerns | Major concerns | No concerns | Major concerns | Low |
| LP299v:PAR | 0 | No concerns | Low risk | No concerns | Major concerns | No concerns | Major concerns | Low |
| LP299v:PROB | 0 | No concerns | Low risk | No concerns | Major concerns | No concerns | Major concerns | Low |
| LP299v:PS128 | 0 | No concerns | Low risk | Some concerns | Major concerns | No concerns | Major concerns | Very Low |
| LP299v:RHO | 0 | No concerns | Low risk | No concerns | Major concerns | No concerns | Major concerns | Low |
| LP299v:RHO+SER | 0 | No concerns | Low risk | No concerns | No concerns | No concerns | Major concerns | Low |
| LP299v:SAF | 0 | No concerns | Low risk | No concerns | Major concerns | No concerns | Major concerns | Low |
| LP299v:SAFF | 0 | No concerns | Low risk | No concerns | Major concerns | No concerns | Major concerns | Low |
| LP299v:SAFF+SCU | 0 | No concerns | Low risk | No concerns | Major concerns | No concerns | Major concerns | Low |
| LP299v:SAMe | 0 | No concerns | Low risk | No concerns | Major concerns | No concerns | Major concerns | Low |
| LP299v:SAMe+HELP9 | 0 | No concerns | Low risk | No concerns | Major concerns | No concerns | Major concerns | Low |
| LP299v:SCU | 0 | No concerns | Low risk | No concerns | Major concerns | No concerns | Major concerns | Low |
| LP299v:SER | 0 | No concerns | Low risk | No concerns | Major concerns | No concerns | Major concerns | Low |
| LP299v:SJW | 0 | No concerns | Low risk | No concerns | Major concerns | No concerns | Major concerns | Low |
| LP299v:SRN | 0 | No concerns | Low risk | No concerns | Major concerns | No concerns | Major concerns | Low |
| LP299v:SZL | 0 | No concerns | Low risk | No concerns | Major concerns | No concerns | Major concerns | Low |
| LP299v:TEN | 0 | No concerns | Low risk | No concerns | Major concerns | No concerns | Major concerns | Low |
| LP299v:WS5570 | 0 | No concerns | Low risk | No concerns | Major concerns | No concerns | Major concerns | Low |
| LP299v:WS5572 | 0 | No concerns | Low risk | No concerns | Major concerns | No concerns | Major concerns | Low |
| LP299v:WS5573 | 0 | No concerns | Low risk | No concerns | Major concerns | No concerns | Major concerns | Low |
| LP299v:ZE | 0 | No concerns | Low risk | No concerns | Major concerns | No concerns | Major concerns | Low |
| LP299v:ZE117 | 0 | No concerns | Low risk | No concerns | Major concerns | No concerns | Major concerns | Low |
| LP299v:vitB | 0 | No concerns | Low risk | No concerns | Major concerns | No concerns | Major concerns | Low |
| LP299v:vitD | 0 | No concerns | Low risk | No concerns | Major concerns | No concerns | Major concerns | Low |
| LP299v:vitD3 | 0 | No concerns | Low risk | No concerns | Major concerns | No concerns | Major concerns | Low |
| LP299v:vitD3+CA | 0 | No concerns | Low risk | No concerns | Major concerns | No concerns | Major concerns | Low |
| LP299v:vitD3+FLUO | 0 | No concerns | Low risk | No concerns | Major concerns | No concerns | Major concerns | Low |
| LS:MEL | 0 | No concerns | Low risk | No concerns | Major concerns | No concerns | Major concerns | Low |
| LS:MG | 0 | No concerns | Low risk | No concerns | Major concerns | No concerns | Major concerns | Low |
| LS:NUT | 0 | No concerns | Low risk | No concerns | Major concerns | No concerns | Major concerns | Low |
| LS:OMB | 0 | No concerns | Low risk | No concerns | Major concerns | No concerns | Major concerns | Low |
| LS:OMEGA3 | 0 | No concerns | Low risk | No concerns | Major concerns | No concerns | Major concerns | Low |
| LS:PAR | 0 | No concerns | Low risk | No concerns | Major concerns | No concerns | Major concerns | Low |
| LS:PROB | 0 | No concerns | Low risk | No concerns | Major concerns | No concerns | Major concerns | Low |
| LS:PS128 | 0 | No concerns | Low risk | No concerns | Major concerns | No concerns | Major concerns | Low |
| LS:RHO | 0 | No concerns | Low risk | No concerns | Some concerns | Some concerns | Major concerns | Low |
| LS:RHO+SER | 0 | No concerns | Low risk | No concerns | No concerns | No concerns | Major concerns | Low |
| LS:SAF | 0 | No concerns | Low risk | No concerns | Major concerns | No concerns | Major concerns | Low |
| LS:SAFF | 0 | No concerns | Low risk | No concerns | Major concerns | No concerns | Major concerns | Low |
| LS:SAFF+SCU | 0 | No concerns | Low risk | No concerns | Major concerns | No concerns | Major concerns | Low |
| LS:SAMe | 0 | No concerns | Low risk | No concerns | Major concerns | No concerns | Major concerns | Low |
| LS:SAMe+HELP9 | 0 | No concerns | Low risk | No concerns | Major concerns | No concerns | Major concerns | Low |
| LS:SCU | 0 | No concerns | Low risk | No concerns | Major concerns | No concerns | Major concerns | Low |
| LS:SER | 0 | No concerns | Low risk | No concerns | Major concerns | No concerns | Major concerns | Low |
| LS:SJW | 0 | No concerns | Low risk | No concerns | Major concerns | No concerns | Major concerns | Low |
| LS:SRN | 0 | No concerns | Low risk | No concerns | Major concerns | No concerns | Major concerns | Low |
| LS:SZL | 0 | No concerns | Low risk | No concerns | Major concerns | No concerns | Major concerns | Low |
| LS:TEN | 0 | No concerns | Low risk | No concerns | Some concerns | Some concerns | Major concerns | Low |
| LS:WS5570 | 0 | No concerns | Low risk | No concerns | Major concerns | No concerns | Major concerns | Low |
| LS:WS5572 | 0 | No concerns | Low risk | No concerns | Major concerns | No concerns | Major concerns | Low |
| LS:WS5573 | 0 | No concerns | Low risk | No concerns | Major concerns | No concerns | Major concerns | Low |
| LS:ZE | 0 | No concerns | Low risk | No concerns | Major concerns | No concerns | Major concerns | Low |
| LS:ZE117 | 0 | No concerns | Low risk | No concerns | Major concerns | No concerns | Major concerns | Low |
| LS:vitB | 0 | No concerns | Low risk | No concerns | Major concerns | No concerns | Major concerns | Low |
| LS:vitD | 0 | No concerns | Low risk | No concerns | Major concerns | No concerns | Major concerns | Low |
| LS:vitD3 | 0 | No concerns | Low risk | No concerns | Major concerns | No concerns | Major concerns | Low |
| LS:vitD3+CA | 0 | No concerns | Low risk | No concerns | Major concerns | No concerns | Major concerns | Low |
| LS:vitD3+FLUO | 0 | No concerns | Low risk | No concerns | Major concerns | No concerns | Major concerns | Low |
| MEL:MG | 0 | No concerns | Low risk | No concerns | Major concerns | No concerns | Major concerns | Low |
| MEL:NUT | 0 | No concerns | Low risk | No concerns | Major concerns | No concerns | Major concerns | Low |
| MEL:OMB | 0 | No concerns | Low risk | No concerns | Major concerns | No concerns | Major concerns | Low |
| MEL:OMEGA3 | 0 | No concerns | Low risk | No concerns | Major concerns | No concerns | Major concerns | Low |
| MEL:PAR | 0 | No concerns | Low risk | No concerns | Major concerns | No concerns | Major concerns | Low |
| MEL:PROB | 0 | No concerns | Low risk | No concerns | Major concerns | No concerns | Major concerns | Low |
| MEL:PS128 | 0 | No concerns | Low risk | No concerns | Major concerns | No concerns | Major concerns | Low |
| MEL:RHO | 0 | No concerns | Low risk | No concerns | Major concerns | No concerns | Major concerns | Low |
| MEL:RHO+SER | 0 | No concerns | Low risk | No concerns | No concerns | No concerns | Major concerns | Low |
| MEL:SAF | 0 | No concerns | Low risk | No concerns | Major concerns | No concerns | Major concerns | Low |
| MEL:SAFF | 0 | No concerns | Low risk | No concerns | Major concerns | No concerns | Major concerns | Low |
| MEL:SAFF+SCU | 0 | No concerns | Low risk | No concerns | Major concerns | No concerns | Major concerns | Low |
| MEL:SAMe | 0 | No concerns | Low risk | No concerns | Major concerns | No concerns | Major concerns | Low |
| MEL:SAMe+HELP9 | 0 | No concerns | Low risk | No concerns | Major concerns | No concerns | Major concerns | Low |
| MEL:SCU | 0 | No concerns | Low risk | No concerns | Major concerns | No concerns | Major concerns | Low |
| MEL:SER | 0 | No concerns | Low risk | No concerns | Major concerns | No concerns | Major concerns | Low |
| MEL:SJW | 0 | No concerns | Low risk | No concerns | Major concerns | No concerns | Major concerns | Low |
| MEL:SRN | 0 | No concerns | Low risk | No concerns | Major concerns | No concerns | Major concerns | Low |
| MEL:SZL | 0 | No concerns | Low risk | No concerns | Major concerns | No concerns | Major concerns | Low |
| MEL:TEN | 0 | No concerns | Low risk | No concerns | Major concerns | No concerns | Major concerns | Low |
| MEL:WS5570 | 0 | No concerns | Low risk | No concerns | Major concerns | No concerns | Major concerns | Low |
| MEL:WS5572 | 0 | No concerns | Low risk | No concerns | Major concerns | No concerns | Major concerns | Low |
| MEL:WS5573 | 0 | No concerns | Low risk | No concerns | Major concerns | No concerns | Major concerns | Low |
| MEL:ZE | 0 | No concerns | Low risk | No concerns | Major concerns | No concerns | Major concerns | Low |
| MEL:ZE117 | 0 | No concerns | Low risk | No concerns | Major concerns | No concerns | Major concerns | Low |
| MEL:vitB | 0 | No concerns | Low risk | No concerns | Major concerns | No concerns | Major concerns | Low |
| MEL:vitD | 0 | No concerns | Low risk | No concerns | Major concerns | No concerns | Major concerns | Low |
| MEL:vitD3 | 0 | No concerns | Low risk | No concerns | Major concerns | No concerns | Major concerns | Low |
| MEL:vitD3+CA | 0 | No concerns | Low risk | No concerns | Major concerns | No concerns | Major concerns | Low |
| MEL:vitD3+FLUO | 0 | No concerns | Low risk | No concerns | Major concerns | No concerns | Major concerns | Low |
| MG:NUT | 0 | No concerns | Low risk | No concerns | Major concerns | No concerns | Major concerns | Low |
| MG:OMB | 0 | No concerns | Low risk | No concerns | Major concerns | No concerns | Major concerns | Low |
| MG:OMEGA3 | 0 | No concerns | Low risk | No concerns | Major concerns | No concerns | Major concerns | Low |
| MG:PAR | 0 | No concerns | Low risk | No concerns | Major concerns | No concerns | Major concerns | Low |
| MG:PROB | 0 | No concerns | Low risk | No concerns | Major concerns | No concerns | Major concerns | Low |
| MG:PS128 | 0 | No concerns | Low risk | No concerns | Major concerns | No concerns | Major concerns | Low |
| MG:RHO | 0 | No concerns | Low risk | No concerns | Some concerns | Some concerns | Major concerns | Low |
| MG:RHO+SER | 0 | No concerns | Low risk | No concerns | No concerns | No concerns | Major concerns | Low |
| MG:SAF | 0 | No concerns | Low risk | No concerns | Major concerns | No concerns | Major concerns | Low |
| MG:SAFF | 0 | No concerns | Low risk | No concerns | Major concerns | No concerns | Major concerns | Low |
| MG:SAFF+SCU | 0 | No concerns | Low risk | No concerns | Major concerns | No concerns | Major concerns | Low |
| MG:SAMe | 0 | No concerns | Low risk | No concerns | Major concerns | No concerns | Major concerns | Low |
| MG:SAMe+HELP9 | 0 | No concerns | Low risk | No concerns | Major concerns | No concerns | Major concerns | Low |
| MG:SCU | 0 | No concerns | Low risk | No concerns | Major concerns | No concerns | Major concerns | Low |
| MG:SER | 0 | No concerns | Low risk | No concerns | Major concerns | No concerns | Major concerns | Low |
| MG:SJW | 0 | No concerns | Low risk | No concerns | Major concerns | No concerns | Major concerns | Low |
| MG:SRN | 0 | No concerns | Low risk | No concerns | Major concerns | No concerns | Major concerns | Low |
| MG:SZL | 0 | No concerns | Low risk | No concerns | Major concerns | No concerns | Major concerns | Low |
| MG:TEN | 0 | No concerns | Low risk | No concerns | Major concerns | No concerns | Major concerns | Low |
| MG:WS5570 | 0 | No concerns | Low risk | No concerns | Major concerns | No concerns | Major concerns | Low |
| MG:WS5572 | 0 | No concerns | Low risk | No concerns | Major concerns | No concerns | Major concerns | Low |
| MG:WS5573 | 0 | No concerns | Low risk | No concerns | Major concerns | No concerns | Major concerns | Low |
| MG:ZE | 0 | No concerns | Low risk | No concerns | Major concerns | No concerns | Major concerns | Low |
| MG:ZE117 | 0 | No concerns | Low risk | No concerns | Major concerns | No concerns | Major concerns | Low |
| MG:vitB | 0 | No concerns | Low risk | No concerns | Major concerns | No concerns | Major concerns | Low |
| MG:vitD | 0 | No concerns | Low risk | No concerns | Major concerns | No concerns | Major concerns | Low |
| MG:vitD3 | 0 | No concerns | Low risk | No concerns | Major concerns | No concerns | Major concerns | Low |
| MG:vitD3+CA | 0 | No concerns | Low risk | No concerns | Major concerns | No concerns | Major concerns | Low |
| MG:vitD3+FLUO | 0 | No concerns | Low risk | No concerns | Major concerns | No concerns | Major concerns | Low |
| NUT:OMB | 0 | No concerns | Low risk | No concerns | Major concerns | No concerns | Major concerns | Low |
| NUT:OMEGA3 | 0 | No concerns | Low risk | No concerns | Major concerns | No concerns | Major concerns | Low |
| NUT:PAR | 0 | No concerns | Low risk | No concerns | Major concerns | No concerns | Major concerns | Low |
| NUT:PROB | 0 | No concerns | Low risk | No concerns | Major concerns | No concerns | Major concerns | Low |
| NUT:PS128 | 0 | No concerns | Low risk | No concerns | Major concerns | No concerns | Major concerns | Low |
| NUT:RHO | 0 | No concerns | Low risk | No concerns | No concerns | Some concerns | Major concerns | Low |
| NUT:RHO+SER | 0 | No concerns | Low risk | No concerns | No concerns | No concerns | Major concerns | Low |
| NUT:SAF | 0 | No concerns | Low risk | No concerns | Major concerns | No concerns | Major concerns | Low |
| NUT:SAFF | 0 | No concerns | Low risk | No concerns | Major concerns | No concerns | Major concerns | Low |
| NUT:SAFF+SCU | 0 | No concerns | Low risk | No concerns | Major concerns | No concerns | Major concerns | Low |
| NUT:SAMe | 0 | No concerns | Low risk | No concerns | Major concerns | No concerns | Major concerns | Low |
| NUT:SAMe+HELP9 | 0 | No concerns | Low risk | Some concerns | Major concerns | No concerns | Major concerns | Very Low |
| NUT:SCU | 0 | No concerns | Low risk | No concerns | Major concerns | No concerns | Major concerns | Low |
| NUT:SER | 0 | No concerns | Low risk | No concerns | Major concerns | No concerns | Major concerns | Low |
| NUT:SJW | 0 | No concerns | Low risk | No concerns | Major concerns | No concerns | Major concerns | Low |
| NUT:SRN | 0 | No concerns | Low risk | No concerns | Major concerns | No concerns | Major concerns | Low |
| NUT:SZL | 0 | No concerns | Low risk | No concerns | Major concerns | No concerns | Major concerns | Low |
| NUT:TEN | 0 | No concerns | Low risk | No concerns | No concerns | Some concerns | Major concerns | Low |
| NUT:WS5570 | 0 | No concerns | Low risk | No concerns | Major concerns | No concerns | Major concerns | Low |
| NUT:WS5572 | 0 | No concerns | Low risk | No concerns | Major concerns | No concerns | Major concerns | Low |
| NUT:WS5573 | 0 | No concerns | Low risk | No concerns | Major concerns | No concerns | Major concerns | Low |
| NUT:ZE | 0 | No concerns | Low risk | No concerns | Major concerns | No concerns | Major concerns | Low |
| NUT:ZE117 | 0 | No concerns | Low risk | No concerns | Major concerns | No concerns | Major concerns | Low |
| NUT:vitB | 0 | No concerns | Low risk | No concerns | No concerns | Major concerns | Major concerns | Low |
| NUT:vitD | 0 | No concerns | Low risk | No concerns | Major concerns | No concerns | Major concerns | Low |
| NUT:vitD3 | 0 | No concerns | Low risk | No concerns | Some concerns | Some concerns | Major concerns | Low |
| NUT:vitD3+CA | 0 | No concerns | Low risk | No concerns | Major concerns | No concerns | Major concerns | Low |
| NUT:vitD3+FLUO | 0 | No concerns | Low risk | No concerns | Major concerns | No concerns | Major concerns | Low |
| OMB:OMEGA3 | 0 | No concerns | Low risk | No concerns | Major concerns | No concerns | Major concerns | Low |
| OMB:PAR | 0 | No concerns | Low risk | No concerns | Major concerns | No concerns | Major concerns | Low |
| OMB:PROB | 0 | No concerns | Low risk | No concerns | Major concerns | No concerns | Major concerns | Low |
| OMB:PS128 | 0 | No concerns | Low risk | No concerns | Major concerns | No concerns | Major concerns | Low |
| OMB:RHO | 0 | No concerns | Low risk | No concerns | No concerns | Some concerns | Major concerns | Low |
| OMB:RHO+SER | 0 | No concerns | Low risk | No concerns | No concerns | No concerns | Major concerns | Low |
| OMB:SAF | 0 | No concerns | Low risk | No concerns | Major concerns | No concerns | Major concerns | Low |
| OMB:SAFF | 0 | No concerns | Low risk | No concerns | Major concerns | No concerns | Major concerns | Low |
| OMB:SAFF+SCU | 0 | No concerns | Low risk | No concerns | Major concerns | No concerns | Major concerns | Low |
| OMB:SAMe | 0 | No concerns | Low risk | No concerns | Major concerns | No concerns | Major concerns | Low |
| OMB:SAMe+HELP9 | 0 | No concerns | Low risk | No concerns | Major concerns | No concerns | Major concerns | Low |
| OMB:SCU | 0 | No concerns | Low risk | No concerns | Major concerns | No concerns | Major concerns | Low |
| OMB:SER | 0 | No concerns | Low risk | No concerns | Major concerns | No concerns | Major concerns | Low |
| OMB:SJW | 0 | No concerns | Low risk | No concerns | Major concerns | No concerns | Major concerns | Low |
| OMB:SRN | 0 | No concerns | Low risk | No concerns | Major concerns | No concerns | Major concerns | Low |
| OMB:SZL | 0 | No concerns | Low risk | No concerns | Major concerns | No concerns | Major concerns | Low |
| OMB:TEN | 0 | No concerns | Low risk | No concerns | No concerns | Major concerns | Major concerns | Low |
| OMB:WS5570 | 0 | No concerns | Low risk | No concerns | Major concerns | No concerns | Major concerns | Low |
| OMB:WS5572 | 0 | No concerns | Low risk | No concerns | Major concerns | No concerns | Major concerns | Low |
| OMB:WS5573 | 0 | No concerns | Low risk | No concerns | Major concerns | No concerns | Major concerns | Low |
| OMB:ZE | 0 | No concerns | Low risk | No concerns | Major concerns | No concerns | Major concerns | Low |
| OMB:ZE117 | 0 | No concerns | Low risk | No concerns | Major concerns | No concerns | Major concerns | Low |
| OMB:vitB | 0 | No concerns | Low risk | No concerns | Some concerns | Some concerns | Major concerns | Low |
| OMB:vitD | 0 | No concerns | Low risk | No concerns | Major concerns | No concerns | Major concerns | Low |
| OMB:vitD3 | 0 | No concerns | Low risk | No concerns | Major concerns | No concerns | Major concerns | Low |
| OMB:vitD3+CA | 0 | No concerns | Low risk | No concerns | Major concerns | No concerns | Major concerns | Low |
| OMB:vitD3+FLUO | 0 | No concerns | Low risk | No concerns | Major concerns | No concerns | Major concerns | Low |
| OMEGA3:PAR | 0 | No concerns | Low risk | No concerns | Major concerns | No concerns | Major concerns | Low |
| OMEGA3:PROB | 0 | No concerns | Low risk | No concerns | Major concerns | No concerns | Major concerns | Low |
| OMEGA3:PS128 | 0 | No concerns | Low risk | No concerns | Major concerns | No concerns | Major concerns | Low |
| OMEGA3:RHO | 0 | No concerns | Low risk | No concerns | No concerns | No concerns | Major concerns | Low |
| OMEGA3:RHO+SER | 0 | No concerns | Low risk | No concerns | No concerns | No concerns | Major concerns | Low |
| OMEGA3:SAF | 0 | No concerns | Low risk | No concerns | Major concerns | No concerns | Major concerns | Low |
| OMEGA3:SAFF | 0 | No concerns | Low risk | Some concerns | Some concerns | Some concerns | Major concerns | Very Low |
| OMEGA3:SAFF+SCU | 0 | No concerns | Low risk | No concerns | Major concerns | No concerns | Major concerns | Low |
| OMEGA3:SAMe | 0 | No concerns | Low risk | No concerns | Major concerns | No concerns | Major concerns | Very Low |
| OMEGA3:SAMe+HELP9 | 0 | No concerns | Low risk | No concerns | Major concerns | No concerns | Major concerns | Low |
| OMEGA3:SCU | 0 | No concerns | Low risk | No concerns | Major concerns | No concerns | Major concerns | Low |
| OMEGA3:SER | 0 | No concerns | Low risk | No concerns | Major concerns | No concerns | Major concerns | Low |
| OMEGA3:SJW | 0 | No concerns | Low risk | No concerns | Major concerns | No concerns | Major concerns | Low |
| OMEGA3:SRN | 0 | No concerns | Low risk | No concerns | Major concerns | No concerns | Major concerns | Low |
| OMEGA3:SZL | 0 | No concerns | Low risk | No concerns | Major concerns | No concerns | Major concerns | Low |
| OMEGA3:TEN | 0 | No concerns | Low risk | No concerns | No concerns | Some concerns | Major concerns | Low |
| OMEGA3:WS5570 | 0 | No concerns | Low risk | No concerns | Major concerns | No concerns | Major concerns | Low |
| OMEGA3:WS5572 | 0 | No concerns | Low risk | No concerns | Major concerns | No concerns | Major concerns | Low |
| OMEGA3:WS5573 | 0 | No concerns | Low risk | No concerns | Major concerns | No concerns | Major concerns | Low |
| OMEGA3:ZE | 0 | No concerns | Low risk | No concerns | Major concerns | No concerns | Major concerns | Low |
| OMEGA3:ZE117 | 0 | No concerns | Low risk | No concerns | Major concerns | No concerns | Major concerns | Low |
| OMEGA3:vitB | 0 | No concerns | Low risk | No concerns | No concerns | Major concerns | Major concerns | Low |
| OMEGA3:vitD | 0 | No concerns | Low risk | No concerns | Major concerns | No concerns | Major concerns | Low |
| OMEGA3:vitD3 | 0 | No concerns | Low risk | No concerns | No concerns | Major concerns | Major concerns | Low |
| OMEGA3:vitD3+CA | 0 | No concerns | Low risk | No concerns | Major concerns | No concerns | Major concerns | Low |
| OMEGA3:vitD3+FLUO | 0 | No concerns | Low risk | No concerns | Major concerns | No concerns | Major concerns | Low |
| PAR:PROB | 0 | No concerns | Low risk | No concerns | Major concerns | No concerns | Major concerns | Low |
| PAR:PLB | 0 | No concerns | Low risk | No concerns | Major concerns | No concerns | Major concerns | Low |
| PAR:PS128 | 0 | No concerns | Low risk | No concerns | Major concerns | No concerns | Major concerns | Low |
| PAR:RHO | 0 | No concerns | Low risk | No concerns | Some concerns | Some concerns | Major concerns | Low |
| PAR:RHO+SER | 0 | No concerns | Low risk | No concerns | No concerns | No concerns | Major concerns | Low |
| PAR:SAF | 0 | No concerns | Low risk | No concerns | Major concerns | No concerns | Major concerns | Low |
| PAR:SAFF | 0 | No concerns | Low risk | No concerns | Major concerns | No concerns | Major concerns | Low |
| PAR:SAFF+SCU | 0 | No concerns | Low risk | No concerns | Major concerns | No concerns | Major concerns | Low |
| PAR:SAMe | 0 | No concerns | Low risk | No concerns | Major concerns | No concerns | Major concerns | Low |
| PAR:SAMe+HELP9 | 0 | No concerns | Low risk | No concerns | Major concerns | No concerns | Major concerns | Low |
| PAR:SCU | 0 | No concerns | Low risk | Some concerns | Major concerns | No concerns | Major concerns | Very Low |
| PAR:SER | 0 | No concerns | Low risk | No concerns | Major concerns | No concerns | Major concerns | Low |
| PAR:SJW | 0 | No concerns | Low risk | No concerns | Major concerns | No concerns | Major concerns | Low |
| PAR:SRN | 0 | No concerns | Low risk | No concerns | Major concerns | No concerns | Major concerns | Low |
| PAR:SZL | 0 | No concerns | Low risk | No concerns | Major concerns | No concerns | Major concerns | Low |
| PAR:TEN | 0 | No concerns | Low risk | No concerns | Some concerns | Some concerns | Major concerns | Low |
| PAR:WS5572 | 0 | No concerns | Low risk | No concerns | Major concerns | No concerns | Major concerns | Low |
| PAR:WS5573 | 0 | No concerns | Low risk | No concerns | Major concerns | No concerns | Major concerns | Low |
| PAR:ZE | 0 | No concerns | Low risk | No concerns | Major concerns | No concerns | Major concerns | Low |
| PAR:ZE117 | 0 | No concerns | Low risk | No concerns | Major concerns | No concerns | Major concerns | Low |
| PAR:vitB | 0 | No concerns | Low risk | No concerns | Major concerns | No concerns | Major concerns | Low |
| PAR:vitD | 0 | No concerns | Low risk | No concerns | Major concerns | No concerns | Major concerns | Low |
| PAR:vitD3 | 0 | No concerns | Low risk | No concerns | Major concerns | No concerns | Major concerns | Low |
| PAR:vitD3+CA | 0 | No concerns | Low risk | No concerns | Major concerns | No concerns | Major concerns | Low |
| PAR:vitD3+FLUO | 0 | No concerns | Low risk | No concerns | Major concerns | No concerns | Major concerns | Low |
| PROB:PS128 | 0 | No concerns | Low risk | No concerns | Major concerns | No concerns | Major concerns | Low |
| PROB:RHO | 0 | No concerns | Low risk | No concerns | Major concerns | No concerns | Major concerns | Low |
| PROB:RHO+SER | 0 | No concerns | Low risk | No concerns | No concerns | No concerns | Major concerns | Low |
| PROB:SAF | 0 | No concerns | Low risk | No concerns | Major concerns | No concerns | Major concerns | Low |
| PROB:SAFF | 0 | No concerns | Low risk | No concerns | Major concerns | No concerns | Major concerns | Low |
| PROB:SAFF+SCU | 0 | No concerns | Low risk | No concerns | Major concerns | No concerns | Major concerns | Low |
| PROB:SAMe | 0 | No concerns | Low risk | No concerns | Major concerns | No concerns | Major concerns | Low |
| PROB:SAMe+HELP9 | 0 | No concerns | Low risk | No concerns | Major concerns | No concerns | Major concerns | Low |
| PROB:SCU | 0 | No concerns | Low risk | Some concerns | Major concerns | No concerns | Major concerns | Very Low |
| PROB:SER | 0 | No concerns | Low risk | No concerns | Major concerns | No concerns | Major concerns | Low |
| PROB:SJW | 0 | No concerns | Low risk | No concerns | Major concerns | No concerns | Major concerns | Low |
| PROB:SRN | 0 | No concerns | Low risk | No concerns | Major concerns | No concerns | Major concerns | Low |
| PROB:SZL | 0 | No concerns | Low risk | No concerns | Major concerns | No concerns | Major concerns | Low |
| PROB:TEN | 0 | No concerns | Low risk | No concerns | Major concerns | No concerns | Major concerns | Low |
| PROB:WS5570 | 0 | No concerns | Low risk | No concerns | Major concerns | No concerns | Major concerns | Low |
| PROB:WS5572 | 0 | No concerns | Low risk | No concerns | Major concerns | No concerns | Major concerns | Low |
| PROB:WS5573 | 0 | No concerns | Low risk | No concerns | Major concerns | No concerns | Major concerns | Low |
| PROB:ZE | 0 | No concerns | Low risk | No concerns | Major concerns | No concerns | Major concerns | Low |
| PROB:ZE117 | 0 | No concerns | Low risk | No concerns | Major concerns | No concerns | Major concerns | Low |
| PROB:vitB | 0 | No concerns | Low risk | No concerns | Major concerns | No concerns | Major concerns | Low |
| PROB:vitD | 0 | No concerns | Low risk | No concerns | Major concerns | No concerns | Major concerns | Low |
| PROB:vitD3 | 0 | No concerns | Low risk | Some concerns | Major concerns | No concerns | Major concerns | Very Low |
| PROB:vitD3+CA | 0 | No concerns | Low risk | No concerns | Major concerns | No concerns | Major concerns | Low |
| PROB:vitD3+FLUO | 0 | No concerns | Low risk | No concerns | Major concerns | No concerns | Major concerns | Low |
| PLB:SZL | 0 | No concerns | Low risk | No concerns | Major concerns | No concerns | Major concerns | Low |
| PLB:ZE | 0 | No concerns | Low risk | No concerns | Major concerns | No concerns | Major concerns | Low |
| PLB:vitD3+FLUO | 0 | No concerns | Low risk | No concerns | Major concerns | No concerns | Major concerns | Low |
| PS128:RHO | 0 | No concerns | Low risk | No concerns | No concerns | Some concerns | Major concerns | Low |
| PS128:RHO+SER | 0 | No concerns | Low risk | No concerns | No concerns | No concerns | Major concerns | Low |
| PS128:SAF | 0 | No concerns | Low risk | No concerns | Major concerns | No concerns | Major concerns | Low |
| PS128:SAFF | 0 | No concerns | Low risk | No concerns | Major concerns | No concerns | Major concerns | Low |
| PS128:SAFF+SCU | 0 | No concerns | Low risk | No concerns | Major concerns | No concerns | Major concerns | Low |
| PS128:SAMe | 0 | No concerns | Low risk | No concerns | Major concerns | No concerns | Major concerns | Low |
| PS128:SAMe+HELP9 | 0 | No concerns | Low risk | No concerns | Major concerns | No concerns | Major concerns | Low |
| PS128:SCU | 0 | No concerns | Low risk | No concerns | Major concerns | No concerns | Major concerns | Low |
| PS128:SER | 0 | No concerns | Low risk | No concerns | Major concerns | No concerns | Major concerns | Low |
| PS128:SJW | 0 | No concerns | Low risk | No concerns | Major concerns | No concerns | Major concerns | Low |
| PS128:SRN | 0 | No concerns | Low risk | No concerns | Major concerns | No concerns | Major concerns | Low |
| PS128:SZL | 0 | No concerns | Low risk | No concerns | Major concerns | No concerns | Major concerns | Low |
| PS128:TEN | 0 | No concerns | Low risk | No concerns | No concerns | Some concerns | Major concerns | Low |
| PS128:WS5570 | 0 | No concerns | Low risk | No concerns | Major concerns | No concerns | Major concerns | Low |
| PS128:WS5572 | 0 | No concerns | Low risk | No concerns | Major concerns | No concerns | Major concerns | Low |
| PS128:WS5573 | 0 | No concerns | Low risk | No concerns | Major concerns | No concerns | Major concerns | Low |
| PS128:ZE | 0 | No concerns | Low risk | No concerns | Major concerns | No concerns | Major concerns | Low |
| PS128:ZE117 | 0 | No concerns | Low risk | No concerns | Major concerns | No concerns | Major concerns | Low |
| PS128:vitB | 0 | No concerns | Low risk | Some concerns | No concerns | Major concerns | Major concerns | Very Low |
| PS128:vitD | 0 | No concerns | Low risk | No concerns | Major concerns | No concerns | Major concerns | Low |
| PS128:vitD3 | 0 | No concerns | Low risk | No concerns | Some concerns | Some concerns | Major concerns | Low |
| PS128:vitD3+CA | 0 | No concerns | Low risk | No concerns | Major concerns | No concerns | Major concerns | Low |
| PS128:vitD3+FLUO | 0 | No concerns | Low risk | No concerns | Major concerns | No concerns | Major concerns | Low |
| RHO:RHO+SER | 0 | No concerns | Low risk | No concerns | Major concerns | No concerns | Major concerns | Low |
| RHO:SAF | 0 | No concerns | Low risk | No concerns | Some concerns | Some concerns | Major concerns | Low |
| RHO:SAFF | 0 | No concerns | Low risk | No concerns | Major concerns | No concerns | Major concerns | Low |
| RHO:SAFF+SCU | 0 | No concerns | Low risk | No concerns | Some concerns | Some concerns | Major concerns | Low |
| RHO:SAMe | 0 | No concerns | Low risk | No concerns | Some concerns | Some concerns | Major concerns | Low |
| RHO:SAMe+HELP9 | 0 | No concerns | Low risk | No concerns | No concerns | No concerns | Major concerns | Low |
| RHO:SCU | 0 | No concerns | Low risk | No concerns | Some concerns | Some concerns | Major concerns | Low |
| RHO:SER | 0 | No concerns | Low risk | No concerns | Some concerns | Some concerns | Major concerns | Low |
| RHO:SJW | 0 | No concerns | Low risk | No concerns | No concerns | Major concerns | Major concerns | Low |
| RHO:SRN | 0 | No concerns | Low risk | No concerns | Some concerns | Some concerns | Major concerns | Low |
| RHO:SZL | 0 | No concerns | Low risk | No concerns | No concerns | Some concerns | Major concerns | Low |
| RHO:TEN | 0 | No concerns | Low risk | No concerns | Major concerns | No concerns | Major concerns | Low |
| RHO:WS5570 | 0 | No concerns | Low risk | No concerns | Some concerns | Some concerns | Major concerns | Low |
| RHO:WS5572 | 0 | No concerns | Low risk | No concerns | Some concerns | Some concerns | Major concerns | Low |
| RHO:WS5573 | 0 | No concerns | Low risk | No concerns | No concerns | Major concerns | Major concerns | Low |
| RHO:ZE | 0 | No concerns | Low risk | No concerns | Major concerns | No concerns | Major concerns | Low |
| RHO:ZE117 | 0 | No concerns | Low risk | No concerns | Major concerns | No concerns | Major concerns | Low |
| RHO:vitB | 0 | No concerns | Low risk | No concerns | Major concerns | No concerns | Major concerns | Low |
| RHO:vitD | 0 | No concerns | Low risk | No concerns | No concerns | Major concerns | Major concerns | Low |
| RHO:vitD3 | 0 | No concerns | Low risk | No concerns | Major concerns | No concerns | Major concerns | Low |
| RHO:vitD3+CA | 0 | No concerns | Low risk | No concerns | Major concerns | No concerns | Major concerns | Low |
| RHO:vitD3+FLUO | 0 | No concerns | Low risk | No concerns | Major concerns | No concerns | Major concerns | Low |
| RHO+SER:SAF | 0 | No concerns | Low risk | No concerns | No concerns | No concerns | Major concerns | Low |
| RHO+SER:SAFF | 0 | No concerns | Low risk | No concerns | No concerns | No concerns | Major concerns | Low |
| RHO+SER:SAFF+SCU | 0 | No concerns | Low risk | No concerns | No concerns | No concerns | Major concerns | Low |
| RHO+SER:SAMe | 0 | No concerns | Low risk | No concerns | No concerns | No concerns | Major concerns | Low |
| RHO+SER:SAMe+HELP9 | 0 | No concerns | Low risk | No concerns | No concerns | No concerns | Major concerns | Low |
| RHO+SER:SCU | 0 | No concerns | Low risk | No concerns | No concerns | No concerns | Major concerns | Low |
| RHO+SER:SER | 0 | No concerns | Low risk | No concerns | No concerns | No concerns | Major concerns | Low |
| RHO+SER:SJW | 0 | No concerns | Low risk | No concerns | No concerns | No concerns | Major concerns | Low |
| RHO+SER:SRN | 0 | No concerns | Low risk | No concerns | No concerns | No concerns | Major concerns | Low |
| RHO+SER:SZL | 0 | No concerns | Low risk | No concerns | No concerns | No concerns | Major concerns | Low |
| RHO+SER:TEN | 0 | No concerns | Low risk | No concerns | Major concerns | No concerns | Major concerns | Low |
| RHO+SER:WS5570 | 0 | No concerns | Low risk | No concerns | No concerns | No concerns | Major concerns | Low |
| RHO+SER:WS5572 | 0 | No concerns | Low risk | No concerns | No concerns | No concerns | Major concerns | Low |
| RHO+SER:WS5573 | 0 | No concerns | Low risk | No concerns | No concerns | No concerns | Major concerns | Low |
| RHO+SER:ZE | 0 | No concerns | Low risk | No concerns | No concerns | No concerns | Major concerns | Low |
| RHO+SER:ZE117 | 0 | No concerns | Low risk | No concerns | No concerns | No concerns | Major concerns | Low |
| RHO+SER:vitB | 0 | No concerns | Low risk | No concerns | No concerns | Major concerns | Major concerns | Low |
| RHO+SER:vitD | 0 | No concerns | Low risk | No concerns | No concerns | No concerns | Major concerns | Low |
| RHO+SER:vitD3 | 0 | No concerns | Low risk | No concerns | No concerns | No concerns | Major concerns | Low |
| RHO+SER:vitD3+CA | 0 | No concerns | Low risk | No concerns | No concerns | No concerns | Major concerns | Low |
| RHO+SER:vitD3+FLUO | 0 | No concerns | Low risk | No concerns | No concerns | No concerns | Major concerns | Low |
| SAF:SAFF | 0 | No concerns | Low risk | No concerns | Major concerns | No concerns | Major concerns | Low |
| SAF:SAFF+SCU | 0 | No concerns | Low risk | No concerns | Major concerns | No concerns | Major concerns | Low |
| SAF:SAMe | 0 | No concerns | Low risk | No concerns | Major concerns | No concerns | Major concerns | Low |
| SAF:SAMe+HELP9 | 0 | No concerns | Low risk | No concerns | Major concerns | No concerns | Major concerns | Low |
| SAF:SCU | 0 | No concerns | Low risk | No concerns | Major concerns | No concerns | Major concerns | Low |
| SAF:SER | 0 | No concerns | Low risk | No concerns | Major concerns | No concerns | Major concerns | Low |
| SAF:SRN | 0 | No concerns | Low risk | No concerns | Major concerns | No concerns | Major concerns | Low |
| SAF:SZL | 0 | No concerns | Low risk | No concerns | Major concerns | No concerns | Major concerns | Low |
| SAF:TEN | 0 | No concerns | Low risk | No concerns | Some concerns | Some concerns | Major concerns | Low |
| SAF:WS5570 | 0 | No concerns | Low risk | No concerns | Major concerns | No concerns | Major concerns | Low |
| SAF:WS5572 | 0 | No concerns | Low risk | Some concerns | Major concerns | No concerns | Major concerns | Very Low |
| SAF:WS5573 | 0 | No concerns | Low risk | No concerns | Major concerns | No concerns | Major concerns | Low |
| SAF:ZE | 0 | No concerns | Low risk | No concerns | Major concerns | No concerns | Major concerns | Low |
| SAF:ZE117 | 0 | No concerns | Low risk | No concerns | Major concerns | No concerns | Major concerns | Low |
| SAF:vitB | 0 | No concerns | Low risk | No concerns | Major concerns | No concerns | Major concerns | Low |
| SAF:vitD | 0 | No concerns | Low risk | No concerns | Major concerns | No concerns | Major concerns | Low |
| SAF:vitD3 | 0 | No concerns | Low risk | No concerns | Major concerns | No concerns | Major concerns | Low |
| SAF:vitD3+CA | 0 | No concerns | Low risk | Some concerns | Major concerns | No concerns | Major concerns | Very Low |
| SAF:vitD3+FLUO | 0 | No concerns | Low risk | No concerns | Major concerns | No concerns | Major concerns | Low |
| SAFF:SAMe | 0 | No concerns | Low risk | No concerns | Major concerns | No concerns | Major concerns | Low |
| SAFF:SAMe+HELP9 | 0 | No concerns | Low risk | No concerns | Some concerns | Some concerns | Major concerns | Low |
| SAFF:SJW | 0 | No concerns | Low risk | No concerns | Major concerns | No concerns | Major concerns | Low |
| SAFF:SRN | 0 | No concerns | Low risk | No concerns | Major concerns | No concerns | Major concerns | Low |
| SAFF:SZL | 0 | No concerns | Low risk | No concerns | Major concerns | No concerns | Major concerns | Low |
| SAFF:TEN | 0 | No concerns | Low risk | No concerns | Major concerns | No concerns | Major concerns | Low |
| SAFF:WS5570 | 0 | No concerns | Low risk | No concerns | Major concerns | No concerns | Major concerns | Low |
| SAFF:WS5572 | 0 | No concerns | Low risk | No concerns | Major concerns | No concerns | Major concerns | Low |
| SAFF:WS5573 | 0 | No concerns | Low risk | No concerns | Major concerns | No concerns | Major concerns | Low |
| SAFF:ZE | 0 | No concerns | Low risk | No concerns | Major concerns | No concerns | Major concerns | Low |
| SAFF:ZE117 | 0 | No concerns | Low risk | No concerns | Major concerns | No concerns | Major concerns | Low |
| SAFF:vitB | 0 | No concerns | Low risk | No concerns | Major concerns | No concerns | Major concerns | Low |
| SAFF:vitD | 0 | No concerns | Low risk | No concerns | Major concerns | No concerns | Major concerns | Low |
| SAFF:vitD3 | 0 | No concerns | Low risk | No concerns | Major concerns | No concerns | Major concerns | Low |
| SAFF:vitD3+CA | 0 | No concerns | Low risk | No concerns | Major concerns | No concerns | Major concerns | Low |
| SAFF:vitD3+FLUO | 0 | No concerns | Low risk | No concerns | Major concerns | No concerns | Major concerns | Low |
| SAFF+SCU:SAMe | 0 | No concerns | Low risk | No concerns | Major concerns | No concerns | Major concerns | Low |
| SAFF+SCU:SAMe+HELP9 | 0 | No concerns | Low risk | No concerns | Major concerns | No concerns | Major concerns | Low |
| SAFF+SCU:SER | 0 | No concerns | Low risk | No concerns | Major concerns | No concerns | Major concerns | Low |
| SAFF+SCU:SJW | 0 | No concerns | Low risk | No concerns | Major concerns | No concerns | Major concerns | Low |
| SAFF+SCU:SRN | 0 | No concerns | Low risk | No concerns | Major concerns | No concerns | Major concerns | Low |
| SAFF+SCU:SZL | 0 | No concerns | Low risk | No concerns | Major concerns | No concerns | Major concerns | Low |
| SAFF+SCU:TEN | 0 | No concerns | Low risk | No concerns | Major concerns | No concerns | Major concerns | Low |
| SAFF+SCU:WS5570 | 0 | No concerns | Low risk | No concerns | Major concerns | No concerns | Major concerns | Low |
| SAFF+SCU:WS5572 | 0 | No concerns | Low risk | No concerns | Major concerns | No concerns | Major concerns | Low |
| SAFF+SCU:WS5573 | 0 | No concerns | Low risk | No concerns | Major concerns | No concerns | Major concerns | Low |
| SAFF+SCU:ZE | 0 | No concerns | Low risk | No concerns | Major concerns | No concerns | Major concerns | Low |
| SAFF+SCU:ZE117 | 0 | No concerns | Low risk | No concerns | Major concerns | No concerns | Major concerns | Low |
| SAFF+SCU:vitB | 0 | No concerns | Low risk | No concerns | Major concerns | No concerns | Major concerns | Low |
| SAFF+SCU:vitD | 0 | No concerns | Low risk | No concerns | Major concerns | No concerns | Major concerns | Low |
| SAFF+SCU:vitD3 | 0 | No concerns | Low risk | No concerns | Major concerns | No concerns | Major concerns | Low |
| SAFF+SCU:vitD3+CA | 0 | No concerns | Low risk | No concerns | Major concerns | No concerns | Major concerns | Low |
| SAFF+SCU:vitD3+FLUO | 0 | No concerns | Low risk | No concerns | Major concerns | No concerns | Major concerns | Low |
| SAMe:SAMe+HELP9 | 0 | No concerns | Low risk | No concerns | Major concerns | No concerns | Major concerns | Low |
| SAMe:SCU | 0 | No concerns | Low risk | No concerns | Major concerns | No concerns | Major concerns | Low |
| SAMe:SER | 0 | No concerns | Low risk | No concerns | Major concerns | No concerns | Major concerns | Low |
| SAMe:SJW | 0 | No concerns | Low risk | No concerns | Major concerns | No concerns | Major concerns | Low |
| SAMe:SRN | 0 | No concerns | Low risk | No concerns | Major concerns | No concerns | Major concerns | Low |
| SAMe:SZL | 0 | No concerns | Low risk | No concerns | Major concerns | No concerns | Major concerns | Low |
| SAMe:TEN | 0 | No concerns | Low risk | No concerns | Some concerns | Some concerns | Major concerns | Low |
| SAMe:WS5570 | 0 | No concerns | Low risk | No concerns | Major concerns | No concerns | Major concerns | Low |
| SAMe:WS5572 | 0 | No concerns | Low risk | No concerns | Major concerns | No concerns | Major concerns | Low |
| SAMe:WS5573 | 0 | No concerns | Low risk | No concerns | Major concerns | No concerns | Major concerns | Low |
| SAMe:ZE | 0 | No concerns | Low risk | No concerns | Major concerns | No concerns | Major concerns | Low |
| SAMe:ZE117 | 0 | No concerns | Low risk | No concerns | Major concerns | No concerns | Major concerns | Low |
| SAMe:vitB | 0 | No concerns | Low risk | No concerns | Major concerns | No concerns | Major concerns | Low |
| SAMe:vitD | 0 | No concerns | Low risk | No concerns | Major concerns | No concerns | Major concerns | Low |
| SAMe:vitD3 | 0 | No concerns | Low risk | No concerns | Major concerns | No concerns | Major concerns | Low |
| SAMe:vitD3+CA | 0 | No concerns | Low risk | No concerns | Major concerns | No concerns | Major concerns | Low |
| SAMe:vitD3+FLUO | 0 | No concerns | Low risk | No concerns | Major concerns | No concerns | Major concerns | Low |
| SAMe+HELP9:SCU | 0 | No concerns | Low risk | No concerns | Major concerns | No concerns | Major concerns | Low |
| SAMe+HELP9:SER | 0 | No concerns | Low risk | No concerns | Major concerns | No concerns | Major concerns | Low |
| SAMe+HELP9:SJW | 0 | No concerns | Low risk | No concerns | Major concerns | No concerns | Major concerns | Low |
| SAMe+HELP9:SRN | 0 | No concerns | Low risk | No concerns | Major concerns | No concerns | Major concerns | Low |
| SAMe+HELP9:SZL | 0 | No concerns | Low risk | No concerns | Major concerns | No concerns | Major concerns | Low |
| SAMe+HELP9:TEN | 0 | No concerns | Low risk | No concerns | No concerns | No concerns | Major concerns | Low |
| SAMe+HELP9:WS5570 | 0 | No concerns | Low risk | No concerns | Major concerns | No concerns | Major concerns | Low |
| SAMe+HELP9:WS5572 | 0 | No concerns | Low risk | No concerns | Major concerns | No concerns | Major concerns | Low |
| SAMe+HELP9:WS5573 | 0 | No concerns | Low risk | No concerns | Major concerns | No concerns | Major concerns | Low |
| SAMe+HELP9:ZE | 0 | No concerns | Low risk | No concerns | Major concerns | No concerns | Major concerns | Low |
| SAMe+HELP9:ZE117 | 0 | No concerns | Low risk | No concerns | Major concerns | No concerns | Major concerns | Low |
| SAMe+HELP9:vitB | 0 | No concerns | Low risk | No concerns | No concerns | Some concerns | Major concerns | Low |
| SAMe+HELP9:vitD | 0 | No concerns | Low risk | No concerns | Major concerns | No concerns | Major concerns | Low |
| SAMe+HELP9:vitD3 | 0 | No concerns | Low risk | No concerns | No concerns | Major concerns | Major concerns | Low |
| SAMe+HELP9:vitD3+CA | 0 | No concerns | Low risk | No concerns | Major concerns | No concerns | Major concerns | Low |
| SAMe+HELP9:vitD3+FLUO | 0 | No concerns | Low risk | No concerns | Major concerns | No concerns | Major concerns | Low |
| SCU:SER | 0 | No concerns | Low risk | No concerns | Major concerns | No concerns | Major concerns | Low |
| SCU:SJW | 0 | No concerns | Low risk | Some concerns | Major concerns | No concerns | Major concerns | Very Low |
| SCU:SRN | 0 | No concerns | Low risk | No concerns | Major concerns | No concerns | Major concerns | Low |
| SCU:SZL | 0 | No concerns | Low risk | No concerns | Major concerns | No concerns | Major concerns | Low |
| SCU:TEN | 0 | No concerns | Low risk | No concerns | Some concerns | Some concerns | Major concerns | Low |
| SCU:WS5570 | 0 | No concerns | Low risk | No concerns | Major concerns | No concerns | Major concerns | Low |
| SCU:WS5572 | 0 | No concerns | Low risk | No concerns | Major concerns | No concerns | Major concerns | Low |
| SCU:WS5573 | 0 | No concerns | Low risk | No concerns | Major concerns | No concerns | Major concerns | Low |
| SCU:ZE | 0 | No concerns | Low risk | No concerns | Major concerns | No concerns | Major concerns | Low |
| SCU:ZE117 | 0 | No concerns | Low risk | No concerns | Major concerns | No concerns | Major concerns | Low |
| SCU:vitB | 0 | No concerns | Low risk | No concerns | Major concerns | No concerns | Major concerns | Low |
| SCU:vitD | 0 | No concerns | Low risk | No concerns | Major concerns | No concerns | Major concerns | Low |
| SCU:vitD3 | 0 | No concerns | Low risk | No concerns | Major concerns | No concerns | Major concerns | Low |
| SCU:vitD3+CA | 0 | No concerns | Low risk | Some concerns | Major concerns | No concerns | Major concerns | Very Low |
| SCU:vitD3+FLUO | 0 | No concerns | Low risk | No concerns | Major concerns | No concerns | Major concerns | Low |
| SER:SRN | 0 | No concerns | Low risk | No concerns | Major concerns | No concerns | Major concerns | Low |
| SER:SZL | 0 | No concerns | Low risk | No concerns | Major concerns | No concerns | Major concerns | Low |
| SER:TEN | 0 | No concerns | Low risk | No concerns | Some concerns | Some concerns | Major concerns | Low |
| SER:WS5570 | 0 | No concerns | Low risk | No concerns | Major concerns | No concerns | Major concerns | Low |
| SER:WS5572 | 0 | No concerns | Low risk | No concerns | Major concerns | No concerns | Major concerns | Low |
| SER:WS5573 | 0 | No concerns | Low risk | No concerns | Major concerns | No concerns | Major concerns | Low |
| SER:ZE | 0 | No concerns | Low risk | No concerns | Major concerns | No concerns | Major concerns | Low |
| SER:ZE117 | 0 | No concerns | Low risk | No concerns | Major concerns | No concerns | Major concerns | Low |
| SER:vitB | 0 | No concerns | Low risk | No concerns | Major concerns | No concerns | Major concerns | Low |
| SER:vitD | 0 | No concerns | Low risk | No concerns | Major concerns | No concerns | Major concerns | Low |
| SER:vitD3 | 0 | No concerns | Low risk | No concerns | Major concerns | No concerns | Major concerns | Low |
| SER:vitD3+CA | 0 | No concerns | Low risk | No concerns | Major concerns | No concerns | Major concerns | Low |
| SER:vitD3+FLUO | 0 | No concerns | Low risk | No concerns | Major concerns | No concerns | Major concerns | Low |
| SJW:SRN | 0 | No concerns | Low risk | No concerns | Major concerns | No concerns | Major concerns | Low |
| SJW:SZL | 0 | No concerns | Low risk | No concerns | Major concerns | No concerns | Major concerns | Low |
| SJW:TEN | 0 | No concerns | Low risk | No concerns | No concerns | Major concerns | Major concerns | Low |
| SJW:WS5570 | 0 | No concerns | Low risk | No concerns | Major concerns | No concerns | Major concerns | Low |
| SJW:WS5572 | 0 | No concerns | Low risk | No concerns | Major concerns | No concerns | Major concerns | Low |
| SJW:WS5573 | 0 | No concerns | Low risk | No concerns | Major concerns | No concerns | Major concerns | Low |
| SJW:ZE | 0 | No concerns | Low risk | No concerns | Major concerns | No concerns | Major concerns | Low |
| SJW:ZE117 | 0 | No concerns | Low risk | No concerns | Major concerns | No concerns | Major concerns | Low |
| SJW:vitB | 0 | No concerns | Low risk | No concerns | Some concerns | Some concerns | Major concerns | Low |
| SJW:vitD | 0 | No concerns | Low risk | No concerns | Major concerns | No concerns | Major concerns | Low |
| SJW:vitD3 | 0 | No concerns | Low risk | No concerns | Major concerns | No concerns | Major concerns | Low |
| SJW:vitD3+CA | 0 | No concerns | Low risk | No concerns | Major concerns | No concerns | Major concerns | Low |
| SJW:vitD3+FLUO | 0 | No concerns | Low risk | No concerns | Major concerns | No concerns | Major concerns | Low |
| SRN:SZL | 0 | No concerns | Low risk | No concerns | Major concerns | No concerns | Major concerns | Low |
| SRN:TEN | 0 | No concerns | Low risk | No concerns | Some concerns | Some concerns | Major concerns | Low |
| SRN:WS5570 | 0 | No concerns | Low risk | No concerns | Major concerns | No concerns | Major concerns | Low |
| SRN:WS5572 | 0 | No concerns | Low risk | No concerns | Major concerns | No concerns | Major concerns | Low |
| SRN:WS5573 | 0 | No concerns | Low risk | No concerns | Major concerns | No concerns | Major concerns | Low |
| SRN:ZE | 0 | No concerns | Low risk | No concerns | Major concerns | No concerns | Major concerns | Low |
| SRN:ZE117 | 0 | No concerns | Low risk | No concerns | Major concerns | No concerns | Major concerns | Low |
| SRN:vitB | 0 | No concerns | Low risk | No concerns | Major concerns | No concerns | Major concerns | Low |
| SRN:vitD | 0 | No concerns | Low risk | No concerns | Major concerns | No concerns | Major concerns | Low |
| SRN:vitD3 | 0 | No concerns | Low risk | No concerns | Major concerns | No concerns | Major concerns | Low |
| SRN:vitD3+CA | 0 | No concerns | Low risk | No concerns | Major concerns | No concerns | Major concerns | Low |
| SRN:vitD3+FLUO | 0 | No concerns | Low risk | No concerns | Major concerns | No concerns | Major concerns | Low |
| SZL:TEN | 0 | No concerns | Low risk | No concerns | No concerns | Major concerns | Major concerns | Low |
| SZL:WS5570 | 0 | No concerns | Low risk | No concerns | Major concerns | No concerns | Major concerns | Low |
| SZL:WS5572 | 0 | No concerns | Low risk | No concerns | Major concerns | No concerns | Major concerns | Low |
| SZL:WS5573 | 0 | No concerns | Low risk | No concerns | Major concerns | No concerns | Major concerns | Low |
| SZL:ZE | 0 | No concerns | Low risk | No concerns | Major concerns | No concerns | Major concerns | Low |
| SZL:ZE117 | 0 | No concerns | Low risk | No concerns | Major concerns | No concerns | Major concerns | Low |
| SZL:vitB | 0 | No concerns | Low risk | No concerns | Some concerns | Some concerns | Major concerns | Low |
| SZL:vitD | 0 | No concerns | Low risk | No concerns | Major concerns | No concerns | Major concerns | Low |
| SZL:vitD3 | 0 | No concerns | Low risk | No concerns | Major concerns | No concerns | Major concerns | Low |
| SZL:vitD3+CA | 0 | No concerns | Low risk | No concerns | Major concerns | No concerns | Major concerns | Low |
| SZL:vitD3+FLUO | 0 | No concerns | Low risk | No concerns | Major concerns | No concerns | Major concerns | Low |
| TEN:WS5570 | 0 | No concerns | Low risk | No concerns | Some concerns | Some concerns | Major concerns | Low |
| TEN:WS5572 | 0 | No concerns | Low risk | No concerns | Some concerns | Some concerns | Major concerns | Low |
| TEN:WS5573 | 0 | No concerns | Low risk | No concerns | Some concerns | Some concerns | Major concerns | Low |
| TEN:ZE | 0 | No concerns | Low risk | No concerns | Major concerns | No concerns | Major concerns | Low |
| TEN:ZE117 | 0 | No concerns | Low risk | No concerns | Major concerns | No concerns | Major concerns | Low |
| TEN:vitB | 0 | No concerns | Low risk | No concerns | Major concerns | No concerns | Major concerns | Low |
| TEN:vitD | 0 | No concerns | Low risk | No concerns | No concerns | Major concerns | Major concerns | Low |
| TEN:vitD3 | 0 | No concerns | Low risk | No concerns | Major concerns | No concerns | Major concerns | Low |
| TEN:vitD3+CA | 0 | No concerns | Low risk | No concerns | Major concerns | No concerns | Major concerns | Low |
| TEN:vitD3+FLUO | 0 | No concerns | Low risk | No concerns | Major concerns | No concerns | Major concerns | Low |
| WS5570:WS5572 | 0 | No concerns | Low risk | No concerns | Major concerns | No concerns | Major concerns | Low |
| WS5570:WS5573 | 0 | No concerns | Low risk | No concerns | Major concerns | No concerns | Major concerns | Low |
| WS5570:ZE | 0 | No concerns | Low risk | No concerns | Major concerns | No concerns | Major concerns | Low |
| WS5570:ZE117 | 0 | No concerns | Low risk | No concerns | Major concerns | No concerns | Major concerns | Low |
| vitB:WS5570 | 0 | No concerns | Low risk | No concerns | Major concerns | No concerns | Major concerns | Low |
| vitD:WS5570 | 0 | No concerns | Low risk | No concerns | Major concerns | No concerns | Major concerns | Low |
| vitD3:WS5570 | 0 | No concerns | Low risk | No concerns | Major concerns | No concerns | Major concerns | Low |
| vitD3+CA:WS5570 | 0 | No concerns | Low risk | No concerns | Major concerns | No concerns | Major concerns | Low |
| vitD3+FLUO:WS5570 | 0 | No concerns | Low risk | No concerns | Major concerns | No concerns | Major concerns | Low |
| WS5572:ZE | 0 | No concerns | Low risk | No concerns | Major concerns | No concerns | Major concerns | Low |
| WS5572:ZE117 | 0 | No concerns | Low risk | No concerns | Major concerns | No concerns | Major concerns | Low |
| vitB:WS5572 | 0 | No concerns | Low risk | No concerns | Major concerns | No concerns | Major concerns | Low |
| vitD:WS5572 | 0 | No concerns | Low risk | No concerns | Major concerns | No concerns | Major concerns | Low |
| vitD3:WS5572 | 0 | No concerns | Low risk | No concerns | Major concerns | No concerns | Major concerns | Low |
| vitD3+CA:WS5572 | 0 | No concerns | Low risk | No concerns | Major concerns | No concerns | Major concerns | Low |
| vitD3+FLUO:WS5572 | 0 | No concerns | Low risk | No concerns | Major concerns | No concerns | Major concerns | Low |
| WS5573:ZE | 0 | No concerns | Low risk | No concerns | Major concerns | No concerns | Major concerns | Low |
| WS5573:ZE117 | 0 | No concerns | Low risk | No concerns | Major concerns | No concerns | Major concerns | Low |
| vitB:WS5573 | 0 | No concerns | Low risk | No concerns | Major concerns | No concerns | Major concerns | Low |
| vitD:WS5573 | 0 | No concerns | Low risk | No concerns | Major concerns | No concerns | Major concerns | Low |
| vitD3:WS5573 | 0 | No concerns | Low risk | No concerns | Major concerns | No concerns | Major concerns | Low |
| vitD3+CA:WS5573 | 0 | No concerns | Low risk | No concerns | Major concerns | No concerns | Major concerns | Low |
| vitD3+FLUO:WS5573 | 0 | No concerns | Low risk | No concerns | Major concerns | No concerns | Major concerns | Low |
| ZE:ZE117 | 0 | No concerns | Low risk | No concerns | Major concerns | No concerns | Major concerns | Low |
| vitB:ZE | 0 | No concerns | Low risk | No concerns | Major concerns | No concerns | Major concerns | Low |
| vitD:ZE | 0 | No concerns | Low risk | No concerns | Major concerns | No concerns | Major concerns | Low |
| vitD3:ZE | 0 | No concerns | Low risk | No concerns | Major concerns | No concerns | Major concerns | Low |
| vitD3+CA:ZE | 0 | No concerns | Low risk | No concerns | Major concerns | No concerns | Major concerns | Low |
| vitD3+FLUO:ZE | 0 | No concerns | Low risk | No concerns | Major concerns | No concerns | Major concerns | Low |
| vitB:ZE117 | 0 | No concerns | Low risk | No concerns | Major concerns | No concerns | Major concerns | Low |
| vitD:ZE117 | 0 | No concerns | Low risk | No concerns | Major concerns | No concerns | Major concerns | Low |
| vitD3:ZE117 | 0 | No concerns | Low risk | No concerns | Major concerns | No concerns | Major concerns | Low |
| vitD3+CA:ZE117 | 0 | No concerns | Low risk | No concerns | Major concerns | No concerns | Major concerns | Low |
| vitD3+FLUO:ZE117 | 0 | No concerns | Low risk | No concerns | Major concerns | No concerns | Major concerns | Low |
| vitB:vitD | 0 | No concerns | Low risk | No concerns | Some concerns | Some concerns | Major concerns | Low |
| vitB:vitD3 | 0 | No concerns | Low risk | No concerns | Major concerns | No concerns | Major concerns | Low |
| vitB:vitD3+CA | 0 | No concerns | Low risk | No concerns | Major concerns | No concerns | Major concerns | Low |
| vitB:vitD3+FLUO | 0 | No concerns | Low risk | No concerns | Major concerns | No concerns | Major concerns | Low |
| vitD:vitD3 | 0 | No concerns | Low risk | Some concerns | Major concerns | No concerns | Major concerns | Very Low |
| vitD:vitD3+CA | 0 | No concerns | Low risk | No concerns | Major concerns | No concerns | Major concerns | Low |
| vitD:vitD3+FLUO | 0 | No concerns | Low risk | No concerns | Major concerns | No concerns | Major concerns | Low |
| vitD3:vitD3+FLUO | 0 | No concerns | Low risk | No concerns | Major concerns | No concerns | Major concerns | Low |
| vitD3+CA:vitD3+FLUO | 0 | No concerns | Low risk | No concerns | Major concerns | No concerns | Major concerns | Low |

## **S11.3** NMA confidence rating for *response*.

**e-figure 65.** Netgraph. Nodes are represented according to the proportion of studies with each level of risk of bias. Edges are described according to the majority risk of bias within the comparison. Node size is equal due to graphical reasons. Edges’ width is proportional to the sample size. Red stands for high risk of bias, yellow for unclear, and green for low risk of bias

**e-table 51:** CINeMA GRADE evaluation of response.

| **Comparison** | **Number of studies** | **Within-study bias** | **Reporting bias** | **Indirectness** | **Imprecision** | **Heterogeneity** | **Incoherence** | **Confidence rating** |
| --- | --- | --- | --- | --- | --- | --- | --- | --- |
| AMI:LI-160 | 1 | No concerns | Low risk | No concerns | Major concerns | No concerns | No concerns | Low |
| AYA:PLB | 1 | Some concerns | Low risk | No concerns | Major concerns | No concerns | No concerns | Very Low |
| CHRO:PLB | 2 | No concerns | Low risk | No concerns | Major concerns | No concerns | No concerns | Low |
| CREA:PLB | 1 | No concerns | Low risk | No concerns | Major concerns | No concerns | No concerns | Low |
| CTP:SAFF | 1 | No concerns | Low risk | No concerns | Major concerns | No concerns | No concerns | Low |
| CUR:CUR+FLUO | 1 | No concerns | Low risk | No concerns | Major concerns | No concerns | No concerns | Low |
| CUR:FLUO | 1 | No concerns | Low risk | No concerns | Major concerns | No concerns | No concerns | Low |
| CUR+FLUO:FLUO | 1 | No concerns | Low risk | No concerns | Major concerns | No concerns | No concerns | Low |
| CUR+SAFF:PLB | 1 | No concerns | Low risk | No concerns | Major concerns | No concerns | No concerns | Low |
| DHA:PLB | 1 | No concerns | Low risk | No concerns | Major concerns | No concerns | No concerns | Low |
| EPA:PLB | 3 | No concerns | Low risk | No concerns | Major concerns | No concerns | No concerns | Low |
| EPA+DHA:PLB | 3 | No concerns | Low risk | No concerns | Major concerns | No concerns | No concerns | Low |
| ESC:ESC+FOL | 1 | No concerns | Low risk | No concerns | Major concerns | No concerns | No concerns | Low |
| ESC:PLB | 2 | No concerns | Low risk | No concerns | Major concerns | No concerns | No concerns | Low |
| ESC:SAMe | 2 | No concerns | Low risk | No concerns | Major concerns | No concerns | No concerns | Low |
| FLUO:LH57 | 1 | No concerns | Low risk | Some concerns | Major concerns | No concerns | No concerns | Very Low |
| FLUO:SAFF | 2 | Some concerns | Low risk | No concerns | Major concerns | No concerns | No concerns | Very Low |
| FLUO:SJW | 1 | No concerns | Low risk | No concerns | Major concerns | No concerns | No concerns | Low |
| FLUO:ZE117 | 1 | No concerns | Low risk | No concerns | Major concerns | No concerns | No concerns | Low |
| GBDP:PLB | 1 | No concerns | Low risk | No concerns | Major concerns | No concerns | No concerns | Low |
| IMI:LI-160 | 1 | No concerns | Low risk | No concerns | Major concerns | No concerns | No concerns | Low |
| IMI:ZE | 1 | No concerns | Low risk | No concerns | Major concerns | No concerns | No concerns | Low |
| INO:PLB | 2 | Some concerns | Low risk | Major concerns | Major concerns | No concerns | No concerns | Very Low |
| LI-160:PLB | 1 | No concerns | Low risk | No concerns | Major concerns | No concerns | No concerns | Low |
| LI-160:SER | 1 | No concerns | Low risk | No concerns | Major concerns | No concerns | No concerns | Low |
| LMF:PLB | 2 | No concerns | Low risk | No concerns | Major concerns | No concerns | No concerns | Low |
| PLB:SAFF | 1 | No concerns | Low risk | No concerns | Major concerns | No concerns | No concerns | Low |
| PLB:SAMe | 3 | No concerns | Low risk | No concerns | Major concerns | No concerns | No concerns | Low |
| PLB:SHU | 1 | No concerns | Low risk | No concerns | Major concerns | No concerns | No concerns | Low |
| PLB:SJW | 1 | No concerns | Low risk | No concerns | Major concerns | No concerns | No concerns | Low |
| PLB:WS5570 | 3 | No concerns | Low risk | No concerns | Major concerns | No concerns | No concerns | Low |
| PLB:WS5572 | 2 | No concerns | Low risk | No concerns | Major concerns | No concerns | No concerns | Low |
| PLB:WS5573 | 1 | No concerns | Low risk | No concerns | Major concerns | No concerns | No concerns | Low |
| PLB:ZE117 | 1 | No concerns | Low risk | No concerns | Major concerns | No concerns | No concerns | Low |
| PLB:ZPG | 1 | No concerns | Low risk | Some concerns | Major concerns | No concerns | No concerns | Very Low |
| PLB:vitD3 | 1 | No concerns | Low risk | Some concerns | Major concerns | No concerns | No concerns | Very Low |
| SAFF:SER | 1 | No concerns | Low risk | Some concerns | Major concerns | No concerns | No concerns | Very Low |
| SAFF:SSRI | 1 | Some concerns | Low risk | Some concerns | Major concerns | No concerns | No concerns | Very Low |
| WS5572:WS5573 | 1 | No concerns | Low risk | No concerns | Major concerns | No concerns | No concerns | Low |
| AMI:AYA | 0 | No concerns | Low risk | No concerns | Major concerns | No concerns | No concerns | Low |
| AMI:CHRO | 0 | No concerns | Low risk | No concerns | Major concerns | No concerns | No concerns | Low |
| AMI:CREA | 0 | No concerns | Low risk | No concerns | Major concerns | No concerns | No concerns | Low |
| AMI:CTP | 0 | No concerns | Low risk | No concerns | Major concerns | No concerns | No concerns | Low |
| AMI:CUR | 0 | No concerns | Low risk | No concerns | Major concerns | No concerns | No concerns | Low |
| AMI:CUR+FLUO | 0 | No concerns | Low risk | No concerns | Major concerns | No concerns | No concerns | Low |
| AMI:CUR+SAFF | 0 | No concerns | Low risk | No concerns | Major concerns | No concerns | No concerns | Low |
| AMI:DHA | 0 | No concerns | Low risk | No concerns | Major concerns | No concerns | No concerns | Low |
| AMI:EPA | 0 | No concerns | Low risk | No concerns | Major concerns | No concerns | No concerns | Low |
| AMI:EPA+DHA | 0 | No concerns | Low risk | No concerns | Major concerns | No concerns | No concerns | Low |
| AMI:ESC | 0 | No concerns | Low risk | No concerns | Major concerns | No concerns | No concerns | Low |
| AMI:ESC+FOL | 0 | No concerns | Low risk | No concerns | Major concerns | No concerns | No concerns | Low |
| AMI:FLUO | 0 | No concerns | Low risk | No concerns | Major concerns | No concerns | No concerns | Low |
| AMI:GBDP | 0 | No concerns | Low risk | No concerns | Major concerns | No concerns | No concerns | Low |
| AMI:IMI | 0 | No concerns | Low risk | No concerns | Major concerns | No concerns | No concerns | Low |
| AMI:INO | 0 | No concerns | Low risk | No concerns | Major concerns | No concerns | No concerns | Low |
| AMI:LH57 | 0 | No concerns | Low risk | No concerns | Major concerns | No concerns | No concerns | Low |
| AMI:LMF | 0 | No concerns | Low risk | No concerns | Major concerns | No concerns | No concerns | Low |
| AMI:PLB | 0 | No concerns | Low risk | No concerns | Major concerns | No concerns | No concerns | Low |
| AMI:SAFF | 0 | No concerns | Low risk | No concerns | Major concerns | No concerns | No concerns | Low |
| AMI:SAMe | 0 | No concerns | Low risk | No concerns | Major concerns | No concerns | No concerns | Low |
| AMI:SER | 0 | No concerns | Low risk | No concerns | Major concerns | No concerns | No concerns | Low |
| AMI:SHU | 0 | No concerns | Low risk | No concerns | Major concerns | No concerns | No concerns | Low |
| AMI:SJW | 0 | No concerns | Low risk | No concerns | Major concerns | No concerns | No concerns | Low |
| AMI:SSRI | 0 | No concerns | Low risk | No concerns | Major concerns | No concerns | No concerns | Low |
| AMI:WS5570 | 0 | No concerns | Low risk | No concerns | Major concerns | No concerns | No concerns | Low |
| AMI:WS5572 | 0 | No concerns | Low risk | No concerns | Major concerns | No concerns | No concerns | Low |
| AMI:WS5573 | 0 | No concerns | Low risk | No concerns | Major concerns | No concerns | No concerns | Low |
| AMI:ZE | 0 | No concerns | Low risk | No concerns | Major concerns | No concerns | No concerns | Low |
| AMI:ZE117 | 0 | No concerns | Low risk | No concerns | Major concerns | No concerns | No concerns | Low |
| AMI:ZPG | 0 | No concerns | Low risk | No concerns | Major concerns | No concerns | No concerns | Low |
| AMI:vitD3 | 0 | No concerns | Low risk | No concerns | Major concerns | No concerns | No concerns | Low |
| AYA:CHRO | 0 | No concerns | Low risk | No concerns | Major concerns | No concerns | No concerns | Low |
| AYA:CREA | 0 | No concerns | Low risk | No concerns | Major concerns | No concerns | No concerns | Low |
| AYA:CTP | 0 | No concerns | Low risk | No concerns | Major concerns | No concerns | No concerns | Low |
| AYA:CUR | 0 | No concerns | Low risk | No concerns | Major concerns | No concerns | No concerns | Low |
| AYA:CUR+FLUO | 0 | No concerns | Low risk | No concerns | Major concerns | No concerns | No concerns | Low |
| AYA:CUR+SAFF | 0 | No concerns | Low risk | No concerns | Major concerns | No concerns | No concerns | Low |
| AYA:DHA | 0 | No concerns | Low risk | No concerns | Major concerns | No concerns | No concerns | Low |
| AYA:EPA | 0 | Some concerns | Low risk | No concerns | Major concerns | No concerns | No concerns | Very Low |
| AYA:EPA+DHA | 0 | No concerns | Low risk | No concerns | Major concerns | No concerns | No concerns | Low |
| AYA:ESC | 0 | Some concerns | Low risk | No concerns | Major concerns | No concerns | No concerns | Very Low |
| AYA:ESC+FOL | 0 | No concerns | Low risk | No concerns | Major concerns | No concerns | No concerns | Low |
| AYA:FLUO | 0 | No concerns | Low risk | No concerns | Major concerns | No concerns | No concerns | Low |
| AYA:GBDP | 0 | No concerns | Low risk | No concerns | Major concerns | No concerns | No concerns | Low |
| AYA:IMI | 0 | No concerns | Low risk | No concerns | Major concerns | No concerns | No concerns | Low |
| AYA:INO | 0 | Some concerns | Low risk | No concerns | Major concerns | No concerns | No concerns | Very Low |
| AYA:LH57 | 0 | No concerns | Low risk | No concerns | Major concerns | No concerns | No concerns | Low |
| AYA:LI-160 | 0 | No concerns | Low risk | No concerns | Major concerns | No concerns | No concerns | Low |
| AYA:LMF | 0 | No concerns | Low risk | No concerns | Major concerns | No concerns | No concerns | Low |
| AYA:SAFF | 0 | No concerns | Low risk | No concerns | Major concerns | No concerns | No concerns | Low |
| AYA:SAMe | 0 | Some concerns | Low risk | No concerns | Major concerns | No concerns | No concerns | Very Low |
| AYA:SER | 0 | No concerns | Low risk | No concerns | Major concerns | No concerns | No concerns | Low |
| AYA:SHU | 0 | No concerns | Low risk | No concerns | Major concerns | No concerns | No concerns | Low |
| AYA:SJW | 0 | No concerns | Low risk | No concerns | Major concerns | No concerns | No concerns | Low |
| AYA:SSRI | 0 | Some concerns | Low risk | No concerns | Major concerns | No concerns | No concerns | Very Low |
| AYA:WS5570 | 0 | No concerns | Low risk | No concerns | Major concerns | No concerns | No concerns | Low |
| AYA:WS5572 | 0 | No concerns | Low risk | No concerns | Major concerns | No concerns | No concerns | Low |
| AYA:WS5573 | 0 | No concerns | Low risk | No concerns | Major concerns | No concerns | No concerns | Low |
| AYA:ZE | 0 | No concerns | Low risk | No concerns | Major concerns | No concerns | No concerns | Low |
| AYA:ZE117 | 0 | No concerns | Low risk | No concerns | Major concerns | No concerns | No concerns | Low |
| AYA:ZPG | 0 | No concerns | Low risk | No concerns | Major concerns | No concerns | No concerns | Low |
| AYA:vitD3 | 0 | No concerns | Low risk | No concerns | Major concerns | No concerns | No concerns | Low |
| CHRO:CREA | 0 | No concerns | Low risk | No concerns | Major concerns | No concerns | No concerns | Low |
| CHRO:CTP | 0 | No concerns | Low risk | No concerns | Major concerns | No concerns | No concerns | Low |
| CHRO:CUR | 0 | No concerns | Low risk | No concerns | Major concerns | No concerns | No concerns | Low |
| CHRO:CUR+FLUO | 0 | No concerns | Low risk | No concerns | Major concerns | No concerns | No concerns | Low |
| CHRO:CUR+SAFF | 0 | No concerns | Low risk | No concerns | Major concerns | No concerns | No concerns | Low |
| CHRO:DHA | 0 | No concerns | Low risk | No concerns | Major concerns | No concerns | No concerns | Low |
| CHRO:EPA | 0 | No concerns | Low risk | No concerns | Major concerns | No concerns | No concerns | Low |
| CHRO:EPA+DHA | 0 | No concerns | Low risk | No concerns | Major concerns | No concerns | No concerns | Low |
| CHRO:ESC | 0 | No concerns | Low risk | No concerns | Major concerns | No concerns | No concerns | Low |
| CHRO:ESC+FOL | 0 | No concerns | Low risk | No concerns | Major concerns | No concerns | No concerns | Low |
| CHRO:FLUO | 0 | No concerns | Low risk | No concerns | Major concerns | No concerns | No concerns | Low |
| CHRO:GBDP | 0 | No concerns | Low risk | No concerns | Major concerns | No concerns | No concerns | Low |
| CHRO:IMI | 0 | No concerns | Low risk | No concerns | Major concerns | No concerns | No concerns | Low |
| CHRO:INO | 0 | No concerns | Low risk | No concerns | Major concerns | No concerns | No concerns | Low |
| CHRO:LH57 | 0 | No concerns | Low risk | No concerns | Major concerns | No concerns | No concerns | Low |
| CHRO:LI-160 | 0 | No concerns | Low risk | No concerns | Major concerns | No concerns | No concerns | Low |
| CHRO:LMF | 0 | No concerns | Low risk | No concerns | Major concerns | No concerns | No concerns | Low |
| CHRO:SAFF | 0 | No concerns | Low risk | No concerns | Major concerns | No concerns | No concerns | Low |
| CHRO:SAMe | 0 | No concerns | Low risk | No concerns | Major concerns | No concerns | No concerns | Low |
| CHRO:SER | 0 | No concerns | Low risk | No concerns | Major concerns | No concerns | No concerns | Low |
| CHRO:SHU | 0 | No concerns | Low risk | No concerns | Major concerns | No concerns | No concerns | Low |
| CHRO:SJW | 0 | No concerns | Low risk | No concerns | Major concerns | No concerns | No concerns | Low |
| CHRO:SSRI | 0 | No concerns | Low risk | No concerns | Major concerns | No concerns | No concerns | Low |
| CHRO:WS5570 | 0 | No concerns | Low risk | No concerns | Major concerns | No concerns | No concerns | Low |
| CHRO:WS5572 | 0 | No concerns | Low risk | No concerns | Major concerns | No concerns | No concerns | Low |
| CHRO:WS5573 | 0 | No concerns | Low risk | No concerns | Major concerns | No concerns | No concerns | Low |
| CHRO:ZE | 0 | No concerns | Low risk | No concerns | Major concerns | No concerns | No concerns | Low |
| CHRO:ZE117 | 0 | No concerns | Low risk | No concerns | Major concerns | No concerns | No concerns | Low |
| CHRO:ZPG | 0 | No concerns | Low risk | No concerns | Major concerns | No concerns | No concerns | Low |
| CHRO:vitD3 | 0 | No concerns | Low risk | No concerns | Major concerns | No concerns | No concerns | Low |
| CREA:CTP | 0 | No concerns | Low risk | No concerns | Major concerns | No concerns | No concerns | Low |
| CREA:CUR | 0 | No concerns | Low risk | No concerns | Major concerns | No concerns | No concerns | Low |
| CREA:CUR+FLUO | 0 | No concerns | Low risk | No concerns | Major concerns | No concerns | No concerns | Low |
| CREA:CUR+SAFF | 0 | No concerns | Low risk | No concerns | Major concerns | No concerns | No concerns | Low |
| CREA:DHA | 0 | No concerns | Low risk | No concerns | Major concerns | No concerns | No concerns | Low |
| CREA:EPA | 0 | No concerns | Low risk | No concerns | Major concerns | No concerns | No concerns | Low |
| CREA:EPA+DHA | 0 | No concerns | Low risk | No concerns | Major concerns | No concerns | No concerns | Low |
| CREA:ESC | 0 | No concerns | Low risk | No concerns | Major concerns | No concerns | No concerns | Low |
| CREA:ESC+FOL | 0 | No concerns | Low risk | No concerns | Major concerns | No concerns | No concerns | Low |
| CREA:FLUO | 0 | No concerns | Low risk | No concerns | Major concerns | No concerns | No concerns | Low |
| CREA:GBDP | 0 | No concerns | Low risk | No concerns | Major concerns | No concerns | No concerns | Low |
| CREA:IMI | 0 | No concerns | Low risk | No concerns | Major concerns | No concerns | No concerns | Low |
| CREA:INO | 0 | No concerns | Low risk | No concerns | Major concerns | No concerns | No concerns | Low |
| CREA:LH57 | 0 | No concerns | Low risk | No concerns | Major concerns | No concerns | No concerns | Low |
| CREA:LI-160 | 0 | No concerns | Low risk | No concerns | Major concerns | No concerns | No concerns | Low |
| CREA:LMF | 0 | No concerns | Low risk | No concerns | Major concerns | No concerns | No concerns | Low |
| CREA:SAFF | 0 | No concerns | Low risk | No concerns | Major concerns | No concerns | No concerns | Low |
| CREA:SAMe | 0 | No concerns | Low risk | No concerns | Major concerns | No concerns | No concerns | Low |
| CREA:SER | 0 | No concerns | Low risk | No concerns | Major concerns | No concerns | No concerns | Low |
| CREA:SHU | 0 | No concerns | Low risk | No concerns | Major concerns | No concerns | No concerns | Low |
| CREA:SJW | 0 | No concerns | Low risk | No concerns | Major concerns | No concerns | No concerns | Low |
| CREA:SSRI | 0 | No concerns | Low risk | No concerns | Major concerns | No concerns | No concerns | Low |
| CREA:WS5570 | 0 | No concerns | Low risk | No concerns | Major concerns | No concerns | No concerns | Low |
| CREA:WS5572 | 0 | No concerns | Low risk | No concerns | Major concerns | No concerns | No concerns | Low |
| CREA:WS5573 | 0 | No concerns | Low risk | No concerns | Major concerns | No concerns | No concerns | Low |
| CREA:ZE | 0 | No concerns | Low risk | No concerns | Major concerns | No concerns | No concerns | Low |
| CREA:ZE117 | 0 | No concerns | Low risk | No concerns | Major concerns | No concerns | No concerns | Low |
| CREA:ZPG | 0 | No concerns | Low risk | No concerns | Major concerns | No concerns | No concerns | Low |
| CREA:vitD3 | 0 | No concerns | Low risk | No concerns | Major concerns | No concerns | No concerns | Low |
| CTP:CUR | 0 | No concerns | Low risk | No concerns | Major concerns | No concerns | No concerns | Low |
| CTP:CUR+FLUO | 0 | No concerns | Low risk | No concerns | Major concerns | No concerns | No concerns | Low |
| CTP:CUR+SAFF | 0 | No concerns | Low risk | No concerns | Major concerns | No concerns | No concerns | Low |
| CTP:DHA | 0 | No concerns | Low risk | No concerns | Major concerns | No concerns | No concerns | Low |
| CTP:EPA | 0 | No concerns | Low risk | No concerns | Major concerns | No concerns | No concerns | Low |
| CTP:EPA+DHA | 0 | No concerns | Low risk | No concerns | Major concerns | No concerns | No concerns | Low |
| CTP:ESC | 0 | No concerns | Low risk | No concerns | Major concerns | No concerns | No concerns | Low |
| CTP:ESC+FOL | 0 | No concerns | Low risk | No concerns | Major concerns | No concerns | No concerns | Low |
| CTP:FLUO | 0 | No concerns | Low risk | No concerns | Major concerns | No concerns | No concerns | Low |
| CTP:GBDP | 0 | No concerns | Low risk | No concerns | Major concerns | No concerns | No concerns | Low |
| CTP:IMI | 0 | No concerns | Low risk | No concerns | Major concerns | No concerns | No concerns | Low |
| CTP:INO | 0 | No concerns | Low risk | No concerns | Major concerns | No concerns | No concerns | Low |
| CTP:LH57 | 0 | No concerns | Low risk | No concerns | Major concerns | No concerns | No concerns | Low |
| CTP:LI-160 | 0 | No concerns | Low risk | No concerns | Major concerns | No concerns | No concerns | Low |
| CTP:LMF | 0 | No concerns | Low risk | No concerns | Major concerns | No concerns | No concerns | Low |
| CTP:PLB | 0 | No concerns | Low risk | No concerns | Major concerns | No concerns | No concerns | Low |
| CTP:SAMe | 0 | No concerns | Low risk | No concerns | Major concerns | No concerns | No concerns | Low |
| CTP:SER | 0 | No concerns | Low risk | No concerns | Major concerns | No concerns | No concerns | Low |
| CTP:SHU | 0 | No concerns | Low risk | No concerns | Major concerns | No concerns | No concerns | Low |
| CTP:SJW | 0 | No concerns | Low risk | No concerns | Major concerns | No concerns | No concerns | Low |
| CTP:SSRI | 0 | No concerns | Low risk | No concerns | Major concerns | No concerns | No concerns | Low |
| CTP:WS5570 | 0 | No concerns | Low risk | No concerns | Major concerns | No concerns | No concerns | Low |
| CTP:WS5572 | 0 | No concerns | Low risk | No concerns | Major concerns | No concerns | No concerns | Low |
| CTP:WS5573 | 0 | No concerns | Low risk | No concerns | Major concerns | No concerns | No concerns | Low |
| CTP:ZE | 0 | No concerns | Low risk | No concerns | Major concerns | No concerns | No concerns | Low |
| CTP:ZE117 | 0 | No concerns | Low risk | No concerns | Major concerns | No concerns | No concerns | Low |
| CTP:ZPG | 0 | No concerns | Low risk | No concerns | Major concerns | No concerns | No concerns | Low |
| CTP:vitD3 | 0 | No concerns | Low risk | No concerns | Major concerns | No concerns | No concerns | Low |
| CUR:CUR+SAFF | 0 | No concerns | Low risk | No concerns | Major concerns | No concerns | No concerns | Low |
| CUR:DHA | 0 | No concerns | Low risk | No concerns | Major concerns | No concerns | No concerns | Low |
| CUR:EPA | 0 | No concerns | Low risk | No concerns | Major concerns | No concerns | No concerns | Low |
| CUR:EPA+DHA | 0 | No concerns | Low risk | No concerns | Major concerns | No concerns | No concerns | Low |
| CUR:ESC | 0 | No concerns | Low risk | No concerns | Major concerns | No concerns | No concerns | Low |
| CUR:ESC+FOL | 0 | No concerns | Low risk | No concerns | Major concerns | No concerns | No concerns | Low |
| CUR:GBDP | 0 | No concerns | Low risk | No concerns | Major concerns | No concerns | No concerns | Low |
| CUR:IMI | 0 | No concerns | Low risk | No concerns | Major concerns | No concerns | No concerns | Low |
| CUR:INO | 0 | No concerns | Low risk | No concerns | Major concerns | No concerns | No concerns | Low |
| CUR:LH57 | 0 | No concerns | Low risk | No concerns | Major concerns | No concerns | No concerns | Low |
| CUR:LI-160 | 0 | No concerns | Low risk | No concerns | Major concerns | No concerns | No concerns | Low |
| CUR:LMF | 0 | No concerns | Low risk | No concerns | Major concerns | No concerns | No concerns | Low |
| CUR:PLB | 0 | No concerns | Low risk | No concerns | Major concerns | No concerns | No concerns | Low |
| CUR:SAFF | 0 | No concerns | Low risk | No concerns | Major concerns | No concerns | No concerns | Low |
| CUR:SAMe | 0 | No concerns | Low risk | No concerns | Major concerns | No concerns | No concerns | Low |
| CUR:SER | 0 | No concerns | Low risk | No concerns | Major concerns | No concerns | No concerns | Low |
| CUR:SHU | 0 | No concerns | Low risk | No concerns | Major concerns | No concerns | No concerns | Low |
| CUR:SJW | 0 | No concerns | Low risk | No concerns | Major concerns | No concerns | No concerns | Low |
| CUR:SSRI | 0 | Some concerns | Low risk | No concerns | Major concerns | No concerns | No concerns | Very Low |
| CUR:WS5570 | 0 | No concerns | Low risk | No concerns | Major concerns | No concerns | No concerns | Low |
| CUR:WS5572 | 0 | No concerns | Low risk | No concerns | Major concerns | No concerns | No concerns | Low |
| CUR:WS5573 | 0 | No concerns | Low risk | No concerns | Major concerns | No concerns | No concerns | Low |
| CUR:ZE | 0 | No concerns | Low risk | No concerns | Major concerns | No concerns | No concerns | Low |
| CUR:ZE117 | 0 | No concerns | Low risk | No concerns | Major concerns | No concerns | No concerns | Low |
| CUR:ZPG | 0 | No concerns | Low risk | No concerns | Major concerns | No concerns | No concerns | Low |
| CUR:vitD3 | 0 | No concerns | Low risk | No concerns | Major concerns | No concerns | No concerns | Low |
| CUR+FLUO:CUR+SAFF | 0 | No concerns | Low risk | No concerns | Major concerns | No concerns | No concerns | Low |
| CUR+FLUO:DHA | 0 | No concerns | Low risk | No concerns | Major concerns | No concerns | No concerns | Low |
| CUR+FLUO:EPA | 0 | No concerns | Low risk | No concerns | Major concerns | No concerns | No concerns | Low |
| CUR+FLUO:EPA+DHA | 0 | No concerns | Low risk | No concerns | Major concerns | No concerns | No concerns | Low |
| CUR+FLUO:ESC | 0 | No concerns | Low risk | No concerns | Major concerns | No concerns | No concerns | Low |
| CUR+FLUO:ESC+FOL | 0 | No concerns | Low risk | No concerns | Major concerns | No concerns | No concerns | Low |
| CUR+FLUO:GBDP | 0 | No concerns | Low risk | No concerns | Major concerns | No concerns | No concerns | Low |
| CUR+FLUO:IMI | 0 | No concerns | Low risk | No concerns | Major concerns | No concerns | No concerns | Low |
| CUR+FLUO:INO | 0 | No concerns | Low risk | No concerns | Major concerns | No concerns | No concerns | Low |
| CUR+FLUO:LH57 | 0 | No concerns | Low risk | No concerns | Major concerns | No concerns | No concerns | Low |
| CUR+FLUO:LI-160 | 0 | No concerns | Low risk | No concerns | Major concerns | No concerns | No concerns | Low |
| CUR+FLUO:LMF | 0 | No concerns | Low risk | No concerns | Major concerns | No concerns | No concerns | Low |
| CUR+FLUO:PLB | 0 | No concerns | Low risk | No concerns | Major concerns | No concerns | No concerns | Low |
| CUR+FLUO:SAFF | 0 | No concerns | Low risk | No concerns | Major concerns | No concerns | No concerns | Low |
| CUR+FLUO:SAMe | 0 | No concerns | Low risk | No concerns | Major concerns | No concerns | No concerns | Low |
| CUR+FLUO:SER | 0 | No concerns | Low risk | No concerns | Major concerns | No concerns | No concerns | Low |
| CUR+FLUO:SHU | 0 | No concerns | Low risk | No concerns | Major concerns | No concerns | No concerns | Low |
| CUR+FLUO:SJW | 0 | No concerns | Low risk | No concerns | Major concerns | No concerns | No concerns | Low |
| CUR+FLUO:SSRI | 0 | Some concerns | Low risk | No concerns | Major concerns | No concerns | No concerns | Very Low |
| CUR+FLUO:WS5570 | 0 | No concerns | Low risk | No concerns | Major concerns | No concerns | No concerns | Low |
| CUR+FLUO:WS5572 | 0 | No concerns | Low risk | No concerns | Major concerns | No concerns | No concerns | Low |
| CUR+FLUO:WS5573 | 0 | No concerns | Low risk | No concerns | Major concerns | No concerns | No concerns | Low |
| CUR+FLUO:ZE | 0 | No concerns | Low risk | No concerns | Major concerns | No concerns | No concerns | Low |
| CUR+FLUO:ZE117 | 0 | No concerns | Low risk | No concerns | Major concerns | No concerns | No concerns | Low |
| CUR+FLUO:ZPG | 0 | No concerns | Low risk | No concerns | Major concerns | No concerns | No concerns | Low |
| CUR+FLUO:vitD3 | 0 | No concerns | Low risk | No concerns | Major concerns | No concerns | No concerns | Low |
| CUR+SAFF:DHA | 0 | No concerns | Low risk | No concerns | Major concerns | No concerns | No concerns | Low |
| CUR+SAFF:EPA | 0 | No concerns | Low risk | No concerns | Major concerns | No concerns | No concerns | Low |
| CUR+SAFF:EPA+DHA | 0 | No concerns | Low risk | No concerns | Major concerns | No concerns | No concerns | Low |
| CUR+SAFF:ESC | 0 | No concerns | Low risk | No concerns | Major concerns | No concerns | No concerns | Low |
| CUR+SAFF:ESC+FOL | 0 | No concerns | Low risk | No concerns | Major concerns | No concerns | No concerns | Low |
| CUR+SAFF:FLUO | 0 | No concerns | Low risk | No concerns | Major concerns | No concerns | No concerns | Low |
| CUR+SAFF:GBDP | 0 | No concerns | Low risk | No concerns | Major concerns | No concerns | No concerns | Low |
| CUR+SAFF:IMI | 0 | No concerns | Low risk | No concerns | Major concerns | No concerns | No concerns | Low |
| CUR+SAFF:INO | 0 | No concerns | Low risk | No concerns | Major concerns | No concerns | No concerns | Low |
| CUR+SAFF:LH57 | 0 | No concerns | Low risk | No concerns | Major concerns | No concerns | No concerns | Low |
| CUR+SAFF:LI-160 | 0 | No concerns | Low risk | No concerns | Major concerns | No concerns | No concerns | Low |
| CUR+SAFF:LMF | 0 | No concerns | Low risk | No concerns | Major concerns | No concerns | No concerns | Low |
| CUR+SAFF:SAFF | 0 | No concerns | Low risk | No concerns | Major concerns | No concerns | No concerns | Low |
| CUR+SAFF:SAMe | 0 | No concerns | Low risk | No concerns | Major concerns | No concerns | No concerns | Low |
| CUR+SAFF:SER | 0 | No concerns | Low risk | No concerns | Major concerns | No concerns | No concerns | Low |
| CUR+SAFF:SHU | 0 | No concerns | Low risk | No concerns | Major concerns | No concerns | No concerns | Low |
| CUR+SAFF:SJW | 0 | No concerns | Low risk | No concerns | Major concerns | No concerns | No concerns | Low |
| CUR+SAFF:SSRI | 0 | No concerns | Low risk | No concerns | Major concerns | No concerns | No concerns | Low |
| CUR+SAFF:WS5570 | 0 | No concerns | Low risk | No concerns | Major concerns | No concerns | No concerns | Low |
| CUR+SAFF:WS5572 | 0 | No concerns | Low risk | No concerns | Major concerns | No concerns | No concerns | Low |
| CUR+SAFF:WS5573 | 0 | No concerns | Low risk | No concerns | Major concerns | No concerns | No concerns | Low |
| CUR+SAFF:ZE | 0 | No concerns | Low risk | No concerns | Major concerns | No concerns | No concerns | Low |
| CUR+SAFF:ZE117 | 0 | No concerns | Low risk | No concerns | Major concerns | No concerns | No concerns | Low |
| CUR+SAFF:ZPG | 0 | No concerns | Low risk | No concerns | Major concerns | No concerns | No concerns | Low |
| CUR+SAFF:vitD3 | 0 | No concerns | Low risk | No concerns | Major concerns | No concerns | No concerns | Low |
| DHA:EPA | 0 | No concerns | Low risk | No concerns | Major concerns | No concerns | No concerns | Low |
| DHA:EPA+DHA | 0 | No concerns | Low risk | No concerns | Major concerns | No concerns | No concerns | Low |
| DHA:ESC | 0 | No concerns | Low risk | No concerns | Major concerns | No concerns | No concerns | Low |
| DHA:ESC+FOL | 0 | No concerns | Low risk | No concerns | Major concerns | No concerns | No concerns | Low |
| DHA:FLUO | 0 | No concerns | Low risk | No concerns | Major concerns | No concerns | No concerns | Low |
| DHA:GBDP | 0 | No concerns | Low risk | No concerns | Major concerns | No concerns | No concerns | Low |
| DHA:IMI | 0 | No concerns | Low risk | No concerns | Major concerns | No concerns | No concerns | Low |
| DHA:INO | 0 | No concerns | Low risk | No concerns | Major concerns | No concerns | No concerns | Low |
| DHA:LH57 | 0 | No concerns | Low risk | No concerns | Major concerns | No concerns | No concerns | Low |
| DHA:LI-160 | 0 | No concerns | Low risk | No concerns | Major concerns | No concerns | No concerns | Low |
| DHA:LMF | 0 | No concerns | Low risk | No concerns | Major concerns | No concerns | No concerns | Low |
| DHA:SAFF | 0 | No concerns | Low risk | No concerns | Major concerns | No concerns | No concerns | Low |
| DHA:SAMe | 0 | No concerns | Low risk | No concerns | Major concerns | No concerns | No concerns | Low |
| DHA:SER | 0 | No concerns | Low risk | No concerns | Major concerns | No concerns | No concerns | Low |
| DHA:SHU | 0 | No concerns | Low risk | No concerns | Major concerns | No concerns | No concerns | Low |
| DHA:SJW | 0 | No concerns | Low risk | No concerns | Major concerns | No concerns | No concerns | Low |
| DHA:SSRI | 0 | No concerns | Low risk | No concerns | Major concerns | No concerns | No concerns | Low |
| DHA:WS5570 | 0 | No concerns | Low risk | No concerns | Major concerns | No concerns | No concerns | Low |
| DHA:WS5572 | 0 | No concerns | Low risk | No concerns | Major concerns | No concerns | No concerns | Low |
| DHA:WS5573 | 0 | No concerns | Low risk | No concerns | Major concerns | No concerns | No concerns | Low |
| DHA:ZE | 0 | No concerns | Low risk | No concerns | Major concerns | No concerns | No concerns | Low |
| DHA:ZE117 | 0 | No concerns | Low risk | No concerns | Major concerns | No concerns | No concerns | Low |
| DHA:ZPG | 0 | No concerns | Low risk | No concerns | Major concerns | No concerns | No concerns | Low |
| DHA:vitD3 | 0 | No concerns | Low risk | No concerns | Major concerns | No concerns | No concerns | Low |
| EPA:EPA+DHA | 0 | No concerns | Low risk | No concerns | Major concerns | No concerns | No concerns | Low |
| EPA:ESC | 0 | No concerns | Low risk | No concerns | Major concerns | No concerns | No concerns | Low |
| EPA:ESC+FOL | 0 | No concerns | Low risk | No concerns | Major concerns | No concerns | No concerns | Low |
| EPA:FLUO | 0 | No concerns | Low risk | No concerns | Major concerns | No concerns | No concerns | Low |
| EPA:GBDP | 0 | No concerns | Low risk | No concerns | Major concerns | No concerns | No concerns | Low |
| EPA:IMI | 0 | No concerns | Low risk | No concerns | Major concerns | No concerns | No concerns | Low |
| EPA:INO | 0 | No concerns | Low risk | No concerns | Major concerns | No concerns | No concerns | Low |
| EPA:LH57 | 0 | No concerns | Low risk | No concerns | Major concerns | No concerns | No concerns | Low |
| EPA:LI-160 | 0 | No concerns | Low risk | No concerns | Major concerns | No concerns | No concerns | Low |
| EPA:LMF | 0 | No concerns | Low risk | No concerns | Major concerns | No concerns | No concerns | Low |
| EPA:SAFF | 0 | No concerns | Low risk | No concerns | Major concerns | No concerns | No concerns | Low |
| EPA:SAMe | 0 | No concerns | Low risk | No concerns | Major concerns | No concerns | No concerns | Low |
| EPA:SER | 0 | No concerns | Low risk | No concerns | Major concerns | No concerns | No concerns | Low |
| EPA:SHU | 0 | No concerns | Low risk | No concerns | Major concerns | No concerns | No concerns | Low |
| EPA:SJW | 0 | No concerns | Low risk | No concerns | Major concerns | No concerns | No concerns | Low |
| EPA:SSRI | 0 | No concerns | Low risk | No concerns | Major concerns | No concerns | No concerns | Low |
| EPA:WS5570 | 0 | No concerns | Low risk | No concerns | Major concerns | No concerns | No concerns | Low |
| EPA:WS5572 | 0 | No concerns | Low risk | No concerns | Major concerns | No concerns | No concerns | Low |
| EPA:WS5573 | 0 | No concerns | Low risk | No concerns | Major concerns | No concerns | No concerns | Low |
| EPA:ZE | 0 | No concerns | Low risk | No concerns | Major concerns | No concerns | No concerns | Low |
| EPA:ZE117 | 0 | No concerns | Low risk | No concerns | Major concerns | No concerns | No concerns | Low |
| EPA:ZPG | 0 | No concerns | Low risk | No concerns | Major concerns | No concerns | No concerns | Low |
| EPA:vitD3 | 0 | No concerns | Low risk | No concerns | Major concerns | No concerns | No concerns | Low |
| EPA+DHA:ESC | 0 | No concerns | Low risk | No concerns | Major concerns | No concerns | No concerns | Low |
| EPA+DHA:ESC+FOL | 0 | No concerns | Low risk | No concerns | Major concerns | No concerns | No concerns | Low |
| EPA+DHA:FLUO | 0 | No concerns | Low risk | No concerns | Major concerns | No concerns | No concerns | Low |
| EPA+DHA:GBDP | 0 | No concerns | Low risk | No concerns | Major concerns | No concerns | No concerns | Low |
| EPA+DHA:IMI | 0 | No concerns | Low risk | No concerns | Major concerns | No concerns | No concerns | Low |
| EPA+DHA:INO | 0 | No concerns | Low risk | No concerns | Major concerns | No concerns | No concerns | Low |
| EPA+DHA:LH57 | 0 | No concerns | Low risk | No concerns | Major concerns | No concerns | No concerns | Low |
| EPA+DHA:LI-160 | 0 | No concerns | Low risk | No concerns | Major concerns | No concerns | No concerns | Low |
| EPA+DHA:LMF | 0 | No concerns | Low risk | No concerns | Major concerns | No concerns | No concerns | Low |
| EPA+DHA:SAFF | 0 | No concerns | Low risk | No concerns | Major concerns | No concerns | No concerns | Low |
| EPA+DHA:SAMe | 0 | No concerns | Low risk | No concerns | Major concerns | No concerns | No concerns | Low |
| EPA+DHA:SER | 0 | No concerns | Low risk | No concerns | Major concerns | No concerns | No concerns | Low |
| EPA+DHA:SHU | 0 | No concerns | Low risk | No concerns | Major concerns | No concerns | No concerns | Low |
| EPA+DHA:SJW | 0 | No concerns | Low risk | No concerns | Major concerns | No concerns | No concerns | Low |
| EPA+DHA:SSRI | 0 | No concerns | Low risk | No concerns | Major concerns | No concerns | No concerns | Low |
| EPA+DHA:WS5570 | 0 | No concerns | Low risk | No concerns | Major concerns | No concerns | No concerns | Low |
| EPA+DHA:WS5572 | 0 | No concerns | Low risk | No concerns | Major concerns | No concerns | No concerns | Low |
| EPA+DHA:WS5573 | 0 | No concerns | Low risk | No concerns | Major concerns | No concerns | No concerns | Low |
| EPA+DHA:ZE | 0 | No concerns | Low risk | No concerns | Major concerns | No concerns | No concerns | Low |
| EPA+DHA:ZE117 | 0 | No concerns | Low risk | No concerns | Major concerns | No concerns | No concerns | Low |
| EPA+DHA:ZPG | 0 | No concerns | Low risk | No concerns | Major concerns | No concerns | No concerns | Low |
| EPA+DHA:vitD3 | 0 | No concerns | Low risk | No concerns | Major concerns | No concerns | No concerns | Low |
| ESC:FLUO | 0 | No concerns | Low risk | No concerns | Major concerns | No concerns | No concerns | Low |
| ESC:GBDP | 0 | No concerns | Low risk | No concerns | Major concerns | No concerns | No concerns | Low |
| ESC:IMI | 0 | No concerns | Low risk | No concerns | Major concerns | No concerns | No concerns | Low |
| ESC:INO | 0 | No concerns | Low risk | No concerns | Major concerns | No concerns | No concerns | Low |
| ESC:LH57 | 0 | No concerns | Low risk | No concerns | Major concerns | No concerns | No concerns | Low |
| ESC:LI-160 | 0 | No concerns | Low risk | No concerns | Major concerns | No concerns | No concerns | Low |
| ESC:LMF | 0 | No concerns | Low risk | No concerns | Major concerns | No concerns | No concerns | Low |
| ESC:SAFF | 0 | No concerns | Low risk | No concerns | Major concerns | No concerns | No concerns | Low |
| ESC:SER | 0 | No concerns | Low risk | No concerns | Major concerns | No concerns | No concerns | Low |
| ESC:SHU | 0 | No concerns | Low risk | No concerns | Major concerns | No concerns | No concerns | Low |
| ESC:SJW | 0 | No concerns | Low risk | No concerns | Major concerns | No concerns | No concerns | Low |
| ESC:SSRI | 0 | No concerns | Low risk | No concerns | Major concerns | No concerns | No concerns | Low |
| ESC:WS5570 | 0 | No concerns | Low risk | No concerns | Major concerns | No concerns | No concerns | Low |
| ESC:WS5572 | 0 | No concerns | Low risk | No concerns | Major concerns | No concerns | No concerns | Low |
| ESC:WS5573 | 0 | No concerns | Low risk | No concerns | Major concerns | No concerns | No concerns | Low |
| ESC:ZE | 0 | No concerns | Low risk | No concerns | Major concerns | No concerns | No concerns | Low |
| ESC:ZE117 | 0 | No concerns | Low risk | No concerns | Major concerns | No concerns | No concerns | Low |
| ESC:ZPG | 0 | No concerns | Low risk | No concerns | Major concerns | No concerns | No concerns | Low |
| ESC:vitD3 | 0 | No concerns | Low risk | No concerns | Major concerns | No concerns | No concerns | Low |
| ESC+FOL:FLUO | 0 | No concerns | Low risk | No concerns | Major concerns | No concerns | No concerns | Low |
| ESC+FOL:GBDP | 0 | No concerns | Low risk | No concerns | Major concerns | No concerns | No concerns | Low |
| ESC+FOL:IMI | 0 | No concerns | Low risk | No concerns | Major concerns | No concerns | No concerns | Low |
| ESC+FOL:INO | 0 | No concerns | Low risk | No concerns | Major concerns | No concerns | No concerns | Low |
| ESC+FOL:LH57 | 0 | No concerns | Low risk | No concerns | Major concerns | No concerns | No concerns | Low |
| ESC+FOL:LI-160 | 0 | No concerns | Low risk | No concerns | Major concerns | No concerns | No concerns | Low |
| ESC+FOL:LMF | 0 | No concerns | Low risk | No concerns | Major concerns | No concerns | No concerns | Low |
| ESC+FOL:PLB | 0 | No concerns | Low risk | No concerns | Major concerns | No concerns | No concerns | Low |
| ESC+FOL:SAFF | 0 | No concerns | Low risk | No concerns | Major concerns | No concerns | No concerns | Low |
| ESC+FOL:SAMe | 0 | No concerns | Low risk | No concerns | Major concerns | No concerns | No concerns | Low |
| ESC+FOL:SER | 0 | No concerns | Low risk | No concerns | Major concerns | No concerns | No concerns | Low |
| ESC+FOL:SHU | 0 | No concerns | Low risk | No concerns | Major concerns | No concerns | No concerns | Low |
| ESC+FOL:SJW | 0 | No concerns | Low risk | No concerns | Major concerns | No concerns | No concerns | Low |
| ESC+FOL:SSRI | 0 | No concerns | Low risk | No concerns | Major concerns | No concerns | No concerns | Low |
| ESC+FOL:WS5570 | 0 | No concerns | Low risk | No concerns | Major concerns | No concerns | No concerns | Low |
| ESC+FOL:WS5572 | 0 | No concerns | Low risk | No concerns | Major concerns | No concerns | No concerns | Low |
| ESC+FOL:WS5573 | 0 | No concerns | Low risk | No concerns | Major concerns | No concerns | No concerns | Low |
| ESC+FOL:ZE | 0 | No concerns | Low risk | No concerns | Major concerns | No concerns | No concerns | Low |
| ESC+FOL:ZE117 | 0 | No concerns | Low risk | No concerns | Major concerns | No concerns | No concerns | Low |
| ESC+FOL:ZPG | 0 | No concerns | Low risk | No concerns | Major concerns | No concerns | No concerns | Low |
| ESC+FOL:vitD3 | 0 | No concerns | Low risk | No concerns | Major concerns | No concerns | No concerns | Low |
| FLUO:GBDP | 0 | No concerns | Low risk | No concerns | Major concerns | No concerns | No concerns | Low |
| FLUO:IMI | 0 | No concerns | Low risk | No concerns | Major concerns | No concerns | No concerns | Low |
| FLUO:INO | 0 | No concerns | Low risk | No concerns | Major concerns | No concerns | No concerns | Low |
| FLUO:LI-160 | 0 | No concerns | Low risk | No concerns | Major concerns | No concerns | No concerns | Low |
| FLUO:LMF | 0 | No concerns | Low risk | No concerns | Major concerns | No concerns | No concerns | Low |
| FLUO:PLB | 0 | No concerns | Low risk | No concerns | Major concerns | No concerns | No concerns | Low |
| FLUO:SAMe | 0 | No concerns | Low risk | No concerns | Major concerns | No concerns | No concerns | Low |
| FLUO:SER | 0 | No concerns | Low risk | No concerns | Major concerns | No concerns | No concerns | Low |
| FLUO:SHU | 0 | No concerns | Low risk | No concerns | Major concerns | No concerns | No concerns | Low |
| FLUO:SSRI | 0 | Some concerns | Low risk | No concerns | Major concerns | No concerns | No concerns | Very Low |
| FLUO:WS5570 | 0 | No concerns | Low risk | No concerns | Major concerns | No concerns | No concerns | Low |
| FLUO:WS5572 | 0 | No concerns | Low risk | No concerns | Major concerns | No concerns | No concerns | Low |
| FLUO:WS5573 | 0 | No concerns | Low risk | No concerns | Major concerns | No concerns | No concerns | Low |
| FLUO:ZE | 0 | No concerns | Low risk | No concerns | Major concerns | No concerns | No concerns | Low |
| FLUO:ZPG | 0 | No concerns | Low risk | No concerns | Major concerns | No concerns | No concerns | Low |
| FLUO:vitD3 | 0 | No concerns | Low risk | No concerns | Major concerns | No concerns | No concerns | Low |
| GBDP:IMI | 0 | No concerns | Low risk | No concerns | Major concerns | No concerns | No concerns | Low |
| GBDP:INO | 0 | No concerns | Low risk | No concerns | Major concerns | No concerns | No concerns | Low |
| GBDP:LH57 | 0 | No concerns | Low risk | No concerns | Major concerns | No concerns | No concerns | Low |
| GBDP:LI-160 | 0 | No concerns | Low risk | No concerns | Major concerns | No concerns | No concerns | Low |
| GBDP:LMF | 0 | No concerns | Low risk | No concerns | Major concerns | No concerns | No concerns | Low |
| GBDP:SAFF | 0 | No concerns | Low risk | No concerns | Major concerns | No concerns | No concerns | Low |
| GBDP:SAMe | 0 | No concerns | Low risk | No concerns | Major concerns | No concerns | No concerns | Low |
| GBDP:SER | 0 | No concerns | Low risk | No concerns | Major concerns | No concerns | No concerns | Low |
| GBDP:SHU | 0 | No concerns | Low risk | No concerns | Major concerns | No concerns | No concerns | Low |
| GBDP:SJW | 0 | No concerns | Low risk | No concerns | Major concerns | No concerns | No concerns | Low |
| GBDP:SSRI | 0 | No concerns | Low risk | No concerns | Major concerns | No concerns | No concerns | Low |
| GBDP:WS5570 | 0 | No concerns | Low risk | No concerns | Major concerns | No concerns | No concerns | Low |
| GBDP:WS5572 | 0 | No concerns | Low risk | No concerns | Major concerns | No concerns | No concerns | Low |
| GBDP:WS5573 | 0 | No concerns | Low risk | No concerns | Major concerns | No concerns | No concerns | Low |
| GBDP:ZE | 0 | No concerns | Low risk | No concerns | Major concerns | No concerns | No concerns | Low |
| GBDP:ZE117 | 0 | No concerns | Low risk | No concerns | Major concerns | No concerns | No concerns | Low |
| GBDP:ZPG | 0 | No concerns | Low risk | No concerns | Major concerns | No concerns | No concerns | Low |
| GBDP:vitD3 | 0 | No concerns | Low risk | No concerns | Major concerns | No concerns | No concerns | Low |
| IMI:INO | 0 | No concerns | Low risk | No concerns | Major concerns | No concerns | No concerns | Low |
| IMI:LH57 | 0 | No concerns | Low risk | No concerns | Major concerns | No concerns | No concerns | Low |
| IMI:LMF | 0 | No concerns | Low risk | No concerns | Major concerns | No concerns | No concerns | Low |
| IMI:PLB | 0 | No concerns | Low risk | No concerns | Major concerns | No concerns | No concerns | Low |
| IMI:SAFF | 0 | No concerns | Low risk | No concerns | Major concerns | No concerns | No concerns | Low |
| IMI:SAMe | 0 | No concerns | Low risk | No concerns | Major concerns | No concerns | No concerns | Low |
| IMI:SER | 0 | No concerns | Low risk | No concerns | Major concerns | No concerns | No concerns | Low |
| IMI:SHU | 0 | No concerns | Low risk | No concerns | Major concerns | No concerns | No concerns | Low |
| IMI:SJW | 0 | No concerns | Low risk | No concerns | Major concerns | No concerns | No concerns | Low |
| IMI:SSRI | 0 | No concerns | Low risk | No concerns | Major concerns | No concerns | No concerns | Low |
| IMI:WS5570 | 0 | No concerns | Low risk | No concerns | Major concerns | No concerns | No concerns | Low |
| IMI:WS5572 | 0 | No concerns | Low risk | No concerns | Major concerns | No concerns | No concerns | Low |
| IMI:WS5573 | 0 | No concerns | Low risk | No concerns | Major concerns | No concerns | No concerns | Low |
| IMI:ZE117 | 0 | No concerns | Low risk | No concerns | Major concerns | No concerns | No concerns | Low |
| IMI:ZPG | 0 | No concerns | Low risk | No concerns | Major concerns | No concerns | No concerns | Low |
| IMI:vitD3 | 0 | No concerns | Low risk | No concerns | Major concerns | No concerns | No concerns | Low |
| INO:LH57 | 0 | No concerns | Low risk | No concerns | Major concerns | No concerns | No concerns | Low |
| INO:LI-160 | 0 | No concerns | Low risk | No concerns | Major concerns | No concerns | No concerns | Low |
| INO:LMF | 0 | No concerns | Low risk | No concerns | Major concerns | No concerns | No concerns | Low |
| INO:SAFF | 0 | No concerns | Low risk | No concerns | Major concerns | No concerns | No concerns | Low |
| INO:SAMe | 0 | No concerns | Low risk | No concerns | Major concerns | No concerns | No concerns | Low |
| INO:SER | 0 | No concerns | Low risk | No concerns | Major concerns | No concerns | No concerns | Low |
| INO:SHU | 0 | No concerns | Low risk | No concerns | Major concerns | No concerns | No concerns | Low |
| INO:SJW | 0 | No concerns | Low risk | No concerns | Major concerns | No concerns | No concerns | Low |
| INO:SSRI | 0 | No concerns | Low risk | No concerns | Major concerns | No concerns | No concerns | Low |
| INO:WS5570 | 0 | No concerns | Low risk | No concerns | Major concerns | No concerns | No concerns | Low |
| INO:WS5572 | 0 | No concerns | Low risk | No concerns | Major concerns | No concerns | No concerns | Low |
| INO:WS5573 | 0 | No concerns | Low risk | No concerns | Major concerns | No concerns | No concerns | Low |
| INO:ZE | 0 | No concerns | Low risk | No concerns | Major concerns | No concerns | No concerns | Low |
| INO:ZE117 | 0 | No concerns | Low risk | No concerns | Major concerns | No concerns | No concerns | Low |
| INO:ZPG | 0 | No concerns | Low risk | Some concerns | Major concerns | No concerns | No concerns | Very Low |
| INO:vitD3 | 0 | No concerns | Low risk | Some concerns | Major concerns | No concerns | No concerns | Very Low |
| LH57:LI-160 | 0 | No concerns | Low risk | No concerns | Major concerns | No concerns | No concerns | Low |
| LH57:LMF | 0 | No concerns | Low risk | No concerns | Major concerns | No concerns | No concerns | Low |
| LH57:PLB | 0 | No concerns | Low risk | No concerns | Major concerns | No concerns | No concerns | Low |
| LH57:SAFF | 0 | No concerns | Low risk | No concerns | Major concerns | No concerns | No concerns | Low |
| LH57:SAMe | 0 | No concerns | Low risk | No concerns | Major concerns | No concerns | No concerns | Low |
| LH57:SER | 0 | No concerns | Low risk | Some concerns | Major concerns | No concerns | No concerns | Very Low |
| LH57:SHU | 0 | No concerns | Low risk | No concerns | Major concerns | No concerns | No concerns | Low |
| LH57:SJW | 0 | No concerns | Low risk | No concerns | Major concerns | No concerns | No concerns | Low |
| LH57:SSRI | 0 | Some concerns | Low risk | Some concerns | Major concerns | No concerns | No concerns | Very Low |
| LH57:WS5570 | 0 | No concerns | Low risk | No concerns | Major concerns | No concerns | No concerns | Low |
| LH57:WS5572 | 0 | No concerns | Low risk | No concerns | Major concerns | No concerns | No concerns | Low |
| LH57:WS5573 | 0 | No concerns | Low risk | No concerns | Major concerns | No concerns | No concerns | Low |
| LH57:ZE | 0 | No concerns | Low risk | No concerns | Major concerns | No concerns | No concerns | Low |
| LH57:ZE117 | 0 | No concerns | Low risk | No concerns | Major concerns | No concerns | No concerns | Low |
| LH57:ZPG | 0 | No concerns | Low risk | No concerns | Major concerns | No concerns | No concerns | Low |
| LH57:vitD3 | 0 | No concerns | Low risk | No concerns | Major concerns | No concerns | No concerns | Low |
| LI-160:LMF | 0 | No concerns | Low risk | No concerns | Major concerns | No concerns | No concerns | Low |
| LI-160:SAFF | 0 | No concerns | Low risk | No concerns | Major concerns | No concerns | No concerns | Low |
| LI-160:SAMe | 0 | No concerns | Low risk | No concerns | Major concerns | No concerns | No concerns | Low |
| LI-160:SHU | 0 | No concerns | Low risk | No concerns | Major concerns | No concerns | No concerns | Low |
| LI-160:SJW | 0 | No concerns | Low risk | No concerns | Major concerns | No concerns | No concerns | Low |
| LI-160:SSRI | 0 | No concerns | Low risk | No concerns | Major concerns | No concerns | No concerns | Low |
| LI-160:WS5570 | 0 | No concerns | Low risk | No concerns | Major concerns | No concerns | No concerns | Low |
| LI-160:WS5572 | 0 | No concerns | Low risk | No concerns | Major concerns | No concerns | No concerns | Low |
| LI-160:WS5573 | 0 | No concerns | Low risk | No concerns | Major concerns | No concerns | No concerns | Low |
| LI-160:ZE | 0 | No concerns | Low risk | No concerns | Major concerns | No concerns | No concerns | Low |
| LI-160:ZE117 | 0 | No concerns | Low risk | No concerns | Major concerns | No concerns | No concerns | Low |
| LI-160:ZPG | 0 | No concerns | Low risk | No concerns | Major concerns | No concerns | No concerns | Low |
| LI-160:vitD3 | 0 | No concerns | Low risk | No concerns | Major concerns | No concerns | No concerns | Low |
| LMF:SAFF | 0 | No concerns | Low risk | No concerns | Major concerns | No concerns | No concerns | Low |
| LMF:SAMe | 0 | No concerns | Low risk | No concerns | Major concerns | No concerns | No concerns | Low |
| LMF:SER | 0 | No concerns | Low risk | No concerns | Major concerns | No concerns | No concerns | Low |
| LMF:SHU | 0 | No concerns | Low risk | No concerns | Major concerns | No concerns | No concerns | Low |
| LMF:SJW | 0 | No concerns | Low risk | No concerns | Major concerns | No concerns | No concerns | Low |
| LMF:SSRI | 0 | No concerns | Low risk | No concerns | Major concerns | No concerns | No concerns | Low |
| LMF:WS5570 | 0 | No concerns | Low risk | No concerns | Major concerns | No concerns | No concerns | Low |
| LMF:WS5572 | 0 | No concerns | Low risk | No concerns | Major concerns | No concerns | No concerns | Low |
| LMF:WS5573 | 0 | No concerns | Low risk | No concerns | Major concerns | No concerns | No concerns | Low |
| LMF:ZE | 0 | No concerns | Low risk | No concerns | Major concerns | No concerns | No concerns | Low |
| LMF:ZE117 | 0 | No concerns | Low risk | No concerns | Major concerns | No concerns | No concerns | Low |
| LMF:ZPG | 0 | No concerns | Low risk | No concerns | Major concerns | No concerns | No concerns | Low |
| LMF:vitD3 | 0 | No concerns | Low risk | No concerns | Major concerns | No concerns | No concerns | Low |
| PLB:SER | 0 | No concerns | Low risk | No concerns | Major concerns | No concerns | No concerns | Low |
| PLB:SSRI | 0 | No concerns | Low risk | No concerns | Major concerns | No concerns | No concerns | Low |
| PLB:ZE | 0 | No concerns | Low risk | No concerns | Major concerns | No concerns | No concerns | Low |
| SAFF:SAMe | 0 | No concerns | Low risk | No concerns | Major concerns | No concerns | No concerns | Low |
| SAFF:SHU | 0 | No concerns | Low risk | No concerns | Major concerns | No concerns | No concerns | Low |
| SAFF:SJW | 0 | No concerns | Low risk | No concerns | Major concerns | No concerns | No concerns | Low |
| SAFF:WS5570 | 0 | No concerns | Low risk | No concerns | Major concerns | No concerns | No concerns | Low |
| SAFF:WS5572 | 0 | No concerns | Low risk | No concerns | Major concerns | No concerns | No concerns | Low |
| SAFF:WS5573 | 0 | No concerns | Low risk | No concerns | Major concerns | No concerns | No concerns | Low |
| SAFF:ZE | 0 | No concerns | Low risk | No concerns | Major concerns | No concerns | No concerns | Low |
| SAFF:ZE117 | 0 | No concerns | Low risk | No concerns | Major concerns | No concerns | No concerns | Low |
| SAFF:ZPG | 0 | No concerns | Low risk | No concerns | Major concerns | No concerns | No concerns | Low |
| SAFF:vitD3 | 0 | No concerns | Low risk | No concerns | Major concerns | No concerns | No concerns | Low |
| SAMe:SER | 0 | No concerns | Low risk | No concerns | Major concerns | No concerns | No concerns | Low |
| SAMe:SHU | 0 | No concerns | Low risk | No concerns | Major concerns | No concerns | No concerns | Low |
| SAMe:SJW | 0 | No concerns | Low risk | No concerns | Major concerns | No concerns | No concerns | Low |
| SAMe:SSRI | 0 | No concerns | Low risk | No concerns | Major concerns | No concerns | No concerns | Low |
| SAMe:WS5570 | 0 | No concerns | Low risk | No concerns | Major concerns | No concerns | No concerns | Low |
| SAMe:WS5572 | 0 | No concerns | Low risk | No concerns | Major concerns | No concerns | No concerns | Low |
| SAMe:WS5573 | 0 | No concerns | Low risk | No concerns | Major concerns | No concerns | No concerns | Low |
| SAMe:ZE | 0 | No concerns | Low risk | No concerns | Major concerns | No concerns | No concerns | Low |
| SAMe:ZE117 | 0 | No concerns | Low risk | No concerns | Major concerns | No concerns | No concerns | Low |
| SAMe:ZPG | 0 | No concerns | Low risk | No concerns | Major concerns | No concerns | No concerns | Low |
| SAMe:vitD3 | 0 | No concerns | Low risk | No concerns | Major concerns | No concerns | No concerns | Low |
| SER:SHU | 0 | No concerns | Low risk | No concerns | Major concerns | No concerns | No concerns | Low |
| SER:SJW | 0 | No concerns | Low risk | No concerns | Major concerns | No concerns | No concerns | Low |
| SER:SSRI | 0 | No concerns | Low risk | Some concerns | Major concerns | No concerns | No concerns | Very Low |
| SER:WS5570 | 0 | No concerns | Low risk | No concerns | Major concerns | No concerns | No concerns | Low |
| SER:WS5572 | 0 | No concerns | Low risk | No concerns | Major concerns | No concerns | No concerns | Low |
| SER:WS5573 | 0 | No concerns | Low risk | No concerns | Major concerns | No concerns | No concerns | Low |
| SER:ZE | 0 | No concerns | Low risk | No concerns | Major concerns | No concerns | No concerns | Low |
| SER:ZE117 | 0 | No concerns | Low risk | No concerns | Major concerns | No concerns | No concerns | Low |
| SER:ZPG | 0 | No concerns | Low risk | No concerns | Major concerns | No concerns | No concerns | Low |
| SER:vitD3 | 0 | No concerns | Low risk | No concerns | Major concerns | No concerns | No concerns | Low |
| SHU:SJW | 0 | No concerns | Low risk | No concerns | Major concerns | No concerns | No concerns | Low |
| SHU:SSRI | 0 | No concerns | Low risk | No concerns | Major concerns | No concerns | No concerns | Low |
| SHU:WS5570 | 0 | No concerns | Low risk | No concerns | Major concerns | No concerns | No concerns | Low |
| SHU:WS5572 | 0 | No concerns | Low risk | No concerns | Major concerns | No concerns | No concerns | Low |
| SHU:WS5573 | 0 | No concerns | Low risk | No concerns | Major concerns | No concerns | No concerns | Low |
| SHU:ZE | 0 | No concerns | Low risk | No concerns | Major concerns | No concerns | No concerns | Low |
| SHU:ZE117 | 0 | No concerns | Low risk | No concerns | Major concerns | No concerns | No concerns | Low |
| SHU:ZPG | 0 | No concerns | Low risk | No concerns | Major concerns | No concerns | No concerns | Low |
| SHU:vitD3 | 0 | No concerns | Low risk | No concerns | Major concerns | No concerns | No concerns | Low |
| SJW:SSRI | 0 | Some concerns | Low risk | No concerns | Major concerns | No concerns | No concerns | Very Low |
| SJW:WS5570 | 0 | No concerns | Low risk | No concerns | Major concerns | No concerns | No concerns | Low |
| SJW:WS5572 | 0 | No concerns | Low risk | No concerns | Major concerns | No concerns | No concerns | Low |
| SJW:WS5573 | 0 | No concerns | Low risk | No concerns | Major concerns | No concerns | No concerns | Low |
| SJW:ZE | 0 | No concerns | Low risk | No concerns | Major concerns | No concerns | No concerns | Low |
| SJW:ZE117 | 0 | No concerns | Low risk | No concerns | Major concerns | No concerns | No concerns | Low |
| SJW:ZPG | 0 | No concerns | Low risk | No concerns | Major concerns | No concerns | No concerns | Low |
| SJW:vitD3 | 0 | No concerns | Low risk | No concerns | Major concerns | No concerns | No concerns | Low |
| SSRI:WS5570 | 0 | No concerns | Low risk | No concerns | Major concerns | No concerns | No concerns | Low |
| SSRI:WS5572 | 0 | No concerns | Low risk | No concerns | Major concerns | No concerns | No concerns | Low |
| SSRI:WS5573 | 0 | No concerns | Low risk | No concerns | Major concerns | No concerns | No concerns | Low |
| SSRI:ZE | 0 | No concerns | Low risk | No concerns | Major concerns | No concerns | No concerns | Low |
| SSRI:ZE117 | 0 | Some concerns | Low risk | No concerns | Major concerns | No concerns | No concerns | Very Low |
| SSRI:ZPG | 0 | No concerns | Low risk | Some concerns | Major concerns | No concerns | No concerns | Very Low |
| SSRI:vitD3 | 0 | No concerns | Low risk | Some concerns | Major concerns | No concerns | No concerns | Very Low |
| WS5570:WS5572 | 0 | No concerns | Low risk | No concerns | Major concerns | No concerns | No concerns | Low |
| WS5570:WS5573 | 0 | No concerns | Low risk | No concerns | Major concerns | No concerns | No concerns | Low |
| WS5570:ZE | 0 | No concerns | Low risk | No concerns | Major concerns | No concerns | No concerns | Low |
| WS5570:ZE117 | 0 | No concerns | Low risk | No concerns | Major concerns | No concerns | No concerns | Low |
| WS5570:ZPG | 0 | No concerns | Low risk | No concerns | Major concerns | No concerns | No concerns | Low |
| vitD3:WS5570 | 0 | No concerns | Low risk | No concerns | Major concerns | No concerns | No concerns | Low |
| WS5572:ZE | 0 | No concerns | Low risk | No concerns | Major concerns | No concerns | No concerns | Low |
| WS5572:ZE117 | 0 | No concerns | Low risk | No concerns | Major concerns | No concerns | No concerns | Low |
| WS5572:ZPG | 0 | No concerns | Low risk | No concerns | Major concerns | No concerns | No concerns | Low |
| vitD3:WS5572 | 0 | No concerns | Low risk | No concerns | Major concerns | No concerns | No concerns | Low |
| WS5573:ZE | 0 | No concerns | Low risk | No concerns | Major concerns | No concerns | No concerns | Low |
| WS5573:ZE117 | 0 | No concerns | Low risk | No concerns | Major concerns | No concerns | No concerns | Low |
| WS5573:ZPG | 0 | No concerns | Low risk | No concerns | Major concerns | No concerns | No concerns | Low |
| vitD3:WS5573 | 0 | No concerns | Low risk | No concerns | Major concerns | No concerns | No concerns | Low |
| ZE:ZE117 | 0 | No concerns | Low risk | No concerns | Major concerns | No concerns | No concerns | Low |
| ZE:ZPG | 0 | No concerns | Low risk | No concerns | Major concerns | No concerns | No concerns | Low |
| vitD3:ZE | 0 | No concerns | Low risk | No concerns | Major concerns | No concerns | No concerns | Low |
| ZE117:ZPG | 0 | No concerns | Low risk | No concerns | Major concerns | No concerns | No concerns | Low |
| vitD3:ZE117 | 0 | No concerns | Low risk | No concerns | Major concerns | No concerns | No concerns | Low |
| vitD3:ZPG | 0 | No concerns | Low risk | Some concerns | Major concerns | No concerns | No concerns | Very Low |

## **S11.4** NMA confidence rating for *acceptability*.

**e-figure 66.** Netgraph. Nodes are represented according to the proportion of studies with each level of risk of bias. Edges are described according to the majority risk of bias within the comparison. Node size is equal due to graphical reasons. Edges’ width is proportional to the sample size. Red stands for high risk of bias, yellow for unclear, and green for low risk of bias

**e-table 52:** CINeMA GRADE overall evaluation of acceptability

| **Comparison** | **Number of studies** | **Within-study bias** | **Reporting bias** | **Indirectness** | **Imprecision** | **Heterogeneity** | **Incoherence** | **Confidence rating** |
| --- | --- | --- | --- | --- | --- | --- | --- | --- |
| AMI:LI-160 | 1 | No concerns | Low risk | No concerns | Major concerns | No concerns | No concerns | Low |
| AYA:PLB | 1 | No concerns | Low risk | No concerns | Major concerns | No concerns | No concerns | Low |
| CHRO:PROB | 1 | No concerns | Low risk | No concerns | Major concerns | No concerns | No concerns | Low |
| CIT:PLB | 1 | No concerns | Low risk | No concerns | Major concerns | No concerns | No concerns | Low |
| CUR:PLB | 2 | No concerns | Low risk | No concerns | Major concerns | No concerns | No concerns | Low |
| EPA:PLB | 2 | No concerns | Low risk | No concerns | Major concerns | No concerns | No concerns | Low |
| EPA+DHA:PLB | 4 | No concerns | Low risk | No concerns | Major concerns | No concerns | No concerns | Low |
| FLUO:LH57 | 1 | No concerns | Low risk | No concerns | Major concerns | No concerns | No concerns | Low |
| FLUO:SAFF | 4 | No concerns | Low risk | No concerns | Major concerns | No concerns | No concerns | Low |
| FLUO:SJW | 1 | No concerns | Low risk | No concerns | Major concerns | No concerns | No concerns | Low |
| INU:PLB | 1 | No concerns | Low risk | No concerns | Major concerns | No concerns | No concerns | Low |
| LI-160:PLB | 1 | No concerns | Low risk | No concerns | Major concerns | No concerns | No concerns | Low |
| LMF:PLB | 2 | No concerns | Low risk | No concerns | Major concerns | No concerns | No concerns | Low |
| NUT:PLB | 1 | No concerns | Low risk | No concerns | Major concerns | No concerns | No concerns | Low |
| PAR:WS5570 | 1 | No concerns | Low risk | No concerns | Major concerns | No concerns | No concerns | Low |
| PROB:PLB | 1 | No concerns | Low risk | No concerns | Major concerns | No concerns | No concerns | Low |
| PROB:SAFF | 1 | No concerns | Low risk | No concerns | Major concerns | No concerns | No concerns | Low |
| PROB:WS5572 | 1 | No concerns | Low risk | No concerns | Major concerns | No concerns | No concerns | Low |
| PLB:RHO | 1 | No concerns | Low risk | No concerns | Major concerns | No concerns | No concerns | Low |
| PLB:SAF | 2 | Some concerns | Low risk | No concerns | Major concerns | No concerns | No concerns | Very Low |
| PLB:SIL | 1 | No concerns | Low risk | No concerns | Major concerns | No concerns | No concerns | Low |
| PLB:SJW | 1 | No concerns | Low risk | No concerns | Major concerns | No concerns | No concerns | Low |
| PLB:WS5570 | 2 | No concerns | Low risk | No concerns | Major concerns | No concerns | No concerns | Low |
| PLB:ZPG | 1 | No concerns | Low risk | No concerns | Major concerns | No concerns | No concerns | Low |
| SAFF:SER | 1 | No concerns | Low risk | No concerns | Major concerns | No concerns | No concerns | Low |
| AMI:AYA | 0 | No concerns | Low risk | No concerns | Major concerns | No concerns | No concerns | Low |
| AMI:CHRO | 0 | No concerns | Low risk | No concerns | Major concerns | No concerns | No concerns | Low |
| AMI:CIT | 0 | No concerns | Low risk | No concerns | Major concerns | No concerns | No concerns | Low |
| AMI:CUR | 0 | No concerns | Low risk | No concerns | Major concerns | No concerns | No concerns | Low |
| AMI:EPA | 0 | No concerns | Low risk | No concerns | Major concerns | No concerns | No concerns | Low |
| AMI:EPA+DHA | 0 | No concerns | Low risk | No concerns | Major concerns | No concerns | No concerns | Low |
| AMI:FLUO | 0 | No concerns | Low risk | No concerns | Major concerns | No concerns | No concerns | Low |
| AMI:INU | 0 | No concerns | Low risk | No concerns | Major concerns | No concerns | No concerns | Low |
| AMI:LH57 | 0 | No concerns | Low risk | No concerns | Major concerns | No concerns | No concerns | Low |
| AMI:LMF | 0 | No concerns | Low risk | No concerns | Major concerns | No concerns | No concerns | Low |
| AMI:NUT | 0 | No concerns | Low risk | No concerns | Major concerns | No concerns | No concerns | Low |
| AMI:PAR | 0 | No concerns | Low risk | No concerns | Major concerns | No concerns | No concerns | Low |
| AMI:PROB | 0 | No concerns | Low risk | No concerns | Major concerns | No concerns | No concerns | Low |
| AMI:PLB | 0 | No concerns | Low risk | No concerns | Major concerns | No concerns | No concerns | Low |
| AMI:RHO | 0 | Some concerns | Low risk | No concerns | Major concerns | No concerns | No concerns | Very Low |
| AMI:SAF | 0 | No concerns | Low risk | No concerns | Major concerns | No concerns | No concerns | Low |
| AMI:SAFF | 0 | No concerns | Low risk | No concerns | Major concerns | No concerns | No concerns | Low |
| AMI:SER | 0 | No concerns | Low risk | No concerns | Major concerns | No concerns | No concerns | Low |
| AMI:SIL | 0 | No concerns | Low risk | No concerns | Major concerns | No concerns | No concerns | Low |
| AMI:SJW | 0 | No concerns | Low risk | No concerns | Major concerns | No concerns | No concerns | Low |
| AMI:WS5570 | 0 | No concerns | Low risk | No concerns | Major concerns | No concerns | No concerns | Low |
| AMI:WS5572 | 0 | No concerns | Low risk | No concerns | Major concerns | No concerns | No concerns | Low |
| AMI:ZPG | 0 | No concerns | Low risk | No concerns | Major concerns | No concerns | No concerns | Low |
| AYA:CHRO | 0 | No concerns | Low risk | No concerns | Major concerns | No concerns | No concerns | Low |
| AYA:CIT | 0 | No concerns | Low risk | No concerns | Major concerns | No concerns | No concerns | Low |
| AYA:CUR | 0 | No concerns | Low risk | No concerns | Major concerns | No concerns | No concerns | Low |
| AYA:EPA | 0 | No concerns | Low risk | No concerns | Major concerns | No concerns | No concerns | Low |
| AYA:EPA+DHA | 0 | No concerns | Low risk | No concerns | Major concerns | No concerns | No concerns | Low |
| AYA:FLUO | 0 | No concerns | Low risk | No concerns | Major concerns | No concerns | No concerns | Low |
| AYA:INU | 0 | No concerns | Low risk | No concerns | Major concerns | No concerns | No concerns | Low |
| AYA:LH57 | 0 | No concerns | Low risk | No concerns | Major concerns | No concerns | No concerns | Low |
| AYA:LI-160 | 0 | No concerns | Low risk | Some concerns | Major concerns | No concerns | No concerns | Very Low |
| AYA:LMF | 0 | No concerns | Low risk | No concerns | Major concerns | No concerns | No concerns | Low |
| AYA:NUT | 0 | No concerns | Low risk | No concerns | Major concerns | No concerns | No concerns | Low |
| AYA:PAR | 0 | No concerns | Low risk | No concerns | Major concerns | No concerns | No concerns | Low |
| AYA:PROB | 0 | No concerns | Low risk | No concerns | Major concerns | No concerns | No concerns | Low |
| AYA:RHO | 0 | No concerns | Low risk | No concerns | Major concerns | No concerns | No concerns | Low |
| AYA:SAF | 0 | No concerns | Low risk | No concerns | Major concerns | No concerns | No concerns | Low |
| AYA:SAFF | 0 | No concerns | Low risk | No concerns | Major concerns | No concerns | No concerns | Low |
| AYA:SER | 0 | No concerns | Low risk | No concerns | Major concerns | No concerns | No concerns | Low |
| AYA:SIL | 0 | No concerns | Low risk | No concerns | Major concerns | No concerns | No concerns | Low |
| AYA:SJW | 0 | No concerns | Low risk | No concerns | Major concerns | No concerns | No concerns | Low |
| AYA:WS5570 | 0 | No concerns | Low risk | No concerns | Major concerns | No concerns | No concerns | Low |
| AYA:WS5572 | 0 | Some concerns | Low risk | No concerns | Major concerns | No concerns | No concerns | Very Low |
| AYA:ZPG | 0 | No concerns | Low risk | No concerns | Major concerns | No concerns | No concerns | Low |
| CHRO:CIT | 0 | No concerns | Low risk | No concerns | Major concerns | No concerns | No concerns | Low |
| CHRO:CUR | 0 | No concerns | Low risk | No concerns | Major concerns | No concerns | No concerns | Low |
| CHRO:EPA | 0 | No concerns | Low risk | No concerns | Major concerns | No concerns | No concerns | Low |
| CHRO:EPA+DHA | 0 | No concerns | Low risk | No concerns | Major concerns | No concerns | No concerns | Low |
| CHRO:FLUO | 0 | No concerns | Low risk | No concerns | Major concerns | No concerns | No concerns | Low |
| CHRO:INU | 0 | No concerns | Low risk | No concerns | Major concerns | No concerns | No concerns | Low |
| CHRO:LH57 | 0 | No concerns | Low risk | No concerns | Major concerns | No concerns | No concerns | Low |
| CHRO:LI-160 | 0 | No concerns | Low risk | No concerns | Major concerns | No concerns | No concerns | Low |
| CHRO:LMF | 0 | No concerns | Low risk | No concerns | Major concerns | No concerns | No concerns | Low |
| CHRO:NUT | 0 | No concerns | Low risk | No concerns | Major concerns | No concerns | No concerns | Low |
| CHRO:PAR | 0 | Some concerns | Low risk | No concerns | Major concerns | No concerns | No concerns | Very Low |
| CHRO:PLB | 0 | No concerns | Low risk | No concerns | Major concerns | No concerns | No concerns | Low |
| CHRO:RHO | 0 | No concerns | Low risk | No concerns | Major concerns | No concerns | No concerns | Low |
| CHRO:SAF | 0 | No concerns | Low risk | No concerns | Major concerns | No concerns | No concerns | Low |
| CHRO:SAFF | 0 | No concerns | Low risk | No concerns | Major concerns | No concerns | No concerns | Low |
| CHRO:SER | 0 | No concerns | Low risk | No concerns | Major concerns | No concerns | No concerns | Low |
| CHRO:SIL | 0 | No concerns | Low risk | No concerns | Major concerns | No concerns | No concerns | Low |
| CHRO:SJW | 0 | No concerns | Low risk | No concerns | Major concerns | No concerns | No concerns | Low |
| CHRO:WS5570 | 0 | No concerns | Low risk | No concerns | Major concerns | No concerns | No concerns | Low |
| CHRO:WS5572 | 0 | No concerns | Low risk | No concerns | Major concerns | No concerns | No concerns | Low |
| CHRO:ZPG | 0 | No concerns | Low risk | No concerns | Major concerns | No concerns | No concerns | Low |
| CIT:CUR | 0 | No concerns | Low risk | No concerns | Major concerns | No concerns | No concerns | Low |
| CIT:EPA | 0 | No concerns | Low risk | No concerns | Major concerns | No concerns | No concerns | Low |
| CIT:EPA+DHA | 0 | No concerns | Low risk | No concerns | Major concerns | No concerns | No concerns | Low |
| CIT:FLUO | 0 | No concerns | Low risk | No concerns | Major concerns | No concerns | No concerns | Low |
| CIT:INU | 0 | No concerns | Low risk | No concerns | Major concerns | No concerns | No concerns | Low |
| CIT:LH57 | 0 | No concerns | Low risk | No concerns | Major concerns | No concerns | No concerns | Low |
| CIT:LI-160 | 0 | No concerns | Low risk | No concerns | Major concerns | No concerns | No concerns | Low |
| CIT:LMF | 0 | Some concerns | Low risk | No concerns | Major concerns | No concerns | No concerns | Very Low |
| CIT:NUT | 0 | No concerns | Low risk | No concerns | Major concerns | No concerns | No concerns | Low |
| CIT:PAR | 0 | No concerns | Low risk | No concerns | Major concerns | No concerns | No concerns | Low |
| CIT:PROB | 0 | No concerns | Low risk | No concerns | Major concerns | No concerns | No concerns | Low |
| CIT:RHO | 0 | No concerns | Low risk | No concerns | Major concerns | No concerns | No concerns | Low |
| CIT:SAF | 0 | No concerns | Low risk | No concerns | Major concerns | No concerns | No concerns | Low |
| CIT:SAFF | 0 | No concerns | Low risk | No concerns | Major concerns | No concerns | No concerns | Low |
| CIT:SER | 0 | No concerns | Low risk | No concerns | Major concerns | No concerns | No concerns | Low |
| CIT:SIL | 0 | No concerns | Low risk | No concerns | Major concerns | No concerns | No concerns | Low |
| CIT:SJW | 0 | No concerns | Low risk | No concerns | Major concerns | No concerns | No concerns | Low |
| CIT:WS5570 | 0 | No concerns | Low risk | No concerns | Major concerns | No concerns | No concerns | Low |
| CIT:WS5572 | 0 | No concerns | Low risk | No concerns | Major concerns | No concerns | No concerns | Low |
| CIT:ZPG | 0 | No concerns | Low risk | No concerns | Major concerns | No concerns | No concerns | Low |
| CUR:EPA | 0 | No concerns | Low risk | No concerns | Major concerns | No concerns | No concerns | Low |
| CUR:EPA+DHA | 0 | No concerns | Low risk | Some concerns | Major concerns | No concerns | No concerns | Very Low |
| CUR:FLUO | 0 | No concerns | Low risk | No concerns | Major concerns | No concerns | No concerns | Low |
| CUR:INU | 0 | No concerns | Low risk | No concerns | Major concerns | No concerns | No concerns | Low |
| CUR:LH57 | 0 | No concerns | Low risk | No concerns | Major concerns | No concerns | No concerns | Low |
| CUR:LI-160 | 0 | No concerns | Low risk | No concerns | Major concerns | No concerns | No concerns | Low |
| CUR:LMF | 0 | No concerns | Low risk | No concerns | Major concerns | No concerns | No concerns | Low |
| CUR:NUT | 0 | No concerns | Low risk | No concerns | Major concerns | No concerns | No concerns | Low |
| CUR:PAR | 0 | No concerns | Low risk | No concerns | Major concerns | No concerns | No concerns | Low |
| CUR:PROB | 0 | No concerns | Low risk | No concerns | Major concerns | No concerns | No concerns | Low |
| CUR:RHO | 0 | No concerns | Low risk | No concerns | Major concerns | No concerns | No concerns | Low |
| CUR:SAF | 0 | No concerns | Low risk | No concerns | Major concerns | No concerns | No concerns | Low |
| CUR:SAFF | 0 | Some concerns | Low risk | No concerns | Major concerns | No concerns | No concerns | Very Low |
| CUR:SER | 0 | No concerns | Low risk | No concerns | Major concerns | No concerns | No concerns | Low |
| CUR:SIL | 0 | No concerns | Low risk | No concerns | Major concerns | No concerns | No concerns | Low |
| CUR:SJW | 0 | No concerns | Low risk | No concerns | Major concerns | No concerns | No concerns | Low |
| CUR:WS5570 | 0 | No concerns | Low risk | No concerns | Major concerns | No concerns | No concerns | Low |
| CUR:WS5572 | 0 | No concerns | Low risk | No concerns | Major concerns | No concerns | No concerns | Low |
| CUR:ZPG | 0 | No concerns | Low risk | No concerns | Major concerns | No concerns | No concerns | Low |
| EPA:EPA+DHA | 0 | No concerns | Low risk | No concerns | Major concerns | No concerns | No concerns | Low |
| EPA:FLUO | 0 | No concerns | Low risk | No concerns | Major concerns | No concerns | No concerns | Low |
| EPA:INU | 0 | No concerns | Low risk | No concerns | Major concerns | No concerns | No concerns | Low |
| EPA:LH57 | 0 | No concerns | Low risk | No concerns | Major concerns | No concerns | No concerns | Low |
| EPA:LI-160 | 0 | No concerns | Low risk | No concerns | Major concerns | No concerns | No concerns | Low |
| EPA:LMF | 0 | No concerns | Low risk | No concerns | Major concerns | No concerns | No concerns | Low |
| EPA:NUT | 0 | No concerns | Low risk | No concerns | Major concerns | No concerns | No concerns | Low |
| EPA:PAR | 0 | No concerns | Low risk | No concerns | Major concerns | No concerns | No concerns | Low |
| EPA:PROB | 0 | No concerns | Low risk | No concerns | Major concerns | No concerns | No concerns | Low |
| EPA:RHO | 0 | No concerns | Low risk | No concerns | Major concerns | No concerns | No concerns | Low |
| EPA:SAF | 0 | No concerns | Low risk | No concerns | Major concerns | No concerns | No concerns | Low |
| EPA:SAFF | 0 | No concerns | Low risk | No concerns | Major concerns | No concerns | No concerns | Low |
| EPA:SER | 0 | No concerns | Low risk | No concerns | Major concerns | No concerns | No concerns | Low |
| EPA:SIL | 0 | Some concerns | Low risk | No concerns | Major concerns | No concerns | No concerns | Very Low |
| EPA:SJW | 0 | No concerns | Low risk | No concerns | Major concerns | No concerns | No concerns | Low |
| EPA:WS5570 | 0 | No concerns | Low risk | No concerns | Major concerns | No concerns | No concerns | Low |
| EPA:WS5572 | 0 | No concerns | Low risk | No concerns | Major concerns | No concerns | No concerns | Low |
| EPA:ZPG | 0 | No concerns | Low risk | No concerns | Major concerns | No concerns | No concerns | Low |
| EPA+DHA:FLUO | 0 | No concerns | Low risk | No concerns | Major concerns | No concerns | No concerns | Low |
| EPA+DHA:INU | 0 | No concerns | Low risk | No concerns | Major concerns | No concerns | No concerns | Low |
| EPA+DHA:LH57 | 0 | No concerns | Low risk | No concerns | Major concerns | No concerns | No concerns | Low |
| EPA+DHA:LI-160 | 0 | No concerns | Low risk | No concerns | Major concerns | No concerns | No concerns | Low |
| EPA+DHA:LMF | 0 | No concerns | Low risk | No concerns | Major concerns | No concerns | No concerns | Low |
| EPA+DHA:NUT | 0 | No concerns | Low risk | No concerns | Major concerns | No concerns | No concerns | Low |
| EPA+DHA:PAR | 0 | No concerns | Low risk | No concerns | Major concerns | No concerns | No concerns | Low |
| EPA+DHA:PROB | 0 | No concerns | Low risk | No concerns | Major concerns | No concerns | No concerns | Low |
| EPA+DHA:RHO | 0 | No concerns | Low risk | No concerns | Major concerns | No concerns | No concerns | Low |
| EPA+DHA:SAF | 0 | No concerns | Low risk | No concerns | Major concerns | No concerns | No concerns | Low |
| EPA+DHA:SAFF | 0 | No concerns | Low risk | No concerns | Major concerns | No concerns | No concerns | Low |
| EPA+DHA:SER | 0 | No concerns | Low risk | No concerns | Major concerns | No concerns | No concerns | Low |
| EPA+DHA:SIL | 0 | Some concerns | Low risk | No concerns | Major concerns | No concerns | No concerns | Very Low |
| EPA+DHA:SJW | 0 | No concerns | Low risk | No concerns | Major concerns | No concerns | No concerns | Low |
| EPA+DHA:WS5570 | 0 | No concerns | Low risk | No concerns | Major concerns | No concerns | No concerns | Low |
| EPA+DHA:WS5572 | 0 | No concerns | Low risk | No concerns | Major concerns | No concerns | No concerns | Low |
| EPA+DHA:ZPG | 0 | No concerns | Low risk | No concerns | Major concerns | No concerns | No concerns | Low |
| FLUO:INU | 0 | No concerns | Low risk | No concerns | Major concerns | No concerns | No concerns | Low |
| FLUO:LI-160 | 0 | No concerns | Low risk | No concerns | Major concerns | No concerns | No concerns | Low |
| FLUO:LMF | 0 | No concerns | Low risk | No concerns | Major concerns | No concerns | No concerns | Low |
| FLUO:NUT | 0 | No concerns | Low risk | Some concerns | Major concerns | No concerns | No concerns | Very Low |
| FLUO:PAR | 0 | No concerns | Low risk | No concerns | Major concerns | No concerns | No concerns | Low |
| FLUO:PROB | 0 | No concerns | Low risk | No concerns | Major concerns | No concerns | No concerns | Low |
| FLUO:PLB | 0 | No concerns | Low risk | No concerns | Major concerns | No concerns | No concerns | Low |
| FLUO:RHO | 0 | No concerns | Low risk | No concerns | Major concerns | No concerns | No concerns | Low |
| FLUO:SAF | 0 | No concerns | Low risk | No concerns | Major concerns | No concerns | No concerns | Low |
| FLUO:SER | 0 | No concerns | Low risk | No concerns | Major concerns | No concerns | No concerns | Low |
| FLUO:SIL | 0 | No concerns | Low risk | No concerns | Major concerns | No concerns | No concerns | Low |
| FLUO:WS5570 | 0 | No concerns | Low risk | No concerns | Major concerns | No concerns | No concerns | Low |
| FLUO:WS5572 | 0 | No concerns | Low risk | No concerns | Major concerns | No concerns | No concerns | Low |
| FLUO:ZPG | 0 | No concerns | Low risk | No concerns | Major concerns | No concerns | No concerns | Low |
| INU:LH57 | 0 | No concerns | Low risk | No concerns | Major concerns | No concerns | No concerns | Low |
| INU:LI-160 | 0 | No concerns | Low risk | No concerns | Major concerns | No concerns | No concerns | Low |
| INU:LMF | 0 | No concerns | Low risk | No concerns | Major concerns | No concerns | No concerns | Low |
| INU:NUT | 0 | No concerns | Low risk | No concerns | Major concerns | No concerns | No concerns | Low |
| INU:PAR | 0 | No concerns | Low risk | No concerns | Major concerns | No concerns | No concerns | Low |
| INU:PROB | 0 | No concerns | Low risk | No concerns | Major concerns | No concerns | No concerns | Low |
| INU:RHO | 0 | No concerns | Low risk | No concerns | Major concerns | No concerns | No concerns | Low |
| INU:SAF | 0 | No concerns | Low risk | No concerns | Major concerns | No concerns | No concerns | Low |
| INU:SAFF | 0 | No concerns | Low risk | No concerns | Major concerns | No concerns | No concerns | Low |
| INU:SER | 0 | No concerns | Low risk | No concerns | Major concerns | No concerns | No concerns | Low |
| INU:SIL | 0 | No concerns | Low risk | No concerns | Major concerns | No concerns | No concerns | Low |
| INU:SJW | 0 | No concerns | Low risk | No concerns | Major concerns | No concerns | No concerns | Low |
| INU:WS5570 | 0 | No concerns | Low risk | No concerns | Major concerns | No concerns | No concerns | Low |
| INU:WS5572 | 0 | No concerns | Low risk | No concerns | Major concerns | No concerns | No concerns | Low |
| INU:ZPG | 0 | No concerns | Low risk | No concerns | Major concerns | No concerns | No concerns | Low |
| LH57:LI-160 | 0 | No concerns | Low risk | No concerns | Major concerns | No concerns | No concerns | Low |
| LH57:LMF | 0 | No concerns | Low risk | No concerns | Major concerns | No concerns | No concerns | Low |
| LH57:NUT | 0 | No concerns | Low risk | No concerns | Major concerns | No concerns | No concerns | Low |
| LH57:PAR | 0 | No concerns | Low risk | No concerns | Major concerns | No concerns | No concerns | Low |
| LH57:PROB | 0 | No concerns | Low risk | No concerns | Major concerns | No concerns | No concerns | Low |
| LH57:PLB | 0 | No concerns | Low risk | No concerns | Major concerns | No concerns | No concerns | Low |
| LH57:RHO | 0 | No concerns | Low risk | No concerns | Major concerns | No concerns | No concerns | Low |
| LH57:SAF | 0 | No concerns | Low risk | No concerns | Major concerns | No concerns | No concerns | Low |
| LH57:SAFF | 0 | No concerns | Low risk | Some concerns | Major concerns | No concerns | No concerns | Very Low |
| LH57:SER | 0 | No concerns | Low risk | No concerns | Major concerns | No concerns | No concerns | Low |
| LH57:SIL | 0 | No concerns | Low risk | No concerns | Major concerns | No concerns | No concerns | Low |
| LH57:SJW | 0 | No concerns | Low risk | No concerns | Major concerns | No concerns | No concerns | Low |
| LH57:WS5570 | 0 | No concerns | Low risk | No concerns | Major concerns | No concerns | No concerns | Low |
| LH57:WS5572 | 0 | Some concerns | Low risk | No concerns | Major concerns | No concerns | No concerns | Very Low |
| LH57:ZPG | 0 | No concerns | Low risk | No concerns | Major concerns | No concerns | No concerns | Low |
| LI-160:LMF | 0 | No concerns | Low risk | No concerns | Major concerns | No concerns | No concerns | Low |
| LI-160:NUT | 0 | No concerns | Low risk | No concerns | Major concerns | No concerns | No concerns | Low |
| LI-160:PAR | 0 | No concerns | Low risk | No concerns | Major concerns | No concerns | No concerns | Low |
| LI-160:PROB | 0 | No concerns | Low risk | No concerns | Major concerns | No concerns | No concerns | Low |
| LI-160:RHO | 0 | No concerns | Low risk | No concerns | Major concerns | No concerns | No concerns | Low |
| LI-160:SAF | 0 | No concerns | Low risk | No concerns | Major concerns | No concerns | No concerns | Low |
| LI-160:SAFF | 0 | No concerns | Low risk | No concerns | Major concerns | No concerns | No concerns | Low |
| LI-160:SER | 0 | No concerns | Low risk | No concerns | Major concerns | No concerns | No concerns | Low |
| LI-160:SIL | 0 | No concerns | Low risk | No concerns | Major concerns | No concerns | No concerns | Low |
| LI-160:SJW | 0 | No concerns | Low risk | No concerns | Major concerns | No concerns | No concerns | Low |
| LI-160:WS5570 | 0 | No concerns | Low risk | No concerns | Major concerns | No concerns | No concerns | Low |
| LI-160:WS5572 | 0 | Some concerns | Low risk | No concerns | Major concerns | No concerns | No concerns | Very Low |
| LI-160:ZPG | 0 | No concerns | Low risk | No concerns | Major concerns | No concerns | No concerns | Low |
| LMF:NUT | 0 | No concerns | Low risk | No concerns | Major concerns | No concerns | No concerns | Low |
| LMF:PAR | 0 | No concerns | Low risk | No concerns | Major concerns | No concerns | No concerns | Low |
| LMF:PROB | 0 | No concerns | Low risk | No concerns | Major concerns | No concerns | No concerns | Low |
| LMF:RHO | 0 | No concerns | Low risk | No concerns | Major concerns | No concerns | No concerns | Low |
| LMF:SAF | 0 | No concerns | Low risk | No concerns | Major concerns | No concerns | No concerns | Low |
| LMF:SAFF | 0 | No concerns | Low risk | No concerns | Major concerns | No concerns | No concerns | Low |
| LMF:SER | 0 | No concerns | Low risk | No concerns | Major concerns | No concerns | No concerns | Low |
| LMF:SIL | 0 | No concerns | Low risk | No concerns | Major concerns | No concerns | No concerns | Low |
| LMF:SJW | 0 | No concerns | Low risk | No concerns | Major concerns | No concerns | No concerns | Low |
| LMF:WS5570 | 0 | No concerns | Low risk | No concerns | Major concerns | No concerns | No concerns | Low |
| LMF:WS5572 | 0 | No concerns | Low risk | No concerns | Major concerns | No concerns | No concerns | Low |
| LMF:ZPG | 0 | No concerns | Low risk | No concerns | Major concerns | No concerns | No concerns | Low |
| NUT:PAR | 0 | No concerns | Low risk | No concerns | Major concerns | No concerns | No concerns | Low |
| NUT:PROB | 0 | No concerns | Low risk | No concerns | Major concerns | No concerns | No concerns | Low |
| NUT:RHO | 0 | No concerns | Low risk | No concerns | Major concerns | No concerns | No concerns | Low |
| NUT:SAF | 0 | No concerns | Low risk | No concerns | Major concerns | No concerns | No concerns | Low |
| NUT:SAFF | 0 | No concerns | Low risk | No concerns | Major concerns | No concerns | No concerns | Low |
| NUT:SER | 0 | No concerns | Low risk | Some concerns | Major concerns | No concerns | No concerns | Very Low |
| NUT:SIL | 0 | No concerns | Low risk | No concerns | Major concerns | No concerns | No concerns | Low |
| NUT:SJW | 0 | No concerns | Low risk | No concerns | Major concerns | No concerns | No concerns | Low |
| NUT:WS5570 | 0 | No concerns | Low risk | No concerns | Major concerns | No concerns | No concerns | Low |
| NUT:WS5572 | 0 | No concerns | Low risk | No concerns | Major concerns | No concerns | No concerns | Low |
| NUT:ZPG | 0 | No concerns | Low risk | No concerns | Major concerns | No concerns | No concerns | Low |
| PAR:PROB | 0 | No concerns | Low risk | No concerns | Major concerns | No concerns | No concerns | Low |
| PAR:PLB | 0 | No concerns | Low risk | No concerns | Major concerns | No concerns | No concerns | Low |
| PAR:RHO | 0 | No concerns | Low risk | No concerns | Major concerns | No concerns | No concerns | Low |
| PAR:SAF | 0 | No concerns | Low risk | No concerns | Major concerns | No concerns | No concerns | Low |
| PAR:SAFF | 0 | No concerns | Low risk | No concerns | Major concerns | No concerns | No concerns | Low |
| PAR:SER | 0 | No concerns | Low risk | No concerns | Major concerns | No concerns | No concerns | Low |
| PAR:SIL | 0 | No concerns | Low risk | No concerns | Major concerns | No concerns | No concerns | Low |
| PAR:SJW | 0 | No concerns | Low risk | No concerns | Major concerns | No concerns | No concerns | Low |
| PAR:WS5572 | 0 | No concerns | Low risk | No concerns | Major concerns | No concerns | No concerns | Low |
| PAR:ZPG | 0 | No concerns | Low risk | No concerns | Major concerns | No concerns | No concerns | Low |
| PROB:RHO | 0 | No concerns | Low risk | No concerns | Major concerns | No concerns | No concerns | Low |
| PROB:SAF | 0 | No concerns | Low risk | No concerns | Major concerns | No concerns | No concerns | Low |
| PROB:SER | 0 | No concerns | Low risk | No concerns | Major concerns | No concerns | No concerns | Low |
| PROB:SIL | 0 | No concerns | Low risk | No concerns | Major concerns | No concerns | No concerns | Low |
| PROB:SJW | 0 | No concerns | Low risk | No concerns | Major concerns | No concerns | No concerns | Low |
| PROB:WS5570 | 0 | No concerns | Low risk | Some concerns | Major concerns | No concerns | No concerns | Very Low |
| PROB:ZPG | 0 | No concerns | Low risk | No concerns | Major concerns | No concerns | No concerns | Low |
| PLB:SAFF | 0 | No concerns | Low risk | No concerns | Major concerns | No concerns | No concerns | Low |
| PLB:SER | 0 | No concerns | Low risk | No concerns | Major concerns | No concerns | No concerns | Low |
| PLB:WS5572 | 0 | No concerns | Low risk | No concerns | Major concerns | No concerns | No concerns | Low |
| RHO:SAF | 0 | No concerns | Low risk | No concerns | Major concerns | No concerns | No concerns | Low |
| RHO:SAFF | 0 | No concerns | Low risk | No concerns | Major concerns | No concerns | No concerns | Low |
| RHO:SER | 0 | No concerns | Low risk | No concerns | Major concerns | No concerns | No concerns | Low |
| RHO:SIL | 0 | No concerns | Low risk | No concerns | Major concerns | No concerns | No concerns | Low |
| RHO:SJW | 0 | No concerns | Low risk | No concerns | Major concerns | No concerns | No concerns | Low |
| RHO:WS5570 | 0 | No concerns | Low risk | No concerns | Major concerns | No concerns | No concerns | Low |
| RHO:WS5572 | 0 | No concerns | Low risk | No concerns | Major concerns | No concerns | No concerns | Low |
| RHO:ZPG | 0 | Some concerns | Low risk | No concerns | Major concerns | No concerns | No concerns | Very Low |
| SAF:SAFF | 0 | No concerns | Low risk | No concerns | Major concerns | No concerns | No concerns | Low |
| SAF:SER | 0 | No concerns | Low risk | Some concerns | Major concerns | No concerns | No concerns | Very Low |
| SAF:SIL | 0 | No concerns | Low risk | No concerns | Major concerns | No concerns | No concerns | Low |
| SAF:SJW | 0 | No concerns | Low risk | No concerns | Major concerns | No concerns | No concerns | Low |
| SAF:WS5570 | 0 | No concerns | Low risk | No concerns | Major concerns | No concerns | No concerns | Low |
| SAF:WS5572 | 0 | No concerns | Low risk | No concerns | Major concerns | No concerns | No concerns | Low |
| SAF:ZPG | 0 | No concerns | Low risk | No concerns | Major concerns | No concerns | No concerns | Low |
| SAFF:SIL | 0 | No concerns | Low risk | No concerns | Major concerns | No concerns | No concerns | Low |
| SAFF:SJW | 0 | No concerns | Low risk | No concerns | Major concerns | No concerns | No concerns | Low |
| SAFF:WS5570 | 0 | No concerns | Low risk | No concerns | Major concerns | No concerns | No concerns | Low |
| SAFF:WS5572 | 0 | No concerns | Low risk | No concerns | Major concerns | No concerns | No concerns | Low |
| SAFF:ZPG | 0 | No concerns | Low risk | No concerns | Major concerns | No concerns | No concerns | Low |
| SER:SIL | 0 | No concerns | Low risk | No concerns | Major concerns | No concerns | No concerns | Low |
| SER:SJW | 0 | No concerns | Low risk | No concerns | Major concerns | No concerns | No concerns | Low |
| SER:WS5570 | 0 | No concerns | Low risk | No concerns | Major concerns | No concerns | No concerns | Low |
| SER:WS5572 | 0 | No concerns | Low risk | No concerns | Major concerns | No concerns | No concerns | Low |
| SER:ZPG | 0 | No concerns | Low risk | No concerns | Major concerns | No concerns | No concerns | Low |
| SIL:SJW | 0 | No concerns | Low risk | No concerns | Major concerns | No concerns | No concerns | Low |
| SIL:WS5570 | 0 | No concerns | Low risk | No concerns | Major concerns | No concerns | No concerns | Low |
| SIL:WS5572 | 0 | No concerns | Low risk | No concerns | Major concerns | No concerns | No concerns | Low |
| SIL:ZPG | 0 | No concerns | Low risk | No concerns | Major concerns | No concerns | No concerns | Low |
| SJW:WS5570 | 0 | No concerns | Low risk | No concerns | Major concerns | No concerns | No concerns | Low |
| SJW:WS5572 | 0 | No concerns | Low risk | No concerns | Major concerns | No concerns | No concerns | Low |
| SJW:ZPG | 0 | No concerns | Low risk | No concerns | Major concerns | No concerns | No concerns | Low |
| WS5570:WS5572 | 0 | No concerns | Low risk | Some concerns | Major concerns | No concerns | No concerns | Very Low |
| WS5570:ZPG | 0 | No concerns | Low risk | No concerns | Major concerns | No concerns | No concerns | Low |
| WS5572:ZPG | 0 | Some concerns | Low risk | No concerns | Major concerns | No concerns | No concerns | Very Low |

# **S12:** Dropouts, adverse outcomes, safety related outcomes.

| **Author(s), year** | **Treatment** | **Mean dose** | **Acceptability (drops-out due to any cause)** | **Adverse events, not necessarily leading to drop-out (n out of total)** | **Safety-related outcomes (n out of total)** | **Inefficacy (n out of total)** | **Tolerability (n out of total)** |
| --- | --- | --- | --- | --- | --- | --- | --- |
| ^455^ | FOIL | 2 ×  1000mg/day | 2 out of 19 (1 pregnancy, 1 migration) | 0 | 0 | 0 | 0 |
| ^455^ | KOIL | 2 ×  1000mg/day | 2 out of 19 (1 pregnancy, 1 migration) | 0 | 0 | 0 | 0 |
| ^455^M. et al., 2025 | PLB |  | 3 out of 19 (2 personal reasons, 1 work issues) | 0 | 0 | 0 | 0 |
| ^456^ | SAFF | 60mg/day | 3 out of 25 (no reason reported) | somnolence (1 out of 25) | 0 | 0 | 0 |
| ^456^ | SER | 100mg/day | 5 out of 25 (3 no reason reported, 2 side effects) | headache (3 out of 25), somnolence (3 out of 25), dizziness (2 out of 25), sleep impairment (2 out of 25) | 0 | 0 | 2 out of 25 |
| ^457^ | SAFF | 30mg/day | 1 out of 20 (consent withdraw) | appetite decrease (5 out of 20), headache (2 out of 20), nausea (3 out of 20), insomnia (3 out of 20), increased sweating (2 out of 20), tremor (2 out of 20), sexual dysfunction (3 out of 20), anxiety (4 out of 20), increased appetite (1 out of 20) | heart pounding (2 out of 20) | 0 | 0 |
| ^457^ | FLUO | 20mg/day | 1 out of 20 (consent withdraw) | appetite decrease (4 out of 20), headache (5 out of 20), nausea (4 out of 20), insomnia (3 out of 20), increased sweating (3 out of 20), tremor (5 out of 20), sexual dysfunction (5 out of 20), anxiety (7 out of 20), increased appetite (3 out of 20), | heart pounding (2 out of 20) | 0 | 0 |
| ^458^ | SAFF | 30 mg/day | 0 | appetite decrease (2 out of 15), headache (3 out of 15), constipation (2 out of 15), dry mouth (1 out of 15), anxiety (4 out of 15), increased appetite (1 out of 15), restlessness (2 out of 15), urinary retention (1 out of 15) | 0 | 0 | 0 |
| ^458^ | IMI | 100 mg/day | 0 | headache (2 out of 15), constipation (5 out of 15), dry mouth (7 out of 15), energy decreased (6 out of 15), anxiety (1 out of 15), increased appetite (5 out of 15), restlessness (1 out of 15), urinary retention (5 out of 15) | 0 | 0 | 0 |
| ^459^ | SAF | 15mg 2/day | 4 out of 36 lost at follow-up (1 for sleep disturbance and increased appetite, 3 became unreachable), 8 out of 36 discontinued intervention at 2nd and 4th weeks of trial and  excluded from analysis (decreased willingness to continue) | sleep impairment (1 out of 36), anxiety (1 out of 36), | 0 | 0 | 1 out of 36 (nightmares and anxiety) |
| ^459^ | PLB | 2/day | 4 out of 37 lost at follow-up (1 for nightmare and anxiety, 2 became unreachable), 6 out of 37 Discontinued intervention at 2nd and 4th weeks of trial and  excluded from analysis (decreased willingness to continue) | sleep impairment (1 out of 37), increased appetite (1 out of 37), | 0 | 0 | 1 out of 37 (sleep disturbance and increased appetite) |
| ^460^ | MEL | 3 mg/day | 5 out of 26 (4 immigration or being unreachable, 1 unwilling to continue) | headache (2 out of 21), somnolence (1 out of 21), sleep impairment (1 out of 21) | 0 | 0 | 0 |
| ^460^ | PLB |  | 4 out of 26 (2 address change or being unreachable, 2 unwilling to continue) | headache (1 out of 22), nausea (2 out of 22), insomnia (1 out of 22) | 0 | 0 | 0 |
| ^460^ | LAV | 60 drops/day | 1 out of 16 (non compliance) | headache (7 out of 15), constipation (8 out of 15), abdominal pain (1 out of 15), dry mouth (2 out of 15), anxiety (2 out of 15), increased appetite (8 out of 15), urinary retention (2 out of 15) | 0 | 0 | 0 |
| ^460^ | IMI | 100mg/day | 1 out of 16 (non compliance) | headache (1 out of 15), constipation (5 out of 15), dry mouth (9 out of 15), anxiety (1 out of 15), increased appetite (7 out of 15), urinary retention (8 out of 15) | heart pounding (3 out of 15) | 0 | 0 |
| ^460^ | LAV+IMI | 60 drops/day+100mg/day | 1 out of 16 (clinical deterioration) | 0 | 0 | 0 | 0 |
| ^461^ | PROB | 1 cp/day | 3 out of 20 (personal reasons) | 0 | 0 | 0 | 0 |
| ^461^ | PLB |  | 2 out of 20 (personal reasons) | 0 | 0 | 0 | 0 |
| ^599^ | FOL+vitB12+vitB6+CTP | 0.5mg+25mg+2mg+20-40mg | 15 out of 77 | headache (10 out of 62), constipation (8 out of 62), diarrhea (7 out of 62), nausea (4 out of 62), dry mouth (19 out of 62), tremor (7 out of 62), sexual dysfunction (12 out of 62), sleep impairment (11 out of 62), anxiety (9 out of 62) | 0 | NA | NA |
| ^599^ | CTP+PLB | 20-40mg | 10 out of 76 | headache (16 out of 66), constipation (8 out of 66), diarrhea (4 out of 66), nausea (7 out of 66), dry mouth (14 out of 66), tremor (6 out of 66), sexual dysfunction (8 out of 66), sleep impairment (7 out of 66), anxiety (10 out of 66) | 0 | NA | NA |
| ^462^ | vitD3-Ca | 50000IU+500mg/day | 1 out of 27 (personal reasons) | 0 | 0 | 0 | 0 |
| ^462^ | vitD3 | 50000IU/day | 1 out of 27 (personal reasons) | 0 | 0 | 0 | 0 |
| ^462^ | PLB | NA | 3 out of 27 (personal reasons) | 0 | 0 | 0 | 0 |
| ^463^ | EACS | 30ml | 4 out of 25 (1 drug intolerance, 3 personal causes) | diarrhea (1 out of 21), insomnia (1 out of 21) | heart pounding (1 out of 21) | 0 | 1 |
| ^463^ | CTP | 20mg/day | 4 out of 25 (3 drug intolerance, 1 personal causes) | headache (3 out of 21), nausea (1 out of 21), reflux (3 out of 21), somnolence (3 out of 21), insomnia (1 out of 21), dizziness (1 out of 21), unpleasant taste (1 out of 21), poor memory (1 out of 21), blurrred vision (1 out of 21), sexual dysfunction (2 out of 21) | heart pounding (3 out of 21) | 0 | 3 |
| ^464^ | MEL | 2g 1/day | 1 out of 16 (personal reason) | appetite decrease (1 out of 16), headache (1 out of 16), somnolence (2 out of 16), insomnia (1 out of 16), dizziness (1 out of 16), dry mouth (1 out of 16), sexual disfunction (1 out of 16), anxiety (1 out of 16), increased appetite (2 out of 16), | 0 | 0 | 0 |
| ^464^ | LAV | 2g 1/day | 2 out of 17 (1 for personal reason, 1 for drowsiness) | headache (2 out of 17), somnolence (2 out of 17), dizziness (1 out of 17), dry mouth (2 out of 17), anxiety (1 out of 17), increased appetite (3 out of 17), | 0 | 0 | 1 out of 17 (drowsiness) |
| ^464^ | FLUO | 20mg 1/day | 2 out of 17 (1 for sexual disfunction, 1 for diarrhea) | appetite decrease (3 out of 17), headache (4 out of 17), diarrhea (1 out of 17), insomnia (3 out of 17), dizziness (5 out of 17), dry mouth (1 out of 17), sexual dysfunction (3 out of 17), anxiety (5 out of 17), increased appetite (1 out of 17), | 0 | 0 | 2 out of 17 (1 for sexual disfunction, 1 for diarrhea) |
| ^465^ | BIOKULT | 4 cp/day | 0 | 0 | 0 | 0 | 0 |
| ^465^ | PLB |  | 0 | 0 | 0 | 0 | 0 |
| ^466^ | ESC+FOL | 10 mg+2,5 mg | 0 | 0 | 0 | 0 | 0 |
| ^466^ | ESC | 10mg | 0 | 0 | 0 | 0 | 0 |
| ^467^ | SJW | 300mg/day | 6 out of 35 (3 failed to report for scheduled visit after day 0, 2 adverse events, 1 simultaneous intake of other drugs) | appetite decrease (2 out of 35), headache (4 out of 35), constipation (2 out of 35), nausea (2 out of 35), dizziness (3 out of 35), sexual dysfunction (1 out of 35), restlessness (1 out of 35) | 0 | 0 | 2 |
| ^467^ | FLUO | 40mg/day | 3 out of 35 ( 1 failed to report for scheduled visit, 2 adverse events) | appetite decrease (1 out of 35), headache (5 out of 35), constipation (1 out of 35), nausea (2 out of 35), dizziness (7 out of 35), sexual dysfunction (1 out of 35), restlessness (5 out of 35) | 0 | 0 | 2 |
| ^468^ | SAMe | 200-400 mg/day | 2 out of 11 | 0 | 0 | 0 | 0 |
| ^468^ | IMI | 150 mg/day | 2 out of 11 | 0 | 0 | 0 | 0 |
| ^469^ | CUR | 500mg/day | 1 out of 20 | 0 | 0 | 0 | 0 |
| ^469^ | PLB |  | 0 | 0 | 0 | 0 | 0 |
| ^470^ | NAC+TAU | 2000mg/day | 19 out of 135 (4 lost to follow-up, 2 non compliant, 10 withdrew consent, 2 withdrawal by investigator, 1 adverse effect) | gastrointestinal discomfort (43 out of 125) | 0 | 0 | 1 |
| ^470^ | PLB+TAU | NA | 26 out of 134 (4 lost to follow-up, 6 non compliant, 12 withdrew consent, 2 withdrawal by investigator, 2 adverse effect) | gastrointestinal discomfort (23 out of 127) | 0 | 0 | 2 |
| ^471^ | EPA+DHA | 4000 mg/day | 0 | 0 | 0 | 0 | 0 |
| ^471^ | PLB |  | 0 | 0 | 0 | 0 | 0 |
| ^472^ | LI-160 | 900mg/day | 9 out of 57 (3 prior to first follow-up) | headache (19 out of 57), limb edema (3 out of 57), nausea (9 out of 57), somnolence (8 out of 57), insomnia (7 out of 57), muscle pain/aches (5 out of 57), gastrointestinal discomfort (9 out of 57), dry mouth (10 out of 57), joint pain (4 out of 57), upper respiratory tract infection (6 out of 57), urinary tract infection (5 out of 57) | 0 | 0 | 4 |
| ^472^ | FLUO | 20mg/day | 8 out of 56 (2 prior to first follow-up) | headache (12 out of 56), limb edema (4 out of 56), nausea (7 out of 56), somnolence (3 out of 56), insomnia (6 out of 56), muscle pain/aches (3 out of 56), gastrointestinal discomfort (5 out of 56), dry mouth (4 out of 56), skin rash (5 out of 56), joint pain (7 out of 56), upper respiratory tract infection (4 out of 56), urinary tract infection (6 out of 56) | 0 | 0 | 4 |
| ^472^ | PLB | NA | 5 out of 57 (2 prior to first follow-up) | headache (12 out of 57), limb edema (7 out of 57), nausea (4 out of 57), somnolence (9 out of 57), insomnia (5 out of 57), muscle pain/aches (4 out of 57), gastrointestinal discomfort (10 out of 57), dry mouth (6 out of 57), joint pain (2 out of 57), upper respiratory tract infection (7 out of 57), urinary tract infection (5 out of 57) | 0 | 0 | 2 |
| ^473^ | EPA | 1g/day | 2 out of 13 (1 lost to follow-up, 1 allergy) | 0 | 0 | 0 | 1 (allergic reaction to fish oil) |
| ^473^ | PLB |  | 0 out of 12 | 0 | 0 | 0 | 0 |
| ^474^ | LI-160 | 900mg/day | 5 out of 13 (2 for adverse events; 3 withdrew consent) | headache (2 out of 15), dizziness (1 out of 15) | 0 | 0 | 2 |
| ^474^ | SER | 75mg/day | 3 out of 15 (2 for adverse events; 1 withdrew consent) | headache (2 out of 13), dizziness (1 out of 13), nausea (1 out of 13) | 0 | 0 | 2 |
| ^475^ | SER + EPA | 50 mg/day + 2g/day | 3 out of 71 (1 severe indigestion, 1 unremitting diarrhea, 1 frequent bowel movements) | headache (11 out of 71), diarrhea (35 out of 71), nausea (35 out of 71), flatulence (35 out of 71), dizziness (11 out of 71), gastrointestinal discomfort (35 out of 71), tremor (11 out of 71), | 0 | 0 | 3 out of 71 (1 severe indigestion, 1 unremitting diarrhea, 1 frequent bowel movements) |
| ^475^ | SER + PLB | 50 mg/day | 0 | headache (14 out of 73), diarrhea (33 out of 73), nausea (33 out of 73), flatulence (33 out of 73), dizziness (14 out of 73), gastrointestinal discomfort (33 out of 73), tremor (14 out of 73), | 0 | 0 | 0 |
| ^476^ | SER+EPA+DHA | 50mg+930mg+750mg/day | NA | 0 | 0 | NA | NA |
| ^476^ | SER+PLB | 50mg/day | NA | 0 | 0 | NA | NA |
| ^477^ | SAMe | 200mg/day | 0 | 0 | 0 | 0 | 0 |
| ^477^ | PLB |  | 1 out of 30 (RA symptoms worsened) | 0 | 0 | 0 | 0 |
| ^478^ | OMEGA3 | 2g EPA+1g DHA/day | 0 | 0 | 0 | 0 | 0 |
| ^478^ | PLB |  | 0 | 0 | 0 | 0 | 0 |
| ^479^ | EPA+DHA | 1,2g+1g/day | 0 | 0 | 0 | 0 | 0 |
| ^479^ | PLB |  | 0 | 0 | 0 | 0 | 0 |
| ^480^ | SAFF | 2 cp/day | 0 | constipation (1 out of 20), dry mouth (1 out of 20) | 0 | 0 | 0 |
| ^480^ | FLUO |  | 0 | constipation (4 out of 20), somnolence (1 out of 20), dry mouth (2 out of 20) | 0 | 0 | 0 |
| ^584^ | FOL+FLUO | 500mcg/day+20mg/day | 11 out of 62 (3 AEs, 7 non attendance, 1 protocol violation) | 0 | 0 | 0 | 3 out of 62 |
| ^584^ | PLB+FLUO | 20mg/day | 7 out of 65 (6 AEs, 1 non attendance) | 0 | 0 | 0 | 6 out of 65 |
| ^481^ | RHO | 340 or 680mg/day | 0 | 0 | 0 | 0 | 0 |
| ^481^ | PLB | NA | 0 | dry mouth (1 out of 29), stiffness (1 out of 29) | 0 | 0 | 0 |
| ^482^ | CHRO | 600 mcg | 0 | insomnia (2 out of 10) | 0 | 0 | 0 |
| ^482^ | PLB |  | 0 | constipation (2 out of 5) | 0 | 0 | 0 |
| ^483^ | LI-160 | 900-1500mg/day | 31 out of 113 (8 lost to follow-up, 7 withdrew consent, 6 insufficient/inadequate response, 2 adverse events, 8 protocol violation) | diarrhea (23 out of 113), nausea (21 out of 113), fluid retention (21 out of 113), increased sweating (20 out of 113), sexual dysfunction (28 out of 113), pollakiuria (30 out of 113), failing memory (28 out of 113) | 0 | 6 | 2 |
| ^483^ | SER | 50-100mg/day | 32 out of 111 (10 lost to follow-up, 8 withdrew consent, 7 insufficient/inadequate response, 5 adverse events, 2 protocol violation) | diarrhea (42 out of 111), nausea (41 out of 111), fluid retention (9 out of 111), increased sweating (32 out of 111), sexual dysfunction (35 out of 111), pollakiuria (23 out of 111), failing memory (13 out of 111) | 0 | 7 | 5 |
| ^483^ | PLB |  | 32 out of 116 (7 lost to follow-up, 8 withdrew consent, 11 insufficient/inadequate response, 3 adverse events, 3 protocol violation) | diarrhea (22 out of 116), nausea (24 out of 116), fluid retention (9 out of 116), increased sweating (14 out of 116), sexual dysfunction (16 out of 116), pollakiuria (13 out of 116), failing memory (26 out of 116) | 0 | 11 | 3 |
| ^484^ | SAMe | 200mg/day | 1 out of 20 (hospitalization) | 0 | 0 | 0 | 0 |
| ^484^ | PLB |  | 3 out of 20 (1 pregnancy, 2 refused to continue) | 0 | 0 | 0 | 0 |
| ^485^ | TEN | 1 cp/day | 0 | 0 | 0 | 0 | 0 |
| ^485^ | PLB |  | 0 | 0 | 0 | 0 | 0 |
| ^486^ | CHRO | 600mcg/day | 20 out of 70 deviated from protocol | headache (2 out of 70), constipation (3 out of 70), limb edema (2 out of 70), insomnia (2 out of 70), dry mouth (5 out of 70), blurred vision (3 out of 70), sleep impairment (3 out of 70), pollakiuria (2 out of 70), difficulties in concentration (4 out of 70), thirst (4 out of 70), dysgeusia (2 out of 70) | 0 | 0 | 0 |
| ^486^ | PROB | NA | 15 out of 40 deviated from protocol | headache (1 out of 40), insomnia (2 out of 40), increased sweating (1 out of 40), dry mouth (3 out of 40), sleep impairment (1 out of 40), pollakiuria (1 out of 40), difficulties in concentration (1 out of 40) | 0 | 0 | 0 |
| ^487^ | SCU | 20mg 1/day | 0 | 0 | 0 | 0 | 0 |
| ^487^ | SAFF+SCU | 20+30mg/day | 0 | 0 | 0 | 0 | 0 |
| ^487^ | SAFF | 30mg/day | 0 | 0 | 0 | 0 | 0 |
| ^487^ | PLB |  | 0 | 0 | 0 | 0 | 0 |
| ^488^ | LI-160 | 900mg/day | 18 out of 45 | headache (19 out of 45), limb edema (3 out of 45), nausea (9 out of 45), somnolence (8 out of 45), insomnia (7 out of 45), muscle pain/aches (5 out of 45), gastrointestinal discomfort (9 out of 45), dry mouth (10 out of 45), joint pain (4 out of 45), upper respiratory tract infection (6 out of 45), urinary tract infection (5 out of 45) | 0 | NA | 1 (heroin overdose and related hospitalization) |
| ^488^ | FLUO | 20mg/day | 23 out of 47 | headache (12 out of 47), limb edema (4 out of 47), nausea (7 out of 47), somnolence (3 out of 47), insomnia (6 out of 47), muscle pain/aches (3 out of 47), gastrointestinal discomfort (5 out of 47), dry mouth (4 out of 47), skin rash (5 out of 47), joint pain (7 out of 47), upper respiratory tract infection (4 out of 47), urinary tract infection (6 out of 47) | 0 | NA | 2 |
| ^488^ | PLB | NA | 22 out of 43 | headache (12 out of 43), limb edema (7 out of 43), nausea (4 out of 43), somnolence (9 out of 43), insomnia (5 out of 43), muscle pain/aches (4 out of 43), gastrointestinal discomfort (10 out of 43), dry mouth (6 out of 43), joint pain (2 out of 43), upper respiratory tract infection (7 out of 43), urinary tract infection (5 out of 43) | 0 | NA | 0 |
| ^489^ | NEP+TAU | 400mg/day | 5 out of 20 | 0 | 0 | NA | NA |
| ^489^ | CUS+TAU | 500mg/day | 11 out of 24 | 0 | 0 | NA | NA |
| ^489^ | TAU | NA | 6 out of 21 | 0 | 0 | NA | NA |
| ^579^ | LAV +PLB | 5mll/day | 3 out of 28 (patients unreachable) | constipation (1 out of 25), somnolence (5 out of 25), insomnia (1 out of 25) | 0 | 0 | 0 |
| ^579^ | CTP + PLB | 20mg/day | 3 out of 28 (2 withdrew consent, 1 gastritis) | headache (2 out of 25), nausea (1 out of 25), somnolence (3 out of 25), insomnia (2 out of 25), dry mouth (2 out of 25), sexual dysfunction (3 out of 25), gastritis (2 out of 25) | 0 | 0 | 1 out of 28 (gastritis) |
| ^490^ | EPA+DHA | 1,9g/day | 3 out of 31 | appetite decrease (1 out of 28), headache (1 out of 28), nausea (1 out of 28), somnolence (1 out of 28), insomnia (1 out of 28), dizziness (2 out of 28), gastrointestinal discomfort (3 out of 28), dry mouth (1 out of 28), weakness (1 out of 28), sexual dysfunction (3 out of 28), restlessness (2 out of 28) | 0 | 0 | 0 |
| ^490^ | PLB |  | 5 out of 28 | nausea (2 out of 23), somnolence (1 out of 23), dizziness (2 out of 23), gastrointestinal discomfort (3 out of 23), weakness (1 out of 23) | 0 | 0 | 0 |
| ^491^ | ZE117 | 500mg/day | 0 | headache (3 out of 126), nausea (9 out of 126), insomnia (3 out of 126), dizziness (4 out of 126), abdominal pain (2 out of 126) | 0 | 0 | 0 |
| ^491^ | FLUO | 20 mg/day | 0 | headache (7 out of 114), nausea (6 out of 114), insomnia (2 out of 114), dizziness (4 out of 114), abdominal pain (4 out of 114) | 0 | 0 | 1 |
| ^492^ | SER+PLB | NA | 2 out of 33 (of whom 1 excluded from analysis for lack of postbaseline data) | appetite decrease (1 out of 32), headache (1 out of 32), nausea (1 out of 32), somnolence (1 out of 32), insomnia (1 out of 32), dizziness (2 out of 32), gastrointestinal discomfort (3 out of 32), dry mouth (1 out of 32), weakness (1 out of 32), sexual dysfunction (3 out of 32), restlessness (2 out of 32) | heart pounding (1 out of 32) | 0 | 0 |
| ^492^ | RHO+SER | 0.3-0.6g/day | 2 out of 67 (of whom 1 excluded from analysis for lack of postbaseline data) | nausea (2 out of 66), somnolence (1 out of 66), dizziness (2 out of 66), gastrointestinal discomfort (3 out of 66), weakness (1 out of 66) | 0 | 0 | 0 |
| ^493^ | LI-160 | 900-1200mg/day | 2 out of 43 (did not provide information) | 0 | 0 | 0 | 0 |
| ^493^ | PLB |  | 1 out of 55 (did not provide information) | 0 | 0 | 0 | 0 |
| ^494^ | SER + PLB | 100mg2/day | 6 out of 25 (6 withdrawn consent) | appetite decrease (2 out of 19), constipation (3 out of 19), somnsolence (3 out of 19) | 0 | 0 | 0 |
| ^494^ | SER+CIN | 100mg2/day+500mg/day | 2 out of 25 (2 withdrawn consent) | somnolence (2 out of 23), dizziness (1 out of 23), dry mouth (1 out of 23), restlessness (1 out of 23) | 0 | 0 | 0 |
| ^495^ | SAFF | 30mg/day | 3 out of 33 (withdrew consent) | headache (2 out of 30), nausea (2 out of 30), vomiting (2 out of 30) | 0 | 0 | 0 |
| ^495^ | CTP | 40mg/day | 3 out of 33 (withdrew consent) | headache (2 out of 30), nausea (2 out of 30), vomiting (2 out of 30), restlessness (3 out of 30), gastritis (2 out of 30), dizziness (5 out of 30) | heart pounding (3 out of 30) | 0 | 0 |
| ^582^ | THI+FLUO | 300mg/day+20mg/day | 0 | 0 | 0 | 0 | 0 |
| ^582^ | PLB+FLUO | 20mg/day | 0 | 0 | 0 | 0 | 0 |
| ^496^ | SYN | 1 cp/day | 0 | constipation (1 out of 20), dry mouth (1 out of 20), cramps (3 out of 20) | 0 | 0 | 0 |
| ^496^ | PLB |  | 0 | constipation (4 out of 20), somnolence (1 out of 20), dry mouth (2 out of 20), bloating (1 out of 20) | 0 | 0 | 0 |
| ^497^ | OMEGA3+TAU | 3g/day | 8 out of 40 | unpleasant taste (18 out of 40), aftertaste in breathe (11 out of 40) | 0 | 0 | 0 |
| ^497^ | PLB+TAU | NA | 15 out of 43 | 0 | 0 | 0 | 0 |
| ^602^ | US | 50ml | 0 | 0 | 0 | 0 | 0 |
| ^602^ | FLUO | 20mg 1/day | 0 | 0 | 0 | 0 | 0 |
| ^602^ | FLUO+US | 20mg+50ml/day | 0 | 0 | 0 | 0 | 0 |
| ^498^ | GGBT | 4000 mg/day | 0 | 0 | 0 | 0 | 0 |
| ^498^ | PLB |  | 0 | 0 | 0 | 0 | 0 |
| ^499^ | LH57 | 800mg/day | 8 out of 77 (6 adverse events) | appetite decrease (1 out of 70), pruritus (1 out of 70), headache (1 out of 70), diarrhea (1 out of 70), nausea (3 out of 70), vomiting (1 out of 70), dizziness (2 out of 70), asthenia (1 out of 70), abdominal pain (1 out of 70), fluid retention (1 out of 70), increased sweating (1 out of 70), dry mouth (1 out of 70), sleep impairment (3 out of 70), restlessness (1 out of 70) | tachycardia (1 out of 70) | 0 | 6 (1 recurrent biliary colic, 1 racing heartbeat at night, 1 nausea, diarrhea, swelling of face and pruritus, 1 excessive rise in blood pressure, increased frequency of difficulty in falling asleep or remaining asleep, pressure sensation on left side of chest, geenral malaise, 1 nausea, inne restlessness, vomitin g) |
| ^499^ | FLUO | 20mg/day | 16 out of 84 (3 inefficacy, 8 adverse events) | appetite decrease (1 out of 79), pruritus (1 out of 79), headache (1 out of 79), diarrhea (1 out of 79), nausea (3 out of 79), vomiting (1 out of 79), dizziness (2 out of 79), asthenia (1 out of 79), abdominal pain (1 out of 79), fluid retention (1 out of 79), increased sweating (1 out of 79), dry mouth (1 out of 79), sleep impairment (3 out of 79), restlessness (1 out of 79) | 0 | 3 | 8 (1 diarrhea, nausea, abdominal symptoms, restlessness, palpitations, 1 constipation, tension of muscles and skin of shoulders and neck, disturbance of concentration and thought, 1 racing heartbeat and rise in blood pressure, 1 sleep disturbance, tremor, restlessness, headache, loss of appetite, 1 vertigo persistent, relpasing, 1 nausea, restlness, diarrhea, headache, 1 upper adbominal symptoms, 1 dry m outh, constipation, eczema) |
| ^500^ | GBDP | 5 cp/3 day | 6 out of 34 (1 adverse events, 5 lack of compliance) | 0 | 0 | 0 | 1 |
| ^500^ | PLB |  | 2 out of 34 (2 lack of compliance) | 0 | 0 | 0 | 0 |
| ^501^ | EA | 200mg | 0 | 0 | 0 | 0 | 0 |
| ^501^ | PLB |  | 0 | 0 | 0 | 0 | 0 |
| ^502^ | SZL + PLB | 3.2g/day | 15 out of 73 | dizziness (1 out of 73), gastrointestinal discomfort (2 out of 73), dry mouth (2 out of 73), | hyperlipemia (1 out of 73) | 0 | 0 |
| ^502^ | FLUO + PLB | 20mg | 8 out of 71 | headache (1 out of 71), dizziness (1 out of 71), asthenia (1 out of 71), gastrointestinal discomfort (5 out of 71), dry mouth (2 out of 71), | hyperlipemia (1 out of 71), granulopenia (1 out of 71) | 0 | 0 |
| ^503^ | vitB12 | 1mg | 0 | 0 | 0 | 0 | 0 |
| ^503^ | PLB |  | 0 | 0 | 0 | 0 | 0 |
| ^592^ | fAA+MIR | NA | 0 | 0 | 0 | 0 | 0 |
| ^592^ | PLB+MIR | NA | 0 | 0 | 0 | 0 | 0 |
| ^587^ | OMEGA3+SER | 1000mg/day+50-200 mg/day | 0 | 0 | 0 | 0 | 0 |
| ^587^ | PLB+SER | 50-200 mg/day | 0 | 0 | 0 | 0 | 0 |
| ^504^ | EPA | 1g/day | 4 out of 20 (1 worsening of depression, 1 non compliance, 2 lost to follow-up( | diarrhea (1 out of 16), somnolence (1 out of 16), unpleasant taste (2 out of 16), anxiety (1 out of 16) | 0 | 1 | 0 |
| ^504^ | FLUO | 20mg7day | 4 out of 20 (1 adverse even, 1 non compliance, 2 lost to follow-up) | appetite decrease (6 out of 16), constipation (1 out of 16), nausea (3 out of 16), reflux (2 out of 16), somnolence (2 out of 16), insomnia (1 out of 16), tremor (1 out of 16), sexual dysfunction (2 out of 16), sleep impairment (1 out of 16), anxiety (9 out of 16) | 0 | 0 | 1 |
| ^504^ | EPA+FLUO | 1g/day+20mg/day | 4 out of 20 (2 adverse events, 1 non compliance, 1 lost to follow-up) | acne (2 out of 16), reflux (1 out of 16), insomnia (2 out of 16), abdominal pain (2 out of 16), unpleasant taste (2 out of 16), skin rash (1 out of 16), tremor (1 out of 16), sexual dysfunction (2 out of 16), dyspepsia (3 out of 16)anxiety (3 out of 16) | 0 | 0 | 2 |
| ^591^ | FLUO+SAFF | 20mg+30mg/day | 0 | 0 | 0 | 0 | 0 |
| ^591^ | FLUO + PLB | 20 mg/day | 0 | 0 | 0 | 0 | 0 |
| ^505^ | SAMe | 200mg/day | 0 | 0 | 0 | 0 | 0 |
| ^505^ | PLB |  | 0 | 0 | 0 | 0 | 0 |
| ^506^ | WS5572 | 900mg/day | 4 out of 37 protocol violation | sinusitis (1 out of 37), upper respiratory tract infection (2 out of 37) | 0 | 0 | 0 |
| ^506^ | PROB | NA | 4 out of 35 protocol violation | gastroenteritis (1 out of 35), upper respiratory tract infection (1 out of 35) | 0 | 0 | 0 |
| ^507^ | CUR | 1500mg/day | 3 out of 33 | ataxia (1 out of 33), headache (7 out of 33), constipation (4 out of 33), diarrhea (2 out of 33), nausea (5 out of 33), vomiting (5 out of 33), flatulence (5 out of 33), somnolence (3 out of 33), insomnia (10 out of 33), dizziness (6 out of 33), skin rash (1 out of 33), increased appetite (4 out of 33) | weight gain (23 out of 33), weight loss (6 out of 33) | NA | NA |
| ^507^ | PLB | NA | 1 out of 32 | ataxia (1 out of 32), headache (5 out of 32), constipation (6 out of 32), diarrhea (2 out of 32), nausea (4 out of 32), vomiting (4 out of 32), flatulence (5 out of 32), somnolence (6 out of 32), insomnia (15 out of 32), dizziness (5 out of 32), increased appetite (1 out of 32) | weight gain (15 out of 32), weight loss (6 out of 32) | NA | NA |
| ^508^ | SAFF | 30mg/day | 2 out of 30 (withdrawn consent) | headache (2 out of 28), constipation (3 out of 28), nausea (3 out of 28), somnsolence (2 out of 28), increased sweating (2 out of 28), dry mouth (3 out of 28) | 0 | 0 | 0 |
| ^508^ | PLB | NA | 2 out of 30 (withdrawn consent) | headache (1 out of 28), constipation (2 out of 28), nausea (1 out of 28), somnsolence (2 out of 28), increased sweating (1 out of 28), dry mouth (2 out of 28) | 0 | 0 | 0 |
| ^509^ | SAFF | 30mg/day | 2 out of 34 (shift to moderate-severe depression) | constipation (4 out of 32), somnolence (1 out of 32), dry mouth (2 out of 32) | 0 | 2 | 0 |
| ^509^ | FLUO | 40mg/day | 2 out of 34 (shift to moderate-severe depression) | headache (1 out of 32), somnolence (3 out of 32), dry mouth 81 out of 32) | 0 | 2 | 0 |
| ^510^ | WS5570 | 600 or 1200mg/day | 31 out of 250 (8 revoked consent, 2 lack of efficacy, 6 adverse event, 13 lost to follow-up, 1 did not meet selection criteria, 1 symptom aggravation) | headache (3 out of 250), nausea (1 out of 250), asthenia (2 out of 250), increased sweating (2 out of 250), dry mouth (13 out of 250) | 0 | 2 | 6 |
| ^510^ | PLB | NA | 8 out of 82 (2 revoked consent, 1 lack of efficacy, 5 lost to follow-up) | headache (6 out of 82), nausea (12 out of 82), dizziness (12 out of 82), asthenia (11 out of 82), increased sweating (13 out of 82), dry mouth (1 out of 82) | 0 | 1 | 0 |
| ^511^ | WS5570 | 1200mg/day | 0 | 0 | 0 | 0 | 0 |
| ^511^ | PLB |  | 0 | 0 | 0 | 0 | 0 |
| ^512^ | SIL | 1cp/day | 18 out of 159 ( 7 Withdrawal leading to exclusion, 5 Violation of visit schedule  3 Violation of inclusion/exclusion criteria, 1 Treatment non-compliance, 2 Non-compliant prior / co-medication) | eructation (16 out of 160), headache (4 out of 160), diarrhea (2 out of 160), nausea (3 out of 160), nasopharyngitis (3 out of 160) | 0 | 0 | 0 |
| ^512^ | PLB |  | 28 out of 156 (4 Withdrawal leading to exclusion, 10  -Violation of visit schedule, 6 Violation of inclusion/exclusion criteria, 5 Treatment non-compliance, 4 Medical history / concomitant diseases) | headache (9 out of 158), diarrhea (4 out of 158), nausea (1 out of 158), nasopharyngitis (8 out of 158) | 0 | 0 | 0 |
| ^597^ | SIL | 80mg/day | 16 out of 170 (8 adverse events, 1 consent revoked, 2 lack of efficacy, 1 lost to follow up, 4 other reasons) | diarrhea (23 out of 154), nausea (21 out of 154), fluid retention (21 out of 154), increased sweating (20 out of 154), sexual dysfunction (28 out of 154), pollakiuria (30 out of 154), failing memory (28 out of 154) | 0 | 2 | 8 |
| ^597^ | SER | 50mg/day | 20 out of 171 (10 adverse events, 3 consent revoked, 2 lack of efficacy, 3 lost to follow-up, 2 other reasons) | diarrhea (42 out of 151), nausea (41 out of 151), fluid retention (9 out of 151), increased sweating (32 out of 151), sexual dysfunction (35 out of 151), pollakiuria (23 out of 151), failing memory (13 out of 151) | 0 | 2 | 10 |
| ^597^ | PLB |  | 16 out of 157 (5 adverse events, 3 consent revoked, 2 inclusion criteria violated, 4 lost to follow-up, 2 other reasons) | diarrhea (22 out of 116), nausea (24 out of 141), fluid retention (9 out of 141), increased sweating (14 out of 141), sexual dysfunction (16 out of 141), pollakiuria (13 out of 141), failing memory (26 out of 141) | 0 | 0 | 5 |
| ^513^ | vitD | 50,000 IU/2wks | 0 | 0 | 0 | 0 | 0 |
| ^513^ | PLB |  | 0 | 0 | 0 | 0 | 0 |
| ^514^ | vitD3+FLUO | 1500IU/day+20mg/day | 1 out of 21 (severe anxiety) | 0 | 0 | 0 | 1 |
| ^514^ | FLUO | 20mg7day | 1 out of 21 (lost to follow-up) | 0 | 0 | 0 | 0 |
| ^515^ | NEP | 400mg/day | 3 out of 36 (1 personal causes, 2 intolerance) | 0 | 0 | 0 | 2 |
| ^515^ | SER | 50mg/day | 1 out of 21 (severe anxiety) | 0 | 0 | 0 | 1 |
| ^516^ | OMB | 3g | 0 | 0 | 0 | 0 | 0 |
| ^516^ | PLB |  | 0 | 0 | 0 | 0 | 0 |
| ^517^ | ESC + vitD | 10-20mg/day + 60000IU every 5 days | 7 out of 31 (4 withdre consent due to lack of perceived benefit, 2 withdre consent without starting reason, 1 lost to follow-up) | 0 | 0 | 4 out of 31 | 0 |
| ^517^ | ESC + PLB | 10-20 mg/day | 11 out of 28 (4 adverse effects, 4 lost to follow-up, 2 withdrew consent due to lack of perceived benefit, 1 withdrew consent without starting reason) | 0 | 0 | 2 out of 28 | 4 out of 28 |
| ^518^ | WS5573 | 3 x 300 mg/day | 0 | headache (19 out of 57), limb edema (3 out of 49), nausea (9 out of 49), somnolence (8 out of 49), insomnia (7 out of 49), muscle pain/aches (5 out of 49), gastrointestinal discomfort (9 out of 49), dry mouth (10 out of 49), joint pain (4 out of 49), upper respiratory tract infection (6 out of 49), urinary tract infection (5 out of 49) | 0 | 0 | 0 |
| ^518^ | WS5572 | 3 x 300 mg/day | 2 out of 49 (revocal of informed consent) | headache (12 out of 49), limb edema (4 out of 49), nausea (7 out of 49), somnolence (3 out of 49), insomnia (6 out of 49), muscle pain/aches (3 out of 49), gastrointestinal discomfort (5 out of 49), dry mouth (4 out of 49), skin rash (5 out of 49), joint pain (7 out of 49), upper respiratory tract infection (4 out of 49), urinary tract infection (6 out of 49) | 0 | 0 | 0 |
| ^518^ | PLB |  | 3 out of 49 (2 revocal of informed consent, 1 adverse events) | headache (12 out of 57), limb edema (7 out of 49), nausea (4 out of 49), somnolence (9 out of 49), insomnia (5 out of 49), muscle pain/aches (4 out of 49), gastrointestinal discomfort (10 out of 49), dry mouth (6 out of 49), joint pain (2 out of 49), upper respiratory tract infection (7 out of 49), urinary tract infection (5 out of 49) | 0 | 0 | 1 |
| ^211^ | WS5570 | 900mg/day | 18 out of 186 (10 lack of efficacy, 4 revocation of informed consent, 2 adverse events) | headache (3 out of 186), nausea (9 out of 186), insomnia (3 out of 186), dizziness (4 out of 186), abdominal pain (2 out of 186) | 0 | 10 | 2 out of 186 (hospitalization) |
| ^211^ | PLB | NA | 25 out of 189 (14 lack of efficacy, 7 revocation of informed consent, 2 adverse events) | headache (7 out of 189), nausea (6 out of 189), insomnia (2 out of 189), dizziness (4 out of 189), abdominal pain (4 out of 189) | 0 | 14 | 2 out of 189 (hospitalization) |
| ^519^ | INO | 12g/day | 7 out of 20 (2 mild psychotic symptoms, 1 weakness and tremor, 1 cutaneous burning sensations, 3 didn't want to continue) | nausea (1 out of 13), flatulence (1 out of 13) | 0 | 0 | 4 |
| ^519^ | PLB |  | 4 out of 19 (1 headache, 1 hypomania, 1 insomnia, 1 lack of desire to continue) | headache (1 out of 15), restlessness (1 out of 15), | 0 | 0 | 3 |
| ^604^ | INO | 12g/day | 0 | 0 | 0 | 0 | 0 |
| ^604^ | PLB |  | 0 | 0 | 0 | 0 | 0 |
| ^520^ | SHU | 6g/day | 0 | 0 | 0 | 0 | 0 |
| ^520^ | PLB |  | 0 | 0 | 0 | 0 | 0 |
| ^521^ | PS128+TAU | 300mg 2/day | 1 out of 17 | 0 | 0 | 0 | 0 |
| ^521^ | PLB+TAU |  | 1 out of 17 | 0 | 0 | 0 | 0 |
| ^522^ | BZT | 250-1500 mg/day | 6 out of 39 (1 poor adherence to protocol, 1 adverse effect, 3 unsatisfied, 1 physical problems) | constipation (2 out of 39), diarrhea (1 out of 39), nausea (1 out of 39), vomiting (1 out of 39), somnolence (4 out of 39), insomnia (5 out of 39), dizziness (2 out of 39), sleep impairment (2 out of 39), difficulties in concentration (2 out of 39), stiffness (1 out of 39), nervousness (2 out of 39), failing memory (1 out of 39) | heart pounding (1 out of 39) | 3 | 1 |
| ^522^ | SER | 25-150 mg/day | 15 out of 39 (2 poor adherence to protocol, 9 adverse effects, 3 unsatisfied, 1 physical problems) | pruritus (1 out of 39), constipation (1 out of 39), nausea (1 out of 39), vomiting (1 out of 39), somnolence ( out of 39), insomnia (5 out of 39), asthenia (5 out of 39), tremor (1 out of 39), akathisia (2 out of 39), sleep impairment (1 out of 39), mervousness (3 out of 39), failing memory (1 out of 39) | heart pounding (1 out of 39) | 3 | 9 |
| ^522^ | PLB |  | 10 out of 39 (2 poor adherence to protovol, 1 adverse effects, 7 unsatisfied) | parasthesias (1 out of 39), somnolence (2 out of 39), insomnia (9 out of 39), asthenia (1 out of 39), dry mouth (1 out of 39), tremor (1 out of 39), epileptic seizures (1 out of 39), sleep impairment (3 out of 39), difficulties in concentration (1 out of 39), stiffness (1 out of 39), nervousness (4 out of 39), failing memory (1 out of 39) | 0 | 7 | 1 |
| ^605^ | SUL+CLO | 600mg/day+75-100mg/day | 7 out of 43 (1 non medical cause, 1 inefficacy, 5 adverse events) | headache (3 out of 43), nausea (1 out of 43), asthenia (2 out of 43), increased sweating (2 out of 43), dry mouth (13 out of 43) | 0 | 1 | 5 |
| ^605^ | PLB+CLO | 75-100mg/day | 12 out of 47 (3 non medical causes, 4 inefficacy, 5 adverse events) | headache (6 out of 47), nausea (12 out of 47), dizziness (12 out of 47), asthenia (11 out of 47), increased sweating (13 out of 47), dry mouth (4 out of 47) | 0 | 4 | 5 |
| ^523^ | CUR | 500 or 1000mg/day | 5 out of 61 | headache (5 out 61), constipation (1 out of 61), diarrhea (9 out of 61), nausea (1 out of 61), abdominal pain (2 out of 61), increased sweating (1 out of 61), unpleasant taste (9 out of 61) | weight gain (2 out of 61) | 0 | 0 |
| ^523^ | CUR+SAFF | 500mg/day+30mg/day | 2 out of 26 | diarrhea (2 out of 26), nausea (1 out of 26), dizziness (2 out of 26), unpleasant taste (2 out of 26) | weight gain (2 out of 26) | 0 | 0 |
| ^523^ | PLB | NA | 5 out of 36 | headache (1 out of 36), constipation (1 out of 36), diarrhea (1 out of 36), nausea (1 out of 36), dizziness (3 out of 36), abdominal pain (2 out of 36), unpleasant taste (1 out of 36), | weight gain (2 out of 36) | 0 | 0 |
| ^221^ | CUR | 1000mg/day | 3 out of 28 | 0 | 0 | 0 | 1 (digestive complaints) |
| ^221^ | PLB | NA | 1 out of 28 | 0 | 0 | 0 | 0 |
| ^593^ | ESC+CREA | 20mg/day+5g/day | 8 out of 25 (2 withdrew consent, 4 lost to follow-up, 2 adverse event) | appetite decrease (1 out of 25), headache (6 out of 25), nausea (9 out of 25), dizziness (5 out of 25), increased sweating (2 out of 25), sleep impairment (6 out of 25), restlessness (2 out of 25), difficulties in concentration (1 out of 25), | 0 | 0 | 2 out of 25 |
| ^593^ | ESC+PLB | 20mg/day | 5 out of 27 (2 withdrew consent, 2 lost to follow-up, 1 adverse event) | appetite decrease (2 out of 27), headache (13 out of 27), nausea (6 out of 27), dizziness (3 out of 27), increased sweating (2 out of 27), sleep impairment (8 out of 27), restlessness (5 out of 27), difficulties in concentration (3 out of 27), | 0 | 0 | 1 out of 27 |
| ^524^ | CQ10+TAU | 200 mg | 10 out of 46 (6 lost to follow-up, 2 non-adherence, 2 adverse effects) | 0 | 0 | 0 | 2 |
| ^524^ | PLB+TAU |  | 13 out of 46 (7 lost to follow-up, 3 non.adherence, 3 adverse effects) | 0 | 0 | 0 | 3 |
| ^525^ | RHO | 340mg/day | 4 out of 20 (2 lost to follow-up, 2 inefficacy) | nausea (10 out of 20), somnolence (3 out of 20), insomnia (4 out of 20), asthenia (2 out of 20), dry mouth (2 out of 20) | 0 | 2 | 0 |
| ^525^ | SER | 50mg/day | 9 out of 19 (4 lost to follow-up; 2 inefficacy, 2 adverse event, 1 withdrew) | somnolence (3 out of 19), insomnia (4 out of 19), asthenia (2 out of 19), dry mouth (2 out of 19) | 0 | 2 | 2 |
| ^525^ | PLB |  | 4 out of 18 (2 lost follow-up, 2 lack of efficacy) | nausea (10 out of 18), somnolence (3 out of 18), insomnia (4 out of 18), asthenia (2 out of 18) | 0 | 2 | 0 |
| ^526^ | DHA | 2g/day | 0 | eructation (3 out of 18), headache (2 out of 18), insomnia (1 out of 18), dizziness (3 out of 18), loose/soft stools (2 out of 18), aftertaste in breathe (13 out of 18) | 0 | 0 | 0 |
| ^526^ | PLB | NA | 0 | insomnia (1 out of 17), asthenia (3 out of 17), loose/soft stools (1 out of 17) | 0 | 0 | 0 |
| ^580^ | CTP + PLB | 20mg 1/day | 0 | 0 | 0 | 0 | 0 |
| ^580^ | CTP+OMEGA3 | 20mg+1g | 0 | 0 | 0 | 0 | 0 |
| ^527^ | SAF | 50 mg 2/day | 6 out of 30 ( 1 due to symptoms of saffron-induced  allergy, 1 due to an operative procedure for an unrelated  reason, and 4 others due to non-cooperation) | hayfever (1 out of 30) | 0 | 0 | 1 out of 30 ( due to symptoms of saffron-induced  allergy) |
| ^527^ | PLB |  | 0 | 0 | 0 | 0 | 0 |
| ^528^ | vitB | 1cp/day | 0 | 0 | 0 | 0 | 0 |
| ^528^ | PLB | NA | 0 | 0 | 0 | 0 | 0 |
| ^254^ | EPA | 1g/day | 5 out of 16 (1 non response, 1 commuting difficulties, 3 lost follow up) | gastrointestinal discomfort (2 out of 16) | 0 | 1 | 0 |
| ^254^ | PLB | NA | 6 out of 19 (2 non response, 1 feeling better, 3 lost follow-up) | gastrointestinal discomfort (5 out of 16) | 0 | 2 | 0 |
| ^603^ | EPA | 4g/day | 2 out of 13 (1 Stopped study meds  because of need for anti-  inflammatory meds, 1 Last visit(s) dropped from  analysis due to poor  compliance) | eructation (1 out of 16), vomiting (1 out of 16), reflux (1 out of 16), gastrointestinal discomfort (1 out of 16), skin rash (1 out of 16), tinnitus (1 out of 16) | 0 | 0 | 0 |
| ^603^ | PLB |  | 1 out of 11 (Last visit(s) dropped from  analysis due to poor  compliance during time  of improved life situation) | eye/visual disorders (1 out of 15), muscoskeletal disorders (2 out of 15), cough (1 out of 15), bloating (1 out of 15), reflux (2 out of 15), somnolence (1 out of 15), dizziness (2 out of 15) | 0 | 0 | 0 |
| ^529^ | SAMe | 1600-3200mg/day | 28 out of 64 (3 clinical worsening, 5 ineffective, 3 adverse events) | sexual dysfunction (2 out of 59), failing memory (1 out of 59) | 0 | 0 | 3 (2 GI upset; 1 leg pain) |
| ^529^ | ESC | 10-20mg/day | 35 out of 65 (4 clinical worsening, 0 ineffective, 8 adverse events) | dizziness (2 out of 55), sexual dysfunction (10 out of 55), hot flushes (4 out of 55) | 0 | 0 | 8 (2 GI upset; 2 agitation; 1 sexual dysfuntion; 1 insomnia; 1 sedation; 1 unspecified) |
| ^529^ | PLB |  | 29 out of 60 (2 clinical worsening, 4 ineffective, 4 adverse events) | dizziness (4 out of 52), sexual dysfunction (4 out of 52), failing memory (6 out of 52) | 0 | 0 | 4 (2 GI upset; 1 agitation; 1 unspecified) |
| ^530^ | SAFF | 30mg/day | 1 out of 20 | appetite decrease (4 out of 20), headache (3 out of 20), nausea (5 out of 20), abdominal pain (4 out of 20), increased sweating (2 out of 20), tremor (3 out of 20), anxiety (4 out of 20) | heart pounding (4 out of 20) | NA | NA |
| ^530^ | PROB | NA | 3 out of 20 | appetite decrease (2 out of 20), headache (1 out of 20), nausea (2 out of 20), abdominal pain (2 out of 20), increased sweating (1 out of 20), tremor (1 out of 20), anxiety (2 out of 20) | heart pounding (2 out of 20) | NA | NA |
| ^272^ | EPA | 1g/day | 6 out of 27 | appetite decrease (1 out of 21), headache (1 out of 21), somnolence (2 out of 21), insomnia (1 out of 21), dizziness (1 out of 21), dry mouth (1 out of 21), sexual disfunction (1 out of 21), anxiety (1 out of 21), increased appetite (2 out of 21), | 0 | 0 | 2 |
| ^272^ | DHA | 1g/day | 7 out of 27 | headache (2 out of 20), somnolence (2 out of 20), dizziness (1 out of 20), dry mouth (2 out of 20), anxiety (1 out of 20), increased appetite (3 out of 20) | 0 | 0 | 3 |
| ^272^ | PLB | NA | 6 out of 27 | appetite decrease (3 out of 21), headache (4 out of 21), diarrhea (1 out of 21), insomnia (3 out of 21), dizziness (5 out of 21), dry mouth (1 out of 21), sexual dysfunction (3 out of 21), anxiety (5 out of 21), increased appetite (1 out of 21) | 0 | 0 | 4 |
| ^531^ | TAU+CREA | 5g | 0 | 0 | 0 | 0 | 0 |
| ^531^ | TAU+PLB |  | 0 | 0 | 0 | 0 | 0 |
| ^531^ | TAU+CREA | 10g | 0 | 0 | 0 | 0 | 0 |
| ^531^ | TAU+PLB |  | 0 | 0 | 0 | 0 | 0 |
| ^296^ | EPA+TAU | 2g/day | 0 out of 10 | 0 | 0 | 0 | 0 |
| ^296^ | PLB+TAU | NA | 1 out of 10 (worsening of depressive symptoms) | 0 | 0 | 0 | 1 |
| ^532^ | ZPG | 6.75 g x 2/day | 15 out of 100 (1 did not take the study medicine, 3 protocol violation, 8 loss to follow-up, 3 adverse events) | dizziness (1 out of 99), gastrointestinal discomfort (2 out of 99), dry mouth (2 out of 99) | 0 | 0 | 3 |
| ^532^ | PLB |  | 14 out of 100 ( 5 protocol violation, 7 loss to follow-up, 2 adverse events) | headache (1 out of 100), dizziness (1 out of 100), asthenia (1 out of 100), gastrointestinal discomfort (5 out of 100), dry mouth (2 out of 100) | 0 | 0 | 2 |
| ^533^ | SAFF | 30mg/day | 1 out of 20 (consent withdraw) | appetite decrease (2 out of 20), headache (3 out of 20), diarrhea (4 out of 20), nausea (3 out of 20), somnolence (2 out of 20), insomnia (1 out of 20), dizziness (3 out of 20), abdominal pain (4 out of 20), increased appetite (3 out of 20) | 0 | 0 | 0 |
| ^533^ | FLUO | 20mg/day | 1 out of 20 (consent withdraw) | appetite decrease (1 out of 20), headache (1 out of 20), diarrhea (2 out of 20), nausea (2 out of 20), somnolence (3 out of 20), insomnia (2 out of 20), dizziness (3 out of 20), abdominal pain (2 out of 20), increased appetite (3 out of 20) | 0 | 0 | 0 |
| ^534^ | ZNC+TAU | 25mg/day | 0 | 0 | 0 | 0 | 0 |
| ^534^ | PLB+TAU |  | 0 | 0 | 0 | 0 | 0 |
| ^535^ | TAU+SAR | 500mg/day | 5 out of 30 | somnolence (2 out of 30), dizziness (1 out of 30), asthenia (1 out of 30) | 0 | 0 | 0 |
| ^535^ | TAU+PLB |  | 4 out of 30 | somnolence (1 out of 30), dizziness (2 out of 30), scleritis (1 out of 30), asthenia (1 out of 30) | 0 | 0 | 0 |
| ^536^ | CVE+TAU | 1800mg/day | 18 out of 60 | 0 | 0 | NA | 0 |
| ^536^ | TAU | NA | 15 out of 65 | 0 | 0 | NA | 0 |
| ^537^ | SAMe | 400mg/die (i.m.) | 13 out of 147 (1 patient no post-baseline: excluded from analysis; 2 Adverse event;) | 0 | 0 | NA | 2 out of 147 |
| ^537^ | IMI | 75mg/day | 19 out of 148 (1 patient didn't take medication: exlcuded from analysis; 7 adverse event) | 0 | 0 | NA | 7 out of 147 |
| ^538^ | SAMe+TAU | 800mg/day | 8 out of 39 (2 inefficacy, 2 intolerance, 3 other reasons, 1 lost to follow-up) | pruritus (1 out of 39), headache (4 out of 39), diarrhea (7 out of 39), somnolence (4 out of 39), insomnia (2 out of 39), epigastric issues (14 out of 39), agitation (3 out of 39) | 0 | 2 | 2 |
| ^538^ | PLB+TAU |  | 10 out of 34 (4 inefficacy, 3 intolerance, 2 other reasons, 1 lost to follow-up) | pruritus (1 out of 34), headache (2 out of 34), diarrhea (5 out of 34), somnolence (2 out of 34), insomnia (2 out of 34), epigastric issues (7 out of 34), sleep impairment (2 out of 34) | 0 | 4 | 3 |
| ^539^ | LMF | 7.5mg/day | 3 out of 36 | 0 | 0 | NA | NA |
| ^539^ | PLB | NA | 14 out of 112 | 0 | 0 | NA | NA |
| ^539^ | LMF | 15mg/day | 2 out of 19 | 0 | 0 | NA | NA |
| ^539^ | PLB | NA | 6 out of 56 | 0 | 0 | NA | NA |
| ^540^ | OMEGA3+TAU | 1.14mgEPA+600mgDHA/day | 6 out of 18 | 0 | 0 | 0 | 0 |
| ^540^ | PLB+TAU | NA | 5 out of 17 | 0 | 0 | 0 | 0 |
| ^541^ | DHA+EPA | 130mg+35mg | 0 | 0 | 0 | 0 | 0 |
| ^541^ | PLB |  | 0 | 0 | 0 | 0 | 0 |
| ^588^ | CAR+SER | 1000mg + 100mg | 1 out of 30 (discontinued intervention) | 0 | 0 | 0 | 0 |
| ^588^ | PLB+SER | 100mg | 2 out of 30 (discontinued intervention) | 0 | 0 | 0 | 0 |
| ^542^ | ZNC+TAU | 25mg/day | 1 out of 22 (failure to complete the questionaire) | 0 | 0 | 0 | 0 |
| ^542^ | TAU | NA | 5 out of 22 (2 travel, 1 broken leg, 1 intolerance, 1 illness unrelated to treatment) | 0 | 0 | 0 | 1 (intolerance) |
| ^332^ | EPA+DHA | 6g/day | 1 out of 13 (possibly hypomanic) | 0 | 0 | 0 | 1 |
| ^332^ | PLB |  | 4 out of 13 (1 nausea, 1 suicidal, 1 non-compliant, 1 other treatment) | nausea (1 out of 13), dry mouth (4 out of 13) | 0 | 0 | 1 |
| ^543^ | EPA+DHA | 1,67g/day+0,83g/day | 0 | eructation (1 out of 22), constipation (2 out of 22), bloating (3 out of 22) | 0 | 0 | 0 |
| ^543^ | PLB |  | 0 | eructation (3 out of 24), headache (1 out of 24), constipation (2 out of 24) | 0 | 0 | 0 |
| ^586^ | CIT+CTP | 200mg/day+20-40mg/day | 2 out of 27 (due to consent withdrawn) | appetite decrease (2 out of 25), headache (3 out of 25), diarrhea (4 out of 25), nausea (3 out of 25), somnolence (2 out of 25), insomnia (1 out of 25), dizziness (3 out of 25), abdominal pain (4 out of 25), increased appetite (3 out of 25) | 0 | 0 | 0 |
| ^586^ | PLB+CTP | 20-40mg/day | 2 out of 27 (due to consent withdrawn) | appetite decrease (1 out of 25), headache (1 out of 25), diarrhea (2 out of 25), nausea (2 out of 25), somnolence (3 out of 25), insomnia (2 out of 25), dizziness (3 out of 25), abdominal pain (2 out of 25), increased appetite (3 out of 25) | 0 | 0 | 0 |
| ^544^ | LP299v+TAU | 2 capsules/day; each capsule contains 10×109CFU of Lactobacillus Plantarum 299v | 10 out of 40 (4 lost follow-up, 3 required psychiatric hospitalisation, 1 difficulties with swallowing capsules, 1 antipsychotic treatment, 1 required antibiotic) | headache (2 out of 30), dizziness (1 out of 30), loose/soft stools (1 out of 30), tremor (1 out of 30), | 0 | 0 | 0 |
| ^544^ | PLB+TAU | NA | 9 out of 39 (5 lost follow-up, 2 required hospitalisation, 1 diagnosi of malignant disease, 1 required antibiotic) | headache (4 out of 30), flatulence (1 out of 30), loose/soft stools (1 out of 30) | heart pounding (1 out of 30) | 0 | 0 |
| ^583^ | MG+FLUO | 120mg/day+20-40mg/day | 0 out of 17 | 0 | 0 | NA | NA |
| ^583^ | PLB+FLUO | 20-40mg/day | 5 out of 20 | 0 | 0 | NA | NA |
| ^545^ | SAMe+HEAL9 | 200mg + 1 cp/day | 0 | appetite decrease (2 out of 46) | 0 | 0 | 0 |
| ^545^ | PLB |  | 0 | pruritus (1 out of 44), skin rash (1 out of 44) | 0 | 0 | 0 |
| ^546^ | EACS+SJW | 100mg+250mg/2a day | 14 out of 37 (lost to follow-up) | nausea (1 out of 23), dry mouth (1 out of 23) | 0 | 0 | 0 |
| ^546^ | FLUO | 20 mg/2 a day | 9 out of 37 (lost to follow-up) | dry mouth (4 out of 28) | 0 | 0 | 0 |
| ^547^ | SER+EPA+DHA | 50mg+1mg/day | 3 out of 33 (lost at follow up) | 0 | 0 | 0 | 0 |
| ^547^ | SER+5HTP | 50mg+100mg/day | 7 out of 37 (lost at follow up) | 0 | 0 | 0 | 0 |
| ^547^ | SER | 50mg/day | 3 out of 33 (lost at follow up) | 0 | 0 | 0 | 0 |
| ^585^ | vitC+CTP | 1g/day+up to 60mg/day | 2 out of 21 (1 lost to follow-up, 1 did not respond to the intervention) | appetite decrease (4 out of 21), nausea (12 out of 21), somnolence (2 out of 21), insomnia (3 out of 21), drooling (1 out of 21), abdominal pain (3 out of 21), dry mouth (7 out of 21), weakness (9 out of 21), increased appetite (3 out of 21), restlessness (3 out of 21), nervousness (1 out of 21) | 0 | 1 | 0 |
| ^585^ | PLB+CTP | up to 60mg/day | 4 out of 22 (2 lost to follow-up, 2 did not respond to the intervention) | appetite decrease (6 out of 22), pruritus (2 out of 22), constipation (1 out of 22), diarrhea (1 out of 22), nausea (11 out of 22), insomnia (1 out of 22), abdominal pain (2 out of 22), increased sweating (4 out of 22), dry mouth (9 out of 22), blurred vision (3 out of 22), weakness (8 out of 22), tremor (2 out of 22), restlessness (2 out of 22), nervousness (1 out of 22) | tachycardia (1 out of 22) | 2 | 0 |
| ^585^ | FLUO+SAFF | 1 cp + 20mg | 1 out of 20 (adverse events) | headache (1 out of 20), nausea (1 out of 20) | 0 | 0 | 1 (headache and nausea) |
| ^585^ | FLUO + PLB | 20mg | 9 out of 20 (adverse events) | headache ( 9 out of 20), nausea (9 out of 20), gastrointestinal discomfort (9 out of 20) | 0 | 0 | 9 (headache, abdominal discomfort, nausea) |
| ^548^ | SAMe | 3,200 mg/day | 0 | abdominal pain (5 out of 16), fluid retention (4 out of 16) | 0 | 0 | 0 |
| ^548^ | ESC | 20 mg/day | 0 | abdominal pain (2 out of 23) | 0 | 0 | 0 |
| ^548^ | PLB |  | 0 | abdominal pain (1 out of 26) | 0 | 0 | 0 |
| ^549^ | CUR | 1000mg/day | 6 out of 20 | 0 | 0 | 0 | 0 |
| ^549^ | FLUO | 20mg/day | 4 out of 20 | 0 | 0 | 0 | 1 |
| ^549^ | CUR+FLUO | 1000mg+20mg/day | 5 out of 20 | 0 | 0 | 0 | 1 |
| ^550^ | SAMe+TAU | 800mg/day | 15 out of 55 | cramps (7 out of 55), headache (2 out of 55), diarrhea (13 out of 55), dizziness (4 out of 55), incrfeased sweating (7 out of 55), confusion (8 out of 55), sleep impairment (3 out of 55), hot flushes (11 out of 55), restlessness (10 out of 55) | 0 | 0 | 5 (1 serotonin syndrome, 1 kidney stones) |
| ^550^ | PLB+TAU | NA | 15 out of 52 | cramps (9 out of 52), headache (11 out of 52), diarrhea (19 out of 52), dizziness (7 out of 52), incrfeased sweating (14 out of 52), confusion (5 out of 52), sleep impairment (4 out of 52), hot flushes (6 out of 52), restlessness (8 out of 52) | 0 | 0 | 2 (1 hypomanic episode, 1 ovarian cystis) |
| ^551^ | NUT | 800mg+500mcg+200mcg+100mgEPA+656mgDHA+200mg+30mg+100mg+60mg+40mg/day | 25 out of 81 | cramps (6 out of 81), headache (11 out of 81), diarrhea (23 out of 81), nausea (15 out of 81), asthenia (12 out of 81), increased sweating (16 out of 81), tremor (8 out of 81) | 0 | 0 | 9 |
| ^551^ | PLB | NA | 20 out of 78 | cramps (111 out of 77), headache (14 out of 77), diarrhea (30 out of 77), nausea (5 out of 77), asthenia (11 out of 77), increased sweating (19 out of 77), tremor (12 out of 77) | 0 | 0 | 3 |
| ^606^ | LI-160 | 900-1500mg/day | 0 | 0 | 0 | 0 | 0 |
| ^606^ | SER | 50-100mg/day | 0 | 0 | 0 | 0 | 0 |
| ^606^ | PLB |  | 0 | 0 | 0 | 0 | 0 |
| ^552^ | SAMe | 800mg/day | 3 out of 25 (2 participant decision, 1 worsening of depression) | appetite decrease (1 out of 25), headache (6 out of 25), nausea (9 out of 25), dizziness (5 out of 25), increased sweating (2 out of 25), sleep impairment (6 out of 25), restlessness (2 out of 25), difficulties in concentration (1 out of 25), | 0 | 1 | 0 |
| ^552^ | PLB | NA | 5 out of 24 (1 adverse event, 4 worsening of depression) | appetite decrease (2 out of 24), headache (13 out of 24), nausea (6 out of 24), dizziness (3 out of 24), increased sweating (2 out of 24), sleep impairment (8 out of 24), restlessness (5 out of 24), difficulties in concentration (3 out of 24), | 0 | 4 | 1 |
| ^553^ | ZE117 | 500mg/day | 1 out of 126 | headache (3 out of 125), nausea (9 out of 125), insomnia (3 out of 125), dizziness (4 out of 125), abdominal pain (2 out of 125) | 0 | 0 | 0 |
| ^553^ | FLUO + PLB | 20mg/day | 1 out of 114 | headache (7 out of 114), nausea (6 out of 114), insomnia (2 out of 114), dizziness (4 out of 114), abdominal pain (4 out of 114) | 0 | 0 | 1 |
| ^554^ | ZE117 | 500mg/day | 1 out of 81 (deterioration of the condition) | dizziness (1 out of 81), gastrointestinal discomfort (2 out of 81), dry mouth (2 out of 81), | 0 | 1 | 0 |
| ^554^ | PROB | NA | 2 out of 81 (1 lost to follow-up, 1 syncope and hospitalization) | headache (1 out of 81), dizziness (1 out of 81), asthenia (1 out of 81), gastrointestinal discomfort (5 out of 81), dry mouth (2 out of 81) | 0 | 0 | 1 |
| ^555^ | SRN+TAU | 2 g/day | 4 out of 26 (2 personal reasons, 2 lost contact) | 0 | 0 | 0 | 0 |
| ^555^ | PLB+TAU |  | 4 out of 26 (1 personal reasons, 1 withdrew surgery, 1 lost contact) | 0 | 0 | 0 | 0 |
| ^581^ | CTP+FOL | 20mg+2,5mg | 0 | 0 | 0 | 0 | 0 |
| ^581^ | CTP + PLB | 20mg | 0 | 0 | 0 | 0 | 0 |
| ^556^ | vitD | 50000IU/week | 2 out of 20 (lost to folow-up) | 0 | 0 | 0 | 0 |
| ^556^ | PLB | NA | 2 out of 20 (lost to folow-up) | 0 | 0 | 0 | 0 |
| ^557^ | SER+LAV+DRA | 100mg+5ml/day | 0 | 0 | 0 | 0 | 0 |
| ^557^ | SER+PLB | 1000mg/day | 0 | 0 | 0 | 0 | 0 |
| ^558^ | SAFF | 30 mg/day | 2 out of 22 (1 withdrawn consent at week 1, 1 at week 3) | appetite decrease (4 out of 20), constipation (1 out of 20), somnolence (1 out of 20), dry mouth (1 out of 20) | 0 | 0 | 0 |
| ^558^ | FLUO | 40 mg/day | 2 out of 22 (1 severe anxiety, 1 suicidal ideation) | appetite decrease (2 out of 20), constipation (3 out of 20), somnolence (2 out of 20), dry mouth (4 out of 20), restlessness (1 out of 20) | 0 | 0 | 2 out of 22 |
| ^559^ | APH | 2 cp/day | 6 out of 27 | 0 | 0 | 0 | 0 |
| ^559^ | PLB |  | 8 out of 27 | 0 | 0 | 0 | 0 |
| ^560^ | SER+THE | 100mg/day+200mg/day | 5 out of 30 (3 COVID-19, 2 withdrawn) | appetite decrease (5 out of 25), headache (4 out of 25), diarrhea (4 out of 25), vomiting (4 out of 25), abdominal pain (5 out of 25) | 0 | 0 | 3 out of 30 (COVID-19) |
| ^560^ | SER+PLB | 100mg/day | 5 out of 30 (2 COVID-19, 3 withdrawn) | appetite decrease (3 out of 25), headache (3 out of 25), diarrhea (2 out of 25), vomiting (2 out of 25), abdominal pain (3 out of 25) | 0 | 0 | 2 out of 30 (COVID-19) |
| ^561^ | EOPF | 434g/week | 0 | 0 | 0 | 0 | 0 |
| ^561^ | PLB |  | 0 | 0 | 0 | 0 | 0 |
| ^562^ | SJW | 900mg/day, increased to 1200mg/day | 18 out of 98 (5 did not take any treatment; 2 personal choice, 8 lost to follow up, 1 adverse event, 4 no reason) | headache (39 out of 98), gastrointestinal discomfort (10 out of 98) | 0 | 0 | 1 |
| ^562^ | PLB | NA | 15 out of 102 (1 personal choide, 6 lost to follow up, 1 non compliance, 1 adverse event, 4 no reason) | headache (25 out of 102), gastrointestinal discomfort (11 out of 102) | 0 | 0 | 1 |
| ^563^ | CIT | 2000 mg/day | 19 out of 28 (14 lost to follow-up, 1 moved out of the  area , 1 entered inpatient drug rehabilitation , 1 trans-  portation problems, 1 stressors at home and 1 advice of  probation officer) | appetite decrease (2 out of 28), constipation (2 out of 28), diarrhea (2 out of 28), insomnia (2 out of 28), | 0 | 17 out of 20 (12 lost to follow-up, 2 moved out of the  area , 1 entered inpatient drug rehabilitation , 1 trans-  portation problems,1 withdrawn due to adverse event) | 1 out of 20 |
| ^563^ | PLB |  | 0 | appetite decrease (1 out of 20), headache (2 out of 20), diarrhea (1 out of 20), somnolence (1 out of 20), tinnitus (1 out of 20), increased appetite (2 out of 20), difficulties in concentration (2 out of 20), | weight gain (1 out of 20) | 0 | 0 |
| ^564^ | EPA+DHA | 1.95g+1.35g/day | 6 out of 21 | constipation (1 out of 21), dry mouth (1 out of 21) | 0 | 0 | 1 (bronchitis) |
| ^564^ | PLB | NA | 2 out of 18 | constipation (4 out of 18), somnolence (1 out of 18), dry mouth (2 out of 18) | 0 | 0 | 1 (colitis) |
| ^598^ | ZNC+IMI | 25mg/day+140mg/day | 3 out of 30 (1 side effects, 2 compliance failure) | headache (3 out of 28), nausea (9 out of 28), insomnia (3 out of 28), dizziness (4 out of 28), abdominal pain (2 out of 28) | 0 | 0 | 1 |
| ^598^ | IMI+PLB | 140mg/day | 5 out of 30 (2 side effects, 3 compliance failure) | headache (7 out of 27), nausea (6 out of 27), insomnia (2 out of 27), dizziness (4 out of 27), abdominal pain (4 out of 27) | 0 | 0 | 2 |
| ^565^ | EPA+DHA | 9.6g/day | 2 out of 14 | 0 | 0 | 0 | 0 |
| ^565^ | PLB | NA | 4 out of 14 | 0 | 0 | 0 | 0 |
| ^387^ | EPA+DHA | 3.4g/day | 5 out of 18 | diarrhea (2 out of 18), constipation (2 out of 18) | 0 | 3 | 0 |
| ^387^ | PLB | NA | 7 out of 18 | constipation (3 out of 18), diarrhea (2 out of 18) | 0 | 3 | 1 |
| ^566^ | MG | 200mg | 5 out of 45 (2 lost to follow-up, 1 treatment change, 2 discontinued treatment) | appetite decrease (2 out of 40), headache (4 out of 40), constipation (2 out of 40), nausea (2 out of 40), dizziness (3 out of 40), sexual dysfunction (1 out of 40), restlessness (1 out of 40) | 0 | 0 | 0 |
| ^566^ | PLB |  | 7 out of 45 (3 treatment change, 3 lost to follow-up, 1 discotninued treatment) | appetite decrease (1 out of 38), headache (5 out of 38), constipation (1 out of 38), nausea (2 out of 38), dizziness (7 out of 38), sexual dysfunction (1 out of 38), restlessness (5 out of 38) | 0 | 0 | 0 |
| ^567^ | WS5570 | 900mg/day | 17 out of 125 (6 lack of efficacy, 4 adverse event, 6 lost to follow-up, 1 remission) | headache (13 out of 125), diarrhea (12 out of 125), nausea (9 out of 125), dizziness (9 out of 125), asthenia (14 out of 125), abdominal pain (12 out of 125), increased sweating (9 out of 125), dry mouth (16 out of 125), sleep impairment (5 out of 125) | 0 | 6 | 4 |
| ^567^ | PAR | 20mg/day | 29 out of 126 (5 withdrew consent, 1 remission, 3 lack of efficacy, 8 adverse event, 1 violation of exclusion criteria, 8 lost to follow-up, 3 other) | headache (14 out of 126), diarrhea (23 out of 126), nausea (21 out of 126), dizziness (24 out of 126), asthenia (16 out of 126), abdominal pain (9 out of 126), increased sweating (13 out of 126), dry mouth (35 out of 126), sleep impairment (10 out of 126) | 0 | 3 | 8 |
| ^590^ | SAFF+TAU | 30mg/day | 3 out of 23 | agitation (1 out of 23), dyspnea (1 out of 23), menometorrhagia (1 out of 23) | tachycardia (1 out of 23) | 0 | 0 |
| ^590^ | PLB+TAU |  | 3 out of 23 | pollakiuria (1 out of 23 ), dyspnea (1 out of 23) | 0 | 0 | 0 |
| ^568^ | SER+CUR | 40mg/day | 3 out of 23 (lost to follow-up) | 0 | 0 | 0 | 0 |
| ^568^ | SER+PLB |  | 2 out of 22 (lost to followw-up) | 0 | 0 | 0 | 0 |
| ^569^ | SAMe+TAU | 800mg/day | 5 out of 118 | headache (4 out of 118), constipation (5 out of 118), diarrhea (13 out of 118), nausea (9 out of 118), vomiting (4 out of 118), abdominal pain (4 out of 118), gastrointestinal discomfort (4 out of 118), epigastric issues (3 out of 118), dyspepsia (4 out of 118), gastroenteritis (3 out of 118), nasopharyngitis (2 out of 118), sinusitis (3 out of 118), upper respiratory tract infection (3 out of 118), back pain (3 out of 118) | 0 | 0 | 1 |
| ^569^ | PLB+TAU | NA | 11 out of 116 | nausea (1 out of 116), diarrhea (5 out of 116), nausea (2 out of 116), vomiting (1 out of 116), gastrointestinal discomfort (1 out of 116), joint pain (3 out of 116), nasopharyngitis (3 out of 116), upper respiratory tract infection (6 out of 116), back pain (2 out of 116), | 0 | 0 | 1 |
| ^570^ | LS | 7g | 0 | 0 | 0 | 0 | 0 |
| ^570^ | PLB |  | 0 | 0 | 0 | 0 | 0 |
| ^571^ | INU | 10g/day | 9 out of 31 (4 did not return to clinic, 5 could not follow dietary plan) | gastrointestinal discomfort (5 out of 31) | 0 | 0 | 0 |
| ^571^ | PLB |  | 8 out of 31 ( 3 did not return to clinic, 1 pregnancy, 4 could not follow dietary plan) | 0 | 0 | 0 | 0 |
| ^572^ | SJW | 900-1800mg/day | 15 out of 45 (3 side effects, 7 unknown, 1 manic episode, 2 increase in depression, 1 suicidal, 1 cancer recurrence) | appetite decrease (10 out of 45), cramps (5 out of 45), headache (18 out of 45), diarrhea (10 out of 45), nausea (4 out of 45), dizziness (5 out of 45), muscle pain/aches (5 out of 45), increased sweating (7 out of 45), dry mouth (16 out of 45), blurred vision (6 out of 45), weakness (19 out of 45), tremor (8 out of 45), epigastric issues (5 out of 45), sleep impairment (23 out of 45), anxiety (18 out of 45), stiffness (8 out of 45), urinary retention (7 out of 45) | heart pounding (4 out of 45) | 2 (increase in depression) | 4 (1 manic episode) |
| ^572^ | SER | 50-100mg/day | 15 out of 43 (7 side effects, 7 unknown, 1 increase in depression) | appetite decrease (11 out of 43), cramps (5 out of 43), headache (14 out of 43), diarrhea (17 out of 43), nausea (17 out of 43), dizziness (11 out of 43), muscle pain/aches (8 out of 43), increased sweating (13 out of 43), dry mouth (20 out of 43), blurred vision (7 out of 43), weakness (21 out of 43), tremor (5 out of 43), epigastric issues (15 out of 43), sleep impairment (24 out of 43), anxiety (18 out of 43), stiffness (5 out of 43), urinary retention (7 out of 43) | heart pounding (7 out of 43) | 1 (increase in depression) | 7 |
| ^596^ | PRO | 1,000mg/day | 0 | constipation (2 out of 28), diarrhea (1 out of 28), nausea (1 out of 28), vomiting (1 out of 28), somnolence (4 out of 28), insomnia (5 out of 28), dizziness (2 out of 28), sleep impairment (2 out of 28), difficulties in concentration (2 out of 28), stiffness (1 out of 28), nervousness (2 out of 28), failing memory (1 out of 28) | 0 | 0 | 0 |
| ^596^ | PLB |  | 0 | pruritus (1 out of 26), constipation (1 out of 26), nausea (1 out of 26), vomiting (1 out of 26), somnolence ( 2out of 26), insomnia (5 out of 26), asthenia (5 out of 26), tremor (1 out of 26), akathisia (2 out of 26), sleep impairment (1 out of 26), nervousness (3 out of 26), failing memory (1 out of 26) | 0 | 0 | 0 |
| ^589^ | vitD3+TAU | 300000IU | 1 out of 23 (lost to follow-up) | 0 | 0 | 0 | 0 |
| ^589^ | PROB+TAU |  | 3 out of 23 (2 lost to follow-up, 1 personal reasons) | 0 | 0 | 0 | 0 |
| ^573^ | ZNC+TAU | 20 mg/day | 0 | 0 | 0 | 0 | 0 |
| ^573^ | TAU |  | 0 | 0 | 0 | 0 | 0 |
| ^574^ | LI-160 | 1800mg/day | 9 out of 107 | somnolence (5 out of 107), dizziness (5 out of 107), gastrointestinal discomfort (5 out of 107), dry mouth (3 out of 107), skin rash (1 out of 107), tremor (2 out of 107), restlessness (6 out of 107) | 0 | NA | 1 out of 107 |
| ^574^ | IMI | 150mg/day | 14 out of 102 | headache (3 out of 102), constipation (5 out of 102), somnolence (8 out of 102), dizziness (2 out of 102), increased sweating (8 out of 102), gastrointestinal discomfort (9 out of 102), dry mouth (16 out of 102), skin rash (2 out of 102), tremor (4 out of 102), sleep impairment (2 out of 102), restlessness (6 out of 102) | heart pounding (3 out of 102) | NA | 8 out of 102 |
| ^575^ | vitD3 | 50000IU/week | 5 out of 362 | 0 | 0 | 0 | 5 (2 bone and joint pain, 1 diarrhea, 1 vomiting, 1 vomiting and nausea) |
| ^575^ | PLB | NA | 0 out of 364 | 0 | 0 | 0 | 0 |
| ^429^ | LI-160 | 2400mg/day | 20 out of 87 | pruritus (2 out of 87), headache (6 out of 87), constipation (4 out of 87), nausea (6 out of 87), somnolence (1 out of 87), dizziness (1 out of 87), dry mouth (4 out of 87) | 0 | 4 | NA |
| ^429^ | AMI | 75mg/day | 29out of 73 | pruritus (1 out of 73), headache (2 out of 73), constipation (1 out of 73), nausea (6 out of 73), somnolence (11 out of 73), dizziness (6 out of 73), dry mouth (32 out of 73) | 0 | 1 | NA |
| ^576^ | ZE | 500mg/day | 15 out of 157 | headache (3 out of 157), nausea (1 out of 157), asthenia (2 out of 157), increased sweating (2 out of 157), dry mouth (13 out of 157) | 0 | 0 | 4 |
| ^576^ | TAU | 150mg/day | 32 out of 167 | headache (6 out of 167), nausea (12 out of 167), dizziness (12 out of 167), asthenia (11 out of 167), increased sweating (13 out of 167), dry mouth (41 out of 167) | 0 | 0 | 26 |
| ^577^ | EPA+DHA | 3,2g | 8 out of 30 (4 study discontinuation, 3 poor compliance, 1 high risk of suicide) | 0 | 0 | 0 | 0 |
| ^577^ | PLB |  | 10 out of 30 (5 study discontinuation, 3 poor compliance, 1 mild side effects, 1 high risk of suicide) | 0 | 0 | 0 | 1 |
| ^601^ | YUE+FLUO | 23 g/day+20mg/day | 0 | diarrhea (2 out of 10), constipation (2 out of 10) | 0 | 0 | 0 |
| ^601^ | FLUO+PLB | 20mg/day | 2 out of 10 (adverse events) | constipation (3 out of 8), diarrhea (2 out of 8) | 0 | 0 | 2 (insomnia and GI symptoms) |
| ^595^ | EPA+DHA+VEN | 1440mg+960mg+75-225mg/day | 15 out of 36 (2 non-compliance, 3 turning to mania, 10 unknown) | 0 | 0 | 0 | 0 |
| ^595^ | PLB+VEN | 75-225mg/day | 9 out of 36 (2 transfer treatment, 3 non compliance, 2 turning to mania, 2 unknown) | 0 | 0 | 0 | 0 |
| ^594^ | CUR+ESC | 2g/day+5-15mg/day | 8 out of 108 lost to follow-up | 0 | 0 | NA | NA |
| ^594^ | PLB+ESC | 5-15mg/day | 8 out of 108 lost to follow-up | 0 | 0 | NA | NA |
| ^578^ | vitD3 | 100,000 IU/week | 0 | 0 | 0 | 0 | 0 |
| ^578^ | PLB |  | 0 | 0 | 0 | 0 | 0 |
| Palhano-Fontes F. et al., ^600^ | AYA | 1ml/kg/day | 3 out of 17 | headache (6 out of 14), nausea (10 out of 14), anxiety (7 out of 14), restlessness (7 out of 14) | 0 | 0 | 0 |
| Palhano-Fontes F. et al., ^600^ | PLB |  | 3 out of 18 | 0 | 0 | 0 | 0 |

1.	NCT00005013. A Placebo Controlled Clinical Trial of a Standardized Extract of Hypericum Perforatum in Major Depressive Disorder. *clinicaltrials.gov*2000.

2.	NCT00042380. A Phase 2A Study of Soy Isoflavones in the Treatment of Major Depression. *clinicaltrials.gov*2002.

3.	12070867 P. St. John's Wort for depression. 900 mg per day should an effective dose. *MMW Fortschr Med* 2002; **Suppl 2:** 87.

4.	12085821 P. Study shows St. John's wort ineffective for major depression. *FDA Consum* 2002; **36**(3)**:** 8.

5.	NCT00066859. A Phase III Double-Blind Randomized Trial Comparing Sertraline (Zoloft) And Hypericum Perforatum (St. John's Wort) In Cancer Patients With Mild To Moderate Depression. *clinicaltrials.gov*2003.

6.	NCT00067301. PUFA Augmentation in Treatment of Major Depression. *clinicaltrials.gov*2003.

7.	20786190 I. St John's wort = SSRI for moderate-severe depression. *S Afr Fam Pract* 2005; **47**(4)**:** 18.

8.	NCT00122954. Fish Oil as an Adjunct Therapy for Depression in Multiple Sclerosis. *clinicaltrials.gov*2005.

9.	NCT00223236. A Double-blind, Placebo-controlled Trial of Citicoline add-on Therapy in Patients With a History of Mania or Hypomania and Cocaine Abuse/Dependence. *clinicaltrials.gov*2005.

10.	NCT00226356. Phase 4 Study: An Open Study of the Efficacy and Tolerability in Unipolar Depression of Augmentation of the One-carbon Cycle With L-methionine, Betaine and Folate. *clinicaltrials.gov*2005.

11.	NCT00256412. Essential Fatty Acids in Management of MDD - A Pilot Study. *clinicaltrials.gov*2005.

12.	NCT00480207. Randomized Double-blind Controlled Clinical Trial to Assess the Impact of Folic Acid and Omega-3 Fatty Acids Supplementation on the Severity of Depressive Symptoms in Older Adults With Identified Depression. *clinicaltrials.gov*2007.

13.	NCT00539513. A Double-Blind Study of N-Acetylcysteine Augmentation in Serotonin Reuptake Inhibitor-Refractory Obsessive-Compulsive Disorder and Depression. *clinicaltrials.gov*2007.

14.	NCT00693680. The Role of Zinc in Therapy of Unipolar Depression. *clinicaltrials.gov*2008.

15.	NCT00711971. Does EPA or DHA Prevent Depressive Symptoms in Pregnancy and Postpartum? *clinicaltrials.gov*2008.

16.	NCT00729755. Efficacy and Safety of Augmentation of Creatine for the Patients With Major Depressive Disorder. *clinicaltrials.gov*2008.

17.	NCT00816322. The Effect of Fish Oil in Major Depressive Disorder: a Double-blind Placebo-controlled Monotherapy Trail and a RCT for Recurrence Prevention. *clinicaltrials.gov*2008.

18.	NCT00861978. St. John's Wort Extract LI 160 for the Treatment of Atypical Depression. *clinicaltrials.gov*2009.

19.	NCT00955955. TRD - 2: A Double-Blind, Placebo Controlled Study of 6(S)-5-MTHF Among SSRI-Resistant Outpatients With Major Depressive Disorder (MDD). *clinicaltrials.gov*2009.

20.	NCT00997490. Phase III Placebo-controlled, Randomised, Single-centre, Double-blind Study of the Antidepressant Efficacy of a Combination Herbal Drug Preparation. *clinicaltrials.gov*2009.

21.	NCT01022632. A Randomized, Active Controlled, Open Label, Parallel Group Study to Compare the Efficacy of Extract of Curcuma Longa (Turmeric) With Fluoxetine and to Study Its Effect as an Add on Therapy to Fluoxetine in Patients of Depression. *clinicaltrials.gov*2009.

22.	Gilbert GEWAH. Omega-3 fatty acids not effective for depression in patients with coronary heart disease. *J Natl Med Assoc* 2010; **102**(2)**:** 151.

23.	NCT01175616. Creatine Augmentation in Female & Male Veterans With Selective Serotonin Reuptake Inhibitor-Resistant Major Depressive Disorder. *clinicaltrials.gov*2010.

24.	NCT01185574. Vitamin D for Improving Metabolic Control and Depressive Symptoms in Women With Diabetes; The Sunshine Study. *clinicaltrials.gov*2010.

25.	NCT01223729. Acetyl-L-Carnitine Reduces Depression and Improves Quality of Life in Patients With Minimal Hepatic Encephalopathy. *clinicaltrials.gov*2010.

26.	NCT01390662. Phase 4 Study of Vitamin D3 Supplementation for Outcomes in Patients With Unipolar Depression. *clinicaltrials.gov*2011.

27.	NCT01416818. A Randomized, Double-Blind, Placebo-Controlled Study of "Xiaoyao Pill" Treatment of Depression in Patients With Parkinson's Disease. *clinicaltrials.gov*2011.

28.	NCT01462058. The Role of Vitamin D Supplementation on Well Being and Symptoms of Depression During the Winter Season in Health Service Staff. *clinicaltrials.gov*2011.

29.	NCT01750359. The Efficacy and Safety of Adjunctive Curcumin for Treatment of Depression: A Randomized, Double-blind, Placebo-controlled Study. *clinicaltrials.gov*2012.

30.	NCT02189915. Open-Label Creatine Monohydrate Supplementation in Depressed Female Methamphetamine Users. *clinicaltrials.gov*2014.

31.	NCT02223299. A Randomized Double-Blind Placebo-Controlled Pilot Study of L-tyrosine and L-tryptophan in Depressed Patients Receiving L-methylfolate and and SSRI. *clinicaltrials.gov*2014.

32.	NCT02359266. Clinical Trial Investigating the Role of Vitamin D in the Treatment of Depression in Patients With Chronic Liver Disease. *clinicaltrials.gov*2015.

33.	NCT02381600. An Evaluation of the Effects of Vitamin D Supplementation on Specific Cognitive Functions and Depressive Symptoms of Older Subjects With Low Levels of This Vitamin. *clinicaltrials.gov*2015.

34.	NCT02568878. An Open-Label Pilot Study of Creatine for Depressed Male and Female Methamphetamine Users. *clinicaltrials.gov*2015.

35.	NCT02793648. Phase II, Double-blind, Randomized, 1-way Cross-over, to Investigate the Effectiveness of the Combination of Ascorbic Acid (Vitamin C) and Tocopherol (Vitamin E) Versus Placebo for the Treatment of Depressive Disorders in Elderly. *clinicaltrials.gov*2015.

36.	The Efficacy, Safety, and Tolerability of Probiotics on the Mood and Cognition of Depressed Patients. *clinicaltrials.gov*2016.

37.	Double Blind, Placebo Controlled Pilot Study to Evaluate the Use of Selenium as an Augmentation Treatment for Sertraline Resistant Major Depression. *clinicaltrials.gov*2016.

38.	A Randomized, Double-blind, Placebo-controlled Trial of 5-hydroxytryptophan and Creatine for SSRI or SNRI Augmentation in Treatment Resistant Depression Associated With Hypobaric Hypoxia in Females. *clinicaltrials.gov*2016.

39.	Nutritional Intervention With Mediterranean Diet in the Prevention of Recurrence of Depression. *clinicaltrials.gov*2017.

40.	The Chinese Herb SZ-05 for the Treatment of Depression and Anxiety Disorders; Efficiency, Safety and Biological Correlates. *clinicaltrials.gov*2017.

41.	Metformin, Vitamin D, and Depression in Polycystic Ovary Syndrome (PCOS) Trial: The MINDD Trial. *clinicaltrials.gov*2017.

42.	Effect of Vitamin D Administration Along With Selective Serotonin Reuptake Inhibitors (SSRIs) in Patients With Major Depressive Disorder (MDD). *clinicaltrials.gov*2018.

43.	Evaluation of Vitamin D Supplementation Effects on Serum 25(OH)D3, PTH, Pro-inflammatory Biomarkers and Neurotransmitters Involved in Depression, and Depression Status in Depressive Patients. *clinicaltrials.gov*2018.

44.	DOUBLE BLIND, RANDOMIZED, PLACEBO CONTROLLED STUDY TO EVALUATE THE EFFICACY OF SAMEUP IN SUBJECTS CHARACTERIZED BY MILD TO MODERATE DEPRESSION SYMPTOMS. *clinicaltrials.gov*2019.

45.	Multistrain Probiotic Product (De Simone Formulation) Reduces Depression and Anxiety Scores: a Randomized Pilot Study in Patients With Ulcerative Colitis. *clinicaltrials.gov*2019.

46.	Evaluation of Efficacy of Lycium Barbarum Polysaccharide in People With Subthreshold Depression: a Randomized, Placebo-controlled Trial. *clinicaltrials.gov*2019.

47.	Evaluation of Efficacy of Lycium Barbarum Polysaccharide in Patients With Major Depressive Disorder: a Randomized,Placebo-controled Trial. *clinicaltrials.gov*2019.

48.	Double-blind Placebo-controlled Clinical Trial of Lactobacillus Helveticus as an add-on Strategy for the Treatment of Major Depression. *clinicaltrials.gov*2020.

49.	A Two-Year Observational Follow-up Study of Subjects With Major Depressive Disorder Following a Randomized, Double-Blind Single-Dose of Psilocybin or Niacin-Control. *clinicaltrials.gov*2020.

50.	Effect of Zinc Supplementation on Depression in Selective Serotonin Reuptake Inhibitors-treated Major Depressive Disorder Patients. *clinicaltrials.gov*2020.

51.	Effect of Probiotic on Depressive Symptom in Chinese Patients. *clinicaltrials.gov*2020.

52.	Effect of Probiotic on Chinese Patients With Depression and Constipated Comorbidity. *clinicaltrials.gov*2020.

53.	Omega-3 Unsaturated Fatty Acid Improves Cognitive Function in Patients With Depression: a 12 Week Randomized, Double-blind, Placebo-controlled Study. *clinicaltrials.gov*2021.

54.	Use of Sumor in the Antidepressant Therapy of Subjects With Major Depression: a Randomized Placebo-controlled Clinical Trial. *clinicaltrials.gov*2021.

55.	Effects of Cholecalciferol on Depressive Symptoms in Type 2 Diabetes Mellitus Patients: Study of Neurotrophin-3, Serotonin, and C-Peptide. *clinicaltrials.gov*2021.

56.	Evaluation of Efficacy and Safety of add-on Sarcosine in Patients With Major Depressive Disorder: A Randomized Controlled Trial. *clinicaltrials.gov*2021.

57.	The Efficacy of Probiotic Compared With Placebo and Acceptance and Commitment Therapy for the Treatment of Major Depressive Disorder Among Adult Females. *clinicaltrials.gov*2022.

58.	Effect of Two Probiotic Formulations on Mental Health and Mood Biomarkers in Adults With Depressive Symptoms: A Pilot Randomized Placebo-controlled Trial. *clinicaltrials.gov*2022.

59.	Multicenter, Randomized, Double-blind, Parallel-group Controlled Nutritional Intervention Study to Evaluate the Effect of a Combination of Probiotic Strain on Postpartum Depression. *clinicaltrials.gov*2022.

60.	The Effect of Peripartum Magnesium Sulfate Use on the Occurrence of Postpartum Depression. *clinicaltrials.gov*2023.

61.	Specialized Pro-resolving Lipid Mediators and Treatment Resistant Depression. *clinicaltrials.gov*2023.

62.	Blueberries, Gut Microbiota, and Metabolites in Depressed Older Adults - A Pilot Study. *clinicaltrials.gov*2023.

63.	Comparative Efficacy of Antidepressants With L-methylfolate, B12, and Magnesium in Depressive Disorders. *clinicaltrials.gov*2023.

64.	Leucine as a Probe of Kynurenine-Induced Glutamate and Neural Circuit Dysfunction in Midlife Depression. *clinicaltrials.gov*2024.

65.	Effectiveness and Safety of Probiotics, BC99 in Relieving Anxiety and Depression in Adults. *clinicaltrials.gov*2024.

66.	The Effect of Stingless Bee Honey on BDNF, Oxidative Stress, Depressive Symptoms and Verbal Learning and Memory in Major Depressive Disorders: A Randomized Clinical Trial. *clinicaltrials.gov*2024.

67.	Actrn. A micronutrient intervention for adults experiencing symptoms of anxiety and depression. 2017.

68.	Adams PW, Rose DP, Folkard J, Wynn V, Seed M, Strong R. Effect of pyridoxine hydrochloride (vitamin B 6 ) upon depression associated with oral contraception. *Lancet* 1973; **1**(7809)**:** 899–904.

69.	Afsharfar M, Shahraki M, Shakiba M, Asbaghi O, Dashipour A. The effects of magnesium supplementation on serum level of brain derived neurotrophic factor (BDNF) and depression status in patients with depression. *Clinical Nutrition ESPEN* 2021; **42:** 381–386.

70.	Afzali A, Vakili Z, Goli S, Bagheri H, Mirhosseini S, Ebrahimi H. A Randomized Clinical Trial of the Effect of Zinc Supplement on Depression and Anxiety in the Elderly. *Open Public Health Journal* 2021; **14**(1)**:** 537–544.

71.	Ahmad S, Khan A, Rafiq H, Tikmani P, Mesiya H, Amin F. Co-administration of saffron and chamomile: to determine the efficacy as an adjuvant therapy for mild to moderate depression in human subjects. A pilot randomized clinical trial. *J Pak Med Assoc* 2023; **73**(6)**:** 1245–1250.

72.	Akhondzadeh Basti A, Choreishi SA, Noorbala AA, Akhondzadeh SH, Rezazadeh S. Petal and stigma of Crocus sativus L. in the treatment of depression: A pilot double - blind randomized trial. *J Med Plants* 2008; **7**(SUPPL. 4)**:** 29–36.

73.	Akhondzadeh S, Fallah-Pour H, Afkham K, Jamshidi AH, Khalighi-Cigaroudi F. Comparison of Crocus sativus L. and imipramine in the treatment of mild to moderate depression: a pilot double-blind randomized trial. 2004; **4**.

74.	Akhondzadeh S, Fallah-Pour H, Afkham K, Jamshidi AH, Khalighi-Cigaroudi F, Miller LG. A comparative trial of Crocus sativus L. (saffron) and imipramine in mild to moderate depression. *Focus Altern Complement Ther* 2005; **10**(1)**:** 22–23.

75.	Alavi NM, Khademalhoseini S, Vakili Z, Assarian F. Effect of vitamin D supplementation on depression in elderly patients: A randomized clinical trial. *Clin Nutr* 2019; **38**(5)**:** 2065–2070.

76.	Alcocer-Gómez E, Sánchez-Alcázar JA, Cordero MD. Coenzyme Q10 regulates serotonin levels and depressive symptoms in fibromyalgia patients: Results of a small clinical trial. *Journal of Clinical Psychopharmacology* 2014; **34**(2)**:** 277–278.

77.	Amminger GP, Nelson B, Yuen HP, Markulev C, Schafer MR, Schlogelhofer M *et al.* Neurapro revisited: increases in long-chain omega-3 fatty acids improve functional and symptomatic outcomes in ultrahigh risk patients. 2018; **44:** S228

C223 – EMBASE 621901637.

78.	Amsterdam JD, Li QS, Xie SX, Mao JJ. Putative Antidepressant Effect of Chamomile (Matricaria chamomilla L.) Oral Extract in Subjects with Comorbid Generalized Anxiety Disorder and Depression. *J Altern Complement Med* 2020; **26**(9)**:** 813–819.

79.	Andrade C. N-Acetylcysteine Augmentation for Patients With Major Depressive Disorder and Bipolar Depression. *Journal of Clinical Psychiatry* 2021; **82**(1 C7 - 21f13891).

80.	Andreeva VA, Galan P, Torrès M, Julia C, Hercberg S, Kesse-Guyot E. Supplementation with B vitamins or N-3 fatty acids and depressive symptoms in cardiovascular disease survivors: Ancillary findings from the SUpplementation with FOLate, vitamins B-6 and B-12 and/or OMega-3 fatty acids (SU.FOL.OM3) randomized trial. *American Journal of Clinical Nutrition* 2012; **96**(1)**:** 208–214.

81.	Antypa N, Smelt AHM, Strengholt A, Van Der Does AJW. Effects of omega-3 fatty acid supplementation on mood and emotional information processing in recovered depressed individuals. *Journal of Psychopharmacology* 2012; **26**(5)**:** 738–743.

82.	Appleton KM, Voyias PD, Sallis HM, Dawson S, Ness AR, Churchill R *et al.* Omega-3 fatty acids for depression in adults. *Cochrane Database of Systematic Reviews* 2021; **2021**(11 C7 - CD004692).

83.	Arnold R, Murphy-Smith J, Ng CH, Mischoulon D, Byrne GJ, Bousman CA *et al.* Predictors of the placebo response in a nutraceutical randomized controlled trial for depression. *Journal of Integrative Medicine* 2024; **22**(1)**:** 46–53.

84.	Arnold LE, Young AS, Belury MA, Cole RM, Gracious B, Seidenfeld AM *et al.* Omega-3 fatty acid plasma levels before and after supplementation: Correlations with mood and clinical outcomes in the omega-3 and therapy studies. *Journal of Child and Adolescent Psychopharmacology* 2017; **27**(3)**:** 223–233.

85.	Asadi S, Gholami MS, Siassi F, Qorbani M, Sotoudeh G. Beneficial effects of nano-curcumin supplement on depression and anxiety in diabetic patients with peripheral neuropathy: A randomized, double-blind, placebo-controlled clinical trial. *Phytother Res* 2019.

86.	Astorg P, Couthouis A, De Courcy GP, Bertrais S, Arnault N, Meneton P *et al.* Association of folate intake with the occurrence of depressive episodes in middle-aged French men and women. *British Journal of Nutrition* 2008; **100**(1)**:** 183–187.

87.	Augusto da Silva Meleiro AM. Treatment of mild and moderate depression: Evaluation of the effectiveness and tolerability of LI 160 S Hypericum perforatum extract. *Rev Bras Med* 2000; **57**(11)**:** 1313–1323.

88.	Badrasawi MM, Shahar S, Abd Manaf Z, Haron H. Effect of Talbinah food consumption on depressive symptoms among elderly individuals in long term care facilities, randomized clinical trial. 2013; **8:** 279–285

C273 – PUBMED 23493965.

89.	Bahrami A, Nikoomanesh F, Dehghani H, Aramjoo H, Ferns GA. Curcumin Ameliorates Depression, and Anxiety in Patients with Premenstrual Syndrome and Dysmenorrhea: A Triple-Blinded Placebo-Controlled Trial. *Journal of Advances in Medical and Biomedical Research* 2024; **32**(152)**:** 174–184.

90.	Barragán-Rodríguez L, Rodríguez-Morán M, Guerrero-Romero F. Efficacy and safety of oral magnesium supplementation in the treatment of depression in the elderly with type 2 diabetes: A randomized, equivalent trial. *Magnes Res* 2008; **21**(4)**:** 218–223.

91.	Bartova L, Dold M, Volz HP, Seifritz E, Möller HJ, Kasper S. Beneficial effects of Silexan on co-occurring depressive symptoms in patients with subthreshold anxiety and anxiety disorders: randomized, placebo-controlled trials revisited. *Eur Arch Psychiatry Clin Neurosci* 2023; **273**(1)**:** 51–63.

92.	Bazinet RP, Metherel AH, Chen CT, Shaikh SR, Nadjar A, Joffre C *et al.* Brain eicosapentaenoic acid metabolism as a lead for novel therapeutics in major depression. *Brain, Behavior, and Immunity* 2020; **85:** 21–28.

93.	Bazrafshan MR, Jokar M, Shokrpour N, Delam H. The effect of lavender herbal tea on the anxiety and depression of the elderly: A randomized clinical trial. *Complement Ther Med* 2020; **50**.

94.	Bitarafan S, Saboor-Yaraghi A, Sahraian MA, Soltani D, Nafissi S, Togha M *et al.* Effect of Vitamin A Supplementation on fatigue and depression in Multiple Sclerosis patients: a Double-Blind Placebo-Controlled Clinical Trial. 2016; **15**(1)**:** 13–19

C13 – PUBMED 26996107,EMBASE 608134953.

95.	Blampied M, Tylianakis JM, Bell C, Gilbert C, Rucklidge JJ. Efficacy and safety of a vitamin-mineral intervention for symptoms of anxiety and depression in adults: A randomised placebo-controlled trial “NoMAD”. *Journal of Affective Disorders* 2023; **339:** 954–964.

96.	Borja-Hart NL, Marino J. Role of omega-3 fatty acids for prevention or treatment of perinatal depression. *Pharmacotherapy* 2010; **30**(2)**:** 210–216.

97.	Bot M, Brouwer IA, Roca M, Kohls E, Penninx BWJH, Watkins E *et al.* Effect of Multinutrient Supplementation and Food-Related Behavioral Activation Therapy on Prevention of Major Depressive Disorder among Overweight or Obese Adults with Subsyndromal Depressive Symptoms: The MooDFOOD Randomized Clinical Trial. *JAMA - Journal of the American Medical Association* 2019; **321**(9)**:** 858–868.

98.	Bradley HA, Moltchanova E, Mulder RT, Dixon L, Henderson J, Rucklidge JJ. Efficacy and safety of a mineral and vitamin treatment on symptoms of antenatal depression: 12-week fully blinded randomised placebo-controlled trial (NUTRIMUM). *BJPsych Open* 2024; **10**(4 C7 - e119).

99.	Bradley HA, Mulder RT, Henderson JMT, Dixon L, Moltchanova E, Rucklidge JJ. A NUTRITIONAL APPROACH TO TREATING SYMPTOMS OF ANTENATAL DEPRESSION AND ANXIETY. THE NUTRIMUM TRIAL: A DOUBLE-BLIND, RANDOMISED CONTROLLED TRIAL. *J Paediatr Child Health* 2022; **58**(SUPPL 2)**:** 23.

100.	Brattström A. Long-term effects of St. John's wort (Hypericum perforatum) treatment: A 1-year safety study in mild to moderate depression. *Phytomedicine* 2009; **16**(4)**:** 277–283.

101.	Brendler T, Brinckmann JA, Feiter U, Gericke N, Lang L, Pozharitskaya ON *et al.* Sceletium for managing anxiety, depression and cognitive impairment: A traditional herbal medicine in modern-day regulatory systems. *Current Neuropharmacology* 2021; **19**(9)**:** 1384–1400.

102.	Brennan BP, Jensen JE, Hudson JI, Coit CE, Beaulieu A, Pope HG *et al.* A placebo-controlled trial of acetyl-l-carnitine and α-lipoic acid in the treatment of bipolar depression. *Journal of Clinical Psychopharmacology* 2013; **33**(5)**:** 627–635.

103.	Browne PD, Bolte AC, Besseling-van der Vaart I, Claassen E, de Weerth C. Probiotics as a treatment for prenatal maternal anxiety and depression: a double-blind randomized pilot trial. *Sci Rep* 2021; **11**(1)**:** 3051.

104.	C. Curtin A, Johnston CS. Vitamin B6 Supplementation Reduces Symptoms of Depression in College Women Taking Oral Contraceptives: A Randomized, Double-Blind Crossover Trial. *Journal of Dietary Supplements* 2023; **20**(4)**:** 550–562.

105.	Cao XW, Guo D, Liu JW, Niu W, Liu J, Pan JK *et al.* The efficacy and safety of the Shaoyao Shujin tablet for knee osteoarthritis: study protocol for a randomized controlled trial. *Trials* 2016; **17:** 3.

106.	Cao XJ, Huang XC, Wang X. Effectiveness of Chinese herbal medicine granules and traditional Chinese medicine-based psychotherapy for perimenopausal depression in Chinese women: a randomized controlled trial. *Menopause* 2019; **26**(10)**:** 1193–1203.

107.	Carnegie R, Borges MC, Jones HJ, Zheng J, Haycock P, Evans J *et al.* Omega-3 fatty acids and major depression: a Mendelian randomization study. *Translational Psychiatry* 2024; **14**(1 C7 - 222).

108.	Carnegie RE, Zheng J, Borges MC, Jones HJ, Wade KH, Sallis HM *et al.* Micronutrients and Major Depression: A Mendelian Randomisation Study. *Nutrients* 2024; **16**(21 C7 - 3690).

109.	Chaban О, Khaustova OO, Assonov DO, Sak LV. SAFETY AND EFFICACY OF THE COMPLEX DEPRILIUM® IN REDUCING SUBCLINICAL SYMPTOMS OF DEPRESSION IN PATIENTS WITH CHRONIC NON-COMMUNICABLE DISEASES: DOUBLE-BLIND RANDOMIZED CONTROLLED STUDY. *Wiad Lek* 2023; **76**(1)**:** 136–144.

110.	Chahwan B, Kwan S, Isik A, van Hemert S, Burke C, Roberts L. Gut feelings: A randomised, triple-blind, placebo-controlled trial of probiotics for depressive symptoms. *Journal of Affective Disorders* 2019; **253:** 317–326.

111.	Chang JPC, Chang SS, Chen HT, Su KP, Pariante CM. A double blind-placebo controlled trial of omega-3 polyunsaturated fatty acids (n-3 PUFAs) in patients with cardiovascular diseases (CVDs) and comorbid major depressive disorder (MDD). *Nutr Neurosci* 2018; **21:** S11.

112.	Chen HM, Kuo PH, Hsu CY, Chiu YH, Liu YW, Lu ML *et al.* Psychophysiological effects of lactobacillus plantarum ps128 in patients with major depressive disorder: A preliminary 8-week open trial. *Nutrients* 2021; **13**(11 C7 - 3731).

113.	Chhetry BT, Hezghia A, Miller JM, Lee S, Rubin-Falcone H, Cooper TB *et al.* Omega-3 polyunsaturated fatty acid supplementation and white matter changes in major depression. *Journal of Psychiatric Research* 2016; **75:** 65–74.

114.	Chi CI. Safety, effectiveness, and mechanism of fish oil as adjunct treatment for Major Depressive Disorder - a 12-month randomized, placebo controlled clinical trial. 2017.

115.	Chinna Meyyappan A, Forth E, Milev R. Microbial Ecosystem Therapeutic-2 Intervention in People With Major Depressive Disorder and Generalized Anxiety Disorder: Phase 1, Open-Label Study. *Interact J Med Res* 2022; **11**(1)**:** e32234.

116.	Chong MFF, Wong JXY, Colega M, Chen LW, van Dam RM, Tan CS *et al.* Relationships of maternal folate and vitamin B12 status during pregnancy with perinatal depression: The GUSTO study. *Journal of Psychiatric Research* 2014; **55**(1)**:** 110–116.

117.	Choukri MA, Conner TS, Haszard JJ, Harper MJ, Houghton LA. Effect of vitamin D supplementation on depressive symptoms and psychological wellbeing in healthy adult women: A double-blind randomised controlled clinical trial. *J Nutr Sci* 2018; **7**.

118.	Coët A, Blampied FM, Rucklidge JJ. 1-year naturalistic follow-up of a Randomised Double-Blind, Placebo-Controlled Trial (“NoMAD”) Exploring the Effectiveness of Micronutrients in Improving Symptoms of Anxiety and Depression. *J Affec Disord Rep* 2025; **20**.

119.	Cropley M, Banks AP, Boyle J. The Effects of Rhodiola rosea L. Extract on Anxiety, Stress, Cognition and Other Mood Symptoms. *Phytother Res* 2015; **29**(12)**:** 1934–1939.

120.	Ctis. EFFECTIVENESS OF SUPPLEMENTATION WITH VITAMIN D IN PATIENTS WITH DEPRESSIVE DISORDER OR BIPOLAR DISORDER: a RANDOMIZED CONTROLLED CLINICAL TRIAL. 2025.

121.	Dabbaghmanesh MH, Vaziri F, Najib F, Nasiri S, Pourahmad S. The effect of vitamin D consumption during pregnancy on maternal thyroid function and depression: A randomized, placebo-controlled, clinical trial. *Jundishapur Journal of Natural Pharmaceutical Products* 2019; **14**(2 C7 - e65328).

122.	de Koning EJ, Lips P, Penninx B, Elders PJM, Heijboer AC, den Heijer M *et al.* Vitamin D supplementation for the prevention of depression and poor physical function in older persons: the D-Vitaal study, a randomized clinical trial. 2019; **110**(5)**:** 1119–1130

C1113 – PUBMED 31340012,EMBASE 628896621.

123.	de Koning EJ, van der Zwaluw NL, van Wijngaarden JP, Sohl E, Brouwer-Brolsma EM, van Marwijk HW *et al.* Effects of Two-Year Vitamin B(12) and Folic Acid Supplementation on Depressive Symptoms and Quality of Life in Older Adults with Elevated Homocysteine Concentrations: Additional Results from the B-PROOF Study, an RCT. *Nutrients* 2016; **8**(11).

124.	de Sousa-Muñoz RL, Filizola RG. Efficacy of soy isoflavones for depressive symptoms of the climacteric syndrome. *Maturitas* 2009; **63**(1)**:** 89–93.

125.	Di Pierro F, Orsi R, Settembre R. Role of betaine in improving the antidepressant effect of S-adenosyl-methionine in patients with mild-to-moderate depression. *J MultidiscipHealthc* 2015; **8:** 39–45.

126.	Effati Daryani F, Charandabi SMA, Zarei S, Mohammadi A, Mirghafourvand M. Effect of lavender cream with or without footbath on anxiety, stress and depression of women in postpartum: A clinical randomized controlled trial. *Iran J Obstet Gynecol Infertil* 2017; **20**(10)**:** 52–61.

127.	Esmaily H, Sahebkar A, Iranshahi M, Ganjali S, Mohammadi A, Ferns G *et al.* An investigation of the effects of curcumin on anxiety and depression in obese individuals: A randomized controlled trial. *Chin J Integr Med* 2015; **21**(5)**:** 332–338.

128.	Fard FE, Mirghafourvand M, Mohammad-Alizadeh Charandabi S, Farshbaf-Khalili A, Javadzadeh Y, Asgharian H. Effects of zinc and magnesium supplements on postpartum depression and anxiety: A randomized controlled clinical trial. *Women and Health* 2017; **57**(9)**:** 1115–1128.

129.	Farioli-Vecchioli S, Sacchetti S, di Robilant NV, Cutuli D. The role of physical exercise and omega-3 fatty acids in depressive illness in the elderly. *Current Neuropharmacology* 2018; **16**(3)**:** 308–326.

130.	Fava M, Borus JS, Alpert JE, Nierenberg AA, Rosenbaum JF, Bottiglieri T. Folate, vitamin B12, and homocysteine in major depressive disorder. *AM J PSYCHIATRY* 1997; **154**(3)**:** 426–428.

131.	Fava M, Gomeni R, Komorowski J, Docherty J, Cusin C, Soskin D *et al.* A re-analysis using a population-enrichment strategy of a double-blind, placebo-controlled study of chromium picolinate in atypical depression. *Neuropsychopharmacology* 2012; **38:** S341–S342.

132.	Fava M, Lamon-Fava S, So J, Mischoulon D, Nierenberg A, Dunlop B *et al.* Eicosapentaenoic acid (EPA) supplementation in major depressive disorder patients with chronic inflammation: Dose-dependent increases in EPA metabolites and specialized pro-resolving lipid mediators. *Neuropsychopharmacology* 2019; **44:** 451–452.

133.	Fedotova J, Bereza Z, Dudnichenko T. Efficacy of escitalopram in combination with vitamin D3 supplementation in the depressed perimenopausal women. *Eur Psychiatry* 2020; **63:** S109–S110.

134.	Firoozabadi A, Kolouri S, Zarshenas MM, Salehi A, Mosavat SH, Dastgheib SA. Efficacy of Nepeta Menthoides Boiss and Buhse Freeze-Dried Aqueous Extract on Anxiety of Patients with Depression: A Double-Blind Randomized Controlled Clinical Trial. 2016; **41**(3 Suppl)**:** S4

C3 – PUBMED 27840470.

135.	Firoozabadi A, Kolouri S, Zarshenas MM, Salehi A, Mosavat SH, Dastgheib SA. Efficacy of a freeze-dried aqueous extract of Nepeta menthoides Boiss. & Buhse in the treatment of anxiety in patients with depression: A double-blind, randomized, controlled trial. *J Herbal Med* 2017; **10:** 17–23.

136.	Foshati S, Ghanizadeh A, Akhlaghi M. Extra-Virgin Olive Oil Improves Depression Symptoms Without Affecting Salivary Cortisol and Brain-Derived Neurotrophic Factor in Patients With Major Depression: a Double-Blind Randomized Controlled Trial. 2022; **122**(2)**:** 284–297.e281

C283 – PUBMED 34358723,EMBASE 635814426.

137.	An open trial of Omega-3 fatty acids for depression in pregnancy. *Proceedings of the Acta Neuropsychiatrica*2006.

138.	Freeman MP, Hibbeln JR, Wisner KL, Brumbach BH, Watchman M, Gelenberg AJ. Randomized dose-ranging pilot trial of omega-3 fatty acids for postpartum depression. *Acta Psychiatrica Scandinavica* 2006; **113**(1)**:** 31–35.

139.	Freeman MP, Hibbeln JR, Silver M, Hirschberg AM, Wang B, Yule AM *et al.* Omega-3 fatty acids for major depressive disorder associated with the menopausal transition: A preliminary open trial. *Menopause* 2011; **18**(3)**:** 279–284.

140.	Freeman MP, Savella GM, Church TR, Goéz-Mogollón L, Sosinsky AZ, Noe OB *et al.* A prenatal supplement with methylfolate for the treatment and prevention of depression in women trying to conceive and during pregnancy. *Annals of Clinical Psychiatry* 2019; **31**(1)**:** 4–16.

141.	Fries LR, Boehme M, Lavalle L, Sakwinska O, Chughlay F, Keddani S *et al.* The impact of ingestion of Bifidobacterium longum NCC3001 on perinatal anxiety and depressive symptoms: a randomized controlled trial. *Sci Rep* 2025; **15**(1)**:** 11250.

142.	Fulzele AV, Ingle N, Huda MN, Mishra DS. Comparative study to evaluate the effect of a herbal preparation & shirodhara in the management of major depressive disorder. *Int J Pharm Res* 2014; **6**(2)**:** 506–511.

143.	Gabbay V, Freed RD, Alonso CM, Senger S, Stadterman J, Davison BA *et al.* A double-blind placebo-controlled trial of omega-3 fatty acids as a monotherapy for adolescent depression. *Journal of Clinical Psychiatry* 2018; **79**(4 C7 - 17m11596).

144.	Gariballa S, Forster S. Effects of dietary supplements on depressive symptoms in older patients: A randomised double-blind placebo-controlled trial. *Clin Nutr* 2007; **26**(5)**:** 545–551.

145.	Gawlik-Kotelnicka O, Margulska A, Skowrońska A, Strzelecki D. PRO-DEMET Randomized Controlled Trial on Probiotics in Depression-Pilot Study Results. *Nutrients* 2023; **15**(6).

146.	Gawlik-Kotelnicka O, Rogalski J, Czarnecka-Chrebelska KH, Burzyński J, Jakubowska P, Skowrońska A *et al.* The Interplay Between Depression, Probiotics, Diet, Immunometabolic Health, the Gut, and the Liver—A Secondary Analysis of the Pro-Demet Randomized Clinical Trial. *Nutrients* 2024; **16**(23 C7 - 4024).

147.	Gawlik-Kotelnicka O, Skowrońska A, Margulska A, Czarnecka-Chrebelska KH, Łoniewski I, Skonieczna-żydecka K *et al.* The influence of probiotic supplementation on depressive symptoms, inflammation, and oxidative stress parameters and fecal microbiota in patients with depression depending on metabolic syndrome comorbidity—pro-demet randomized study protocol. *Journal of Clinical Medicine* 2021; **10**(7 C7 - 1342).

148.	Gawlik-Kotelnicka O, Skowrońska A, Margulska A, Strzelecki D. The Influence of Probiotic Supplementation on Depression, Anxiety, and Stress Level, as well as Inflammation, Anthropometric and Metabolic Parameters in Patients with Depressive Disorders-preliminary results of an RCT. *Eur Psychiatry* 2023; **66:** S841–S842.

149.	Gecele M, Francesetti G, Meluzzi A. Acetyl-L-carnitine in aged subjects with major depression: Clinical efficacy and effects on the circadian rhythm of cortisol. *DEMENTIA* 1991; **2**(6)**:** 333–337.

150.	Ghamchini VM, Salami M, Mohammadi GR, Moradi Z, Kavosi A, Movahedi A *et al.* The effect of chamomile tea on anxiety and depression in cancer patients treated with chemotherapy. *J Young Pharm* 2019; **11**(3)**:** 309–312.

151.	Gharekhani A, Khatami MR, Dashti-Khavidaki S, Razeghi E, Noorbala AA, Hashemi-Nazari SS *et al.* The effect of omega-3 fatty acids on depressive symptoms and inflammatory markers in maintenance hemodialysis patients: A randomized, placebo-controlled clinical trial. *European Journal of Clinical Pharmacology* 2014; **70**(6)**:** 655–665.

152.	Ginsberg DL. Chromium Picolinate Treatment of Atypical Depression. *Prim Psychiatry* 2003; **10**(4)**:** 23–24.

153.	Grobler AC, Matthews G, Molenberghs G. The impact of missing data on clinical trials: A re-analysis of a placebo controlled trial of Hypericum perforatum (St Johns wort) and sertraline in major depressive disorder. *Psychopharmacology* 2014; **231**(9)**:** 1987–1999.

154.	Haberka M, Mizia-Stec K, Mizia M, Gieszczyk K, Chmiel A, Sitnik-Warchulska K *et al.* Effects of n-3 polyunsaturated fatty acids on depressive symptoms, anxiety and emotional state in patients with acute myocardial infarction. 2012; **19**(1)**:** S42

C43 – EMBASE 71706389.

155.	Haghighat N, Rajabi S, Mohammadshahi M. Effect of synbiotic and probiotic supplementation on serum brain-derived neurotrophic factor level, depression and anxiety symptoms in hemodialysis patients: a randomized, double-blinded, clinical trial. *Nutr Neurosci* 2021; **24**(6)**:** 490–499.

156.	Hallahan B, Ryan T, Hibbeln JR, Murray IT, Glynn S, Ramsden CE *et al.* Efficacy of omega-3 highly unsaturated fatty acids in the treatment of depression. *British Journal of Psychiatry* 2016; **209**(3)**:** 192–201.

157.	Hamlbar EP, Mirghafourvand M, Shaseb E, Kamalifard M. The effect of curcumin on postpartum depression and anxiety in primiparous women: a double-blind randomized placebo-controlled clinical trial. *BMC Compl MedTherapies* 2025; **25**(1).

158.	Hatton DC, Harrison-Hohner J, Matarazzo J, Edwards P, Lewy A, Davis L. Missed antenatal depression among high risk women: A secondary analysis. *Archives of Women's Mental Health* 2007; **10**(3)**:** 121–123.

159.	Haybar H, Javid AZ, Haghighizadeh MH, Valizadeh E, Mohaghegh SM, Mohammadzadeh A. The effects of Melissa officinalis supplementation on depression, anxiety, stress, and sleep disorder in patients with chronic stable angina. *Clinical Nutrition ESPEN* 2018; **26:** 47–52.

160.	Heaton JL, Campbell SA, Bradley HA, Mulder RT, Dixon L, Henderson J *et al.* Broad-Spectrum Micronutrients or Antidepressants for Antenatal Depression: Effect on Maternal and Infant Birth Outcomes in an Observational Secondary Analysis of NUTRIMUM. *Journal of Clinical Psychopharmacology* 2025; **45**(1)**:** 4–15.

161.	Hellem TL, Sung Y-H, Shi X-F, Pett MA, Latendresse G, Morgan J *et al.* Creatine as a novel treatment for depression in females using methamphetamine: A pilot study. *Journal of Dual Diagnosis* 2015; **11**(3-4)**:** 189–202.

162.	Hidese S, Ota M, Wakabayashi C, Noda T, Ozawa H, Okubo T *et al.* Effects of chronic l-theanine administration in patients with major depressive disorder: An open-label study. *Acta Neuropsychiatr* 2017; **29**(2)**:** 72–79.

163.	Ho YT, Tsai YC, Kuo TBJ, Yang CCH. Effects of lactobacillus plantarum ps128 on depressive symptoms and sleep quality in self-reported insomniacs: A randomized, double-blind, placebo-controlled pilot trial. *Nutrients* 2021; **13**(8 C7 - 2820).

164.	Holsboen F, Benkert O, Meier L, Kreuz-Kersting A. Combined estradiol and vitamin B6 treatment in women with major depression. *Am J Psychiatry* 1985; **142**(5)**:** 658.

165.	Hulkkonen P, Kataja EL, Vahlberg T, Koivuniemi E, Houttu N, Pellonperä O *et al.* The efficacy of probiotics and/or n-3 long-chain polyunsaturated fatty acids intervention on maternal prenatal and postnatal depressive and anxiety symptoms among overweight and obese women. *Journal of Affective Disorders* 2021; **289:** 21–30.

166.	Ibero-Baraibar I, Perez-Cornago A, Ramirez MJ, Martinez JA, Zulet MA. An increase in plasma homovanillic acid with cocoa extract consumption is associated with the alleviation of depressive symptoms in overweight or obese adults on an energy restricted diet in a randomized controlled trial. 2015; **146**(4)**:** 897S–904S

C893 – PUBMED 26962189,EMBASE 610003794.

167.	Irct138901233695N. Effect of vitamins in treatment of depression. 2010.

168.	Irct201109297669N. The impact of Omga-3 fatty acids on depression. 2011.

169.	Irct2014041916465N. Evaluating the effects of Crocus sativus L. (Saffron) on weight and depression. 2014.

170.	Irct2014082518922N. Effect of Folic acid supplementation on the therapeutic regimen for major depressive disorder. 2014.

171.	Irct20170311033004N. Effects of alkaloids of harmala seeds on depression. 2022.

172.	Irct2017082612438N. The effect of barij deprex capsule on Depressive symptoms. 2017.

173.	Irct20240107060642N. Investigating the effect of folic acid supplementation on postpartum depression in women. 2024.

174.	Irct20240619062173N. Effect of Cuscuta epithymum on depression. 2024.

175.	Islambulchilar M, Morovati M, Morsali Y, Rezaee S, Esmaeilzadeh A. Could one-milligram daily folic acid supplementation change the intensity of the disorder in outpatients with depression who use citalopram? *Koomesh* 2023; **25**(5).

176.	Isrctn. Probiotic supplementation for maternal depression. 2024.

177.	j57vj RBR. Effect of Magnesium Chloride supplementation on Depression. 2024.

178.	Jam IN, Sahebkar AH, Eslami S, Mokhber N, Nosrati M, Khademi M *et al.* The effects of crocin on the symptoms of depression in subjects with metabolic syndrome. *Adv Clin Exp Med* 2017; **26**(6)**:** 925–930.

179.	Javadinia SA, Salek R, Dehghani M, Mohajeri SA, Talaei A, Fanipakdel A. Amelioration of anxiety, depression, and chemotherapy related toxicity after crocin administration during chemotherapy of breast cancer: A double blind, randomized clinical trial. *Ann Oncol* 2021; **32:** S439–S440.

180.	Jeong HG, Ko YH, Oh SY, Han C, Kim T, Joe SH. Effect of Korean Red Ginseng as an adjuvant treatment for women with residual symptoms of major depression. *Asia-Pacific Psychiatry* 2015; **7**(3)**:** 330–336.

181.	Jiang W, Whellan DJ, Adams KF, Babyak MA, Boyle SH, Wilson JL *et al.* Long-Chain Omega-3 Fatty Acid Supplements in Depressed Heart Failure Patients: Results of the OCEAN Trial. *JACC: Heart Failure* 2018; **6**(10)**:** 833–843.

182.	Jokar A, Farahi F. Effect of vitamin C on depression in menopausal women with balanced diet: A randomized clinical trial. *Iran J Obstet Gynecol Infertil* 2014; **17**(121)**:** 18–23.

183.	Jorde R, Kubiak J. No improvement in depressive symptoms by vitamin D supplementation: Results from a randomised controlled trial. *J Nutr Sci* 2018; **7**.

184.	Jorde R, Sneve M, Figenschau Y, Svartberg J, Waterloo K. Effects of vitamin D supplementation on symptoms of depression in overweight and obese subjects: Randomized double blind trial. *Journal of Internal Medicine* 2008; **264**(6)**:** 599–609.

185.	Jurcic J, Pereira JA, Kavanaugh D. St John's wort versus paroxetine for depression. *Can Fam Phys* 2007; **53**(9)**:** 1511–1513.

186.	Karakula-Juchnowicz H, Rog J, Juchnowicz D, Łoniewski I, Skonieczna-Ydecka K, Krukow P *et al.* The study evaluating the effect of probiotic supplementation on the mental status, inflammation, and intestinal barrier in major depressive disorder patients using gluten-free or gluten-containing diet (SANGUT study): A 12-week, randomized, double-blind, and placebo-controlled clinical study protocol. *Nutr J* 2019; **18**(1).

187.	Karimi FZ, Hosseini H, Mazloom SR, Rakhshandeh H. Effect of oral capsule of Ocimum basilicum leaf extract on depression in menopausal women: A randomized clinical trial. *Iran J Obstet Gynecol Infertil* 2021; **24**(2)**:** 50–58.

188.	Kasper S, Caraci F, Forti B, Drago F, Aguglia E. Efficacy and tolerability of Hypericum extract for the treatment of mild to moderate depression. *Eur Neuropsychopharmacol* 2010; **20**(11)**:** 747–765.

189.	Kasper S, Gastpar M, Müller WE, Volz HP, Dienel A, Möller HJ. Treatment of depression with St. John's wort extract WS® 5570. No influence on patients' weight. *Psychopharmakotherapie* 2008; **15**(1)**:** 6–10.

190.	Kasper S, Seifritz E, Volz HP. Efficacy of Silexan in Patients with a Major Depressive Episode - First Results from a Multi-centre, Doubleblind, Randomised, Placebo- and Reference-controlled Phase III Trial. *Eur Psychiatry* 2024; **67:** S224–S225.

191.	Kasper DC, Widness JA, Haiden N, Berger A, Hayde M, Pollak A *et al.* Characterization and differentiation of iron status in anemic very low birth weight infants using a diagnostic nomogram. *Neonatology* 2009; **95**(2)**:** 164–171.

192.	Katrenčíková B, Vaváková M, Paduchová Z, Nagyová Z, Garaiova I, Muchová J *et al.* Oxidative stress markers and antioxidant enzymes in children and adolescents with depressive disorder and impact of omega-3 fatty acids in randomised clinical trial. *Antioxidants* 2021; **10**(8 C7 - 1256).

193.	Kazemi A, Noorbala AA, Azam K, Eskandari MH, Djafarian K. Effect of probiotic and prebiotic vs placebo on psychological outcomes in patients with major depressive disorder: A randomized clinical trial. *Clin Nutr* 2019; **38**(2)**:** 522–528.

194.	Kazemi A, Noorbala AA, Djafarian K. Effect of probiotic and prebiotic versus placebo on appetite in patients with major depressive disorder: post hoc analysis of a randomised clinical trial. *Journal of Human Nutrition and Dietetics* 2020; **33**(1)**:** 56–65.

195.	Keshavarz SA, Mostafavi SA, Akhondzadeh S, Mohammadi MR, Hosseini S, Eshraghian MR *et al.* Omega-3 supplementation effects on body weight and depression among dieter women with co-morbidity of depression and obesity compared with the placebo: A randomized clinical trial. *Clinical Nutrition ESPEN* 2018; **25:** 37–43.

196.	Khaddage R, Bassil M, Rahi B. Vitamin D Supplementation Improves Depressive Symptomatology Among Vitamin D Deficient Saudi Arabian Adults - A Randomized Clinical Trial. *Curr Dev Nutr* 2023; **7**.

197.	Khajehnasiri F, Akhondzadeh S, Mortazavi SB, Allameh A, Sotoudeh G, Khavanin A *et al.* Are supplementation of omega-3 and ascorbic acid effective in reducing oxidative stress and depression among depressed shift workers? *International Journal for Vitamin and Nutrition Research* 2015; **85**(5-6)**:** 299–310.

198.	Khalatbari Mohseni G, Hosseini SA, Majdinasab N, Cheraghian B. Effects of N-acetylcysteine on oxidative stress biomarkers, depression, and anxiety symptoms in patients with multiple sclerosis. *Neuropsychopharmacology Reports* 2023; **43**(3)**:** 382–390.

199.	Khalili M, Karimi H, Sadri S. Effect of fish oil supplement and weight loss diet on depression in obese women. *Obes Rev* 2011; **12:** 70.

200.	Khanna A, John F, Das S, Thomas J, Rao J, Maliakel B *et al.* Efficacy of a novel extract of fenugreek seeds in alleviating vasomotor symptoms and depression in perimenopausal women: A randomized, double-blinded, placebo-controlled study. *J Food Biochem* 2020; **44**(12)**:** e13507.

201.	Khodadad M, Bahadoran P, Kheirabadi G, Sabzghabaee A. Can Vitamin B6 help to prevent postpartum depression? A randomized controlled trial. *International Journal of Preventive Medicine* 2021; **12**(1 C7 - 12).

202.	Khorvash F, Hajihashemi P, Askari G, Noorian M, Maracy MR. The effects of combined supplementation of coenzyme Q10 with L-carnitine on migraine-related disability and depression among patient with migraine: A randomized, placebo-controlled, double-blind trial. *J Neurol Sci* 2019; **405:** 106.

203.	Kieser M, Szegedi A. Predicting stable treatment response in patients with major depression treated with hypericum extract WS® 5570/5572. *Pharmacopsychiatry* 2005; **38**(5)**:** 194–200.

204.	Kim Y, Choi Y, Lee MY, Cho SH, Jung IC, Kang DH *et al.* Bangpungtongsung-san for patients with major depressive disorder: study protocol for a randomized controlled phase II clinical trial. *BMC Complement Med Ther* 2023; **23**(1)**:** 114.

205.	Kious BM, Sabic H, Sung Y-H, Kondo DG, Renshaw P. An open-label pilot study of combined augmentation with creatine monohydrate and 5-hydroxytryptophan for selective serotonin reuptake inhibitor–or serotonin-norepinephrine reuptake inhibitor–resistant depression in adult women. *Journal of clinical psychopharmacology* 2017; **37**(5)**:** 578–583.

206.	Komorniak N, Kaczmarczyk M, Łoniewski I, Martynova-Van Kley A, Nalian A, Wroński M *et al.* Analysis of the Efficacy of Diet and Short-Term Probiotic Intervention on Depressive Symptoms in Patients after Bariatric Surgery: A Randomized Double-Blind Placebo Controlled Pilot Study. *Nutrients* 2023; **15**(23 C7 - 4905).

207.	Kotlega D, Zembron-Lacny A, Golab-Janowska M, Nowacki P, Szczuko M. The association of free fatty acids and eicosanoids with the severity of depressive symptoms in stroke patients. *International Journal of Molecular Sciences* 2020; **21**(15 C7 - 5220)**:** 1–18.

208.	Lagzi N, Bateni A, Goli R, Talebiazar N. The effect of multivitamins on anxiety and depression in patients undergoing methadone maintenance treatment: A double-blind randomized controlled trial. *International Journal of Psychiatry in Medicine* 2023; **58**(6)**:** 576–590.

209.	Lamon-Fava S, Liu M, Dunlop BW, Kinkead B, Schettler PJ, Felger JC *et al.* Clinical response to EPA supplementation in patients with major depressive disorder is associated with higher plasma concentrations of pro-resolving lipid mediators. *Neuropsychopharmacology* 2023; **48**(6)**:** 929–935.

210.	Lc R, Em B, Db H, H S, Ar P. A pilot randomized controlled trial testing supplements of omega-3 fatty acids, probiotics, combination or placebo on symptoms of depression, anxiety and stress. *J Affec Disord Rep* 2021; **5 C7 - 100141**.

211.	Lecrubier Y, Clerc G, Didi R, Kieser M. Efficacy of St. John's wort extract WS 5570 in major depression: A double-blind, placebo-controlled trial. *AM J PSYCHIATRY* 2002; **159**(8)**:** 1361–1366.

212.	Lee YJ, Sohng KY. Effects of Vitamin D on depression, cognitive function, and physical function in elderly individuals living alone. *International Journal of Gerontology* 2019; **13**(3)**:** 196–201.

213.	Lespérance F, Frasure-Smith N, St-André E, Turecki G, Lespérance P, Wisniewski SR. The efficacy of omega-3 supplementation for major depression: A randomized controlled trial. *Journal of Clinical Psychiatry* 2011; **72**(8)**:** 1054–1062.

214.	Levant B. Brain N-3 (Omega-3) fatty acids in neurochemistry, behavior and mood: Implications for the treatment and prevention of non-puerperal and postpartum depression. *Lipids: Categories, Biological Functions and Metabolism, Nutrition and Health*2011, pp 127–152.

215.	Liang ZH, Jia YB, Wang ML, Li ZR, Li M, Yun YL *et al.* Efficacy of ginkgo biloba extract as augmentation of venlafaxine in treating post-stroke depression. *Neuropsychiatr Dis Treat* 2019; **15:** 2551–2557.

216.	Lin C, Lee SH, Huang CM, Wu YW, Chang YX, Liu HL *et al.* Cognitive protection and brain entropy changes from omega-3 polyunsaturated fatty acids supplement in late-life depression: A 52-week randomized controlled trial. *Journal of Affective Disorders* 2024; **351:** 15–23.

217.	Lin K, Li Y, Toit ED, Wendt L, Sun J. Effects of Polyphenol Supplementations on Improving Depression, Anxiety, and Quality of Life in Patients With Depression. *Frontiers in Psychiatry* 2021; **12 C7 - 765485**.

218.	Lingærde O, Føreland AR, Magnusson A. Can winter depression be prevented by Ginkgo biloba extract? A placebo- controlled trial. *Acta Psychiatrica Scandinavica* 1999; **100**(1)**:** 62–66.

219.	Lipovac M, Chedraui P, Gruenhut C, Gocan A, Stammler M, Imhof M. Improvement of postmenopausal depressive and anxiety symptoms after treatment with isoflavones derived from red clover extracts. *Maturitas* 2010; **65**(3)**:** 258–261.

220.	Loas G, Rose D, Nowaczkowski P, Lernout P, Duron B. Proportion of non-responsive patient to amitriptyline: Exploratory pilot study of the French center. A report from the WHO project on the combined utilization of an antidepressant (amitriptyline) and an antioxidant (ginkgo biloba) in the treatment of therapy-resistant depression. *ANN MED-PSYCHOL* 1996; **154**(3)**:** 202–203.

221.	Lopresti AL, Maes M, Maker GL, Hood SD, Drummond PD. Curcumin for the treatment of major depression: A randomised, double-blind, placebo controlled study. *Journal of Affective Disorders* 2014; **167:** 368–375.

222.	Lopresti AL, Smith SJ, Malvi H, Kodgule R. An investigation into the stress-relieving and pharmacological actions of an ashwagandha (Withania somnifera) extract: a randomized, double-blind, placebo-controlled study. 2019; **98**(37)**:** e17186

C17183 – PUBMED 31517876.

223.	Lopresti AL, Smith SJ, Hood SD, Drummond PD. Efficacy of a standardised saffron extract (affron®) as an add-on to antidepressant medication for the treatment of persistent depressive symptoms in adults: A randomised, double-blind, placebo-controlled study. *Journal of Psychopharmacology* 2019; **33**(11)**:** 1415–1427.

224.	Lopresti AL, Smith SJ, Marx W, Díez-Municio M, Morán-Valero MI. An Examination into the Effects of a Saffron Extract (Affron) on Mood and General Wellbeing in Adults Experiencing Low Mood: A Randomized, Double-Blind, Placebo-Controlled Trial. *J Nutr* 2025.

225.	Loria-Kohen V, Gómez-Candela C, Palma-Milla S, Amador-Sastre B, Hernanz A, Bermejo LM. A pilot study of folic acid supplementation for improving homocysteine levels, cognitive and depressive status in eating disorders. *Nutricion Hospitalaria* 2013; **28**(3)**:** 807–815.

226.	Lu J, Mao H, Tan Y, Luo G. Associations of Dietary Intake of Vitamin B6 and Plasma Pyridoxal 5′-Phosphate Level With Depression in US Adults: Findings From NHANES 2005–2010. *Brain and Behavior* 2024; **14**(11 C7 - e70128).

227.	Lucas M, Asselin G, Mérette C, Poulin MJ, Dodin S. Ethyl-eicosapentaenoic acid for the treatment of psychological distress and depressive symptoms in middle-aged women: A double-blind, placebo-controlled, randomized clinical trial. *American Journal of Clinical Nutrition* 2009; **89**(2)**:** 641–651.

228.	Lucas M, Mirzaei F, O'Reilly EJ, Pan A, Willett WC, Kawachi I *et al.* Dietary intake of n-3 and n-6 fatty acids and the risk of clinical depression in women: A 10-y prospective follow-up study. *American Journal of Clinical Nutrition* 2011; **93**(6)**:** 1337–1343.

229.	Luger M, Kruschitz R, Marculescu R, Klammer C, Adelfang M, Kral M *et al.* Depressive symptoms and associations with low vitamin d levels and adherence to mediterranean diet in morbidly obese patients. 2015; **8:** 188

C183 – EMBASE 71907835.

230.	Luke AK, Evans EW, Bond DS, Thomas JG. Associations between omega fatty acid consumption and depressive symptoms among individuals seeking behavioural weight loss treatment. *Obesity Science and Practice* 2016; **2**(1)**:** 75–82.

231.	Mac Giollabhui N, Mischoulon D, Dunlop BW, Kinkead B, Schettler PJ, Liu RT *et al.* Individuals with depression exhibiting a pro-inflammatory phenotype receiving omega-3 polyunsaturated fatty acids experience improved motivation-related cognitive function: Preliminary results from a randomized controlled trial. *Brain, Behavior, and Immunity - Health* 2023; **32 C7 - 100666**.

232.	Mahboobi S, Ghasvarian M, Ghaem H, Alipour H, Alipour S, Eftekhari MH. Effects of probiotic and magnesium co-supplementation on mood, cognition, intestinal barrier function and inflammation in individuals with obesity and depressed mood: A randomized, double-blind placebo-controlled clinical trial. *Frontiers in Nutrition* 2022; **9 C7 - 1018357**.

233.	Majeed M, Nagabhushanam K, Murali A, Vishwanathan DT, Mamidala RV, Mundkur L. A Standardized Withania somnifera (Linn.) Root Extract with Piperine Alleviates the Symptoms of Anxiety and Depression by Increasing Serotonin Levels: A Double-Blind, Randomized, Placebo-Controlled Study. *J Integr Complement Med* 2024; **30**(5)**:** 459–468.

234.	Malaguarnera G, Bertino G, Chisari G, Motta M, Vecchio M, Vacante M *et al.* Silybin supplementation during HCV therapy with pegylated interferon-a plus ribavirin reduces depression and anxiety and increases work ability. 2016; **16**(1).

235.	Mannel M, Kuhn U, Schmidt U, Ploch M, Murck H. St. John's wort extract LI160 for the treatment of depression with atypical features - A double-blind, randomized, and placebo-controlled trial. *Journal of Psychiatric Research* 2010; **44**(12)**:** 760–767.

236.	Maroufi M, Kianvash F, Alavi MR. Comparison of Citrus aurantium and imipramine for treatment of major depressive disorder. *Eur Neuropsychopharmacol* 2010; **20:** S369.

237.	Matthews J, Torres SJ, Milte CM, Hopkins I, Kukuljan S, Nowson CA *et al.* Effects of a multicomponent exercise program combined with calcium–vitamin D3-enriched milk on health-related quality of life and depressive symptoms in older men: secondary analysis of a randomized controlled trial. *European Journal of Nutrition* 2020; **59**(3)**:** 1081–1091.

238.	Mayers AG, Baldwin DS, Dyson R, Middleton RW, Mustapha A. Use of St. John's wort (Hypericum perforatum L) in members of a depression self-help organisation: A 12-week open prospective pilot study using the HADS scale. *Prim Care Psychiatry* 2003; **9**(1)**:** 15–20.

239.	Mazaherioun M, Saedisomeolia A, Javanbakht MH, Koohdani F, Zarei M, Ansari S *et al.* Long chain n-3 fatty acids improve depression syndrome in type 2 diabetes mellitus. *Iranian Journal of Public Health* 2018; **47**(4)**:** 575–583.

240.	Mazereeuw G, Herrmann N, Oh PI, Ma DWL, Wang CT, Kiss A *et al.* Omega-3 fatty acids, depressive symptoms, and cognitive performance in patients with coronary artery disease. *Journal of Clinical Psychopharmacology* 2016; **36**(5)**:** 436–444.

241.	Mazloom Z, Ekramzadeh M, Hejazi N. Efficacy of supplementary vitamins C and E on anxiety, depression and stress in type 2 diabetic patients: A randomized, single-blind, placebo-controlled trial. *Pakistan Journal of Biological Sciences* 2013; **16**(22)**:** 1597–1600.

242.	McKetin R, Dean OM, Turner A, Kelly PJ, Quinn B, Lubman DI *et al.* A study protocol for the N-ICE trial: a randomised double-blind placebo-controlled study of the safety and efficacy of N-acetyl-cysteine (NAC) as a pharmacotherapy for methamphetamine ("ice") dependence. 2019; **20**(1)**:** 325

C323 – PUBMED 31164169.

243.	Mech AW, Farah BA. Reduced B vitamin therapy for MDD in MTHFR C677t/A1298c patients-clinical response correlates with homocysteine reduction: A double-blind, placebo-controlled study. *CNS Spectr* 2016; **21**(1)**:** 116–117.

244.	Mehdi SM, Atlas SE, Qadir S, Musselman D, Goldberg S, Woolger JM *et al.* Double-blind, randomized crossover study of intravenous infusion of magnesium sulfate versus 5% dextrose on depressive symptoms in adults with treatment-resistant depression. 2017; **71**(3)**:** 204–211

C203 – PUBMED 27862658,EMBASE 613942099.

245.	Mehdi SMA, Atlas SE, Qadir S, Musselman D, Goldberg S, Woolger JM *et al.* Double-blind, randomized crossover study of intravenous infusion of magnesium sulfate versus 5% dextrose on depressive symptoms in adults with treatment-resistant depression. *Psychiatry Clin Neurosci* 2017; **71**(3)**:** 204–211.

246.	Mehdi S, Manohar K, Shariff A, Kinattingal N, Wani SUD, Alshehri S *et al.* Omega-3 Fatty Acids Supplementation in the Treatment of Depression: An Observational Study. *Journal of Personalized Medicine* 2023; **13**(2 C7 - 224).

247.	Melzer J, Brignoli R, Keck ME, Saller R. A hypericum extract in the treatment of depressive symptoms in outpatients: an open study. *Forsch Komplementmed* 2010; **17**(1)**:** 7–14.

248.	Merens W, Booij L, Markus R, Zitman FG, Onkenhout W, Van der Does AJ. The effects of a diet enriched with alpha-lactalbumin on mood and cortisol response in unmedicated recovered depressed subjects and controls. 2005; **94**(3)**:** 415–422

C413 – PUBMED 16176613.

249.	Mesbahi SF, Sadegh AM, Asl EM, Boostani H, Irani NS. Effectiveness of Ginseng on the Symptoms of Depression and Anxiety in Patients Referred to Addiction Treatment Clinics in Ahvaz: A Randomized Clinical Trial. *Jundishapur Journal of Natural Pharmaceutical Products* 2025; **20**(1).

250.	Mikola T, Lehto SM, Honkalampi K, Valkonen-Korhonen M, Koivumaa-Honkanen H, Tolmunen T *et al.* A follow-up study with a double-blinded, randomized controlled vitamin D supplementation trial in patients with major depressive episode (DepFuD): a study protocol and baseline characteristics. 2024; **12**(10)**:** 8454–8469

C8453 – PUBMED 39479665.

251.	Millar CL, Wolfe A, Baldyga K, Dufour AB, Lipsitz LA. Berries and Steps: a protocol of a randomized, placebo-controlled pilot study testing freeze-dried blueberry powder in sedentary older adults with mild depressive symptoms. *Nutr J* 2025; **24**(1 C7 - 87).

252.	Mirzavandi F, Babaie S, Rahimpour S, Razmpoosh E, Talenezhad N, Aghaei Zarch SM *et al.* The effect of high dose of intramuscular vitamin D supplement injections on depression in patients with type 2 diabetes and vitamin D deficiency: A randomized controlled clinical trial. *Obes Med* 2020; **17**.

253.	Mischoulon D, Fava M. Docosahexanoic acid and ω-3 fatty acids in depression. *Psychiatric Clinics of North America* 2000; **23**(4)**:** 785–794.

254.	Mischoulon D, Best-Popescu C, Laposata M, Merens W, Murakami JL, Wu SL *et al.* A double-blind dose-finding pilot study of docosahexaenoic acid (DHA) for major depressive disorder. *Eur Neuropsychopharmacol* 2008; **18**(9)**:** 639–645.

255.	Mischoulon D, Papakostas GI, Dording CM, Farabaugh AH, Sonawalla SB, Agoston AM *et al.* A double-blind, randomized controlled trial of ethyl-eicosapentaenoate for major depressive disorder. *Journal of Clinical Psychiatry* 2009; **70**(12)**:** 1636–1644.

256.	Missaoui M, Hassine A, Naija S, Emna J, Ben Amor S. Effect of vitamin D supplementation on anxiety-depressive disorder at six months of stroke: A randomized clinical trial. *Eur J Neurol* 2021; **28**(SUPPL 1)**:** 864.

257.	Mitchell M, Bradley H, Blampied NM, Mulder RT, Rucklidge JJ. Protective effect of micronutrients used to treat antenatal depression on rates of postnatal depression at six months: A secondary analysis of NUTRIMUM. *Journal of Affective Disorders* 2025; **388 C7 - 119560**.

258.	Moazen-Zadeh E, Abbasi SH, Safi-Aghdam H, Shahmansouri N, Arjmandi-Beglar A, Hajhosseinn Talasaz A *et al.* Effects of Saffron on Cognition, Anxiety, and Depression in Patients Undergoing Coronary Artery Bypass Grafting: A Randomized Double-Blind Placebo-Controlled Trial. *J Altern Complement Med* 2018; **24**(4)**:** 361–368.

259.	Mocking RJT, Assies J, Bot M, Jansen EHJM, Schene AH, Pouwer F. Biological Effects of Add-On Eicosapentaenoic Acid Supplementation in Diabetes Mellitus and Co-Morbid Depression: A Randomized Controlled Trial. *PLoS ONE* 2012; **7**(11 C7 - e49431).

260.	Mohajeri S, Hassanpour Moghadam M, Talaei A, Sajadi Tabassi S. Effect of crocin on major depressive disorder: a double blind clinical trial. 2014; **115:** 187

C183 – EMBASE 71549613.

261.	Mohamed S-A, Khafagy GM, Alsaid ITA, Hussein HA. The gut brain axis, effect of dietary changes and probiotics supplement on depression symptoms. 2024; **30**.

262.	Mohsenpour MA, Mohammadi F, Sohrabi Z, Niakousari M, Jeddi M, Eftekhari MH. Mental Health and Health-related Quality of Life Response to Curcumin-Probiotic Drink Powder Co-supplementation in Adults with Overweight/Obesity and Metabolic Syndrome: a Four-arm Randomized, Double-blind, Placebo-controlled, Clinical Trial. *British Journal of Nutrition* 2025.

263.	Mokhber N, Namjoo M, Tara F, Boskabadi H, Rayman MP, Ghayour-Mobarhan M *et al.* Effect of supplementation with selenium on postpartum depression: a randomized double-blind placebo-controlled trial. 2011; **24**(1)**:** 104–108

C103 – PUBMED 20528216.

264.	Moludi J, Alizadeh M, Mohammadzad MHS, Davari M. The Effect of Probiotic Supplementation on Depressive Symptoms and Quality of Life in Patients after Myocardial Infarction: Results of a Preliminary Double-Blind Clinical Trial. *Psychosomatic Medicine* 2019; **81**(9)**:** 770–777.

265.	Moludi J, Khedmatgozar H, Nachvak SM, Abdollahzad H, Moradinazar M, Sadeghpour tabaei A. The effects of co-administration of probiotics and prebiotics on chronic inflammation, and depression symptoms in patients with coronary artery diseases: a randomized clinical trial. *Nutr Neurosci* 2022; **25**(8)**:** 1659–1668.

266.	Moniello G, Bonfitto I, Simonetti F, Dimalta S, Rizzo MR, Bellomo A. Role of L-Arginine supplementation in Long Covidrelated Fatigue and Depression in Elderly Outpatients. *Eur Psychiatry* 2024; **67:** S106.

267.	Moore K, Hughes CF, Hoey L, Ward M, Cunningham C, Molloy AM *et al.* B-vitamins in Relation to Depression in Older Adults Over 60 Years of Age: The Trinity Ulster Department of Agriculture (TUDA) Cohort Study. *Journal of the American Medical Directors Association* 2019; **20**(5)**:** 551–557.e551.

268.	Moosavi SM, Ahmadi M, Amini M, Vazirzadeh B. The effects of 40 and 80 mg hydro- alcoholic extract of crocus sativus in the treatment of mild to moderate depression. *J Mazandaran Univ Med Sci* 2014; **24**(113)**:** 47–53.

269.	Moschonis G, Sarapis K, Resciniti S, Hall R, Yim K, Tonkovic M *et al.* The effectiveness of a Lactobacillus probiotic on measures of psychosocial health in adults diagnosed with subthreshold depression: a double-blind, randomised, placebo-controlled trial. *Proc Nutr Soc* 2024; **83**(OCE1)**:** E58.

270.	Moschonis G, Sarapis K, Resciniti S, Hall R, Yim K, Tonkovic M *et al.* Evaluation of a probiotic blend on psychosocial health and biomarkers of inflammatory, immune and stress response in adults with subthreshold depression: A double-blind, randomised, placebo-controlled trial. *British Journal of Nutrition* 2024.

271.	Mousa A, Naderpoor N, de Courten MPJ, de Courten B. Vitamin D and symptoms of depression in overweight or obese adults: A cross-sectional study and randomized placebo-controlled trial. *Journal of Steroid Biochemistry and Molecular Biology* 2018; **177:** 200–208.

272.	Mozaffari-Khosravi H, Nabizade L, Yassini-Ardakani SM, Hadinedoushan H, Barzegar K. The effect of 2 different single injections of high dose of vitamin D on improving the depression in depressed patients with vitamin D deficiency: A randomized clinical trial. *Journal of Clinical Psychopharmacology* 2013; **33**(3)**:** 378–385.

273.	Mueller BM. St. John's wort for depressive disorders: Results of an outpatient study with the hypericum preparation HYP 811. *Adv Ther* 1998; **15**(2)**:** 109–116.

274.	Mueller M, Ganesh R, Bonnes S. Gut Health = Mental Health? The Impact of Diet and Dietary Supplements on Mood Disorders. *Current Nutrition Reports* 2020; **9**(4)**:** 361–368.

275.	Nabimeybodi R, Ranjbar AM, Javaheri A, Heydari B, Hosseini F, Kardan S *et al.* Assessment of the Effect of Grape Molasses on Preventing Postpartum Depression in Women Referred to Shahid Sadoughi Hospital in Yazd. *Tradit Integr Med* 2024; **9:** 4.

276.	Nagayasu Y, Fujita D, Daimon A, Nunode M, Sawada M, Sano T *et al.* Possible prevention of post-partum depression by intake of omega-3 polyunsaturated fatty acids and its relationship with interleukin 6. *Journal of Obstetrics and Gynaecology Research* 2021; **47**(4)**:** 1371–1379.

277.	Najafabady MT, Baghbadoranee PY, Moghimipour E, Haghighizadeh MH, Boostani H. Comparison the effect of Echium amoenum extract with fluoxetine on depression in menopausal women. A double-blind randomized controlled trial. *Asian J Pharm* 2019; **13**(3)**:** 271–275.

278.	Nct. Examination of a Soy-Based Supplement for Major Depression. 2002.

279.	Nct. Folate Augmentation of Treatment - Evaluation for Depression: a Randomised Controlled Trial. 2007.

280.	Nct. Omega-3 Fatty Acids as Adjunct Treatment for Major Depressive Disorder. 2007.

281.	Nct. Omega-3 Fatty Acids for Treating Adults With Major Depression. 2007.

282.	Nct. St. John's Wort And Kava In The Treatment Of Major Depressive Disorder With Comorbid Anxiety. 2007.

283.	Nct. Comparison of Vitamin B12 Supplementation to Selective Serotonin Reuptake Inhibitor (SSRI) Versus SSRI Antidepressant Treatment Alone. 2009.

284.	Nct. Rhodiola Rosea Therapy of Major Depressive Disorder. 2010.

285.	Nct. Vitamin B6, B12, Folic Acid and Exercise in Parkinson's Disease. 2010.

286.	Nct. N-3 Polyunsaturated Fatty Acids in the Prevention and Treatment for IFN-induced Depression. 2012.

287.	Nct. Effect of Lactobacillus Plantarum 299v Supplementation on Major Depression Treatment. 2015.

288.	Nct. Selenium as Augmentation Treatment for Sertraline Resistant Major Depression. 2016.

289.	Nct. Role of Omega-3 Polyunsaturated Fatty Acid in the Management of Major Depressive Disorder. 2018.

290.	Nct. Effects of Nutrients Supplementation in Antidepressant Treated Depressive Disorder Patients. 2019.

291.	Nct. Cholecalciferol on Depressive Symptoms in Type 2 Diabetes Mellitus Patients. 2021.

292.	Nct. RCT of Adjunctive Curcumin and the Meru Health Program. 2021.

293.	Nct. Supplementation and Depressive Symptomatology Incidence Among Saudi Arabian Adults. 2023.

294.	Nct. Comparison of Krill and Fish Oil on Clinical and Biochemical Outcomes in Depression. 2024.

295.	Nemets B. A Double-Blind, Parallel, Randomized, Add-on Clinical Trial of Creatine Versus Placebo Added to Antidepressant Treatment of Patients With Major Depressive Episode. 2006.

296.	Nemets B, Stahl Z, Belmaker RH. Addition of omega-3 fatty acid to maintenance medication treatment for recurrent unipolar depressive disorder. *AM J PSYCHIATRY* 2002; **159**(3)**:** 477–479.

297.	Nikfarjam M, Goudarzi I, Heidari S, Vardanjani LR, Parvin N. Effect of Ginkgo biloba pill on patients with major depression treated with electroconvulsive therapy. *J Mazandaran Univ Med Sci* 2012; **22**(88)**:** 64–72.

298.	Nikfarjam M, Rakhshan R, Ghaderi H. Comparison of effect of Lavandula officinalis and venlafaxine in treating depression: A double blind clinical trial. *J Clin Diagn Res* 2017; **11**(7)**:** KC01–KC04.

299.	Nikolova V, Cleare A, Young A, Stone J. Probiotics as Adjunctive Treatment in Major Depressive Disorder: Estimates of Treatment Effect and Underlying Mechanisms From a Double-Blind Placebo-Controlled Randomised Pilot Trial. *BJPsych Open* 2023; **9:** S64–S65.

300.	Nikolova VL, Cleare AJ, Young AH, Stone JM. Acceptability, Tolerability, and Estimates of Putative Treatment Effects of Probiotics as Adjunctive Treatment in Patients with Depression: A Randomized Clinical Trial. *JAMA Psychiatry* 2023; **80**(8)**:** 842–847.

301.	Nikolova VL, Cleare AJ, Young AH, Stone JM. Exploring the mechanisms of action of probiotics in depression: Results from a randomized controlled pilot trial. *Journal of Affective Disorders* 2025; **376:** 241–250.

302.	Ning H, Zhou H, Yang N, Ren J, Wang H, Liu W *et al.* Effect of Zishen pingchan granules combined with pramipexole on serum BDNF, IL-1β, IL-6, CRP, TNF-α levels in depressed patients with Parkinson's disease: Results of a randomized, double-blind, controlled study. *Exp Gerontol* 2023; **182**.

303.	Are omega-3 PUFAs effective for depression during pregnancy in countries where fish consumptions are high? *Proceedings of the PSYCHOTHERAPY AND PSYCHOSOMATICS*2015. KARGER ALLSCHWILERSTRASSE 10, CH-4009 BASEL, SWITZERLAND.

304.	Nishi D, Su KP, Usuda K, Chang JPC, Hamazaki K, Ishima T *et al.* The effect of omega-3 fatty acids for depressive symptoms among pregnant women in Japan and Taiwan: A randomized, double-blind, placebo-controlled trial and biomarker study. *Psychother Psychosom* 2019; **88:** 96–97.

305.	Nishi D, Su KP, Usuda K, Chang JPC, Hamazaki K, Ishima T *et al.* Plasma estradiol levels and antidepressant effects of omega-3 fatty acids in pregnant women. *Brain, Behavior, and Immunity* 2020; **85:** 29–34.

306.	Noori M, Shateri Z, Babajafari S, Eskandari MH, Parastouei K, Ghasemi M *et al.* The effect of probiotic-fortified kefir on depression, appetite, oxidative stress, and inflammatory parameters in Iranian overweight and obese elderly: a randomized, double-blind, placebo-controlled clinical trial. *J Health Popul Nutr* 2025; **44**(1)**:** 30.

307.	Novak F, Vecka M, Meisnerova E, Sevela S, Vavrova L, Rychlikova J *et al.* Fish oil supplementation with various lipid emulsions suppresses in vitro cytokine release in home parenteral nutrition patients: a crossover study. 2019; **72:** 70–79

C73 – PUBMED 31759770,EMBASE 2003873276.

308.	Novak F, Vecka M, Meisnerova E, Sevela S, Vavrova L, Rychlikova J *et al.* Fish oil supplementation with various lipid emulsions suppresses in vitro cytokine release in home parenteral nutrition patients: a crossover study. *Nutrition Research* 2019; **72:** 70–79.

309.	Odebrecht Vargas Nunes S, Porcu M, Odebrecht Vargas H, Urbano M. N-acetylcysteine as an adjunctive treatment to reduce depressive symptom severity when C-reactive protein levels are increased. *Eur Neuropsychopharmacol* 2017; **27:** S788.

310.	Okronipa H, Adu-Afarwuah S, Lartey A, Ashorn P, Vosti SA, Young RR *et al.* Maternal supplementation with small-quantity lipid-based nutrient supplements during pregnancy and lactation does not reduce depressive symptoms at 6 months postpartum in Ghanaian women: a randomized controlled trial. *Archives of Women's Mental Health* 2018; **21**(1)**:** 55–63.

311.	Olagunju AE, Gaddey H. Leveraging CAM to treat depression and anxiety. *Journal of Family Practice* 2020; **69**(5)**:** 221–227.

312.	Omidian M, Mahmoudi M, Abshirini M, Eshraghian MR, Javanbakht MH, Zarei M *et al.* Effects of vitamin D supplementation on depressive symptoms in type 2 diabetes mellitus patients: Randomized placebo-controlled double-blind clinical trial. *Diabetes Metab Syndr* 2019; **13**(4)**:** 2375–2380.

313.	Opiyo RO, Nyasulu PS, Koigi RK, Obondo A, Ogoyi D, Kogi-Makau W. Effect of fish oil omega-3 fatty acids on reduction of depressive symptoms among HIV-seropositive pregnant women: a randomized, double-blind controlled trial. *Annals of General Psychiatry* 2018; **17**(1 C7 - 49).

314.	Osali A, Rostami A. Effect of 6 weeks of aerobic training with nanocurcumin consumption on IL1β, nitric oxide, and depression in women with metabolic syndrome. *Int J Diabetes Dev Countries* 2023; **43**(6)**:** 1007–1014.

315.	Pakseresht S, Boustani H, Azemi ME, Nilsaz J, Babapour R, Haghdust MR. Evaluation of pharmaceutical products of st. john's wort efficacy added on tricyclic antidepressants in treating major depressive disorder: A double blind randomized control trial. *Jundishapur Journal of Natural Pharmaceutical Products* 2012; **7**(3)**:** 106–110.

316.	Parletta N, Zarnowiecki D, Cho J, Wilson A, Bogomolova S, Villani A *et al.* Effects of a Mediterranean-style diet supplemented with fish oil on mental health and quality of life in people with depression. *Nutr Neurosci* 2018; **21:** S41.

317.	Parvin N, Farzaneh S, Nikfarjam M, Shahinfard N, Asarzadegan N. The effect of lavandula angustifolia in the treatment of depression. *Eur Psychiatry* 2011; **26**.

318.	Peet M, Horrobin DF. A dose-ranging study of the effects of ethyl-eicosapentaenoate in patients with ongoing depression despite apparently adequate treatment with standard drugs. *Archives of General Psychiatry* 2002; **59**(10)**:** 913–919.

319.	Penckofer S, Ridosh M, Adams W, Grzesiak M, Woo J, Byrn M *et al.* Vitamin D Supplementation for the Treatment of Depressive Symptoms in Women with Type 2 Diabetes: a Randomized Clinical Trial. 2022; **2022:** 4090807

C4090803 – PUBMED 35280228,EMBASE 2017280621.

320.	Pinto-Sanchez MI, Hall GB, Ghajar K, Nardelli A, Bolino C, Lau JT *et al.* Probiotic Bifidobacterium longum NCC3001 Reduces Depression Scores and Alters Brain Activity: a Pilot Study in Patients With Irritable Bowel Syndrome. 2017; **153**(2)**:** 448–459.e448

C443 – PUBMED 28483500,EMBASE 617425533.

321.	Putranto R, Setiati S, Nasrun MW, Witjaksono F, Immanuel S, Subekti I *et al.* Effects of cholecalciferol supplementation on depressive symptoms, C-peptide, serotonin, and neurotrophin-3 in type 2 diabetes mellitus: a double-blind, randomized, placebo-controlled trial. 2024; **4**(3)**:** e1342

C1343 – PUBMED 39816051.

322.	Raeder MB, Steen VM, Vollset SE, Bjelland I. Associations between cod liver oil use and symptoms of depression: The Hordaland Health Study. *Journal of Affective Disorders* 2007; **101**(1-3)**:** 245–249.

323.	Rahman ST, Waterhouse M, Romero BD, Baxter C, English DR, Almeida OP *et al.* Effect of vitamin D supplementation on depression in older Australian adults. *Int J Geriatr Psychiatry* 2023; **38**(1)**:** e5847.

324.	Rahman ST, Waterhouse M, Romero BD, Baxter C, English DR, Almeida OP *et al.* Effect of vitamin D supplementation on depression in older Australian adults. *International Journal of Geriatric Psychiatry* 2023; **38**(1 C7 - e5847).

325.	Rajizadeh A, Mozaffari-Khosravi H, Yassini-Ardakani M, Dehghani A. Effect of magnesium supplementation on depression status in depressed patients with magnesium deficiency: A randomized, double-blind, placebo-controlled trial. *Nutrition* 2017; **35:** 56–60.

326.	Ranjbar M, Firoozabadi A, Salehi A, Ghorbanifar Z, Zarshenas MM, Sadeghniiat-Haghighi K *et al.* Effects of Herbal combination (Melissa officinalis L. and Nepeta menthoides Boiss. & Buhse) on insomnia severity, anxiety and depression in insomniacs: Randomized placebo controlled trial. *Integr Med Res* 2018; **7**(4)**:** 328–332.

327.	Rao GP, Kariya S, Chaudhari P, Bhondave M. A STUDY TO ASSESS EFFICACY AND SAFETY OF ADD-ON ACETYL-L-CARNITINE AND AGMATINE (REJIYANA®) WITH STANDARD OF CARE (SOC) TO VALIDATE VISIBLE CHANGES IN MAJOR DEPRESSIVE DISORDER A RANDOMIZED CONTROLLED TRIAL: REVIVE TRIAL. *Int J Med Public Health* 2024; **14**(2)**:** 597–604.

328.	Rashmi K, Madhuri P. Evaluation of therapeutic effect of Vachadi ghrita in patients of mild to moderate depression. *Int J Res Ayurveda Pharm* 2017; **8:** 137–140.

329.	Rastegar M, Shoorab NJ, Salari R, Saki A. The Effect of Oral Probiotic Capsules on Postpartum Depression in Primiparous Women: A Triple-blind Randomized Control trial. *Iran J Obstet Gynecol Infertil* 2024; **27**(3)**:** 64–75.

330.	Ravi S, Khalili H, Abbasian L, Arbabi M, Ghaeli P. Effect of Omega-3 Fatty Acids on Depressive Symptoms in HIV-Positive Individuals: A Randomized, Placebo-Controlled Clinical Trial. *Annals of Pharmacotherapy* 2016; **50**(10)**:** 797–807.

331.	Raza ML, Hassan ST, Jamil S, Fatima W, Fatima M. Nutritional interventions in depression: The role of vitamin D and omega-3 fatty acids in neuropsychiatric health. *Clin Nutr* 2025; **45:** 270–280.

332.	Rees AM, Austin MP, Parker GB. Omega-3 fatty acids as a treatment for perinatal depression: Randomized double-blind placebo-controlled trial. *Australian and New Zealand Journal of Psychiatry* 2008; **42**(3)**:** 199–205.

333.	Rees AM, Parker G, Austin MP. A randomized controlled trial to assess the efficacy of omega-3 fatty acids as a treatment for depression during the perinatal period. *Acta Neuropsychiatr* 2006; **18**(6)**:** 267–268.

334.	Regueme SC, Ostan R, Pinto A, Valentini L, Durrieu J, Blanc-Bisson C *et al.* Effects of a 2-month dietand nutraceutical intervention on quality of life and depression with respect to inflammaging: Ristomed, an open label intervention trial. *Clin Nutr* 2016; **35:** S84.

335.	Reininghaus EZ, Platzer M, Kohlhammer-Dohr A, Hamm C, Mörkl S, Bengesser SA *et al.* PROVIT: Supplementary Probiotic Treatment and Vitamin B7 in Depression-A Randomized Controlled Trial. *Nutrients* 2020; **12**(11).

336.	Reininghaus E, Riedrich K, Bengesser S, Mörkl S, Hamm C, Queissner R *et al.* Intake of probiotic supplements for the treatment of depression. *Bipolar Disord* 2020; **22:** 101.

337.	Rocco AD, Rogers JD, Brown R, Werner P, Bottiglieri T. S-adenosyl-methionine improves depression in patients with Parkinson's disease in an open-label clinical trial. *Mov Disord* 2000; **15**(6)**:** 1225–1229.

338.	Rodríguez-Morán M, Guerrero-Romero F, Barragán-Zuñiga J, Gamboa-Gómez CI, Weyman-Vela Y, Arce-Quiñones M *et al.* Combined oral supplementation with magnesium plus vitamin D alleviates mild to moderate depressive symptoms related to long-COVID: an open-label randomized, controlled clinical trial. *Magnes Res* 2024; **37**(3)**:** 49–57.

339.	Rolf L, Muris AH, Bol Y, Damoiseaux J, Smolders J, Hupperts R. High dose vitamin D3 supplementation does not ameliorate depressive symptoms in relapsing remitting multiple sclerosis: Results of a randomized placebo-controlled trial. *Mult Scler* 2016; **22:** 379.

340.	Romijn AR, Rucklidge JJ, Kuijer RG, Frampton C. A double-blind, randomized, placebo-controlled trial of Lactobacillus helveticus and Bifidobacterium longum for the symptoms of depression. *Australian and New Zealand Journal of Psychiatry* 2017; **51**(8)**:** 810–821.

341.	Rondanelli M, Giacosa A, Opizzi A, Pelucchi C, La Vecchia C, Montorfano G *et al.* Long chain omega 3 polyunsaturated fatty acids supplementation in the treatment of elderly depression: Effects on depressive symptoms, on phospholipids fatty acids profile and on health-related quality of life. *Journal of Nutrition, Health and Aging* 2011; **15**(1)**:** 37–44.

342.	Rosenbaum JF, Fava M, Falk WE, Pollack MH, Cohen LS, Cohen BM *et al.* An open-label pilot study of oral S-adenosylmethionine in major depression. An interim report. *Ala J Med Sci* 1988; **25**(3)**:** 301–306.

343.	Ross SM. Rhodiola rosea (SHR-5), Part 2: A standardized extract of Rhodiola rosea is shown to be effective in the treatment of mild to moderate depression. *Holist Nurs Pract* 2014; **28**(3)**:** 217–221.

344.	Rucklidge JJ, Richardson AJ. Multinutrient Supplementation for Prevention of Major Depressive Disorder in Overweight Adults. *JAMA - Journal of the American Medical Association* 2019; **322**(4)**:** 366.

345.	Russell SE, Skvarc DR, Mohebbi M, Camfield D, Byrne LK, Turner A *et al.* The Impact of N-acetylcysteine on Major Depression: Qualitative Observation and Mixed Methods Analysis of Participant Change during a 12-week Randomised Controlled Trial. *Clin Psychopharmacol Neurosci* 2023; **21**(2)**:** 320–331.

346.	Rychlik R, Siedentop H, von den Driesch V, Kasper S. St. John's wort extract WS 5572 in minor to moderately severe depression. Effectiveness and tolerance of 600 and 1200 mg active ingredient daily. *Fortschr Med Orig* 2001; **119**(3-4)**:** 119–128.

347.	Safa M, Fallah Tafti S, Ghassem Boroujerdi F, Talischi F. Clinical trial in the treatment of 80 Iranian patients with major depression disorder by the combination of omega 3 fatty acid and a selective serotonin reuptake inhibitor. *Therapeutic Advances in Psychopharmacology* 2013; **3**(4)**:** 186–190.

348.	Safari M, Asadi A, Aryaeian N, Huseini HF, shidfar F, Jazayeri S *et al.* The effects of melissa officinalis on depression and anxiety in type 2 diabetes patients with depression: a randomized double-blinded placebo-controlled clinical trial. *BMC Compl MedTherapies* 2023; **23**(1 C7 - 140).

349.	Salehi L, Farajnejad M, Esmailzadeh Saieh S. The effect of aromatherapy with Polianthes’s tuberosa on postpartum depression in high-risk pregnancies: A double blind randomized placebo-controlled clinical trial. *The Iranian Journal of Obstetrics, Gynecology and Infertility* 2024; **27**(4)**:** 18–27.

350.	Sánchez-Villegas A, Álvarez-Pérez J, Toledo E, Salas-Salvadó J, Ortega-Azorín C, Zomeño MD *et al.* Seafood consumption, omega-3 fatty acids intake, and life-time prevalence of depression in the PREDIMED-plus trial. *Nutrients* 2018; **10**(12 C7 - 2000).

351.	Sánchez-Villegas A, Martínez-González MA, Estruch R, Salas-Salvadó J, Corella D, Covas MI *et al.* Mediterranean dietary pattern and depression: the PREDIMED randomized trial. 2013; **11:** 208

C203 – PUBMED 24229349,EMBASE 52789647.

352.	Sanders AE, Weatherspoon ED, Ehrmann BM, Soma PS, Shaikh SR, Preisser JS *et al.* Ratio of Omega-6/Omega-3 Polyunsaturated Fatty Acids Associated With Somatic and Depressive Symptoms in People With Painful Temporomandibular Disorder and Irritable Bowel Syndrome. *Journal of Pain* 2022; **23**(10)**:** 1737–1748.

353.	Sarkawi M, Raja Ali RA, Abdul Wahab N, Abdul Rathi ND, Mokhtar NM. A randomized, double-blinded, placebo-controlled clinical trial on Lactobacillus-containing cultured milk drink as adjuvant therapy for depression in irritable bowel syndrome. *Scientific reports* 2024; **14**(1)**:** 9478.

354.	Sarris J. Positive effects of Piper methysticum (kava) in patients with anxiety and depression. *Focus Altern Complement Ther* 2010; **15**(1)**:** 27–28.

355.	Sarris J, Kavanagh DJ, Adams J, Bone K, Byrne G. Kava Anxiety Depression Spectrum Study (KADSS): A mixed methods RCT using an aqueous extract of Piper methysticum. *Complement Ther Med* 2009; **17**(3)**:** 176–178.

356.	Sarris J, Kavanagh DJ, Byrne G, Bone KM, Adams J, Deed G. The Kava Anxiety Depression Spectrum Study (KADSS): A randomized, placebo-controlled crossover trial using an aqueous extract of Piper methysticum. *Psychopharmacology* 2009; **205**(3)**:** 399–407.

357.	Savović J. Neither Hypericum perforatum nor fluoxetine are more effective than placebo in short-term treatment of mild to moderate depression. *Focus Altern Complement Ther* 2005; **10**(4)**:** 286–287.

358.	Schifano F, Garofoli F. Dothiepin vs. S-adenosylmethionine (SAMe) for the treatment of major depression in weaned alcoholics: A pilot study. *Eur Neuropsychopharmacol* 1993; **3**(3)**:** 330.

359.	Schmidt U, Sommer H. St. John's wort extract in the ambulatory therapy of depression. Attention and reaction ability are preserved. *Fortschr Med* 1993; **111**(19)**:** 339–342.

360.	Schmidt U, Maisenbacher J, Harrer G, Kuhn U. Psychomotoric performance improvement: Antidepressant therapy with St John's wort. *THERAPIEWOCHE* 1995; **45**(2)**:** 106+108+110+112.

361.	Schneider E, Doll JPK, Schweinfurth N, Kettelhack C, Schaub AC, Yamanbaeva G *et al.* Effect of short-term, high-dose probiotic supplementation on cognition, related brain functions and BDNF in patients with depression: a secondary analysis of a randomized controlled trial. 2023; **48**(1)**:** E23–E33

C23 – PUBMED 36653035.

362.	Schulz V. Clinical study with hypericum extracts in depressive patients. Results, comparison, conclusions, for anti-depressive pharmacotherapy. *Z Phytother* 2002; **23**(1)**:** 11–15.

363.	Schulz V. St John's wort in the treatment of moderate to severe depression. *Z Phytother* 2005; **26**(3)**:** 127–128.

364.	Schulz V. Single dose of St John's wort versus citalopram in patients with moderate depression. *Z Phytother* 2008; **29**(3)**:** 131–132.

365.	Schulzz V. Rhodiola rosea radix extract for the treatment of mild to moderate depression? Results of a placebo-controlled clinical study. *KIM Komplemetare Integr Med Artztezeitschrift Naturheilverfahren* 2009; **50**(3)**:** 53–54.

366.	Sempels C, Sienaert P. The role of omega-3 fatty acids in the treatment of bipolar disorders: The current situation. *Tijdschrift voor Psychiatrie* 2007; **49**(9)**:** 639–647.

367.	Severus WE. Effects of omega-3 polyunsaturated fatty acids on depression. *Herz* 2006; **31 Suppl 3:** 69–74.

368.	Sharifi A, Vahedi H, Nedjat S, Mohamadkhani A, Hosseinzadeh Attar MJ. Vitamin D Decreases Beck Depression Inventory Score in Patients with Mild to Moderate Ulcerative Colitis: A Double-Blind Randomized Placebo-Controlled Trial. *Journal of Dietary Supplements* 2019; **16**(5)**:** 541–549.

369.	Sheikh M, Hantoushzadeh S, Shariat M, Farahani Z, Ebrahiminasab O. The efficacy of early iron supplementation on postpartum depression, a randomized double-blind placebo-controlled trial. *European Journal of Nutrition* 2017; **56**(2)**:** 901–908.

370.	Shemshian M, Sadeghi A, Kermani T, Seyed HM, Tavalaie S, Norouzi A. Saffron (crocus sativus) in the treatment of anxiety and depression: a double-blind, randomized and placebo-controlled trial. 2011.

371.	Sherpa NN, De Giorgi R, Ostinelli EG, Choudhury A, Dolma T, Dorjee S. Efficacy and safety profile of oral creatine monohydrate in add-on to cognitive-behavioural therapy in depression: An 8-week pilot, double-blind, randomised, placebo-controlled feasibility and exploratory trial in an under-resourced area. *Eur Neuropsychopharmacol* 2025; **90:** 28–35.

372.	Shiloh R, Weizman A, Weizer N, Dorfman-Etrog P, Munitz H. Antidepressive effect of pyridoxine (vitamin B6) in neuroleptic-treated schizophrenic patients with co-morbid minor depression - Preliminary open-label trial. *Harefuah* 2001; **140**(5)**:** 369–373+456.

373.	Shipowick CD, Moore CB, Corbett C, Bindler R. Vitamin D and depressive symptoms in women during the winter: A pilot study. *Appl Nurs Res* 2009; **22**(3)**:** 221–225.

374.	Shippy RA, Mendez D, Jones K, Cergnul I, Karpiak SE. S-adenosylmethionine (SAM-e) for the treatment of depression in people living with HIV/AIDS. *BMC Psychiatry* 2004; **4**.

375.	Silvers KM, Woolley CC, Hamilton FC, Watts PM, Watson RA. Randomised double-blind placebo-controlled trial of fish oil in the treatment of depression. *Prostaglandins Leukotrienes and Essential Fatty Acids* 2005; **72**(3)**:** 211–218.

376.	Singer A, Schmidt M, Hauke W, Stade K. Duration of response after treatment of mild to moderate depression with Hypericum extract STW 3-VI, citalopram and placebo: A reanalysis of data from a controlled clinical trial. *Phytomedicine* 2011.

377.	Sinn N, Milte CM, Street SJ, Buckley JD, Coates AM, Petkov J *et al.* Effects of n-3 fatty acids, EPA v. DHA, on depressive symptoms, quality of life, memory and executive function in older adults with mild cognitive impairment: a 6-month randomised controlled trial. 2012; **107**(11)**:** 1682–1693

C1683 – PUBMED 21929835.

378.	Slykerman RF, Hood F, Wickens K, Thompson JMD, Barthow C, Murphy R *et al.* Effect of Lactobacillus rhamnosus HN001 in Pregnancy on Postpartum Symptoms of Depression and Anxiety: A Randomised Double-blind Placebo-controlled Trial. *EBioMedicine* 2017; **24:** 159–165.

379.	Smith DJ, Sarris J, Dowling N, O'Connor M, Ng CH. Adjunctive low-dose docosahexaenoic acid (DHA) for major depression: An open-label pilot trial. *Nutr Neurosci* 2018; **21**(3)**:** 224–228.

380.	Solati Z, Jazayeri S, Tehrani-Doost M, Mahmoodianfard S, Gohari MR. Zinc monotherapy increases serum brain-derived neurotrophic factor (BDNF) levels and decreases depressive symptoms in overweight or obese subjects: a double-blind, randomized, placebo-controlled trial. 2015; **18**(4)**:** 162–168

C163 – PUBMED 24621065,EMBASE 603838110.

381.	Soltani M, Hosseinzadeh-Attar MJ, Rezaei M, Alipoor E, Vasheghani-Farahani A, Yaseri M *et al.* Effect of nano-curcumin supplementation on cardiometabolic risk factors, physical and psychological quality of life, and depression in patients with coronary slow flow phenomenon: a randomized double-blind clinical trial. *Trials* 2024; **25**(1 C7 - 515).

382.	Song YK, Lee JY, Park W. Modulatory Effect of Eui-E-In-Tang on Serum Leptin Concentration in Obese Korean Female Adults: A Randomized Controlled Trial. *Evid Based Complement Alternat Med* 2016; **2016:** 1894837.

383.	Sousa TMD, Santos LCD. Dietary fatty acids, omega-6/omega-3 ratio and cholesterol intake associated with depressive symptoms in low-risk pregnancy. *Nutr Neurosci* 2020**:** 1–6.

384.	Stevinson C, Ernst E. Hypericum for depression: An update of the clinical evidence. *Eur Neuropsychopharmacol* 1999; **9**(6)**:** 501–505.

385.	Stewart RC, Ashorn P, Umar E, Dewey KG, Ashorn U, Creed F *et al.* The impact of maternal diet fortification with lipid-based nutrient supplements on postpartum depression in rural Malawi: a randomised-controlled trial. *Maternal and Child Nutrition* 2017; **13**(2 C7 - e12299).

386.	Strodl E, Bambling M, Parnam S, Ritchie G, Cramb S, Vitetta L. Probiotics and magnesium orotate for the treatment of major depressive disorder: a randomised double blind controlled trial. *Scientific reports* 2024; **14**(1)**:** 20841.

387.	Su KP, Huang SY, Chiu TH, Huang KC, Huang CL, Chang HC *et al.* Omega-3 fatty acids for major depressive disorder during pregnancy: Results from a randomized, double-blind, placebo-controlled trial. *Journal of Clinical Psychiatry* 2008; **69**(4)**:** 644–651.

388.	Su KP, Lai HC, Yang HT, Su WP, Peng CY, Chang JP *et al.* Omega-3 fatty acids in the prevention of interferon-alpha-induced depression: results from a randomized, controlled trial. *Biol Psychiatry* 2014; **76**(7)**:** 559–566.

389.	Suzuki H, Masuki S, Morikawa A, Ogawa Y, Kamijo YI, Takahashi K *et al.* Effects of 5-aminolevulinic acid supplementation on home-based walking training achievement in middle-aged depressive women: Randomized, double-blind, crossover pilot study. *Scientific Reports* 2018; **8**(1 C7 - 7151).

390.	Syed EU, Wasay M, Awan S. Vitamin B12 supplementation in treating major depressive disorder: A randomized controlled trial. *Open Neurology Journal* 2013; **7**(1)**:** 44–48.

391.	Tabasi M, Mostafavi SA, Oreyzi H, Mohammadi MR, Khaleghi A. The Effectiveness of Transcranial Direct Current Stimulation (tDCS) and Omega-3 on Food Craving, Executive Functions, Weight, and Depressive Symptoms in Patients with Depression and Overweight: A Randomized Controlled Trial. *Iranian Journal of Psychiatry* 2024; **19**(2)**:** 158–170.

392.	Tabeshpour J, Sobhani F, Sadjadi SA, Hosseinzadeh H, Mohajeri SA, Rajabi O *et al.* A double-blind, randomized, placebo-controlled trial of saffron stigma (Crocus sativus L.) in mothers suffering from mild-to-moderate postpartum depression. *Phytomedicine* 2017; **36:** 145–152.

393.	Taheri M, Irandoust K, Sadeghi A, Yari S. The effect of omega-3 fatty acid supplement and aerobic exercise on lipid profile and depression in obese women. *Acta Medica Mediterranea* 2018; **34**(3)**:** 865–870.

394.	Tajalizadekhoob Y, Sharifi F, Fakhrzadeh H, Mirarefin M, Ghaderpanahi M, Badamchizade Z *et al.* The effect of low-dose omega 3 fatty acids on the treatment of mild to moderate depression in the elderly: A double-blind, randomized, placebo-controlled study. *Eur Arch Psychiatry Clin Neurosci* 2011; **261**(8)**:** 539–549.

395.	Tarkesh F, Namavar Jahromi B, Hejazi N, Hoseini G. Effect of vitamin K2 administration on depression status in patients with polycystic ovary syndrome: a randomized clinical trial. *BMC Women's Health* 2022; **22**(1).

396.	Tarleton EK, Littenberg B, MacLean CD, Kennedy AG, Daley C. Role of magnesium supplementation in the treatment of depression: A randomized clinical trial. *PLoS ONE* 2017; **12**(6 C7 - e0180067).

397.	Tatum P, Lindbloom EJ. Is St. John's wort an effective treatment for major depression? *Journal of Family Practice* 2001; **50**(7)**:** 624.

398.	Tayama J, Ogawa S, Nakaya N, Sone T, Hamaguchi T, Takeoka A *et al.* Omega-3 polyunsaturated fatty acids and psychological intervention for workers with mild to moderate depression: a double-blind randomized controlled trial. 2019; **245:** 364–370

C363 – PUBMED 30423463,EMBASE 2001261185.

399.	Tayebi N, Emamghoreishi M, Akbarzadeh M. Effect of vitex agnus-castus on depression of postmenopausal women: A randomized clinical trial. *Shiraz E Med J* 2021; **22**(6).

400.	Tayebi N, Emamghoreishi M, Akbarzadeh M. The Impact of Salvia officinalis Extract on Depression in Postmenopausal Women: A Randomized Clinical Trial. *Int J Women's Health Reproduction Sci* 2024; **12**(2).

401.	Taylor CL, Thomas PR, Aloia JF, Millard PS, Rosen CJ. Questions about Vitamin D for Primary Care Practice: Input from an NIH Conference. *American Journal of Medicine* 2015; **128**(11)**:** 1167–1170.

402.	Tessou KD, Lemus H, Hsu FC, Pierce J, Hong S, Brown L *et al.* Independent and joint impacts of acid-producing diets and depression on physical health among breast cancer survivors. *Nutrients* 2021; **13**(7 C7 - 2422).

403.	Thesing CS, Milaneschi Y, Bot M, Brouwer IA, Owens M, Hegerl U *et al.* Supplementation-induced increase in circulating omega-3 serum levels is not associated with a reduction in depressive symptoms: Results from the MooDFOOD depression prevention trial. *Depress Anxiety* 2020; **37**(11)**:** 1079–1088.

404.	Thesing CS, Milaneschi Y, Bot M, Brouwer IA, Owens M, Hegerl U *et al.* Supplementation-induced increase in circulating omega-3 serum levels is not associated with a reduction in depressive symptoms: results from the MooDFOOD depression prevention trial. 2020; **37**(11)**:** 1079–1088

C1073 – PUBMED 32845021,EMBASE 2005970673.

405.	Thomson M, Sharma V. Alternative Treatments for Postpartum Depression. *Current Treatment Options in Psychiatry* 2023; **10**(3)**:** 288–312.

406.	Tian P, Chen Y, Zhu H, Wang L, Qian X, Zou R *et al.* Bifidobacterium breve CCFM1025 attenuates major depression disorder via regulating gut microbiome and tryptophan metabolism: a randomized clinical trial. 2022; **100:** 233–241

C233 – PUBMED 34875345.

407.	Tian P, Yang H, Hang F, Wang G, Mao X, Jin X *et al.* Evaluation of the clinical efficacy of Pediococcus acidilactici CCFM6432 in alleviating depression. *Microbiome Research Reports* 2024; **3**(4 C7 - 49).

408.	Tiemeier H, Ruud van Tuijl H, Hofman A, Meijer J, Kiliaan AJ, Breteler MMB. Vitamin B12, folate, and homocysteine in depression: The Rotterdam study. *AM J PSYCHIATRY* 2002; **159**(12)**:** 2099–2101.

409.	Tomko RL, Baker NL, Hood CO, Gilmore AK, McClure EA, Squeglia LM *et al.* Depressive symptoms and cannabis use in a placebo-controlled trial of N-Acetylcysteine for adult cannabis use disorder. *Psychopharmacology* 2020; **237**(2)**:** 479–490.

410.	Tomko RL, Gilmore AK, Gray KM. The role of depressive symptoms in treatment of adolescent cannabis use disorder with N-Acetylcysteine. *Addict Behav* 2018; **85:** 26–30.

411.	Tzikos G, Chamalidou E, Christopoulou D, Apostolopoulou A, Gkarmiri S, Pertsikapa M *et al.* Psychobiotics Ameliorate Depression and Anxiety Status in Surgical Oncology Patients: Results from the ProDeCa Study. *Nutrients* 2025; **17**(5).

412.	Ullah H, Di Minno A, Esposito C, El-Seedi HR, Khalifa SAM, Baldi A *et al.* Efficacy of a food supplement based on S-adenosyl methionine and probiotic strains in subjects with subthreshold depression and mild-to-moderate depression: a monocentric, randomized, cross-over, double-blind, placebo-controlled clinical trial. 2022; **156:** 113930

C113933 – PUBMED 36411659.

413.	Van De Rest O, De Goede J, Sytsma F, Oude Griep LM, Geleijnse JM, Kromhout D *et al.* Association of n-3 long-chain PUFA and fish intake with depressive symptoms and low dispositional optimism in older subjects with a history of myocardial infarction. *British Journal of Nutrition* 2010; **103**(9)**:** 1381–1387.

414.	Vaz JDS, Farias DR, Adegboye ARA, Nardi AE, Kac G. Omega-3 supplementation from pregnancy to postpartum to prevent depressive symptoms: a randomized placebo-controlled trial. *BMC Pregnancy Childbirth* 2017; **17**(1)**:** 180.

415.	Vaz J, Farias D, Adegboye A, Nardi A, Kac G. N-3 polyunsaturated fatty acids (PUFAs) supplementation from pregnancy to postpartum to prevent depressive symptoms: A randomized placebo-controlled trial. *Nutr Neurosci* 2018; **21:** S1.

416.	Vaziri F, Nasiri S, Tavana Z, Dabbaghmanesh MH, Sharif F, Jafari P. A randomized controlled trial of vitamin D supplementation on perinatal depression: In Iranian pregnant mothers. *BMC Pregnancy and Childbirth* 2016; **16**(1 C7 - 239).

417.	Velichkov M, Bezur Z, van Reekum CM, Williams CM. A biphasic response to blueberry supplementation on depressive symptoms in emerging adults: a double-blind randomized controlled trial. *European Journal of Nutrition* 2024; **63**(4)**:** 1071–1088.

418.	Venkatasubramanian R, Kumar CN, Pandey RS. A randomized double-blind comparison of fluoxetine augmentation by high and low dosage folic acid in patients with depressive episodes. *Journal of Affective Disorders* 2013; **150**(2)**:** 644–648.

419.	Vreijling SR, Penninx B, Bot M, Watkins E, Owens M, Kohls E *et al.* Effects of dietary interventions on depressive symptom profiles: results from the MooDFOOD depression prevention study. 2021; **52**(15)**:** 1–10

C13 – PUBMED 33823960,EMBASE 634757999.

420.	Vyas CM, Mischoulon D, Chang G, Cook NR, Weinberg A, Copeland T *et al.* Effects of Vitamin D3 and Marine Omega-3 Fatty Acids Supplementation on Indicated and Selective Prevention of Depression in Older Adults: results From the Clinical Center Sub-Cohort of the VITamin D and OmegA-3 TriaL (VITAL). 2023; **84**(4).

421.	Wadud S, Fatima S, Irfan M. ROLE OF ASCORBIC ACID IN IMPROVING MOOD IN PATIENTS WITH DEPRESSION: A RANDOMIZED CONTROLLED TRIAL. *J Postgrad Med Inst* 2024; **38**(2)**:** 97–101.

422.	Walker JG, Batterham PJ, Mackinnon AJ, Jorm AF, Hickie I, Fenech M *et al.* Oral folic acid and vitamin B-12 supplementation to prevent cognitive decline in community-dwelling older adults with depressive symptoms - The Beyond Ageing Project: A randomized controlled trial (American Journal of Clinical Nutrition (2012) 95, (194-203)). *American Journal of Clinical Nutrition* 2012; **96**(2)**:** 448.

423.	Walker SP, Chang SM, Powell CA, Simonoff E, Grantham-McGregor SM. Effects of psychosocial stimulation and dietary supplementation in early childhood on psychosocial functioning in late adolescence: follow-up of randomised controlled trial. 2006; **333**(7566)**:** 472

C473 – PUBMED 16877454,EMBASE 44343443.

424.	Walker JG, Mackinnon AJ, Batterham P, Jorm AF, Hickie I, McCarthy A *et al.* Mental health literacy, folic acid and vitamin B12, and physical activity for the prevention of depression in older adults: Randomised controlled trial. *British Journal of Psychiatry* 2010; **197**(1)**:** 45–54.

425.	Wallace CJK, Milev RV. The Efficacy, Safety, and Tolerability of Probiotics on Depression: Clinical Results From an Open-Label Pilot Study. *Frontiers in Psychiatry* 2021; **12 C7 - 618279**.

426.	Wang XJ, Li J, Zou QD, Jin L. Wuling Capsule for climacteric patients with depression and anxiety state: A randomized, positive parallel controlled trial. *J Chin Integr Med* 2009; **7**(11)**:** 1042–1046.

427.	Wang L, Zhang Y, Chen L, Yuan D, Feng X, Zheng J *et al.* A two-center randomized controlled trial of a repairing mask as an adjunctive treatment for mild to moderate rosacea. *J Cosmet Dermatol* 2024; **23**(10)**:** 3281–3286.

428.	Wasielewski S. Herbal psychotropic drugs for the treatment of mild depression? *Dtsch Apoth Ztg* 2003; **143**(39)**:** 38–39.

429.	Wheatley D. LI 160, an extract of St. John's wort, versus amitriptyline in mildly to moderately depressed outpatients--a controlled 6-week clinical trial. 1997; **30 Suppl 2:** 77–80

C73 – PUBMED 9342764.

430.	Wu SK, Yang KJ, Liu WC, Malau IA, Zailani H, Chang CH *et al.* The Efficacy of Omega-3 Fatty Acids as the Monotherapy for Depression: A Randomized, Double-Blind, Placebo-Controlled Pilot Study. *Nutrients* 2024; **16**(21 C7 - 3688).

431.	Xiao F, Liu L, Zhang WP, Chang XY, Zhang YF. Effect of adding magnesium sulfate to intrathecal low-dose of bupivacaine for patients with severe pre-eclampsia undergoing cesarean delivery. 2016; **9**(10)**:** 19749–19756

C19743 – EMBASE 612964624.

432.	Xue Y, Wang L, Liu T, Zhao T, Xie K, Guo J *et al.* Omega-3 polyunsaturated fatty acids supplementation improves memory in first-diagnosed, drug-naïve patients with depression: Secondary analysis of data from a randomized controlled trial. *Journal of Affective Disorders* 2024; **350:** 403–410.

433.	Yalamanchili V, Gallagher JC. Comparison of the effect of vitamin D and 1,25 dihydroxyvitamin D treatment on depression scores in older women. *Endocr Rev* 2016; **37**(2).

434.	Yalamanchili V, Gallagher JC. Effect of vitamin D supplementation on depression scores in younger caucasian and Africa-American women. *Endocr Rev* 2017; **38**(3).

435.	Yalamanchili V, Gallagher JC. Dose ranging effects of vitamin D3 on the geriatric depression score: A clinical trial. *Journal of Steroid Biochemistry and Molecular Biology* 2018; **178:** 60–64.

436.	Yamada K, Yagi G, Kanba S. Effectiveness of herbal medicine (Rokumigan and Hachimijiogan) for fatigue or loss of energy in patients with partial remitted major depressive disorder. *Psychiatry Clin Neurosci* 2005; **59**(5)**:** 610–612.

437.	Yang C, Bosker FJ, Li J, Schoevers RA. N-acetylcysteine as add-on to antidepressant medication in therapy refractory major depressive disorder patients with increased inflammatory activity: Study protocol of a double-blind randomized placebo-controlled trial. *BMC Psychiatry* 2018; **18**(1).

438.	Yang TJ, Chiu CH, Wu CL, Liao YS, Chang CK. Thirty minutes of moderate-intensity downhill or level running has no effect on postprandial lipemia: A randomized controlled trial. *Chin J Physiol* 2021; **64**(5)**:** 244–250.

439.	Yang B, Lin L, Bazinet RP, Chien YC, Chang JPC, Satyanarayanan SK *et al.* Clinical Efficacy and Biological Regulations of ω-3 PUFA-Derived Endocannabinoids in Major Depressive Disorder. *Psychother Psychosom* 2019; **88**(4)**:** 215–224.

440.	Yoon S, Kim JE, Hwang J, Kim TS, Kang HJ, Namgung E *et al.* Effects of Creatine Monohydrate Augmentation on Brain Metabolic and Network Outcome Measures in Women With Major Depressive Disorder. *Biol Psychiatry* 2016; **80**(6)**:** 439–447.

441.	Yosaee S, Soltani S, Esteghamati A, Motevalian SA, Tehrani-Doost M, Clark CCT *et al.* Effects of zinc, vitamin D, and their co-supplementation on mood, serum cortisol, and brain-derived neurotrophic factor in patients with obesity and mild to moderate depressive symptoms: A phase II, 12-wk, 2 × 2 factorial design, double-blind, randomized, placebo-controlled trial. *Nutrition* 2020; **71 C7 - 110601**.

442.	Yu J, Chukaew P, Rasenick M. A Molecular Rationale for n-3 PUFA Augmentation of Antidepressant Action. *Biol Psychiatry* 2020; **87**(9)**:** S40.

443.	Zailani H, Wu SK, Yang KJ, Malau IA, Liao HF, Chung YL *et al.* Omega-3 polyunsaturated fatty acids in the prevention of relapse in patients with stable bipolar disorder: A 6-month pilot randomized controlled trial. *Psychiatry Res* 2024; **331:** 115633.

444.	Zajecka JM, Fava M, Shelton RC, Barrentine LW, Young P, Papakostas GI. Long-Term Efficacy, Safety, and Tolerability of l-Methylfolate Calcium 15 mg as Adjunctive Therapy With Selective Serotonin Reuptake Inhibitors: A 12-Month, Open-Label Study Following a Placebo-Controlled Acute Study. *Journal of Clinical Psychiatry* 2016; **77**(5)**:** 654–660.

445.	Zaromytidou E, Koufakis T, Dimakopoulos G, Drivakou D, Meristoudis G, Konstantinidou S *et al.* Vitamin D Supplementation Improves Anxiety and Depression Status in Elderly People with Prediabetes: an Open-Label 12-Month Randomized Controlled Study. 2022; **71 C3 - EMBASE 641933907**.

446.	Zaromytidou E, Koufakis T, Dimakopoulos G, Drivakou D, Konstantinidou S, Rakitzi P *et al.* Vitamin D Alleviates Anxiety and Depression in Elderly People with Prediabetes: a Randomized Controlled Study. 2022; **12**(10 C3 - PUBMED 36295786,EMBASE 2019781038).

447.	Zhang ZJ. Herbal medicine and acupuncture for unipolar and bipolar depression: Randomized controlled trials. *Bipolar Disord* 2012; **14:** 137.

448.	Zhang Y, Cao Y, Wang L. The effects of a new, improved Chinese medicine, Gengnianchun formula granules, on hot flushes, depression, anxiety, and sleep in Chinese peri-and postmenopausal women: A randomized placebo-controlled trial. *Menopause* 2020; **27**(8)**:** 899–905.

449.	Zhao AM, Qiu WR, Mao LJ, Ren JG, Xu L, Yao MJ *et al.* The efficacy and safety of Jiedu Tongluo granules for treating post-stroke depression with qi deficiency and blood stasis syndrome: study protocol for a randomized controlled trial. *Trials* 2018; **19**(1)**:** 275.

450.	Zheng S, Tu L, Cicuttini F, Han W, Zhu Z, Antony B *et al.* Vitamin D supplementation improves depressive symptoms in knee osteoarthritis patients over 24 months. *Eur J Immunol* 2019; **49:** 1421.

451.	Zheng S, Tu L, Cicuttini F, Han W, Zhu Z, Antony B *et al.* Effect of Vitamin D Supplementation on Depressive Symptoms in Patients With Knee Osteoarthritis. *Journal of the American Medical Directors Association* 2019; **20**(12)**:** 1634–1640.e1631.

452.	Zheng Y, Xu LJ, Shi L, Pang B, Xu FQ. Modified erxian decoction for peri-menopausal depression: A randomized controlled clinical trial. *Psychother Psychosom* 2019; **88:** 142.

453.	Zhou Z, Fu S, Liu Y, Wang Y, Bu H, Mei Y *et al.* Study of efficacy and safety of Jiaotai pill in the treatment of depression. *Medicine* 2020; **99**(18)**:** E19999.

454.	Zimmer R, Riemer T, Rauch B, Schneider S, Schiele R, Gohlke H *et al.* Effects of 1-year treatment with highly purified omega-3 fatty acids on depression after myocardial infarction: Results from the OMEGA trial. *Journal of Clinical Psychiatry* 2013; **74**(11)**:** e1037–e1045.

455.	Açik M, Çakiroğlu FP, Tekin A, Egeli A. The effect of fish oil versus krill oil intervention on clinical symptoms and cardiometabolic risk factors in patients with major depressive disorder: A randomized placebo-controlled double-blind trial. *Journal of Affective Disorders* 2025; **380:** 171–180.

456.	Ahmadpanah M, Ramezanshams F, Ghaleiha A, Akhondzadeh S, Sadeghi Bahmani D, Brand S. Crocus Sativus L. (saffron) versus sertraline on symptoms of depression among older people with major depressive disorders–a double-blind, randomized intervention study. *Psychiatry Res* 2019; **282**.

457.	Akhondzadeh Basti A, Moshiri E, Noorbala AA, Jamshidi AH, Abbasi SH, Akhondzadeh S. Comparison of petal of Crocus sativus L. and fluoxetine in the treatment of depressed outpatients: A pilot double-blind randomized trial. *Prog Neuro-Psychopharmacol Biol Psychiatry* 2007; **31**(2)**:** 439–442.

458.	Akhondzadeh S, Fallah-Pour H, Afkham K, Jamshidi AH, Khalighi-Cigaroudi F. Comparison of Crocus sativus L. and imipramine in the treatment of mild to moderate depression: A pilot double-blind randomized trial [ISRCTN45683816]. *BMC Complement Altern Med* 2004; **4**.

459.	Akhondzadeh S, Mostafavi SA, Keshavarz SA, Mohammadi MR, Hosseini S, Eshraghian MR. A placebo controlled randomized clinical trial of Crocus sativus L. (saffron) on depression and food craving among overweight women with mild to moderate depression. *Journal of Clinical Pharmacy and Therapeutics* 2020; **45**(1)**:** 134–143.

460.	Akhondzadeh S, Mostafavi SA, Keshavarz SA, Mohammadi MR, Chamari M. Melatonin Effects in Women With Comorbidities of Overweight, Depression, and Sleep Disturbance: A Randomized Placebo Controlled Clinical Trial. *Sleep Med Res* 2022; **13**(1)**:** 22–30.

461.	Akkasheh G, Kashani-Poor Z, Tajabadi-Ebrahimi M, Jafari P, Akbari H, Taghizadeh M *et al.* Clinical and metabolic response to probiotic administration in patients with major depressive disorder: A randomized, double-blind, placebo-controlled trial. *Nutrition* 2016; **32**(3)**:** 315–320.

462.	Amini L, Yaghini O, Ghazavi M, Aslani N. L-carnitine versus Propranolol for pediatric migraine prophylaxis. *Iran J Child Neurol* 2021; **15**(2)**:** 77–86.

463.	Anushiravani M, Manteghi AA, Taghipur A, Eslami M. Comparing Effectiveness of a Combined Herbal Drug Based on Echium Amoenum with Citalopram in the Treatment of Major Depressive Disorder. *Curr Drug Discov Technol* 2019; **16**(2)**:** 232–238.

464.	Araj-Khodaei M, Noorbala AA, Yarani R, Emadi F, Emaratkar E, Faghihzadeh S *et al.* A double-blind, randomized pilot study for comparison of Melissa officinalis L. And Lavandula angustifolia Mill. And Fluoxetine for the treatment of depression. *BMC Compl MedTherapies* 2020; **20**(1).

465.	Baião R, Capitão LP, Higgins C, Browning M, Harmer CJ, Burnet PWJ. Multispecies probiotic administration reduces emotional salience and improves mood in subjects with moderate depression: a randomised, double-blind, placebo-controlled study. *Psychol Med* 2023; **53**(8)**:** 3437–3447.

466.	Basoglu C, Ates MA, Algul A, Ipcioglu OM, Gecici O, Yilmaz O *et al.* Adjuvant folate with escitalopram treatment and homocystein, folate, vitamin B-12 levels in patients with major depressive disorder. 2009; **19**(2)**:** 135–142

C133 – EMBASE 354832073.

467.	Behnke K, Jensen GS, Graubaum HJ, Gruenwald J. Hypericum perforatum versus fluoxetine in the treatment of mild to moderate depression. *Adv Ther* 2002; **19**(1)**:** 43–52.

468.	Bell KM, Plon L, Bunney Jr WE, Potkin SG. S-adenosylmethionine treatment of depression: A controlled clinical trial. *AM J PSYCHIATRY* 1988; **145**(9)**:** 1110–1114.

469.	Bergman J, Miodownik C, Bersudsky Y, Sokolik S, Lerner PP, Kreinin A *et al.* Curcumin as an add-on to antidepressive treatment: A randomized, double-blind, placebo-controlled, pilot clinical study. *Clin Neuropharmacol* 2013; **36**(3)**:** 73–77.

470.	Berk M, Dean OM, Cotton SM, Jeavons S, Tanious M, Kohlmann K *et al.* The efficacy of adjunctive N-acetylcysteine in major depressive disorder: A double-blind, randomized, placebo-controlled trial. *Journal of Clinical Psychiatry* 2014; **75**(6)**:** 628–636.

471.	Bidzan-Wiącek M, Tomczyk M, Błażek M, Mika A, Antosiewicz J. No Effects of Omega-3 Supplementation on Kynurenine Pathway, Inflammation, Depressive Symptoms, and Stress Response in Males: A Placebo-Controlled Trial. *Nutrients* 2024; **16**(21 C7 - 3744).

472.	Bjerkenstedt L, Edman GV, Alken RG, Mannel M. Hypericum extract LI 160 and fluoxetine in mild to moderate depression: A randomized, placebo-controlled multi-center study in outpatients. *Eur Arch Psychiatry Clin Neurosci* 2005; **255**(1)**:** 40–47.

473.	Bot M, Pouwer F, Assies J, Jansen EH, Diamant M, Snoek FJ *et al.* Eicosapentaenoic acid as an add-on to antidepressant medication for co-morbid major depression in patients with diabetes mellitus: a randomized, double-blind placebo-controlled study. *J Affect Disord* 2010; **126**(1-2)**:** 282–286.

474.	Brenner R, Azbel V, Madhusoodanan S, Pawlowska M. Comparison of an extract of hypericum (LI 160) and sertraline in the treatment of depression: A double-blind, randomized pilot study. *Clin Ther* 2000; **22**(4)**:** 411–419.

475.	Carney RM, Freedland KE, Rubin EH, Rich MW, Steinmeyer BC, Harris WS. A randomized placebo-controlled trial of omega-3 and sertraline in depressed patients with or at risk for coronary heart disease. *Journal of Clinical Psychiatry* 2019; **80**(4 C7 - 19m12742).

476.	Carney RM, Steinmeyer BC, Freedland KE, Rubin EH, Rich MW, Harris WS. Baseline blood levels of omega-3 and depression remission: A secondary analysis of data from a placebo-controlled trial of omega-3 supplements. *Journal of Clinical Psychiatry* 2016; **77**(2)**:** e138–e143.

477.	Caruso I, Fumagalli M, Boccassini L, Puttini SP, Santandrea S, Giniselli G *et al.* Treatment of depression in rheumatoid arthritic patients. A comparison of S-adenosylmethionine (Samyr) and placebo in a double-blind study. *CLIN TRIALS J* 1987; **24**(4)**:** 305–310.

478.	Chang JP, Chang SS, Yang HT, Chen HT, Chien YC, Yang B *et al.* Omega-3 polyunsaturated fatty acids in cardiovascular diseases comorbid major depressive disorder - Results from a randomized controlled trial. *Brain Behav Immun* 2020; **85:** 14–20.

479.	Cheng YC, Chen WY, Lin C, Lee SH, Chiu CC, Kuo PH. The N-3 polyunsaturated fatty acids supplementation to prevent depression recurrence in patients with late-life depression: A 52-week double-blind, placebo-controlled trial. *Journal of Affective Disorders* 2025; **369:** 8–15.

480.	Chopra A, Kalyani AK. Comparison of efficacy of Crocus sativus L. with fluoxetine for improving depression among patients with cardiovascular diseases. *Int J Life Sci Biotechnol Pharma Res* 2020; **9**(2)**:** 151–154.

481.	Darbinyan V, Aslanyan G, Amroyan E, Gabrielyan E, Malmström C, Panossian A. Clinical trial of Rhodiola rosea L. extract SHR-5 in the treatment of mild to moderate depression. *Nord J Psychiatry* 2007; **61**(5)**:** 343–348.

482.	Davidson JR, Gadde KM, Fairbank JA, Krishnan KRR, Califf RM, Binanay C *et al.* Effect of Hypericum perforatum (St John's wort) in major depressive disorder: a randomized controlled trial. 2002; **287**(14)**:** 1807–1814

C1803 – PUBMED 11939866,EMBASE 34303625.

483.	Davidson JRT, Gadde KM, Fairbank JA, Ranga Rama Krishnan K, Califf RM, Binanay C *et al.* Effect of Hypericum perforatum (St John's wort) in major depressive disorder: A randomized controlled trial. *J Am Med Assoc* 2002; **287**(14)**:** 1807–1814.

484.	De Leo D. S-Adenosylmethionine as an antidepressant. A double-blind trial versus placebo. *CURR THER RES CLIN EXP* 1987; **41**(6)**:** 865–870.

485.	Djokic G, Korcok D, Djordjevic V, Agic A, Rankovic A, Djukic Dejanovic S. The effects of s-adenosyl-L-methionine-vitamin b complex on mild and moderate depressive symptoms. *Hippokratia* 2017; **21**(3)**:** 140–143.

486.	Docherty JP, Sack DA, Roffman M, Finch M, Komorowski JR. A double-blind, placebo-controlled, exploratory trial of chromium picolinate in atypical depression: Effect on carbohydrate craving. *Journal of Psychiatric Practice* 2005; **11**(5)**:** 302–314.

487.	Dormal V, Jonniaux L, Buchet M, Simar L, Copine S, Deldicque L. Effect of Hydroponically Grown Red Panax Ginseng on Perceived Stress Level, Emotional Processing, and Cognitive Functions in Moderately Stressed Adults: A Randomized, Double-Blind, Placebo-Controlled Study. *Nutrients* 2025; **17**(6 C7 - 955).

488.	Fava M, Alpert J, Nierenberg AA, Mischoulon D, Otto MW, Zajecka J *et al.* A double-blind, randomized trial of St John's wort, fluoxetine, and placebo in major depressive disorder. *Journal of Clinical Psychopharmacology* 2005; **25**(5)**:** 441–447.

489.	Firoozabadi A, Zarshenas MM, Salehi A, Jahanbin S, Mohagheghzadeh A. Effectiveness of Cuscuta planiflora Ten. and Nepeta menthoides Boiss. & Buhse in major depression: a triple-blind randomized controlled trial study. *J Evid Based Complementary Altern Med* 2015; **20**(2)**:** 94–97.

490.	Freeman MP, Davis M, Sinha P, Wisner KL, Hibbeln JR, Gelenberg AJ. Omega-3 fatty acids and supportive psychotherapy for perinatal depression: A randomized placebo-controlled study. *Journal of Affective Disorders* 2008; **110**(1-2)**:** 142–148.

491.	Friede M, Henneicke von Zepelin HH, Freudenstein J. Differential therapy of mild to moderate depressive episodes (ICD-10 F 32.0; F 32.1) with St. John's wort. *Pharmacopsychiatry* 2001; **34**(SUPPL. 1)**:** S38–S41.

492.	Gao L, Wu C, Liao Y, Wang J. Antidepressants effects of Rhodiola capsule combined with sertraline for major depressive disorder: A randomized double-blind placebo-controlled clinical trial. *Journal of Affective Disorders* 2020; **265:** 99–103.

493.	Gelenberg AJ, Shelton RC, Crits-Christoph P, Keller MB, Dunner DL, Hirschfeld RM *et al.* The effectiveness of St. John's Wort in major depressive disorder: a naturalistic phase 2 follow-up in which nonresponders were provided alternate medication. 2004; **65**(8)**:** 1114–1119

C1113 – PUBMED 15323598.

494.	Ghaffari S, Ghobadi A, Jamshidi AH, Mortazavi SH, Naderi S, Aqamolaei A *et al.* Cinnamomum tamala as an adjuvant therapy in the treatment of major depressive disorder: A double-blind, randomized, placebo-controlled clinical trial with placebo control. *Adv Integr Med* 2020; **7**(3)**:** 141–147.

495.	Ghajar A, Neishabouri SM, Velayati N, Jahangard L, Matinnia N, Haghighi M *et al.* Crocus sativus L versus Citalopram in the Treatment of Major Depressive Disorder with Anxious Distress: A Double-Blind, Controlled Clinical Trial. *Pharmacopsychiatry* 2017; **50**(4)**:** 152–160.

496.	Ghorbani Z, Nazari S, Etesam F, Nourimajd S, Ahmadpanah M, Jahromi SR. The effect of synbiotic as an adjuvant therapy to fluoxetine in moderate depression: A randomized multicenter trial. *Arch Neurosci* 2018; **5**(2).

497.	Grenyer BF, Crowe T, Meyer B, Owen AJ, Grigonis-Deane EM, Caputi P *et al.* Fish oil supplementation in the treatment of major depression: a randomised double-blind placebo-controlled trial. 2007; **31**(7)**:** 1393–1396

C1393 – PUBMED 17659823.

498.	Han CH, Chung MI, Bose S, Lim C, Kim H, Koo BS *et al.* The effect of Gami Guibitang (GGBT), a medicinal herbal formulation, on moderate depression. *Eur J Integr Med* 2012; **4**(4)**:** e400–e407.

499.	Harrer G, Schmidt U, Kuhn U, Biller A. Comparison of equivalence between the St. John's wort extract LoHyp-57 and fluoxetine. *Arzneim-Forsch Drug Res* 1999; **49**(4)**:** 289–296.

500.	He X, Liu D, Ni S, Li Z, Li S, Wu T *et al.* Efficacy and safety evaluation of Ginkgo biloba dropping pill (GBDP) on stable angina pectoris complicated with depression: A placebo-controlled, randomized, double-blind, multicenter study. *Phytomedicine* 2024; **126**.

501.	Hosseini H, Bagherniya M, Sahebkar A, Iraj B, Majeed M, Askari G. The effect of curcumin-piperine supplementation on lipid profile, glycemic index, inflammation, and blood pressure in patients with type 2 diabetes mellitus and hypertriglyceridemia. *Phytother Res* 2024; **38**(11)**:** 5150–5161.

502.	Hu Y, Chen C, Wang Y, Yang W, Wang Y, Zhu W *et al.* The effects of KaiXinSan on depression and its association with lipid profiles: a randomized, double-blinded, placebo-controlled trial. 2021; **83:** 153467

C153463 – PUBMED 33516143,EMBASE 2010802711.

503.	Hvas AM, Juul S, Lauritzen L, Nexø E, Ellegaard J. No effect of vitamin B-12 treatment on cognitive function and depression: A randomized placebo controlled study. *Journal of Affective Disorders* 2004; **81**(3)**:** 269–273.

504.	Jazayeri S, Tehrani-Doost M, Keshavarz SA, Hosseini M, Djazayery A, Amini H *et al.* Comparison of therapeutic effects of omega-3 fatty acid eicosapentaenoic acid and fluoxetine, separately and in combination, in major depressive disorder. *Australian and New Zealand Journal of Psychiatry* 2008; **42**(3)**:** 192–198.

505.	Kagan BL, Sultzer DL, Rosenlicht N, Gerner RH. Oral S-adenosylmethionine in depression: a randomized, double-blind, placebo-controlled trial. 1990; **147**(5)**:** 591–595

C593 – PUBMED 2183633.

506.	Kalb R, Trautmann-Sponsel RD, Kieser M. Efficacy and tolerability of Hypericum extract WS 5572 versus placebo in mildly to moderately depressed patients: A randomized double-blind multicenter clinical trial. *Pharmacopsychiatry* 2001; **34**(3)**:** 96–103.

507.	Kanchanatawan B, Tangwongchai S, Sughondhabhirom A, Suppapitiporn S, Hemrunrojn S, Carvalho AF *et al.* Add-on Treatment with Curcumin Has Antidepressive Effects in Thai Patients with Major Depression: Results of a Randomized Double-Blind Placebo-Controlled Study. *Neurotoxic Res* 2018; **33**(3)**:** 621–633.

508.	Kashani L, Esalatmanesh S, Eftekhari F, Salimi S, Foroughifar T, Etesam F *et al.* Efficacy of Crocus sativus (saffron) in treatment of major depressive disorder associated with post-menopausal hot flashes: a double-blind, randomized, placebo-controlled trial. *Arch Gynecol Obstet* 2018; **297**(3)**:** 717–724.

509.	Kashani L, Eslatmanesh S, Saedi N, Niroomand N, Ebrahimi M, Hosseinian M *et al.* Comparison of Saffron versus Fluoxetine in Treatment of Mild to Moderate Postpartum Depression: A Double-Blind, Randomized Clinical Trial. *Pharmacopsychiatry* 2017; **50**(2)**:** 64–68.

510.	Kasper S, Anghelescu IG, Szegedi A, Dienel A, Kieser M. Superior efficacy of St John's wort extract WS 5570 compared to placebo in patients with major depression: a randomized, double-blind, placebo-controlled, multi-center trial. 2006; **4:** 14

C13 – PUBMED 16796730,EMBASE 44227549.

511.	Kasper S, Anghelescu IG, Szegedi A, Dienel A, Kieser M. Placebo controlled continuation treatment with Hypericum extract WS® 5570 after recovery from a mild or moderate depressive episode. *Wien Med Wochenschr* 2007; **157**(13-14)**:** 362–366.

512.	Kasper S, Anghelescu I, Dienel A. Efficacy of orally administered Silexan in patients with anxiety-related restlessness and disturbed sleep--A randomized, placebo-controlled trial. 2015; **25**(11)**:** 1960–1967

C1963 – PUBMED 26293583,EMBASE 605643089.

513.	Kaviani M, Nikooyeh B, Etesam F, Behnagh SJ, Kangarani HM, Arefi M *et al.* Effects of vitamin D supplementation on depression and some selected pro-inflammatory biomarkers: a double-blind randomized clinical trial. *BMC Psychiatry* 2022; **22**(1 C7 - 694).

514.	Khoraminya N, Tehrani-Doost M, Jazayeri S, Hosseini A, Djazayery A. Therapeutic effects of vitamin D as adjunctive therapy to fluoxetine in patients with major depressive disorder. *Australian and New Zealand Journal of Psychiatry* 2013; **47**(3)**:** 271–275.

515.	Kolouri S, Firoozabadi A, Salehi A, Zarshenas MM, Dastgheib SA, Heydari M *et al.* Nepeta menthoides Boiss. & Buhse freeze-dried aqueous extract versus sertraline in the treatment of major depression: A double blind randomized controlled trial. *Complement Ther Med* 2016; **26:** 164–170.

516.	Kreuzer K, Reiter A, Birkl-Töglhofer AM, Dalkner N, Mörkl S, Mairinger M *et al.* The PROVIT Study—Effects of Multispecies Probiotic Add-on Treatment on Metabolomics in Major Depressive Disorder—A Randomized, Placebo-Controlled Trial. *Metabolites* 2022; **12**(8).

517.	Kumar PNS, Menon V, Andrade C. A randomized, double-blind, placebo-controlled, 12-week trial of vitamin D augmentation in major depressive disorder associated with vitamin D deficiency. *Journal of Affective Disorders* 2022; **314:** 143–149.

518.	Laakmann G. St. John's Wort in mild to moderate depression: The relevance of hyperforin for the clinical efficacy. *Pharmacopsychiatry* 1998; **31**(SUPPL. 1)**:** 54–59.

519.	Levine J, Barak Y, Gonzalves M, Szor H, Elizur A, Kofman O *et al.* Double-blind, controlled trial of inositol treatment of depression. *AM J PSYCHIATRY* 1995; **152**(5)**:** 792–794.

520.	Li Y, Li L, Guo R, Yu H, Wang X, Wang B *et al.* Clinical efficacy of Shugan granule in the treatment of mixed anxiety-depressive disorder: a multicenter, randomized, double-blind, placebo-controlled trial. 2022; **290:** 115032

C115033 – PUBMED 35085742,EMBASE 637131287.

521.	Lin SKK, Kuo PH, Hsu CY, Chiu YH, Chen CH. The effects of Lactobacillus plantarum PS128 in patients with major depressive disorder: an eight-week double-blind, placebo-controlled study. *Asian Journal of Psychiatry* 2024; **101 C7 - 104210**.

522.	Lin CH, Wang SH, Lane HY. Effects of Sodium Benzoate, a D-Amino Acid Oxidase Inhibitor, on Perceived Stress and Cognitive Function Among Patients With Late-Life Depression: A Randomized, Double-Blind, Sertraline- and Placebo-Controlled Trial. *Int J Neuropsychopharmacol* 2022; **25**(7)**:** 545–555.

523.	Lopresti AL, Drummond PD. Efficacy of curcumin, and a saffron/curcumin combination for the treatment of major depression: A randomised, double-blind, placebo-controlled study. *Journal of Affective Disorders* 2017; **207:** 188–196.

524.	Majmasanaye M, Mehrpooya M, Amiri H, Eshraghi A. Discovering the Potential Value of Coenzyme Q10 as an Adjuvant Treatment in Patients with Depression. *Journal of Clinical Psychopharmacology* 2024; **44**(3)**:** 232–239.

525.	Mao JJ, Xie SX, Zee J, Soeller I, Li QS, Rockwell K *et al.* Rhodiola rosea versus sertraline for major depressive disorder: A randomized placebo-controlled trial. *Phytomedicine* 2015; **22**(3)**:** 394–399.

526.	Marangell LB, Martinez JM, Zboyan HA, Kertz B, Kim HFS, Puryear LJ. A double-blind, placebo-controlled study of the omega-3 fatty acid docosahexaenoic acid in the treatment of major depression. *AM J PSYCHIATRY* 2003; **160**(5)**:** 996–998.

527.	Mazidi M, Shemshian M, Mousavi SH, Norouzy A, Kermani T, Moghiman T *et al.* A double-blind, randomized and placebo-controlled trial of Saffron (Crocus sativus L.) in the treatment of anxiety and depression. 2016; **13**(2)**:** 195–199

C193 – PUBMED 27101556,EMBASE 610483123.

528.	Mech AW, Farah A. Correlation of clinical response with homocysteine reduction during therapy with reduced b vitamins in patients with mdd who are positive for MTHFR C677T or A1298C polymorphism: A Randomized, double-blind, placebo-controlled study. *Journal of Clinical Psychiatry* 2016; **77**(5)**:** 668–671.

529.	Mischoulon D, Price LH, Carpenter LL, Tyrka AR, Papakostas GI, Baer L *et al.* A double-blind, randomized, placebo-controlled clinical trial of S-adenosyl-L-methionine (SAMe) versus escitalopram in major depressive disorder. *J Clin Psychiatry* 2014; **75**(4)**:** 370–376.

530.	Moshiri E, Basti AA, Noorbala AA, Jamshidi AH, Hesameddin Abbasi S, Akhondzadeh S. Crocus sativus L. (petal) in the treatment of mild-to-moderate depression: A double-blind, randomized and placebo-controlled trial. *Phytomedicine* 2006; **13**(9-10)**:** 607–611.

531.	Nemets B, Levine J. A pilot dose-finding clinical trial of creatine monohydrate augmentation to SSRIs/SNRIs/NASA antidepressant treatment in major depression. *Int Clin Psychopharmacol* 2013; **28**(3)**:** 127–133.

532.	Ning H, Zhou H, Ren J, Zhou G, Yang N, Wang Z *et al.* Zishen pingchan granules combined with pramipexole in the improvement of depressive symptoms in Parkinson's disease: a prospective, multicenter, randomized, double-blind, controlled clinical study. *J Transl Med* 2022; **20**(1)**:** 357.

533.	Noorbala AA, Akhondzadeh S, Tahmacebi-Pour N, Jamshidi AH. Hydro-alcoholic extract of Crocus sativus L. versus fluoxetine in the treatment of mild to moderate depression: A double-blind, randomized pilot trial. *J Ethnopharmacol* 2005; **97**(2)**:** 281–284.

534.	Nowak G, Siwek M, Dudek D, Zieba A, Pilc A. Effect of zinc supplementation on antidepressant therapy in unipolar depression: A preliminary placebo-controlled study. *Polish Journal of Pharmacology* 2003; **55**(6)**:** 1143–1147.

535.	Padhan M, Mohapatra D, Mishra BR, Maiti R, Jena M. Efficacy and safety of add-on sarcosine in patients with major depressive disorder: A randomized controlled trial. *Journal of Psychiatric Research* 2024; **178:** 298–304.

536.	Panahi Y, Badeli R, Karami GR, Badeli Z, Sahebkar A. A randomized controlled trial of 6-week Chlorella vulgaris supplementation in patients with major depressive disorder. *Complement Ther Med* 2015; **23**(4)**:** 598–602.

537.	Pancheri P, Scapicchio P, Delle Chiaie R. A double-blind, randomized parallel-group, efficacy and safety study of intramuscular S-adenosyl-L-methionine 1, 4-butanedisulphonate (SAMe) versus imipramine in patients with major depressive disorder. *Int J Neuropsychopharmacol* 2002; **5**(4)**:** 287–294.

538.	Papakostas GI, Mischoulon D, Shyu I, Alpert JE, Fava M. S-adenosyl methionine (SAMe) augmentation of serotonin reuptake inhibitors for antidepressant nonresponders with major depressive disorder: a double-blind, randomized clinical trial. 2010; **167**(8)**:** 942–948

C943 – PUBMED 20595412.

539.	Papakostas GI, Shelton RC, Zajecka JM, Etemad B, Rickels K, Clain A *et al.* L-methylfolate as adjunctive therapy for SSRI-resistant major depression: results of two randomized, double-blind, parallel-sequential trials. 2012; **169**(12)**:** 1267–1274

C1263 – PUBMED 23212058.

540.	Park Y, Park YS, Kim SH, Oh DH, Park YC. Supplementation of n-3 polyunsaturated fatty acids for major depressive disorder: A randomized, double-blind, 12-week, placebo-controlled trial in Korea. *Annals of Nutrition and Metabolism* 2015; **66**(2-3)**:** 141–148.

541.	Piperoglou M, Hopwood M, Norman TR. Adjunctive Docosahexaenoic Acid in Residual Symptoms of Depression and Anxiety. *Journal of Clinical Psychopharmacology* 2023; **43**(6)**:** 493–497.

542.	Ranjbar E, Shams J, Sabetkasaei M, M-Shirazi M, Rashidkhani B, Mostafavi A *et al.* Effects of zinc supplementation on efficacy of antidepressant therapy, inflammatory cytokines, and brain-derived neurotrophic factor in patients with major depression. *Nutr Neurosci* 2014; **17**(2)**:** 65–71.

543.	Rondanelli M, Giacosa A, Opizzi A, Pelucchi C, Vecchia CL, Montorfano G *et al.* Effect of omega-3 fatty acids supplementation on depressive symptoms and on health-related quality of life in the treatment of elderly women with depression: A double-blind, placebo-controlled, randomized clinical trial. *J Am Coll Nutr* 2010; **29**(1)**:** 55–64.

544.	Rudzki L, Ostrowska L, Pawlak D, Małus A, Pawlak K, Waszkiewicz N *et al.* Probiotic Lactobacillus Plantarum 299v decreases kynurenine concentration and improves cognitive functions in patients with major depression: A double-blind, randomized, placebo controlled study. *Psychoneuroendocrinology* 2019; **100:** 213–222.

545.	Saccarello A, Montarsolo P, Massardo I, Picciotto R, Pedemonte A, Castagnaro R *et al.* Oral Administration of S-Adenosylmethionine (SAMe) and Lactobacillus Plantarum HEAL9 Improves the Mild-To-Moderate Symptoms of Depression: a Randomized, Double-Blind, Placebo-Controlled Study. 2020; **22**(4 C3 - PUBMED 32589828,EMBASE 632201612).

546.	Sadeghi A, Ghorayshi F, Baghshahi H, Akbari H, Memarzadeh MR, Taghizadeh M *et al.* The antidepressant effect of combined extracts of Hypericum perforatum and Echium amoenum supplementation in patients with depression symptoms: A randomized clinical trial. *Avicenna J Phytomedicine* 2023; **13**(4)**:** 328–337.

547.	Sahoo JP, Jarnail S, Khurana H, Singh P. Omega-3 Fatty Acids Versus 5-hydroxytryptophan as Add on Therapy in Patients with Depression: A Comparative Study. *Indian J Pharmacol* 2015; **47**(7)**:** S23–S24.

548.	Sakurai H, L. Carpenter L, R. Tyrka A, Price LH, I. Papakostas G, Dording CM *et al.* Dose increase of S-Adenosyl-Methionine and escitalopram in a randomized clinical trial for major depressive disorder. *Journal of Affective Disorders* 2020; **262:** 118–125.

549.	Sanmukhani J, Satodia V, Trivedi J, Patel T, Tiwari D, Panchal B *et al.* Efficacy and safety of curcumin in major depressive disorder: a randomized controlled trial. *Phytother Res* 2014; **28**(4)**:** 579–585.

550.	Sarris J, Byrne GJ, Bousman C, Stough C, Murphy J, MacDonald P *et al.* Adjunctive S-adenosylmethionine (SAMe) in treating non-remittent major depressive disorder: An 8-week double-blind, randomized, controlled trial. *Eur Neuropsychopharmacol* 2018; **28**(10)**:** 1126–1136.

551.	Sarris J, Byrne GJ, Stough C, Bousman C, Mischoulon D, Murphy J *et al.* Nutraceuticals for major depressive disorder- more is not merrier: An 8-week double-blind, randomised, controlled trial. *Journal of Affective Disorders* 2019; **245:** 1007–1015.

552.	Sarris J, Murphy J, Stough C, Mischoulon D, Bousman C, MacDonald P *et al.* S-Adenosylmethionine (SAMe) monotherapy for depression: an 8-week double-blind, randomised, controlled trial. 2020; **237**(1)**:** 209–218

C203 – PUBMED 31712971,EMBASE 2003633556.

553.	Schrader E. Equivalence of St John's wort extract (Ze 117) and fluoxetine: A randomized, controlled study in mild-moderate depression. *Int Clin Psychopharmacol* 2000; **15**(2)**:** 61–68.

554.	Schrader E, Meier B, Brattström A. Hypericum treatment of mild-moderate depression in a placebo-controlled study. A prospective, double-blind, randomized, placebo-controlled, multicentre study. *Hum Psychopharmacol* 1998; **13**(3)**:** 163–169.

555.	Sempach L, Schaub A-C, Brühl AB, Sterzer P, Lang UE, Schmidt A. Adjunctive d-serine treatment for major depressive disorder: A randomized clinical trial. *Journal of Affective Disorders* 2025**:** 119504.

556.	Sepehrmanesh Z, Kolahdooz F, Abedi F, Mazroii N, Assarian A, Asemi Z *et al.* Vitamin D supplementation affects the beck depression inventory, insulin resistance, and biomarkers of oxidative stress in patients with major depressive disorder: A randomized, controlled clinical trial. *J Nutr* 2016; **146**(2)**:** 243–248.

557.	Shafiee-Kandjani A, Khalili M, Malek A, Farhang S, Ranjbari Y, Khalili Y. The therapeutic effect of the extracts of Lavandula angustifolia and Dracocephalum ruyschiana besides sertraline on patients with mild to moderate depression: A double-blind controlled trial. *Phytomedicine Plus* 2023; **3**(2).

558.	Shahmansouri N, Farokhnia M, Abbasi SH, Kassaian SE, Noorbala Tafti AA, Gougol A *et al.* A randomized, double-blind, clinical trial comparing the efficacy and safety of Crocus sativus L. with fluoxetine for improving mild to moderate depression in post percutaneous coronary intervention patients. 2014; **155:** 216–222

C213 – PUBMED 24289892,EMBASE 52891114.

559.	Shahmoradi N, Davarinejad O, Brühl AB, Brand S. Effects of Aphrodite (an Herbal Compound) on SSRI-Induced Sexual Dysfunctions and Depression in Females with Major Depressive Disorder: Findings from a Randomized Clinical Trial. *Medicina (Kaunas)* 2023; **59**(9).

560.	Shamabadi A, Fattollahzadeh-Noor S, Fallahpour B, A. Basti F, Khodaei Ardakani MR, Akhondzadeh S. L-Theanine adjunct to risperidone in the treatment of chronic schizophrenia inpatients: a randomized, double-blind, placebo-controlled clinical trial. *Psychopharmacology* 2023; **240**(12)**:** 2631–2640.

561.	Sharifan P, Hosseini MS, Sharifan A. The interventional relationship between frequent fish consumption and depression symptoms in aging adults: a randomized controlled trial. *International Journal of Geriatric Psychiatry* 2017; **32**(12)**:** e116–e122.

562.	Shelton RC, Keller MB, Gelenberg A, Dunner DL, Hirschfeld R, Thase ME *et al.* Effectiveness of St John's wort in major depression: A randomized controlled trial. *J Am Med Assoc* 2001; **285**(15)**:** 1978–1986.

563.	Sherwood Brown E, Gabrielson B. A randomized, double-blind, placebo-controlled trial of citicoline for bipolar and unipolar depression and methamphetamine dependence. *Journal of Affective Disorders* 2012; **143**(1-3)**:** 257–260.

564.	Shinto L, Marracci G, Mohr DC, Bumgarner L, Murchison C, Senders A *et al.* Omega-3 fatty acids for depression in multiple sclerosis: A randomized pilot study. *PLoS ONE* 2016; **11**(1 C7 - e0147195).

565.	Su KP, Huang SY, Chiu CC, Shen WW. Omega-3 fatty acids in major depressive disorder: A preliminary double-blind, placebo-controlled trial. *Eur Neuropsychopharmacol* 2003; **13**(4)**:** 267–271.

566.	Sultana S, Rahman MS. Effect of magnesium supplementation in selective serotonin reuptake inhibitors treated major depressive disorder patients: A randomized, double-blind, placebo-controlled trial. *EC Nutr* 2023; **18:** 25–34.

567.	Szegedi A, Kohnen R, Dienel A, Kieser M. Acute treatment of moderate to severe depression with hypericum extract WS 5570 (St John's wort): Randomised controlled double blind non-inferiority trial versus paroxetine. *Br Med J* 2005; **330**(7490)**:** 503–506.

568.	Talaei A, Noori R, Rezaei Ardani A, Mohammadpour AH, Azimipoor Z, Afzaljavan F. The Effect of Adding Curcumin to Sertraline in the Treatment of Severe Major Depressive Disorder: A Randomized, Double-Blind Clinical Trial. *Clin Neuropharmacol* 2023; **46**(4)**:** 135–139.

569.	Targum SD, Cameron BR, Ferreira L, MacDonald ID. An augmentation study of MSI-195 (S-adenosylmethionine) in Major Depressive Disorder. *Journal of Psychiatric Research* 2018; **107:** 86–96.

570.	Tarutani S, Omori M, Ido Y, Yano M, Komatsu T, Okamura T. Effects of 4G-beta-D-Galactosylsucrose in patients with depression: A randomized, double-blinded, placebo-controlled, parallel-group comparative study. *J Psychiatr Res* 2022; **148:** 110–120.

571.	Vaghef-Mehrabany E, Ranjbar F, Asghari-Jafarabadi M, Hosseinpour-Arjmand S, Ebrahimi-Mameghani M. Calorie restriction in combination with prebiotic supplementation in obese women with depression: effects on metabolic and clinical response. *Nutr Neurosci* 2021; **24**(5)**:** 339–353.

572.	van Gurp G, Meterissian GB, Haiek LN, McCusker J, Bellavance F. St John's wort or sertraline? Randomized controlled trial in primary care. *Can Fam Physician* 2002; **48:** 905–912.

573.	Verma DK, Das B. Effect of zinc supplementation on antidepressant therapy and serum zinc level in unipolar depression: A randomized double blind controlled study. *Indian J Psychiatry* 2016; **58**(5)**:** S47.

574.	Vorbach EU, Arnoldt KH, Hubner WD. Efficacy and tolerability of St. John's wort extract LI 160 versus imipramine in patients with severe depressive episodes according to ICD-10. *Pharmacopsychiatry* 1997; **30**(SUPPL.)**:** 81–85.

575.	Wang L, Bordi PL, Fleming JA, Hill AM, Kris-Etherton PM. Effect of a moderate fat diet with and without avocados on lipoprotein particle number, size and subclasses in overweight and obese adults: a randomized, controlled trial. *J Am Heart Assoc* 2015; **4**(1)**:** e001355.

576.	Woelk H. Comparison of St John's wort and imipramine for treating depression: Randomised controlled trial. *Br Med J* 2000; **321**(7260)**:** 536–539.

577.	Wu SK, Yang KJ, Liu WC, Malau IA, Zailani H, Chang CH *et al.* The Efficacy of Omega-3 Fatty Acids as the Monotherapy for Depression: A Randomized, Double-Blind, Placebo-Controlled Pilot Study. *Nutrients* 2024; **16**(21).

578.	Zhang L, Wang S, Zhu Y, Yang T. Vitamin D3 as adjunctive therapy in the treatment of depression in tuberculosis patients: A short-term pilot randomized double-blind controlled study. *Neuropsychiatr Dis Treat* 2018; **14:** 3103–3109.

579.	Firoozeei TS, Barekatain M, Karimi M, Zargaran A, Akhondzadeh S, Rezaeizadeh H. Lavender and dodder combined herbal syrup versus citalopram in major depressive disorder with anxious distress: A double-blind randomized trial. *Journal of Integrative Medicine* 2020; **18**(5)**:** 409–415.

580.	Masoumi SZ, Kazemi F, Tavakolian S, Rahimi A, Oshvandi K, Soltanian A *et al.* Effect of citalopram in combination with omega-3 on depression in post-menopausal women: A triple blind randomized controlled trial. *J Clin Diagn Res* 2016; **10**(10)**:** QC01–QC05.

581.	Sepehrmanesh Z, Kolahdooz F, Abedi F, Mazroii N, Assarian A, Asemi Z *et al.* Vitamin D Supplementation Affects the Beck Depression Inventory, Insulin Resistance, and Biomarkers of Oxidative Stress in Patients with Major Depressive Disorder: A Randomized, Controlled Clinical Trial [Retraction notice in CN-02298463]. 2016; **146**(2)**:** 243–248

C243 – PUBMED 26609167,EMBASE 608794678.

582.	Ghaleiha A, Davari H, Jahangard L, Haghighi M, Ahmadpanah M, Seifrabie MA *et al.* Adjuvant thiamine improved standard treatment in patients with major depressive disorder: results from a randomized, double-blind, and placebo-controlled clinical trial. *Eur Arch Psychiatry Clin Neurosci* 2016; **266**(8)**:** 695–702.

583.	Ryszewska-Pokraśniewicz B, Mach A, Skalski M, Januszko P, Wawrzyniak ZM, Poleszak E *et al.* Effects of magnesium supplementation on unipolar depression: A placebo-controlled study and review of the importance of dosing and magnesium status in the therapeutic response. *Nutrients* 2018; **10**(8 C7 - 1014).

584.	Coppen A, Bailey J. Enhancement of the antidepressant action of fluoxetine by folic acid: A randomised, placebo controlled trial. *Journal of Affective Disorders* 2000; **60**(2)**:** 121–130.

585.	Sahraian A, Ghanizadeh A, Kazemeini F. Vitamin C as an adjuvant for treating major depressive disorder and suicidal behavior, a randomized placebo-controlled clinical trial. *Trials* 2015; **16**(1 C7 - 94).

586.	Roohi-Azizi M, Arabzadeh S, Amidfar M, Salimi S, Zarindast MR, Talaei A *et al.* Citicoline combination therapy for major depressive disorder: A randomized, double-blind, placebo-controlled trial. *Clin Neuropharmacol* 2017; **40**(1)**:** 1–5.

587.	Jahangard L, Sadeghi A, Ahmadpanah M, Holsboer-Trachsler E, Sadeghi Bahmani D, Haghighi M *et al.* Influence of adjuvant omega-3-polyunsaturated fatty acids on depression, sleep, and emotion regulation among outpatients with major depressive disorders - Results from a double-blind, randomized and placebo-controlled clinical trial. *Journal of Psychiatric Research* 2018; **107:** 48–56.

588.	Ramezani A, Kheirkhah F, Zabihi E, Shirafkan H, Moudi S. Effectiveness of L-carnitine Supplementation to Sertraline for Treatment of Major Depressive Disorder: A Double-blind Randomized Placebo-controlled Trial. *Glob J Med Pharm Biomed Update* 2022; **17**.

589.	Vellekkatt F, Menon V, Rajappa M, Sahoo J. Effect of adjunctive single dose parenteral Vitamin D supplementation in major depressive disorder with concurrent vitamin D deficiency: A double-blind randomized placebo-controlled trial. *J Psychiatr Res* 2020; **129:** 250–256.

590.	Talaei A, Hassanpour Moghadam M, Sajadi Tabassi SA, Mohajeri SA. Crocin, the main active saffron constituent, as an adjunctive treatment in major depressive disorder: A randomized, double-blind, placebo-controlled, pilot clinical trial. *Journal of Affective Disorders* 2015; **174:** 51–56.

591.	Jelodar G, Javid Z, Sahraian A, Jelodar S. Saffron improved depression and reduced homocysteine level in patients with major depression: A Randomized, double-blind study. *Avicenna J Phytomedicine* 2018; **8**(1)**:** 43–50.

592.	Ille R, Spona J, Zickl M, Hofmann P, Lahousen T, Dittrich N *et al.* "Add-On"-therapy with an individualized preparation consisting of free amino acids for patients with a major depression. *Eur Arch Psychiatry Clin Neurosci* 2007; **257**(4)**:** 222–229.

593.	Lyoo IK, Yoon S, Kim TS, Hwang J, Kim JE, Won W *et al.* A randomized, double-blind placebo-controlled trial of oral creatine monohydrate augmentation for enhanced response to a selective serotonin reuptake inhibitor in women with major depressive disorder. *AM J PSYCHIATRY* 2012; **169**(9)**:** 937–945.

594.	Yu JJ, Pei LB, Zhang Y, Wen ZY, Yang JL. Chronic Supplementation of Curcumin Enhances the Efficacy of Antidepressants in Major Depressive Disorder: A Randomized, Double-Blind, Placebo-Controlled Pilot Study. *Journal of Clinical Psychopharmacology* 2015; **35**(4)**:** 406–410.

595.	Yang R, Wang L, Jin K, Cao S, Wu C, Guo J *et al.* Omega-3 Polyunsaturated Fatty Acids Supplementation Alleviate Anxiety Rather Than Depressive Symptoms Among First-Diagnosed, Drug-Naïve Major Depressive Disorder Patients: A Randomized Clinical Trial. *Frontiers in Nutrition* 2022; **9 C7 - 876152**.

596.	Varzaghani V, Sharifi M, Hajiaghaee R, Bagheri S, Momtaz S, Tarassoli Z *et al.* Propolis add-on therapy alleviates depressive symptoms; A randomized placebo-controlled clinical trial. *Phytother Res* 2022; **36**(3)**:** 1258–1267.

597.	Kasper S, Volz HP, Möller HJ, Schläfke S, Klement S, Anghelescu IG *et al.* Lavender oil preparation Silexan is effective in mild-to-moderate major depression: a randomized, placebo- and reference-controlled trial. *Eur Arch Psychiatry Clin Neurosci* 2024.

598.	Siwek M, Dudek D, Schlegel-Zawadzka M, Morawska A, Piekoszewski W, Opoka W *et al.* Serum zinc level in depressed patients during zinc supplementation of imipramine treatment. *Journal of Affective Disorders* 2010; **126**(3)**:** 447–452.

599.	Almeida OP, Ford AH, Hirani V, Singh V, VanBockxmeer FM, McCaul K *et al.* B vitamins to enhance treatment response to antidepressants in middle-aged and older adults: Results from the B-VITAGE randomised, double-blind, placebo-controlled trial. *British Journal of Psychiatry* 2014; **205**(6)**:** 450–457.

600.	Antidepressant Effects of Ayahuasca: a Randomized Placebo Controlled Trial in Treatment Resistant Depression. *clinicaltrials.gov*2016.

601.	Wu R, Zhu D, Xia Y, Wang H, Tao W, Xue W *et al.* A role of Yueju in fast-onset antidepressant action on major depressive disorder and serum BDNF expression: A randomly double-blind, fluoxetine-adjunct, placebo-controlled, pilot clinical study. *Neuropsychiatr Dis Treat* 2015; **11:** 2013–2021.

602.	Gul A, Memtily N, Mijit P, Imin M, Rui H, Wushuer P *et al.* The Üstikuddus sherbiti in the treatment of abnormal cold and dry-type depression patients with comorbid anxiety: A randomized and controlled clinical trial. *Trad Med Mod Med* 2019; **2**(3)**:** 127–133.

603.	Mischoulon D, Dunlop BW, Kinkead B, Schettler PJ, Lamon-Fava S, Rakofsky JJ *et al.* Omega-3 Fatty Acids for Major Depressive Disorder With High Inflammation: A Randomized Dose-Finding Clinical Trial. *Journal of Clinical Psychiatry* 2022; **83**(5 C7 - 21m14074).

604.	Levine J, Barak Y, Kofman O, Belmaker RH. Follow-up and relapse analysis of an inositol study of depression. *Isr J Psychiatry Relat Sci* 1995; **32**(1)**:** 14–21.

605.	Lôo H, Poirier MF, Ollat H, Elatki S. Effects of sulbutiamine (Arcalion 200) on psycho-behavioral inhibition in major depressive episodes. 2000; **26**(2)**:** 70–75

C73 – PUBMED 10858919,EMBASE 30337124.

606.	Sarris J, Fava M, Schweitzer I, Mischoulon D. St Johns Wort (Hypericum perforatum) versus sertraline and placebo in major depressive disorder: Continuation data from a 26-week RCT. *Pharmacopsychiatry* 2012; **45**(7)**:** 275–278.

607.	Davidson JRT, Gadde KM, Fairbank JA, Ranga Rama Krishnan K, Califf RM, Binanay C *et al.* Effect of Hypericum perforatum (St John's wort) in major depressive disorder: A randomized controlled trial. *J Am Med Assoc* 2002; **287**(14)**:** 1807–1814.

608.	Kreuzer K, Reiter A, Birkl-Töglhofer AM, Dalkner N, Mörkl S, Mairinger M *et al.* The PROVIT Study—Effects of Multispecies Probiotic Add-on Treatment on Metabolomics in Major Depressive Disorder—A Randomized, Placebo-Controlled Trial. *Metabolites* 2022; **12**(8).

609.	Leucht S, Fennema H, Engel RR, Kaspers-Janssen M, Szegedi A. Translating the HAM-D into the MADRS and vice versa with equipercentile linking. *Journal of affective disorders* 2018; **226:** 326–331.

610.	Furukawa TA, Reijnders M, Kishimoto S, Sakata M, DeRubeis RJ, Dimidjian S *et al.* Translating the BDI and BDI-II into the HAMD and vice versa with equipercentile linking. 2019.

**S13:** PRISMA NMA Checklist of Items to Include When Reporting A Systematic Review Involving a Network Meta-analysis

| **Section/Topic** | **Item #** | **Checklist Item** | **Reported on Page #** |
| --- | --- | --- | --- |
| **TITLE** |  |  |  |
| Title | 1 | Identify the report as a systematic review *incorporating a network meta-analysis (or related form of meta-analysis).* | ***1*** |
|  |  |  |  |
| **ABSTRACT** |  |  |  |
| Structured summary | 2 | Provide a structured summary including, as applicable:  **Background:** main objectives  **Methods:** data sources; study eligibility criteria, participants, and interventions; study appraisal; and *synthesis methods, such as network meta-analysis.*  **Results:** number of studies and participants identified; summary estimates with corresponding confidence/credible intervals; *treatment rankings may also be discussed. Authors may choose to summarize pairwise comparisons against a chosen treatment included in their analyses for brevity.*  **Discussion/Conclusions:** limitations; conclusions and implications of findings.  **Other:** primary source of funding; systematic review registration number with registry name. | 2-3 |
|  |  |  |  |
| **INTRODUCTION** |  |  |  |
| Rationale | 3 | Describe the rationale for the review in the context of what is already known*, including mention of why a network meta-analysis has been conducted.* | ***4*** |
| Objectives | 4 | Provide an explicit statement of questions being addressed, with reference to participants, interventions, comparisons, outcomes, and study design (PICOS). | 5 |
|  |  |  |  |
| **METHODS** |  |  |  |
| Protocol and registration | 5 | Indicate whether a review protocol exists and if and where it can be accessed (e.g., Web address); and, if available, provide registration information, including registration number. | 6 |
| Eligibility criteria | 6 | Specify study characteristics (e.g., PICOS, length of follow-up) and report characteristics (e.g., years considered, language, publication status) used as criteria for eligibility, giving rationale. *Clearly describe eligible treatments included in the treatment network and note whether any have been clustered or merged into the same node (with justification).* | 6 |
| Information sources | 7 | Describe all information sources (e.g., databases with dates of coverage, contact with study authors to identify additional studies) in the search and date last searched. | 6 |
| Search | 8 | Present full electronic search strategy for at least one database, including any limits used, such that it could be repeated. | 6 and Supplementary |
| Study selection | 9 | State the process for selecting studies (i.e., screening, eligibility, included in systematic review, and, if applicable, included in the meta-analysis). | Figure 1 |
| Data collection process | 10 | Describe method of data extraction from reports (e.g., piloted forms, independently, in duplicate) and processes for obtaining and confirming data from investigators. | 7 |
| Data items | 11 | List and define all variables for which data were sought (e.g., PICOS, funding sources) and any assumptions and simplifications made. | 7 |
| **Geometry of the network** | S1 | Describe methods used to explore the geometry of the treatment network under study and potential biases related to it. This should include how the evidence base has been graphically summarized for presentation, and what characteristics were compiled and used to describe the evidence base to readers. | ***8*** |
| Risk of bias within individual studies | 12 | Describe methods used for assessing risk of bias of individual studies (including specification of whether this was done at the study or outcome level), and how this information is to be used in any data synthesis. | 8 |
| Summary measures | 13 | State the principal summary measures (e.g., risk ratio, difference in means). *Also describe the use of additional summary measures assessed, such as treatment rankings and surface under the cumulative ranking curve (P-SCORE) values, as well as modified approaches used to present summary findings from meta-analyses.* | 7-8 |
| Planned methods of analysis | 14 | Describe the methods of handling data and combining results of studies for each network meta-analysis. This should include, but not be limited to:   - *Handling of multi-arm trials;* - *Selection of variance structure;* - *Selection of prior distributions in Bayesian analyses; and* - *Assessment of model fit.* | 8-9 |
| **Assessment of Inconsistency** | **S2** | Describe the statistical methods used to evaluate the agreement of direct and indirect evidence in the treatment network(s) studied. Describe efforts taken to address its presence when found. | 9 |
| Risk of bias across studies | 15 | Specify any assessment of risk of bias that may affect the cumulative evidence (e.g., publication bias, selective reporting within studies). | **8 and Supplementary** |
| Additional analyses | 16 | Describe methods of additional analyses if done, indicating which were pre-specified. This may include, but not be limited to, the following:   - Sensitivity or subgroup analyses; - Meta-regression analyses; - *Alternative formulations of the treatment network;* - *Use of alternative prior distributions for Bayesian analyses* | ***8-9*** |
| **RESULTS†** |  |  |  |
| Study selection | 17 | Give numbers of studies screened, assessed for eligibility, and included in the review, with reasons for exclusions at each stage, ideally with a flow diagram. | 11 |
| **Presentation of network structure** | **S3** | Provide a network graph of the included studies to enable visualization of the geometry of the treatment network. | ***Figures 2a, 2b, 3a, 3b, 4a, 4b, 5a, 5b, 6a, 6b*** |
| **Summary of network geometry** | **S4** | Provide a brief overview of characteristics of the treatment network. This may include commentary on the abundance of trials and randomized patients for the different interventions and pairwise comparisons in the network, gaps of evidence in the treatment network, and potential biases reflected by the network structure. | **11** |
| Study characteristics | 18 | For each study, present characteristics for which data were extracted (e.g., study size, PICOS, follow-up period) and provide the citations. | Table 1 |
| Risk of bias within studies | 19 | Present data on risk of bias of each study and, if available, any outcome level assessment. | 11 |
| Results of individual studies | 20 | For all outcomes considered (benefits or harms), present, for each study: 1) simple summary data for each intervention group, and 2) effect estimates and confidence intervals. *Modified approaches may be needed to deal with information from larger networks.* | ***Table 1*** |
| Synthesis of results | 21 | Present results of each meta-analysis done, including confidence/credible intervals. *In larger networks, authors may focus on comparisons versus a particular comparator (e.g. placebo or standard care), with full findings presented in an appendix. League tables and forest plots may be considered to summarize pairwise comparisons.* If additional summary measures were explored (such as treatment rankings), these should also be presented. | ***11-20, Supplementary*** |
| **Exploration for inconsistency** | **S5** | Describe results from investigations of inconsistency. This may include such information as measures of model fit to compare consistency and inconsistency models, *P* values from statistical tests, or summary of inconsistency estimates from different parts of the treatment network. | ***11-20 and Supplementary*** |
| Risk of bias across studies | 22 | Present results of any assessment of risk of bias across studies for the evidence base being studied. | 11, Supplementary |
| Results of additional analyses | 23 | Give results of additional analyses, if done (e.g., sensitivity or subgroup analyses, meta-regression analyses*, alternative network geometries studied, alternative choice of prior distributions for Bayesian analyses,* and so forth). | ***11-20 and Supplementary*** |
|  |  |  |  |
| **DISCUSSION** |  |  |  |
| Summary of evidence | 24 | Summarize the main findings, including the strength of evidence for each main outcome; consider their relevance to key groups (e.g., healthcare providers, users, and policy-makers). | 21-24 |
| Limitations | 25 | Discuss limitations at study and outcome level (e.g., risk of bias), and at review level (e.g., incomplete retrieval of identified research, reporting bias). *Comment on the validity of the assumptions, such as transitivity and consistency. Comment on any concerns regarding network geometry (e.g., avoidance of certain comparisons).* | 24 |
| Conclusions | 26 | Provide a general interpretation of the results in the context of other evidence, and implications for future research. | 24-25 |
|  |  |  |  |
| **FUNDING** |  |  |  |
| Funding | 27 | Describe sources of funding for the systematic review and other support (e.g., supply of data); role of funders for the systematic review. This should also include information regarding whether funding has been received from manufacturers of treatments in the network and/or whether some of the authors are content experts with professional conflicts of interest that could affect use of treatments in the network. | ***25*** |
